# Supplementary material for: A Humanized Yeast Phenomic Model of Deoxycytidine Kinase to Predict Genetic Buffering of Nucleoside Analog Cytotoxicity
Source: Genes (Basel). 2019 Sep 30;10(10):770. doi: 10.3390/genes10100770 (PMC6826991; doi:10.3390/genes10100770)
Supplement: Supplementary file 1 [file genes-10-00770-s001.zip › Additional_Files/Additional_File4_InteractionCytarabine.zip!/Additional_File4_InteractionCytarabine/A - InteractionPlots_Cytarabine.pdf]

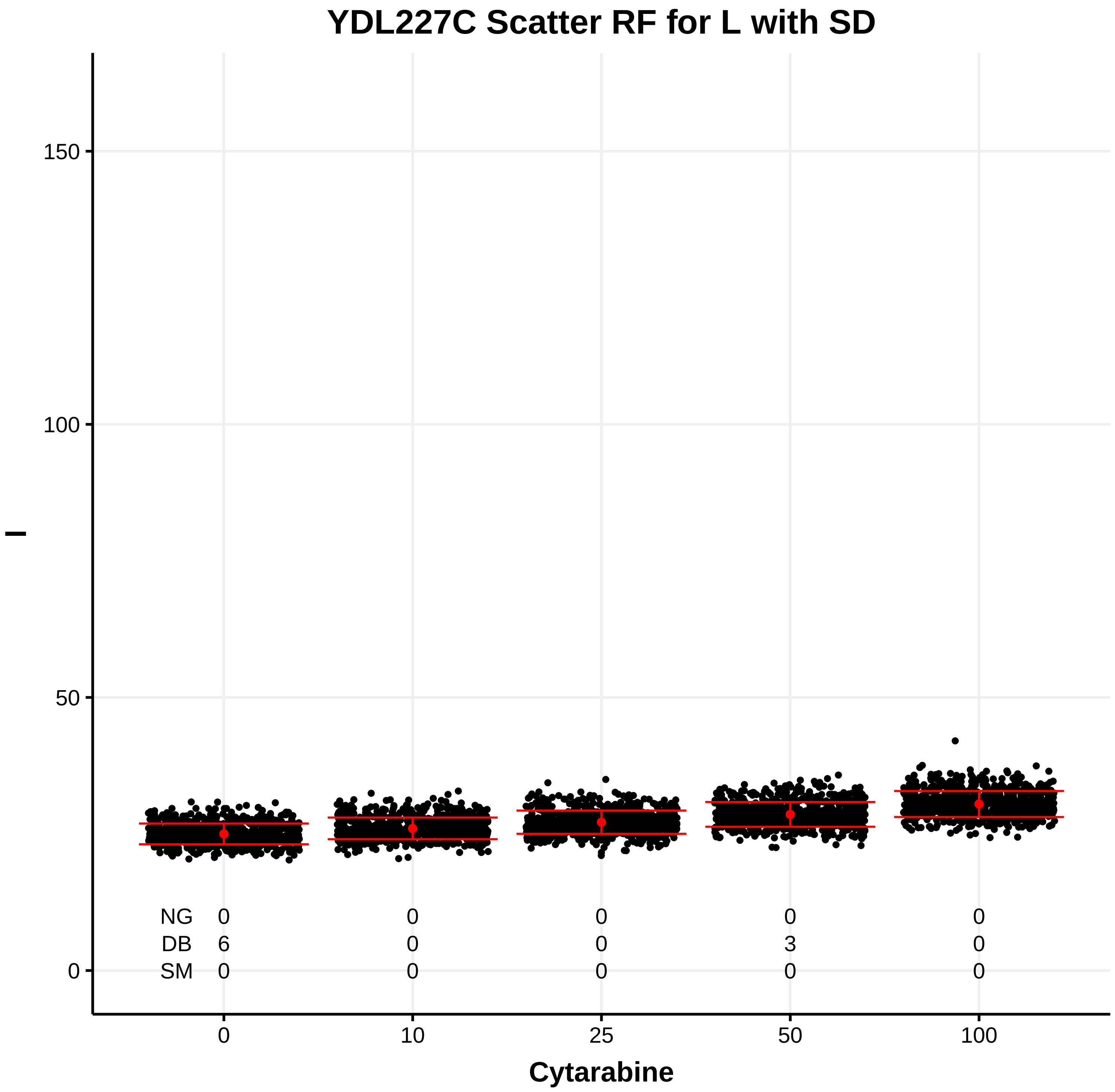

YDL227C Scatter RF for L with SD
l
150
100
50
0
NG 0 0 0 0 0
DB 6 0 0 3 0
SM 0 0 0 0 0
0 10 25 50 100
Cytarabine

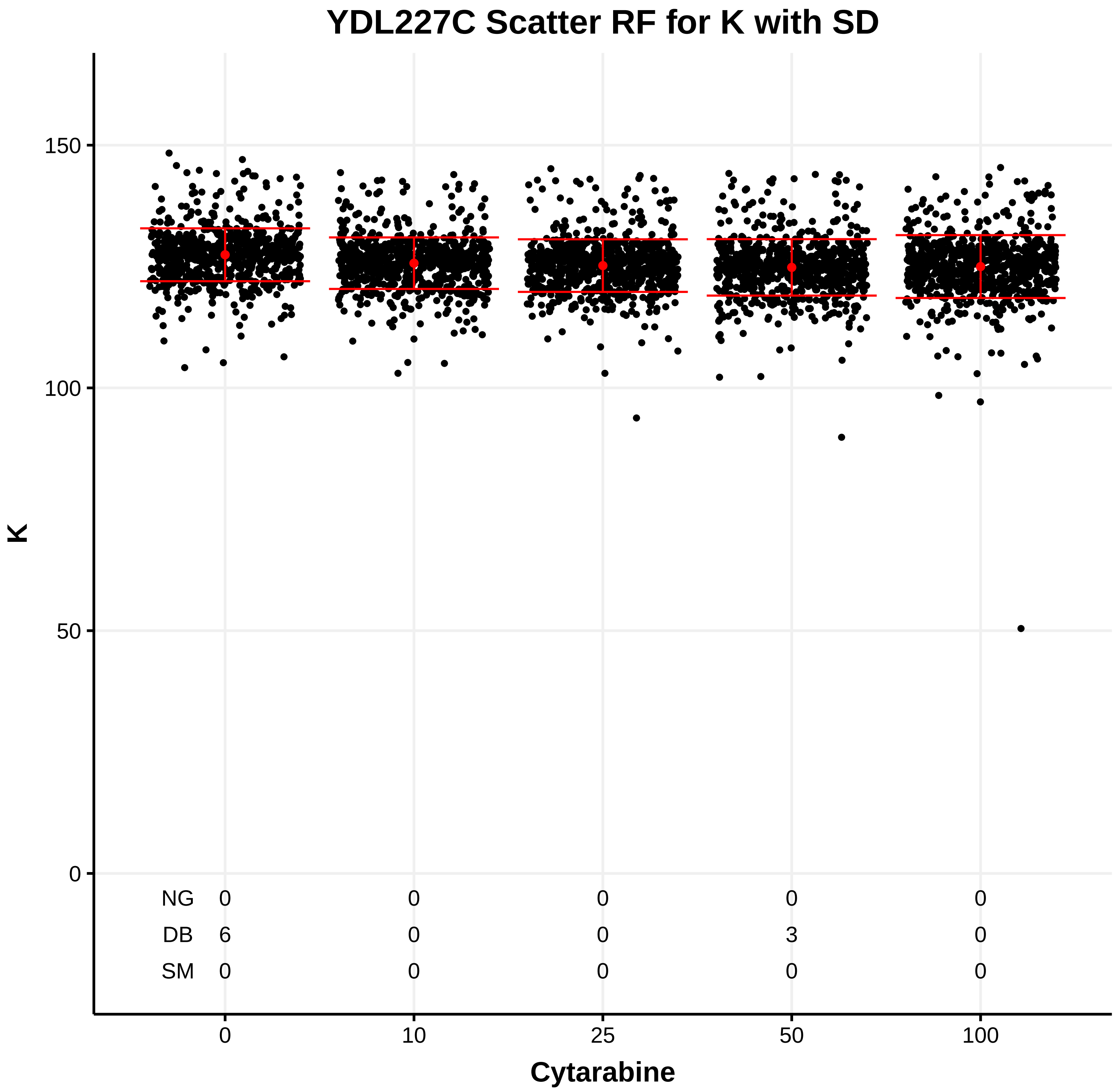

YDL227C Scatter RF for K with SD
K
150
100
50
0
NG 0 0 0 0 0
DB 6 0 0 3 0
SM 0 0 0 0 0
0 10 25 50 100
Cytarabine

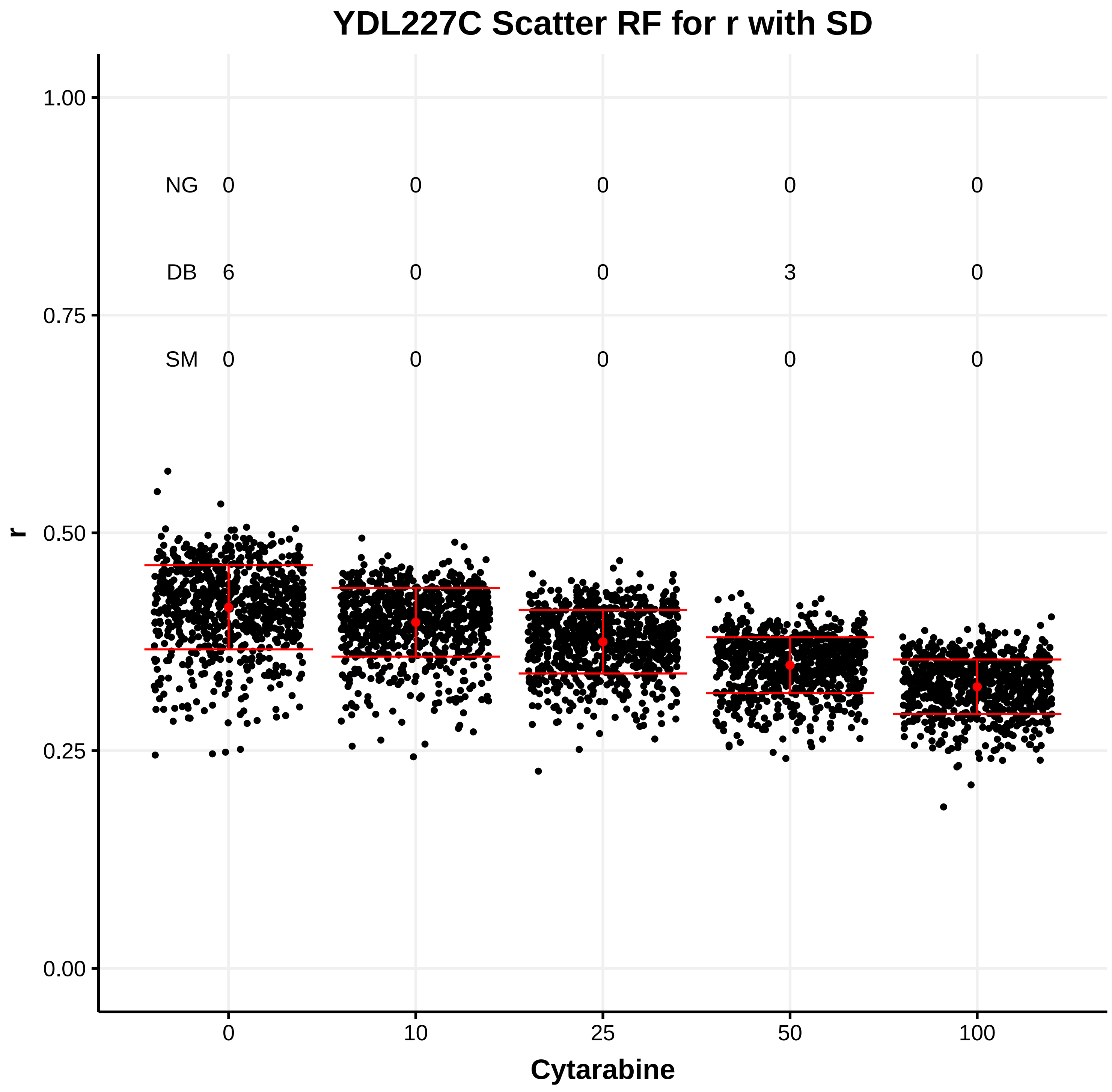

YDL227C Scatter RF for r with SD
r
1.00
0.75
0.50
0.25
0.00
NG 0 0 0 0 0
DB 6 0 0 3 0
SM 0 0 0 0 0
0 10 25 50 100
Cytarabine

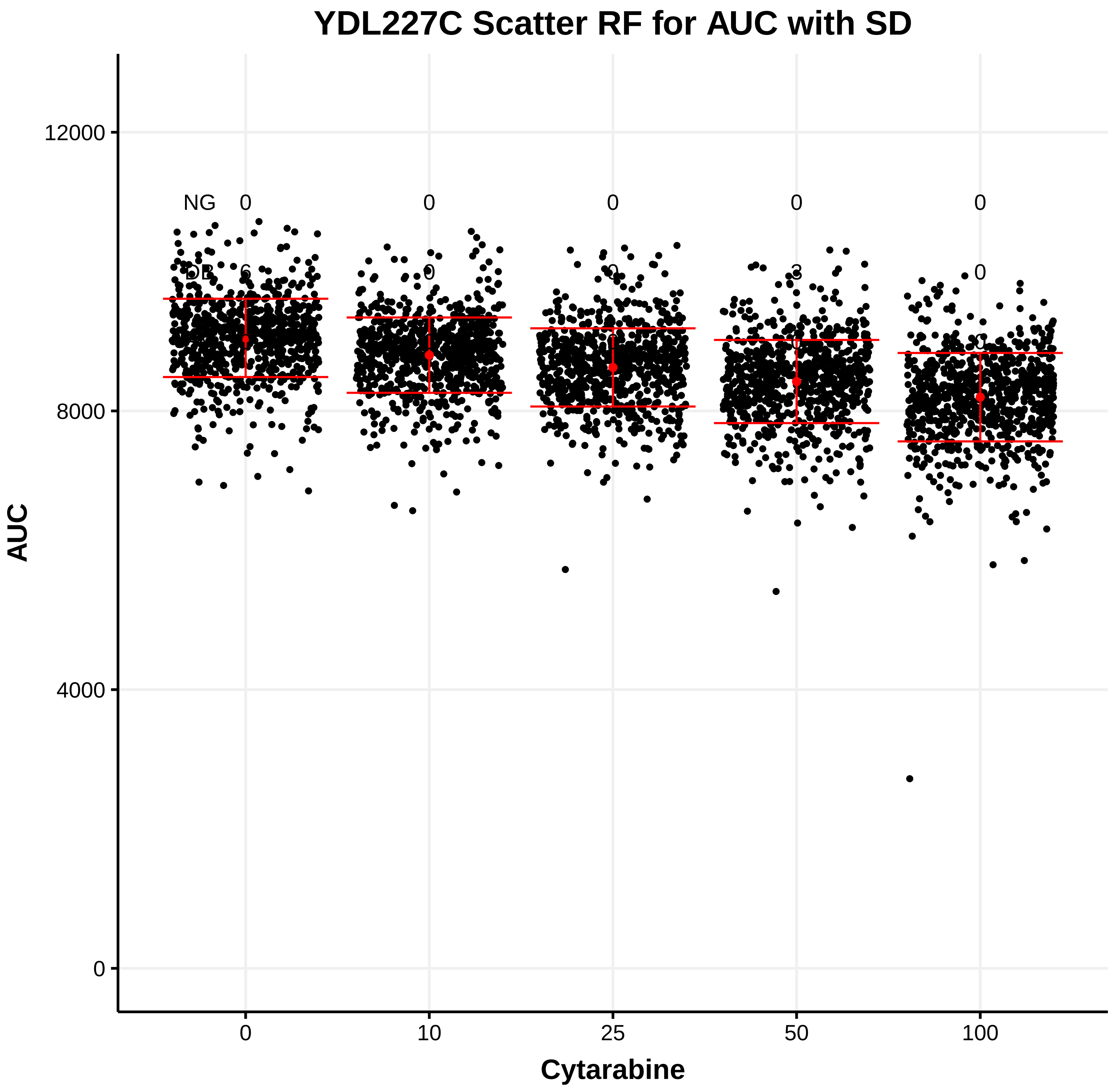

YDL227C Scatter RF for AUC with SD
AUC
12000
8000
4000
0
NG 0 0 0 0 0
0 10 25 50 100
Cytarabine

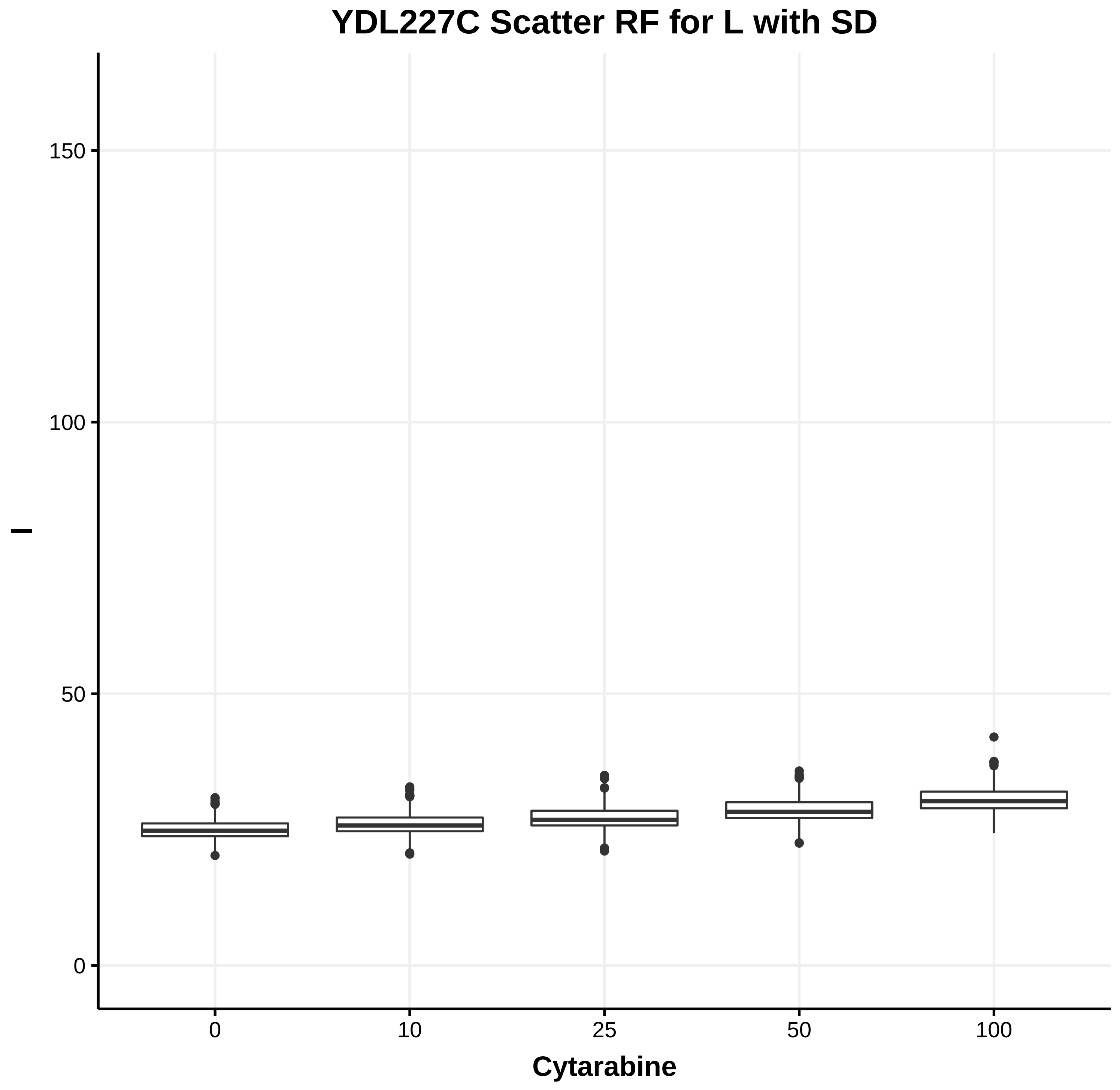
YDL227C Scatter RF for L with SD
l
0
50
100
150
0
10
25
50
100
Cytarabine

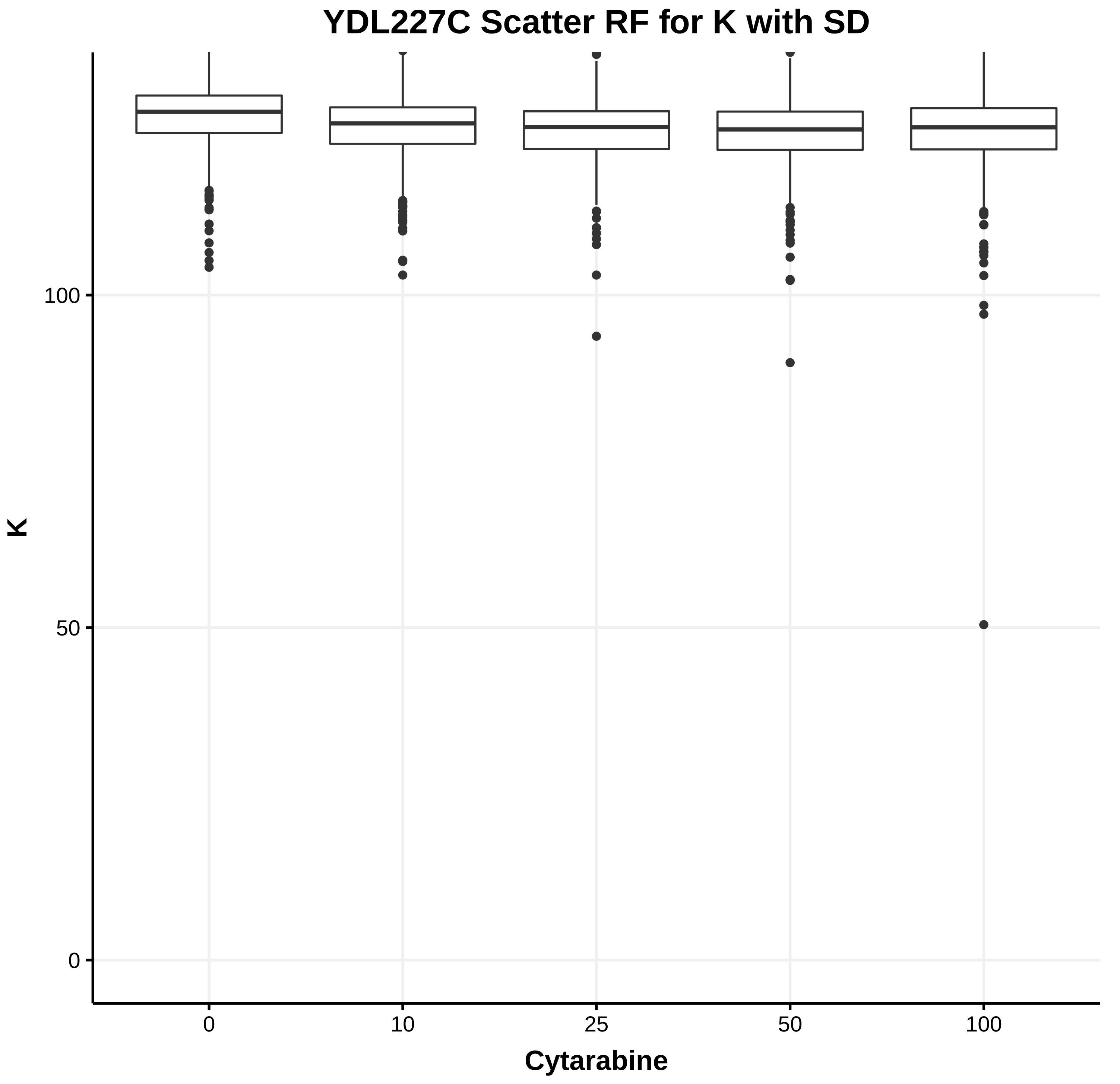
YDL227C Scatter RF for K with SD
K
0
50
100
0
10
25
50
100
Cytarabine

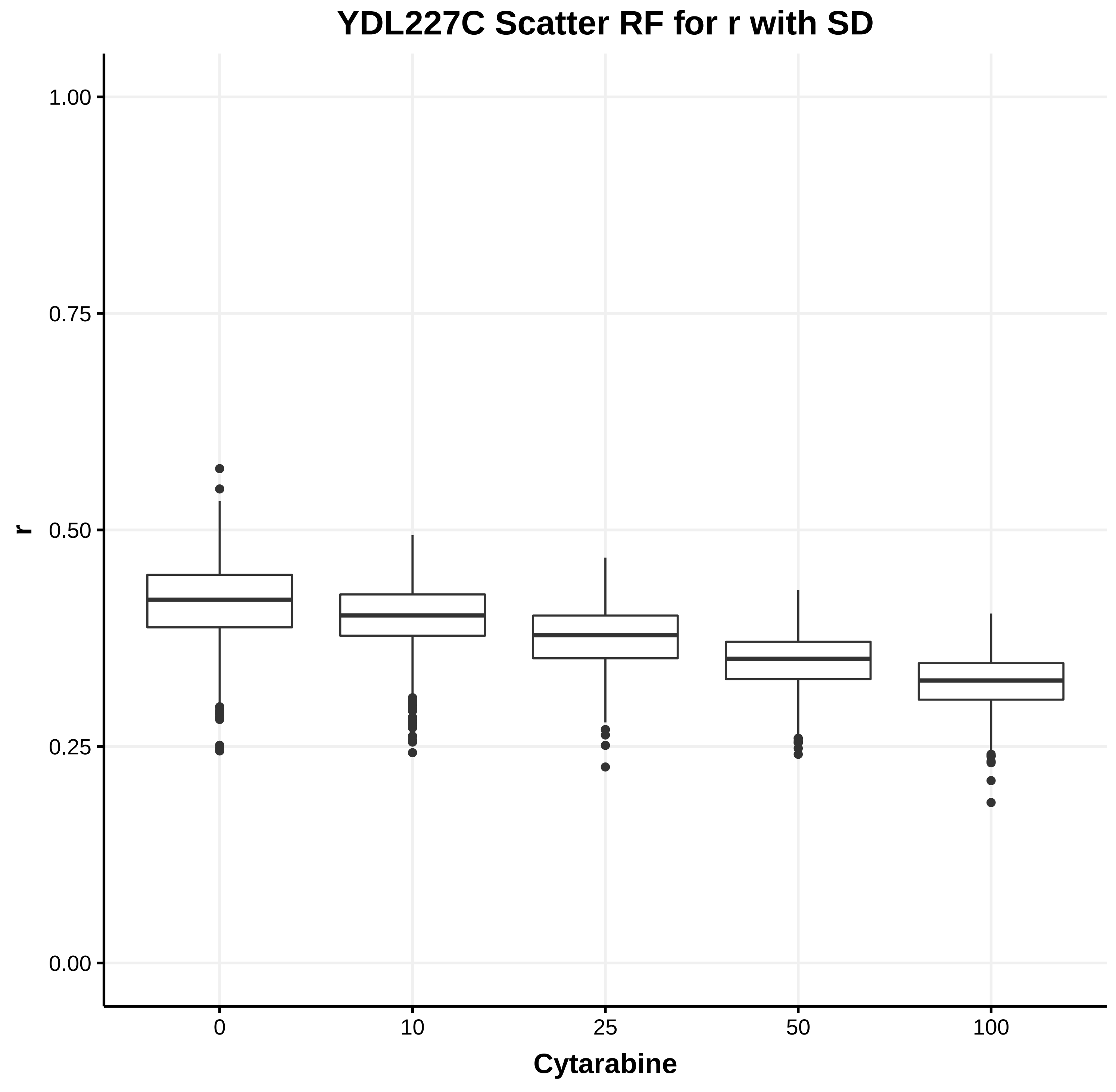
YDL227C Scatter RF for r with SD
r
0.00
0.25
0.50
0.75
1.00
0
10
25
50
100
Cytarabine

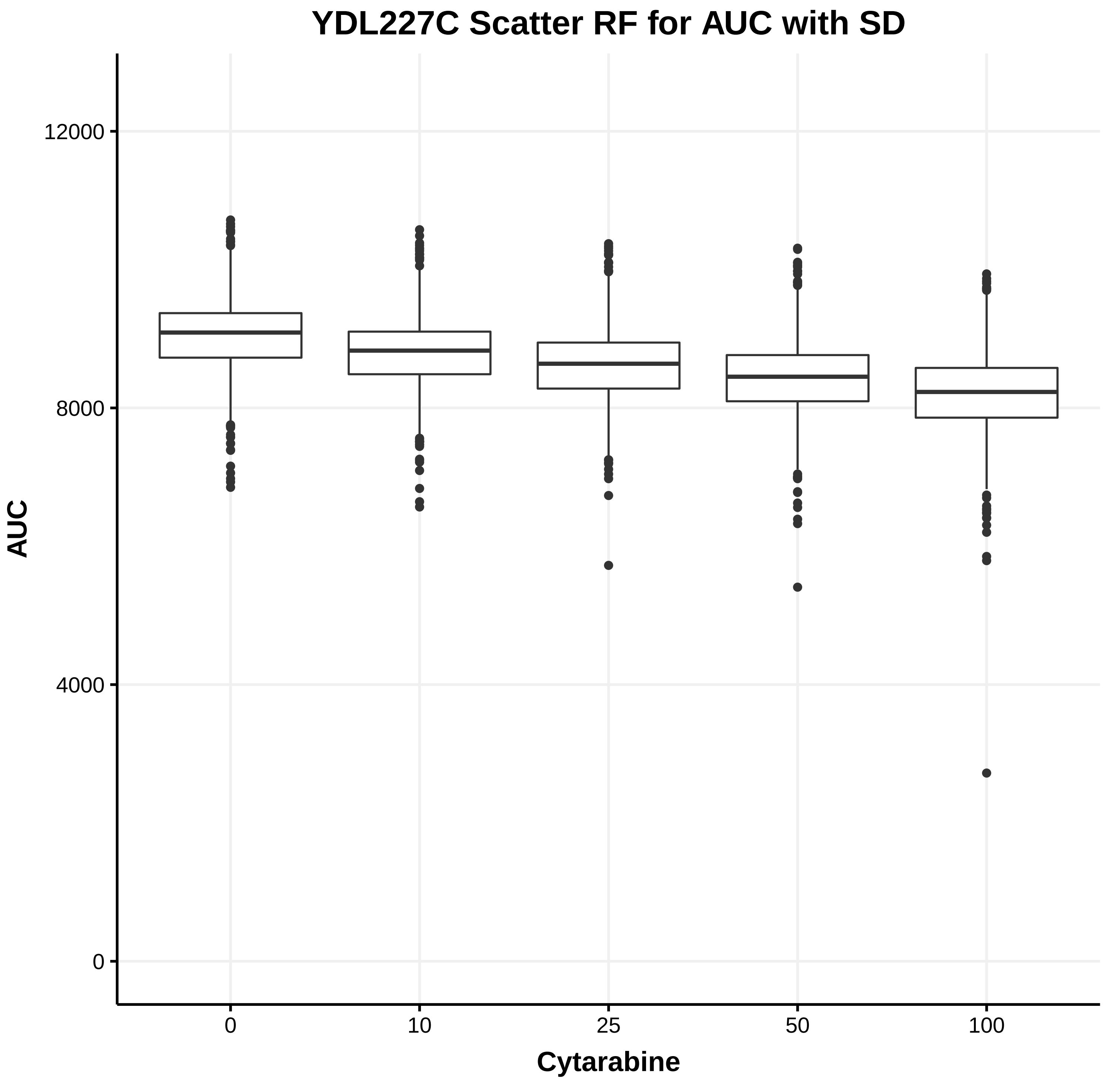
YDL227C Scatter RF for AUC with SD
AUC
0
4000
8000
12000
0
10
25
50
100
Cytarabine

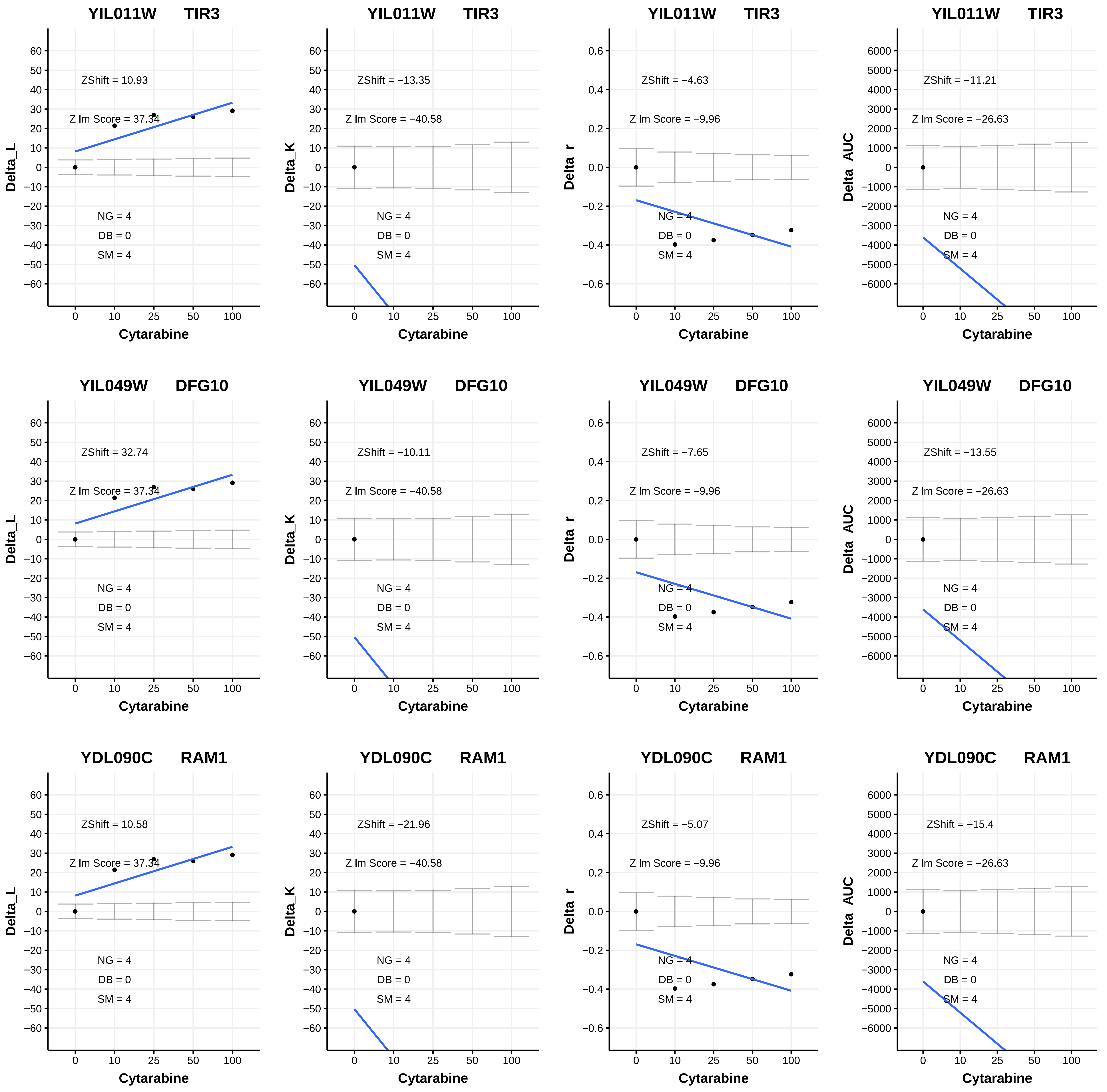
YIL011W TIR3
ZShift = 10.93
Z lm Score = 37.34
NG = 4
DB = 0
SM = 4
Delta_L
Cytarabine
YIL011W TIR3
ZShift = −13.35
Z lm Score = −40.58
NG = 4
DB = 0
SM = 4
Delta_K
Cytarabine
YIL011W TIR3
ZShift = −4.63
Z lm Score = −9.96
NG = 4
DB = 0
SM = 4
Delta_r
Cytarabine
YIL011W TIR3
ZShift = −11.21
Z lm Score = −26.63
NG = 4
DB = 0
SM = 4
Delta_AUC
Cytarabine
YIL049W DFG10
ZShift = 32.74
Z lm Score = 37.34
NG = 4
DB = 0
SM = 4
Delta_L
Cytarabine
YIL049W DFG10
ZShift = −10.11
Z lm Score = −40.58
NG = 4
DB = 0
SM = 4
Delta_K
Cytarabine
YIL049W DFG10
ZShift = −7.65
Z lm Score = −9.96
NG = 4
DB = 0
SM = 4
Delta_r
Cytarabine
YIL049W DFG10
ZShift = −13.55
Z lm Score = −26.63
NG = 4
DB = 0
SM = 4
Delta_AUC
Cytarabine
YDL090C RAM1
ZShift = 10.58
Z lm Score = 37.34
NG = 4
DB = 0
SM = 4
Delta_L
Cytarabine
YDL090C RAM1
ZShift = −21.96
Z lm Score = −40.58
NG = 4
DB = 0
SM = 4
Delta_K
Cytarabine
YDL090C RAM1
ZShift = −5.07
Z lm Score = −9.96
NG = 4
DB = 0
SM = 4
Delta_r
Cytarabine
YDL090C RAM1
ZShift = −15.4
Z lm Score = −26.63
NG = 4
DB = 0
SM = 4
Delta_AUC
Cytarabine

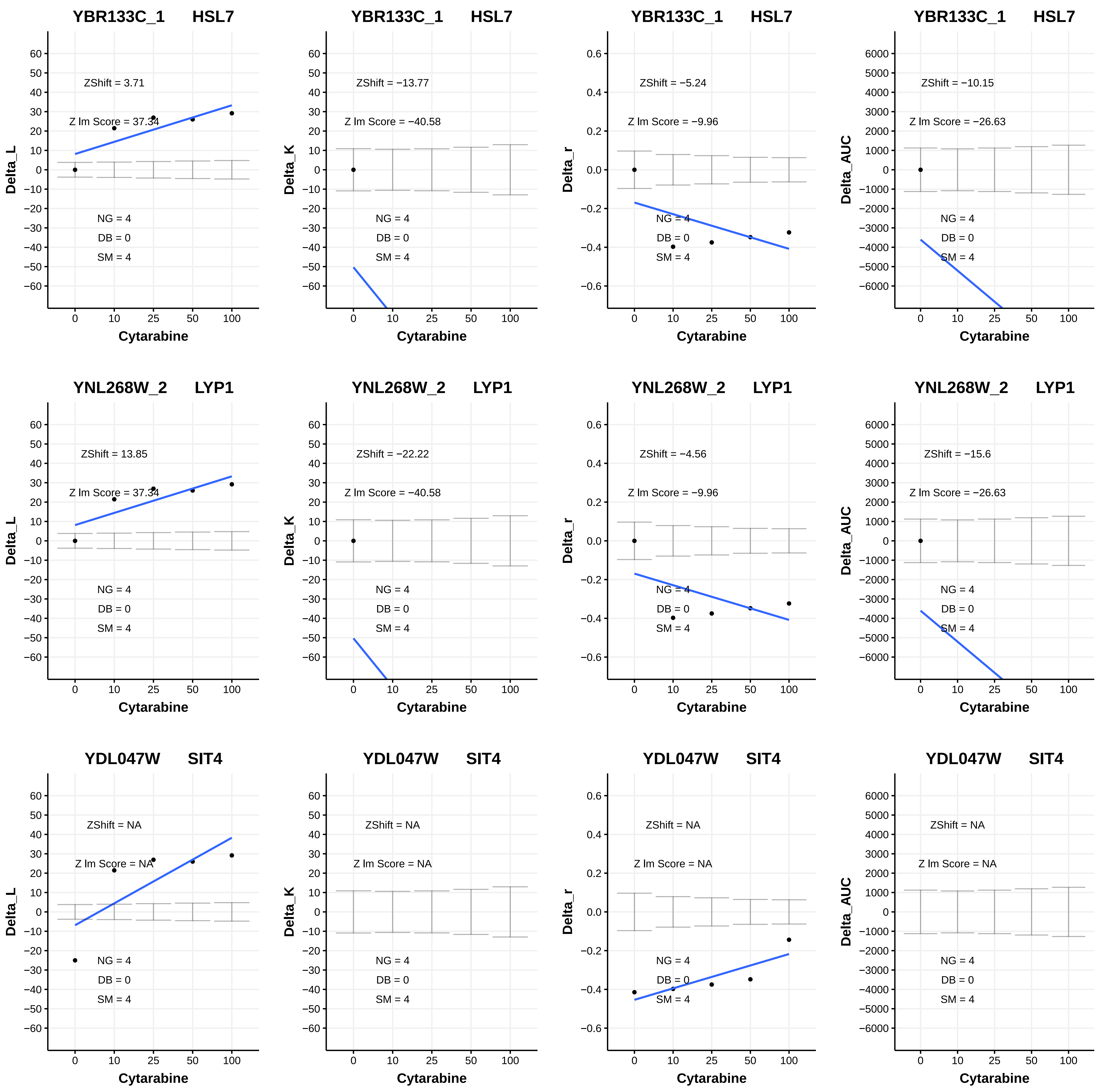
YBR133C_1 HSL7
ZShift = 3.71
Z lm Score = 37.34
NG = 4
DB = 0
SM = 4
Delta_L
Cytarabine
YBR133C_1 HSL7
ZShift = −13.77
Z lm Score = −40.58
NG = 4
DB = 0
SM = 4
Delta_K
Cytarabine
YBR133C_1 HSL7
ZShift = −5.24
Z lm Score = −9.96
NG = 4
DB = 0
SM = 4
Delta_r
Cytarabine
YBR133C_1 HSL7
ZShift = −10.15
Z lm Score = −26.63
NG = 4
DB = 0
SM = 4
Delta_AUC
Cytarabine
YNL268W_2 LYP1
ZShift = 13.85
Z lm Score = 37.34
NG = 4
DB = 0
SM = 4
Delta_L
Cytarabine
YNL268W_2 LYP1
ZShift = −22.22
Z lm Score = −40.58
NG = 4
DB = 0
SM = 4
Delta_K
Cytarabine
YNL268W_2 LYP1
ZShift = −4.56
Z lm Score = −9.96
NG = 4
DB = 0
SM = 4
Delta_r
Cytarabine
YNL268W_2 LYP1
ZShift = −15.6
Z lm Score = −26.63
NG = 4
DB = 0
SM = 4
Delta_AUC
Cytarabine
YDL047W SIT4
ZShift = NA
Z lm Score = NA
NG = 4
DB = 0
SM = 4
Delta_L
Cytarabine
YDL047W SIT4
ZShift = NA
Z lm Score = NA
NG = 4
DB = 0
SM = 4
Delta_K
Cytarabine
YDL047W SIT4
ZShift = NA
Z lm Score = NA
NG = 4
DB = 0
SM = 4
Delta_r
Cytarabine
YDL047W SIT4
ZShift = NA
Z lm Score = NA
NG = 4
DB = 0
SM = 4
Delta_AUC
Cytarabine

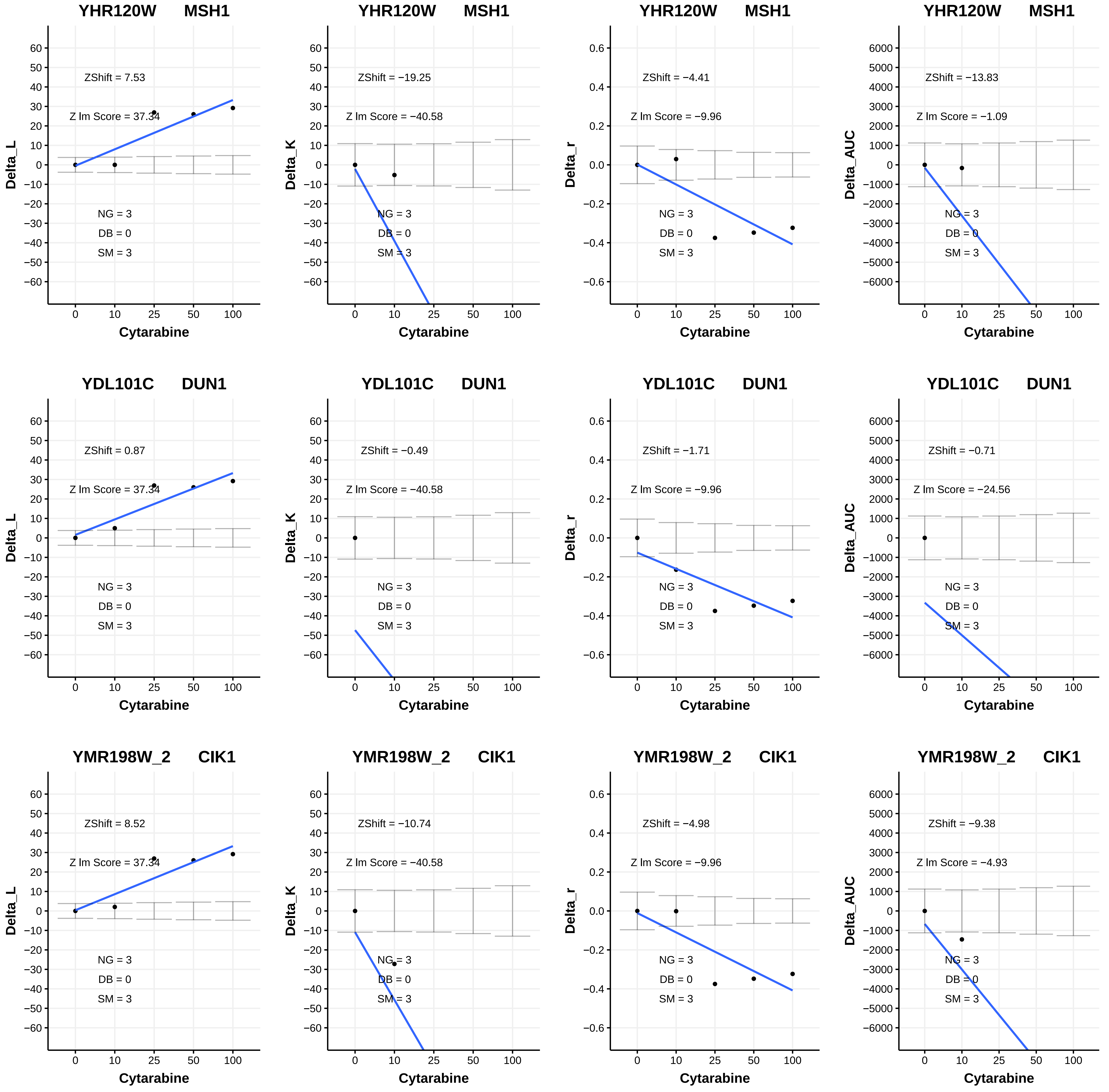
YHR120W MSH1
ZShift = 7.53
Z lm Score = 37.34
NG = 3
DB = 0
SM = 3
Delta_L
Cytarabine
YHR120W MSH1
ZShift = −19.25
Z lm Score = −40.58
NG = 3
DB = 0
SM = 3
Delta_K
Cytarabine
YHR120W MSH1
ZShift = −4.41
Z lm Score = −9.96
NG = 3
DB = 0
SM = 3
Delta_r
Cytarabine
YHR120W MSH1
ZShift = −13.83
Z lm Score = −1.09
NG = 3
DB = 0
SM = 3
Delta_AUC
Cytarabine
YDL101C DUN1
ZShift = 0.87
Z lm Score = 37.34
NG = 3
DB = 0
SM = 3
Delta_L
Cytarabine
YDL101C DUN1
ZShift = −0.49
Z lm Score = −40.58
NG = 3
DB = 0
SM = 3
Delta_K
Cytarabine
YDL101C DUN1
ZShift = −1.71
Z lm Score = −9.96
NG = 3
DB = 0
SM = 3
Delta_r
Cytarabine
YDL101C DUN1
ZShift = −0.71
Z lm Score = −24.56
NG = 3
DB = 0
SM = 3
Delta_AUC
Cytarabine
YMR198W_2 CIK1
ZShift = 8.52
Z lm Score = 37.34
NG = 3
DB = 0
SM = 3
Delta_L
Cytarabine
YMR198W_2 CIK1
ZShift = −10.74
Z lm Score = −40.58
NG = 3
DB = 0
SM = 3
Delta_K
Cytarabine
YMR198W_2 CIK1
ZShift = −4.98
Z lm Score = −9.96
NG = 3
DB = 0
SM = 3
Delta_r
Cytarabine
YMR198W_2 CIK1
ZShift = −9.38
Z lm Score = −4.93
NG = 3
DB = 0
SM = 3
Delta_AUC
Cytarabine

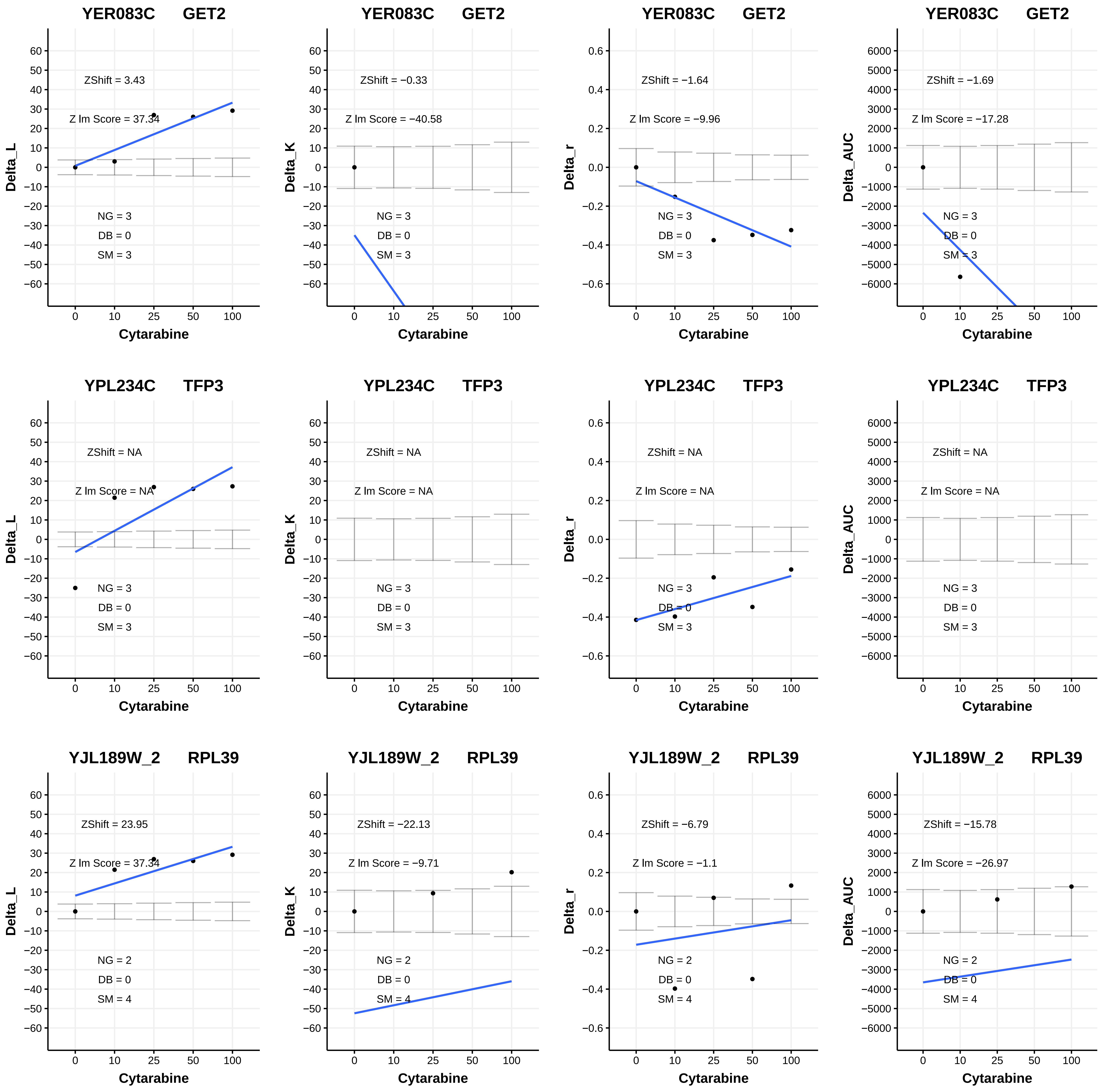

YER083C GET2
ZShift = 3.43
Z lm Score = 37.34
NG = 3
DB = 0
SM = 3
Delta_L
Cytarabine
YER083C GET2
ZShift = −0.33
Z lm Score = −40.58
NG = 3
DB = 0
SM = 3
Delta_K
Cytarabine
YER083C GET2
ZShift = −1.64
Z lm Score = −9.96
NG = 3
DB = 0
SM = 3
Delta_r
Cytarabine
YER083C GET2
ZShift = −1.69
Z lm Score = −17.28
NG = 3
DB = 0
SM = 3
Delta_AUC
Cytarabine
YPL234C TFP3
ZShift = NA
Z lm Score = NA
NG = 3
DB = 0
SM = 3
Delta_L
Cytarabine
YPL234C TFP3
ZShift = NA
Z lm Score = NA
NG = 3
DB = 0
SM = 3
Delta_K
Cytarabine
YPL234C TFP3
ZShift = NA
Z lm Score = NA
NG = 3
DB = 0
SM = 3
Delta_r
Cytarabine
YPL234C TFP3
ZShift = NA
Z lm Score = NA
NG = 3
DB = 0
SM = 3
Delta_AUC
Cytarabine
YJL189W_2 RPL39
ZShift = 23.95
Z lm Score = 37.34
NG = 2
DB = 0
SM = 4
Delta_L
Cytarabine
YJL189W_2 RPL39
ZShift = −22.13
Z lm Score = −9.71
NG = 2
DB = 0
SM = 4
Delta_K
Cytarabine
YJL189W_2 RPL39
ZShift = −6.79
Z lm Score = −1.1
NG = 2
DB = 0
SM = 4
Delta_r
Cytarabine
YJL189W_2 RPL39
ZShift = −15.78
Z lm Score = −26.97
NG = 2
DB = 0
SM = 4
Delta_AUC
Cytarabine

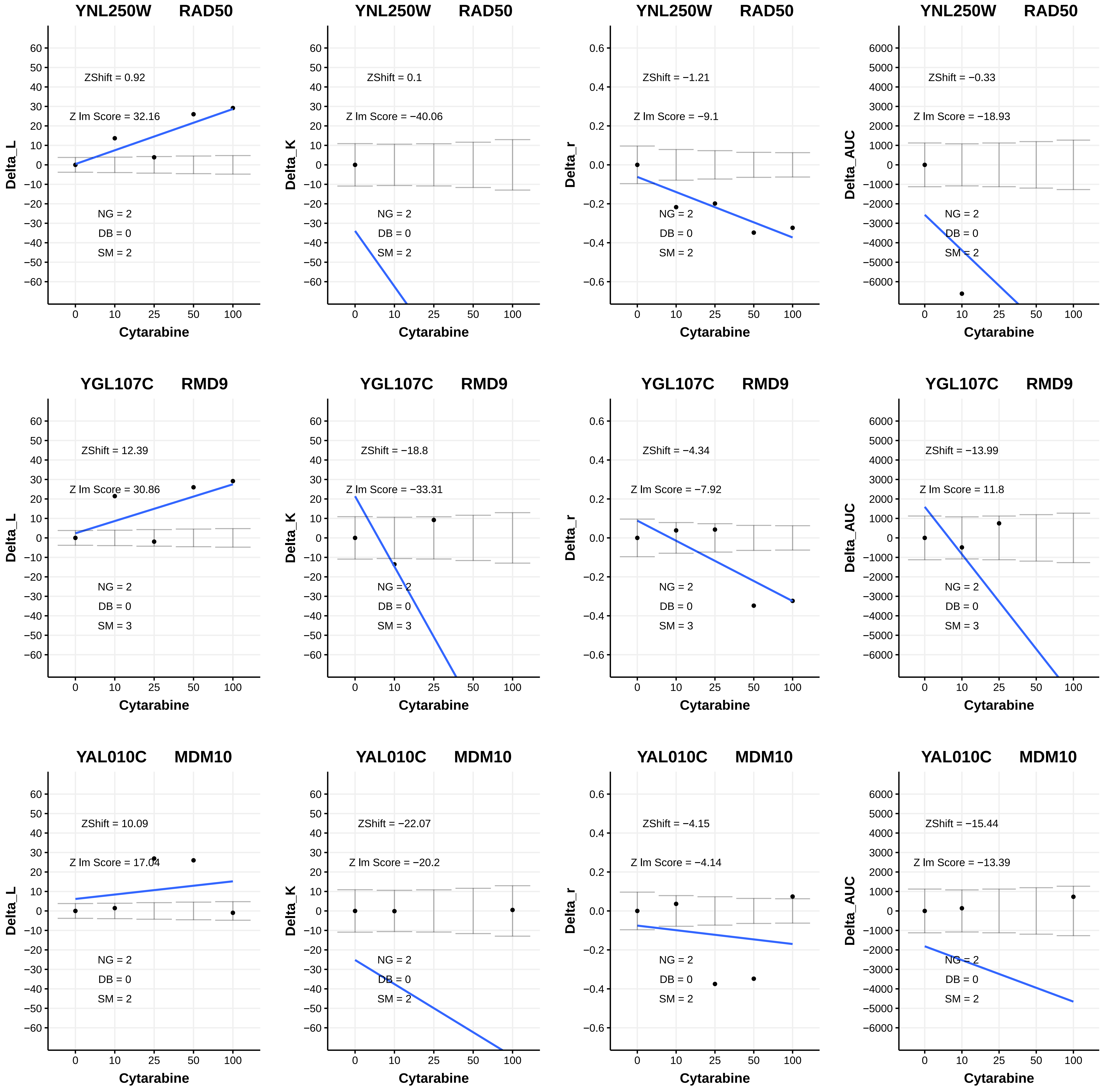

YNL250W RAD50
ZShift = 0.92
Z lm Score = 32.16
NG = 2
DB = 0
SM = 2
Delta_L
Cytarabine
YNL250W RAD50
ZShift = 0.1
Z lm Score = −40.06
NG = 2
DB = 0
SM = 2
Delta_K
Cytarabine
YNL250W RAD50
ZShift = −1.21
Z lm Score = −9.1
NG = 2
DB = 0
SM = 2
Delta_r
Cytarabine
YNL250W RAD50
ZShift = −0.33
Z lm Score = −18.93
NG = 2
DB = 0
SM = 2
Delta_AUC
Cytarabine
YGL107C RMD9
ZShift = 12.39
Z lm Score = 30.86
NG = 2
DB = 0
SM = 3
Delta_L
Cytarabine
YGL107C RMD9
ZShift = −18.8
Z lm Score = −33.31
NG = 2
DB = 0
SM = 3
Delta_K
Cytarabine
YGL107C RMD9
ZShift = −4.34
Z lm Score = −7.92
NG = 2
DB = 0
SM = 3
Delta_r
Cytarabine
YGL107C RMD9
ZShift = −13.99
Z lm Score = 11.8
NG = 2
DB = 0
SM = 3
Delta_AUC
Cytarabine
YAL010C MDM10
ZShift = 10.09
Z lm Score = 17.04
NG = 2
DB = 0
SM = 2
Delta_L
Cytarabine
YAL010C MDM10
ZShift = −22.07
Z lm Score = −20.2
NG = 2
DB = 0
SM = 2
Delta_K
Cytarabine
YAL010C MDM10
ZShift = −4.15
Z lm Score = −4.14
NG = 2
DB = 0
SM = 2
Delta_r
Cytarabine
YAL010C MDM10
ZShift = −15.44
Z lm Score = −13.39
NG = 2
DB = 0
SM = 2
Delta_AUC
Cytarabine
0 10 25 50 100

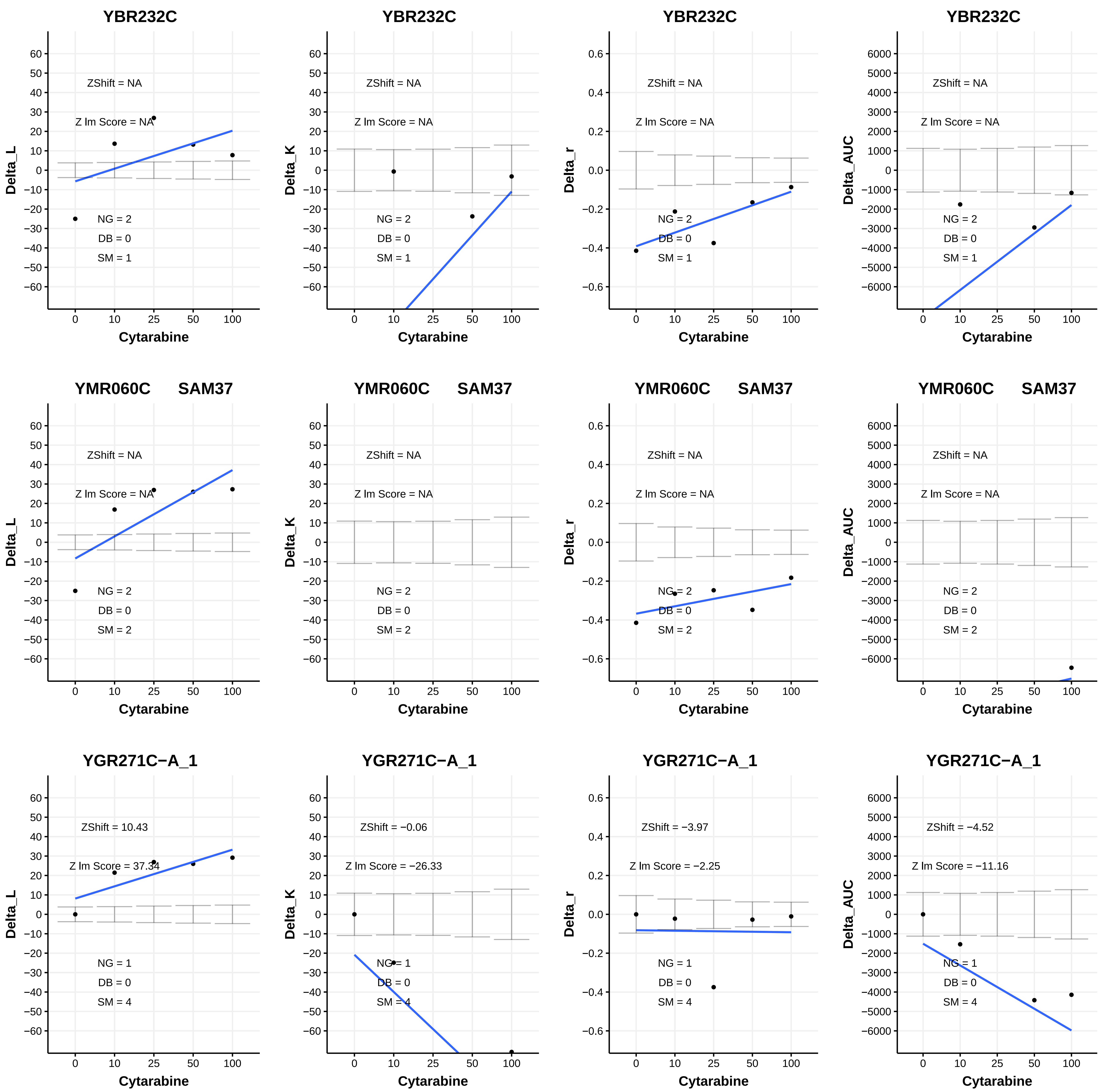
YBR232C
ZShift = NA
Z lm Score = NA
NG = 2
DB = 0
SM = 1
Delta_L
Delta_K
Delta_r
Delta_AUC
Cytarabine
0
10
25
50
100
YMR060C SAM37
ZShift = NA
Z lm Score = NA
NG = 2
DB = 0
SM = 2
YGR271C−A_1
ZShift = 10.43
Z lm Score = 37.34
ZShift = −0.06
Z lm Score = −26.33
ZShift = −3.97
Z lm Score = −2.25
ZShift = −4.52
Z lm Score = −11.16
NG = 1
DB = 0
SM = 4

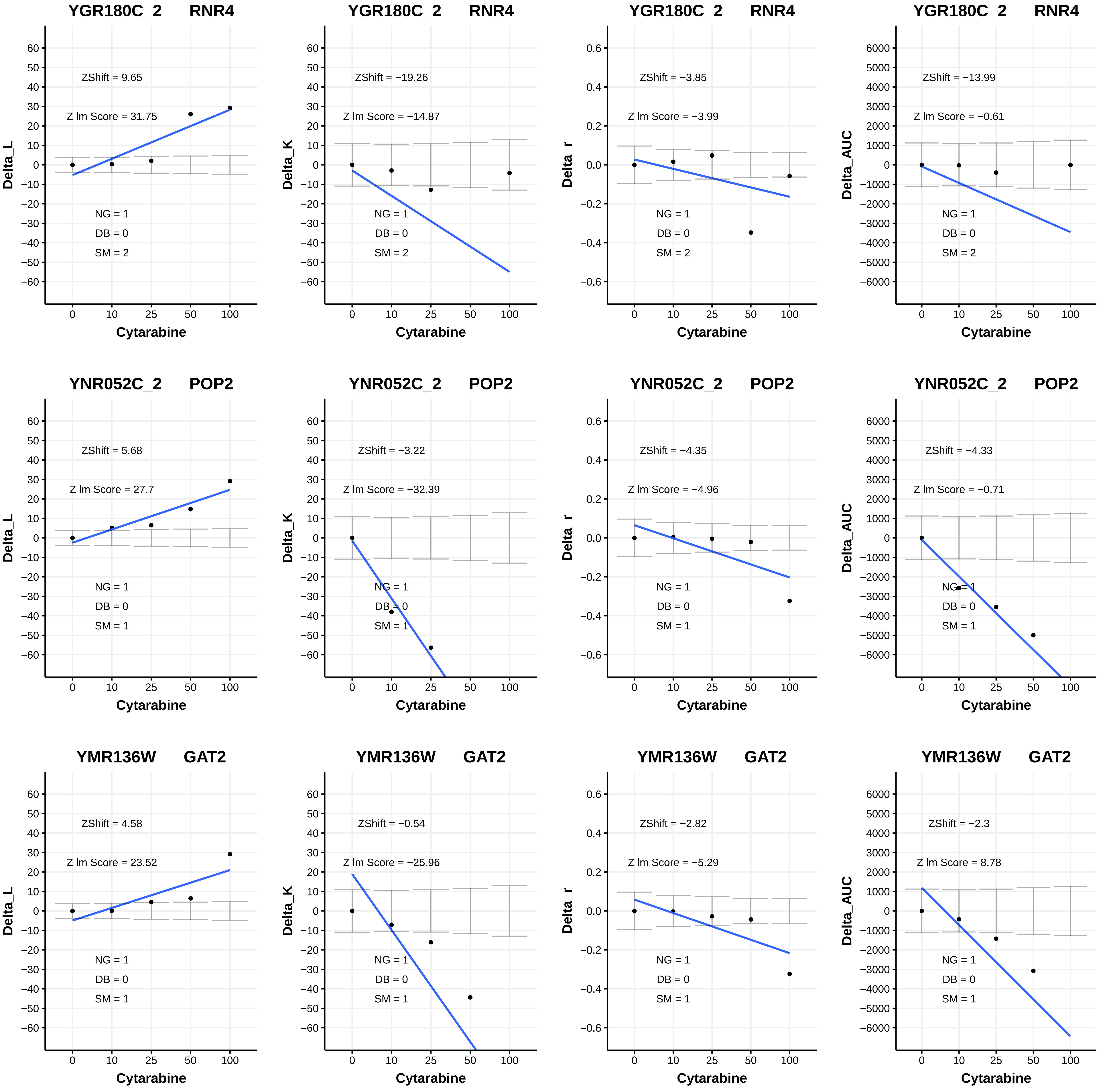

YGR180C_2 RNR4
ZShift = 9.65
Z lm Score = 31.75
NG = 1
DB = 0
SM = 2
Delta_L
Cytarabine
YGR180C_2 RNR4
ZShift = −19.26
Z lm Score = −14.87
NG = 1
DB = 0
SM = 2
Delta_K
Cytarabine
YGR180C_2 RNR4
ZShift = −3.85
Z lm Score = −3.99
NG = 1
DB = 0
SM = 2
Delta_r
Cytarabine
YGR180C_2 RNR4
ZShift = −13.99
Z lm Score = −0.61
NG = 1
DB = 0
SM = 2
Delta_AUC
Cytarabine
YNR052C_2 POP2
ZShift = 5.68
Z lm Score = 27.7
NG = 1
DB = 0
SM = 1
Delta_L
Cytarabine
YNR052C_2 POP2
ZShift = −3.22
Z lm Score = −32.39
NG = 1
DB = 0
SM = 1
Delta_K
Cytarabine
YNR052C_2 POP2
ZShift = −4.35
Z lm Score = −4.96
NG = 1
DB = 0
SM = 1
Delta_r
Cytarabine
YNR052C_2 POP2
ZShift = −4.33
Z lm Score = −0.71
NG = 1
DB = 0
SM = 1
Delta_AUC
Cytarabine
YMR136W GAT2
ZShift = 4.58
Z lm Score = 23.52
NG = 1
DB = 0
SM = 1
Delta_L
Cytarabine
YMR136W GAT2
ZShift = −0.54
Z lm Score = −25.96
NG = 1
DB = 0
SM = 1
Delta_K
Cytarabine
YMR136W GAT2
ZShift = −2.82
Z lm Score = −5.29
NG = 1
DB = 0
SM = 1
Delta_r
Cytarabine
YMR136W GAT2
ZShift = −2.3
Z lm Score = 8.78
NG = 1
DB = 0
SM = 1
Delta_AUC
Cytarabine

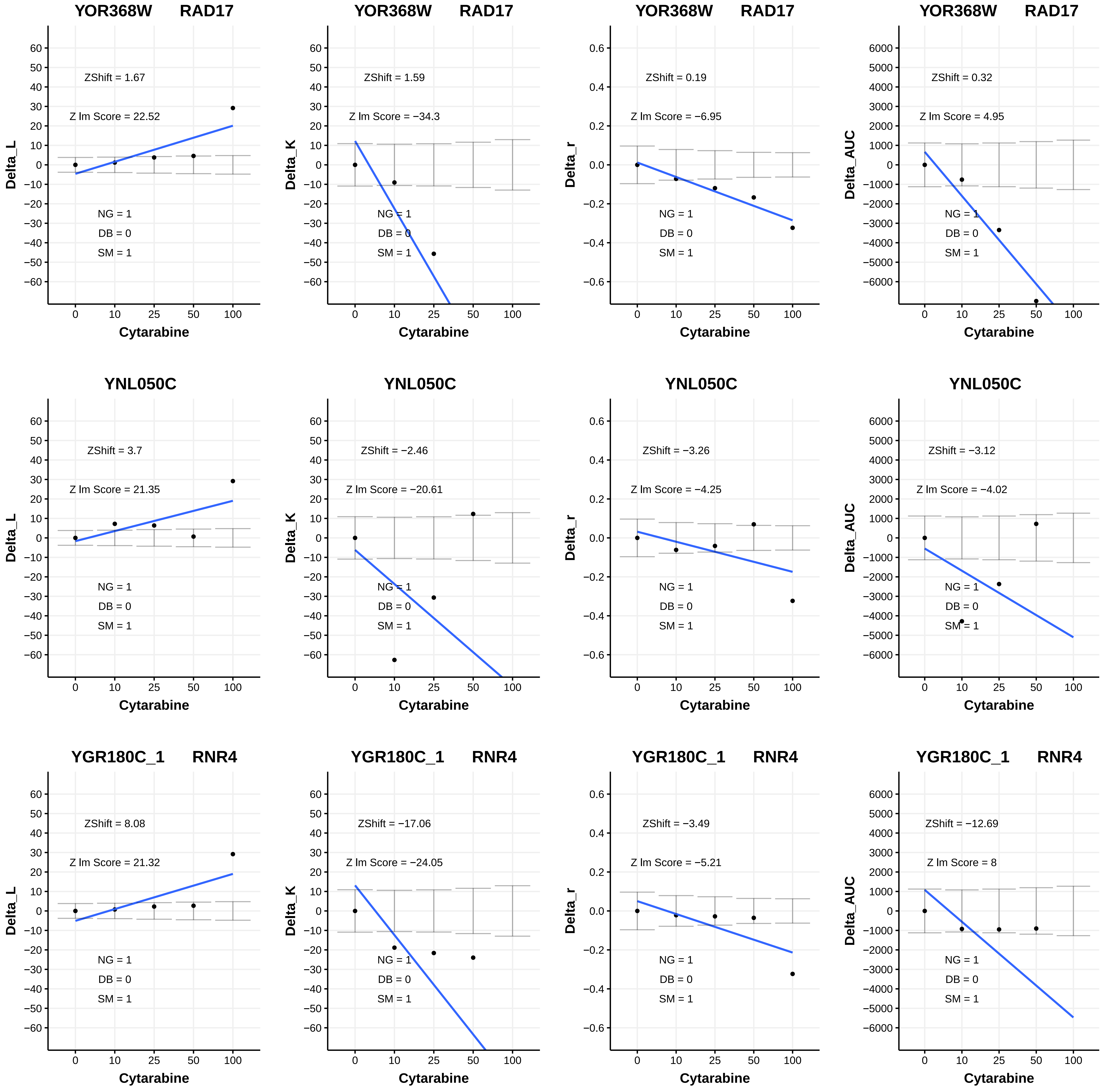

YOR368W RAD17
ZShift = 1.67
Z lm Score = 22.52
NG = 1
DB = 0
SM = 1
Delta_L
Cytarabine
YOR368W RAD17
ZShift = 1.59
Z lm Score = −34.3
NG = 1
DB = 0
SM = 1
Delta_K
Cytarabine
YOR368W RAD17
ZShift = 0.19
Z lm Score = −6.95
NG = 1
DB = 0
SM = 1
Delta_r
Cytarabine
YOR368W RAD17
ZShift = 0.32
Z lm Score = 4.95
NG = 1
DB = 0
SM = 1
Delta_AUC
Cytarabine
YNL050C
ZShift = 3.7
Z lm Score = 21.35
NG = 1
DB = 0
SM = 1
Delta_L
Cytarabine
YNL050C
ZShift = −2.46
Z lm Score = −20.61
NG = 1
DB = 0
SM = 1
Delta_K
Cytarabine
YNL050C
ZShift = −3.26
Z lm Score = −4.25
NG = 1
DB = 0
SM = 1
Delta_r
Cytarabine
YNL050C
ZShift = −3.12
Z lm Score = −4.02
NG = 1
DB = 0
SM = 1
Delta_AUC
Cytarabine
YGR180C_1 RNR4
ZShift = 8.08
Z lm Score = 21.32
NG = 1
DB = 0
SM = 1
Delta_L
Cytarabine
YGR180C_1 RNR4
ZShift = −17.06
Z lm Score = −24.05
NG = 1
DB = 0
SM = 1
Delta_K
Cytarabine
YGR180C_1 RNR4
ZShift = −3.49
Z lm Score = −5.21
NG = 1
DB = 0
SM = 1
Delta_r
Cytarabine
YGR180C_1 RNR4
ZShift = −12.69
Z lm Score = 8
NG = 1
DB = 0
SM = 1
Delta_AUC
Cytarabine

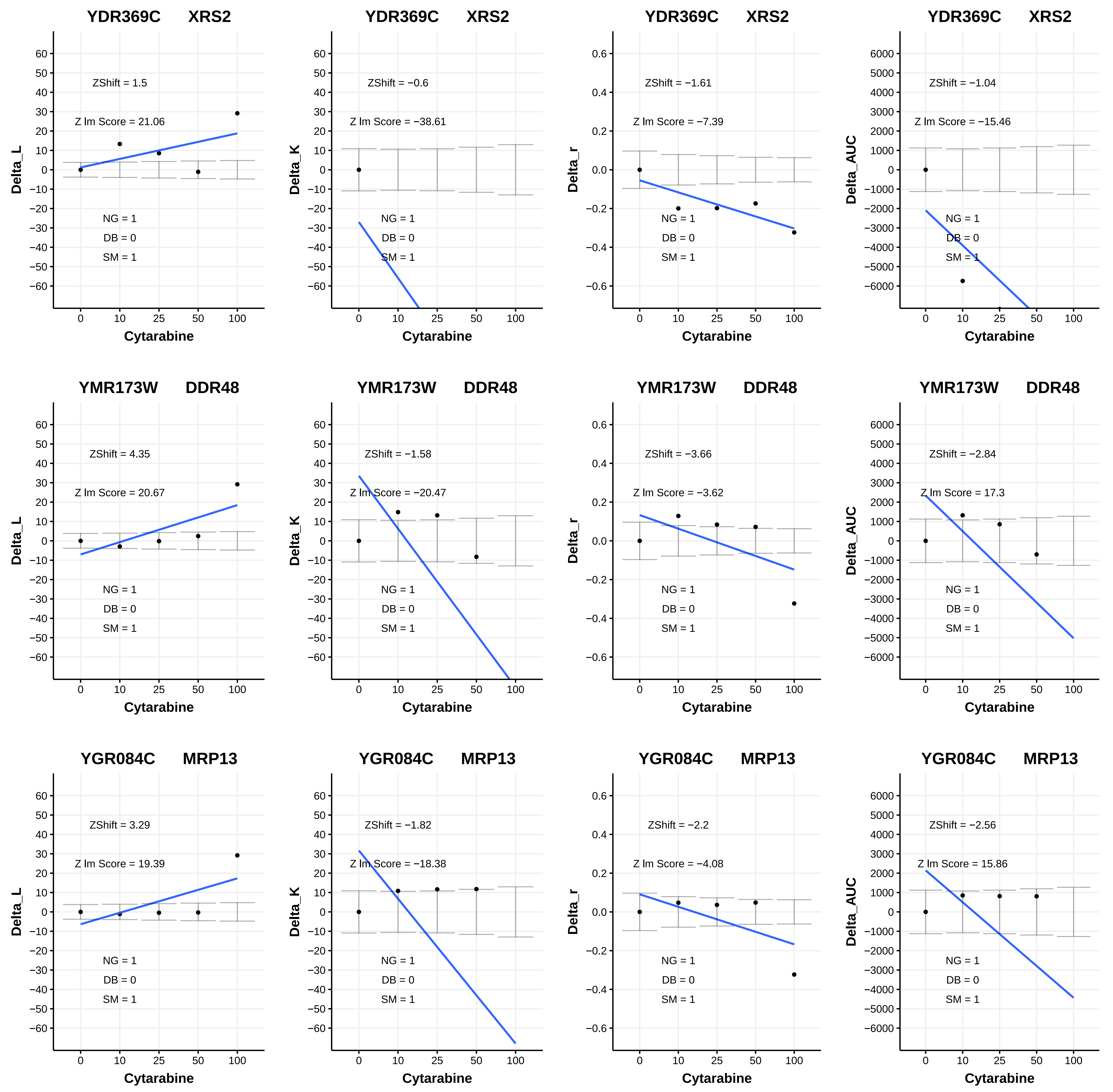

YDR369C XRS2
ZShift = 1.5
Z lm Score = 21.06
NG = 1
DB = 0
SM = 1
Delta_L
Cytarabine
YDR369C XRS2
ZShift = −0.6
Z lm Score = −38.61
NG = 1
DB = 0
SM = 1
Delta_K
Cytarabine
YDR369C XRS2
ZShift = −1.61
Z lm Score = −7.39
NG = 1
DB = 0
SM = 1
Delta_r
Cytarabine
YDR369C XRS2
ZShift = −1.04
Z lm Score = −15.46
NG = 1
DB = 0
SM = 1
Delta_AUC
Cytarabine
YMR173W DDR48
ZShift = 4.35
Z lm Score = 20.67
NG = 1
DB = 0
SM = 1
Delta_L
Cytarabine
YMR173W DDR48
ZShift = −1.58
Z lm Score = −20.47
NG = 1
DB = 0
SM = 1
Delta_K
Cytarabine
YMR173W DDR48
ZShift = −3.66
Z lm Score = −3.62
NG = 1
DB = 0
SM = 1
Delta_r
Cytarabine
YMR173W DDR48
ZShift = −2.84
Z lm Score = 17.3
NG = 1
DB = 0
SM = 1
Delta_AUC
Cytarabine
YGR084C MRP13
ZShift = 3.29
Z lm Score = 19.39
NG = 1
DB = 0
SM = 1
Delta_L
Cytarabine
YGR084C MRP13
ZShift = −1.82
Z lm Score = −18.38
NG = 1
DB = 0
SM = 1
Delta_K
Cytarabine
YGR084C MRP13
ZShift = −2.2
Z lm Score = −4.08
NG = 1
DB = 0
SM = 1
Delta_r
Cytarabine
YGR084C MRP13
ZShift = −2.56
Z lm Score = 15.86
NG = 1
DB = 0
SM = 1
Delta_AUC
Cytarabine

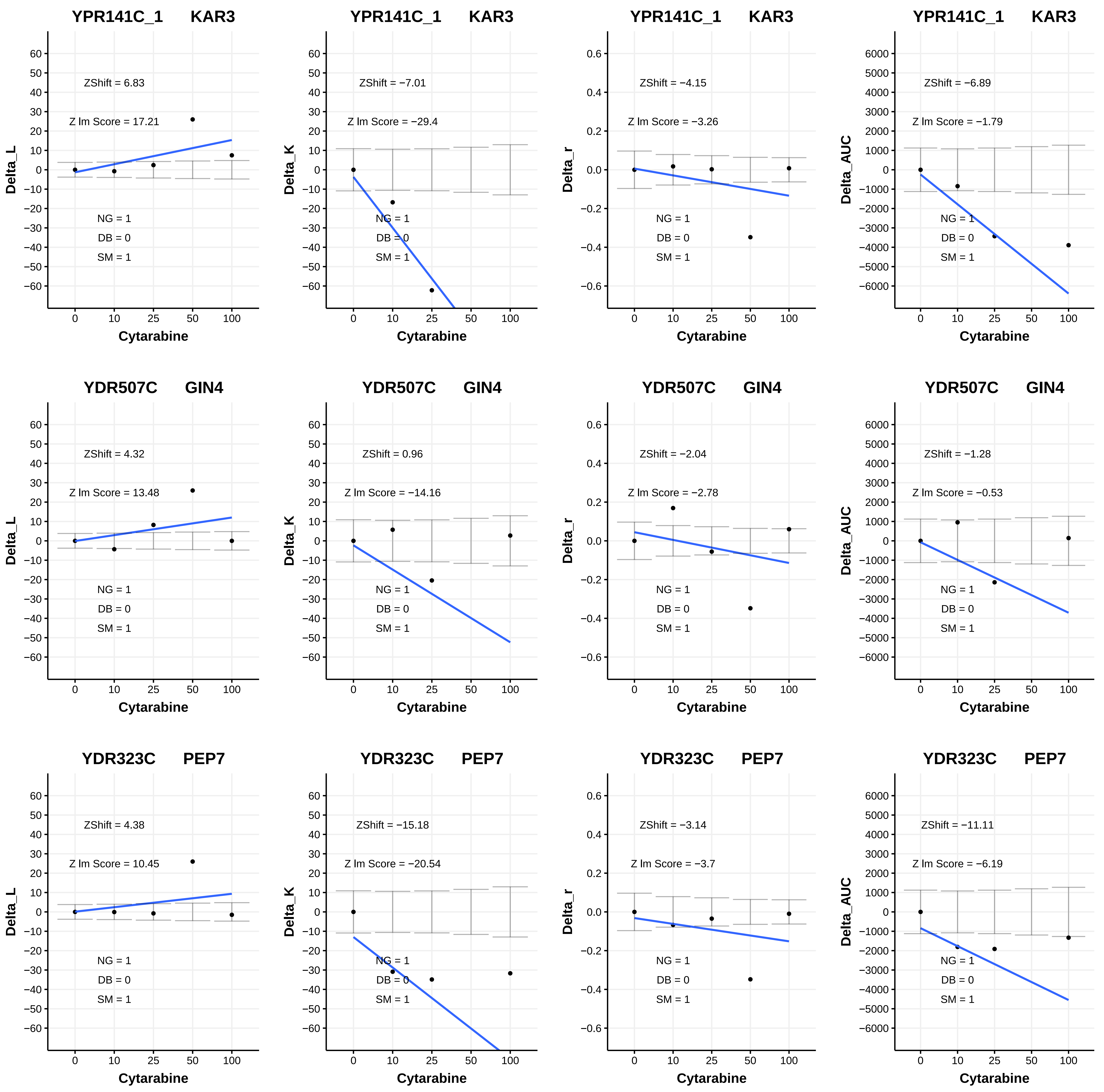

YPR141C_1 KAR3
ZShift = 6.83
Z lm Score = 17.21
NG = 1
DB = 0
SM = 1
Delta_L
Cytarabine
YPR141C_1 KAR3
ZShift = −7.01
Z lm Score = −29.4
NG = 1
DB = 0
SM = 1
Delta_K
Cytarabine
YPR141C_1 KAR3
ZShift = −4.15
Z lm Score = −3.26
NG = 1
DB = 0
SM = 1
Delta_r
Cytarabine
YPR141C_1 KAR3
ZShift = −6.89
Z lm Score = −1.79
NG = 1
DB = 0
SM = 1
Delta_AUC
Cytarabine
YDR507C GIN4
ZShift = 4.32
Z lm Score = 13.48
NG = 1
DB = 0
SM = 1
Delta_L
Cytarabine
YDR507C GIN4
ZShift = 0.96
Z lm Score = −14.16
NG = 1
DB = 0
SM = 1
Delta_K
Cytarabine
YDR507C GIN4
ZShift = −2.04
Z lm Score = −2.78
NG = 1
DB = 0
SM = 1
Delta_r
Cytarabine
YDR507C GIN4
ZShift = −1.28
Z lm Score = −0.53
NG = 1
DB = 0
SM = 1
Delta_AUC
Cytarabine
YDR323C PEP7
ZShift = 4.38
Z lm Score = 10.45
NG = 1
DB = 0
SM = 1
Delta_L
Cytarabine
YDR323C PEP7
ZShift = −15.18
Z lm Score = −20.54
NG = 1
DB = 0
SM = 1
Delta_K
Cytarabine
YDR323C PEP7
ZShift = −3.14
Z lm Score = −3.7
NG = 1
DB = 0
SM = 1
Delta_r
Cytarabine
YDR323C PEP7
ZShift = −11.11
Z lm Score = −6.19
NG = 1
DB = 0
SM = 1
Delta_AUC
Cytarabine

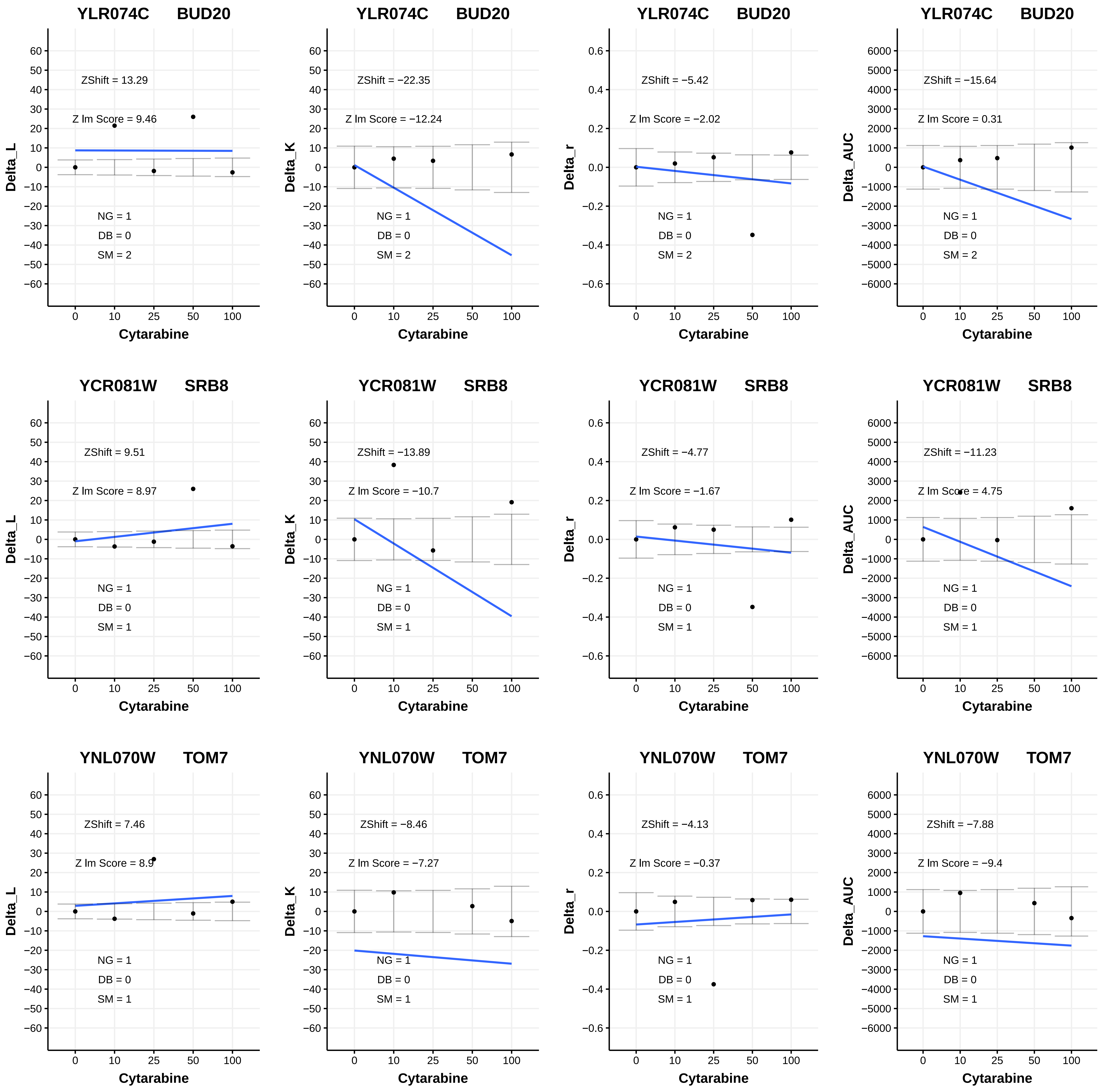

YLR074C BUD20
ZShift = 13.29
Z lm Score = 9.46
NG = 1
DB = 0
SM = 2
Delta_L
ZShift = −22.35
Z lm Score = −12.24
NG = 1
DB = 0
SM = 2
Delta_K
ZShift = −5.42
Z lm Score = −2.02
NG = 1
DB = 0
SM = 2
Delta_r
ZShift = −15.64
Z lm Score = 0.31
NG = 1
DB = 0
SM = 2
Delta_AUC
YCR081W SRB8
ZShift = 9.51
Z lm Score = 8.97
NG = 1
DB = 0
SM = 1
ZShift = −13.89
Z lm Score = −10.7
NG = 1
DB = 0
SM = 1
ZShift = −4.77
Z lm Score = −1.67
NG = 1
DB = 0
SM = 1
ZShift = −11.23
Z lm Score = 4.75
NG = 1
DB = 0
SM = 1
YNL070W TOM7
ZShift = 7.46
Z lm Score = 8.9
NG = 1
DB = 0
SM = 1
ZShift = −8.46
Z lm Score = −7.27
NG = 1
DB = 0
SM = 1
ZShift = −4.13
Z lm Score = −0.37
NG = 1
DB = 0
SM = 1
ZShift = −7.88
Z lm Score = −9.4
NG = 1
DB = 0
SM = 1
Cytarabine
0
10
25
50
100

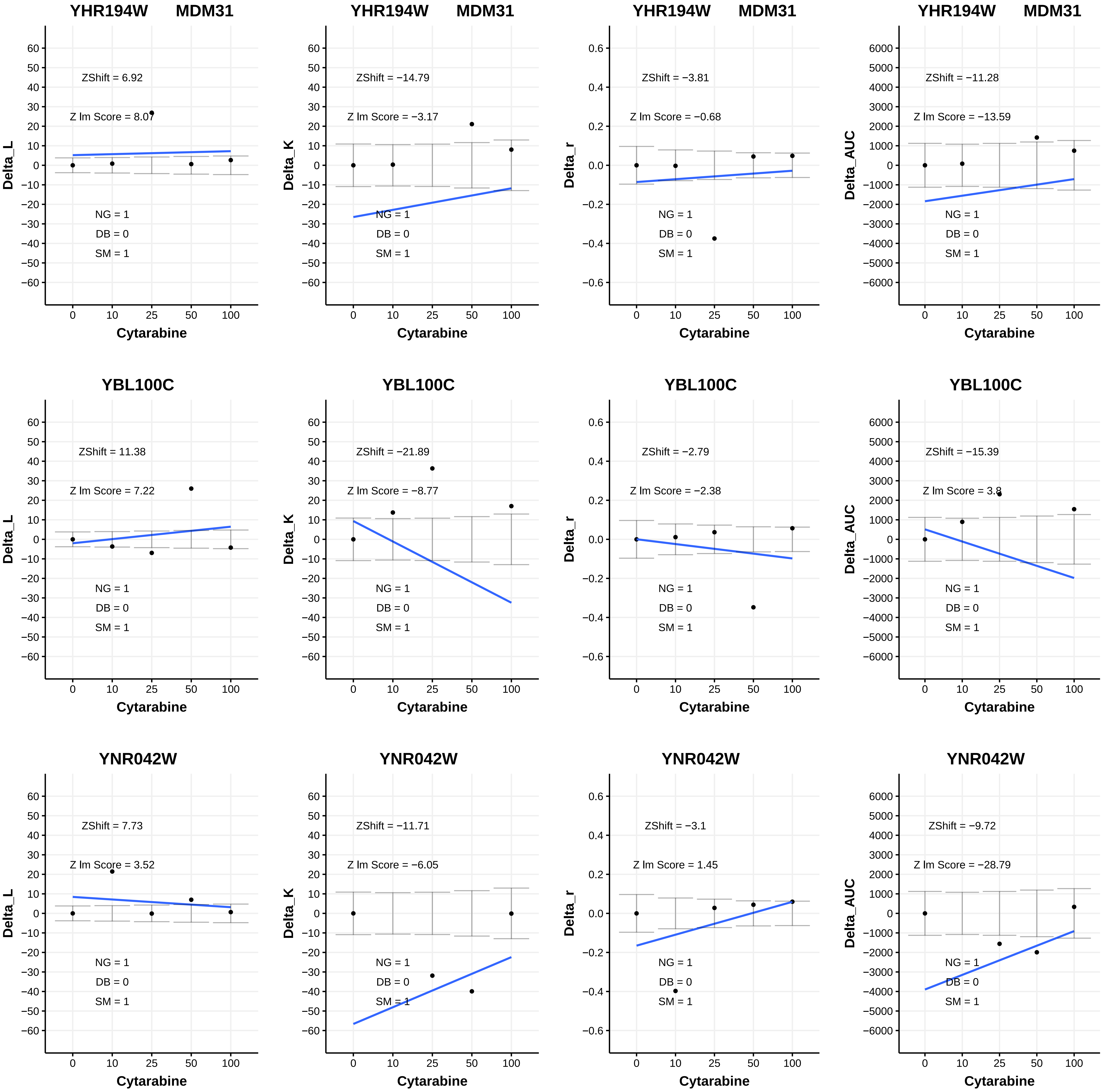
YHR194W MDM31
ZShift = 6.92
Z lm Score = 8.07
NG = 1
DB = 0
SM = 1
Delta_L
Cytarabine
YHR194W MDM31
ZShift = −14.79
Z lm Score = −3.17
NG = 1
DB = 0
SM = 1
Delta_K
Cytarabine
YHR194W MDM31
ZShift = −3.81
Z lm Score = −0.68
NG = 1
DB = 0
SM = 1
Delta_r
Cytarabine
YHR194W MDM31
ZShift = −11.28
Z lm Score = −13.59
NG = 1
DB = 0
SM = 1
Delta_AUC
Cytarabine
YBL100C
ZShift = 11.38
Z lm Score = 7.22
NG = 1
DB = 0
SM = 1
Delta_L
Cytarabine
YBL100C
ZShift = −21.89
Z lm Score = −8.77
NG = 1
DB = 0
SM = 1
Delta_K
Cytarabine
YBL100C
ZShift = −2.79
Z lm Score = −2.38
NG = 1
DB = 0
SM = 1
Delta_r
Cytarabine
YBL100C
ZShift = −15.39
Z lm Score = 3.8
NG = 1
DB = 0
SM = 1
Delta_AUC
Cytarabine
YNR042W
ZShift = 7.73
Z lm Score = 3.52
NG = 1
DB = 0
SM = 1
Delta_L
Cytarabine
YNR042W
ZShift = −11.71
Z lm Score = −6.05
NG = 1
DB = 0
SM = 1
Delta_K
Cytarabine
YNR042W
ZShift = −3.1
Z lm Score = 1.45
NG = 1
DB = 0
SM = 1
Delta_r
Cytarabine
YNR042W
ZShift = −9.72
Z lm Score = −28.79
NG = 1
DB = 0
SM = 1
Delta_AUC
Cytarabine

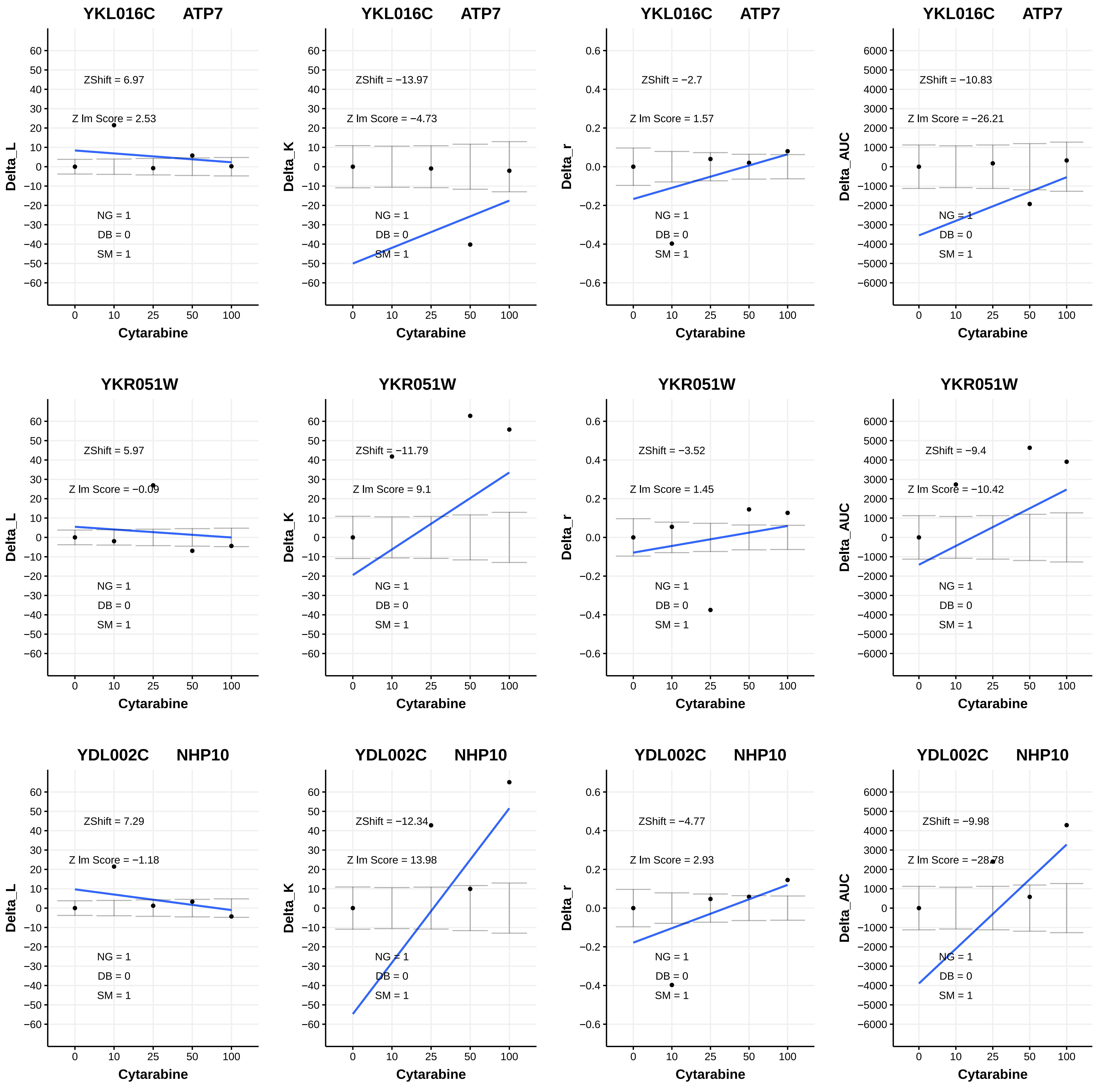
YKL016C ATP7
ZShift = 6.97
Z lm Score = 2.53
NG = 1
DB = 0
SM = 1
Delta_L
Cytarabine
YKL016C ATP7
ZShift = −13.97
Z lm Score = −4.73
NG = 1
DB = 0
SM = 1
Delta_K
Cytarabine
YKL016C ATP7
ZShift = −2.7
Z lm Score = 1.57
NG = 1
DB = 0
SM = 1
Delta_r
Cytarabine
YKL016C ATP7
ZShift = −10.83
Z lm Score = −26.21
NG = 1
DB = 0
SM = 1
Delta_AUC
Cytarabine
YKR051W
ZShift = 5.97
Z lm Score = −0.09
NG = 1
DB = 0
SM = 1
Delta_L
Cytarabine
YKR051W
ZShift = −11.79
Z lm Score = 9.1
NG = 1
DB = 0
SM = 1
Delta_K
Cytarabine
YKR051W
ZShift = −3.52
Z lm Score = 1.45
NG = 1
DB = 0
SM = 1
Delta_r
Cytarabine
YKR051W
ZShift = −9.4
Z lm Score = −10.42
NG = 1
DB = 0
SM = 1
Delta_AUC
Cytarabine
YDL002C NHP10
ZShift = 7.29
Z lm Score = −1.18
NG = 1
DB = 0
SM = 1
Delta_L
Cytarabine
YDL002C NHP10
ZShift = −12.34
Z lm Score = 13.98
NG = 1
DB = 0
SM = 1
Delta_K
Cytarabine
YDL002C NHP10
ZShift = −4.77
Z lm Score = 2.93
NG = 1
DB = 0
SM = 1
Delta_r
Cytarabine
YDL002C NHP10
ZShift = −9.98
Z lm Score = −28.78
NG = 1
DB = 0
SM = 1
Delta_AUC
Cytarabine

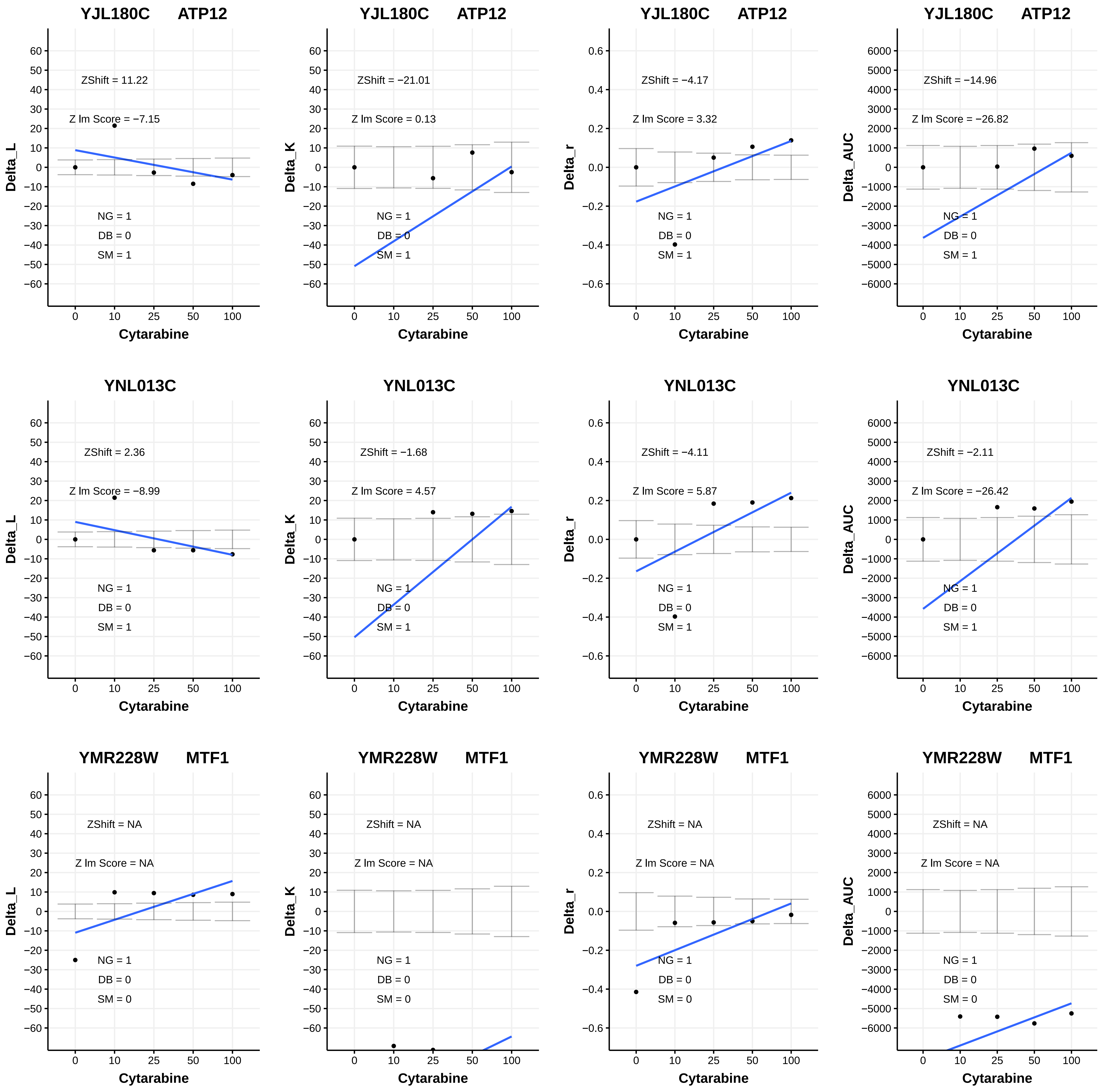

YJL180C ATP12
ZShift = 11.22
Z lm Score = −7.15
NG = 1
DB = 0
SM = 1
Delta_L
Cytarabine
YJL180C ATP12
ZShift = −21.01
Z lm Score = 0.13
NG = 1
DB = 0
SM = 1
Delta_K
Cytarabine
YJL180C ATP12
ZShift = −4.17
Z lm Score = 3.32
NG = 1
DB = 0
SM = 1
Delta_r
Cytarabine
YJL180C ATP12
ZShift = −14.96
Z lm Score = −26.82
NG = 1
DB = 0
SM = 1
Delta_AUC
Cytarabine
YNL013C
ZShift = 2.36
Z lm Score = −8.99
NG = 1
DB = 0
SM = 1
Delta_L
Cytarabine
YNL013C
ZShift = −1.68
Z lm Score = 4.57
NG = 1
DB = 0
SM = 1
Delta_K
Cytarabine
YNL013C
ZShift = −4.11
Z lm Score = 5.87
NG = 1
DB = 0
SM = 1
Delta_r
Cytarabine
YNL013C
ZShift = −2.11
Z lm Score = −26.42
NG = 1
DB = 0
SM = 1
Delta_AUC
Cytarabine
YMR228W MTF1
ZShift = NA
Z lm Score = NA
NG = 1
DB = 0
SM = 0
Delta_L
Cytarabine
YMR228W MTF1
ZShift = NA
Z lm Score = NA
NG = 1
DB = 0
SM = 0
Delta_K
Cytarabine
YMR228W MTF1
ZShift = NA
Z lm Score = NA
NG = 1
DB = 0
SM = 0
Delta_r
Cytarabine
YMR228W MTF1
ZShift = NA
Z lm Score = NA
NG = 1
DB = 0
SM = 0
Delta_AUC
Cytarabine

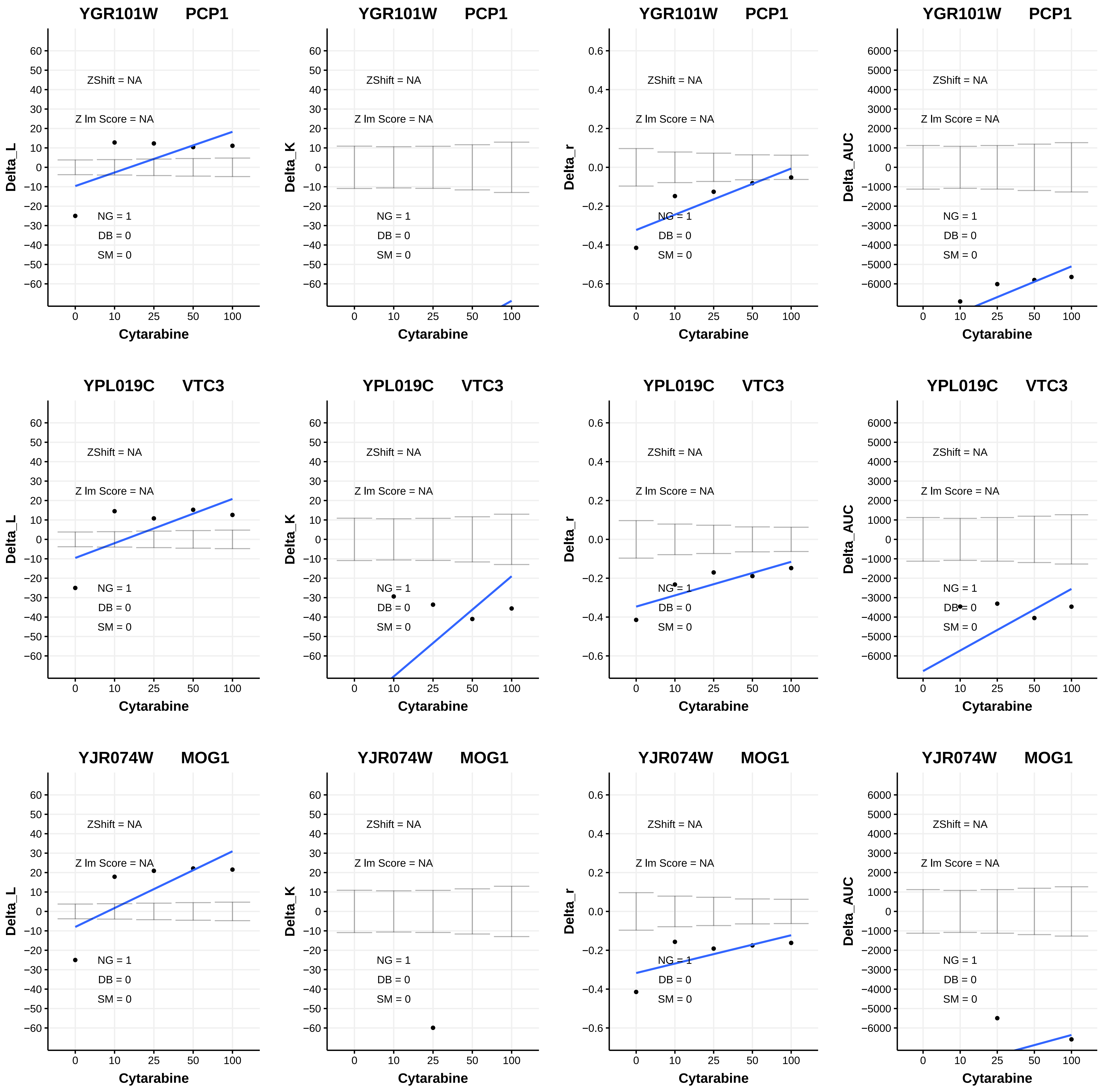

YGR101W PCP1
ZShift = NA
Z lm Score = NA
NG = 1
DB = 0
SM = 0
Delta_L
Delta_K
Delta_r
Delta_AUC
Cytarabine
0 10 25 50 100
YPL019C VTC3
YJR074W MOG1

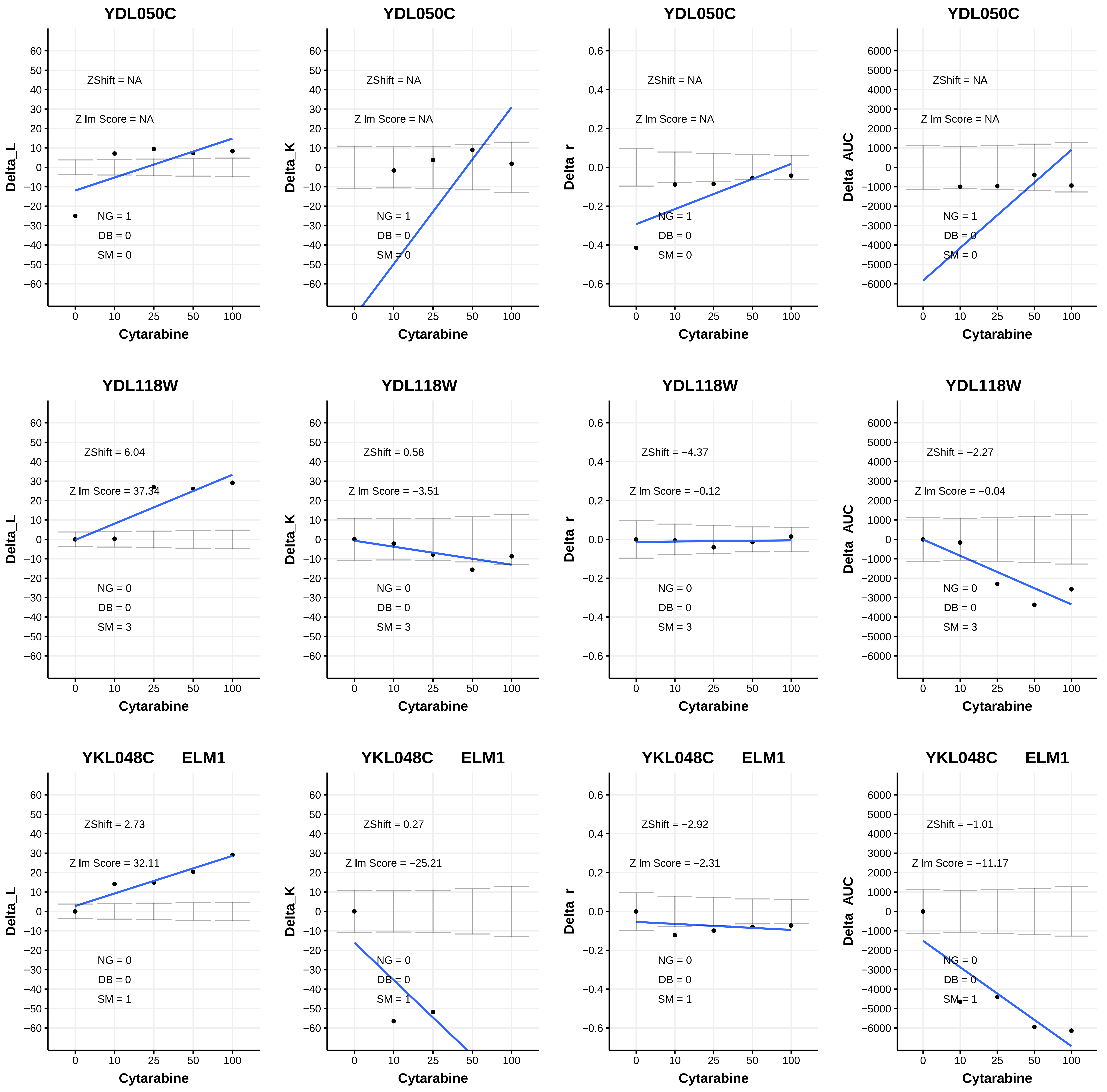
YDL050C
ZShift = NA
Z lm Score = NA
NG = 1
DB = 0
SM = 0
Delta_L
Delta_K
Delta_r
Delta_AUC
Cytarabine
YDL118W
ZShift = 6.04
Z lm Score = 37.34
ZShift = 0.58
Z lm Score = −3.51
ZShift = −4.37
Z lm Score = −0.12
ZShift = −2.27
Z lm Score = −0.04
NG = 0
DB = 0
SM = 3
YKL048C ELM1
ZShift = 2.73
Z lm Score = 32.11
ZShift = 0.27
Z lm Score = −25.21
ZShift = −2.92
Z lm Score = −2.31
ZShift = −1.01
Z lm Score = −11.17
NG = 0
DB = 0
SM = 1

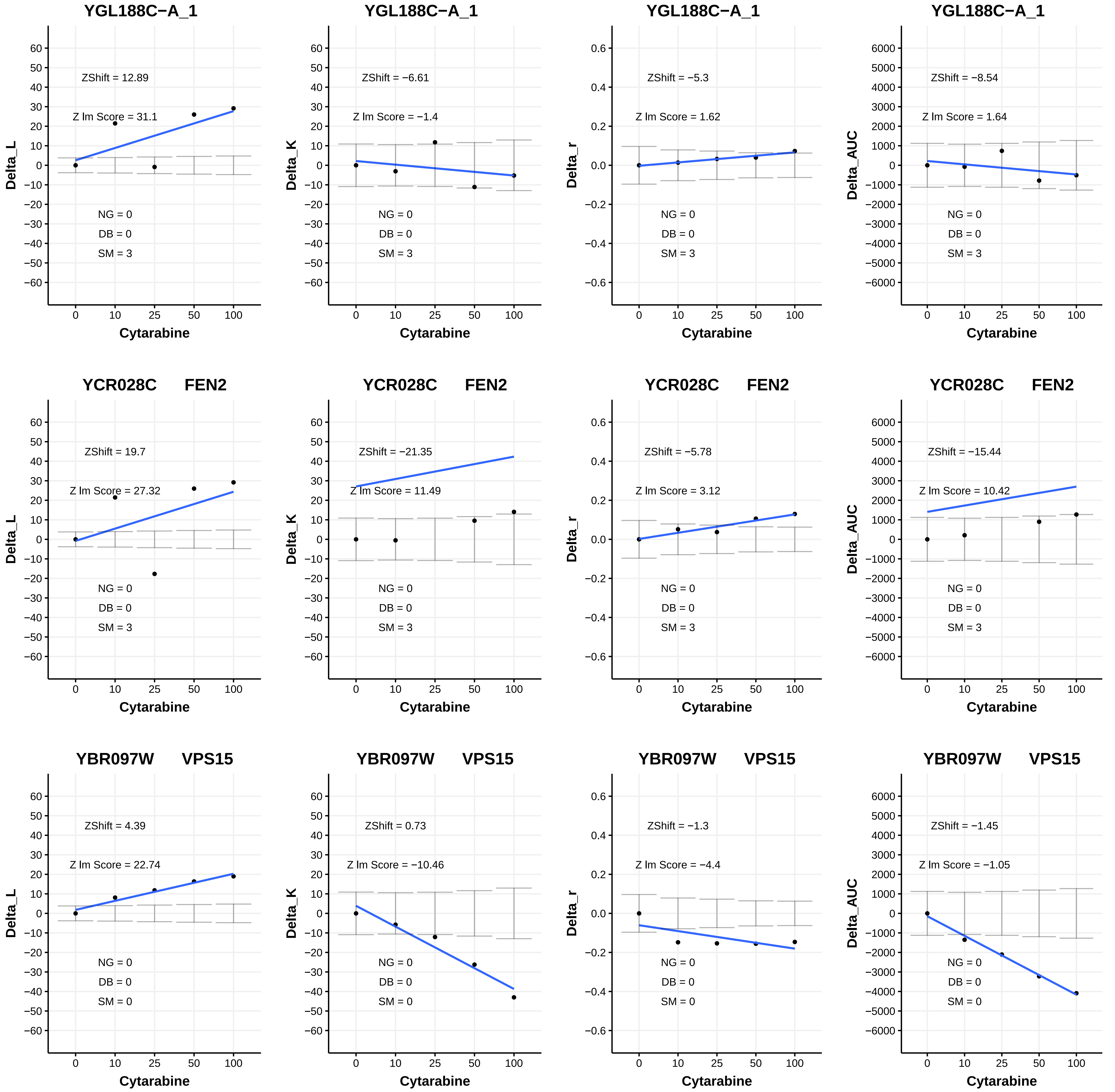
YGL188C−A_1
ZShift = 12.89
Z lm Score = 31.1
NG = 0
DB = 0
SM = 3
Delta_L
Cytarabine
YGL188C−A_1
ZShift = −6.61
Z lm Score = −1.4
NG = 0
DB = 0
SM = 3
Delta_K
Cytarabine
YGL188C−A_1
ZShift = −5.3
Z lm Score = 1.62
NG = 0
DB = 0
SM = 3
Delta_r
Cytarabine
YGL188C−A_1
ZShift = −8.54
Z lm Score = 1.64
NG = 0
DB = 0
SM = 3
Delta_AUC
Cytarabine
YCR028C FEN2
ZShift = 19.7
Z lm Score = 27.32
NG = 0
DB = 0
SM = 3
Delta_L
Cytarabine
YCR028C FEN2
ZShift = −21.35
Z lm Score = 11.49
NG = 0
DB = 0
SM = 3
Delta_K
Cytarabine
YCR028C FEN2
ZShift = −5.78
Z lm Score = 3.12
NG = 0
DB = 0
SM = 3
Delta_r
Cytarabine
YCR028C FEN2
ZShift = −15.44
Z lm Score = 10.42
NG = 0
DB = 0
SM = 3
Delta_AUC
Cytarabine
YBR097W VPS15
ZShift = 4.39
Z lm Score = 22.74
NG = 0
DB = 0
SM = 0
Delta_L
Cytarabine
YBR097W VPS15
ZShift = 0.73
Z lm Score = −10.46
NG = 0
DB = 0
SM = 0
Delta_K
Cytarabine
YBR097W VPS15
ZShift = −1.3
Z lm Score = −4.4
NG = 0
DB = 0
SM = 0
Delta_r
Cytarabine
YBR097W VPS15
ZShift = −1.45
Z lm Score = −1.05
NG = 0
DB = 0
SM = 0
Delta_AUC
Cytarabine

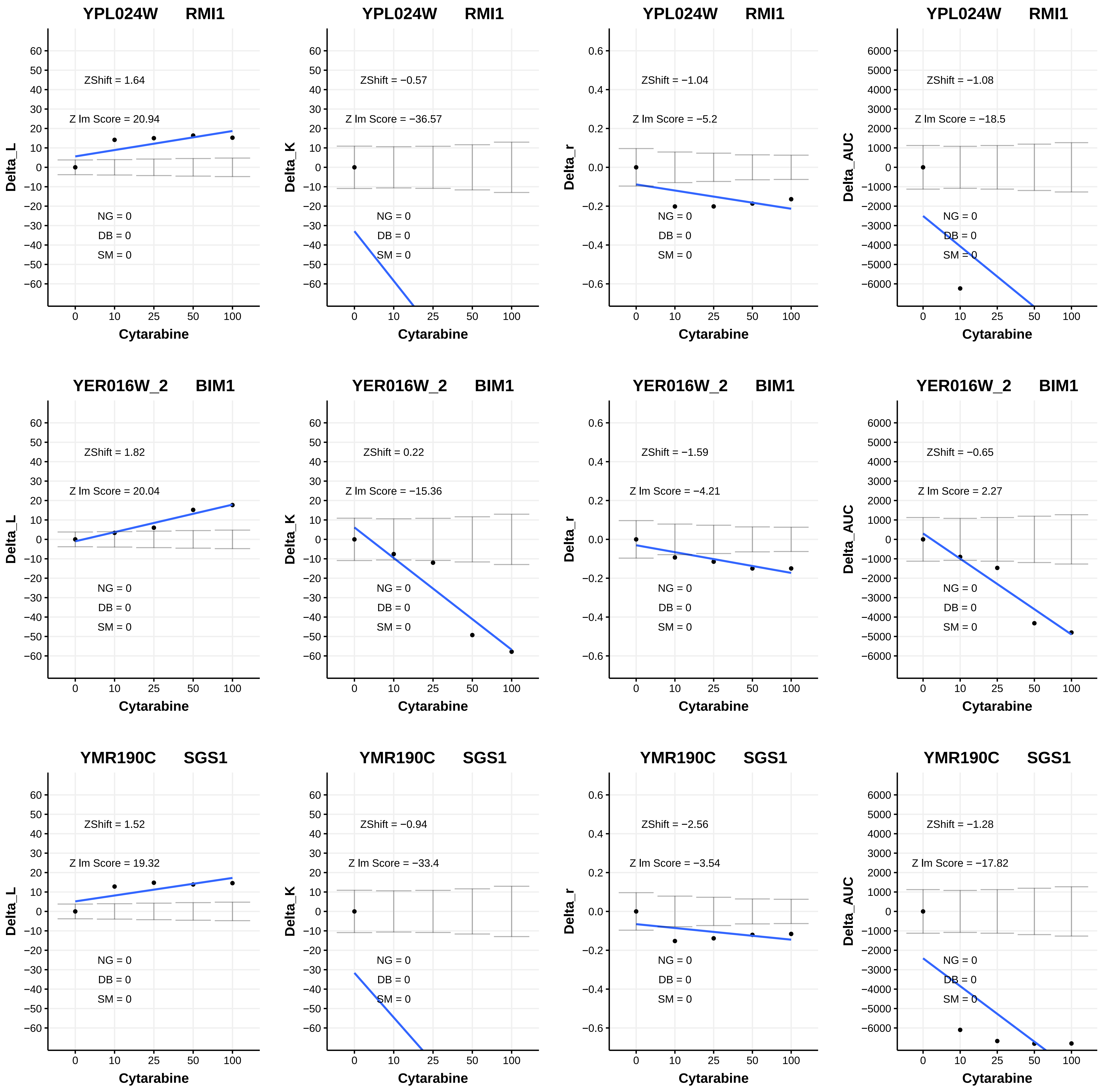
YPL024W RMI1
ZShift = 1.64
Z lm Score = 20.94
NG = 0
DB = 0
SM = 0
Delta_L
Cytarabine
YPL024W RMI1
ZShift = −0.57
Z lm Score = −36.57
NG = 0
DB = 0
SM = 0
Delta_K
Cytarabine
YPL024W RMI1
ZShift = −1.04
Z lm Score = −5.2
NG = 0
DB = 0
SM = 0
Delta_r
Cytarabine
YPL024W RMI1
ZShift = −1.08
Z lm Score = −18.5
NG = 0
DB = 0
SM = 0
Delta_AUC
Cytarabine
YER016W_2 BIM1
ZShift = 1.82
Z lm Score = 20.04
NG = 0
DB = 0
SM = 0
Delta_L
Cytarabine
YER016W_2 BIM1
ZShift = 0.22
Z lm Score = −15.36
NG = 0
DB = 0
SM = 0
Delta_K
Cytarabine
YER016W_2 BIM1
ZShift = −1.59
Z lm Score = −4.21
NG = 0
DB = 0
SM = 0
Delta_r
Cytarabine
YER016W_2 BIM1
ZShift = −0.65
Z lm Score = 2.27
NG = 0
DB = 0
SM = 0
Delta_AUC
Cytarabine
YMR190C SGS1
ZShift = 1.52
Z lm Score = 19.32
NG = 0
DB = 0
SM = 0
Delta_L
Cytarabine
YMR190C SGS1
ZShift = −0.94
Z lm Score = −33.4
NG = 0
DB = 0
SM = 0
Delta_K
Cytarabine
YMR190C SGS1
ZShift = −2.56
Z lm Score = −3.54
NG = 0
DB = 0
SM = 0
Delta_r
Cytarabine
YMR190C SGS1
ZShift = −1.28
Z lm Score = −17.82
NG = 0
DB = 0
SM = 0
Delta_AUC
Cytarabine

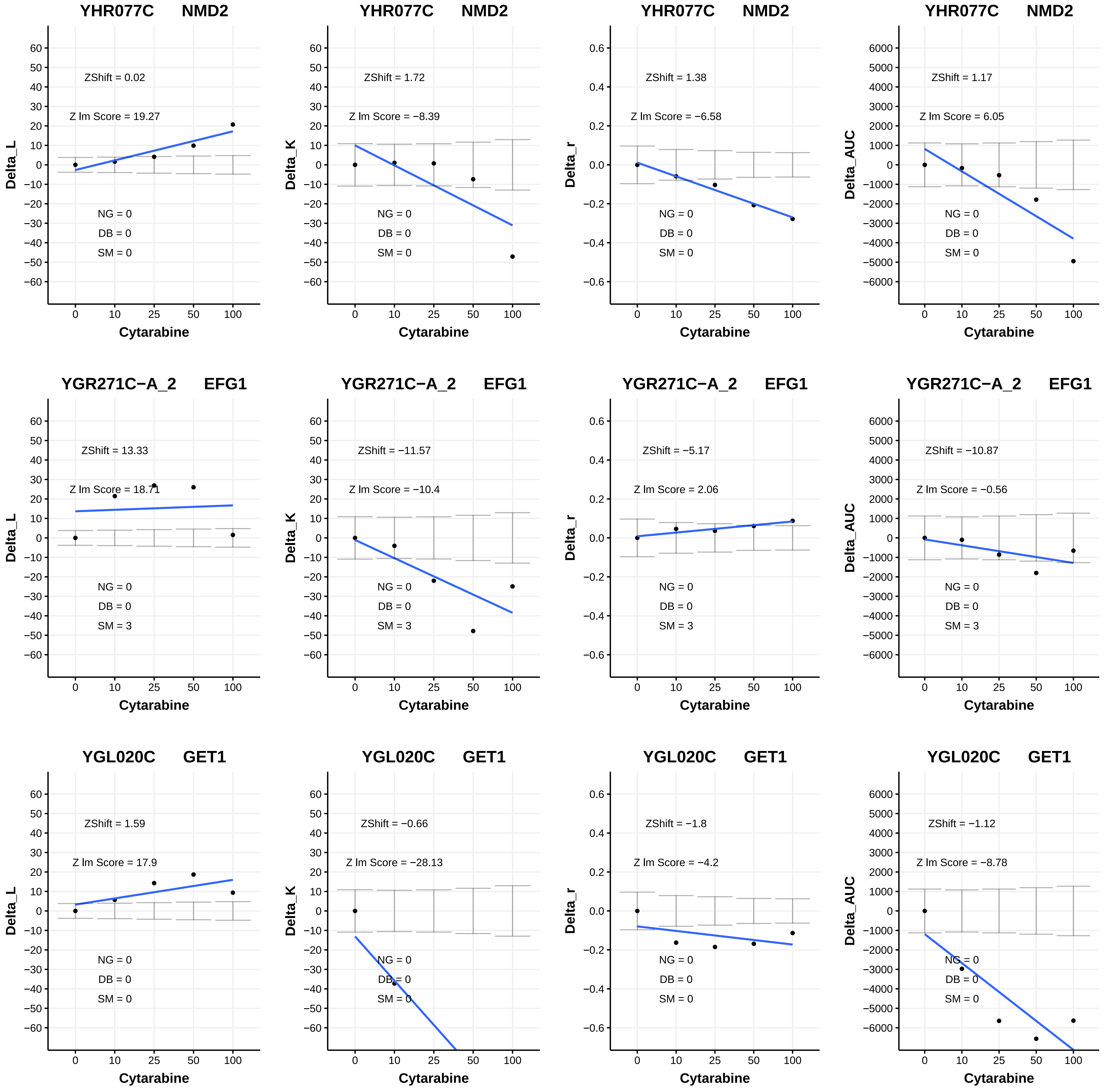

YHR077C NMD2
ZShift = 0.02
Z lm Score = 19.27
NG = 0
DB = 0
SM = 0
Delta_L
Cytarabine
YHR077C NMD2
ZShift = 1.72
Z lm Score = −8.39
NG = 0
DB = 0
SM = 0
Delta_K
Cytarabine
YHR077C NMD2
ZShift = 1.38
Z lm Score = −6.58
NG = 0
DB = 0
SM = 0
Delta_r
Cytarabine
YHR077C NMD2
ZShift = 1.17
Z lm Score = 6.05
NG = 0
DB = 0
SM = 0
Delta_AUC
Cytarabine
YGR271C−A_2 EFG1
ZShift = 13.33
Z lm Score = 18.71
NG = 0
DB = 0
SM = 3
Delta_L
Cytarabine
YGR271C−A_2 EFG1
ZShift = −11.57
Z lm Score = −10.4
NG = 0
DB = 0
SM = 3
Delta_K
Cytarabine
YGR271C−A_2 EFG1
ZShift = −5.17
Z lm Score = 2.06
NG = 0
DB = 0
SM = 3
Delta_r
Cytarabine
YGR271C−A_2 EFG1
ZShift = −10.87
Z lm Score = −0.56
NG = 0
DB = 0
SM = 3
Delta_AUC
Cytarabine
YGL020C GET1
ZShift = 1.59
Z lm Score = 17.9
NG = 0
DB = 0
SM = 0
Delta_L
Cytarabine
YGL020C GET1
ZShift = −0.66
Z lm Score = −28.13
NG = 0
DB = 0
SM = 0
Delta_K
Cytarabine
YGL020C GET1
ZShift = −1.8
Z lm Score = −4.2
NG = 0
DB = 0
SM = 0
Delta_r
Cytarabine
YGL020C GET1
ZShift = −1.12
Z lm Score = −8.78
NG = 0
DB = 0
SM = 0
Delta_AUC
Cytarabine

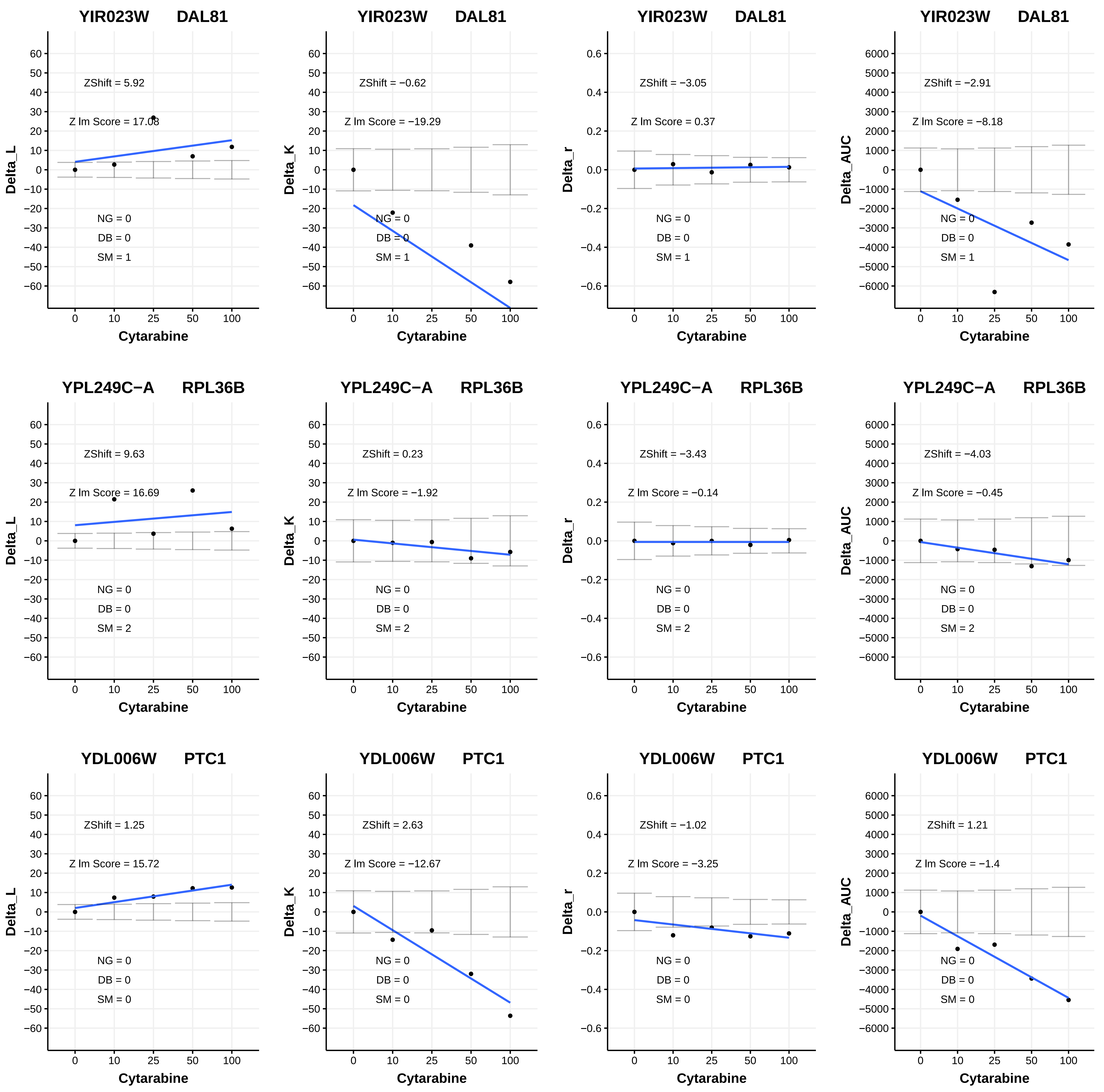
YIR023W DAL81
ZShift = 5.92
Z lm Score = 17.08
NG = 0
DB = 0
SM = 1
Delta_L
Cytarabine
YIR023W DAL81
ZShift = −0.62
Z lm Score = −19.29
NG = 0
DB = 0
SM = 1
Delta_K
Cytarabine
YIR023W DAL81
ZShift = −3.05
Z lm Score = 0.37
NG = 0
DB = 0
SM = 1
Delta_r
Cytarabine
YIR023W DAL81
ZShift = −2.91
Z lm Score = −8.18
NG = 0
DB = 0
SM = 1
Delta_AUC
Cytarabine
YPL249C−A RPL36B
ZShift = 9.63
Z lm Score = 16.69
NG = 0
DB = 0
SM = 2
Delta_L
Cytarabine
YPL249C−A RPL36B
ZShift = 0.23
Z lm Score = −1.92
NG = 0
DB = 0
SM = 2
Delta_K
Cytarabine
YPL249C−A RPL36B
ZShift = −3.43
Z lm Score = −0.14
NG = 0
DB = 0
SM = 2
Delta_r
Cytarabine
YPL249C−A RPL36B
ZShift = −4.03
Z lm Score = −0.45
NG = 0
DB = 0
SM = 2
Delta_AUC
Cytarabine
YDL006W PTC1
ZShift = 1.25
Z lm Score = 15.72
NG = 0
DB = 0
SM = 0
Delta_L
Cytarabine
YDL006W PTC1
ZShift = 2.63
Z lm Score = −12.67
NG = 0
DB = 0
SM = 0
Delta_K
Cytarabine
YDL006W PTC1
ZShift = −1.02
Z lm Score = −3.25
NG = 0
DB = 0
SM = 0
Delta_r
Cytarabine
YDL006W PTC1
ZShift = 1.21
Z lm Score = −1.4
NG = 0
DB = 0
SM = 0
Delta_AUC
Cytarabine

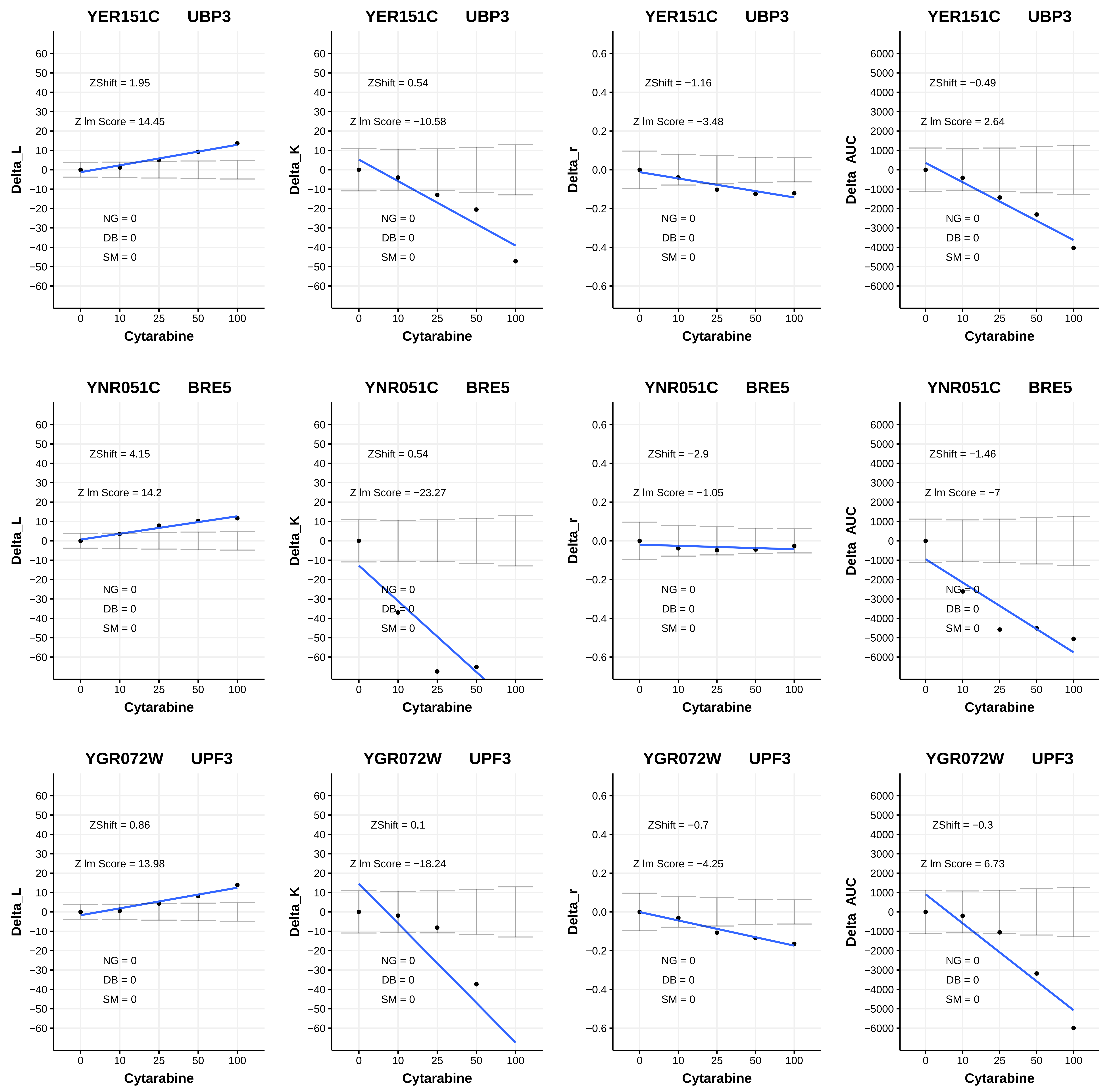
YER151C UBP3
ZShift = 1.95
Z lm Score = 14.45
NG = 0
DB = 0
SM = 0
Delta_L
Cytarabine
YER151C UBP3
ZShift = 0.54
Z lm Score = −10.58
NG = 0
DB = 0
SM = 0
Delta_K
Cytarabine
YER151C UBP3
ZShift = −1.16
Z lm Score = −3.48
NG = 0
DB = 0
SM = 0
Delta_r
Cytarabine
YER151C UBP3
ZShift = −0.49
Z lm Score = 2.64
NG = 0
DB = 0
SM = 0
Delta_AUC
Cytarabine
YNR051C BRE5
ZShift = 4.15
Z lm Score = 14.2
NG = 0
DB = 0
SM = 0
Delta_L
Cytarabine
YNR051C BRE5
ZShift = 0.54
Z lm Score = −23.27
NG = 0
DB = 0
SM = 0
Delta_K
Cytarabine
YNR051C BRE5
ZShift = −2.9
Z lm Score = −1.05
NG = 0
DB = 0
SM = 0
Delta_r
Cytarabine
YNR051C BRE5
ZShift = −1.46
Z lm Score = −7
NG = 0
DB = 0
SM = 0
Delta_AUC
Cytarabine
YGR072W UPF3
ZShift = 0.86
Z lm Score = 13.98
NG = 0
DB = 0
SM = 0
Delta_L
Cytarabine
YGR072W UPF3
ZShift = 0.1
Z lm Score = −18.24
NG = 0
DB = 0
SM = 0
Delta_K
Cytarabine
YGR072W UPF3
ZShift = −0.7
Z lm Score = −4.25
NG = 0
DB = 0
SM = 0
Delta_r
Cytarabine
YGR072W UPF3
ZShift = −0.3
Z lm Score = 6.73
NG = 0
DB = 0
SM = 0
Delta_AUC
Cytarabine

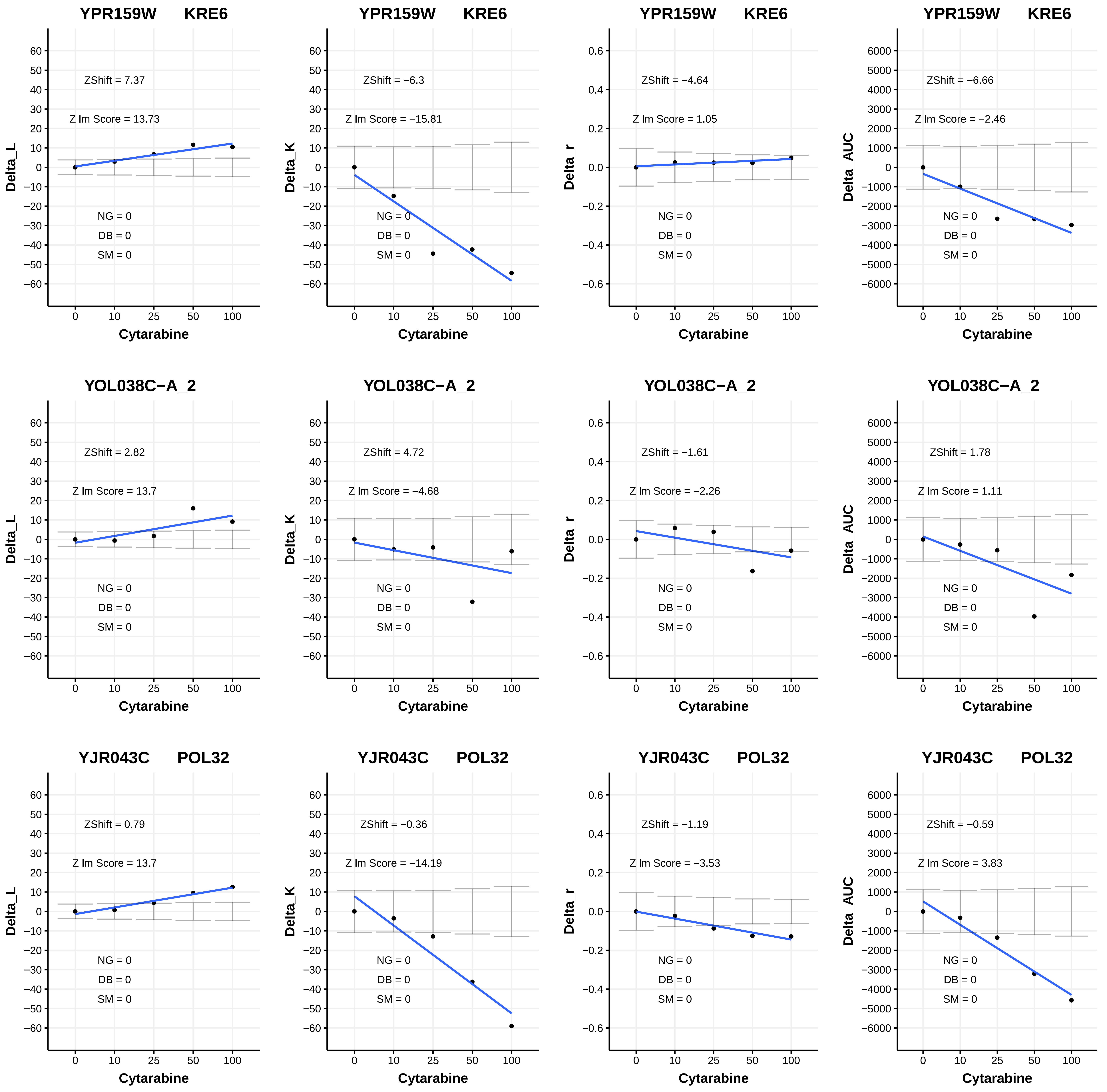

YPR159W KRE6
ZShift = 7.37
Z lm Score = 13.73
NG = 0
DB = 0
SM = 0
Delta_L
Cytarabine
YPR159W KRE6
ZShift = −6.3
Z lm Score = −15.81
NG = 0
DB = 0
SM = 0
Delta_K
Cytarabine
YPR159W KRE6
ZShift = −4.64
Z lm Score = 1.05
NG = 0
DB = 0
SM = 0
Delta_r
Cytarabine
YPR159W KRE6
ZShift = −6.66
Z lm Score = −2.46
NG = 0
DB = 0
SM = 0
Delta_AUC
Cytarabine
YOL038C−A_2
ZShift = 2.82
Z lm Score = 13.7
NG = 0
DB = 0
SM = 0
Delta_L
Cytarabine
YOL038C−A_2
ZShift = 4.72
Z lm Score = −4.68
NG = 0
DB = 0
SM = 0
Delta_K
Cytarabine
YOL038C−A_2
ZShift = −1.61
Z lm Score = −2.26
NG = 0
DB = 0
SM = 0
Delta_r
Cytarabine
YOL038C−A_2
ZShift = 1.78
Z lm Score = 1.11
NG = 0
DB = 0
SM = 0
Delta_AUC
Cytarabine
YJR043C POL32
ZShift = 0.79
Z lm Score = 13.7
NG = 0
DB = 0
SM = 0
Delta_L
Cytarabine
YJR043C POL32
ZShift = −0.36
Z lm Score = −14.19
NG = 0
DB = 0
SM = 0
Delta_K
Cytarabine
YJR043C POL32
ZShift = −1.19
Z lm Score = −3.53
NG = 0
DB = 0
SM = 0
Delta_r
Cytarabine
YJR043C POL32
ZShift = −0.59
Z lm Score = 3.83
NG = 0
DB = 0
SM = 0
Delta_AUC
Cytarabine

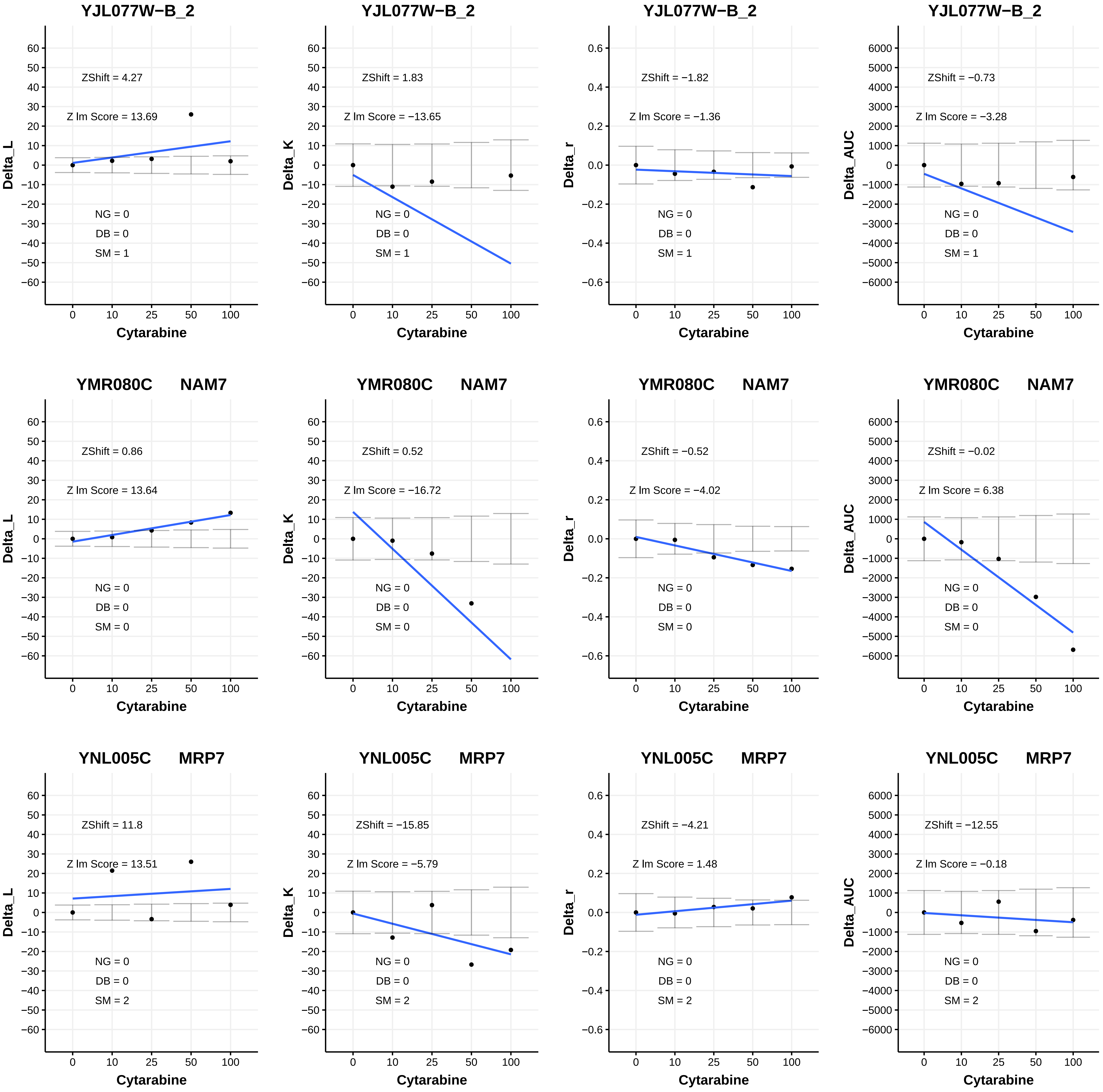

YJL077W−B_2
ZShift = 4.27
Z lm Score = 13.69
NG = 0
DB = 0
SM = 1
Delta_L
Cytarabine
YJL077W−B_2
ZShift = 1.83
Z lm Score = −13.65
NG = 0
DB = 0
SM = 1
Delta_K
Cytarabine
YJL077W−B_2
ZShift = −1.82
Z lm Score = −1.36
NG = 0
DB = 0
SM = 1
Delta_r
Cytarabine
YJL077W−B_2
ZShift = −0.73
Z lm Score = −3.28
NG = 0
DB = 0
SM = 1
Delta_AUC
Cytarabine
YMR080C NAM7
ZShift = 0.86
Z lm Score = 13.64
NG = 0
DB = 0
SM = 0
Delta_L
Cytarabine
YMR080C NAM7
ZShift = 0.52
Z lm Score = −16.72
NG = 0
DB = 0
SM = 0
Delta_K
Cytarabine
YMR080C NAM7
ZShift = −0.52
Z lm Score = −4.02
NG = 0
DB = 0
SM = 0
Delta_r
Cytarabine
YMR080C NAM7
ZShift = −0.02
Z lm Score = 6.38
NG = 0
DB = 0
SM = 0
Delta_AUC
Cytarabine
YNL005C MRP7
ZShift = 11.8
Z lm Score = 13.51
NG = 0
DB = 0
SM = 2
Delta_L
Cytarabine
YNL005C MRP7
ZShift = −15.85
Z lm Score = −5.79
NG = 0
DB = 0
SM = 2
Delta_K
Cytarabine
YNL005C MRP7
ZShift = −4.21
Z lm Score = 1.48
NG = 0
DB = 0
SM = 2
Delta_r
Cytarabine
YNL005C MRP7
ZShift = −12.55
Z lm Score = −0.18
NG = 0
DB = 0
SM = 2
Delta_AUC
Cytarabine

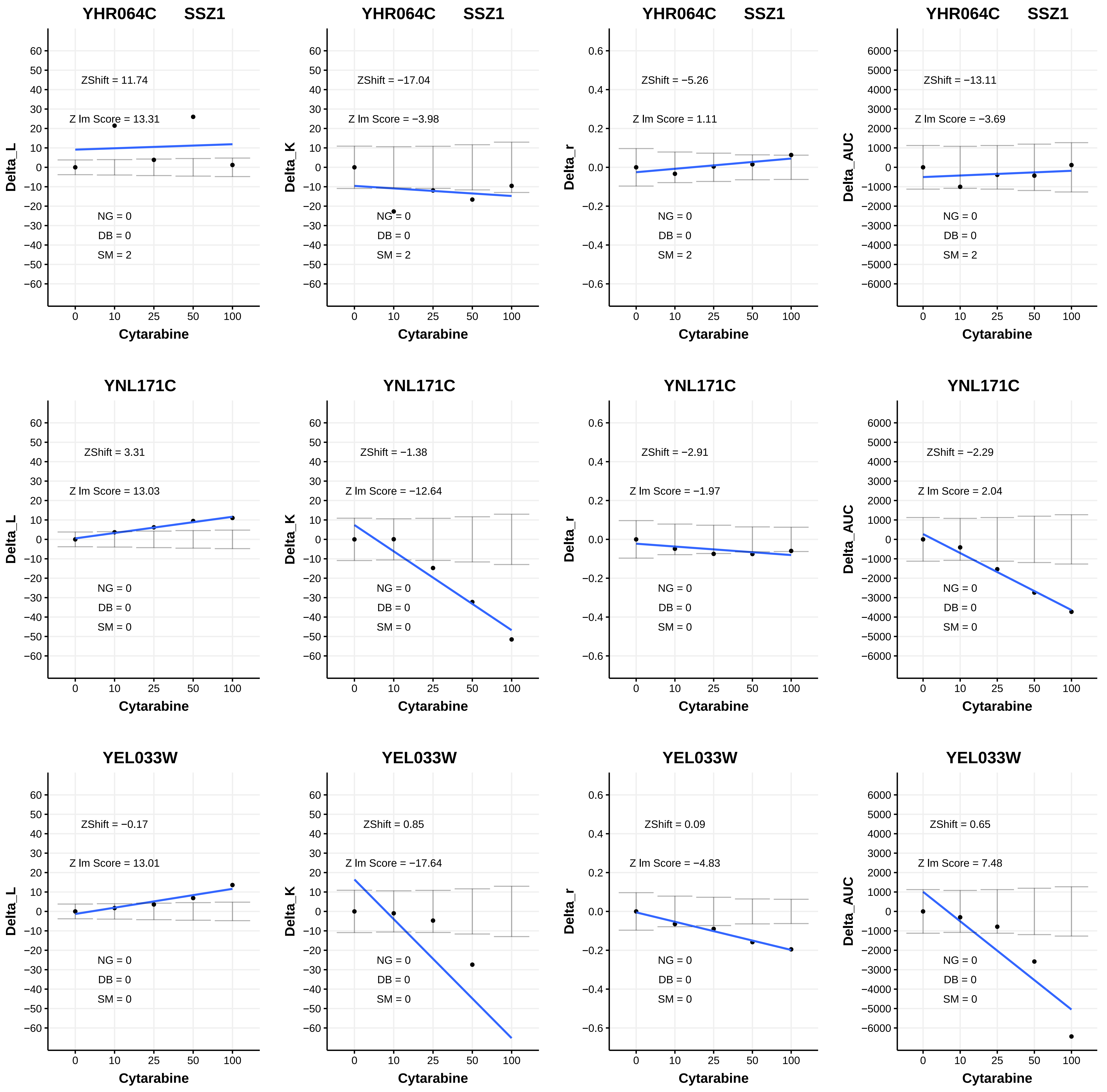

YHR064C SSZ1
ZShift = 11.74
Z lm Score = 13.31
NG = 0
DB = 0
SM = 2
Delta_L
Cytarabine
YHR064C SSZ1
ZShift = −17.04
Z lm Score = −3.98
NG = 0
DB = 0
SM = 2
Delta_K
Cytarabine
YHR064C SSZ1
ZShift = −5.26
Z lm Score = 1.11
NG = 0
DB = 0
SM = 2
Delta_r
Cytarabine
YHR064C SSZ1
ZShift = −13.11
Z lm Score = −3.69
NG = 0
DB = 0
SM = 2
Delta_AUC
Cytarabine
YNL171C
ZShift = 3.31
Z lm Score = 13.03
NG = 0
DB = 0
SM = 0
Delta_L
Cytarabine
YNL171C
ZShift = −1.38
Z lm Score = −12.64
NG = 0
DB = 0
SM = 0
Delta_K
Cytarabine
YNL171C
ZShift = −2.91
Z lm Score = −1.97
NG = 0
DB = 0
SM = 0
Delta_r
Cytarabine
YNL171C
ZShift = −2.29
Z lm Score = 2.04
NG = 0
DB = 0
SM = 0
Delta_AUC
Cytarabine
YEL033W
ZShift = −0.17
Z lm Score = 13.01
NG = 0
DB = 0
SM = 0
Delta_L
Cytarabine
YEL033W
ZShift = 0.85
Z lm Score = −17.64
NG = 0
DB = 0
SM = 0
Delta_K
Cytarabine
YEL033W
ZShift = 0.09
Z lm Score = −4.83
NG = 0
DB = 0
SM = 0
Delta_r
Cytarabine
YEL033W
ZShift = 0.65
Z lm Score = 7.48
NG = 0
DB = 0
SM = 0
Delta_AUC
Cytarabine

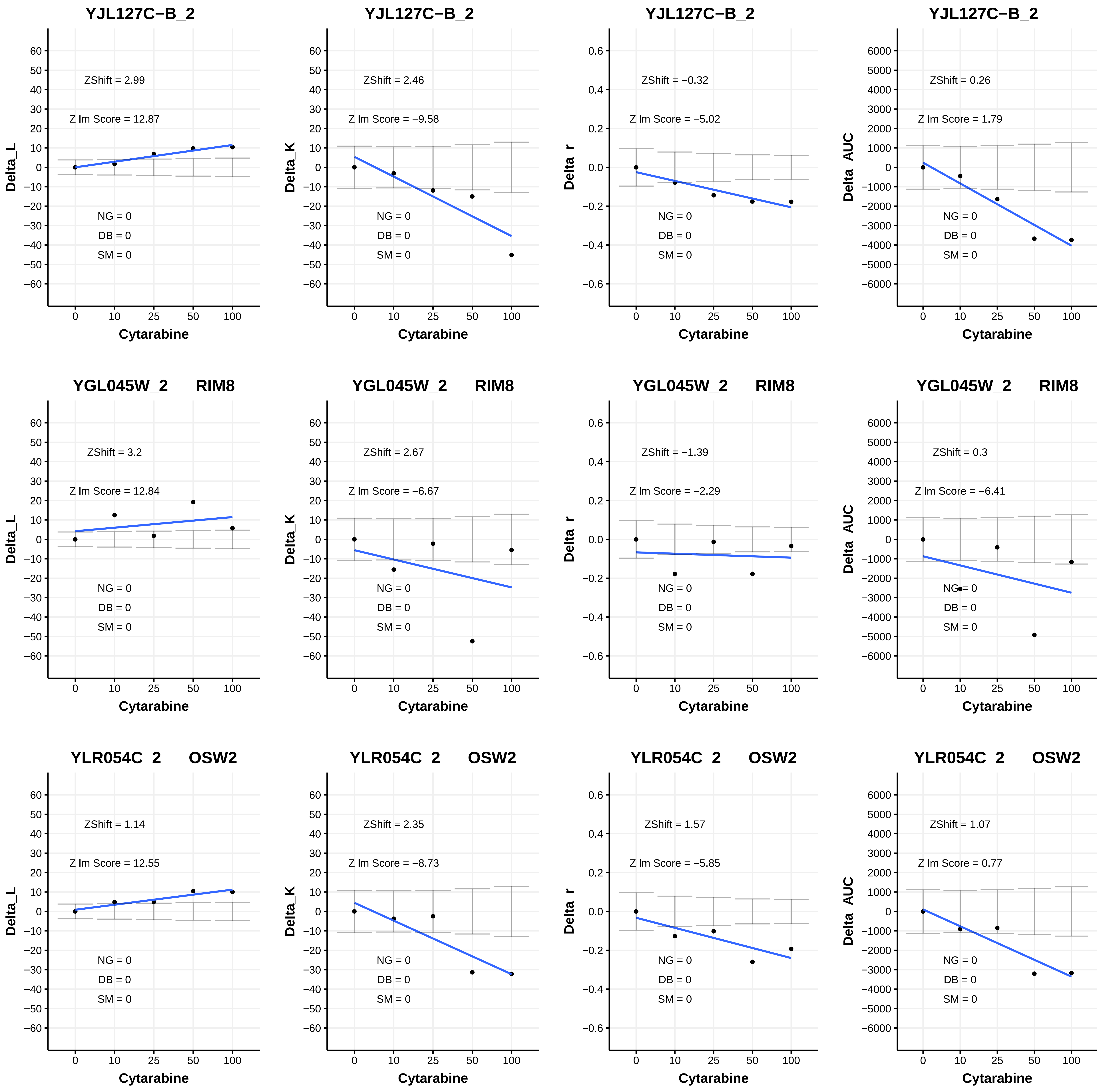

YJL127C−B_2
ZShift = 2.99
Z lm Score = 12.87
NG = 0
DB = 0
SM = 0
Delta_L
Cytarabine
YJL127C−B_2
ZShift = 2.46
Z lm Score = −9.58
NG = 0
DB = 0
SM = 0
Delta_K
Cytarabine
YJL127C−B_2
ZShift = −0.32
Z lm Score = −5.02
NG = 0
DB = 0
SM = 0
Delta_r
Cytarabine
YJL127C−B_2
ZShift = 0.26
Z lm Score = 1.79
NG = 0
DB = 0
SM = 0
Delta_AUC
Cytarabine
YGL045W_2 RIM8
ZShift = 3.2
Z lm Score = 12.84
NG = 0
DB = 0
SM = 0
Delta_L
Cytarabine
YGL045W_2 RIM8
ZShift = 2.67
Z lm Score = −6.67
NG = 0
DB = 0
SM = 0
Delta_K
Cytarabine
YGL045W_2 RIM8
ZShift = −1.39
Z lm Score = −2.29
NG = 0
DB = 0
SM = 0
Delta_r
Cytarabine
YGL045W_2 RIM8
ZShift = 0.3
Z lm Score = −6.41
NG = 0
DB = 0
SM = 0
Delta_AUC
Cytarabine
YLR054C_2 OSW2
ZShift = 1.14
Z lm Score = 12.55
NG = 0
DB = 0
SM = 0
Delta_L
Cytarabine
YLR054C_2 OSW2
ZShift = 2.35
Z lm Score = −8.73
NG = 0
DB = 0
SM = 0
Delta_K
Cytarabine
YLR054C_2 OSW2
ZShift = 1.57
Z lm Score = −5.85
NG = 0
DB = 0
SM = 0
Delta_r
Cytarabine
YLR054C_2 OSW2
ZShift = 1.07
Z lm Score = 0.77
NG = 0
DB = 0
SM = 0
Delta_AUC
Cytarabine

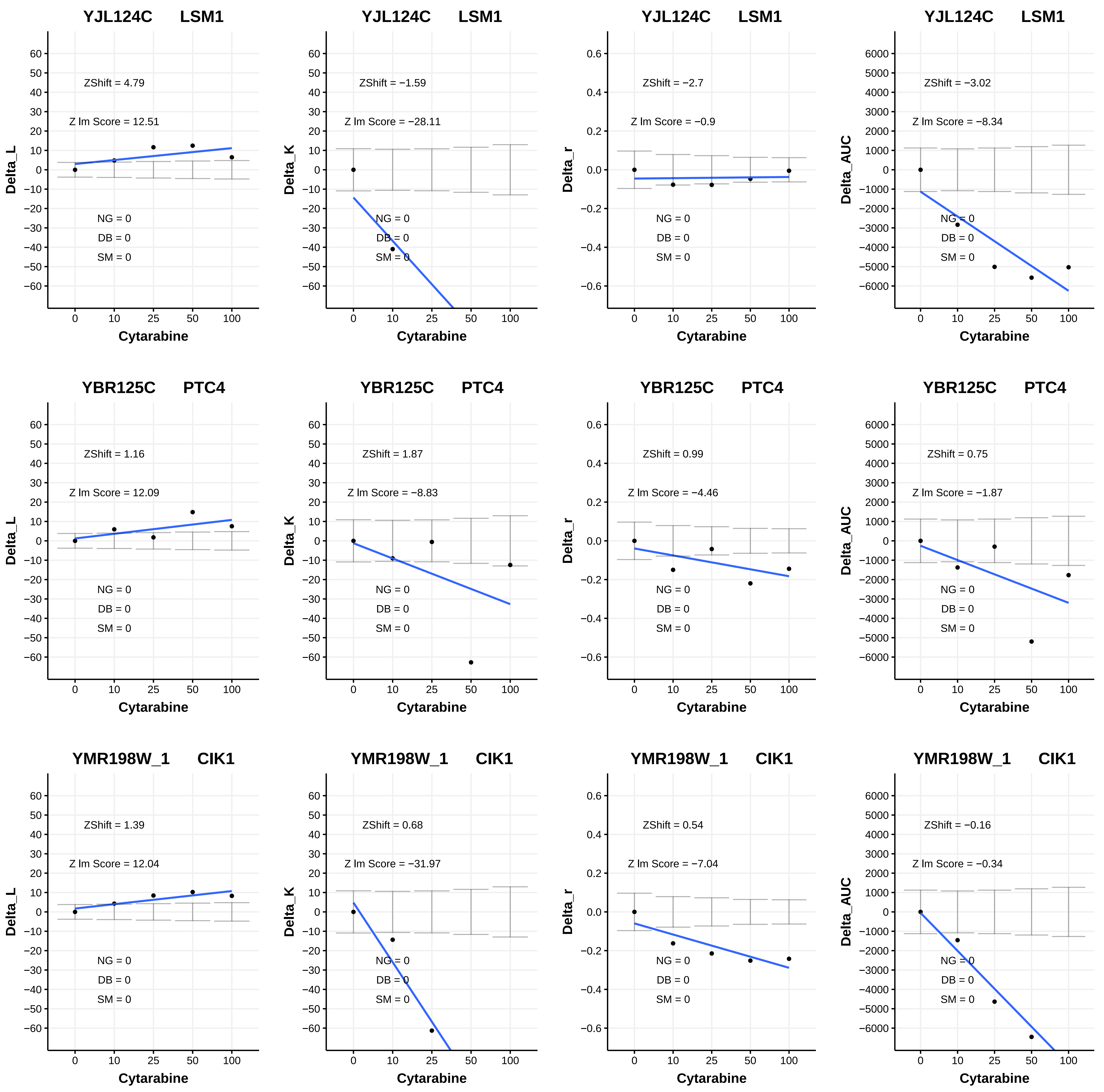

YJL124C LSM1
ZShift = 4.79
Z lm Score = 12.51
NG = 0
DB = 0
SM = 0
Delta_L
Cytarabine
YJL124C LSM1
ZShift = −1.59
Z lm Score = −28.11
NG = 0
DB = 0
SM = 0
Delta_K
Cytarabine
YJL124C LSM1
ZShift = −2.7
Z lm Score = −0.9
NG = 0
DB = 0
SM = 0
Delta_r
Cytarabine
YJL124C LSM1
ZShift = −3.02
Z lm Score = −8.34
NG = 0
DB = 0
SM = 0
Delta_AUC
Cytarabine
YBR125C PTC4
ZShift = 1.16
Z lm Score = 12.09
NG = 0
DB = 0
SM = 0
Delta_L
Cytarabine
YBR125C PTC4
ZShift = 1.87
Z lm Score = −8.83
NG = 0
DB = 0
SM = 0
Delta_K
Cytarabine
YBR125C PTC4
ZShift = 0.99
Z lm Score = −4.46
NG = 0
DB = 0
SM = 0
Delta_r
Cytarabine
YBR125C PTC4
ZShift = 0.75
Z lm Score = −1.87
NG = 0
DB = 0
SM = 0
Delta_AUC
Cytarabine
YMR198W_1 CIK1
ZShift = 1.39
Z lm Score = 12.04
NG = 0
DB = 0
SM = 0
Delta_L
Cytarabine
YMR198W_1 CIK1
ZShift = 0.68
Z lm Score = −31.97
NG = 0
DB = 0
SM = 0
Delta_K
Cytarabine
YMR198W_1 CIK1
ZShift = 0.54
Z lm Score = −7.04
NG = 0
DB = 0
SM = 0
Delta_r
Cytarabine
YMR198W_1 CIK1
ZShift = −0.16
Z lm Score = −0.34
NG = 0
DB = 0
SM = 0
Delta_AUC
Cytarabine

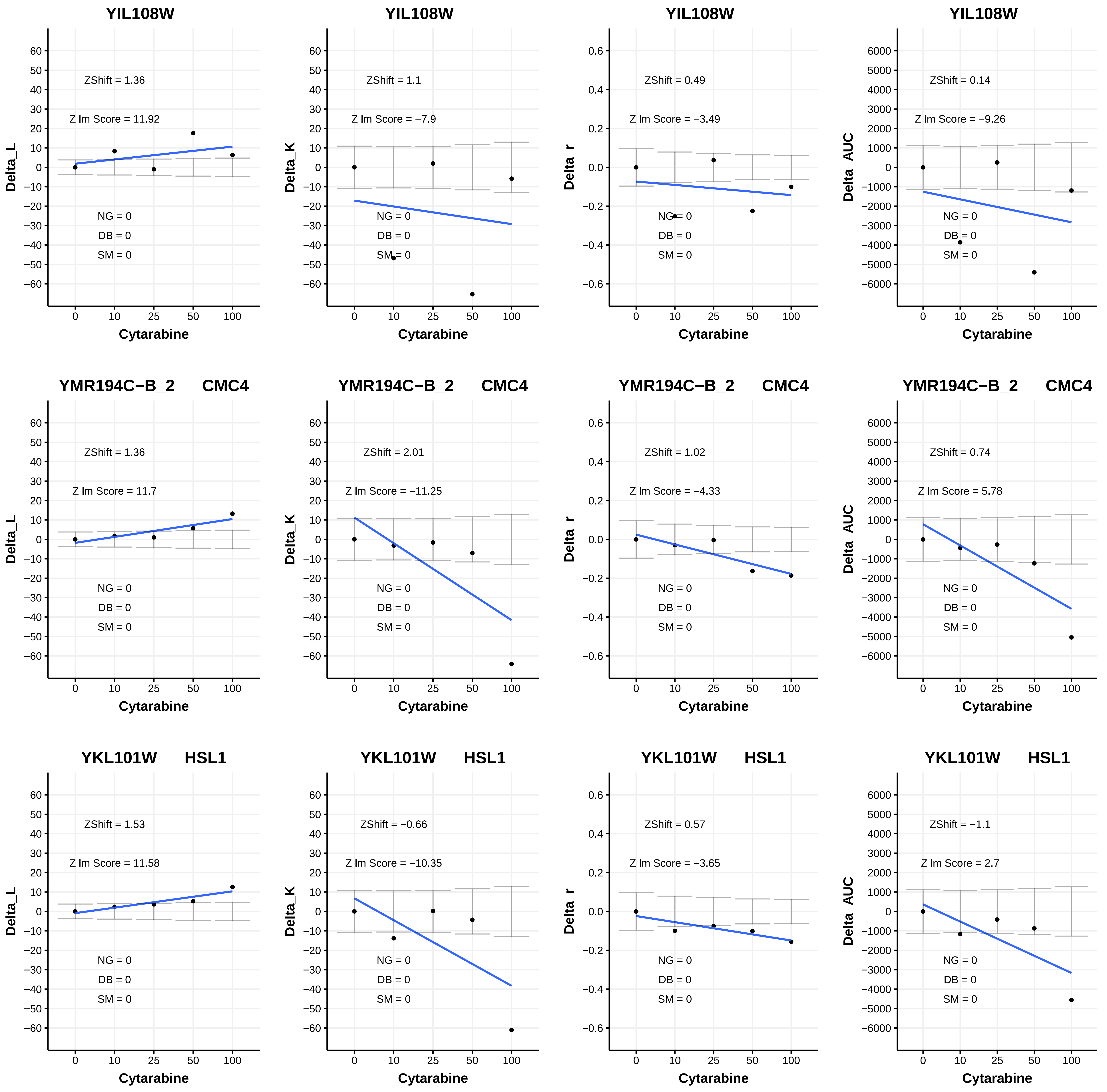
YIL108W
ZShift = 1.36
Z lm Score = 11.92
NG = 0
DB = 0
SM = 0
Delta_L
Cytarabine
YIL108W
ZShift = 1.1
Z lm Score = −7.9
NG = 0
DB = 0
SM = 0
Delta_K
Cytarabine
YIL108W
ZShift = 0.49
Z lm Score = −3.49
NG = 0
DB = 0
SM = 0
Delta_r
Cytarabine
YIL108W
ZShift = 0.14
Z lm Score = −9.26
NG = 0
DB = 0
SM = 0
Delta_AUC
Cytarabine
YMR194C−B_2 CMC4
ZShift = 1.36
Z lm Score = 11.7
NG = 0
DB = 0
SM = 0
Delta_L
Cytarabine
YMR194C−B_2 CMC4
ZShift = 2.01
Z lm Score = −11.25
NG = 0
DB = 0
SM = 0
Delta_K
Cytarabine
YMR194C−B_2 CMC4
ZShift = 1.02
Z lm Score = −4.33
NG = 0
DB = 0
SM = 0
Delta_r
Cytarabine
YMR194C−B_2 CMC4
ZShift = 0.74
Z lm Score = 5.78
NG = 0
DB = 0
SM = 0
Delta_AUC
Cytarabine
YKL101W HSL1
ZShift = 1.53
Z lm Score = 11.58
NG = 0
DB = 0
SM = 0
Delta_L
Cytarabine
YKL101W HSL1
ZShift = −0.66
Z lm Score = −10.35
NG = 0
DB = 0
SM = 0
Delta_K
Cytarabine
YKL101W HSL1
ZShift = 0.57
Z lm Score = −3.65
NG = 0
DB = 0
SM = 0
Delta_r
Cytarabine
YKL101W HSL1
ZShift = −1.1
Z lm Score = 2.7
NG = 0
DB = 0
SM = 0
Delta_AUC
Cytarabine

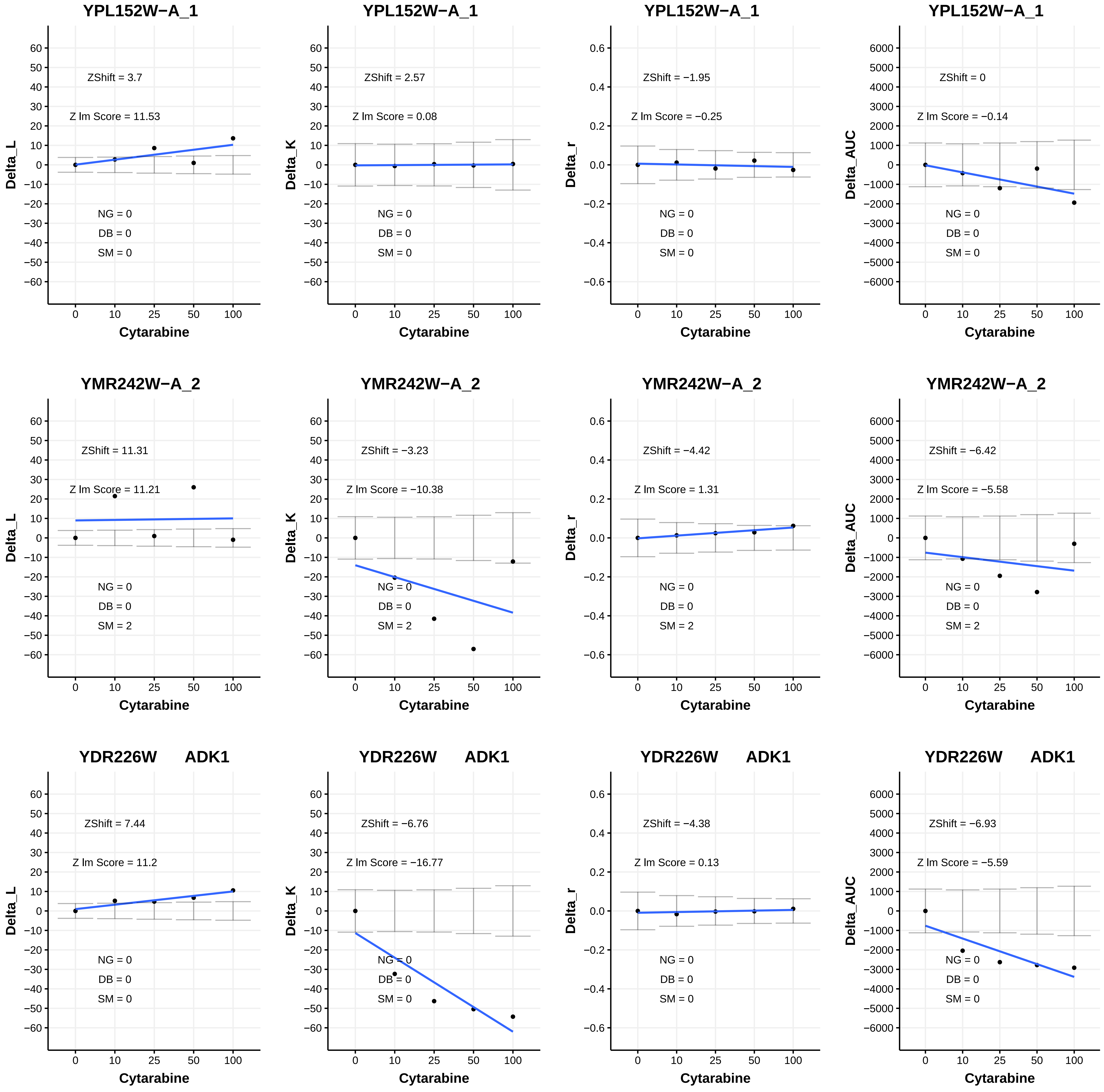
YPL152W−A_1
ZShift = 3.7
Z lm Score = 11.53
NG = 0
DB = 0
SM = 0
Delta_L
Cytarabine
YPL152W−A_1
ZShift = 2.57
Z lm Score = 0.08
NG = 0
DB = 0
SM = 0
Delta_K
Cytarabine
YPL152W−A_1
ZShift = −1.95
Z lm Score = −0.25
NG = 0
DB = 0
SM = 0
Delta_r
Cytarabine
YPL152W−A_1
ZShift = 0
Z lm Score = −0.14
NG = 0
DB = 0
SM = 0
Delta_AUC
Cytarabine
YMR242W−A_2
ZShift = 11.31
Z lm Score = 11.21
NG = 0
DB = 0
SM = 2
Delta_L
Cytarabine
YMR242W−A_2
ZShift = −3.23
Z lm Score = −10.38
NG = 0
DB = 0
SM = 2
Delta_K
Cytarabine
YMR242W−A_2
ZShift = −4.42
Z lm Score = 1.31
NG = 0
DB = 0
SM = 2
Delta_r
Cytarabine
YMR242W−A_2
ZShift = −6.42
Z lm Score = −5.58
NG = 0
DB = 0
SM = 2
Delta_AUC
Cytarabine
YDR226W ADK1
ZShift = 7.44
Z lm Score = 11.2
NG = 0
DB = 0
SM = 0
Delta_L
Cytarabine
YDR226W ADK1
ZShift = −6.76
Z lm Score = −16.77
NG = 0
DB = 0
SM = 0
Delta_K
Cytarabine
YDR226W ADK1
ZShift = −4.38
Z lm Score = 0.13
NG = 0
DB = 0
SM = 0
Delta_r
Cytarabine
YDR226W ADK1
ZShift = −6.93
Z lm Score = −5.59
NG = 0
DB = 0
SM = 0
Delta_AUC
Cytarabine

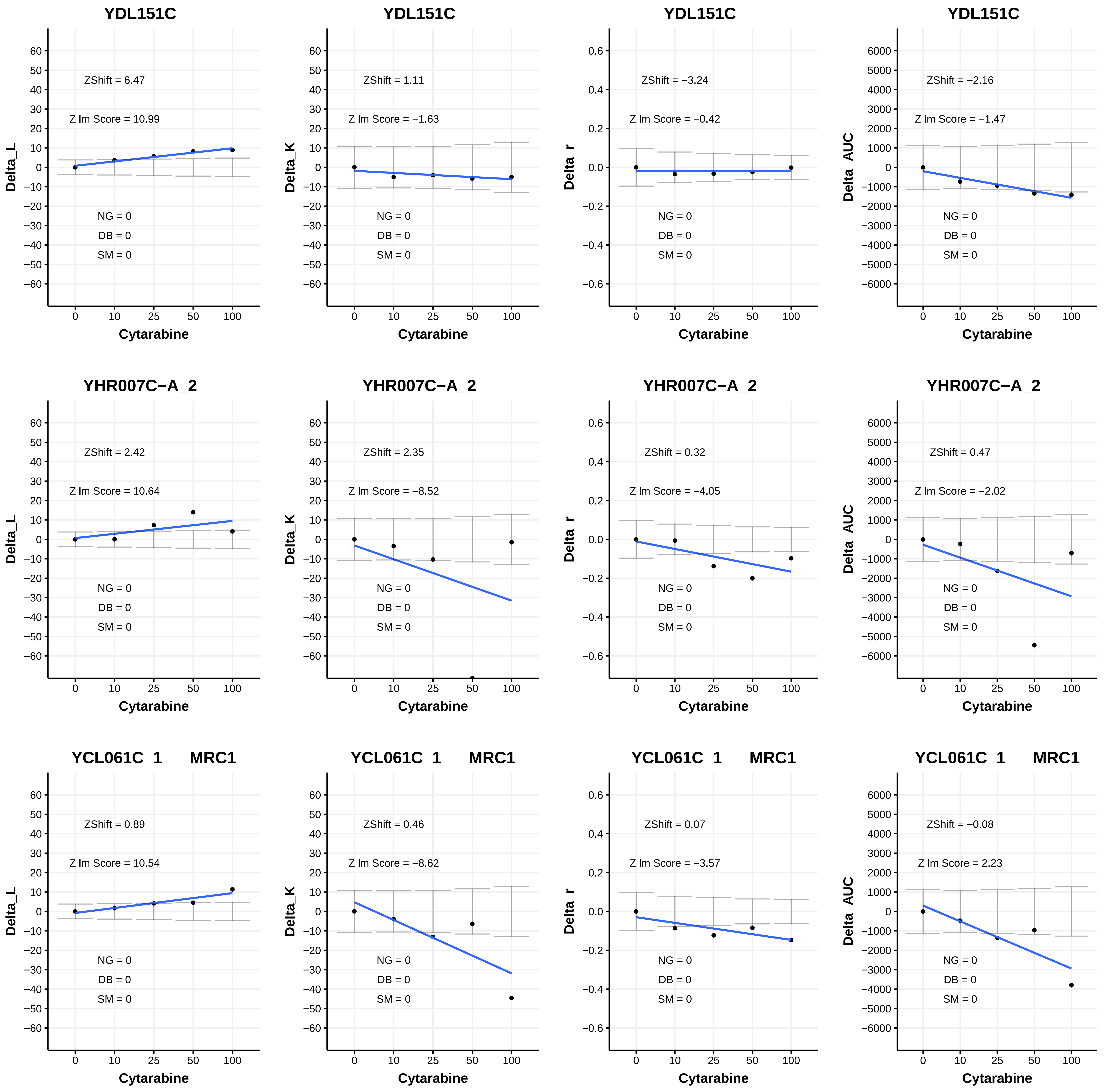

YDL151C
ZShift = 6.47
Z lm Score = 10.99
NG = 0
DB = 0
SM = 0
Delta_L
Cytarabine
YDL151C
ZShift = 1.11
Z lm Score = −1.63
NG = 0
DB = 0
SM = 0
Delta_K
Cytarabine
YDL151C
ZShift = −3.24
Z lm Score = −0.42
NG = 0
DB = 0
SM = 0
Delta_r
Cytarabine
YDL151C
ZShift = −2.16
Z lm Score = −1.47
NG = 0
DB = 0
SM = 0
Delta_AUC
Cytarabine
YHR007C−A_2
ZShift = 2.42
Z lm Score = 10.64
NG = 0
DB = 0
SM = 0
Delta_L
Cytarabine
YHR007C−A_2
ZShift = 2.35
Z lm Score = −8.52
NG = 0
DB = 0
SM = 0
Delta_K
Cytarabine
YHR007C−A_2
ZShift = 0.32
Z lm Score = −4.05
NG = 0
DB = 0
SM = 0
Delta_r
Cytarabine
YHR007C−A_2
ZShift = 0.47
Z lm Score = −2.02
NG = 0
DB = 0
SM = 0
Delta_AUC
Cytarabine
YCL061C_1 MRC1
ZShift = 0.89
Z lm Score = 10.54
NG = 0
DB = 0
SM = 0
Delta_L
Cytarabine
YCL061C_1 MRC1
ZShift = 0.46
Z lm Score = −8.62
NG = 0
DB = 0
SM = 0
Delta_K
Cytarabine
YCL061C_1 MRC1
ZShift = 0.07
Z lm Score = −3.57
NG = 0
DB = 0
SM = 0
Delta_r
Cytarabine
YCL061C_1 MRC1
ZShift = −0.08
Z lm Score = 2.23
NG = 0
DB = 0
SM = 0
Delta_AUC
Cytarabine

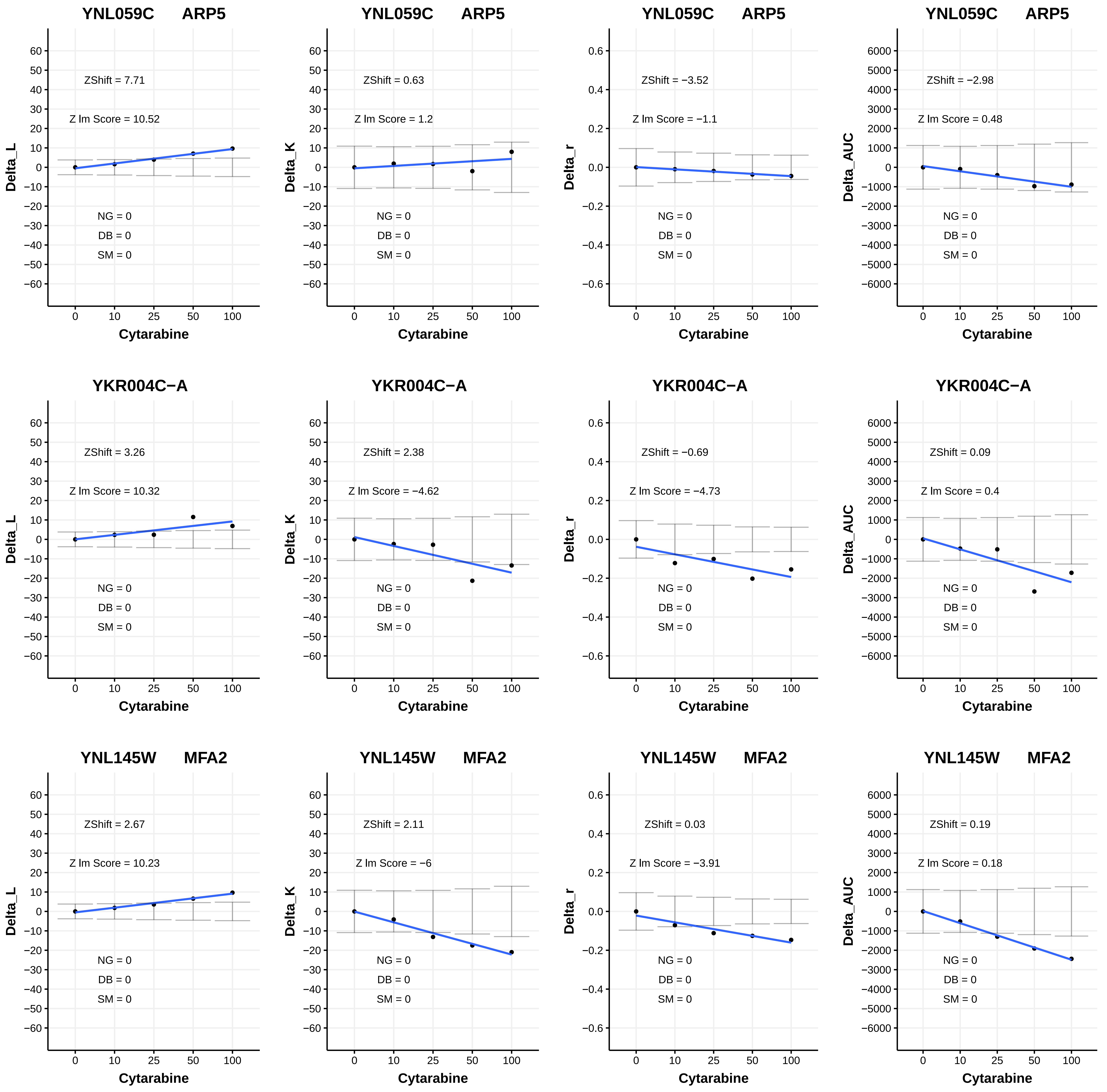

YNL059C ARP5
ZShift = 7.71
Z lm Score = 10.52
NG = 0
DB = 0
SM = 0
Delta_L
Cytarabine
YNL059C ARP5
ZShift = 0.63
Z lm Score = 1.2
NG = 0
DB = 0
SM = 0
Delta_K
Cytarabine
YNL059C ARP5
ZShift = −3.52
Z lm Score = −1.1
NG = 0
DB = 0
SM = 0
Delta_r
Cytarabine
YNL059C ARP5
ZShift = −2.98
Z lm Score = 0.48
NG = 0
DB = 0
SM = 0
Delta_AUC
Cytarabine
YKR004C−A
ZShift = 3.26
Z lm Score = 10.32
NG = 0
DB = 0
SM = 0
Delta_L
Cytarabine
YKR004C−A
ZShift = 2.38
Z lm Score = −4.62
NG = 0
DB = 0
SM = 0
Delta_K
Cytarabine
YKR004C−A
ZShift = −0.69
Z lm Score = −4.73
NG = 0
DB = 0
SM = 0
Delta_r
Cytarabine
YKR004C−A
ZShift = 0.09
Z lm Score = 0.4
NG = 0
DB = 0
SM = 0
Delta_AUC
Cytarabine
YNL145W MFA2
ZShift = 2.67
Z lm Score = 10.23
NG = 0
DB = 0
SM = 0
Delta_L
Cytarabine
YNL145W MFA2
ZShift = 2.11
Z lm Score = −6
NG = 0
DB = 0
SM = 0
Delta_K
Cytarabine
YNL145W MFA2
ZShift = 0.03
Z lm Score = −3.91
NG = 0
DB = 0
SM = 0
Delta_r
Cytarabine
YNL145W MFA2
ZShift = 0.19
Z lm Score = 0.18
NG = 0
DB = 0
SM = 0
Delta_AUC
Cytarabine

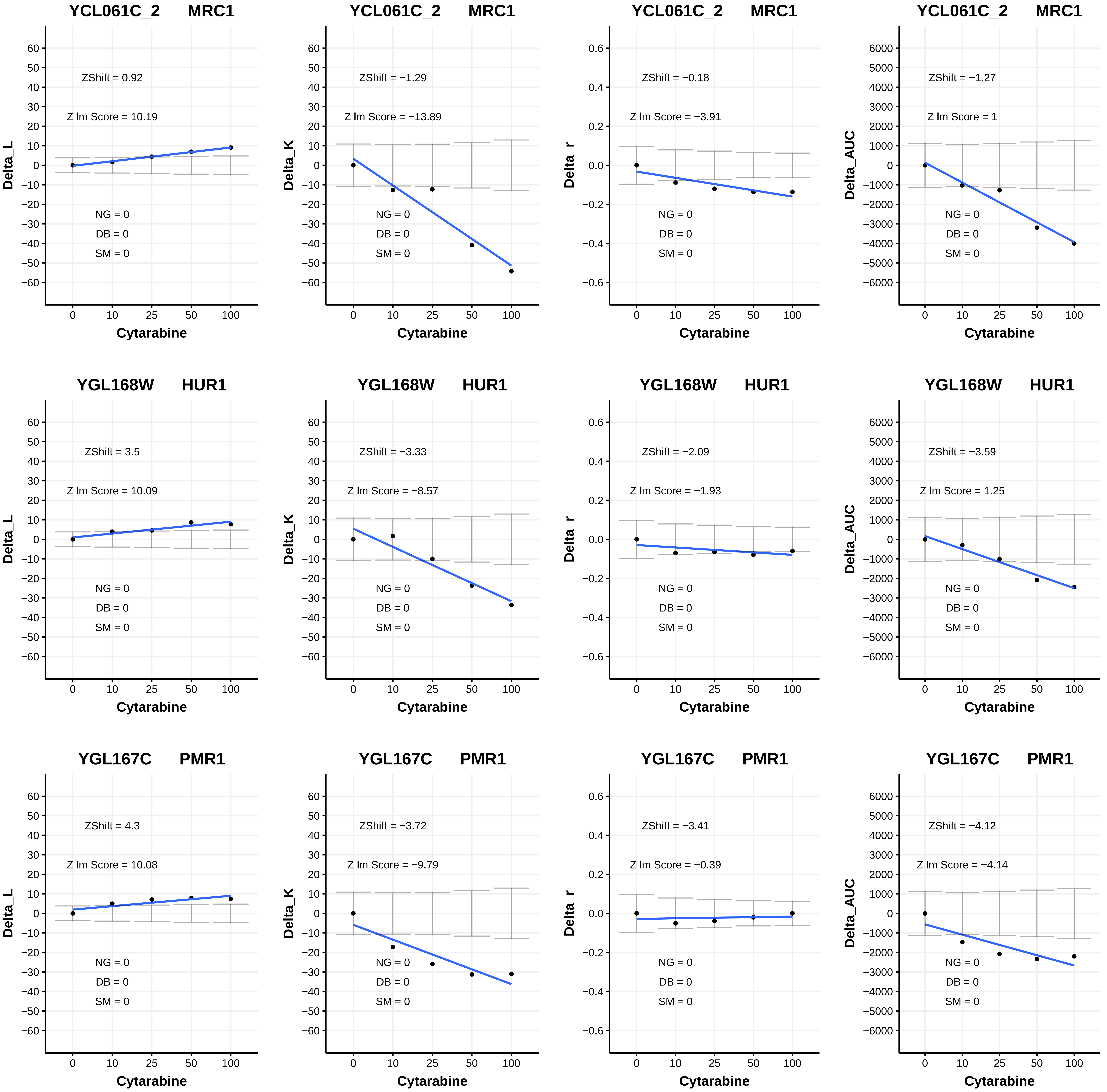

YCL061C_2 MRC1
ZShift = 0.92
Z lm Score = 10.19
NG = 0
DB = 0
SM = 0
Delta_L
Cytarabine
YCL061C_2 MRC1
ZShift = −1.29
Z lm Score = −13.89
NG = 0
DB = 0
SM = 0
Delta_K
Cytarabine
YCL061C_2 MRC1
ZShift = −0.18
Z lm Score = −3.91
NG = 0
DB = 0
SM = 0
Delta_r
Cytarabine
YCL061C_2 MRC1
ZShift = −1.27
Z lm Score = 1
NG = 0
DB = 0
SM = 0
Delta_AUC
Cytarabine
YGL168W HUR1
ZShift = 3.5
Z lm Score = 10.09
NG = 0
DB = 0
SM = 0
Delta_L
Cytarabine
YGL168W HUR1
ZShift = −3.33
Z lm Score = −8.57
NG = 0
DB = 0
SM = 0
Delta_K
Cytarabine
YGL168W HUR1
ZShift = −2.09
Z lm Score = −1.93
NG = 0
DB = 0
SM = 0
Delta_r
Cytarabine
YGL168W HUR1
ZShift = −3.59
Z lm Score = 1.25
NG = 0
DB = 0
SM = 0
Delta_AUC
Cytarabine
YGL167C PMR1
ZShift = 4.3
Z lm Score = 10.08
NG = 0
DB = 0
SM = 0
Delta_L
Cytarabine
YGL167C PMR1
ZShift = −3.72
Z lm Score = −9.79
NG = 0
DB = 0
SM = 0
Delta_K
Cytarabine
YGL167C PMR1
ZShift = −3.41
Z lm Score = −0.39
NG = 0
DB = 0
SM = 0
Delta_r
Cytarabine
YGL167C PMR1
ZShift = −4.12
Z lm Score = −4.14
NG = 0
DB = 0
SM = 0
Delta_AUC
Cytarabine

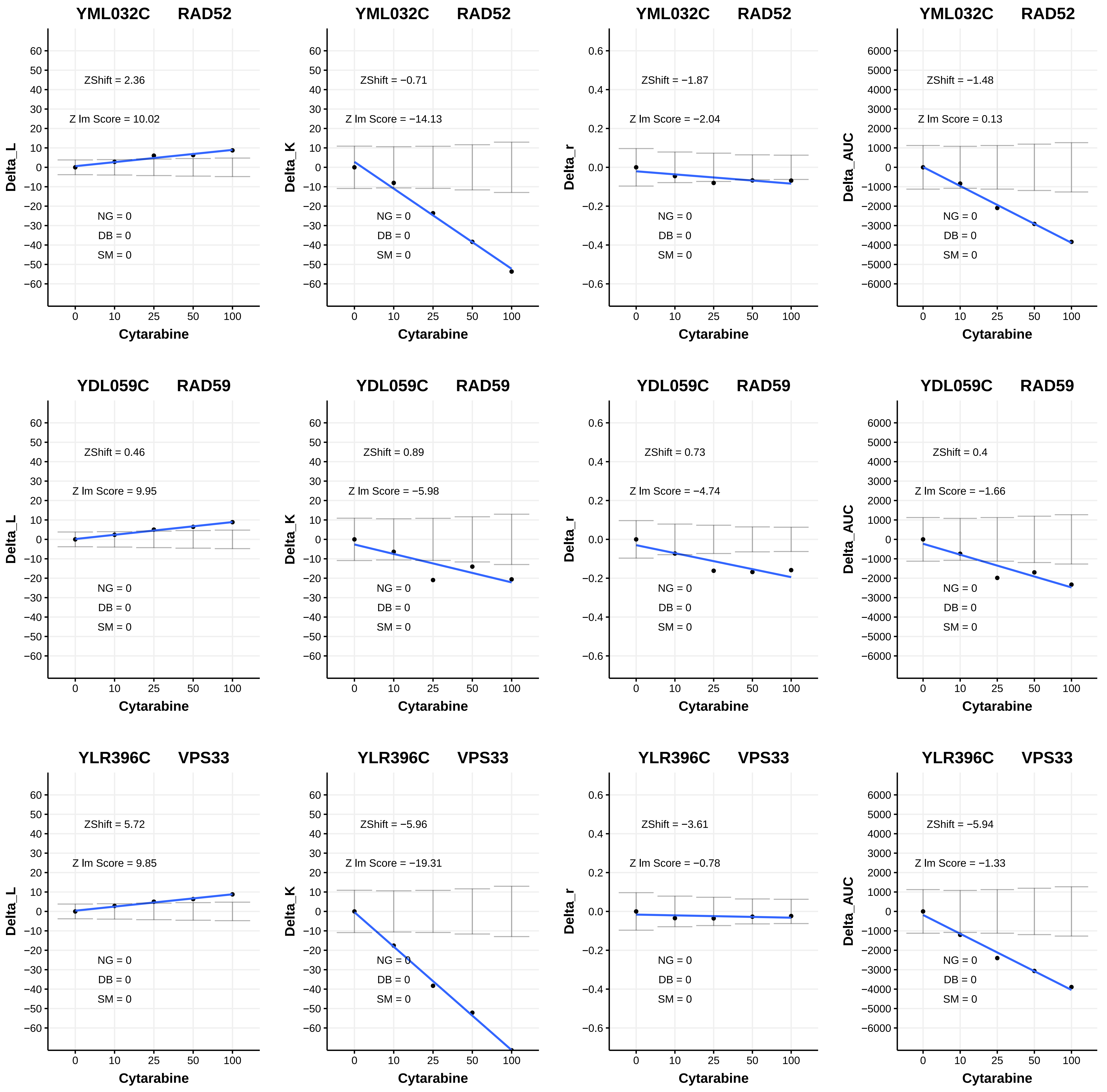
YML032C RAD52
ZShift = 2.36
Z lm Score = 10.02
NG = 0
DB = 0
SM = 0
Delta_L
Cytarabine
YML032C RAD52
ZShift = −0.71
Z lm Score = −14.13
NG = 0
DB = 0
SM = 0
Delta_K
Cytarabine
YML032C RAD52
ZShift = −1.87
Z lm Score = −2.04
NG = 0
DB = 0
SM = 0
Delta_r
Cytarabine
YML032C RAD52
ZShift = −1.48
Z lm Score = 0.13
NG = 0
DB = 0
SM = 0
Delta_AUC
Cytarabine
YDL059C RAD59
ZShift = 0.46
Z lm Score = 9.95
NG = 0
DB = 0
SM = 0
Delta_L
Cytarabine
YDL059C RAD59
ZShift = 0.89
Z lm Score = −5.98
NG = 0
DB = 0
SM = 0
Delta_K
Cytarabine
YDL059C RAD59
ZShift = 0.73
Z lm Score = −4.74
NG = 0
DB = 0
SM = 0
Delta_r
Cytarabine
YDL059C RAD59
ZShift = 0.4
Z lm Score = −1.66
NG = 0
DB = 0
SM = 0
Delta_AUC
Cytarabine
YLR396C VPS33
ZShift = 5.72
Z lm Score = 9.85
NG = 0
DB = 0
SM = 0
Delta_L
Cytarabine
YLR396C VPS33
ZShift = −5.96
Z lm Score = −19.31
NG = 0
DB = 0
SM = 0
Delta_K
Cytarabine
YLR396C VPS33
ZShift = −3.61
Z lm Score = −0.78
NG = 0
DB = 0
SM = 0
Delta_r
Cytarabine
YLR396C VPS33
ZShift = −5.94
Z lm Score = −1.33
NG = 0
DB = 0
SM = 0
Delta_AUC
Cytarabine

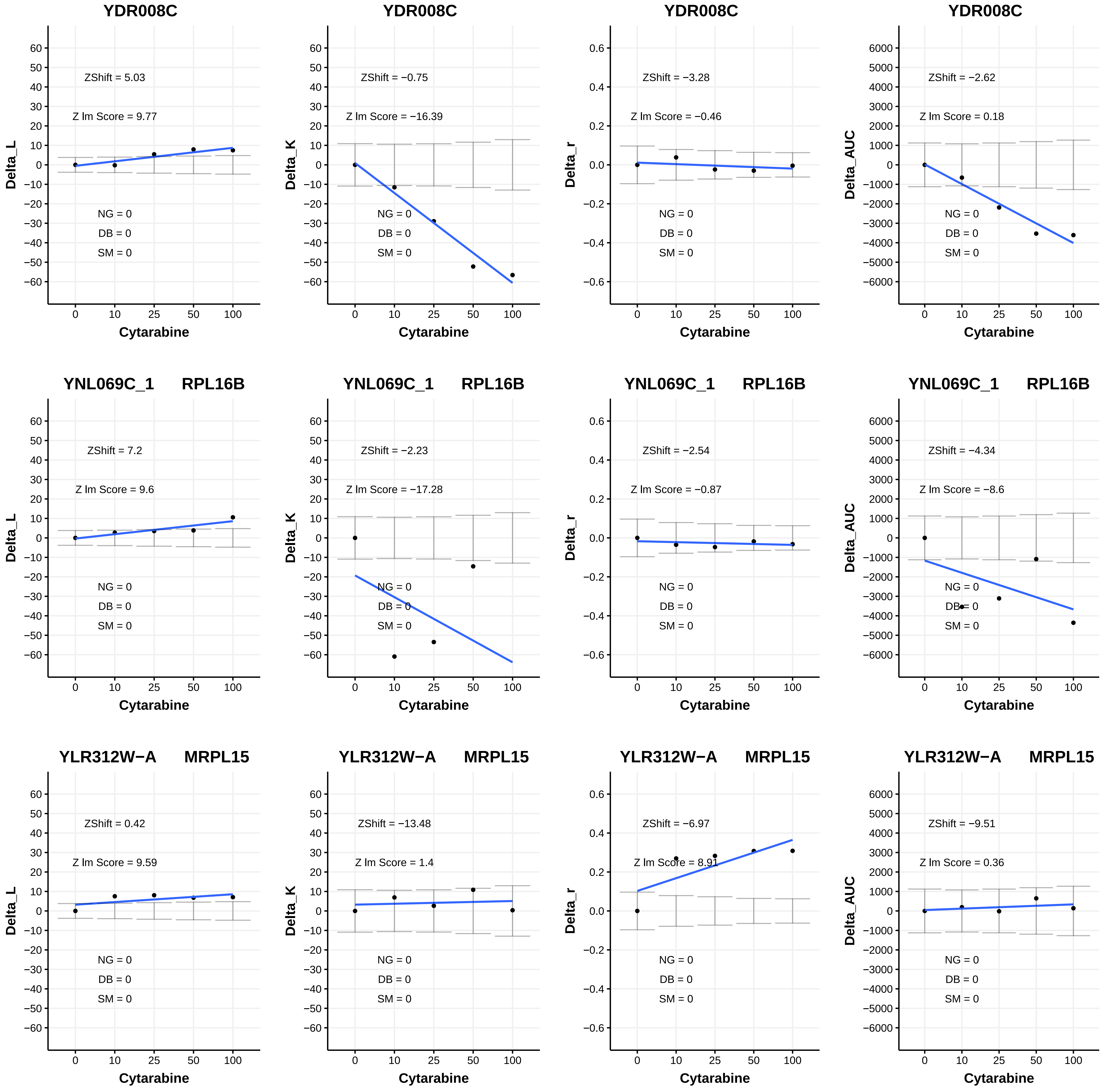

YDR008C
ZShift = 5.03
Z lm Score = 9.77
NG = 0
DB = 0
SM = 0
Delta_L
Cytarabine
YDR008C
ZShift = −0.75
Z lm Score = −16.39
NG = 0
DB = 0
SM = 0
Delta_K
Cytarabine
YDR008C
ZShift = −3.28
Z lm Score = −0.46
NG = 0
DB = 0
SM = 0
Delta_r
Cytarabine
YDR008C
ZShift = −2.62
Z lm Score = 0.18
NG = 0
DB = 0
SM = 0
Delta_AUC
Cytarabine
YNL069C_1 RPL16B
ZShift = 7.2
Z lm Score = 9.6
NG = 0
DB = 0
SM = 0
Delta_L
Cytarabine
YNL069C_1 RPL16B
ZShift = −2.23
Z lm Score = −17.28
NG = 0
DB = 0
SM = 0
Delta_K
Cytarabine
YNL069C_1 RPL16B
ZShift = −2.54
Z lm Score = −0.87
NG = 0
DB = 0
SM = 0
Delta_r
Cytarabine
YNL069C_1 RPL16B
ZShift = −4.34
Z lm Score = −8.6
NG = 0
DB = 0
SM = 0
Delta_AUC
Cytarabine
YLR312W−A MRPL15
ZShift = 0.42
Z lm Score = 9.59
NG = 0
DB = 0
SM = 0
Delta_L
Cytarabine
YLR312W−A MRPL15
ZShift = −13.48
Z lm Score = 1.4
NG = 0
DB = 0
SM = 0
Delta_K
Cytarabine
YLR312W−A MRPL15
ZShift = −6.97
Z lm Score = 8.91
NG = 0
DB = 0
SM = 0
Delta_r
Cytarabine
YLR312W−A MRPL15
ZShift = −9.51
Z lm Score = 0.36
NG = 0
DB = 0
SM = 0
Delta_AUC
Cytarabine

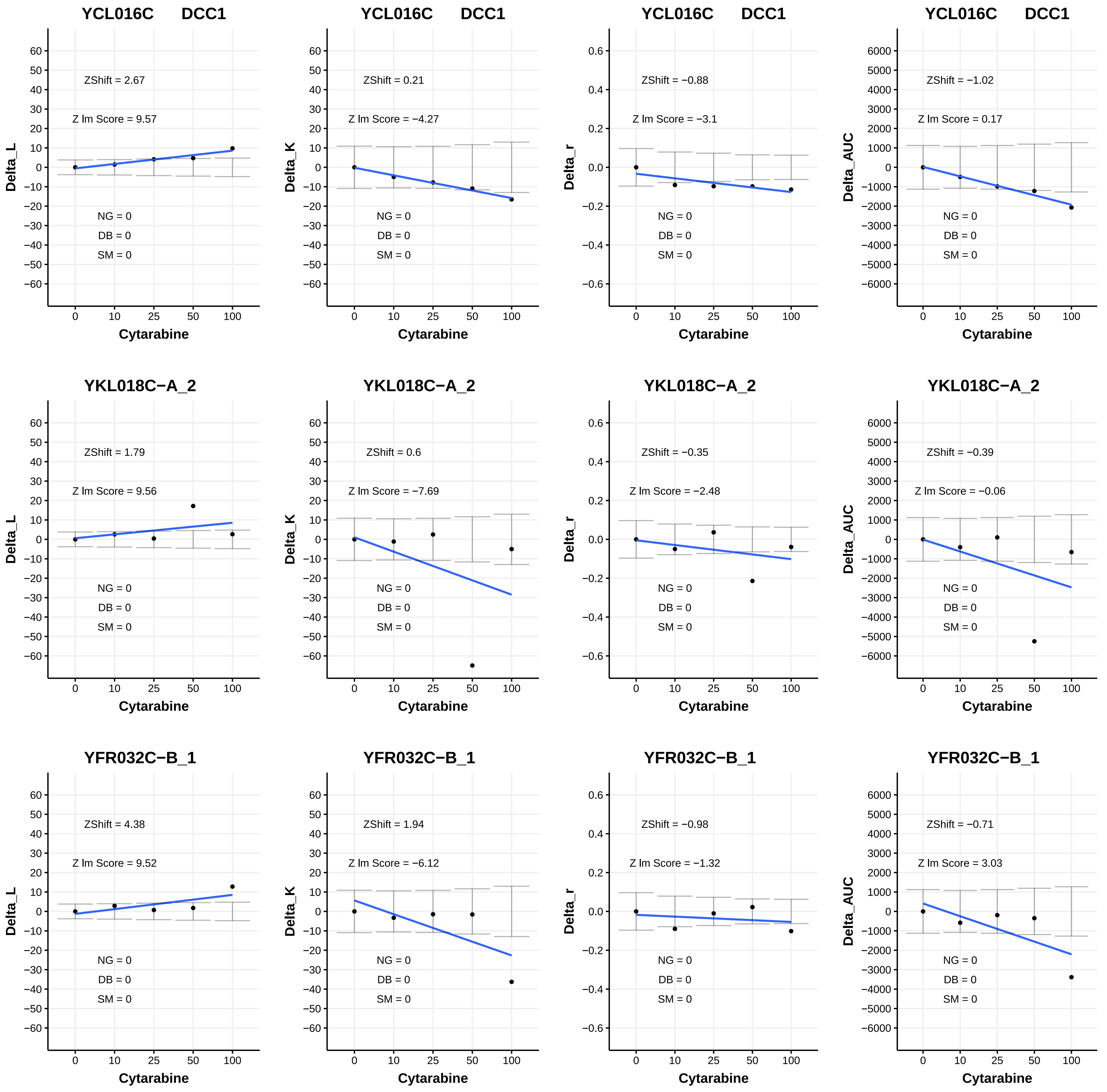

YCL016C DCC1
ZShift = 2.67
Z lm Score = 9.57
NG = 0
DB = 0
SM = 0
Delta_L
Cytarabine
YCL016C DCC1
ZShift = 0.21
Z lm Score = −4.27
NG = 0
DB = 0
SM = 0
Delta_K
Cytarabine
YCL016C DCC1
ZShift = −0.88
Z lm Score = −3.1
NG = 0
DB = 0
SM = 0
Delta_r
Cytarabine
YCL016C DCC1
ZShift = −1.02
Z lm Score = 0.17
NG = 0
DB = 0
SM = 0
Delta_AUC
Cytarabine
YKL018C−A_2
ZShift = 1.79
Z lm Score = 9.56
NG = 0
DB = 0
SM = 0
Delta_L
Cytarabine
YKL018C−A_2
ZShift = 0.6
Z lm Score = −7.69
NG = 0
DB = 0
SM = 0
Delta_K
Cytarabine
YKL018C−A_2
ZShift = −0.35
Z lm Score = −2.48
NG = 0
DB = 0
SM = 0
Delta_r
Cytarabine
YKL018C−A_2
ZShift = −0.39
Z lm Score = −0.06
NG = 0
DB = 0
SM = 0
Delta_AUC
Cytarabine
YFR032C−B_1
ZShift = 4.38
Z lm Score = 9.52
NG = 0
DB = 0
SM = 0
Delta_L
Cytarabine
YFR032C−B_1
ZShift = 1.94
Z lm Score = −6.12
NG = 0
DB = 0
SM = 0
Delta_K
Cytarabine
YFR032C−B_1
ZShift = −0.98
Z lm Score = −1.32
NG = 0
DB = 0
SM = 0
Delta_r
Cytarabine
YFR032C−B_1
ZShift = −0.71
Z lm Score = 3.03
NG = 0
DB = 0
SM = 0
Delta_AUC
Cytarabine

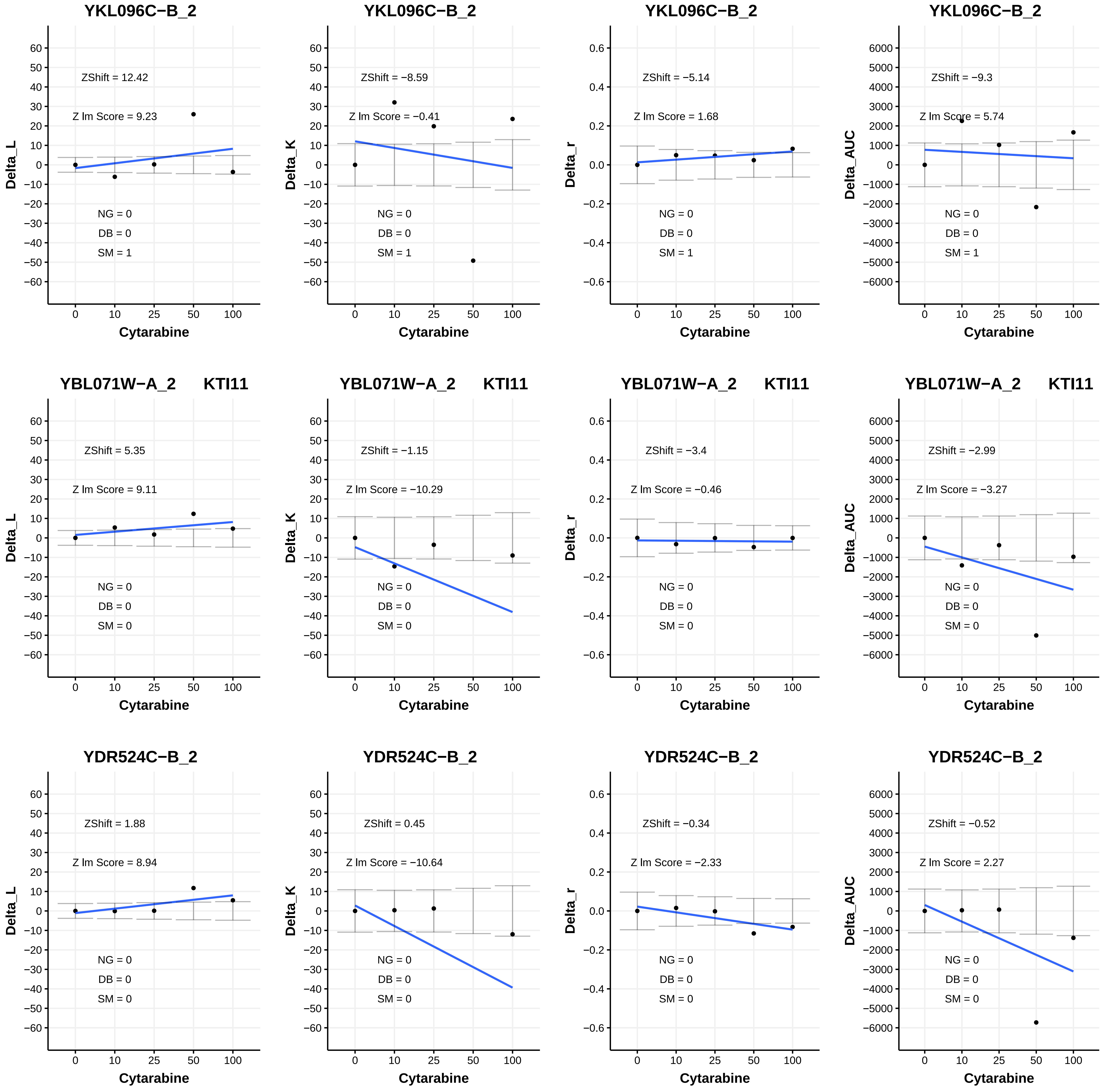

YKL096C−B_2
ZShift = 12.42
Z lm Score = 9.23
NG = 0
DB = 0
SM = 1
Delta_L
Cytarabine
YKL096C−B_2
ZShift = −8.59
Z lm Score = −0.41
NG = 0
DB = 0
SM = 1
Delta_K
Cytarabine
YKL096C−B_2
ZShift = −5.14
Z lm Score = 1.68
NG = 0
DB = 0
SM = 1
Delta_r
Cytarabine
YKL096C−B_2
ZShift = −9.3
Z lm Score = 5.74
NG = 0
DB = 0
SM = 1
Delta_AUC
Cytarabine
YBL071W−A_2 KTI11
ZShift = 5.35
Z lm Score = 9.11
NG = 0
DB = 0
SM = 0
Delta_L
Cytarabine
YBL071W−A_2 KTI11
ZShift = −1.15
Z lm Score = −10.29
NG = 0
DB = 0
SM = 0
Delta_K
Cytarabine
YBL071W−A_2 KTI11
ZShift = −3.4
Z lm Score = −0.46
NG = 0
DB = 0
SM = 0
Delta_r
Cytarabine
YBL071W−A_2 KTI11
ZShift = −2.99
Z lm Score = −3.27
NG = 0
DB = 0
SM = 0
Delta_AUC
Cytarabine
YDR524C−B_2
ZShift = 1.88
Z lm Score = 8.94
NG = 0
DB = 0
SM = 0
Delta_L
Cytarabine
YDR524C−B_2
ZShift = 0.45
Z lm Score = −10.64
NG = 0
DB = 0
SM = 0
Delta_K
Cytarabine
YDR524C−B_2
ZShift = −0.34
Z lm Score = −2.33
NG = 0
DB = 0
SM = 0
Delta_r
Cytarabine
YDR524C−B_2
ZShift = −0.52
Z lm Score = 2.27
NG = 0
DB = 0
SM = 0
Delta_AUC
Cytarabine

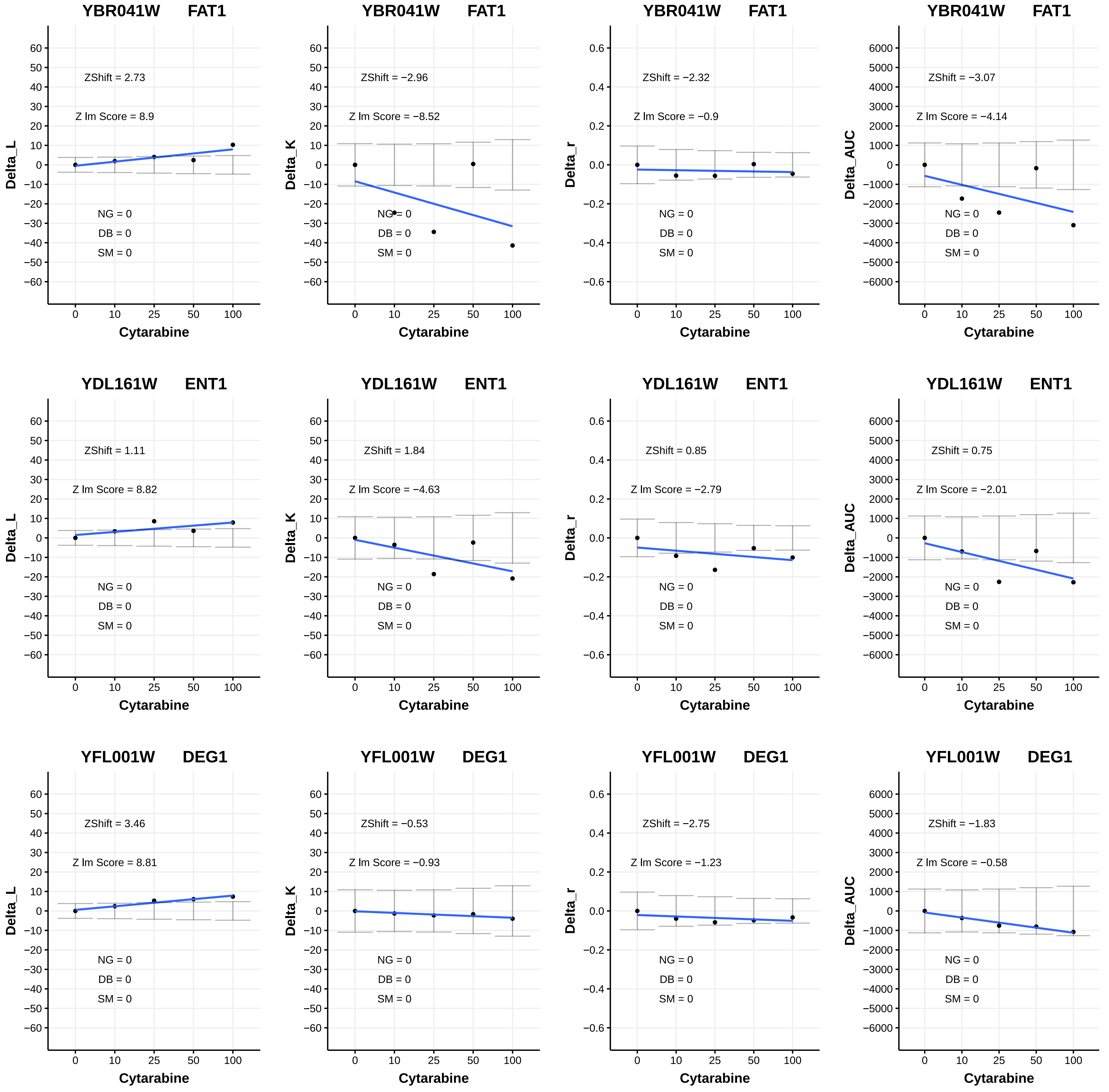

YBR041W FAT1
ZShift = 2.73
Z lm Score = 8.9
NG = 0
DB = 0
SM = 0
Delta_L
Cytarabine
YBR041W FAT1
ZShift = −2.96
Z lm Score = −8.52
NG = 0
DB = 0
SM = 0
Delta_K
Cytarabine
YBR041W FAT1
ZShift = −2.32
Z lm Score = −0.9
NG = 0
DB = 0
SM = 0
Delta_r
Cytarabine
YBR041W FAT1
ZShift = −3.07
Z lm Score = −4.14
NG = 0
DB = 0
SM = 0
Delta_AUC
Cytarabine
YDL161W ENT1
ZShift = 1.11
Z lm Score = 8.82
NG = 0
DB = 0
SM = 0
Delta_L
Cytarabine
YDL161W ENT1
ZShift = 1.84
Z lm Score = −4.63
NG = 0
DB = 0
SM = 0
Delta_K
Cytarabine
YDL161W ENT1
ZShift = 0.85
Z lm Score = −2.79
NG = 0
DB = 0
SM = 0
Delta_r
Cytarabine
YDL161W ENT1
ZShift = 0.75
Z lm Score = −2.01
NG = 0
DB = 0
SM = 0
Delta_AUC
Cytarabine
YFL001W DEG1
ZShift = 3.46
Z lm Score = 8.81
NG = 0
DB = 0
SM = 0
Delta_L
Cytarabine
YFL001W DEG1
ZShift = −0.53
Z lm Score = −0.93
NG = 0
DB = 0
SM = 0
Delta_K
Cytarabine
YFL001W DEG1
ZShift = −2.75
Z lm Score = −1.23
NG = 0
DB = 0
SM = 0
Delta_r
Cytarabine
YFL001W DEG1
ZShift = −1.83
Z lm Score = −0.58
NG = 0
DB = 0
SM = 0
Delta_AUC
Cytarabine

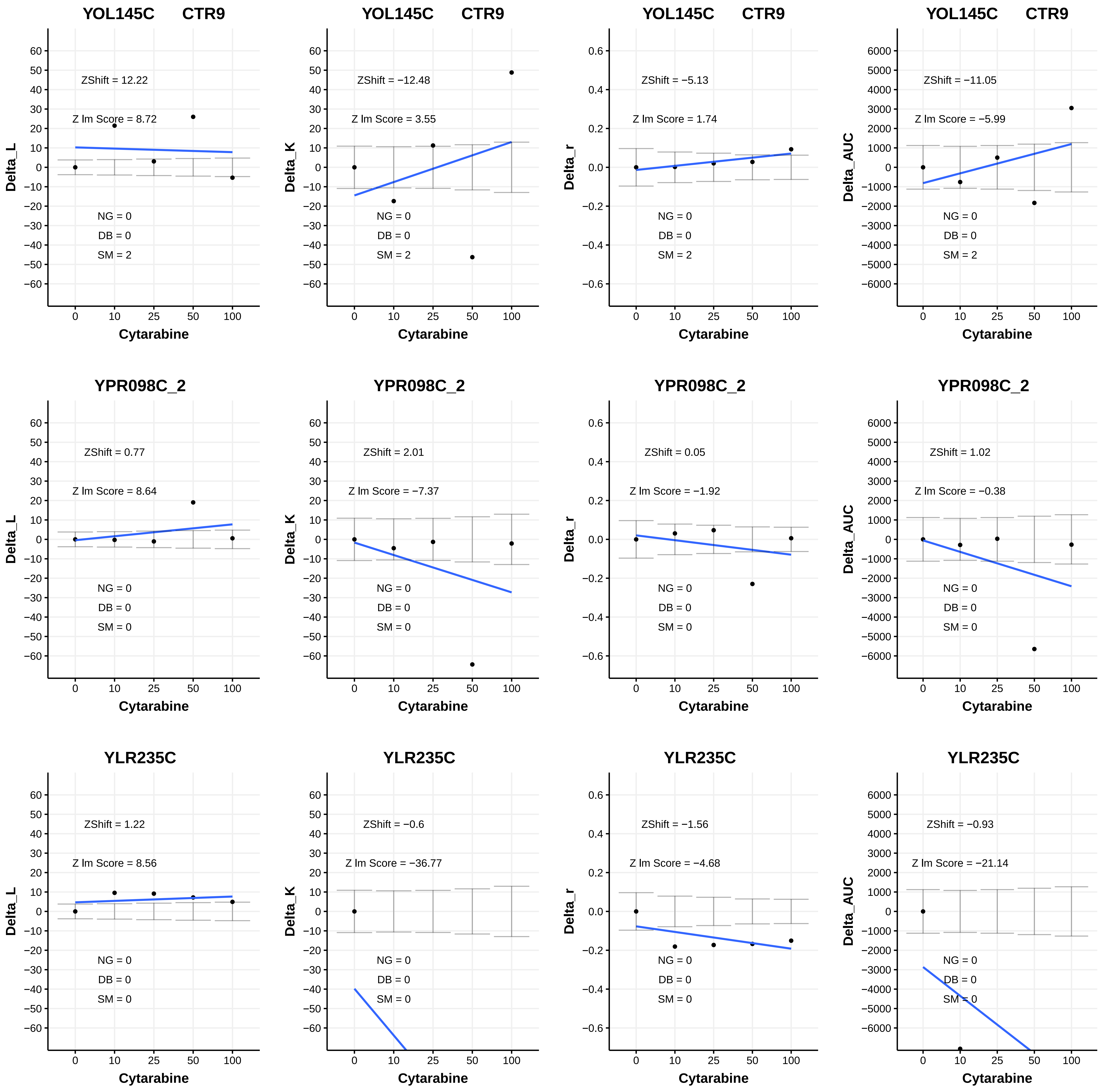

YOL145C CTR9
ZShift = 12.22
Z lm Score = 8.72
NG = 0
DB = 0
SM = 2
Delta_L
Cytarabine
YOL145C CTR9
ZShift = −12.48
Z lm Score = 3.55
NG = 0
DB = 0
SM = 2
Delta_K
Cytarabine
YOL145C CTR9
ZShift = −5.13
Z lm Score = 1.74
NG = 0
DB = 0
SM = 2
Delta_r
Cytarabine
YOL145C CTR9
ZShift = −11.05
Z lm Score = −5.99
NG = 0
DB = 0
SM = 2
Delta_AUC
Cytarabine
YPR098C_2
ZShift = 0.77
Z lm Score = 8.64
NG = 0
DB = 0
SM = 0
Delta_L
Cytarabine
YPR098C_2
ZShift = 2.01
Z lm Score = −7.37
NG = 0
DB = 0
SM = 0
Delta_K
Cytarabine
YPR098C_2
ZShift = 0.05
Z lm Score = −1.92
NG = 0
DB = 0
SM = 0
Delta_r
Cytarabine
YPR098C_2
ZShift = 1.02
Z lm Score = −0.38
NG = 0
DB = 0
SM = 0
Delta_AUC
Cytarabine
YLR235C
ZShift = 1.22
Z lm Score = 8.56
NG = 0
DB = 0
SM = 0
Delta_L
Cytarabine
YLR235C
ZShift = −0.6
Z lm Score = −36.77
NG = 0
DB = 0
SM = 0
Delta_K
Cytarabine
YLR235C
ZShift = −1.56
Z lm Score = −4.68
NG = 0
DB = 0
SM = 0
Delta_r
Cytarabine
YLR235C
ZShift = −0.93
Z lm Score = −21.14
NG = 0
DB = 0
SM = 0
Delta_AUC
Cytarabine

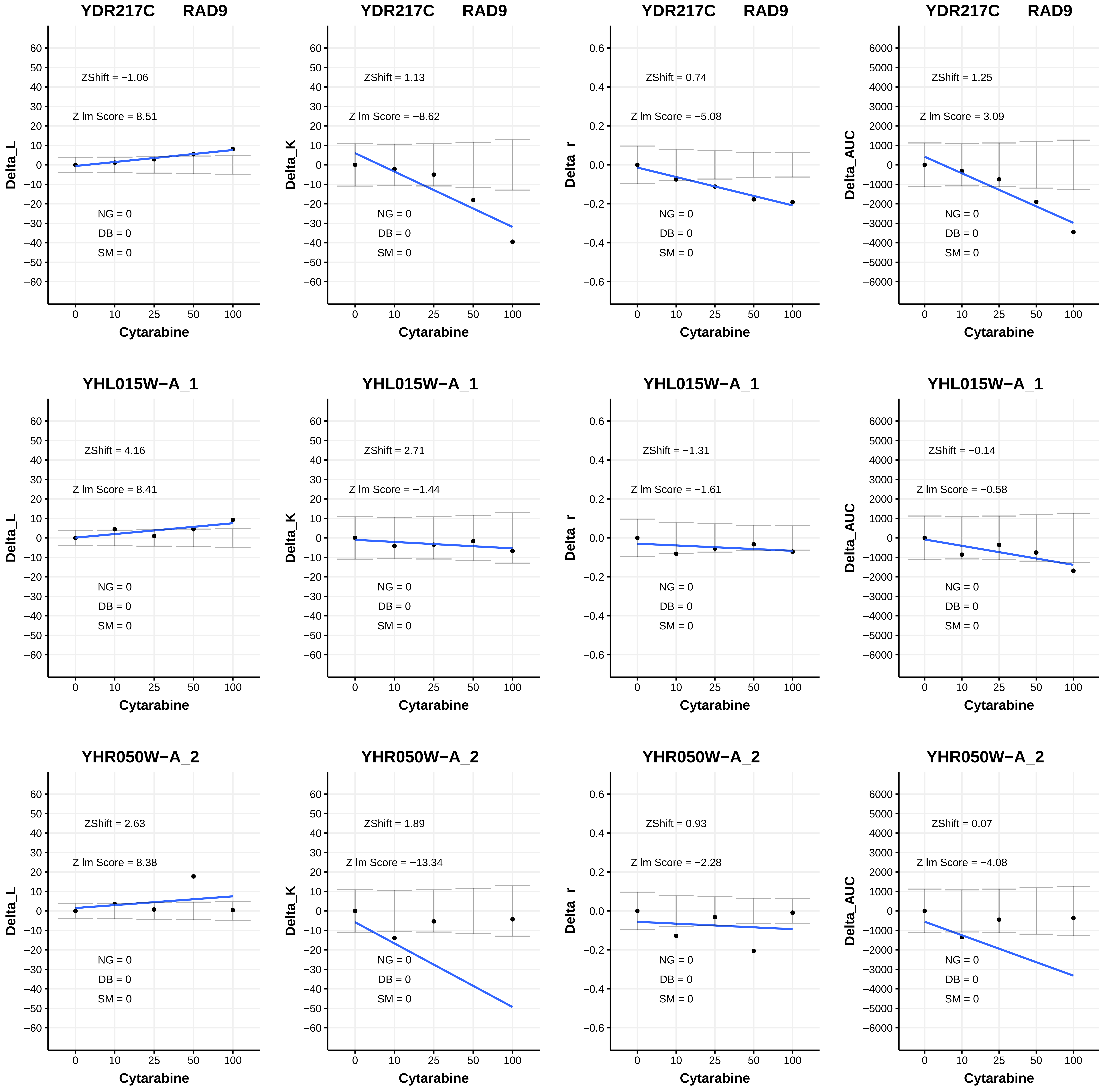

YDR217C RAD9
ZShift = −1.06
Z lm Score = 8.51
NG = 0
DB = 0
SM = 0
Delta_L
Cytarabine
YDR217C RAD9
ZShift = 1.13
Z lm Score = −8.62
NG = 0
DB = 0
SM = 0
Delta_K
Cytarabine
YDR217C RAD9
ZShift = 0.74
Z lm Score = −5.08
NG = 0
DB = 0
SM = 0
Delta_r
Cytarabine
YDR217C RAD9
ZShift = 1.25
Z lm Score = 3.09
NG = 0
DB = 0
SM = 0
Delta_AUC
Cytarabine
YHL015W−A_1
ZShift = 4.16
Z lm Score = 8.41
NG = 0
DB = 0
SM = 0
Delta_L
Cytarabine
YHL015W−A_1
ZShift = 2.71
Z lm Score = −1.44
NG = 0
DB = 0
SM = 0
Delta_K
Cytarabine
YHL015W−A_1
ZShift = −1.31
Z lm Score = −1.61
NG = 0
DB = 0
SM = 0
Delta_r
Cytarabine
YHL015W−A_1
ZShift = −0.14
Z lm Score = −0.58
NG = 0
DB = 0
SM = 0
Delta_AUC
Cytarabine
YHR050W−A_2
ZShift = 2.63
Z lm Score = 8.38
NG = 0
DB = 0
SM = 0
Delta_L
Cytarabine
YHR050W−A_2
ZShift = 1.89
Z lm Score = −13.34
NG = 0
DB = 0
SM = 0
Delta_K
Cytarabine
YHR050W−A_2
ZShift = 0.93
Z lm Score = −2.28
NG = 0
DB = 0
SM = 0
Delta_r
Cytarabine
YHR050W−A_2
ZShift = 0.07
Z lm Score = −4.08
NG = 0
DB = 0
SM = 0
Delta_AUC
Cytarabine

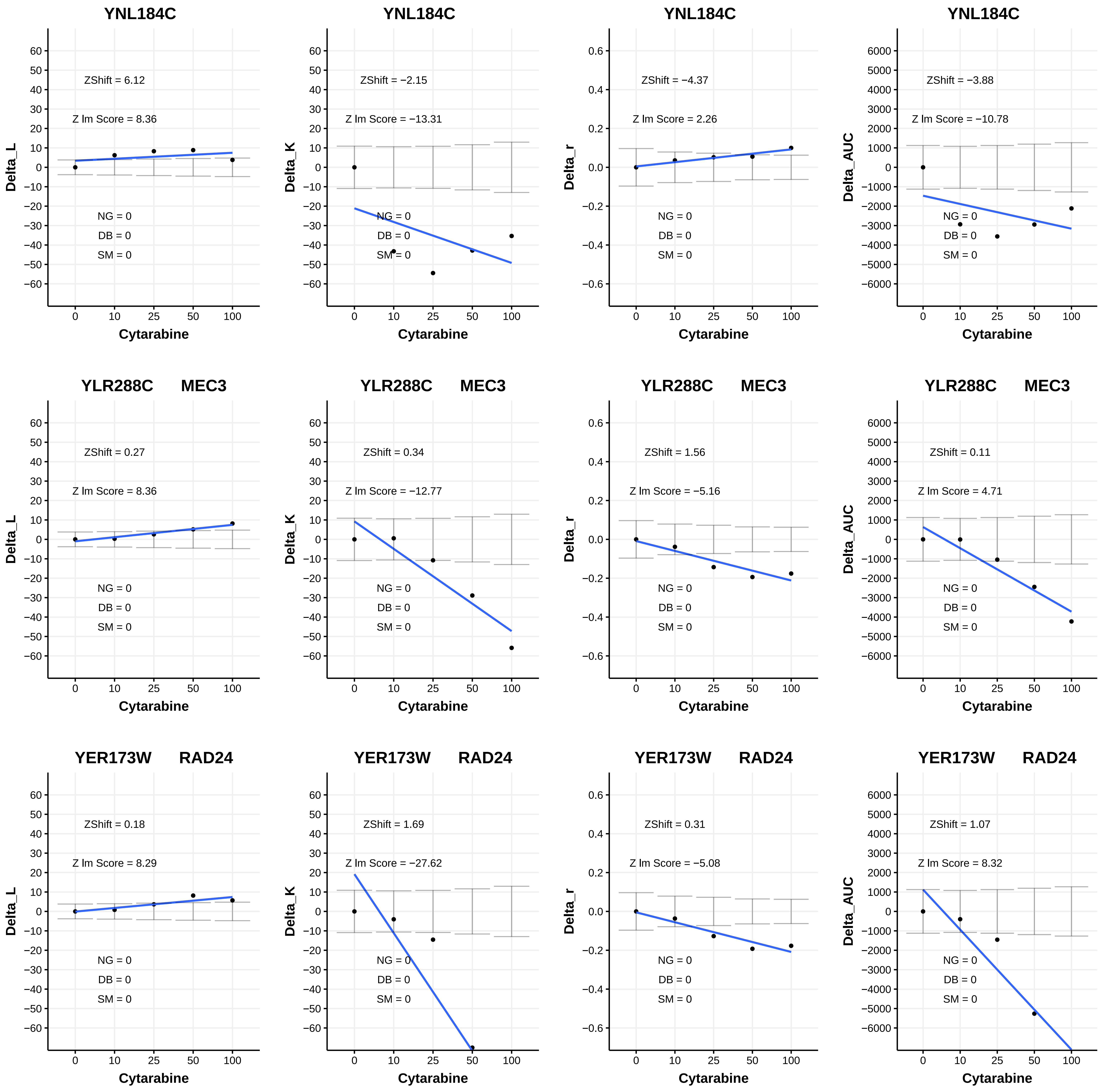
YNL184C
ZShift = 6.12
Z lm Score = 8.36
NG = 0
DB = 0
SM = 0
Delta_L
Cytarabine
YNL184C
ZShift = −2.15
Z lm Score = −13.31
NG = 0
DB = 0
SM = 0
Delta_K
Cytarabine
YNL184C
ZShift = −4.37
Z lm Score = 2.26
NG = 0
DB = 0
SM = 0
Delta_r
Cytarabine
YNL184C
ZShift = −3.88
Z lm Score = −10.78
NG = 0
DB = 0
SM = 0
Delta_AUC
Cytarabine
YLR288C MEC3
ZShift = 0.27
Z lm Score = 8.36
NG = 0
DB = 0
SM = 0
Delta_L
Cytarabine
YLR288C MEC3
ZShift = 0.34
Z lm Score = −12.77
NG = 0
DB = 0
SM = 0
Delta_K
Cytarabine
YLR288C MEC3
ZShift = 1.56
Z lm Score = −5.16
NG = 0
DB = 0
SM = 0
Delta_r
Cytarabine
YLR288C MEC3
ZShift = 0.11
Z lm Score = 4.71
NG = 0
DB = 0
SM = 0
Delta_AUC
Cytarabine
YER173W RAD24
ZShift = 0.18
Z lm Score = 8.29
NG = 0
DB = 0
SM = 0
Delta_L
Cytarabine
YER173W RAD24
ZShift = 1.69
Z lm Score = −27.62
NG = 0
DB = 0
SM = 0
Delta_K
Cytarabine
YER173W RAD24
ZShift = 0.31
Z lm Score = −5.08
NG = 0
DB = 0
SM = 0
Delta_r
Cytarabine
YER173W RAD24
ZShift = 1.07
Z lm Score = 8.32
NG = 0
DB = 0
SM = 0
Delta_AUC
Cytarabine

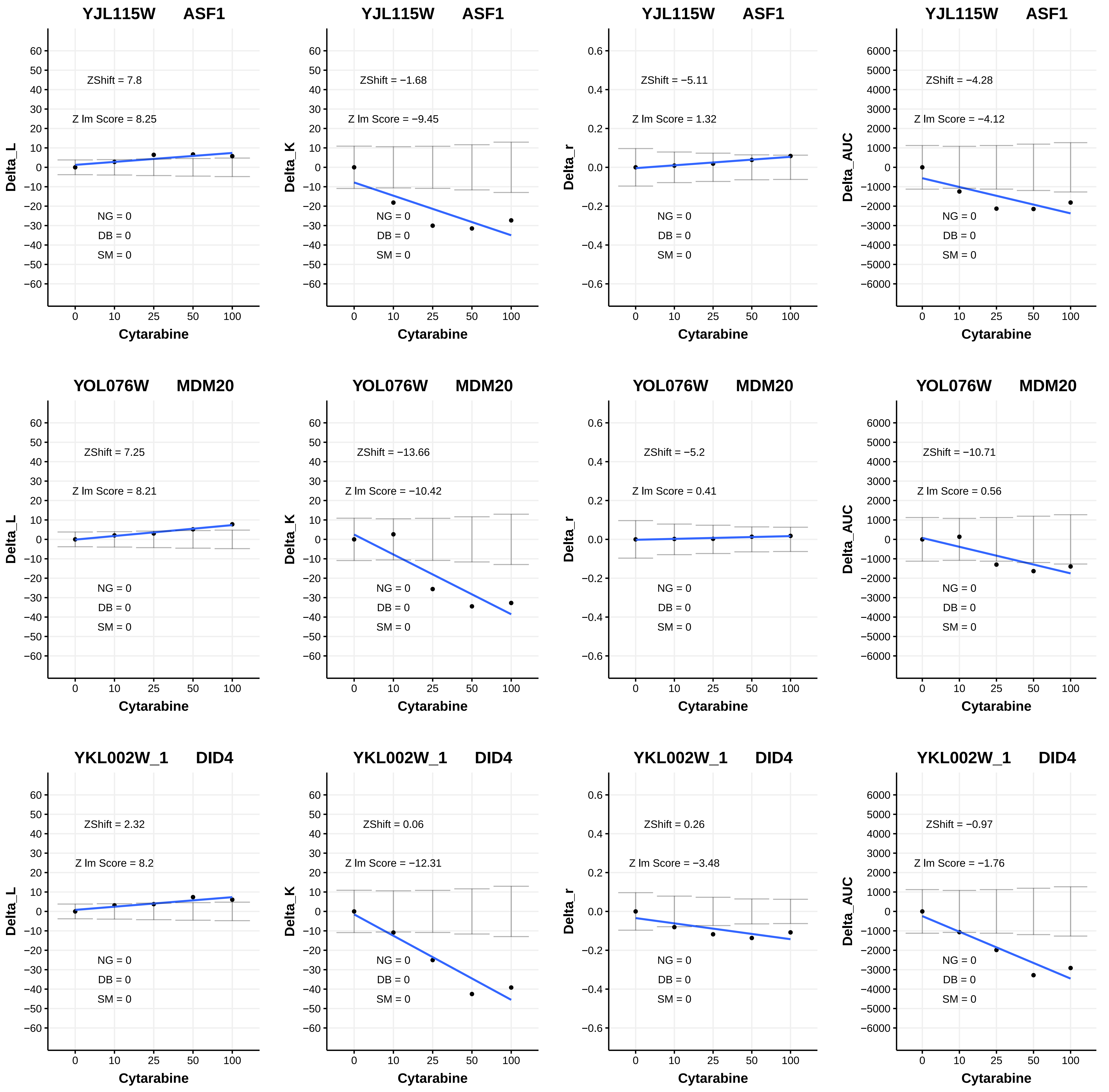

YJL115W ASF1
ZShift = 7.8
Z lm Score = 8.25
Delta_L
NG = 0
DB = 0
SM = 0
Cytarabine
YJL115W ASF1
ZShift = −1.68
Z lm Score = −9.45
Delta_K
NG = 0
DB = 0
SM = 0
Cytarabine
YJL115W ASF1
ZShift = −5.11
Z lm Score = 1.32
Delta_r
NG = 0
DB = 0
SM = 0
Cytarabine
YJL115W ASF1
ZShift = −4.28
Z lm Score = −4.12
Delta_AUC
NG = 0
DB = 0
SM = 0
Cytarabine
YOL076W MDM20
ZShift = 7.25
Z lm Score = 8.21
Delta_L
NG = 0
DB = 0
SM = 0
Cytarabine
YOL076W MDM20
ZShift = −13.66
Z lm Score = −10.42
Delta_K
NG = 0
DB = 0
SM = 0
Cytarabine
YOL076W MDM20
ZShift = −5.2
Z lm Score = 0.41
Delta_r
NG = 0
DB = 0
SM = 0
Cytarabine
YOL076W MDM20
ZShift = −10.71
Z lm Score = 0.56
Delta_AUC
NG = 0
DB = 0
SM = 0
Cytarabine
YKL002W_1 DID4
ZShift = 2.32
Z lm Score = 8.2
Delta_L
NG = 0
DB = 0
SM = 0
Cytarabine
YKL002W_1 DID4
ZShift = 0.06
Z lm Score = −12.31
Delta_K
NG = 0
DB = 0
SM = 0
Cytarabine
YKL002W_1 DID4
ZShift = 0.26
Z lm Score = −3.48
Delta_r
NG = 0
DB = 0
SM = 0
Cytarabine
YKL002W_1 DID4
ZShift = −0.97
Z lm Score = −1.76
Delta_AUC
NG = 0
DB = 0
SM = 0
Cytarabine

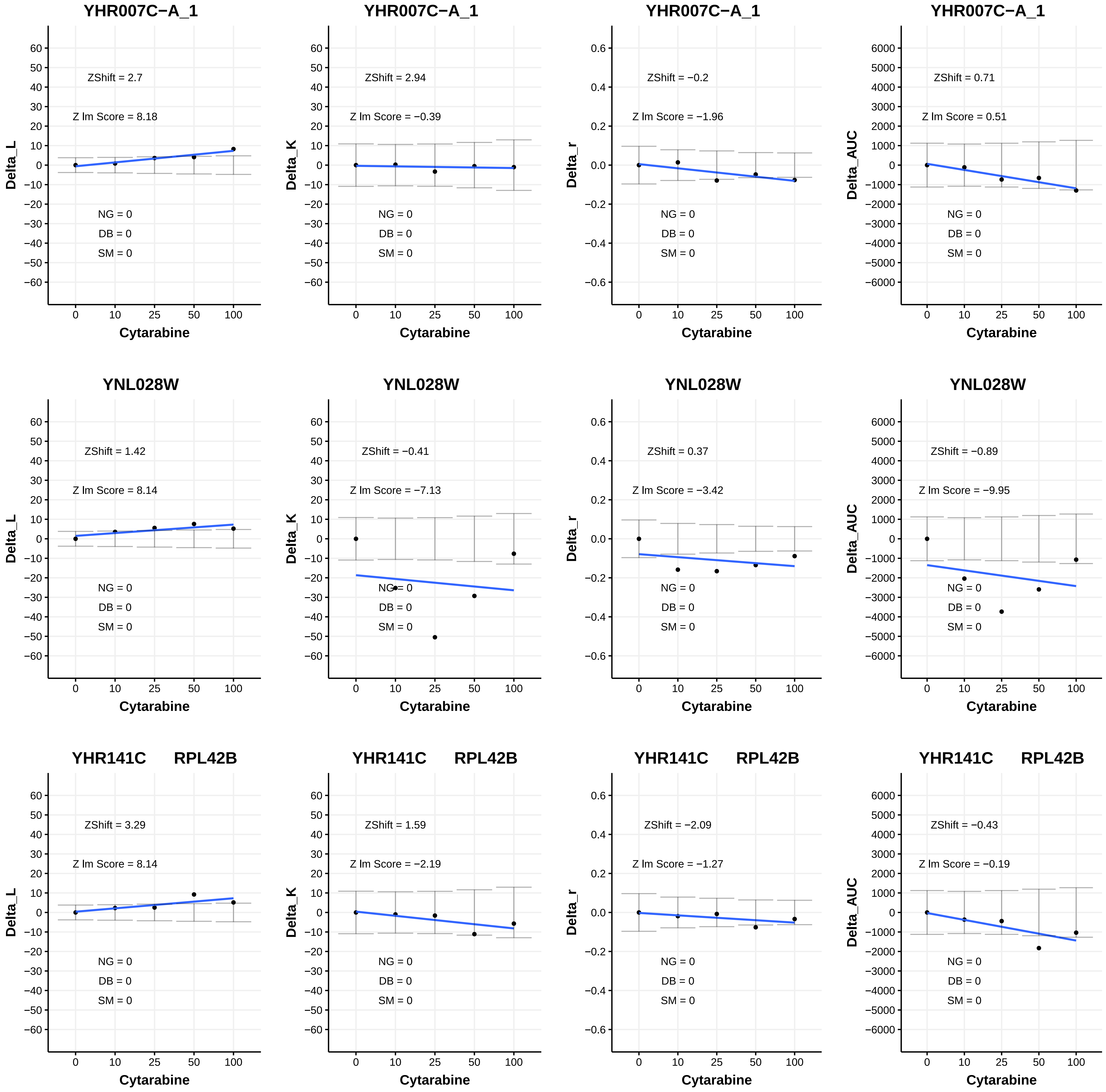

YHR007C−A_1
ZShift = 2.7
Z lm Score = 8.18
NG = 0
DB = 0
SM = 0
Delta_L
YHR007C−A_1
ZShift = 2.94
Z lm Score = −0.39
NG = 0
DB = 0
SM = 0
Delta_K
YHR007C−A_1
ZShift = −0.2
Z lm Score = −1.96
NG = 0
DB = 0
SM = 0
Delta_r
YHR007C−A_1
ZShift = 0.71
Z lm Score = 0.51
NG = 0
DB = 0
SM = 0
Delta_AUC
YNL028W
ZShift = 1.42
Z lm Score = 8.14
NG = 0
DB = 0
SM = 0
Delta_L
YNL028W
ZShift = −0.41
Z lm Score = −7.13
NG = 0
DB = 0
SM = 0
Delta_K
YNL028W
ZShift = 0.37
Z lm Score = −3.42
NG = 0
DB = 0
SM = 0
Delta_r
YNL028W
ZShift = −0.89
Z lm Score = −9.95
NG = 0
DB = 0
SM = 0
Delta_AUC
YHR141C RPL42B
ZShift = 3.29
Z lm Score = 8.14
NG = 0
DB = 0
SM = 0
Delta_L
YHR141C RPL42B
ZShift = 1.59
Z lm Score = −2.19
NG = 0
DB = 0
SM = 0
Delta_K
YHR141C RPL42B
ZShift = −2.09
Z lm Score = −1.27
NG = 0
DB = 0
SM = 0
Delta_r
YHR141C RPL42B
ZShift = −0.43
Z lm Score = −0.19
NG = 0
DB = 0
SM = 0
Delta_AUC
0 10 25 50 100
Cytarabine

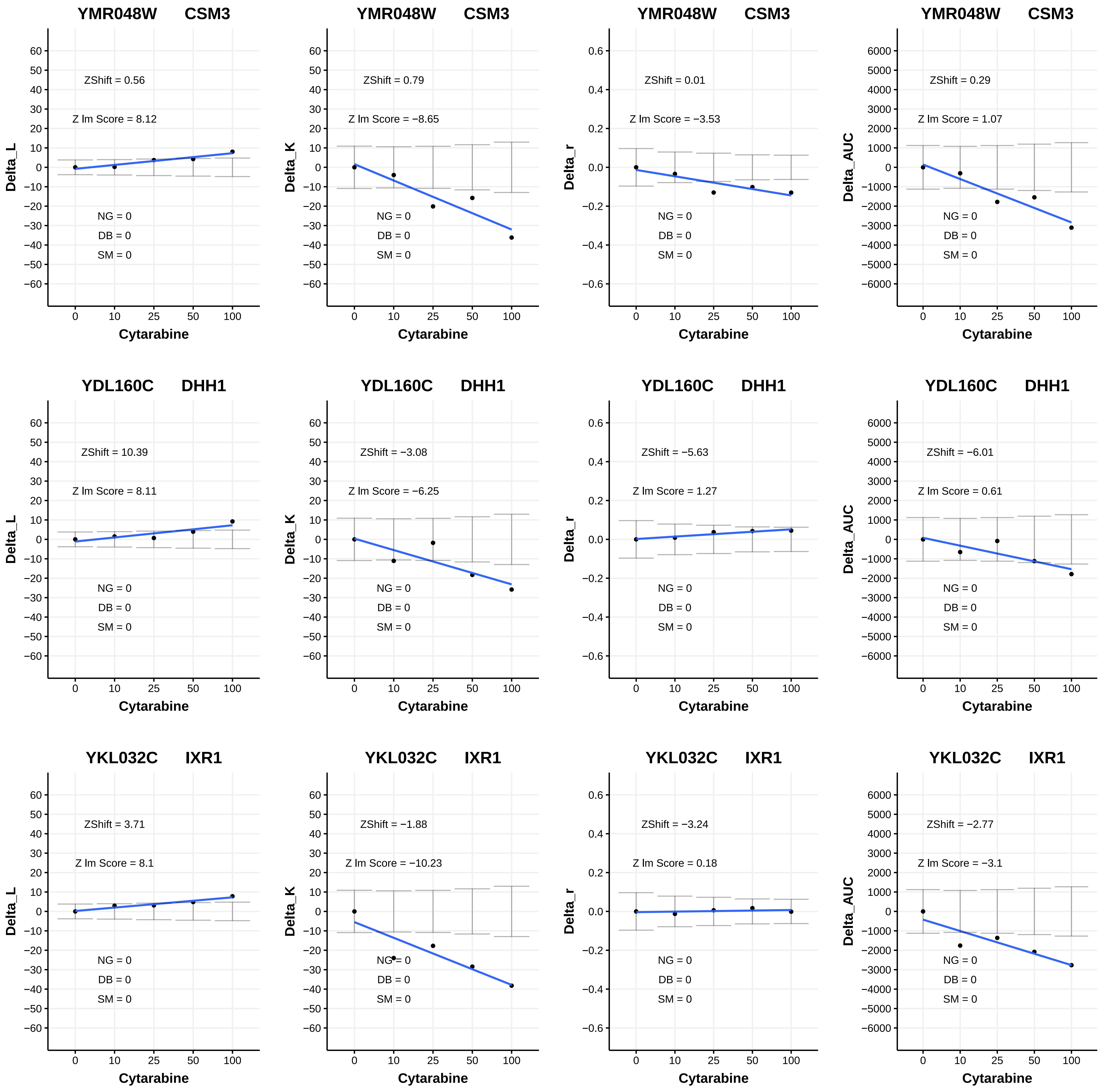

YMR048W CSM3
ZShift = 0.56
Z lm Score = 8.12
NG = 0
DB = 0
SM = 0
Delta_L
Cytarabine
YMR048W CSM3
ZShift = 0.79
Z lm Score = −8.65
NG = 0
DB = 0
SM = 0
Delta_K
Cytarabine
YMR048W CSM3
ZShift = 0.01
Z lm Score = −3.53
NG = 0
DB = 0
SM = 0
Delta_r
Cytarabine
YMR048W CSM3
ZShift = 0.29
Z lm Score = 1.07
NG = 0
DB = 0
SM = 0
Delta_AUC
Cytarabine
YDL160C DHH1
ZShift = 10.39
Z lm Score = 8.11
NG = 0
DB = 0
SM = 0
Delta_L
Cytarabine
YDL160C DHH1
ZShift = −3.08
Z lm Score = −6.25
NG = 0
DB = 0
SM = 0
Delta_K
Cytarabine
YDL160C DHH1
ZShift = −5.63
Z lm Score = 1.27
NG = 0
DB = 0
SM = 0
Delta_r
Cytarabine
YDL160C DHH1
ZShift = −6.01
Z lm Score = 0.61
NG = 0
DB = 0
SM = 0
Delta_AUC
Cytarabine
YKL032C IXR1
ZShift = 3.71
Z lm Score = 8.1
NG = 0
DB = 0
SM = 0
Delta_L
Cytarabine
YKL032C IXR1
ZShift = −1.88
Z lm Score = −10.23
NG = 0
DB = 0
SM = 0
Delta_K
Cytarabine
YKL032C IXR1
ZShift = −3.24
Z lm Score = 0.18
NG = 0
DB = 0
SM = 0
Delta_r
Cytarabine
YKL032C IXR1
ZShift = −2.77
Z lm Score = −3.1
NG = 0
DB = 0
SM = 0
Delta_AUC
Cytarabine

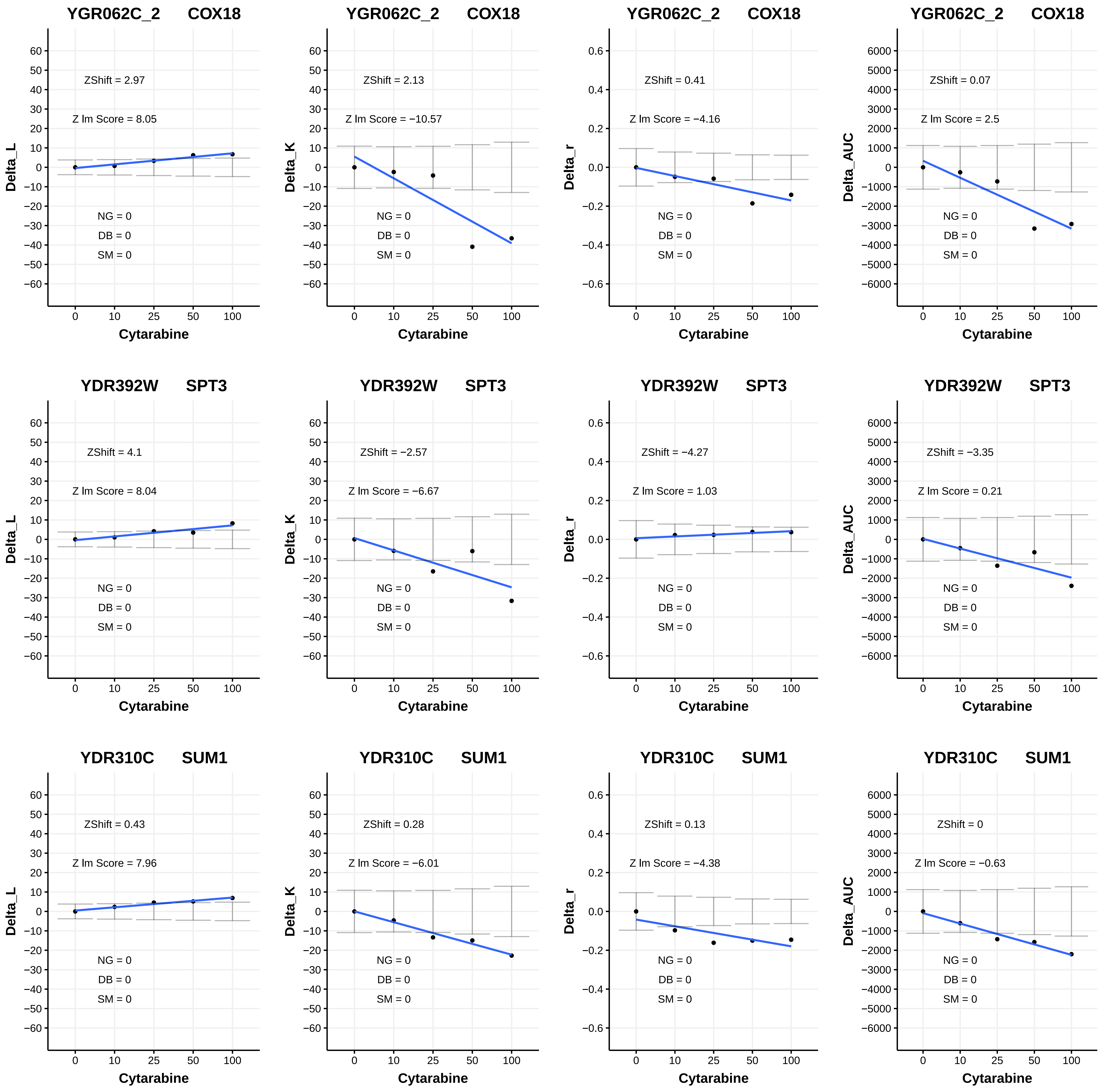
YGR062C_2 COX18
ZShift = 2.97
Z lm Score = 8.05
NG = 0
DB = 0
SM = 0
Delta_L
Cytarabine
YGR062C_2 COX18
ZShift = 2.13
Z lm Score = −10.57
NG = 0
DB = 0
SM = 0
Delta_K
Cytarabine
YGR062C_2 COX18
ZShift = 0.41
Z lm Score = −4.16
NG = 0
DB = 0
SM = 0
Delta_r
Cytarabine
YGR062C_2 COX18
ZShift = 0.07
Z lm Score = 2.5
NG = 0
DB = 0
SM = 0
Delta_AUC
Cytarabine
YDR392W SPT3
ZShift = 4.1
Z lm Score = 8.04
NG = 0
DB = 0
SM = 0
Delta_L
Cytarabine
YDR392W SPT3
ZShift = −2.57
Z lm Score = −6.67
NG = 0
DB = 0
SM = 0
Delta_K
Cytarabine
YDR392W SPT3
ZShift = −4.27
Z lm Score = 1.03
NG = 0
DB = 0
SM = 0
Delta_r
Cytarabine
YDR392W SPT3
ZShift = −3.35
Z lm Score = 0.21
NG = 0
DB = 0
SM = 0
Delta_AUC
Cytarabine
YDR310C SUM1
ZShift = 0.43
Z lm Score = 7.96
NG = 0
DB = 0
SM = 0
Delta_L
Cytarabine
YDR310C SUM1
ZShift = 0.28
Z lm Score = −6.01
NG = 0
DB = 0
SM = 0
Delta_K
Cytarabine
YDR310C SUM1
ZShift = 0.13
Z lm Score = −4.38
NG = 0
DB = 0
SM = 0
Delta_r
Cytarabine
YDR310C SUM1
ZShift = 0
Z lm Score = −0.63
NG = 0
DB = 0
SM = 0
Delta_AUC
Cytarabine

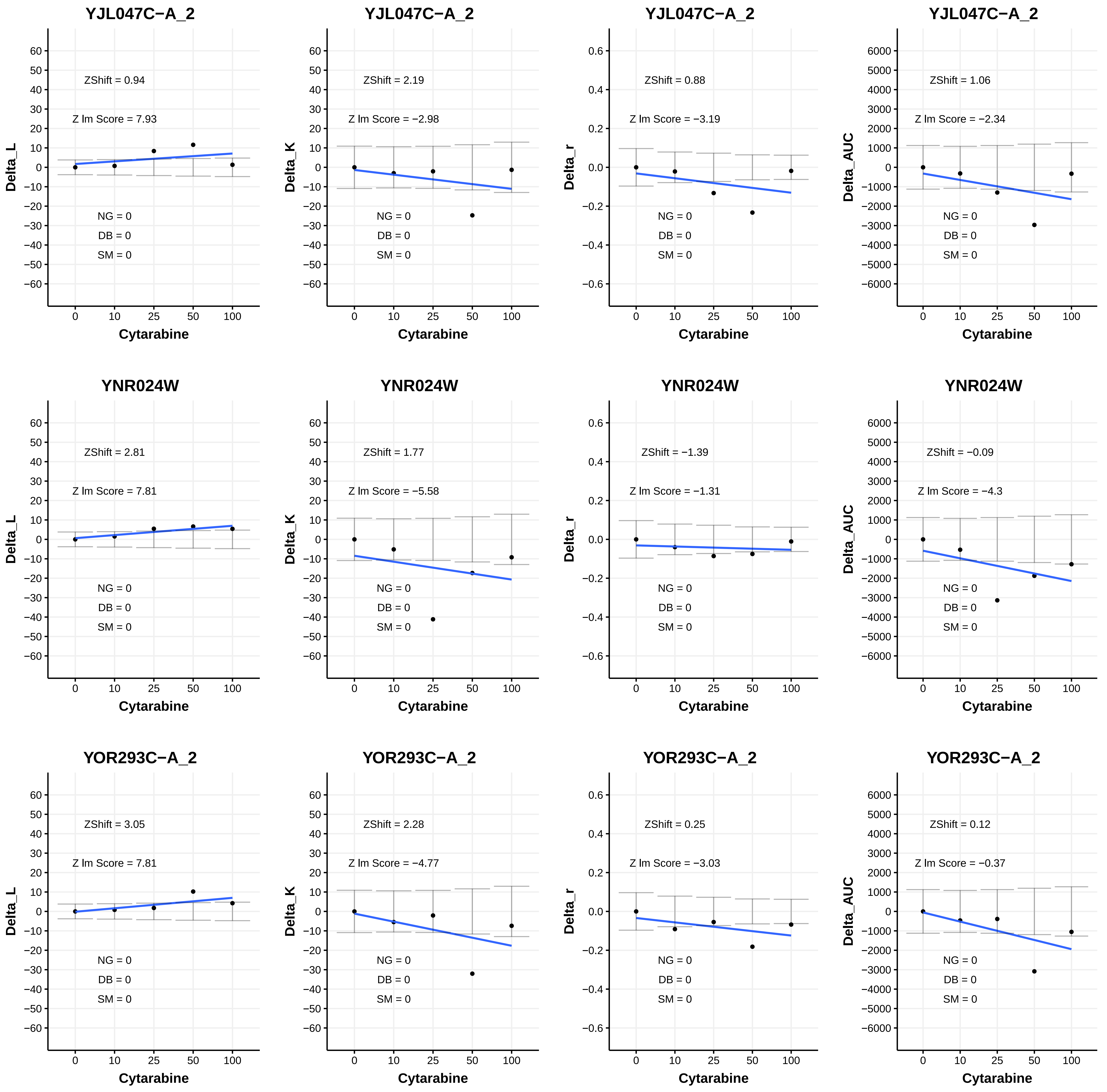
YJL047C−A_2
ZShift = 0.94
Z lm Score = 7.93
NG = 0
DB = 0
SM = 0
Delta_L
Cytarabine
YJL047C−A_2
ZShift = 2.19
Z lm Score = −2.98
NG = 0
DB = 0
SM = 0
Delta_K
Cytarabine
YJL047C−A_2
ZShift = 0.88
Z lm Score = −3.19
NG = 0
DB = 0
SM = 0
Delta_r
Cytarabine
YJL047C−A_2
ZShift = 1.06
Z lm Score = −2.34
NG = 0
DB = 0
SM = 0
Delta_AUC
Cytarabine
YNR024W
ZShift = 2.81
Z lm Score = 7.81
NG = 0
DB = 0
SM = 0
Delta_L
Cytarabine
YNR024W
ZShift = 1.77
Z lm Score = −5.58
NG = 0
DB = 0
SM = 0
Delta_K
Cytarabine
YNR024W
ZShift = −1.39
Z lm Score = −1.31
NG = 0
DB = 0
SM = 0
Delta_r
Cytarabine
YNR024W
ZShift = −0.09
Z lm Score = −4.3
NG = 0
DB = 0
SM = 0
Delta_AUC
Cytarabine
YOR293C−A_2
ZShift = 3.05
Z lm Score = 7.81
NG = 0
DB = 0
SM = 0
Delta_L
Cytarabine
YOR293C−A_2
ZShift = 2.28
Z lm Score = −4.77
NG = 0
DB = 0
SM = 0
Delta_K
Cytarabine
YOR293C−A_2
ZShift = 0.25
Z lm Score = −3.03
NG = 0
DB = 0
SM = 0
Delta_r
Cytarabine
YOR293C−A_2
ZShift = 0.12
Z lm Score = −0.37
NG = 0
DB = 0
SM = 0
Delta_AUC
Cytarabine

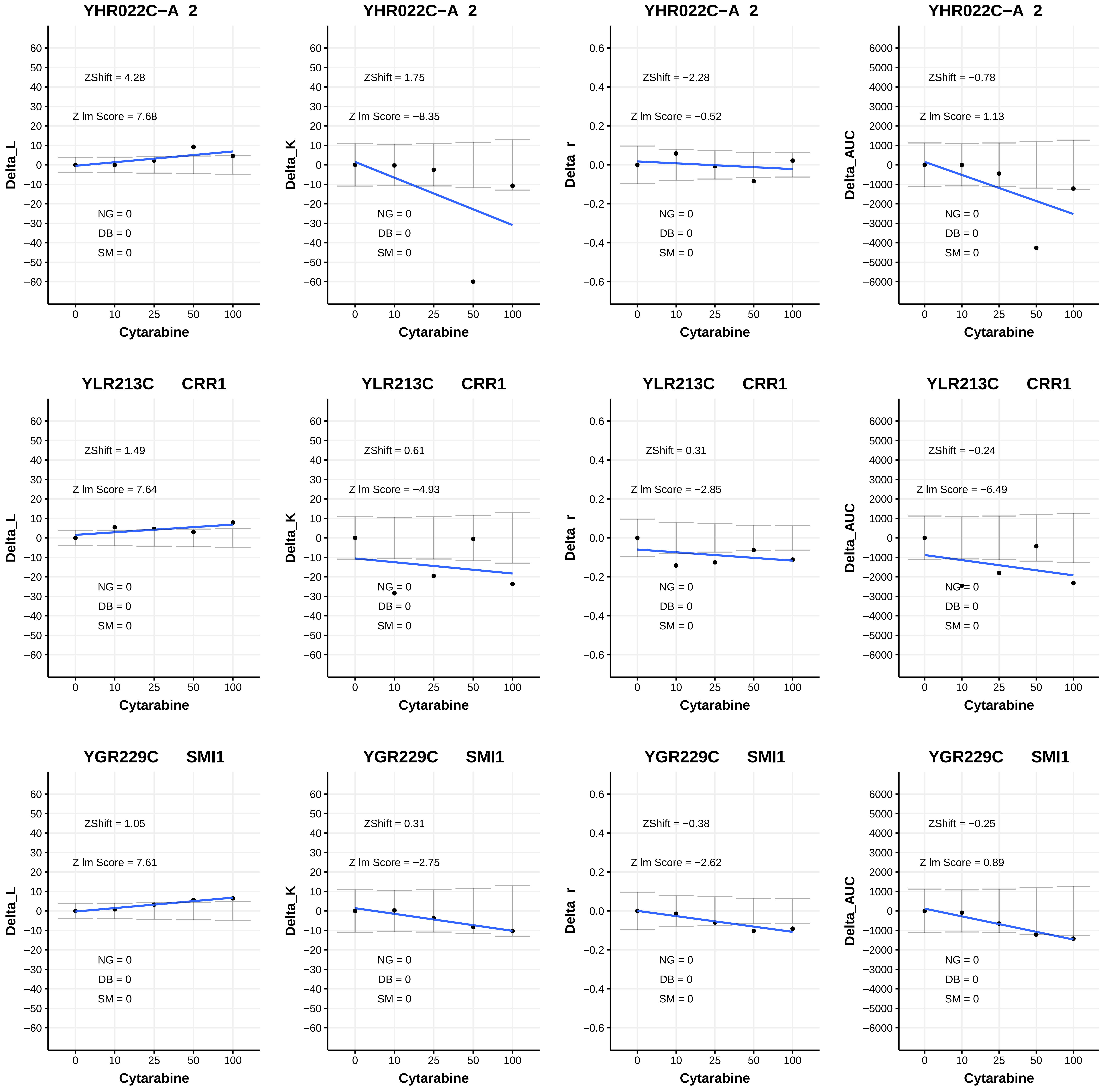

YHR022C−A_2
ZShift = 4.28
Z lm Score = 7.68
NG = 0
DB = 0
SM = 0
Delta_L
Cytarabine
YHR022C−A_2
ZShift = 1.75
Z lm Score = −8.35
NG = 0
DB = 0
SM = 0
Delta_K
Cytarabine
YHR022C−A_2
ZShift = −2.28
Z lm Score = −0.52
NG = 0
DB = 0
SM = 0
Delta_r
Cytarabine
YHR022C−A_2
ZShift = −0.78
Z lm Score = 1.13
NG = 0
DB = 0
SM = 0
Delta_AUC
Cytarabine
YLR213C CRR1
ZShift = 1.49
Z lm Score = 7.64
NG = 0
DB = 0
SM = 0
Delta_L
Cytarabine
YLR213C CRR1
ZShift = 0.61
Z lm Score = −4.93
NG = 0
DB = 0
SM = 0
Delta_K
Cytarabine
YLR213C CRR1
ZShift = 0.31
Z lm Score = −2.85
NG = 0
DB = 0
SM = 0
Delta_r
Cytarabine
YLR213C CRR1
ZShift = −0.24
Z lm Score = −6.49
NG = 0
DB = 0
SM = 0
Delta_AUC
Cytarabine
YGR229C SMI1
ZShift = 1.05
Z lm Score = 7.61
NG = 0
DB = 0
SM = 0
Delta_L
Cytarabine
YGR229C SMI1
ZShift = 0.31
Z lm Score = −2.75
NG = 0
DB = 0
SM = 0
Delta_K
Cytarabine
YGR229C SMI1
ZShift = −0.38
Z lm Score = −2.62
NG = 0
DB = 0
SM = 0
Delta_r
Cytarabine
YGR229C SMI1
ZShift = −0.25
Z lm Score = 0.89
NG = 0
DB = 0
SM = 0
Delta_AUC
Cytarabine

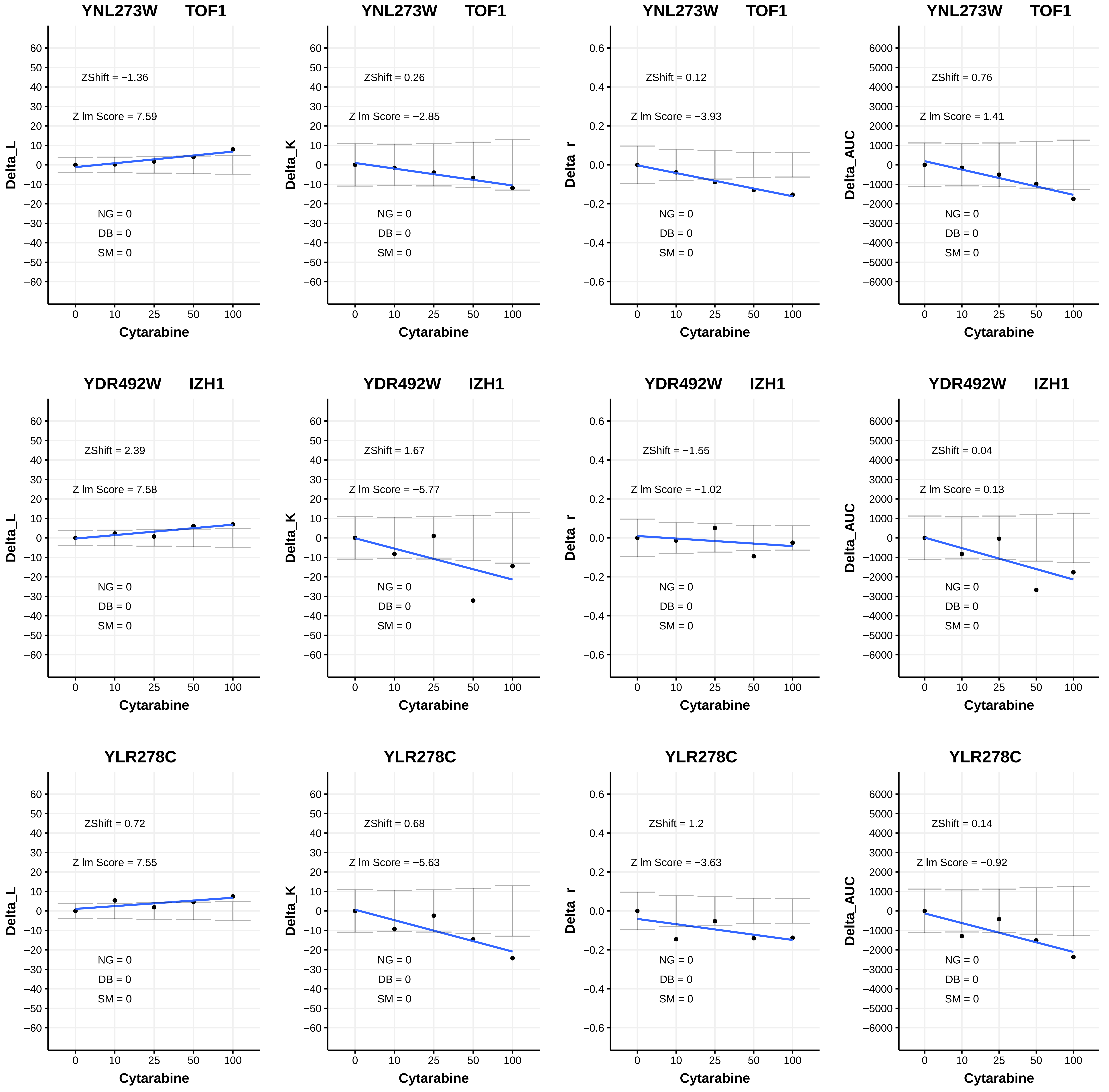
YNL273W TOF1
ZShift = −1.36
Z lm Score = 7.59
NG = 0
DB = 0
SM = 0
Delta_L
Cytarabine
YNL273W TOF1
ZShift = 0.26
Z lm Score = −2.85
NG = 0
DB = 0
SM = 0
Delta_K
Cytarabine
YNL273W TOF1
ZShift = 0.12
Z lm Score = −3.93
NG = 0
DB = 0
SM = 0
Delta_r
Cytarabine
YNL273W TOF1
ZShift = 0.76
Z lm Score = 1.41
NG = 0
DB = 0
SM = 0
Delta_AUC
Cytarabine
YDR492W IZH1
ZShift = 2.39
Z lm Score = 7.58
NG = 0
DB = 0
SM = 0
Delta_L
Cytarabine
YDR492W IZH1
ZShift = 1.67
Z lm Score = −5.77
NG = 0
DB = 0
SM = 0
Delta_K
Cytarabine
YDR492W IZH1
ZShift = −1.55
Z lm Score = −1.02
NG = 0
DB = 0
SM = 0
Delta_r
Cytarabine
YDR492W IZH1
ZShift = 0.04
Z lm Score = 0.13
NG = 0
DB = 0
SM = 0
Delta_AUC
Cytarabine
YLR278C
ZShift = 0.72
Z lm Score = 7.55
NG = 0
DB = 0
SM = 0
Delta_L
Cytarabine
YLR278C
ZShift = 0.68
Z lm Score = −5.63
NG = 0
DB = 0
SM = 0
Delta_K
Cytarabine
YLR278C
ZShift = 1.2
Z lm Score = −3.63
NG = 0
DB = 0
SM = 0
Delta_r
Cytarabine
YLR278C
ZShift = 0.14
Z lm Score = −0.92
NG = 0
DB = 0
SM = 0
Delta_AUC
Cytarabine

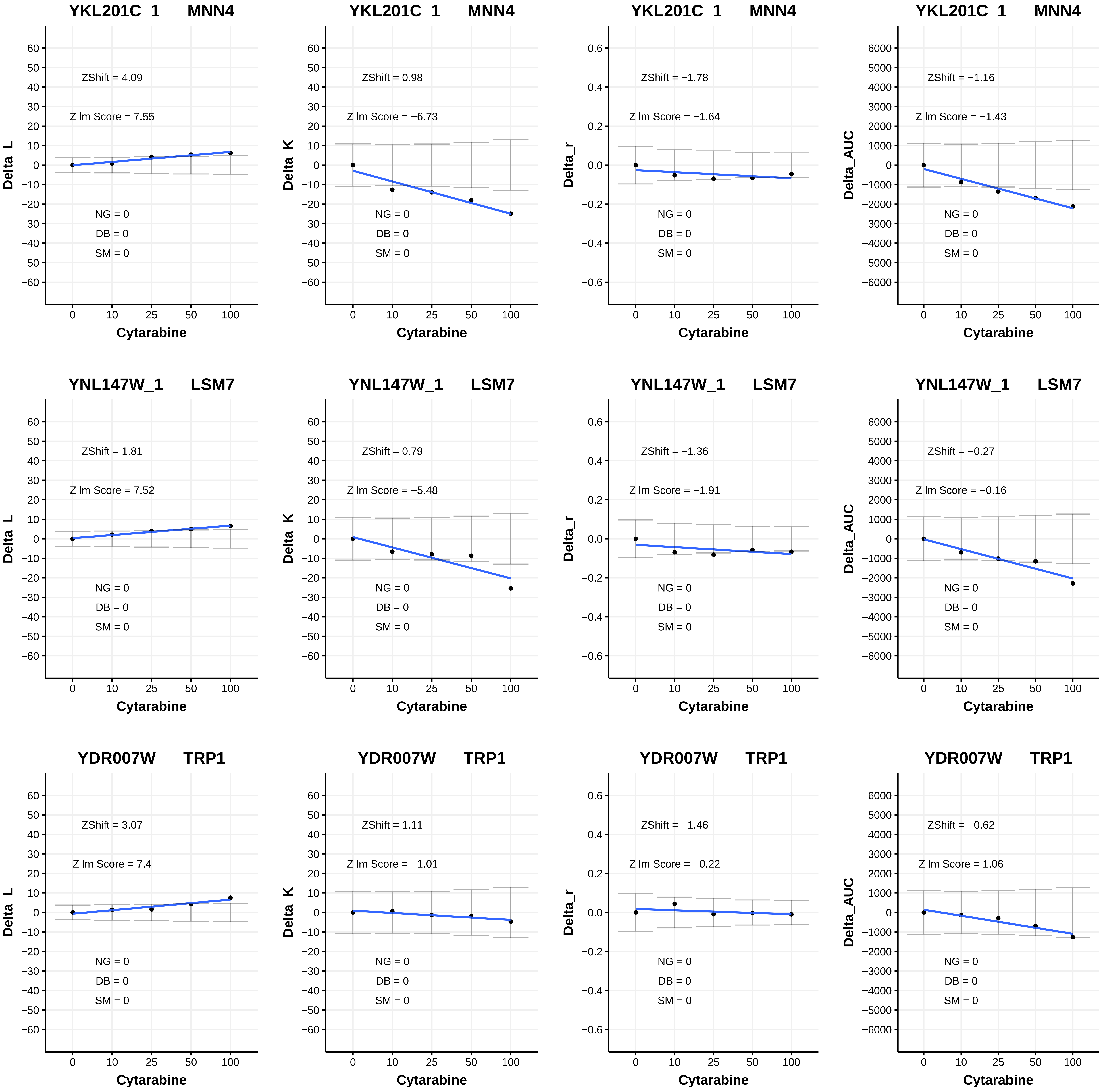
YKL201C_1 MNN4
ZShift = 4.09
Z lm Score = 7.55
NG = 0
DB = 0
SM = 0
Delta_L
Cytarabine
YKL201C_1 MNN4
ZShift = 0.98
Z lm Score = −6.73
NG = 0
DB = 0
SM = 0
Delta_K
Cytarabine
YKL201C_1 MNN4
ZShift = −1.78
Z lm Score = −1.64
NG = 0
DB = 0
SM = 0
Delta_r
Cytarabine
YKL201C_1 MNN4
ZShift = −1.16
Z lm Score = −1.43
NG = 0
DB = 0
SM = 0
Delta_AUC
Cytarabine
YNL147W_1 LSM7
ZShift = 1.81
Z lm Score = 7.52
NG = 0
DB = 0
SM = 0
Delta_L
Cytarabine
YNL147W_1 LSM7
ZShift = 0.79
Z lm Score = −5.48
NG = 0
DB = 0
SM = 0
Delta_K
Cytarabine
YNL147W_1 LSM7
ZShift = −1.36
Z lm Score = −1.91
NG = 0
DB = 0
SM = 0
Delta_r
Cytarabine
YNL147W_1 LSM7
ZShift = −0.27
Z lm Score = −0.16
NG = 0
DB = 0
SM = 0
Delta_AUC
Cytarabine
YDR007W TRP1
ZShift = 3.07
Z lm Score = 7.4
NG = 0
DB = 0
SM = 0
Delta_L
Cytarabine
YDR007W TRP1
ZShift = 1.11
Z lm Score = −1.01
NG = 0
DB = 0
SM = 0
Delta_K
Cytarabine
YDR007W TRP1
ZShift = −1.46
Z lm Score = −0.22
NG = 0
DB = 0
SM = 0
Delta_r
Cytarabine
YDR007W TRP1
ZShift = −0.62
Z lm Score = 1.06
NG = 0
DB = 0
SM = 0
Delta_AUC
Cytarabine

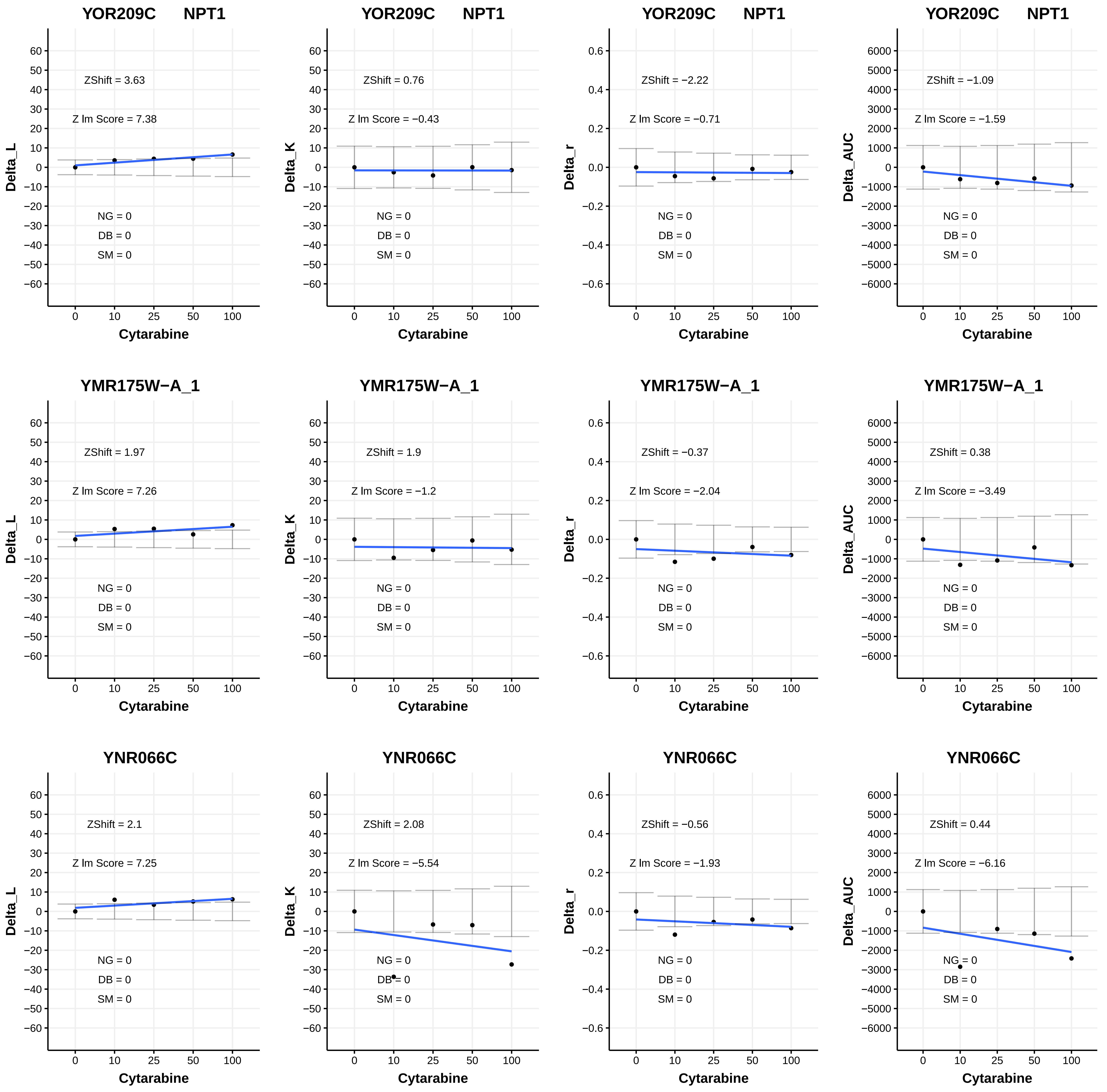
YOR209C NPT1
ZShift = 3.63
Z lm Score = 7.38
NG = 0
DB = 0
SM = 0
Delta_L
Cytarabine
YOR209C NPT1
ZShift = 0.76
Z lm Score = −0.43
NG = 0
DB = 0
SM = 0
Delta_K
Cytarabine
YOR209C NPT1
ZShift = −2.22
Z lm Score = −0.71
NG = 0
DB = 0
SM = 0
Delta_r
Cytarabine
YOR209C NPT1
ZShift = −1.09
Z lm Score = −1.59
NG = 0
DB = 0
SM = 0
Delta_AUC
Cytarabine
YMR175W−A_1
ZShift = 1.97
Z lm Score = 7.26
NG = 0
DB = 0
SM = 0
Delta_L
Cytarabine
YMR175W−A_1
ZShift = 1.9
Z lm Score = −1.2
NG = 0
DB = 0
SM = 0
Delta_K
Cytarabine
YMR175W−A_1
ZShift = −0.37
Z lm Score = −2.04
NG = 0
DB = 0
SM = 0
Delta_r
Cytarabine
YMR175W−A_1
ZShift = 0.38
Z lm Score = −3.49
NG = 0
DB = 0
SM = 0
Delta_AUC
Cytarabine
YNR066C
ZShift = 2.1
Z lm Score = 7.25
NG = 0
DB = 0
SM = 0
Delta_L
Cytarabine
YNR066C
ZShift = 2.08
Z lm Score = −5.54
NG = 0
DB = 0
SM = 0
Delta_K
Cytarabine
YNR066C
ZShift = −0.56
Z lm Score = −1.93
NG = 0
DB = 0
SM = 0
Delta_r
Cytarabine
YNR066C
ZShift = 0.44
Z lm Score = −6.16
NG = 0
DB = 0
SM = 0
Delta_AUC
Cytarabine

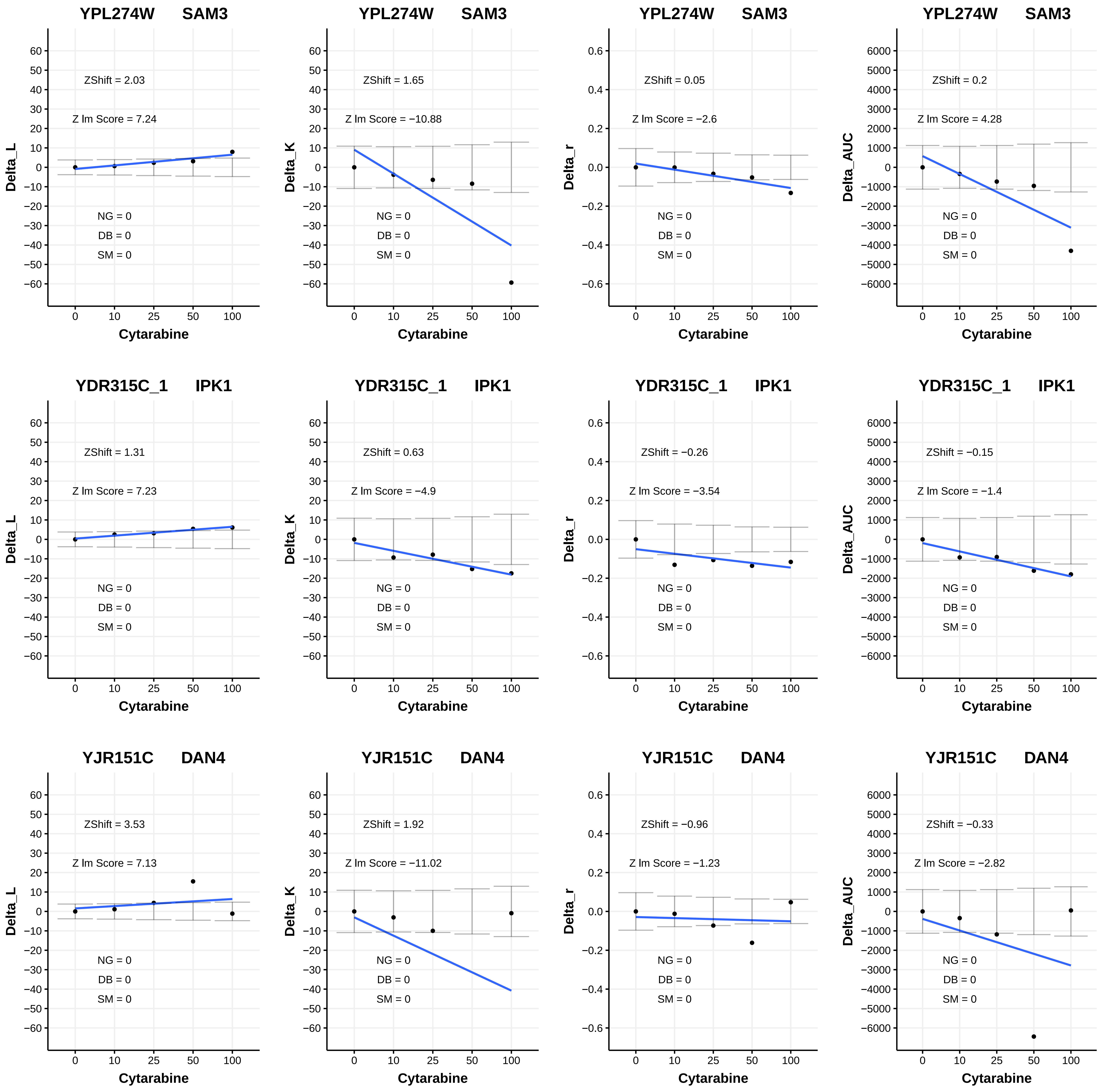
YPL274W SAM3
ZShift = 2.03
Z lm Score = 7.24
NG = 0
DB = 0
SM = 0
Delta_L
Cytarabine
YPL274W SAM3
ZShift = 1.65
Z lm Score = −10.88
NG = 0
DB = 0
SM = 0
Delta_K
Cytarabine
YPL274W SAM3
ZShift = 0.05
Z lm Score = −2.6
NG = 0
DB = 0
SM = 0
Delta_r
Cytarabine
YPL274W SAM3
ZShift = 0.2
Z lm Score = 4.28
NG = 0
DB = 0
SM = 0
Delta_AUC
Cytarabine
YDR315C_1 IPK1
ZShift = 1.31
Z lm Score = 7.23
NG = 0
DB = 0
SM = 0
Delta_L
Cytarabine
YDR315C_1 IPK1
ZShift = 0.63
Z lm Score = −4.9
NG = 0
DB = 0
SM = 0
Delta_K
Cytarabine
YDR315C_1 IPK1
ZShift = −0.26
Z lm Score = −3.54
NG = 0
DB = 0
SM = 0
Delta_r
Cytarabine
YDR315C_1 IPK1
ZShift = −0.15
Z lm Score = −1.4
NG = 0
DB = 0
SM = 0
Delta_AUC
Cytarabine
YJR151C DAN4
ZShift = 3.53
Z lm Score = 7.13
NG = 0
DB = 0
SM = 0
Delta_L
Cytarabine
YJR151C DAN4
ZShift = 1.92
Z lm Score = −11.02
NG = 0
DB = 0
SM = 0
Delta_K
Cytarabine
YJR151C DAN4
ZShift = −0.96
Z lm Score = −1.23
NG = 0
DB = 0
SM = 0
Delta_r
Cytarabine
YJR151C DAN4
ZShift = −0.33
Z lm Score = −2.82
NG = 0
DB = 0
SM = 0
Delta_AUC
Cytarabine

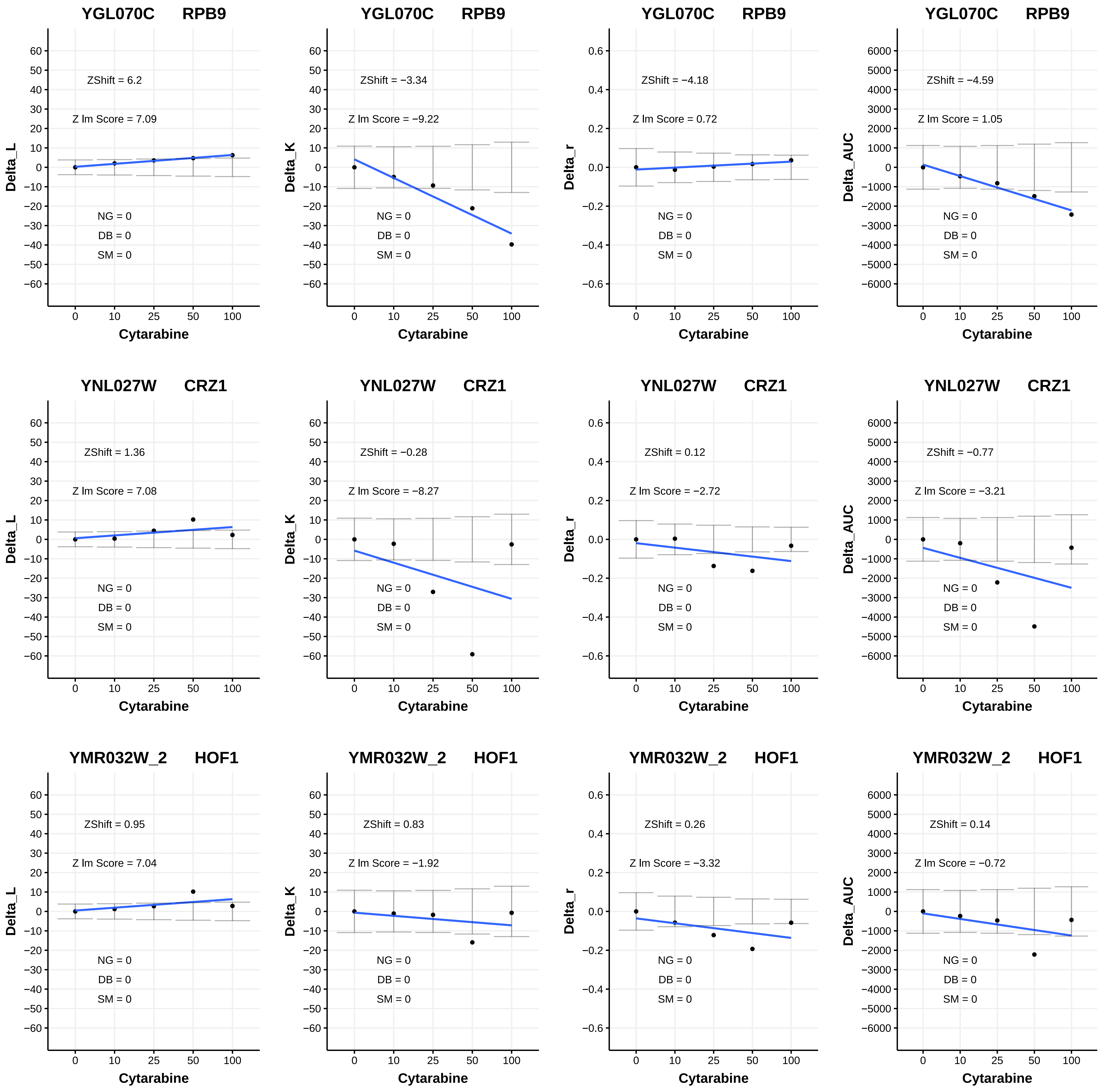

YGL070C RPB9
ZShift = 6.2
Z lm Score = 7.09
NG = 0
DB = 0
SM = 0
Delta_L
Cytarabine
YGL070C RPB9
ZShift = −3.34
Z lm Score = −9.22
NG = 0
DB = 0
SM = 0
Delta_K
Cytarabine
YGL070C RPB9
ZShift = −4.18
Z lm Score = 0.72
NG = 0
DB = 0
SM = 0
Delta_r
Cytarabine
YGL070C RPB9
ZShift = −4.59
Z lm Score = 1.05
NG = 0
DB = 0
SM = 0
Delta_AUC
Cytarabine
YNL027W CRZ1
ZShift = 1.36
Z lm Score = 7.08
NG = 0
DB = 0
SM = 0
Delta_L
Cytarabine
YNL027W CRZ1
ZShift = −0.28
Z lm Score = −8.27
NG = 0
DB = 0
SM = 0
Delta_K
Cytarabine
YNL027W CRZ1
ZShift = 0.12
Z lm Score = −2.72
NG = 0
DB = 0
SM = 0
Delta_r
Cytarabine
YNL027W CRZ1
ZShift = −0.77
Z lm Score = −3.21
NG = 0
DB = 0
SM = 0
Delta_AUC
Cytarabine
YMR032W_2 HOF1
ZShift = 0.95
Z lm Score = 7.04
NG = 0
DB = 0
SM = 0
Delta_L
Cytarabine
YMR032W_2 HOF1
ZShift = 0.83
Z lm Score = −1.92
NG = 0
DB = 0
SM = 0
Delta_K
Cytarabine
YMR032W_2 HOF1
ZShift = 0.26
Z lm Score = −3.32
NG = 0
DB = 0
SM = 0
Delta_r
Cytarabine
YMR032W_2 HOF1
ZShift = 0.14
Z lm Score = −0.72
NG = 0
DB = 0
SM = 0
Delta_AUC
Cytarabine

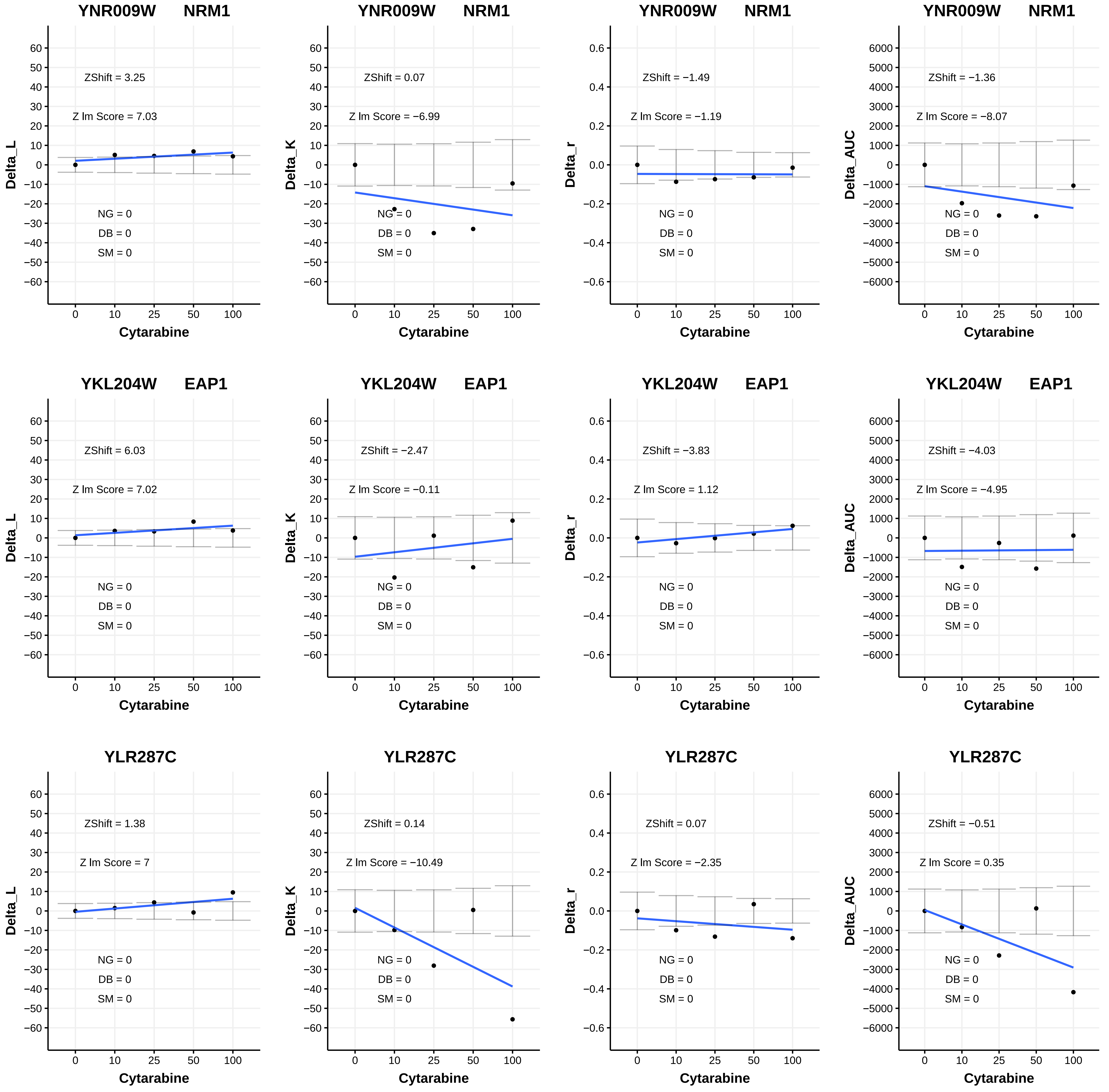
YNR009W NRM1
ZShift = 3.25
Z lm Score = 7.03
NG = 0
DB = 0
SM = 0
Delta_L
Cytarabine
YNR009W NRM1
ZShift = 0.07
Z lm Score = −6.99
NG = 0
DB = 0
SM = 0
Delta_K
Cytarabine
YNR009W NRM1
ZShift = −1.49
Z lm Score = −1.19
NG = 0
DB = 0
SM = 0
Delta_r
Cytarabine
YNR009W NRM1
ZShift = −1.36
Z lm Score = −8.07
NG = 0
DB = 0
SM = 0
Delta_AUC
Cytarabine
YKL204W EAP1
ZShift = 6.03
Z lm Score = 7.02
NG = 0
DB = 0
SM = 0
Delta_L
Cytarabine
YKL204W EAP1
ZShift = −2.47
Z lm Score = −0.11
NG = 0
DB = 0
SM = 0
Delta_K
Cytarabine
YKL204W EAP1
ZShift = −3.83
Z lm Score = 1.12
NG = 0
DB = 0
SM = 0
Delta_r
Cytarabine
YKL204W EAP1
ZShift = −4.03
Z lm Score = −4.95
NG = 0
DB = 0
SM = 0
Delta_AUC
Cytarabine
YLR287C
ZShift = 1.38
Z lm Score = 7
NG = 0
DB = 0
SM = 0
Delta_L
Cytarabine
YLR287C
ZShift = 0.14
Z lm Score = −10.49
NG = 0
DB = 0
SM = 0
Delta_K
Cytarabine
YLR287C
ZShift = 0.07
Z lm Score = −2.35
NG = 0
DB = 0
SM = 0
Delta_r
Cytarabine
YLR287C
ZShift = −0.51
Z lm Score = 0.35
NG = 0
DB = 0
SM = 0
Delta_AUC
Cytarabine

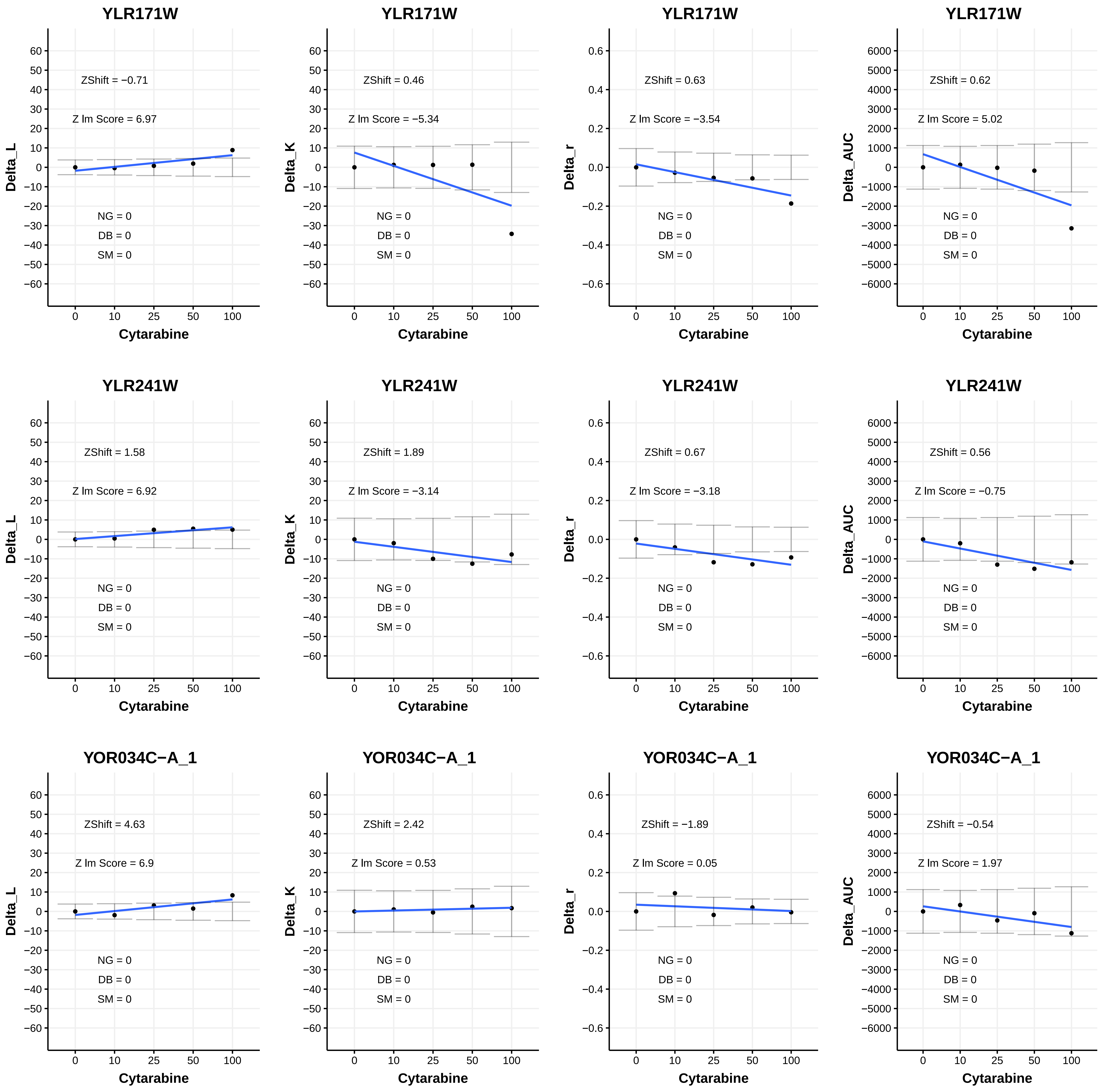
YLR171W
ZShift = −0.71
Z lm Score = 6.97
NG = 0
DB = 0
SM = 0
Delta_L
Cytarabine
YLR171W
ZShift = 0.46
Z lm Score = −5.34
NG = 0
DB = 0
SM = 0
Delta_K
Cytarabine
YLR171W
ZShift = 0.63
Z lm Score = −3.54
NG = 0
DB = 0
SM = 0
Delta_r
Cytarabine
YLR171W
ZShift = 0.62
Z lm Score = 5.02
NG = 0
DB = 0
SM = 0
Delta_AUC
Cytarabine
YLR241W
ZShift = 1.58
Z lm Score = 6.92
NG = 0
DB = 0
SM = 0
Delta_L
Cytarabine
YLR241W
ZShift = 1.89
Z lm Score = −3.14
NG = 0
DB = 0
SM = 0
Delta_K
Cytarabine
YLR241W
ZShift = 0.67
Z lm Score = −3.18
NG = 0
DB = 0
SM = 0
Delta_r
Cytarabine
YLR241W
ZShift = 0.56
Z lm Score = −0.75
NG = 0
DB = 0
SM = 0
Delta_AUC
Cytarabine
YOR034C−A_1
ZShift = 4.63
Z lm Score = 6.9
NG = 0
DB = 0
SM = 0
Delta_L
Cytarabine
YOR034C−A_1
ZShift = 2.42
Z lm Score = 0.53
NG = 0
DB = 0
SM = 0
Delta_K
Cytarabine
YOR034C−A_1
ZShift = −1.89
Z lm Score = 0.05
NG = 0
DB = 0
SM = 0
Delta_r
Cytarabine
YOR034C−A_1
ZShift = −0.54
Z lm Score = 1.97
NG = 0
DB = 0
SM = 0
Delta_AUC
Cytarabine

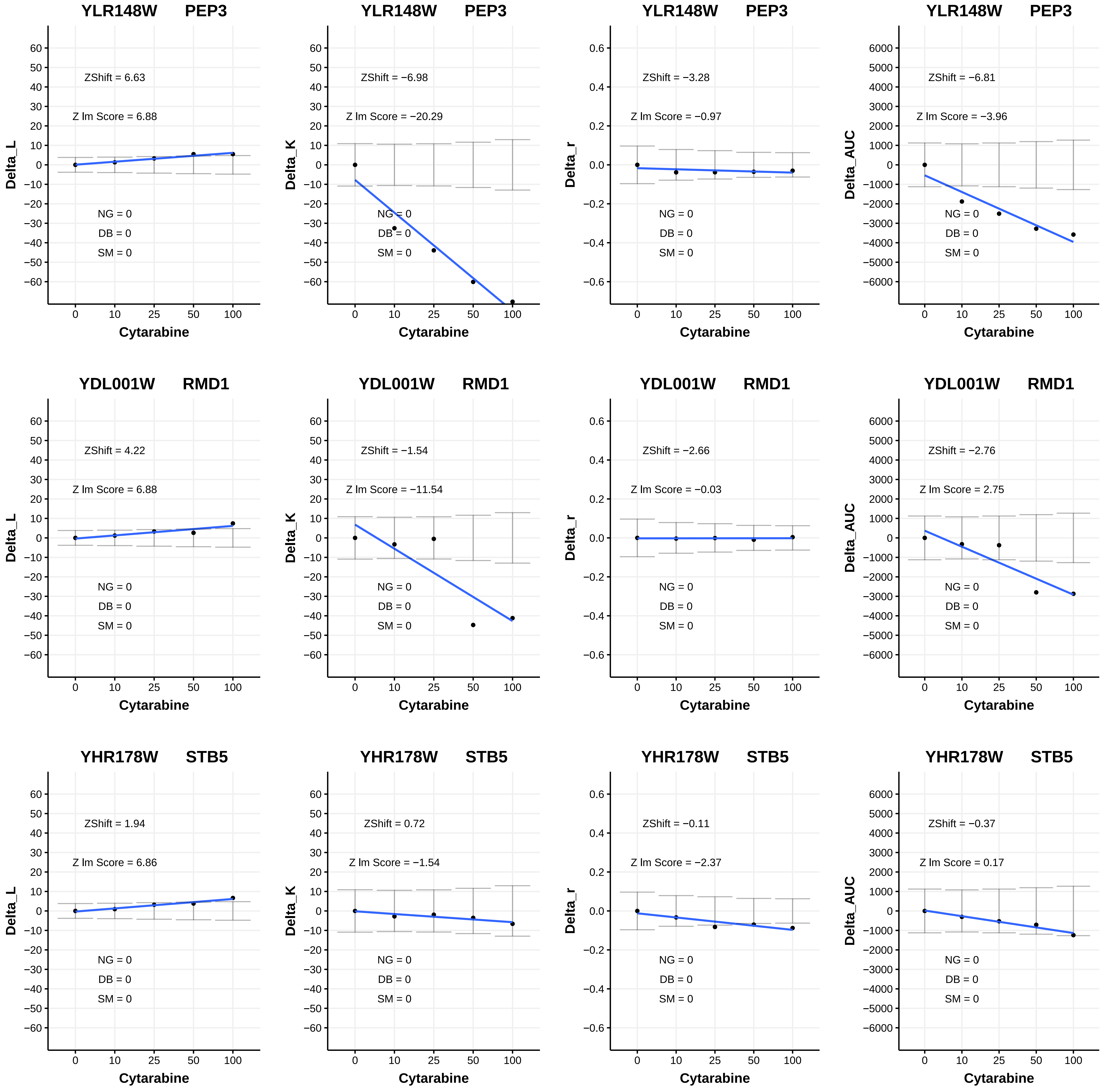

YLR148W PEP3
ZShift = 6.63
Z lm Score = 6.88
NG = 0
DB = 0
SM = 0
Delta_L
Cytarabine
YLR148W PEP3
ZShift = −6.98
Z lm Score = −20.29
NG = 0
DB = 0
SM = 0
Delta_K
Cytarabine
YLR148W PEP3
ZShift = −3.28
Z lm Score = −0.97
NG = 0
DB = 0
SM = 0
Delta_r
Cytarabine
YLR148W PEP3
ZShift = −6.81
Z lm Score = −3.96
NG = 0
DB = 0
SM = 0
Delta_AUC
Cytarabine
YDL001W RMD1
ZShift = 4.22
Z lm Score = 6.88
NG = 0
DB = 0
SM = 0
Delta_L
Cytarabine
YDL001W RMD1
ZShift = −1.54
Z lm Score = −11.54
NG = 0
DB = 0
SM = 0
Delta_K
Cytarabine
YDL001W RMD1
ZShift = −2.66
Z lm Score = −0.03
NG = 0
DB = 0
SM = 0
Delta_r
Cytarabine
YDL001W RMD1
ZShift = −2.76
Z lm Score = 2.75
NG = 0
DB = 0
SM = 0
Delta_AUC
Cytarabine
YHR178W STB5
ZShift = 1.94
Z lm Score = 6.86
NG = 0
DB = 0
SM = 0
Delta_L
Cytarabine
YHR178W STB5
ZShift = 0.72
Z lm Score = −1.54
NG = 0
DB = 0
SM = 0
Delta_K
Cytarabine
YHR178W STB5
ZShift = −0.11
Z lm Score = −2.37
NG = 0
DB = 0
SM = 0
Delta_r
Cytarabine
YHR178W STB5
ZShift = −0.37
Z lm Score = 0.17
NG = 0
DB = 0
SM = 0
Delta_AUC
Cytarabine

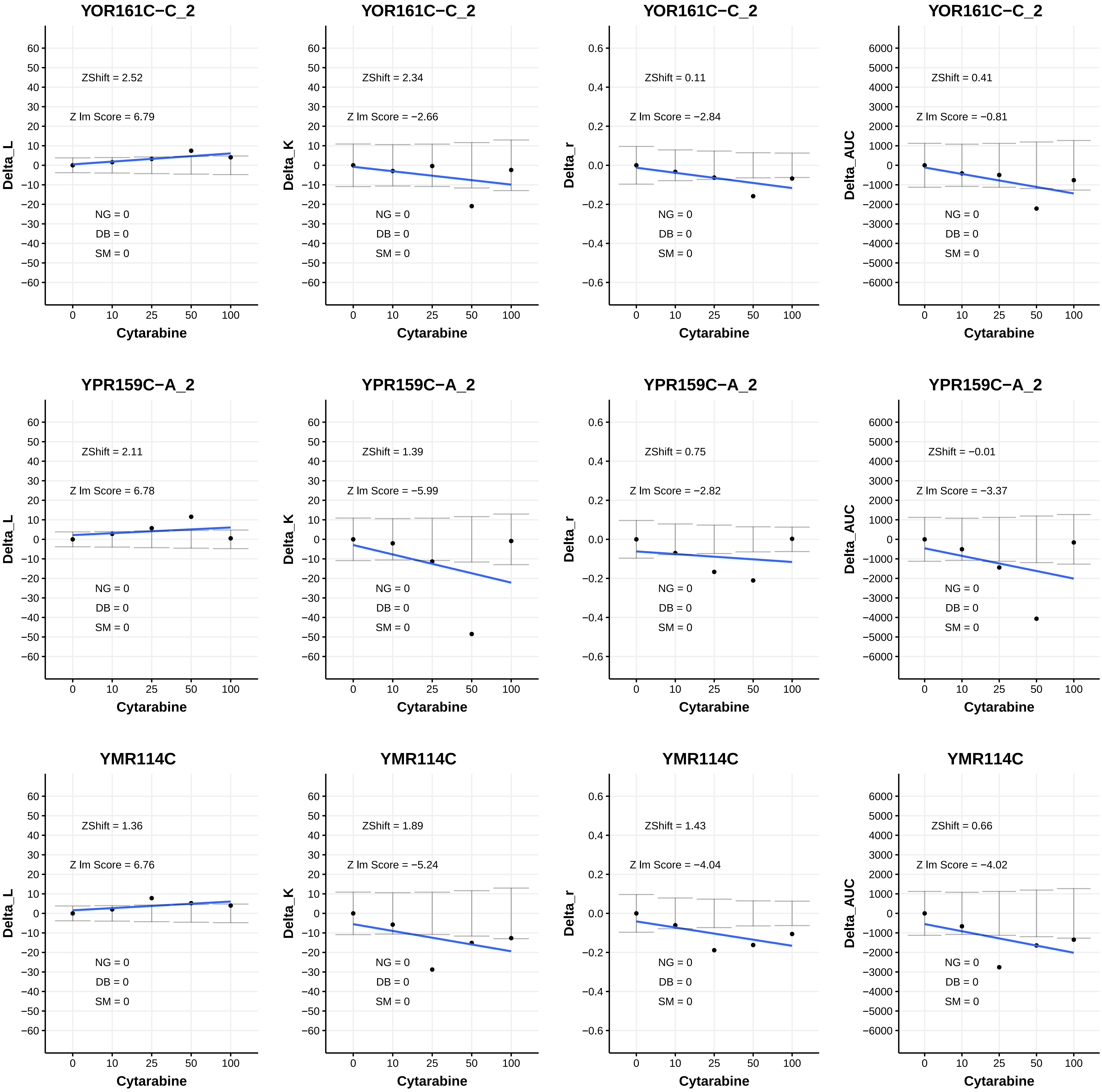
YOR161C−C_2
ZShift = 2.52
Z lm Score = 6.79
NG = 0
DB = 0
SM = 0
Delta_L
Cytarabine
YOR161C−C_2
ZShift = 2.34
Z lm Score = −2.66
NG = 0
DB = 0
SM = 0
Delta_K
Cytarabine
YOR161C−C_2
ZShift = 0.11
Z lm Score = −2.84
NG = 0
DB = 0
SM = 0
Delta_r
Cytarabine
YOR161C−C_2
ZShift = 0.41
Z lm Score = −0.81
NG = 0
DB = 0
SM = 0
Delta_AUC
Cytarabine
YPR159C−A_2
ZShift = 2.11
Z lm Score = 6.78
NG = 0
DB = 0
SM = 0
Delta_L
Cytarabine
YPR159C−A_2
ZShift = 1.39
Z lm Score = −5.99
NG = 0
DB = 0
SM = 0
Delta_K
Cytarabine
YPR159C−A_2
ZShift = 0.75
Z lm Score = −2.82
NG = 0
DB = 0
SM = 0
Delta_r
Cytarabine
YPR159C−A_2
ZShift = −0.01
Z lm Score = −3.37
NG = 0
DB = 0
SM = 0
Delta_AUC
Cytarabine
YMR114C
ZShift = 1.36
Z lm Score = 6.76
NG = 0
DB = 0
SM = 0
Delta_L
Cytarabine
YMR114C
ZShift = 1.89
Z lm Score = −5.24
NG = 0
DB = 0
SM = 0
Delta_K
Cytarabine
YMR114C
ZShift = 1.43
Z lm Score = −4.04
NG = 0
DB = 0
SM = 0
Delta_r
Cytarabine
YMR114C
ZShift = 0.66
Z lm Score = −4.02
NG = 0
DB = 0
SM = 0
Delta_AUC
Cytarabine

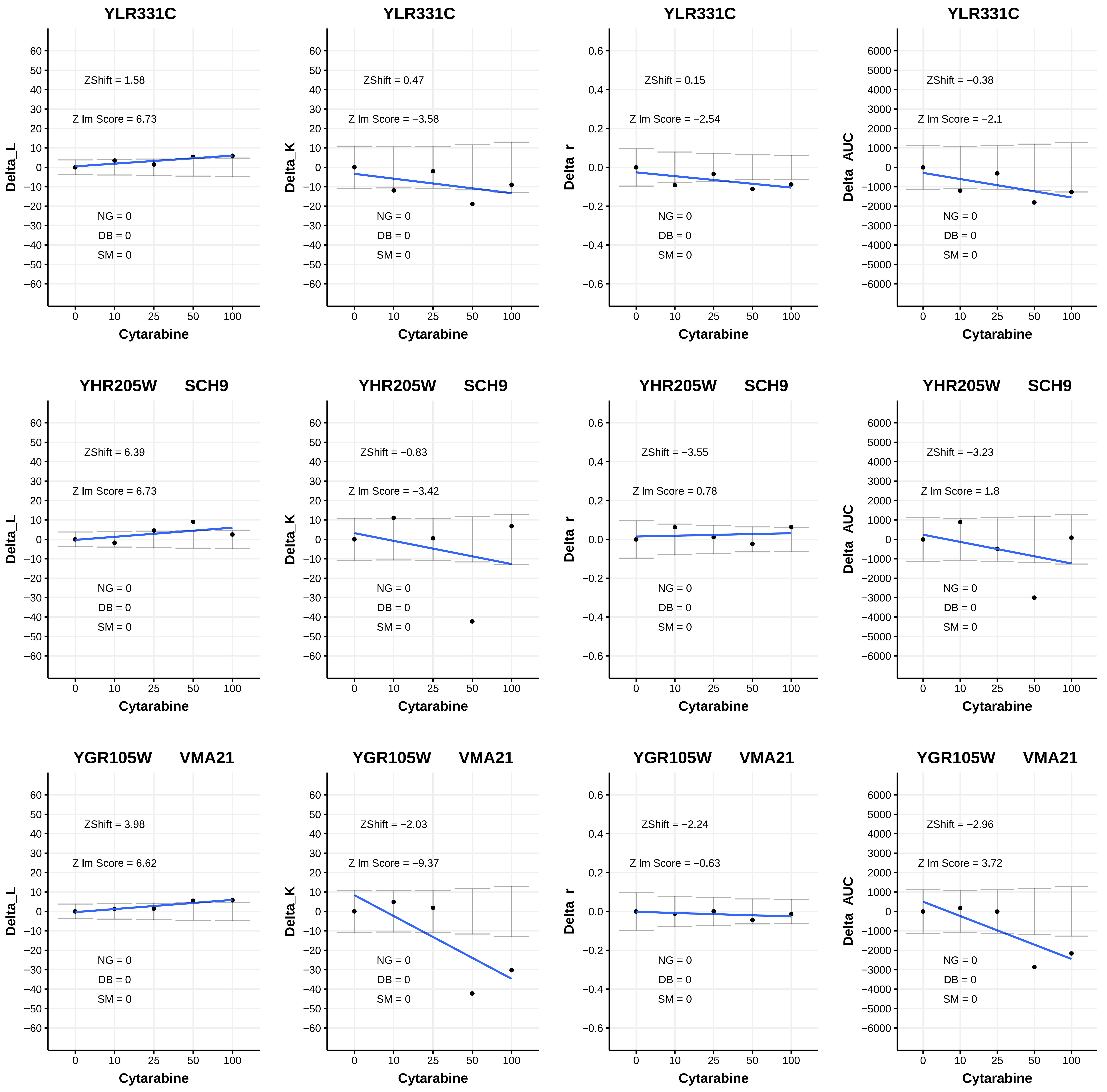

YLR331C
ZShift = 1.58
Z lm Score = 6.73
NG = 0
DB = 0
SM = 0
Delta_L
Cytarabine
YLR331C
ZShift = 0.47
Z lm Score = −3.58
NG = 0
DB = 0
SM = 0
Delta_K
Cytarabine
YLR331C
ZShift = 0.15
Z lm Score = −2.54
NG = 0
DB = 0
SM = 0
Delta_r
Cytarabine
YLR331C
ZShift = −0.38
Z lm Score = −2.1
NG = 0
DB = 0
SM = 0
Delta_AUC
Cytarabine
YHR205W SCH9
ZShift = 6.39
Z lm Score = 6.73
NG = 0
DB = 0
SM = 0
Delta_L
Cytarabine
YHR205W SCH9
ZShift = −0.83
Z lm Score = −3.42
NG = 0
DB = 0
SM = 0
Delta_K
Cytarabine
YHR205W SCH9
ZShift = −3.55
Z lm Score = 0.78
NG = 0
DB = 0
SM = 0
Delta_r
Cytarabine
YHR205W SCH9
ZShift = −3.23
Z lm Score = 1.8
NG = 0
DB = 0
SM = 0
Delta_AUC
Cytarabine
YGR105W VMA21
ZShift = 3.98
Z lm Score = 6.62
NG = 0
DB = 0
SM = 0
Delta_L
Cytarabine
YGR105W VMA21
ZShift = −2.03
Z lm Score = −9.37
NG = 0
DB = 0
SM = 0
Delta_K
Cytarabine
YGR105W VMA21
ZShift = −2.24
Z lm Score = −0.63
NG = 0
DB = 0
SM = 0
Delta_r
Cytarabine
YGR105W VMA21
ZShift = −2.96
Z lm Score = 3.72
NG = 0
DB = 0
SM = 0
Delta_AUC
Cytarabine

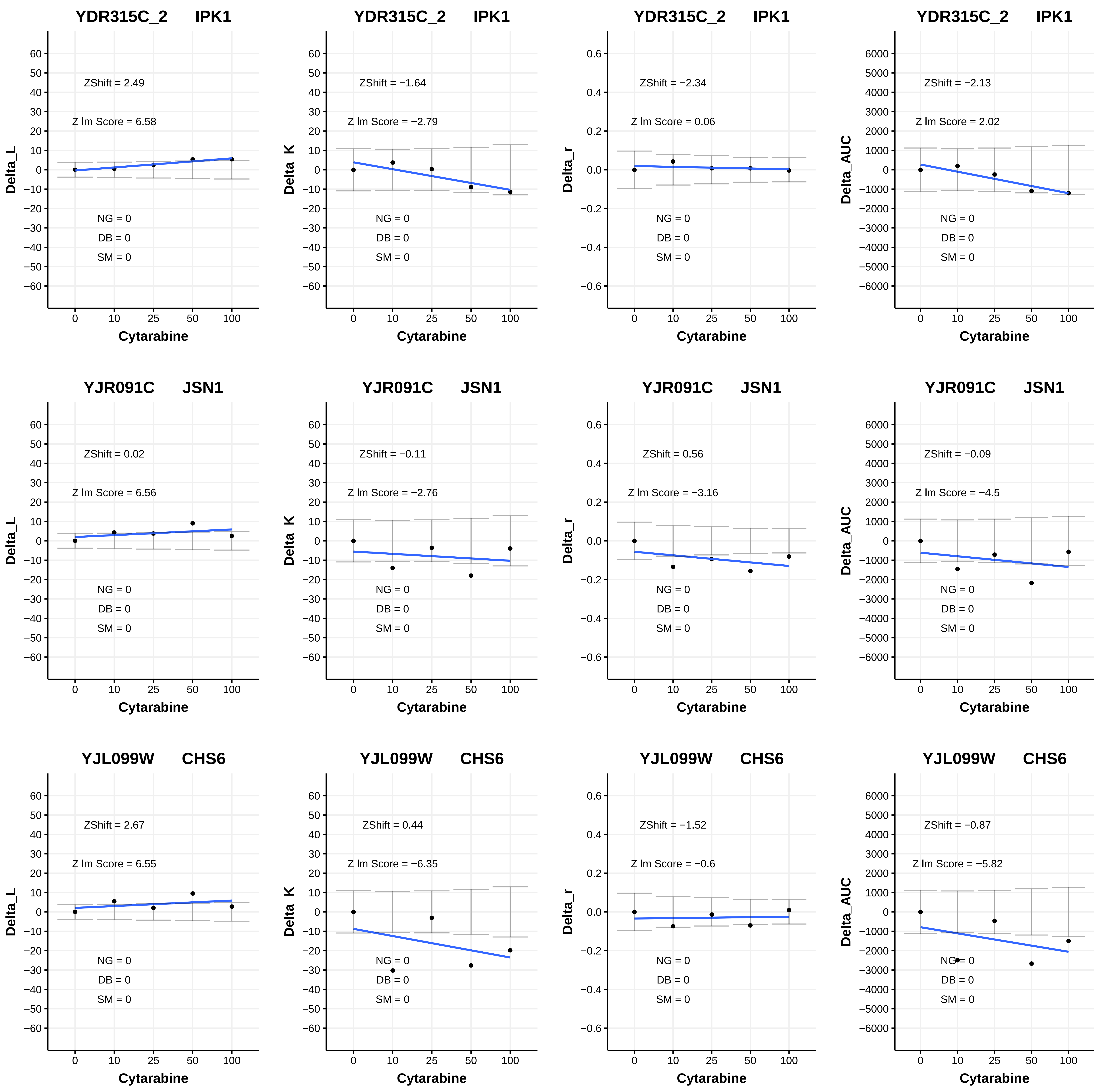

YDR315C_2 IPK1
ZShift = 2.49
Z lm Score = 6.58
NG = 0
DB = 0
SM = 0
Delta_L
Cytarabine
YDR315C_2 IPK1
ZShift = −1.64
Z lm Score = −2.79
NG = 0
DB = 0
SM = 0
Delta_K
Cytarabine
YDR315C_2 IPK1
ZShift = −2.34
Z lm Score = 0.06
NG = 0
DB = 0
SM = 0
Delta_r
Cytarabine
YDR315C_2 IPK1
ZShift = −2.13
Z lm Score = 2.02
NG = 0
DB = 0
SM = 0
Delta_AUC
Cytarabine
YJR091C JSN1
ZShift = 0.02
Z lm Score = 6.56
NG = 0
DB = 0
SM = 0
Delta_L
Cytarabine
YJR091C JSN1
ZShift = −0.11
Z lm Score = −2.76
NG = 0
DB = 0
SM = 0
Delta_K
Cytarabine
YJR091C JSN1
ZShift = 0.56
Z lm Score = −3.16
NG = 0
DB = 0
SM = 0
Delta_r
Cytarabine
YJR091C JSN1
ZShift = −0.09
Z lm Score = −4.5
NG = 0
DB = 0
SM = 0
Delta_AUC
Cytarabine
YJL099W CHS6
ZShift = 2.67
Z lm Score = 6.55
NG = 0
DB = 0
SM = 0
Delta_L
Cytarabine
YJL099W CHS6
ZShift = 0.44
Z lm Score = −6.35
NG = 0
DB = 0
SM = 0
Delta_K
Cytarabine
YJL099W CHS6
ZShift = −1.52
Z lm Score = −0.6
NG = 0
DB = 0
SM = 0
Delta_r
Cytarabine
YJL099W CHS6
ZShift = −0.87
Z lm Score = −5.82
NG = 0
DB = 0
SM = 0
Delta_AUC
Cytarabine

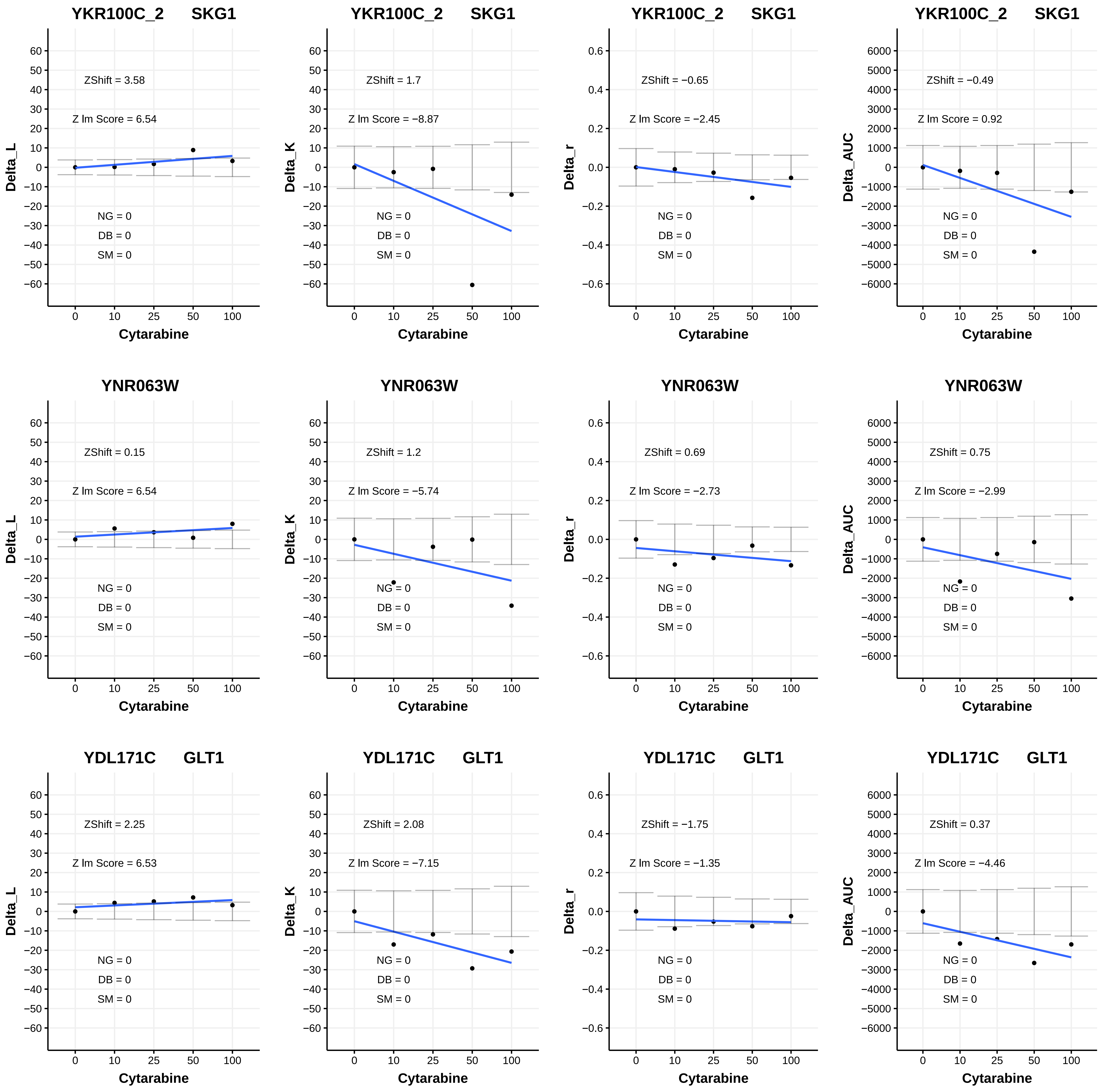
YKR100C_2 SKG1
ZShift = 3.58
Z lm Score = 6.54
NG = 0
DB = 0
SM = 0
Delta_L
Cytarabine
YKR100C_2 SKG1
ZShift = 1.7
Z lm Score = −8.87
NG = 0
DB = 0
SM = 0
Delta_K
Cytarabine
YKR100C_2 SKG1
ZShift = −0.65
Z lm Score = −2.45
NG = 0
DB = 0
SM = 0
Delta_r
Cytarabine
YKR100C_2 SKG1
ZShift = −0.49
Z lm Score = 0.92
NG = 0
DB = 0
SM = 0
Delta_AUC
Cytarabine
YNR063W
ZShift = 0.15
Z lm Score = 6.54
NG = 0
DB = 0
SM = 0
Delta_L
Cytarabine
YNR063W
ZShift = 1.2
Z lm Score = −5.74
NG = 0
DB = 0
SM = 0
Delta_K
Cytarabine
YNR063W
ZShift = 0.69
Z lm Score = −2.73
NG = 0
DB = 0
SM = 0
Delta_r
Cytarabine
YNR063W
ZShift = 0.75
Z lm Score = −2.99
NG = 0
DB = 0
SM = 0
Delta_AUC
Cytarabine
YDL171C GLT1
ZShift = 2.25
Z lm Score = 6.53
NG = 0
DB = 0
SM = 0
Delta_L
Cytarabine
YDL171C GLT1
ZShift = 2.08
Z lm Score = −7.15
NG = 0
DB = 0
SM = 0
Delta_K
Cytarabine
YDL171C GLT1
ZShift = −1.75
Z lm Score = −1.35
NG = 0
DB = 0
SM = 0
Delta_r
Cytarabine
YDL171C GLT1
ZShift = 0.37
Z lm Score = −4.46
NG = 0
DB = 0
SM = 0
Delta_AUC
Cytarabine
0 10 25 50 100

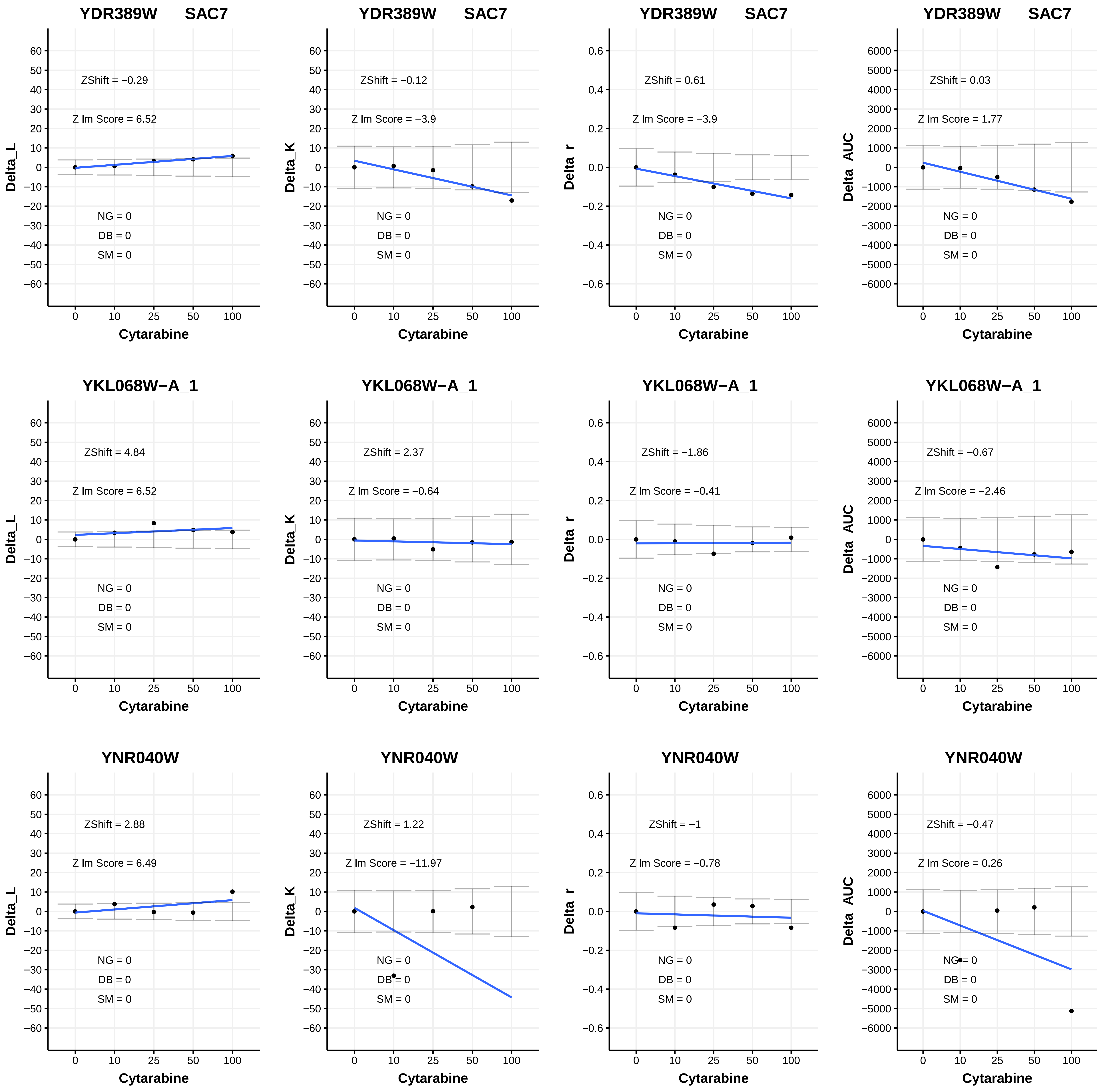
YDR389W SAC7
ZShift = −0.29
Z lm Score = 6.52
NG = 0
DB = 0
SM = 0
Delta_L
Cytarabine
YDR389W SAC7
ZShift = −0.12
Z lm Score = −3.9
NG = 0
DB = 0
SM = 0
Delta_K
Cytarabine
YDR389W SAC7
ZShift = 0.61
Z lm Score = −3.9
NG = 0
DB = 0
SM = 0
Delta_r
Cytarabine
YDR389W SAC7
ZShift = 0.03
Z lm Score = 1.77
NG = 0
DB = 0
SM = 0
Delta_AUC
Cytarabine
YKL068W−A_1
ZShift = 4.84
Z lm Score = 6.52
NG = 0
DB = 0
SM = 0
Delta_L
Cytarabine
YKL068W−A_1
ZShift = 2.37
Z lm Score = −0.64
NG = 0
DB = 0
SM = 0
Delta_K
Cytarabine
YKL068W−A_1
ZShift = −1.86
Z lm Score = −0.41
NG = 0
DB = 0
SM = 0
Delta_r
Cytarabine
YKL068W−A_1
ZShift = −0.67
Z lm Score = −2.46
NG = 0
DB = 0
SM = 0
Delta_AUC
Cytarabine
YNR040W
ZShift = 2.88
Z lm Score = 6.49
NG = 0
DB = 0
SM = 0
Delta_L
Cytarabine
YNR040W
ZShift = 1.22
Z lm Score = −11.97
NG = 0
DB = 0
SM = 0
Delta_K
Cytarabine
YNR040W
ZShift = −1
Z lm Score = −0.78
NG = 0
DB = 0
SM = 0
Delta_r
Cytarabine
YNR040W
ZShift = −0.47
Z lm Score = 0.26
NG = 0
DB = 0
SM = 0
Delta_AUC
Cytarabine

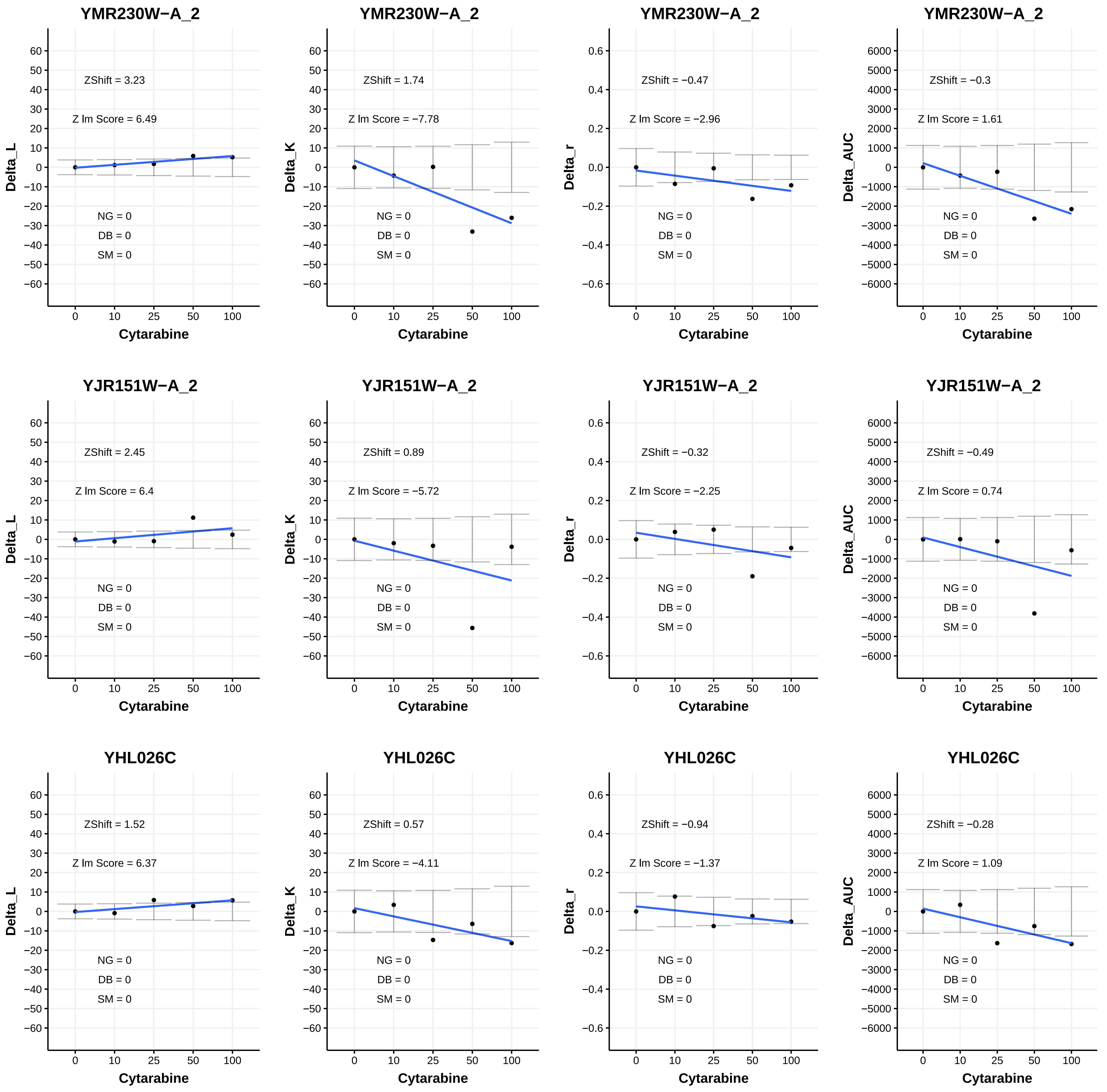
YMR230W−A_2
ZShift = 3.23
Z lm Score = 6.49
NG = 0
DB = 0
SM = 0
Delta_L
Cytarabine
YMR230W−A_2
ZShift = 1.74
Z lm Score = −7.78
NG = 0
DB = 0
SM = 0
Delta_K
Cytarabine
YMR230W−A_2
ZShift = −0.47
Z lm Score = −2.96
NG = 0
DB = 0
SM = 0
Delta_r
Cytarabine
YMR230W−A_2
ZShift = −0.3
Z lm Score = 1.61
NG = 0
DB = 0
SM = 0
Delta_AUC
Cytarabine
YJR151W−A_2
ZShift = 2.45
Z lm Score = 6.4
NG = 0
DB = 0
SM = 0
Delta_L
Cytarabine
YJR151W−A_2
ZShift = 0.89
Z lm Score = −5.72
NG = 0
DB = 0
SM = 0
Delta_K
Cytarabine
YJR151W−A_2
ZShift = −0.32
Z lm Score = −2.25
NG = 0
DB = 0
SM = 0
Delta_r
Cytarabine
YJR151W−A_2
ZShift = −0.49
Z lm Score = 0.74
NG = 0
DB = 0
SM = 0
Delta_AUC
Cytarabine
YHL026C
ZShift = 1.52
Z lm Score = 6.37
NG = 0
DB = 0
SM = 0
Delta_L
Cytarabine
YHL026C
ZShift = 0.57
Z lm Score = −4.11
NG = 0
DB = 0
SM = 0
Delta_K
Cytarabine
YHL026C
ZShift = −0.94
Z lm Score = −1.37
NG = 0
DB = 0
SM = 0
Delta_r
Cytarabine
YHL026C
ZShift = −0.28
Z lm Score = 1.09
NG = 0
DB = 0
SM = 0
Delta_AUC
Cytarabine

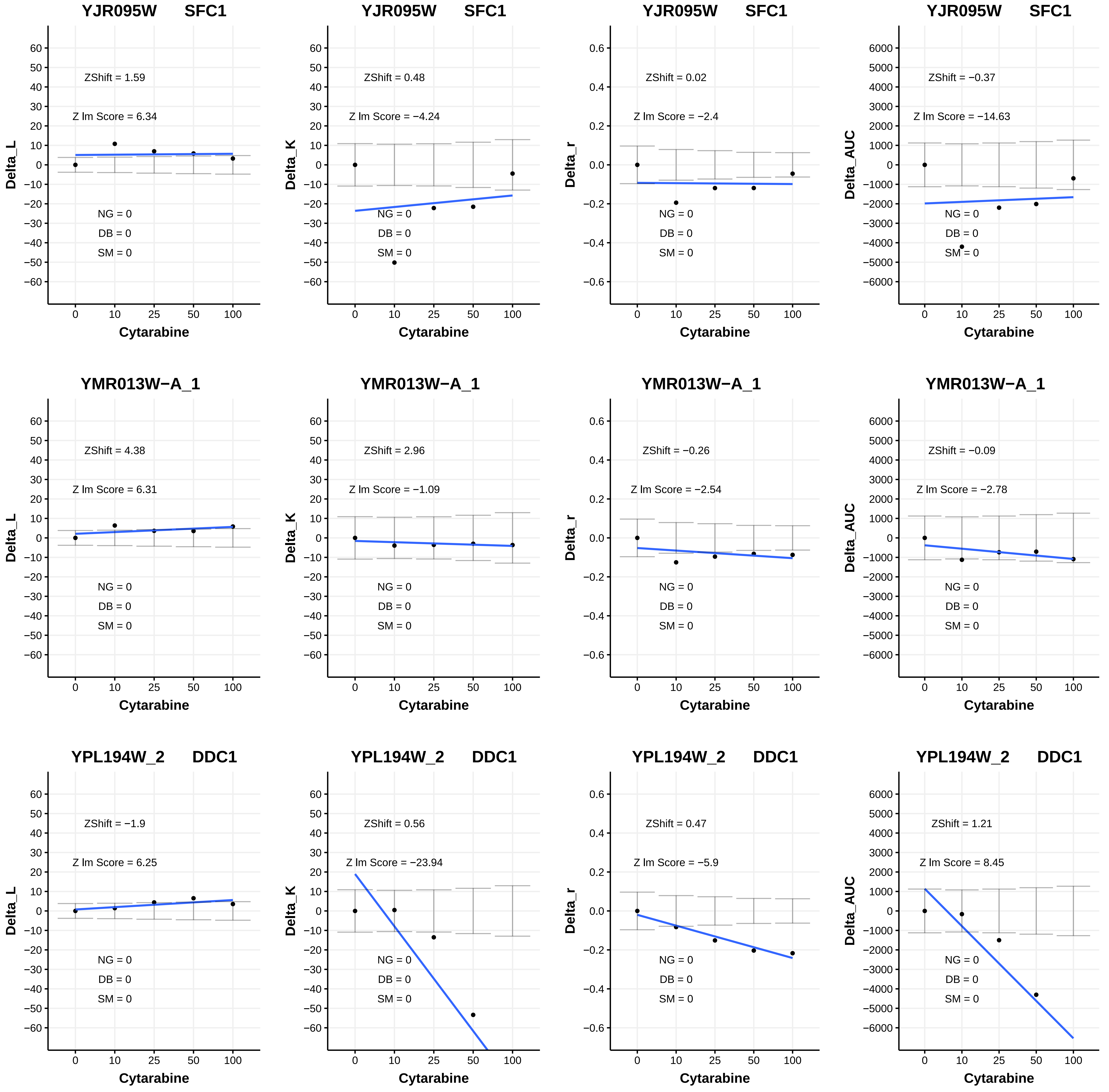
YJR095W SFC1
ZShift = 1.59
Z lm Score = 6.34
NG = 0
DB = 0
SM = 0
Delta_L
Cytarabine
YJR095W SFC1
ZShift = 0.48
Z lm Score = −4.24
NG = 0
DB = 0
SM = 0
Delta_K
Cytarabine
YJR095W SFC1
ZShift = 0.02
Z lm Score = −2.4
NG = 0
DB = 0
SM = 0
Delta_r
Cytarabine
YJR095W SFC1
ZShift = −0.37
Z lm Score = −14.63
NG = 0
DB = 0
SM = 0
Delta_AUC
Cytarabine
YMR013W−A_1
ZShift = 4.38
Z lm Score = 6.31
NG = 0
DB = 0
SM = 0
Delta_L
Cytarabine
YMR013W−A_1
ZShift = 2.96
Z lm Score = −1.09
NG = 0
DB = 0
SM = 0
Delta_K
Cytarabine
YMR013W−A_1
ZShift = −0.26
Z lm Score = −2.54
NG = 0
DB = 0
SM = 0
Delta_r
Cytarabine
YMR013W−A_1
ZShift = −0.09
Z lm Score = −2.78
NG = 0
DB = 0
SM = 0
Delta_AUC
Cytarabine
YPL194W_2 DDC1
ZShift = −1.9
Z lm Score = 6.25
NG = 0
DB = 0
SM = 0
Delta_L
Cytarabine
YPL194W_2 DDC1
ZShift = 0.56
Z lm Score = −23.94
NG = 0
DB = 0
SM = 0
Delta_K
Cytarabine
YPL194W_2 DDC1
ZShift = 0.47
Z lm Score = −5.9
NG = 0
DB = 0
SM = 0
Delta_r
Cytarabine
YPL194W_2 DDC1
ZShift = 1.21
Z lm Score = 8.45
NG = 0
DB = 0
SM = 0
Delta_AUC
Cytarabine

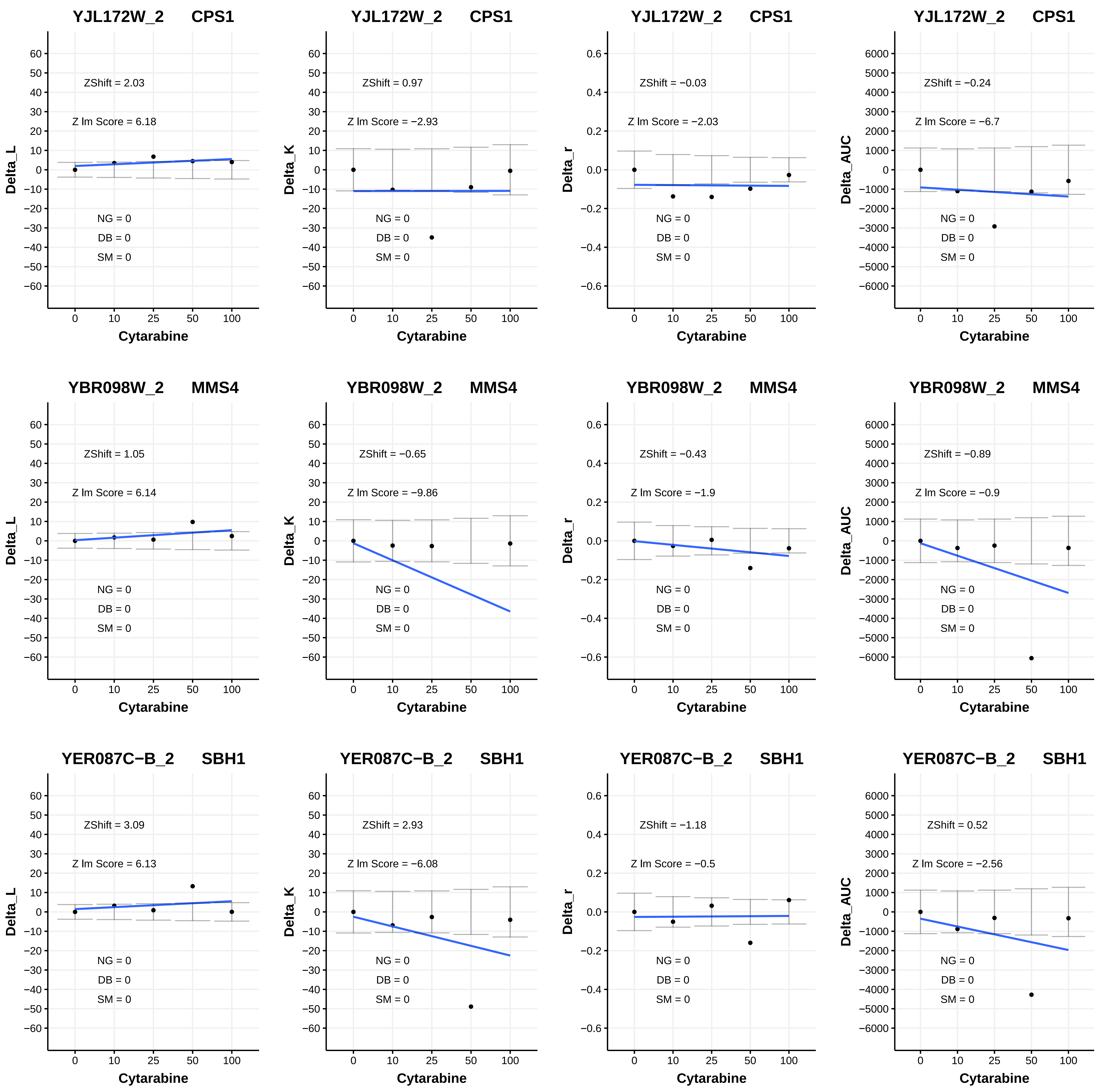

YJL172W_2 CPS1
ZShift = 2.03
Z lm Score = 6.18
NG = 0
DB = 0
SM = 0
Delta_L
Cytarabine
YJL172W_2 CPS1
ZShift = 0.97
Z lm Score = −2.93
NG = 0
DB = 0
SM = 0
Delta_K
Cytarabine
YJL172W_2 CPS1
ZShift = −0.03
Z lm Score = −2.03
NG = 0
DB = 0
SM = 0
Delta_r
Cytarabine
YJL172W_2 CPS1
ZShift = −0.24
Z lm Score = −6.7
NG = 0
DB = 0
SM = 0
Delta_AUC
Cytarabine
YBR098W_2 MMS4
ZShift = 1.05
Z lm Score = 6.14
NG = 0
DB = 0
SM = 0
Delta_L
Cytarabine
YBR098W_2 MMS4
ZShift = −0.65
Z lm Score = −9.86
NG = 0
DB = 0
SM = 0
Delta_K
Cytarabine
YBR098W_2 MMS4
ZShift = −0.43
Z lm Score = −1.9
NG = 0
DB = 0
SM = 0
Delta_r
Cytarabine
YBR098W_2 MMS4
ZShift = −0.89
Z lm Score = −0.9
NG = 0
DB = 0
SM = 0
Delta_AUC
Cytarabine
YER087C−B_2 SBH1
ZShift = 3.09
Z lm Score = 6.13
NG = 0
DB = 0
SM = 0
Delta_L
Cytarabine
YER087C−B_2 SBH1
ZShift = 2.93
Z lm Score = −6.08
NG = 0
DB = 0
SM = 0
Delta_K
Cytarabine
YER087C−B_2 SBH1
ZShift = −1.18
Z lm Score = −0.5
NG = 0
DB = 0
SM = 0
Delta_r
Cytarabine
YER087C−B_2 SBH1
ZShift = 0.52
Z lm Score = −2.56
NG = 0
DB = 0
SM = 0
Delta_AUC
Cytarabine

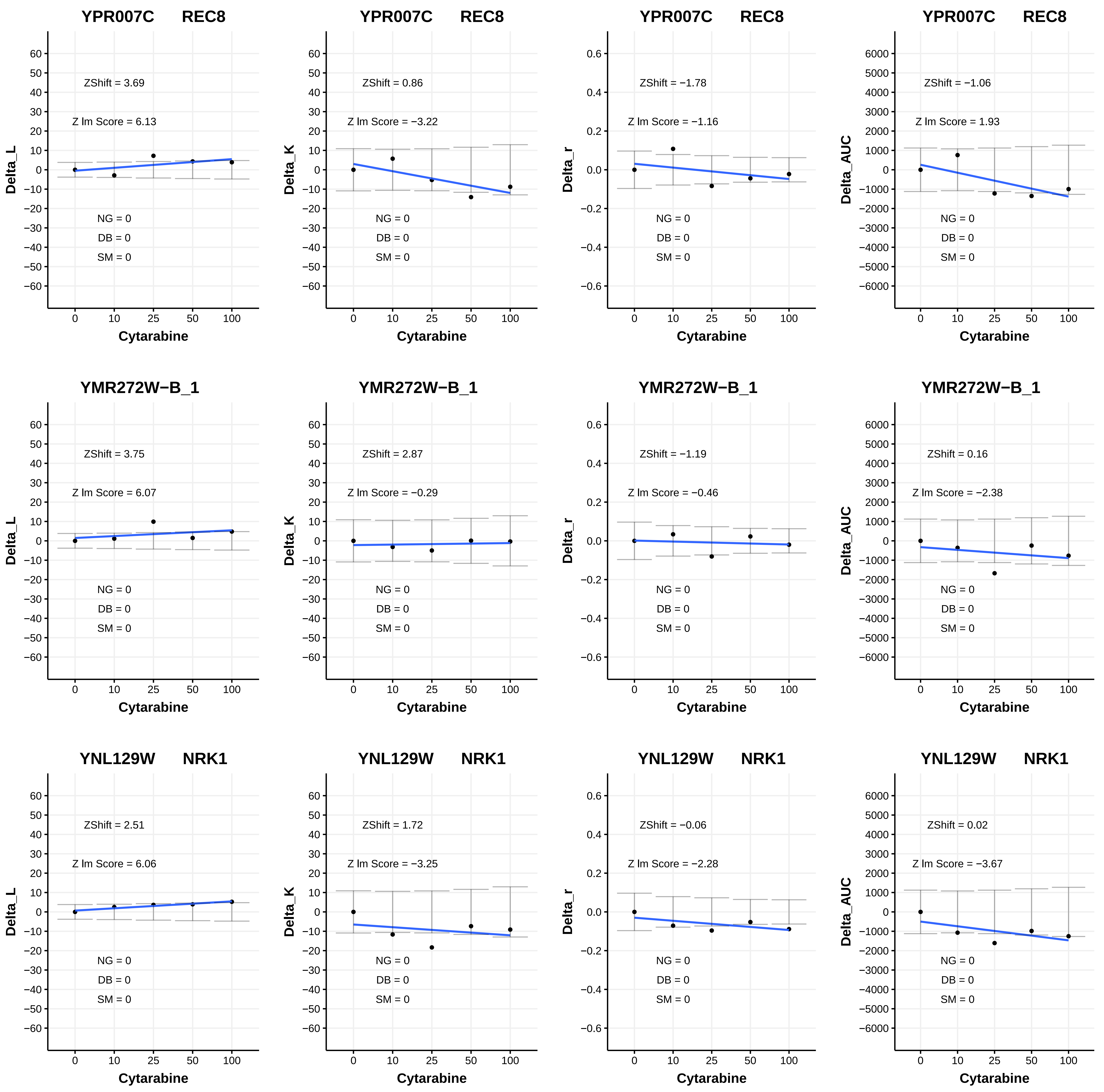

YPR007C REC8
ZShift = 3.69
Z lm Score = 6.13
NG = 0
DB = 0
SM = 0
Delta_L
Cytarabine
YPR007C REC8
ZShift = 0.86
Z lm Score = −3.22
NG = 0
DB = 0
SM = 0
Delta_K
Cytarabine
YPR007C REC8
ZShift = −1.78
Z lm Score = −1.16
NG = 0
DB = 0
SM = 0
Delta_r
Cytarabine
YPR007C REC8
ZShift = −1.06
Z lm Score = 1.93
NG = 0
DB = 0
SM = 0
Delta_AUC
Cytarabine
YMR272W−B_1
ZShift = 3.75
Z lm Score = 6.07
NG = 0
DB = 0
SM = 0
Delta_L
Cytarabine
YMR272W−B_1
ZShift = 2.87
Z lm Score = −0.29
NG = 0
DB = 0
SM = 0
Delta_K
Cytarabine
YMR272W−B_1
ZShift = −1.19
Z lm Score = −0.46
NG = 0
DB = 0
SM = 0
Delta_r
Cytarabine
YMR272W−B_1
ZShift = 0.16
Z lm Score = −2.38
NG = 0
DB = 0
SM = 0
Delta_AUC
Cytarabine
YNL129W NRK1
ZShift = 2.51
Z lm Score = 6.06
NG = 0
DB = 0
SM = 0
Delta_L
Cytarabine
YNL129W NRK1
ZShift = 1.72
Z lm Score = −3.25
NG = 0
DB = 0
SM = 0
Delta_K
Cytarabine
YNL129W NRK1
ZShift = −0.06
Z lm Score = −2.28
NG = 0
DB = 0
SM = 0
Delta_r
Cytarabine
YNL129W NRK1
ZShift = 0.02
Z lm Score = −3.67
NG = 0
DB = 0
SM = 0
Delta_AUC
Cytarabine

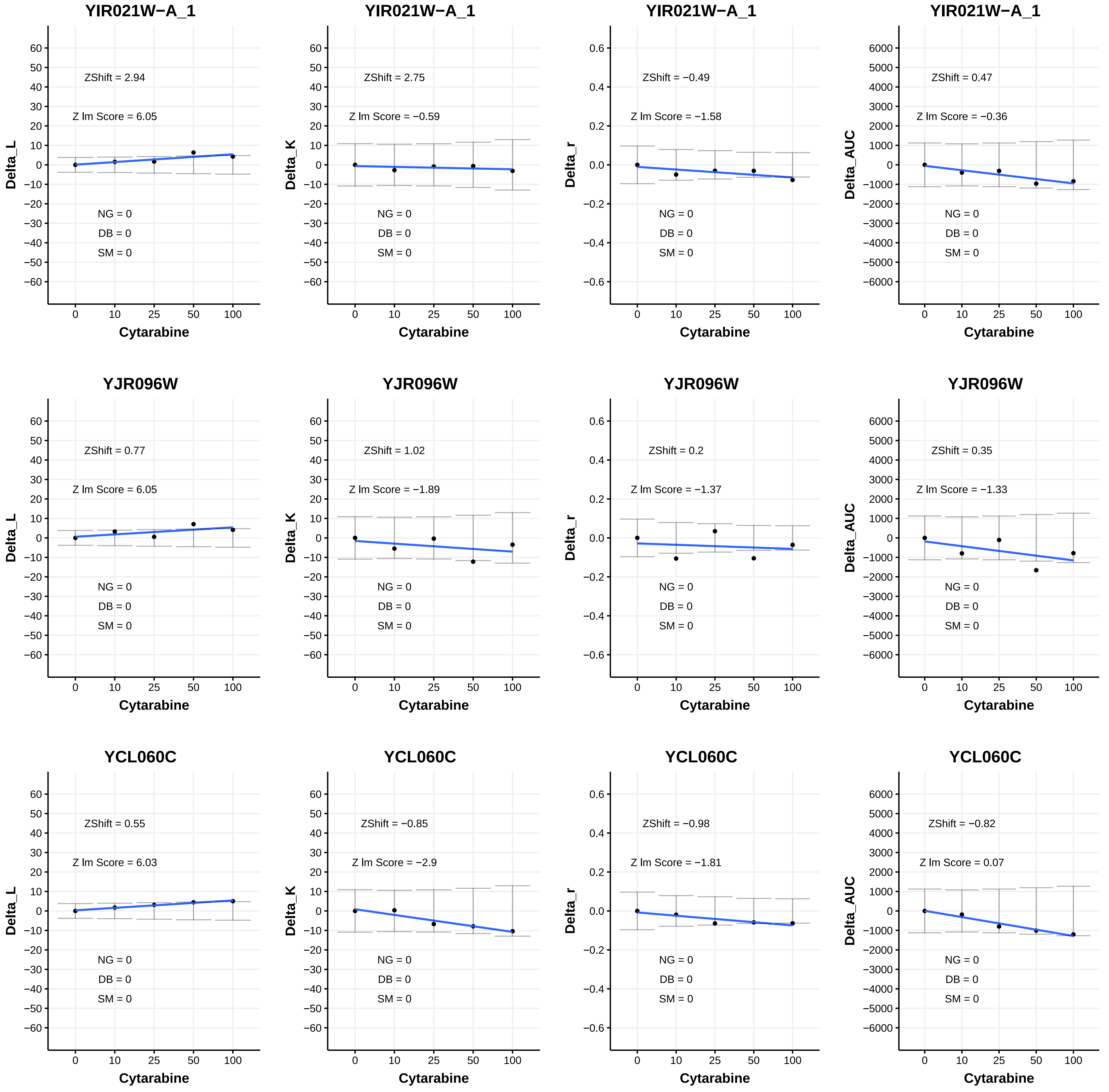
YIR021W−A_1
ZShift = 2.94
Z lm Score = 6.05
NG = 0
DB = 0
SM = 0
Delta_L
Cytarabine
YIR021W−A_1
ZShift = 2.75
Z lm Score = −0.59
NG = 0
DB = 0
SM = 0
Delta_K
Cytarabine
YIR021W−A_1
ZShift = −0.49
Z lm Score = −1.58
NG = 0
DB = 0
SM = 0
Delta_r
Cytarabine
YIR021W−A_1
ZShift = 0.47
Z lm Score = −0.36
NG = 0
DB = 0
SM = 0
Delta_AUC
Cytarabine
YJR096W
ZShift = 0.77
Z lm Score = 6.05
NG = 0
DB = 0
SM = 0
Delta_L
Cytarabine
YJR096W
ZShift = 1.02
Z lm Score = −1.89
NG = 0
DB = 0
SM = 0
Delta_K
Cytarabine
YJR096W
ZShift = 0.2
Z lm Score = −1.37
NG = 0
DB = 0
SM = 0
Delta_r
Cytarabine
YJR096W
ZShift = 0.35
Z lm Score = −1.33
NG = 0
DB = 0
SM = 0
Delta_AUC
Cytarabine
YCL060C
ZShift = 0.55
Z lm Score = 6.03
NG = 0
DB = 0
SM = 0
Delta_L
Cytarabine
YCL060C
ZShift = −0.85
Z lm Score = −2.9
NG = 0
DB = 0
SM = 0
Delta_K
Cytarabine
YCL060C
ZShift = −0.98
Z lm Score = −1.81
NG = 0
DB = 0
SM = 0
Delta_r
Cytarabine
YCL060C
ZShift = −0.82
Z lm Score = 0.07
NG = 0
DB = 0
SM = 0
Delta_AUC
Cytarabine

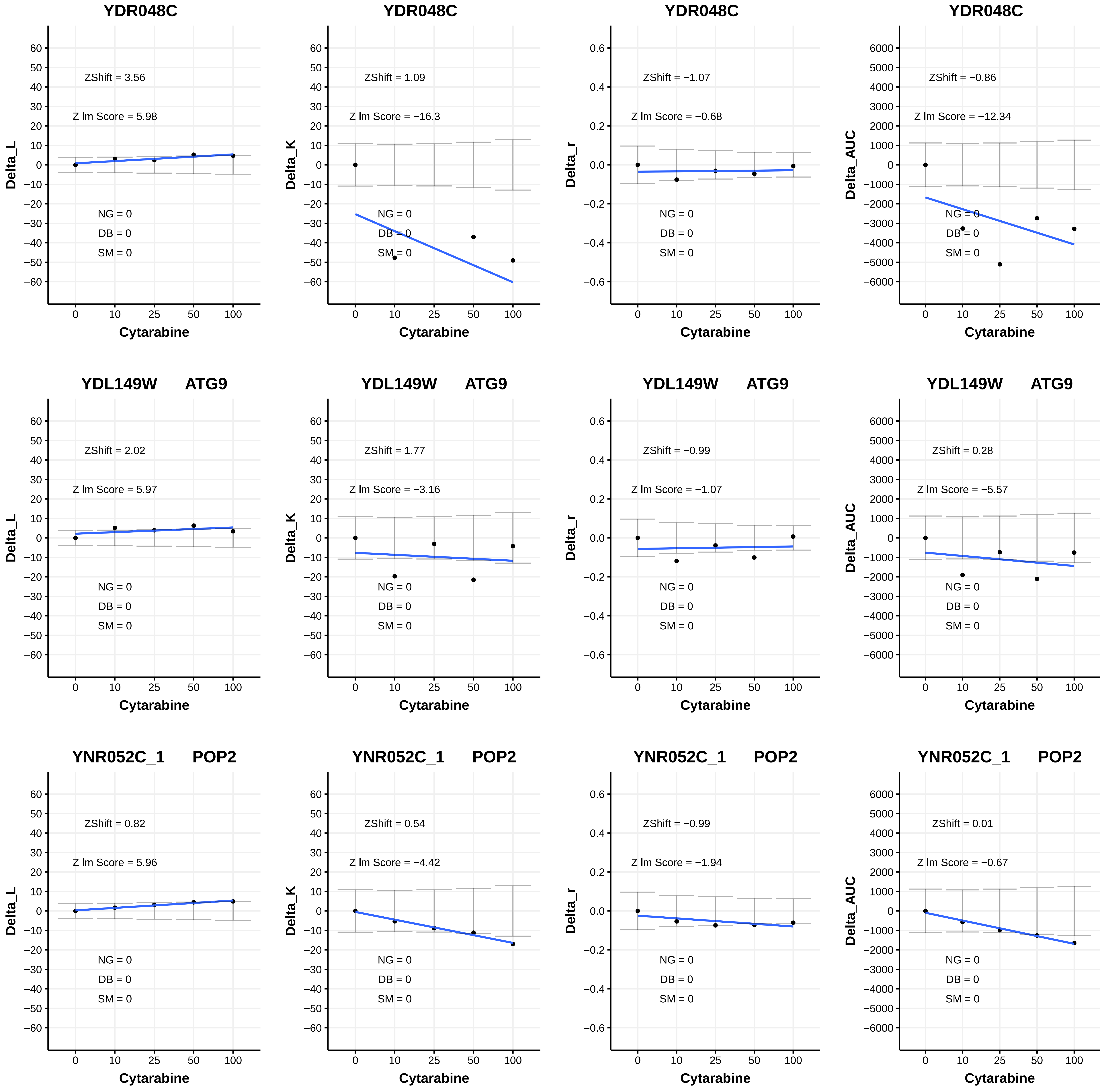
YDR048C
ZShift = 3.56
Z lm Score = 5.98
NG = 0
DB = 0
SM = 0
Delta_L
Cytarabine
YDR048C
ZShift = 1.09
Z lm Score = −16.3
NG = 0
DB = 0
SM = 0
Delta_K
Cytarabine
YDR048C
ZShift = −1.07
Z lm Score = −0.68
NG = 0
DB = 0
SM = 0
Delta_r
Cytarabine
YDR048C
ZShift = −0.86
Z lm Score = −12.34
NG = 0
DB = 0
SM = 0
Delta_AUC
Cytarabine
YDL149W ATG9
ZShift = 2.02
Z lm Score = 5.97
NG = 0
DB = 0
SM = 0
Delta_L
Cytarabine
YDL149W ATG9
ZShift = 1.77
Z lm Score = −3.16
NG = 0
DB = 0
SM = 0
Delta_K
Cytarabine
YDL149W ATG9
ZShift = −0.99
Z lm Score = −1.07
NG = 0
DB = 0
SM = 0
Delta_r
Cytarabine
YDL149W ATG9
ZShift = 0.28
Z lm Score = −5.57
NG = 0
DB = 0
SM = 0
Delta_AUC
Cytarabine
YNR052C_1 POP2
ZShift = 0.82
Z lm Score = 5.96
NG = 0
DB = 0
SM = 0
Delta_L
Cytarabine
YNR052C_1 POP2
ZShift = 0.54
Z lm Score = −4.42
NG = 0
DB = 0
SM = 0
Delta_K
Cytarabine
YNR052C_1 POP2
ZShift = −0.99
Z lm Score = −1.94
NG = 0
DB = 0
SM = 0
Delta_r
Cytarabine
YNR052C_1 POP2
ZShift = 0.01
Z lm Score = −0.67
NG = 0
DB = 0
SM = 0
Delta_AUC
Cytarabine

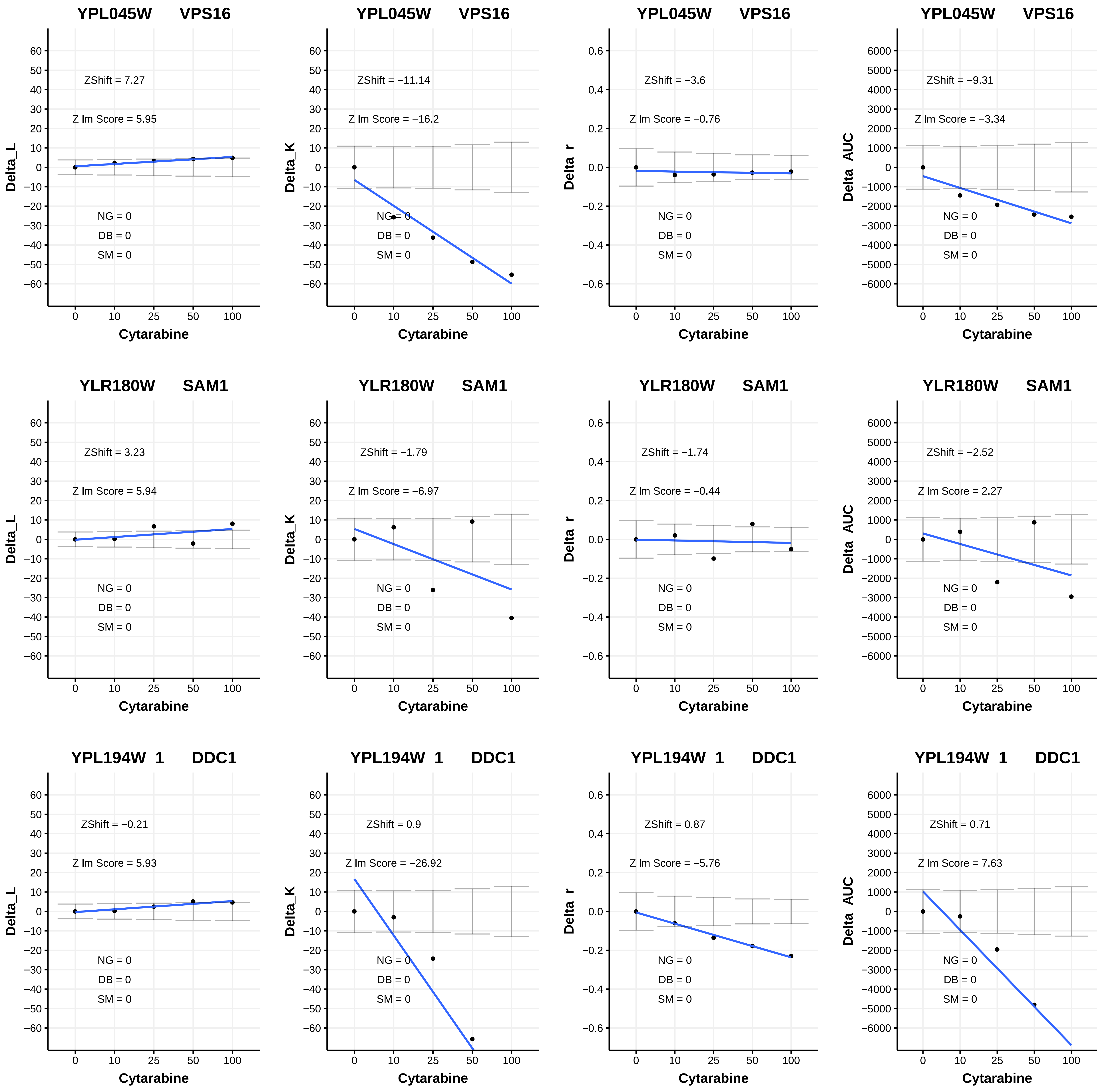
YPL045W VPS16
ZShift = 7.27
Z lm Score = 5.95
NG = 0
DB = 0
SM = 0
Delta_L
Cytarabine
YPL045W VPS16
ZShift = −11.14
Z lm Score = −16.2
NG = 0
DB = 0
SM = 0
Delta_K
Cytarabine
YPL045W VPS16
ZShift = −3.6
Z lm Score = −0.76
NG = 0
DB = 0
SM = 0
Delta_r
Cytarabine
YPL045W VPS16
ZShift = −9.31
Z lm Score = −3.34
NG = 0
DB = 0
SM = 0
Delta_AUC
Cytarabine
YLR180W SAM1
ZShift = 3.23
Z lm Score = 5.94
NG = 0
DB = 0
SM = 0
Delta_L
Cytarabine
YLR180W SAM1
ZShift = −1.79
Z lm Score = −6.97
NG = 0
DB = 0
SM = 0
Delta_K
Cytarabine
YLR180W SAM1
ZShift = −1.74
Z lm Score = −0.44
NG = 0
DB = 0
SM = 0
Delta_r
Cytarabine
YLR180W SAM1
ZShift = −2.52
Z lm Score = 2.27
NG = 0
DB = 0
SM = 0
Delta_AUC
Cytarabine
YPL194W_1 DDC1
ZShift = −0.21
Z lm Score = 5.93
NG = 0
DB = 0
SM = 0
Delta_L
Cytarabine
YPL194W_1 DDC1
ZShift = 0.9
Z lm Score = −26.92
NG = 0
DB = 0
SM = 0
Delta_K
Cytarabine
YPL194W_1 DDC1
ZShift = 0.87
Z lm Score = −5.76
NG = 0
DB = 0
SM = 0
Delta_r
Cytarabine
YPL194W_1 DDC1
ZShift = 0.71
Z lm Score = 7.63
NG = 0
DB = 0
SM = 0
Delta_AUC
Cytarabine

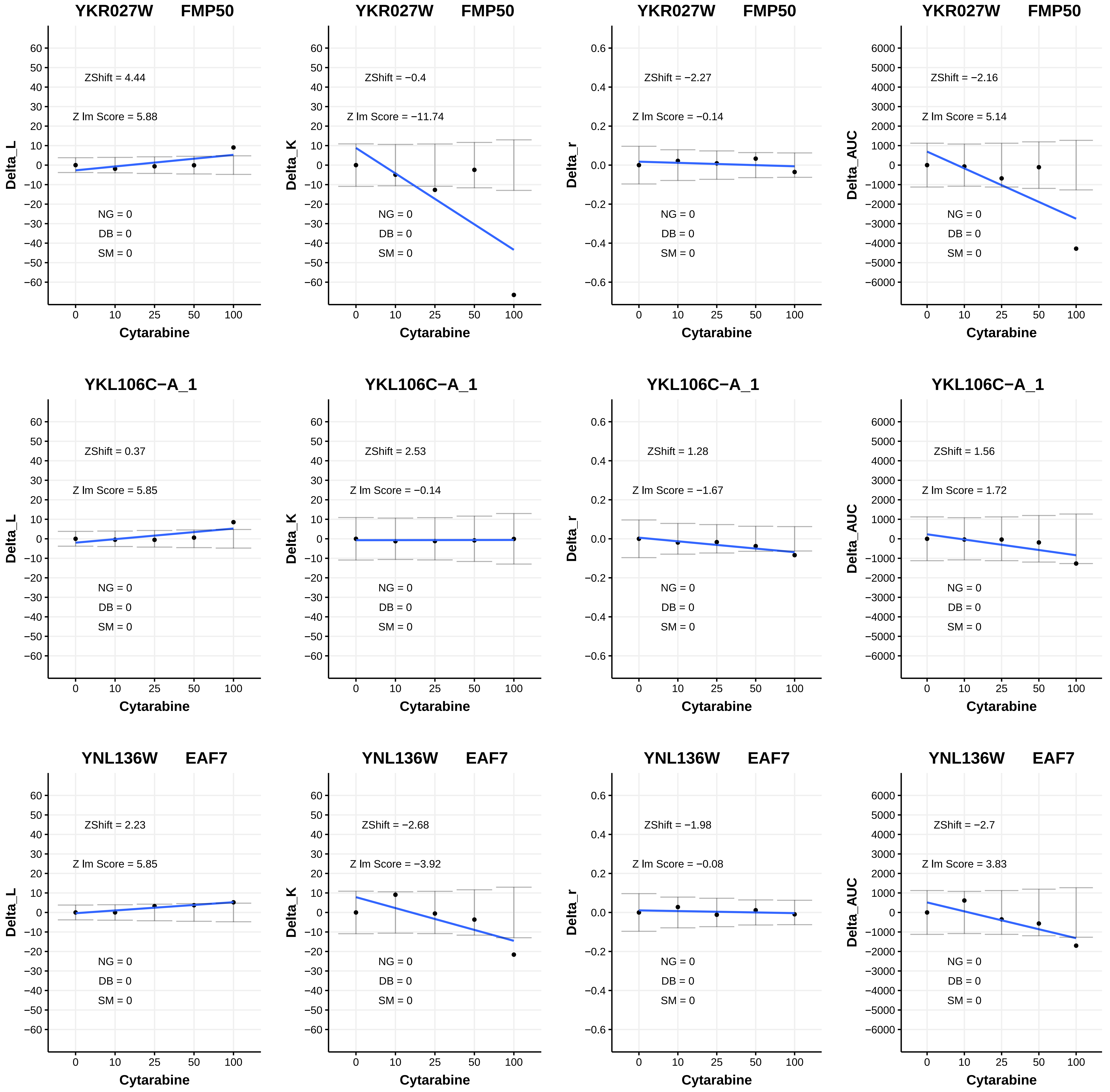

YKR027W FMP50
ZShift = 4.44
Z lm Score = 5.88
NG = 0
DB = 0
SM = 0
Delta_L
Cytarabine
YKR027W FMP50
ZShift = −0.4
Z lm Score = −11.74
NG = 0
DB = 0
SM = 0
Delta_K
Cytarabine
YKR027W FMP50
ZShift = −2.27
Z lm Score = −0.14
NG = 0
DB = 0
SM = 0
Delta_r
Cytarabine
YKR027W FMP50
ZShift = −2.16
Z lm Score = 5.14
NG = 0
DB = 0
SM = 0
Delta_AUC
Cytarabine
YKL106C−A_1
ZShift = 0.37
Z lm Score = 5.85
NG = 0
DB = 0
SM = 0
Delta_L
Cytarabine
YKL106C−A_1
ZShift = 2.53
Z lm Score = −0.14
NG = 0
DB = 0
SM = 0
Delta_K
Cytarabine
YKL106C−A_1
ZShift = 1.28
Z lm Score = −1.67
NG = 0
DB = 0
SM = 0
Delta_r
Cytarabine
YKL106C−A_1
ZShift = 1.56
Z lm Score = 1.72
NG = 0
DB = 0
SM = 0
Delta_AUC
Cytarabine
YNL136W EAF7
ZShift = 2.23
Z lm Score = 5.85
NG = 0
DB = 0
SM = 0
Delta_L
Cytarabine
YNL136W EAF7
ZShift = −2.68
Z lm Score = −3.92
NG = 0
DB = 0
SM = 0
Delta_K
Cytarabine
YNL136W EAF7
ZShift = −1.98
Z lm Score = −0.08
NG = 0
DB = 0
SM = 0
Delta_r
Cytarabine
YNL136W EAF7
ZShift = −2.7
Z lm Score = 3.83
NG = 0
DB = 0
SM = 0
Delta_AUC
Cytarabine

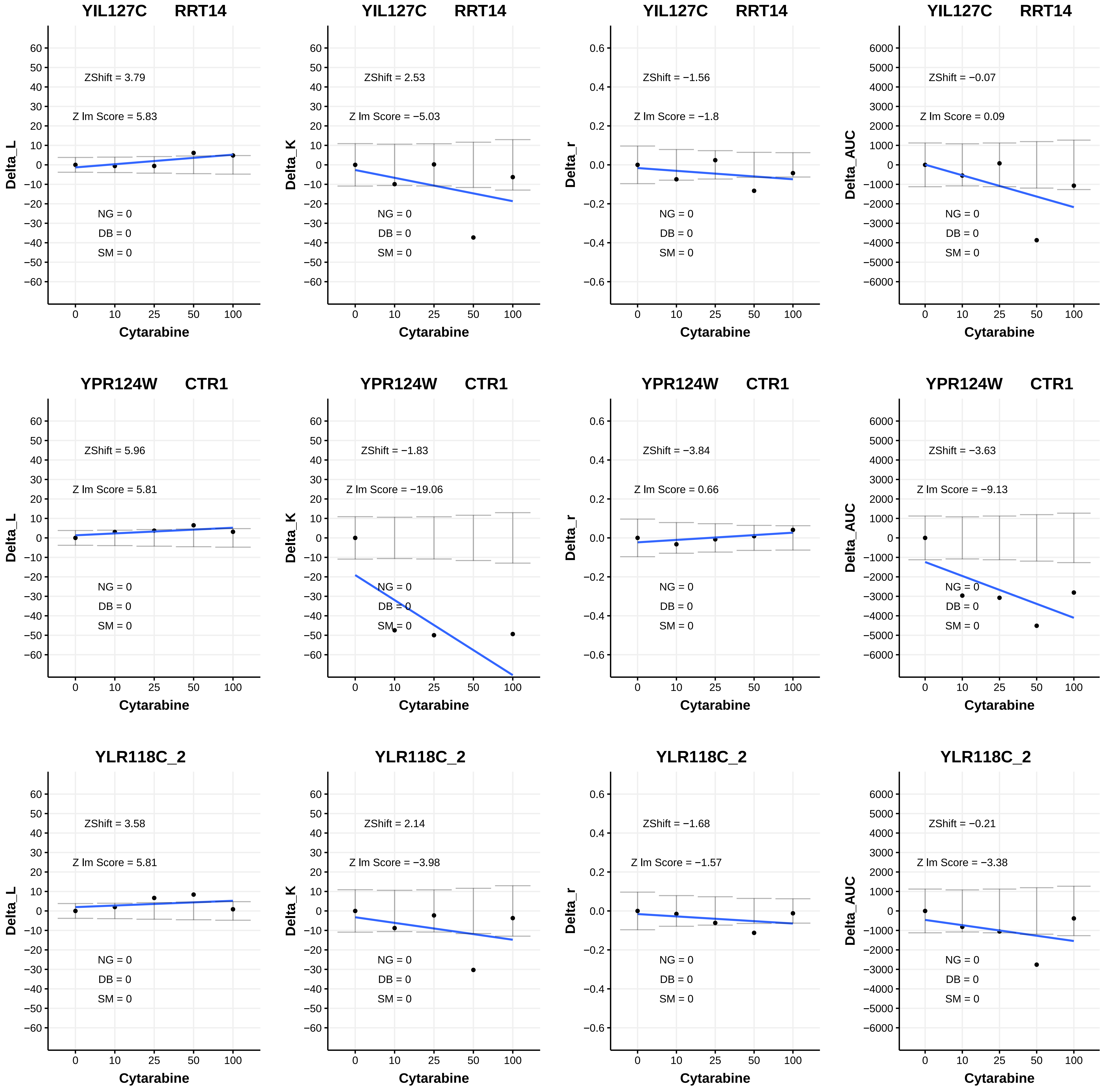
YIL127C RRT14
ZShift = 3.79
Z lm Score = 5.83
NG = 0
DB = 0
SM = 0
Delta_L
Cytarabine
YIL127C RRT14
ZShift = 2.53
Z lm Score = −5.03
NG = 0
DB = 0
SM = 0
Delta_K
Cytarabine
YIL127C RRT14
ZShift = −1.56
Z lm Score = −1.8
NG = 0
DB = 0
SM = 0
Delta_r
Cytarabine
YIL127C RRT14
ZShift = −0.07
Z lm Score = 0.09
NG = 0
DB = 0
SM = 0
Delta_AUC
Cytarabine
YPR124W CTR1
ZShift = 5.96
Z lm Score = 5.81
NG = 0
DB = 0
SM = 0
Delta_L
Cytarabine
YPR124W CTR1
ZShift = −1.83
Z lm Score = −19.06
NG = 0
DB = 0
SM = 0
Delta_K
Cytarabine
YPR124W CTR1
ZShift = −3.84
Z lm Score = 0.66
NG = 0
DB = 0
SM = 0
Delta_r
Cytarabine
YPR124W CTR1
ZShift = −3.63
Z lm Score = −9.13
NG = 0
DB = 0
SM = 0
Delta_AUC
Cytarabine
YLR118C_2
ZShift = 3.58
Z lm Score = 5.81
NG = 0
DB = 0
SM = 0
Delta_L
Cytarabine
YLR118C_2
ZShift = 2.14
Z lm Score = −3.98
NG = 0
DB = 0
SM = 0
Delta_K
Cytarabine
YLR118C_2
ZShift = −1.68
Z lm Score = −1.57
NG = 0
DB = 0
SM = 0
Delta_r
Cytarabine
YLR118C_2
ZShift = −0.21
Z lm Score = −3.38
NG = 0
DB = 0
SM = 0
Delta_AUC
Cytarabine

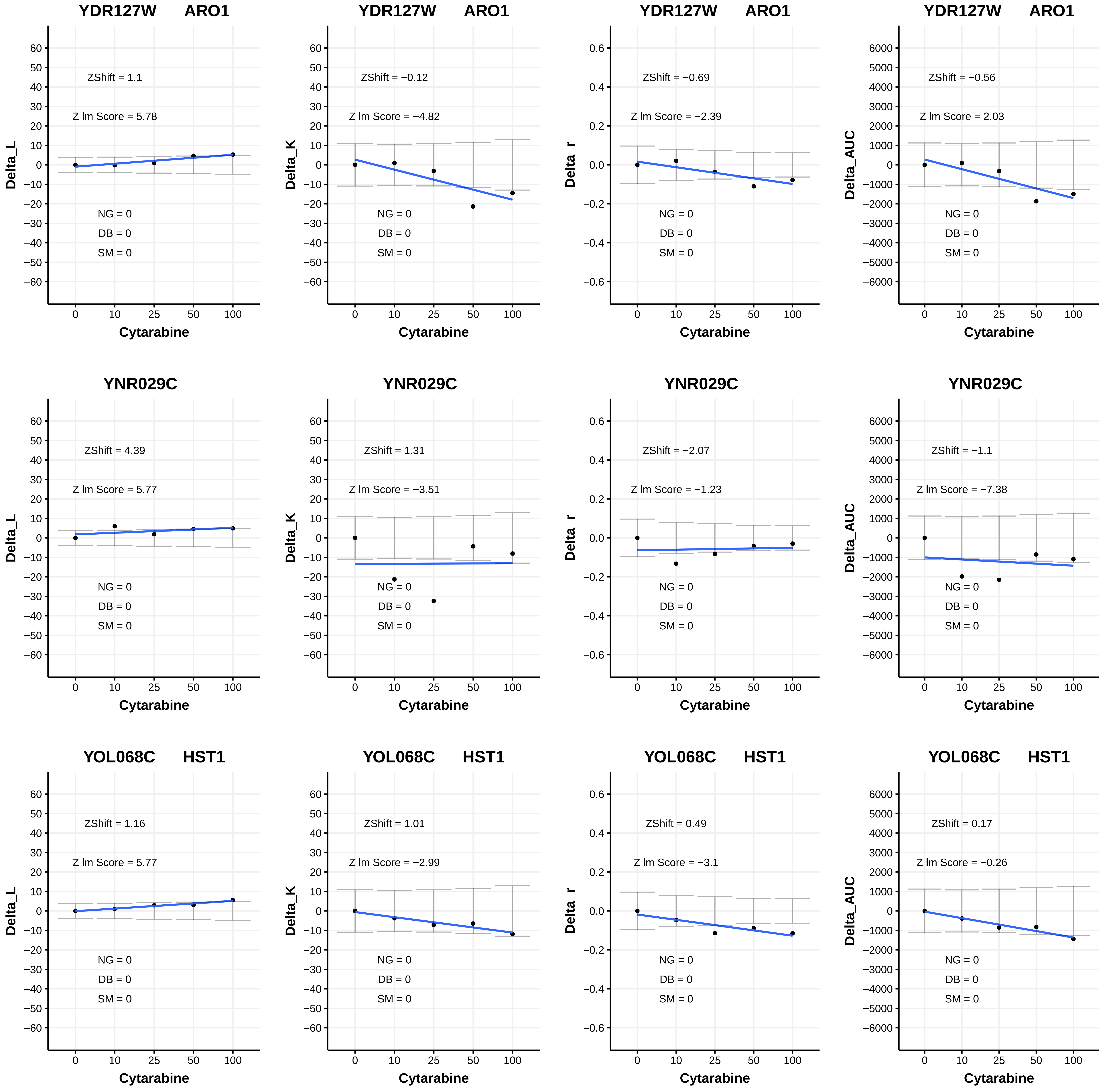
YDR127W ARO1
ZShift = 1.1
Z lm Score = 5.78
NG = 0
DB = 0
SM = 0
Delta_L
Cytarabine
YDR127W ARO1
ZShift = −0.12
Z lm Score = −4.82
NG = 0
DB = 0
SM = 0
Delta_K
Cytarabine
YDR127W ARO1
ZShift = −0.69
Z lm Score = −2.39
NG = 0
DB = 0
SM = 0
Delta_r
Cytarabine
YDR127W ARO1
ZShift = −0.56
Z lm Score = 2.03
NG = 0
DB = 0
SM = 0
Delta_AUC
Cytarabine
YNR029C
ZShift = 4.39
Z lm Score = 5.77
NG = 0
DB = 0
SM = 0
Delta_L
Cytarabine
YNR029C
ZShift = 1.31
Z lm Score = −3.51
NG = 0
DB = 0
SM = 0
Delta_K
Cytarabine
YNR029C
ZShift = −2.07
Z lm Score = −1.23
NG = 0
DB = 0
SM = 0
Delta_r
Cytarabine
YNR029C
ZShift = −1.1
Z lm Score = −7.38
NG = 0
DB = 0
SM = 0
Delta_AUC
Cytarabine
YOL068C HST1
ZShift = 1.16
Z lm Score = 5.77
NG = 0
DB = 0
SM = 0
Delta_L
Cytarabine
YOL068C HST1
ZShift = 1.01
Z lm Score = −2.99
NG = 0
DB = 0
SM = 0
Delta_K
Cytarabine
YOL068C HST1
ZShift = 0.49
Z lm Score = −3.1
NG = 0
DB = 0
SM = 0
Delta_r
Cytarabine
YOL068C HST1
ZShift = 0.17
Z lm Score = −0.26
NG = 0
DB = 0
SM = 0
Delta_AUC
Cytarabine

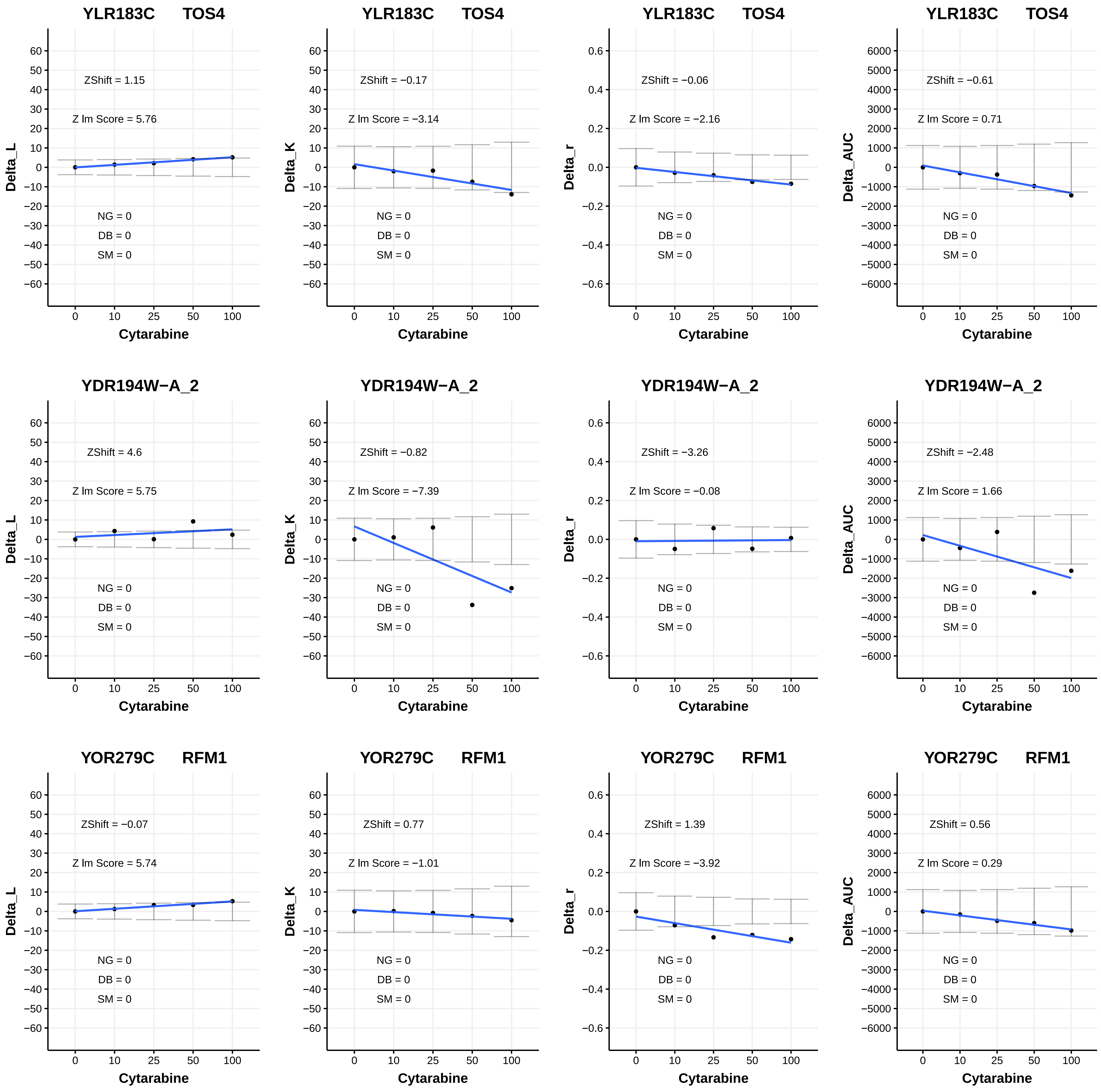

YLR183C TOS4
ZShift = 1.15
Z lm Score = 5.76
NG = 0
DB = 0
SM = 0
Delta_L
Cytarabine
YLR183C TOS4
ZShift = −0.17
Z lm Score = −3.14
NG = 0
DB = 0
SM = 0
Delta_K
Cytarabine
YLR183C TOS4
ZShift = −0.06
Z lm Score = −2.16
NG = 0
DB = 0
SM = 0
Delta_r
Cytarabine
YLR183C TOS4
ZShift = −0.61
Z lm Score = 0.71
NG = 0
DB = 0
SM = 0
Delta_AUC
Cytarabine
YDR194W−A_2
ZShift = 4.6
Z lm Score = 5.75
NG = 0
DB = 0
SM = 0
Delta_L
Cytarabine
YDR194W−A_2
ZShift = −0.82
Z lm Score = −7.39
NG = 0
DB = 0
SM = 0
Delta_K
Cytarabine
YDR194W−A_2
ZShift = −3.26
Z lm Score = −0.08
NG = 0
DB = 0
SM = 0
Delta_r
Cytarabine
YDR194W−A_2
ZShift = −2.48
Z lm Score = 1.66
NG = 0
DB = 0
SM = 0
Delta_AUC
Cytarabine
YOR279C RFM1
ZShift = −0.07
Z lm Score = 5.74
NG = 0
DB = 0
SM = 0
Delta_L
Cytarabine
YOR279C RFM1
ZShift = 0.77
Z lm Score = −1.01
NG = 0
DB = 0
SM = 0
Delta_K
Cytarabine
YOR279C RFM1
ZShift = 1.39
Z lm Score = −3.92
NG = 0
DB = 0
SM = 0
Delta_r
Cytarabine
YOR279C RFM1
ZShift = 0.56
Z lm Score = 0.29
NG = 0
DB = 0
SM = 0
Delta_AUC
Cytarabine

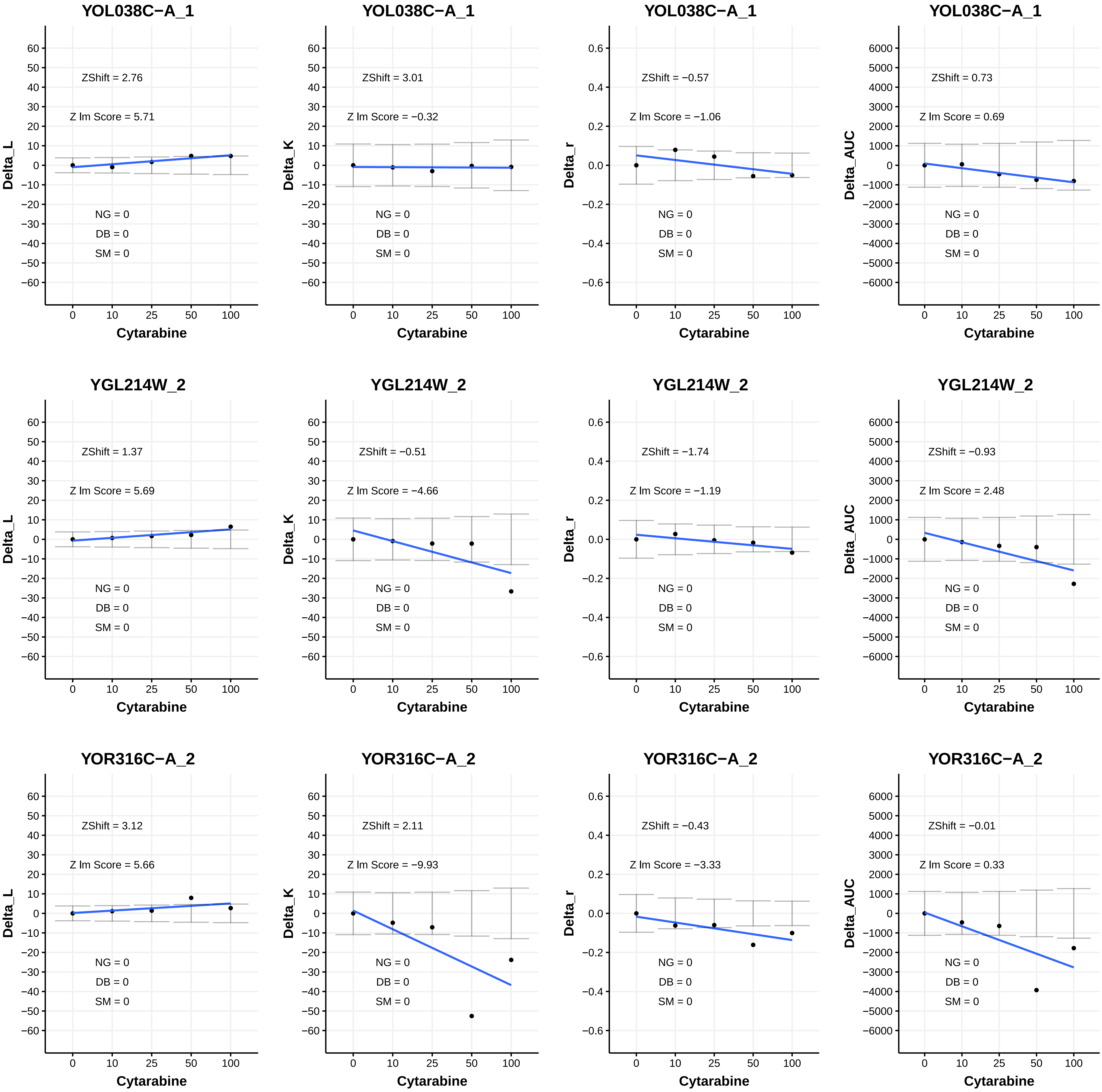
YOL038C−A_1
ZShift = 2.76
Z lm Score = 5.71
NG = 0
DB = 0
SM = 0
Delta_L
Cytarabine
YOL038C−A_1
ZShift = 3.01
Z lm Score = −0.32
NG = 0
DB = 0
SM = 0
Delta_K
Cytarabine
YOL038C−A_1
ZShift = −0.57
Z lm Score = −1.06
NG = 0
DB = 0
SM = 0
Delta_r
Cytarabine
YOL038C−A_1
ZShift = 0.73
Z lm Score = 0.69
NG = 0
DB = 0
SM = 0
Delta_AUC
Cytarabine
YGL214W_2
ZShift = 1.37
Z lm Score = 5.69
NG = 0
DB = 0
SM = 0
Delta_L
Cytarabine
YGL214W_2
ZShift = −0.51
Z lm Score = −4.66
NG = 0
DB = 0
SM = 0
Delta_K
Cytarabine
YGL214W_2
ZShift = −1.74
Z lm Score = −1.19
NG = 0
DB = 0
SM = 0
Delta_r
Cytarabine
YGL214W_2
ZShift = −0.93
Z lm Score = 2.48
NG = 0
DB = 0
SM = 0
Delta_AUC
Cytarabine
YOR316C−A_2
ZShift = 3.12
Z lm Score = 5.66
NG = 0
DB = 0
SM = 0
Delta_L
Cytarabine
YOR316C−A_2
ZShift = 2.11
Z lm Score = −9.93
NG = 0
DB = 0
SM = 0
Delta_K
Cytarabine
YOR316C−A_2
ZShift = −0.43
Z lm Score = −3.33
NG = 0
DB = 0
SM = 0
Delta_r
Cytarabine
YOR316C−A_2
ZShift = −0.01
Z lm Score = 0.33
NG = 0
DB = 0
SM = 0
Delta_AUC
Cytarabine

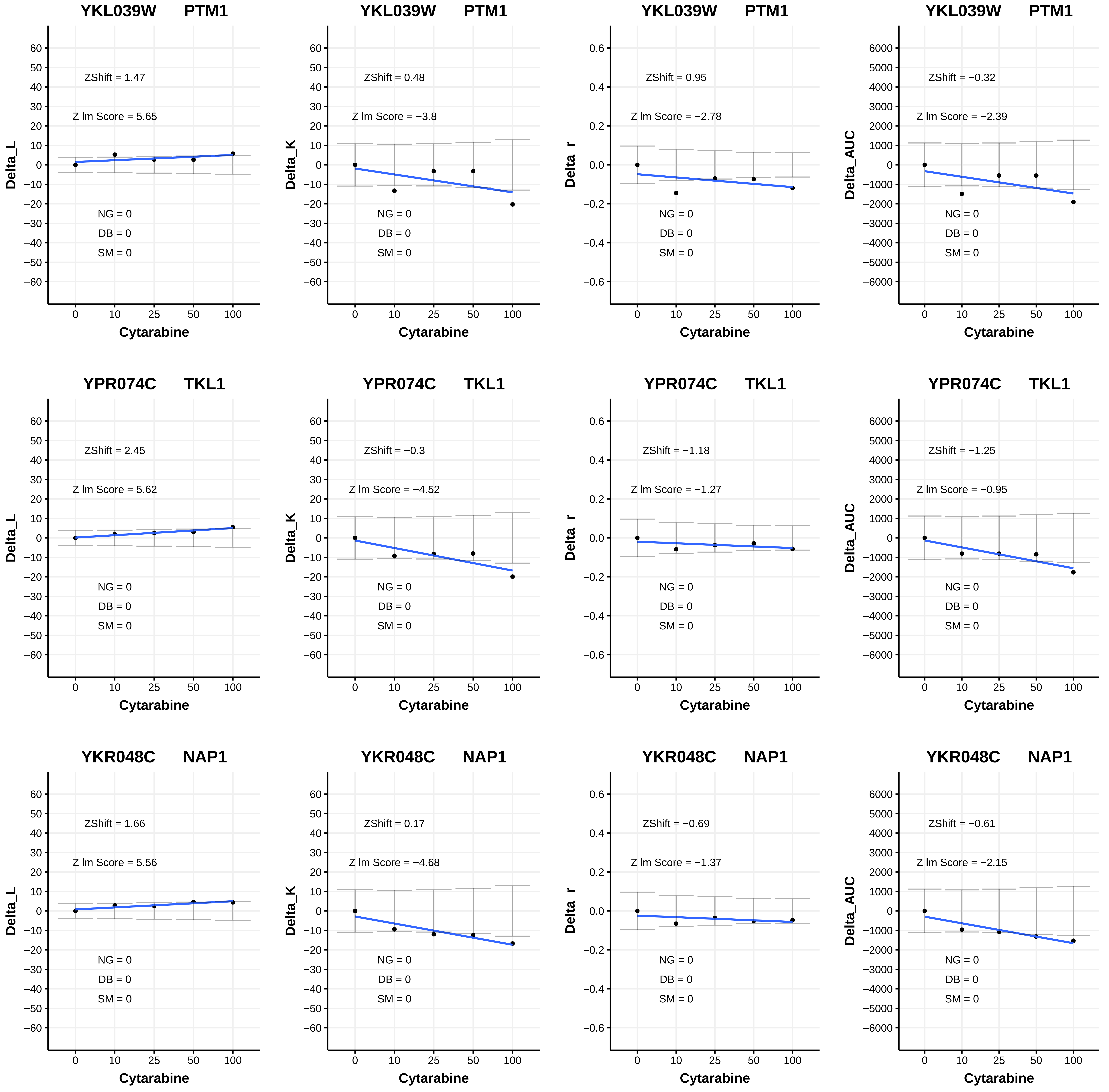

YKL039W PTM1
ZShift = 1.47
Z lm Score = 5.65
NG = 0
DB = 0
SM = 0
Delta_L
Cytarabine
YKL039W PTM1
ZShift = 0.48
Z lm Score = −3.8
NG = 0
DB = 0
SM = 0
Delta_K
Cytarabine
YKL039W PTM1
ZShift = 0.95
Z lm Score = −2.78
NG = 0
DB = 0
SM = 0
Delta_r
Cytarabine
YKL039W PTM1
ZShift = −0.32
Z lm Score = −2.39
NG = 0
DB = 0
SM = 0
Delta_AUC
Cytarabine
YPR074C TKL1
ZShift = 2.45
Z lm Score = 5.62
NG = 0
DB = 0
SM = 0
Delta_L
Cytarabine
YPR074C TKL1
ZShift = −0.3
Z lm Score = −4.52
NG = 0
DB = 0
SM = 0
Delta_K
Cytarabine
YPR074C TKL1
ZShift = −1.18
Z lm Score = −1.27
NG = 0
DB = 0
SM = 0
Delta_r
Cytarabine
YPR074C TKL1
ZShift = −1.25
Z lm Score = −0.95
NG = 0
DB = 0
SM = 0
Delta_AUC
Cytarabine
YKR048C NAP1
ZShift = 1.66
Z lm Score = 5.56
NG = 0
DB = 0
SM = 0
Delta_L
Cytarabine
YKR048C NAP1
ZShift = 0.17
Z lm Score = −4.68
NG = 0
DB = 0
SM = 0
Delta_K
Cytarabine
YKR048C NAP1
ZShift = −0.69
Z lm Score = −1.37
NG = 0
DB = 0
SM = 0
Delta_r
Cytarabine
YKR048C NAP1
ZShift = −0.61
Z lm Score = −2.15
NG = 0
DB = 0
SM = 0
Delta_AUC
Cytarabine

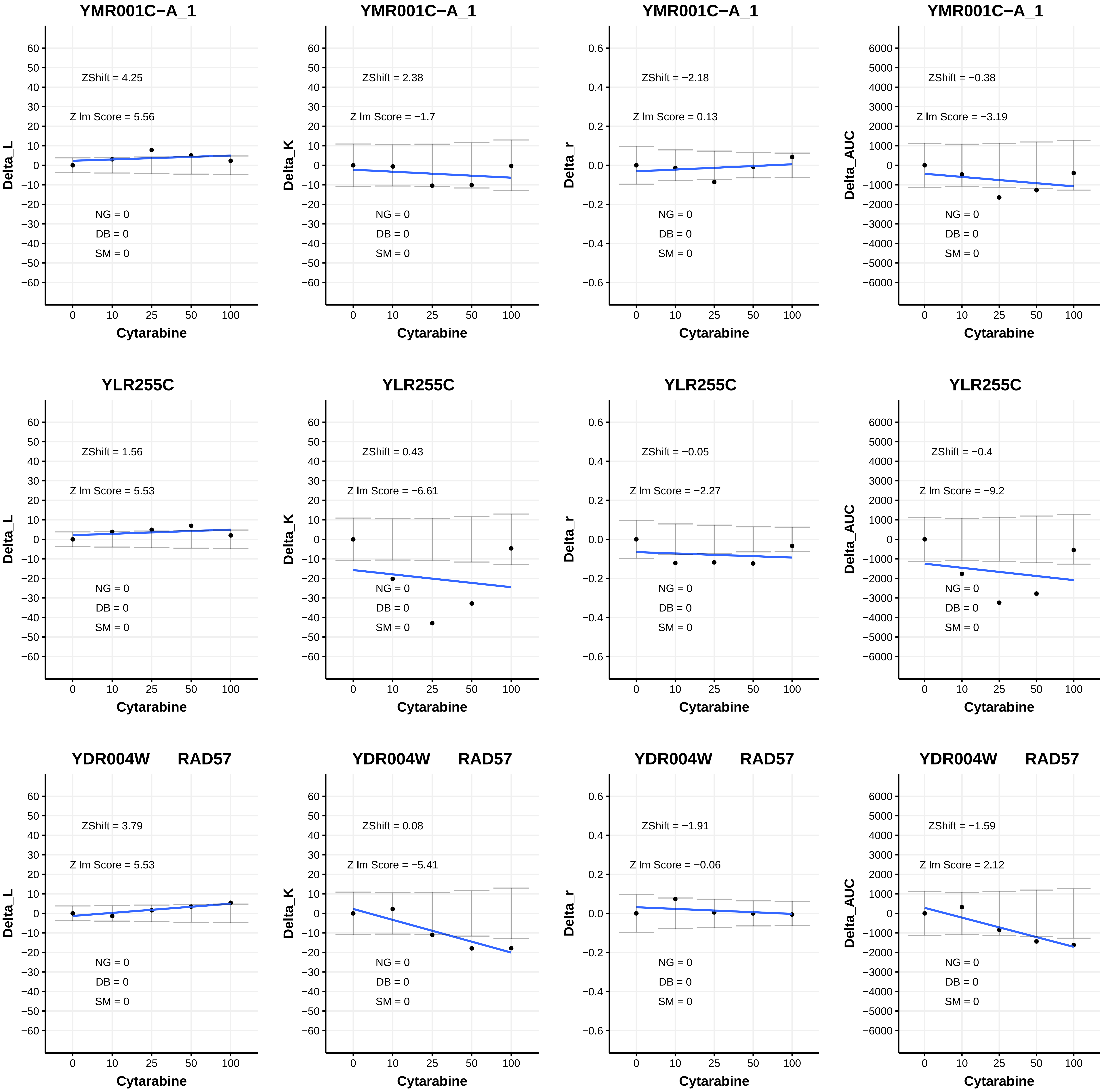
YMR001C−A_1
ZShift = 4.25
Z lm Score = 5.56
NG = 0
DB = 0
SM = 0
Delta_L
Cytarabine
YMR001C−A_1
ZShift = 2.38
Z lm Score = −1.7
NG = 0
DB = 0
SM = 0
Delta_K
Cytarabine
YMR001C−A_1
ZShift = −2.18
Z lm Score = 0.13
NG = 0
DB = 0
SM = 0
Delta_r
Cytarabine
YMR001C−A_1
ZShift = −0.38
Z lm Score = −3.19
NG = 0
DB = 0
SM = 0
Delta_AUC
Cytarabine
YLR255C
ZShift = 1.56
Z lm Score = 5.53
NG = 0
DB = 0
SM = 0
Delta_L
Cytarabine
YLR255C
ZShift = 0.43
Z lm Score = −6.61
NG = 0
DB = 0
SM = 0
Delta_K
Cytarabine
YLR255C
ZShift = −0.05
Z lm Score = −2.27
NG = 0
DB = 0
SM = 0
Delta_r
Cytarabine
YLR255C
ZShift = −0.4
Z lm Score = −9.2
NG = 0
DB = 0
SM = 0
Delta_AUC
Cytarabine
YDR004W RAD57
ZShift = 3.79
Z lm Score = 5.53
NG = 0
DB = 0
SM = 0
Delta_L
Cytarabine
YDR004W RAD57
ZShift = 0.08
Z lm Score = −5.41
NG = 0
DB = 0
SM = 0
Delta_K
Cytarabine
YDR004W RAD57
ZShift = −1.91
Z lm Score = −0.06
NG = 0
DB = 0
SM = 0
Delta_r
Cytarabine
YDR004W RAD57
ZShift = −1.59
Z lm Score = 2.12
NG = 0
DB = 0
SM = 0
Delta_AUC
Cytarabine
0 10 25 50 100

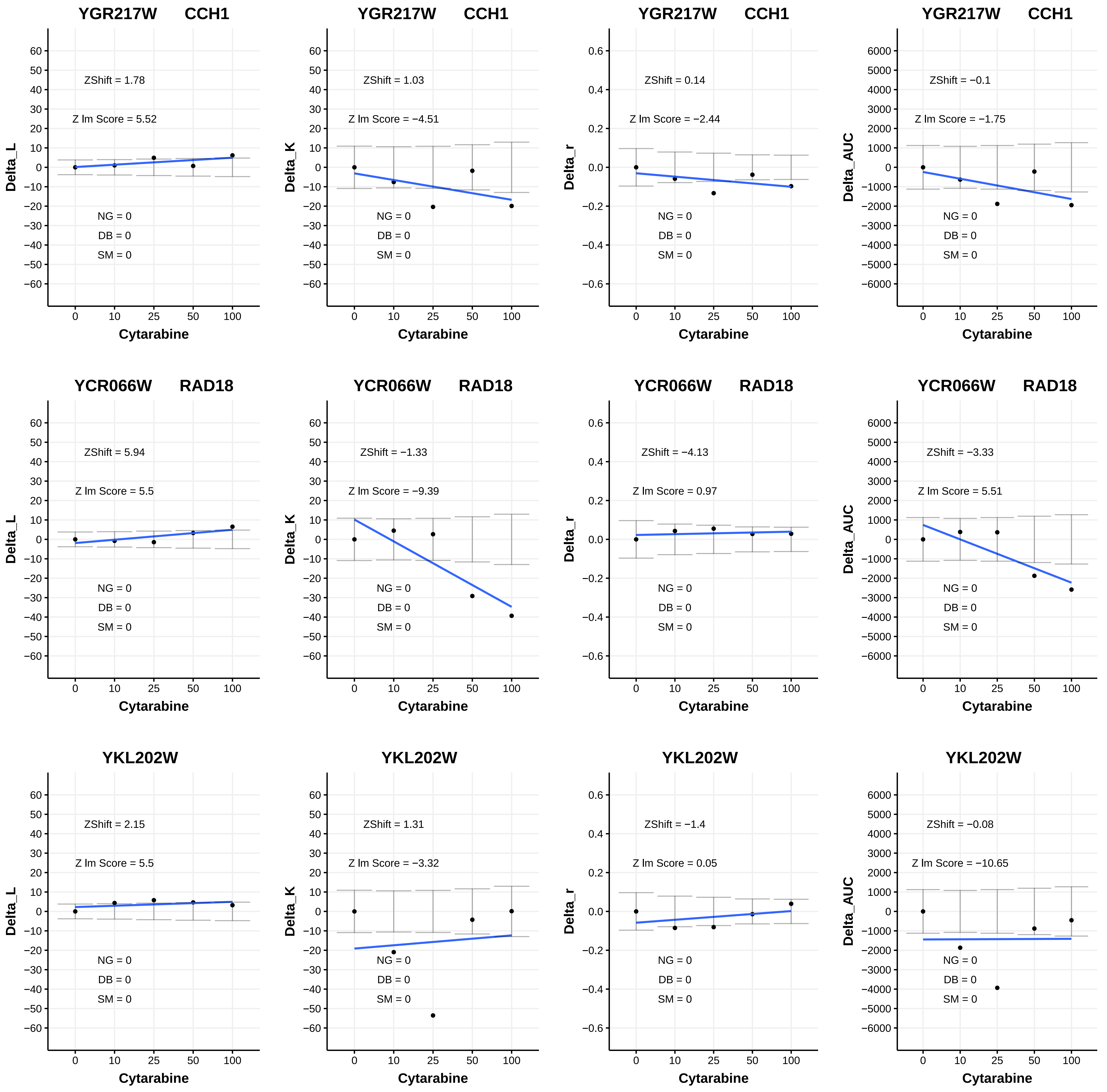
YGR217W CCH1
ZShift = 1.78
Z lm Score = 5.52
NG = 0
DB = 0
SM = 0
Delta_L
Cytarabine
YGR217W CCH1
ZShift = 1.03
Z lm Score = −4.51
NG = 0
DB = 0
SM = 0
Delta_K
Cytarabine
YGR217W CCH1
ZShift = 0.14
Z lm Score = −2.44
NG = 0
DB = 0
SM = 0
Delta_r
Cytarabine
YGR217W CCH1
ZShift = −0.1
Z lm Score = −1.75
NG = 0
DB = 0
SM = 0
Delta_AUC
Cytarabine
YCR066W RAD18
ZShift = 5.94
Z lm Score = 5.5
NG = 0
DB = 0
SM = 0
Delta_L
Cytarabine
YCR066W RAD18
ZShift = −1.33
Z lm Score = −9.39
NG = 0
DB = 0
SM = 0
Delta_K
Cytarabine
YCR066W RAD18
ZShift = −4.13
Z lm Score = 0.97
NG = 0
DB = 0
SM = 0
Delta_r
Cytarabine
YCR066W RAD18
ZShift = −3.33
Z lm Score = 5.51
NG = 0
DB = 0
SM = 0
Delta_AUC
Cytarabine
YKL202W
ZShift = 2.15
Z lm Score = 5.5
NG = 0
DB = 0
SM = 0
Delta_L
Cytarabine
YKL202W
ZShift = 1.31
Z lm Score = −3.32
NG = 0
DB = 0
SM = 0
Delta_K
Cytarabine
YKL202W
ZShift = −1.4
Z lm Score = 0.05
NG = 0
DB = 0
SM = 0
Delta_r
Cytarabine
YKL202W
ZShift = −0.08
Z lm Score = −10.65
NG = 0
DB = 0
SM = 0
Delta_AUC
Cytarabine

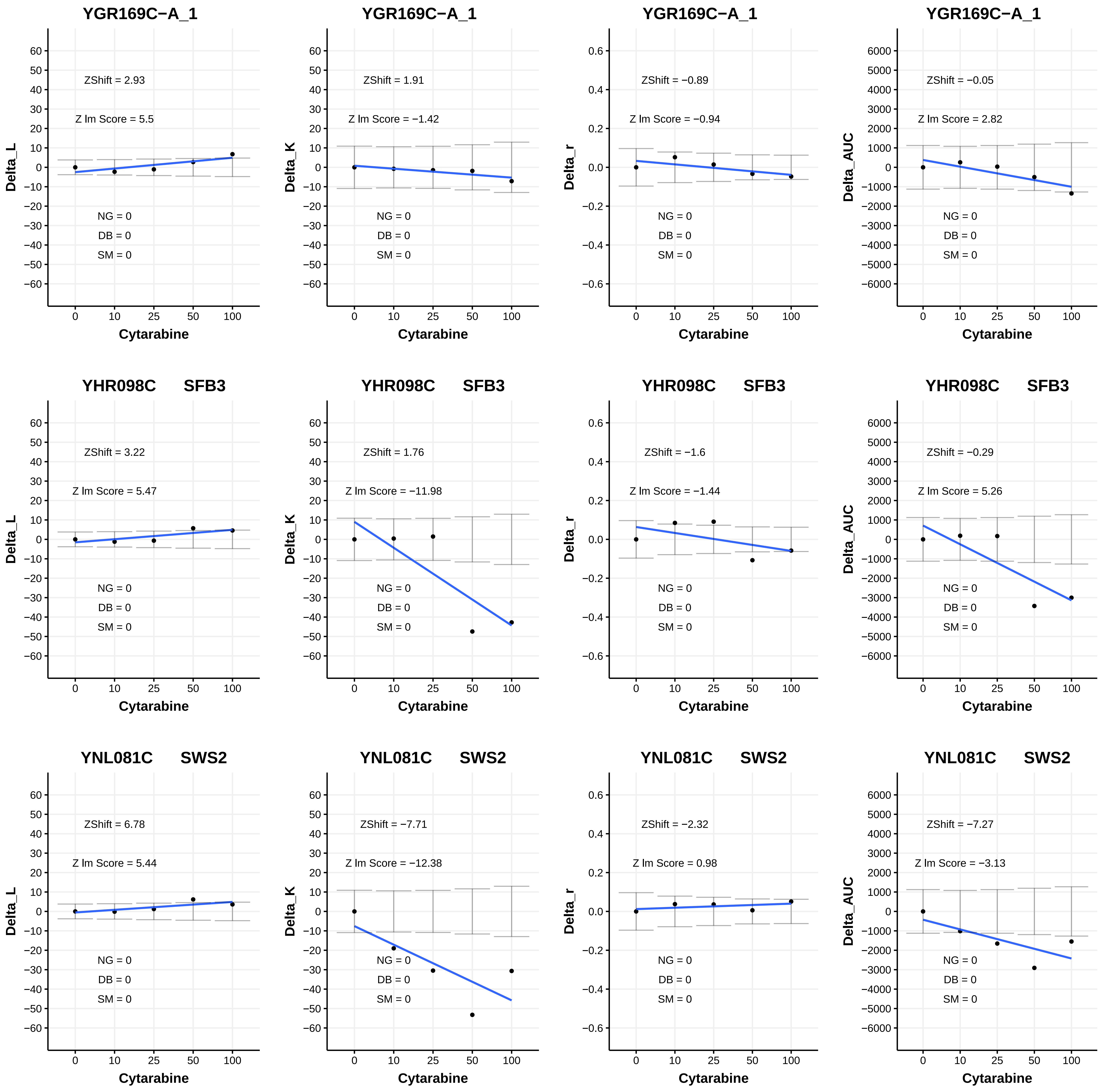
YGR169C−A_1
ZShift = 2.93
Z lm Score = 5.5
NG = 0
DB = 0
SM = 0
Delta_L
Cytarabine
YGR169C−A_1
ZShift = 1.91
Z lm Score = −1.42
NG = 0
DB = 0
SM = 0
Delta_K
Cytarabine
YGR169C−A_1
ZShift = −0.89
Z lm Score = −0.94
NG = 0
DB = 0
SM = 0
Delta_r
Cytarabine
YGR169C−A_1
ZShift = −0.05
Z lm Score = 2.82
NG = 0
DB = 0
SM = 0
Delta_AUC
Cytarabine
YHR098C SFB3
ZShift = 3.22
Z lm Score = 5.47
NG = 0
DB = 0
SM = 0
Delta_L
Cytarabine
YHR098C SFB3
ZShift = 1.76
Z lm Score = −11.98
NG = 0
DB = 0
SM = 0
Delta_K
Cytarabine
YHR098C SFB3
ZShift = −1.6
Z lm Score = −1.44
NG = 0
DB = 0
SM = 0
Delta_r
Cytarabine
YHR098C SFB3
ZShift = −0.29
Z lm Score = 5.26
NG = 0
DB = 0
SM = 0
Delta_AUC
Cytarabine
YNL081C SWS2
ZShift = 6.78
Z lm Score = 5.44
NG = 0
DB = 0
SM = 0
Delta_L
Cytarabine
YNL081C SWS2
ZShift = −7.71
Z lm Score = −12.38
NG = 0
DB = 0
SM = 0
Delta_K
Cytarabine
YNL081C SWS2
ZShift = −2.32
Z lm Score = 0.98
NG = 0
DB = 0
SM = 0
Delta_r
Cytarabine
YNL081C SWS2
ZShift = −7.27
Z lm Score = −3.13
NG = 0
DB = 0
SM = 0
Delta_AUC
Cytarabine

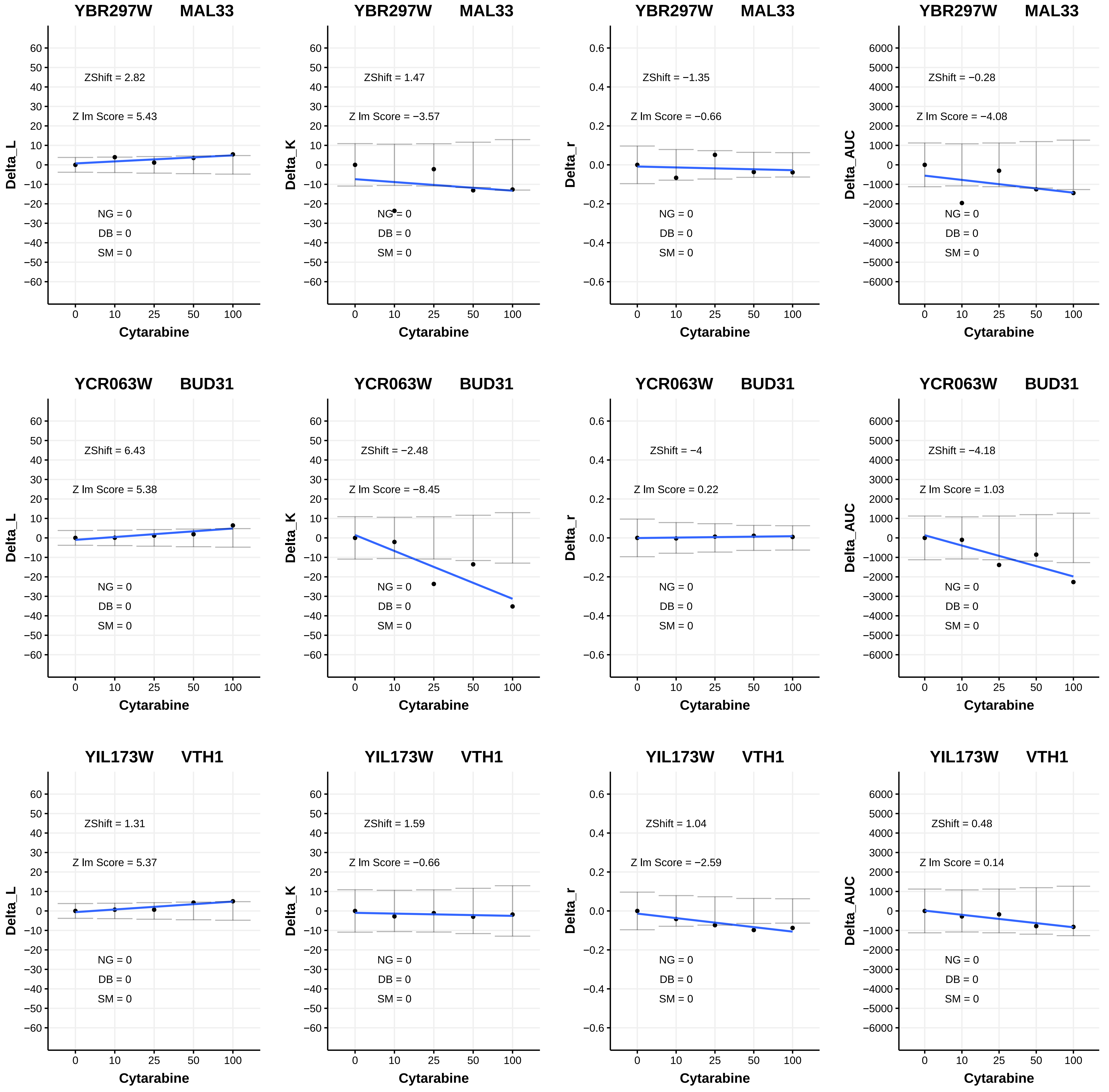
YBR297W MAL33
ZShift = 2.82
Z lm Score = 5.43
NG = 0
DB = 0
SM = 0
Delta_L
Cytarabine
YBR297W MAL33
ZShift = 1.47
Z lm Score = −3.57
NG = 0
DB = 0
SM = 0
Delta_K
Cytarabine
YBR297W MAL33
ZShift = −1.35
Z lm Score = −0.66
NG = 0
DB = 0
SM = 0
Delta_r
Cytarabine
YBR297W MAL33
ZShift = −0.28
Z lm Score = −4.08
NG = 0
DB = 0
SM = 0
Delta_AUC
Cytarabine
YCR063W BUD31
ZShift = 6.43
Z lm Score = 5.38
NG = 0
DB = 0
SM = 0
Delta_L
Cytarabine
YCR063W BUD31
ZShift = −2.48
Z lm Score = −8.45
NG = 0
DB = 0
SM = 0
Delta_K
Cytarabine
YCR063W BUD31
ZShift = −4
Z lm Score = 0.22
NG = 0
DB = 0
SM = 0
Delta_r
Cytarabine
YCR063W BUD31
ZShift = −4.18
Z lm Score = 1.03
NG = 0
DB = 0
SM = 0
Delta_AUC
Cytarabine
YIL173W VTH1
ZShift = 1.31
Z lm Score = 5.37
NG = 0
DB = 0
SM = 0
Delta_L
Cytarabine
YIL173W VTH1
ZShift = 1.59
Z lm Score = −0.66
NG = 0
DB = 0
SM = 0
Delta_K
Cytarabine
YIL173W VTH1
ZShift = 1.04
Z lm Score = −2.59
NG = 0
DB = 0
SM = 0
Delta_r
Cytarabine
YIL173W VTH1
ZShift = 0.48
Z lm Score = 0.14
NG = 0
DB = 0
SM = 0
Delta_AUC
Cytarabine

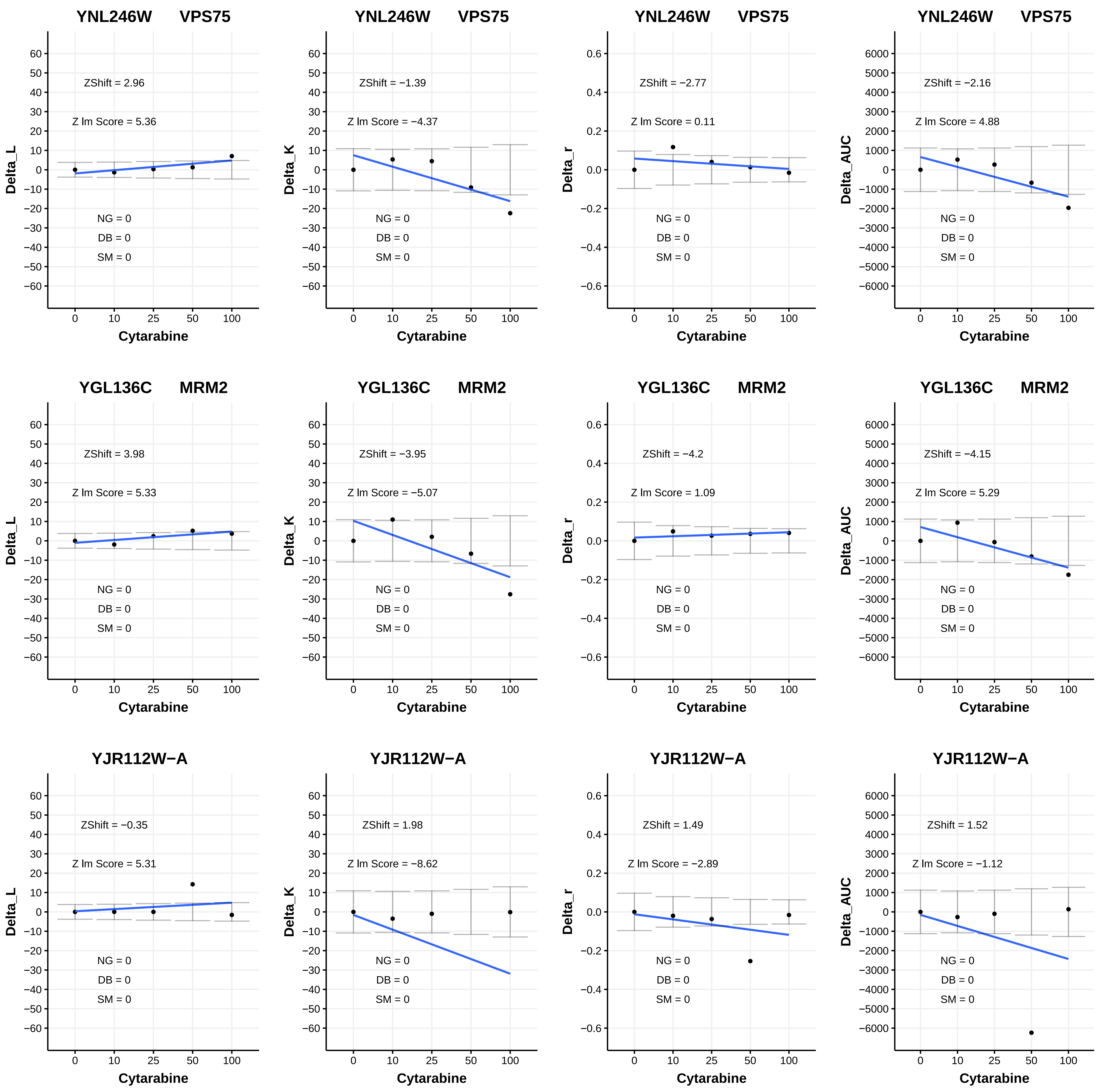
YNL246W VPS75
ZShift = 2.96
Z lm Score = 5.36
NG = 0
DB = 0
SM = 0
Delta_L
Cytarabine
YNL246W VPS75
ZShift = −1.39
Z lm Score = −4.37
NG = 0
DB = 0
SM = 0
Delta_K
Cytarabine
YNL246W VPS75
ZShift = −2.77
Z lm Score = 0.11
NG = 0
DB = 0
SM = 0
Delta_r
Cytarabine
YNL246W VPS75
ZShift = −2.16
Z lm Score = 4.88
NG = 0
DB = 0
SM = 0
Delta_AUC
Cytarabine
YGL136C MRM2
ZShift = 3.98
Z lm Score = 5.33
NG = 0
DB = 0
SM = 0
Delta_L
Cytarabine
YGL136C MRM2
ZShift = −3.95
Z lm Score = −5.07
NG = 0
DB = 0
SM = 0
Delta_K
Cytarabine
YGL136C MRM2
ZShift = −4.2
Z lm Score = 1.09
NG = 0
DB = 0
SM = 0
Delta_r
Cytarabine
YGL136C MRM2
ZShift = −4.15
Z lm Score = 5.29
NG = 0
DB = 0
SM = 0
Delta_AUC
Cytarabine
YJR112W−A
ZShift = −0.35
Z lm Score = 5.31
NG = 0
DB = 0
SM = 0
Delta_L
Cytarabine
YJR112W−A
ZShift = 1.98
Z lm Score = −8.62
NG = 0
DB = 0
SM = 0
Delta_K
Cytarabine
YJR112W−A
ZShift = 1.49
Z lm Score = −2.89
NG = 0
DB = 0
SM = 0
Delta_r
Cytarabine
YJR112W−A
ZShift = 1.52
Z lm Score = −1.12
NG = 0
DB = 0
SM = 0
Delta_AUC
Cytarabine

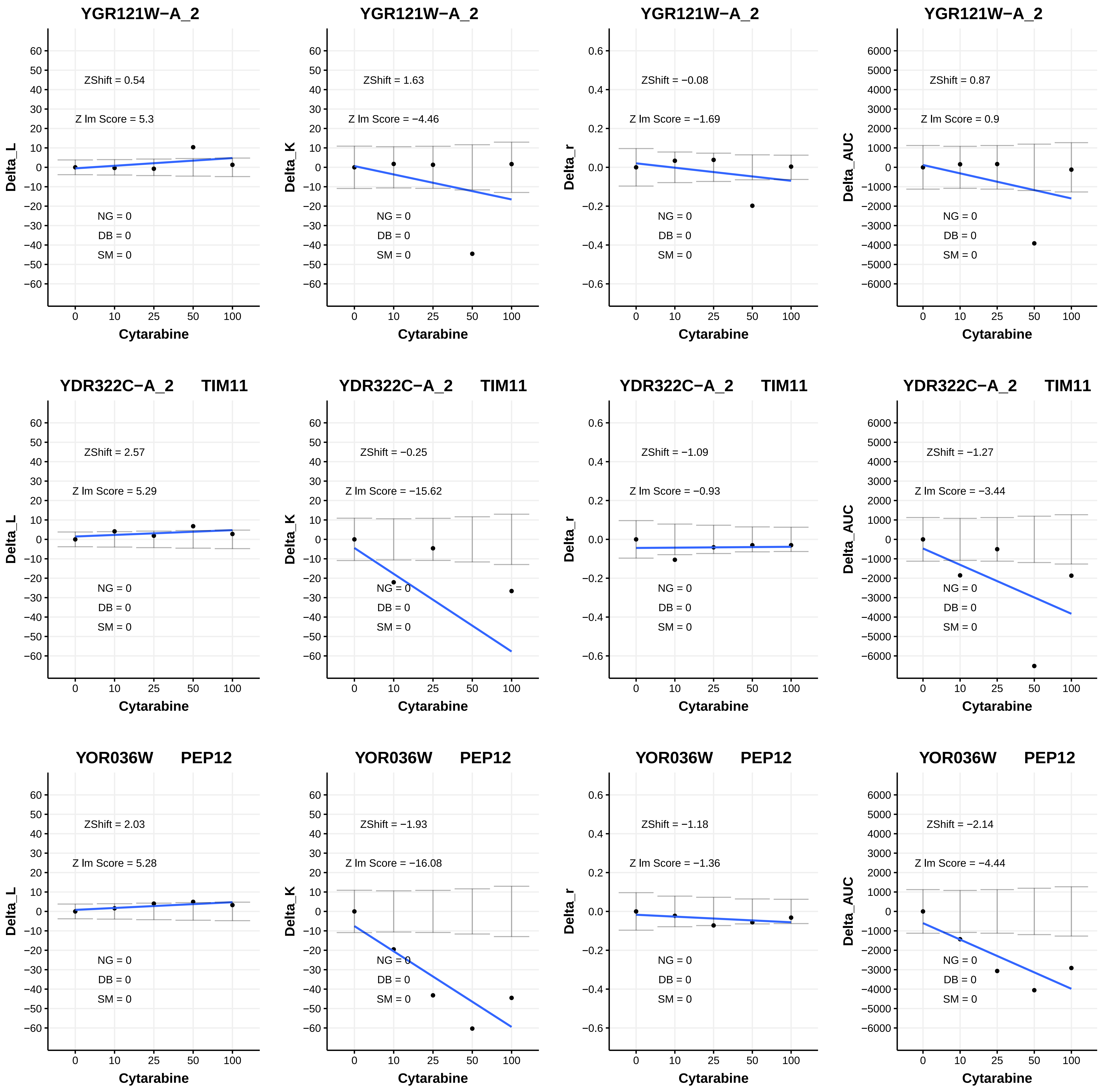

YGR121W−A_2
ZShift = 0.54
Z lm Score = 5.3
NG = 0
DB = 0
SM = 0
Delta_L
Cytarabine
YGR121W−A_2
ZShift = 1.63
Z lm Score = −4.46
NG = 0
DB = 0
SM = 0
Delta_K
Cytarabine
YGR121W−A_2
ZShift = −0.08
Z lm Score = −1.69
NG = 0
DB = 0
SM = 0
Delta_r
Cytarabine
YGR121W−A_2
ZShift = 0.87
Z lm Score = 0.9
NG = 0
DB = 0
SM = 0
Delta_AUC
Cytarabine
YDR322C−A_2 TIM11
ZShift = 2.57
Z lm Score = 5.29
NG = 0
DB = 0
SM = 0
Delta_L
Cytarabine
YDR322C−A_2 TIM11
ZShift = −0.25
Z lm Score = −15.62
NG = 0
DB = 0
SM = 0
Delta_K
Cytarabine
YDR322C−A_2 TIM11
ZShift = −1.09
Z lm Score = −0.93
NG = 0
DB = 0
SM = 0
Delta_r
Cytarabine
YDR322C−A_2 TIM11
ZShift = −1.27
Z lm Score = −3.44
NG = 0
DB = 0
SM = 0
Delta_AUC
Cytarabine
YOR036W PEP12
ZShift = 2.03
Z lm Score = 5.28
NG = 0
DB = 0
SM = 0
Delta_L
Cytarabine
YOR036W PEP12
ZShift = −1.93
Z lm Score = −16.08
NG = 0
DB = 0
SM = 0
Delta_K
Cytarabine
YOR036W PEP12
ZShift = −1.18
Z lm Score = −1.36
NG = 0
DB = 0
SM = 0
Delta_r
Cytarabine
YOR036W PEP12
ZShift = −2.14
Z lm Score = −4.44
NG = 0
DB = 0
SM = 0
Delta_AUC
Cytarabine

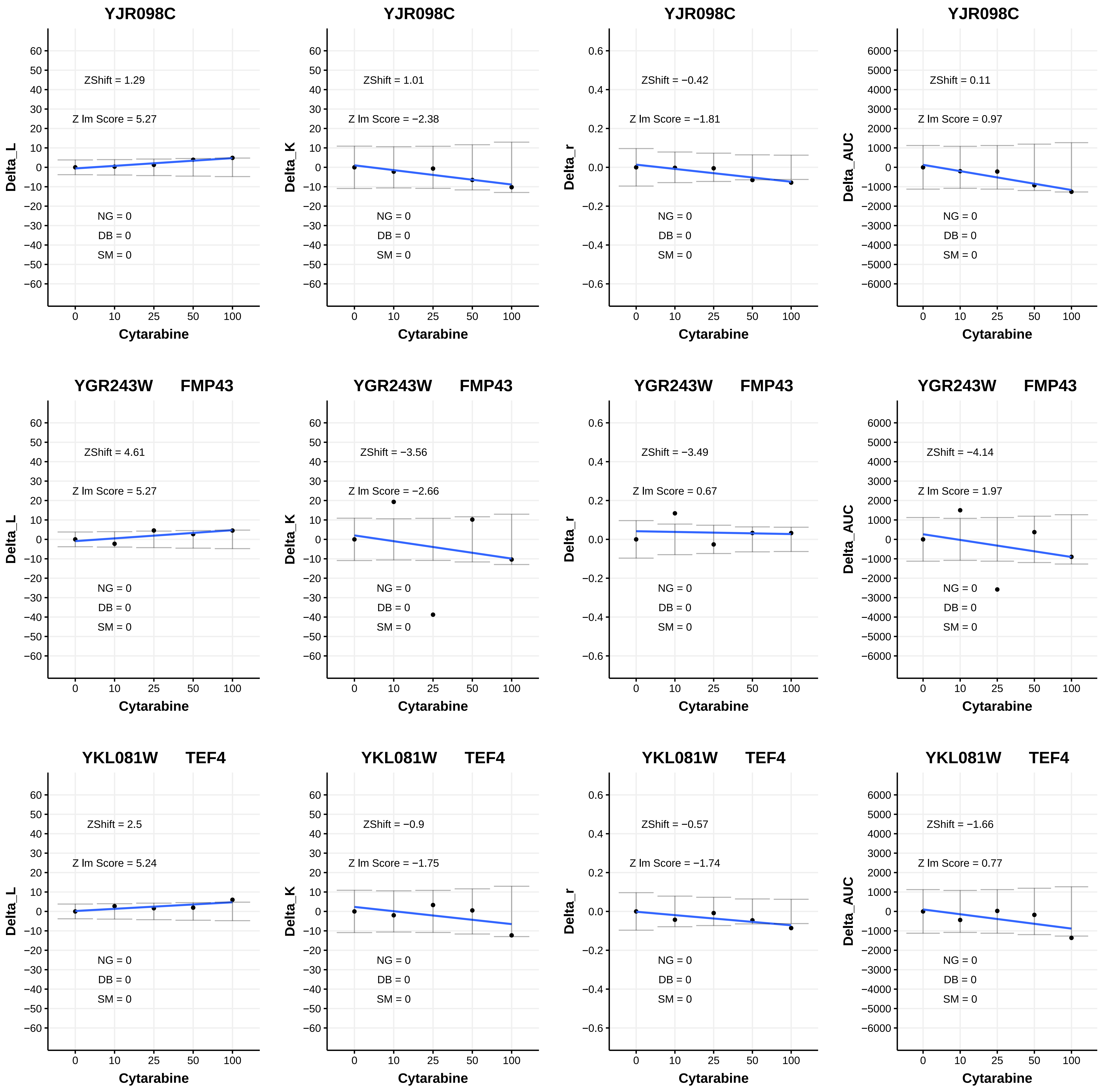
YJR098C
ZShift = 1.29
Z lm Score = 5.27
NG = 0
DB = 0
SM = 0
Delta_L
Cytarabine
YJR098C
ZShift = 1.01
Z lm Score = −2.38
NG = 0
DB = 0
SM = 0
Delta_K
Cytarabine
YJR098C
ZShift = −0.42
Z lm Score = −1.81
NG = 0
DB = 0
SM = 0
Delta_r
Cytarabine
YJR098C
ZShift = 0.11
Z lm Score = 0.97
NG = 0
DB = 0
SM = 0
Delta_AUC
Cytarabine
YGR243W FMP43
ZShift = 4.61
Z lm Score = 5.27
NG = 0
DB = 0
SM = 0
Delta_L
Cytarabine
YGR243W FMP43
ZShift = −3.56
Z lm Score = −2.66
NG = 0
DB = 0
SM = 0
Delta_K
Cytarabine
YGR243W FMP43
ZShift = −3.49
Z lm Score = 0.67
NG = 0
DB = 0
SM = 0
Delta_r
Cytarabine
YGR243W FMP43
ZShift = −4.14
Z lm Score = 1.97
NG = 0
DB = 0
SM = 0
Delta_AUC
Cytarabine
YKL081W TEF4
ZShift = 2.5
Z lm Score = 5.24
NG = 0
DB = 0
SM = 0
Delta_L
Cytarabine
YKL081W TEF4
ZShift = −0.9
Z lm Score = −1.75
NG = 0
DB = 0
SM = 0
Delta_K
Cytarabine
YKL081W TEF4
ZShift = −0.57
Z lm Score = −1.74
NG = 0
DB = 0
SM = 0
Delta_r
Cytarabine
YKL081W TEF4
ZShift = −1.66
Z lm Score = 0.77
NG = 0
DB = 0
SM = 0
Delta_AUC
Cytarabine

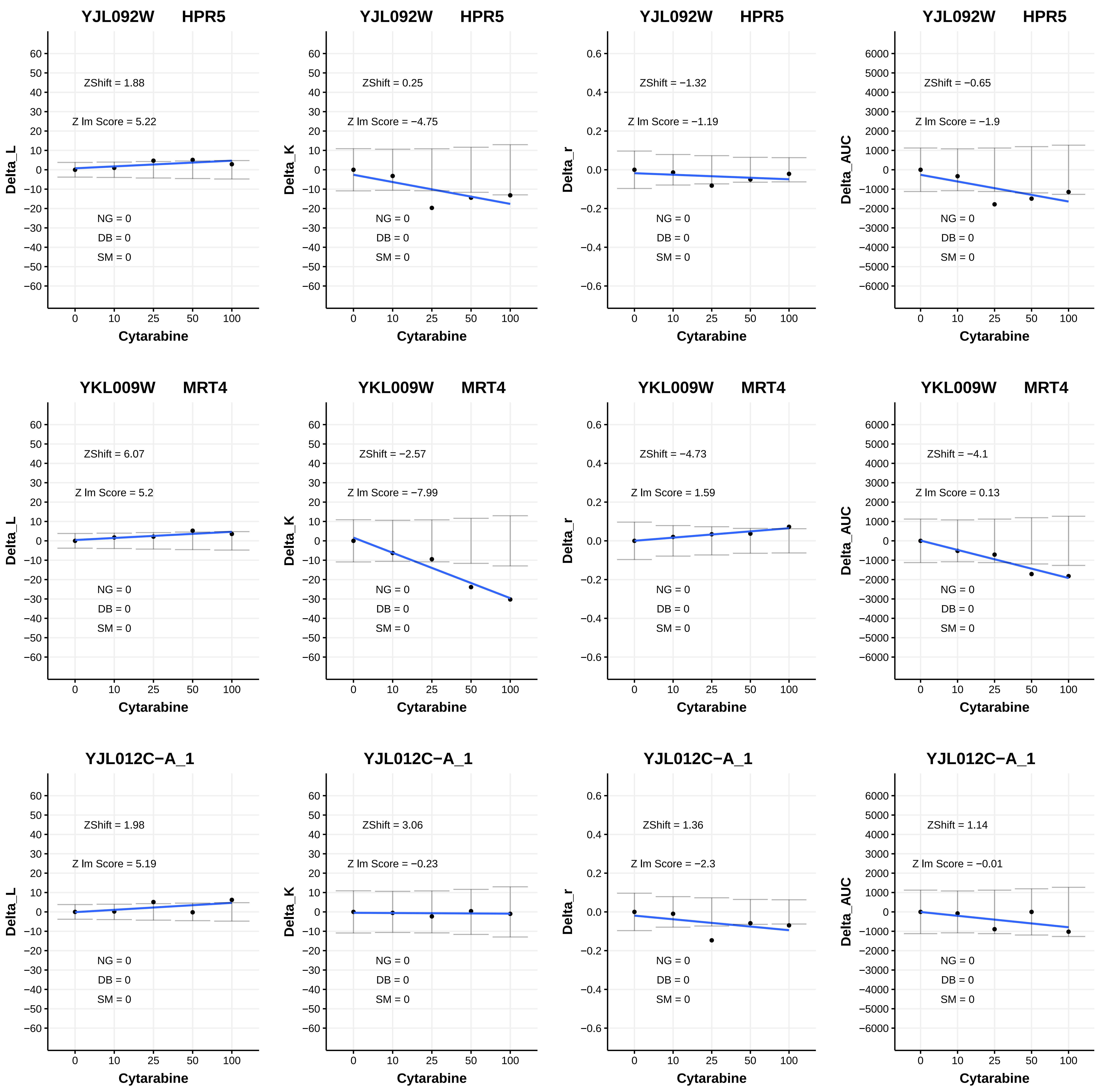
YJL092W HPR5
ZShift = 1.88
Z lm Score = 5.22
NG = 0
DB = 0
SM = 0
Delta_L
Cytarabine
YJL092W HPR5
ZShift = 0.25
Z lm Score = −4.75
NG = 0
DB = 0
SM = 0
Delta_K
Cytarabine
YJL092W HPR5
ZShift = −1.32
Z lm Score = −1.19
NG = 0
DB = 0
SM = 0
Delta_r
Cytarabine
YJL092W HPR5
ZShift = −0.65
Z lm Score = −1.9
NG = 0
DB = 0
SM = 0
Delta_AUC
Cytarabine
YKL009W MRT4
ZShift = 6.07
Z lm Score = 5.2
NG = 0
DB = 0
SM = 0
Delta_L
Cytarabine
YKL009W MRT4
ZShift = −2.57
Z lm Score = −7.99
NG = 0
DB = 0
SM = 0
Delta_K
Cytarabine
YKL009W MRT4
ZShift = −4.73
Z lm Score = 1.59
NG = 0
DB = 0
SM = 0
Delta_r
Cytarabine
YKL009W MRT4
ZShift = −4.1
Z lm Score = 0.13
NG = 0
DB = 0
SM = 0
Delta_AUC
Cytarabine
YJL012C−A_1
ZShift = 1.98
Z lm Score = 5.19
NG = 0
DB = 0
SM = 0
Delta_L
Cytarabine
YJL012C−A_1
ZShift = 3.06
Z lm Score = −0.23
NG = 0
DB = 0
SM = 0
Delta_K
Cytarabine
YJL012C−A_1
ZShift = 1.36
Z lm Score = −2.3
NG = 0
DB = 0
SM = 0
Delta_r
Cytarabine
YJL012C−A_1
ZShift = 1.14
Z lm Score = −0.01
NG = 0
DB = 0
SM = 0
Delta_AUC
Cytarabine

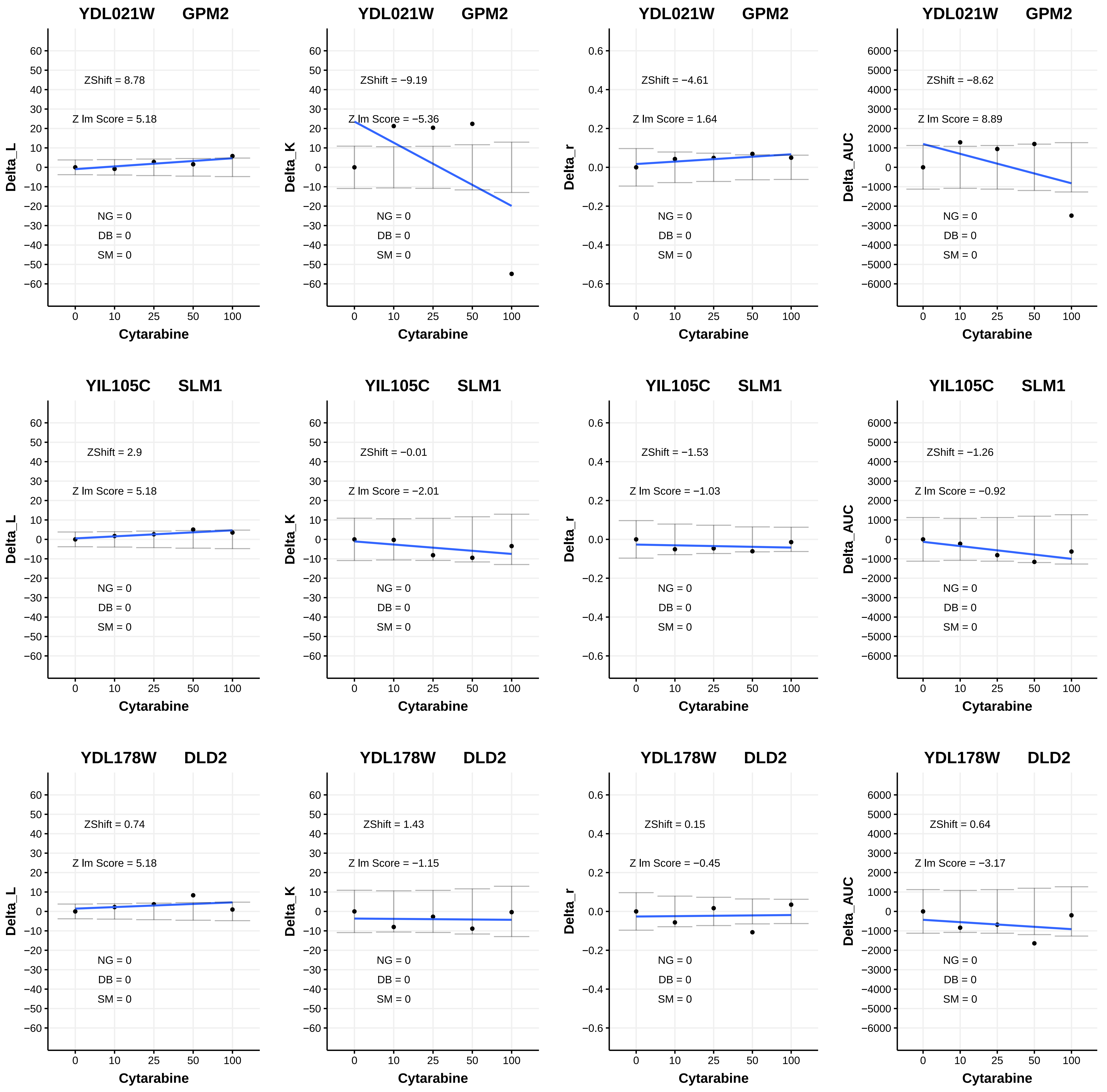

YDL021W GPM2
ZShift = 8.78
Z lm Score = 5.18
NG = 0
DB = 0
SM = 0
Delta_L
Cytarabine
YDL021W GPM2
ZShift = −9.19
Z lm Score = −5.36
NG = 0
DB = 0
SM = 0
Delta_K
Cytarabine
YDL021W GPM2
ZShift = −4.61
Z lm Score = 1.64
NG = 0
DB = 0
SM = 0
Delta_r
Cytarabine
YDL021W GPM2
ZShift = −8.62
Z lm Score = 8.89
NG = 0
DB = 0
SM = 0
Delta_AUC
Cytarabine
YIL105C SLM1
ZShift = 2.9
Z lm Score = 5.18
NG = 0
DB = 0
SM = 0
Delta_L
Cytarabine
YIL105C SLM1
ZShift = −0.01
Z lm Score = −2.01
NG = 0
DB = 0
SM = 0
Delta_K
Cytarabine
YIL105C SLM1
ZShift = −1.53
Z lm Score = −1.03
NG = 0
DB = 0
SM = 0
Delta_r
Cytarabine
YIL105C SLM1
ZShift = −1.26
Z lm Score = −0.92
NG = 0
DB = 0
SM = 0
Delta_AUC
Cytarabine
YDL178W DLD2
ZShift = 0.74
Z lm Score = 5.18
NG = 0
DB = 0
SM = 0
Delta_L
Cytarabine
YDL178W DLD2
ZShift = 1.43
Z lm Score = −1.15
NG = 0
DB = 0
SM = 0
Delta_K
Cytarabine
YDL178W DLD2
ZShift = 0.15
Z lm Score = −0.45
NG = 0
DB = 0
SM = 0
Delta_r
Cytarabine
YDL178W DLD2
ZShift = 0.64
Z lm Score = −3.17
NG = 0
DB = 0
SM = 0
Delta_AUC
Cytarabine

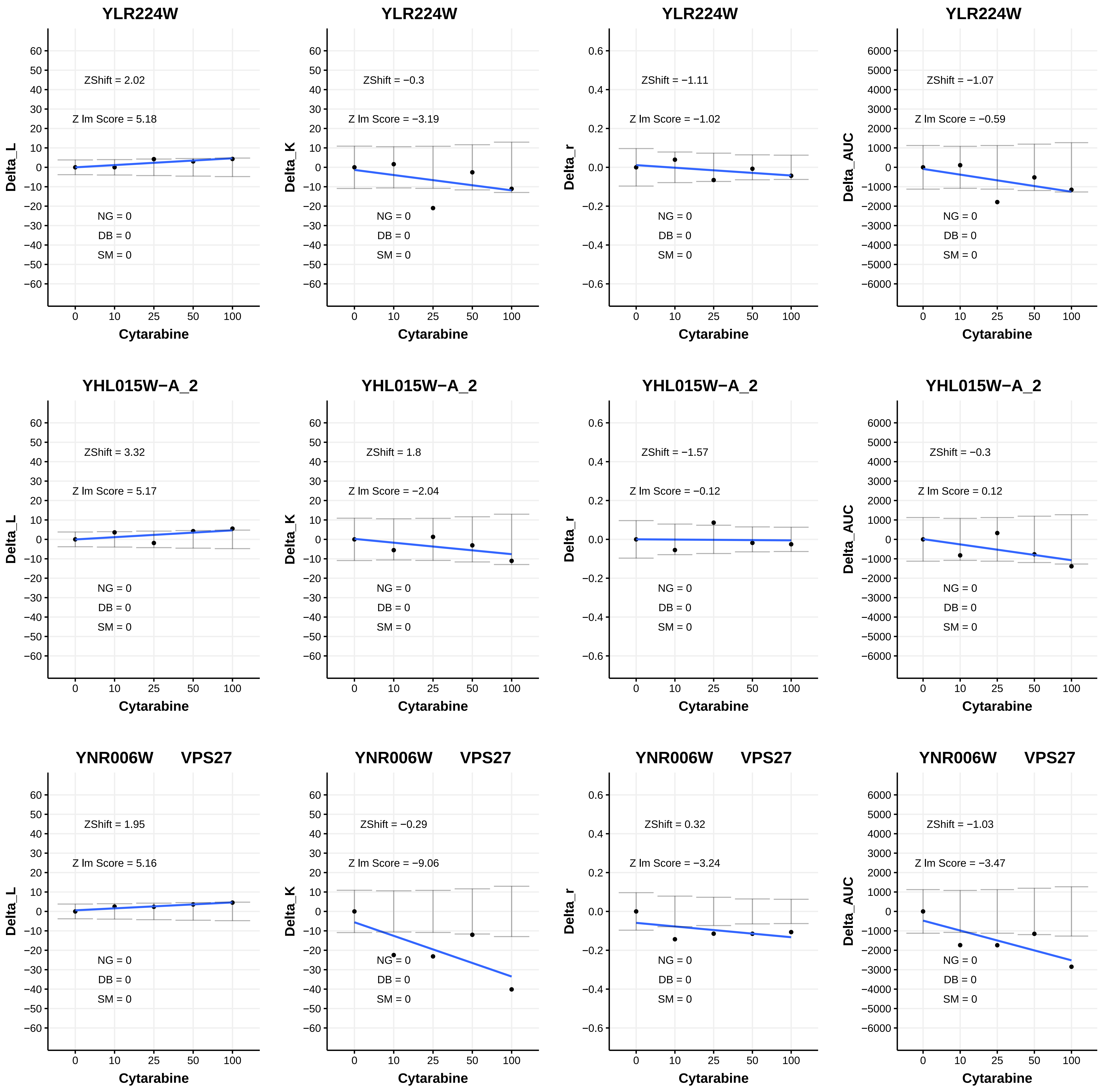

YLR224W
ZShift = 2.02
Z lm Score = 5.18
NG = 0
DB = 0
SM = 0
Delta_L
Cytarabine
YLR224W
ZShift = −0.3
Z lm Score = −3.19
NG = 0
DB = 0
SM = 0
Delta_K
Cytarabine
YLR224W
ZShift = −1.11
Z lm Score = −1.02
NG = 0
DB = 0
SM = 0
Delta_r
Cytarabine
YLR224W
ZShift = −1.07
Z lm Score = −0.59
NG = 0
DB = 0
SM = 0
Delta_AUC
Cytarabine
YHL015W−A_2
ZShift = 3.32
Z lm Score = 5.17
NG = 0
DB = 0
SM = 0
Delta_L
Cytarabine
YHL015W−A_2
ZShift = 1.8
Z lm Score = −2.04
NG = 0
DB = 0
SM = 0
Delta_K
Cytarabine
YHL015W−A_2
ZShift = −1.57
Z lm Score = −0.12
NG = 0
DB = 0
SM = 0
Delta_r
Cytarabine
YHL015W−A_2
ZShift = −0.3
Z lm Score = 0.12
NG = 0
DB = 0
SM = 0
Delta_AUC
Cytarabine
YNR006W VPS27
ZShift = 1.95
Z lm Score = 5.16
NG = 0
DB = 0
SM = 0
Delta_L
Cytarabine
YNR006W VPS27
ZShift = −0.29
Z lm Score = −9.06
NG = 0
DB = 0
SM = 0
Delta_K
Cytarabine
YNR006W VPS27
ZShift = 0.32
Z lm Score = −3.24
NG = 0
DB = 0
SM = 0
Delta_r
Cytarabine
YNR006W VPS27
ZShift = −1.03
Z lm Score = −3.47
NG = 0
DB = 0
SM = 0
Delta_AUC
Cytarabine

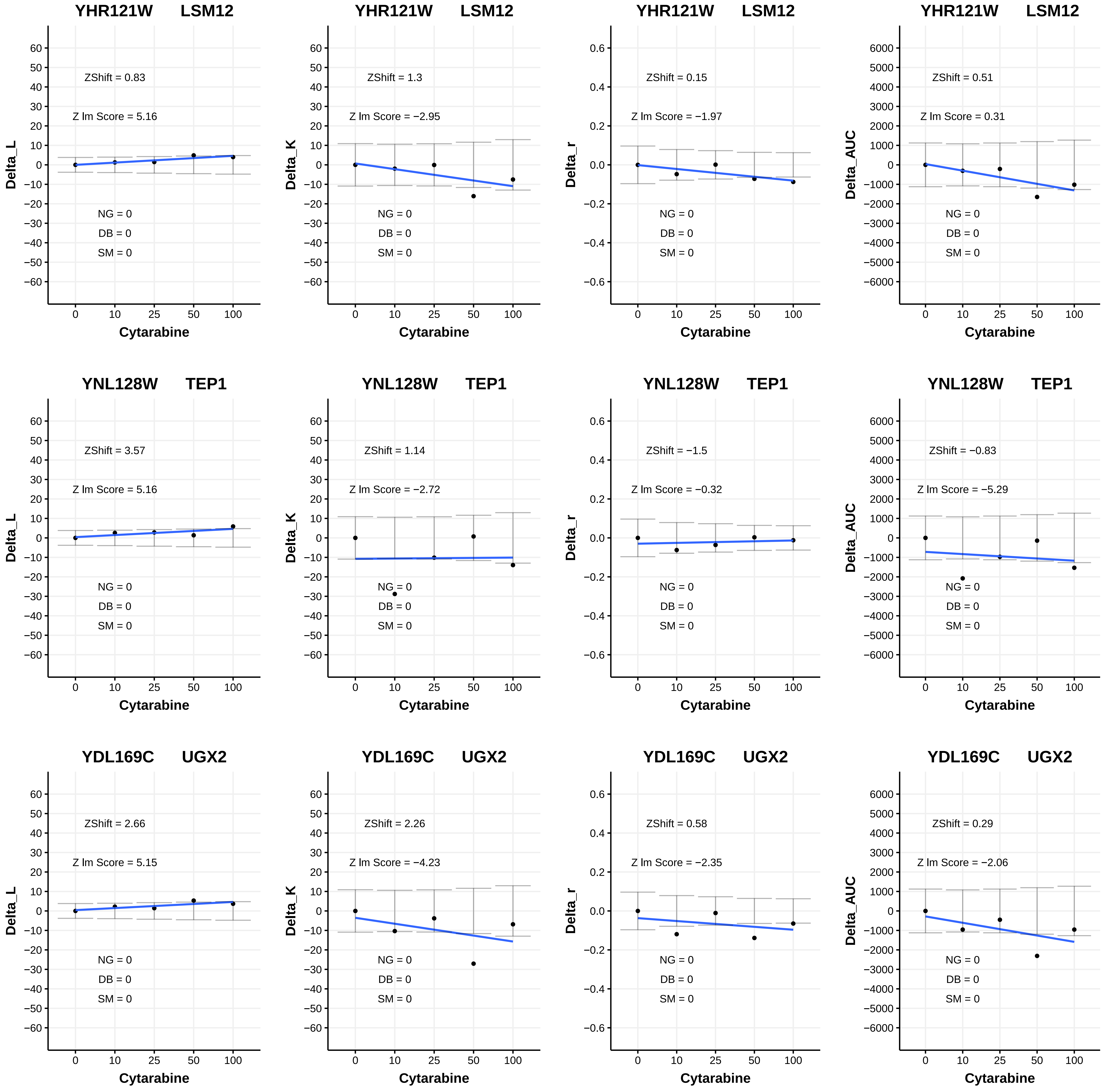

YHR121W LSM12
ZShift = 0.83
Z lm Score = 5.16
NG = 0
DB = 0
SM = 0
Delta_L
Cytarabine
YHR121W LSM12
ZShift = 1.3
Z lm Score = −2.95
NG = 0
DB = 0
SM = 0
Delta_K
Cytarabine
YHR121W LSM12
ZShift = 0.15
Z lm Score = −1.97
NG = 0
DB = 0
SM = 0
Delta_r
Cytarabine
YHR121W LSM12
ZShift = 0.51
Z lm Score = 0.31
NG = 0
DB = 0
SM = 0
Delta_AUC
Cytarabine
YNL128W TEP1
ZShift = 3.57
Z lm Score = 5.16
NG = 0
DB = 0
SM = 0
Delta_L
Cytarabine
YNL128W TEP1
ZShift = 1.14
Z lm Score = −2.72
NG = 0
DB = 0
SM = 0
Delta_K
Cytarabine
YNL128W TEP1
ZShift = −1.5
Z lm Score = −0.32
NG = 0
DB = 0
SM = 0
Delta_r
Cytarabine
YNL128W TEP1
ZShift = −0.83
Z lm Score = −5.29
NG = 0
DB = 0
SM = 0
Delta_AUC
Cytarabine
YDL169C UGX2
ZShift = 2.66
Z lm Score = 5.15
NG = 0
DB = 0
SM = 0
Delta_L
Cytarabine
YDL169C UGX2
ZShift = 2.26
Z lm Score = −4.23
NG = 0
DB = 0
SM = 0
Delta_K
Cytarabine
YDL169C UGX2
ZShift = 0.58
Z lm Score = −2.35
NG = 0
DB = 0
SM = 0
Delta_r
Cytarabine
YDL169C UGX2
ZShift = 0.29
Z lm Score = −2.06
NG = 0
DB = 0
SM = 0
Delta_AUC
Cytarabine

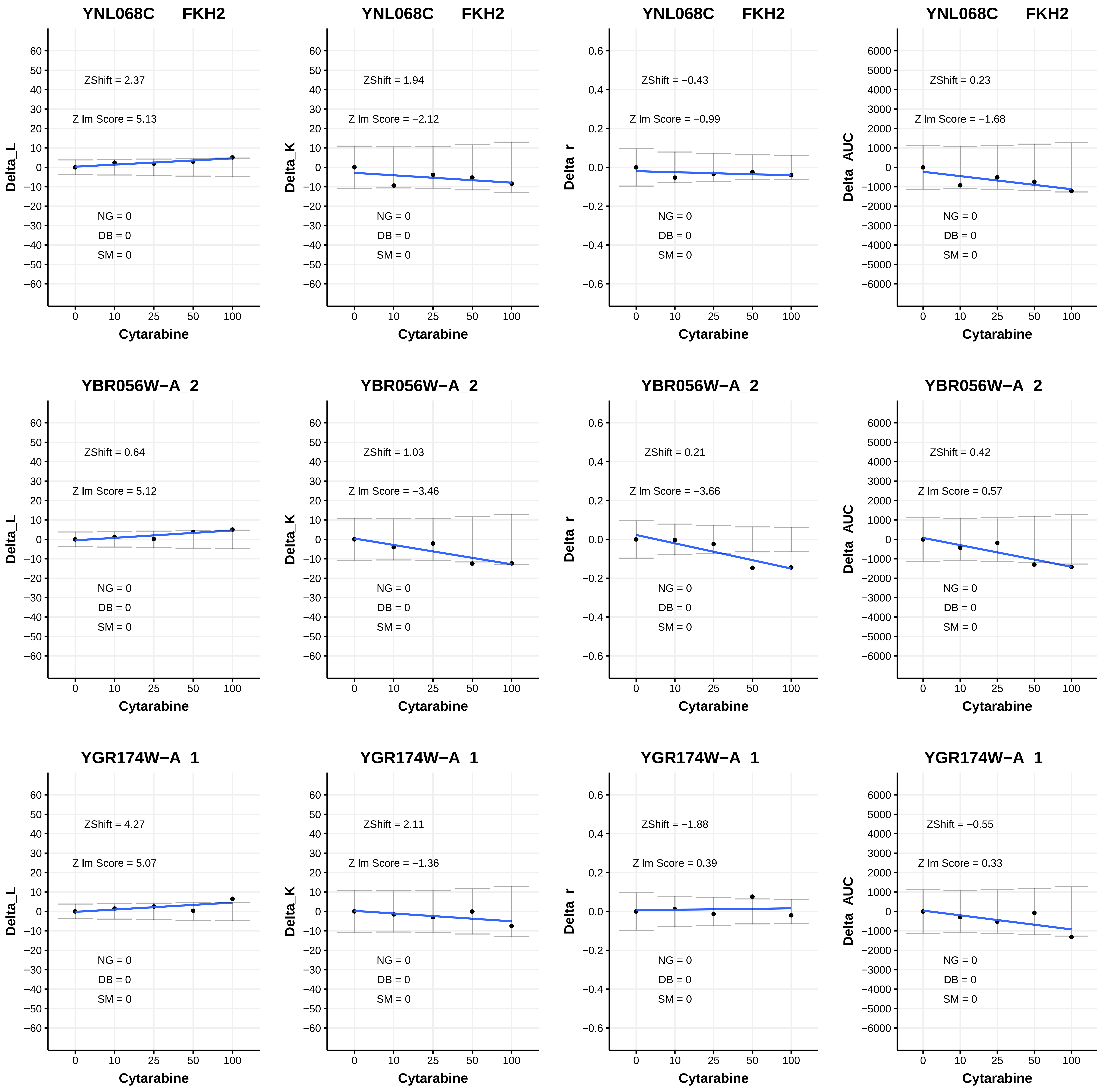

YNL068C FKH2
ZShift = 2.37
Z lm Score = 5.13
NG = 0
DB = 0
SM = 0
Delta_L
Cytarabine
YNL068C FKH2
ZShift = 1.94
Z lm Score = −2.12
NG = 0
DB = 0
SM = 0
Delta_K
Cytarabine
YNL068C FKH2
ZShift = −0.43
Z lm Score = −0.99
NG = 0
DB = 0
SM = 0
Delta_r
Cytarabine
YNL068C FKH2
ZShift = 0.23
Z lm Score = −1.68
NG = 0
DB = 0
SM = 0
Delta_AUC
Cytarabine
YBR056W−A_2
ZShift = 0.64
Z lm Score = 5.12
NG = 0
DB = 0
SM = 0
Delta_L
Cytarabine
YBR056W−A_2
ZShift = 1.03
Z lm Score = −3.46
NG = 0
DB = 0
SM = 0
Delta_K
Cytarabine
YBR056W−A_2
ZShift = 0.21
Z lm Score = −3.66
NG = 0
DB = 0
SM = 0
Delta_r
Cytarabine
YBR056W−A_2
ZShift = 0.42
Z lm Score = 0.57
NG = 0
DB = 0
SM = 0
Delta_AUC
Cytarabine
YGR174W−A_1
ZShift = 4.27
Z lm Score = 5.07
NG = 0
DB = 0
SM = 0
Delta_L
Cytarabine
YGR174W−A_1
ZShift = 2.11
Z lm Score = −1.36
NG = 0
DB = 0
SM = 0
Delta_K
Cytarabine
YGR174W−A_1
ZShift = −1.88
Z lm Score = 0.39
NG = 0
DB = 0
SM = 0
Delta_r
Cytarabine
YGR174W−A_1
ZShift = −0.55
Z lm Score = 0.33
NG = 0
DB = 0
SM = 0
Delta_AUC
Cytarabine

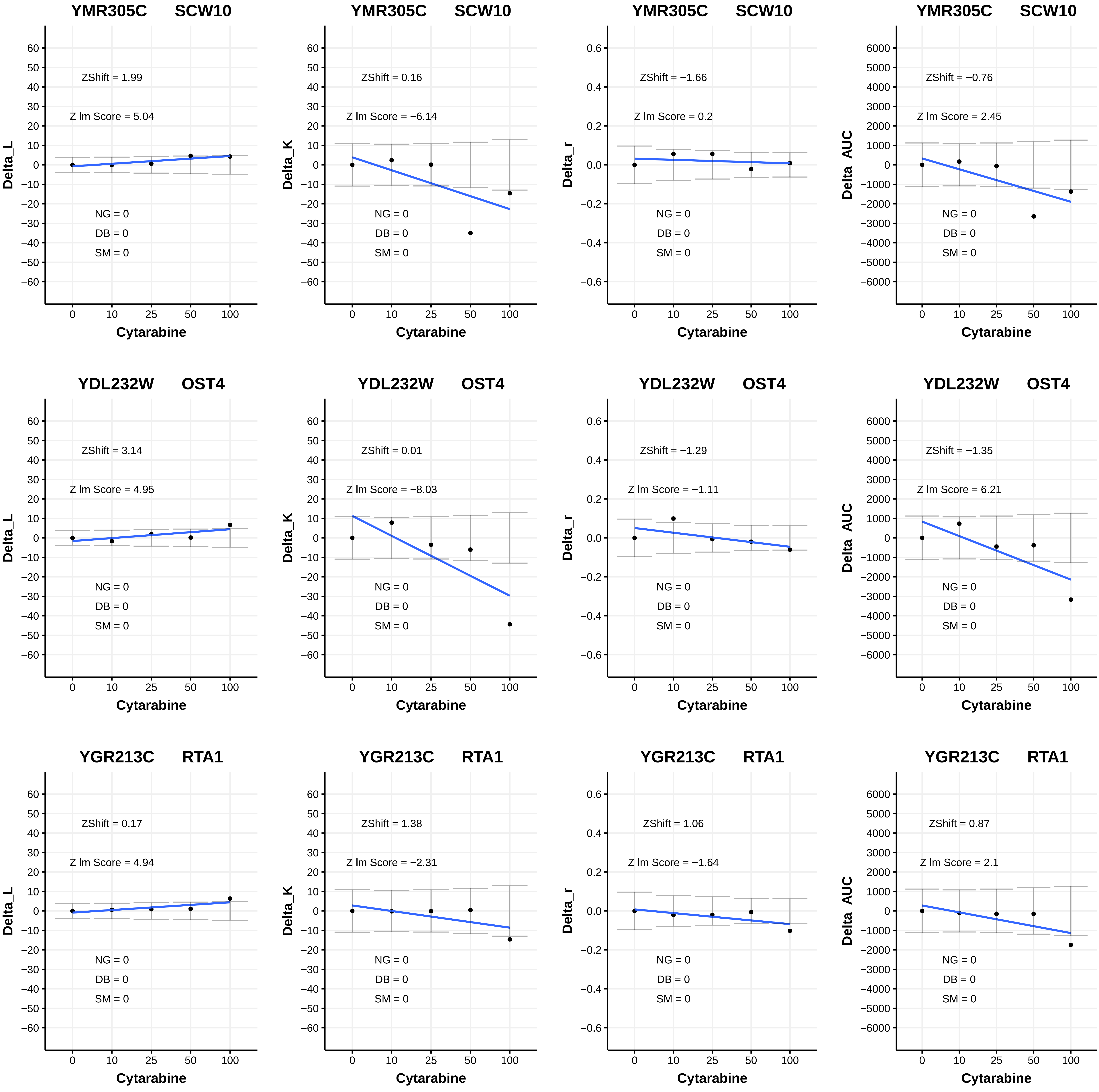

YMR305C SCW10
ZShift = 1.99
Z lm Score = 5.04
NG = 0
DB = 0
SM = 0
Delta_L
Cytarabine
YMR305C SCW10
ZShift = 0.16
Z lm Score = −6.14
NG = 0
DB = 0
SM = 0
Delta_K
Cytarabine
YMR305C SCW10
ZShift = −1.66
Z lm Score = 0.2
NG = 0
DB = 0
SM = 0
Delta_r
Cytarabine
YMR305C SCW10
ZShift = −0.76
Z lm Score = 2.45
NG = 0
DB = 0
SM = 0
Delta_AUC
Cytarabine
YDL232W OST4
ZShift = 3.14
Z lm Score = 4.95
NG = 0
DB = 0
SM = 0
Delta_L
Cytarabine
YDL232W OST4
ZShift = 0.01
Z lm Score = −8.03
NG = 0
DB = 0
SM = 0
Delta_K
Cytarabine
YDL232W OST4
ZShift = −1.29
Z lm Score = −1.11
NG = 0
DB = 0
SM = 0
Delta_r
Cytarabine
YDL232W OST4
ZShift = −1.35
Z lm Score = 6.21
NG = 0
DB = 0
SM = 0
Delta_AUC
Cytarabine
YGR213C RTA1
ZShift = 0.17
Z lm Score = 4.94
NG = 0
DB = 0
SM = 0
Delta_L
Cytarabine
YGR213C RTA1
ZShift = 1.38
Z lm Score = −2.31
NG = 0
DB = 0
SM = 0
Delta_K
Cytarabine
YGR213C RTA1
ZShift = 1.06
Z lm Score = −1.64
NG = 0
DB = 0
SM = 0
Delta_r
Cytarabine
YGR213C RTA1
ZShift = 0.87
Z lm Score = 2.1
NG = 0
DB = 0
SM = 0
Delta_AUC
Cytarabine

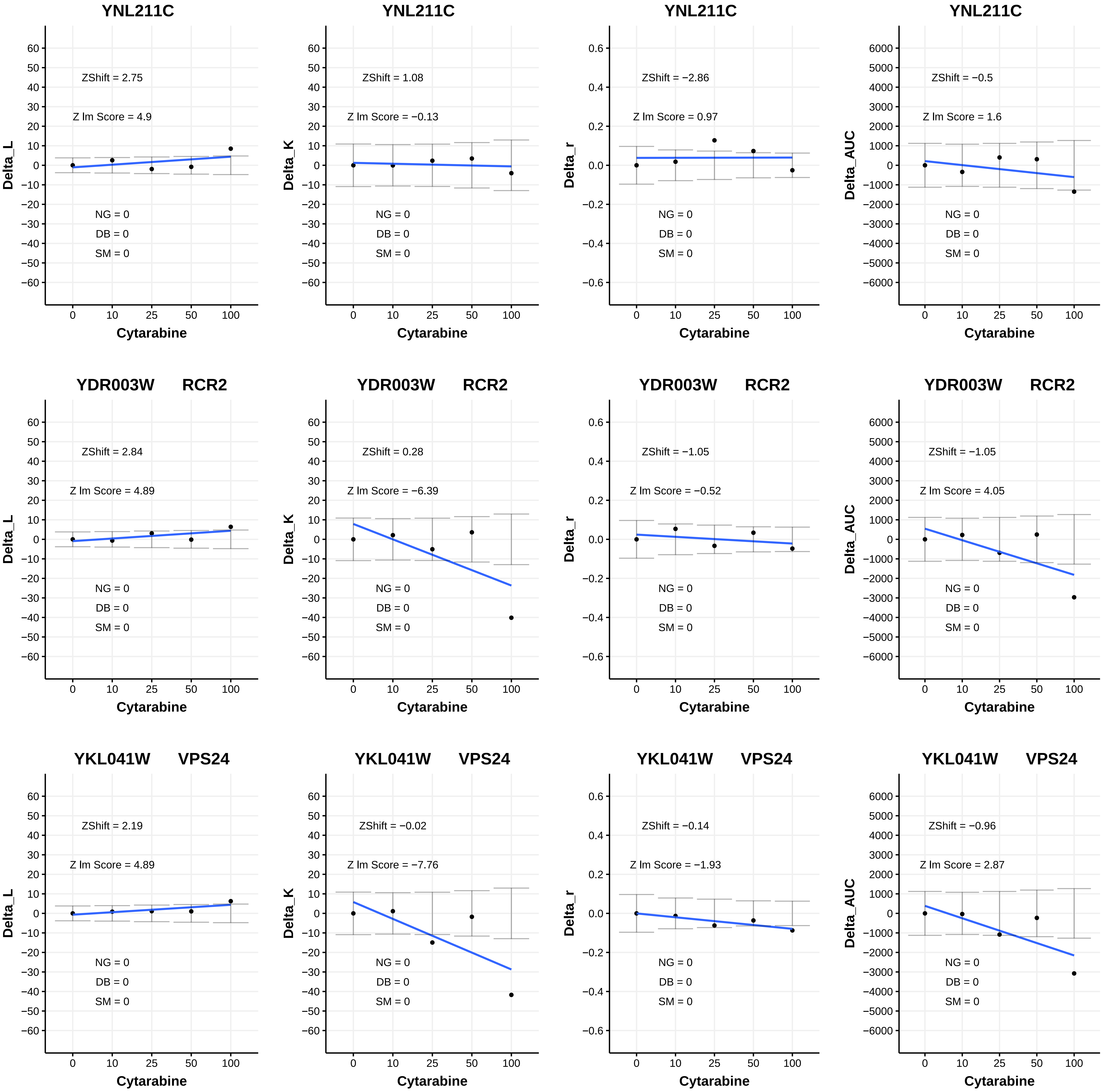

YNL211C
Delta_L
ZShift = 2.75
Z lm Score = 4.9
NG = 0
DB = 0
SM = 0
Cytarabine
YNL211C
Delta_K
ZShift = 1.08
Z lm Score = −0.13
NG = 0
DB = 0
SM = 0
Cytarabine
YNL211C
Delta_r
ZShift = −2.86
Z lm Score = 0.97
NG = 0
DB = 0
SM = 0
Cytarabine
YNL211C
Delta_AUC
ZShift = −0.5
Z lm Score = 1.6
NG = 0
DB = 0
SM = 0
Cytarabine
YDR003W RCR2
Delta_L
ZShift = 2.84
Z lm Score = 4.89
NG = 0
DB = 0
SM = 0
Cytarabine
YDR003W RCR2
Delta_K
ZShift = 0.28
Z lm Score = −6.39
NG = 0
DB = 0
SM = 0
Cytarabine
YDR003W RCR2
Delta_r
ZShift = −1.05
Z lm Score = −0.52
NG = 0
DB = 0
SM = 0
Cytarabine
YDR003W RCR2
Delta_AUC
ZShift = −1.05
Z lm Score = 4.05
NG = 0
DB = 0
SM = 0
Cytarabine
YKL041W VPS24
Delta_L
ZShift = 2.19
Z lm Score = 4.89
NG = 0
DB = 0
SM = 0
Cytarabine
YKL041W VPS24
Delta_K
ZShift = −0.02
Z lm Score = −7.76
NG = 0
DB = 0
SM = 0
Cytarabine
YKL041W VPS24
Delta_r
ZShift = −0.14
Z lm Score = −1.93
NG = 0
DB = 0
SM = 0
Cytarabine
YKL041W VPS24
Delta_AUC
ZShift = −0.96
Z lm Score = 2.87
NG = 0
DB = 0
SM = 0
Cytarabine

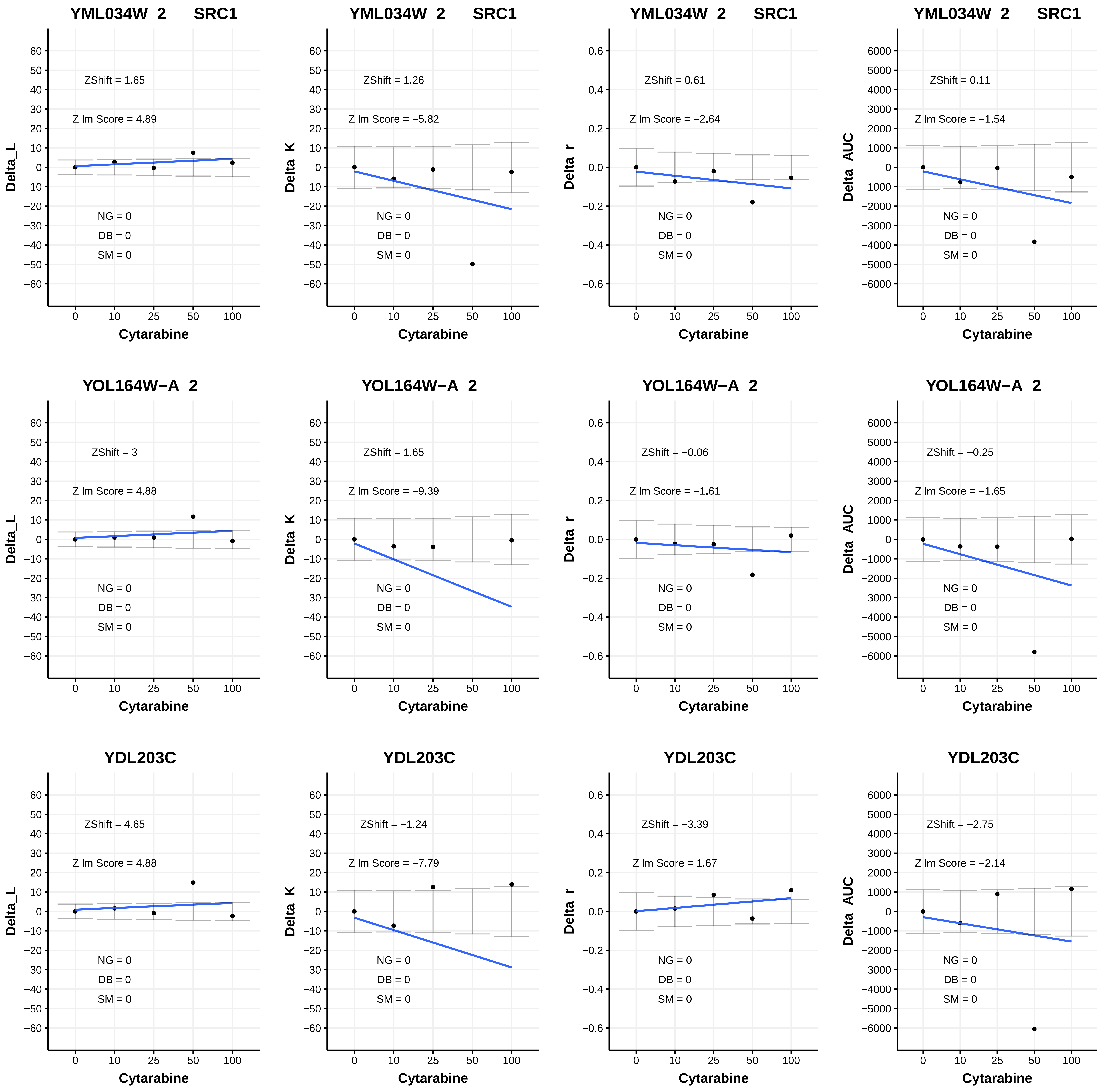

YML034W_2 SRC1
ZShift = 1.65
Z lm Score = 4.89
NG = 0
DB = 0
SM = 0
Delta_L
Cytarabine
YML034W_2 SRC1
ZShift = 1.26
Z lm Score = −5.82
NG = 0
DB = 0
SM = 0
Delta_K
Cytarabine
YML034W_2 SRC1
ZShift = 0.61
Z lm Score = −2.64
NG = 0
DB = 0
SM = 0
Delta_r
Cytarabine
YML034W_2 SRC1
ZShift = 0.11
Z lm Score = −1.54
NG = 0
DB = 0
SM = 0
Delta_AUC
Cytarabine
YOL164W−A_2
ZShift = 3
Z lm Score = 4.88
NG = 0
DB = 0
SM = 0
Delta_L
Cytarabine
YOL164W−A_2
ZShift = 1.65
Z lm Score = −9.39
NG = 0
DB = 0
SM = 0
Delta_K
Cytarabine
YOL164W−A_2
ZShift = −0.06
Z lm Score = −1.61
NG = 0
DB = 0
SM = 0
Delta_r
Cytarabine
YOL164W−A_2
ZShift = −0.25
Z lm Score = −1.65
NG = 0
DB = 0
SM = 0
Delta_AUC
Cytarabine
YDL203C
ZShift = 4.65
Z lm Score = 4.88
NG = 0
DB = 0
SM = 0
Delta_L
Cytarabine
YDL203C
ZShift = −1.24
Z lm Score = −7.79
NG = 0
DB = 0
SM = 0
Delta_K
Cytarabine
YDL203C
ZShift = −3.39
Z lm Score = 1.67
NG = 0
DB = 0
SM = 0
Delta_r
Cytarabine
YDL203C
ZShift = −2.75
Z lm Score = −2.14
NG = 0
DB = 0
SM = 0
Delta_AUC
Cytarabine

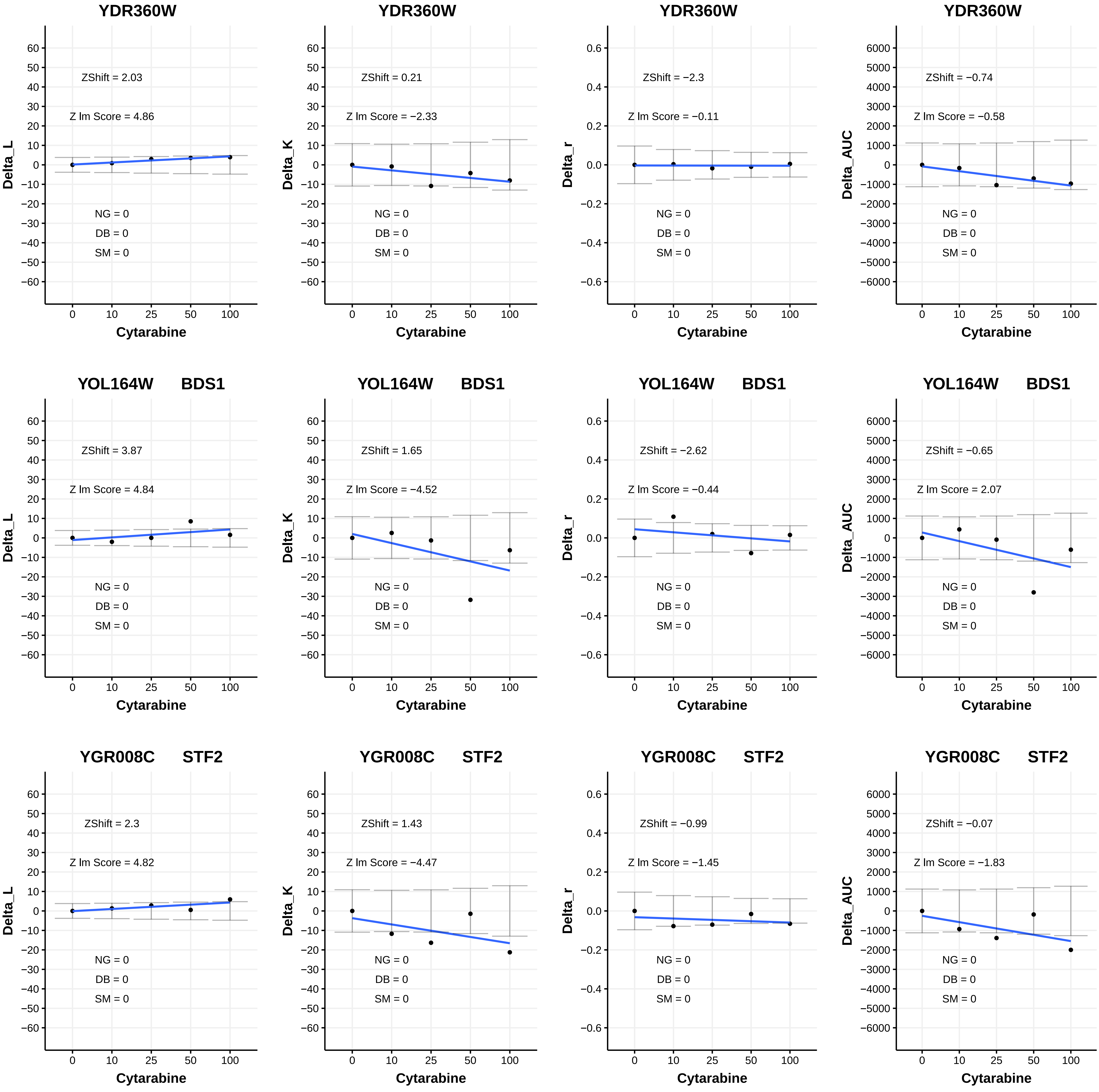
YDR360W
ZShift = 2.03
Z lm Score = 4.86
NG = 0
DB = 0
SM = 0
Delta_L
Cytarabine
YDR360W
ZShift = 0.21
Z lm Score = −2.33
NG = 0
DB = 0
SM = 0
Delta_K
Cytarabine
YDR360W
ZShift = −2.3
Z lm Score = −0.11
NG = 0
DB = 0
SM = 0
Delta_r
Cytarabine
YDR360W
ZShift = −0.74
Z lm Score = −0.58
NG = 0
DB = 0
SM = 0
Delta_AUC
Cytarabine
YOL164W BDS1
ZShift = 3.87
Z lm Score = 4.84
NG = 0
DB = 0
SM = 0
Delta_L
Cytarabine
YOL164W BDS1
ZShift = 1.65
Z lm Score = −4.52
NG = 0
DB = 0
SM = 0
Delta_K
Cytarabine
YOL164W BDS1
ZShift = −2.62
Z lm Score = −0.44
NG = 0
DB = 0
SM = 0
Delta_r
Cytarabine
YOL164W BDS1
ZShift = −0.65
Z lm Score = 2.07
NG = 0
DB = 0
SM = 0
Delta_AUC
Cytarabine
YGR008C STF2
ZShift = 2.3
Z lm Score = 4.82
NG = 0
DB = 0
SM = 0
Delta_L
Cytarabine
YGR008C STF2
ZShift = 1.43
Z lm Score = −4.47
NG = 0
DB = 0
SM = 0
Delta_K
Cytarabine
YGR008C STF2
ZShift = −0.99
Z lm Score = −1.45
NG = 0
DB = 0
SM = 0
Delta_r
Cytarabine
YGR008C STF2
ZShift = −0.07
Z lm Score = −1.83
NG = 0
DB = 0
SM = 0
Delta_AUC
Cytarabine

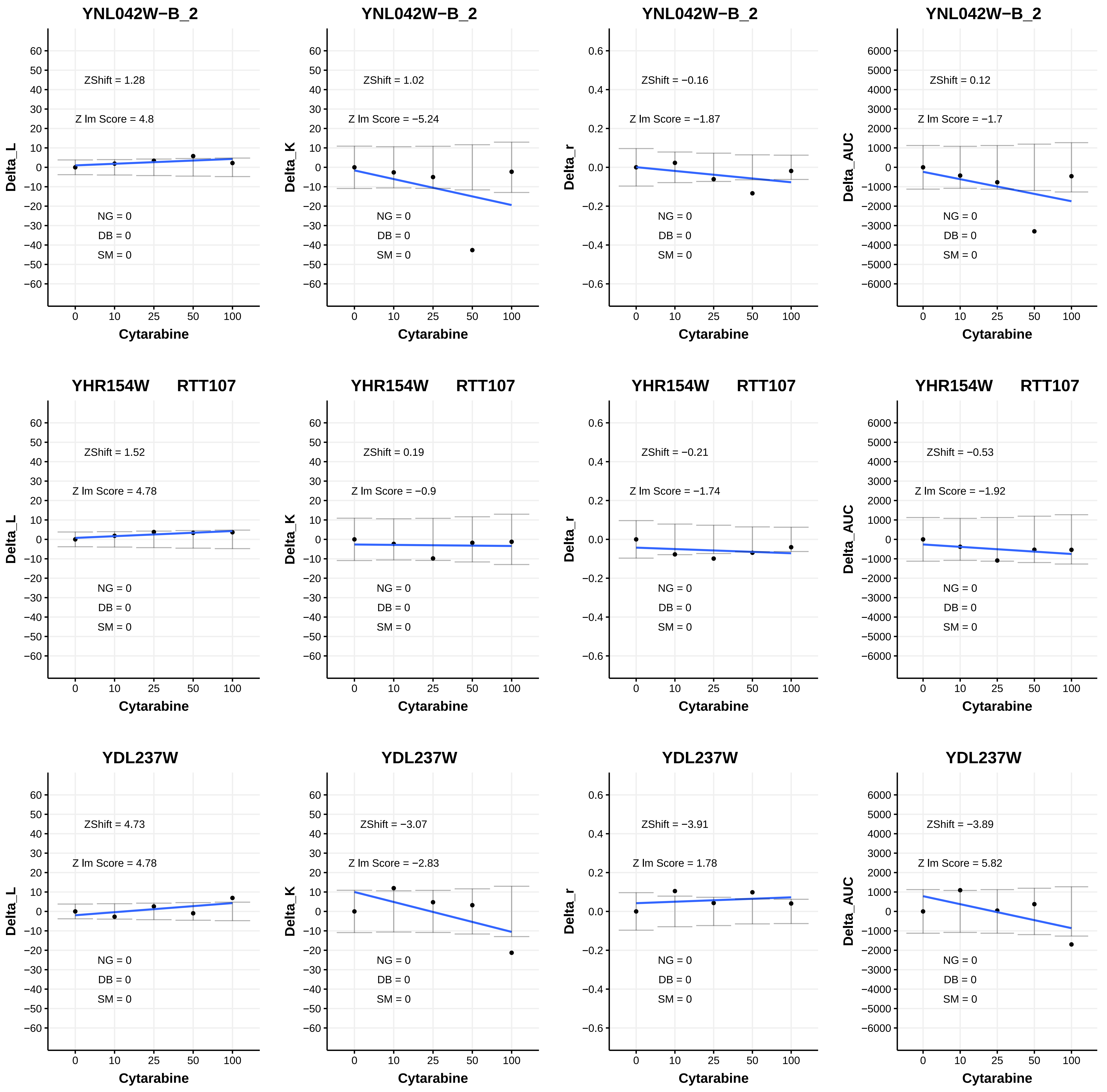

YNL042W−B_2
ZShift = 1.28
Z lm Score = 4.8
NG = 0
DB = 0
SM = 0
Delta_L
Cytarabine
YNL042W−B_2
ZShift = 1.02
Z lm Score = −5.24
NG = 0
DB = 0
SM = 0
Delta_K
Cytarabine
YNL042W−B_2
ZShift = −0.16
Z lm Score = −1.87
NG = 0
DB = 0
SM = 0
Delta_r
Cytarabine
YNL042W−B_2
ZShift = 0.12
Z lm Score = −1.7
NG = 0
DB = 0
SM = 0
Delta_AUC
Cytarabine
YHR154W RTT107
ZShift = 1.52
Z lm Score = 4.78
NG = 0
DB = 0
SM = 0
Delta_L
Cytarabine
YHR154W RTT107
ZShift = 0.19
Z lm Score = −0.9
NG = 0
DB = 0
SM = 0
Delta_K
Cytarabine
YHR154W RTT107
ZShift = −0.21
Z lm Score = −1.74
NG = 0
DB = 0
SM = 0
Delta_r
Cytarabine
YHR154W RTT107
ZShift = −0.53
Z lm Score = −1.92
NG = 0
DB = 0
SM = 0
Delta_AUC
Cytarabine
YDL237W
ZShift = 4.73
Z lm Score = 4.78
NG = 0
DB = 0
SM = 0
Delta_L
Cytarabine
YDL237W
ZShift = −3.07
Z lm Score = −2.83
NG = 0
DB = 0
SM = 0
Delta_K
Cytarabine
YDL237W
ZShift = −3.91
Z lm Score = 1.78
NG = 0
DB = 0
SM = 0
Delta_r
Cytarabine
YDL237W
ZShift = −3.89
Z lm Score = 5.82
NG = 0
DB = 0
SM = 0
Delta_AUC
Cytarabine

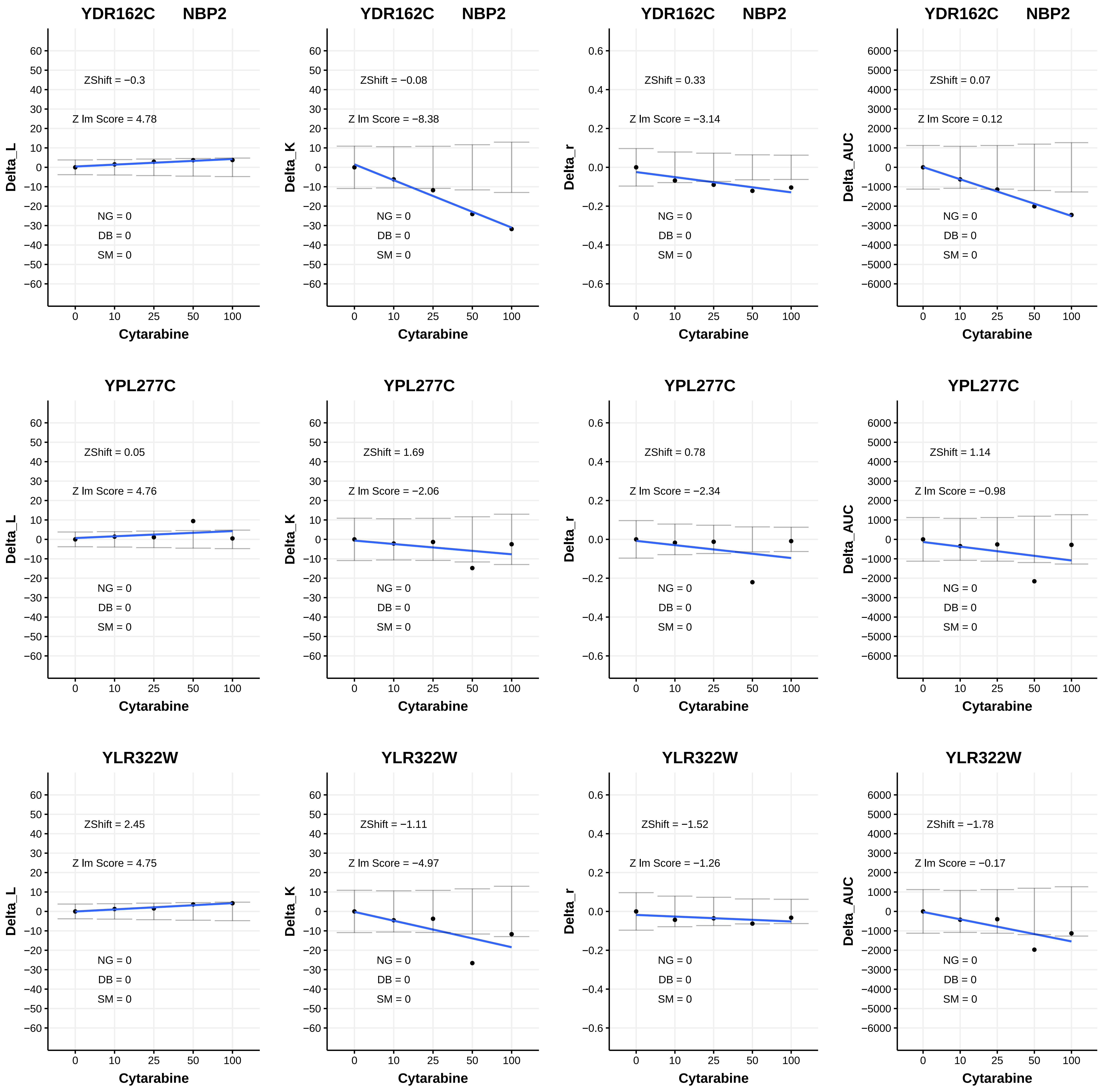
YDR162C NBP2
ZShift = −0.3
Z lm Score = 4.78
NG = 0
DB = 0
SM = 0
Delta_L
Cytarabine
YDR162C NBP2
ZShift = −0.08
Z lm Score = −8.38
NG = 0
DB = 0
SM = 0
Delta_K
Cytarabine
YDR162C NBP2
ZShift = 0.33
Z lm Score = −3.14
NG = 0
DB = 0
SM = 0
Delta_r
Cytarabine
YDR162C NBP2
ZShift = 0.07
Z lm Score = 0.12
NG = 0
DB = 0
SM = 0
Delta_AUC
Cytarabine
YPL277C
ZShift = 0.05
Z lm Score = 4.76
NG = 0
DB = 0
SM = 0
Delta_L
Cytarabine
YPL277C
ZShift = 1.69
Z lm Score = −2.06
NG = 0
DB = 0
SM = 0
Delta_K
Cytarabine
YPL277C
ZShift = 0.78
Z lm Score = −2.34
NG = 0
DB = 0
SM = 0
Delta_r
Cytarabine
YPL277C
ZShift = 1.14
Z lm Score = −0.98
NG = 0
DB = 0
SM = 0
Delta_AUC
Cytarabine
YLR322W
ZShift = 2.45
Z lm Score = 4.75
NG = 0
DB = 0
SM = 0
Delta_L
Cytarabine
YLR322W
ZShift = −1.11
Z lm Score = −4.97
NG = 0
DB = 0
SM = 0
Delta_K
Cytarabine
YLR322W
ZShift = −1.52
Z lm Score = −1.26
NG = 0
DB = 0
SM = 0
Delta_r
Cytarabine
YLR322W
ZShift = −1.78
Z lm Score = −0.17
NG = 0
DB = 0
SM = 0
Delta_AUC
Cytarabine

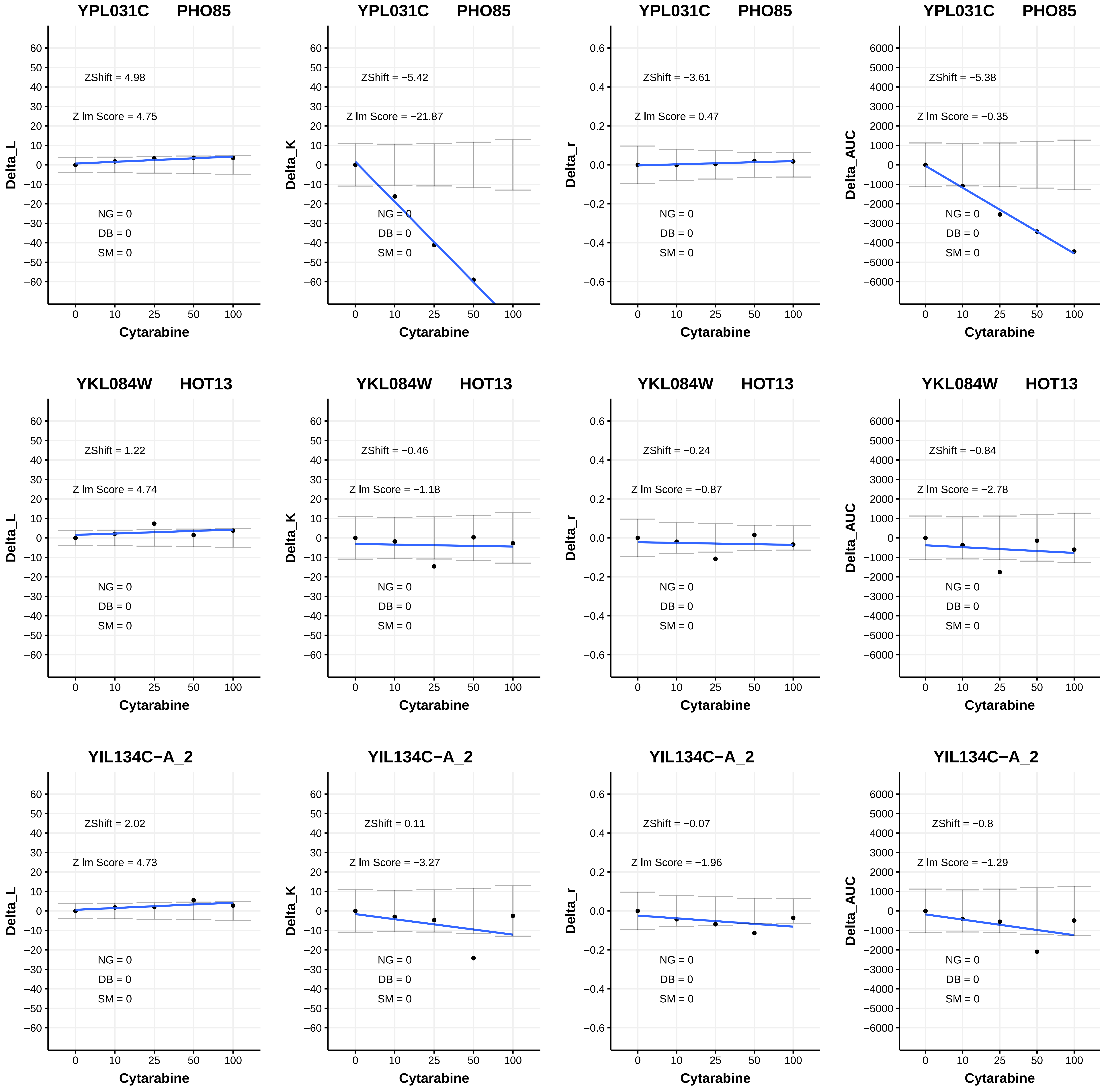

YPL031C PHO85
ZShift = 4.98
Z lm Score = 4.75
NG = 0
DB = 0
SM = 0
Delta_L
Cytarabine
YPL031C PHO85
ZShift = −5.42
Z lm Score = −21.87
NG = 0
DB = 0
SM = 0
Delta_K
Cytarabine
YPL031C PHO85
ZShift = −3.61
Z lm Score = 0.47
NG = 0
DB = 0
SM = 0
Delta_r
Cytarabine
YPL031C PHO85
ZShift = −5.38
Z lm Score = −0.35
NG = 0
DB = 0
SM = 0
Delta_AUC
Cytarabine
YKL084W HOT13
ZShift = 1.22
Z lm Score = 4.74
NG = 0
DB = 0
SM = 0
Delta_L
Cytarabine
YKL084W HOT13
ZShift = −0.46
Z lm Score = −1.18
NG = 0
DB = 0
SM = 0
Delta_K
Cytarabine
YKL084W HOT13
ZShift = −0.24
Z lm Score = −0.87
NG = 0
DB = 0
SM = 0
Delta_r
Cytarabine
YKL084W HOT13
ZShift = −0.84
Z lm Score = −2.78
NG = 0
DB = 0
SM = 0
Delta_AUC
Cytarabine
YIL134C−A_2
ZShift = 2.02
Z lm Score = 4.73
NG = 0
DB = 0
SM = 0
Delta_L
Cytarabine
YIL134C−A_2
ZShift = 0.11
Z lm Score = −3.27
NG = 0
DB = 0
SM = 0
Delta_K
Cytarabine
YIL134C−A_2
ZShift = −0.07
Z lm Score = −1.96
NG = 0
DB = 0
SM = 0
Delta_r
Cytarabine
YIL134C−A_2
ZShift = −0.8
Z lm Score = −1.29
NG = 0
DB = 0
SM = 0
Delta_AUC
Cytarabine

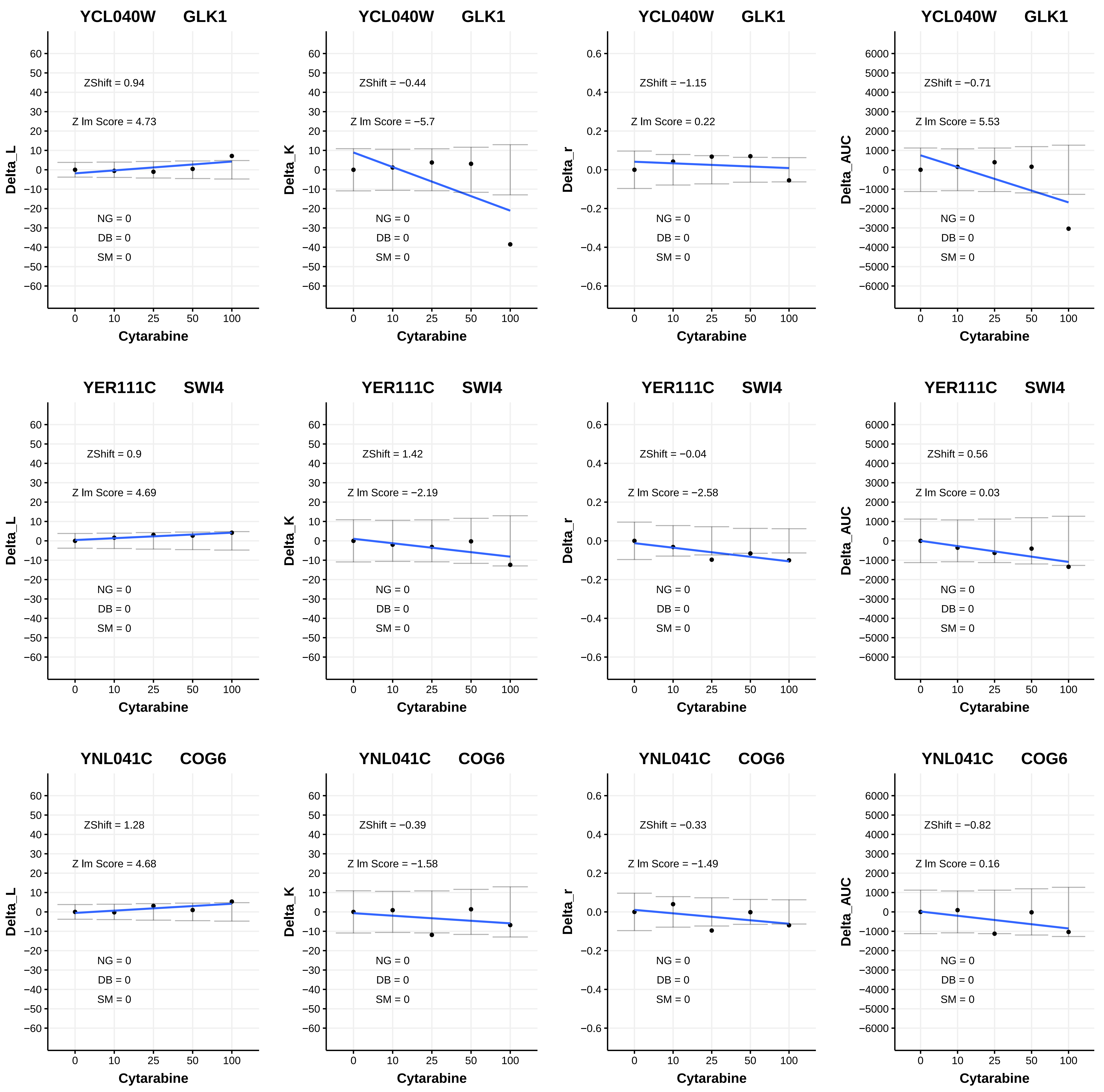

YCL040W GLK1
ZShift = 0.94
Z lm Score = 4.73
NG = 0
DB = 0
SM = 0
Delta_L
Cytarabine
YCL040W GLK1
ZShift = −0.44
Z lm Score = −5.7
NG = 0
DB = 0
SM = 0
Delta_K
Cytarabine
YCL040W GLK1
ZShift = −1.15
Z lm Score = 0.22
NG = 0
DB = 0
SM = 0
Delta_r
Cytarabine
YCL040W GLK1
ZShift = −0.71
Z lm Score = 5.53
NG = 0
DB = 0
SM = 0
Delta_AUC
Cytarabine
YER111C SWI4
ZShift = 0.9
Z lm Score = 4.69
NG = 0
DB = 0
SM = 0
Delta_L
Cytarabine
YER111C SWI4
ZShift = 1.42
Z lm Score = −2.19
NG = 0
DB = 0
SM = 0
Delta_K
Cytarabine
YER111C SWI4
ZShift = −0.04
Z lm Score = −2.58
NG = 0
DB = 0
SM = 0
Delta_r
Cytarabine
YER111C SWI4
ZShift = 0.56
Z lm Score = 0.03
NG = 0
DB = 0
SM = 0
Delta_AUC
Cytarabine
YNL041C COG6
ZShift = 1.28
Z lm Score = 4.68
NG = 0
DB = 0
SM = 0
Delta_L
Cytarabine
YNL041C COG6
ZShift = −0.39
Z lm Score = −1.58
NG = 0
DB = 0
SM = 0
Delta_K
Cytarabine
YNL041C COG6
ZShift = −0.33
Z lm Score = −1.49
NG = 0
DB = 0
SM = 0
Delta_r
Cytarabine
YNL041C COG6
ZShift = −0.82
Z lm Score = 0.16
NG = 0
DB = 0
SM = 0
Delta_AUC
Cytarabine

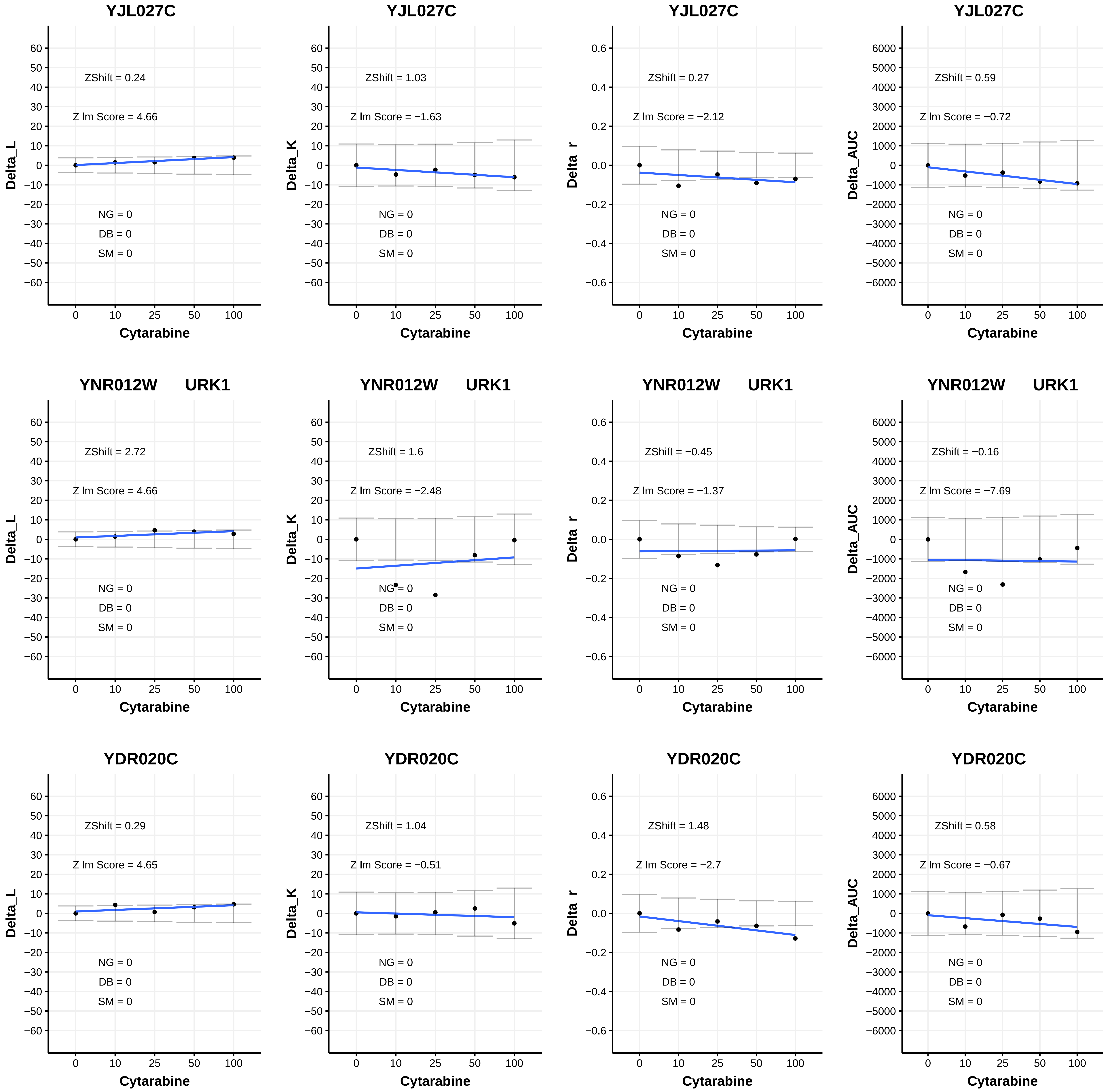
YJL027C
ZShift = 0.24
Z lm Score = 4.66
NG = 0
DB = 0
SM = 0
Delta_L
Cytarabine
YJL027C
ZShift = 1.03
Z lm Score = −1.63
NG = 0
DB = 0
SM = 0
Delta_K
Cytarabine
YJL027C
ZShift = 0.27
Z lm Score = −2.12
NG = 0
DB = 0
SM = 0
Delta_r
Cytarabine
YJL027C
ZShift = 0.59
Z lm Score = −0.72
NG = 0
DB = 0
SM = 0
Delta_AUC
Cytarabine
YNR012W URK1
ZShift = 2.72
Z lm Score = 4.66
NG = 0
DB = 0
SM = 0
Delta_L
Cytarabine
YNR012W URK1
ZShift = 1.6
Z lm Score = −2.48
NG = 0
DB = 0
SM = 0
Delta_K
Cytarabine
YNR012W URK1
ZShift = −0.45
Z lm Score = −1.37
NG = 0
DB = 0
SM = 0
Delta_r
Cytarabine
YNR012W URK1
ZShift = −0.16
Z lm Score = −7.69
NG = 0
DB = 0
SM = 0
Delta_AUC
Cytarabine
YDR020C
ZShift = 0.29
Z lm Score = 4.65
NG = 0
DB = 0
SM = 0
Delta_L
Cytarabine
YDR020C
ZShift = 1.04
Z lm Score = −0.51
NG = 0
DB = 0
SM = 0
Delta_K
Cytarabine
YDR020C
ZShift = 1.48
Z lm Score = −2.7
NG = 0
DB = 0
SM = 0
Delta_r
Cytarabine
YDR020C
ZShift = 0.58
Z lm Score = −0.67
NG = 0
DB = 0
SM = 0
Delta_AUC
Cytarabine

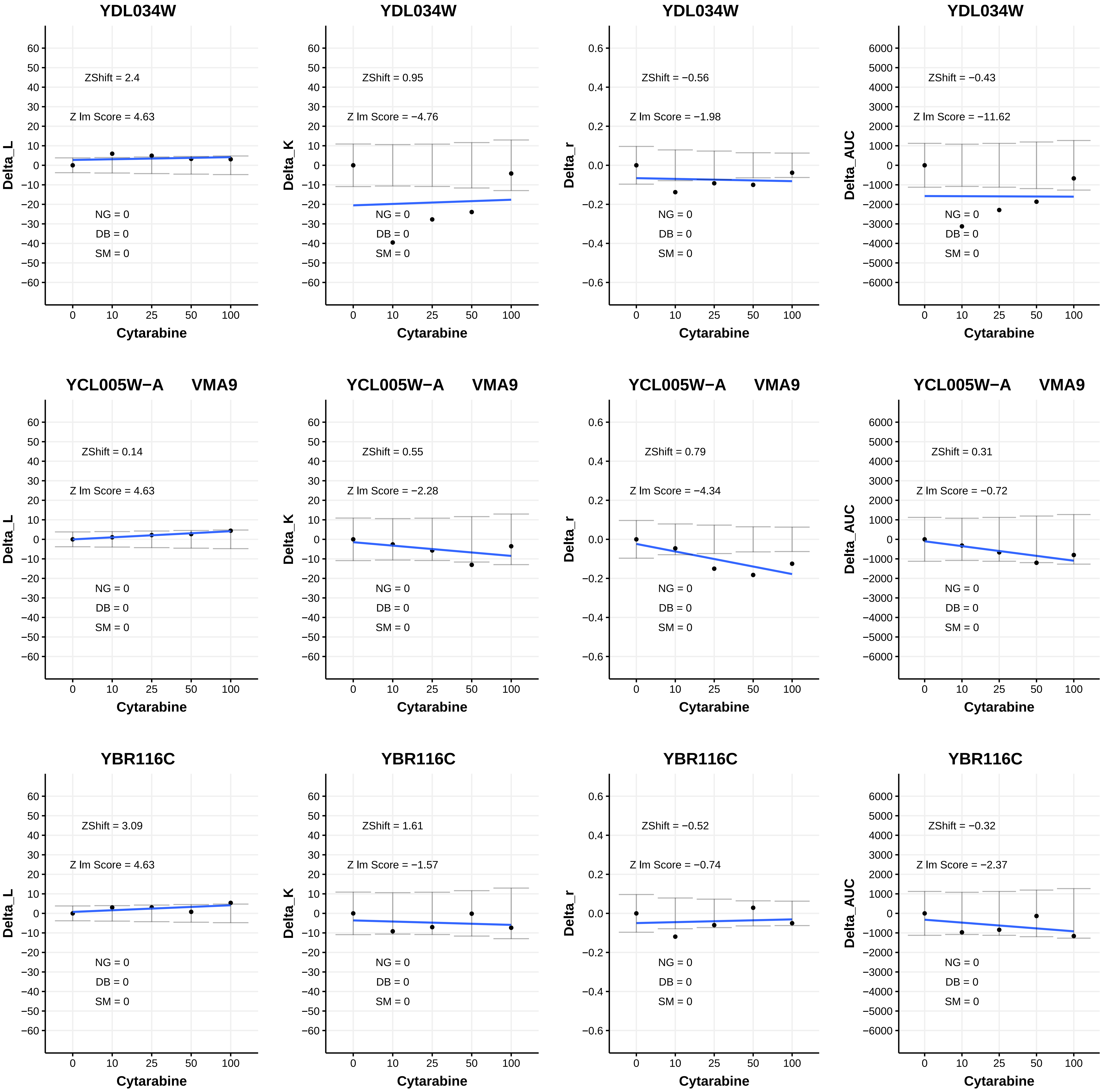
YDL034W
ZShift = 2.4
Z lm Score = 4.63
NG = 0
DB = 0
SM = 0
Delta_L
Cytarabine
YDL034W
ZShift = 0.95
Z lm Score = −4.76
NG = 0
DB = 0
SM = 0
Delta_K
Cytarabine
YDL034W
ZShift = −0.56
Z lm Score = −1.98
NG = 0
DB = 0
SM = 0
Delta_r
Cytarabine
YDL034W
ZShift = −0.43
Z lm Score = −11.62
NG = 0
DB = 0
SM = 0
Delta_AUC
Cytarabine
YCL005W−A VMA9
ZShift = 0.14
Z lm Score = 4.63
NG = 0
DB = 0
SM = 0
Delta_L
Cytarabine
YCL005W−A VMA9
ZShift = 0.55
Z lm Score = −2.28
NG = 0
DB = 0
SM = 0
Delta_K
Cytarabine
YCL005W−A VMA9
ZShift = 0.79
Z lm Score = −4.34
NG = 0
DB = 0
SM = 0
Delta_r
Cytarabine
YCL005W−A VMA9
ZShift = 0.31
Z lm Score = −0.72
NG = 0
DB = 0
SM = 0
Delta_AUC
Cytarabine
YBR116C
ZShift = 3.09
Z lm Score = 4.63
NG = 0
DB = 0
SM = 0
Delta_L
Cytarabine
YBR116C
ZShift = 1.61
Z lm Score = −1.57
NG = 0
DB = 0
SM = 0
Delta_K
Cytarabine
YBR116C
ZShift = −0.52
Z lm Score = −0.74
NG = 0
DB = 0
SM = 0
Delta_r
Cytarabine
YBR116C
ZShift = −0.32
Z lm Score = −2.37
NG = 0
DB = 0
SM = 0
Delta_AUC
Cytarabine

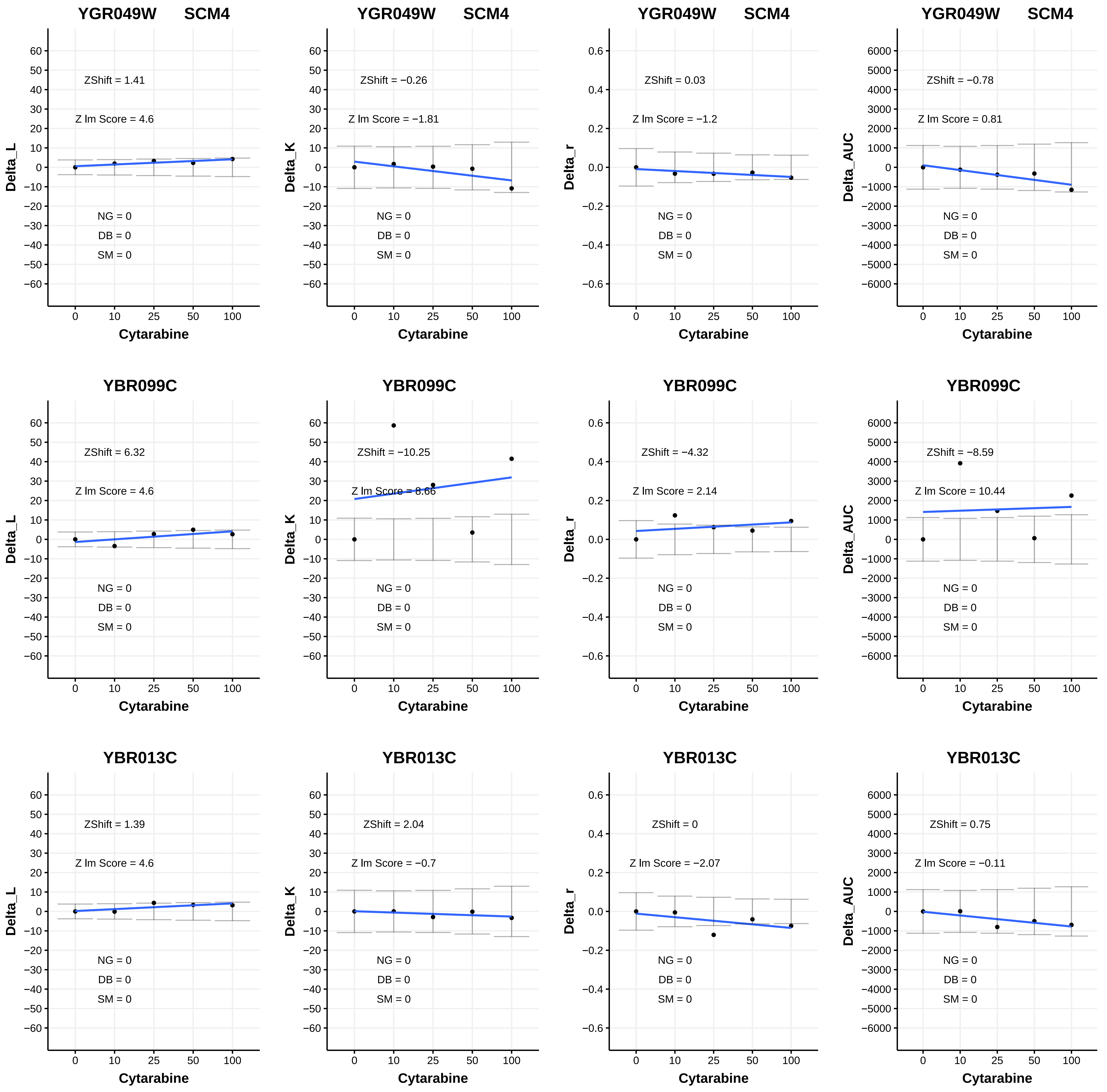

YGR049W SCM4
ZShift = 1.41
Z lm Score = 4.6
NG = 0
DB = 0
SM = 0
Delta_L
Cytarabine
YGR049W SCM4
ZShift = −0.26
Z lm Score = −1.81
NG = 0
DB = 0
SM = 0
Delta_K
Cytarabine
YGR049W SCM4
ZShift = 0.03
Z lm Score = −1.2
NG = 0
DB = 0
SM = 0
Delta_r
Cytarabine
YGR049W SCM4
ZShift = −0.78
Z lm Score = 0.81
NG = 0
DB = 0
SM = 0
Delta_AUC
Cytarabine
YBR099C
ZShift = 6.32
Z lm Score = 4.6
NG = 0
DB = 0
SM = 0
Delta_L
Cytarabine
YBR099C
ZShift = −10.25
Z lm Score = 8.66
NG = 0
DB = 0
SM = 0
Delta_K
Cytarabine
YBR099C
ZShift = −4.32
Z lm Score = 2.14
NG = 0
DB = 0
SM = 0
Delta_r
Cytarabine
YBR099C
ZShift = −8.59
Z lm Score = 10.44
NG = 0
DB = 0
SM = 0
Delta_AUC
Cytarabine
YBR013C
ZShift = 1.39
Z lm Score = 4.6
NG = 0
DB = 0
SM = 0
Delta_L
Cytarabine
YBR013C
ZShift = 2.04
Z lm Score = −0.7
NG = 0
DB = 0
SM = 0
Delta_K
Cytarabine
YBR013C
ZShift = 0
Z lm Score = −2.07
NG = 0
DB = 0
SM = 0
Delta_r
Cytarabine
YBR013C
ZShift = 0.75
Z lm Score = −0.11
NG = 0
DB = 0
SM = 0
Delta_AUC
Cytarabine

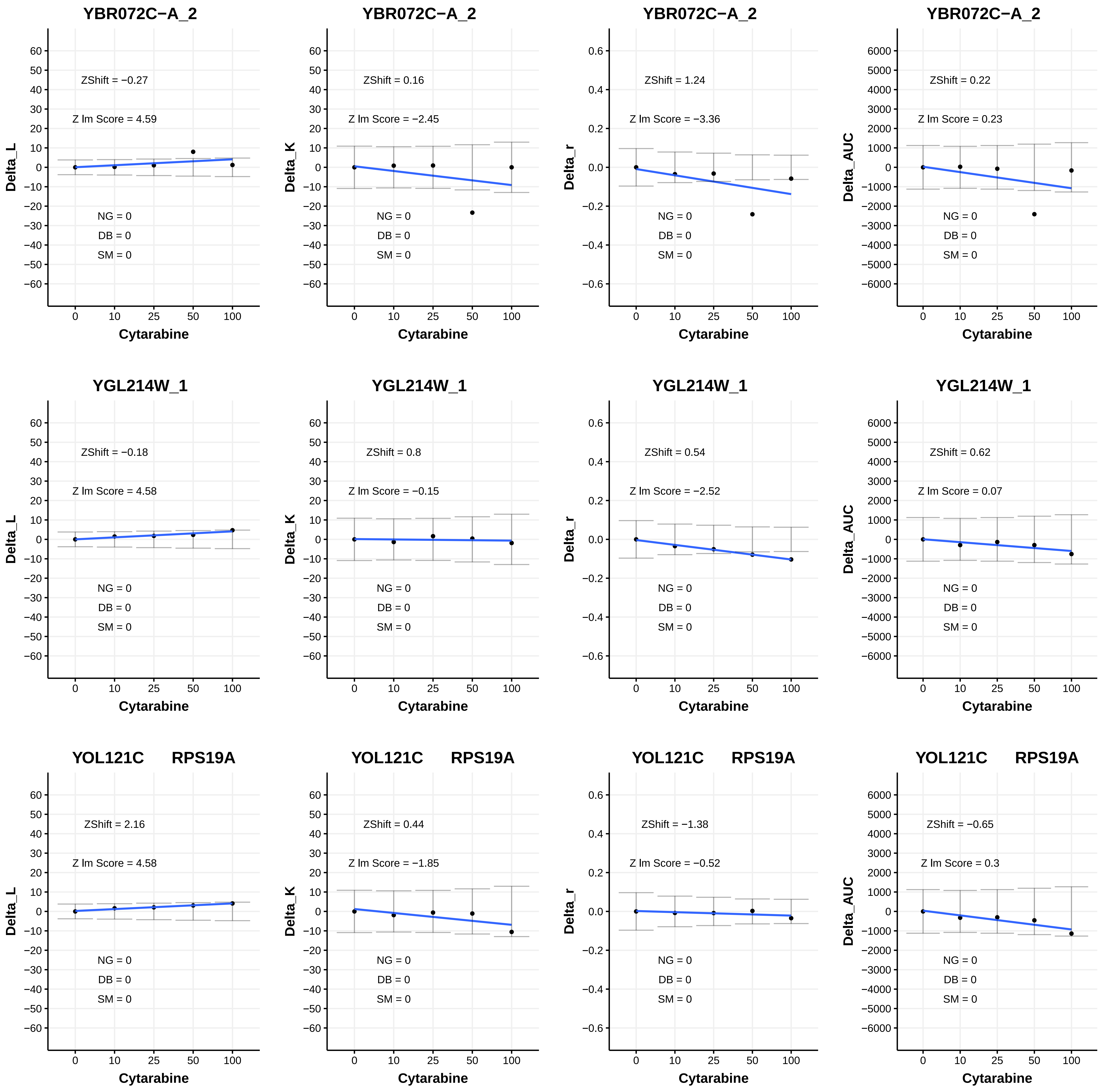
YBR072C−A_2
ZShift = −0.27
Z lm Score = 4.59
NG = 0
DB = 0
SM = 0
Delta_L
Cytarabine
YBR072C−A_2
ZShift = 0.16
Z lm Score = −2.45
NG = 0
DB = 0
SM = 0
Delta_K
Cytarabine
YBR072C−A_2
ZShift = 1.24
Z lm Score = −3.36
NG = 0
DB = 0
SM = 0
Delta_r
Cytarabine
YBR072C−A_2
ZShift = 0.22
Z lm Score = 0.23
NG = 0
DB = 0
SM = 0
Delta_AUC
Cytarabine
YGL214W_1
ZShift = −0.18
Z lm Score = 4.58
NG = 0
DB = 0
SM = 0
Delta_L
Cytarabine
YGL214W_1
ZShift = 0.8
Z lm Score = −0.15
NG = 0
DB = 0
SM = 0
Delta_K
Cytarabine
YGL214W_1
ZShift = 0.54
Z lm Score = −2.52
NG = 0
DB = 0
SM = 0
Delta_r
Cytarabine
YGL214W_1
ZShift = 0.62
Z lm Score = 0.07
NG = 0
DB = 0
SM = 0
Delta_AUC
Cytarabine
YOL121C RPS19A
ZShift = 2.16
Z lm Score = 4.58
NG = 0
DB = 0
SM = 0
Delta_L
Cytarabine
YOL121C RPS19A
ZShift = 0.44
Z lm Score = −1.85
NG = 0
DB = 0
SM = 0
Delta_K
Cytarabine
YOL121C RPS19A
ZShift = −1.38
Z lm Score = −0.52
NG = 0
DB = 0
SM = 0
Delta_r
Cytarabine
YOL121C RPS19A
ZShift = −0.65
Z lm Score = 0.3
NG = 0
DB = 0
SM = 0
Delta_AUC
Cytarabine

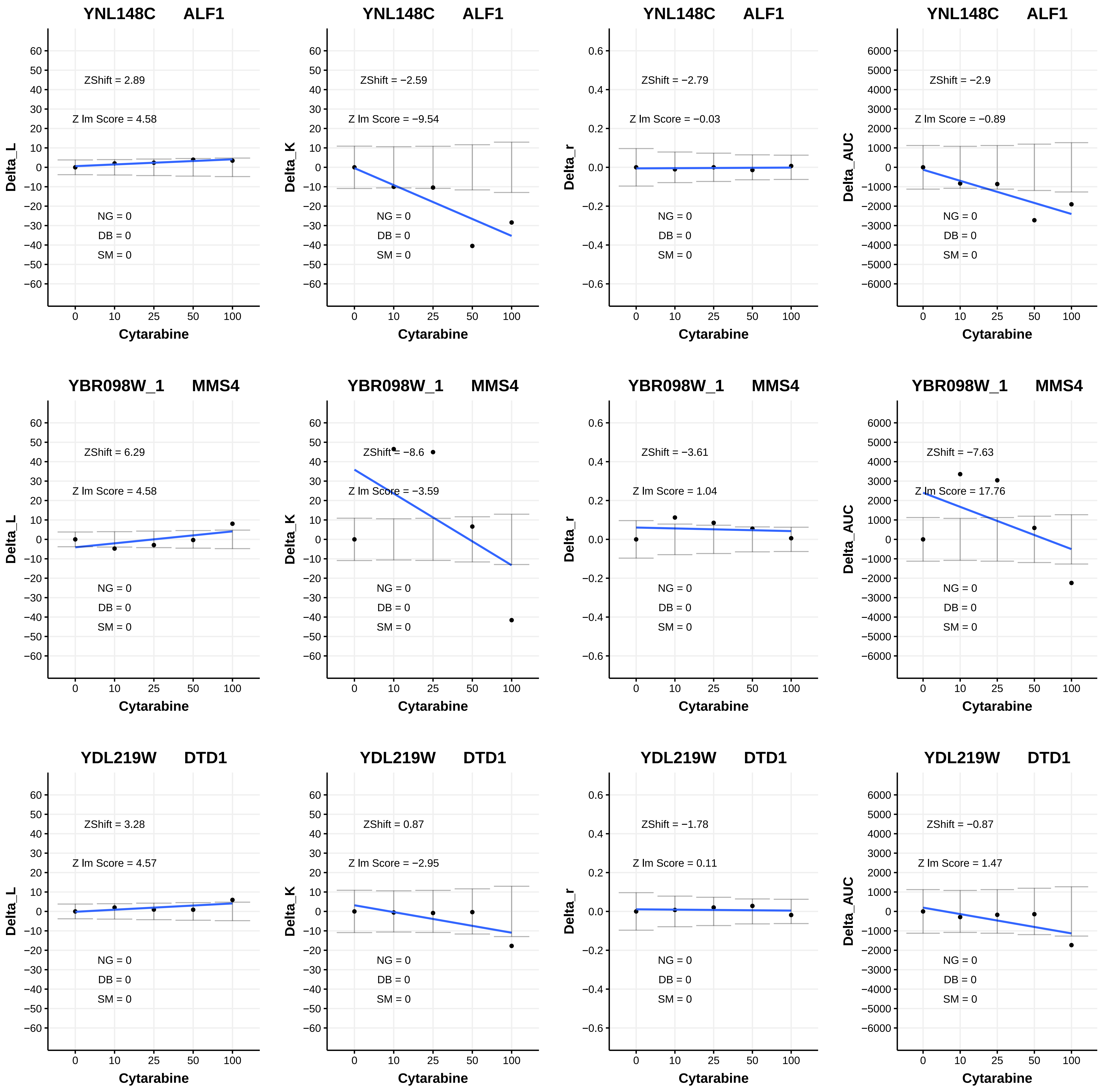
YNL148C ALF1
ZShift = 2.89
Z lm Score = 4.58
NG = 0
DB = 0
SM = 0
Delta_L
Cytarabine
YNL148C ALF1
ZShift = −2.59
Z lm Score = −9.54
NG = 0
DB = 0
SM = 0
Delta_K
Cytarabine
YNL148C ALF1
ZShift = −2.79
Z lm Score = −0.03
NG = 0
DB = 0
SM = 0
Delta_r
Cytarabine
YNL148C ALF1
ZShift = −2.9
Z lm Score = −0.89
NG = 0
DB = 0
SM = 0
Delta_AUC
Cytarabine
YBR098W_1 MMS4
ZShift = 6.29
Z lm Score = 4.58
NG = 0
DB = 0
SM = 0
Delta_L
Cytarabine
YBR098W_1 MMS4
ZShift = −8.6
Z lm Score = −3.59
NG = 0
DB = 0
SM = 0
Delta_K
Cytarabine
YBR098W_1 MMS4
ZShift = −3.61
Z lm Score = 1.04
NG = 0
DB = 0
SM = 0
Delta_r
Cytarabine
YBR098W_1 MMS4
ZShift = −7.63
Z lm Score = 17.76
NG = 0
DB = 0
SM = 0
Delta_AUC
Cytarabine
YDL219W DTD1
ZShift = 3.28
Z lm Score = 4.57
NG = 0
DB = 0
SM = 0
Delta_L
Cytarabine
YDL219W DTD1
ZShift = 0.87
Z lm Score = −2.95
NG = 0
DB = 0
SM = 0
Delta_K
Cytarabine
YDL219W DTD1
ZShift = −1.78
Z lm Score = 0.11
NG = 0
DB = 0
SM = 0
Delta_r
Cytarabine
YDL219W DTD1
ZShift = −0.87
Z lm Score = 1.47
NG = 0
DB = 0
SM = 0
Delta_AUC
Cytarabine

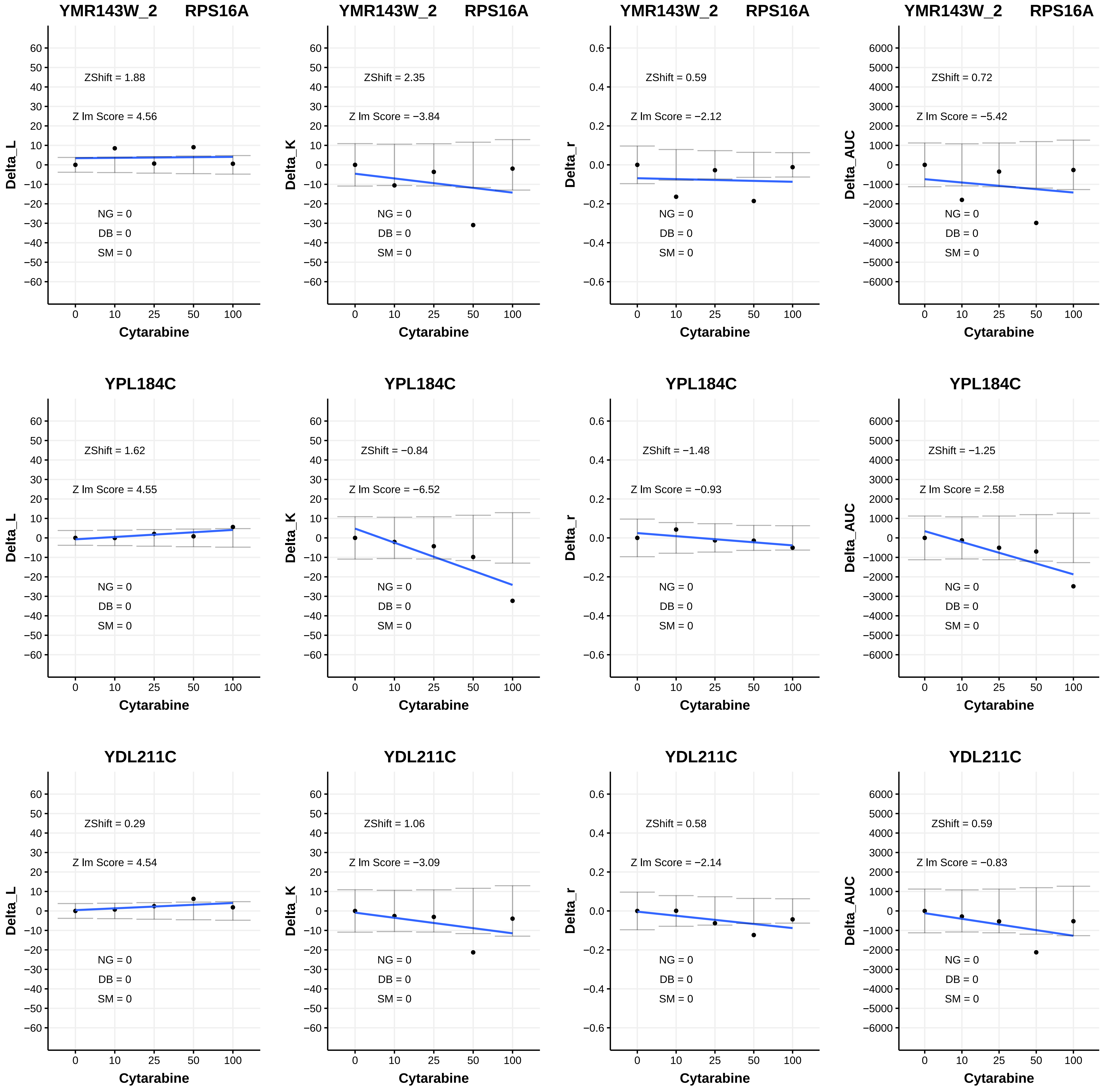
YMR143W_2 RPS16A
ZShift = 1.88
Z lm Score = 4.56
NG = 0
DB = 0
SM = 0
Delta_L
Cytarabine
YMR143W_2 RPS16A
ZShift = 2.35
Z lm Score = −3.84
NG = 0
DB = 0
SM = 0
Delta_K
Cytarabine
YMR143W_2 RPS16A
ZShift = 0.59
Z lm Score = −2.12
NG = 0
DB = 0
SM = 0
Delta_r
Cytarabine
YMR143W_2 RPS16A
ZShift = 0.72
Z lm Score = −5.42
NG = 0
DB = 0
SM = 0
Delta_AUC
Cytarabine
YPL184C
ZShift = 1.62
Z lm Score = 4.55
NG = 0
DB = 0
SM = 0
Delta_L
Cytarabine
YPL184C
ZShift = −0.84
Z lm Score = −6.52
NG = 0
DB = 0
SM = 0
Delta_K
Cytarabine
YPL184C
ZShift = −1.48
Z lm Score = −0.93
NG = 0
DB = 0
SM = 0
Delta_r
Cytarabine
YPL184C
ZShift = −1.25
Z lm Score = 2.58
NG = 0
DB = 0
SM = 0
Delta_AUC
Cytarabine
YDL211C
ZShift = 0.29
Z lm Score = 4.54
NG = 0
DB = 0
SM = 0
Delta_L
Cytarabine
YDL211C
ZShift = 1.06
Z lm Score = −3.09
NG = 0
DB = 0
SM = 0
Delta_K
Cytarabine
YDL211C
ZShift = 0.58
Z lm Score = −2.14
NG = 0
DB = 0
SM = 0
Delta_r
Cytarabine
YDL211C
ZShift = 0.59
Z lm Score = −0.83
NG = 0
DB = 0
SM = 0
Delta_AUC
Cytarabine

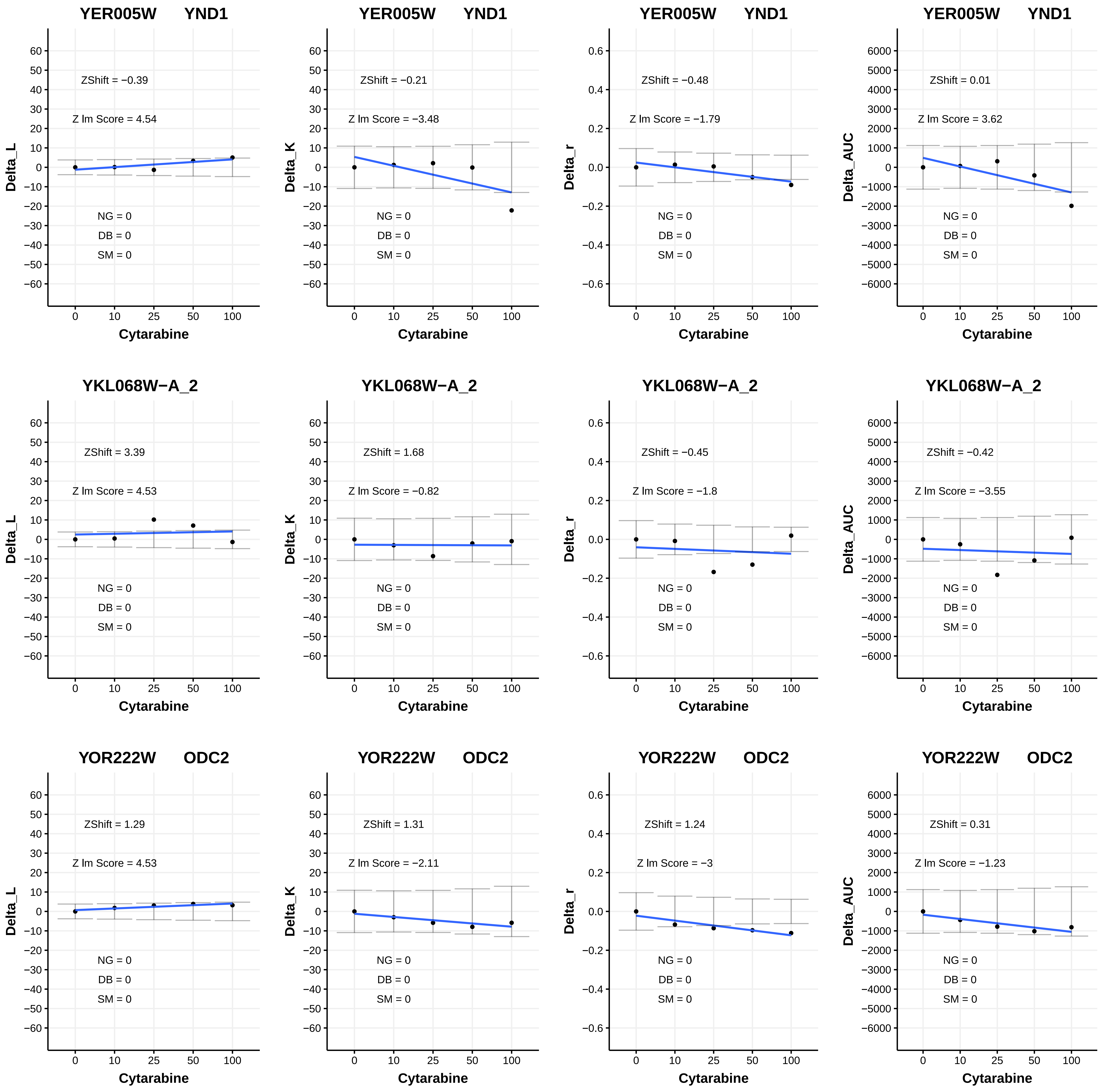

YER005W YND1
ZShift = −0.39
Z lm Score = 4.54
NG = 0
DB = 0
SM = 0
Delta_L
Cytarabine
YER005W YND1
ZShift = −0.21
Z lm Score = −3.48
NG = 0
DB = 0
SM = 0
Delta_K
Cytarabine
YER005W YND1
ZShift = −0.48
Z lm Score = −1.79
NG = 0
DB = 0
SM = 0
Delta_r
Cytarabine
YER005W YND1
ZShift = 0.01
Z lm Score = 3.62
NG = 0
DB = 0
SM = 0
Delta_AUC
Cytarabine
YKL068W−A_2
ZShift = 3.39
Z lm Score = 4.53
NG = 0
DB = 0
SM = 0
Delta_L
Cytarabine
YKL068W−A_2
ZShift = 1.68
Z lm Score = −0.82
NG = 0
DB = 0
SM = 0
Delta_K
Cytarabine
YKL068W−A_2
ZShift = −0.45
Z lm Score = −1.8
NG = 0
DB = 0
SM = 0
Delta_r
Cytarabine
YKL068W−A_2
ZShift = −0.42
Z lm Score = −3.55
NG = 0
DB = 0
SM = 0
Delta_AUC
Cytarabine
YOR222W ODC2
ZShift = 1.29
Z lm Score = 4.53
NG = 0
DB = 0
SM = 0
Delta_L
Cytarabine
YOR222W ODC2
ZShift = 1.31
Z lm Score = −2.11
NG = 0
DB = 0
SM = 0
Delta_K
Cytarabine
YOR222W ODC2
ZShift = 1.24
Z lm Score = −3
NG = 0
DB = 0
SM = 0
Delta_r
Cytarabine
YOR222W ODC2
ZShift = 0.31
Z lm Score = −1.23
NG = 0
DB = 0
SM = 0
Delta_AUC
Cytarabine

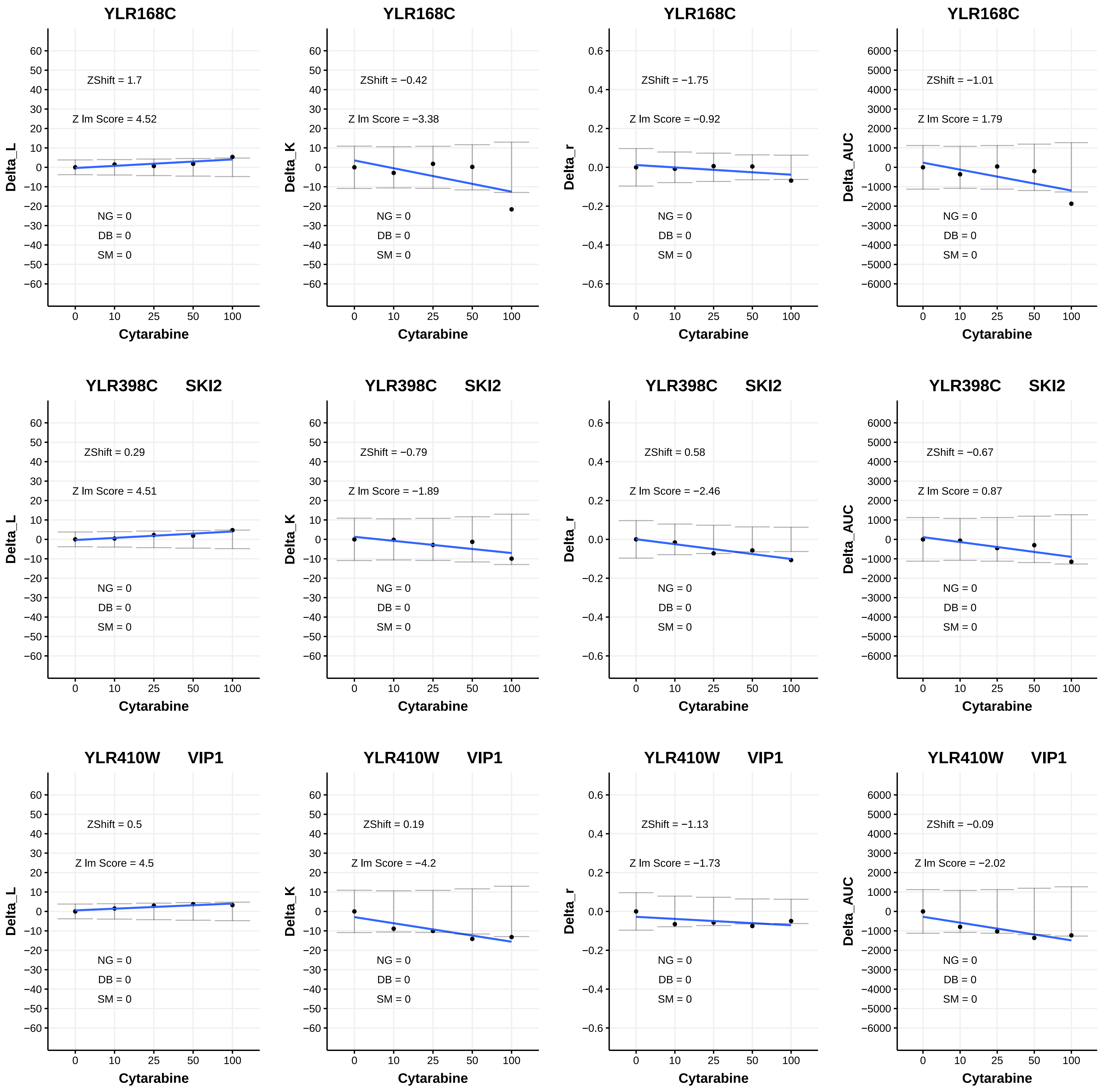

YLR168C
ZShift = 1.7
Z lm Score = 4.52
NG = 0
DB = 0
SM = 0
Delta_L
Cytarabine
YLR168C
ZShift = −0.42
Z lm Score = −3.38
NG = 0
DB = 0
SM = 0
Delta_K
Cytarabine
YLR168C
ZShift = −1.75
Z lm Score = −0.92
NG = 0
DB = 0
SM = 0
Delta_r
Cytarabine
YLR168C
ZShift = −1.01
Z lm Score = 1.79
NG = 0
DB = 0
SM = 0
Delta_AUC
Cytarabine
YLR398C SKI2
ZShift = 0.29
Z lm Score = 4.51
NG = 0
DB = 0
SM = 0
Delta_L
Cytarabine
YLR398C SKI2
ZShift = −0.79
Z lm Score = −1.89
NG = 0
DB = 0
SM = 0
Delta_K
Cytarabine
YLR398C SKI2
ZShift = 0.58
Z lm Score = −2.46
NG = 0
DB = 0
SM = 0
Delta_r
Cytarabine
YLR398C SKI2
ZShift = −0.67
Z lm Score = 0.87
NG = 0
DB = 0
SM = 0
Delta_AUC
Cytarabine
YLR410W VIP1
ZShift = 0.5
Z lm Score = 4.5
NG = 0
DB = 0
SM = 0
Delta_L
Cytarabine
YLR410W VIP1
ZShift = 0.19
Z lm Score = −4.2
NG = 0
DB = 0
SM = 0
Delta_K
Cytarabine
YLR410W VIP1
ZShift = −1.13
Z lm Score = −1.73
NG = 0
DB = 0
SM = 0
Delta_r
Cytarabine
YLR410W VIP1
ZShift = −0.09
Z lm Score = −2.02
NG = 0
DB = 0
SM = 0
Delta_AUC
Cytarabine

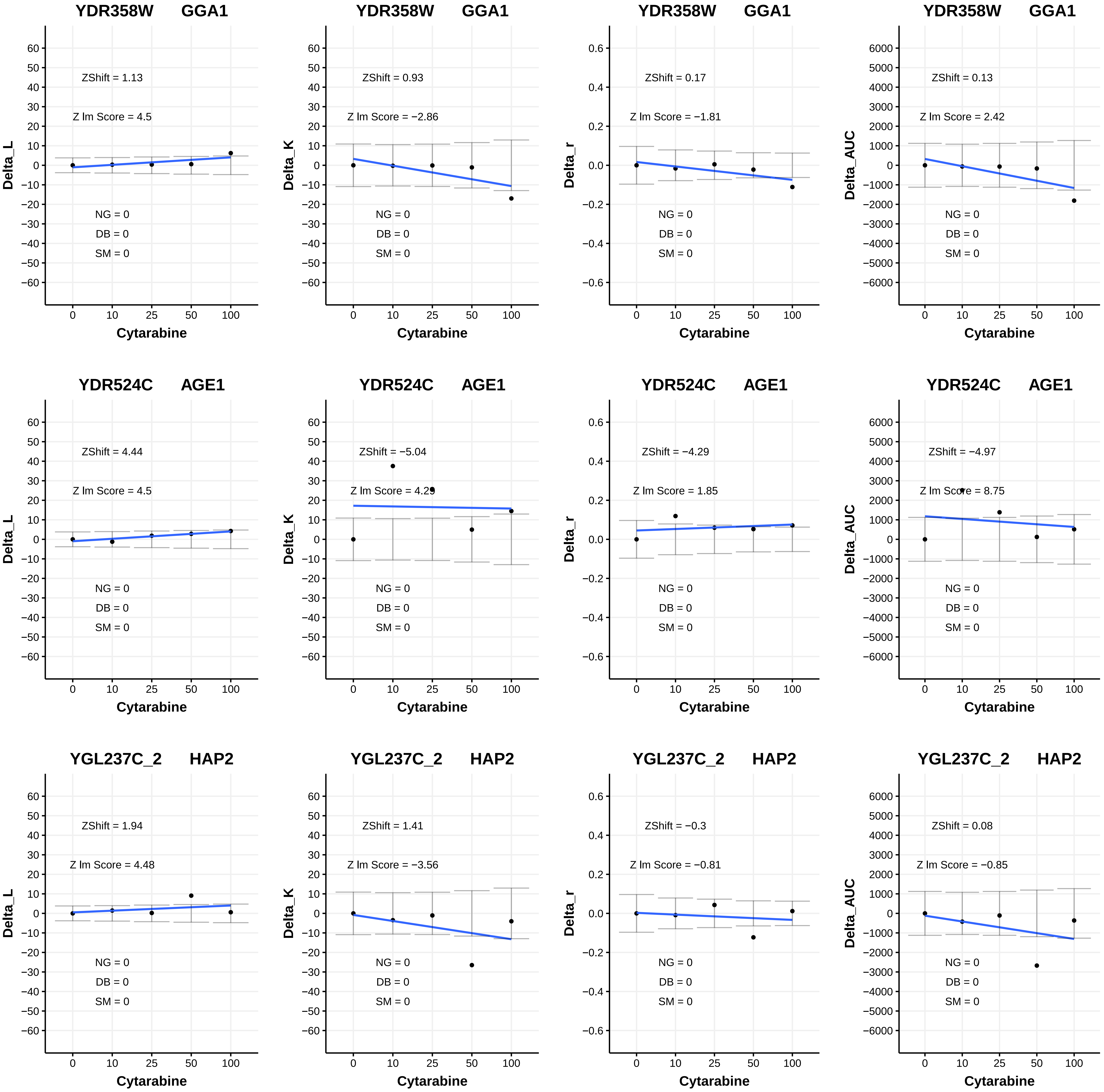

YDR358W GGA1
ZShift = 1.13
Z lm Score = 4.5
NG = 0
DB = 0
SM = 0
Delta_L
Cytarabine
YDR358W GGA1
ZShift = 0.93
Z lm Score = −2.86
NG = 0
DB = 0
SM = 0
Delta_K
Cytarabine
YDR358W GGA1
ZShift = 0.17
Z lm Score = −1.81
NG = 0
DB = 0
SM = 0
Delta_r
Cytarabine
YDR358W GGA1
ZShift = 0.13
Z lm Score = 2.42
NG = 0
DB = 0
SM = 0
Delta_AUC
Cytarabine
YDR524C AGE1
ZShift = 4.44
Z lm Score = 4.5
NG = 0
DB = 0
SM = 0
Delta_L
Cytarabine
YDR524C AGE1
ZShift = −5.04
Z lm Score = 4.29
NG = 0
DB = 0
SM = 0
Delta_K
Cytarabine
YDR524C AGE1
ZShift = −4.29
Z lm Score = 1.85
NG = 0
DB = 0
SM = 0
Delta_r
Cytarabine
YDR524C AGE1
ZShift = −4.97
Z lm Score = 8.75
NG = 0
DB = 0
SM = 0
Delta_AUC
Cytarabine
YGL237C_2 HAP2
ZShift = 1.94
Z lm Score = 4.48
NG = 0
DB = 0
SM = 0
Delta_L
Cytarabine
YGL237C_2 HAP2
ZShift = 1.41
Z lm Score = −3.56
NG = 0
DB = 0
SM = 0
Delta_K
Cytarabine
YGL237C_2 HAP2
ZShift = −0.3
Z lm Score = −0.81
NG = 0
DB = 0
SM = 0
Delta_r
Cytarabine
YGL237C_2 HAP2
ZShift = 0.08
Z lm Score = −0.85
NG = 0
DB = 0
SM = 0
Delta_AUC
Cytarabine

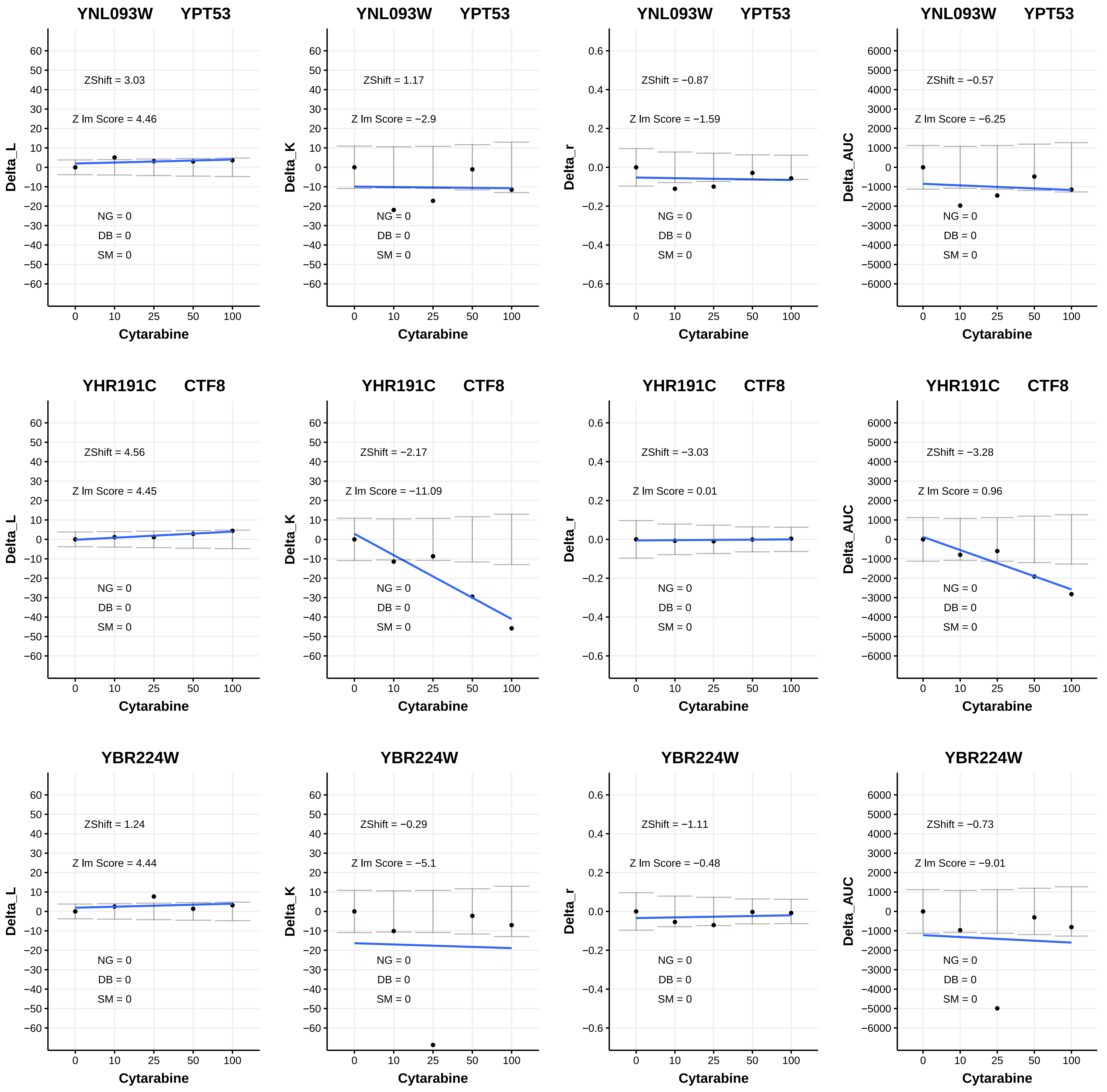

YNL093W YPT53
ZShift = 3.03
Z lm Score = 4.46
NG = 0
DB = 0
SM = 0
Delta_L
Cytarabine
YNL093W YPT53
ZShift = 1.17
Z lm Score = −2.9
NG = 0
DB = 0
SM = 0
Delta_K
Cytarabine
YNL093W YPT53
ZShift = −0.87
Z lm Score = −1.59
NG = 0
DB = 0
SM = 0
Delta_r
Cytarabine
YNL093W YPT53
ZShift = −0.57
Z lm Score = −6.25
NG = 0
DB = 0
SM = 0
Delta_AUC
Cytarabine
YHR191C CTF8
ZShift = 4.56
Z lm Score = 4.45
NG = 0
DB = 0
SM = 0
Delta_L
Cytarabine
YHR191C CTF8
ZShift = −2.17
Z lm Score = −11.09
NG = 0
DB = 0
SM = 0
Delta_K
Cytarabine
YHR191C CTF8
ZShift = −3.03
Z lm Score = 0.01
NG = 0
DB = 0
SM = 0
Delta_r
Cytarabine
YHR191C CTF8
ZShift = −3.28
Z lm Score = 0.96
NG = 0
DB = 0
SM = 0
Delta_AUC
Cytarabine
YBR224W
ZShift = 1.24
Z lm Score = 4.44
NG = 0
DB = 0
SM = 0
Delta_L
Cytarabine
YBR224W
ZShift = −0.29
Z lm Score = −5.1
NG = 0
DB = 0
SM = 0
Delta_K
Cytarabine
YBR224W
ZShift = −1.11
Z lm Score = −0.48
NG = 0
DB = 0
SM = 0
Delta_r
Cytarabine
YBR224W
ZShift = −0.73
Z lm Score = −9.01
NG = 0
DB = 0
SM = 0
Delta_AUC
Cytarabine

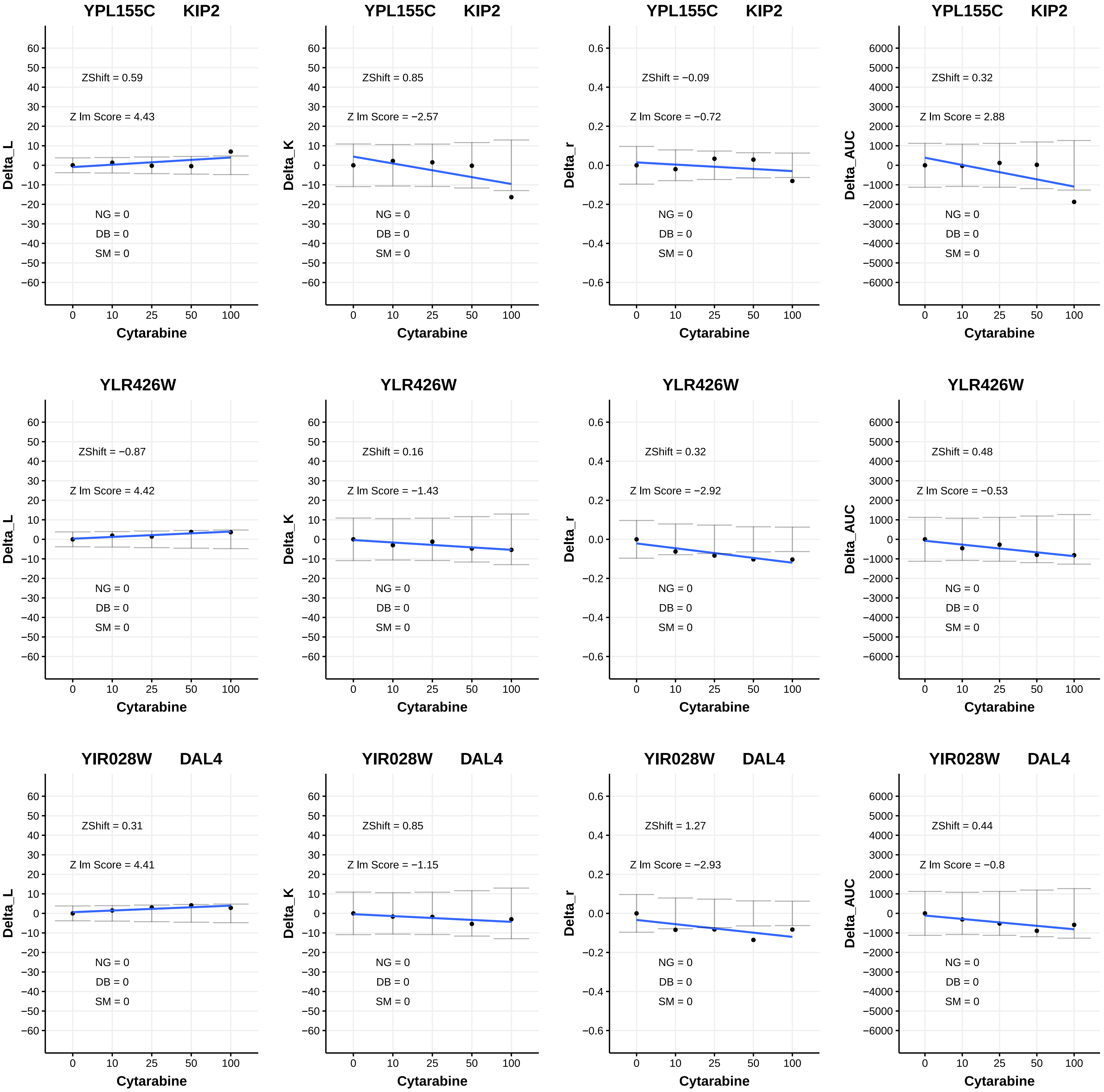
YPL155C KIP2
ZShift = 0.59
Z lm Score = 4.43
NG = 0
DB = 0
SM = 0
Delta_L
Cytarabine
YPL155C KIP2
ZShift = 0.85
Z lm Score = −2.57
NG = 0
DB = 0
SM = 0
Delta_K
Cytarabine
YPL155C KIP2
ZShift = −0.09
Z lm Score = −0.72
NG = 0
DB = 0
SM = 0
Delta_r
Cytarabine
YPL155C KIP2
ZShift = 0.32
Z lm Score = 2.88
NG = 0
DB = 0
SM = 0
Delta_AUC
Cytarabine
YLR426W
ZShift = −0.87
Z lm Score = 4.42
NG = 0
DB = 0
SM = 0
Delta_L
Cytarabine
YLR426W
ZShift = 0.16
Z lm Score = −1.43
NG = 0
DB = 0
SM = 0
Delta_K
Cytarabine
YLR426W
ZShift = 0.32
Z lm Score = −2.92
NG = 0
DB = 0
SM = 0
Delta_r
Cytarabine
YLR426W
ZShift = 0.48
Z lm Score = −0.53
NG = 0
DB = 0
SM = 0
Delta_AUC
Cytarabine
YIR028W DAL4
ZShift = 0.31
Z lm Score = 4.41
NG = 0
DB = 0
SM = 0
Delta_L
Cytarabine
YIR028W DAL4
ZShift = 0.85
Z lm Score = −1.15
NG = 0
DB = 0
SM = 0
Delta_K
Cytarabine
YIR028W DAL4
ZShift = 1.27
Z lm Score = −2.93
NG = 0
DB = 0
SM = 0
Delta_r
Cytarabine
YIR028W DAL4
ZShift = 0.44
Z lm Score = −0.8
NG = 0
DB = 0
SM = 0
Delta_AUC
Cytarabine

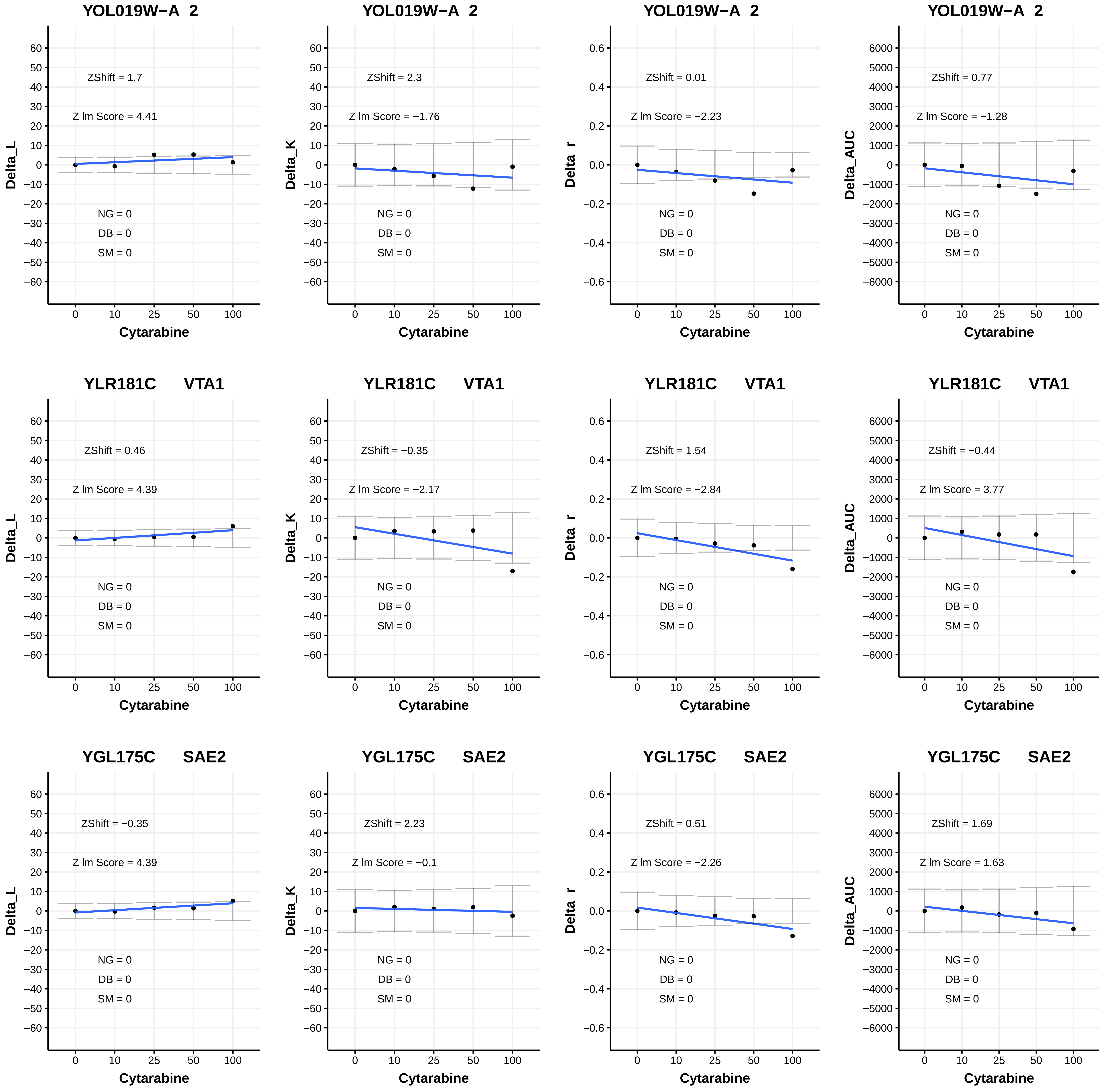
YOL019W−A_2
ZShift = 1.7
Z lm Score = 4.41
NG = 0
DB = 0
SM = 0
Delta_L
Cytarabine
YOL019W−A_2
ZShift = 2.3
Z lm Score = −1.76
NG = 0
DB = 0
SM = 0
Delta_K
Cytarabine
YOL019W−A_2
ZShift = 0.01
Z lm Score = −2.23
NG = 0
DB = 0
SM = 0
Delta_r
Cytarabine
YOL019W−A_2
ZShift = 0.77
Z lm Score = −1.28
NG = 0
DB = 0
SM = 0
Delta_AUC
Cytarabine
YLR181C VTA1
ZShift = 0.46
Z lm Score = 4.39
NG = 0
DB = 0
SM = 0
Delta_L
Cytarabine
YLR181C VTA1
ZShift = −0.35
Z lm Score = −2.17
NG = 0
DB = 0
SM = 0
Delta_K
Cytarabine
YLR181C VTA1
ZShift = 1.54
Z lm Score = −2.84
NG = 0
DB = 0
SM = 0
Delta_r
Cytarabine
YLR181C VTA1
ZShift = −0.44
Z lm Score = 3.77
NG = 0
DB = 0
SM = 0
Delta_AUC
Cytarabine
YGL175C SAE2
ZShift = −0.35
Z lm Score = 4.39
NG = 0
DB = 0
SM = 0
Delta_L
Cytarabine
YGL175C SAE2
ZShift = 2.23
Z lm Score = −0.1
NG = 0
DB = 0
SM = 0
Delta_K
Cytarabine
YGL175C SAE2
ZShift = 0.51
Z lm Score = −2.26
NG = 0
DB = 0
SM = 0
Delta_r
Cytarabine
YGL175C SAE2
ZShift = 1.69
Z lm Score = 1.63
NG = 0
DB = 0
SM = 0
Delta_AUC
Cytarabine

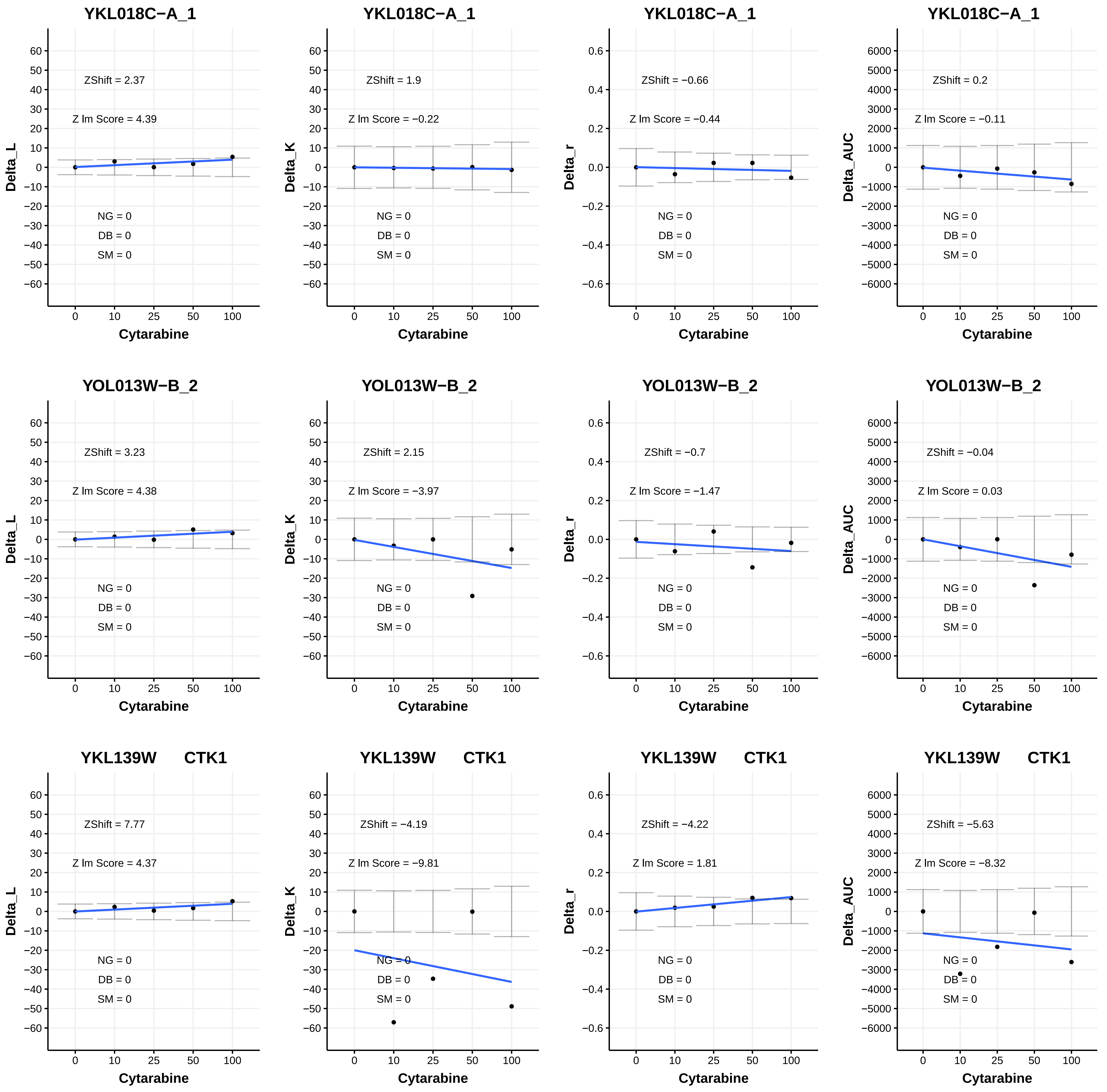
YKL018C−A_1
ZShift = 2.37
Z lm Score = 4.39
NG = 0
DB = 0
SM = 0
Delta_L
Cytarabine
YKL018C−A_1
ZShift = 1.9
Z lm Score = −0.22
NG = 0
DB = 0
SM = 0
Delta_K
Cytarabine
YKL018C−A_1
ZShift = −0.66
Z lm Score = −0.44
NG = 0
DB = 0
SM = 0
Delta_r
Cytarabine
YKL018C−A_1
ZShift = 0.2
Z lm Score = −0.11
NG = 0
DB = 0
SM = 0
Delta_AUC
Cytarabine
YOL013W−B_2
ZShift = 3.23
Z lm Score = 4.38
NG = 0
DB = 0
SM = 0
Delta_L
Cytarabine
YOL013W−B_2
ZShift = 2.15
Z lm Score = −3.97
NG = 0
DB = 0
SM = 0
Delta_K
Cytarabine
YOL013W−B_2
ZShift = −0.7
Z lm Score = −1.47
NG = 0
DB = 0
SM = 0
Delta_r
Cytarabine
YOL013W−B_2
ZShift = −0.04
Z lm Score = 0.03
NG = 0
DB = 0
SM = 0
Delta_AUC
Cytarabine
YKL139W CTK1
ZShift = 7.77
Z lm Score = 4.37
NG = 0
DB = 0
SM = 0
Delta_L
Cytarabine
YKL139W CTK1
ZShift = −4.19
Z lm Score = −9.81
NG = 0
DB = 0
SM = 0
Delta_K
Cytarabine
YKL139W CTK1
ZShift = −4.22
Z lm Score = 1.81
NG = 0
DB = 0
SM = 0
Delta_r
Cytarabine
YKL139W CTK1
ZShift = −5.63
Z lm Score = −8.32
NG = 0
DB = 0
SM = 0
Delta_AUC
Cytarabine

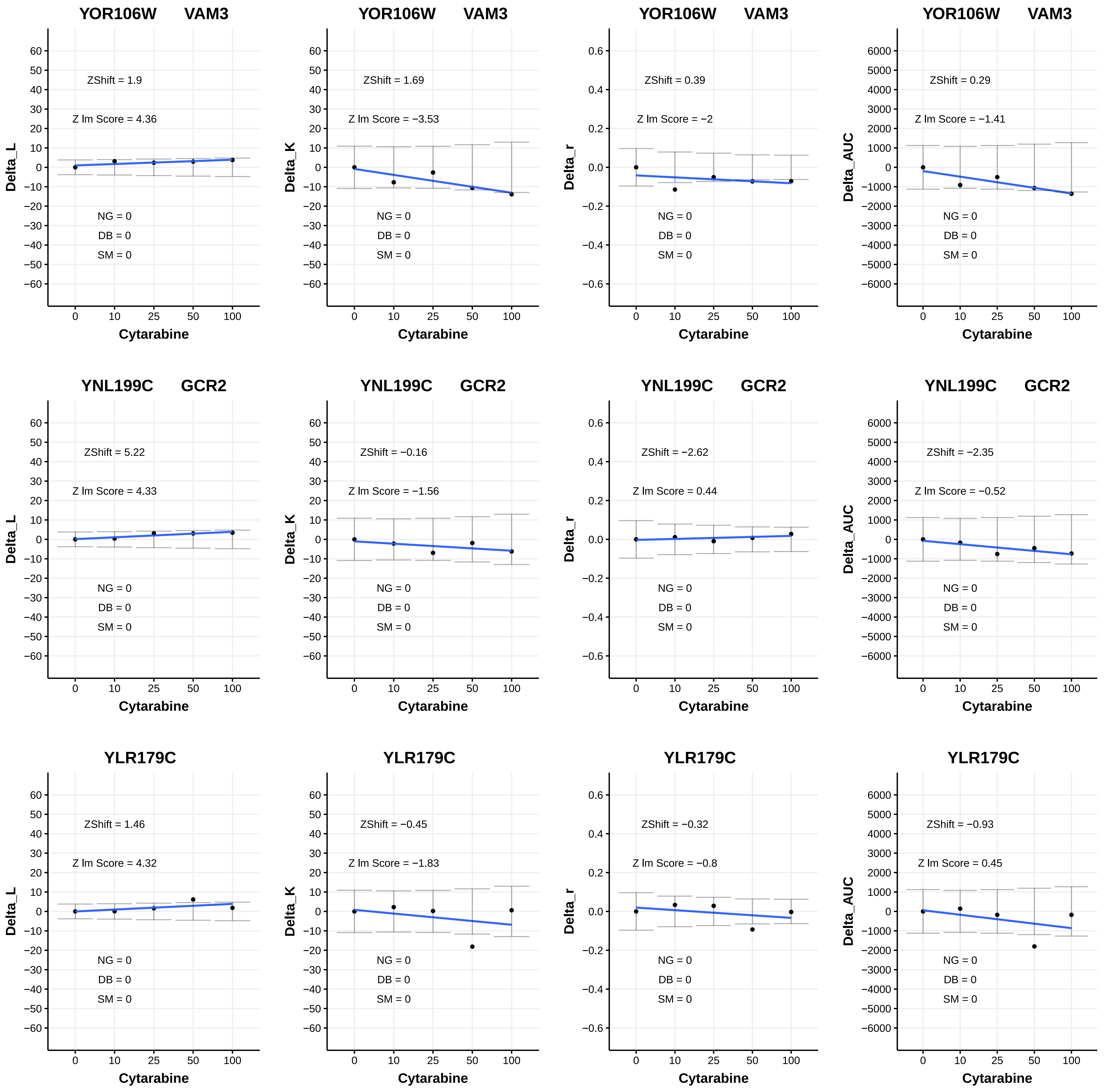

YOR106W VAM3
ZShift = 1.9
Z lm Score = 4.36
Delta_L
NG = 0
DB = 0
SM = 0
Cytarabine
YOR106W VAM3
ZShift = 1.69
Z lm Score = −3.53
Delta_K
NG = 0
DB = 0
SM = 0
Cytarabine
YOR106W VAM3
ZShift = 0.39
Z lm Score = −2
Delta_r
NG = 0
DB = 0
SM = 0
Cytarabine
YOR106W VAM3
ZShift = 0.29
Z lm Score = −1.41
Delta_AUC
NG = 0
DB = 0
SM = 0
Cytarabine
YNL199C GCR2
ZShift = 5.22
Z lm Score = 4.33
Delta_L
NG = 0
DB = 0
SM = 0
Cytarabine
YNL199C GCR2
ZShift = −0.16
Z lm Score = −1.56
Delta_K
NG = 0
DB = 0
SM = 0
Cytarabine
YNL199C GCR2
ZShift = −2.62
Z lm Score = 0.44
Delta_r
NG = 0
DB = 0
SM = 0
Cytarabine
YNL199C GCR2
ZShift = −2.35
Z lm Score = −0.52
Delta_AUC
NG = 0
DB = 0
SM = 0
Cytarabine
YLR179C
ZShift = 1.46
Z lm Score = 4.32
Delta_L
NG = 0
DB = 0
SM = 0
Cytarabine
YLR179C
ZShift = −0.45
Z lm Score = −1.83
Delta_K
NG = 0
DB = 0
SM = 0
Cytarabine
YLR179C
ZShift = −0.32
Z lm Score = −0.8
Delta_r
NG = 0
DB = 0
SM = 0
Cytarabine
YLR179C
ZShift = −0.93
Z lm Score = 0.45
Delta_AUC
NG = 0
DB = 0
SM = 0
Cytarabine

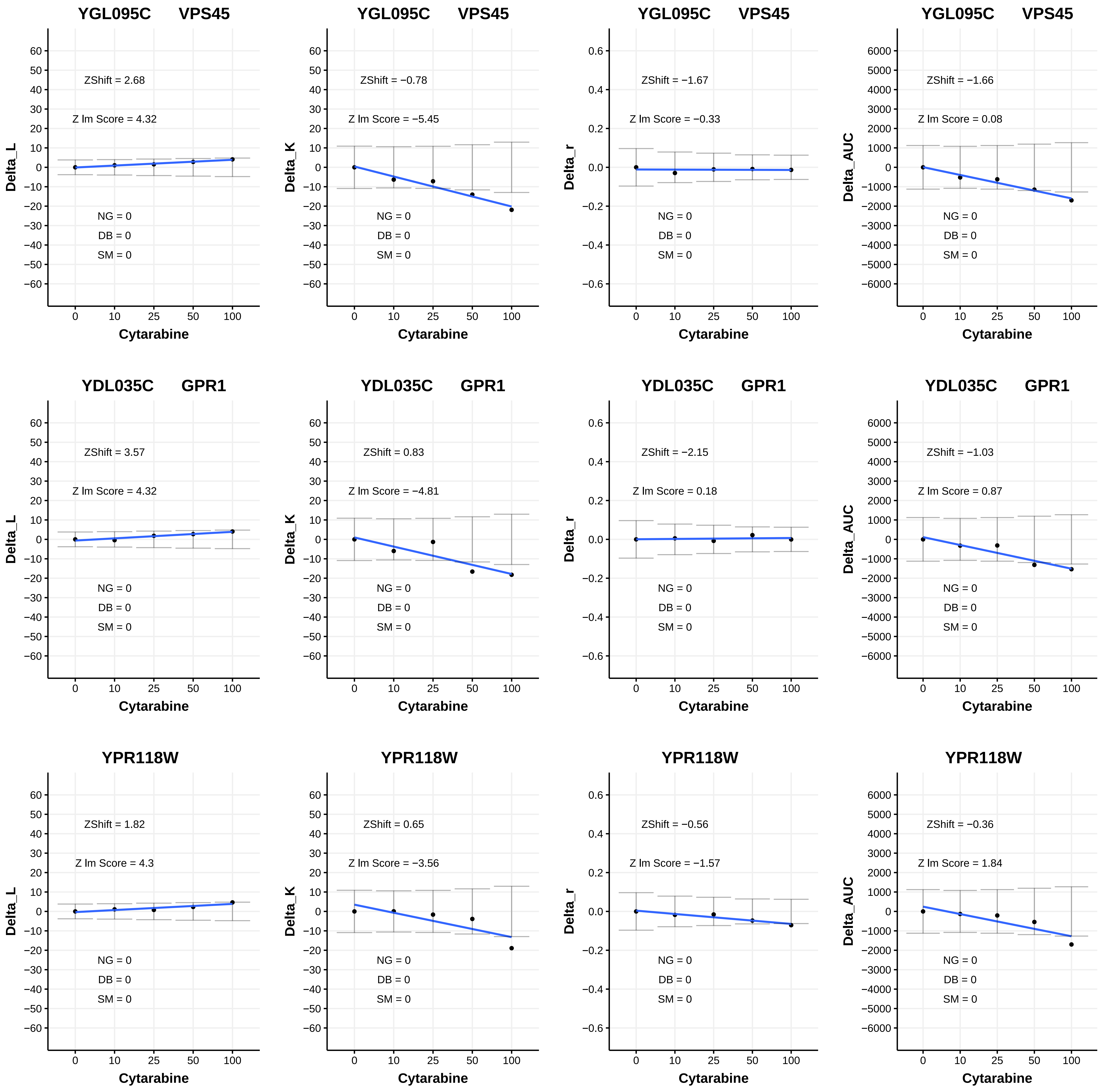
YGL095C VPS45
ZShift = 2.68
Z lm Score = 4.32
Delta_L
NG = 0
DB = 0
SM = 0
Cytarabine
YGL095C VPS45
ZShift = −0.78
Z lm Score = −5.45
Delta_K
NG = 0
DB = 0
SM = 0
Cytarabine
YGL095C VPS45
ZShift = −1.67
Z lm Score = −0.33
Delta_r
NG = 0
DB = 0
SM = 0
Cytarabine
YGL095C VPS45
ZShift = −1.66
Z lm Score = 0.08
Delta_AUC
NG = 0
DB = 0
SM = 0
Cytarabine
YDL035C GPR1
ZShift = 3.57
Z lm Score = 4.32
Delta_L
NG = 0
DB = 0
SM = 0
Cytarabine
YDL035C GPR1
ZShift = 0.83
Z lm Score = −4.81
Delta_K
NG = 0
DB = 0
SM = 0
Cytarabine
YDL035C GPR1
ZShift = −2.15
Z lm Score = 0.18
Delta_r
NG = 0
DB = 0
SM = 0
Cytarabine
YDL035C GPR1
ZShift = −1.03
Z lm Score = 0.87
Delta_AUC
NG = 0
DB = 0
SM = 0
Cytarabine
YPR118W
ZShift = 1.82
Z lm Score = 4.3
Delta_L
NG = 0
DB = 0
SM = 0
Cytarabine
YPR118W
ZShift = 0.65
Z lm Score = −3.56
Delta_K
NG = 0
DB = 0
SM = 0
Cytarabine
YPR118W
ZShift = −0.56
Z lm Score = −1.57
Delta_r
NG = 0
DB = 0
SM = 0
Cytarabine
YPR118W
ZShift = −0.36
Z lm Score = 1.84
Delta_AUC
NG = 0
DB = 0
SM = 0
Cytarabine

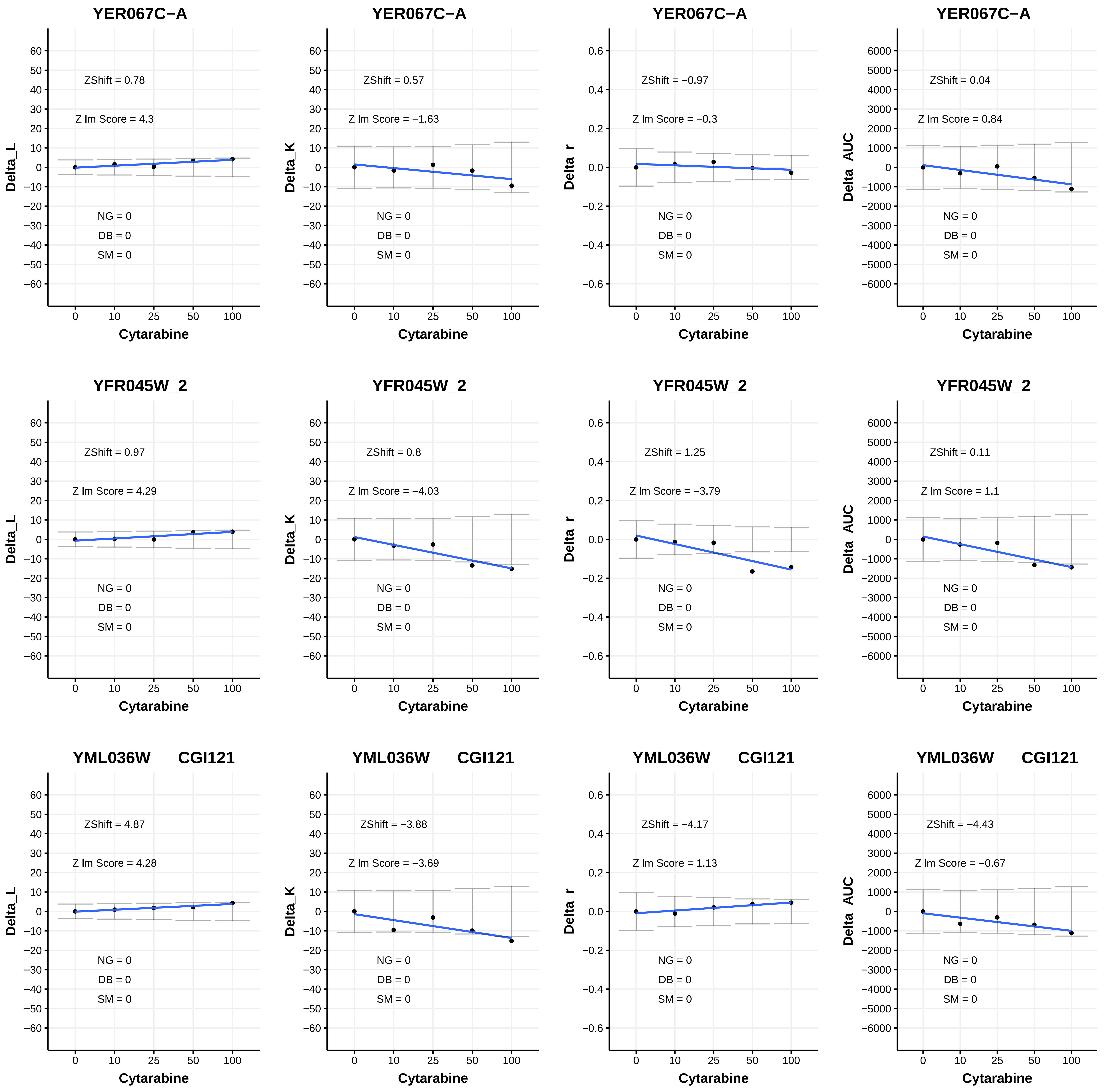

YER067C−A
ZShift = 0.78
Z lm Score = 4.3
NG = 0
DB = 0
SM = 0
Delta_L
Cytarabine
YER067C−A
ZShift = 0.57
Z lm Score = −1.63
NG = 0
DB = 0
SM = 0
Delta_K
Cytarabine
YER067C−A
ZShift = −0.97
Z lm Score = −0.3
NG = 0
DB = 0
SM = 0
Delta_r
Cytarabine
YER067C−A
ZShift = 0.04
Z lm Score = 0.84
NG = 0
DB = 0
SM = 0
Delta_AUC
Cytarabine
YFR045W_2
ZShift = 0.97
Z lm Score = 4.29
NG = 0
DB = 0
SM = 0
Delta_L
Cytarabine
YFR045W_2
ZShift = 0.8
Z lm Score = −4.03
NG = 0
DB = 0
SM = 0
Delta_K
Cytarabine
YFR045W_2
ZShift = 1.25
Z lm Score = −3.79
NG = 0
DB = 0
SM = 0
Delta_r
Cytarabine
YFR045W_2
ZShift = 0.11
Z lm Score = 1.1
NG = 0
DB = 0
SM = 0
Delta_AUC
Cytarabine
YML036W CGI121
ZShift = 4.87
Z lm Score = 4.28
NG = 0
DB = 0
SM = 0
Delta_L
Cytarabine
YML036W CGI121
ZShift = −3.88
Z lm Score = −3.69
NG = 0
DB = 0
SM = 0
Delta_K
Cytarabine
YML036W CGI121
ZShift = −4.17
Z lm Score = 1.13
NG = 0
DB = 0
SM = 0
Delta_r
Cytarabine
YML036W CGI121
ZShift = −4.43
Z lm Score = −0.67
NG = 0
DB = 0
SM = 0
Delta_AUC
Cytarabine

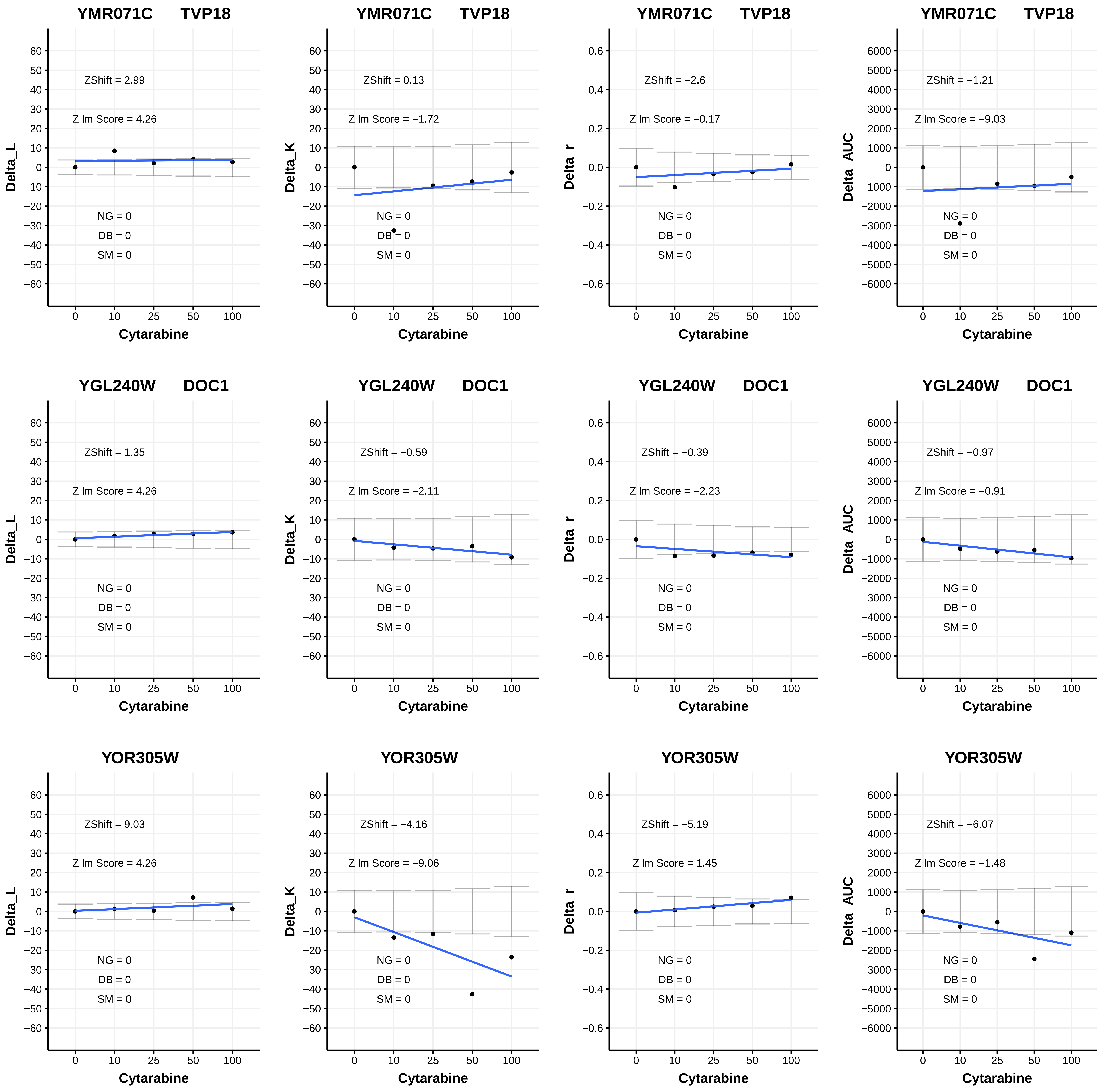

YMR071C TVP18
ZShift = 2.99
Z lm Score = 4.26
NG = 0
DB = 0
SM = 0
Delta_L
Cytarabine
YMR071C TVP18
ZShift = 0.13
Z lm Score = −1.72
NG = 0
DB = 0
SM = 0
Delta_K
Cytarabine
YMR071C TVP18
ZShift = −2.6
Z lm Score = −0.17
NG = 0
DB = 0
SM = 0
Delta_r
Cytarabine
YMR071C TVP18
ZShift = −1.21
Z lm Score = −9.03
NG = 0
DB = 0
SM = 0
Delta_AUC
Cytarabine
YGL240W DOC1
ZShift = 1.35
Z lm Score = 4.26
NG = 0
DB = 0
SM = 0
Delta_L
Cytarabine
YGL240W DOC1
ZShift = −0.59
Z lm Score = −2.11
NG = 0
DB = 0
SM = 0
Delta_K
Cytarabine
YGL240W DOC1
ZShift = −0.39
Z lm Score = −2.23
NG = 0
DB = 0
SM = 0
Delta_r
Cytarabine
YGL240W DOC1
ZShift = −0.97
Z lm Score = −0.91
NG = 0
DB = 0
SM = 0
Delta_AUC
Cytarabine
YOR305W
ZShift = 9.03
Z lm Score = 4.26
NG = 0
DB = 0
SM = 0
Delta_L
Cytarabine
YOR305W
ZShift = −4.16
Z lm Score = −9.06
NG = 0
DB = 0
SM = 0
Delta_K
Cytarabine
YOR305W
ZShift = −5.19
Z lm Score = 1.45
NG = 0
DB = 0
SM = 0
Delta_r
Cytarabine
YOR305W
ZShift = −6.07
Z lm Score = −1.48
NG = 0
DB = 0
SM = 0
Delta_AUC
Cytarabine

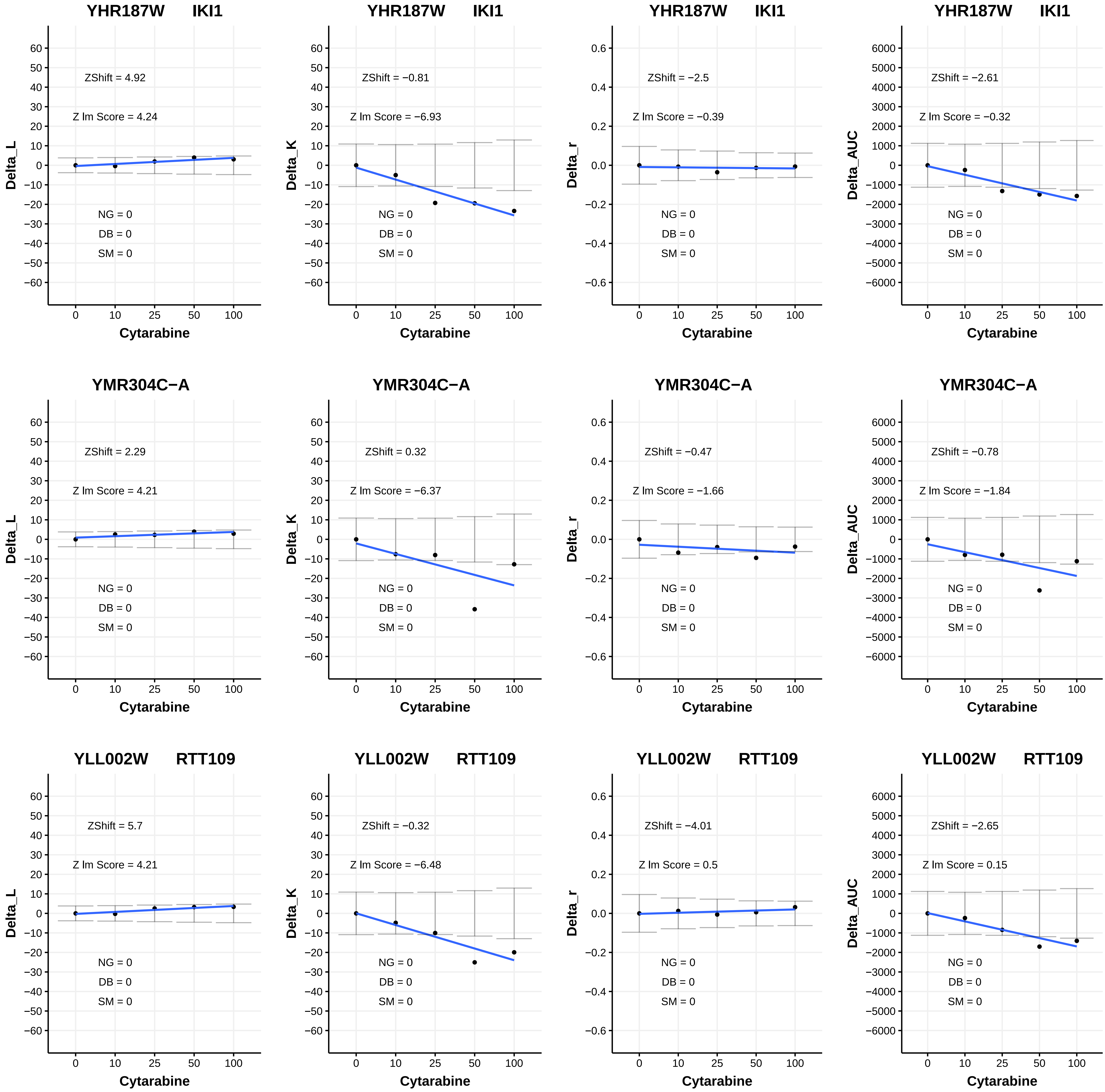
YHR187W IKI1
ZShift = 4.92
Z lm Score = 4.24
NG = 0
DB = 0
SM = 0
Delta_L
Cytarabine
YHR187W IKI1
ZShift = −0.81
Z lm Score = −6.93
NG = 0
DB = 0
SM = 0
Delta_K
Cytarabine
YHR187W IKI1
ZShift = −2.5
Z lm Score = −0.39
NG = 0
DB = 0
SM = 0
Delta_r
Cytarabine
YHR187W IKI1
ZShift = −2.61
Z lm Score = −0.32
NG = 0
DB = 0
SM = 0
Delta_AUC
Cytarabine
YMR304C−A
ZShift = 2.29
Z lm Score = 4.21
NG = 0
DB = 0
SM = 0
Delta_L
Cytarabine
YMR304C−A
ZShift = 0.32
Z lm Score = −6.37
NG = 0
DB = 0
SM = 0
Delta_K
Cytarabine
YMR304C−A
ZShift = −0.47
Z lm Score = −1.66
NG = 0
DB = 0
SM = 0
Delta_r
Cytarabine
YMR304C−A
ZShift = −0.78
Z lm Score = −1.84
NG = 0
DB = 0
SM = 0
Delta_AUC
Cytarabine
YLL002W RTT109
ZShift = 5.7
Z lm Score = 4.21
NG = 0
DB = 0
SM = 0
Delta_L
Cytarabine
YLL002W RTT109
ZShift = −0.32
Z lm Score = −6.48
NG = 0
DB = 0
SM = 0
Delta_K
Cytarabine
YLL002W RTT109
ZShift = −4.01
Z lm Score = 0.5
NG = 0
DB = 0
SM = 0
Delta_r
Cytarabine
YLL002W RTT109
ZShift = −2.65
Z lm Score = 0.15
NG = 0
DB = 0
SM = 0
Delta_AUC
Cytarabine

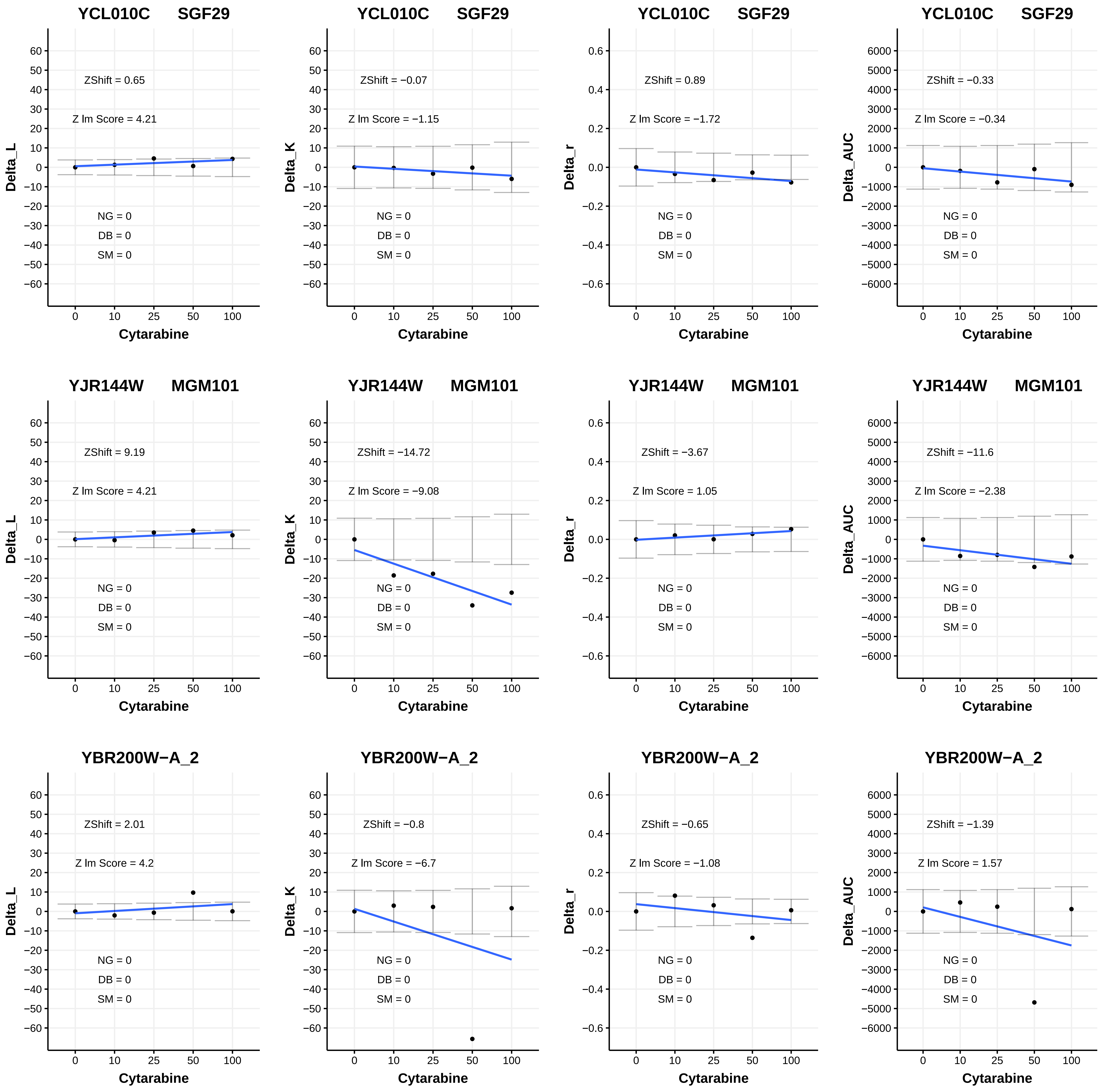

YCL010C SGF29
ZShift = 0.65
Z lm Score = 4.21
NG = 0
DB = 0
SM = 0
Delta_L
Cytarabine
YCL010C SGF29
ZShift = −0.07
Z lm Score = −1.15
NG = 0
DB = 0
SM = 0
Delta_K
Cytarabine
YCL010C SGF29
ZShift = 0.89
Z lm Score = −1.72
NG = 0
DB = 0
SM = 0
Delta_r
Cytarabine
YCL010C SGF29
ZShift = −0.33
Z lm Score = −0.34
NG = 0
DB = 0
SM = 0
Delta_AUC
Cytarabine
YJR144W MGM101
ZShift = 9.19
Z lm Score = 4.21
NG = 0
DB = 0
SM = 0
Delta_L
Cytarabine
YJR144W MGM101
ZShift = −14.72
Z lm Score = −9.08
NG = 0
DB = 0
SM = 0
Delta_K
Cytarabine
YJR144W MGM101
ZShift = −3.67
Z lm Score = 1.05
NG = 0
DB = 0
SM = 0
Delta_r
Cytarabine
YJR144W MGM101
ZShift = −11.6
Z lm Score = −2.38
NG = 0
DB = 0
SM = 0
Delta_AUC
Cytarabine
YBR200W−A_2
ZShift = 2.01
Z lm Score = 4.2
NG = 0
DB = 0
SM = 0
Delta_L
Cytarabine
YBR200W−A_2
ZShift = −0.8
Z lm Score = −6.7
NG = 0
DB = 0
SM = 0
Delta_K
Cytarabine
YBR200W−A_2
ZShift = −0.65
Z lm Score = −1.08
NG = 0
DB = 0
SM = 0
Delta_r
Cytarabine
YBR200W−A_2
ZShift = −1.39
Z lm Score = 1.57
NG = 0
DB = 0
SM = 0
Delta_AUC
Cytarabine

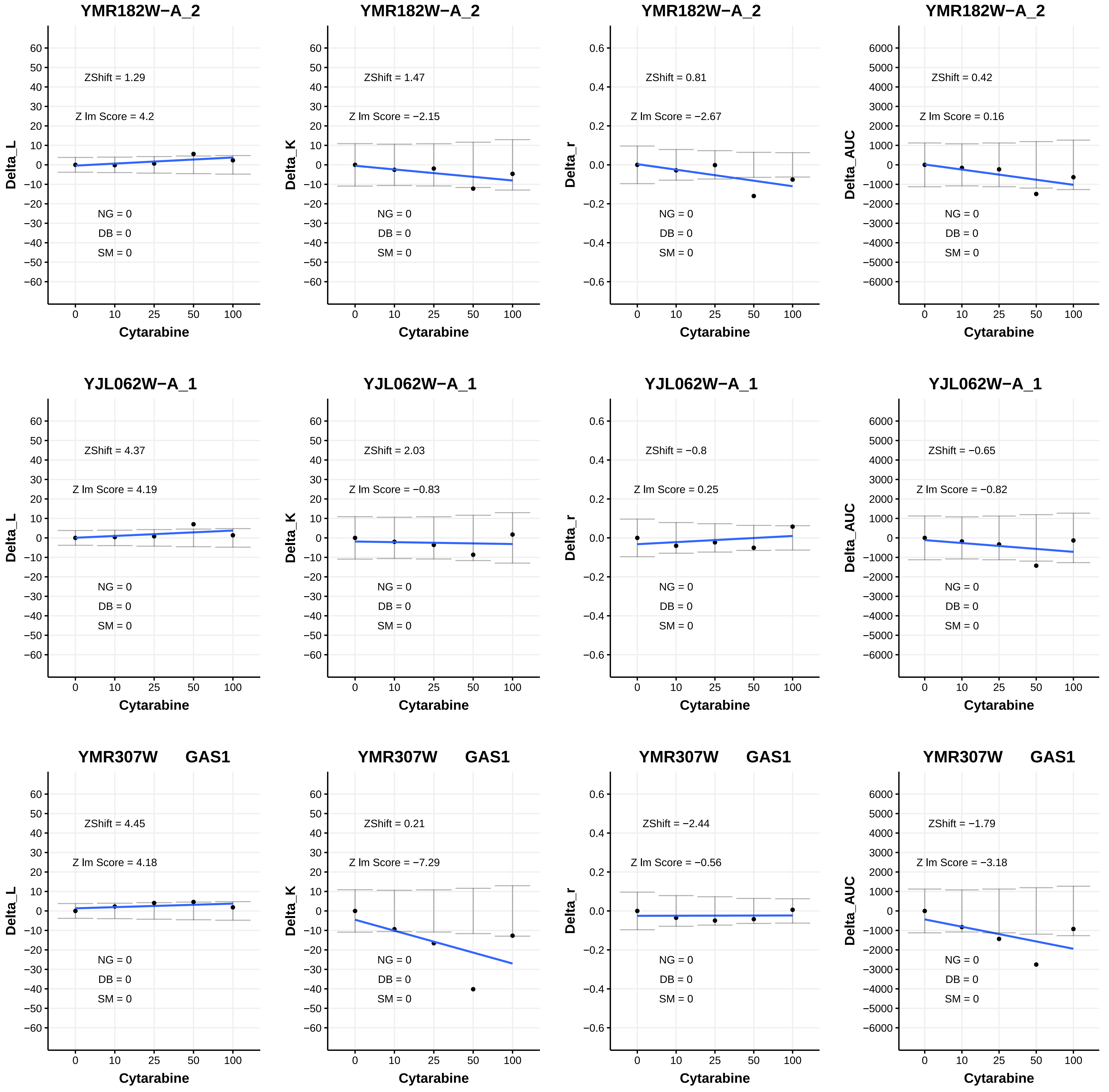
YMR182W−A_2
ZShift = 1.29
Z lm Score = 4.2
NG = 0
DB = 0
SM = 0
Delta_L
Cytarabine
YMR182W−A_2
ZShift = 1.47
Z lm Score = −2.15
NG = 0
DB = 0
SM = 0
Delta_K
Cytarabine
YMR182W−A_2
ZShift = 0.81
Z lm Score = −2.67
NG = 0
DB = 0
SM = 0
Delta_r
Cytarabine
YMR182W−A_2
ZShift = 0.42
Z lm Score = 0.16
NG = 0
DB = 0
SM = 0
Delta_AUC
Cytarabine
YJL062W−A_1
ZShift = 4.37
Z lm Score = 4.19
NG = 0
DB = 0
SM = 0
Delta_L
Cytarabine
YJL062W−A_1
ZShift = 2.03
Z lm Score = −0.83
NG = 0
DB = 0
SM = 0
Delta_K
Cytarabine
YJL062W−A_1
ZShift = −0.8
Z lm Score = 0.25
NG = 0
DB = 0
SM = 0
Delta_r
Cytarabine
YJL062W−A_1
ZShift = −0.65
Z lm Score = −0.82
NG = 0
DB = 0
SM = 0
Delta_AUC
Cytarabine
YMR307W GAS1
ZShift = 4.45
Z lm Score = 4.18
NG = 0
DB = 0
SM = 0
Delta_L
Cytarabine
YMR307W GAS1
ZShift = 0.21
Z lm Score = −7.29
NG = 0
DB = 0
SM = 0
Delta_K
Cytarabine
YMR307W GAS1
ZShift = −2.44
Z lm Score = −0.56
NG = 0
DB = 0
SM = 0
Delta_r
Cytarabine
YMR307W GAS1
ZShift = −1.79
Z lm Score = −3.18
NG = 0
DB = 0
SM = 0
Delta_AUC
Cytarabine

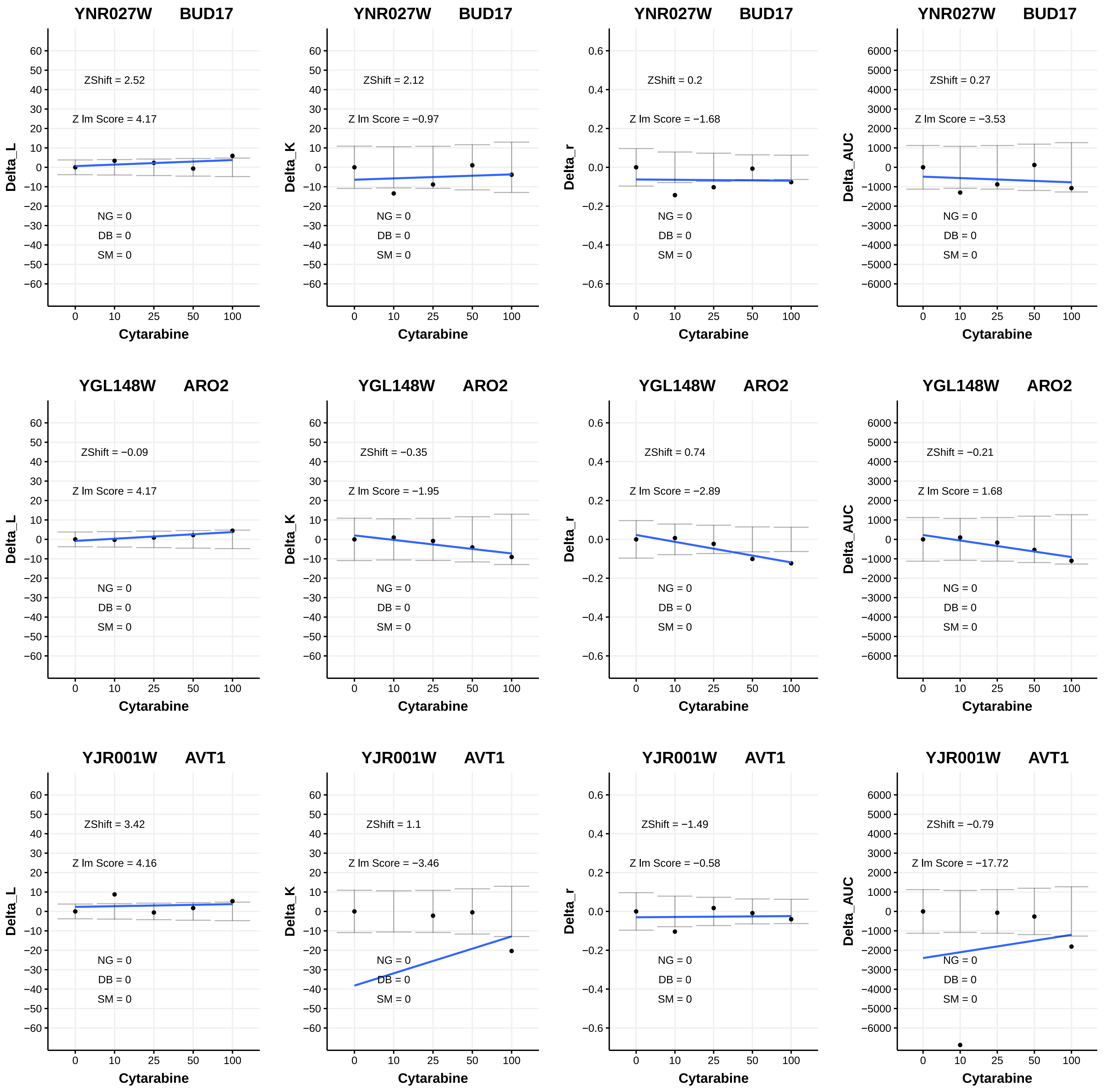

YNR027W BUD17
ZShift = 2.52
Z lm Score = 4.17
NG = 0
DB = 0
SM = 0
Delta_L
Cytarabine
YNR027W BUD17
ZShift = 2.12
Z lm Score = −0.97
NG = 0
DB = 0
SM = 0
Delta_K
Cytarabine
YNR027W BUD17
ZShift = 0.2
Z lm Score = −1.68
NG = 0
DB = 0
SM = 0
Delta_r
Cytarabine
YNR027W BUD17
ZShift = 0.27
Z lm Score = −3.53
NG = 0
DB = 0
SM = 0
Delta_AUC
Cytarabine
YGL148W ARO2
ZShift = −0.09
Z lm Score = 4.17
NG = 0
DB = 0
SM = 0
Delta_L
Cytarabine
YGL148W ARO2
ZShift = −0.35
Z lm Score = −1.95
NG = 0
DB = 0
SM = 0
Delta_K
Cytarabine
YGL148W ARO2
ZShift = 0.74
Z lm Score = −2.89
NG = 0
DB = 0
SM = 0
Delta_r
Cytarabine
YGL148W ARO2
ZShift = −0.21
Z lm Score = 1.68
NG = 0
DB = 0
SM = 0
Delta_AUC
Cytarabine
YJR001W AVT1
ZShift = 3.42
Z lm Score = 4.16
NG = 0
DB = 0
SM = 0
Delta_L
Cytarabine
YJR001W AVT1
ZShift = 1.1
Z lm Score = −3.46
NG = 0
DB = 0
SM = 0
Delta_K
Cytarabine
YJR001W AVT1
ZShift = −1.49
Z lm Score = −0.58
NG = 0
DB = 0
SM = 0
Delta_r
Cytarabine
YJR001W AVT1
ZShift = −0.79
Z lm Score = −17.72
NG = 0
DB = 0
SM = 0
Delta_AUC
Cytarabine

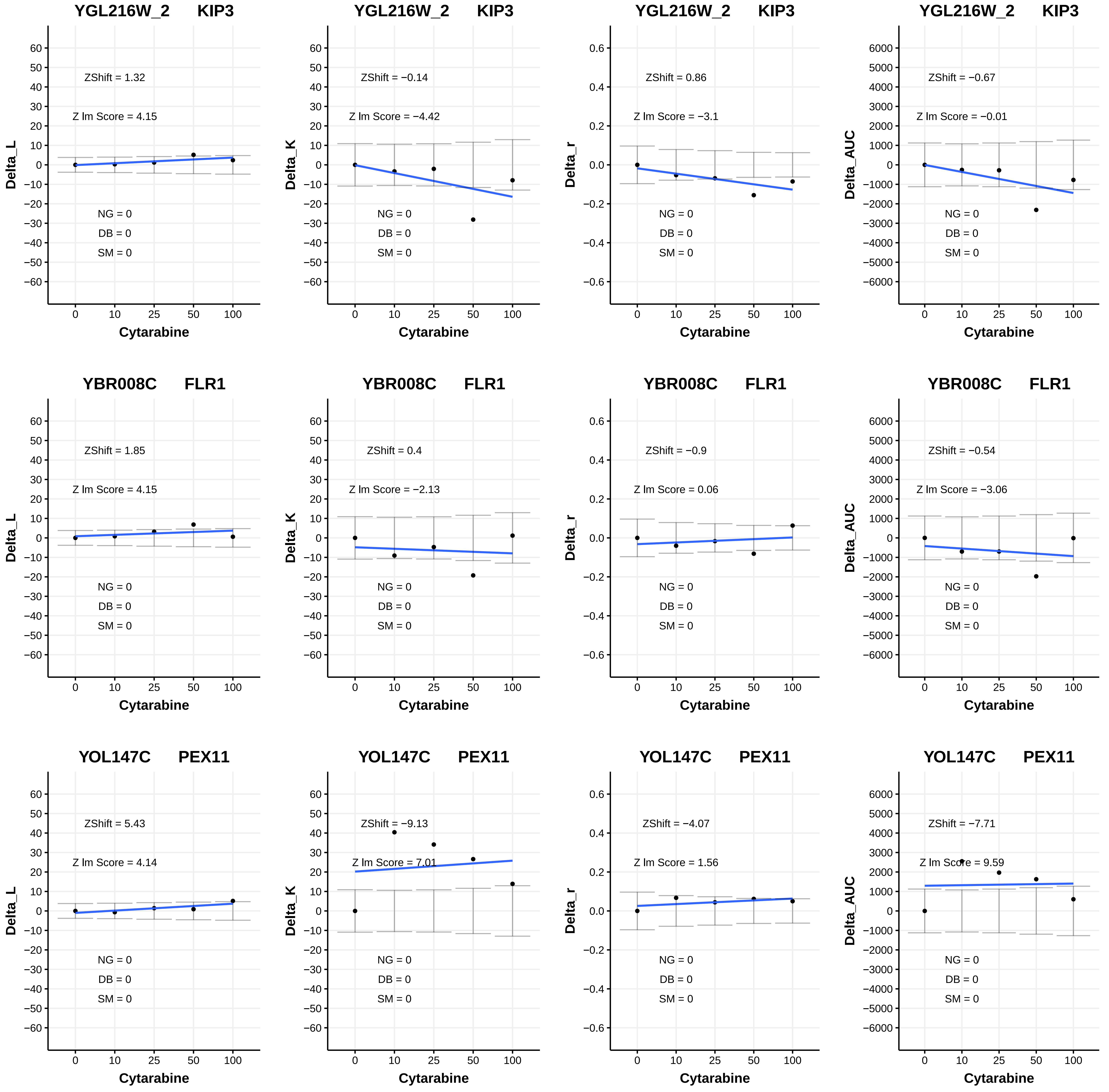

YGL216W_2 KIP3
ZShift = 1.32
Z lm Score = 4.15
NG = 0
DB = 0
SM = 0
Delta_L
Cytarabine
YGL216W_2 KIP3
ZShift = −0.14
Z lm Score = −4.42
NG = 0
DB = 0
SM = 0
Delta_K
Cytarabine
YGL216W_2 KIP3
ZShift = 0.86
Z lm Score = −3.1
NG = 0
DB = 0
SM = 0
Delta_r
Cytarabine
YGL216W_2 KIP3
ZShift = −0.67
Z lm Score = −0.01
NG = 0
DB = 0
SM = 0
Delta_AUC
Cytarabine
YBR008C FLR1
ZShift = 1.85
Z lm Score = 4.15
NG = 0
DB = 0
SM = 0
Delta_L
Cytarabine
YBR008C FLR1
ZShift = 0.4
Z lm Score = −2.13
NG = 0
DB = 0
SM = 0
Delta_K
Cytarabine
YBR008C FLR1
ZShift = −0.9
Z lm Score = 0.06
NG = 0
DB = 0
SM = 0
Delta_r
Cytarabine
YBR008C FLR1
ZShift = −0.54
Z lm Score = −3.06
NG = 0
DB = 0
SM = 0
Delta_AUC
Cytarabine
YOL147C PEX11
ZShift = 5.43
Z lm Score = 4.14
NG = 0
DB = 0
SM = 0
Delta_L
Cytarabine
YOL147C PEX11
ZShift = −9.13
Z lm Score = 7.01
NG = 0
DB = 0
SM = 0
Delta_K
Cytarabine
YOL147C PEX11
ZShift = −4.07
Z lm Score = 1.56
NG = 0
DB = 0
SM = 0
Delta_r
Cytarabine
YOL147C PEX11
ZShift = −7.71
Z lm Score = 9.59
NG = 0
DB = 0
SM = 0
Delta_AUC
Cytarabine

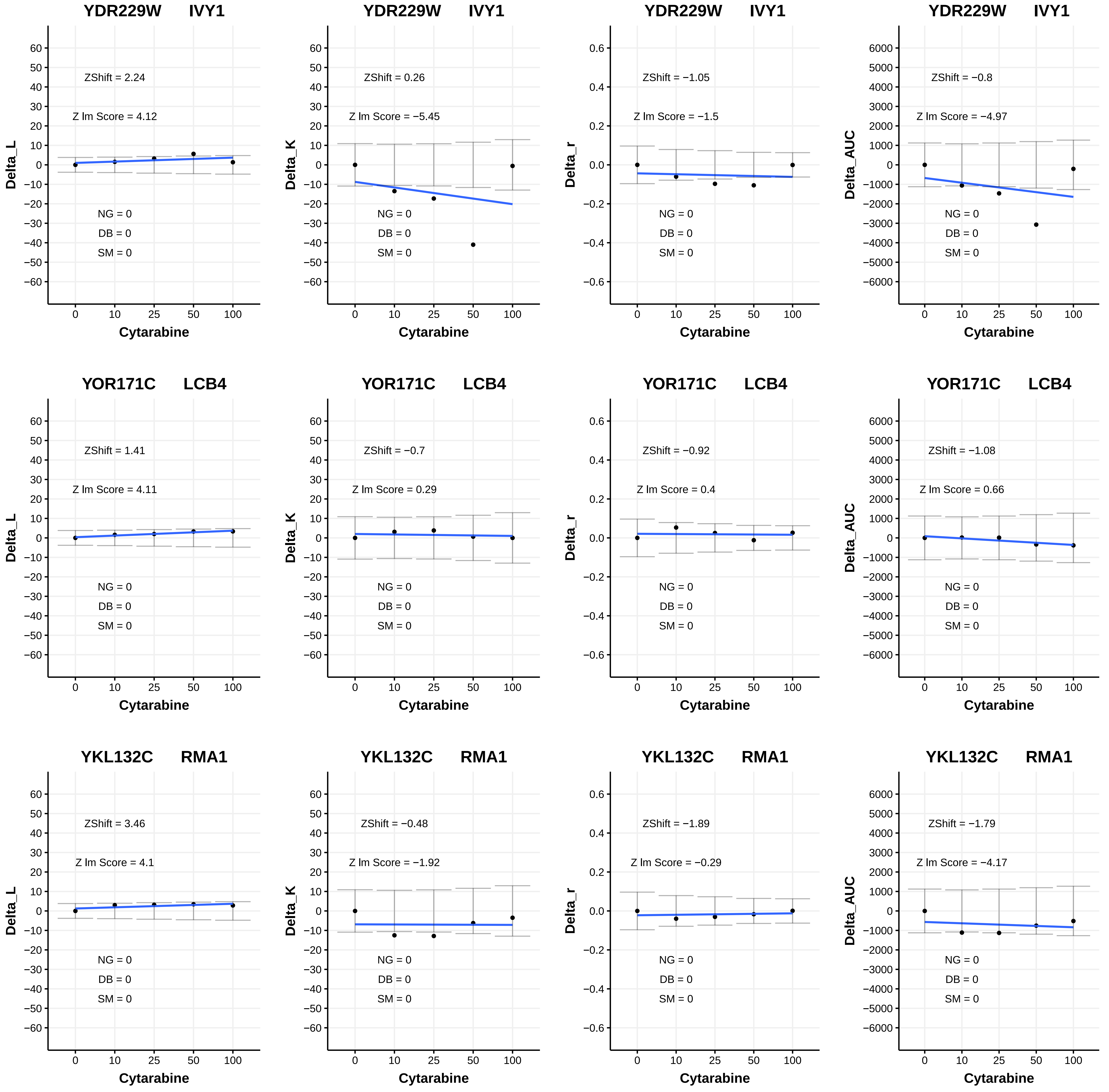
YDR229W IVY1
ZShift = 2.24
Z lm Score = 4.12
NG = 0
DB = 0
SM = 0
Delta_L
Cytarabine
YDR229W IVY1
ZShift = 0.26
Z lm Score = −5.45
NG = 0
DB = 0
SM = 0
Delta_K
Cytarabine
YDR229W IVY1
ZShift = −1.05
Z lm Score = −1.5
NG = 0
DB = 0
SM = 0
Delta_r
Cytarabine
YDR229W IVY1
ZShift = −0.8
Z lm Score = −4.97
NG = 0
DB = 0
SM = 0
Delta_AUC
Cytarabine
YOR171C LCB4
ZShift = 1.41
Z lm Score = 4.11
NG = 0
DB = 0
SM = 0
Delta_L
Cytarabine
YOR171C LCB4
ZShift = −0.7
Z lm Score = 0.29
NG = 0
DB = 0
SM = 0
Delta_K
Cytarabine
YOR171C LCB4
ZShift = −0.92
Z lm Score = 0.4
NG = 0
DB = 0
SM = 0
Delta_r
Cytarabine
YOR171C LCB4
ZShift = −1.08
Z lm Score = 0.66
NG = 0
DB = 0
SM = 0
Delta_AUC
Cytarabine
YKL132C RMA1
ZShift = 3.46
Z lm Score = 4.1
NG = 0
DB = 0
SM = 0
Delta_L
Cytarabine
YKL132C RMA1
ZShift = −0.48
Z lm Score = −1.92
NG = 0
DB = 0
SM = 0
Delta_K
Cytarabine
YKL132C RMA1
ZShift = −1.89
Z lm Score = −0.29
NG = 0
DB = 0
SM = 0
Delta_r
Cytarabine
YKL132C RMA1
ZShift = −1.79
Z lm Score = −4.17
NG = 0
DB = 0
SM = 0
Delta_AUC
Cytarabine

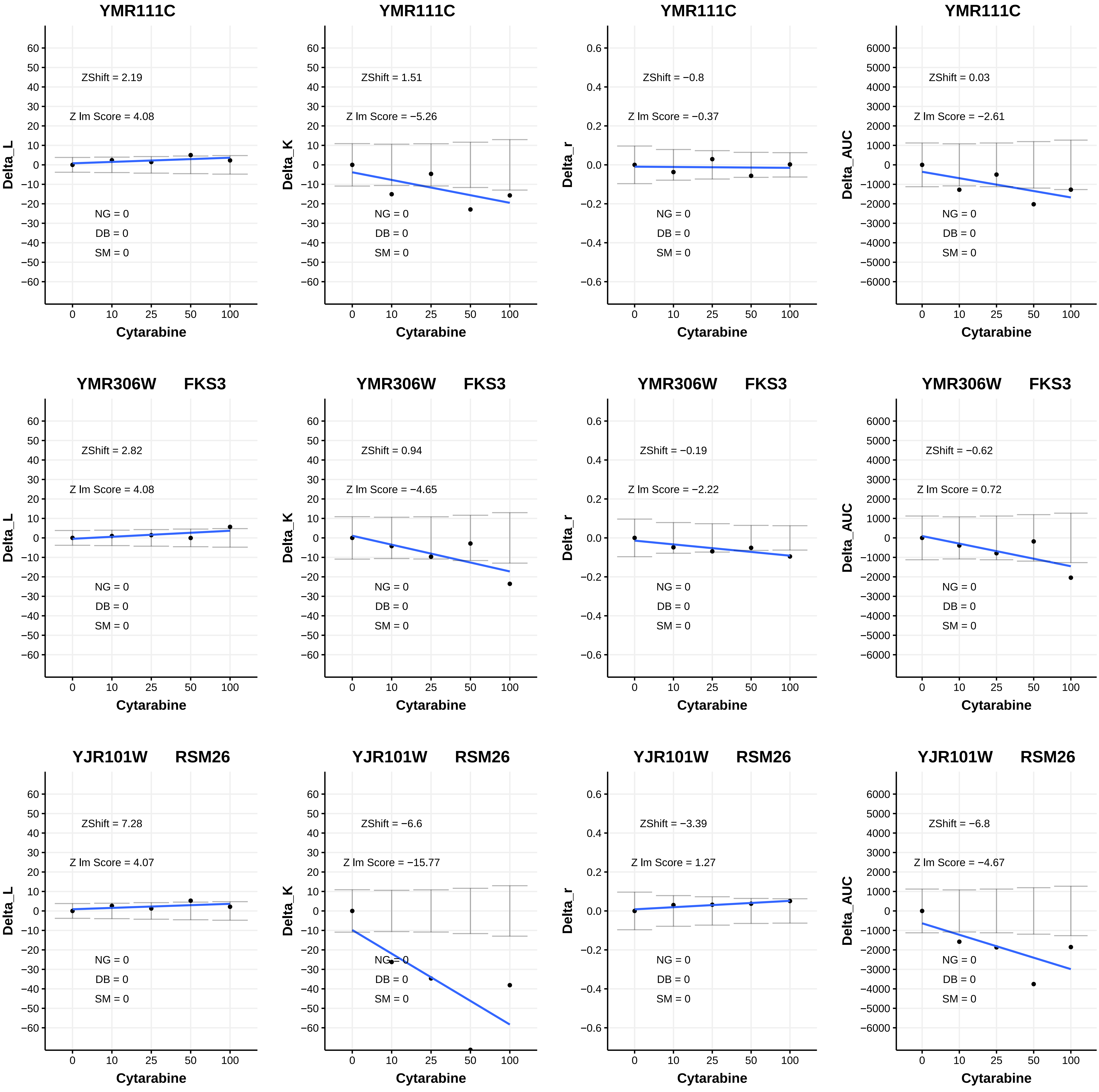
YMR111C
ZShift = 2.19
Z lm Score = 4.08
NG = 0
DB = 0
SM = 0
Delta_L
Cytarabine
YMR111C
ZShift = 1.51
Z lm Score = −5.26
NG = 0
DB = 0
SM = 0
Delta_K
Cytarabine
YMR111C
ZShift = −0.8
Z lm Score = −0.37
NG = 0
DB = 0
SM = 0
Delta_r
Cytarabine
YMR111C
ZShift = 0.03
Z lm Score = −2.61
NG = 0
DB = 0
SM = 0
Delta_AUC
Cytarabine
YMR306W FKS3
ZShift = 2.82
Z lm Score = 4.08
NG = 0
DB = 0
SM = 0
Delta_L
Cytarabine
YMR306W FKS3
ZShift = 0.94
Z lm Score = −4.65
NG = 0
DB = 0
SM = 0
Delta_K
Cytarabine
YMR306W FKS3
ZShift = −0.19
Z lm Score = −2.22
NG = 0
DB = 0
SM = 0
Delta_r
Cytarabine
YMR306W FKS3
ZShift = −0.62
Z lm Score = 0.72
NG = 0
DB = 0
SM = 0
Delta_AUC
Cytarabine
YJR101W RSM26
ZShift = 7.28
Z lm Score = 4.07
NG = 0
DB = 0
SM = 0
Delta_L
Cytarabine
YJR101W RSM26
ZShift = −6.6
Z lm Score = −15.77
NG = 0
DB = 0
SM = 0
Delta_K
Cytarabine
YJR101W RSM26
ZShift = −3.39
Z lm Score = 1.27
NG = 0
DB = 0
SM = 0
Delta_r
Cytarabine
YJR101W RSM26
ZShift = −6.8
Z lm Score = −4.67
NG = 0
DB = 0
SM = 0
Delta_AUC
Cytarabine

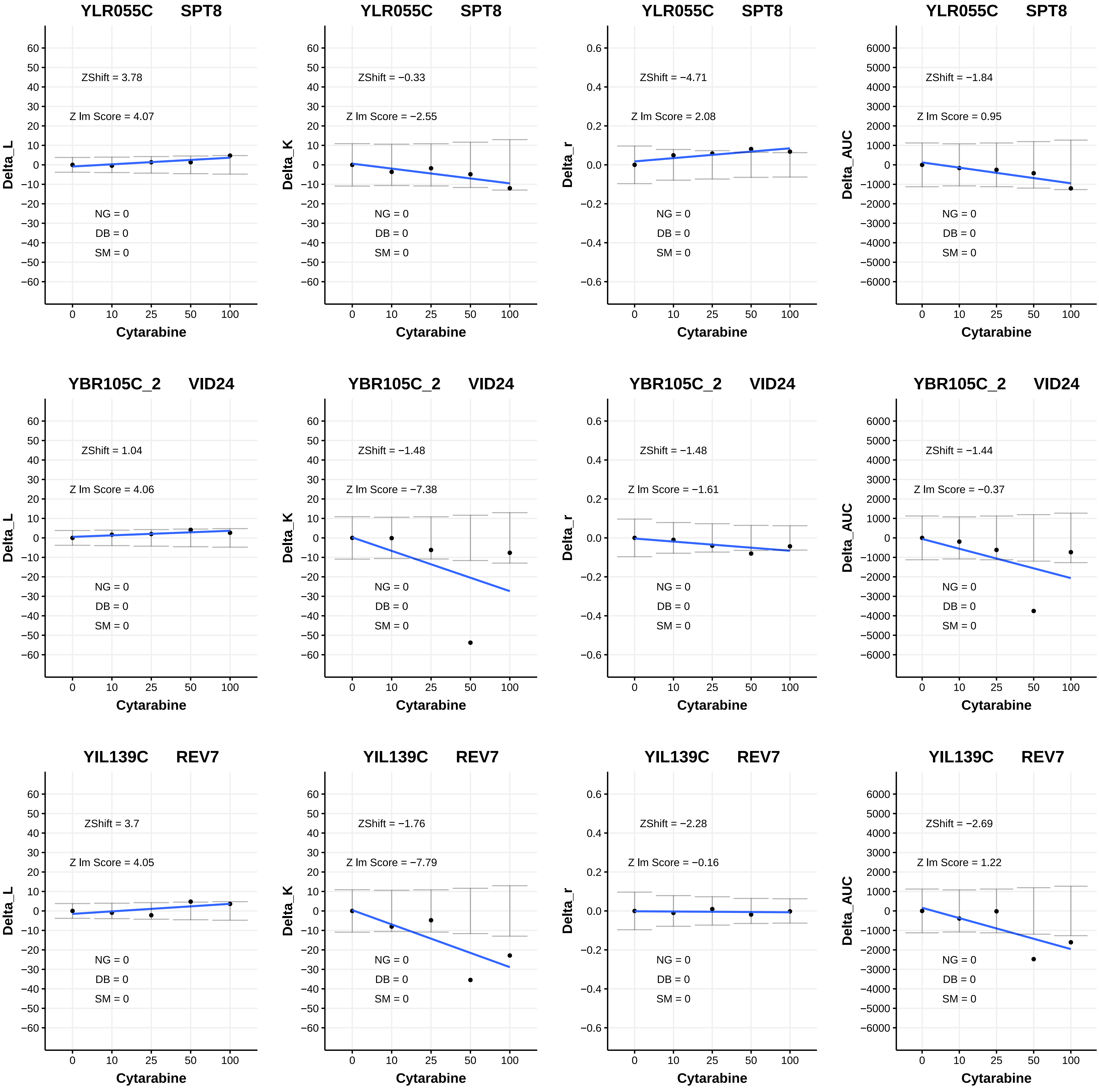
YLR055C SPT8
ZShift = 3.78
Z lm Score = 4.07
NG = 0
DB = 0
SM = 0
Delta_L
Cytarabine
YLR055C SPT8
ZShift = −0.33
Z lm Score = −2.55
NG = 0
DB = 0
SM = 0
Delta_K
Cytarabine
YLR055C SPT8
ZShift = −4.71
Z lm Score = 2.08
NG = 0
DB = 0
SM = 0
Delta_r
Cytarabine
YLR055C SPT8
ZShift = −1.84
Z lm Score = 0.95
NG = 0
DB = 0
SM = 0
Delta_AUC
Cytarabine
YBR105C_2 VID24
ZShift = 1.04
Z lm Score = 4.06
NG = 0
DB = 0
SM = 0
Delta_L
Cytarabine
YBR105C_2 VID24
ZShift = −1.48
Z lm Score = −7.38
NG = 0
DB = 0
SM = 0
Delta_K
Cytarabine
YBR105C_2 VID24
ZShift = −1.48
Z lm Score = −1.61
NG = 0
DB = 0
SM = 0
Delta_r
Cytarabine
YBR105C_2 VID24
ZShift = −1.44
Z lm Score = −0.37
NG = 0
DB = 0
SM = 0
Delta_AUC
Cytarabine
YIL139C REV7
ZShift = 3.7
Z lm Score = 4.05
NG = 0
DB = 0
SM = 0
Delta_L
Cytarabine
YIL139C REV7
ZShift = −1.76
Z lm Score = −7.79
NG = 0
DB = 0
SM = 0
Delta_K
Cytarabine
YIL139C REV7
ZShift = −2.28
Z lm Score = −0.16
NG = 0
DB = 0
SM = 0
Delta_r
Cytarabine
YIL139C REV7
ZShift = −2.69
Z lm Score = 1.22
NG = 0
DB = 0
SM = 0
Delta_AUC
Cytarabine

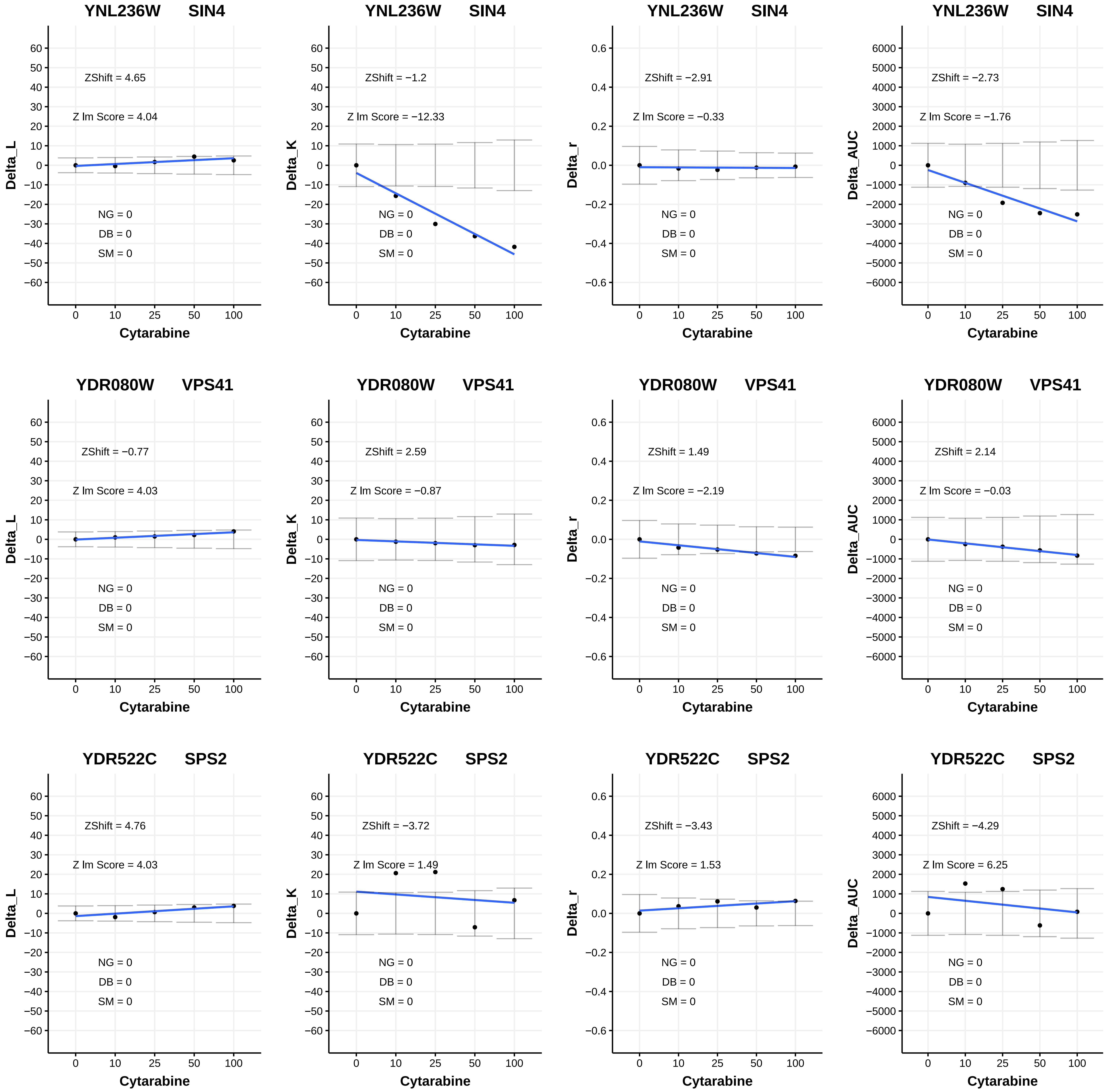

YNL236W SIN4
ZShift = 4.65
Z lm Score = 4.04
NG = 0
DB = 0
SM = 0
Delta_L
Cytarabine
YNL236W SIN4
ZShift = −1.2
Z lm Score = −12.33
NG = 0
DB = 0
SM = 0
Delta_K
Cytarabine
YNL236W SIN4
ZShift = −2.91
Z lm Score = −0.33
NG = 0
DB = 0
SM = 0
Delta_r
Cytarabine
YNL236W SIN4
ZShift = −2.73
Z lm Score = −1.76
NG = 0
DB = 0
SM = 0
Delta_AUC
Cytarabine
YDR080W VPS41
ZShift = −0.77
Z lm Score = 4.03
NG = 0
DB = 0
SM = 0
Delta_L
Cytarabine
YDR080W VPS41
ZShift = 2.59
Z lm Score = −0.87
NG = 0
DB = 0
SM = 0
Delta_K
Cytarabine
YDR080W VPS41
ZShift = 1.49
Z lm Score = −2.19
NG = 0
DB = 0
SM = 0
Delta_r
Cytarabine
YDR080W VPS41
ZShift = 2.14
Z lm Score = −0.03
NG = 0
DB = 0
SM = 0
Delta_AUC
Cytarabine
YDR522C SPS2
ZShift = 4.76
Z lm Score = 4.03
NG = 0
DB = 0
SM = 0
Delta_L
Cytarabine
YDR522C SPS2
ZShift = −3.72
Z lm Score = 1.49
NG = 0
DB = 0
SM = 0
Delta_K
Cytarabine
YDR522C SPS2
ZShift = −3.43
Z lm Score = 1.53
NG = 0
DB = 0
SM = 0
Delta_r
Cytarabine
YDR522C SPS2
ZShift = −4.29
Z lm Score = 6.25
NG = 0
DB = 0
SM = 0
Delta_AUC
Cytarabine

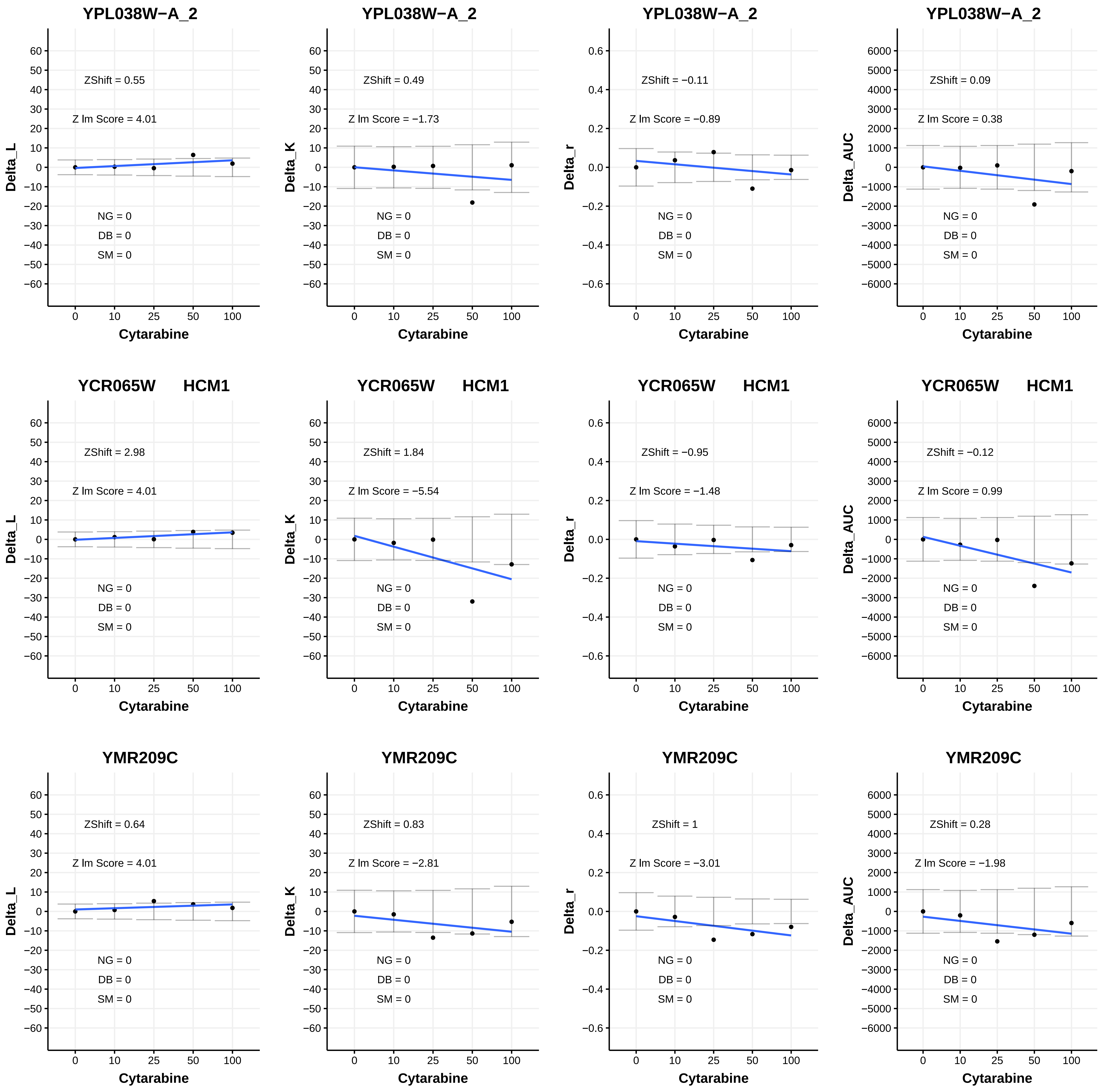
YPL038W−A_2
ZShift = 0.55
Z lm Score = 4.01
NG = 0
DB = 0
SM = 0
Delta_L
Cytarabine
YPL038W−A_2
ZShift = 0.49
Z lm Score = −1.73
NG = 0
DB = 0
SM = 0
Delta_K
Cytarabine
YPL038W−A_2
ZShift = −0.11
Z lm Score = −0.89
NG = 0
DB = 0
SM = 0
Delta_r
Cytarabine
YPL038W−A_2
ZShift = 0.09
Z lm Score = 0.38
NG = 0
DB = 0
SM = 0
Delta_AUC
Cytarabine
YCR065W HCM1
ZShift = 2.98
Z lm Score = 4.01
NG = 0
DB = 0
SM = 0
Delta_L
Cytarabine
YCR065W HCM1
ZShift = 1.84
Z lm Score = −5.54
NG = 0
DB = 0
SM = 0
Delta_K
Cytarabine
YCR065W HCM1
ZShift = −0.95
Z lm Score = −1.48
NG = 0
DB = 0
SM = 0
Delta_r
Cytarabine
YCR065W HCM1
ZShift = −0.12
Z lm Score = 0.99
NG = 0
DB = 0
SM = 0
Delta_AUC
Cytarabine
YMR209C
ZShift = 0.64
Z lm Score = 4.01
NG = 0
DB = 0
SM = 0
Delta_L
Cytarabine
YMR209C
ZShift = 0.83
Z lm Score = −2.81
NG = 0
DB = 0
SM = 0
Delta_K
Cytarabine
YMR209C
ZShift = 1
Z lm Score = −3.01
NG = 0
DB = 0
SM = 0
Delta_r
Cytarabine
YMR209C
ZShift = 0.28
Z lm Score = −1.98
NG = 0
DB = 0
SM = 0
Delta_AUC
Cytarabine

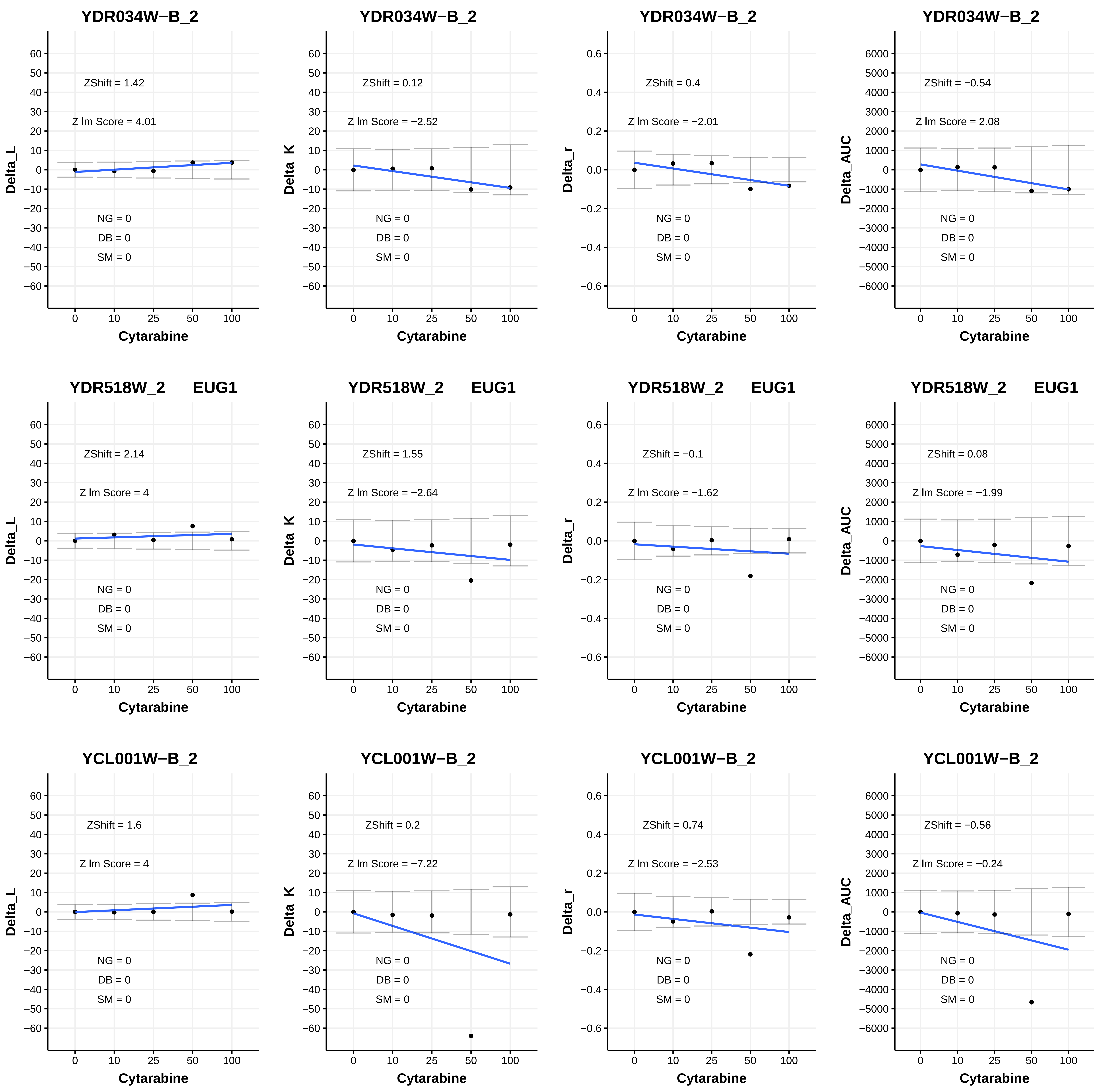
YDR034W−B_2
ZShift = 1.42
Z lm Score = 4.01
NG = 0
DB = 0
SM = 0
Delta_L
Cytarabine
YDR034W−B_2
ZShift = 0.12
Z lm Score = −2.52
NG = 0
DB = 0
SM = 0
Delta_K
Cytarabine
YDR034W−B_2
ZShift = 0.4
Z lm Score = −2.01
NG = 0
DB = 0
SM = 0
Delta_r
Cytarabine
YDR034W−B_2
ZShift = −0.54
Z lm Score = 2.08
NG = 0
DB = 0
SM = 0
Delta_AUC
Cytarabine
YDR518W_2 EUG1
ZShift = 2.14
Z lm Score = 4
NG = 0
DB = 0
SM = 0
Delta_L
Cytarabine
YDR518W_2 EUG1
ZShift = 1.55
Z lm Score = −2.64
NG = 0
DB = 0
SM = 0
Delta_K
Cytarabine
YDR518W_2 EUG1
ZShift = −0.1
Z lm Score = −1.62
NG = 0
DB = 0
SM = 0
Delta_r
Cytarabine
YDR518W_2 EUG1
ZShift = 0.08
Z lm Score = −1.99
NG = 0
DB = 0
SM = 0
Delta_AUC
Cytarabine
YCL001W−B_2
ZShift = 1.6
Z lm Score = 4
NG = 0
DB = 0
SM = 0
Delta_L
Cytarabine
YCL001W−B_2
ZShift = 0.2
Z lm Score = −7.22
NG = 0
DB = 0
SM = 0
Delta_K
Cytarabine
YCL001W−B_2
ZShift = 0.74
Z lm Score = −2.53
NG = 0
DB = 0
SM = 0
Delta_r
Cytarabine
YCL001W−B_2
ZShift = −0.56
Z lm Score = −0.24
NG = 0
DB = 0
SM = 0
Delta_AUC
Cytarabine

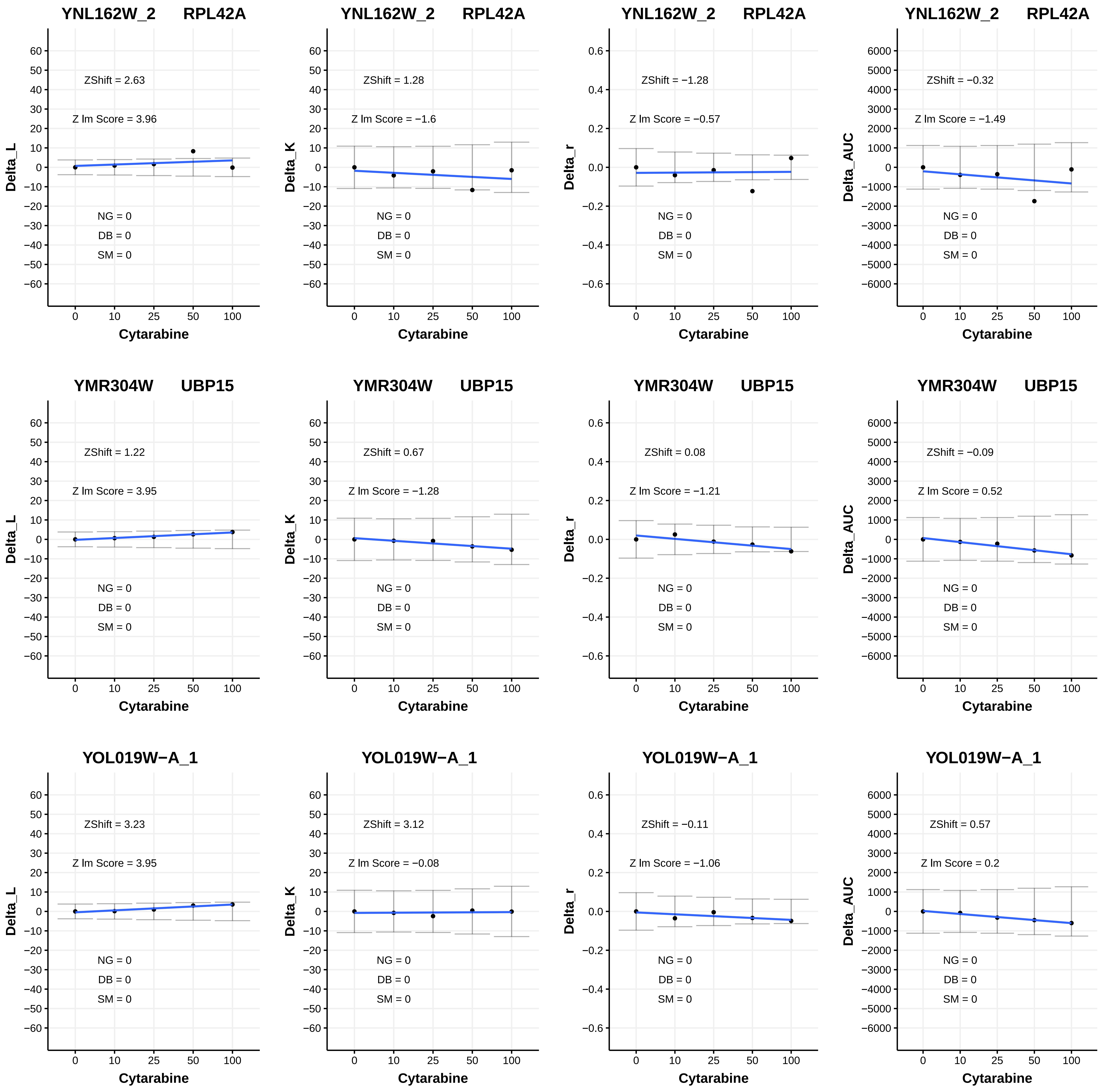
YNL162W_2 RPL42A
ZShift = 2.63
Z lm Score = 3.96
NG = 0
DB = 0
SM = 0
Delta_L
Cytarabine
0 10 25 50 100
YNL162W_2 RPL42A
ZShift = 1.28
Z lm Score = −1.6
NG = 0
DB = 0
SM = 0
Delta_K
Cytarabine
YNL162W_2 RPL42A
ZShift = −1.28
Z lm Score = −0.57
NG = 0
DB = 0
SM = 0
Delta_r
Cytarabine
YNL162W_2 RPL42A
ZShift = −0.32
Z lm Score = −1.49
NG = 0
DB = 0
SM = 0
Delta_AUC
Cytarabine
YMR304W UBP15
ZShift = 1.22
Z lm Score = 3.95
NG = 0
DB = 0
SM = 0
Delta_L
Cytarabine
YMR304W UBP15
ZShift = 0.67
Z lm Score = −1.28
NG = 0
DB = 0
SM = 0
Delta_K
Cytarabine
YMR304W UBP15
ZShift = 0.08
Z lm Score = −1.21
NG = 0
DB = 0
SM = 0
Delta_r
Cytarabine
YMR304W UBP15
ZShift = −0.09
Z lm Score = 0.52
NG = 0
DB = 0
SM = 0
Delta_AUC
Cytarabine
YOL019W−A_1
ZShift = 3.23
Z lm Score = 3.95
NG = 0
DB = 0
SM = 0
Delta_L
Cytarabine
YOL019W−A_1
ZShift = 3.12
Z lm Score = −0.08
NG = 0
DB = 0
SM = 0
Delta_K
Cytarabine
YOL019W−A_1
ZShift = −0.11
Z lm Score = −1.06
NG = 0
DB = 0
SM = 0
Delta_r
Cytarabine
YOL019W−A_1
ZShift = 0.57
Z lm Score = 0.2
NG = 0
DB = 0
SM = 0
Delta_AUC
Cytarabine

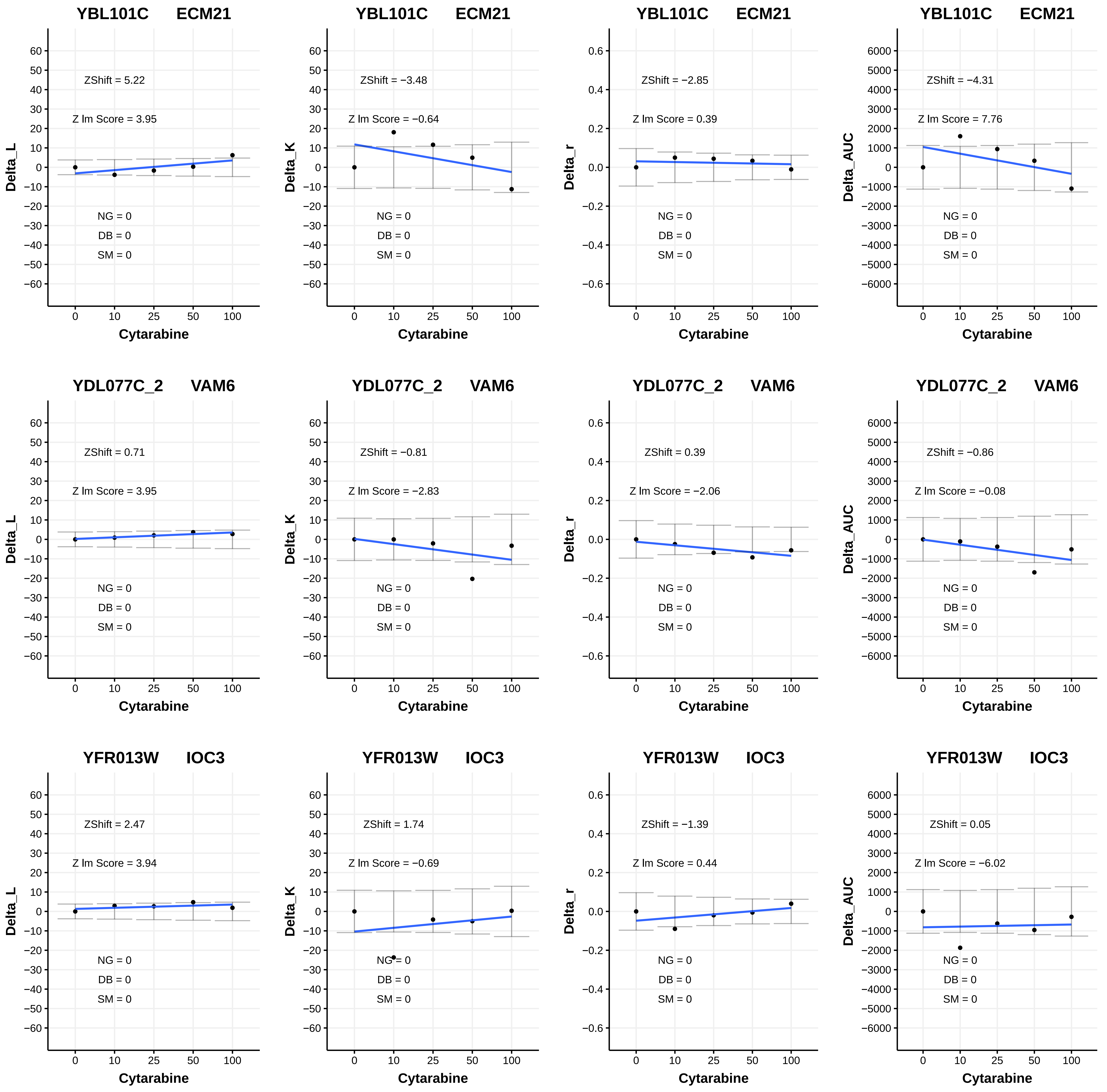

YBL101C ECM21
ZShift = 5.22
Z lm Score = 3.95
NG = 0
DB = 0
SM = 0
Delta_L
Cytarabine
YBL101C ECM21
ZShift = −3.48
Z lm Score = −0.64
NG = 0
DB = 0
SM = 0
Delta_K
Cytarabine
YBL101C ECM21
ZShift = −2.85
Z lm Score = 0.39
NG = 0
DB = 0
SM = 0
Delta_r
Cytarabine
YBL101C ECM21
ZShift = −4.31
Z lm Score = 7.76
NG = 0
DB = 0
SM = 0
Delta_AUC
Cytarabine
YDL077C_2 VAM6
ZShift = 0.71
Z lm Score = 3.95
NG = 0
DB = 0
SM = 0
Delta_L
Cytarabine
YDL077C_2 VAM6
ZShift = −0.81
Z lm Score = −2.83
NG = 0
DB = 0
SM = 0
Delta_K
Cytarabine
YDL077C_2 VAM6
ZShift = 0.39
Z lm Score = −2.06
NG = 0
DB = 0
SM = 0
Delta_r
Cytarabine
YDL077C_2 VAM6
ZShift = −0.86
Z lm Score = −0.08
NG = 0
DB = 0
SM = 0
Delta_AUC
Cytarabine
YFR013W IOC3
ZShift = 2.47
Z lm Score = 3.94
NG = 0
DB = 0
SM = 0
Delta_L
Cytarabine
YFR013W IOC3
ZShift = 1.74
Z lm Score = −0.69
NG = 0
DB = 0
SM = 0
Delta_K
Cytarabine
YFR013W IOC3
ZShift = −1.39
Z lm Score = 0.44
NG = 0
DB = 0
SM = 0
Delta_r
Cytarabine
YFR013W IOC3
ZShift = 0.05
Z lm Score = −6.02
NG = 0
DB = 0
SM = 0
Delta_AUC
Cytarabine

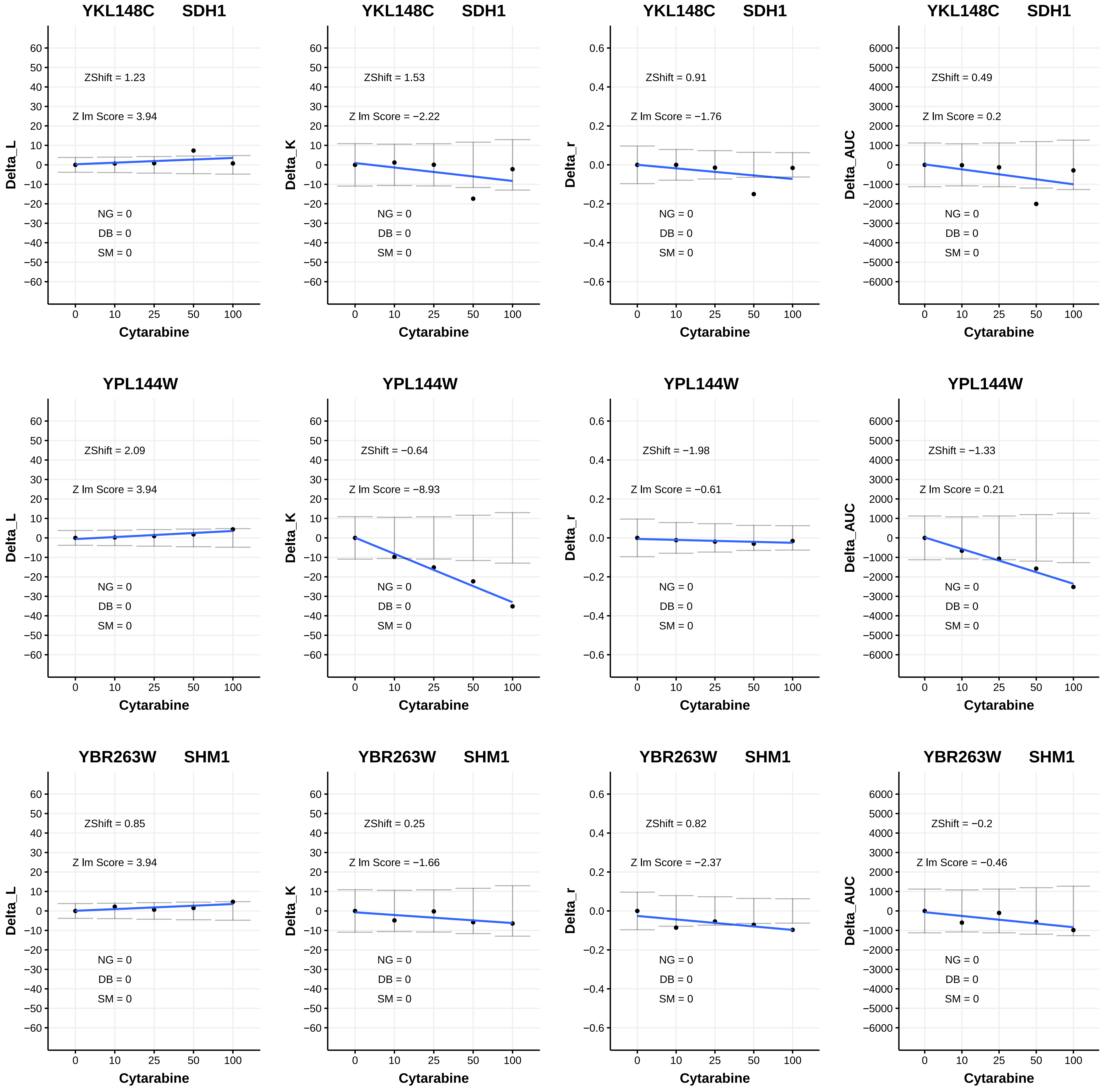
YKL148C SDH1
ZShift = 1.23
Z lm Score = 3.94
NG = 0
DB = 0
SM = 0
Delta_L
Cytarabine
YKL148C SDH1
ZShift = 1.53
Z lm Score = −2.22
NG = 0
DB = 0
SM = 0
Delta_K
Cytarabine
YKL148C SDH1
ZShift = 0.91
Z lm Score = −1.76
NG = 0
DB = 0
SM = 0
Delta_r
Cytarabine
YKL148C SDH1
ZShift = 0.49
Z lm Score = 0.2
NG = 0
DB = 0
SM = 0
Delta_AUC
Cytarabine
YPL144W
ZShift = 2.09
Z lm Score = 3.94
NG = 0
DB = 0
SM = 0
Delta_L
Cytarabine
YPL144W
ZShift = −0.64
Z lm Score = −8.93
NG = 0
DB = 0
SM = 0
Delta_K
Cytarabine
YPL144W
ZShift = −1.98
Z lm Score = −0.61
NG = 0
DB = 0
SM = 0
Delta_r
Cytarabine
YPL144W
ZShift = −1.33
Z lm Score = 0.21
NG = 0
DB = 0
SM = 0
Delta_AUC
Cytarabine
YBR263W SHM1
ZShift = 0.85
Z lm Score = 3.94
NG = 0
DB = 0
SM = 0
Delta_L
Cytarabine
YBR263W SHM1
ZShift = 0.25
Z lm Score = −1.66
NG = 0
DB = 0
SM = 0
Delta_K
Cytarabine
YBR263W SHM1
ZShift = 0.82
Z lm Score = −2.37
NG = 0
DB = 0
SM = 0
Delta_r
Cytarabine
YBR263W SHM1
ZShift = −0.2
Z lm Score = −0.46
NG = 0
DB = 0
SM = 0
Delta_AUC
Cytarabine

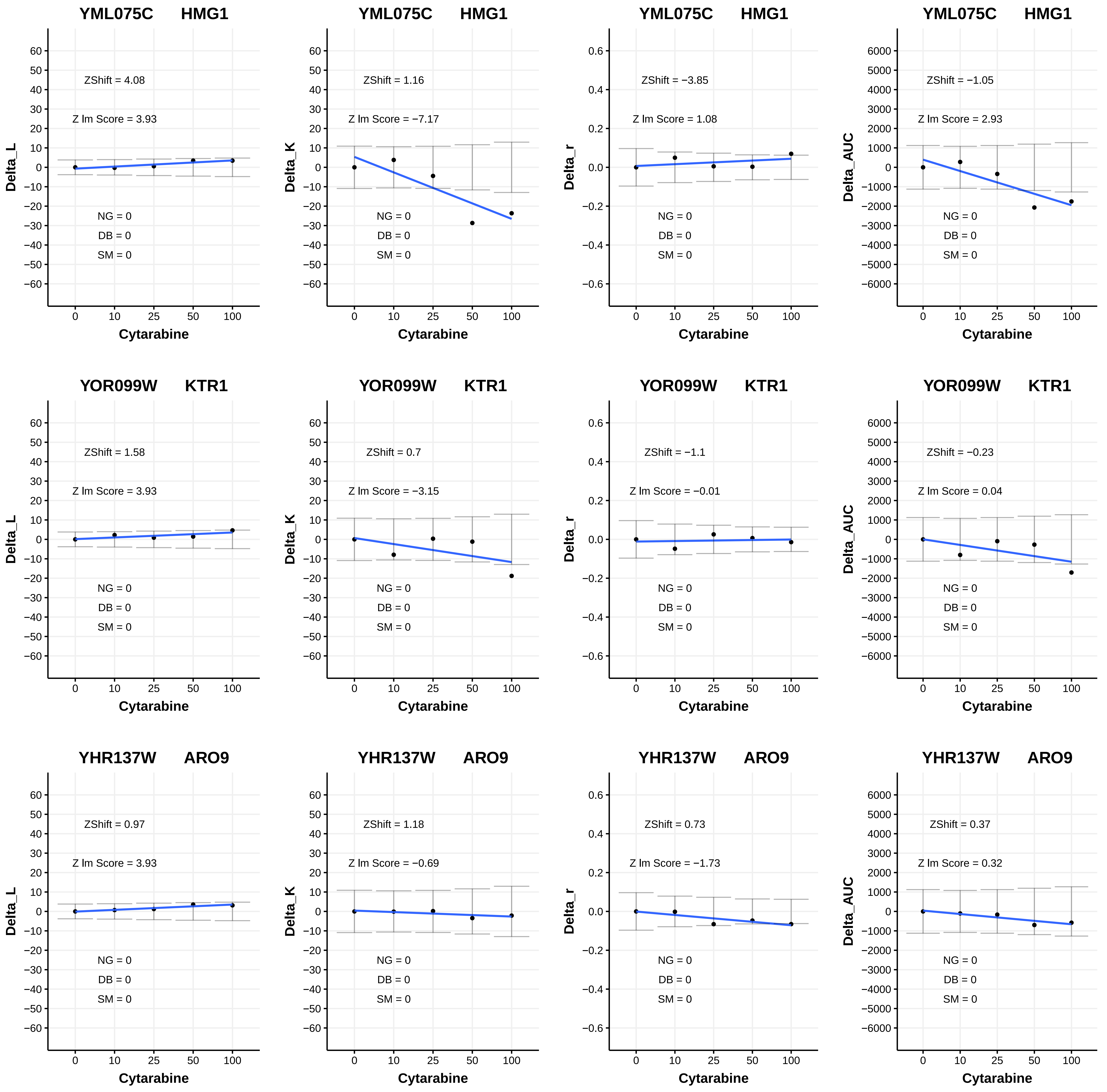

YML075C HMG1
ZShift = 4.08
Z lm Score = 3.93
NG = 0
DB = 0
SM = 0
Delta_L
Cytarabine
YML075C HMG1
ZShift = 1.16
Z lm Score = −7.17
NG = 0
DB = 0
SM = 0
Delta_K
Cytarabine
YML075C HMG1
ZShift = −3.85
Z lm Score = 1.08
NG = 0
DB = 0
SM = 0
Delta_r
Cytarabine
YML075C HMG1
ZShift = −1.05
Z lm Score = 2.93
NG = 0
DB = 0
SM = 0
Delta_AUC
Cytarabine
YOR099W KTR1
ZShift = 1.58
Z lm Score = 3.93
NG = 0
DB = 0
SM = 0
Delta_L
Cytarabine
YOR099W KTR1
ZShift = 0.7
Z lm Score = −3.15
NG = 0
DB = 0
SM = 0
Delta_K
Cytarabine
YOR099W KTR1
ZShift = −1.1
Z lm Score = −0.01
NG = 0
DB = 0
SM = 0
Delta_r
Cytarabine
YOR099W KTR1
ZShift = −0.23
Z lm Score = 0.04
NG = 0
DB = 0
SM = 0
Delta_AUC
Cytarabine
YHR137W ARO9
ZShift = 0.97
Z lm Score = 3.93
NG = 0
DB = 0
SM = 0
Delta_L
Cytarabine
YHR137W ARO9
ZShift = 1.18
Z lm Score = −0.69
NG = 0
DB = 0
SM = 0
Delta_K
Cytarabine
YHR137W ARO9
ZShift = 0.73
Z lm Score = −1.73
NG = 0
DB = 0
SM = 0
Delta_r
Cytarabine
YHR137W ARO9
ZShift = 0.37
Z lm Score = 0.32
NG = 0
DB = 0
SM = 0
Delta_AUC
Cytarabine

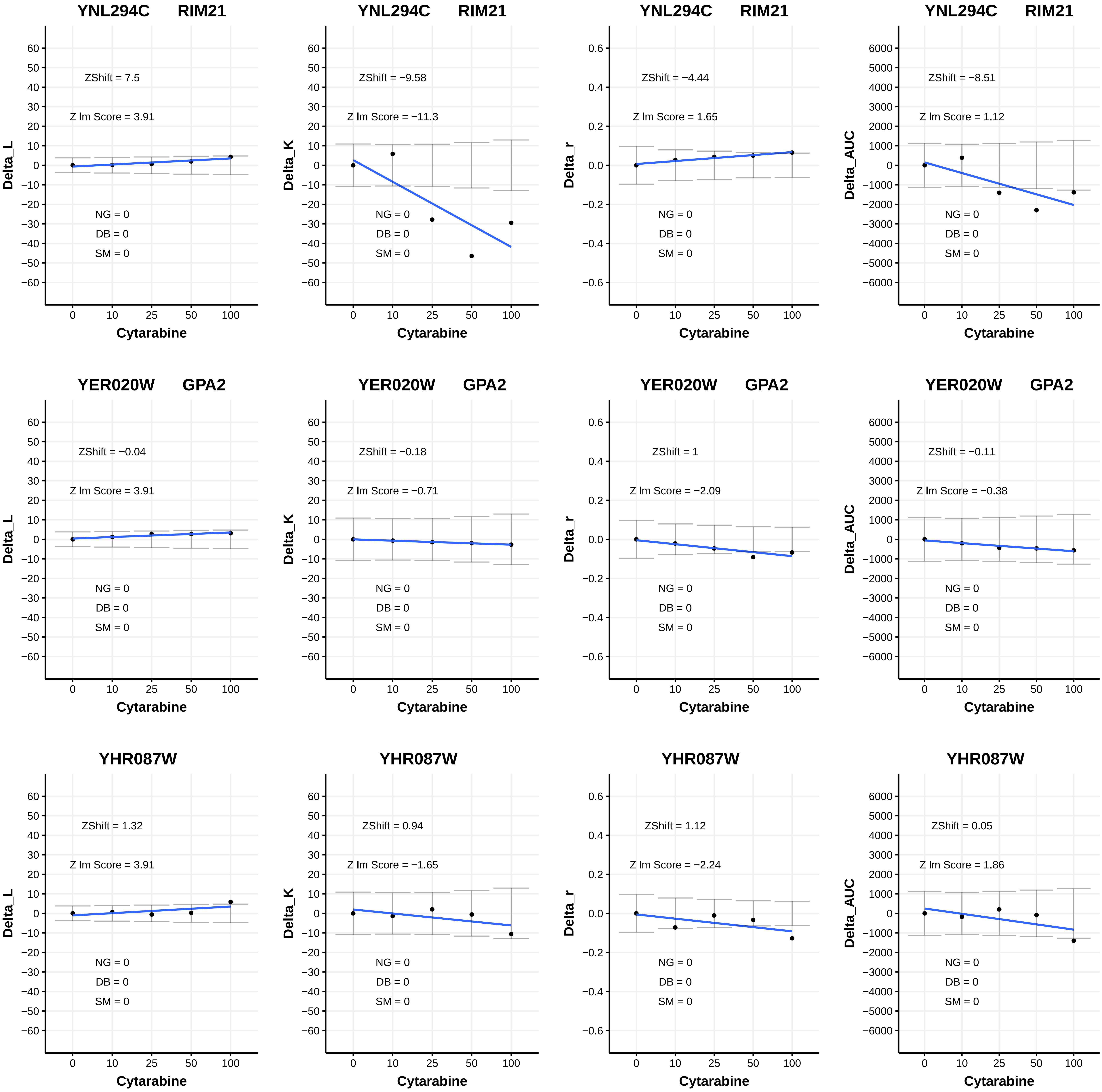

YNL294C RIM21
ZShift = 7.5
Z lm Score = 3.91
NG = 0
DB = 0
SM = 0
Delta_L
Cytarabine
YNL294C RIM21
ZShift = −9.58
Z lm Score = −11.3
NG = 0
DB = 0
SM = 0
Delta_K
Cytarabine
YNL294C RIM21
ZShift = −4.44
Z lm Score = 1.65
NG = 0
DB = 0
SM = 0
Delta_r
Cytarabine
YNL294C RIM21
ZShift = −8.51
Z lm Score = 1.12
NG = 0
DB = 0
SM = 0
Delta_AUC
Cytarabine
YER020W GPA2
ZShift = −0.04
Z lm Score = 3.91
NG = 0
DB = 0
SM = 0
Delta_L
Cytarabine
YER020W GPA2
ZShift = −0.18
Z lm Score = −0.71
NG = 0
DB = 0
SM = 0
Delta_K
Cytarabine
YER020W GPA2
ZShift = 1
Z lm Score = −2.09
NG = 0
DB = 0
SM = 0
Delta_r
Cytarabine
YER020W GPA2
ZShift = −0.11
Z lm Score = −0.38
NG = 0
DB = 0
SM = 0
Delta_AUC
Cytarabine
YHR087W
ZShift = 1.32
Z lm Score = 3.91
NG = 0
DB = 0
SM = 0
Delta_L
Cytarabine
YHR087W
ZShift = 0.94
Z lm Score = −1.65
NG = 0
DB = 0
SM = 0
Delta_K
Cytarabine
YHR087W
ZShift = 1.12
Z lm Score = −2.24
NG = 0
DB = 0
SM = 0
Delta_r
Cytarabine
YHR087W
ZShift = 0.05
Z lm Score = 1.86
NG = 0
DB = 0
SM = 0
Delta_AUC
Cytarabine

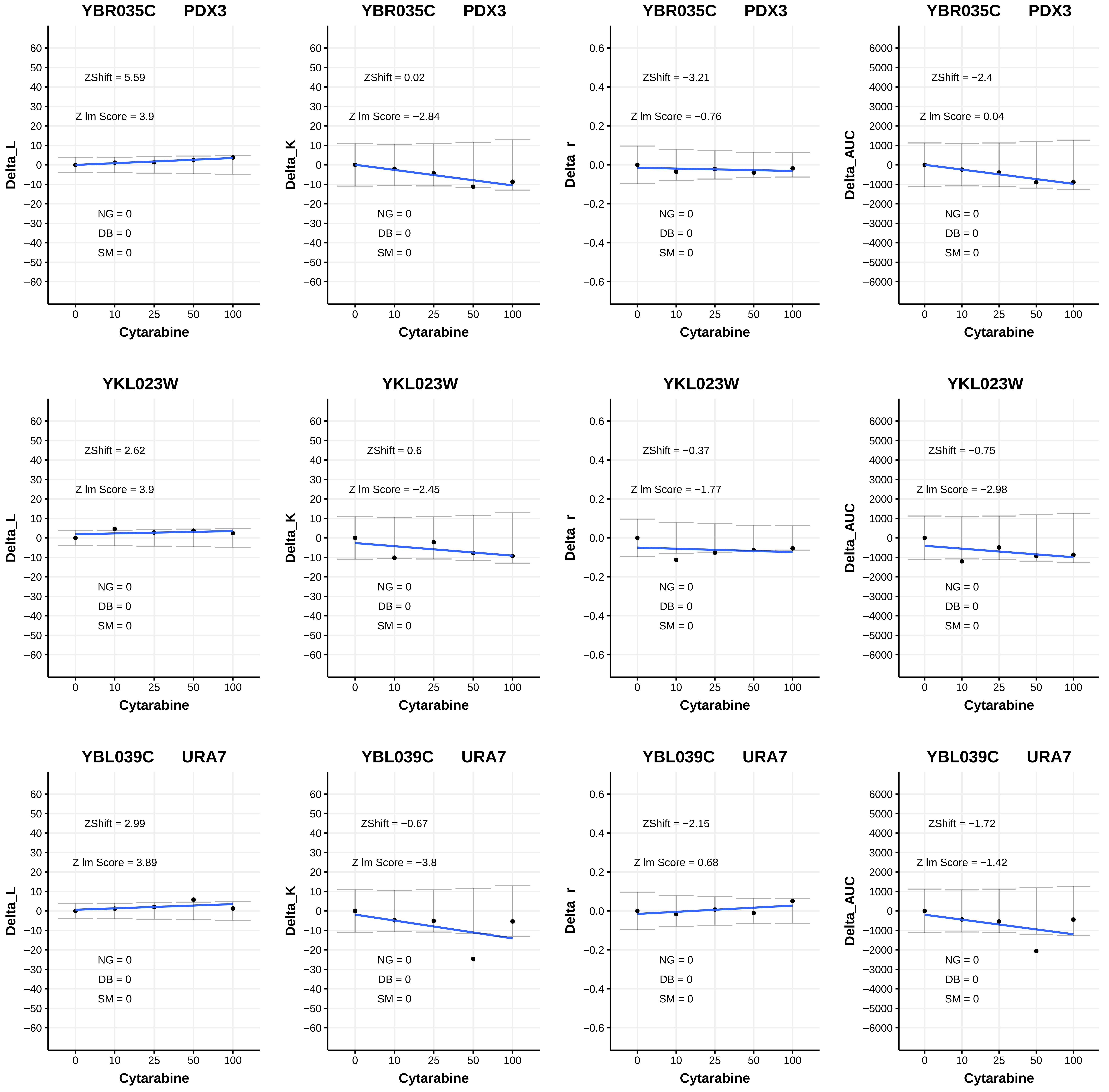

YBR035C PDX3
ZShift = 5.59
Z lm Score = 3.9
NG = 0
DB = 0
SM = 0
Delta_L
Cytarabine
YBR035C PDX3
ZShift = 0.02
Z lm Score = −2.84
NG = 0
DB = 0
SM = 0
Delta_K
Cytarabine
YBR035C PDX3
ZShift = −3.21
Z lm Score = −0.76
NG = 0
DB = 0
SM = 0
Delta_r
Cytarabine
YBR035C PDX3
ZShift = −2.4
Z lm Score = 0.04
NG = 0
DB = 0
SM = 0
Delta_AUC
Cytarabine
YKL023W
ZShift = 2.62
Z lm Score = 3.9
NG = 0
DB = 0
SM = 0
Delta_L
Cytarabine
YKL023W
ZShift = 0.6
Z lm Score = −2.45
NG = 0
DB = 0
SM = 0
Delta_K
Cytarabine
YKL023W
ZShift = −0.37
Z lm Score = −1.77
NG = 0
DB = 0
SM = 0
Delta_r
Cytarabine
YKL023W
ZShift = −0.75
Z lm Score = −2.98
NG = 0
DB = 0
SM = 0
Delta_AUC
Cytarabine
YBL039C URA7
ZShift = 2.99
Z lm Score = 3.89
NG = 0
DB = 0
SM = 0
Delta_L
Cytarabine
YBL039C URA7
ZShift = −0.67
Z lm Score = −3.8
NG = 0
DB = 0
SM = 0
Delta_K
Cytarabine
YBL039C URA7
ZShift = −2.15
Z lm Score = 0.68
NG = 0
DB = 0
SM = 0
Delta_r
Cytarabine
YBL039C URA7
ZShift = −1.72
Z lm Score = −1.42
NG = 0
DB = 0
SM = 0
Delta_AUC
Cytarabine

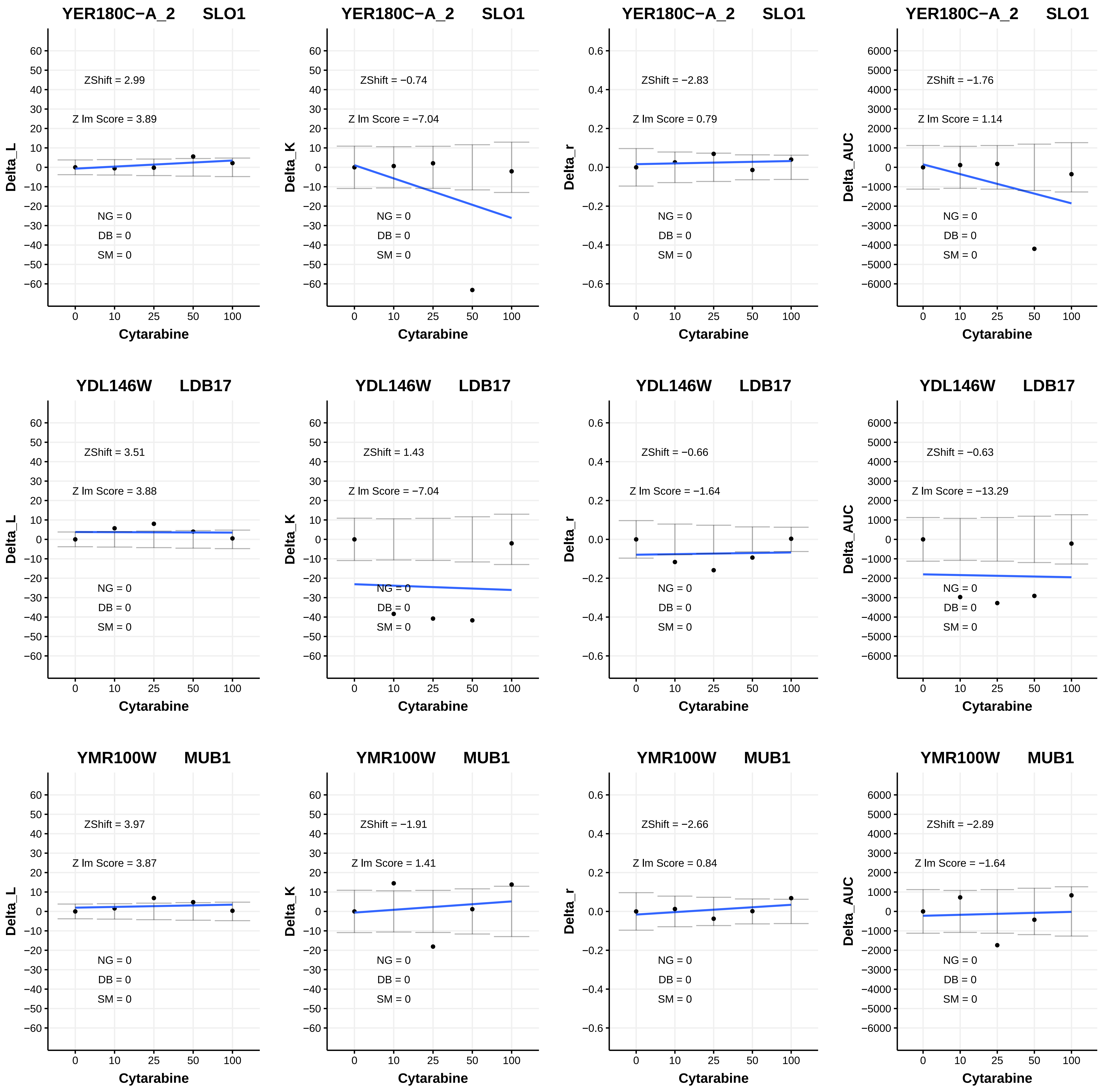

YER180C−A_2 SLO1
ZShift = 2.99
Z lm Score = 3.89
NG = 0
DB = 0
SM = 0
Delta_L
Cytarabine
YER180C−A_2 SLO1
ZShift = −0.74
Z lm Score = −7.04
NG = 0
DB = 0
SM = 0
Delta_K
Cytarabine
YER180C−A_2 SLO1
ZShift = −2.83
Z lm Score = 0.79
NG = 0
DB = 0
SM = 0
Delta_r
Cytarabine
YER180C−A_2 SLO1
ZShift = −1.76
Z lm Score = 1.14
NG = 0
DB = 0
SM = 0
Delta_AUC
Cytarabine
YDL146W LDB17
ZShift = 3.51
Z lm Score = 3.88
NG = 0
DB = 0
SM = 0
Delta_L
Cytarabine
YDL146W LDB17
ZShift = 1.43
Z lm Score = −7.04
NG = 0
DB = 0
SM = 0
Delta_K
Cytarabine
YDL146W LDB17
ZShift = −0.66
Z lm Score = −1.64
NG = 0
DB = 0
SM = 0
Delta_r
Cytarabine
YDL146W LDB17
ZShift = −0.63
Z lm Score = −13.29
NG = 0
DB = 0
SM = 0
Delta_AUC
Cytarabine
YMR100W MUB1
ZShift = 3.97
Z lm Score = 3.87
NG = 0
DB = 0
SM = 0
Delta_L
Cytarabine
YMR100W MUB1
ZShift = −1.91
Z lm Score = 1.41
NG = 0
DB = 0
SM = 0
Delta_K
Cytarabine
YMR100W MUB1
ZShift = −2.66
Z lm Score = 0.84
NG = 0
DB = 0
SM = 0
Delta_r
Cytarabine
YMR100W MUB1
ZShift = −2.89
Z lm Score = −1.64
NG = 0
DB = 0
SM = 0
Delta_AUC
Cytarabine

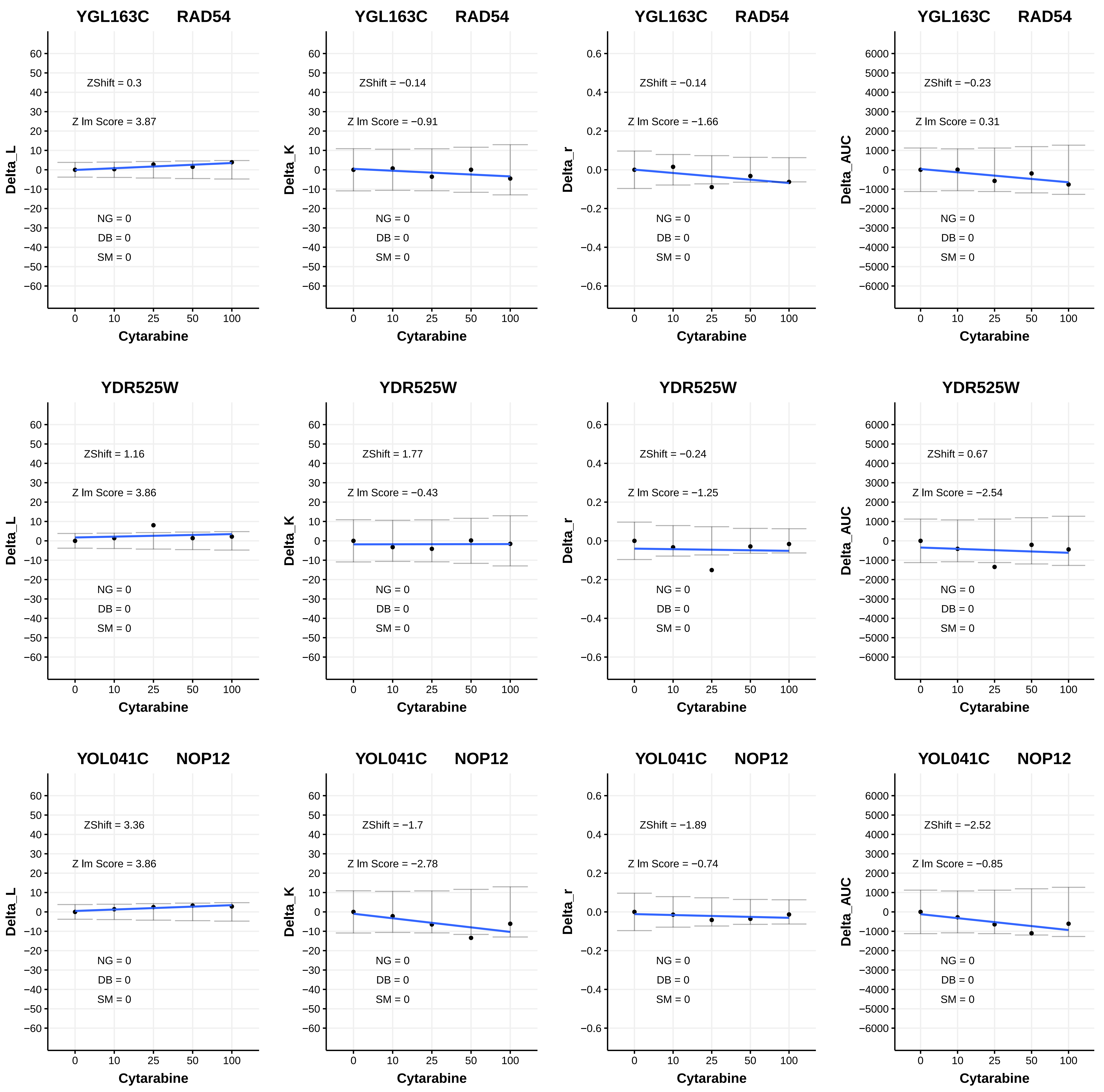

YGL163C RAD54
ZShift = 0.3
Z lm Score = 3.87
NG = 0
DB = 0
SM = 0
Delta_L
Cytarabine
YGL163C RAD54
ZShift = −0.14
Z lm Score = −0.91
NG = 0
DB = 0
SM = 0
Delta_K
Cytarabine
YGL163C RAD54
ZShift = −0.14
Z lm Score = −1.66
NG = 0
DB = 0
SM = 0
Delta_r
Cytarabine
YGL163C RAD54
ZShift = −0.23
Z lm Score = 0.31
NG = 0
DB = 0
SM = 0
Delta_AUC
Cytarabine
YDR525W
ZShift = 1.16
Z lm Score = 3.86
NG = 0
DB = 0
SM = 0
Delta_L
Cytarabine
YDR525W
ZShift = 1.77
Z lm Score = −0.43
NG = 0
DB = 0
SM = 0
Delta_K
Cytarabine
YDR525W
ZShift = −0.24
Z lm Score = −1.25
NG = 0
DB = 0
SM = 0
Delta_r
Cytarabine
YDR525W
ZShift = 0.67
Z lm Score = −2.54
NG = 0
DB = 0
SM = 0
Delta_AUC
Cytarabine
YOL041C NOP12
ZShift = 3.36
Z lm Score = 3.86
NG = 0
DB = 0
SM = 0
Delta_L
Cytarabine
YOL041C NOP12
ZShift = −1.7
Z lm Score = −2.78
NG = 0
DB = 0
SM = 0
Delta_K
Cytarabine
YOL041C NOP12
ZShift = −1.89
Z lm Score = −0.74
NG = 0
DB = 0
SM = 0
Delta_r
Cytarabine
YOL041C NOP12
ZShift = −2.52
Z lm Score = −0.85
NG = 0
DB = 0
SM = 0
Delta_AUC
Cytarabine

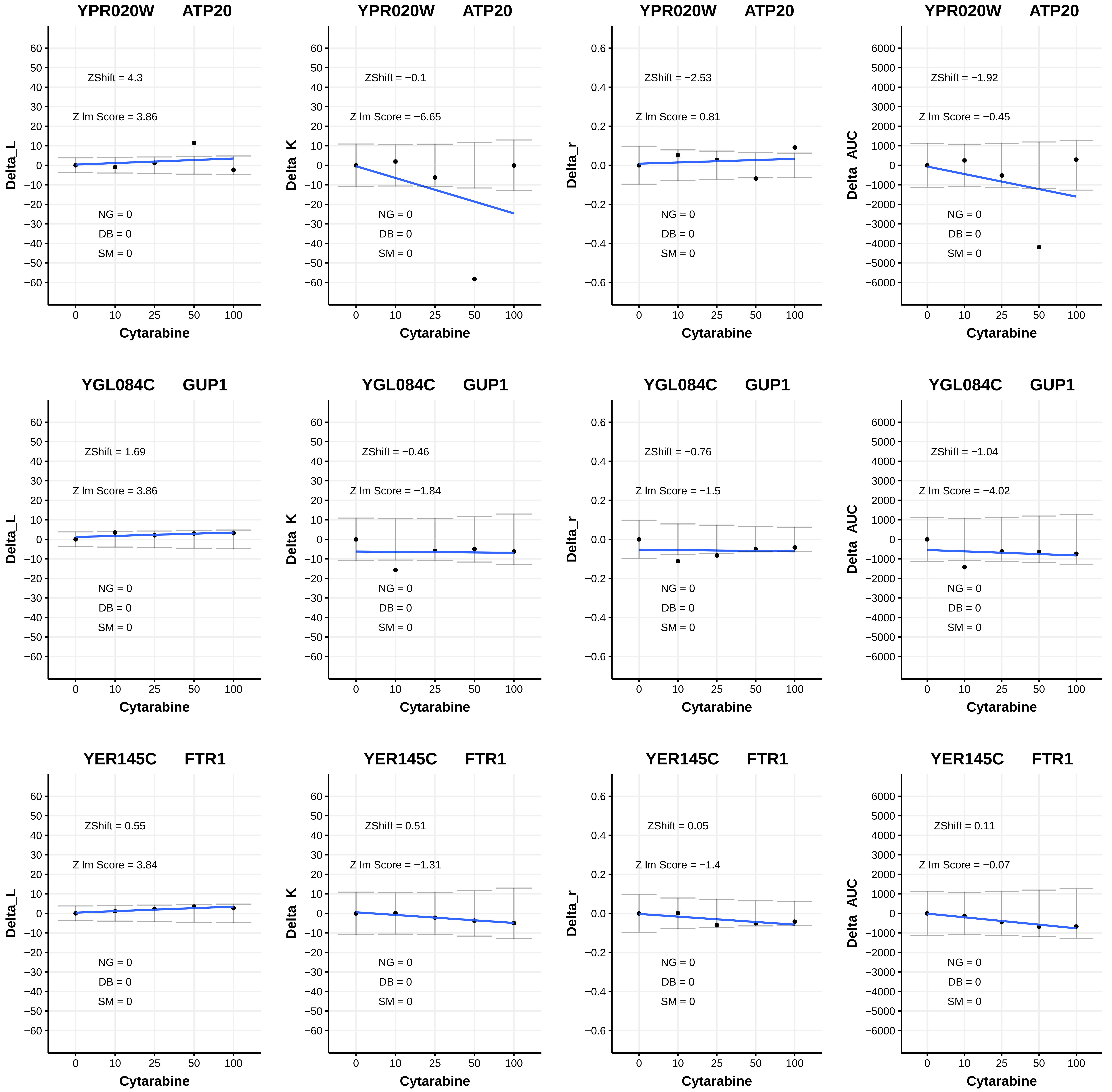

YPR020W ATP20
ZShift = 4.3
Z lm Score = 3.86
NG = 0
DB = 0
SM = 0
Delta_L
Cytarabine
YPR020W ATP20
ZShift = −0.1
Z lm Score = −6.65
NG = 0
DB = 0
SM = 0
Delta_K
Cytarabine
YPR020W ATP20
ZShift = −2.53
Z lm Score = 0.81
NG = 0
DB = 0
SM = 0
Delta_r
Cytarabine
YPR020W ATP20
ZShift = −1.92
Z lm Score = −0.45
NG = 0
DB = 0
SM = 0
Delta_AUC
Cytarabine
YGL084C GUP1
ZShift = 1.69
Z lm Score = 3.86
NG = 0
DB = 0
SM = 0
Delta_L
Cytarabine
YGL084C GUP1
ZShift = −0.46
Z lm Score = −1.84
NG = 0
DB = 0
SM = 0
Delta_K
Cytarabine
YGL084C GUP1
ZShift = −0.76
Z lm Score = −1.5
NG = 0
DB = 0
SM = 0
Delta_r
Cytarabine
YGL084C GUP1
ZShift = −1.04
Z lm Score = −4.02
NG = 0
DB = 0
SM = 0
Delta_AUC
Cytarabine
YER145C FTR1
ZShift = 0.55
Z lm Score = 3.84
NG = 0
DB = 0
SM = 0
Delta_L
Cytarabine
YER145C FTR1
ZShift = 0.51
Z lm Score = −1.31
NG = 0
DB = 0
SM = 0
Delta_K
Cytarabine
YER145C FTR1
ZShift = 0.05
Z lm Score = −1.4
NG = 0
DB = 0
SM = 0
Delta_r
Cytarabine
YER145C FTR1
ZShift = 0.11
Z lm Score = −0.07
NG = 0
DB = 0
SM = 0
Delta_AUC
Cytarabine

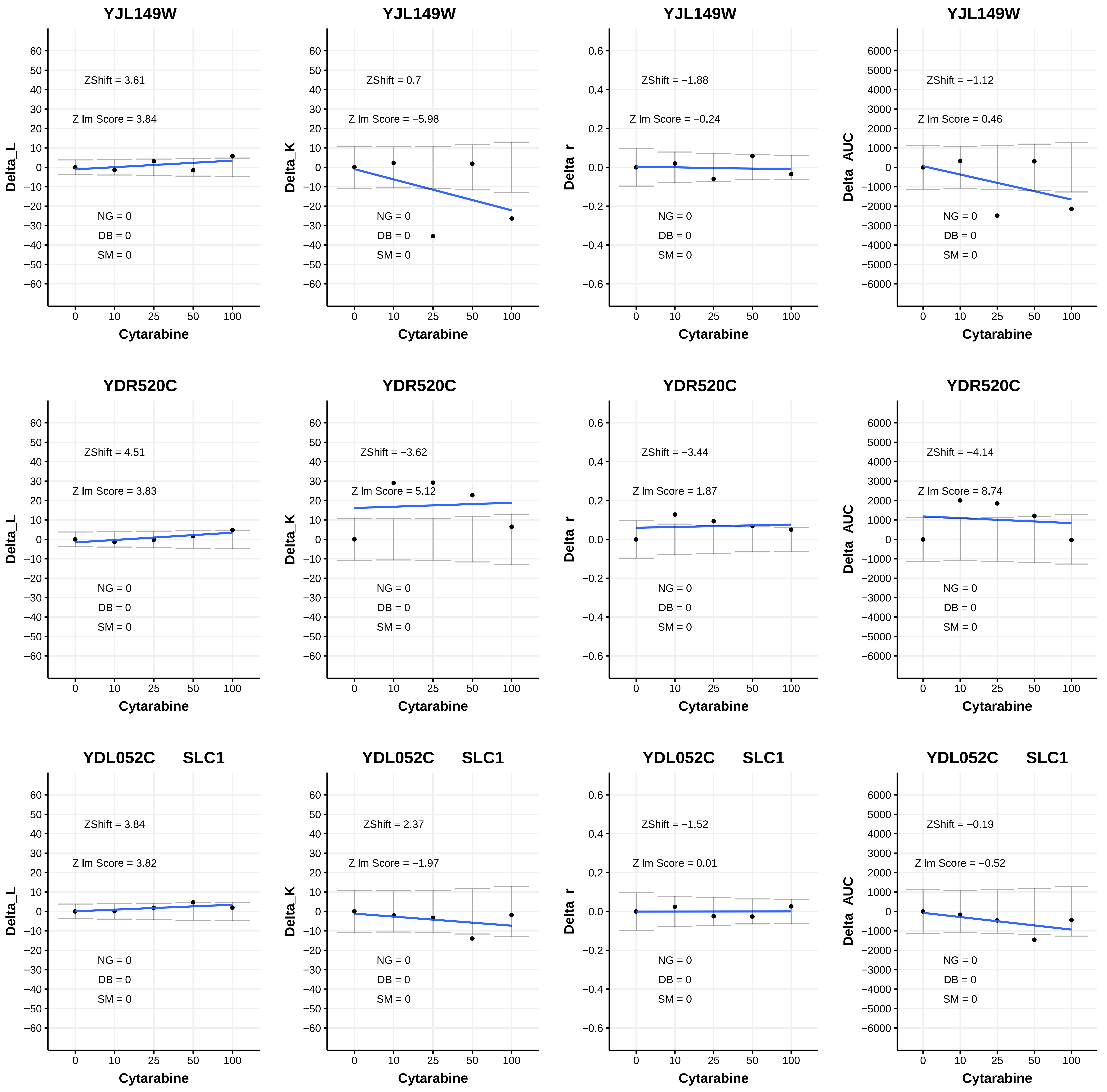

YJL149W
ZShift = 3.61
Z lm Score = 3.84
NG = 0
DB = 0
SM = 0
Delta_L
Cytarabine
YJL149W
ZShift = 0.7
Z lm Score = −5.98
NG = 0
DB = 0
SM = 0
Delta_K
Cytarabine
YJL149W
ZShift = −1.88
Z lm Score = −0.24
NG = 0
DB = 0
SM = 0
Delta_r
Cytarabine
YJL149W
ZShift = −1.12
Z lm Score = 0.46
NG = 0
DB = 0
SM = 0
Delta_AUC
Cytarabine
YDR520C
ZShift = 4.51
Z lm Score = 3.83
NG = 0
DB = 0
SM = 0
Delta_L
Cytarabine
YDR520C
ZShift = −3.62
Z lm Score = 5.12
NG = 0
DB = 0
SM = 0
Delta_K
Cytarabine
YDR520C
ZShift = −3.44
Z lm Score = 1.87
NG = 0
DB = 0
SM = 0
Delta_r
Cytarabine
YDR520C
ZShift = −4.14
Z lm Score = 8.74
NG = 0
DB = 0
SM = 0
Delta_AUC
Cytarabine
YDL052C SLC1
ZShift = 3.84
Z lm Score = 3.82
NG = 0
DB = 0
SM = 0
Delta_L
Cytarabine
YDL052C SLC1
ZShift = 2.37
Z lm Score = −1.97
NG = 0
DB = 0
SM = 0
Delta_K
Cytarabine
YDL052C SLC1
ZShift = −1.52
Z lm Score = 0.01
NG = 0
DB = 0
SM = 0
Delta_r
Cytarabine
YDL052C SLC1
ZShift = −0.19
Z lm Score = −0.52
NG = 0
DB = 0
SM = 0
Delta_AUC
Cytarabine

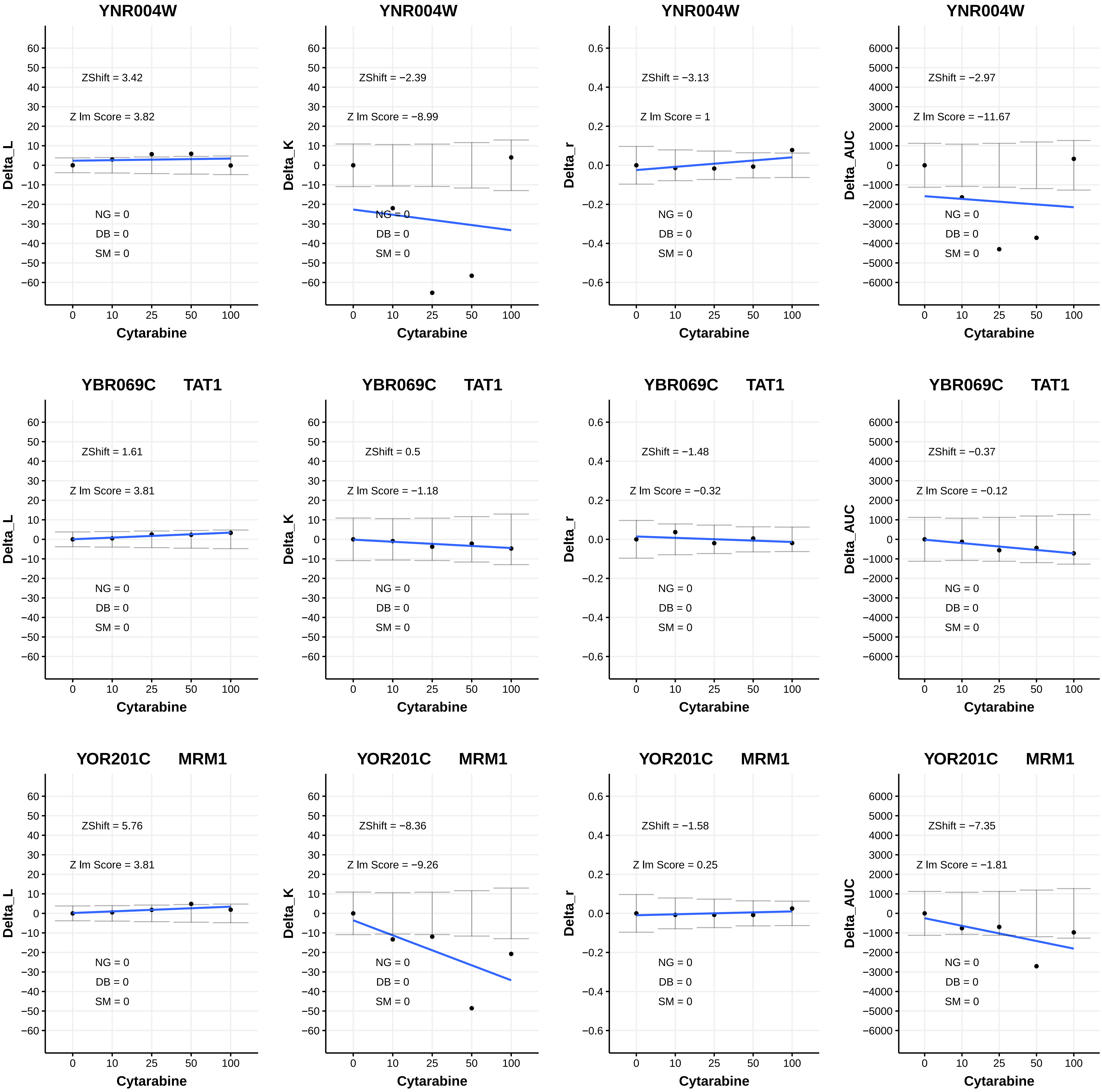

YNR004W
ZShift = 3.42
Z lm Score = 3.82
NG = 0
DB = 0
SM = 0
Delta_L
Cytarabine
YNR004W
ZShift = −2.39
Z lm Score = −8.99
NG = 0
DB = 0
SM = 0
Delta_K
Cytarabine
YNR004W
ZShift = −3.13
Z lm Score = 1
NG = 0
DB = 0
SM = 0
Delta_r
Cytarabine
YNR004W
ZShift = −2.97
Z lm Score = −11.67
NG = 0
DB = 0
SM = 0
Delta_AUC
Cytarabine
YBR069C TAT1
ZShift = 1.61
Z lm Score = 3.81
NG = 0
DB = 0
SM = 0
Delta_L
Cytarabine
YBR069C TAT1
ZShift = 0.5
Z lm Score = −1.18
NG = 0
DB = 0
SM = 0
Delta_K
Cytarabine
YBR069C TAT1
ZShift = −1.48
Z lm Score = −0.32
NG = 0
DB = 0
SM = 0
Delta_r
Cytarabine
YBR069C TAT1
ZShift = −0.37
Z lm Score = −0.12
NG = 0
DB = 0
SM = 0
Delta_AUC
Cytarabine
YOR201C MRM1
ZShift = 5.76
Z lm Score = 3.81
NG = 0
DB = 0
SM = 0
Delta_L
Cytarabine
YOR201C MRM1
ZShift = −8.36
Z lm Score = −9.26
NG = 0
DB = 0
SM = 0
Delta_K
Cytarabine
YOR201C MRM1
ZShift = −1.58
Z lm Score = 0.25
NG = 0
DB = 0
SM = 0
Delta_r
Cytarabine
YOR201C MRM1
ZShift = −7.35
Z lm Score = −1.81
NG = 0
DB = 0
SM = 0
Delta_AUC
Cytarabine

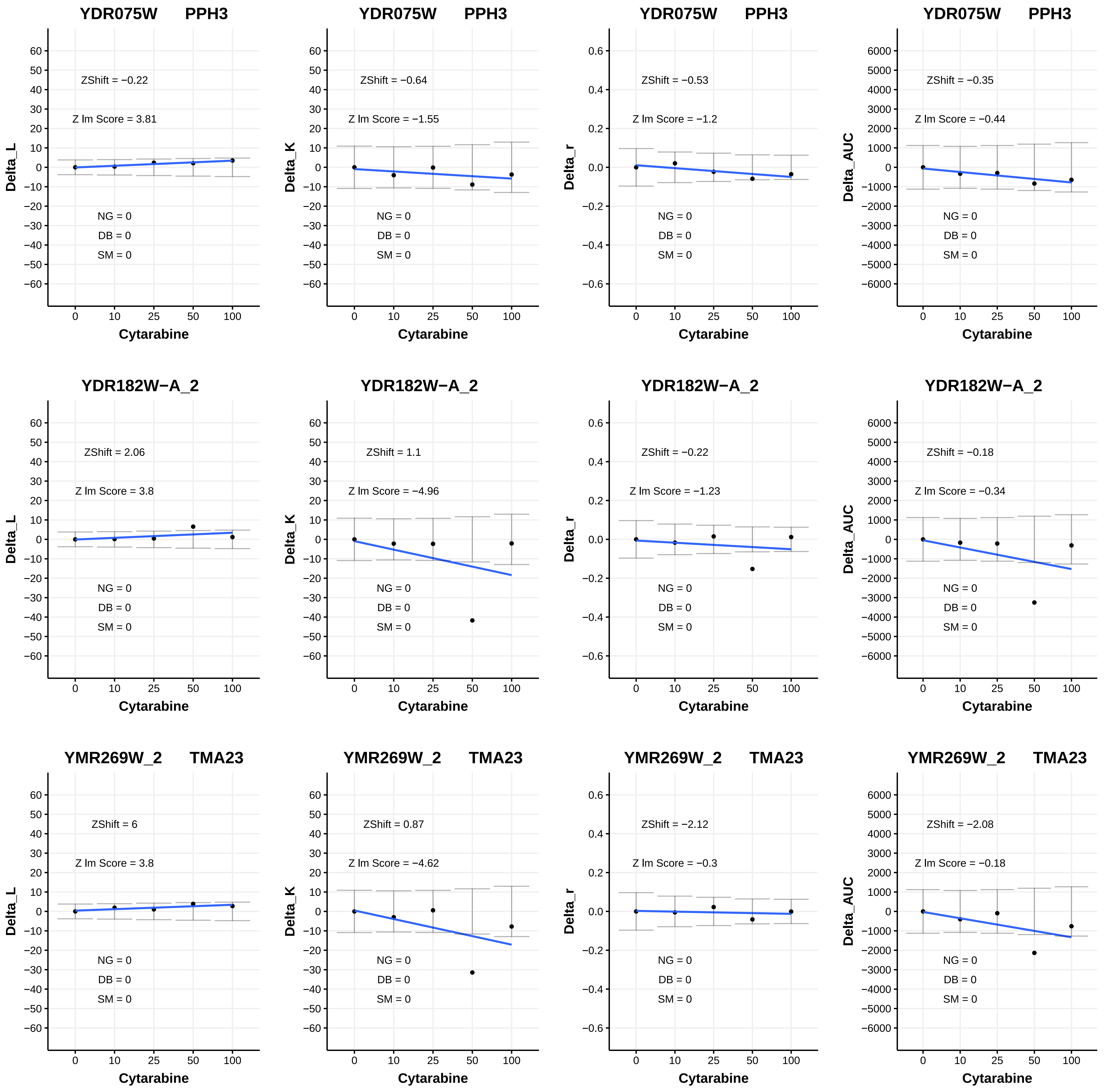

YDR075W PPH3
Delta_L
ZShift = −0.22
Z lm Score = 3.81
NG = 0
DB = 0
SM = 0
Cytarabine
YDR075W PPH3
Delta_K
ZShift = −0.64
Z lm Score = −1.55
NG = 0
DB = 0
SM = 0
Cytarabine
YDR075W PPH3
Delta_r
ZShift = −0.53
Z lm Score = −1.2
NG = 0
DB = 0
SM = 0
Cytarabine
YDR075W PPH3
Delta_AUC
ZShift = −0.35
Z lm Score = −0.44
NG = 0
DB = 0
SM = 0
Cytarabine
YDR182W−A_2
Delta_L
ZShift = 2.06
Z lm Score = 3.8
NG = 0
DB = 0
SM = 0
Cytarabine
YDR182W−A_2
Delta_K
ZShift = 1.1
Z lm Score = −4.96
NG = 0
DB = 0
SM = 0
Cytarabine
YDR182W−A_2
Delta_r
ZShift = −0.22
Z lm Score = −1.23
NG = 0
DB = 0
SM = 0
Cytarabine
YDR182W−A_2
Delta_AUC
ZShift = −0.18
Z lm Score = −0.34
NG = 0
DB = 0
SM = 0
Cytarabine
YMR269W_2 TMA23
Delta_L
ZShift = 6
Z lm Score = 3.8
NG = 0
DB = 0
SM = 0
Cytarabine
YMR269W_2 TMA23
Delta_K
ZShift = 0.87
Z lm Score = −4.62
NG = 0
DB = 0
SM = 0
Cytarabine
YMR269W_2 TMA23
Delta_r
ZShift = −2.12
Z lm Score = −0.3
NG = 0
DB = 0
SM = 0
Cytarabine
YMR269W_2 TMA23
Delta_AUC
ZShift = −2.08
Z lm Score = −0.18
NG = 0
DB = 0
SM = 0
Cytarabine

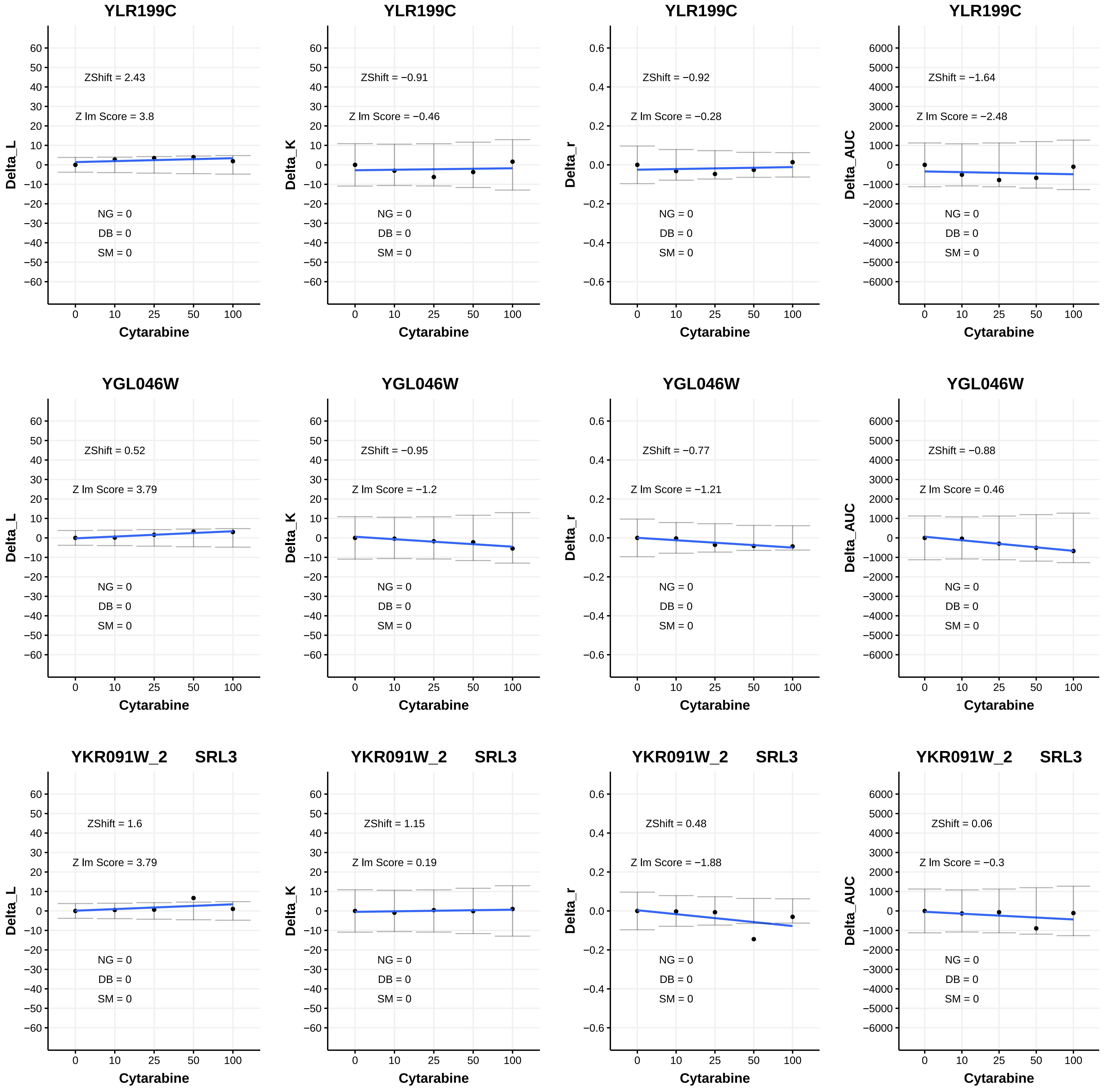

YLR199C
ZShift = 2.43
Z lm Score = 3.8
NG = 0
DB = 0
SM = 0
Delta_L
Cytarabine
YLR199C
ZShift = −0.91
Z lm Score = −0.46
NG = 0
DB = 0
SM = 0
Delta_K
Cytarabine
YLR199C
ZShift = −0.92
Z lm Score = −0.28
NG = 0
DB = 0
SM = 0
Delta_r
Cytarabine
YLR199C
ZShift = −1.64
Z lm Score = −2.48
NG = 0
DB = 0
SM = 0
Delta_AUC
Cytarabine
YGL046W
ZShift = 0.52
Z lm Score = 3.79
NG = 0
DB = 0
SM = 0
Delta_L
Cytarabine
YGL046W
ZShift = −0.95
Z lm Score = −1.2
NG = 0
DB = 0
SM = 0
Delta_K
Cytarabine
YGL046W
ZShift = −0.77
Z lm Score = −1.21
NG = 0
DB = 0
SM = 0
Delta_r
Cytarabine
YGL046W
ZShift = −0.88
Z lm Score = 0.46
NG = 0
DB = 0
SM = 0
Delta_AUC
Cytarabine
YKR091W_2 SRL3
ZShift = 1.6
Z lm Score = 3.79
NG = 0
DB = 0
SM = 0
Delta_L
Cytarabine
YKR091W_2 SRL3
ZShift = 1.15
Z lm Score = 0.19
NG = 0
DB = 0
SM = 0
Delta_K
Cytarabine
YKR091W_2 SRL3
ZShift = 0.48
Z lm Score = −1.88
NG = 0
DB = 0
SM = 0
Delta_r
Cytarabine
YKR091W_2 SRL3
ZShift = 0.06
Z lm Score = −0.3
NG = 0
DB = 0
SM = 0
Delta_AUC
Cytarabine

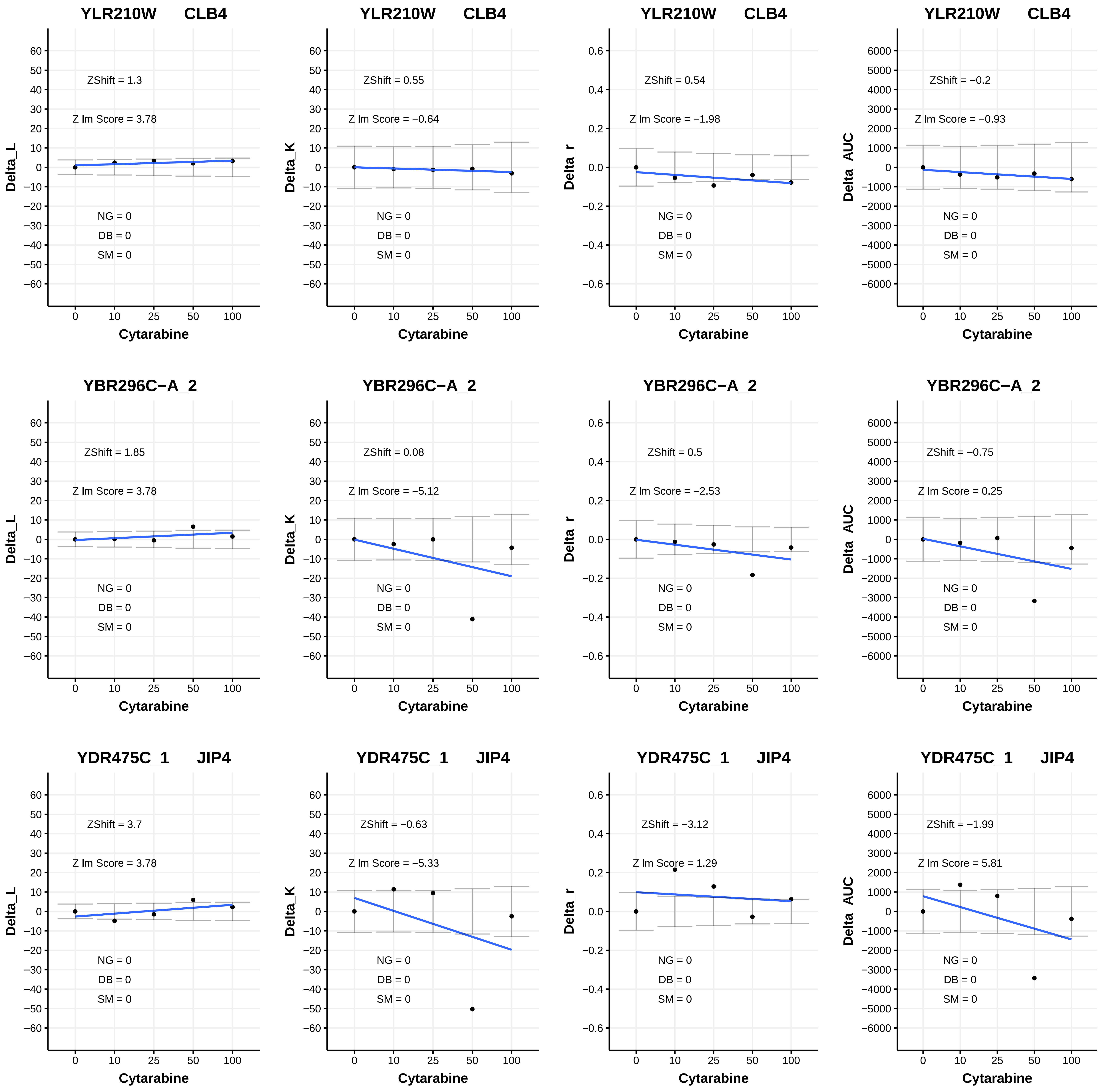

YLR210W CLB4
ZShift = 1.3
Z lm Score = 3.78
NG = 0
DB = 0
SM = 0
Delta_L
Cytarabine
YLR210W CLB4
ZShift = 0.55
Z lm Score = −0.64
NG = 0
DB = 0
SM = 0
Delta_K
Cytarabine
YLR210W CLB4
ZShift = 0.54
Z lm Score = −1.98
NG = 0
DB = 0
SM = 0
Delta_r
Cytarabine
YLR210W CLB4
ZShift = −0.2
Z lm Score = −0.93
NG = 0
DB = 0
SM = 0
Delta_AUC
Cytarabine
YBR296C−A_2
ZShift = 1.85
Z lm Score = 3.78
NG = 0
DB = 0
SM = 0
Delta_L
Cytarabine
YBR296C−A_2
ZShift = 0.08
Z lm Score = −5.12
NG = 0
DB = 0
SM = 0
Delta_K
Cytarabine
YBR296C−A_2
ZShift = 0.5
Z lm Score = −2.53
NG = 0
DB = 0
SM = 0
Delta_r
Cytarabine
YBR296C−A_2
ZShift = −0.75
Z lm Score = 0.25
NG = 0
DB = 0
SM = 0
Delta_AUC
Cytarabine
YDR475C_1 JIP4
ZShift = 3.7
Z lm Score = 3.78
NG = 0
DB = 0
SM = 0
Delta_L
Cytarabine
YDR475C_1 JIP4
ZShift = −0.63
Z lm Score = −5.33
NG = 0
DB = 0
SM = 0
Delta_K
Cytarabine
YDR475C_1 JIP4
ZShift = −3.12
Z lm Score = 1.29
NG = 0
DB = 0
SM = 0
Delta_r
Cytarabine
YDR475C_1 JIP4
ZShift = −1.99
Z lm Score = 5.81
NG = 0
DB = 0
SM = 0
Delta_AUC
Cytarabine

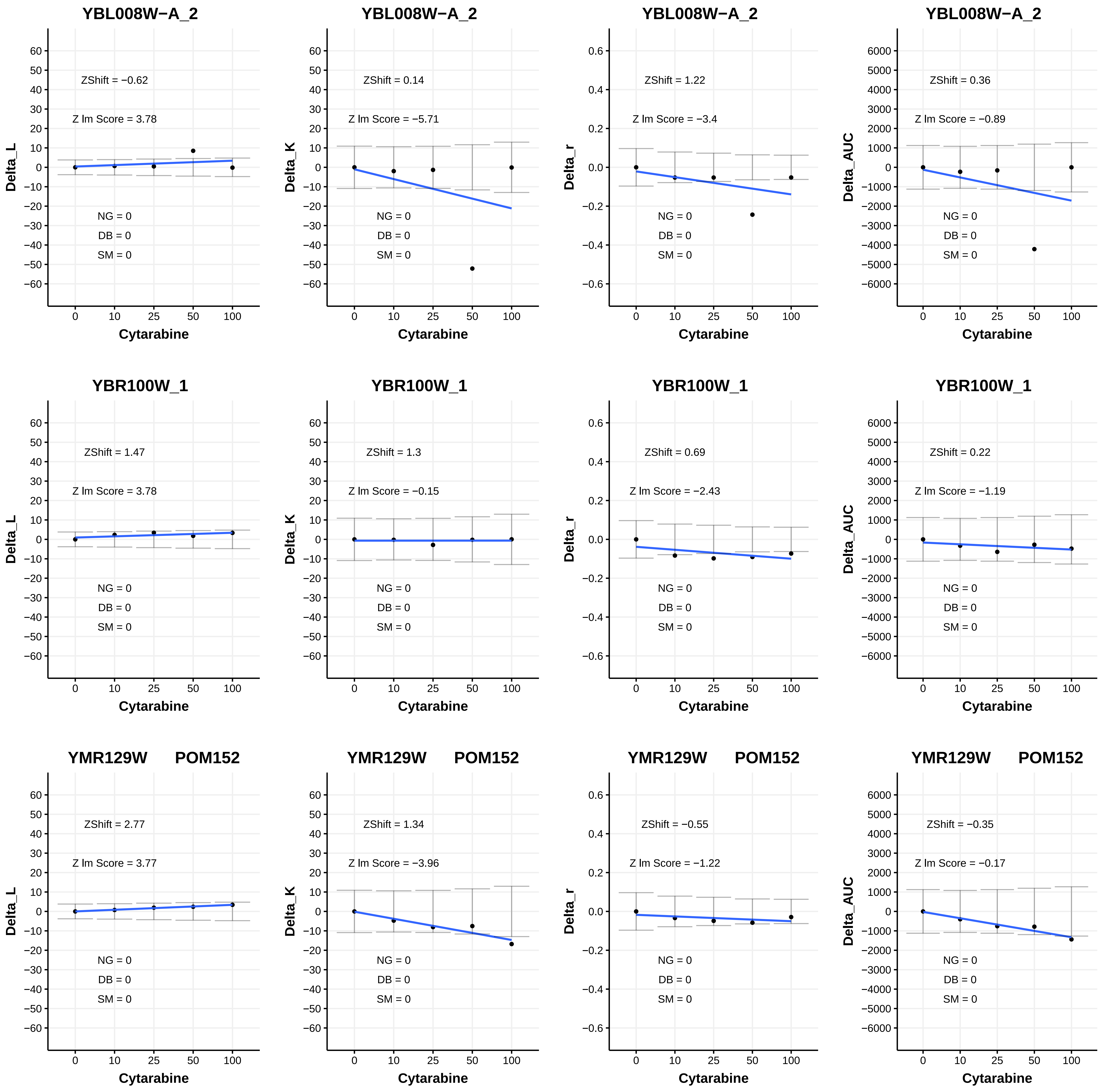
YBL008W−A_2
ZShift = −0.62
Z lm Score = 3.78
NG = 0
DB = 0
SM = 0
Delta_L
Cytarabine
YBL008W−A_2
ZShift = 0.14
Z lm Score = −5.71
NG = 0
DB = 0
SM = 0
Delta_K
Cytarabine
YBL008W−A_2
ZShift = 1.22
Z lm Score = −3.4
NG = 0
DB = 0
SM = 0
Delta_r
Cytarabine
YBL008W−A_2
ZShift = 0.36
Z lm Score = −0.89
NG = 0
DB = 0
SM = 0
Delta_AUC
Cytarabine
YBR100W_1
ZShift = 1.47
Z lm Score = 3.78
NG = 0
DB = 0
SM = 0
Delta_L
Cytarabine
YBR100W_1
ZShift = 1.3
Z lm Score = −0.15
NG = 0
DB = 0
SM = 0
Delta_K
Cytarabine
YBR100W_1
ZShift = 0.69
Z lm Score = −2.43
NG = 0
DB = 0
SM = 0
Delta_r
Cytarabine
YBR100W_1
ZShift = 0.22
Z lm Score = −1.19
NG = 0
DB = 0
SM = 0
Delta_AUC
Cytarabine
YMR129W POM152
ZShift = 2.77
Z lm Score = 3.77
NG = 0
DB = 0
SM = 0
Delta_L
Cytarabine
YMR129W POM152
ZShift = 1.34
Z lm Score = −3.96
NG = 0
DB = 0
SM = 0
Delta_K
Cytarabine
YMR129W POM152
ZShift = −0.55
Z lm Score = −1.22
NG = 0
DB = 0
SM = 0
Delta_r
Cytarabine
YMR129W POM152
ZShift = −0.35
Z lm Score = −0.17
NG = 0
DB = 0
SM = 0
Delta_AUC
Cytarabine

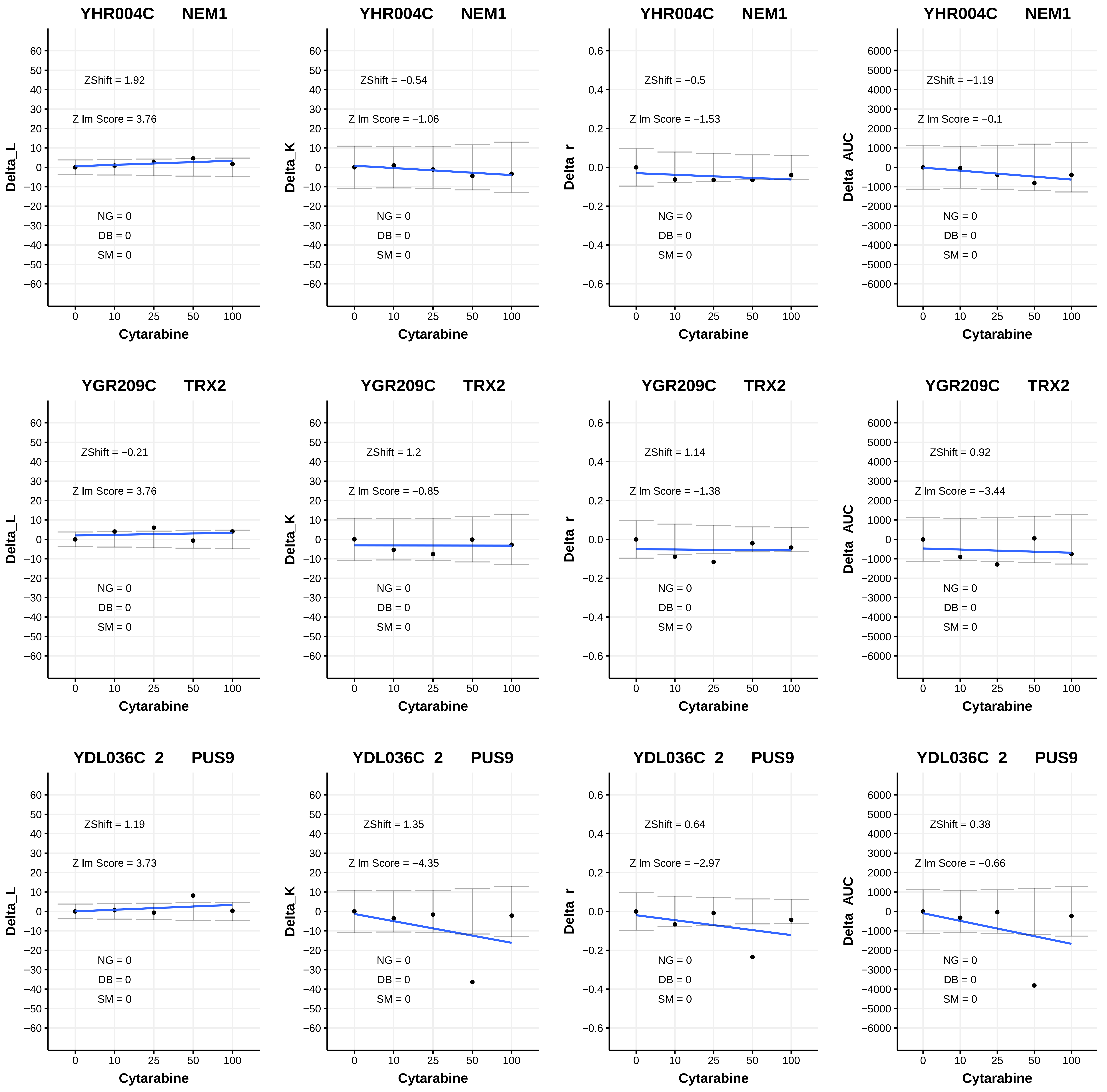

YHR004C NEM1
ZShift = 1.92
Z lm Score = 3.76
NG = 0
DB = 0
SM = 0
Delta_L
Cytarabine
YHR004C NEM1
ZShift = −0.54
Z lm Score = −1.06
NG = 0
DB = 0
SM = 0
Delta_K
Cytarabine
YHR004C NEM1
ZShift = −0.5
Z lm Score = −1.53
NG = 0
DB = 0
SM = 0
Delta_r
Cytarabine
YHR004C NEM1
ZShift = −1.19
Z lm Score = −0.1
NG = 0
DB = 0
SM = 0
Delta_AUC
Cytarabine
YGR209C TRX2
ZShift = −0.21
Z lm Score = 3.76
NG = 0
DB = 0
SM = 0
Delta_L
Cytarabine
YGR209C TRX2
ZShift = 1.2
Z lm Score = −0.85
NG = 0
DB = 0
SM = 0
Delta_K
Cytarabine
YGR209C TRX2
ZShift = 1.14
Z lm Score = −1.38
NG = 0
DB = 0
SM = 0
Delta_r
Cytarabine
YGR209C TRX2
ZShift = 0.92
Z lm Score = −3.44
NG = 0
DB = 0
SM = 0
Delta_AUC
Cytarabine
YDL036C_2 PUS9
ZShift = 1.19
Z lm Score = 3.73
NG = 0
DB = 0
SM = 0
Delta_L
Cytarabine
YDL036C_2 PUS9
ZShift = 1.35
Z lm Score = −4.35
NG = 0
DB = 0
SM = 0
Delta_K
Cytarabine
YDL036C_2 PUS9
ZShift = 0.64
Z lm Score = −2.97
NG = 0
DB = 0
SM = 0
Delta_r
Cytarabine
YDL036C_2 PUS9
ZShift = 0.38
Z lm Score = −0.66
NG = 0
DB = 0
SM = 0
Delta_AUC
Cytarabine

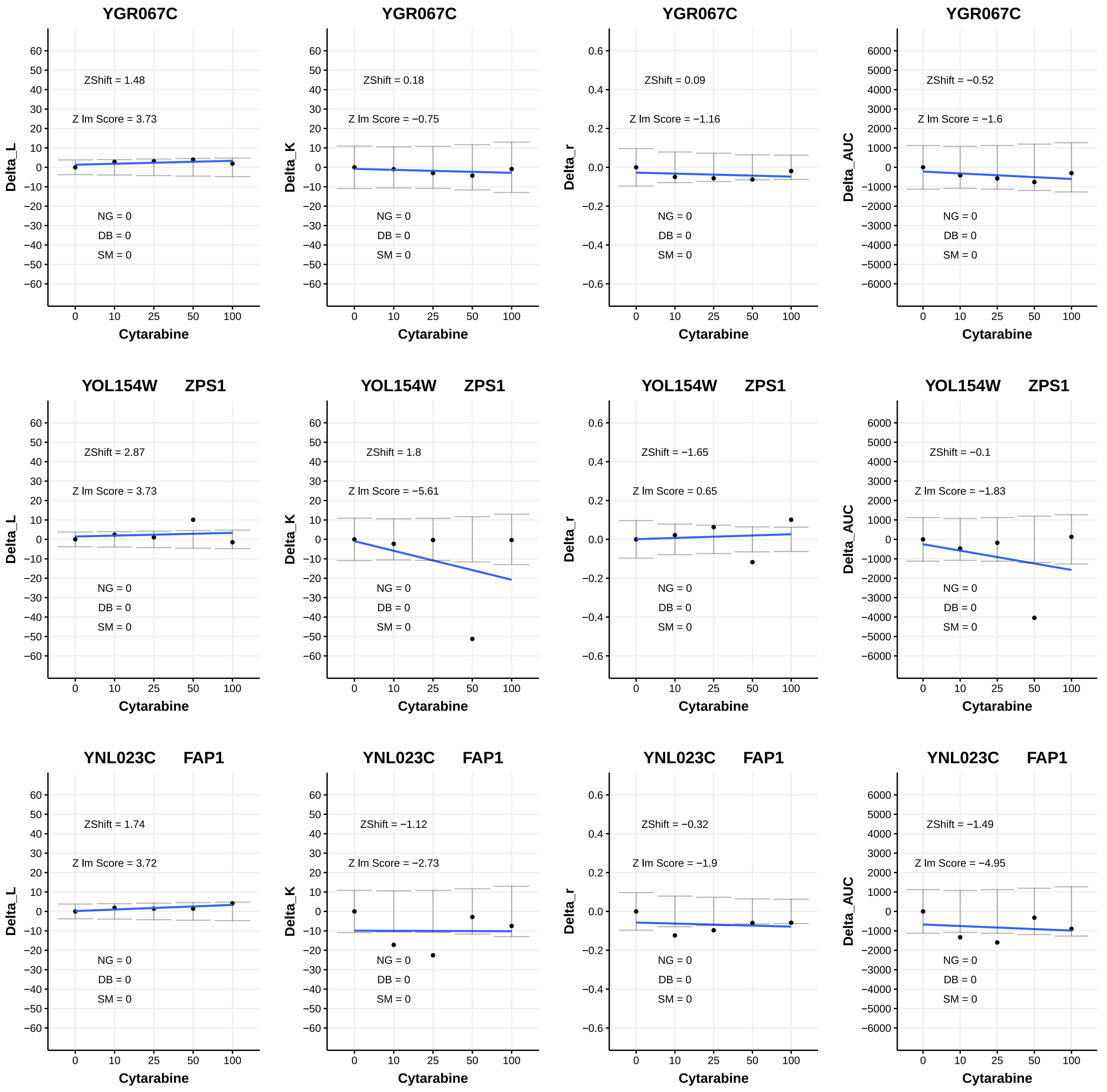

YGR067C
ZShift = 1.48
Z lm Score = 3.73
NG = 0
DB = 0
SM = 0
Delta_L
Cytarabine
YGR067C
ZShift = 0.18
Z lm Score = −0.75
NG = 0
DB = 0
SM = 0
Delta_K
Cytarabine
YGR067C
ZShift = 0.09
Z lm Score = −1.16
NG = 0
DB = 0
SM = 0
Delta_r
Cytarabine
YGR067C
ZShift = −0.52
Z lm Score = −1.6
NG = 0
DB = 0
SM = 0
Delta_AUC
Cytarabine
YOL154W ZPS1
ZShift = 2.87
Z lm Score = 3.73
NG = 0
DB = 0
SM = 0
Delta_L
Cytarabine
YOL154W ZPS1
ZShift = 1.8
Z lm Score = −5.61
NG = 0
DB = 0
SM = 0
Delta_K
Cytarabine
YOL154W ZPS1
ZShift = −1.65
Z lm Score = 0.65
NG = 0
DB = 0
SM = 0
Delta_r
Cytarabine
YOL154W ZPS1
ZShift = −0.1
Z lm Score = −1.83
NG = 0
DB = 0
SM = 0
Delta_AUC
Cytarabine
YNL023C FAP1
ZShift = 1.74
Z lm Score = 3.72
NG = 0
DB = 0
SM = 0
Delta_L
Cytarabine
YNL023C FAP1
ZShift = −1.12
Z lm Score = −2.73
NG = 0
DB = 0
SM = 0
Delta_K
Cytarabine
YNL023C FAP1
ZShift = −0.32
Z lm Score = −1.9
NG = 0
DB = 0
SM = 0
Delta_r
Cytarabine
YNL023C FAP1
ZShift = −1.49
Z lm Score = −4.95
NG = 0
DB = 0
SM = 0
Delta_AUC
Cytarabine

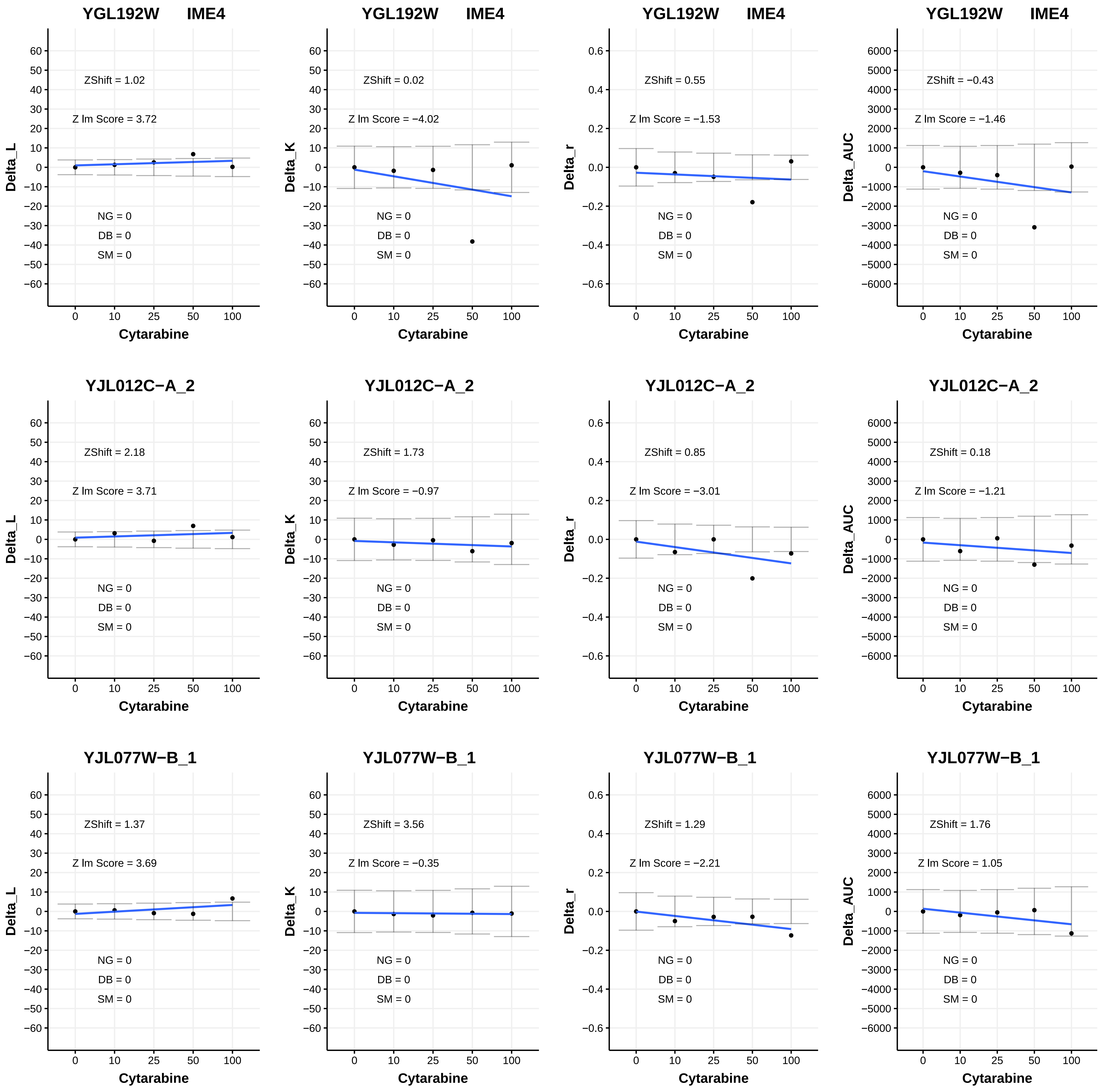

YGL192W IME4
ZShift = 1.02
Z lm Score = 3.72
NG = 0
DB = 0
SM = 0
Delta_L
Cytarabine
YGL192W IME4
ZShift = 0.02
Z lm Score = −4.02
NG = 0
DB = 0
SM = 0
Delta_K
Cytarabine
YGL192W IME4
ZShift = 0.55
Z lm Score = −1.53
NG = 0
DB = 0
SM = 0
Delta_r
Cytarabine
YGL192W IME4
ZShift = −0.43
Z lm Score = −1.46
NG = 0
DB = 0
SM = 0
Delta_AUC
Cytarabine
YJL012C−A_2
ZShift = 2.18
Z lm Score = 3.71
NG = 0
DB = 0
SM = 0
Delta_L
Cytarabine
YJL012C−A_2
ZShift = 1.73
Z lm Score = −0.97
NG = 0
DB = 0
SM = 0
Delta_K
Cytarabine
YJL012C−A_2
ZShift = 0.85
Z lm Score = −3.01
NG = 0
DB = 0
SM = 0
Delta_r
Cytarabine
YJL012C−A_2
ZShift = 0.18
Z lm Score = −1.21
NG = 0
DB = 0
SM = 0
Delta_AUC
Cytarabine
YJL077W−B_1
ZShift = 1.37
Z lm Score = 3.69
NG = 0
DB = 0
SM = 0
Delta_L
Cytarabine
YJL077W−B_1
ZShift = 3.56
Z lm Score = −0.35
NG = 0
DB = 0
SM = 0
Delta_K
Cytarabine
YJL077W−B_1
ZShift = 1.29
Z lm Score = −2.21
NG = 0
DB = 0
SM = 0
Delta_r
Cytarabine
YJL077W−B_1
ZShift = 1.76
Z lm Score = 1.05
NG = 0
DB = 0
SM = 0
Delta_AUC
Cytarabine

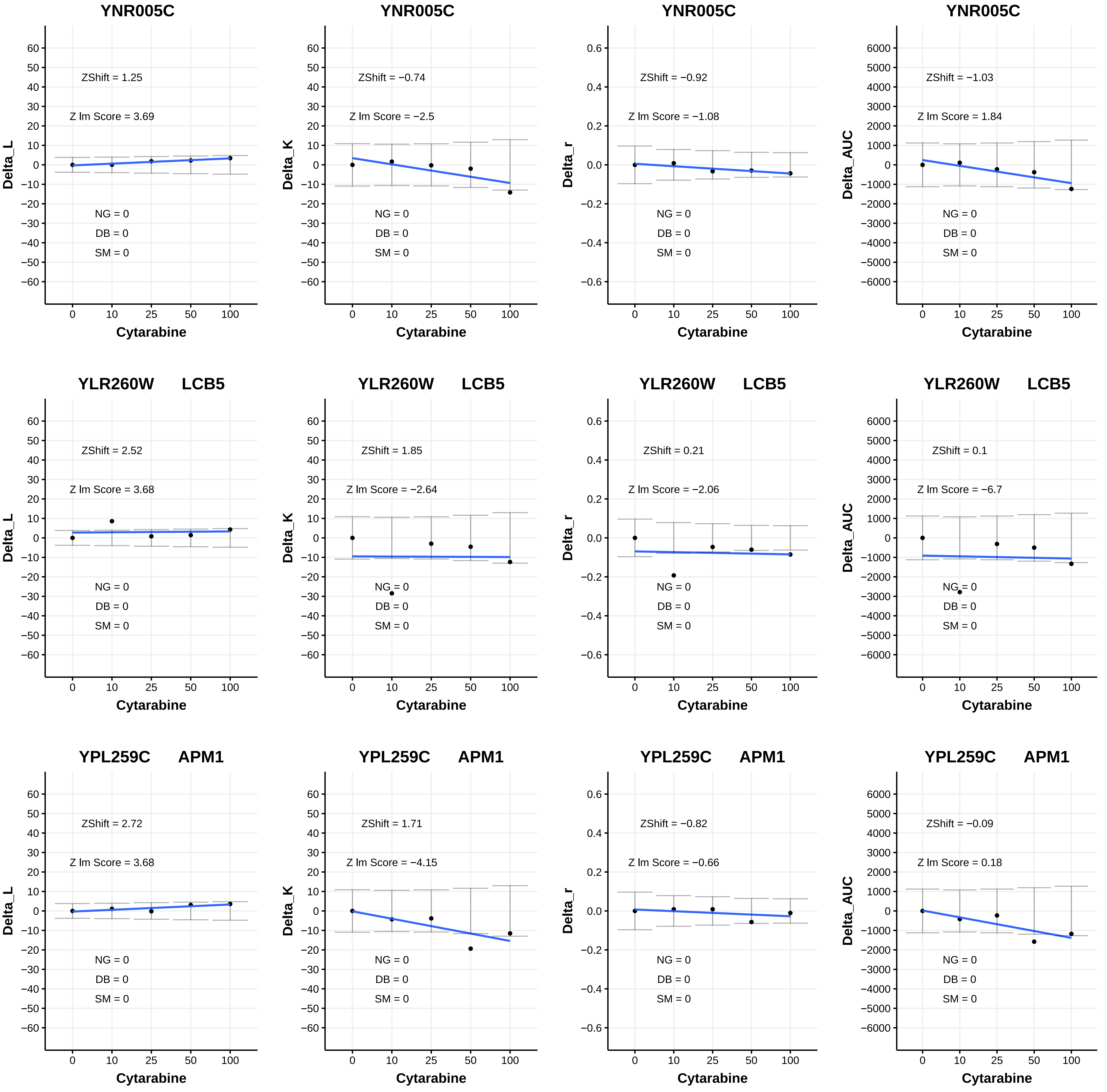

YNR005C
ZShift = 1.25
Z lm Score = 3.69
NG = 0
DB = 0
SM = 0
Delta_L
Cytarabine
YNR005C
ZShift = −0.74
Z lm Score = −2.5
NG = 0
DB = 0
SM = 0
Delta_K
Cytarabine
YNR005C
ZShift = −0.92
Z lm Score = −1.08
NG = 0
DB = 0
SM = 0
Delta_r
Cytarabine
YNR005C
ZShift = −1.03
Z lm Score = 1.84
NG = 0
DB = 0
SM = 0
Delta_AUC
Cytarabine
YLR260W LCB5
ZShift = 2.52
Z lm Score = 3.68
NG = 0
DB = 0
SM = 0
Delta_L
Cytarabine
YLR260W LCB5
ZShift = 1.85
Z lm Score = −2.64
NG = 0
DB = 0
SM = 0
Delta_K
Cytarabine
YLR260W LCB5
ZShift = 0.21
Z lm Score = −2.06
NG = 0
DB = 0
SM = 0
Delta_r
Cytarabine
YLR260W LCB5
ZShift = 0.1
Z lm Score = −6.7
NG = 0
DB = 0
SM = 0
Delta_AUC
Cytarabine
YPL259C APM1
ZShift = 2.72
Z lm Score = 3.68
NG = 0
DB = 0
SM = 0
Delta_L
Cytarabine
YPL259C APM1
ZShift = 1.71
Z lm Score = −4.15
NG = 0
DB = 0
SM = 0
Delta_K
Cytarabine
YPL259C APM1
ZShift = −0.82
Z lm Score = −0.66
NG = 0
DB = 0
SM = 0
Delta_r
Cytarabine
YPL259C APM1
ZShift = −0.09
Z lm Score = 0.18
NG = 0
DB = 0
SM = 0
Delta_AUC
Cytarabine

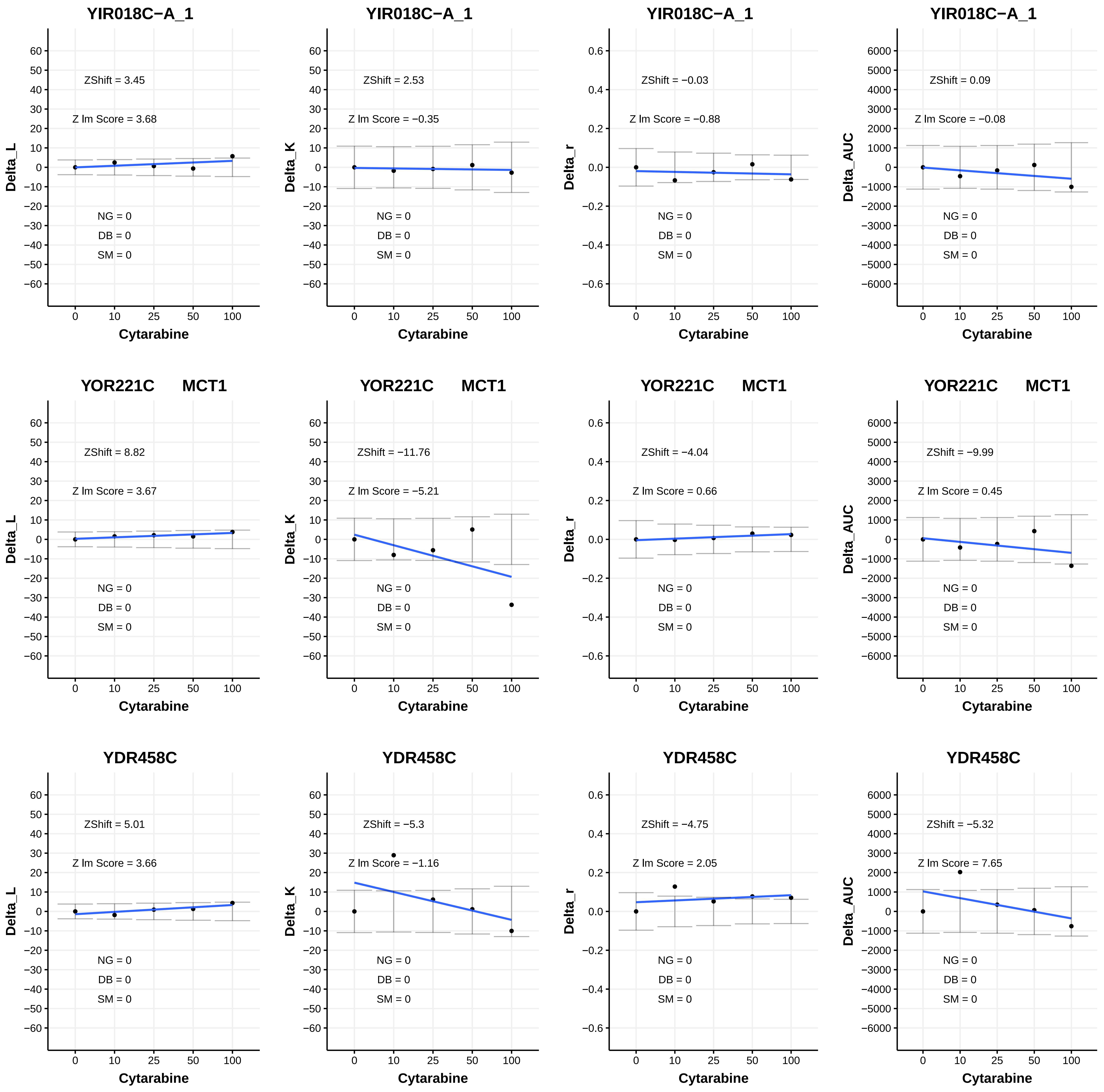

YIR018C−A_1
ZShift = 3.45
Z lm Score = 3.68
NG = 0
DB = 0
SM = 0
Delta_L
Cytarabine
YIR018C−A_1
ZShift = 2.53
Z lm Score = −0.35
NG = 0
DB = 0
SM = 0
Delta_K
Cytarabine
YIR018C−A_1
ZShift = −0.03
Z lm Score = −0.88
NG = 0
DB = 0
SM = 0
Delta_r
Cytarabine
YIR018C−A_1
ZShift = 0.09
Z lm Score = −0.08
NG = 0
DB = 0
SM = 0
Delta_AUC
Cytarabine
YOR221C MCT1
ZShift = 8.82
Z lm Score = 3.67
NG = 0
DB = 0
SM = 0
Delta_L
Cytarabine
YOR221C MCT1
ZShift = −11.76
Z lm Score = −5.21
NG = 0
DB = 0
SM = 0
Delta_K
Cytarabine
YOR221C MCT1
ZShift = −4.04
Z lm Score = 0.66
NG = 0
DB = 0
SM = 0
Delta_r
Cytarabine
YOR221C MCT1
ZShift = −9.99
Z lm Score = 0.45
NG = 0
DB = 0
SM = 0
Delta_AUC
Cytarabine
YDR458C
ZShift = 5.01
Z lm Score = 3.66
NG = 0
DB = 0
SM = 0
Delta_L
Cytarabine
YDR458C
ZShift = −5.3
Z lm Score = −1.16
NG = 0
DB = 0
SM = 0
Delta_K
Cytarabine
YDR458C
ZShift = −4.75
Z lm Score = 2.05
NG = 0
DB = 0
SM = 0
Delta_r
Cytarabine
YDR458C
ZShift = −5.32
Z lm Score = 7.65
NG = 0
DB = 0
SM = 0
Delta_AUC
Cytarabine

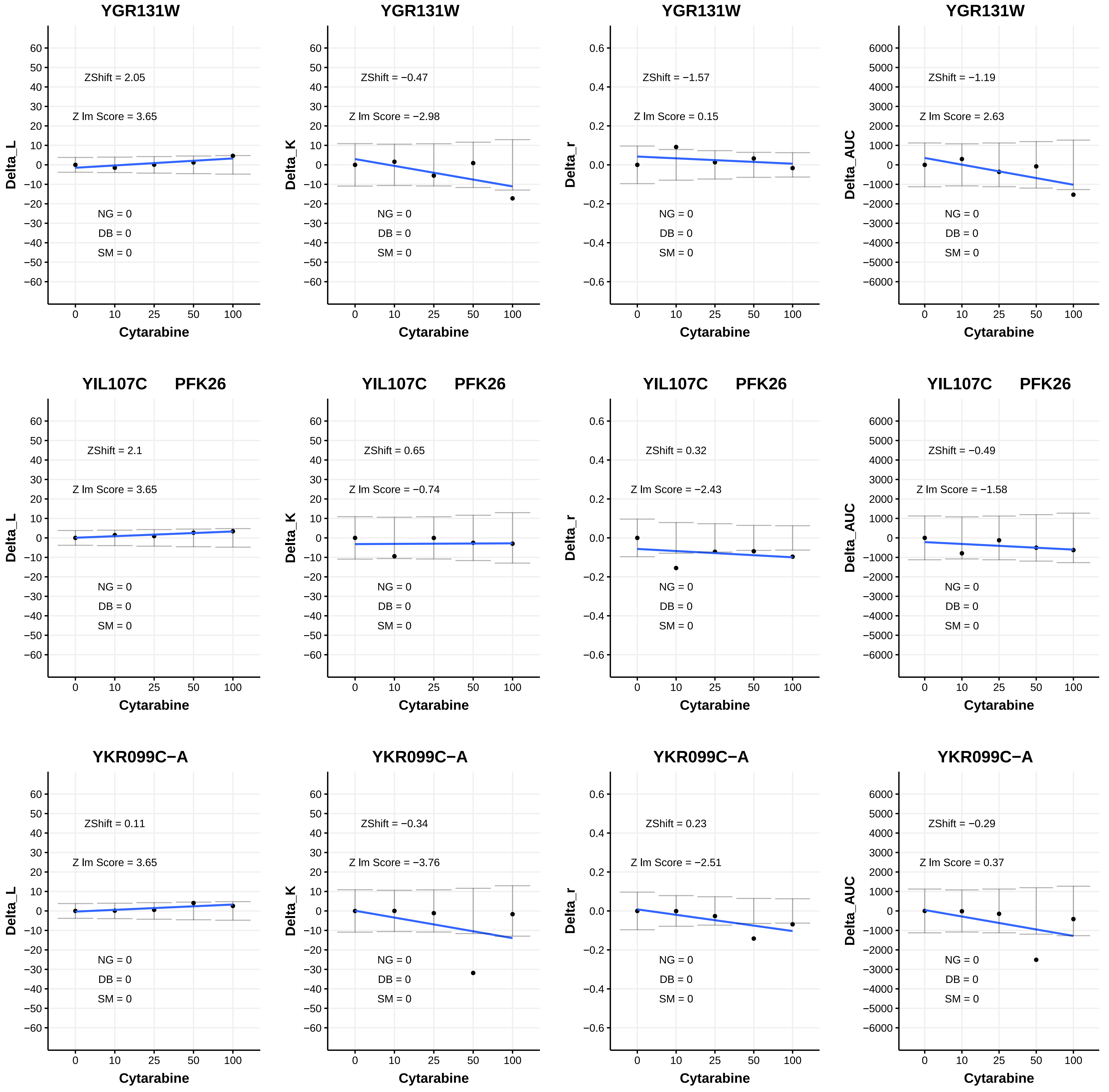
YGR131W
ZShift = 2.05
Z lm Score = 3.65
NG = 0
DB = 0
SM = 0
Delta_L
Cytarabine
YGR131W
ZShift = −0.47
Z lm Score = −2.98
NG = 0
DB = 0
SM = 0
Delta_K
Cytarabine
YGR131W
ZShift = −1.57
Z lm Score = 0.15
NG = 0
DB = 0
SM = 0
Delta_r
Cytarabine
YGR131W
ZShift = −1.19
Z lm Score = 2.63
NG = 0
DB = 0
SM = 0
Delta_AUC
Cytarabine
YIL107C PFK26
ZShift = 2.1
Z lm Score = 3.65
NG = 0
DB = 0
SM = 0
Delta_L
Cytarabine
YIL107C PFK26
ZShift = 0.65
Z lm Score = −0.74
NG = 0
DB = 0
SM = 0
Delta_K
Cytarabine
YIL107C PFK26
ZShift = 0.32
Z lm Score = −2.43
NG = 0
DB = 0
SM = 0
Delta_r
Cytarabine
YIL107C PFK26
ZShift = −0.49
Z lm Score = −1.58
NG = 0
DB = 0
SM = 0
Delta_AUC
Cytarabine
YKR099C−A
ZShift = 0.11
Z lm Score = 3.65
NG = 0
DB = 0
SM = 0
Delta_L
Cytarabine
YKR099C−A
ZShift = −0.34
Z lm Score = −3.76
NG = 0
DB = 0
SM = 0
Delta_K
Cytarabine
YKR099C−A
ZShift = 0.23
Z lm Score = −2.51
NG = 0
DB = 0
SM = 0
Delta_r
Cytarabine
YKR099C−A
ZShift = −0.29
Z lm Score = 0.37
NG = 0
DB = 0
SM = 0
Delta_AUC
Cytarabine

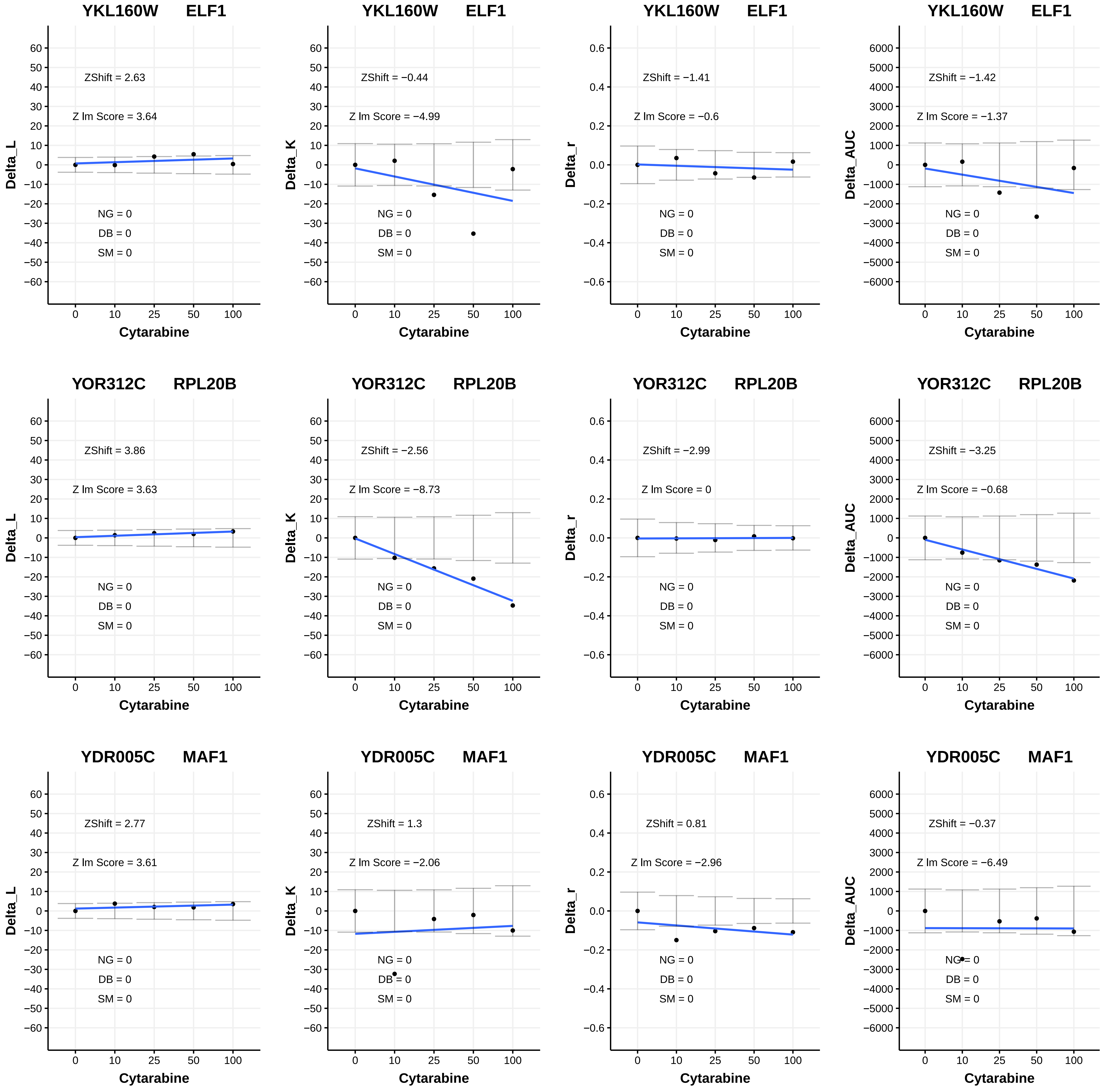

YKL160W ELF1
ZShift = 2.63
Z lm Score = 3.64
NG = 0
DB = 0
SM = 0
Delta_L
Cytarabine
YKL160W ELF1
ZShift = −0.44
Z lm Score = −4.99
NG = 0
DB = 0
SM = 0
Delta_K
Cytarabine
YKL160W ELF1
ZShift = −1.41
Z lm Score = −0.6
NG = 0
DB = 0
SM = 0
Delta_r
Cytarabine
YKL160W ELF1
ZShift = −1.42
Z lm Score = −1.37
NG = 0
DB = 0
SM = 0
Delta_AUC
Cytarabine
YOR312C RPL20B
ZShift = 3.86
Z lm Score = 3.63
NG = 0
DB = 0
SM = 0
Delta_L
Cytarabine
YOR312C RPL20B
ZShift = −2.56
Z lm Score = −8.73
NG = 0
DB = 0
SM = 0
Delta_K
Cytarabine
YOR312C RPL20B
ZShift = −2.99
Z lm Score = 0
NG = 0
DB = 0
SM = 0
Delta_r
Cytarabine
YOR312C RPL20B
ZShift = −3.25
Z lm Score = −0.68
NG = 0
DB = 0
SM = 0
Delta_AUC
Cytarabine
YDR005C MAF1
ZShift = 2.77
Z lm Score = 3.61
NG = 0
DB = 0
SM = 0
Delta_L
Cytarabine
YDR005C MAF1
ZShift = 1.3
Z lm Score = −2.06
NG = 0
DB = 0
SM = 0
Delta_K
Cytarabine
YDR005C MAF1
ZShift = 0.81
Z lm Score = −2.96
NG = 0
DB = 0
SM = 0
Delta_r
Cytarabine
YDR005C MAF1
ZShift = −0.37
Z lm Score = −6.49
NG = 0
DB = 0
SM = 0
Delta_AUC
Cytarabine

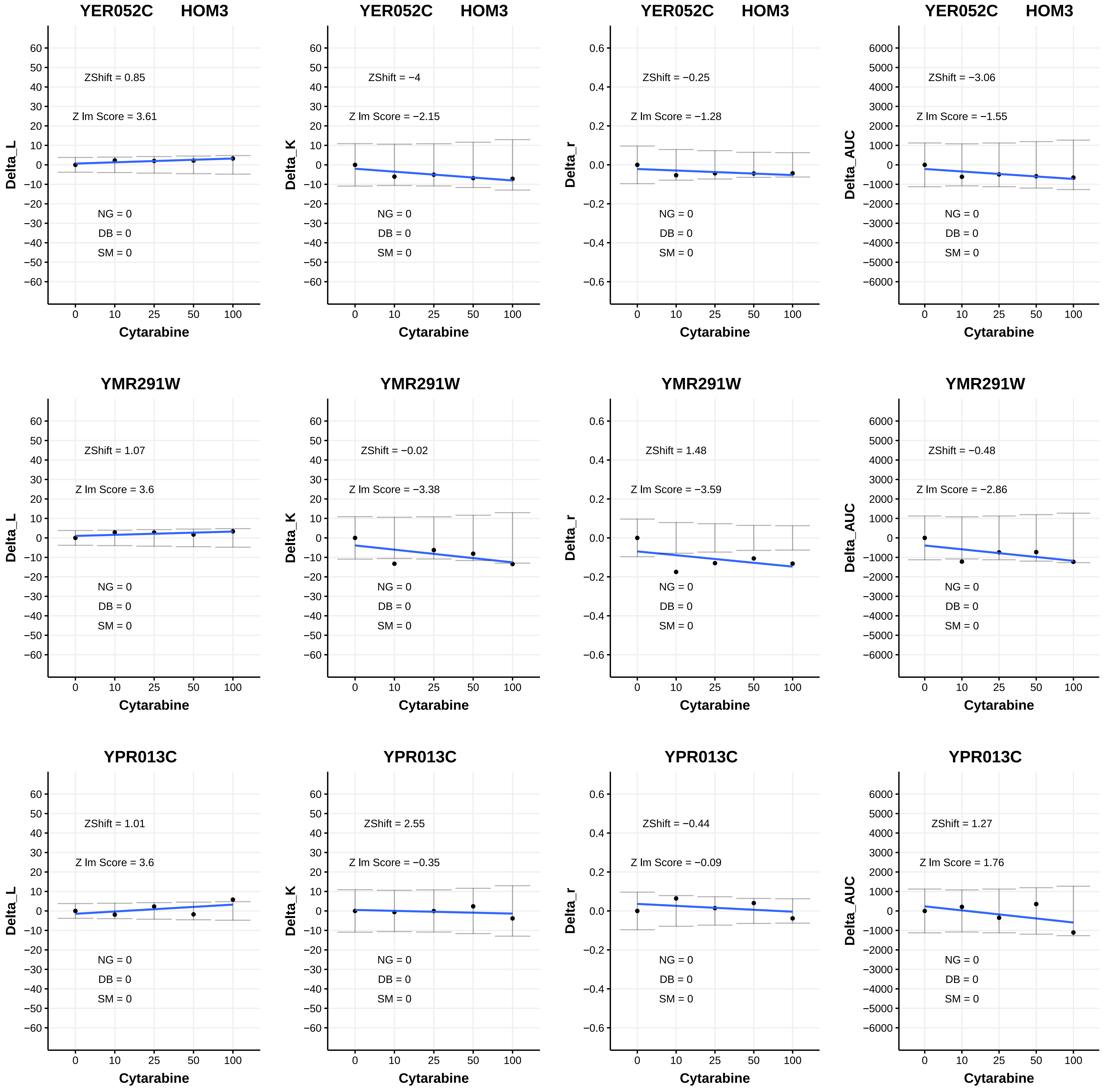
YER052C HOM3
ZShift = 0.85
Z lm Score = 3.61
NG = 0
DB = 0
SM = 0
Delta_L
Cytarabine
YER052C HOM3
ZShift = −4
Z lm Score = −2.15
NG = 0
DB = 0
SM = 0
Delta_K
Cytarabine
YER052C HOM3
ZShift = −0.25
Z lm Score = −1.28
NG = 0
DB = 0
SM = 0
Delta_r
Cytarabine
YER052C HOM3
ZShift = −3.06
Z lm Score = −1.55
NG = 0
DB = 0
SM = 0
Delta_AUC
Cytarabine
YMR291W
ZShift = 1.07
Z lm Score = 3.6
NG = 0
DB = 0
SM = 0
Delta_L
Cytarabine
YMR291W
ZShift = −0.02
Z lm Score = −3.38
NG = 0
DB = 0
SM = 0
Delta_K
Cytarabine
YMR291W
ZShift = 1.48
Z lm Score = −3.59
NG = 0
DB = 0
SM = 0
Delta_r
Cytarabine
YMR291W
ZShift = −0.48
Z lm Score = −2.86
NG = 0
DB = 0
SM = 0
Delta_AUC
Cytarabine
YPR013C
ZShift = 1.01
Z lm Score = 3.6
NG = 0
DB = 0
SM = 0
Delta_L
Cytarabine
YPR013C
ZShift = 2.55
Z lm Score = −0.35
NG = 0
DB = 0
SM = 0
Delta_K
Cytarabine
YPR013C
ZShift = −0.44
Z lm Score = −0.09
NG = 0
DB = 0
SM = 0
Delta_r
Cytarabine
YPR013C
ZShift = 1.27
Z lm Score = 1.76
NG = 0
DB = 0
SM = 0
Delta_AUC
Cytarabine

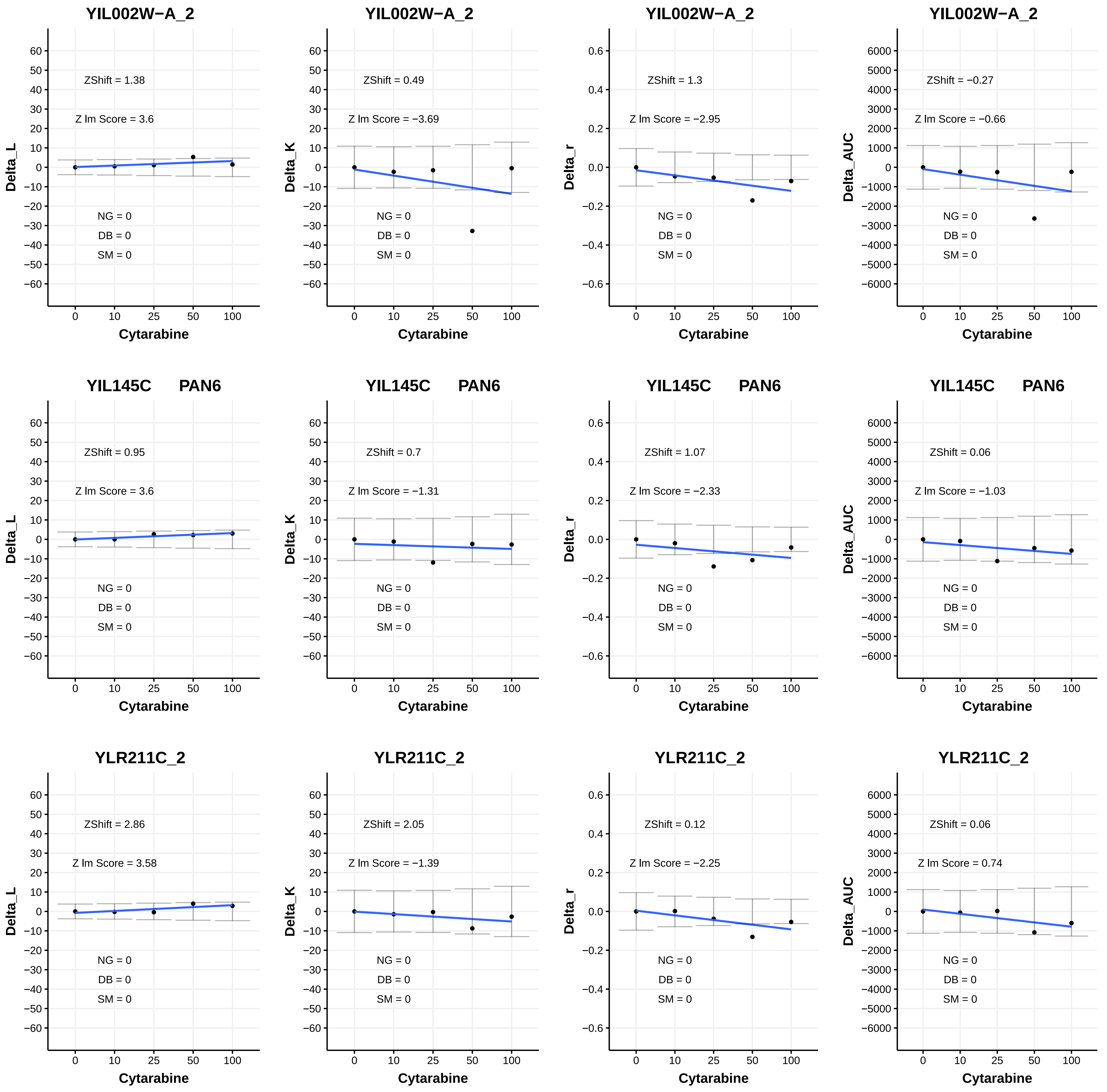

YIL002W−A_2
ZShift = 1.38
Z lm Score = 3.6
NG = 0
DB = 0
SM = 0
Delta_L
Cytarabine
YIL002W−A_2
ZShift = 0.49
Z lm Score = −3.69
NG = 0
DB = 0
SM = 0
Delta_K
Cytarabine
YIL002W−A_2
ZShift = 1.3
Z lm Score = −2.95
NG = 0
DB = 0
SM = 0
Delta_r
Cytarabine
YIL002W−A_2
ZShift = −0.27
Z lm Score = −0.66
NG = 0
DB = 0
SM = 0
Delta_AUC
Cytarabine
YIL145C PAN6
ZShift = 0.95
Z lm Score = 3.6
NG = 0
DB = 0
SM = 0
Delta_L
Cytarabine
YIL145C PAN6
ZShift = 0.7
Z lm Score = −1.31
NG = 0
DB = 0
SM = 0
Delta_K
Cytarabine
YIL145C PAN6
ZShift = 1.07
Z lm Score = −2.33
NG = 0
DB = 0
SM = 0
Delta_r
Cytarabine
YIL145C PAN6
ZShift = 0.06
Z lm Score = −1.03
NG = 0
DB = 0
SM = 0
Delta_AUC
Cytarabine
YLR211C_2
ZShift = 2.86
Z lm Score = 3.58
NG = 0
DB = 0
SM = 0
Delta_L
Cytarabine
YLR211C_2
ZShift = 2.05
Z lm Score = −1.39
NG = 0
DB = 0
SM = 0
Delta_K
Cytarabine
YLR211C_2
ZShift = 0.12
Z lm Score = −2.25
NG = 0
DB = 0
SM = 0
Delta_r
Cytarabine
YLR211C_2
ZShift = 0.06
Z lm Score = 0.74
NG = 0
DB = 0
SM = 0
Delta_AUC
Cytarabine

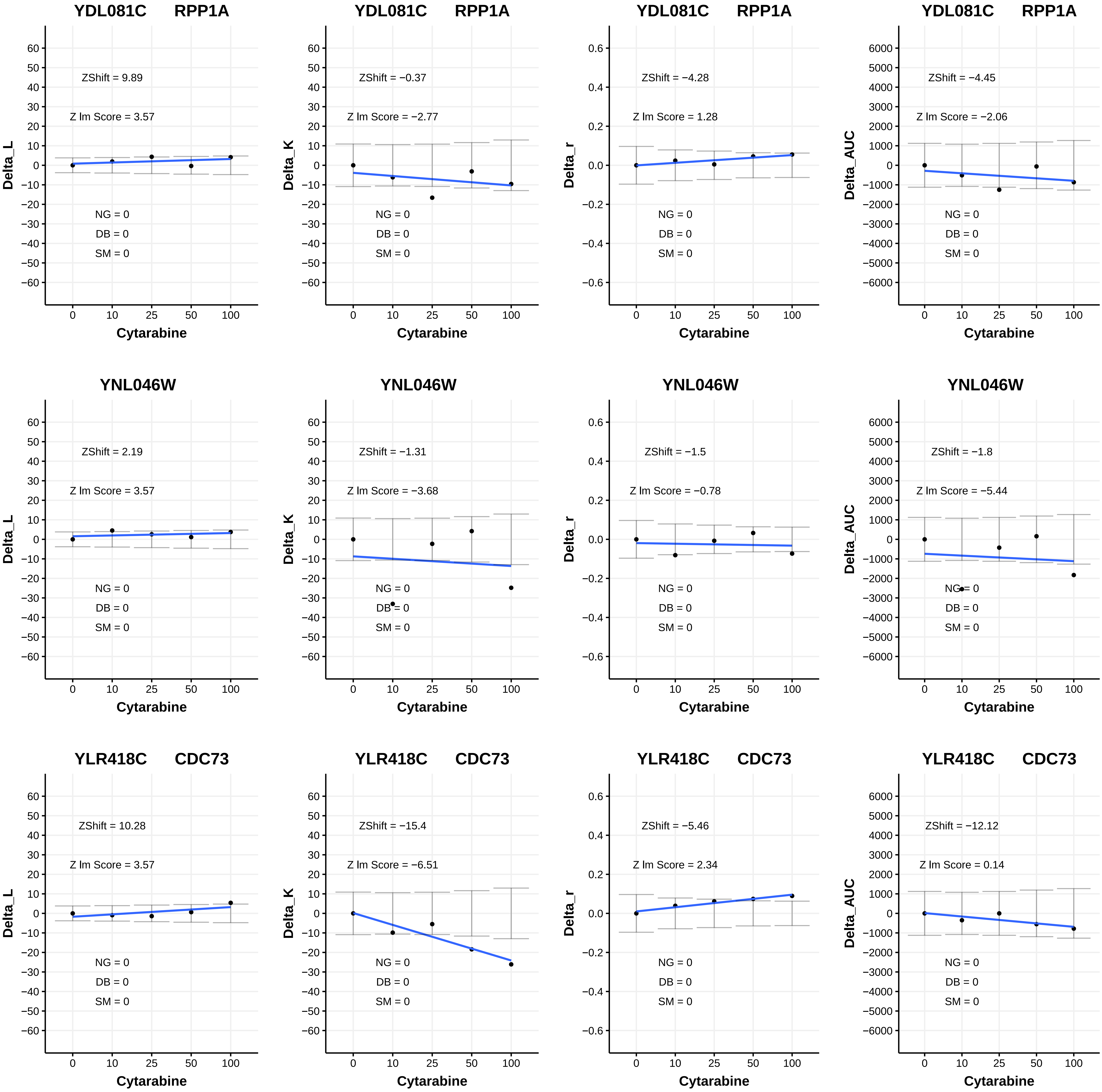

YDL081C RPP1A
ZShift = 9.89
Z lm Score = 3.57
NG = 0
DB = 0
SM = 0
Delta_L
Cytarabine
YDL081C RPP1A
ZShift = −0.37
Z lm Score = −2.77
NG = 0
DB = 0
SM = 0
Delta_K
Cytarabine
YDL081C RPP1A
ZShift = −4.28
Z lm Score = 1.28
NG = 0
DB = 0
SM = 0
Delta_r
Cytarabine
YDL081C RPP1A
ZShift = −4.45
Z lm Score = −2.06
NG = 0
DB = 0
SM = 0
Delta_AUC
Cytarabine
YNL046W
ZShift = 2.19
Z lm Score = 3.57
NG = 0
DB = 0
SM = 0
Delta_L
Cytarabine
YNL046W
ZShift = −1.31
Z lm Score = −3.68
NG = 0
DB = 0
SM = 0
Delta_K
Cytarabine
YNL046W
ZShift = −1.5
Z lm Score = −0.78
NG = 0
DB = 0
SM = 0
Delta_r
Cytarabine
YNL046W
ZShift = −1.8
Z lm Score = −5.44
NG = 0
DB = 0
SM = 0
Delta_AUC
Cytarabine
YLR418C CDC73
ZShift = 10.28
Z lm Score = 3.57
NG = 0
DB = 0
SM = 0
Delta_L
Cytarabine
YLR418C CDC73
ZShift = −15.4
Z lm Score = −6.51
NG = 0
DB = 0
SM = 0
Delta_K
Cytarabine
YLR418C CDC73
ZShift = −5.46
Z lm Score = 2.34
NG = 0
DB = 0
SM = 0
Delta_r
Cytarabine
YLR418C CDC73
ZShift = −12.12
Z lm Score = 0.14
NG = 0
DB = 0
SM = 0
Delta_AUC
Cytarabine

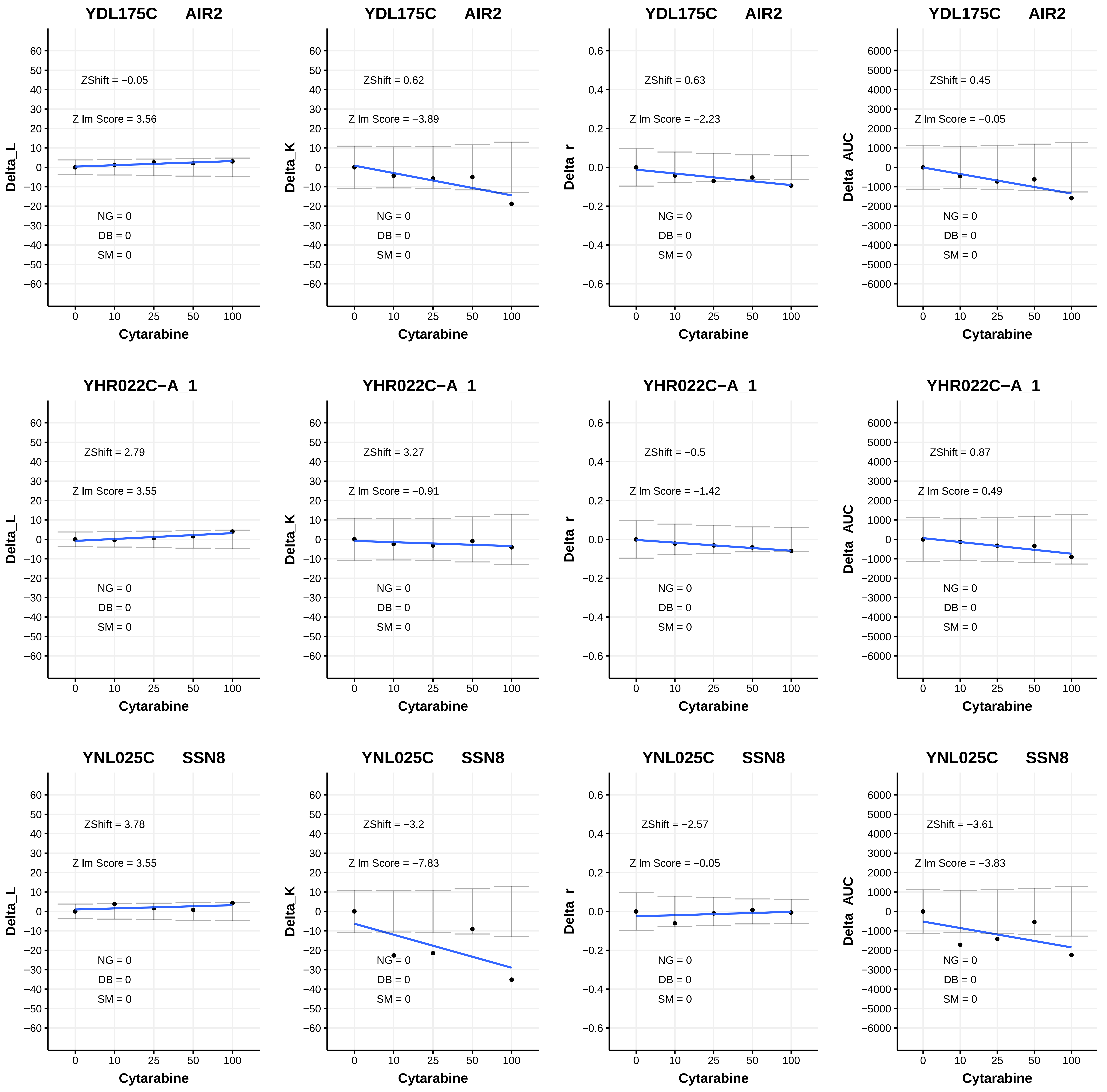

YDL175C AIR2
ZShift = −0.05
Z lm Score = 3.56
NG = 0
DB = 0
SM = 0
Delta_L
Cytarabine
YDL175C AIR2
ZShift = 0.62
Z lm Score = −3.89
NG = 0
DB = 0
SM = 0
Delta_K
Cytarabine
YDL175C AIR2
ZShift = 0.63
Z lm Score = −2.23
NG = 0
DB = 0
SM = 0
Delta_r
Cytarabine
YDL175C AIR2
ZShift = 0.45
Z lm Score = −0.05
NG = 0
DB = 0
SM = 0
Delta_AUC
Cytarabine
YHR022C−A_1
ZShift = 2.79
Z lm Score = 3.55
NG = 0
DB = 0
SM = 0
Delta_L
Cytarabine
YHR022C−A_1
ZShift = 3.27
Z lm Score = −0.91
NG = 0
DB = 0
SM = 0
Delta_K
Cytarabine
YHR022C−A_1
ZShift = −0.5
Z lm Score = −1.42
NG = 0
DB = 0
SM = 0
Delta_r
Cytarabine
YHR022C−A_1
ZShift = 0.87
Z lm Score = 0.49
NG = 0
DB = 0
SM = 0
Delta_AUC
Cytarabine
YNL025C SSN8
ZShift = 3.78
Z lm Score = 3.55
NG = 0
DB = 0
SM = 0
Delta_L
Cytarabine
YNL025C SSN8
ZShift = −3.2
Z lm Score = −7.83
NG = 0
DB = 0
SM = 0
Delta_K
Cytarabine
YNL025C SSN8
ZShift = −2.57
Z lm Score = −0.05
NG = 0
DB = 0
SM = 0
Delta_r
Cytarabine
YNL025C SSN8
ZShift = −3.61
Z lm Score = −3.83
NG = 0
DB = 0
SM = 0
Delta_AUC
Cytarabine

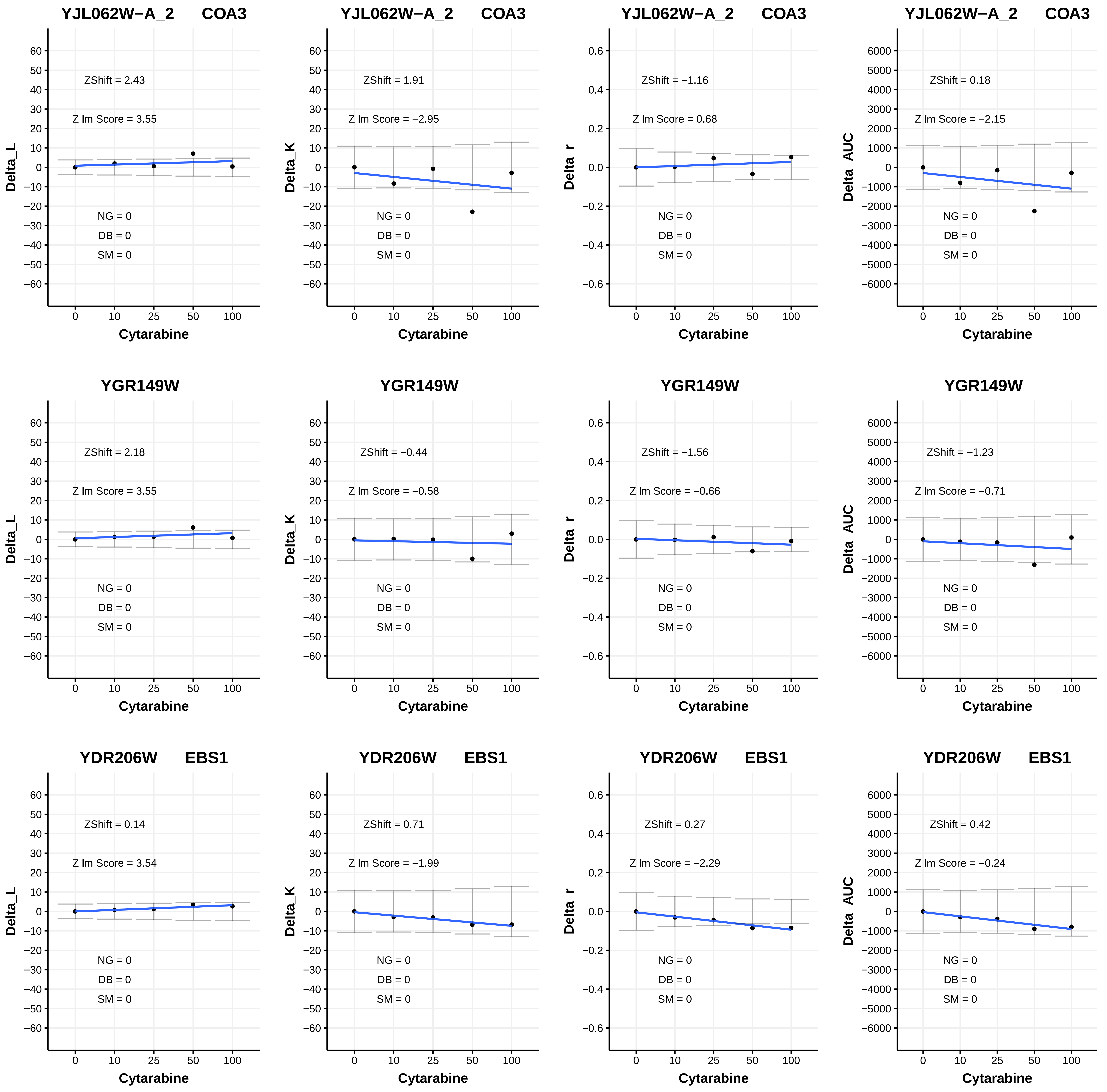
YJL062W−A_2 COA3
ZShift = 2.43
Z lm Score = 3.55
NG = 0
DB = 0
SM = 0
Delta_L
Cytarabine
YJL062W−A_2 COA3
ZShift = 1.91
Z lm Score = −2.95
NG = 0
DB = 0
SM = 0
Delta_K
Cytarabine
YJL062W−A_2 COA3
ZShift = −1.16
Z lm Score = 0.68
NG = 0
DB = 0
SM = 0
Delta_r
Cytarabine
YJL062W−A_2 COA3
ZShift = 0.18
Z lm Score = −2.15
NG = 0
DB = 0
SM = 0
Delta_AUC
Cytarabine
YGR149W
ZShift = 2.18
Z lm Score = 3.55
NG = 0
DB = 0
SM = 0
Delta_L
Cytarabine
YGR149W
ZShift = −0.44
Z lm Score = −0.58
NG = 0
DB = 0
SM = 0
Delta_K
Cytarabine
YGR149W
ZShift = −1.56
Z lm Score = −0.66
NG = 0
DB = 0
SM = 0
Delta_r
Cytarabine
YGR149W
ZShift = −1.23
Z lm Score = −0.71
NG = 0
DB = 0
SM = 0
Delta_AUC
Cytarabine
YDR206W EBS1
ZShift = 0.14
Z lm Score = 3.54
NG = 0
DB = 0
SM = 0
Delta_L
Cytarabine
YDR206W EBS1
ZShift = 0.71
Z lm Score = −1.99
NG = 0
DB = 0
SM = 0
Delta_K
Cytarabine
YDR206W EBS1
ZShift = 0.27
Z lm Score = −2.29
NG = 0
DB = 0
SM = 0
Delta_r
Cytarabine
YDR206W EBS1
ZShift = 0.42
Z lm Score = −0.24
NG = 0
DB = 0
SM = 0
Delta_AUC
Cytarabine

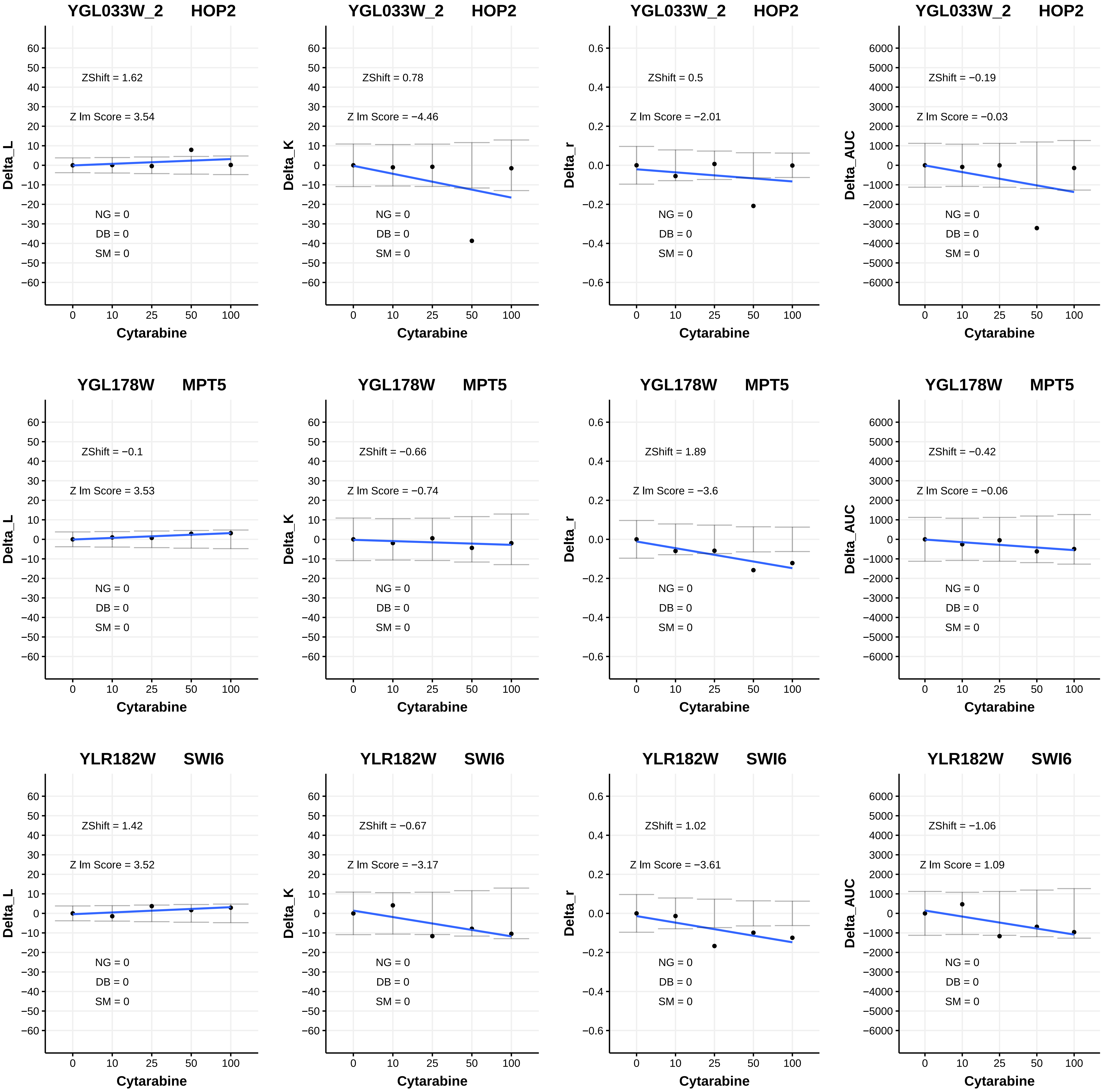

YGL033W_2 HOP2
ZShift = 1.62
Z lm Score = 3.54
NG = 0
DB = 0
SM = 0
Delta_L
Cytarabine
YGL033W_2 HOP2
ZShift = 0.78
Z lm Score = −4.46
NG = 0
DB = 0
SM = 0
Delta_K
Cytarabine
YGL033W_2 HOP2
ZShift = 0.5
Z lm Score = −2.01
NG = 0
DB = 0
SM = 0
Delta_r
Cytarabine
YGL033W_2 HOP2
ZShift = −0.19
Z lm Score = −0.03
NG = 0
DB = 0
SM = 0
Delta_AUC
Cytarabine
YGL178W MPT5
ZShift = −0.1
Z lm Score = 3.53
NG = 0
DB = 0
SM = 0
Delta_L
Cytarabine
YGL178W MPT5
ZShift = −0.66
Z lm Score = −0.74
NG = 0
DB = 0
SM = 0
Delta_K
Cytarabine
YGL178W MPT5
ZShift = 1.89
Z lm Score = −3.6
NG = 0
DB = 0
SM = 0
Delta_r
Cytarabine
YGL178W MPT5
ZShift = −0.42
Z lm Score = −0.06
NG = 0
DB = 0
SM = 0
Delta_AUC
Cytarabine
YLR182W SWI6
ZShift = 1.42
Z lm Score = 3.52
NG = 0
DB = 0
SM = 0
Delta_L
Cytarabine
YLR182W SWI6
ZShift = −0.67
Z lm Score = −3.17
NG = 0
DB = 0
SM = 0
Delta_K
Cytarabine
YLR182W SWI6
ZShift = 1.02
Z lm Score = −3.61
NG = 0
DB = 0
SM = 0
Delta_r
Cytarabine
YLR182W SWI6
ZShift = −1.06
Z lm Score = 1.09
NG = 0
DB = 0
SM = 0
Delta_AUC
Cytarabine

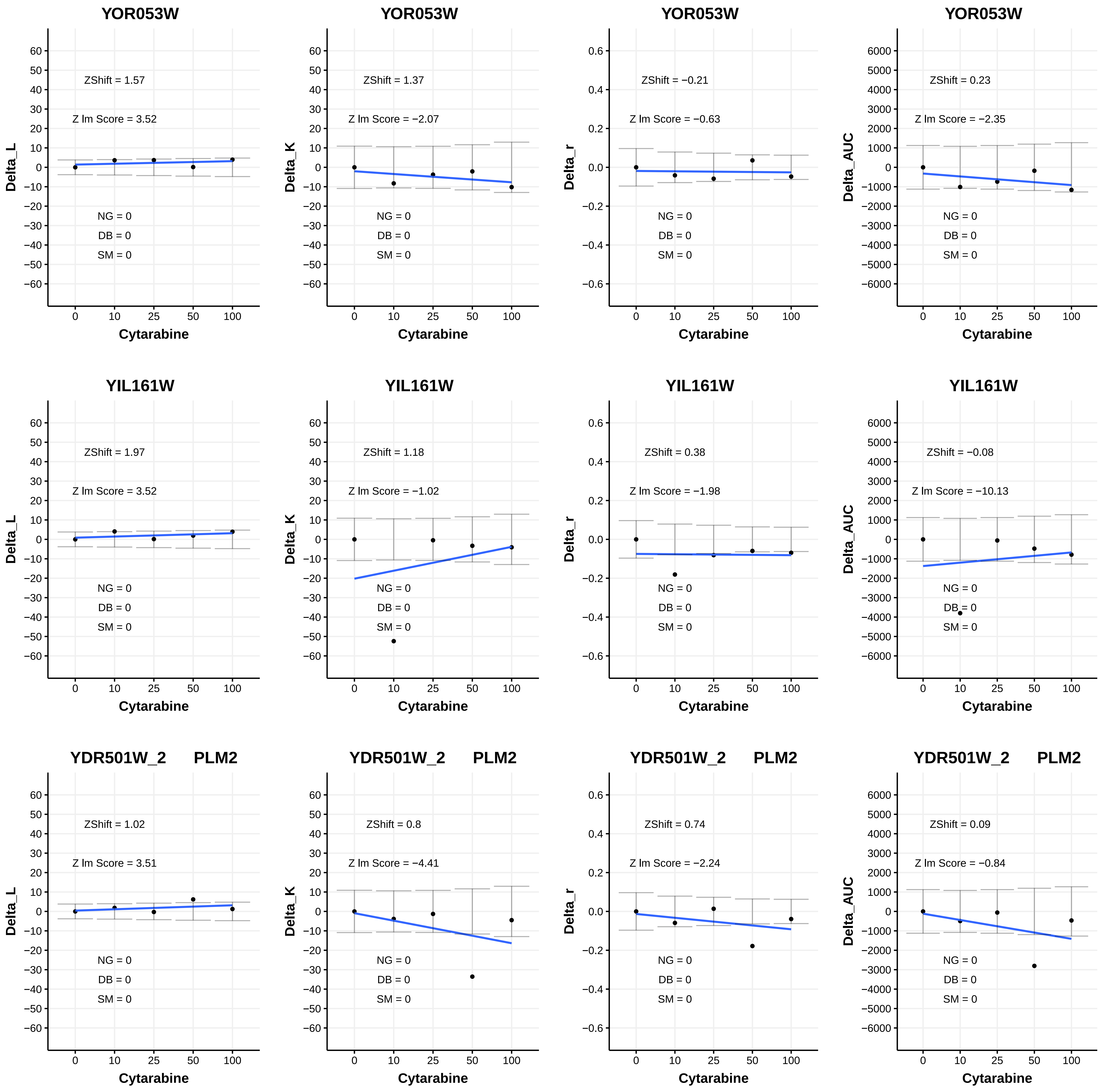

YOR053W
ZShift = 1.57
Z lm Score = 3.52
NG = 0
DB = 0
SM = 0
Delta_L
Cytarabine
YOR053W
ZShift = 1.37
Z lm Score = −2.07
NG = 0
DB = 0
SM = 0
Delta_K
Cytarabine
YOR053W
ZShift = −0.21
Z lm Score = −0.63
NG = 0
DB = 0
SM = 0
Delta_r
Cytarabine
YOR053W
ZShift = 0.23
Z lm Score = −2.35
NG = 0
DB = 0
SM = 0
Delta_AUC
Cytarabine
YIL161W
ZShift = 1.97
Z lm Score = 3.52
NG = 0
DB = 0
SM = 0
Delta_L
Cytarabine
YIL161W
ZShift = 1.18
Z lm Score = −1.02
NG = 0
DB = 0
SM = 0
Delta_K
Cytarabine
YIL161W
ZShift = 0.38
Z lm Score = −1.98
NG = 0
DB = 0
SM = 0
Delta_r
Cytarabine
YIL161W
ZShift = −0.08
Z lm Score = −10.13
NG = 0
DB = 0
SM = 0
Delta_AUC
Cytarabine
YDR501W_2 PLM2
ZShift = 1.02
Z lm Score = 3.51
NG = 0
DB = 0
SM = 0
Delta_L
Cytarabine
YDR501W_2 PLM2
ZShift = 0.8
Z lm Score = −4.41
NG = 0
DB = 0
SM = 0
Delta_K
Cytarabine
YDR501W_2 PLM2
ZShift = 0.74
Z lm Score = −2.24
NG = 0
DB = 0
SM = 0
Delta_r
Cytarabine
YDR501W_2 PLM2
ZShift = 0.09
Z lm Score = −0.84
NG = 0
DB = 0
SM = 0
Delta_AUC
Cytarabine

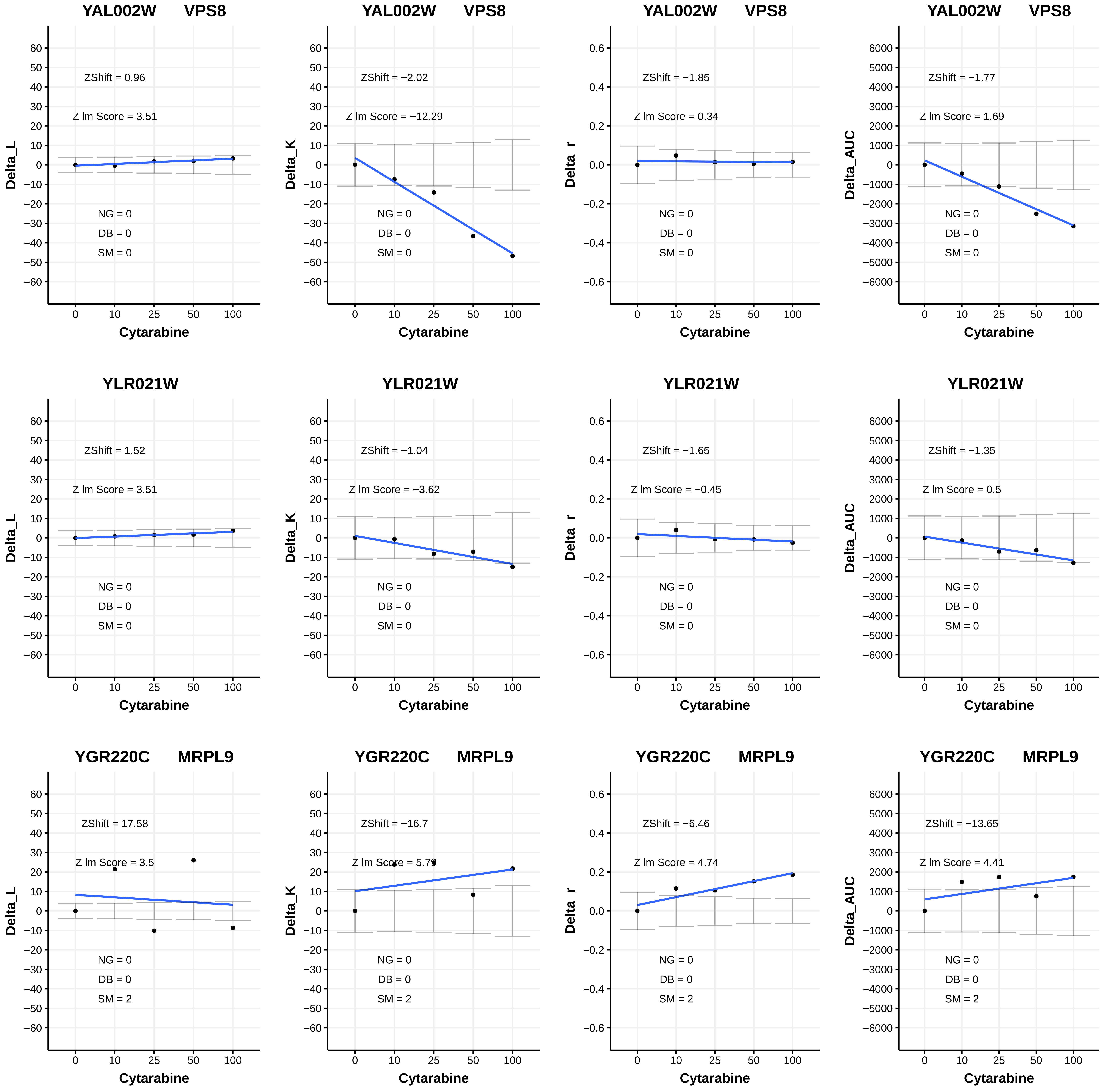

YAL002W VPS8
ZShift = 0.96
Z lm Score = 3.51
NG = 0
DB = 0
SM = 0
Delta_L
Cytarabine
YAL002W VPS8
ZShift = −2.02
Z lm Score = −12.29
NG = 0
DB = 0
SM = 0
Delta_K
Cytarabine
YAL002W VPS8
ZShift = −1.85
Z lm Score = 0.34
NG = 0
DB = 0
SM = 0
Delta_r
Cytarabine
YAL002W VPS8
ZShift = −1.77
Z lm Score = 1.69
NG = 0
DB = 0
SM = 0
Delta_AUC
Cytarabine
YLR021W
ZShift = 1.52
Z lm Score = 3.51
NG = 0
DB = 0
SM = 0
Delta_L
Cytarabine
YLR021W
ZShift = −1.04
Z lm Score = −3.62
NG = 0
DB = 0
SM = 0
Delta_K
Cytarabine
YLR021W
ZShift = −1.65
Z lm Score = −0.45
NG = 0
DB = 0
SM = 0
Delta_r
Cytarabine
YLR021W
ZShift = −1.35
Z lm Score = 0.5
NG = 0
DB = 0
SM = 0
Delta_AUC
Cytarabine
YGR220C MRPL9
ZShift = 17.58
Z lm Score = 3.5
NG = 0
DB = 0
SM = 2
Delta_L
Cytarabine
YGR220C MRPL9
ZShift = −16.7
Z lm Score = 5.79
NG = 0
DB = 0
SM = 2
Delta_K
Cytarabine
YGR220C MRPL9
ZShift = −6.46
Z lm Score = 4.74
NG = 0
DB = 0
SM = 2
Delta_r
Cytarabine
YGR220C MRPL9
ZShift = −13.65
Z lm Score = 4.41
NG = 0
DB = 0
SM = 2
Delta_AUC
Cytarabine

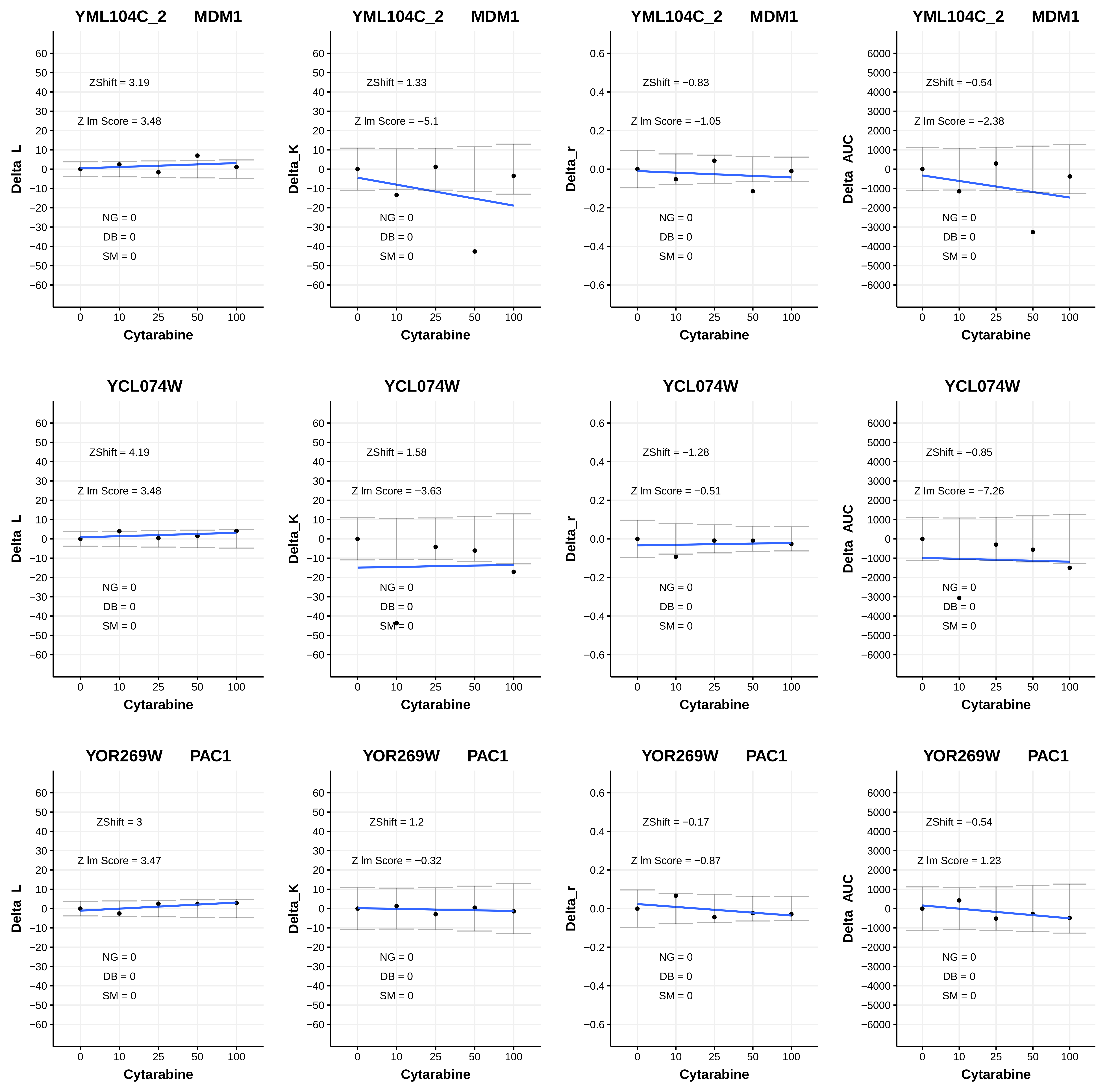
YML104C_2 MDM1
ZShift = 3.19
Z lm Score = 3.48
NG = 0
DB = 0
SM = 0
Delta_L
Cytarabine
YML104C_2 MDM1
ZShift = 1.33
Z lm Score = −5.1
NG = 0
DB = 0
SM = 0
Delta_K
Cytarabine
YML104C_2 MDM1
ZShift = −0.83
Z lm Score = −1.05
NG = 0
DB = 0
SM = 0
Delta_r
Cytarabine
YML104C_2 MDM1
ZShift = −0.54
Z lm Score = −2.38
NG = 0
DB = 0
SM = 0
Delta_AUC
Cytarabine
YCL074W
ZShift = 4.19
Z lm Score = 3.48
NG = 0
DB = 0
SM = 0
Delta_L
Cytarabine
YCL074W
ZShift = 1.58
Z lm Score = −3.63
NG = 0
DB = 0
SM = 0
Delta_K
Cytarabine
YCL074W
ZShift = −1.28
Z lm Score = −0.51
NG = 0
DB = 0
SM = 0
Delta_r
Cytarabine
YCL074W
ZShift = −0.85
Z lm Score = −7.26
NG = 0
DB = 0
SM = 0
Delta_AUC
Cytarabine
YOR269W PAC1
ZShift = 3
Z lm Score = 3.47
NG = 0
DB = 0
SM = 0
Delta_L
Cytarabine
YOR269W PAC1
ZShift = 1.2
Z lm Score = −0.32
NG = 0
DB = 0
SM = 0
Delta_K
Cytarabine
YOR269W PAC1
ZShift = −0.17
Z lm Score = −0.87
NG = 0
DB = 0
SM = 0
Delta_r
Cytarabine
YOR269W PAC1
ZShift = −0.54
Z lm Score = 1.23
NG = 0
DB = 0
SM = 0
Delta_AUC
Cytarabine

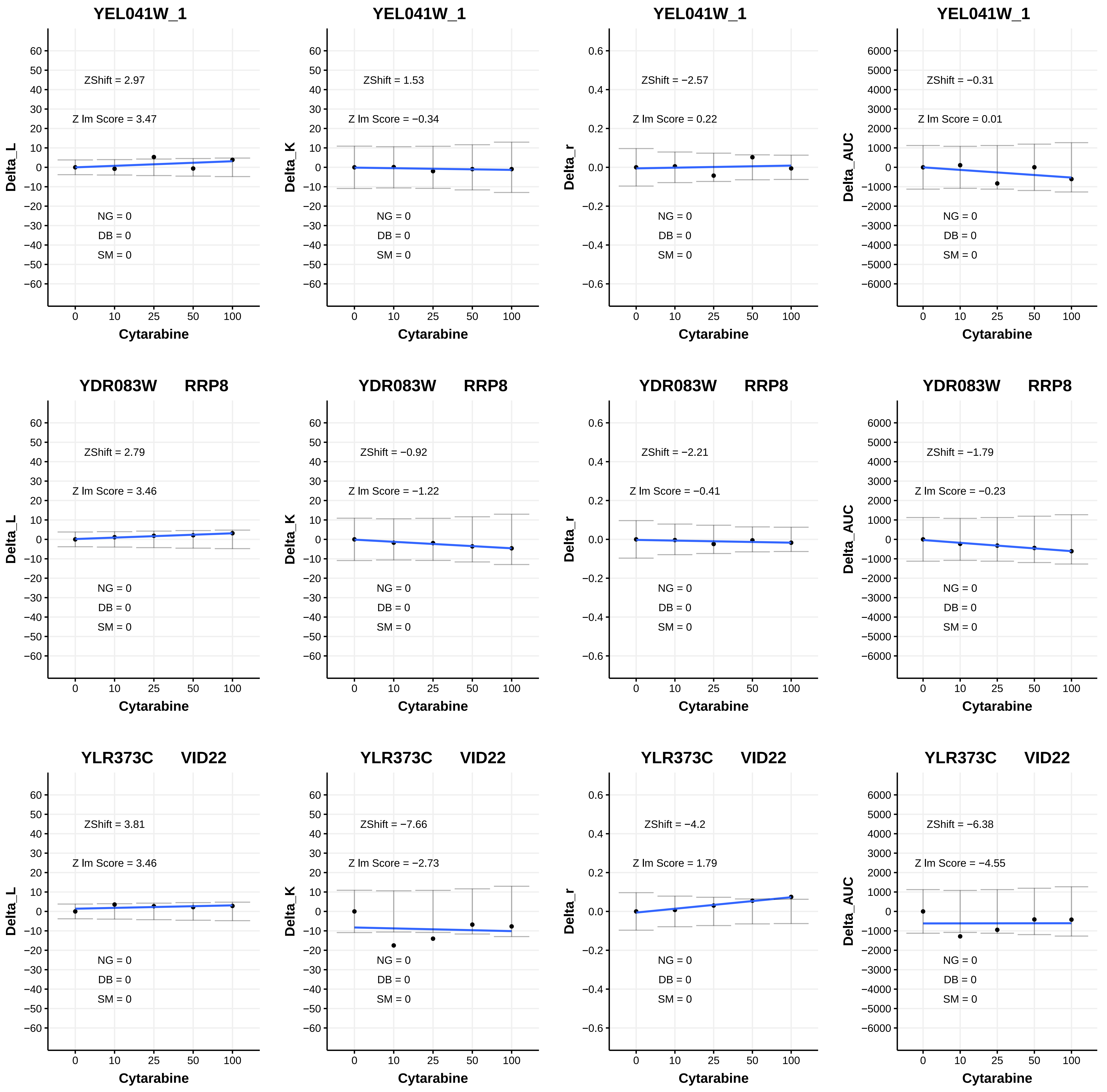
YEL041W_1
ZShift = 2.97
Z lm Score = 3.47
NG = 0
DB = 0
SM = 0
Delta_L
Cytarabine
YEL041W_1
ZShift = 1.53
Z lm Score = −0.34
NG = 0
DB = 0
SM = 0
Delta_K
Cytarabine
YEL041W_1
ZShift = −2.57
Z lm Score = 0.22
NG = 0
DB = 0
SM = 0
Delta_r
Cytarabine
YEL041W_1
ZShift = −0.31
Z lm Score = 0.01
NG = 0
DB = 0
SM = 0
Delta_AUC
Cytarabine
YDR083W RRP8
ZShift = 2.79
Z lm Score = 3.46
NG = 0
DB = 0
SM = 0
Delta_L
Cytarabine
YDR083W RRP8
ZShift = −0.92
Z lm Score = −1.22
NG = 0
DB = 0
SM = 0
Delta_K
Cytarabine
YDR083W RRP8
ZShift = −2.21
Z lm Score = −0.41
NG = 0
DB = 0
SM = 0
Delta_r
Cytarabine
YDR083W RRP8
ZShift = −1.79
Z lm Score = −0.23
NG = 0
DB = 0
SM = 0
Delta_AUC
Cytarabine
YLR373C VID22
ZShift = 3.81
Z lm Score = 3.46
NG = 0
DB = 0
SM = 0
Delta_L
Cytarabine
YLR373C VID22
ZShift = −7.66
Z lm Score = −2.73
NG = 0
DB = 0
SM = 0
Delta_K
Cytarabine
YLR373C VID22
ZShift = −4.2
Z lm Score = 1.79
NG = 0
DB = 0
SM = 0
Delta_r
Cytarabine
YLR373C VID22
ZShift = −6.38
Z lm Score = −4.55
NG = 0
DB = 0
SM = 0
Delta_AUC
Cytarabine

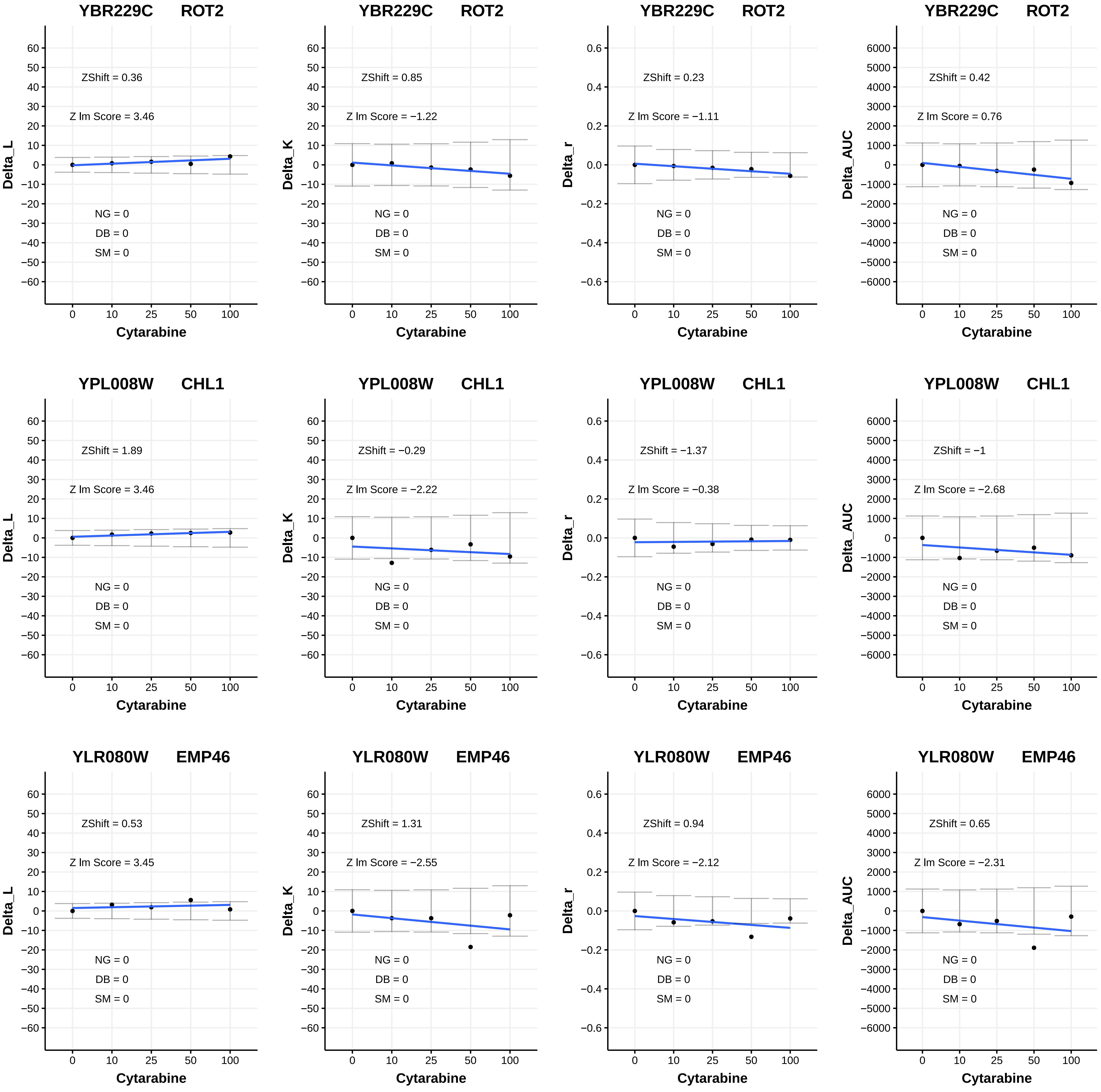
YBR229C ROT2
ZShift = 0.36
Z lm Score = 3.46
NG = 0
DB = 0
SM = 0
Delta_L
Cytarabine
YBR229C ROT2
ZShift = 0.85
Z lm Score = −1.22
NG = 0
DB = 0
SM = 0
Delta_K
Cytarabine
YBR229C ROT2
ZShift = 0.23
Z lm Score = −1.11
NG = 0
DB = 0
SM = 0
Delta_r
Cytarabine
YBR229C ROT2
ZShift = 0.42
Z lm Score = 0.76
NG = 0
DB = 0
SM = 0
Delta_AUC
Cytarabine
YPL008W CHL1
ZShift = 1.89
Z lm Score = 3.46
NG = 0
DB = 0
SM = 0
Delta_L
Cytarabine
YPL008W CHL1
ZShift = −0.29
Z lm Score = −2.22
NG = 0
DB = 0
SM = 0
Delta_K
Cytarabine
YPL008W CHL1
ZShift = −1.37
Z lm Score = −0.38
NG = 0
DB = 0
SM = 0
Delta_r
Cytarabine
YPL008W CHL1
ZShift = −1
Z lm Score = −2.68
NG = 0
DB = 0
SM = 0
Delta_AUC
Cytarabine
YLR080W EMP46
ZShift = 0.53
Z lm Score = 3.45
NG = 0
DB = 0
SM = 0
Delta_L
Cytarabine
YLR080W EMP46
ZShift = 1.31
Z lm Score = −2.55
NG = 0
DB = 0
SM = 0
Delta_K
Cytarabine
YLR080W EMP46
ZShift = 0.94
Z lm Score = −2.12
NG = 0
DB = 0
SM = 0
Delta_r
Cytarabine
YLR080W EMP46
ZShift = 0.65
Z lm Score = −2.31
NG = 0
DB = 0
SM = 0
Delta_AUC
Cytarabine

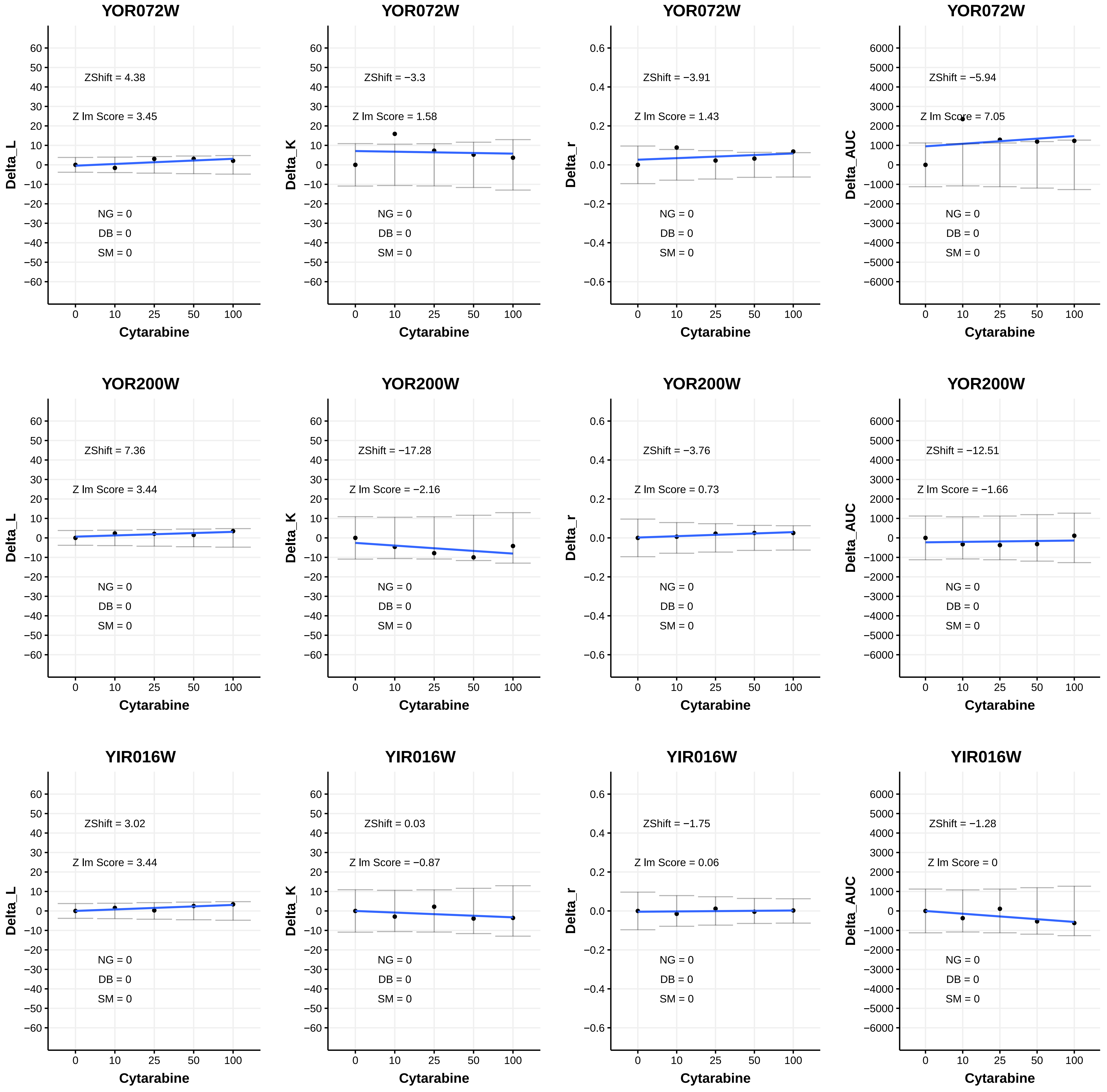

YOR072W
Delta_L
Cytarabine
ZShift = 4.38
Z lm Score = 3.45
NG = 0
DB = 0
SM = 0
YOR072W
Delta_K
Cytarabine
ZShift = −3.3
Z lm Score = 1.58
NG = 0
DB = 0
SM = 0
YOR072W
Delta_r
Cytarabine
ZShift = −3.91
Z lm Score = 1.43
NG = 0
DB = 0
SM = 0
YOR072W
Delta_AUC
Cytarabine
ZShift = −5.94
Z lm Score = 7.05
NG = 0
DB = 0
SM = 0
YOR200W
Delta_L
Cytarabine
ZShift = 7.36
Z lm Score = 3.44
NG = 0
DB = 0
SM = 0
YOR200W
Delta_K
Cytarabine
ZShift = −17.28
Z lm Score = −2.16
NG = 0
DB = 0
SM = 0
YOR200W
Delta_r
Cytarabine
ZShift = −3.76
Z lm Score = 0.73
NG = 0
DB = 0
SM = 0
YOR200W
Delta_AUC
Cytarabine
ZShift = −12.51
Z lm Score = −1.66
NG = 0
DB = 0
SM = 0
YIR016W
Delta_L
Cytarabine
ZShift = 3.02
Z lm Score = 3.44
NG = 0
DB = 0
SM = 0
YIR016W
Delta_K
Cytarabine
ZShift = 0.03
Z lm Score = −0.87
NG = 0
DB = 0
SM = 0
YIR016W
Delta_r
Cytarabine
ZShift = −1.75
Z lm Score = 0.06
NG = 0
DB = 0
SM = 0
YIR016W
Delta_AUC
Cytarabine
ZShift = −1.28
Z lm Score = 0
NG = 0
DB = 0
SM = 0

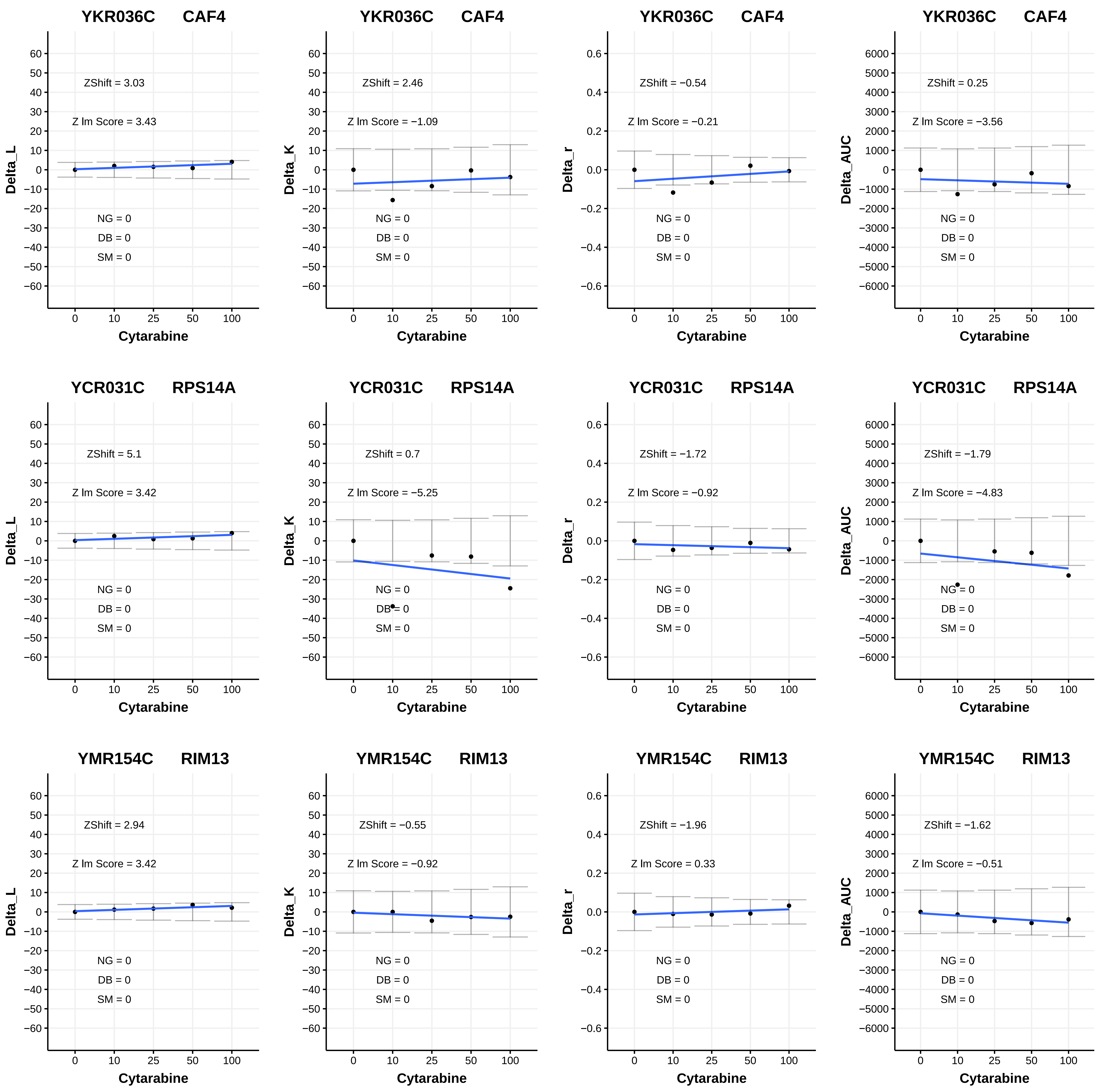

YKR036C CAF4
ZShift = 3.03
Z lm Score = 3.43
NG = 0
DB = 0
SM = 0
Delta_L
Cytarabine
YKR036C CAF4
ZShift = 2.46
Z lm Score = −1.09
NG = 0
DB = 0
SM = 0
Delta_K
Cytarabine
YKR036C CAF4
ZShift = −0.54
Z lm Score = −0.21
NG = 0
DB = 0
SM = 0
Delta_r
Cytarabine
YKR036C CAF4
ZShift = 0.25
Z lm Score = −3.56
NG = 0
DB = 0
SM = 0
Delta_AUC
Cytarabine
YCR031C RPS14A
ZShift = 5.1
Z lm Score = 3.42
NG = 0
DB = 0
SM = 0
Delta_L
Cytarabine
YCR031C RPS14A
ZShift = 0.7
Z lm Score = −5.25
NG = 0
DB = 0
SM = 0
Delta_K
Cytarabine
YCR031C RPS14A
ZShift = −1.72
Z lm Score = −0.92
NG = 0
DB = 0
SM = 0
Delta_r
Cytarabine
YCR031C RPS14A
ZShift = −1.79
Z lm Score = −4.83
NG = 0
DB = 0
SM = 0
Delta_AUC
Cytarabine
YMR154C RIM13
ZShift = 2.94
Z lm Score = 3.42
NG = 0
DB = 0
SM = 0
Delta_L
Cytarabine
YMR154C RIM13
ZShift = −0.55
Z lm Score = −0.92
NG = 0
DB = 0
SM = 0
Delta_K
Cytarabine
YMR154C RIM13
ZShift = −1.96
Z lm Score = 0.33
NG = 0
DB = 0
SM = 0
Delta_r
Cytarabine
YMR154C RIM13
ZShift = −1.62
Z lm Score = −0.51
NG = 0
DB = 0
SM = 0
Delta_AUC
Cytarabine

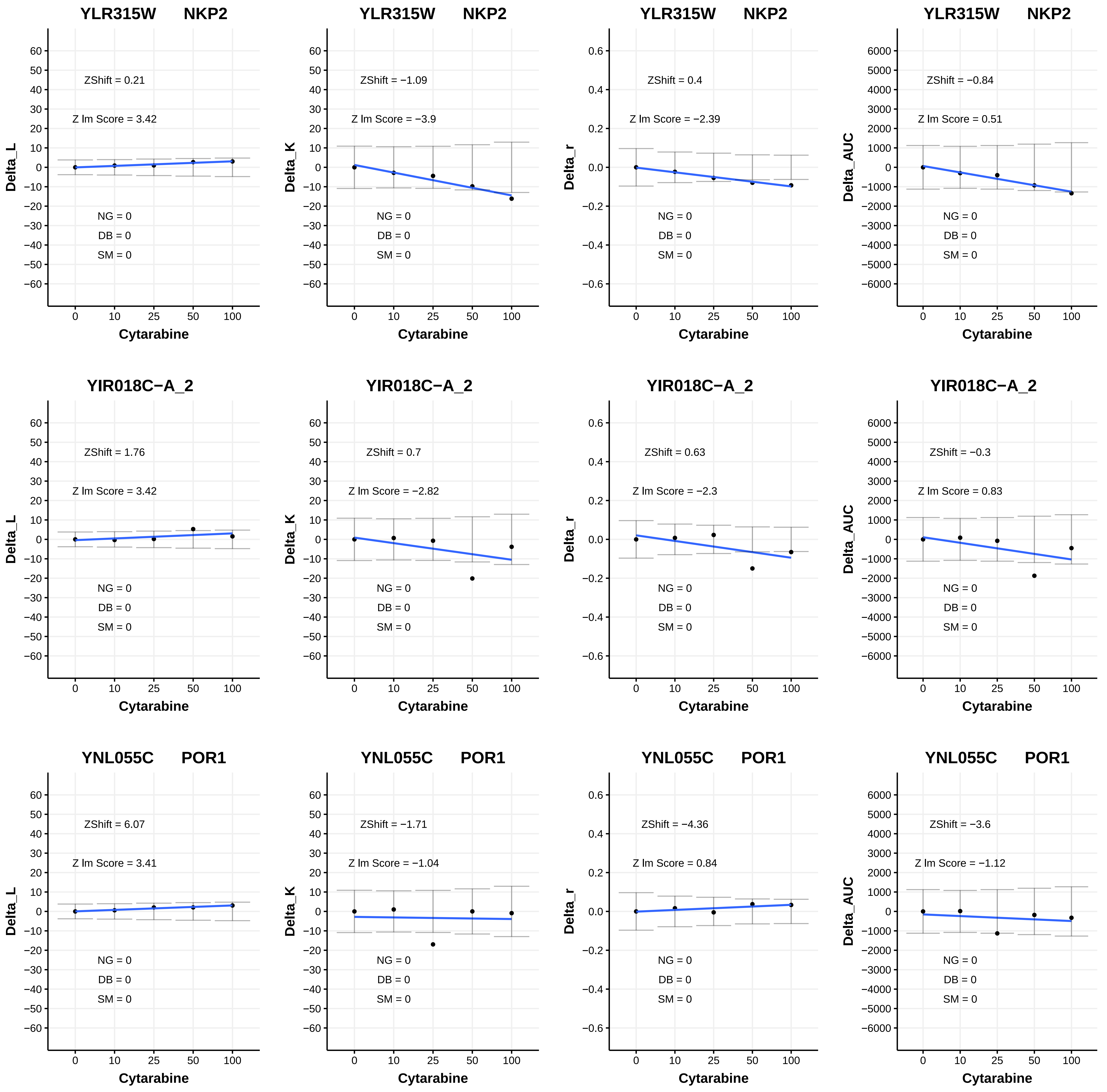
YLR315W NKP2
ZShift = 0.21
Z lm Score = 3.42
NG = 0
DB = 0
SM = 0
Delta_L
Cytarabine
YLR315W NKP2
ZShift = −1.09
Z lm Score = −3.9
NG = 0
DB = 0
SM = 0
Delta_K
Cytarabine
YLR315W NKP2
ZShift = 0.4
Z lm Score = −2.39
NG = 0
DB = 0
SM = 0
Delta_r
Cytarabine
YLR315W NKP2
ZShift = −0.84
Z lm Score = 0.51
NG = 0
DB = 0
SM = 0
Delta_AUC
Cytarabine
YIR018C−A_2
ZShift = 1.76
Z lm Score = 3.42
NG = 0
DB = 0
SM = 0
Delta_L
Cytarabine
YIR018C−A_2
ZShift = 0.7
Z lm Score = −2.82
NG = 0
DB = 0
SM = 0
Delta_K
Cytarabine
YIR018C−A_2
ZShift = 0.63
Z lm Score = −2.3
NG = 0
DB = 0
SM = 0
Delta_r
Cytarabine
YIR018C−A_2
ZShift = −0.3
Z lm Score = 0.83
NG = 0
DB = 0
SM = 0
Delta_AUC
Cytarabine
YNL055C POR1
ZShift = 6.07
Z lm Score = 3.41
NG = 0
DB = 0
SM = 0
Delta_L
Cytarabine
YNL055C POR1
ZShift = −1.71
Z lm Score = −1.04
NG = 0
DB = 0
SM = 0
Delta_K
Cytarabine
YNL055C POR1
ZShift = −4.36
Z lm Score = 0.84
NG = 0
DB = 0
SM = 0
Delta_r
Cytarabine
YNL055C POR1
ZShift = −3.6
Z lm Score = −1.12
NG = 0
DB = 0
SM = 0
Delta_AUC
Cytarabine

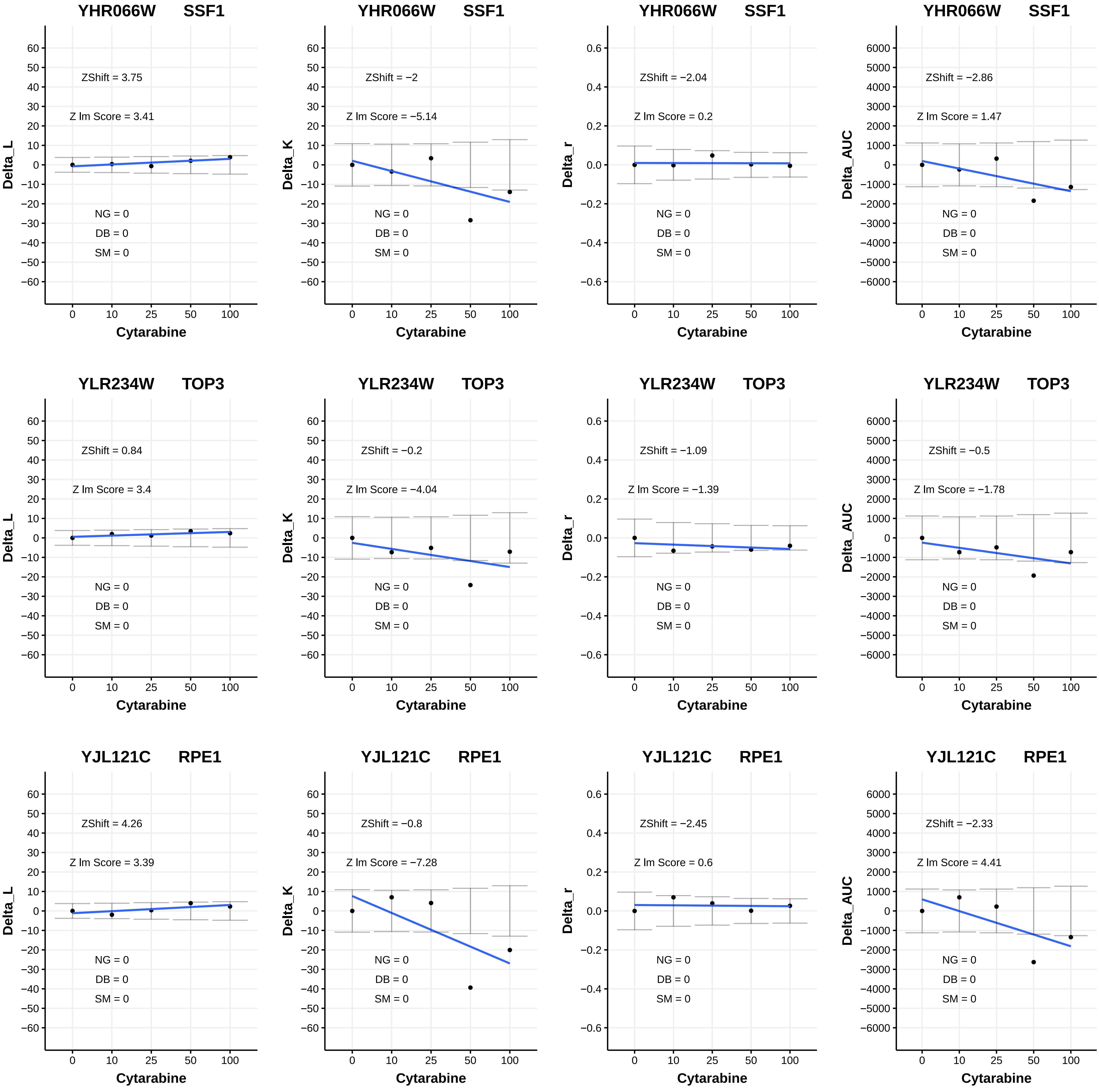
YHR066W SSF1
ZShift = 3.75
Z lm Score = 3.41
NG = 0
DB = 0
SM = 0
Delta_L
Cytarabine
YHR066W SSF1
ZShift = −2
Z lm Score = −5.14
NG = 0
DB = 0
SM = 0
Delta_K
Cytarabine
YHR066W SSF1
ZShift = −2.04
Z lm Score = 0.2
NG = 0
DB = 0
SM = 0
Delta_r
Cytarabine
YHR066W SSF1
ZShift = −2.86
Z lm Score = 1.47
NG = 0
DB = 0
SM = 0
Delta_AUC
Cytarabine
YLR234W TOP3
ZShift = 0.84
Z lm Score = 3.4
NG = 0
DB = 0
SM = 0
Delta_L
Cytarabine
YLR234W TOP3
ZShift = −0.2
Z lm Score = −4.04
NG = 0
DB = 0
SM = 0
Delta_K
Cytarabine
YLR234W TOP3
ZShift = −1.09
Z lm Score = −1.39
NG = 0
DB = 0
SM = 0
Delta_r
Cytarabine
YLR234W TOP3
ZShift = −0.5
Z lm Score = −1.78
NG = 0
DB = 0
SM = 0
Delta_AUC
Cytarabine
YJL121C RPE1
ZShift = 4.26
Z lm Score = 3.39
NG = 0
DB = 0
SM = 0
Delta_L
Cytarabine
YJL121C RPE1
ZShift = −0.8
Z lm Score = −7.28
NG = 0
DB = 0
SM = 0
Delta_K
Cytarabine
YJL121C RPE1
ZShift = −2.45
Z lm Score = 0.6
NG = 0
DB = 0
SM = 0
Delta_r
Cytarabine
YJL121C RPE1
ZShift = −2.33
Z lm Score = 4.41
NG = 0
DB = 0
SM = 0
Delta_AUC
Cytarabine

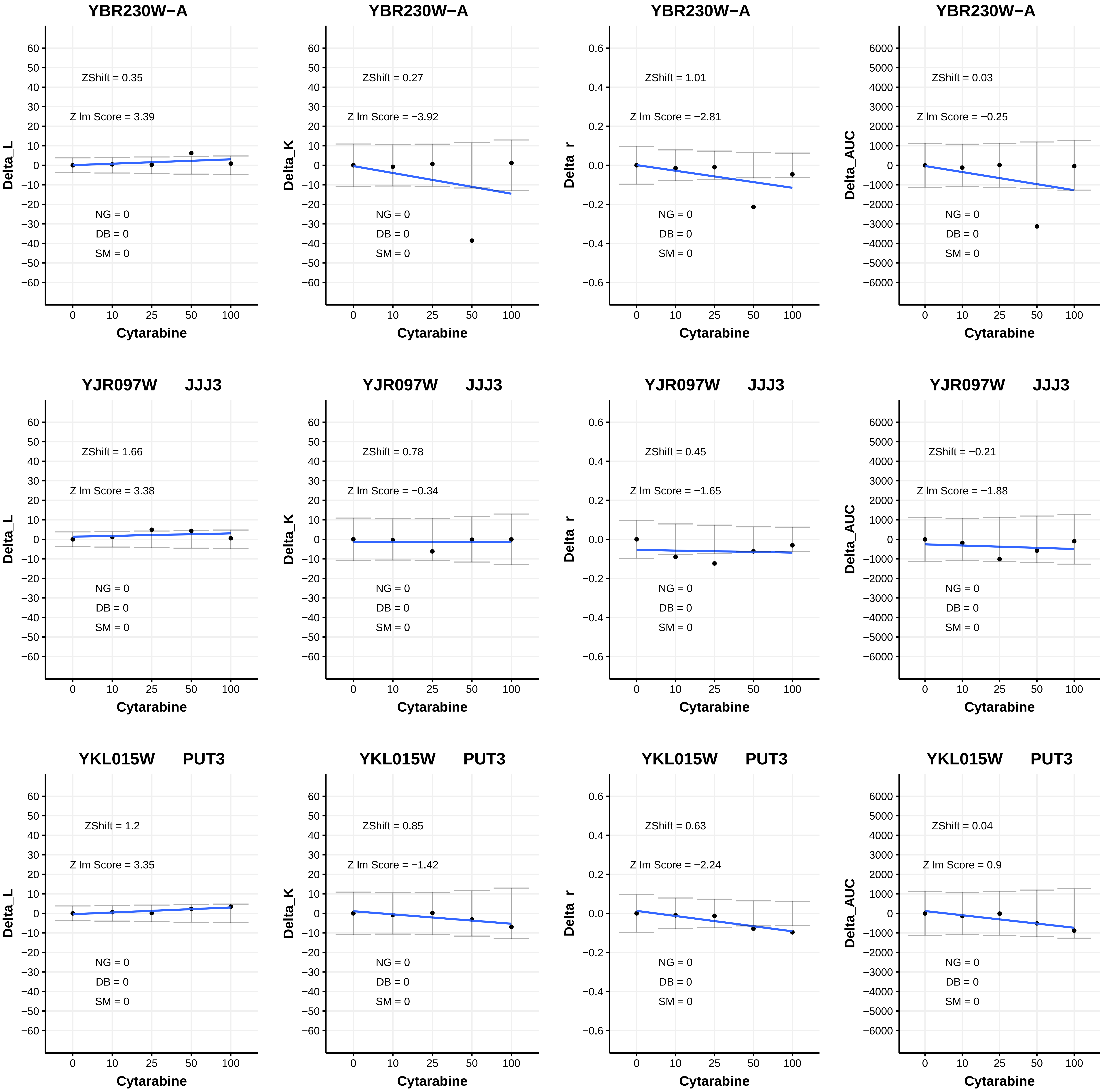

YBR230W−A
ZShift = 0.35
Z lm Score = 3.39
NG = 0
DB = 0
SM = 0
Delta_L
Cytarabine
YBR230W−A
ZShift = 0.27
Z lm Score = −3.92
NG = 0
DB = 0
SM = 0
Delta_K
Cytarabine
YBR230W−A
ZShift = 1.01
Z lm Score = −2.81
NG = 0
DB = 0
SM = 0
Delta_r
Cytarabine
YBR230W−A
ZShift = 0.03
Z lm Score = −0.25
NG = 0
DB = 0
SM = 0
Delta_AUC
Cytarabine
YJR097W JJJ3
ZShift = 1.66
Z lm Score = 3.38
NG = 0
DB = 0
SM = 0
Delta_L
Cytarabine
YJR097W JJJ3
ZShift = 0.78
Z lm Score = −0.34
NG = 0
DB = 0
SM = 0
Delta_K
Cytarabine
YJR097W JJJ3
ZShift = 0.45
Z lm Score = −1.65
NG = 0
DB = 0
SM = 0
Delta_r
Cytarabine
YJR097W JJJ3
ZShift = −0.21
Z lm Score = −1.88
NG = 0
DB = 0
SM = 0
Delta_AUC
Cytarabine
YKL015W PUT3
ZShift = 1.2
Z lm Score = 3.35
NG = 0
DB = 0
SM = 0
Delta_L
Cytarabine
YKL015W PUT3
ZShift = 0.85
Z lm Score = −1.42
NG = 0
DB = 0
SM = 0
Delta_K
Cytarabine
YKL015W PUT3
ZShift = 0.63
Z lm Score = −2.24
NG = 0
DB = 0
SM = 0
Delta_r
Cytarabine
YKL015W PUT3
ZShift = 0.04
Z lm Score = 0.9
NG = 0
DB = 0
SM = 0
Delta_AUC
Cytarabine

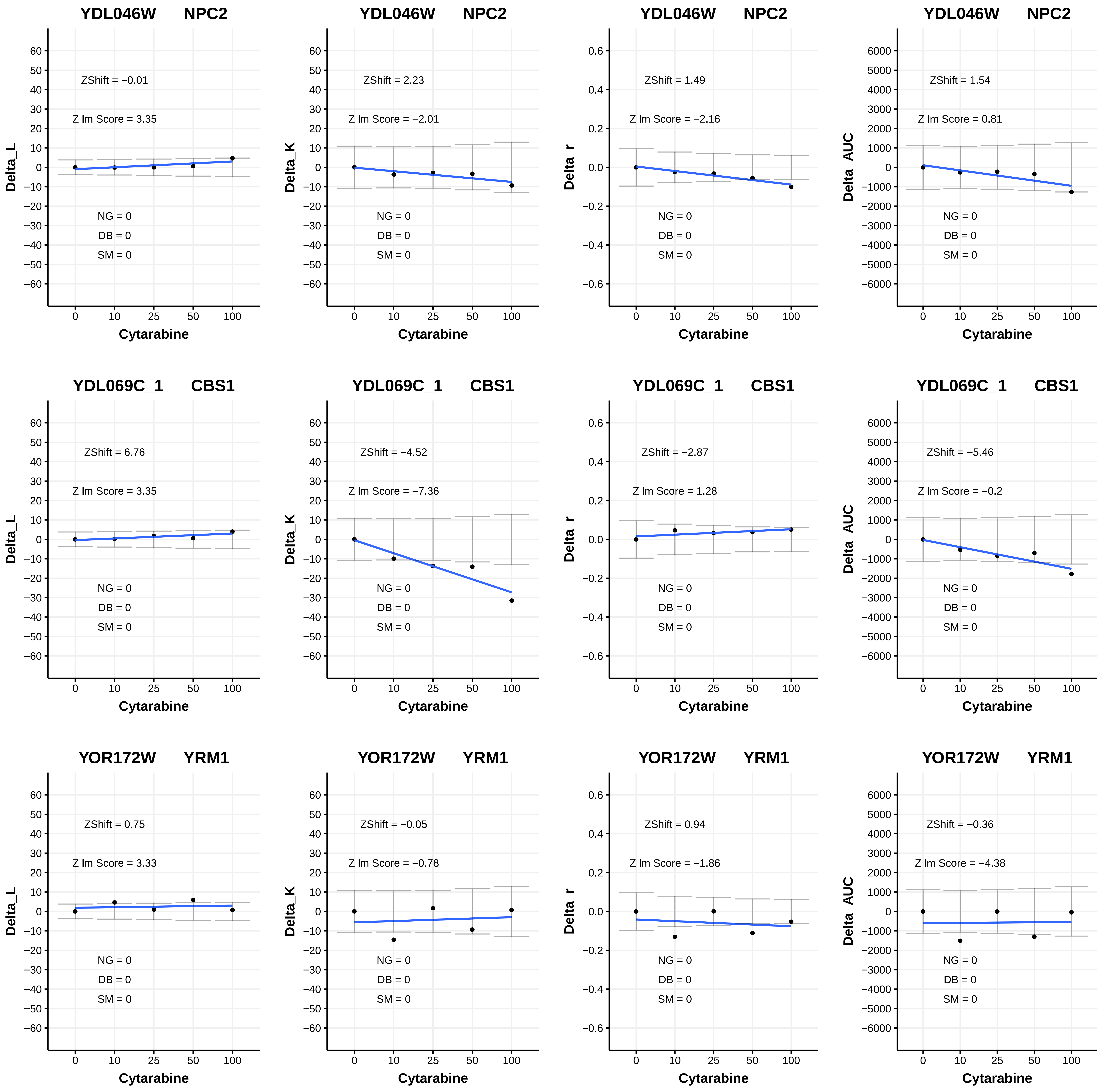
YDL046W NPC2
ZShift = −0.01
Z lm Score = 3.35
NG = 0
DB = 0
SM = 0
Delta_L
Cytarabine
YDL046W NPC2
ZShift = 2.23
Z lm Score = −2.01
NG = 0
DB = 0
SM = 0
Delta_K
Cytarabine
YDL046W NPC2
ZShift = 1.49
Z lm Score = −2.16
NG = 0
DB = 0
SM = 0
Delta_r
Cytarabine
YDL046W NPC2
ZShift = 1.54
Z lm Score = 0.81
NG = 0
DB = 0
SM = 0
Delta_AUC
Cytarabine
YDL069C_1 CBS1
ZShift = 6.76
Z lm Score = 3.35
NG = 0
DB = 0
SM = 0
Delta_L
Cytarabine
YDL069C_1 CBS1
ZShift = −4.52
Z lm Score = −7.36
NG = 0
DB = 0
SM = 0
Delta_K
Cytarabine
YDL069C_1 CBS1
ZShift = −2.87
Z lm Score = 1.28
NG = 0
DB = 0
SM = 0
Delta_r
Cytarabine
YDL069C_1 CBS1
ZShift = −5.46
Z lm Score = −0.2
NG = 0
DB = 0
SM = 0
Delta_AUC
Cytarabine
YOR172W YRM1
ZShift = 0.75
Z lm Score = 3.33
NG = 0
DB = 0
SM = 0
Delta_L
Cytarabine
YOR172W YRM1
ZShift = −0.05
Z lm Score = −0.78
NG = 0
DB = 0
SM = 0
Delta_K
Cytarabine
YOR172W YRM1
ZShift = 0.94
Z lm Score = −1.86
NG = 0
DB = 0
SM = 0
Delta_r
Cytarabine
YOR172W YRM1
ZShift = −0.36
Z lm Score = −4.38
NG = 0
DB = 0
SM = 0
Delta_AUC
Cytarabine

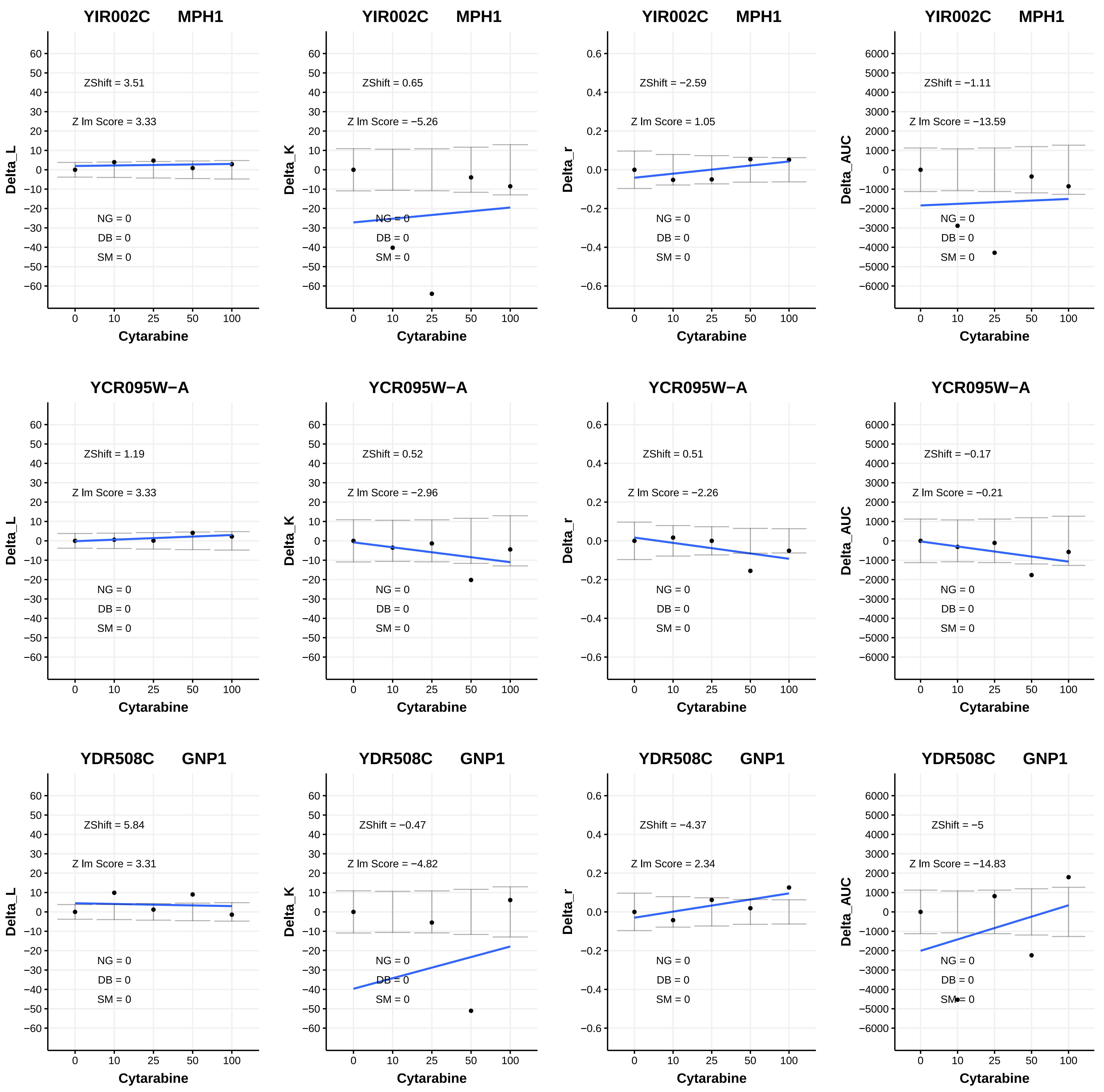
YIR002C MPH1
ZShift = 3.51
Z lm Score = 3.33
NG = 0
DB = 0
SM = 0
Delta_L
Cytarabine
YIR002C MPH1
ZShift = 0.65
Z lm Score = −5.26
NG = 0
DB = 0
SM = 0
Delta_K
Cytarabine
YIR002C MPH1
ZShift = −2.59
Z lm Score = 1.05
NG = 0
DB = 0
SM = 0
Delta_r
Cytarabine
YIR002C MPH1
ZShift = −1.11
Z lm Score = −13.59
NG = 0
DB = 0
SM = 0
Delta_AUC
Cytarabine
YCR095W−A
ZShift = 1.19
Z lm Score = 3.33
NG = 0
DB = 0
SM = 0
Delta_L
Cytarabine
YCR095W−A
ZShift = 0.52
Z lm Score = −2.96
NG = 0
DB = 0
SM = 0
Delta_K
Cytarabine
YCR095W−A
ZShift = 0.51
Z lm Score = −2.26
NG = 0
DB = 0
SM = 0
Delta_r
Cytarabine
YCR095W−A
ZShift = −0.17
Z lm Score = −0.21
NG = 0
DB = 0
SM = 0
Delta_AUC
Cytarabine
YDR508C GNP1
ZShift = 5.84
Z lm Score = 3.31
NG = 0
DB = 0
SM = 0
Delta_L
Cytarabine
YDR508C GNP1
ZShift = −0.47
Z lm Score = −4.82
NG = 0
DB = 0
SM = 0
Delta_K
Cytarabine
YDR508C GNP1
ZShift = −4.37
Z lm Score = 2.34
NG = 0
DB = 0
SM = 0
Delta_r
Cytarabine
YDR508C GNP1
ZShift = −5
Z lm Score = −14.83
NG = 0
DB = 0
SM = 0
Delta_AUC
Cytarabine

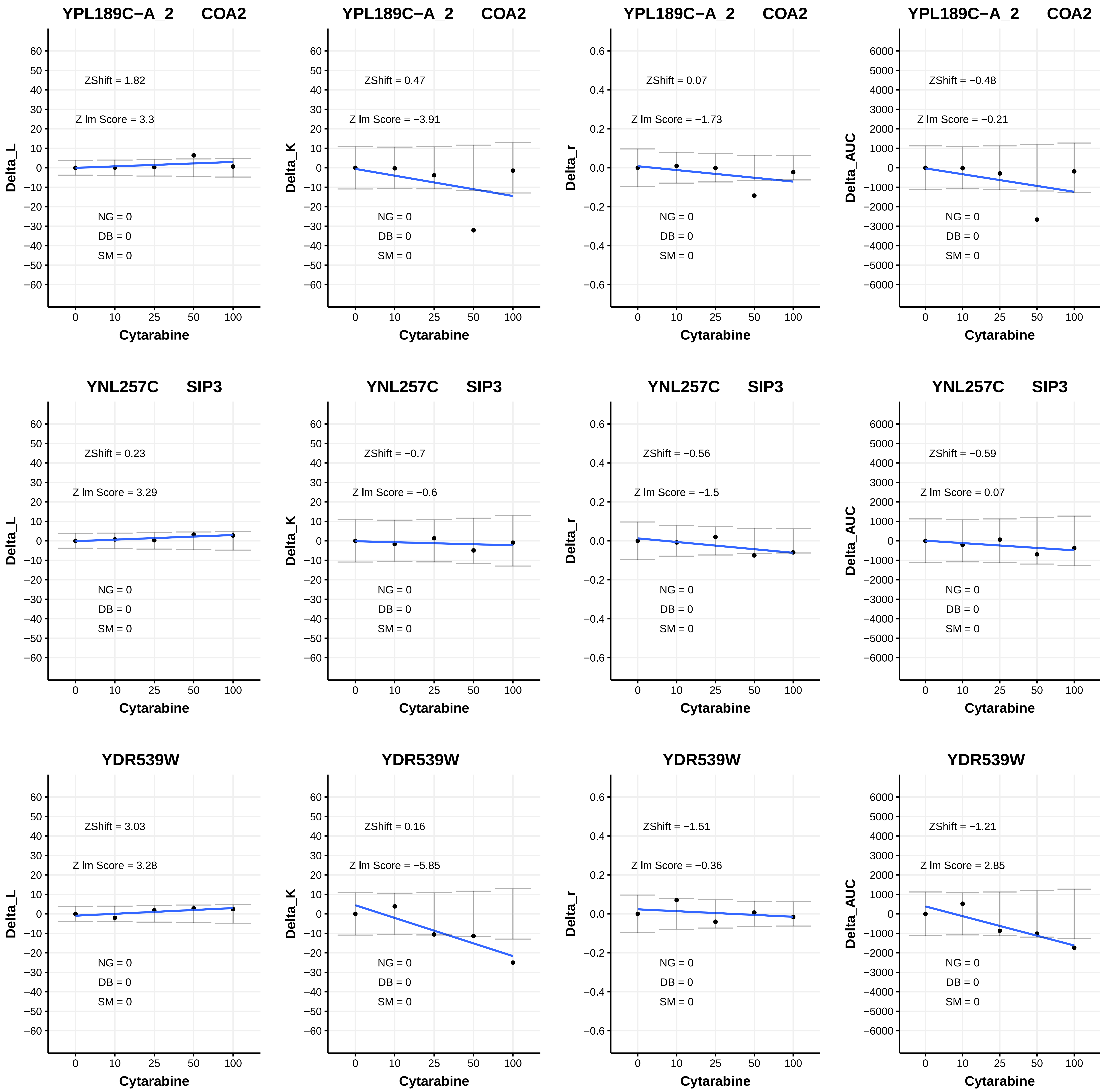
YPL189C−A_2 COA2
ZShift = 1.82
Z lm Score = 3.3
NG = 0
DB = 0
SM = 0
Delta_L
Cytarabine
YPL189C−A_2 COA2
ZShift = 0.47
Z lm Score = −3.91
NG = 0
DB = 0
SM = 0
Delta_K
Cytarabine
YPL189C−A_2 COA2
ZShift = 0.07
Z lm Score = −1.73
NG = 0
DB = 0
SM = 0
Delta_r
Cytarabine
YPL189C−A_2 COA2
ZShift = −0.48
Z lm Score = −0.21
NG = 0
DB = 0
SM = 0
Delta_AUC
Cytarabine
YNL257C SIP3
ZShift = 0.23
Z lm Score = 3.29
NG = 0
DB = 0
SM = 0
Delta_L
Cytarabine
YNL257C SIP3
ZShift = −0.7
Z lm Score = −0.6
NG = 0
DB = 0
SM = 0
Delta_K
Cytarabine
YNL257C SIP3
ZShift = −0.56
Z lm Score = −1.5
NG = 0
DB = 0
SM = 0
Delta_r
Cytarabine
YNL257C SIP3
ZShift = −0.59
Z lm Score = 0.07
NG = 0
DB = 0
SM = 0
Delta_AUC
Cytarabine
YDR539W
ZShift = 3.03
Z lm Score = 3.28
NG = 0
DB = 0
SM = 0
Delta_L
Cytarabine
YDR539W
ZShift = 0.16
Z lm Score = −5.85
NG = 0
DB = 0
SM = 0
Delta_K
Cytarabine
YDR539W
ZShift = −1.51
Z lm Score = −0.36
NG = 0
DB = 0
SM = 0
Delta_r
Cytarabine
YDR539W
ZShift = −1.21
Z lm Score = 2.85
NG = 0
DB = 0
SM = 0
Delta_AUC
Cytarabine

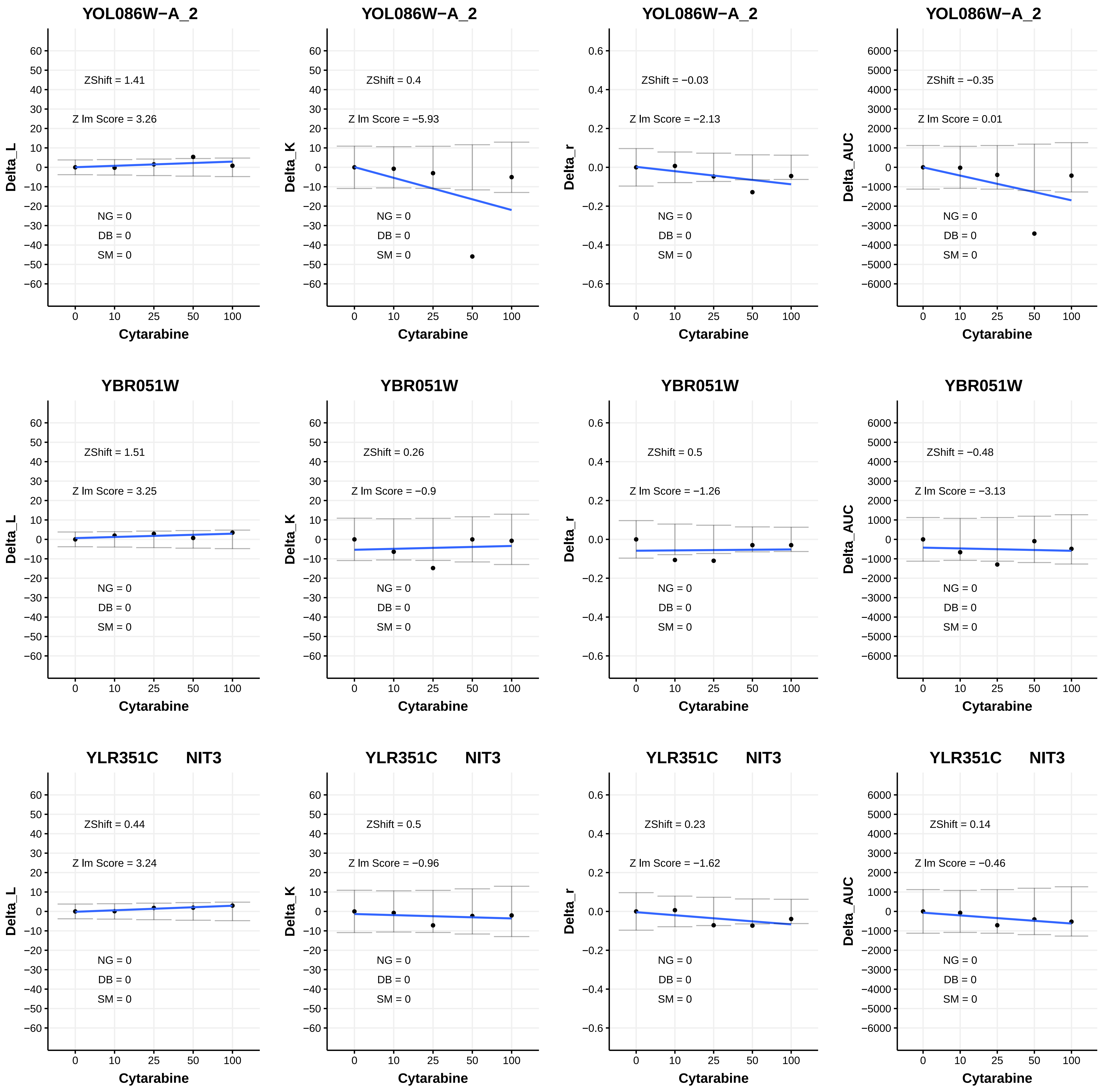
YOL086W−A_2
ZShift = 1.41
Z lm Score = 3.26
NG = 0
DB = 0
SM = 0
Delta_L
Cytarabine
YOL086W−A_2
ZShift = 0.4
Z lm Score = −5.93
NG = 0
DB = 0
SM = 0
Delta_K
Cytarabine
YOL086W−A_2
ZShift = −0.03
Z lm Score = −2.13
NG = 0
DB = 0
SM = 0
Delta_r
Cytarabine
YOL086W−A_2
ZShift = −0.35
Z lm Score = 0.01
NG = 0
DB = 0
SM = 0
Delta_AUC
Cytarabine
YBR051W
ZShift = 1.51
Z lm Score = 3.25
NG = 0
DB = 0
SM = 0
Delta_L
Cytarabine
YBR051W
ZShift = 0.26
Z lm Score = −0.9
NG = 0
DB = 0
SM = 0
Delta_K
Cytarabine
YBR051W
ZShift = 0.5
Z lm Score = −1.26
NG = 0
DB = 0
SM = 0
Delta_r
Cytarabine
YBR051W
ZShift = −0.48
Z lm Score = −3.13
NG = 0
DB = 0
SM = 0
Delta_AUC
Cytarabine
YLR351C NIT3
ZShift = 0.44
Z lm Score = 3.24
NG = 0
DB = 0
SM = 0
Delta_L
Cytarabine
YLR351C NIT3
ZShift = 0.5
Z lm Score = −0.96
NG = 0
DB = 0
SM = 0
Delta_K
Cytarabine
YLR351C NIT3
ZShift = 0.23
Z lm Score = −1.62
NG = 0
DB = 0
SM = 0
Delta_r
Cytarabine
YLR351C NIT3
ZShift = 0.14
Z lm Score = −0.46
NG = 0
DB = 0
SM = 0
Delta_AUC
Cytarabine

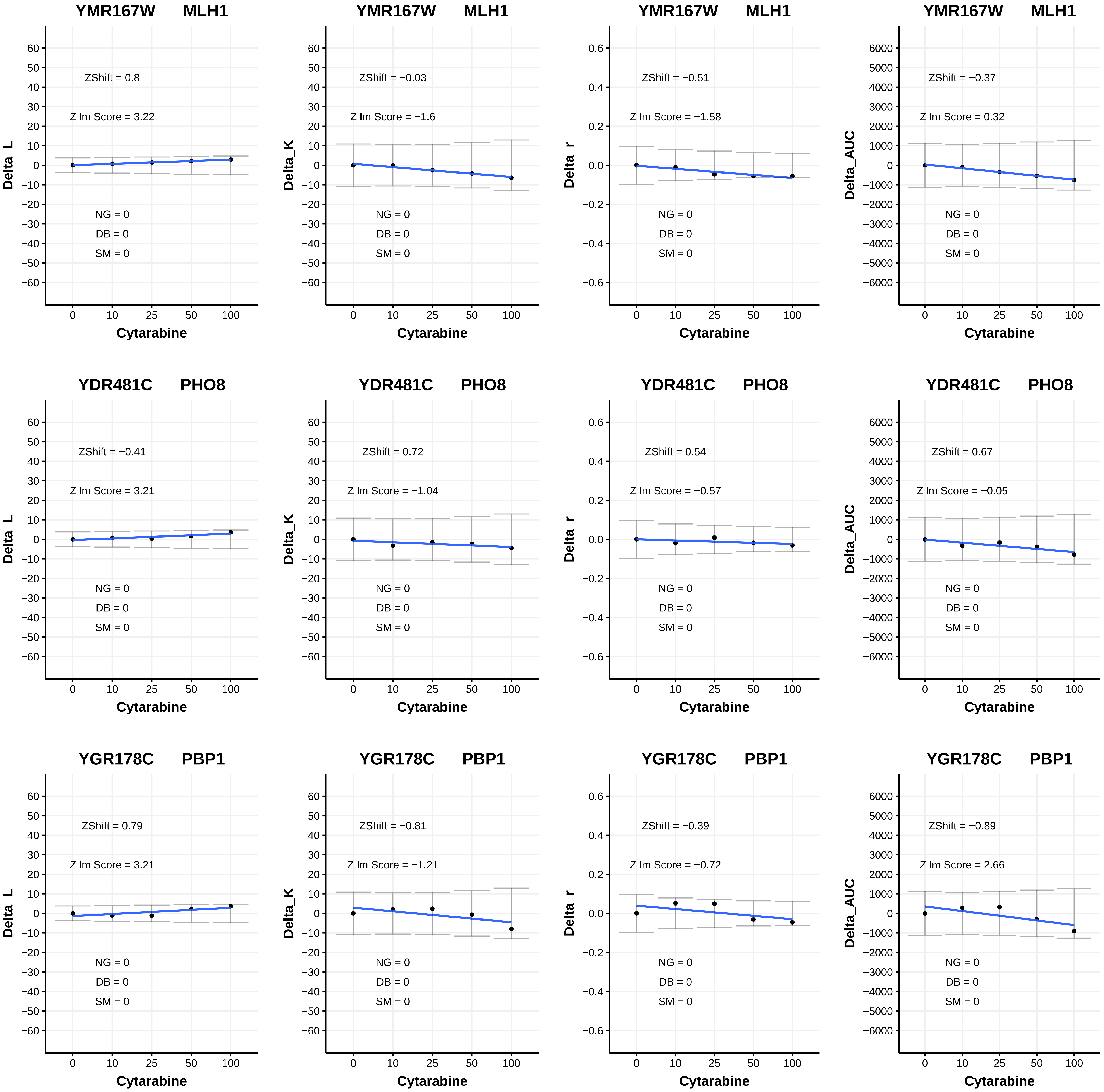

YMR167W MLH1
ZShift = 0.8
Z lm Score = 3.22
NG = 0
DB = 0
SM = 0
Delta_L
Cytarabine
YMR167W MLH1
ZShift = −0.03
Z lm Score = −1.6
NG = 0
DB = 0
SM = 0
Delta_K
Cytarabine
YMR167W MLH1
ZShift = −0.51
Z lm Score = −1.58
NG = 0
DB = 0
SM = 0
Delta_r
Cytarabine
YMR167W MLH1
ZShift = −0.37
Z lm Score = 0.32
NG = 0
DB = 0
SM = 0
Delta_AUC
Cytarabine
YDR481C PHO8
ZShift = −0.41
Z lm Score = 3.21
NG = 0
DB = 0
SM = 0
Delta_L
Cytarabine
YDR481C PHO8
ZShift = 0.72
Z lm Score = −1.04
NG = 0
DB = 0
SM = 0
Delta_K
Cytarabine
YDR481C PHO8
ZShift = 0.54
Z lm Score = −0.57
NG = 0
DB = 0
SM = 0
Delta_r
Cytarabine
YDR481C PHO8
ZShift = 0.67
Z lm Score = −0.05
NG = 0
DB = 0
SM = 0
Delta_AUC
Cytarabine
YGR178C PBP1
ZShift = 0.79
Z lm Score = 3.21
NG = 0
DB = 0
SM = 0
Delta_L
Cytarabine
YGR178C PBP1
ZShift = −0.81
Z lm Score = −1.21
NG = 0
DB = 0
SM = 0
Delta_K
Cytarabine
YGR178C PBP1
ZShift = −0.39
Z lm Score = −0.72
NG = 0
DB = 0
SM = 0
Delta_r
Cytarabine
YGR178C PBP1
ZShift = −0.89
Z lm Score = 2.66
NG = 0
DB = 0
SM = 0
Delta_AUC
Cytarabine

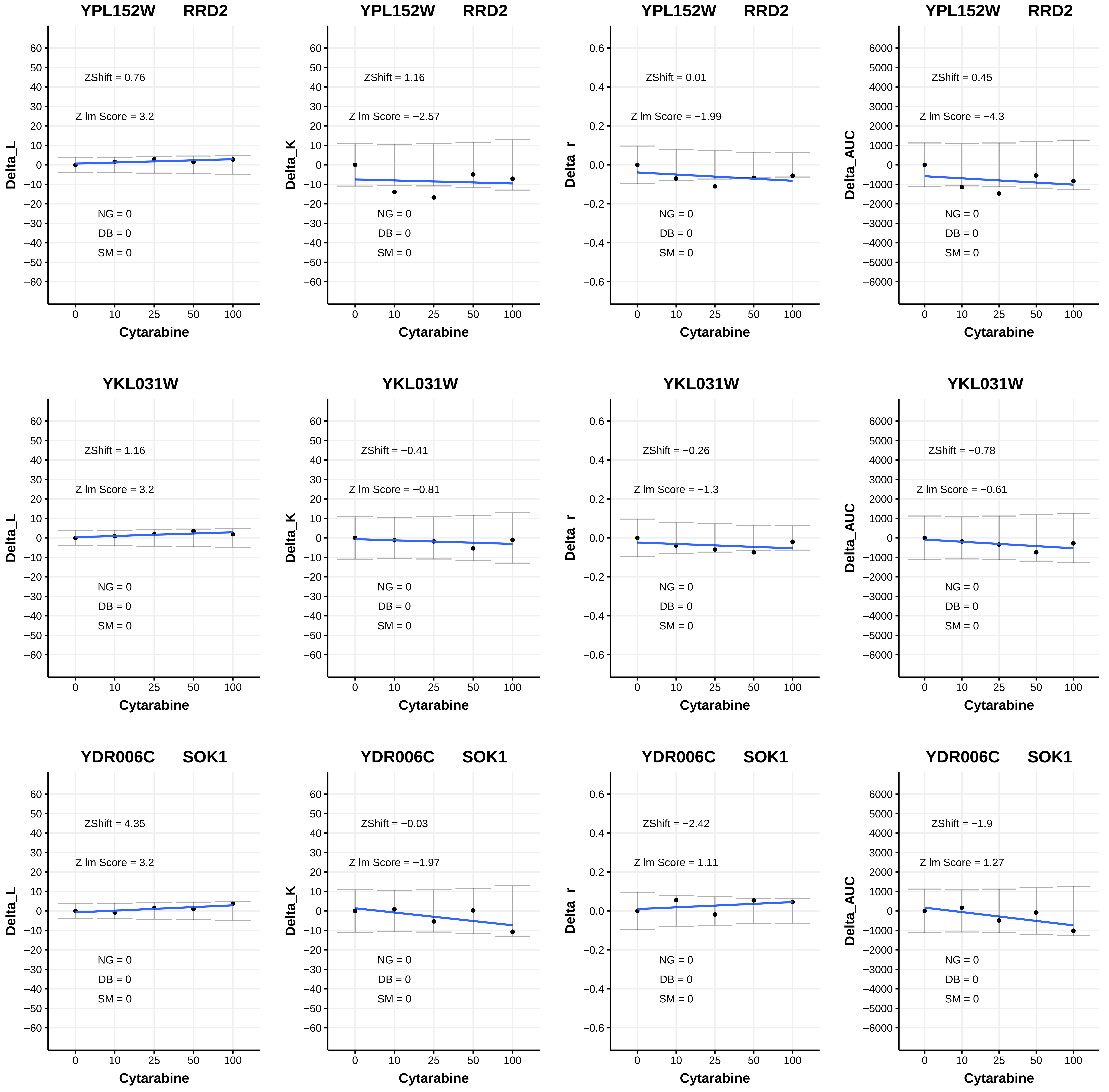

YPL152W RRD2
ZShift = 0.76
Z lm Score = 3.2
NG = 0
DB = 0
SM = 0
Delta_L
Cytarabine
YPL152W RRD2
ZShift = 1.16
Z lm Score = −2.57
NG = 0
DB = 0
SM = 0
Delta_K
Cytarabine
YPL152W RRD2
ZShift = 0.01
Z lm Score = −1.99
NG = 0
DB = 0
SM = 0
Delta_r
Cytarabine
YPL152W RRD2
ZShift = 0.45
Z lm Score = −4.3
NG = 0
DB = 0
SM = 0
Delta_AUC
Cytarabine
YKL031W
ZShift = 1.16
Z lm Score = 3.2
NG = 0
DB = 0
SM = 0
Delta_L
Cytarabine
YKL031W
ZShift = −0.41
Z lm Score = −0.81
NG = 0
DB = 0
SM = 0
Delta_K
Cytarabine
YKL031W
ZShift = −0.26
Z lm Score = −1.3
NG = 0
DB = 0
SM = 0
Delta_r
Cytarabine
YKL031W
ZShift = −0.78
Z lm Score = −0.61
NG = 0
DB = 0
SM = 0
Delta_AUC
Cytarabine
YDR006C SOK1
ZShift = 4.35
Z lm Score = 3.2
NG = 0
DB = 0
SM = 0
Delta_L
Cytarabine
YDR006C SOK1
ZShift = −0.03
Z lm Score = −1.97
NG = 0
DB = 0
SM = 0
Delta_K
Cytarabine
YDR006C SOK1
ZShift = −2.42
Z lm Score = 1.11
NG = 0
DB = 0
SM = 0
Delta_r
Cytarabine
YDR006C SOK1
ZShift = −1.9
Z lm Score = 1.27
NG = 0
DB = 0
SM = 0
Delta_AUC
Cytarabine

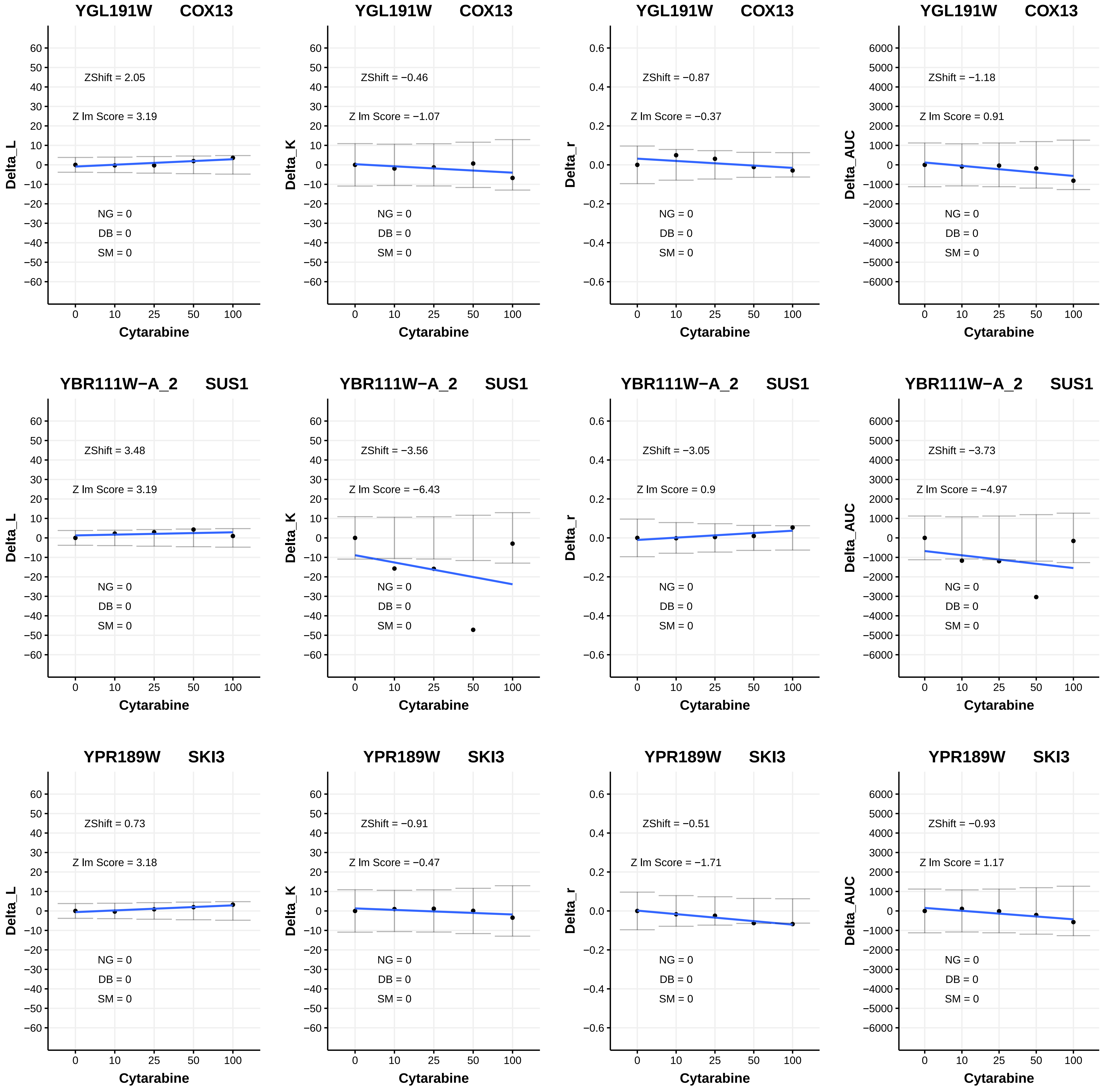

YGL191W COX13
ZShift = 2.05
Z lm Score = 3.19
NG = 0
DB = 0
SM = 0
Delta_L
Cytarabine
YGL191W COX13
ZShift = −0.46
Z lm Score = −1.07
NG = 0
DB = 0
SM = 0
Delta_K
Cytarabine
YGL191W COX13
ZShift = −0.87
Z lm Score = −0.37
NG = 0
DB = 0
SM = 0
Delta_r
Cytarabine
YGL191W COX13
ZShift = −1.18
Z lm Score = 0.91
NG = 0
DB = 0
SM = 0
Delta_AUC
Cytarabine
YBR111W−A_2 SUS1
ZShift = 3.48
Z lm Score = 3.19
NG = 0
DB = 0
SM = 0
Delta_L
Cytarabine
YBR111W−A_2 SUS1
ZShift = −3.56
Z lm Score = −6.43
NG = 0
DB = 0
SM = 0
Delta_K
Cytarabine
YBR111W−A_2 SUS1
ZShift = −3.05
Z lm Score = 0.9
NG = 0
DB = 0
SM = 0
Delta_r
Cytarabine
YBR111W−A_2 SUS1
ZShift = −3.73
Z lm Score = −4.97
NG = 0
DB = 0
SM = 0
Delta_AUC
Cytarabine
YPR189W SKI3
ZShift = 0.73
Z lm Score = 3.18
NG = 0
DB = 0
SM = 0
Delta_L
Cytarabine
YPR189W SKI3
ZShift = −0.91
Z lm Score = −0.47
NG = 0
DB = 0
SM = 0
Delta_K
Cytarabine
YPR189W SKI3
ZShift = −0.51
Z lm Score = −1.71
NG = 0
DB = 0
SM = 0
Delta_r
Cytarabine
YPR189W SKI3
ZShift = −0.93
Z lm Score = 1.17
NG = 0
DB = 0
SM = 0
Delta_AUC
Cytarabine

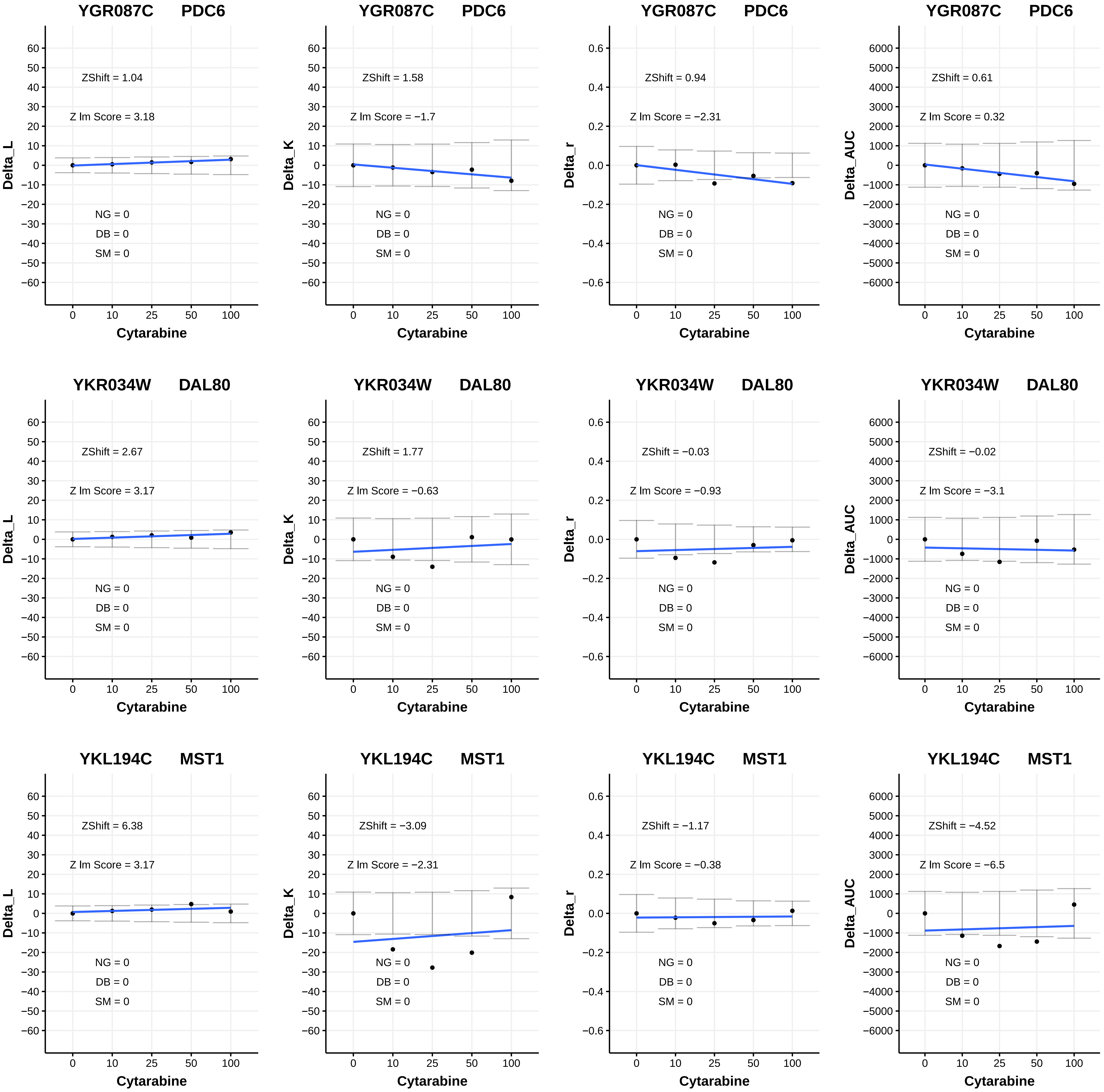

YGR087C PDC6
ZShift = 1.04
Z lm Score = 3.18
NG = 0
DB = 0
SM = 0
Delta_L
Cytarabine
YGR087C PDC6
ZShift = 1.58
Z lm Score = −1.7
NG = 0
DB = 0
SM = 0
Delta_K
Cytarabine
YGR087C PDC6
ZShift = 0.94
Z lm Score = −2.31
NG = 0
DB = 0
SM = 0
Delta_r
Cytarabine
YGR087C PDC6
ZShift = 0.61
Z lm Score = 0.32
NG = 0
DB = 0
SM = 0
Delta_AUC
Cytarabine
YKR034W DAL80
ZShift = 2.67
Z lm Score = 3.17
NG = 0
DB = 0
SM = 0
Delta_L
Cytarabine
YKR034W DAL80
ZShift = 1.77
Z lm Score = −0.63
NG = 0
DB = 0
SM = 0
Delta_K
Cytarabine
YKR034W DAL80
ZShift = −0.03
Z lm Score = −0.93
NG = 0
DB = 0
SM = 0
Delta_r
Cytarabine
YKR034W DAL80
ZShift = −0.02
Z lm Score = −3.1
NG = 0
DB = 0
SM = 0
Delta_AUC
Cytarabine
YKL194C MST1
ZShift = 6.38
Z lm Score = 3.17
NG = 0
DB = 0
SM = 0
Delta_L
Cytarabine
YKL194C MST1
ZShift = −3.09
Z lm Score = −2.31
NG = 0
DB = 0
SM = 0
Delta_K
Cytarabine
YKL194C MST1
ZShift = −1.17
Z lm Score = −0.38
NG = 0
DB = 0
SM = 0
Delta_r
Cytarabine
YKL194C MST1
ZShift = −4.52
Z lm Score = −6.5
NG = 0
DB = 0
SM = 0
Delta_AUC
Cytarabine

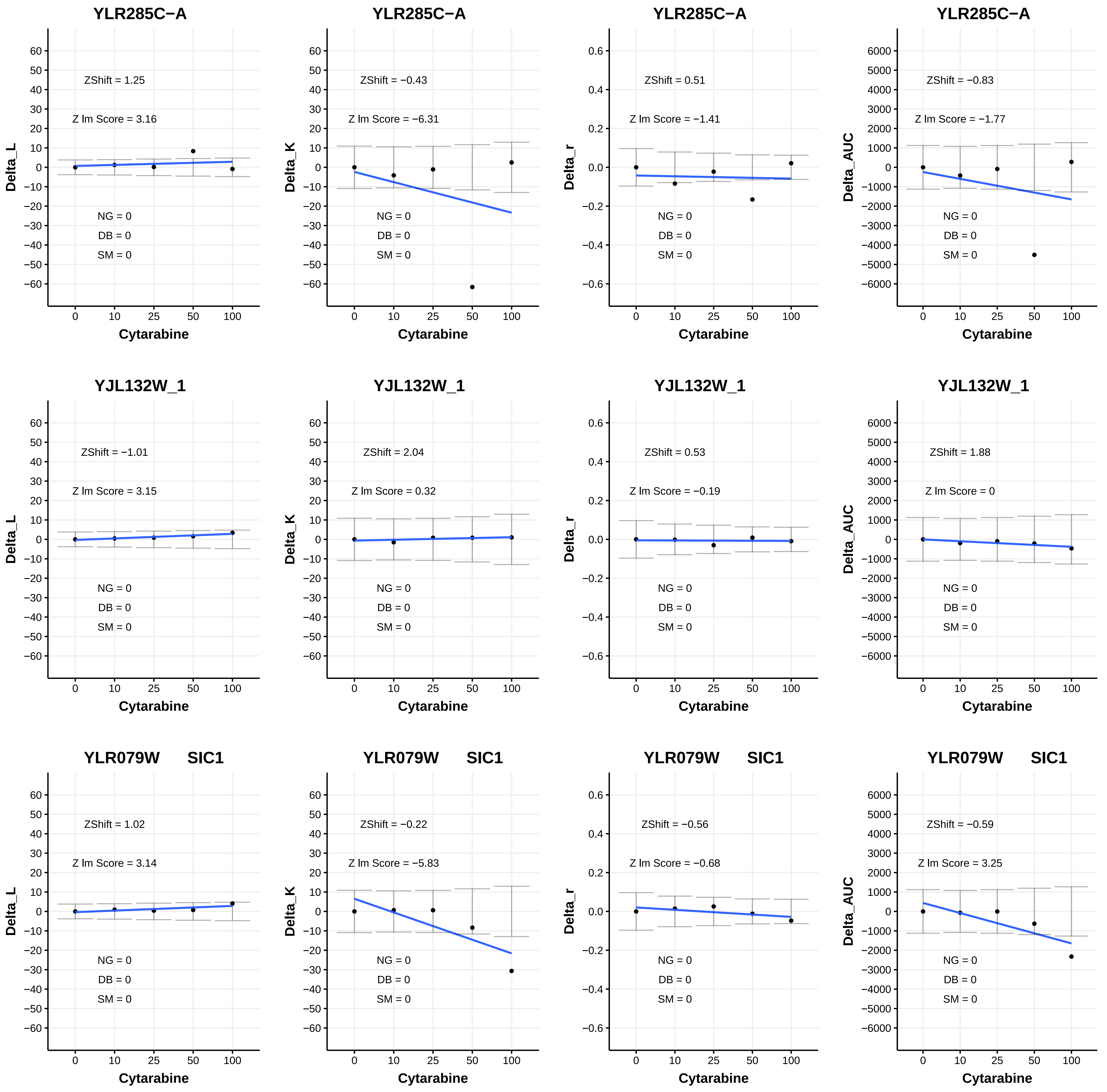
YLR285C−A
ZShift = 1.25
Z lm Score = 3.16
NG = 0
DB = 0
SM = 0
Delta_L
Cytarabine
YLR285C−A
ZShift = −0.43
Z lm Score = −6.31
NG = 0
DB = 0
SM = 0
Delta_K
Cytarabine
YLR285C−A
ZShift = 0.51
Z lm Score = −1.41
NG = 0
DB = 0
SM = 0
Delta_r
Cytarabine
YLR285C−A
ZShift = −0.83
Z lm Score = −1.77
NG = 0
DB = 0
SM = 0
Delta_AUC
Cytarabine
YJL132W_1
ZShift = −1.01
Z lm Score = 3.15
NG = 0
DB = 0
SM = 0
Delta_L
Cytarabine
YJL132W_1
ZShift = 2.04
Z lm Score = 0.32
NG = 0
DB = 0
SM = 0
Delta_K
Cytarabine
YJL132W_1
ZShift = 0.53
Z lm Score = −0.19
NG = 0
DB = 0
SM = 0
Delta_r
Cytarabine
YJL132W_1
ZShift = 1.88
Z lm Score = 0
NG = 0
DB = 0
SM = 0
Delta_AUC
Cytarabine
YLR079W SIC1
ZShift = 1.02
Z lm Score = 3.14
NG = 0
DB = 0
SM = 0
Delta_L
Cytarabine
YLR079W SIC1
ZShift = −0.22
Z lm Score = −5.83
NG = 0
DB = 0
SM = 0
Delta_K
Cytarabine
YLR079W SIC1
ZShift = −0.56
Z lm Score = −0.68
NG = 0
DB = 0
SM = 0
Delta_r
Cytarabine
YLR079W SIC1
ZShift = −0.59
Z lm Score = 3.25
NG = 0
DB = 0
SM = 0
Delta_AUC
Cytarabine

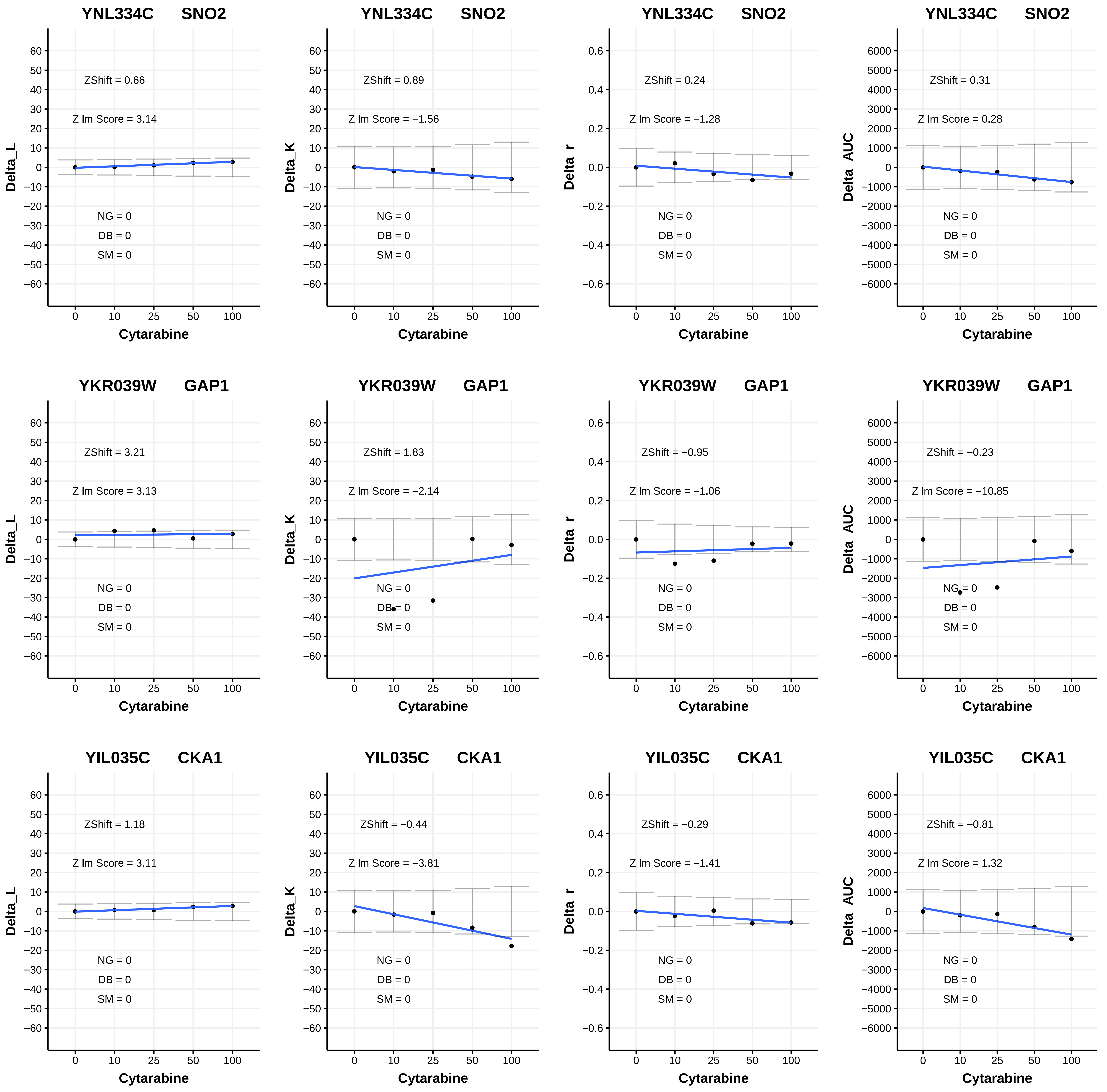
YNL334C SNO2
ZShift = 0.66
Z lm Score = 3.14
NG = 0
DB = 0
SM = 0
Delta_L
Cytarabine
YNL334C SNO2
ZShift = 0.89
Z lm Score = −1.56
NG = 0
DB = 0
SM = 0
Delta_K
Cytarabine
YNL334C SNO2
ZShift = 0.24
Z lm Score = −1.28
NG = 0
DB = 0
SM = 0
Delta_r
Cytarabine
YNL334C SNO2
ZShift = 0.31
Z lm Score = 0.28
NG = 0
DB = 0
SM = 0
Delta_AUC
Cytarabine
YKR039W GAP1
ZShift = 3.21
Z lm Score = 3.13
NG = 0
DB = 0
SM = 0
Delta_L
Cytarabine
YKR039W GAP1
ZShift = 1.83
Z lm Score = −2.14
NG = 0
DB = 0
SM = 0
Delta_K
Cytarabine
YKR039W GAP1
ZShift = −0.95
Z lm Score = −1.06
NG = 0
DB = 0
SM = 0
Delta_r
Cytarabine
YKR039W GAP1
ZShift = −0.23
Z lm Score = −10.85
NG = 0
DB = 0
SM = 0
Delta_AUC
Cytarabine
YIL035C CKA1
ZShift = 1.18
Z lm Score = 3.11
NG = 0
DB = 0
SM = 0
Delta_L
Cytarabine
YIL035C CKA1
ZShift = −0.44
Z lm Score = −3.81
NG = 0
DB = 0
SM = 0
Delta_K
Cytarabine
YIL035C CKA1
ZShift = −0.29
Z lm Score = −1.41
NG = 0
DB = 0
SM = 0
Delta_r
Cytarabine
YIL035C CKA1
ZShift = −0.81
Z lm Score = 1.32
NG = 0
DB = 0
SM = 0
Delta_AUC
Cytarabine

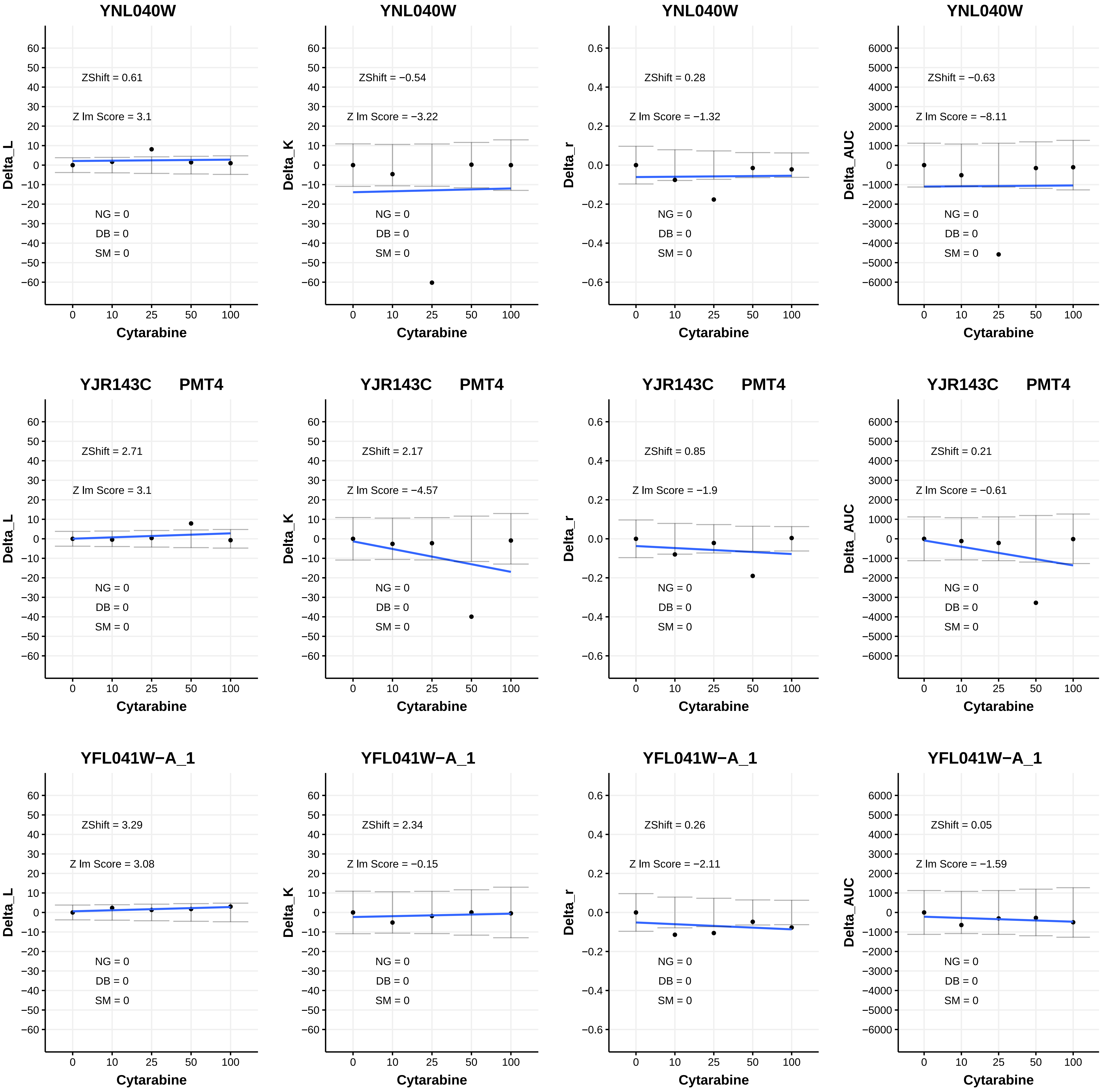
YNL040W
ZShift = 0.61
Z lm Score = 3.1
NG = 0
DB = 0
SM = 0
Delta_L
Cytarabine
YNL040W
ZShift = −0.54
Z lm Score = −3.22
NG = 0
DB = 0
SM = 0
Delta_K
Cytarabine
YNL040W
ZShift = 0.28
Z lm Score = −1.32
NG = 0
DB = 0
SM = 0
Delta_r
Cytarabine
YNL040W
ZShift = −0.63
Z lm Score = −8.11
NG = 0
DB = 0
SM = 0
Delta_AUC
Cytarabine
YJR143C PMT4
ZShift = 2.71
Z lm Score = 3.1
NG = 0
DB = 0
SM = 0
Delta_L
Cytarabine
YJR143C PMT4
ZShift = 2.17
Z lm Score = −4.57
NG = 0
DB = 0
SM = 0
Delta_K
Cytarabine
YJR143C PMT4
ZShift = 0.85
Z lm Score = −1.9
NG = 0
DB = 0
SM = 0
Delta_r
Cytarabine
YJR143C PMT4
ZShift = 0.21
Z lm Score = −0.61
NG = 0
DB = 0
SM = 0
Delta_AUC
Cytarabine
YFL041W−A_1
ZShift = 3.29
Z lm Score = 3.08
NG = 0
DB = 0
SM = 0
Delta_L
Cytarabine
YFL041W−A_1
ZShift = 2.34
Z lm Score = −0.15
NG = 0
DB = 0
SM = 0
Delta_K
Cytarabine
YFL041W−A_1
ZShift = 0.26
Z lm Score = −2.11
NG = 0
DB = 0
SM = 0
Delta_r
Cytarabine
YFL041W−A_1
ZShift = 0.05
Z lm Score = −1.59
NG = 0
DB = 0
SM = 0
Delta_AUC
Cytarabine

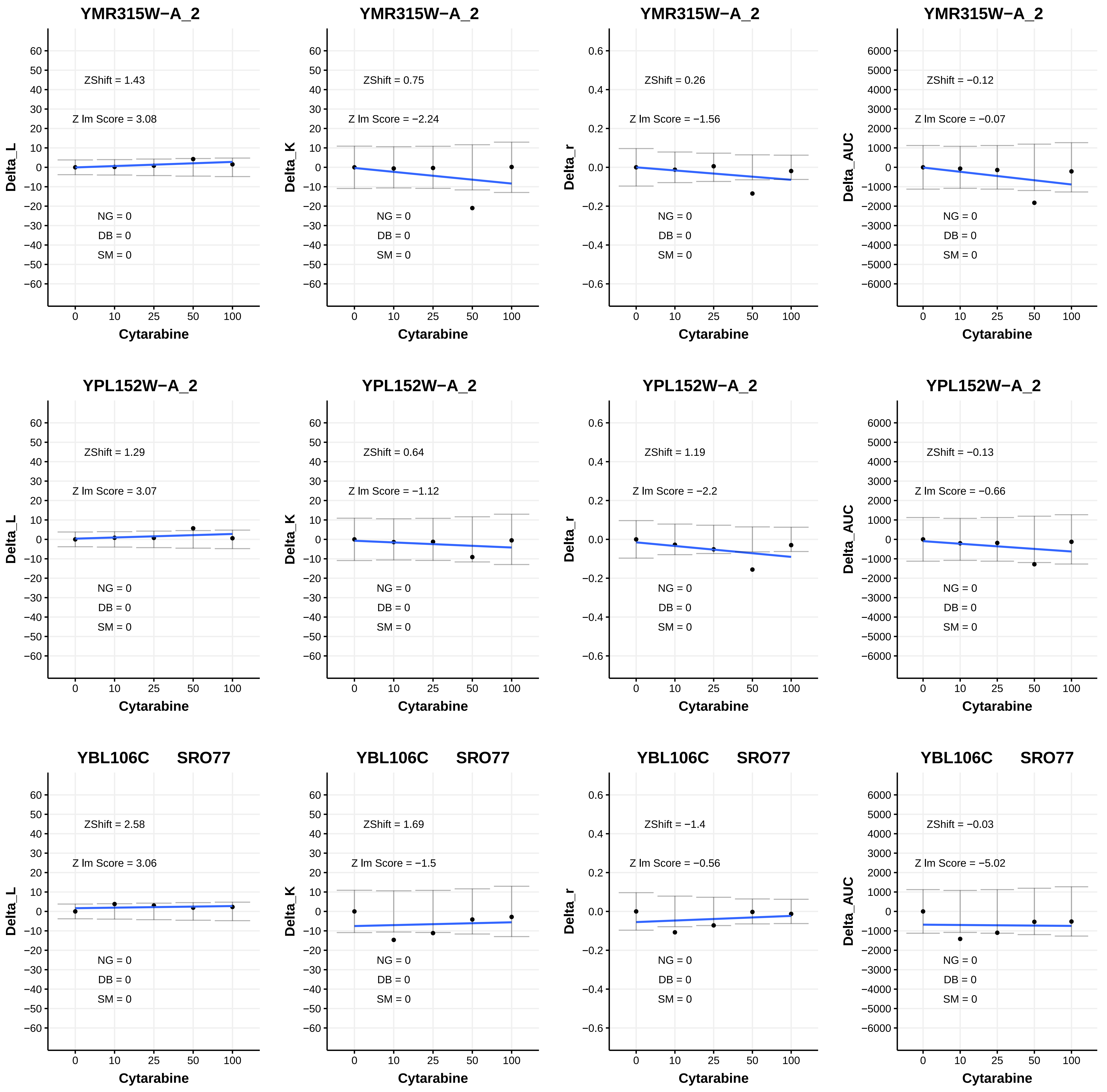
YMR315W−A_2
ZShift = 1.43
Z lm Score = 3.08
NG = 0
DB = 0
SM = 0
Delta_L
Cytarabine
YMR315W−A_2
ZShift = 0.75
Z lm Score = −2.24
NG = 0
DB = 0
SM = 0
Delta_K
Cytarabine
YMR315W−A_2
ZShift = 0.26
Z lm Score = −1.56
NG = 0
DB = 0
SM = 0
Delta_r
Cytarabine
YMR315W−A_2
ZShift = −0.12
Z lm Score = −0.07
NG = 0
DB = 0
SM = 0
Delta_AUC
Cytarabine
YPL152W−A_2
ZShift = 1.29
Z lm Score = 3.07
NG = 0
DB = 0
SM = 0
Delta_L
Cytarabine
YPL152W−A_2
ZShift = 0.64
Z lm Score = −1.12
NG = 0
DB = 0
SM = 0
Delta_K
Cytarabine
YPL152W−A_2
ZShift = 1.19
Z lm Score = −2.2
NG = 0
DB = 0
SM = 0
Delta_r
Cytarabine
YPL152W−A_2
ZShift = −0.13
Z lm Score = −0.66
NG = 0
DB = 0
SM = 0
Delta_AUC
Cytarabine
YBL106C SRO77
ZShift = 2.58
Z lm Score = 3.06
NG = 0
DB = 0
SM = 0
Delta_L
Cytarabine
YBL106C SRO77
ZShift = 1.69
Z lm Score = −1.5
NG = 0
DB = 0
SM = 0
Delta_K
Cytarabine
YBL106C SRO77
ZShift = −1.4
Z lm Score = −0.56
NG = 0
DB = 0
SM = 0
Delta_r
Cytarabine
YBL106C SRO77
ZShift = −0.03
Z lm Score = −5.02
NG = 0
DB = 0
SM = 0
Delta_AUC
Cytarabine

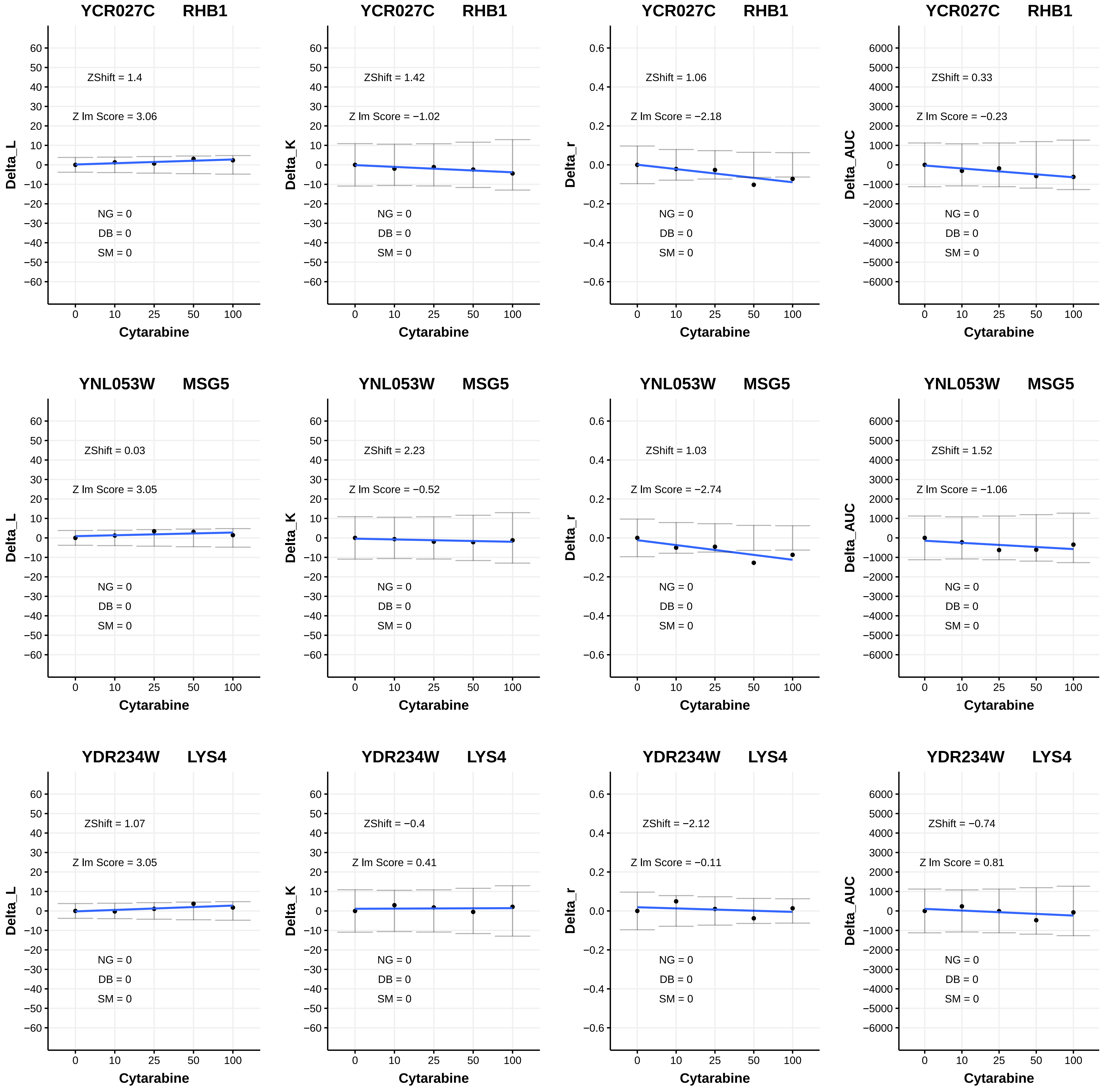
YCR027C RHB1
ZShift = 1.4
Z lm Score = 3.06
NG = 0
DB = 0
SM = 0
Delta_L
Cytarabine
YCR027C RHB1
ZShift = 1.42
Z lm Score = −1.02
NG = 0
DB = 0
SM = 0
Delta_K
Cytarabine
YCR027C RHB1
ZShift = 1.06
Z lm Score = −2.18
NG = 0
DB = 0
SM = 0
Delta_r
Cytarabine
YCR027C RHB1
ZShift = 0.33
Z lm Score = −0.23
NG = 0
DB = 0
SM = 0
Delta_AUC
Cytarabine
YNL053W MSG5
ZShift = 0.03
Z lm Score = 3.05
NG = 0
DB = 0
SM = 0
Delta_L
Cytarabine
YNL053W MSG5
ZShift = 2.23
Z lm Score = −0.52
NG = 0
DB = 0
SM = 0
Delta_K
Cytarabine
YNL053W MSG5
ZShift = 1.03
Z lm Score = −2.74
NG = 0
DB = 0
SM = 0
Delta_r
Cytarabine
YNL053W MSG5
ZShift = 1.52
Z lm Score = −1.06
NG = 0
DB = 0
SM = 0
Delta_AUC
Cytarabine
YDR234W LYS4
ZShift = 1.07
Z lm Score = 3.05
NG = 0
DB = 0
SM = 0
Delta_L
Cytarabine
YDR234W LYS4
ZShift = −0.4
Z lm Score = 0.41
NG = 0
DB = 0
SM = 0
Delta_K
Cytarabine
YDR234W LYS4
ZShift = −2.12
Z lm Score = −0.11
NG = 0
DB = 0
SM = 0
Delta_r
Cytarabine
YDR234W LYS4
ZShift = −0.74
Z lm Score = 0.81
NG = 0
DB = 0
SM = 0
Delta_AUC
Cytarabine

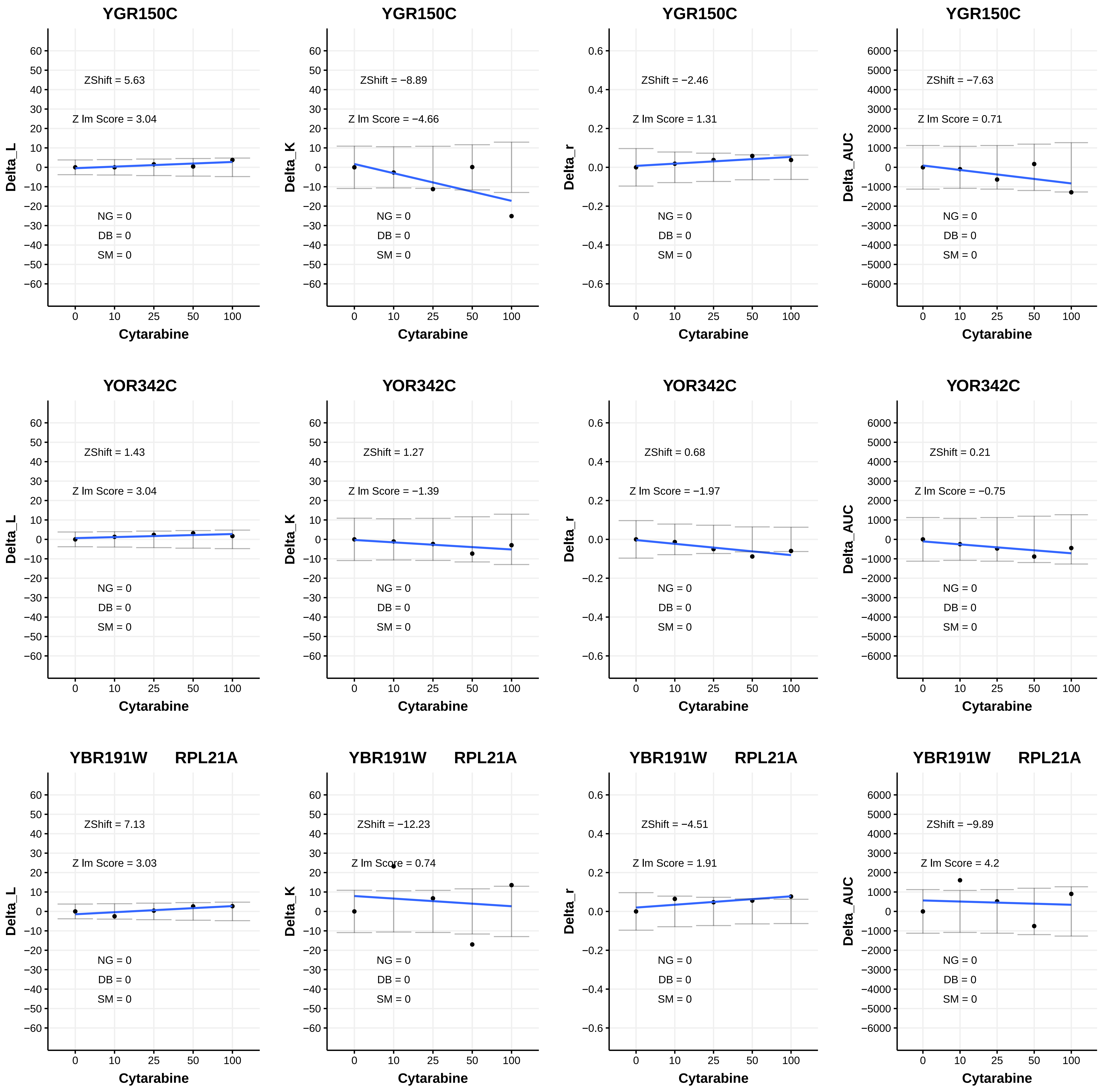
YGR150C
ZShift = 5.63
Z lm Score = 3.04
NG = 0
DB = 0
SM = 0
Delta_L
Cytarabine
YGR150C
ZShift = −8.89
Z lm Score = −4.66
NG = 0
DB = 0
SM = 0
Delta_K
Cytarabine
YGR150C
ZShift = −2.46
Z lm Score = 1.31
NG = 0
DB = 0
SM = 0
Delta_r
Cytarabine
YGR150C
ZShift = −7.63
Z lm Score = 0.71
NG = 0
DB = 0
SM = 0
Delta_AUC
Cytarabine
YOR342C
ZShift = 1.43
Z lm Score = 3.04
NG = 0
DB = 0
SM = 0
Delta_L
Cytarabine
YOR342C
ZShift = 1.27
Z lm Score = −1.39
NG = 0
DB = 0
SM = 0
Delta_K
Cytarabine
YOR342C
ZShift = 0.68
Z lm Score = −1.97
NG = 0
DB = 0
SM = 0
Delta_r
Cytarabine
YOR342C
ZShift = 0.21
Z lm Score = −0.75
NG = 0
DB = 0
SM = 0
Delta_AUC
Cytarabine
YBR191W RPL21A
ZShift = 7.13
Z lm Score = 3.03
NG = 0
DB = 0
SM = 0
Delta_L
Cytarabine
YBR191W RPL21A
ZShift = −12.23
Z lm Score = 0.74
NG = 0
DB = 0
SM = 0
Delta_K
Cytarabine
YBR191W RPL21A
ZShift = −4.51
Z lm Score = 1.91
NG = 0
DB = 0
SM = 0
Delta_r
Cytarabine
YBR191W RPL21A
ZShift = −9.89
Z lm Score = 4.2
NG = 0
DB = 0
SM = 0
Delta_AUC
Cytarabine

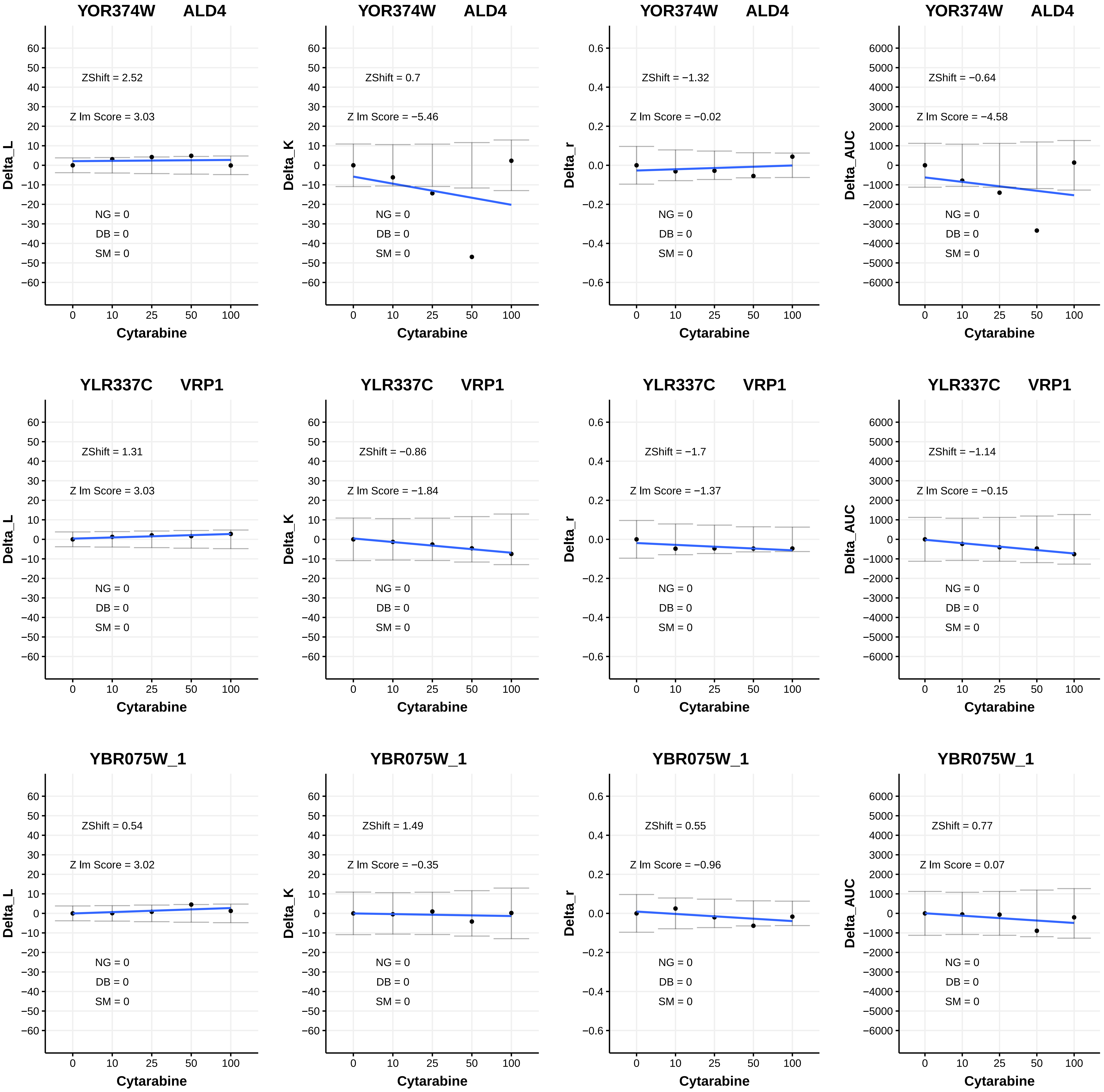

YOR374W ALD4
ZShift = 2.52
Z lm Score = 3.03
NG = 0
DB = 0
SM = 0
Delta_L
Cytarabine
YOR374W ALD4
ZShift = 0.7
Z lm Score = −5.46
NG = 0
DB = 0
SM = 0
Delta_K
Cytarabine
YOR374W ALD4
ZShift = −1.32
Z lm Score = −0.02
NG = 0
DB = 0
SM = 0
Delta_r
Cytarabine
YOR374W ALD4
ZShift = −0.64
Z lm Score = −4.58
NG = 0
DB = 0
SM = 0
Delta_AUC
Cytarabine
YLR337C VRP1
ZShift = 1.31
Z lm Score = 3.03
NG = 0
DB = 0
SM = 0
Delta_L
Cytarabine
YLR337C VRP1
ZShift = −0.86
Z lm Score = −1.84
NG = 0
DB = 0
SM = 0
Delta_K
Cytarabine
YLR337C VRP1
ZShift = −1.7
Z lm Score = −1.37
NG = 0
DB = 0
SM = 0
Delta_r
Cytarabine
YLR337C VRP1
ZShift = −1.14
Z lm Score = −0.15
NG = 0
DB = 0
SM = 0
Delta_AUC
Cytarabine
YBR075W_1
ZShift = 0.54
Z lm Score = 3.02
NG = 0
DB = 0
SM = 0
Delta_L
Cytarabine
YBR075W_1
ZShift = 1.49
Z lm Score = −0.35
NG = 0
DB = 0
SM = 0
Delta_K
Cytarabine
YBR075W_1
ZShift = 0.55
Z lm Score = −0.96
NG = 0
DB = 0
SM = 0
Delta_r
Cytarabine
YBR075W_1
ZShift = 0.77
Z lm Score = 0.07
NG = 0
DB = 0
SM = 0
Delta_AUC
Cytarabine

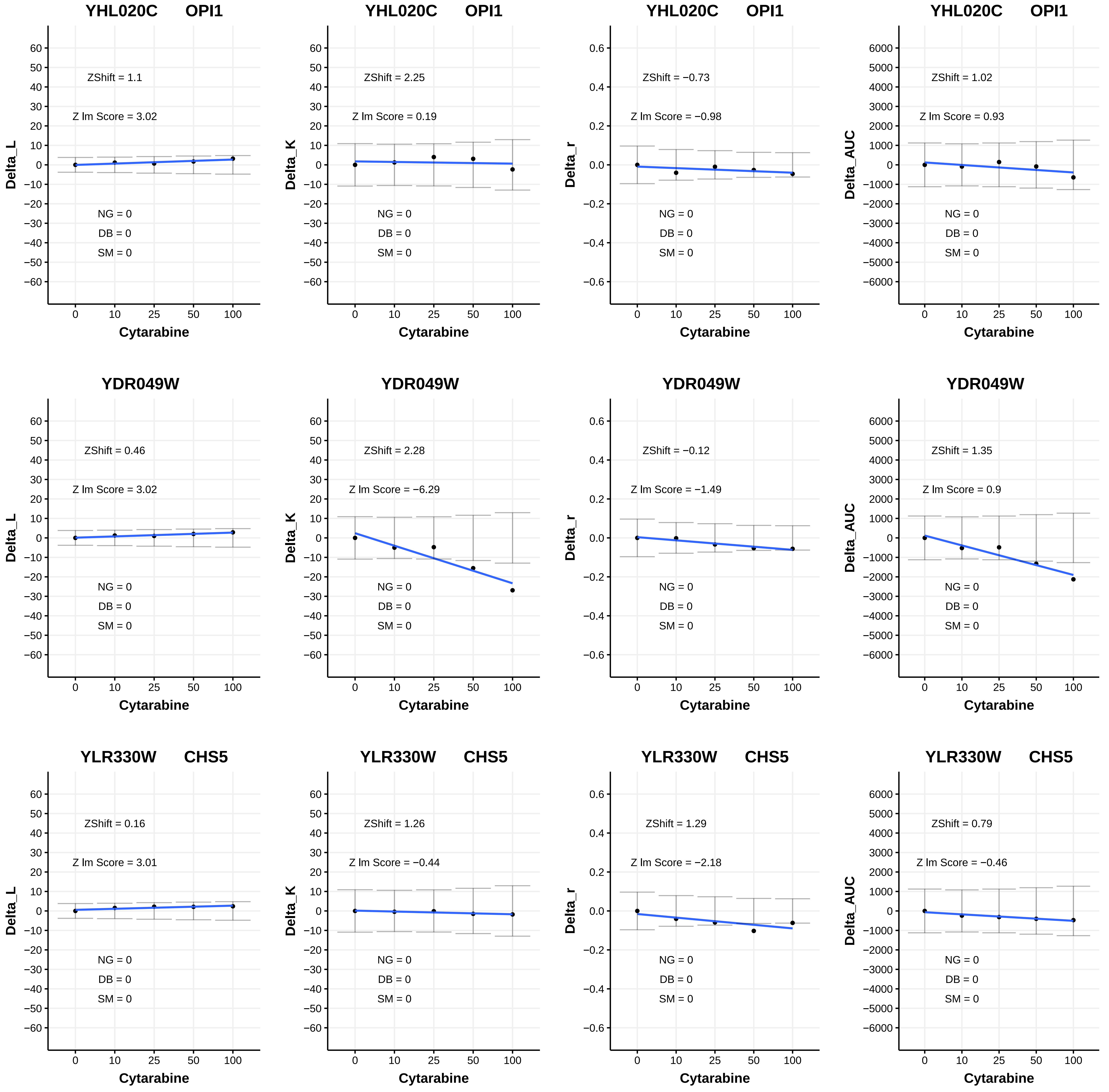
YHL020C OPI1
ZShift = 1.1
Z lm Score = 3.02
NG = 0
DB = 0
SM = 0
Delta_L
Cytarabine
YHL020C OPI1
ZShift = 2.25
Z lm Score = 0.19
NG = 0
DB = 0
SM = 0
Delta_K
Cytarabine
YHL020C OPI1
ZShift = −0.73
Z lm Score = −0.98
NG = 0
DB = 0
SM = 0
Delta_r
Cytarabine
YHL020C OPI1
ZShift = 1.02
Z lm Score = 0.93
NG = 0
DB = 0
SM = 0
Delta_AUC
Cytarabine
YDR049W
ZShift = 0.46
Z lm Score = 3.02
NG = 0
DB = 0
SM = 0
Delta_L
Cytarabine
YDR049W
ZShift = 2.28
Z lm Score = −6.29
NG = 0
DB = 0
SM = 0
Delta_K
Cytarabine
YDR049W
ZShift = −0.12
Z lm Score = −1.49
NG = 0
DB = 0
SM = 0
Delta_r
Cytarabine
YDR049W
ZShift = 1.35
Z lm Score = 0.9
NG = 0
DB = 0
SM = 0
Delta_AUC
Cytarabine
YLR330W CHS5
ZShift = 0.16
Z lm Score = 3.01
NG = 0
DB = 0
SM = 0
Delta_L
Cytarabine
YLR330W CHS5
ZShift = 1.26
Z lm Score = −0.44
NG = 0
DB = 0
SM = 0
Delta_K
Cytarabine
YLR330W CHS5
ZShift = 1.29
Z lm Score = −2.18
NG = 0
DB = 0
SM = 0
Delta_r
Cytarabine
YLR330W CHS5
ZShift = 0.79
Z lm Score = −0.46
NG = 0
DB = 0
SM = 0
Delta_AUC
Cytarabine

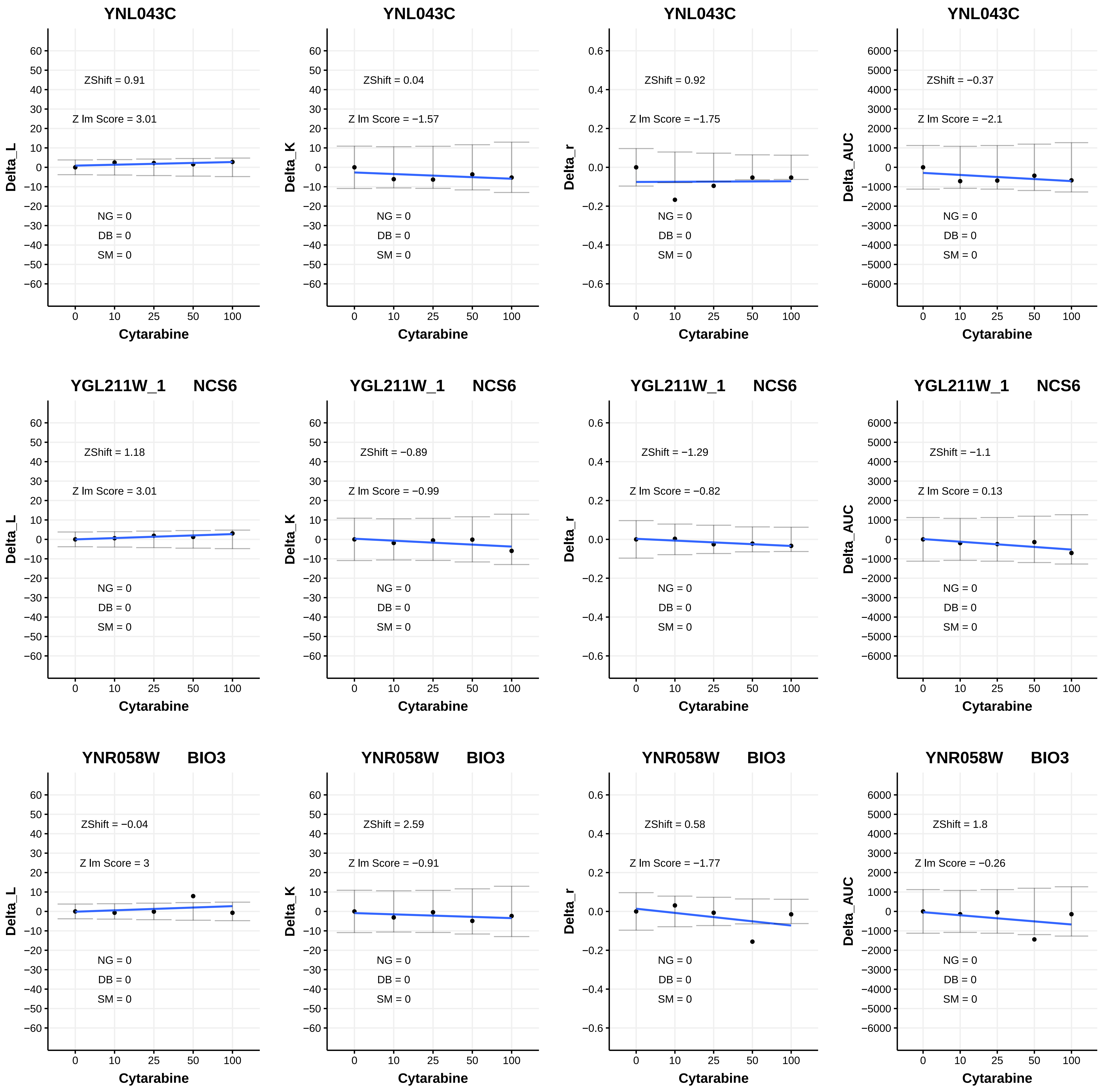
YNL043C
Delta_L
ZShift = 0.91
Z lm Score = 3.01
NG = 0
DB = 0
SM = 0
Cytarabine
YNL043C
Delta_K
ZShift = 0.04
Z lm Score = −1.57
NG = 0
DB = 0
SM = 0
Cytarabine
YNL043C
Delta_r
ZShift = 0.92
Z lm Score = −1.75
NG = 0
DB = 0
SM = 0
Cytarabine
YNL043C
Delta_AUC
ZShift = −0.37
Z lm Score = −2.1
NG = 0
DB = 0
SM = 0
Cytarabine
YGL211W_1 NCS6
Delta_L
ZShift = 1.18
Z lm Score = 3.01
NG = 0
DB = 0
SM = 0
Cytarabine
YGL211W_1 NCS6
Delta_K
ZShift = −0.89
Z lm Score = −0.99
NG = 0
DB = 0
SM = 0
Cytarabine
YGL211W_1 NCS6
Delta_r
ZShift = −1.29
Z lm Score = −0.82
NG = 0
DB = 0
SM = 0
Cytarabine
YGL211W_1 NCS6
Delta_AUC
ZShift = −1.1
Z lm Score = 0.13
NG = 0
DB = 0
SM = 0
Cytarabine
YNR058W BIO3
Delta_L
ZShift = −0.04
Z lm Score = 3
NG = 0
DB = 0
SM = 0
Cytarabine
YNR058W BIO3
Delta_K
ZShift = 2.59
Z lm Score = −0.91
NG = 0
DB = 0
SM = 0
Cytarabine
YNR058W BIO3
Delta_r
ZShift = 0.58
Z lm Score = −1.77
NG = 0
DB = 0
SM = 0
Cytarabine
YNR058W BIO3
Delta_AUC
ZShift = 1.8
Z lm Score = −0.26
NG = 0
DB = 0
SM = 0
Cytarabine

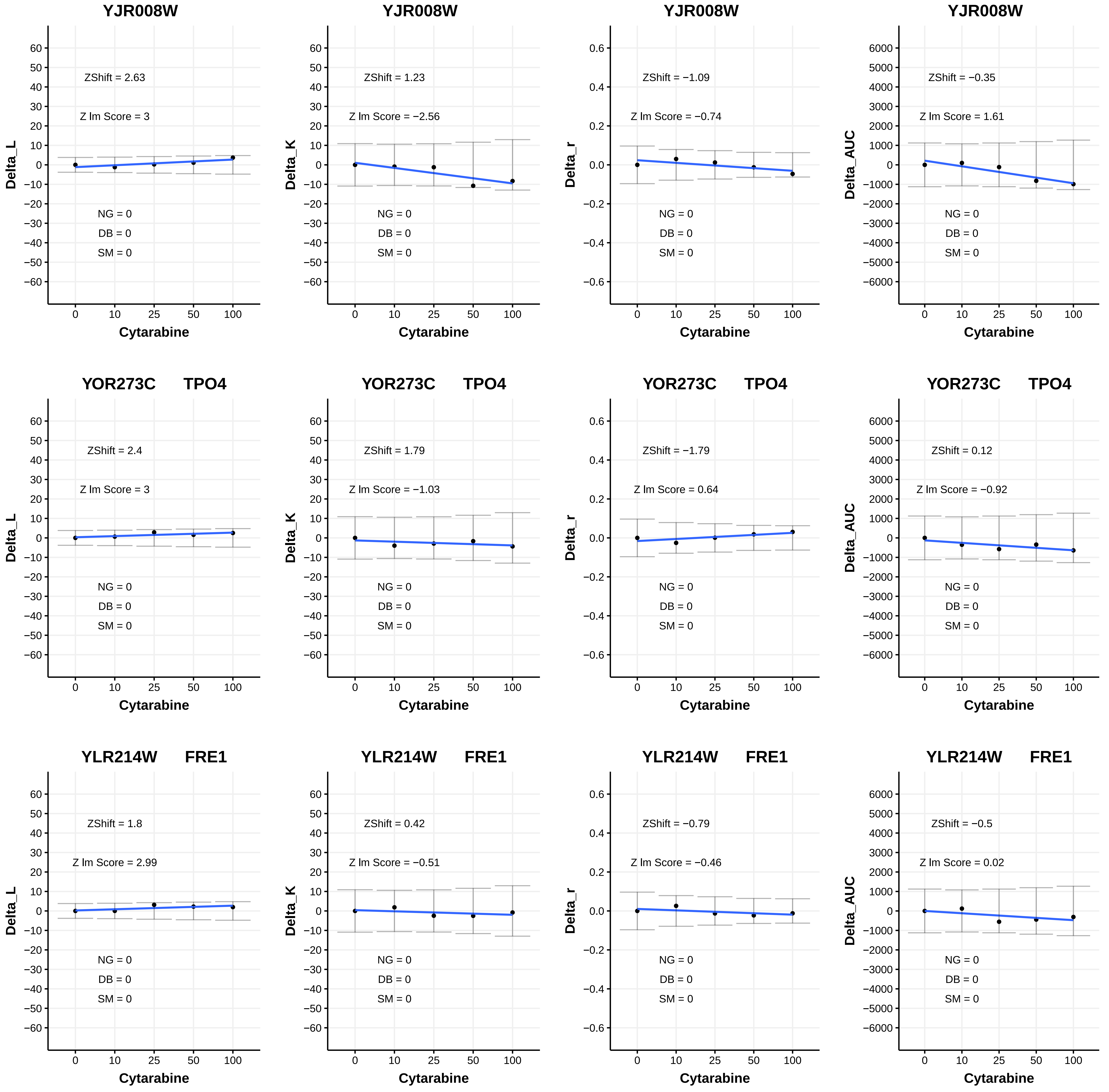

YJR008W
ZShift = 2.63
Z lm Score = 3
NG = 0
DB = 0
SM = 0
Delta_L
Cytarabine
YJR008W
ZShift = 1.23
Z lm Score = −2.56
NG = 0
DB = 0
SM = 0
Delta_K
Cytarabine
YJR008W
ZShift = −1.09
Z lm Score = −0.74
NG = 0
DB = 0
SM = 0
Delta_r
Cytarabine
YJR008W
ZShift = −0.35
Z lm Score = 1.61
NG = 0
DB = 0
SM = 0
Delta_AUC
Cytarabine
YOR273C TPO4
ZShift = 2.4
Z lm Score = 3
NG = 0
DB = 0
SM = 0
Delta_L
Cytarabine
YOR273C TPO4
ZShift = 1.79
Z lm Score = −1.03
NG = 0
DB = 0
SM = 0
Delta_K
Cytarabine
YOR273C TPO4
ZShift = −1.79
Z lm Score = 0.64
NG = 0
DB = 0
SM = 0
Delta_r
Cytarabine
YOR273C TPO4
ZShift = 0.12
Z lm Score = −0.92
NG = 0
DB = 0
SM = 0
Delta_AUC
Cytarabine
YLR214W FRE1
ZShift = 1.8
Z lm Score = 2.99
NG = 0
DB = 0
SM = 0
Delta_L
Cytarabine
YLR214W FRE1
ZShift = 0.42
Z lm Score = −0.51
NG = 0
DB = 0
SM = 0
Delta_K
Cytarabine
YLR214W FRE1
ZShift = −0.79
Z lm Score = −0.46
NG = 0
DB = 0
SM = 0
Delta_r
Cytarabine
YLR214W FRE1
ZShift = −0.5
Z lm Score = 0.02
NG = 0
DB = 0
SM = 0
Delta_AUC
Cytarabine

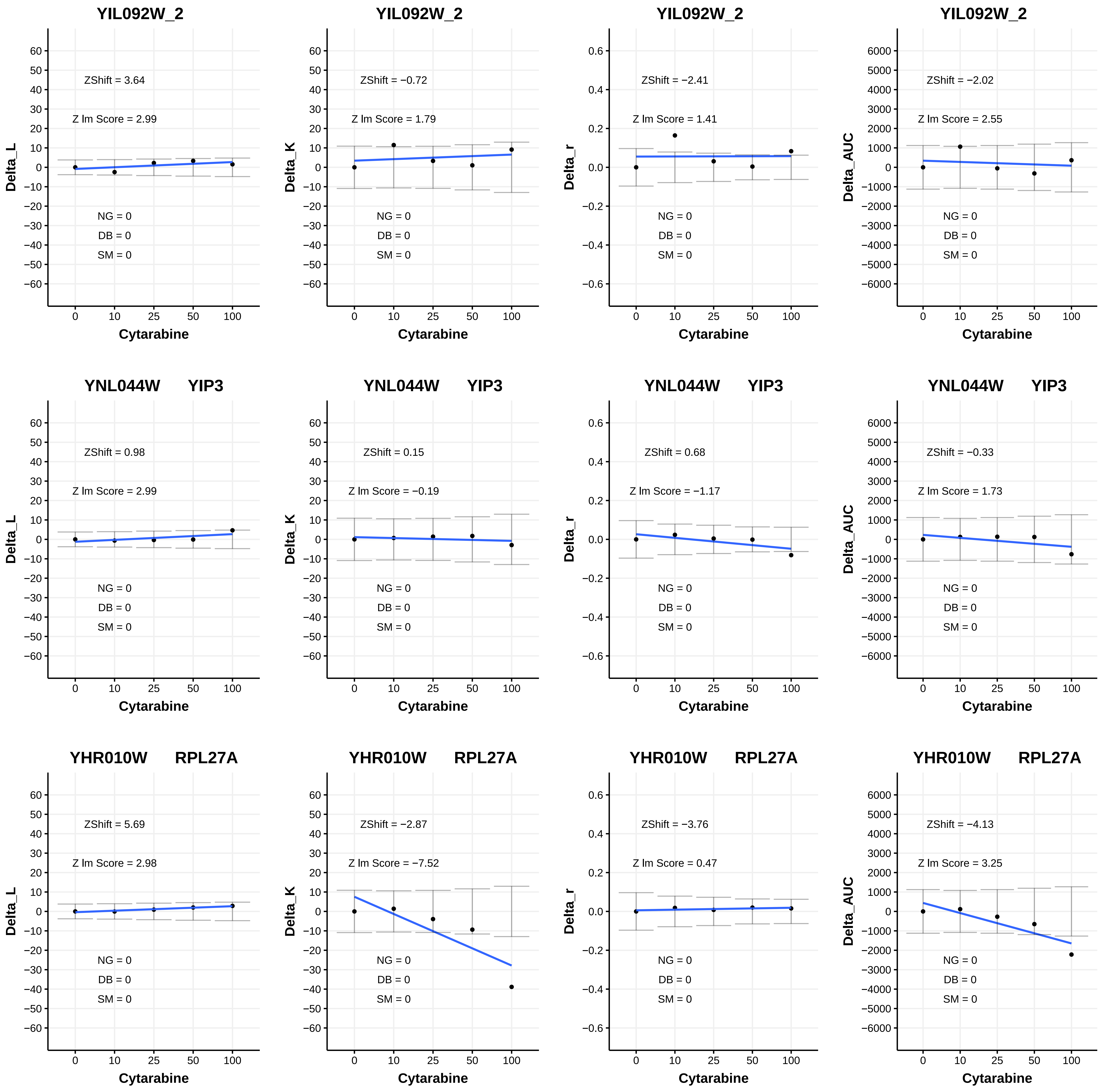

YIL092W_2
ZShift = 3.64
Z lm Score = 2.99
NG = 0
DB = 0
SM = 0
Delta_L
Cytarabine
YIL092W_2
ZShift = −0.72
Z lm Score = 1.79
NG = 0
DB = 0
SM = 0
Delta_K
Cytarabine
YIL092W_2
ZShift = −2.41
Z lm Score = 1.41
NG = 0
DB = 0
SM = 0
Delta_r
Cytarabine
YIL092W_2
ZShift = −2.02
Z lm Score = 2.55
NG = 0
DB = 0
SM = 0
Delta_AUC
Cytarabine
YNL044W YIP3
ZShift = 0.98
Z lm Score = 2.99
NG = 0
DB = 0
SM = 0
Delta_L
Cytarabine
YNL044W YIP3
ZShift = 0.15
Z lm Score = −0.19
NG = 0
DB = 0
SM = 0
Delta_K
Cytarabine
YNL044W YIP3
ZShift = 0.68
Z lm Score = −1.17
NG = 0
DB = 0
SM = 0
Delta_r
Cytarabine
YNL044W YIP3
ZShift = −0.33
Z lm Score = 1.73
NG = 0
DB = 0
SM = 0
Delta_AUC
Cytarabine
YHR010W RPL27A
ZShift = 5.69
Z lm Score = 2.98
NG = 0
DB = 0
SM = 0
Delta_L
Cytarabine
YHR010W RPL27A
ZShift = −2.87
Z lm Score = −7.52
NG = 0
DB = 0
SM = 0
Delta_K
Cytarabine
YHR010W RPL27A
ZShift = −3.76
Z lm Score = 0.47
NG = 0
DB = 0
SM = 0
Delta_r
Cytarabine
YHR010W RPL27A
ZShift = −4.13
Z lm Score = 3.25
NG = 0
DB = 0
SM = 0
Delta_AUC
Cytarabine

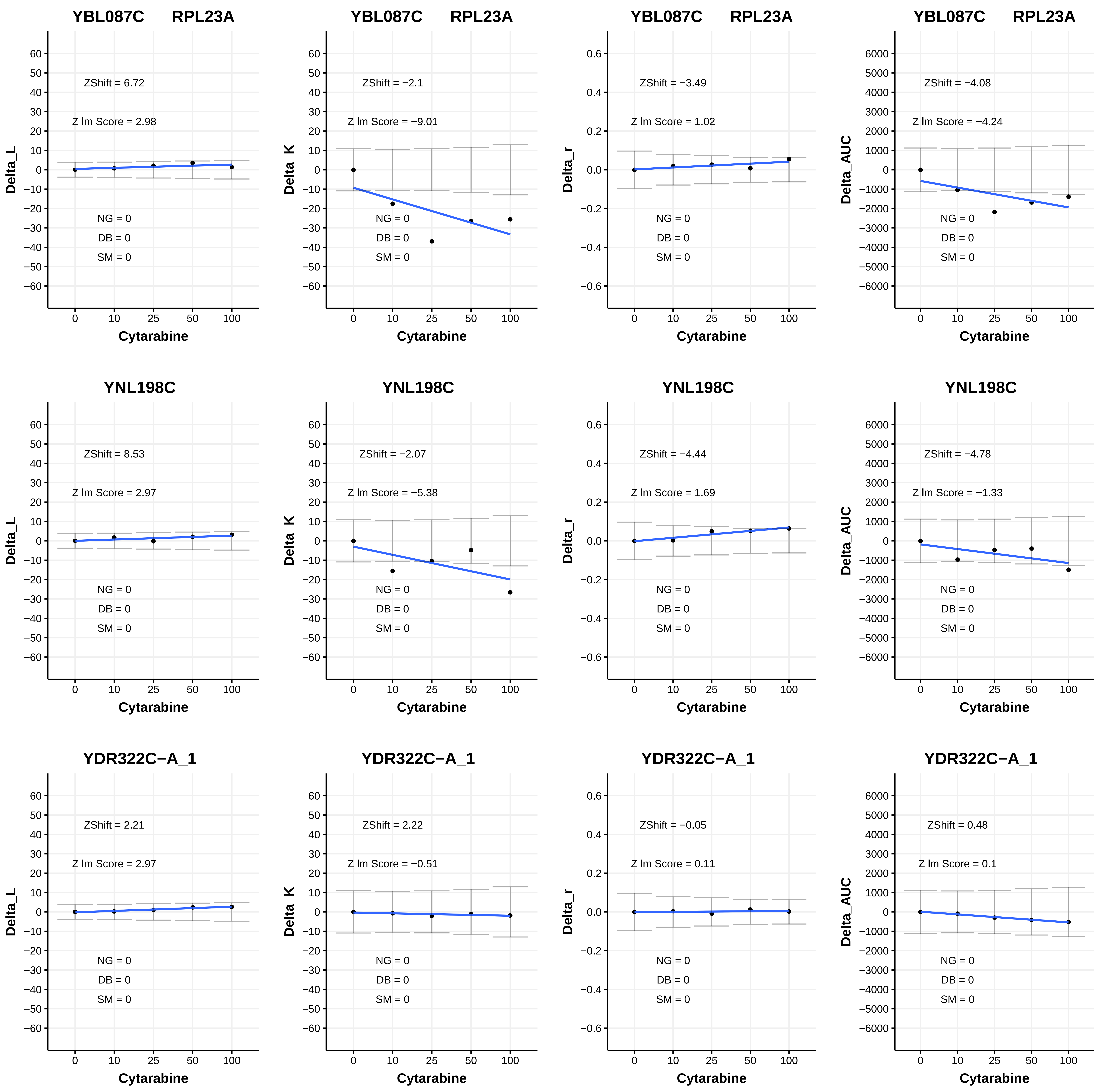
YBL087C RPL23A
ZShift = 6.72
Z lm Score = 2.98
NG = 0
DB = 0
SM = 0
Delta_L
Cytarabine
YBL087C RPL23A
ZShift = −2.1
Z lm Score = −9.01
NG = 0
DB = 0
SM = 0
Delta_K
Cytarabine
YBL087C RPL23A
ZShift = −3.49
Z lm Score = 1.02
NG = 0
DB = 0
SM = 0
Delta_r
Cytarabine
YBL087C RPL23A
ZShift = −4.08
Z lm Score = −4.24
NG = 0
DB = 0
SM = 0
Delta_AUC
Cytarabine
YNL198C
ZShift = 8.53
Z lm Score = 2.97
NG = 0
DB = 0
SM = 0
Delta_L
Cytarabine
YNL198C
ZShift = −2.07
Z lm Score = −5.38
NG = 0
DB = 0
SM = 0
Delta_K
Cytarabine
YNL198C
ZShift = −4.44
Z lm Score = 1.69
NG = 0
DB = 0
SM = 0
Delta_r
Cytarabine
YNL198C
ZShift = −4.78
Z lm Score = −1.33
NG = 0
DB = 0
SM = 0
Delta_AUC
Cytarabine
YDR322C−A_1
ZShift = 2.21
Z lm Score = 2.97
NG = 0
DB = 0
SM = 0
Delta_L
Cytarabine
YDR322C−A_1
ZShift = 2.22
Z lm Score = −0.51
NG = 0
DB = 0
SM = 0
Delta_K
Cytarabine
YDR322C−A_1
ZShift = −0.05
Z lm Score = 0.11
NG = 0
DB = 0
SM = 0
Delta_r
Cytarabine
YDR322C−A_1
ZShift = 0.48
Z lm Score = 0.1
NG = 0
DB = 0
SM = 0
Delta_AUC
Cytarabine

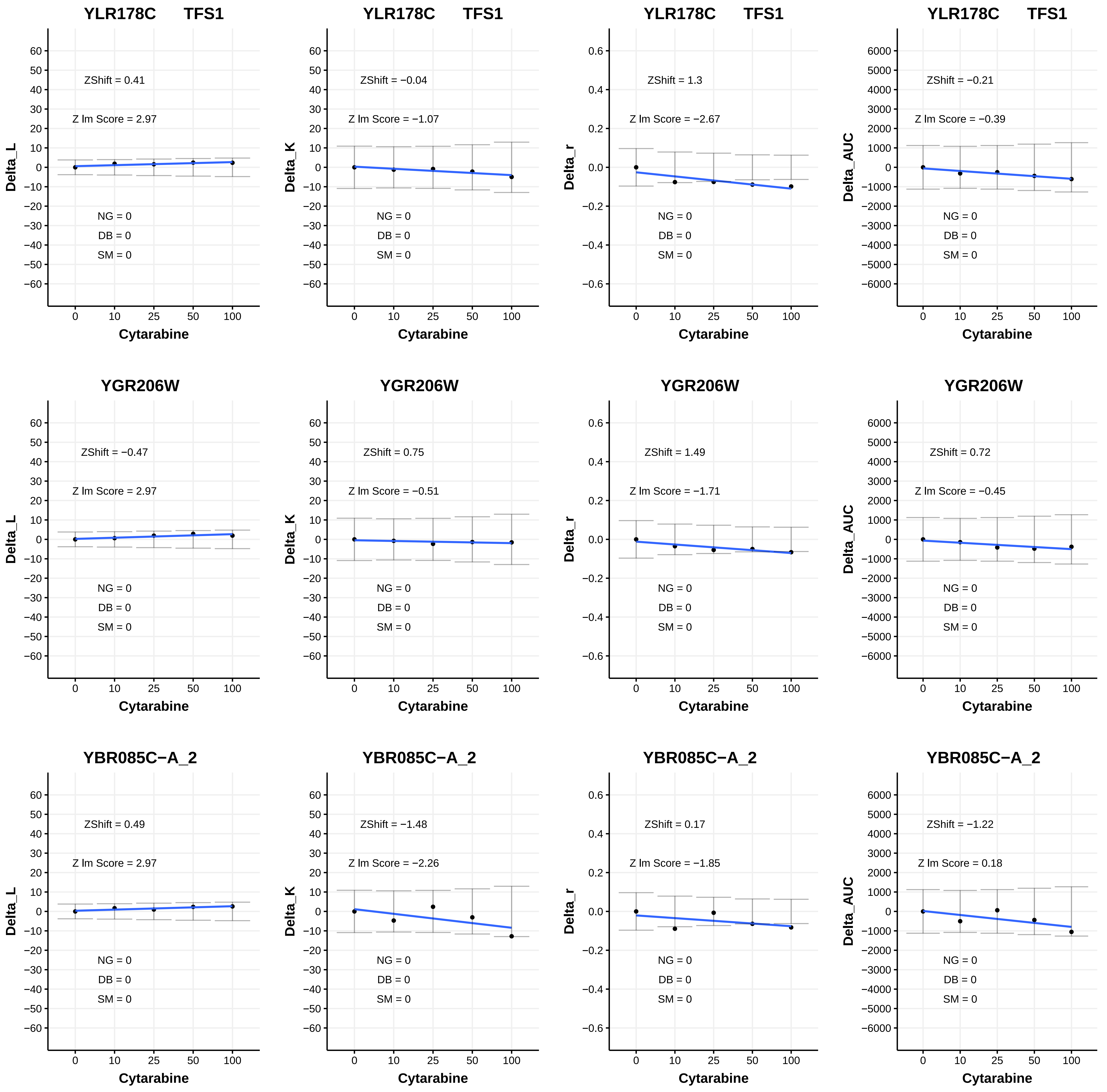

YLR178C TFS1
ZShift = 0.41
Z lm Score = 2.97
NG = 0
DB = 0
SM = 0
Delta_L
Cytarabine
YLR178C TFS1
ZShift = −0.04
Z lm Score = −1.07
NG = 0
DB = 0
SM = 0
Delta_K
Cytarabine
YLR178C TFS1
ZShift = 1.3
Z lm Score = −2.67
NG = 0
DB = 0
SM = 0
Delta_r
Cytarabine
YLR178C TFS1
ZShift = −0.21
Z lm Score = −0.39
NG = 0
DB = 0
SM = 0
Delta_AUC
Cytarabine
YGR206W
ZShift = −0.47
Z lm Score = 2.97
NG = 0
DB = 0
SM = 0
Delta_L
Cytarabine
YGR206W
ZShift = 0.75
Z lm Score = −0.51
NG = 0
DB = 0
SM = 0
Delta_K
Cytarabine
YGR206W
ZShift = 1.49
Z lm Score = −1.71
NG = 0
DB = 0
SM = 0
Delta_r
Cytarabine
YGR206W
ZShift = 0.72
Z lm Score = −0.45
NG = 0
DB = 0
SM = 0
Delta_AUC
Cytarabine
YBR085C−A_2
ZShift = 0.49
Z lm Score = 2.97
NG = 0
DB = 0
SM = 0
Delta_L
Cytarabine
YBR085C−A_2
ZShift = −1.48
Z lm Score = −2.26
NG = 0
DB = 0
SM = 0
Delta_K
Cytarabine
YBR085C−A_2
ZShift = 0.17
Z lm Score = −1.85
NG = 0
DB = 0
SM = 0
Delta_r
Cytarabine
YBR085C−A_2
ZShift = −1.22
Z lm Score = 0.18
NG = 0
DB = 0
SM = 0
Delta_AUC
Cytarabine

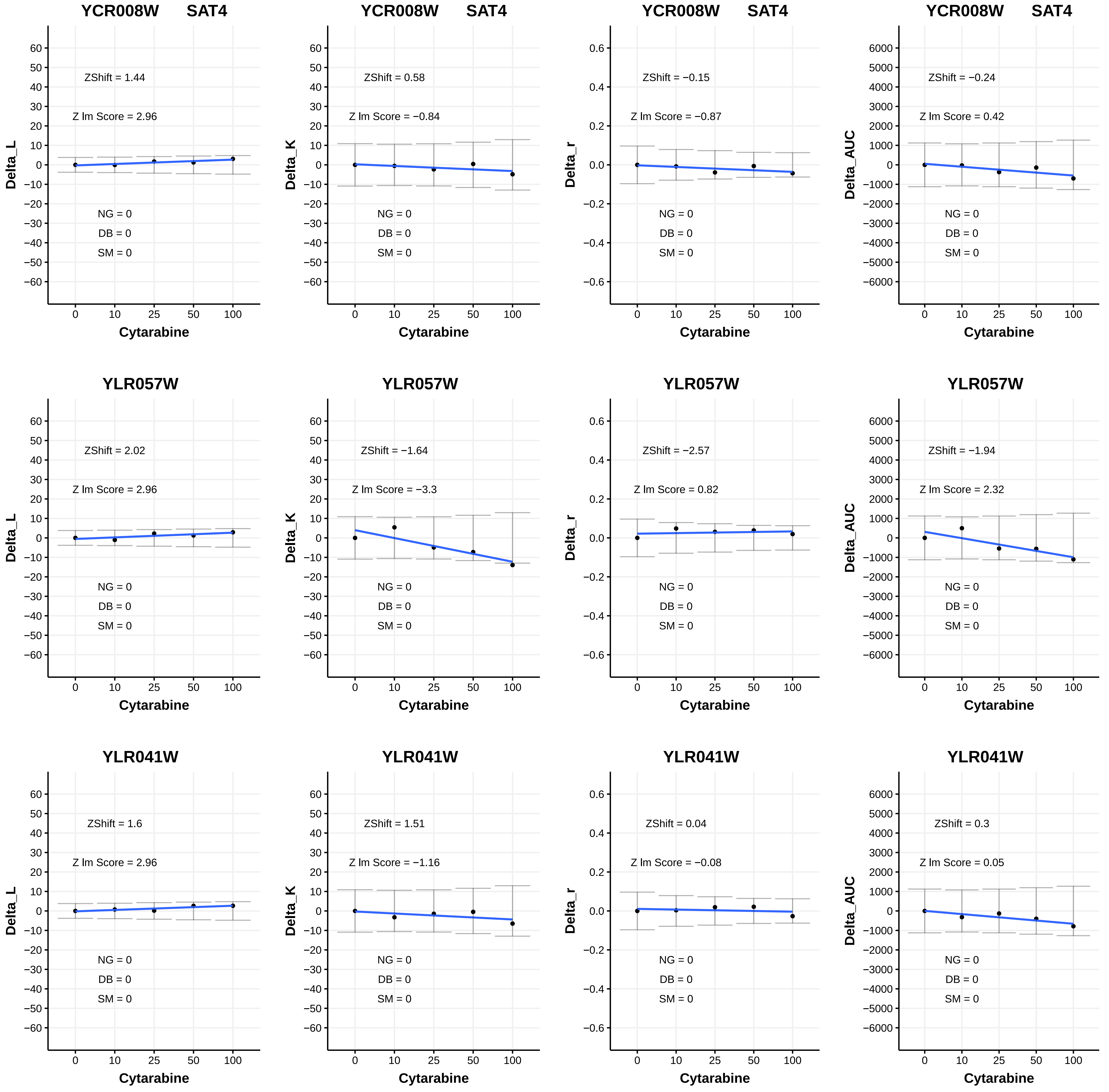

YCR008W SAT4
ZShift = 1.44
Z lm Score = 2.96
NG = 0
DB = 0
SM = 0
Delta_L
Cytarabine
YCR008W SAT4
ZShift = 0.58
Z lm Score = −0.84
NG = 0
DB = 0
SM = 0
Delta_K
Cytarabine
YCR008W SAT4
ZShift = −0.15
Z lm Score = −0.87
NG = 0
DB = 0
SM = 0
Delta_r
Cytarabine
YCR008W SAT4
ZShift = −0.24
Z lm Score = 0.42
NG = 0
DB = 0
SM = 0
Delta_AUC
Cytarabine
YLR057W
ZShift = 2.02
Z lm Score = 2.96
NG = 0
DB = 0
SM = 0
Delta_L
Cytarabine
YLR057W
ZShift = −1.64
Z lm Score = −3.3
NG = 0
DB = 0
SM = 0
Delta_K
Cytarabine
YLR057W
ZShift = −2.57
Z lm Score = 0.82
NG = 0
DB = 0
SM = 0
Delta_r
Cytarabine
YLR057W
ZShift = −1.94
Z lm Score = 2.32
NG = 0
DB = 0
SM = 0
Delta_AUC
Cytarabine
YLR041W
ZShift = 1.6
Z lm Score = 2.96
NG = 0
DB = 0
SM = 0
Delta_L
Cytarabine
YLR041W
ZShift = 1.51
Z lm Score = −1.16
NG = 0
DB = 0
SM = 0
Delta_K
Cytarabine
YLR041W
ZShift = 0.04
Z lm Score = −0.08
NG = 0
DB = 0
SM = 0
Delta_r
Cytarabine
YLR041W
ZShift = 0.3
Z lm Score = 0.05
NG = 0
DB = 0
SM = 0
Delta_AUC
Cytarabine

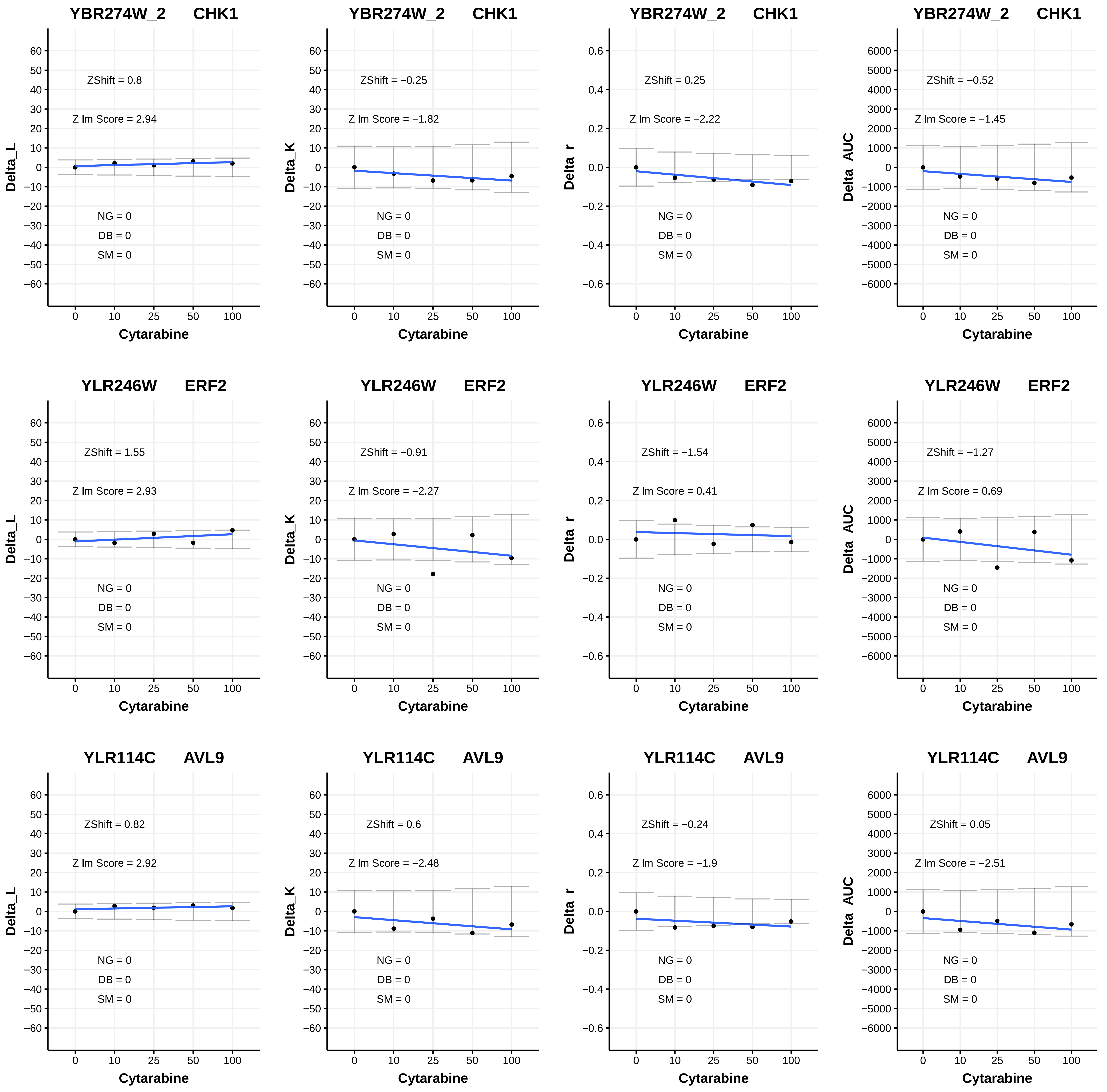
YBR274W_2 CHK1
ZShift = 0.8
Z lm Score = 2.94
NG = 0
DB = 0
SM = 0
Delta_L
Cytarabine
YBR274W_2 CHK1
ZShift = −0.25
Z lm Score = −1.82
NG = 0
DB = 0
SM = 0
Delta_K
Cytarabine
YBR274W_2 CHK1
ZShift = 0.25
Z lm Score = −2.22
NG = 0
DB = 0
SM = 0
Delta_r
Cytarabine
YBR274W_2 CHK1
ZShift = −0.52
Z lm Score = −1.45
NG = 0
DB = 0
SM = 0
Delta_AUC
Cytarabine
YLR246W ERF2
ZShift = 1.55
Z lm Score = 2.93
NG = 0
DB = 0
SM = 0
Delta_L
Cytarabine
YLR246W ERF2
ZShift = −0.91
Z lm Score = −2.27
NG = 0
DB = 0
SM = 0
Delta_K
Cytarabine
YLR246W ERF2
ZShift = −1.54
Z lm Score = 0.41
NG = 0
DB = 0
SM = 0
Delta_r
Cytarabine
YLR246W ERF2
ZShift = −1.27
Z lm Score = 0.69
NG = 0
DB = 0
SM = 0
Delta_AUC
Cytarabine
YLR114C AVL9
ZShift = 0.82
Z lm Score = 2.92
NG = 0
DB = 0
SM = 0
Delta_L
Cytarabine
YLR114C AVL9
ZShift = 0.6
Z lm Score = −2.48
NG = 0
DB = 0
SM = 0
Delta_K
Cytarabine
YLR114C AVL9
ZShift = −0.24
Z lm Score = −1.9
NG = 0
DB = 0
SM = 0
Delta_r
Cytarabine
YLR114C AVL9
ZShift = 0.05
Z lm Score = −2.51
NG = 0
DB = 0
SM = 0
Delta_AUC
Cytarabine
0
10
25
50
100

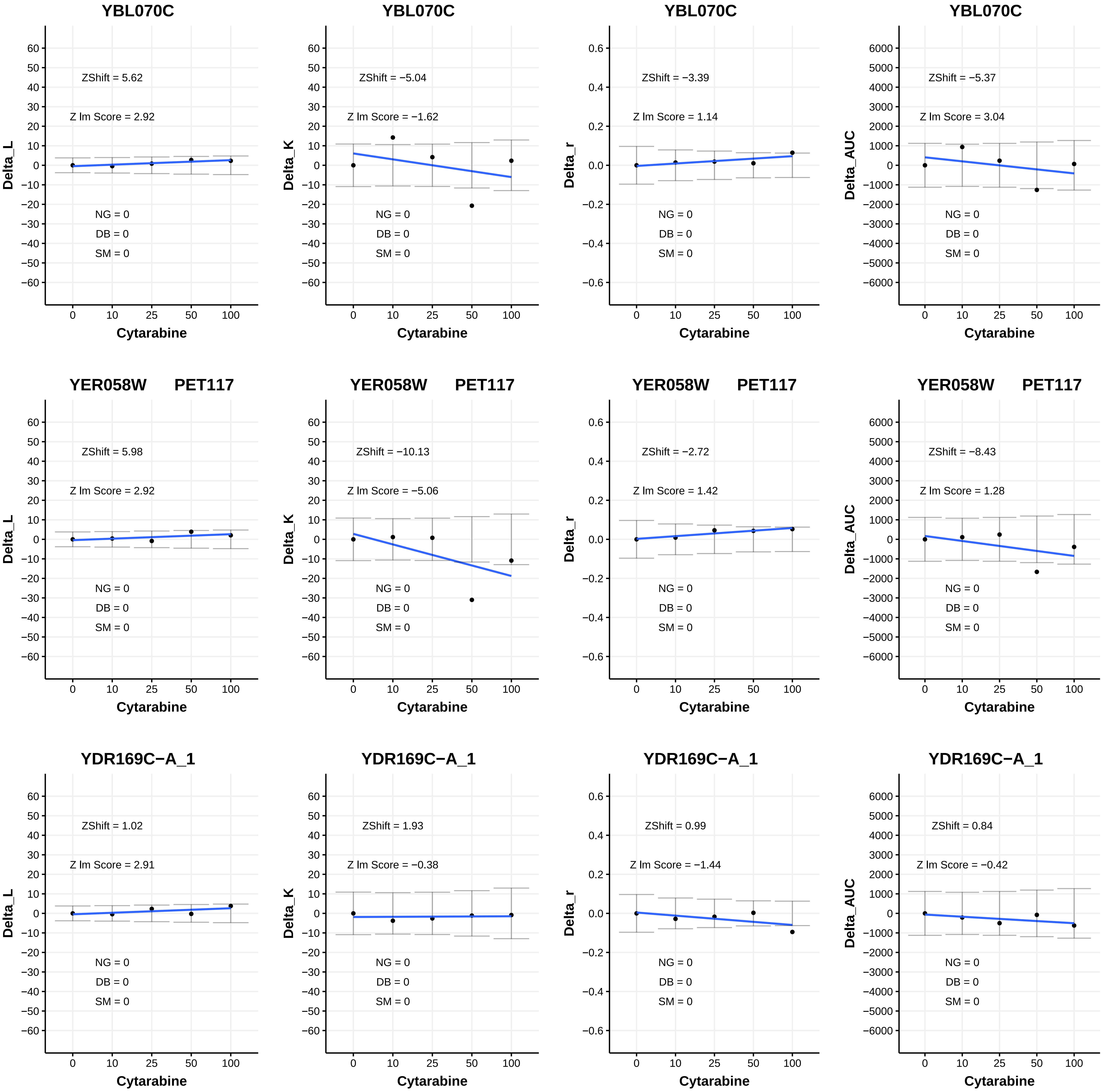
YBL070C
ZShift = 5.62
Z lm Score = 2.92
NG = 0
DB = 0
SM = 0
Delta_L
Cytarabine
YBL070C
ZShift = −5.04
Z lm Score = −1.62
NG = 0
DB = 0
SM = 0
Delta_K
Cytarabine
YBL070C
ZShift = −3.39
Z lm Score = 1.14
NG = 0
DB = 0
SM = 0
Delta_r
Cytarabine
YBL070C
ZShift = −5.37
Z lm Score = 3.04
NG = 0
DB = 0
SM = 0
Delta_AUC
Cytarabine
YER058W PET117
ZShift = 5.98
Z lm Score = 2.92
NG = 0
DB = 0
SM = 0
Delta_L
Cytarabine
YER058W PET117
ZShift = −10.13
Z lm Score = −5.06
NG = 0
DB = 0
SM = 0
Delta_K
Cytarabine
YER058W PET117
ZShift = −2.72
Z lm Score = 1.42
NG = 0
DB = 0
SM = 0
Delta_r
Cytarabine
YER058W PET117
ZShift = −8.43
Z lm Score = 1.28
NG = 0
DB = 0
SM = 0
Delta_AUC
Cytarabine
YDR169C−A_1
ZShift = 1.02
Z lm Score = 2.91
NG = 0
DB = 0
SM = 0
Delta_L
Cytarabine
YDR169C−A_1
ZShift = 1.93
Z lm Score = −0.38
NG = 0
DB = 0
SM = 0
Delta_K
Cytarabine
YDR169C−A_1
ZShift = 0.99
Z lm Score = −1.44
NG = 0
DB = 0
SM = 0
Delta_r
Cytarabine
YDR169C−A_1
ZShift = 0.84
Z lm Score = −0.42
NG = 0
DB = 0
SM = 0
Delta_AUC
Cytarabine

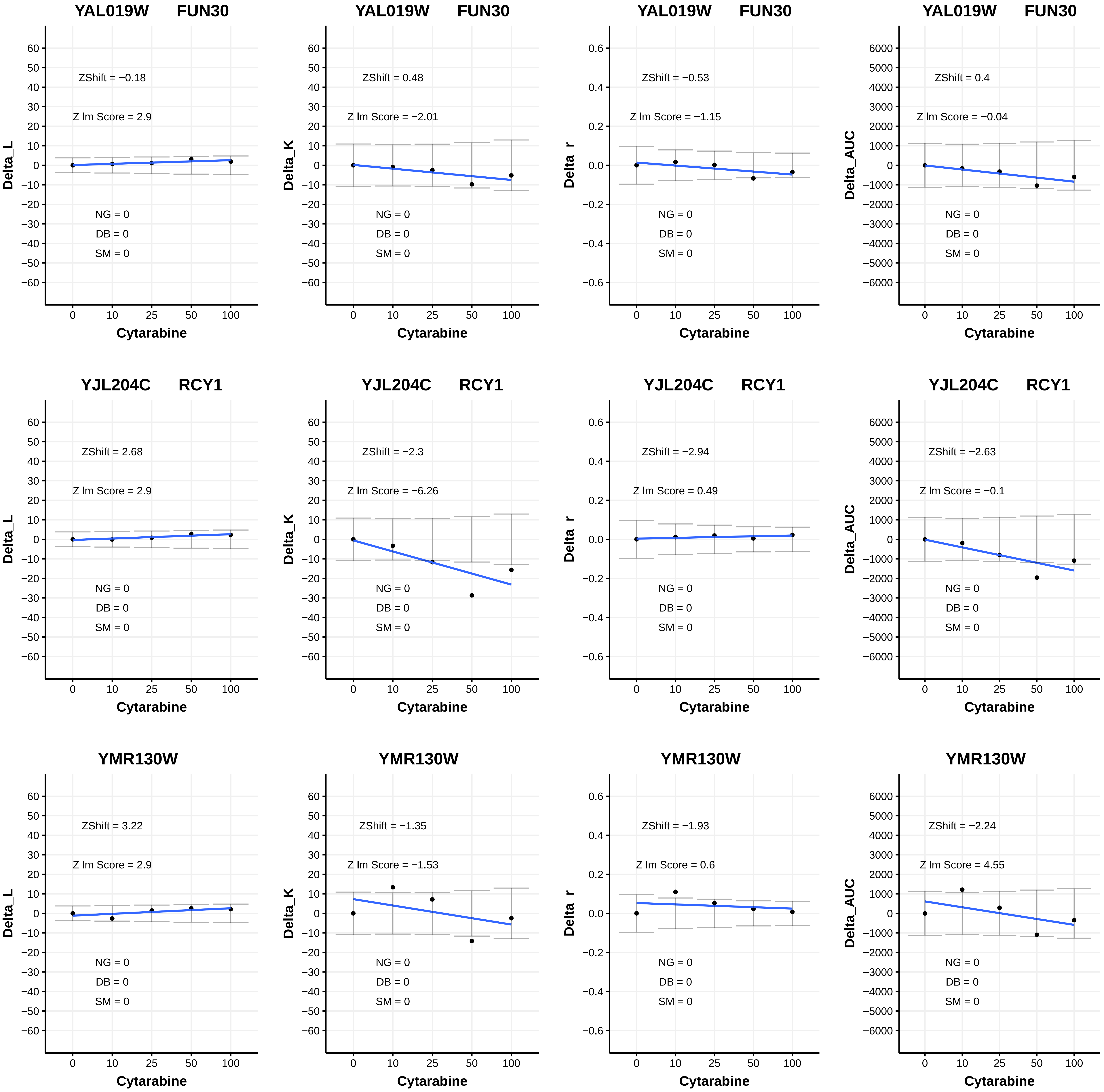
YAL019W FUN30
ZShift = −0.18
Z lm Score = 2.9
NG = 0
DB = 0
SM = 0
Delta_L
Cytarabine
YAL019W FUN30
ZShift = 0.48
Z lm Score = −2.01
NG = 0
DB = 0
SM = 0
Delta_K
Cytarabine
YAL019W FUN30
ZShift = −0.53
Z lm Score = −1.15
NG = 0
DB = 0
SM = 0
Delta_r
Cytarabine
YAL019W FUN30
ZShift = 0.4
Z lm Score = −0.04
NG = 0
DB = 0
SM = 0
Delta_AUC
Cytarabine
YJL204C RCY1
ZShift = 2.68
Z lm Score = 2.9
NG = 0
DB = 0
SM = 0
Delta_L
Cytarabine
YJL204C RCY1
ZShift = −2.3
Z lm Score = −6.26
NG = 0
DB = 0
SM = 0
Delta_K
Cytarabine
YJL204C RCY1
ZShift = −2.94
Z lm Score = 0.49
NG = 0
DB = 0
SM = 0
Delta_r
Cytarabine
YJL204C RCY1
ZShift = −2.63
Z lm Score = −0.1
NG = 0
DB = 0
SM = 0
Delta_AUC
Cytarabine
YMR130W
ZShift = 3.22
Z lm Score = 2.9
NG = 0
DB = 0
SM = 0
Delta_L
Cytarabine
YMR130W
ZShift = −1.35
Z lm Score = −1.53
NG = 0
DB = 0
SM = 0
Delta_K
Cytarabine
YMR130W
ZShift = −1.93
Z lm Score = 0.6
NG = 0
DB = 0
SM = 0
Delta_r
Cytarabine
YMR130W
ZShift = −2.24
Z lm Score = 4.55
NG = 0
DB = 0
SM = 0
Delta_AUC
Cytarabine
0 10 25 50 100

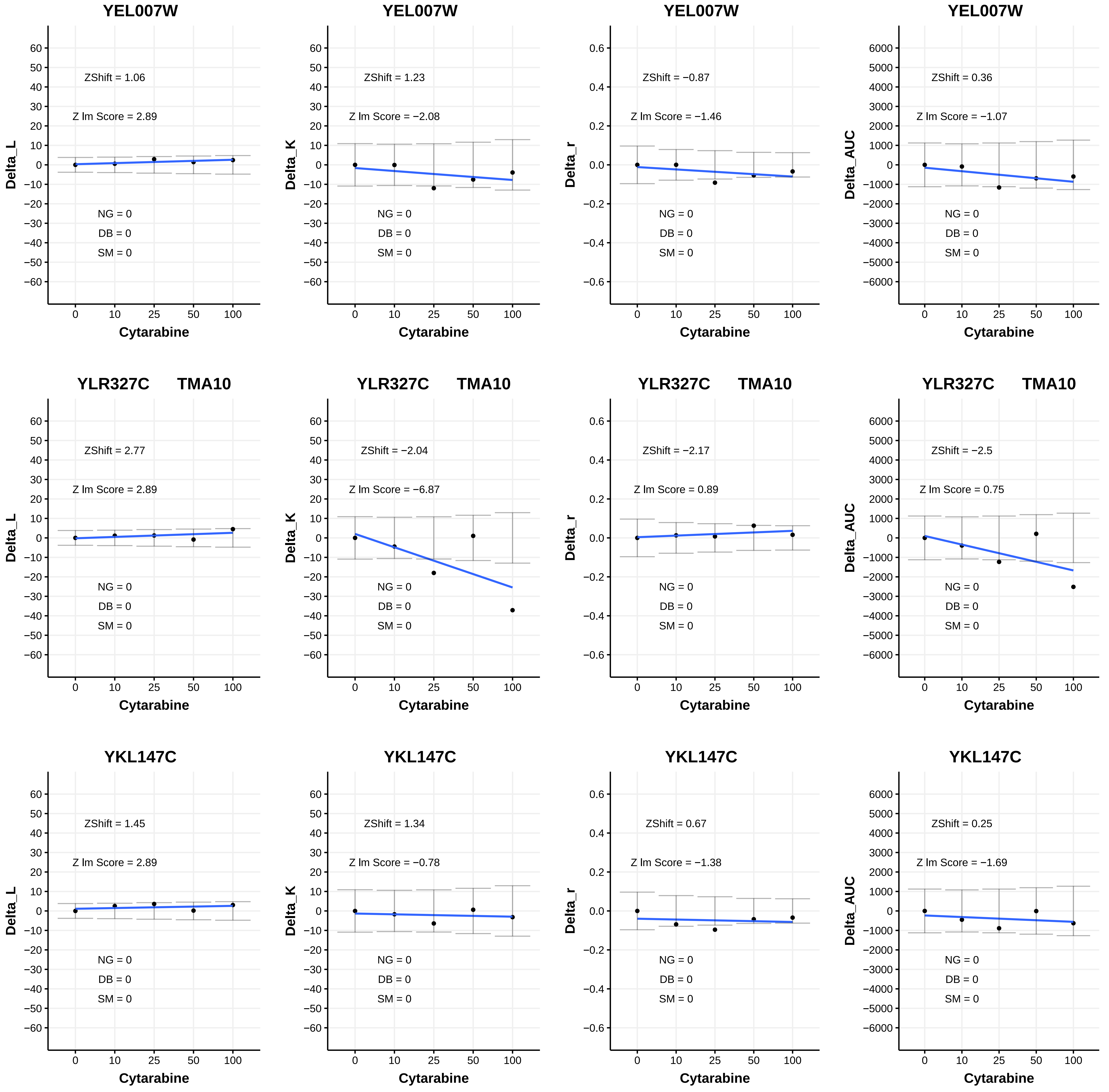

YEL007W
Delta_L
ZShift = 1.06
Z lm Score = 2.89
NG = 0
DB = 0
SM = 0
Cytarabine
YEL007W
Delta_K
ZShift = 1.23
Z lm Score = −2.08
NG = 0
DB = 0
SM = 0
Cytarabine
YEL007W
Delta_r
ZShift = −0.87
Z lm Score = −1.46
NG = 0
DB = 0
SM = 0
Cytarabine
YEL007W
Delta_AUC
ZShift = 0.36
Z lm Score = −1.07
NG = 0
DB = 0
SM = 0
Cytarabine
YLR327C TMA10
Delta_L
ZShift = 2.77
Z lm Score = 2.89
NG = 0
DB = 0
SM = 0
Cytarabine
YLR327C TMA10
Delta_K
ZShift = −2.04
Z lm Score = −6.87
NG = 0
DB = 0
SM = 0
Cytarabine
YLR327C TMA10
Delta_r
ZShift = −2.17
Z lm Score = 0.89
NG = 0
DB = 0
SM = 0
Cytarabine
YLR327C TMA10
Delta_AUC
ZShift = −2.5
Z lm Score = 0.75
NG = 0
DB = 0
SM = 0
Cytarabine
YKL147C
Delta_L
ZShift = 1.45
Z lm Score = 2.89
NG = 0
DB = 0
SM = 0
Cytarabine
YKL147C
Delta_K
ZShift = 1.34
Z lm Score = −0.78
NG = 0
DB = 0
SM = 0
Cytarabine
YKL147C
Delta_r
ZShift = 0.67
Z lm Score = −1.38
NG = 0
DB = 0
SM = 0
Cytarabine
YKL147C
Delta_AUC
ZShift = 0.25
Z lm Score = −1.69
NG = 0
DB = 0
SM = 0
Cytarabine

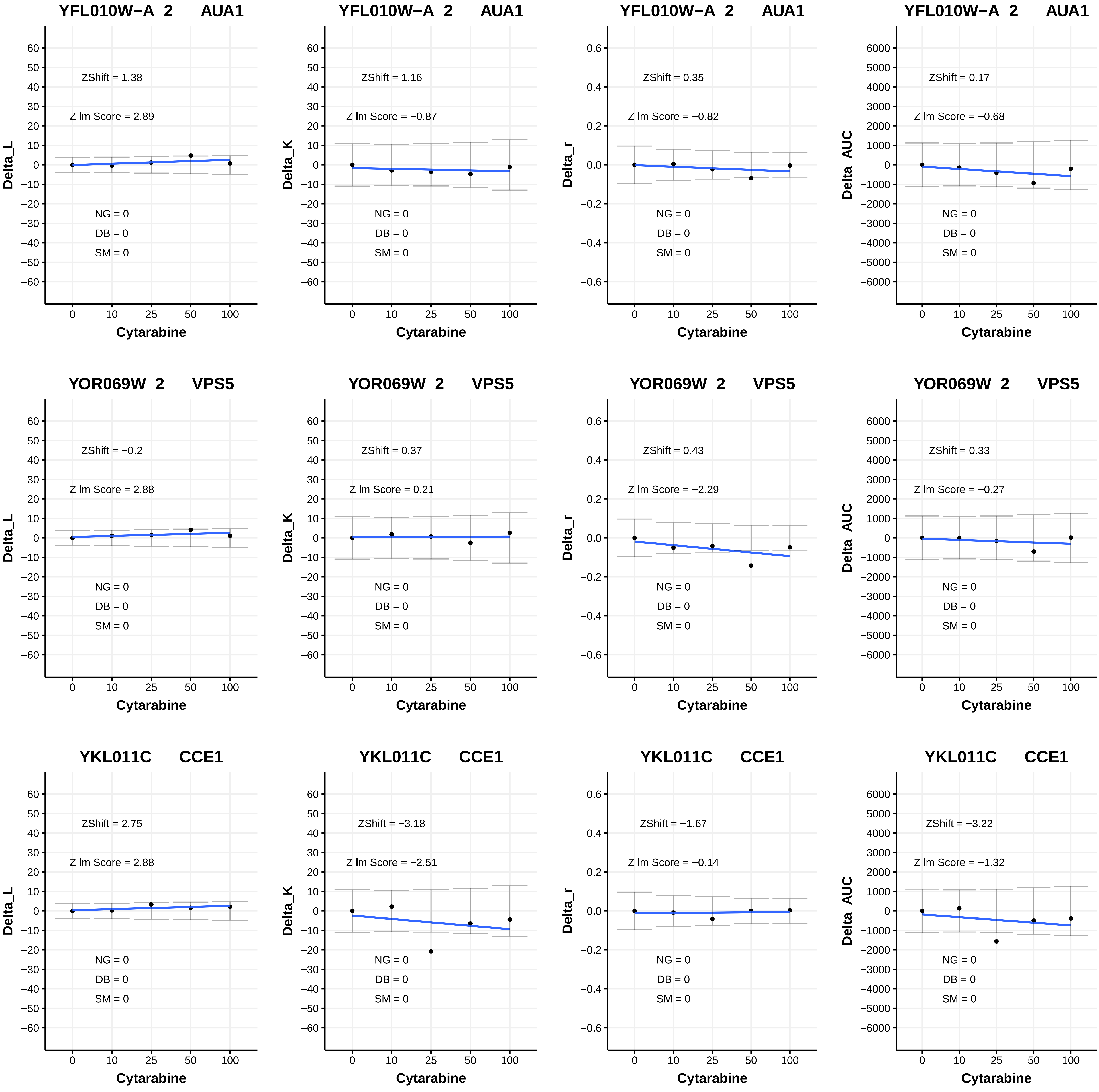

YFL010W−A_2 AUA1
ZShift = 1.38
Z lm Score = 2.89
NG = 0
DB = 0
SM = 0
Delta_L
Cytarabine
YFL010W−A_2 AUA1
ZShift = 1.16
Z lm Score = −0.87
NG = 0
DB = 0
SM = 0
Delta_K
Cytarabine
YFL010W−A_2 AUA1
ZShift = 0.35
Z lm Score = −0.82
NG = 0
DB = 0
SM = 0
Delta_r
Cytarabine
YFL010W−A_2 AUA1
ZShift = 0.17
Z lm Score = −0.68
NG = 0
DB = 0
SM = 0
Delta_AUC
Cytarabine
YOR069W_2 VPS5
ZShift = −0.2
Z lm Score = 2.88
NG = 0
DB = 0
SM = 0
Delta_L
Cytarabine
YOR069W_2 VPS5
ZShift = 0.37
Z lm Score = 0.21
NG = 0
DB = 0
SM = 0
Delta_K
Cytarabine
YOR069W_2 VPS5
ZShift = 0.43
Z lm Score = −2.29
NG = 0
DB = 0
SM = 0
Delta_r
Cytarabine
YOR069W_2 VPS5
ZShift = 0.33
Z lm Score = −0.27
NG = 0
DB = 0
SM = 0
Delta_AUC
Cytarabine
YKL011C CCE1
ZShift = 2.75
Z lm Score = 2.88
NG = 0
DB = 0
SM = 0
Delta_L
Cytarabine
YKL011C CCE1
ZShift = −3.18
Z lm Score = −2.51
NG = 0
DB = 0
SM = 0
Delta_K
Cytarabine
YKL011C CCE1
ZShift = −1.67
Z lm Score = −0.14
NG = 0
DB = 0
SM = 0
Delta_r
Cytarabine
YKL011C CCE1
ZShift = −3.22
Z lm Score = −1.32
NG = 0
DB = 0
SM = 0
Delta_AUC
Cytarabine

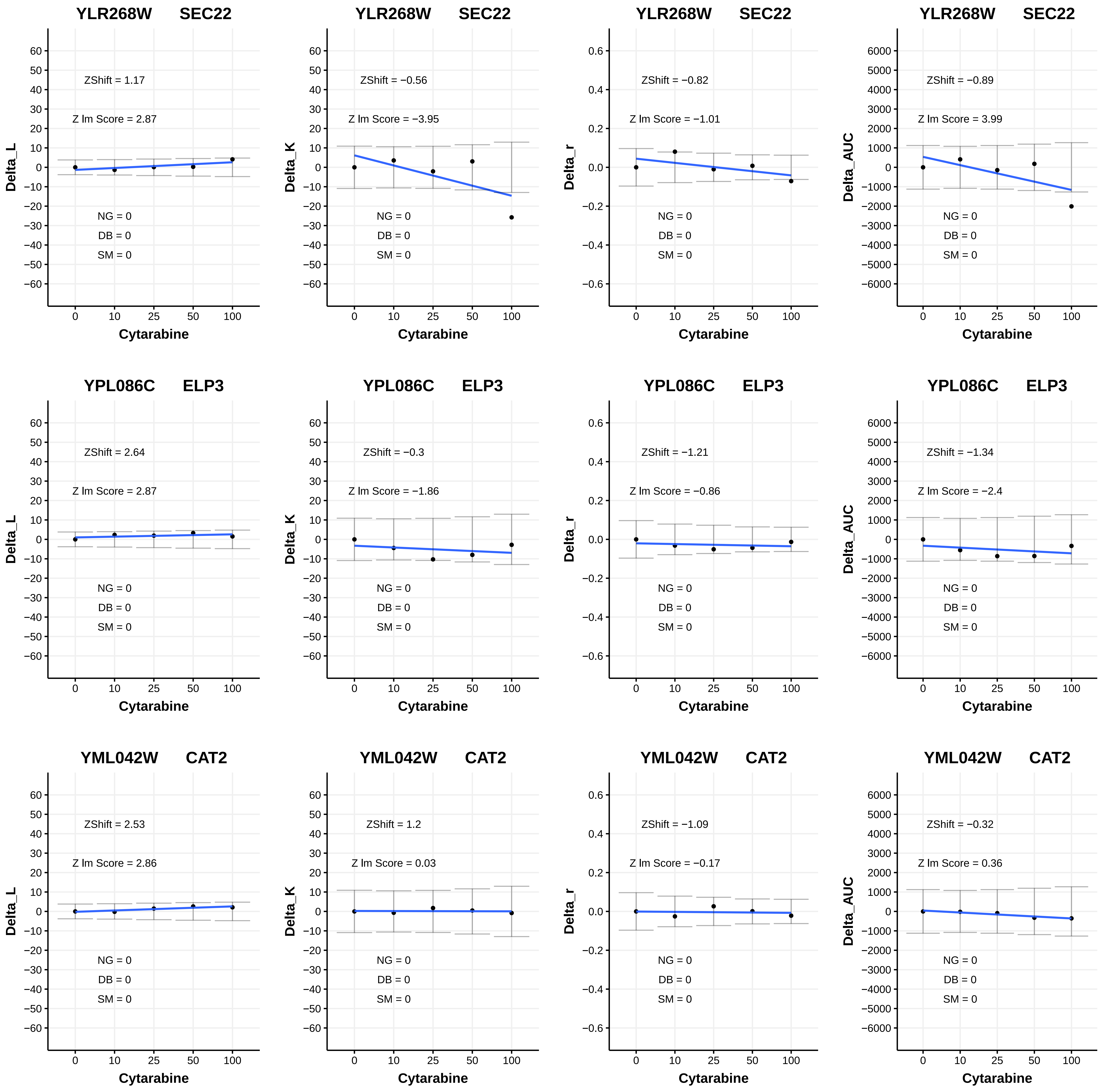

YLR268W SEC22
ZShift = 1.17
Z lm Score = 2.87
NG = 0
DB = 0
SM = 0
Delta_L
Cytarabine
YLR268W SEC22
ZShift = −0.56
Z lm Score = −3.95
NG = 0
DB = 0
SM = 0
Delta_K
Cytarabine
YLR268W SEC22
ZShift = −0.82
Z lm Score = −1.01
NG = 0
DB = 0
SM = 0
Delta_r
Cytarabine
YLR268W SEC22
ZShift = −0.89
Z lm Score = 3.99
NG = 0
DB = 0
SM = 0
Delta_AUC
Cytarabine
YPL086C ELP3
ZShift = 2.64
Z lm Score = 2.87
NG = 0
DB = 0
SM = 0
Delta_L
Cytarabine
YPL086C ELP3
ZShift = −0.3
Z lm Score = −1.86
NG = 0
DB = 0
SM = 0
Delta_K
Cytarabine
YPL086C ELP3
ZShift = −1.21
Z lm Score = −0.86
NG = 0
DB = 0
SM = 0
Delta_r
Cytarabine
YPL086C ELP3
ZShift = −1.34
Z lm Score = −2.4
NG = 0
DB = 0
SM = 0
Delta_AUC
Cytarabine
YML042W CAT2
ZShift = 2.53
Z lm Score = 2.86
NG = 0
DB = 0
SM = 0
Delta_L
Cytarabine
YML042W CAT2
ZShift = 1.2
Z lm Score = 0.03
NG = 0
DB = 0
SM = 0
Delta_K
Cytarabine
YML042W CAT2
ZShift = −1.09
Z lm Score = −0.17
NG = 0
DB = 0
SM = 0
Delta_r
Cytarabine
YML042W CAT2
ZShift = −0.32
Z lm Score = 0.36
NG = 0
DB = 0
SM = 0
Delta_AUC
Cytarabine

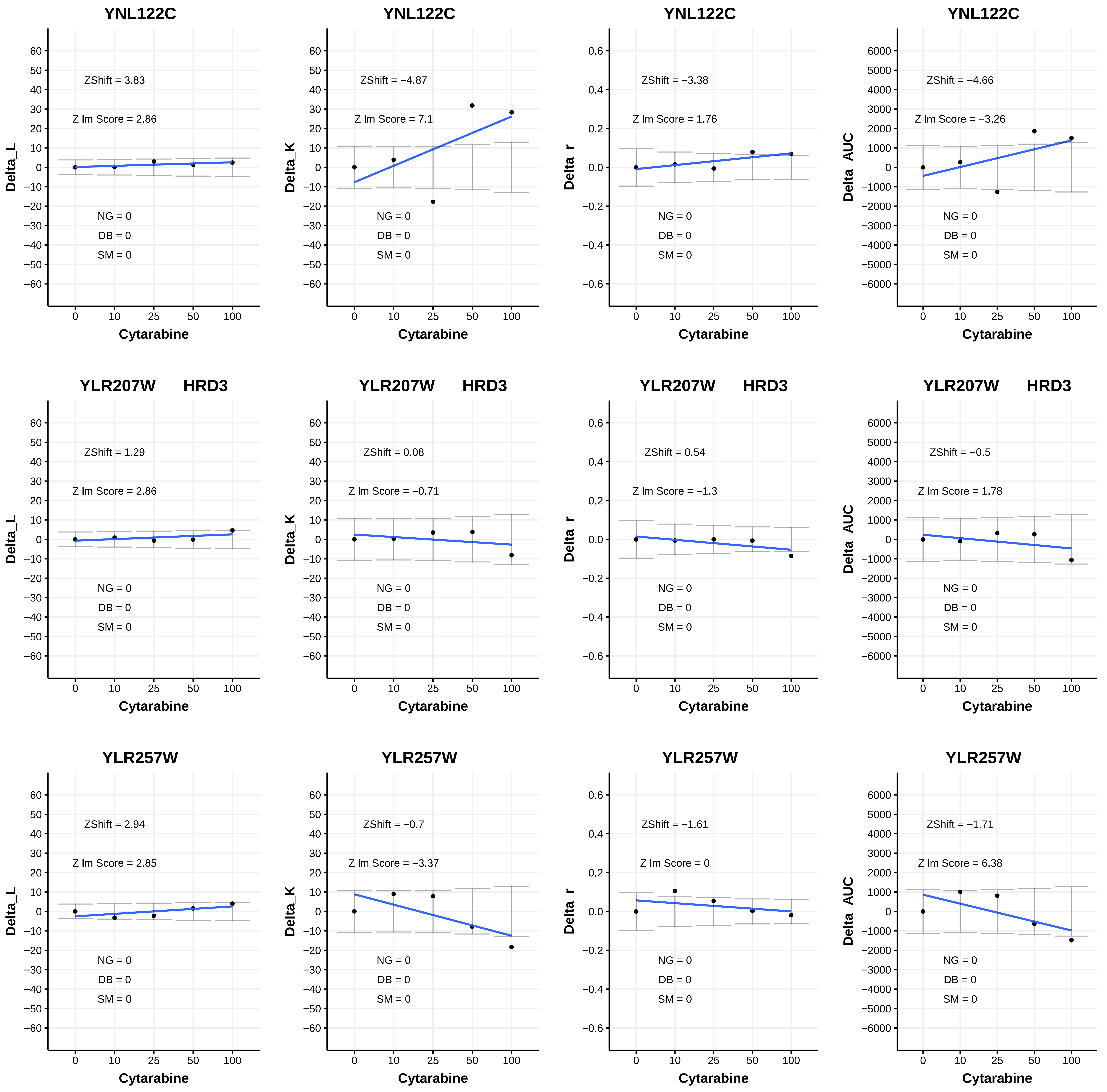
YNL122C
ZShift = 3.83
Z lm Score = 2.86
NG = 0
DB = 0
SM = 0
Delta_L
Cytarabine
YNL122C
ZShift = −4.87
Z lm Score = 7.1
NG = 0
DB = 0
SM = 0
Delta_K
Cytarabine
YNL122C
ZShift = −3.38
Z lm Score = 1.76
NG = 0
DB = 0
SM = 0
Delta_r
Cytarabine
YNL122C
ZShift = −4.66
Z lm Score = −3.26
NG = 0
DB = 0
SM = 0
Delta_AUC
Cytarabine
YLR207W HRD3
ZShift = 1.29
Z lm Score = 2.86
NG = 0
DB = 0
SM = 0
Delta_L
Cytarabine
YLR207W HRD3
ZShift = 0.08
Z lm Score = −0.71
NG = 0
DB = 0
SM = 0
Delta_K
Cytarabine
YLR207W HRD3
ZShift = 0.54
Z lm Score = −1.3
NG = 0
DB = 0
SM = 0
Delta_r
Cytarabine
YLR207W HRD3
ZShift = −0.5
Z lm Score = 1.78
NG = 0
DB = 0
SM = 0
Delta_AUC
Cytarabine
YLR257W
ZShift = 2.94
Z lm Score = 2.85
NG = 0
DB = 0
SM = 0
Delta_L
Cytarabine
YLR257W
ZShift = −0.7
Z lm Score = −3.37
NG = 0
DB = 0
SM = 0
Delta_K
Cytarabine
YLR257W
ZShift = −1.61
Z lm Score = 0
NG = 0
DB = 0
SM = 0
Delta_r
Cytarabine
YLR257W
ZShift = −1.71
Z lm Score = 6.38
NG = 0
DB = 0
SM = 0
Delta_AUC
Cytarabine

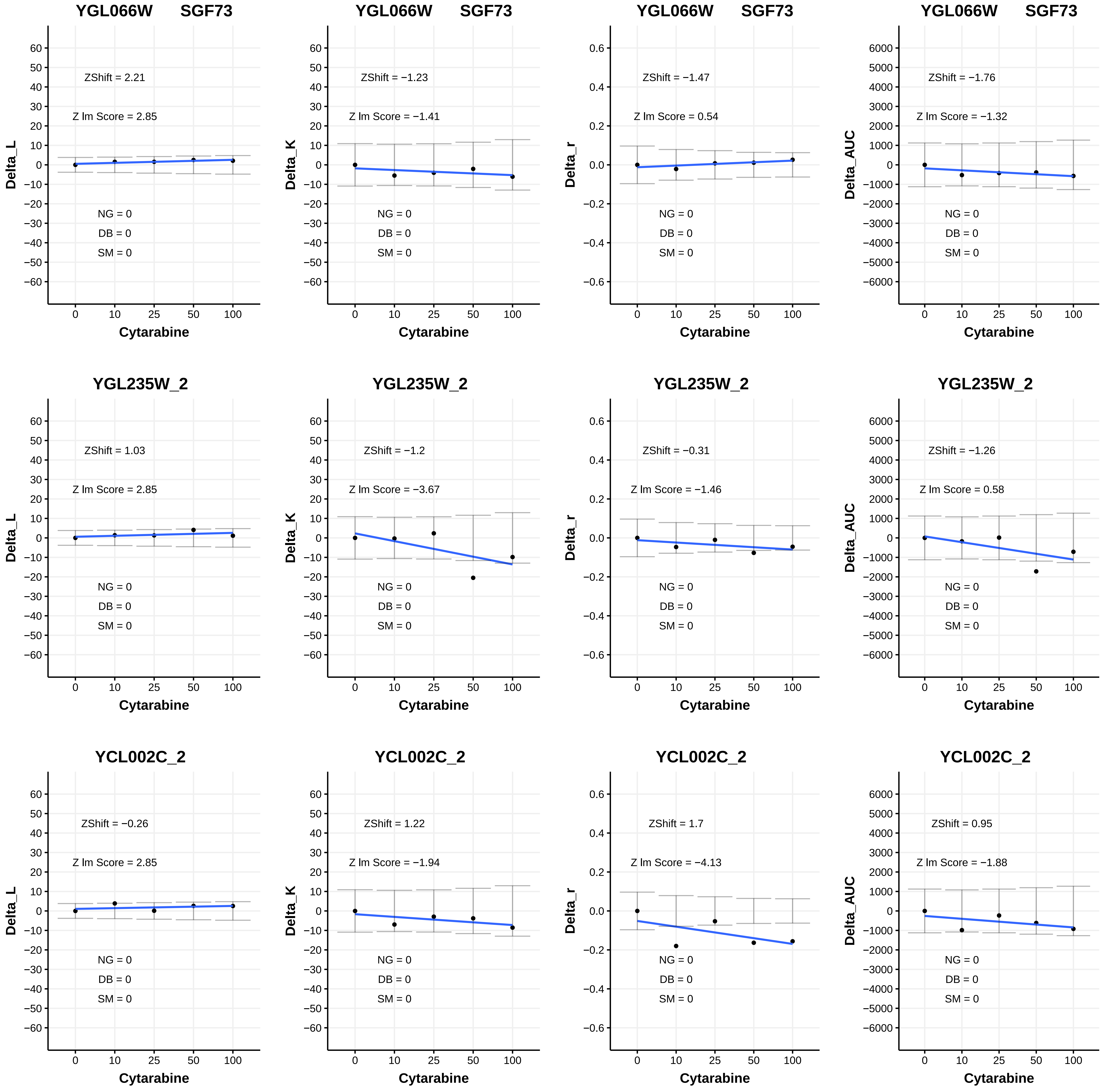
YGL066W SGF73
ZShift = 2.21
Z lm Score = 2.85
NG = 0
DB = 0
SM = 0
Delta_L
Cytarabine
YGL066W SGF73
ZShift = −1.23
Z lm Score = −1.41
NG = 0
DB = 0
SM = 0
Delta_K
Cytarabine
YGL066W SGF73
ZShift = −1.47
Z lm Score = 0.54
NG = 0
DB = 0
SM = 0
Delta_r
Cytarabine
YGL066W SGF73
ZShift = −1.76
Z lm Score = −1.32
NG = 0
DB = 0
SM = 0
Delta_AUC
Cytarabine
YGL235W_2
ZShift = 1.03
Z lm Score = 2.85
NG = 0
DB = 0
SM = 0
Delta_L
Cytarabine
YGL235W_2
ZShift = −1.2
Z lm Score = −3.67
NG = 0
DB = 0
SM = 0
Delta_K
Cytarabine
YGL235W_2
ZShift = −0.31
Z lm Score = −1.46
NG = 0
DB = 0
SM = 0
Delta_r
Cytarabine
YGL235W_2
ZShift = −1.26
Z lm Score = 0.58
NG = 0
DB = 0
SM = 0
Delta_AUC
Cytarabine
YCL002C_2
ZShift = −0.26
Z lm Score = 2.85
NG = 0
DB = 0
SM = 0
Delta_L
Cytarabine
YCL002C_2
ZShift = 1.22
Z lm Score = −1.94
NG = 0
DB = 0
SM = 0
Delta_K
Cytarabine
YCL002C_2
ZShift = 1.7
Z lm Score = −4.13
NG = 0
DB = 0
SM = 0
Delta_r
Cytarabine
YCL002C_2
ZShift = 0.95
Z lm Score = −1.88
NG = 0
DB = 0
SM = 0
Delta_AUC
Cytarabine

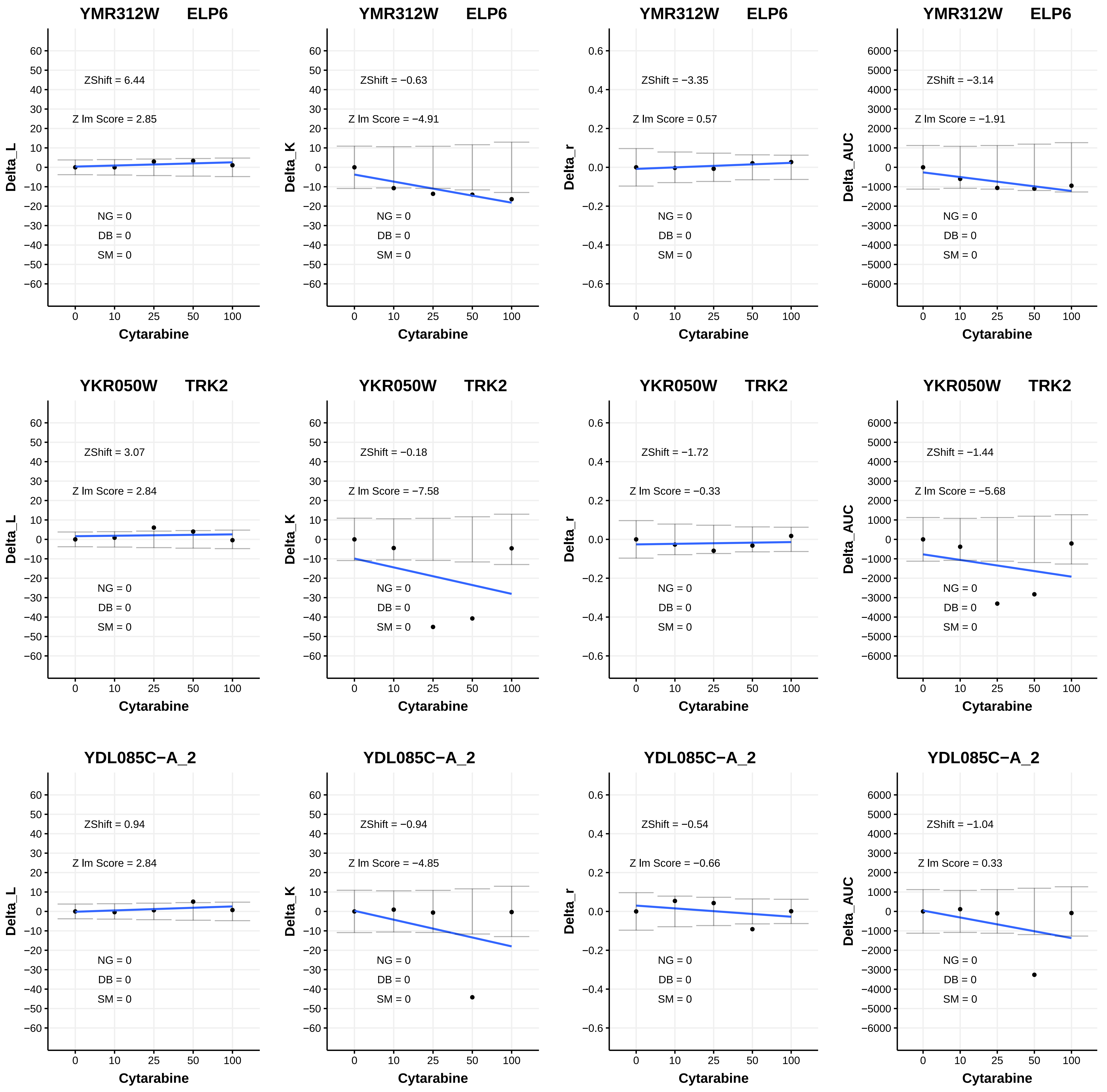

YMR312W ELP6
ZShift = 6.44
Z lm Score = 2.85
NG = 0
DB = 0
SM = 0
Delta_L
Cytarabine
YMR312W ELP6
ZShift = −0.63
Z lm Score = −4.91
NG = 0
DB = 0
SM = 0
Delta_K
Cytarabine
YMR312W ELP6
ZShift = −3.35
Z lm Score = 0.57
NG = 0
DB = 0
SM = 0
Delta_r
Cytarabine
YMR312W ELP6
ZShift = −3.14
Z lm Score = −1.91
NG = 0
DB = 0
SM = 0
Delta_AUC
Cytarabine
YKR050W TRK2
ZShift = 3.07
Z lm Score = 2.84
NG = 0
DB = 0
SM = 0
Delta_L
Cytarabine
YKR050W TRK2
ZShift = −0.18
Z lm Score = −7.58
NG = 0
DB = 0
SM = 0
Delta_K
Cytarabine
YKR050W TRK2
ZShift = −1.72
Z lm Score = −0.33
NG = 0
DB = 0
SM = 0
Delta_r
Cytarabine
YKR050W TRK2
ZShift = −1.44
Z lm Score = −5.68
NG = 0
DB = 0
SM = 0
Delta_AUC
Cytarabine
YDL085C−A_2
ZShift = 0.94
Z lm Score = 2.84
NG = 0
DB = 0
SM = 0
Delta_L
Cytarabine
YDL085C−A_2
ZShift = −0.94
Z lm Score = −4.85
NG = 0
DB = 0
SM = 0
Delta_K
Cytarabine
YDL085C−A_2
ZShift = −0.54
Z lm Score = −0.66
NG = 0
DB = 0
SM = 0
Delta_r
Cytarabine
YDL085C−A_2
ZShift = −1.04
Z lm Score = 0.33
NG = 0
DB = 0
SM = 0
Delta_AUC
Cytarabine

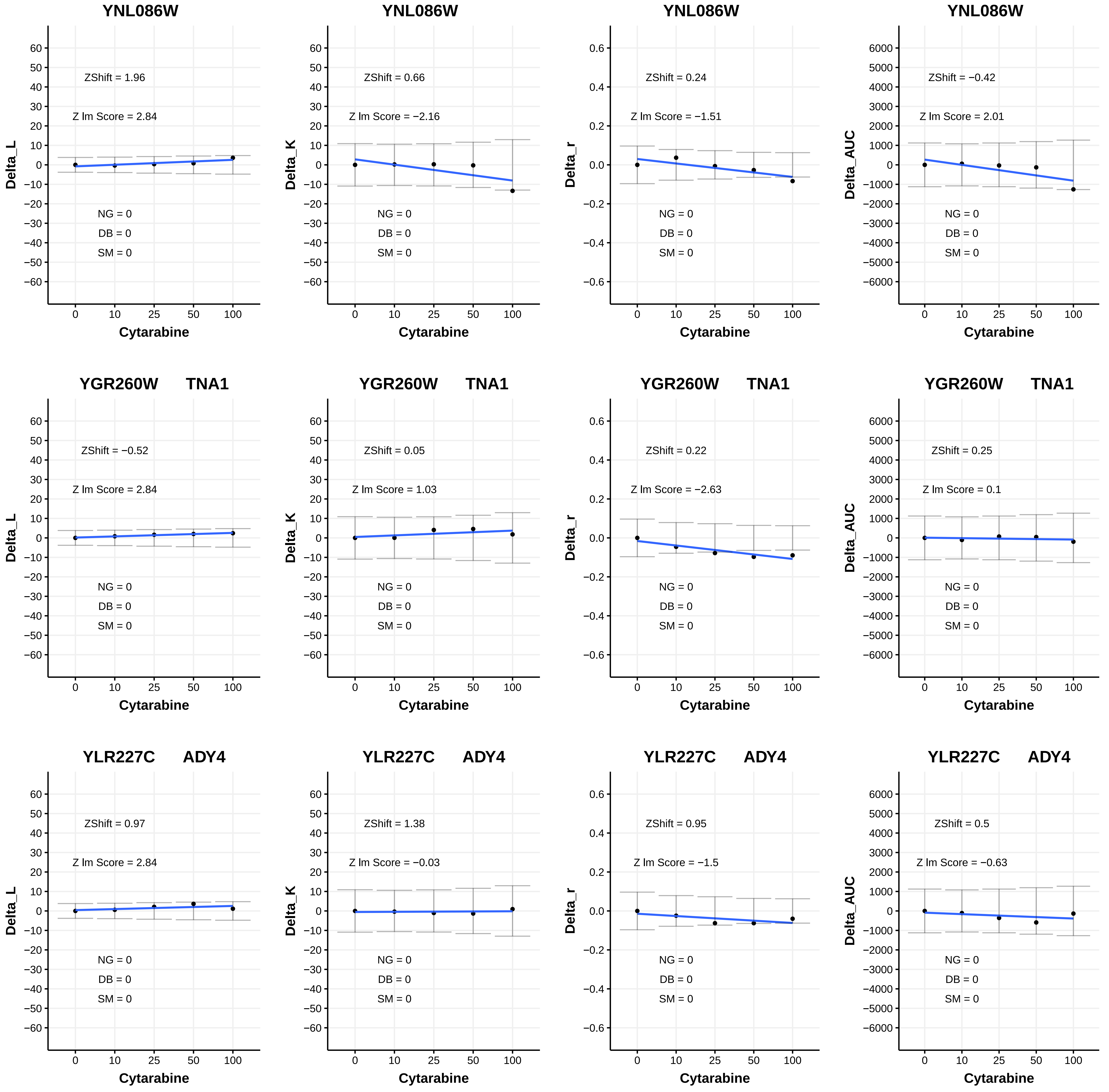

YNL086W
ZShift = 1.96
Z lm Score = 2.84
NG = 0
DB = 0
SM = 0
Delta_L
Cytarabine
YNL086W
ZShift = 0.66
Z lm Score = −2.16
NG = 0
DB = 0
SM = 0
Delta_K
Cytarabine
YNL086W
ZShift = 0.24
Z lm Score = −1.51
NG = 0
DB = 0
SM = 0
Delta_r
Cytarabine
YNL086W
ZShift = −0.42
Z lm Score = 2.01
NG = 0
DB = 0
SM = 0
Delta_AUC
Cytarabine
YGR260W TNA1
ZShift = −0.52
Z lm Score = 2.84
NG = 0
DB = 0
SM = 0
Delta_L
Cytarabine
YGR260W TNA1
ZShift = 0.05
Z lm Score = 1.03
NG = 0
DB = 0
SM = 0
Delta_K
Cytarabine
YGR260W TNA1
ZShift = 0.22
Z lm Score = −2.63
NG = 0
DB = 0
SM = 0
Delta_r
Cytarabine
YGR260W TNA1
ZShift = 0.25
Z lm Score = 0.1
NG = 0
DB = 0
SM = 0
Delta_AUC
Cytarabine
YLR227C ADY4
ZShift = 0.97
Z lm Score = 2.84
NG = 0
DB = 0
SM = 0
Delta_L
Cytarabine
YLR227C ADY4
ZShift = 1.38
Z lm Score = −0.03
NG = 0
DB = 0
SM = 0
Delta_K
Cytarabine
YLR227C ADY4
ZShift = 0.95
Z lm Score = −1.5
NG = 0
DB = 0
SM = 0
Delta_r
Cytarabine
YLR227C ADY4
ZShift = 0.5
Z lm Score = −0.63
NG = 0
DB = 0
SM = 0
Delta_AUC
Cytarabine

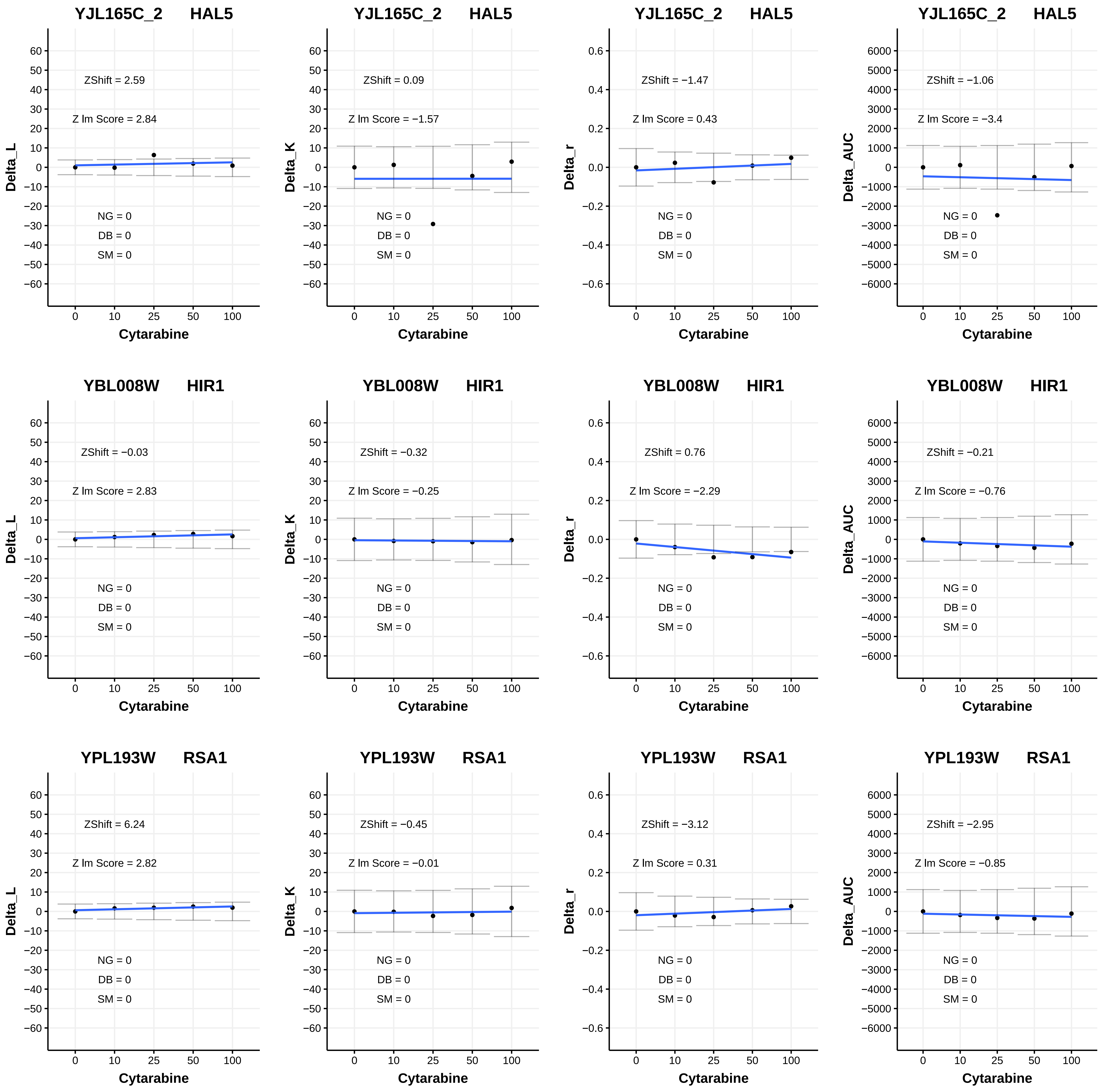

YJL165C_2 HAL5
ZShift = 2.59
Z lm Score = 2.84
NG = 0
DB = 0
SM = 0
Delta_L
Cytarabine
YJL165C_2 HAL5
ZShift = 0.09
Z lm Score = −1.57
NG = 0
DB = 0
SM = 0
Delta_K
Cytarabine
YJL165C_2 HAL5
ZShift = −1.47
Z lm Score = 0.43
NG = 0
DB = 0
SM = 0
Delta_r
Cytarabine
YJL165C_2 HAL5
ZShift = −1.06
Z lm Score = −3.4
NG = 0
DB = 0
SM = 0
Delta_AUC
Cytarabine
YBL008W HIR1
ZShift = −0.03
Z lm Score = 2.83
NG = 0
DB = 0
SM = 0
Delta_L
Cytarabine
YBL008W HIR1
ZShift = −0.32
Z lm Score = −0.25
NG = 0
DB = 0
SM = 0
Delta_K
Cytarabine
YBL008W HIR1
ZShift = 0.76
Z lm Score = −2.29
NG = 0
DB = 0
SM = 0
Delta_r
Cytarabine
YBL008W HIR1
ZShift = −0.21
Z lm Score = −0.76
NG = 0
DB = 0
SM = 0
Delta_AUC
Cytarabine
YPL193W RSA1
ZShift = 6.24
Z lm Score = 2.82
NG = 0
DB = 0
SM = 0
Delta_L
Cytarabine
YPL193W RSA1
ZShift = −0.45
Z lm Score = −0.01
NG = 0
DB = 0
SM = 0
Delta_K
Cytarabine
YPL193W RSA1
ZShift = −3.12
Z lm Score = 0.31
NG = 0
DB = 0
SM = 0
Delta_r
Cytarabine
YPL193W RSA1
ZShift = −2.95
Z lm Score = −0.85
NG = 0
DB = 0
SM = 0
Delta_AUC
Cytarabine

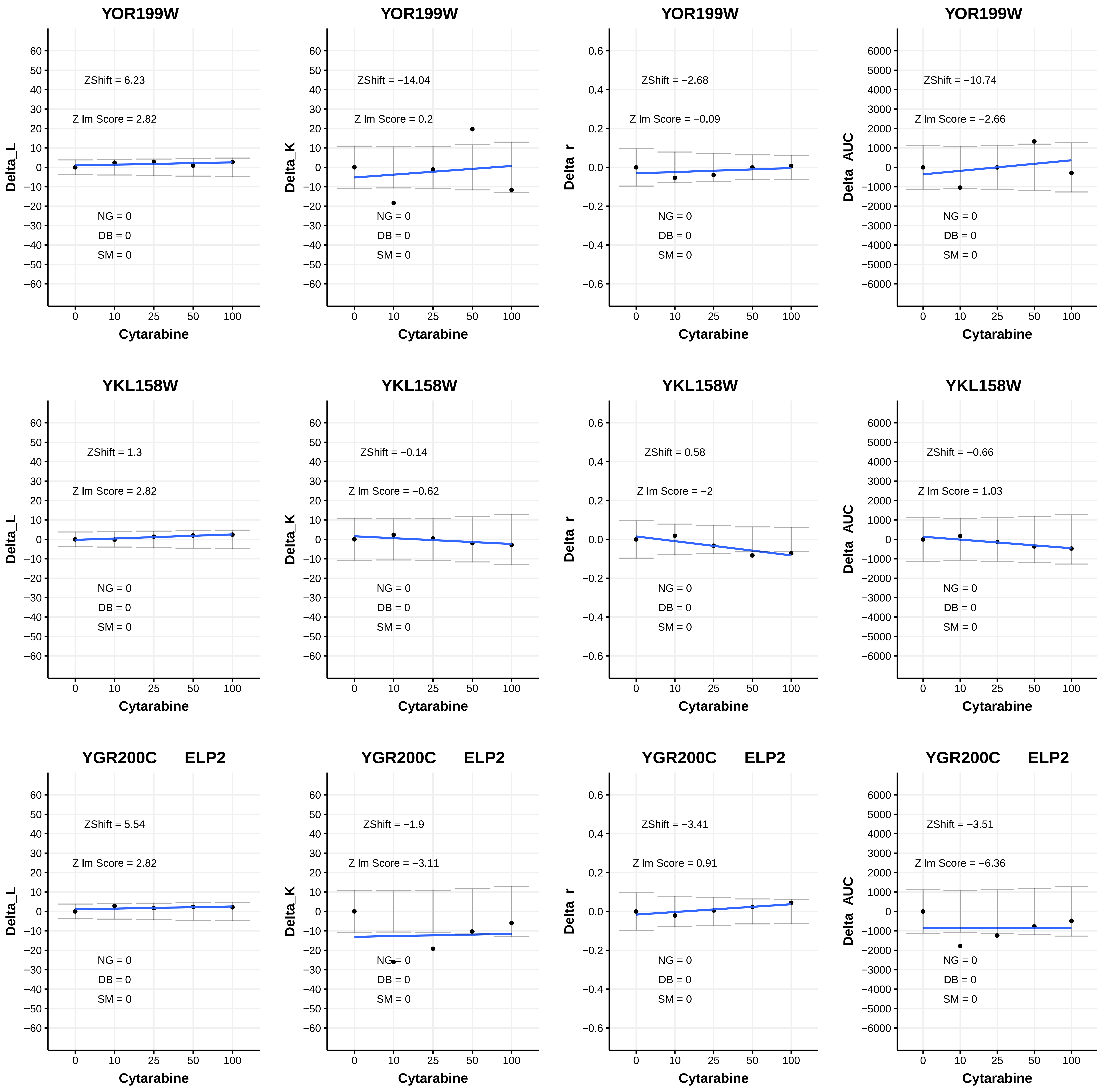

YOR199W
ZShift = 6.23
Z lm Score = 2.82
NG = 0
DB = 0
SM = 0
Delta_L
Cytarabine
YOR199W
ZShift = −14.04
Z lm Score = 0.2
NG = 0
DB = 0
SM = 0
Delta_K
Cytarabine
YOR199W
ZShift = −2.68
Z lm Score = −0.09
NG = 0
DB = 0
SM = 0
Delta_r
Cytarabine
YOR199W
ZShift = −10.74
Z lm Score = −2.66
NG = 0
DB = 0
SM = 0
Delta_AUC
Cytarabine
YKL158W
ZShift = 1.3
Z lm Score = 2.82
NG = 0
DB = 0
SM = 0
Delta_L
Cytarabine
YKL158W
ZShift = −0.14
Z lm Score = −0.62
NG = 0
DB = 0
SM = 0
Delta_K
Cytarabine
YKL158W
ZShift = 0.58
Z lm Score = −2
NG = 0
DB = 0
SM = 0
Delta_r
Cytarabine
YKL158W
ZShift = −0.66
Z lm Score = 1.03
NG = 0
DB = 0
SM = 0
Delta_AUC
Cytarabine
YGR200C ELP2
ZShift = 5.54
Z lm Score = 2.82
NG = 0
DB = 0
SM = 0
Delta_L
Cytarabine
YGR200C ELP2
ZShift = −1.9
Z lm Score = −3.11
NG = 0
DB = 0
SM = 0
Delta_K
Cytarabine
YGR200C ELP2
ZShift = −3.41
Z lm Score = 0.91
NG = 0
DB = 0
SM = 0
Delta_r
Cytarabine
YGR200C ELP2
ZShift = −3.51
Z lm Score = −6.36
NG = 0
DB = 0
SM = 0
Delta_AUC
Cytarabine

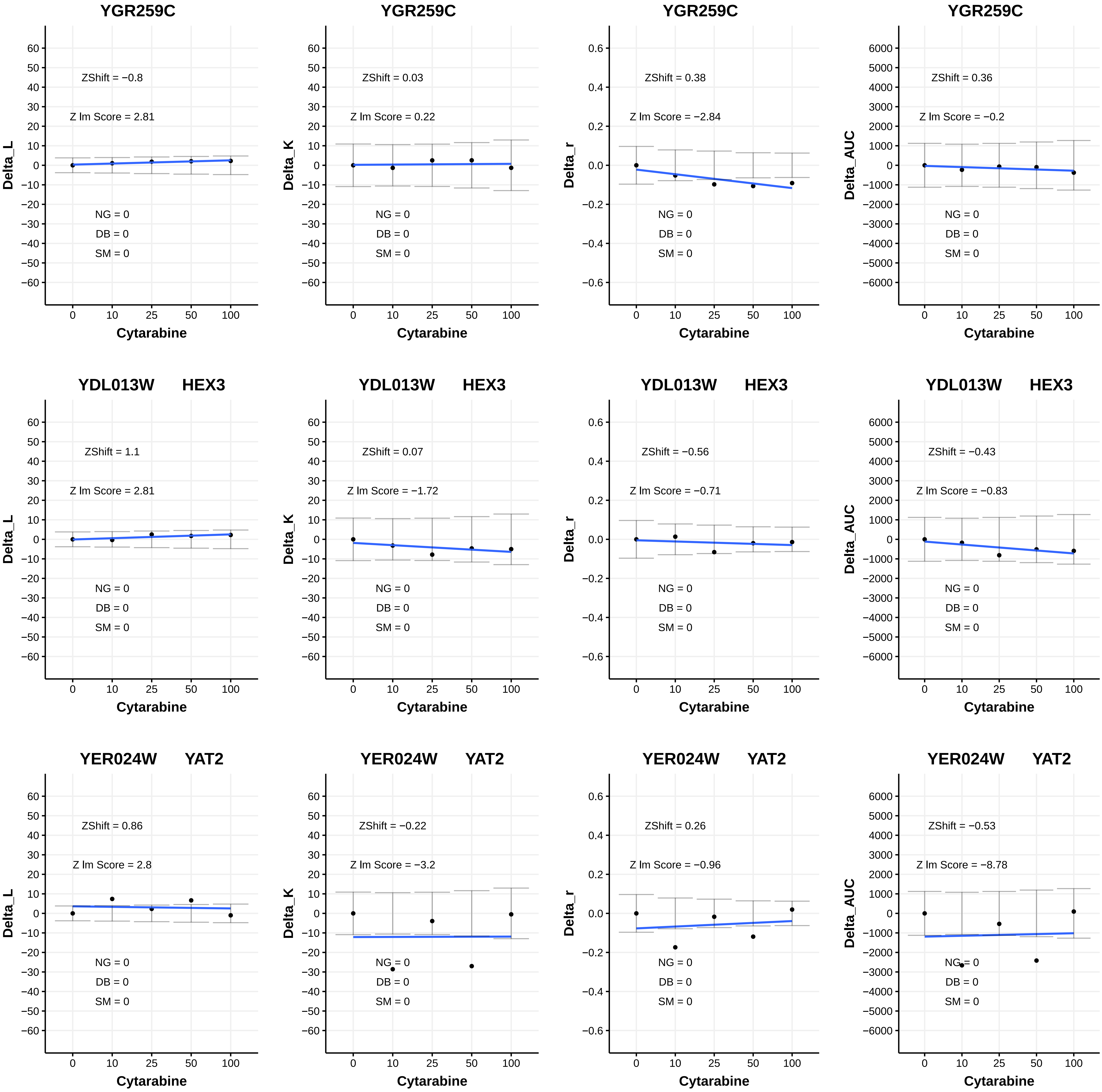
YGR259C
Delta_L
ZShift = −0.8
Z lm Score = 2.81
NG = 0
DB = 0
SM = 0
Cytarabine
YGR259C
Delta_K
ZShift = 0.03
Z lm Score = 0.22
NG = 0
DB = 0
SM = 0
Cytarabine
YGR259C
Delta_r
ZShift = 0.38
Z lm Score = −2.84
NG = 0
DB = 0
SM = 0
Cytarabine
YGR259C
Delta_AUC
ZShift = 0.36
Z lm Score = −0.2
NG = 0
DB = 0
SM = 0
Cytarabine
YDL013W HEX3
Delta_L
ZShift = 1.1
Z lm Score = 2.81
NG = 0
DB = 0
SM = 0
Cytarabine
YDL013W HEX3
Delta_K
ZShift = 0.07
Z lm Score = −1.72
NG = 0
DB = 0
SM = 0
Cytarabine
YDL013W HEX3
Delta_r
ZShift = −0.56
Z lm Score = −0.71
NG = 0
DB = 0
SM = 0
Cytarabine
YDL013W HEX3
Delta_AUC
ZShift = −0.43
Z lm Score = −0.83
NG = 0
DB = 0
SM = 0
Cytarabine
YER024W YAT2
Delta_L
ZShift = 0.86
Z lm Score = 2.8
NG = 0
DB = 0
SM = 0
Cytarabine
YER024W YAT2
Delta_K
ZShift = −0.22
Z lm Score = −3.2
NG = 0
DB = 0
SM = 0
Cytarabine
YER024W YAT2
Delta_r
ZShift = 0.26
Z lm Score = −0.96
NG = 0
DB = 0
SM = 0
Cytarabine
YER024W YAT2
Delta_AUC
ZShift = −0.53
Z lm Score = −8.78
NG = 0
DB = 0
SM = 0
Cytarabine

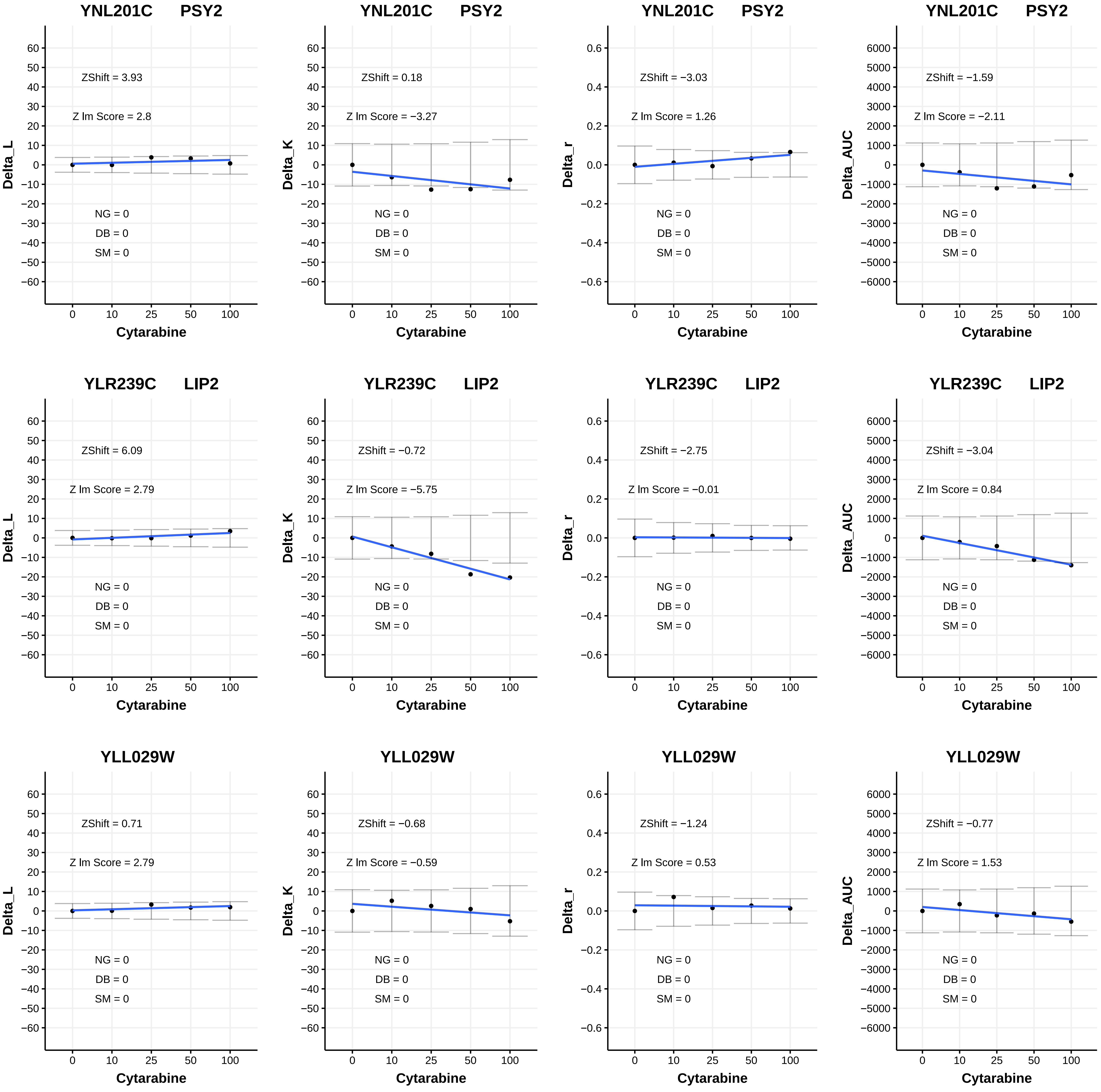

YNL201C PSY2
ZShift = 3.93
Z lm Score = 2.8
NG = 0
DB = 0
SM = 0
Delta_L
Cytarabine
YNL201C PSY2
ZShift = 0.18
Z lm Score = −3.27
NG = 0
DB = 0
SM = 0
Delta_K
Cytarabine
YNL201C PSY2
ZShift = −3.03
Z lm Score = 1.26
NG = 0
DB = 0
SM = 0
Delta_r
Cytarabine
YNL201C PSY2
ZShift = −1.59
Z lm Score = −2.11
NG = 0
DB = 0
SM = 0
Delta_AUC
Cytarabine
YLR239C LIP2
ZShift = 6.09
Z lm Score = 2.79
NG = 0
DB = 0
SM = 0
Delta_L
Cytarabine
YLR239C LIP2
ZShift = −0.72
Z lm Score = −5.75
NG = 0
DB = 0
SM = 0
Delta_K
Cytarabine
YLR239C LIP2
ZShift = −2.75
Z lm Score = −0.01
NG = 0
DB = 0
SM = 0
Delta_r
Cytarabine
YLR239C LIP2
ZShift = −3.04
Z lm Score = 0.84
NG = 0
DB = 0
SM = 0
Delta_AUC
Cytarabine
YLL029W
ZShift = 0.71
Z lm Score = 2.79
NG = 0
DB = 0
SM = 0
Delta_L
Cytarabine
YLL029W
ZShift = −0.68
Z lm Score = −0.59
NG = 0
DB = 0
SM = 0
Delta_K
Cytarabine
YLL029W
ZShift = −1.24
Z lm Score = 0.53
NG = 0
DB = 0
SM = 0
Delta_r
Cytarabine
YLL029W
ZShift = −0.77
Z lm Score = 1.53
NG = 0
DB = 0
SM = 0
Delta_AUC
Cytarabine

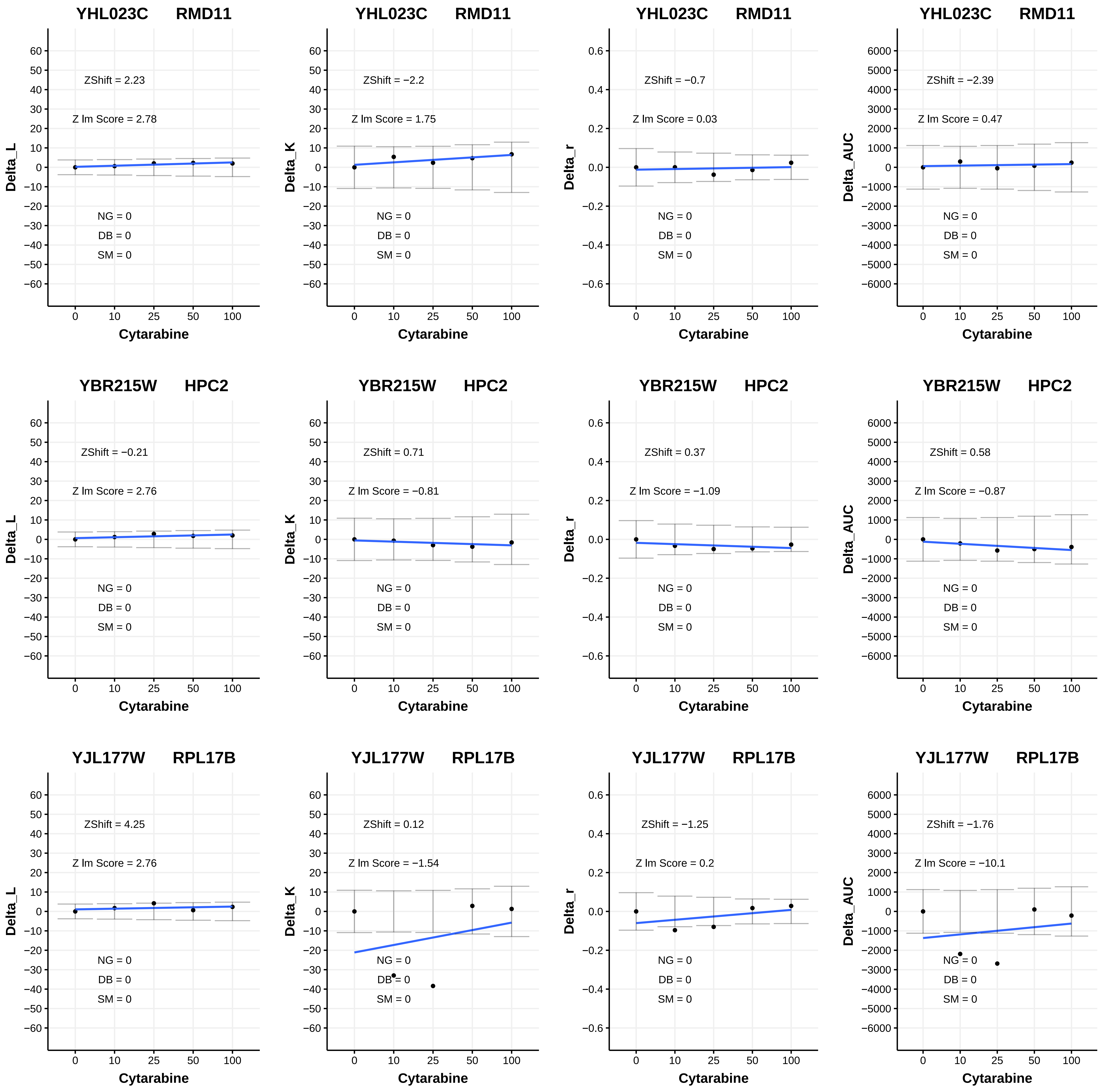
YHL023C RMD11
ZShift = 2.23
Z lm Score = 2.78
NG = 0
DB = 0
SM = 0
Delta_L
Cytarabine
YHL023C RMD11
ZShift = −2.2
Z lm Score = 1.75
NG = 0
DB = 0
SM = 0
Delta_K
Cytarabine
YHL023C RMD11
ZShift = −0.7
Z lm Score = 0.03
NG = 0
DB = 0
SM = 0
Delta_r
Cytarabine
YHL023C RMD11
ZShift = −2.39
Z lm Score = 0.47
NG = 0
DB = 0
SM = 0
Delta_AUC
Cytarabine
YBR215W HPC2
ZShift = −0.21
Z lm Score = 2.76
NG = 0
DB = 0
SM = 0
Delta_L
Cytarabine
YBR215W HPC2
ZShift = 0.71
Z lm Score = −0.81
NG = 0
DB = 0
SM = 0
Delta_K
Cytarabine
YBR215W HPC2
ZShift = 0.37
Z lm Score = −1.09
NG = 0
DB = 0
SM = 0
Delta_r
Cytarabine
YBR215W HPC2
ZShift = 0.58
Z lm Score = −0.87
NG = 0
DB = 0
SM = 0
Delta_AUC
Cytarabine
YJL177W RPL17B
ZShift = 4.25
Z lm Score = 2.76
NG = 0
DB = 0
SM = 0
Delta_L
Cytarabine
YJL177W RPL17B
ZShift = 0.12
Z lm Score = −1.54
NG = 0
DB = 0
SM = 0
Delta_K
Cytarabine
YJL177W RPL17B
ZShift = −1.25
Z lm Score = 0.2
NG = 0
DB = 0
SM = 0
Delta_r
Cytarabine
YJL177W RPL17B
ZShift = −1.76
Z lm Score = −10.1
NG = 0
DB = 0
SM = 0
Delta_AUC
Cytarabine

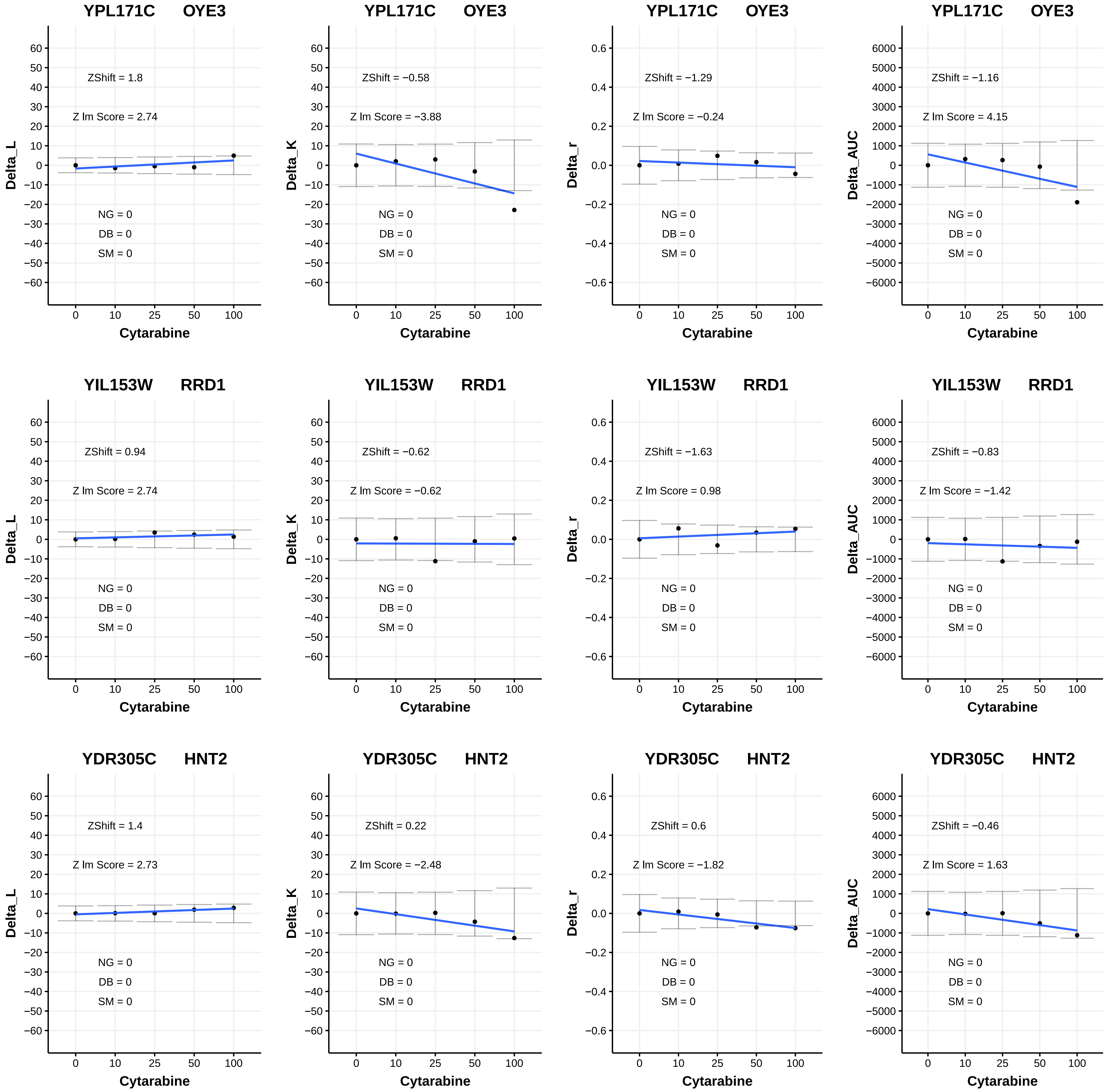
YPL171C OYE3
ZShift = 1.8
Z lm Score = 2.74
NG = 0
DB = 0
SM = 0
Delta_L
Cytarabine
YPL171C OYE3
ZShift = −0.58
Z lm Score = −3.88
NG = 0
DB = 0
SM = 0
Delta_K
Cytarabine
YPL171C OYE3
ZShift = −1.29
Z lm Score = −0.24
NG = 0
DB = 0
SM = 0
Delta_r
Cytarabine
YPL171C OYE3
ZShift = −1.16
Z lm Score = 4.15
NG = 0
DB = 0
SM = 0
Delta_AUC
Cytarabine
YIL153W RRD1
ZShift = 0.94
Z lm Score = 2.74
NG = 0
DB = 0
SM = 0
Delta_L
Cytarabine
YIL153W RRD1
ZShift = −0.62
Z lm Score = −0.62
NG = 0
DB = 0
SM = 0
Delta_K
Cytarabine
YIL153W RRD1
ZShift = −1.63
Z lm Score = 0.98
NG = 0
DB = 0
SM = 0
Delta_r
Cytarabine
YIL153W RRD1
ZShift = −0.83
Z lm Score = −1.42
NG = 0
DB = 0
SM = 0
Delta_AUC
Cytarabine
YDR305C HNT2
ZShift = 1.4
Z lm Score = 2.73
NG = 0
DB = 0
SM = 0
Delta_L
Cytarabine
YDR305C HNT2
ZShift = 0.22
Z lm Score = −2.48
NG = 0
DB = 0
SM = 0
Delta_K
Cytarabine
YDR305C HNT2
ZShift = 0.6
Z lm Score = −1.82
NG = 0
DB = 0
SM = 0
Delta_r
Cytarabine
YDR305C HNT2
ZShift = −0.46
Z lm Score = 1.63
NG = 0
DB = 0
SM = 0
Delta_AUC
Cytarabine

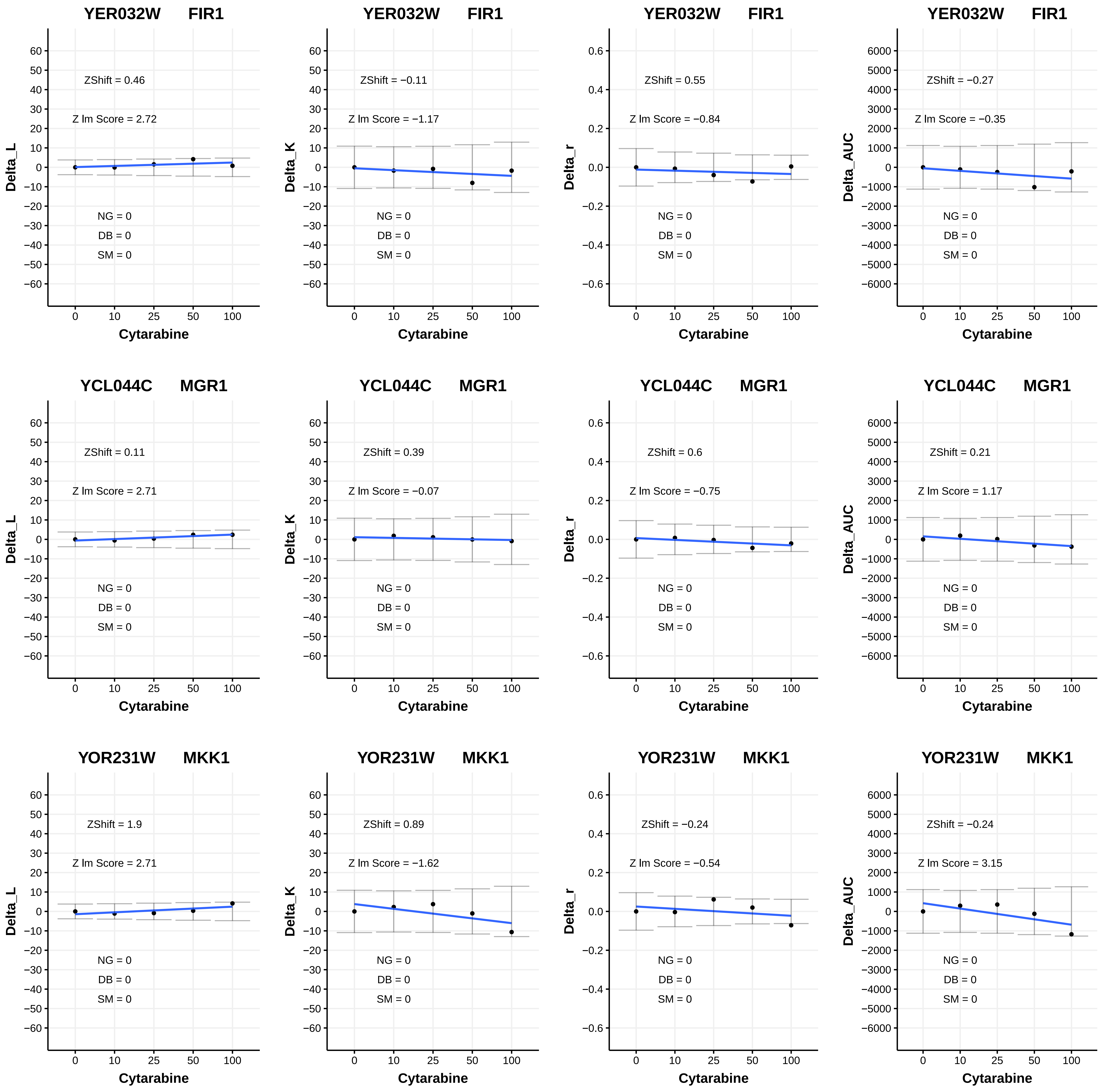
YER032W FIR1
ZShift = 0.46
Z lm Score = 2.72
NG = 0
DB = 0
SM = 0
Delta_L
Cytarabine
YER032W FIR1
ZShift = −0.11
Z lm Score = −1.17
NG = 0
DB = 0
SM = 0
Delta_K
Cytarabine
YER032W FIR1
ZShift = 0.55
Z lm Score = −0.84
NG = 0
DB = 0
SM = 0
Delta_r
Cytarabine
YER032W FIR1
ZShift = −0.27
Z lm Score = −0.35
NG = 0
DB = 0
SM = 0
Delta_AUC
Cytarabine
YCL044C MGR1
ZShift = 0.11
Z lm Score = 2.71
NG = 0
DB = 0
SM = 0
Delta_L
Cytarabine
YCL044C MGR1
ZShift = 0.39
Z lm Score = −0.07
NG = 0
DB = 0
SM = 0
Delta_K
Cytarabine
YCL044C MGR1
ZShift = 0.6
Z lm Score = −0.75
NG = 0
DB = 0
SM = 0
Delta_r
Cytarabine
YCL044C MGR1
ZShift = 0.21
Z lm Score = 1.17
NG = 0
DB = 0
SM = 0
Delta_AUC
Cytarabine
YOR231W MKK1
ZShift = 1.9
Z lm Score = 2.71
NG = 0
DB = 0
SM = 0
Delta_L
Cytarabine
YOR231W MKK1
ZShift = 0.89
Z lm Score = −1.62
NG = 0
DB = 0
SM = 0
Delta_K
Cytarabine
YOR231W MKK1
ZShift = −0.24
Z lm Score = −0.54
NG = 0
DB = 0
SM = 0
Delta_r
Cytarabine
YOR231W MKK1
ZShift = −0.24
Z lm Score = 3.15
NG = 0
DB = 0
SM = 0
Delta_AUC
Cytarabine

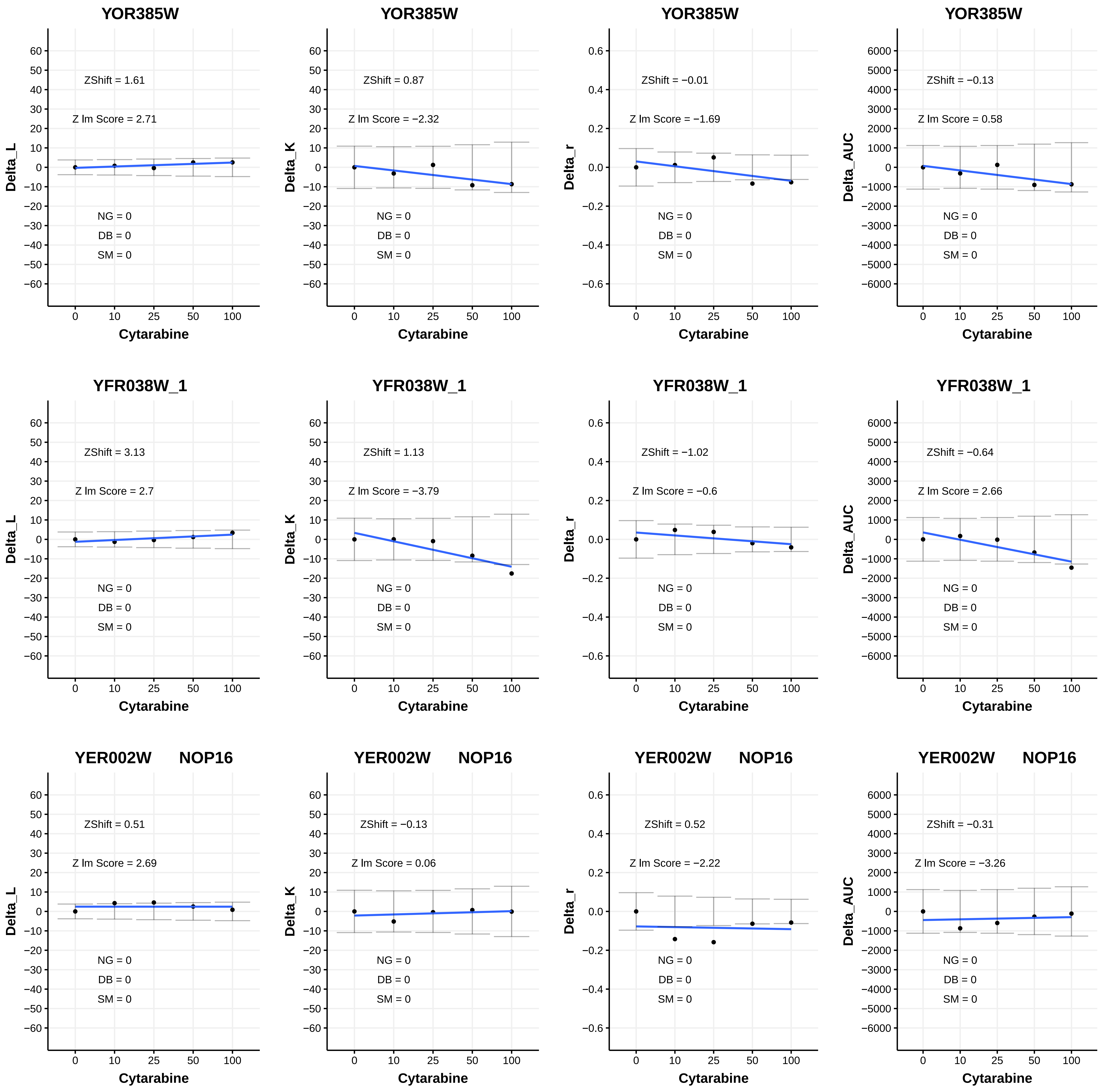

YOR385W
ZShift = 1.61
Z lm Score = 2.71
NG = 0
DB = 0
SM = 0
Delta_L
Cytarabine
YOR385W
ZShift = 0.87
Z lm Score = −2.32
NG = 0
DB = 0
SM = 0
Delta_K
Cytarabine
YOR385W
ZShift = −0.01
Z lm Score = −1.69
NG = 0
DB = 0
SM = 0
Delta_r
Cytarabine
YOR385W
ZShift = −0.13
Z lm Score = 0.58
NG = 0
DB = 0
SM = 0
Delta_AUC
Cytarabine
YFR038W_1
ZShift = 3.13
Z lm Score = 2.7
NG = 0
DB = 0
SM = 0
Delta_L
Cytarabine
YFR038W_1
ZShift = 1.13
Z lm Score = −3.79
NG = 0
DB = 0
SM = 0
Delta_K
Cytarabine
YFR038W_1
ZShift = −1.02
Z lm Score = −0.6
NG = 0
DB = 0
SM = 0
Delta_r
Cytarabine
YFR038W_1
ZShift = −0.64
Z lm Score = 2.66
NG = 0
DB = 0
SM = 0
Delta_AUC
Cytarabine
YER002W NOP16
ZShift = 0.51
Z lm Score = 2.69
NG = 0
DB = 0
SM = 0
Delta_L
Cytarabine
YER002W NOP16
ZShift = −0.13
Z lm Score = 0.06
NG = 0
DB = 0
SM = 0
Delta_K
Cytarabine
YER002W NOP16
ZShift = 0.52
Z lm Score = −2.22
NG = 0
DB = 0
SM = 0
Delta_r
Cytarabine
YER002W NOP16
ZShift = −0.31
Z lm Score = −3.26
NG = 0
DB = 0
SM = 0
Delta_AUC
Cytarabine

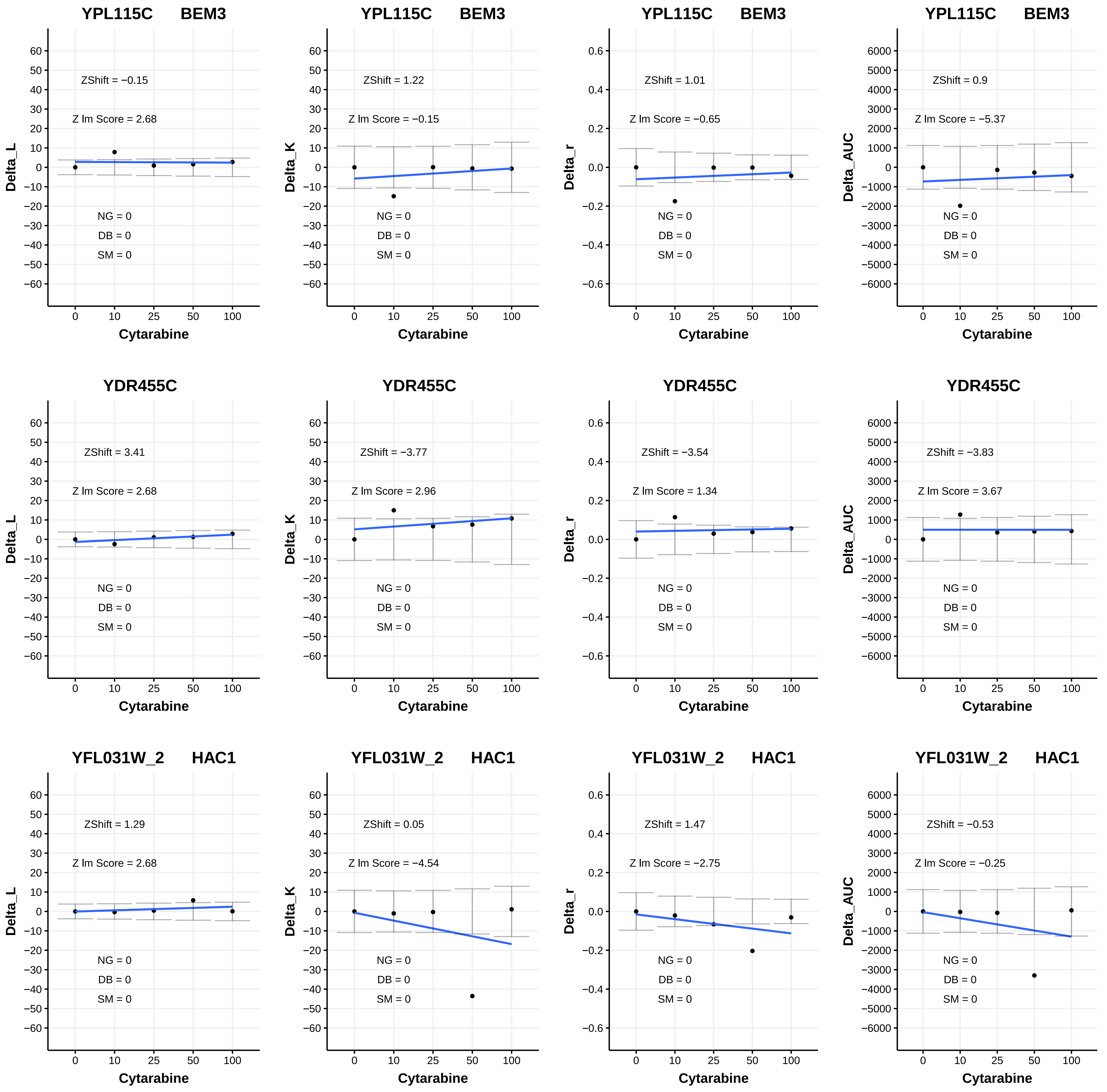

YPL115C BEM3
ZShift = −0.15
Z lm Score = 2.68
NG = 0
DB = 0
SM = 0
Delta_L
Cytarabine
YPL115C BEM3
ZShift = 1.22
Z lm Score = −0.15
NG = 0
DB = 0
SM = 0
Delta_K
Cytarabine
YPL115C BEM3
ZShift = 1.01
Z lm Score = −0.65
NG = 0
DB = 0
SM = 0
Delta_r
Cytarabine
YPL115C BEM3
ZShift = 0.9
Z lm Score = −5.37
NG = 0
DB = 0
SM = 0
Delta_AUC
Cytarabine
YDR455C
ZShift = 3.41
Z lm Score = 2.68
NG = 0
DB = 0
SM = 0
Delta_L
Cytarabine
YDR455C
ZShift = −3.77
Z lm Score = 2.96
NG = 0
DB = 0
SM = 0
Delta_K
Cytarabine
YDR455C
ZShift = −3.54
Z lm Score = 1.34
NG = 0
DB = 0
SM = 0
Delta_r
Cytarabine
YDR455C
ZShift = −3.83
Z lm Score = 3.67
NG = 0
DB = 0
SM = 0
Delta_AUC
Cytarabine
YFL031W_2 HAC1
ZShift = 1.29
Z lm Score = 2.68
NG = 0
DB = 0
SM = 0
Delta_L
Cytarabine
YFL031W_2 HAC1
ZShift = 0.05
Z lm Score = −4.54
NG = 0
DB = 0
SM = 0
Delta_K
Cytarabine
YFL031W_2 HAC1
ZShift = 1.47
Z lm Score = −2.75
NG = 0
DB = 0
SM = 0
Delta_r
Cytarabine
YFL031W_2 HAC1
ZShift = −0.53
Z lm Score = −0.25
NG = 0
DB = 0
SM = 0
Delta_AUC
Cytarabine

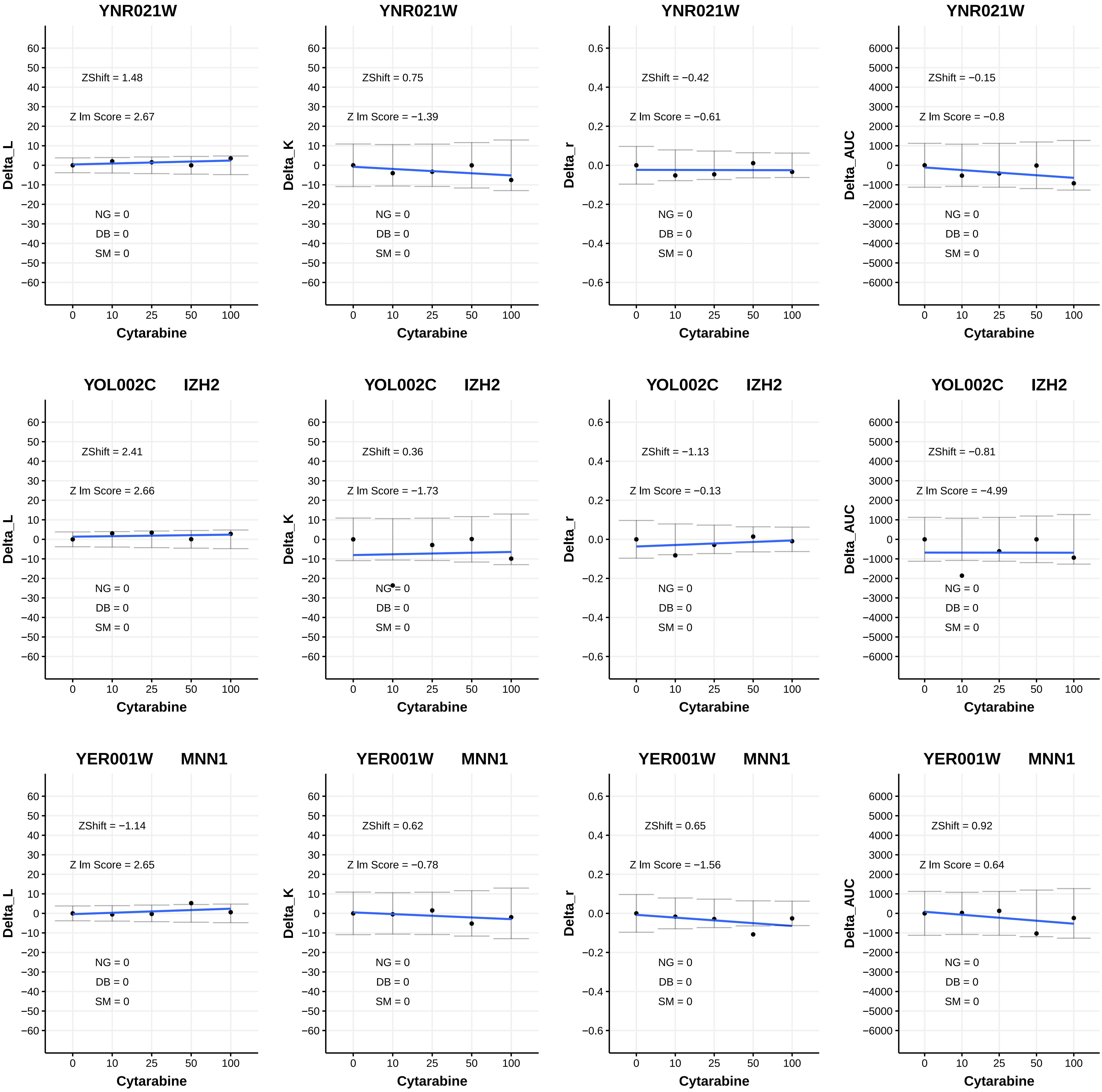
YNR021W
ZShift = 1.48
Z lm Score = 2.67
NG = 0
DB = 0
SM = 0
Delta_L
Cytarabine
YNR021W
ZShift = 0.75
Z lm Score = −1.39
NG = 0
DB = 0
SM = 0
Delta_K
Cytarabine
YNR021W
ZShift = −0.42
Z lm Score = −0.61
NG = 0
DB = 0
SM = 0
Delta_r
Cytarabine
YNR021W
ZShift = −0.15
Z lm Score = −0.8
NG = 0
DB = 0
SM = 0
Delta_AUC
Cytarabine
YOL002C IZH2
ZShift = 2.41
Z lm Score = 2.66
NG = 0
DB = 0
SM = 0
Delta_L
Cytarabine
YOL002C IZH2
ZShift = 0.36
Z lm Score = −1.73
NG = 0
DB = 0
SM = 0
Delta_K
Cytarabine
YOL002C IZH2
ZShift = −1.13
Z lm Score = −0.13
NG = 0
DB = 0
SM = 0
Delta_r
Cytarabine
YOL002C IZH2
ZShift = −0.81
Z lm Score = −4.99
NG = 0
DB = 0
SM = 0
Delta_AUC
Cytarabine
YER001W MNN1
ZShift = −1.14
Z lm Score = 2.65
NG = 0
DB = 0
SM = 0
Delta_L
Cytarabine
YER001W MNN1
ZShift = 0.62
Z lm Score = −0.78
NG = 0
DB = 0
SM = 0
Delta_K
Cytarabine
YER001W MNN1
ZShift = 0.65
Z lm Score = −1.56
NG = 0
DB = 0
SM = 0
Delta_r
Cytarabine
YER001W MNN1
ZShift = 0.92
Z lm Score = 0.64
NG = 0
DB = 0
SM = 0
Delta_AUC
Cytarabine

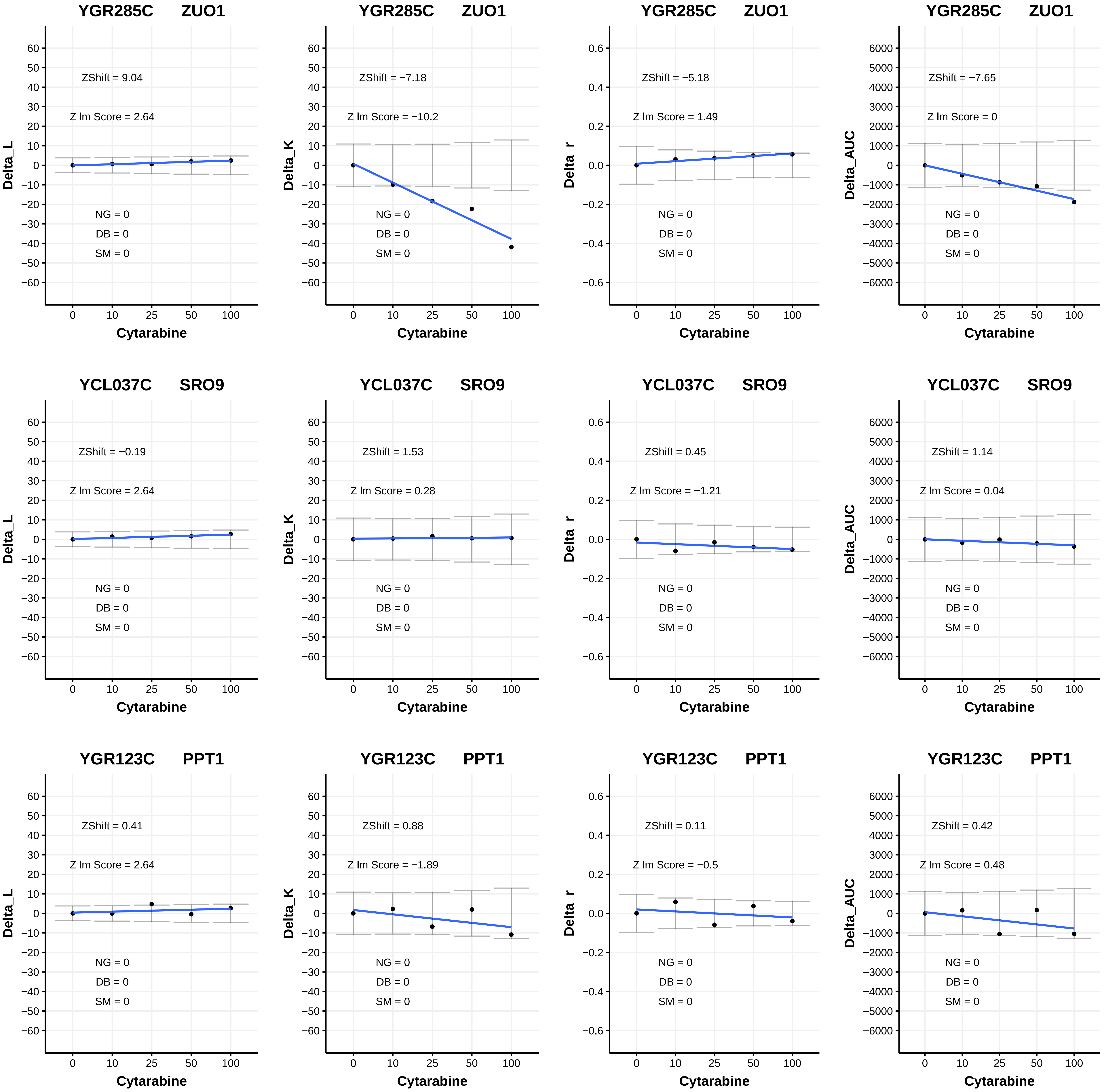

YGR285C ZUO1
ZShift = 9.04
Z lm Score = 2.64
NG = 0
DB = 0
SM = 0
Delta_L
Cytarabine
YGR285C ZUO1
ZShift = −7.18
Z lm Score = −10.2
NG = 0
DB = 0
SM = 0
Delta_K
Cytarabine
YGR285C ZUO1
ZShift = −5.18
Z lm Score = 1.49
NG = 0
DB = 0
SM = 0
Delta_r
Cytarabine
YGR285C ZUO1
ZShift = −7.65
Z lm Score = 0
NG = 0
DB = 0
SM = 0
Delta_AUC
Cytarabine
YCL037C SRO9
ZShift = −0.19
Z lm Score = 2.64
NG = 0
DB = 0
SM = 0
Delta_L
Cytarabine
YCL037C SRO9
ZShift = 1.53
Z lm Score = 0.28
NG = 0
DB = 0
SM = 0
Delta_K
Cytarabine
YCL037C SRO9
ZShift = 0.45
Z lm Score = −1.21
NG = 0
DB = 0
SM = 0
Delta_r
Cytarabine
YCL037C SRO9
ZShift = 1.14
Z lm Score = 0.04
NG = 0
DB = 0
SM = 0
Delta_AUC
Cytarabine
YGR123C PPT1
ZShift = 0.41
Z lm Score = 2.64
NG = 0
DB = 0
SM = 0
Delta_L
Cytarabine
YGR123C PPT1
ZShift = 0.88
Z lm Score = −1.89
NG = 0
DB = 0
SM = 0
Delta_K
Cytarabine
YGR123C PPT1
ZShift = 0.11
Z lm Score = −0.5
NG = 0
DB = 0
SM = 0
Delta_r
Cytarabine
YGR123C PPT1
ZShift = 0.42
Z lm Score = 0.48
NG = 0
DB = 0
SM = 0
Delta_AUC
Cytarabine

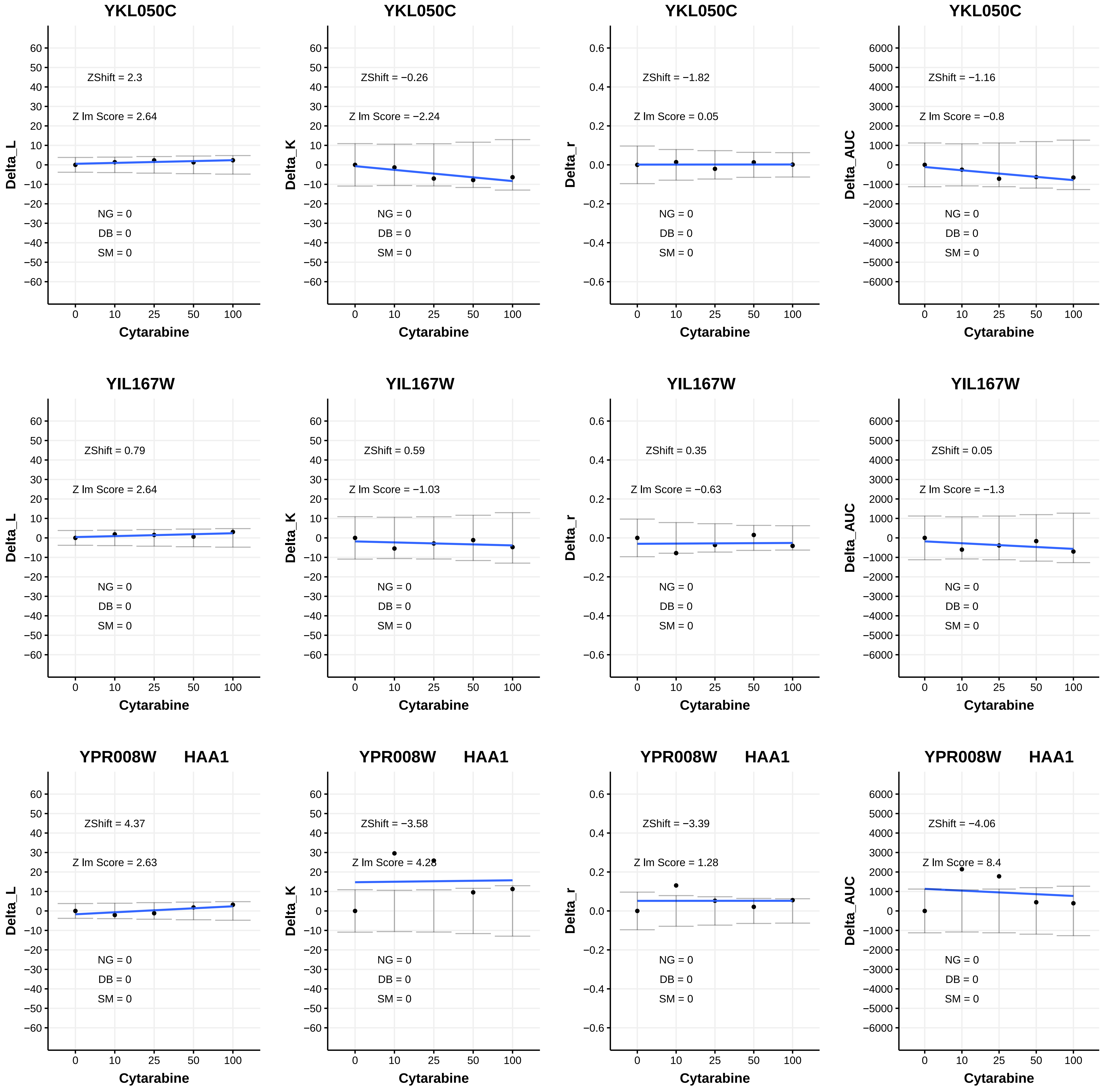

YKL050C
ZShift = 2.3
Z lm Score = 2.64
NG = 0
DB = 0
SM = 0
Delta_L
Cytarabine
YKL050C
ZShift = −0.26
Z lm Score = −2.24
NG = 0
DB = 0
SM = 0
Delta_K
Cytarabine
YKL050C
ZShift = −1.82
Z lm Score = 0.05
NG = 0
DB = 0
SM = 0
Delta_r
Cytarabine
YKL050C
ZShift = −1.16
Z lm Score = −0.8
NG = 0
DB = 0
SM = 0
Delta_AUC
Cytarabine
YIL167W
ZShift = 0.79
Z lm Score = 2.64
NG = 0
DB = 0
SM = 0
Delta_L
Cytarabine
YIL167W
ZShift = 0.59
Z lm Score = −1.03
NG = 0
DB = 0
SM = 0
Delta_K
Cytarabine
YIL167W
ZShift = 0.35
Z lm Score = −0.63
NG = 0
DB = 0
SM = 0
Delta_r
Cytarabine
YIL167W
ZShift = 0.05
Z lm Score = −1.3
NG = 0
DB = 0
SM = 0
Delta_AUC
Cytarabine
YPR008W HAA1
ZShift = 4.37
Z lm Score = 2.63
NG = 0
DB = 0
SM = 0
Delta_L
Cytarabine
YPR008W HAA1
ZShift = −3.58
Z lm Score = 4.28
NG = 0
DB = 0
SM = 0
Delta_K
Cytarabine
YPR008W HAA1
ZShift = −3.39
Z lm Score = 1.28
NG = 0
DB = 0
SM = 0
Delta_r
Cytarabine
YPR008W HAA1
ZShift = −4.06
Z lm Score = 8.4
NG = 0
DB = 0
SM = 0
Delta_AUC
Cytarabine
0
10
25
50
100

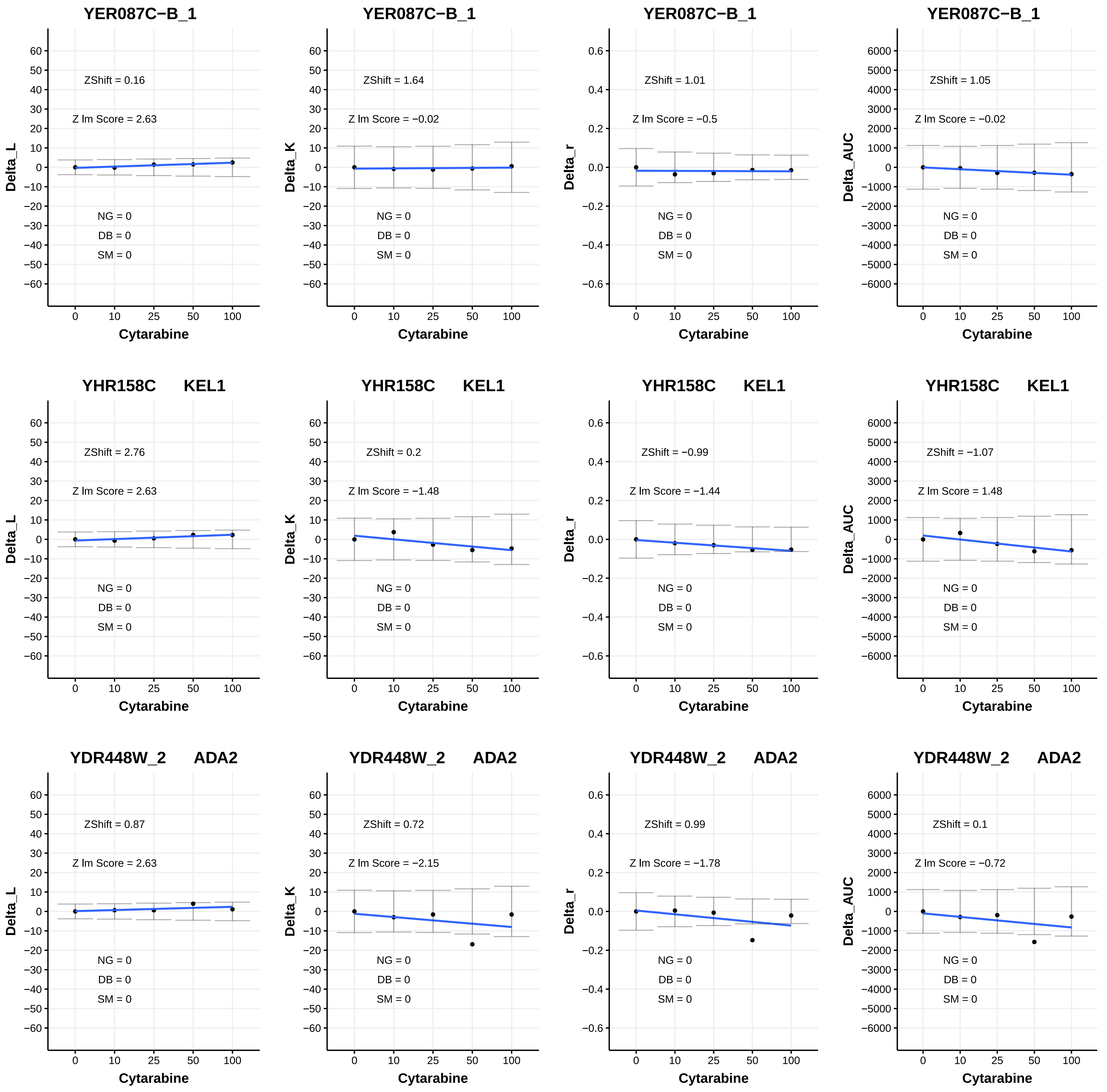

YER087C−B_1
ZShift = 0.16
Z lm Score = 2.63
NG = 0
DB = 0
SM = 0
Delta_L
Cytarabine
YER087C−B_1
ZShift = 1.64
Z lm Score = −0.02
NG = 0
DB = 0
SM = 0
Delta_K
Cytarabine
YER087C−B_1
ZShift = 1.01
Z lm Score = −0.5
NG = 0
DB = 0
SM = 0
Delta_r
Cytarabine
YER087C−B_1
ZShift = 1.05
Z lm Score = −0.02
NG = 0
DB = 0
SM = 0
Delta_AUC
Cytarabine
YHR158C KEL1
ZShift = 2.76
Z lm Score = 2.63
NG = 0
DB = 0
SM = 0
Delta_L
Cytarabine
YHR158C KEL1
ZShift = 0.2
Z lm Score = −1.48
NG = 0
DB = 0
SM = 0
Delta_K
Cytarabine
YHR158C KEL1
ZShift = −0.99
Z lm Score = −1.44
NG = 0
DB = 0
SM = 0
Delta_r
Cytarabine
YHR158C KEL1
ZShift = −1.07
Z lm Score = 1.48
NG = 0
DB = 0
SM = 0
Delta_AUC
Cytarabine
YDR448W_2 ADA2
ZShift = 0.87
Z lm Score = 2.63
NG = 0
DB = 0
SM = 0
Delta_L
Cytarabine
YDR448W_2 ADA2
ZShift = 0.72
Z lm Score = −2.15
NG = 0
DB = 0
SM = 0
Delta_K
Cytarabine
YDR448W_2 ADA2
ZShift = 0.99
Z lm Score = −1.78
NG = 0
DB = 0
SM = 0
Delta_r
Cytarabine
YDR448W_2 ADA2
ZShift = 0.1
Z lm Score = −0.72
NG = 0
DB = 0
SM = 0
Delta_AUC
Cytarabine

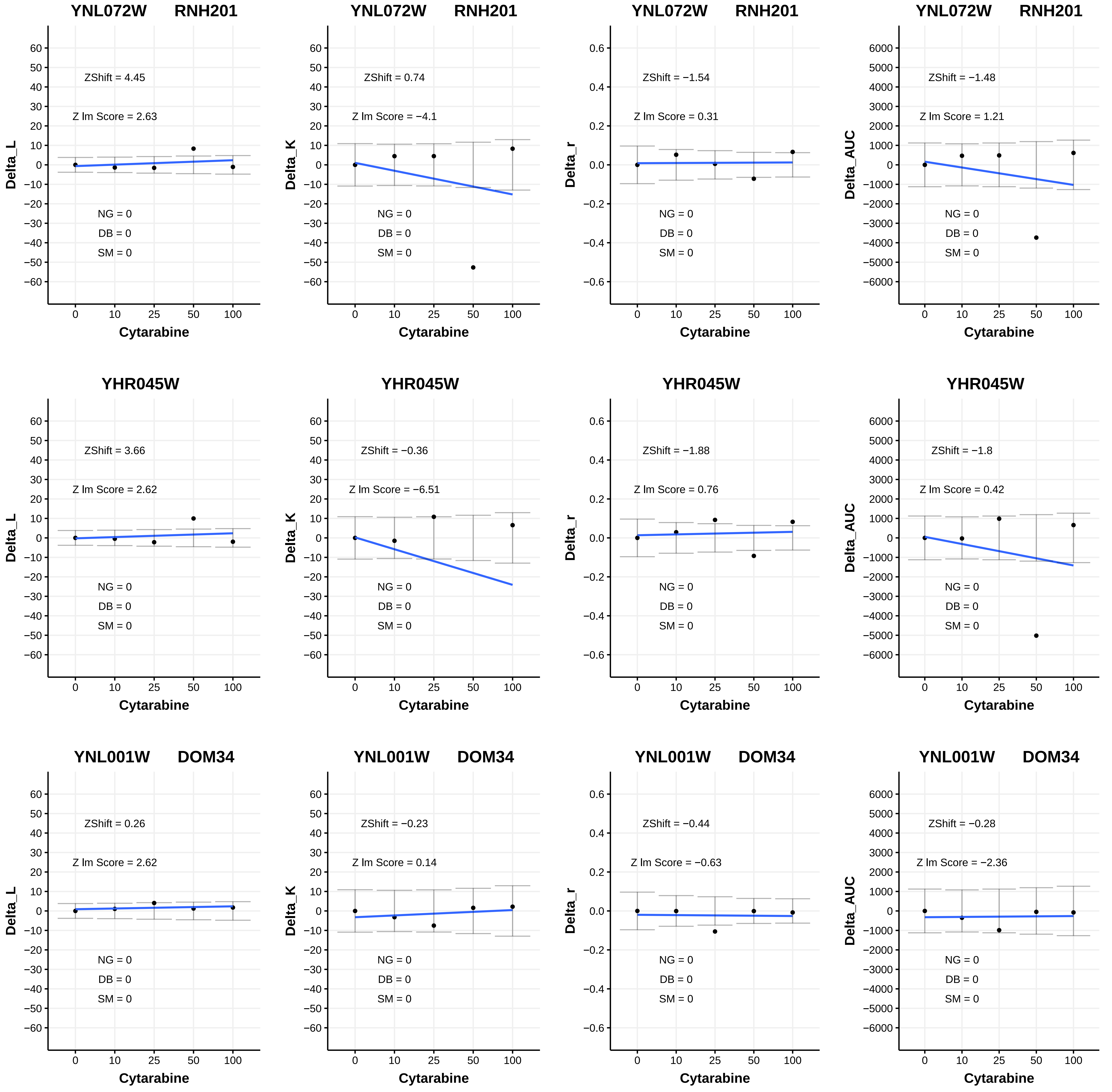
YNL072W RNH201
ZShift = 4.45
Z lm Score = 2.63
NG = 0
DB = 0
SM = 0
Delta_L
Cytarabine
YNL072W RNH201
ZShift = 0.74
Z lm Score = −4.1
NG = 0
DB = 0
SM = 0
Delta_K
Cytarabine
YNL072W RNH201
ZShift = −1.54
Z lm Score = 0.31
NG = 0
DB = 0
SM = 0
Delta_r
Cytarabine
YNL072W RNH201
ZShift = −1.48
Z lm Score = 1.21
NG = 0
DB = 0
SM = 0
Delta_AUC
Cytarabine
YHR045W
ZShift = 3.66
Z lm Score = 2.62
NG = 0
DB = 0
SM = 0
Delta_L
Cytarabine
YHR045W
ZShift = −0.36
Z lm Score = −6.51
NG = 0
DB = 0
SM = 0
Delta_K
Cytarabine
YHR045W
ZShift = −1.88
Z lm Score = 0.76
NG = 0
DB = 0
SM = 0
Delta_r
Cytarabine
YHR045W
ZShift = −1.8
Z lm Score = 0.42
NG = 0
DB = 0
SM = 0
Delta_AUC
Cytarabine
YNL001W DOM34
ZShift = 0.26
Z lm Score = 2.62
NG = 0
DB = 0
SM = 0
Delta_L
Cytarabine
YNL001W DOM34
ZShift = −0.23
Z lm Score = 0.14
NG = 0
DB = 0
SM = 0
Delta_K
Cytarabine
YNL001W DOM34
ZShift = −0.44
Z lm Score = −0.63
NG = 0
DB = 0
SM = 0
Delta_r
Cytarabine
YNL001W DOM34
ZShift = −0.28
Z lm Score = −2.36
NG = 0
DB = 0
SM = 0
Delta_AUC
Cytarabine

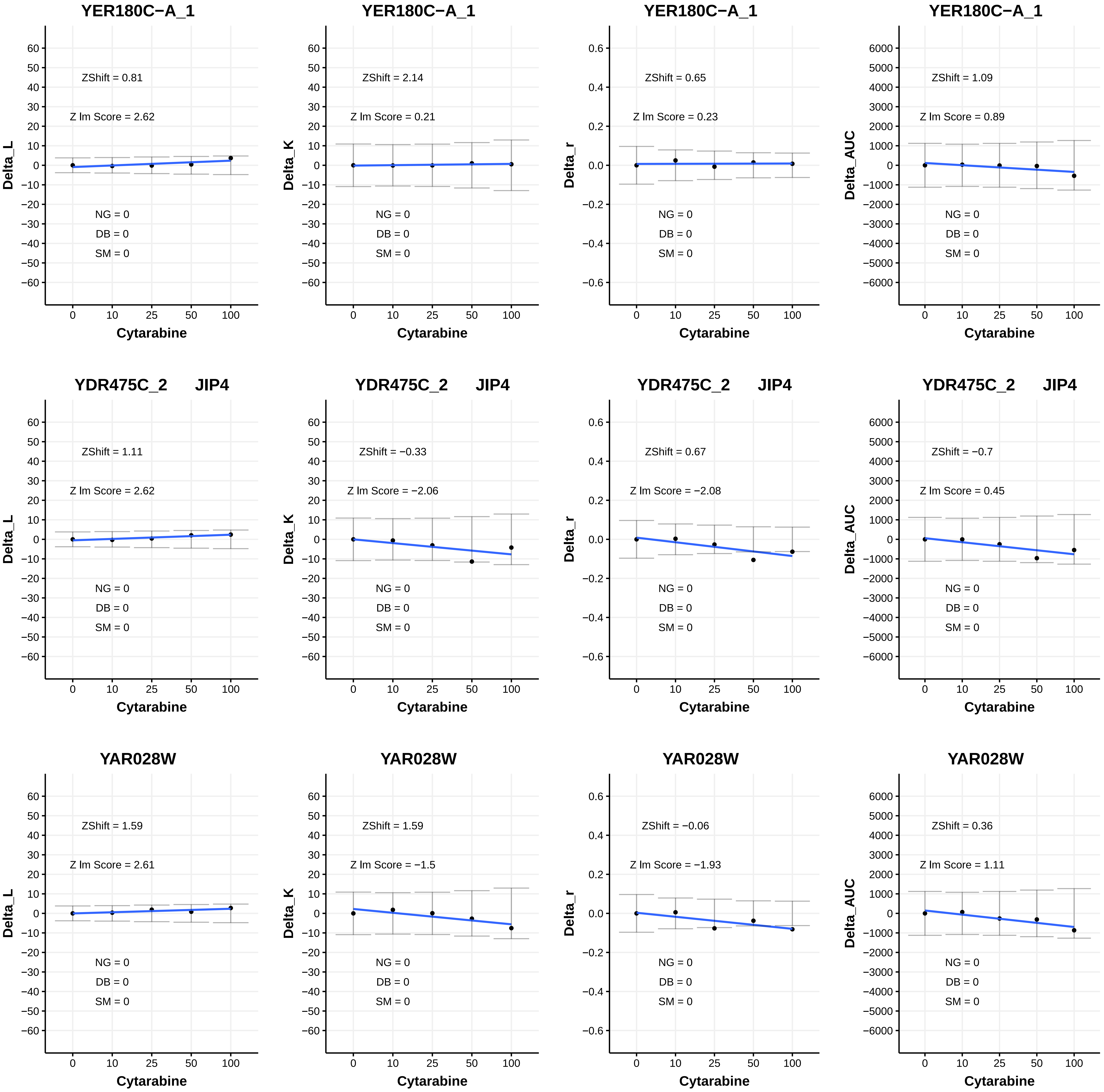
YER180C−A_1
ZShift = 0.81
Z lm Score = 2.62
NG = 0
DB = 0
SM = 0
Delta_L
Cytarabine
YER180C−A_1
ZShift = 2.14
Z lm Score = 0.21
NG = 0
DB = 0
SM = 0
Delta_K
Cytarabine
YER180C−A_1
ZShift = 0.65
Z lm Score = 0.23
NG = 0
DB = 0
SM = 0
Delta_r
Cytarabine
YER180C−A_1
ZShift = 1.09
Z lm Score = 0.89
NG = 0
DB = 0
SM = 0
Delta_AUC
Cytarabine
YDR475C_2 JIP4
ZShift = 1.11
Z lm Score = 2.62
NG = 0
DB = 0
SM = 0
Delta_L
Cytarabine
YDR475C_2 JIP4
ZShift = −0.33
Z lm Score = −2.06
NG = 0
DB = 0
SM = 0
Delta_K
Cytarabine
YDR475C_2 JIP4
ZShift = 0.67
Z lm Score = −2.08
NG = 0
DB = 0
SM = 0
Delta_r
Cytarabine
YDR475C_2 JIP4
ZShift = −0.7
Z lm Score = 0.45
NG = 0
DB = 0
SM = 0
Delta_AUC
Cytarabine
YAR028W
ZShift = 1.59
Z lm Score = 2.61
NG = 0
DB = 0
SM = 0
Delta_L
Cytarabine
YAR028W
ZShift = 1.59
Z lm Score = −1.5
NG = 0
DB = 0
SM = 0
Delta_K
Cytarabine
YAR028W
ZShift = −0.06
Z lm Score = −1.93
NG = 0
DB = 0
SM = 0
Delta_r
Cytarabine
YAR028W
ZShift = 0.36
Z lm Score = 1.11
NG = 0
DB = 0
SM = 0
Delta_AUC
Cytarabine

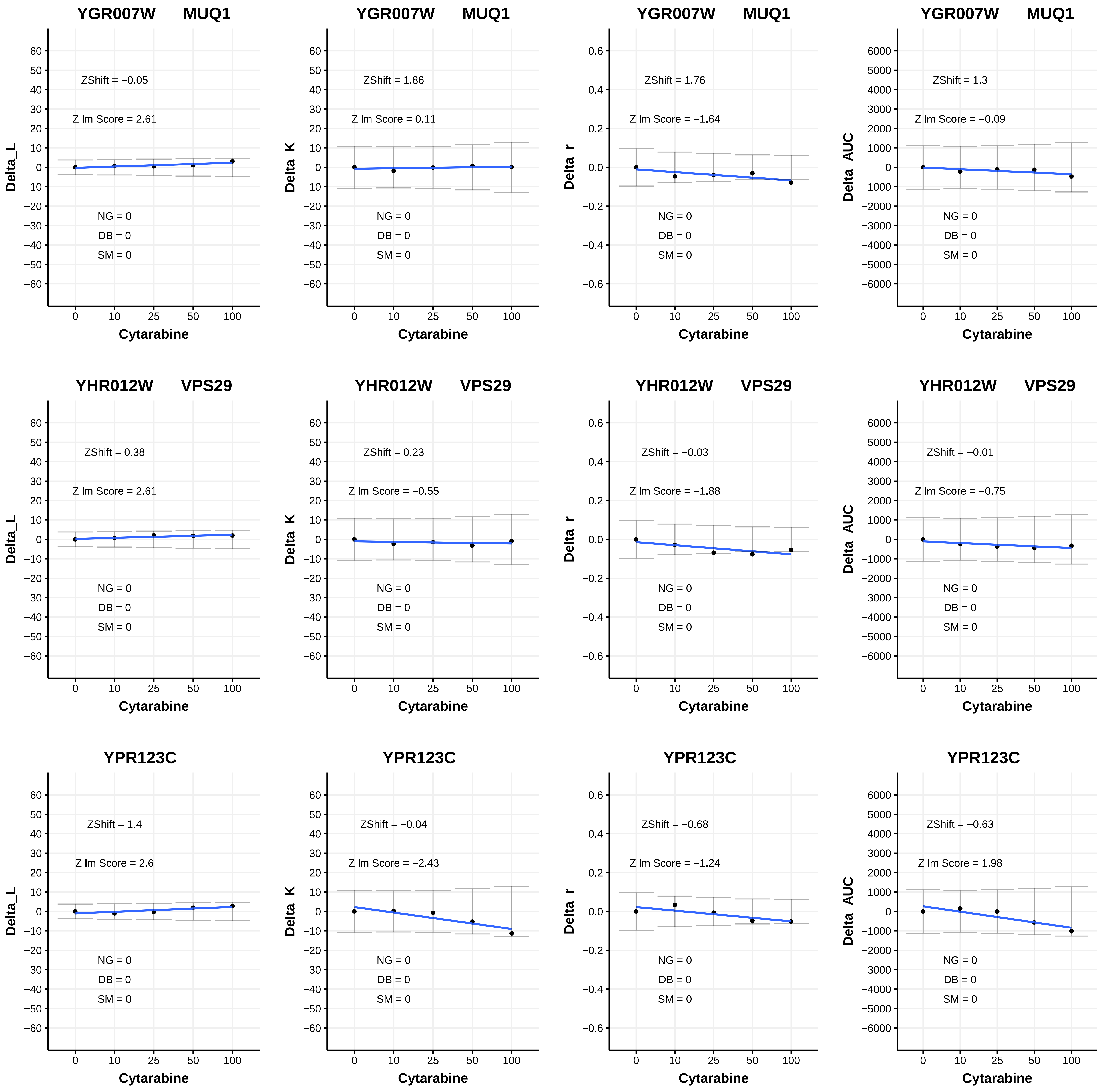

YGR007W MUQ1
ZShift = −0.05
Z lm Score = 2.61
NG = 0
DB = 0
SM = 0
Delta_L
Cytarabine
YGR007W MUQ1
ZShift = 1.86
Z lm Score = 0.11
NG = 0
DB = 0
SM = 0
Delta_K
Cytarabine
YGR007W MUQ1
ZShift = 1.76
Z lm Score = −1.64
NG = 0
DB = 0
SM = 0
Delta_r
Cytarabine
YGR007W MUQ1
ZShift = 1.3
Z lm Score = −0.09
NG = 0
DB = 0
SM = 0
Delta_AUC
Cytarabine
YHR012W VPS29
ZShift = 0.38
Z lm Score = 2.61
NG = 0
DB = 0
SM = 0
Delta_L
Cytarabine
YHR012W VPS29
ZShift = 0.23
Z lm Score = −0.55
NG = 0
DB = 0
SM = 0
Delta_K
Cytarabine
YHR012W VPS29
ZShift = −0.03
Z lm Score = −1.88
NG = 0
DB = 0
SM = 0
Delta_r
Cytarabine
YHR012W VPS29
ZShift = −0.01
Z lm Score = −0.75
NG = 0
DB = 0
SM = 0
Delta_AUC
Cytarabine
YPR123C
ZShift = 1.4
Z lm Score = 2.6
NG = 0
DB = 0
SM = 0
Delta_L
Cytarabine
YPR123C
ZShift = −0.04
Z lm Score = −2.43
NG = 0
DB = 0
SM = 0
Delta_K
Cytarabine
YPR123C
ZShift = −0.68
Z lm Score = −1.24
NG = 0
DB = 0
SM = 0
Delta_r
Cytarabine
YPR123C
ZShift = −0.63
Z lm Score = 1.98
NG = 0
DB = 0
SM = 0
Delta_AUC
Cytarabine

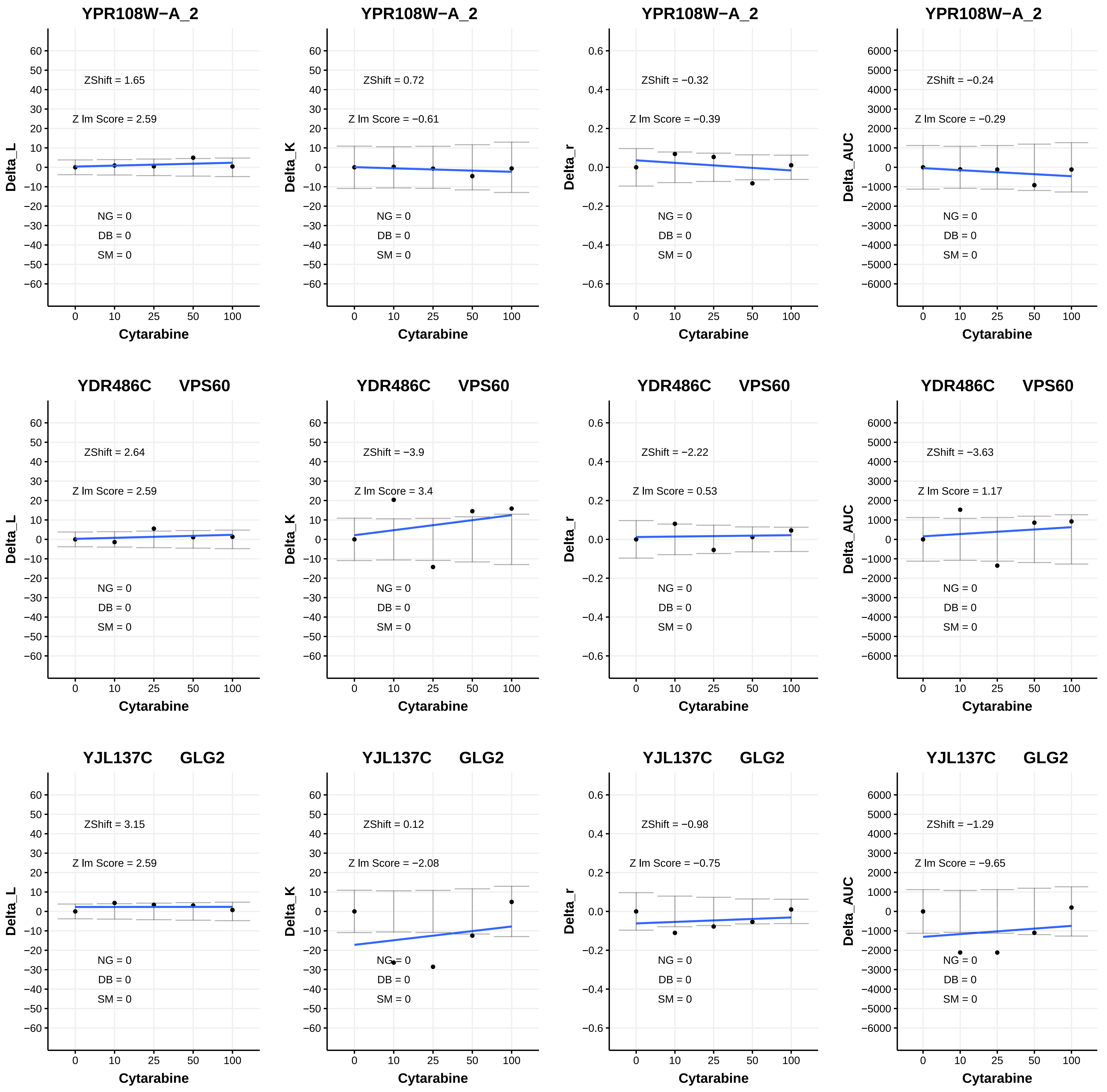

YPR108W−A_2
ZShift = 1.65
Z lm Score = 2.59
NG = 0
DB = 0
SM = 0
Delta_L
Cytarabine
YPR108W−A_2
ZShift = 0.72
Z lm Score = −0.61
NG = 0
DB = 0
SM = 0
Delta_K
Cytarabine
YPR108W−A_2
ZShift = −0.32
Z lm Score = −0.39
NG = 0
DB = 0
SM = 0
Delta_r
Cytarabine
YPR108W−A_2
ZShift = −0.24
Z lm Score = −0.29
NG = 0
DB = 0
SM = 0
Delta_AUC
Cytarabine
YDR486C VPS60
ZShift = 2.64
Z lm Score = 2.59
NG = 0
DB = 0
SM = 0
Delta_L
Cytarabine
YDR486C VPS60
ZShift = −3.9
Z lm Score = 3.4
NG = 0
DB = 0
SM = 0
Delta_K
Cytarabine
YDR486C VPS60
ZShift = −2.22
Z lm Score = 0.53
NG = 0
DB = 0
SM = 0
Delta_r
Cytarabine
YDR486C VPS60
ZShift = −3.63
Z lm Score = 1.17
NG = 0
DB = 0
SM = 0
Delta_AUC
Cytarabine
YJL137C GLG2
ZShift = 3.15
Z lm Score = 2.59
NG = 0
DB = 0
SM = 0
Delta_L
Cytarabine
YJL137C GLG2
ZShift = 0.12
Z lm Score = −2.08
NG = 0
DB = 0
SM = 0
Delta_K
Cytarabine
YJL137C GLG2
ZShift = −0.98
Z lm Score = −0.75
NG = 0
DB = 0
SM = 0
Delta_r
Cytarabine
YJL137C GLG2
ZShift = −1.29
Z lm Score = −9.65
NG = 0
DB = 0
SM = 0
Delta_AUC
Cytarabine

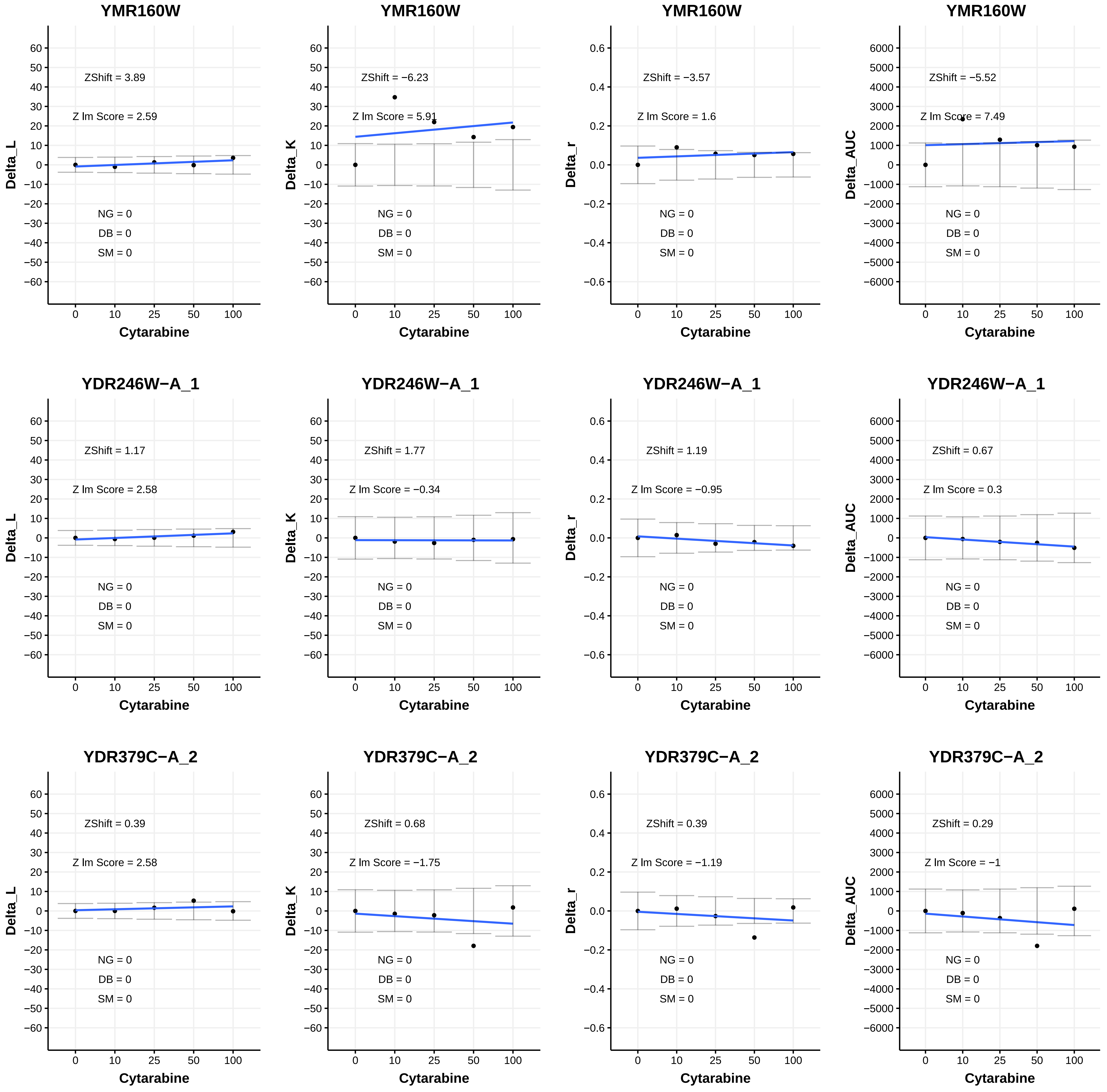

YMR160W
ZShift = 3.89
Z lm Score = 2.59
NG = 0
DB = 0
SM = 0
Delta_L
Cytarabine
YMR160W
ZShift = −6.23
Z lm Score = 5.91
NG = 0
DB = 0
SM = 0
Delta_K
Cytarabine
YMR160W
ZShift = −3.57
Z lm Score = 1.6
NG = 0
DB = 0
SM = 0
Delta_r
Cytarabine
YMR160W
ZShift = −5.52
Z lm Score = 7.49
NG = 0
DB = 0
SM = 0
Delta_AUC
Cytarabine
YDR246W−A_1
ZShift = 1.17
Z lm Score = 2.58
NG = 0
DB = 0
SM = 0
Delta_L
Cytarabine
YDR246W−A_1
ZShift = 1.77
Z lm Score = −0.34
NG = 0
DB = 0
SM = 0
Delta_K
Cytarabine
YDR246W−A_1
ZShift = 1.19
Z lm Score = −0.95
NG = 0
DB = 0
SM = 0
Delta_r
Cytarabine
YDR246W−A_1
ZShift = 0.67
Z lm Score = 0.3
NG = 0
DB = 0
SM = 0
Delta_AUC
Cytarabine
YDR379C−A_2
ZShift = 0.39
Z lm Score = 2.58
NG = 0
DB = 0
SM = 0
Delta_L
Cytarabine
YDR379C−A_2
ZShift = 0.68
Z lm Score = −1.75
NG = 0
DB = 0
SM = 0
Delta_K
Cytarabine
YDR379C−A_2
ZShift = 0.39
Z lm Score = −1.19
NG = 0
DB = 0
SM = 0
Delta_r
Cytarabine
YDR379C−A_2
ZShift = 0.29
Z lm Score = −1
NG = 0
DB = 0
SM = 0
Delta_AUC
Cytarabine

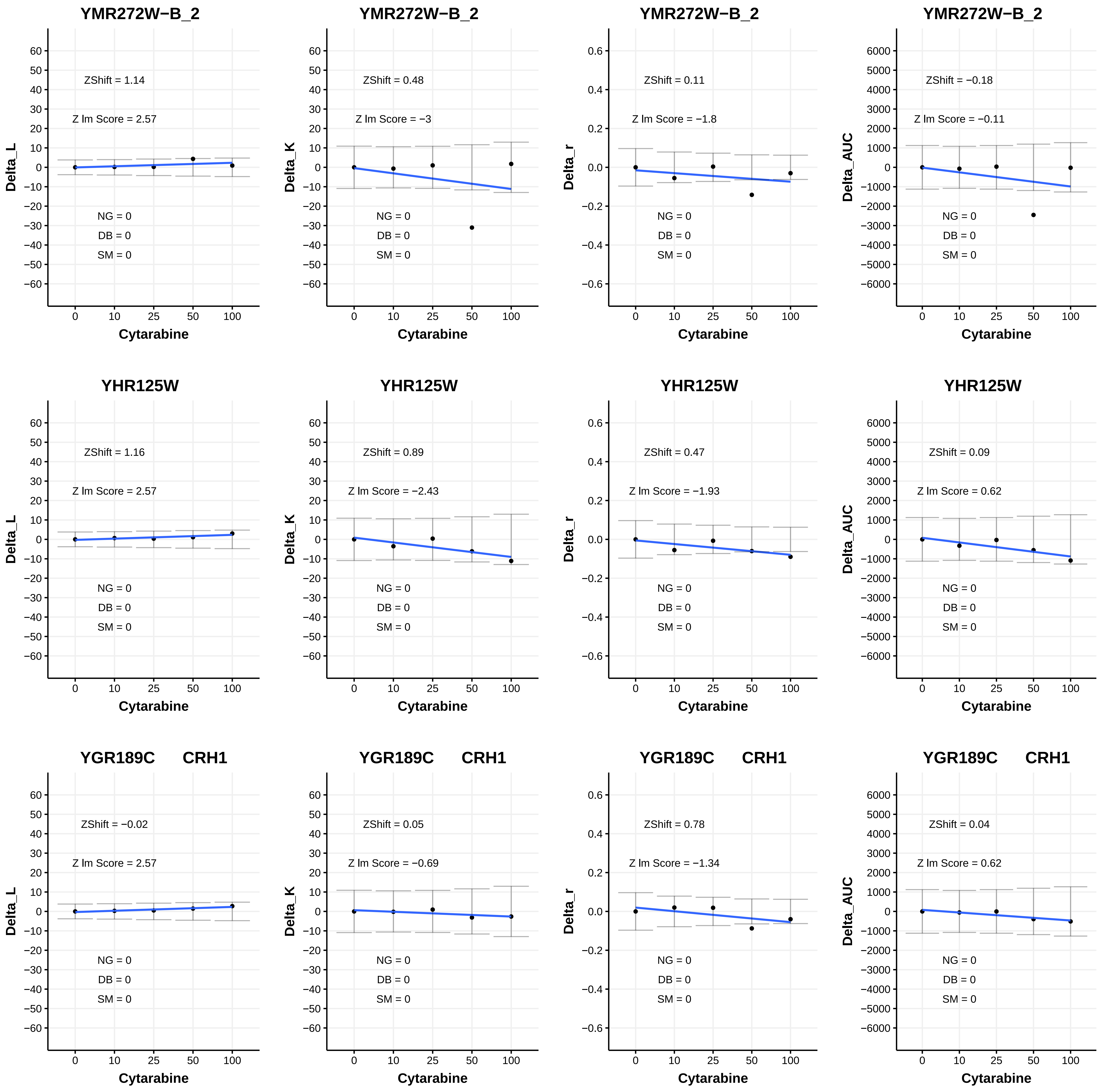

YMR272W−B_2
ZShift = 1.14
Z lm Score = 2.57
NG = 0
DB = 0
SM = 0
Delta_L
Cytarabine
YMR272W−B_2
ZShift = 0.48
Z lm Score = −3
NG = 0
DB = 0
SM = 0
Delta_K
Cytarabine
YMR272W−B_2
ZShift = 0.11
Z lm Score = −1.8
NG = 0
DB = 0
SM = 0
Delta_r
Cytarabine
YMR272W−B_2
ZShift = −0.18
Z lm Score = −0.11
NG = 0
DB = 0
SM = 0
Delta_AUC
Cytarabine
YHR125W
ZShift = 1.16
Z lm Score = 2.57
NG = 0
DB = 0
SM = 0
Delta_L
Cytarabine
YHR125W
ZShift = 0.89
Z lm Score = −2.43
NG = 0
DB = 0
SM = 0
Delta_K
Cytarabine
YHR125W
ZShift = 0.47
Z lm Score = −1.93
NG = 0
DB = 0
SM = 0
Delta_r
Cytarabine
YHR125W
ZShift = 0.09
Z lm Score = 0.62
NG = 0
DB = 0
SM = 0
Delta_AUC
Cytarabine
YGR189C CRH1
ZShift = −0.02
Z lm Score = 2.57
NG = 0
DB = 0
SM = 0
Delta_L
Cytarabine
YGR189C CRH1
ZShift = 0.05
Z lm Score = −0.69
NG = 0
DB = 0
SM = 0
Delta_K
Cytarabine
YGR189C CRH1
ZShift = 0.78
Z lm Score = −1.34
NG = 0
DB = 0
SM = 0
Delta_r
Cytarabine
YGR189C CRH1
ZShift = 0.04
Z lm Score = 0.62
NG = 0
DB = 0
SM = 0
Delta_AUC
Cytarabine

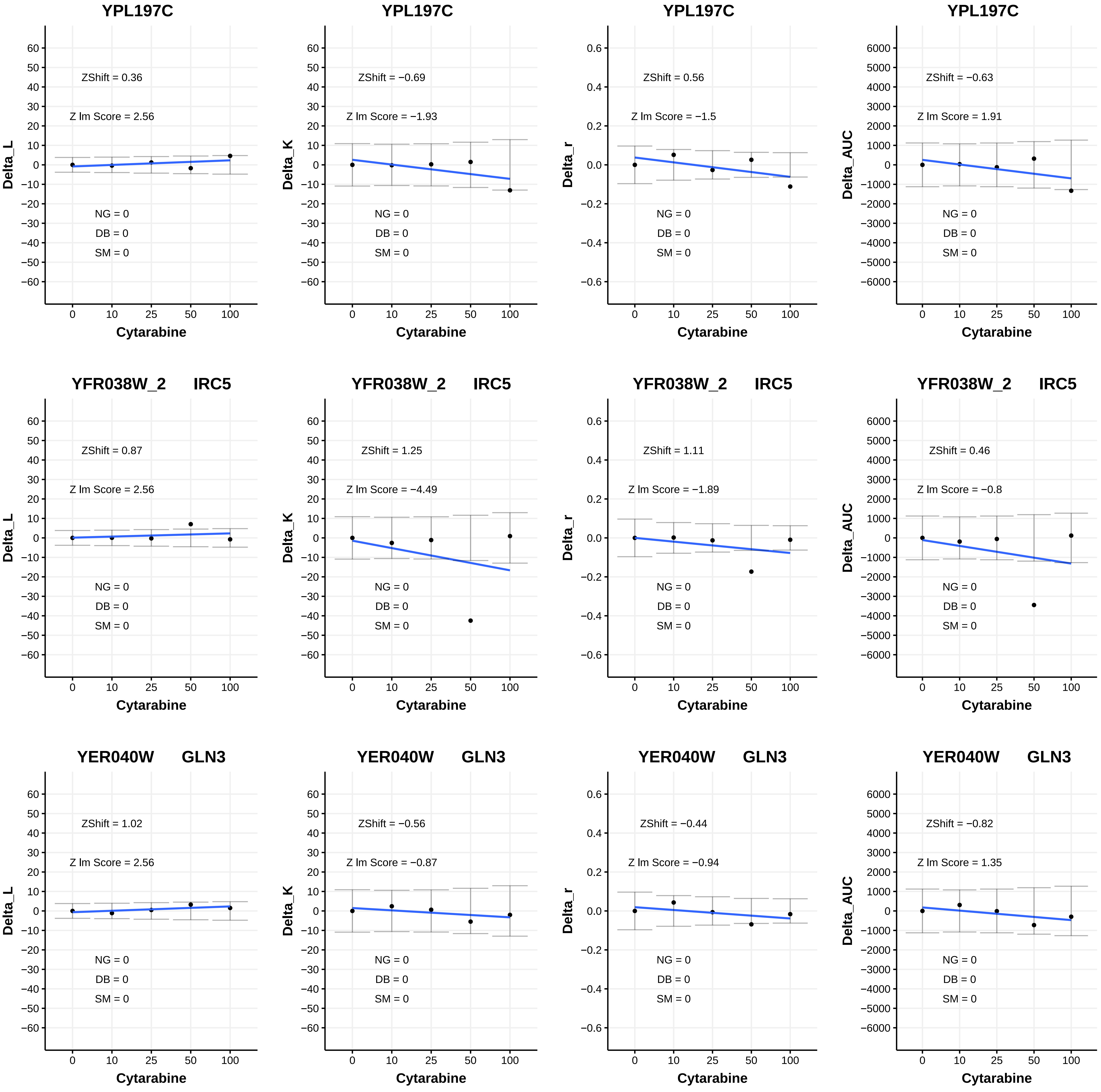
YPL197C
ZShift = 0.36
Z lm Score = 2.56
NG = 0
DB = 0
SM = 0
Delta_L
Cytarabine
YPL197C
ZShift = −0.69
Z lm Score = −1.93
NG = 0
DB = 0
SM = 0
Delta_K
Cytarabine
YPL197C
ZShift = 0.56
Z lm Score = −1.5
NG = 0
DB = 0
SM = 0
Delta_r
Cytarabine
YPL197C
ZShift = −0.63
Z lm Score = 1.91
NG = 0
DB = 0
SM = 0
Delta_AUC
Cytarabine
YFR038W_2 IRC5
ZShift = 0.87
Z lm Score = 2.56
NG = 0
DB = 0
SM = 0
Delta_L
Cytarabine
YFR038W_2 IRC5
ZShift = 1.25
Z lm Score = −4.49
NG = 0
DB = 0
SM = 0
Delta_K
Cytarabine
YFR038W_2 IRC5
ZShift = 1.11
Z lm Score = −1.89
NG = 0
DB = 0
SM = 0
Delta_r
Cytarabine
YFR038W_2 IRC5
ZShift = 0.46
Z lm Score = −0.8
NG = 0
DB = 0
SM = 0
Delta_AUC
Cytarabine
YER040W GLN3
ZShift = 1.02
Z lm Score = 2.56
NG = 0
DB = 0
SM = 0
Delta_L
Cytarabine
YER040W GLN3
ZShift = −0.56
Z lm Score = −0.87
NG = 0
DB = 0
SM = 0
Delta_K
Cytarabine
YER040W GLN3
ZShift = −0.44
Z lm Score = −0.94
NG = 0
DB = 0
SM = 0
Delta_r
Cytarabine
YER040W GLN3
ZShift = −0.82
Z lm Score = 1.35
NG = 0
DB = 0
SM = 0
Delta_AUC
Cytarabine

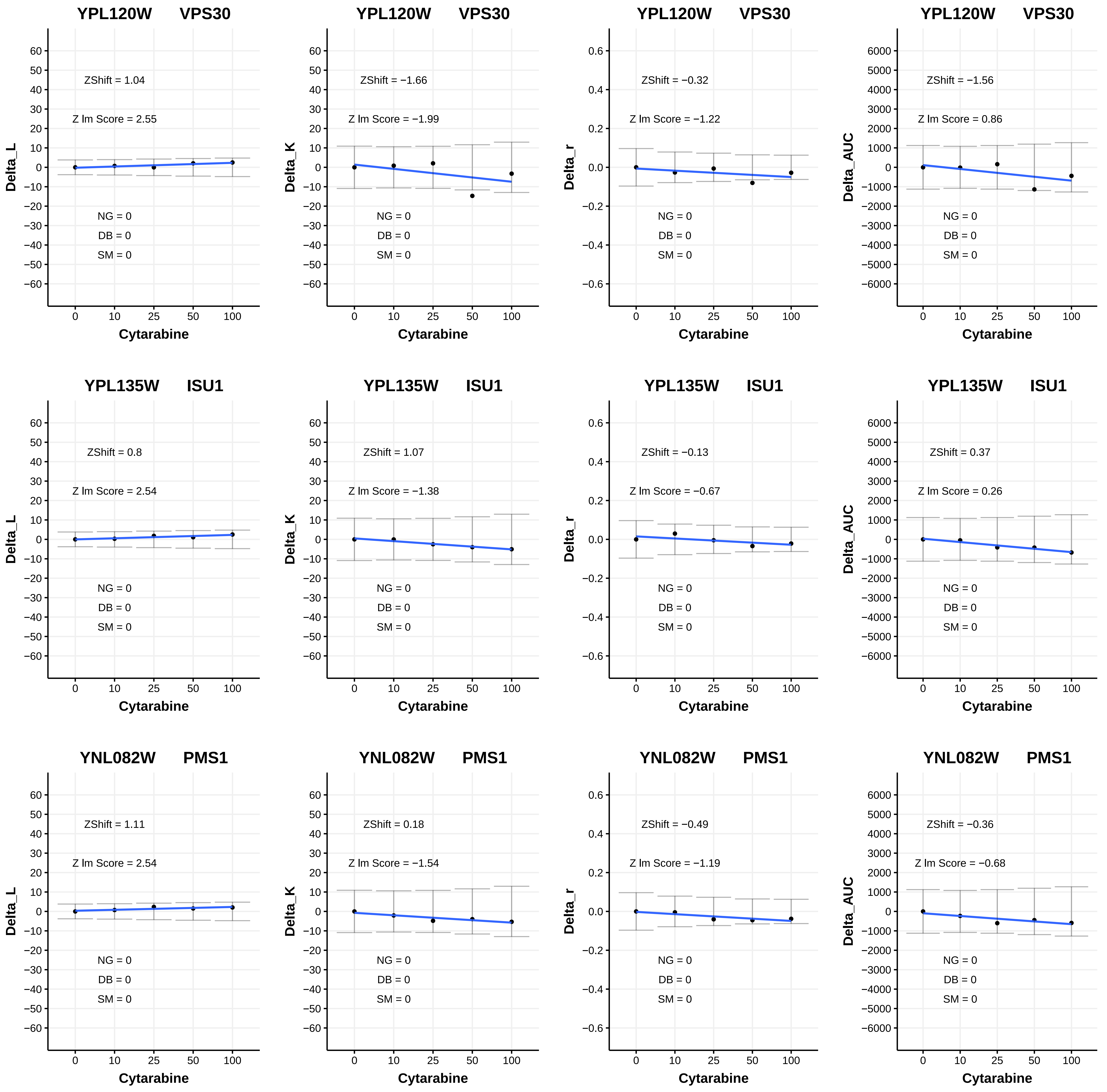

YPL120W VPS30
ZShift = 1.04
Z lm Score = 2.55
NG = 0
DB = 0
SM = 0
Delta_L
Cytarabine
YPL120W VPS30
ZShift = −1.66
Z lm Score = −1.99
NG = 0
DB = 0
SM = 0
Delta_K
Cytarabine
YPL120W VPS30
ZShift = −0.32
Z lm Score = −1.22
NG = 0
DB = 0
SM = 0
Delta_r
Cytarabine
YPL120W VPS30
ZShift = −1.56
Z lm Score = 0.86
NG = 0
DB = 0
SM = 0
Delta_AUC
Cytarabine
YPL135W ISU1
ZShift = 0.8
Z lm Score = 2.54
NG = 0
DB = 0
SM = 0
Delta_L
Cytarabine
YPL135W ISU1
ZShift = 1.07
Z lm Score = −1.38
NG = 0
DB = 0
SM = 0
Delta_K
Cytarabine
YPL135W ISU1
ZShift = −0.13
Z lm Score = −0.67
NG = 0
DB = 0
SM = 0
Delta_r
Cytarabine
YPL135W ISU1
ZShift = 0.37
Z lm Score = 0.26
NG = 0
DB = 0
SM = 0
Delta_AUC
Cytarabine
YNL082W PMS1
ZShift = 1.11
Z lm Score = 2.54
NG = 0
DB = 0
SM = 0
Delta_L
Cytarabine
YNL082W PMS1
ZShift = 0.18
Z lm Score = −1.54
NG = 0
DB = 0
SM = 0
Delta_K
Cytarabine
YNL082W PMS1
ZShift = −0.49
Z lm Score = −1.19
NG = 0
DB = 0
SM = 0
Delta_r
Cytarabine
YNL082W PMS1
ZShift = −0.36
Z lm Score = −0.68
NG = 0
DB = 0
SM = 0
Delta_AUC
Cytarabine

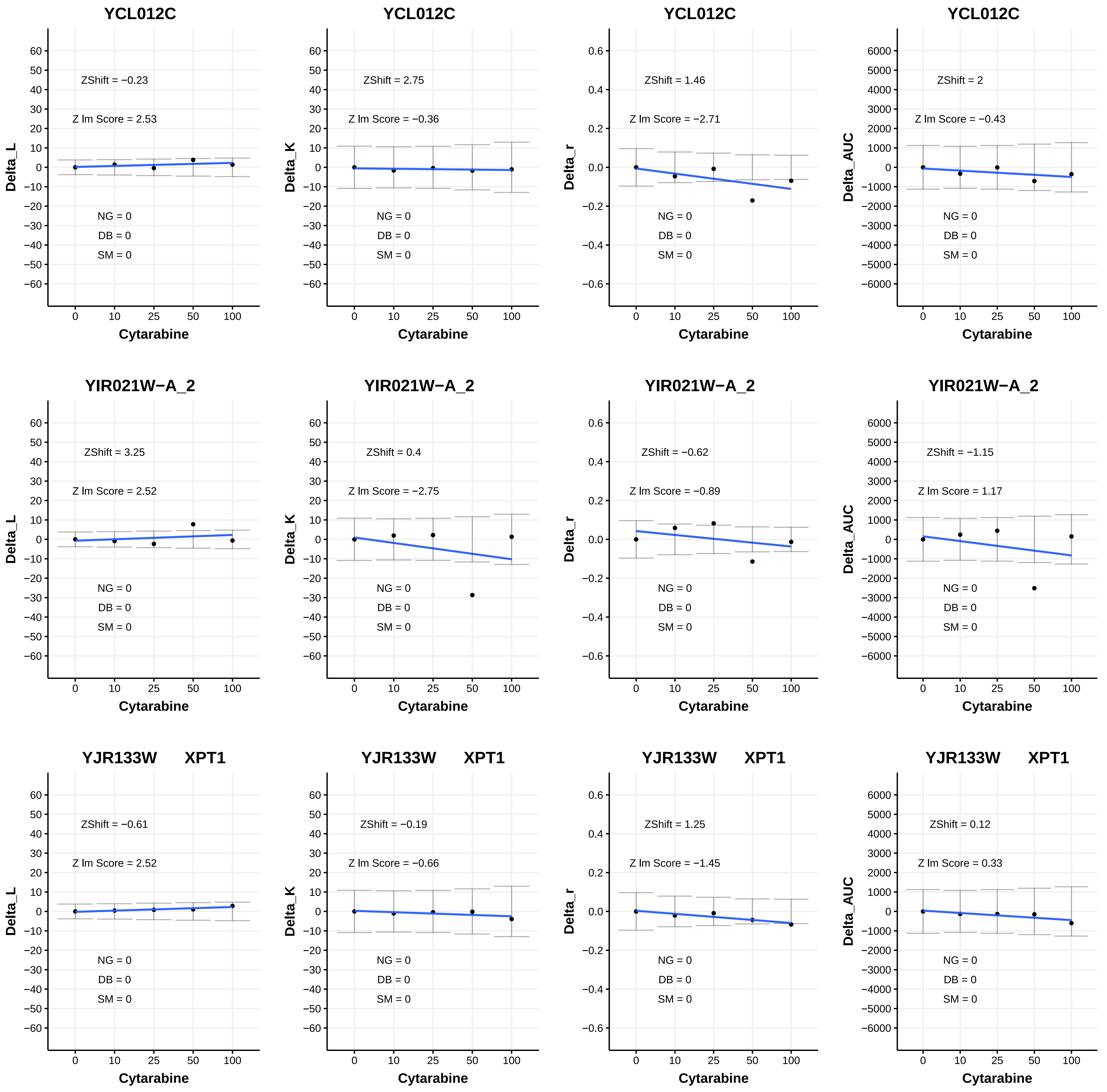
YCL012C
ZShift = −0.23
Z lm Score = 2.53
NG = 0
DB = 0
SM = 0
Delta_L
Cytarabine
YCL012C
ZShift = 2.75
Z lm Score = −0.36
NG = 0
DB = 0
SM = 0
Delta_K
Cytarabine
YCL012C
ZShift = 1.46
Z lm Score = −2.71
NG = 0
DB = 0
SM = 0
Delta_r
Cytarabine
YCL012C
ZShift = 2
Z lm Score = −0.43
NG = 0
DB = 0
SM = 0
Delta_AUC
Cytarabine
YIR021W−A_2
ZShift = 3.25
Z lm Score = 2.52
NG = 0
DB = 0
SM = 0
Delta_L
Cytarabine
YIR021W−A_2
ZShift = 0.4
Z lm Score = −2.75
NG = 0
DB = 0
SM = 0
Delta_K
Cytarabine
YIR021W−A_2
ZShift = −0.62
Z lm Score = −0.89
NG = 0
DB = 0
SM = 0
Delta_r
Cytarabine
YIR021W−A_2
ZShift = −1.15
Z lm Score = 1.17
NG = 0
DB = 0
SM = 0
Delta_AUC
Cytarabine
YJR133W XPT1
ZShift = −0.61
Z lm Score = 2.52
NG = 0
DB = 0
SM = 0
Delta_L
Cytarabine
YJR133W XPT1
ZShift = −0.19
Z lm Score = −0.66
NG = 0
DB = 0
SM = 0
Delta_K
Cytarabine
YJR133W XPT1
ZShift = 1.25
Z lm Score = −1.45
NG = 0
DB = 0
SM = 0
Delta_r
Cytarabine
YJR133W XPT1
ZShift = 0.12
Z lm Score = 0.33
NG = 0
DB = 0
SM = 0
Delta_AUC
Cytarabine

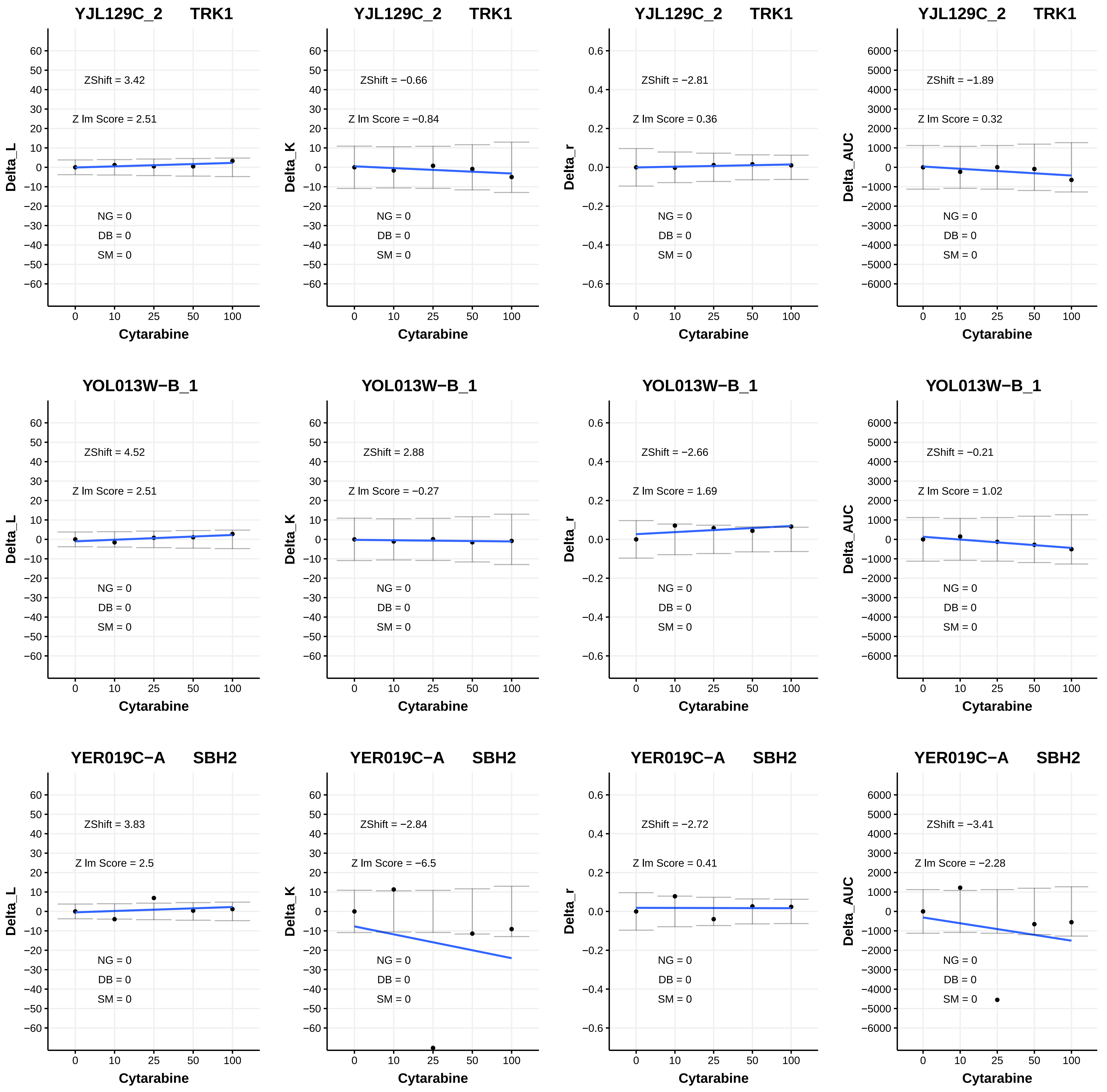

YJL129C_2 TRK1
ZShift = 3.42
Z lm Score = 2.51
NG = 0
DB = 0
SM = 0
Delta_L
Cytarabine
YJL129C_2 TRK1
ZShift = −0.66
Z lm Score = −0.84
NG = 0
DB = 0
SM = 0
Delta_K
Cytarabine
YJL129C_2 TRK1
ZShift = −2.81
Z lm Score = 0.36
NG = 0
DB = 0
SM = 0
Delta_r
Cytarabine
YJL129C_2 TRK1
ZShift = −1.89
Z lm Score = 0.32
NG = 0
DB = 0
SM = 0
Delta_AUC
Cytarabine
YOL013W−B_1
ZShift = 4.52
Z lm Score = 2.51
NG = 0
DB = 0
SM = 0
Delta_L
Cytarabine
YOL013W−B_1
ZShift = 2.88
Z lm Score = −0.27
NG = 0
DB = 0
SM = 0
Delta_K
Cytarabine
YOL013W−B_1
ZShift = −2.66
Z lm Score = 1.69
NG = 0
DB = 0
SM = 0
Delta_r
Cytarabine
YOL013W−B_1
ZShift = −0.21
Z lm Score = 1.02
NG = 0
DB = 0
SM = 0
Delta_AUC
Cytarabine
YER019C−A SBH2
ZShift = 3.83
Z lm Score = 2.5
NG = 0
DB = 0
SM = 0
Delta_L
Cytarabine
YER019C−A SBH2
ZShift = −2.84
Z lm Score = −6.5
NG = 0
DB = 0
SM = 0
Delta_K
Cytarabine
YER019C−A SBH2
ZShift = −2.72
Z lm Score = 0.41
NG = 0
DB = 0
SM = 0
Delta_r
Cytarabine
YER019C−A SBH2
ZShift = −3.41
Z lm Score = −2.28
NG = 0
DB = 0
SM = 0
Delta_AUC
Cytarabine

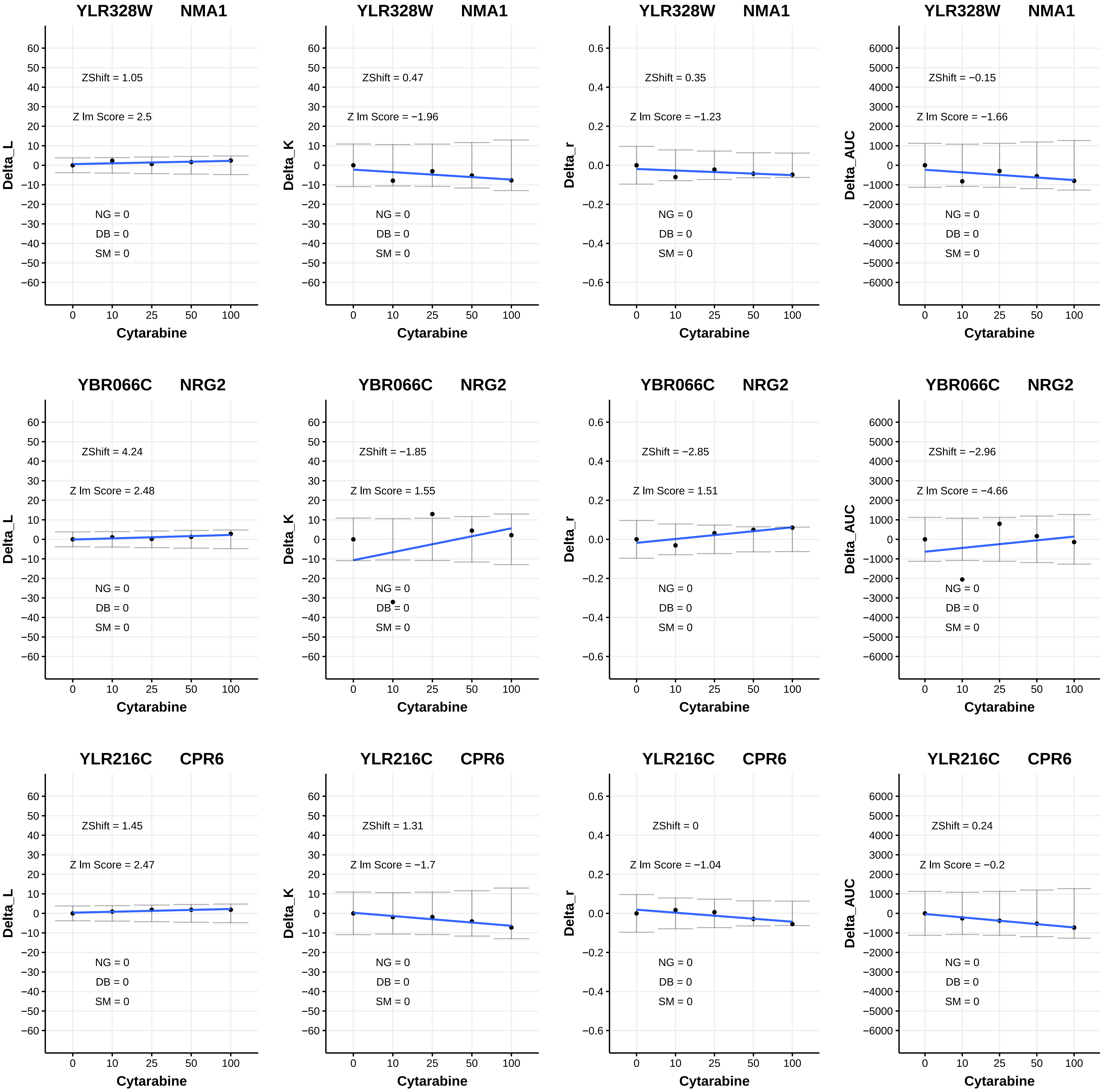

YLR328W NMA1
ZShift = 1.05
Z lm Score = 2.5
NG = 0
DB = 0
SM = 0
Delta_L
Cytarabine
YLR328W NMA1
ZShift = 0.47
Z lm Score = −1.96
NG = 0
DB = 0
SM = 0
Delta_K
Cytarabine
YLR328W NMA1
ZShift = 0.35
Z lm Score = −1.23
NG = 0
DB = 0
SM = 0
Delta_r
Cytarabine
YLR328W NMA1
ZShift = −0.15
Z lm Score = −1.66
NG = 0
DB = 0
SM = 0
Delta_AUC
Cytarabine
YBR066C NRG2
ZShift = 4.24
Z lm Score = 2.48
NG = 0
DB = 0
SM = 0
Delta_L
Cytarabine
YBR066C NRG2
ZShift = −1.85
Z lm Score = 1.55
NG = 0
DB = 0
SM = 0
Delta_K
Cytarabine
YBR066C NRG2
ZShift = −2.85
Z lm Score = 1.51
NG = 0
DB = 0
SM = 0
Delta_r
Cytarabine
YBR066C NRG2
ZShift = −2.96
Z lm Score = −4.66
NG = 0
DB = 0
SM = 0
Delta_AUC
Cytarabine
YLR216C CPR6
ZShift = 1.45
Z lm Score = 2.47
NG = 0
DB = 0
SM = 0
Delta_L
Cytarabine
YLR216C CPR6
ZShift = 1.31
Z lm Score = −1.7
NG = 0
DB = 0
SM = 0
Delta_K
Cytarabine
YLR216C CPR6
ZShift = 0
Z lm Score = −1.04
NG = 0
DB = 0
SM = 0
Delta_r
Cytarabine
YLR216C CPR6
ZShift = 0.24
Z lm Score = −0.2
NG = 0
DB = 0
SM = 0
Delta_AUC
Cytarabine

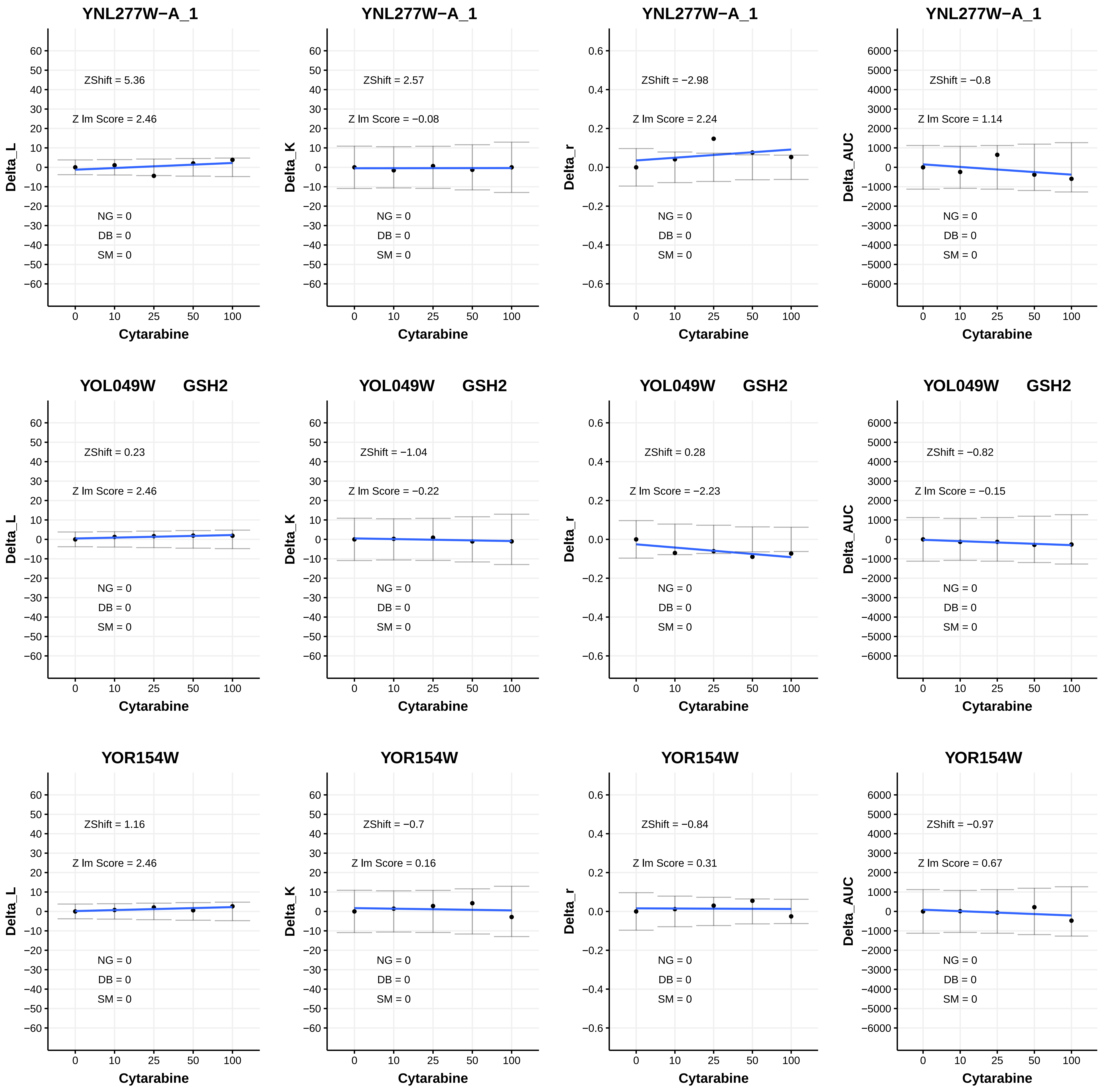
YNL277W−A_1
ZShift = 5.36
Z lm Score = 2.46
NG = 0
DB = 0
SM = 0
Delta_L
Cytarabine
YNL277W−A_1
ZShift = 2.57
Z lm Score = −0.08
NG = 0
DB = 0
SM = 0
Delta_K
Cytarabine
YNL277W−A_1
ZShift = −2.98
Z lm Score = 2.24
NG = 0
DB = 0
SM = 0
Delta_r
Cytarabine
YNL277W−A_1
ZShift = −0.8
Z lm Score = 1.14
NG = 0
DB = 0
SM = 0
Delta_AUC
Cytarabine
YOL049W GSH2
ZShift = 0.23
Z lm Score = 2.46
NG = 0
DB = 0
SM = 0
Delta_L
Cytarabine
YOL049W GSH2
ZShift = −1.04
Z lm Score = −0.22
NG = 0
DB = 0
SM = 0
Delta_K
Cytarabine
YOL049W GSH2
ZShift = 0.28
Z lm Score = −2.23
NG = 0
DB = 0
SM = 0
Delta_r
Cytarabine
YOL049W GSH2
ZShift = −0.82
Z lm Score = −0.15
NG = 0
DB = 0
SM = 0
Delta_AUC
Cytarabine
YOR154W
ZShift = 1.16
Z lm Score = 2.46
NG = 0
DB = 0
SM = 0
Delta_L
Cytarabine
YOR154W
ZShift = −0.7
Z lm Score = 0.16
NG = 0
DB = 0
SM = 0
Delta_K
Cytarabine
YOR154W
ZShift = −0.84
Z lm Score = 0.31
NG = 0
DB = 0
SM = 0
Delta_r
Cytarabine
YOR154W
ZShift = −0.97
Z lm Score = 0.67
NG = 0
DB = 0
SM = 0
Delta_AUC
Cytarabine

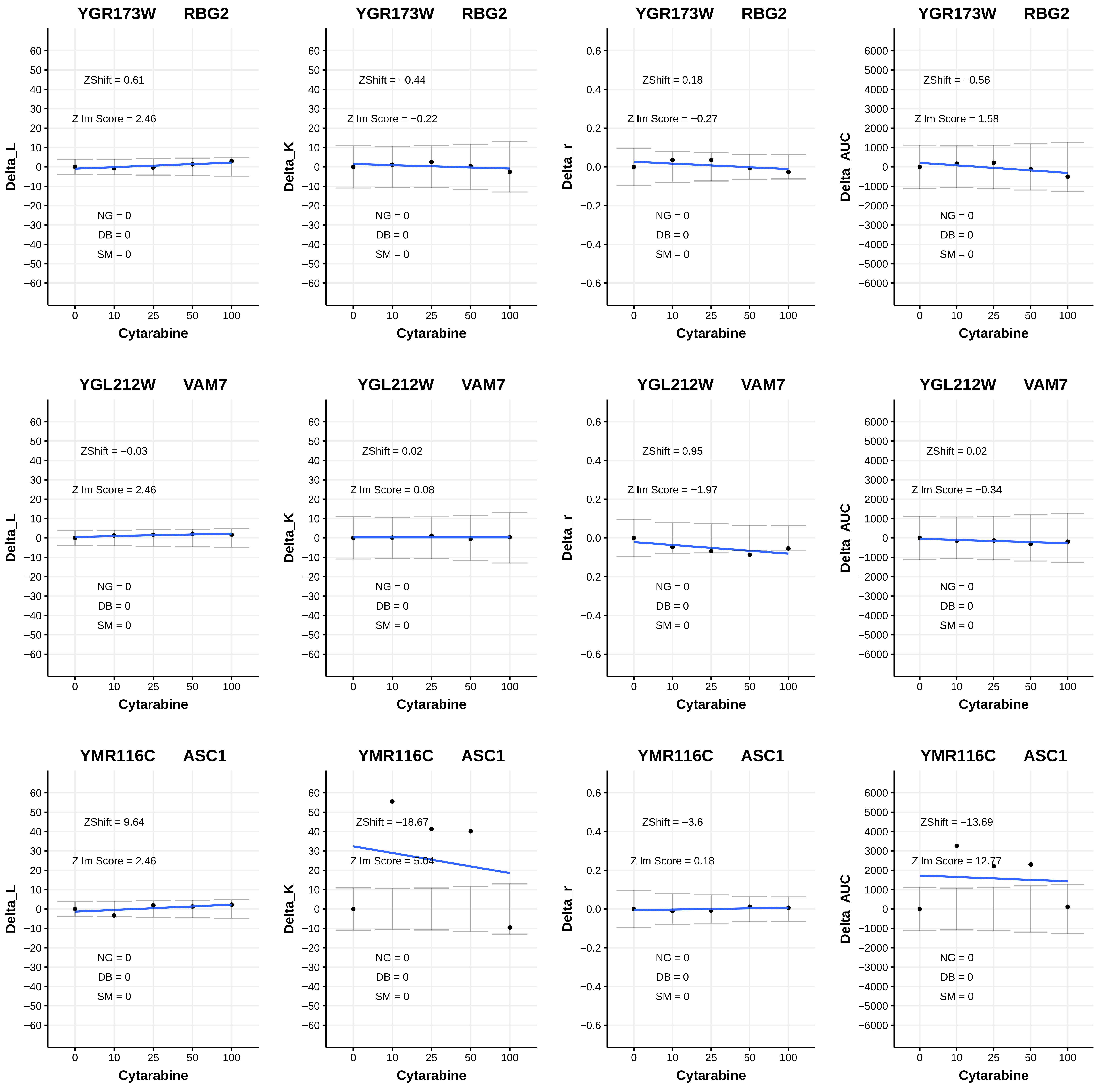
YGR173W RBG2
ZShift = 0.61
Z lm Score = 2.46
NG = 0
DB = 0
SM = 0
Delta_L
Cytarabine
YGR173W RBG2
ZShift = −0.44
Z lm Score = −0.22
NG = 0
DB = 0
SM = 0
Delta_K
Cytarabine
YGR173W RBG2
ZShift = 0.18
Z lm Score = −0.27
NG = 0
DB = 0
SM = 0
Delta_r
Cytarabine
YGR173W RBG2
ZShift = −0.56
Z lm Score = 1.58
NG = 0
DB = 0
SM = 0
Delta_AUC
Cytarabine
YGL212W VAM7
ZShift = −0.03
Z lm Score = 2.46
NG = 0
DB = 0
SM = 0
Delta_L
Cytarabine
YGL212W VAM7
ZShift = 0.02
Z lm Score = 0.08
NG = 0
DB = 0
SM = 0
Delta_K
Cytarabine
YGL212W VAM7
ZShift = 0.95
Z lm Score = −1.97
NG = 0
DB = 0
SM = 0
Delta_r
Cytarabine
YGL212W VAM7
ZShift = 0.02
Z lm Score = −0.34
NG = 0
DB = 0
SM = 0
Delta_AUC
Cytarabine
YMR116C ASC1
ZShift = 9.64
Z lm Score = 2.46
NG = 0
DB = 0
SM = 0
Delta_L
Cytarabine
YMR116C ASC1
ZShift = −18.67
Z lm Score = 5.04
NG = 0
DB = 0
SM = 0
Delta_K
Cytarabine
YMR116C ASC1
ZShift = −3.6
Z lm Score = 0.18
NG = 0
DB = 0
SM = 0
Delta_r
Cytarabine
YMR116C ASC1
ZShift = −13.69
Z lm Score = 12.77
NG = 0
DB = 0
SM = 0
Delta_AUC
Cytarabine

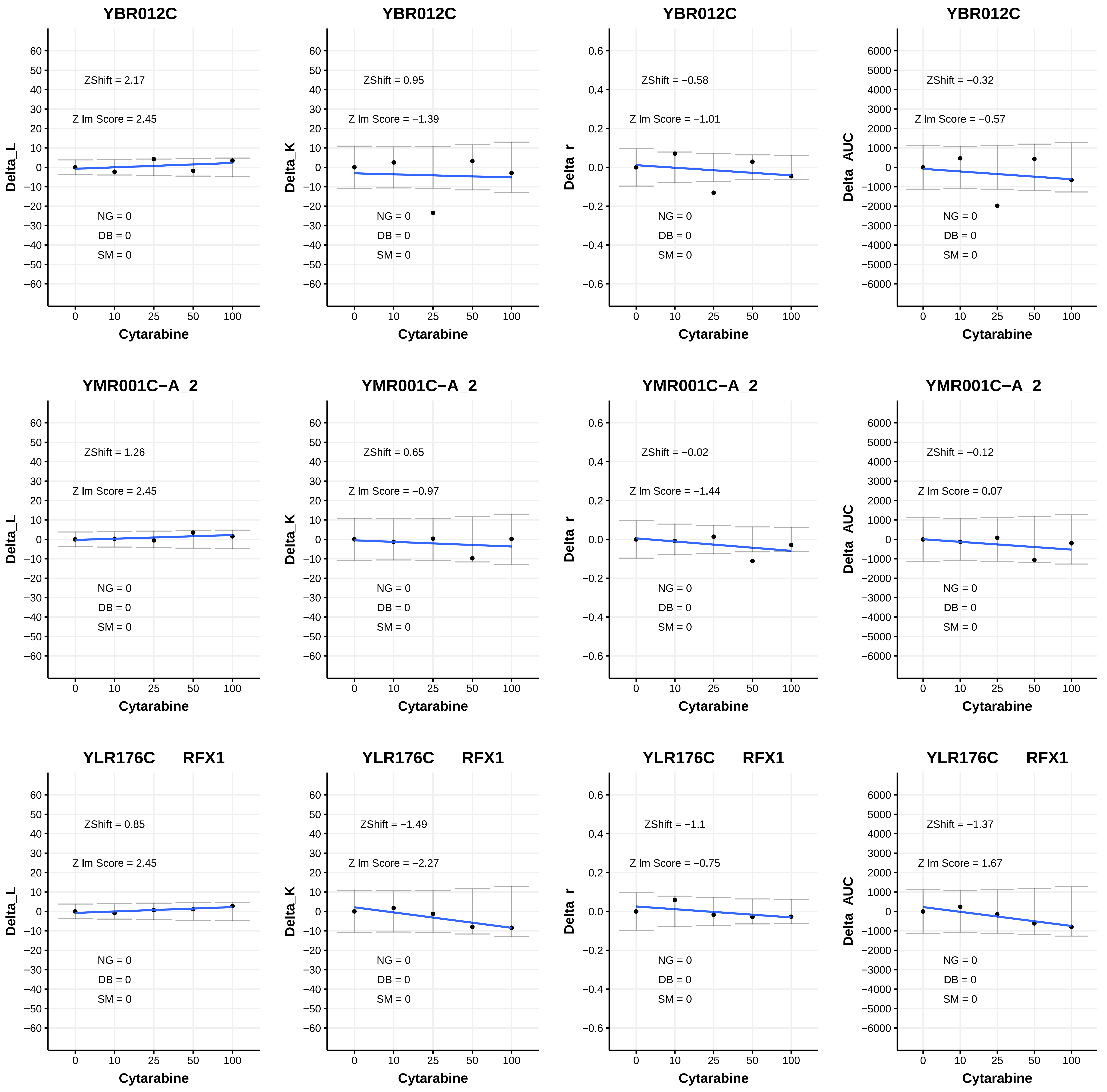

YBR012C
ZShift = 2.17
Z lm Score = 2.45
NG = 0
DB = 0
SM = 0
Delta_L
Cytarabine
YBR012C
ZShift = 0.95
Z lm Score = −1.39
NG = 0
DB = 0
SM = 0
Delta_K
Cytarabine
YBR012C
ZShift = −0.58
Z lm Score = −1.01
NG = 0
DB = 0
SM = 0
Delta_r
Cytarabine
YBR012C
ZShift = −0.32
Z lm Score = −0.57
NG = 0
DB = 0
SM = 0
Delta_AUC
Cytarabine
YMR001C−A_2
ZShift = 1.26
Z lm Score = 2.45
NG = 0
DB = 0
SM = 0
Delta_L
Cytarabine
YMR001C−A_2
ZShift = 0.65
Z lm Score = −0.97
NG = 0
DB = 0
SM = 0
Delta_K
Cytarabine
YMR001C−A_2
ZShift = −0.02
Z lm Score = −1.44
NG = 0
DB = 0
SM = 0
Delta_r
Cytarabine
YMR001C−A_2
ZShift = −0.12
Z lm Score = 0.07
NG = 0
DB = 0
SM = 0
Delta_AUC
Cytarabine
YLR176C RFX1
ZShift = 0.85
Z lm Score = 2.45
NG = 0
DB = 0
SM = 0
Delta_L
Cytarabine
YLR176C RFX1
ZShift = −1.49
Z lm Score = −2.27
NG = 0
DB = 0
SM = 0
Delta_K
Cytarabine
YLR176C RFX1
ZShift = −1.1
Z lm Score = −0.75
NG = 0
DB = 0
SM = 0
Delta_r
Cytarabine
YLR176C RFX1
ZShift = −1.37
Z lm Score = 1.67
NG = 0
DB = 0
SM = 0
Delta_AUC
Cytarabine

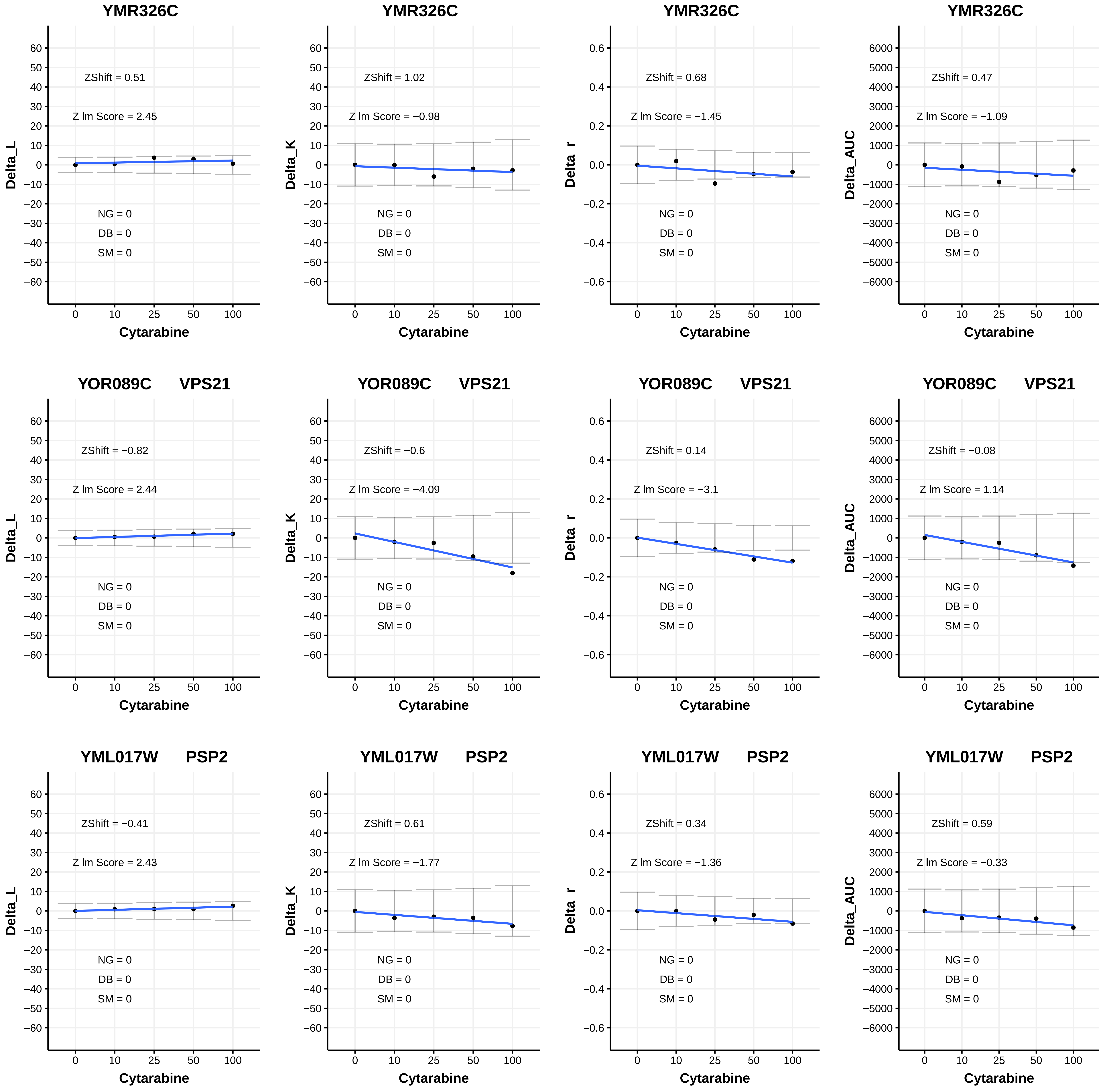

YMR326C
ZShift = 0.51
Z lm Score = 2.45
NG = 0
DB = 0
SM = 0
Delta_L
Cytarabine
YMR326C
ZShift = 1.02
Z lm Score = −0.98
NG = 0
DB = 0
SM = 0
Delta_K
Cytarabine
YMR326C
ZShift = 0.68
Z lm Score = −1.45
NG = 0
DB = 0
SM = 0
Delta_r
Cytarabine
YMR326C
ZShift = 0.47
Z lm Score = −1.09
NG = 0
DB = 0
SM = 0
Delta_AUC
Cytarabine
YOR089C VPS21
ZShift = −0.82
Z lm Score = 2.44
NG = 0
DB = 0
SM = 0
Delta_L
Cytarabine
YOR089C VPS21
ZShift = −0.6
Z lm Score = −4.09
NG = 0
DB = 0
SM = 0
Delta_K
Cytarabine
YOR089C VPS21
ZShift = 0.14
Z lm Score = −3.1
NG = 0
DB = 0
SM = 0
Delta_r
Cytarabine
YOR089C VPS21
ZShift = −0.08
Z lm Score = 1.14
NG = 0
DB = 0
SM = 0
Delta_AUC
Cytarabine
YML017W PSP2
ZShift = −0.41
Z lm Score = 2.43
NG = 0
DB = 0
SM = 0
Delta_L
Cytarabine
YML017W PSP2
ZShift = 0.61
Z lm Score = −1.77
NG = 0
DB = 0
SM = 0
Delta_K
Cytarabine
YML017W PSP2
ZShift = 0.34
Z lm Score = −1.36
NG = 0
DB = 0
SM = 0
Delta_r
Cytarabine
YML017W PSP2
ZShift = 0.59
Z lm Score = −0.33
NG = 0
DB = 0
SM = 0
Delta_AUC
Cytarabine

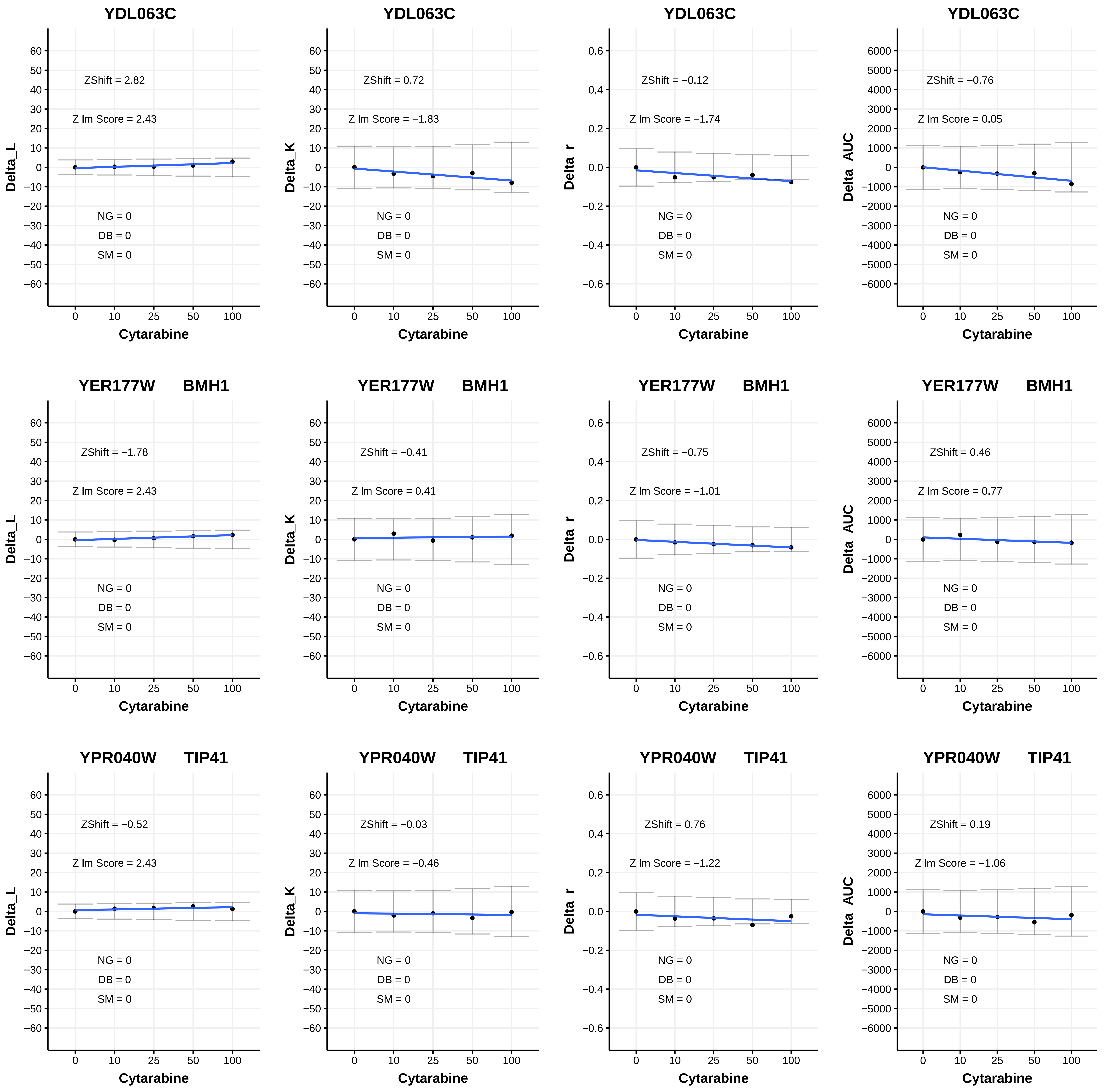
YDL063C
ZShift = 2.82
Z lm Score = 2.43
NG = 0
DB = 0
SM = 0
Delta_L
Cytarabine
YDL063C
ZShift = 0.72
Z lm Score = −1.83
NG = 0
DB = 0
SM = 0
Delta_K
Cytarabine
YDL063C
ZShift = −0.12
Z lm Score = −1.74
NG = 0
DB = 0
SM = 0
Delta_r
Cytarabine
YDL063C
ZShift = −0.76
Z lm Score = 0.05
NG = 0
DB = 0
SM = 0
Delta_AUC
Cytarabine
YER177W BMH1
ZShift = −1.78
Z lm Score = 2.43
NG = 0
DB = 0
SM = 0
Delta_L
Cytarabine
YER177W BMH1
ZShift = −0.41
Z lm Score = 0.41
NG = 0
DB = 0
SM = 0
Delta_K
Cytarabine
YER177W BMH1
ZShift = −0.75
Z lm Score = −1.01
NG = 0
DB = 0
SM = 0
Delta_r
Cytarabine
YER177W BMH1
ZShift = 0.46
Z lm Score = 0.77
NG = 0
DB = 0
SM = 0
Delta_AUC
Cytarabine
YPR040W TIP41
ZShift = −0.52
Z lm Score = 2.43
NG = 0
DB = 0
SM = 0
Delta_L
Cytarabine
YPR040W TIP41
ZShift = −0.03
Z lm Score = −0.46
NG = 0
DB = 0
SM = 0
Delta_K
Cytarabine
YPR040W TIP41
ZShift = 0.76
Z lm Score = −1.22
NG = 0
DB = 0
SM = 0
Delta_r
Cytarabine
YPR040W TIP41
ZShift = 0.19
Z lm Score = −1.06
NG = 0
DB = 0
SM = 0
Delta_AUC
Cytarabine

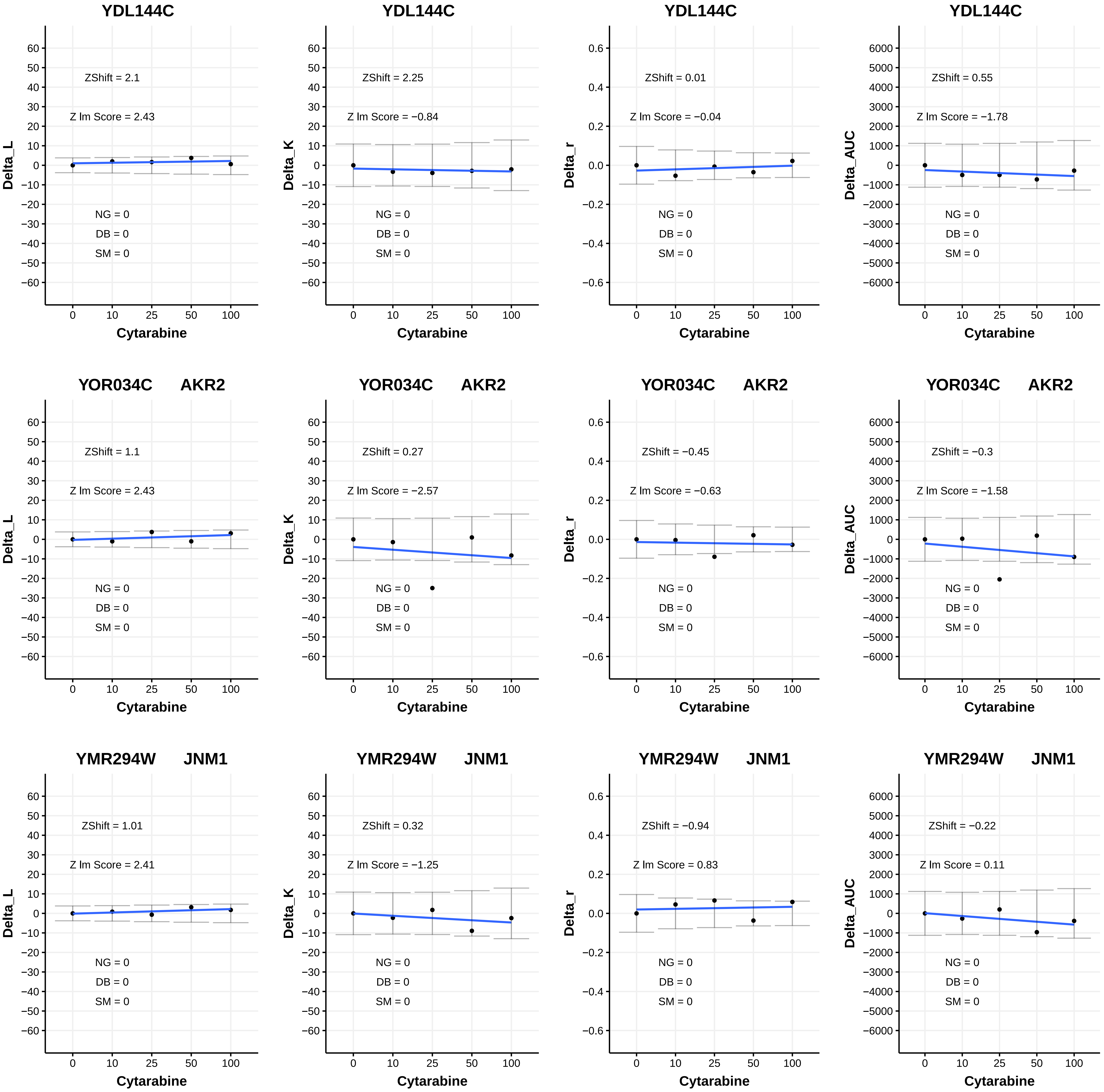

YDL144C
ZShift = 2.1
Z lm Score = 2.43
NG = 0
DB = 0
SM = 0
Delta_L
Cytarabine
YDL144C
ZShift = 2.25
Z lm Score = −0.84
NG = 0
DB = 0
SM = 0
Delta_K
Cytarabine
YDL144C
ZShift = 0.01
Z lm Score = −0.04
NG = 0
DB = 0
SM = 0
Delta_r
Cytarabine
YDL144C
ZShift = 0.55
Z lm Score = −1.78
NG = 0
DB = 0
SM = 0
Delta_AUC
Cytarabine
YOR034C AKR2
ZShift = 1.1
Z lm Score = 2.43
NG = 0
DB = 0
SM = 0
Delta_L
Cytarabine
YOR034C AKR2
ZShift = 0.27
Z lm Score = −2.57
NG = 0
DB = 0
SM = 0
Delta_K
Cytarabine
YOR034C AKR2
ZShift = −0.45
Z lm Score = −0.63
NG = 0
DB = 0
SM = 0
Delta_r
Cytarabine
YOR034C AKR2
ZShift = −0.3
Z lm Score = −1.58
NG = 0
DB = 0
SM = 0
Delta_AUC
Cytarabine
YMR294W JNM1
ZShift = 1.01
Z lm Score = 2.41
NG = 0
DB = 0
SM = 0
Delta_L
Cytarabine
YMR294W JNM1
ZShift = 0.32
Z lm Score = −1.25
NG = 0
DB = 0
SM = 0
Delta_K
Cytarabine
YMR294W JNM1
ZShift = −0.94
Z lm Score = 0.83
NG = 0
DB = 0
SM = 0
Delta_r
Cytarabine
YMR294W JNM1
ZShift = −0.22
Z lm Score = 0.11
NG = 0
DB = 0
SM = 0
Delta_AUC
Cytarabine

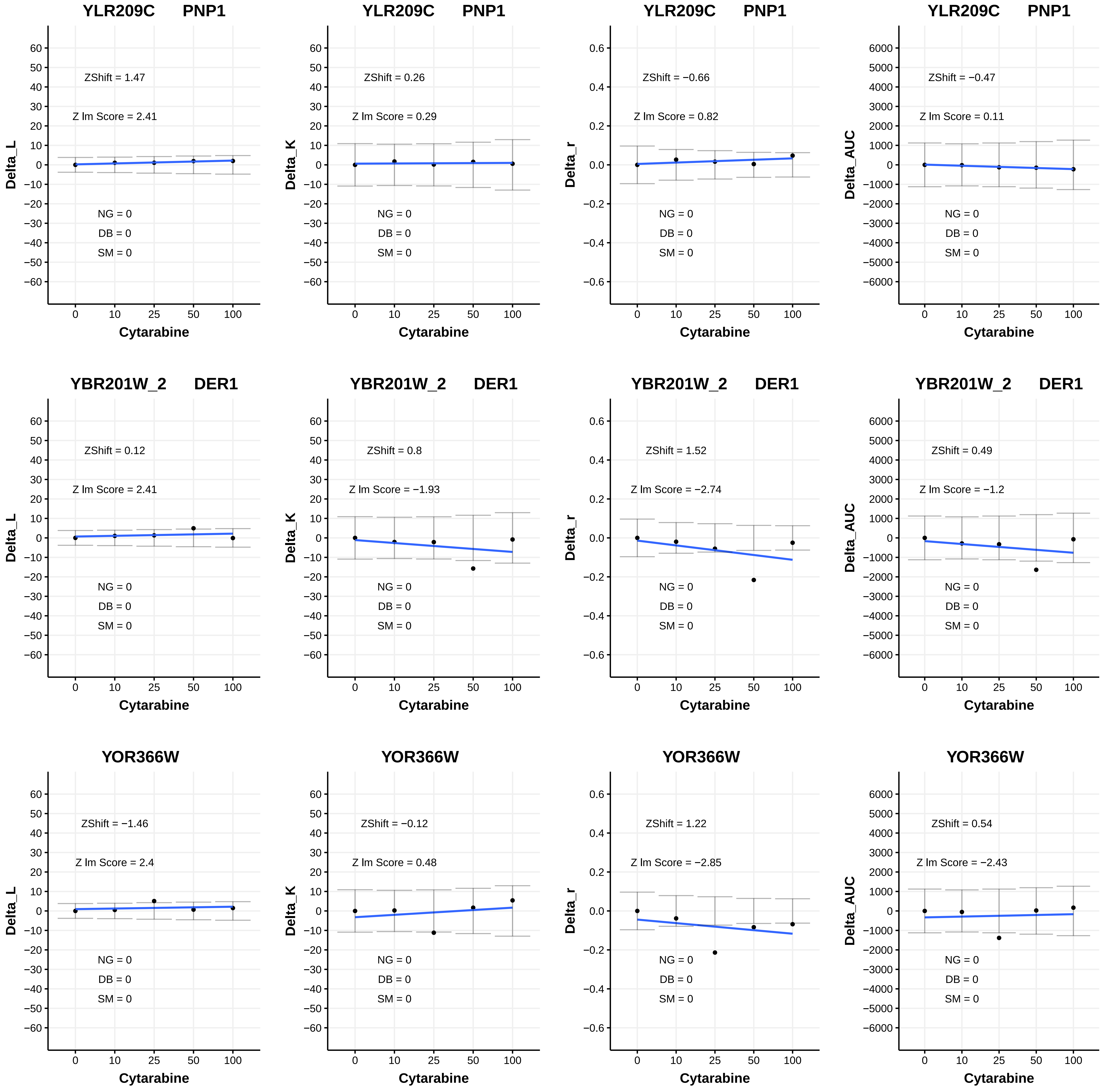
YLR209C PNP1
ZShift = 1.47
Z lm Score = 2.41
NG = 0
DB = 0
SM = 0
Delta_L
Cytarabine
YLR209C PNP1
ZShift = 0.26
Z lm Score = 0.29
NG = 0
DB = 0
SM = 0
Delta_K
Cytarabine
YLR209C PNP1
ZShift = −0.66
Z lm Score = 0.82
NG = 0
DB = 0
SM = 0
Delta_r
Cytarabine
YLR209C PNP1
ZShift = −0.47
Z lm Score = 0.11
NG = 0
DB = 0
SM = 0
Delta_AUC
Cytarabine
YBR201W_2 DER1
ZShift = 0.12
Z lm Score = 2.41
NG = 0
DB = 0
SM = 0
Delta_L
Cytarabine
YBR201W_2 DER1
ZShift = 0.8
Z lm Score = −1.93
NG = 0
DB = 0
SM = 0
Delta_K
Cytarabine
YBR201W_2 DER1
ZShift = 1.52
Z lm Score = −2.74
NG = 0
DB = 0
SM = 0
Delta_r
Cytarabine
YBR201W_2 DER1
ZShift = 0.49
Z lm Score = −1.2
NG = 0
DB = 0
SM = 0
Delta_AUC
Cytarabine
YOR366W
ZShift = −1.46
Z lm Score = 2.4
NG = 0
DB = 0
SM = 0
Delta_L
Cytarabine
YOR366W
ZShift = −0.12
Z lm Score = 0.48
NG = 0
DB = 0
SM = 0
Delta_K
Cytarabine
YOR366W
ZShift = 1.22
Z lm Score = −2.85
NG = 0
DB = 0
SM = 0
Delta_r
Cytarabine
YOR366W
ZShift = 0.54
Z lm Score = −2.43
NG = 0
DB = 0
SM = 0
Delta_AUC
Cytarabine

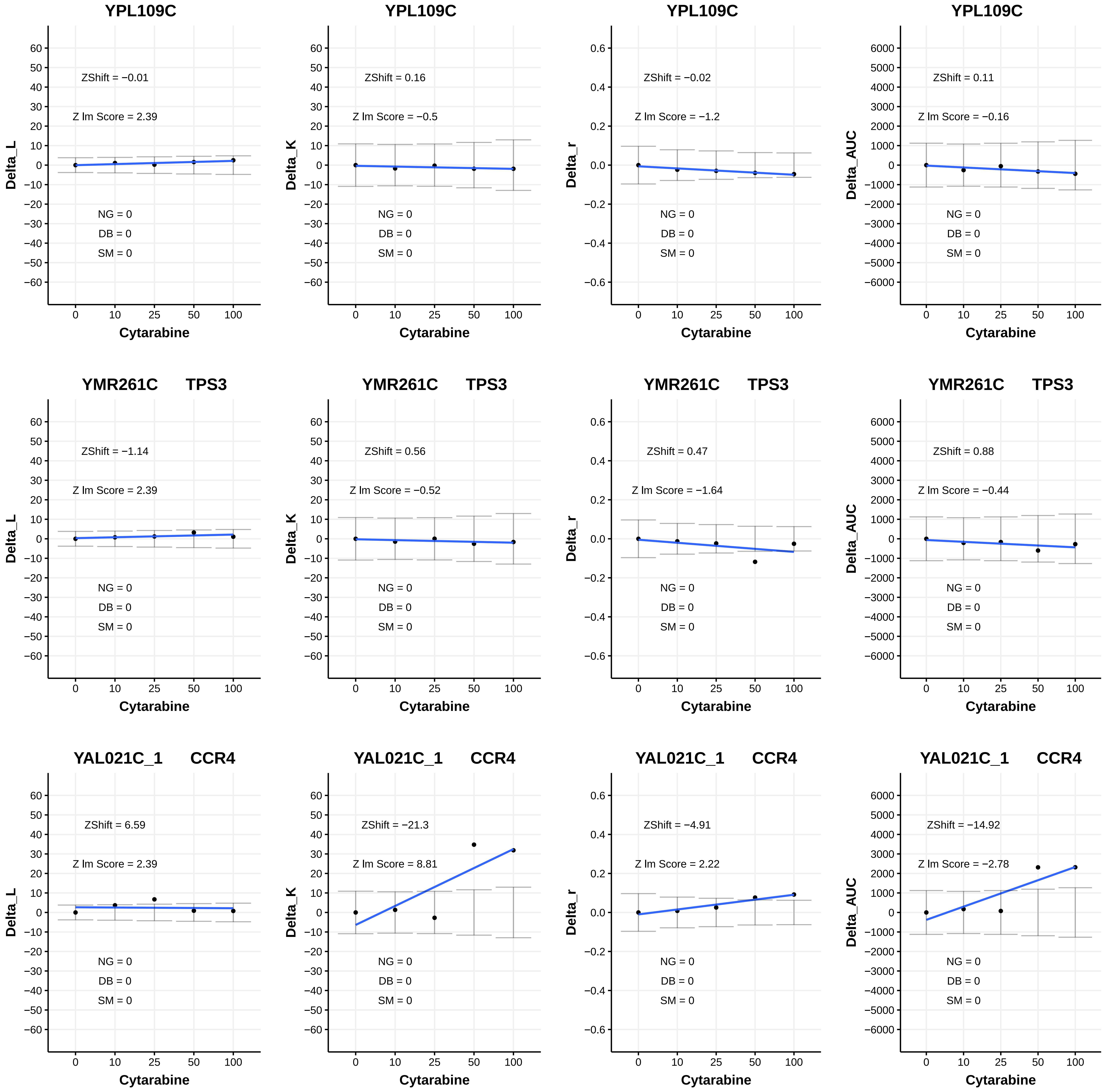

YPL109C
ZShift = −0.01
Z lm Score = 2.39
NG = 0
DB = 0
SM = 0
Delta_L
Cytarabine
YPL109C
ZShift = 0.16
Z lm Score = −0.5
NG = 0
DB = 0
SM = 0
Delta_K
Cytarabine
YPL109C
ZShift = −0.02
Z lm Score = −1.2
NG = 0
DB = 0
SM = 0
Delta_r
Cytarabine
YPL109C
ZShift = 0.11
Z lm Score = −0.16
NG = 0
DB = 0
SM = 0
Delta_AUC
Cytarabine
YMR261C TPS3
ZShift = −1.14
Z lm Score = 2.39
NG = 0
DB = 0
SM = 0
Delta_L
Cytarabine
YMR261C TPS3
ZShift = 0.56
Z lm Score = −0.52
NG = 0
DB = 0
SM = 0
Delta_K
Cytarabine
YMR261C TPS3
ZShift = 0.47
Z lm Score = −1.64
NG = 0
DB = 0
SM = 0
Delta_r
Cytarabine
YMR261C TPS3
ZShift = 0.88
Z lm Score = −0.44
NG = 0
DB = 0
SM = 0
Delta_AUC
Cytarabine
YAL021C_1 CCR4
ZShift = 6.59
Z lm Score = 2.39
NG = 0
DB = 0
SM = 0
Delta_L
Cytarabine
YAL021C_1 CCR4
ZShift = −21.3
Z lm Score = 8.81
NG = 0
DB = 0
SM = 0
Delta_K
Cytarabine
YAL021C_1 CCR4
ZShift = −4.91
Z lm Score = 2.22
NG = 0
DB = 0
SM = 0
Delta_r
Cytarabine
YAL021C_1 CCR4
ZShift = −14.92
Z lm Score = −2.78
NG = 0
DB = 0
SM = 0
Delta_AUC
Cytarabine

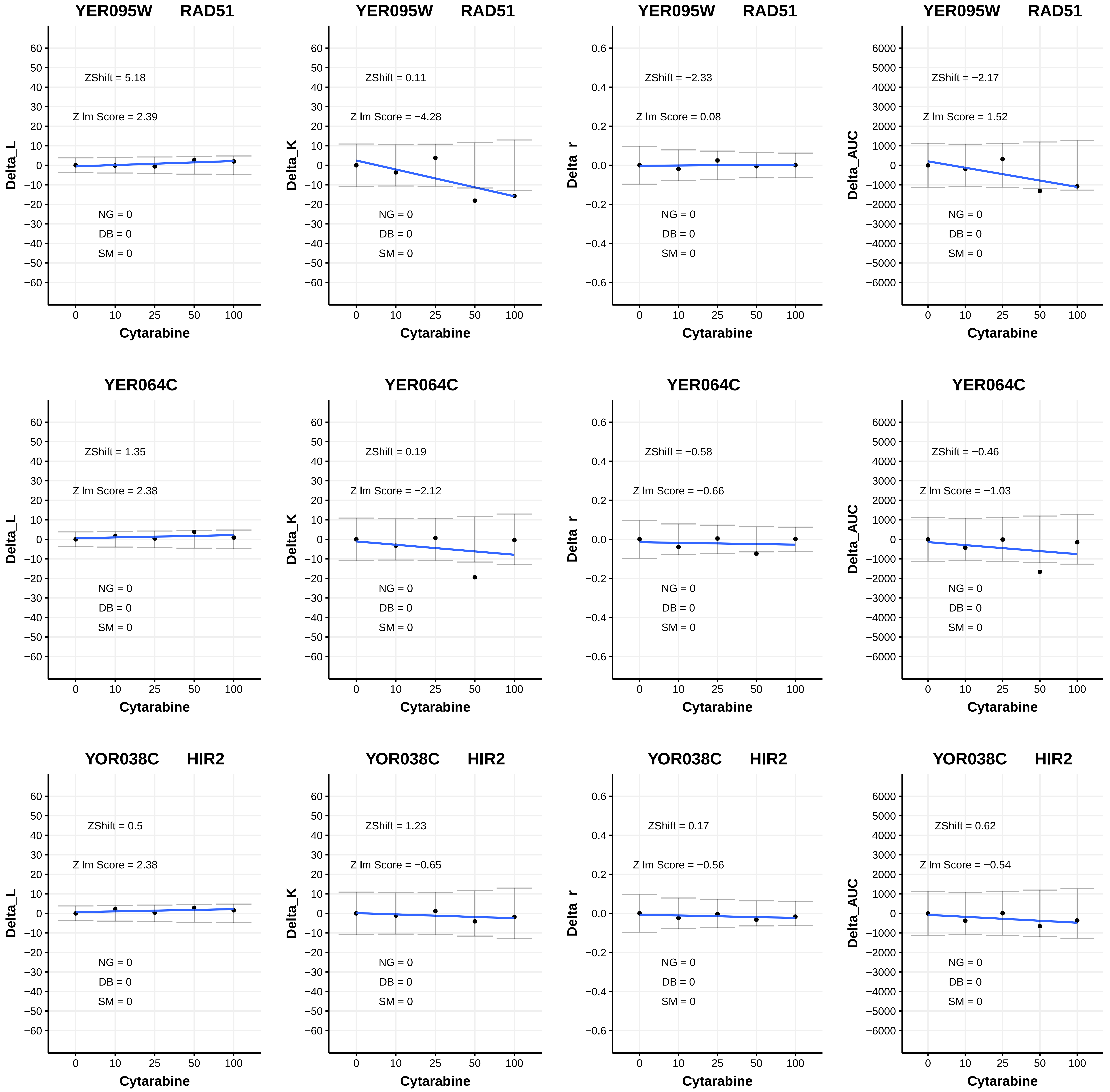
YER095W RAD51
ZShift = 5.18
Z lm Score = 2.39
NG = 0
DB = 0
SM = 0
Delta_L
Cytarabine
YER095W RAD51
ZShift = 0.11
Z lm Score = −4.28
NG = 0
DB = 0
SM = 0
Delta_K
Cytarabine
YER095W RAD51
ZShift = −2.33
Z lm Score = 0.08
NG = 0
DB = 0
SM = 0
Delta_r
Cytarabine
YER095W RAD51
ZShift = −2.17
Z lm Score = 1.52
NG = 0
DB = 0
SM = 0
Delta_AUC
Cytarabine
YER064C
ZShift = 1.35
Z lm Score = 2.38
NG = 0
DB = 0
SM = 0
Delta_L
Cytarabine
YER064C
ZShift = 0.19
Z lm Score = −2.12
NG = 0
DB = 0
SM = 0
Delta_K
Cytarabine
YER064C
ZShift = −0.58
Z lm Score = −0.66
NG = 0
DB = 0
SM = 0
Delta_r
Cytarabine
YER064C
ZShift = −0.46
Z lm Score = −1.03
NG = 0
DB = 0
SM = 0
Delta_AUC
Cytarabine
YOR038C HIR2
ZShift = 0.5
Z lm Score = 2.38
NG = 0
DB = 0
SM = 0
Delta_L
Cytarabine
YOR038C HIR2
ZShift = 1.23
Z lm Score = −0.65
NG = 0
DB = 0
SM = 0
Delta_K
Cytarabine
YOR038C HIR2
ZShift = 0.17
Z lm Score = −0.56
NG = 0
DB = 0
SM = 0
Delta_r
Cytarabine
YOR038C HIR2
ZShift = 0.62
Z lm Score = −0.54
NG = 0
DB = 0
SM = 0
Delta_AUC
Cytarabine

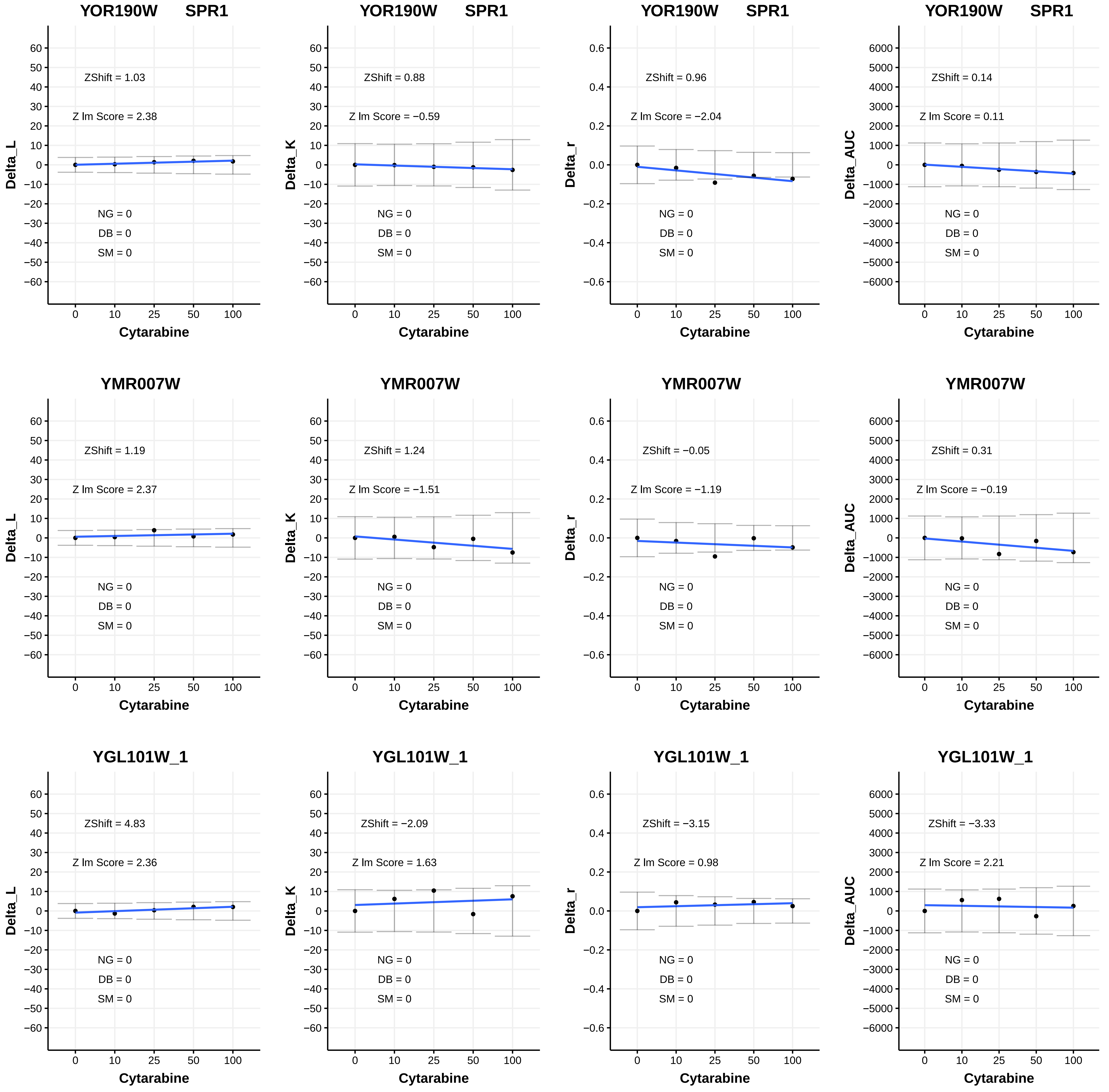

YOR190W SPR1
ZShift = 1.03
Z lm Score = 2.38
NG = 0
DB = 0
SM = 0
Delta_L
Cytarabine
YOR190W SPR1
ZShift = 0.88
Z lm Score = −0.59
NG = 0
DB = 0
SM = 0
Delta_K
Cytarabine
YOR190W SPR1
ZShift = 0.96
Z lm Score = −2.04
NG = 0
DB = 0
SM = 0
Delta_r
Cytarabine
YOR190W SPR1
ZShift = 0.14
Z lm Score = 0.11
NG = 0
DB = 0
SM = 0
Delta_AUC
Cytarabine
YMR007W
ZShift = 1.19
Z lm Score = 2.37
NG = 0
DB = 0
SM = 0
Delta_L
Cytarabine
YMR007W
ZShift = 1.24
Z lm Score = −1.51
NG = 0
DB = 0
SM = 0
Delta_K
Cytarabine
YMR007W
ZShift = −0.05
Z lm Score = −1.19
NG = 0
DB = 0
SM = 0
Delta_r
Cytarabine
YMR007W
ZShift = 0.31
Z lm Score = −0.19
NG = 0
DB = 0
SM = 0
Delta_AUC
Cytarabine
YGL101W_1
ZShift = 4.83
Z lm Score = 2.36
NG = 0
DB = 0
SM = 0
Delta_L
Cytarabine
YGL101W_1
ZShift = −2.09
Z lm Score = 1.63
NG = 0
DB = 0
SM = 0
Delta_K
Cytarabine
YGL101W_1
ZShift = −3.15
Z lm Score = 0.98
NG = 0
DB = 0
SM = 0
Delta_r
Cytarabine
YGL101W_1
ZShift = −3.33
Z lm Score = 2.21
NG = 0
DB = 0
SM = 0
Delta_AUC
Cytarabine

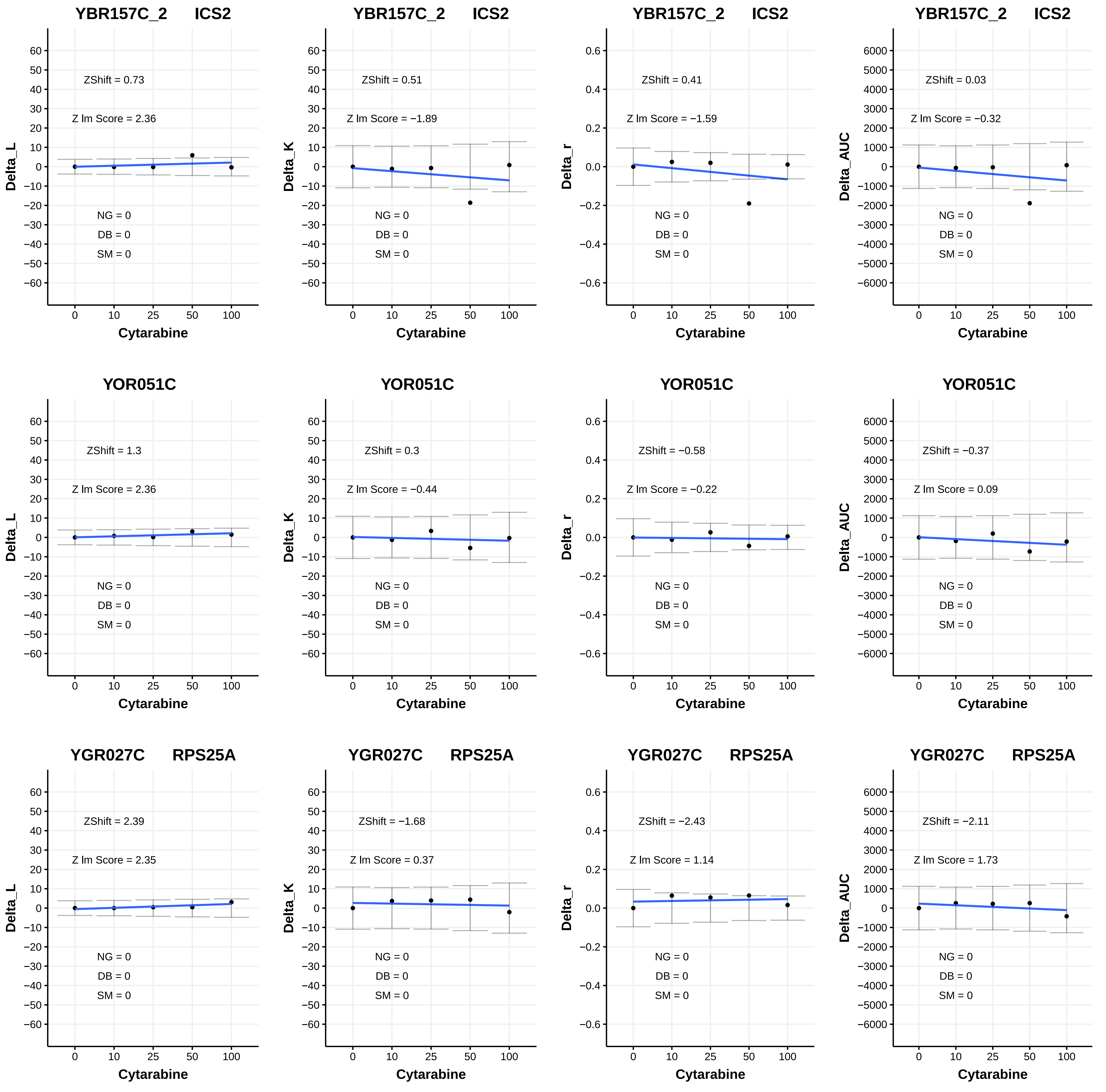
YBR157C_2 ICS2
Delta_L
ZShift = 0.73
Z lm Score = 2.36
NG = 0
DB = 0
SM = 0
Cytarabine
YBR157C_2 ICS2
Delta_K
ZShift = 0.51
Z lm Score = −1.89
NG = 0
DB = 0
SM = 0
Cytarabine
YBR157C_2 ICS2
Delta_r
ZShift = 0.41
Z lm Score = −1.59
NG = 0
DB = 0
SM = 0
Cytarabine
YBR157C_2 ICS2
Delta_AUC
ZShift = 0.03
Z lm Score = −0.32
NG = 0
DB = 0
SM = 0
Cytarabine
YOR051C
Delta_L
ZShift = 1.3
Z lm Score = 2.36
NG = 0
DB = 0
SM = 0
Cytarabine
YOR051C
Delta_K
ZShift = 0.3
Z lm Score = −0.44
NG = 0
DB = 0
SM = 0
Cytarabine
YOR051C
Delta_r
ZShift = −0.58
Z lm Score = −0.22
NG = 0
DB = 0
SM = 0
Cytarabine
YOR051C
Delta_AUC
ZShift = −0.37
Z lm Score = 0.09
NG = 0
DB = 0
SM = 0
Cytarabine
YGR027C RPS25A
Delta_L
ZShift = 2.39
Z lm Score = 2.35
NG = 0
DB = 0
SM = 0
Cytarabine
YGR027C RPS25A
Delta_K
ZShift = −1.68
Z lm Score = 0.37
NG = 0
DB = 0
SM = 0
Cytarabine
YGR027C RPS25A
Delta_r
ZShift = −2.43
Z lm Score = 1.14
NG = 0
DB = 0
SM = 0
Cytarabine
YGR027C RPS25A
Delta_AUC
ZShift = −2.11
Z lm Score = 1.73
NG = 0
DB = 0
SM = 0
Cytarabine

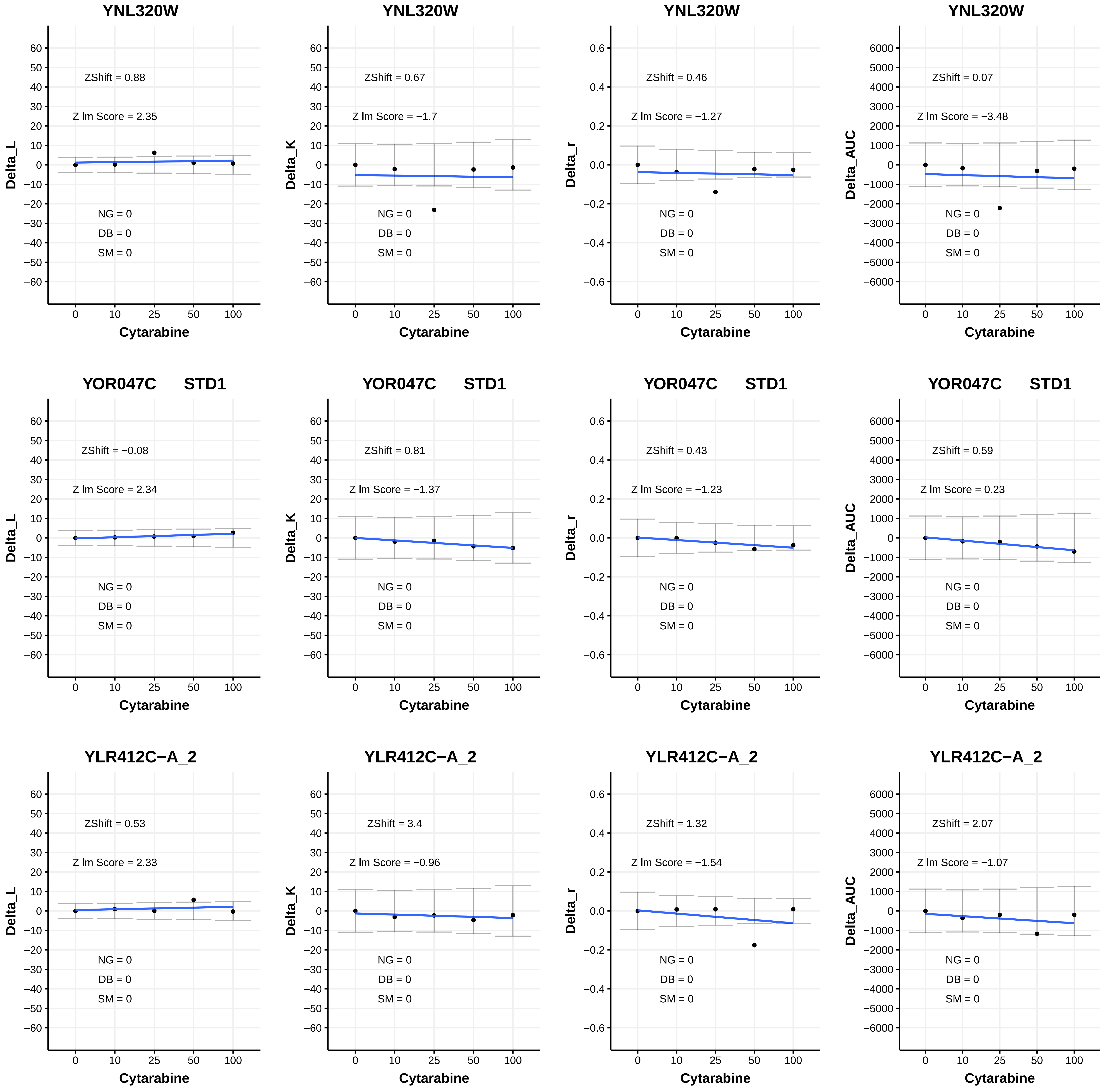
YNL320W
ZShift = 0.88
Z lm Score = 2.35
NG = 0
DB = 0
SM = 0
Delta_L
Cytarabine
YNL320W
ZShift = 0.67
Z lm Score = −1.7
NG = 0
DB = 0
SM = 0
Delta_K
Cytarabine
YNL320W
ZShift = 0.46
Z lm Score = −1.27
NG = 0
DB = 0
SM = 0
Delta_r
Cytarabine
YNL320W
ZShift = 0.07
Z lm Score = −3.48
NG = 0
DB = 0
SM = 0
Delta_AUC
Cytarabine
YOR047C STD1
ZShift = −0.08
Z lm Score = 2.34
NG = 0
DB = 0
SM = 0
Delta_L
Cytarabine
YOR047C STD1
ZShift = 0.81
Z lm Score = −1.37
NG = 0
DB = 0
SM = 0
Delta_K
Cytarabine
YOR047C STD1
ZShift = 0.43
Z lm Score = −1.23
NG = 0
DB = 0
SM = 0
Delta_r
Cytarabine
YOR047C STD1
ZShift = 0.59
Z lm Score = 0.23
NG = 0
DB = 0
SM = 0
Delta_AUC
Cytarabine
YLR412C−A_2
ZShift = 0.53
Z lm Score = 2.33
NG = 0
DB = 0
SM = 0
Delta_L
Cytarabine
YLR412C−A_2
ZShift = 3.4
Z lm Score = −0.96
NG = 0
DB = 0
SM = 0
Delta_K
Cytarabine
YLR412C−A_2
ZShift = 1.32
Z lm Score = −1.54
NG = 0
DB = 0
SM = 0
Delta_r
Cytarabine
YLR412C−A_2
ZShift = 2.07
Z lm Score = −1.07
NG = 0
DB = 0
SM = 0
Delta_AUC
Cytarabine

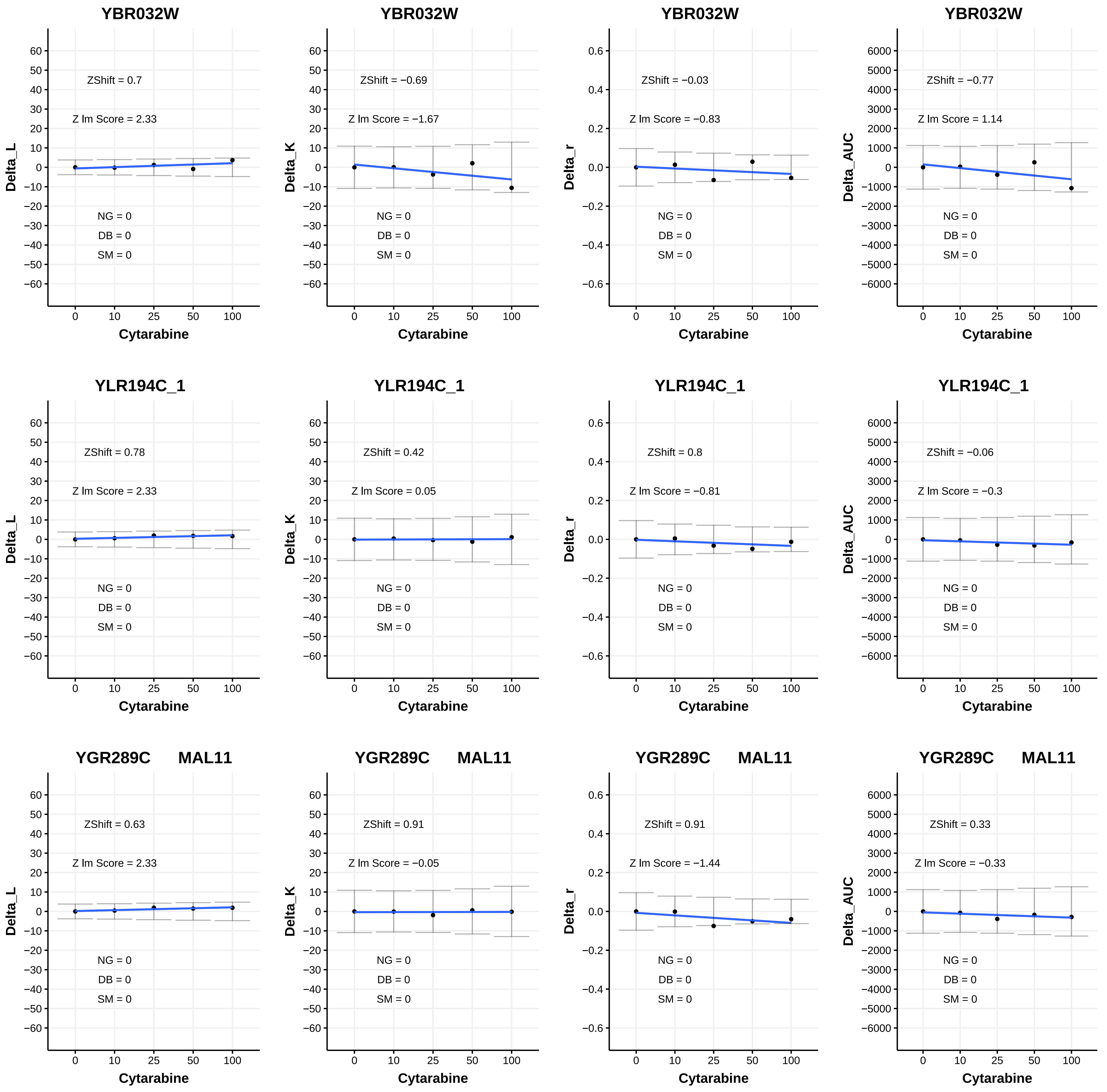

YBR032W
ZShift = 0.7
Z lm Score = 2.33
NG = 0
DB = 0
SM = 0
Delta_L
Cytarabine
YBR032W
ZShift = −0.69
Z lm Score = −1.67
NG = 0
DB = 0
SM = 0
Delta_K
Cytarabine
YBR032W
ZShift = −0.03
Z lm Score = −0.83
NG = 0
DB = 0
SM = 0
Delta_r
Cytarabine
YBR032W
ZShift = −0.77
Z lm Score = 1.14
NG = 0
DB = 0
SM = 0
Delta_AUC
Cytarabine
YLR194C_1
ZShift = 0.78
Z lm Score = 2.33
NG = 0
DB = 0
SM = 0
Delta_L
Cytarabine
YLR194C_1
ZShift = 0.42
Z lm Score = 0.05
NG = 0
DB = 0
SM = 0
Delta_K
Cytarabine
YLR194C_1
ZShift = 0.8
Z lm Score = −0.81
NG = 0
DB = 0
SM = 0
Delta_r
Cytarabine
YLR194C_1
ZShift = −0.06
Z lm Score = −0.3
NG = 0
DB = 0
SM = 0
Delta_AUC
Cytarabine
YGR289C MAL11
ZShift = 0.63
Z lm Score = 2.33
NG = 0
DB = 0
SM = 0
Delta_L
Cytarabine
YGR289C MAL11
ZShift = 0.91
Z lm Score = −0.05
NG = 0
DB = 0
SM = 0
Delta_K
Cytarabine
YGR289C MAL11
ZShift = 0.91
Z lm Score = −1.44
NG = 0
DB = 0
SM = 0
Delta_r
Cytarabine
YGR289C MAL11
ZShift = 0.33
Z lm Score = −0.33
NG = 0
DB = 0
SM = 0
Delta_AUC
Cytarabine

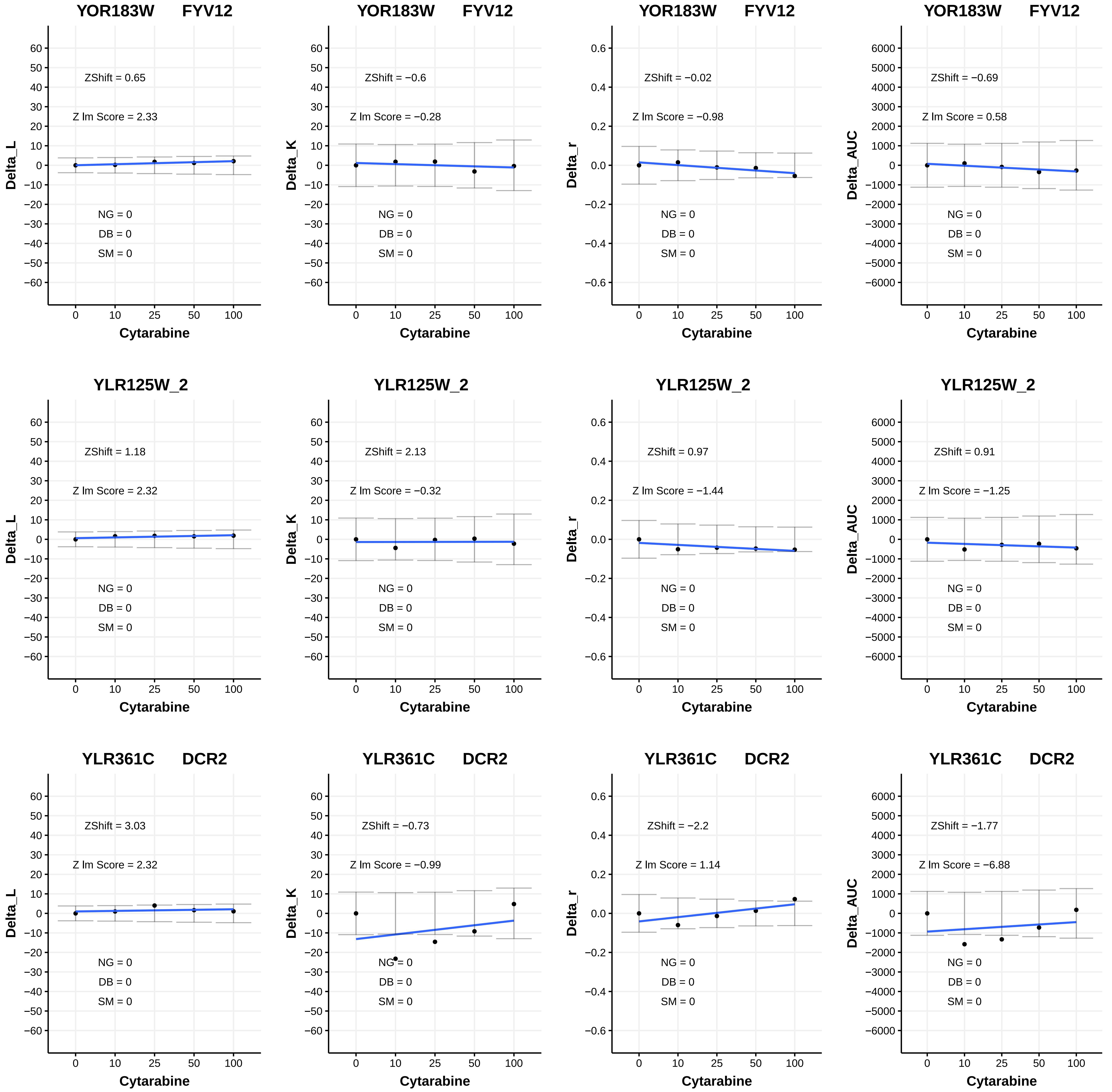
YOR183W FYV12
ZShift = 0.65
Z lm Score = 2.33
NG = 0
DB = 0
SM = 0
Delta_L
Cytarabine
YOR183W FYV12
ZShift = −0.6
Z lm Score = −0.28
NG = 0
DB = 0
SM = 0
Delta_K
Cytarabine
YOR183W FYV12
ZShift = −0.02
Z lm Score = −0.98
NG = 0
DB = 0
SM = 0
Delta_r
Cytarabine
YOR183W FYV12
ZShift = −0.69
Z lm Score = 0.58
NG = 0
DB = 0
SM = 0
Delta_AUC
Cytarabine
YLR125W_2
ZShift = 1.18
Z lm Score = 2.32
NG = 0
DB = 0
SM = 0
Delta_L
Cytarabine
YLR125W_2
ZShift = 2.13
Z lm Score = −0.32
NG = 0
DB = 0
SM = 0
Delta_K
Cytarabine
YLR125W_2
ZShift = 0.97
Z lm Score = −1.44
NG = 0
DB = 0
SM = 0
Delta_r
Cytarabine
YLR125W_2
ZShift = 0.91
Z lm Score = −1.25
NG = 0
DB = 0
SM = 0
Delta_AUC
Cytarabine
YLR361C DCR2
ZShift = 3.03
Z lm Score = 2.32
NG = 0
DB = 0
SM = 0
Delta_L
Cytarabine
YLR361C DCR2
ZShift = −0.73
Z lm Score = −0.99
NG = 0
DB = 0
SM = 0
Delta_K
Cytarabine
YLR361C DCR2
ZShift = −2.2
Z lm Score = 1.14
NG = 0
DB = 0
SM = 0
Delta_r
Cytarabine
YLR361C DCR2
ZShift = −1.77
Z lm Score = −6.88
NG = 0
DB = 0
SM = 0
Delta_AUC
Cytarabine

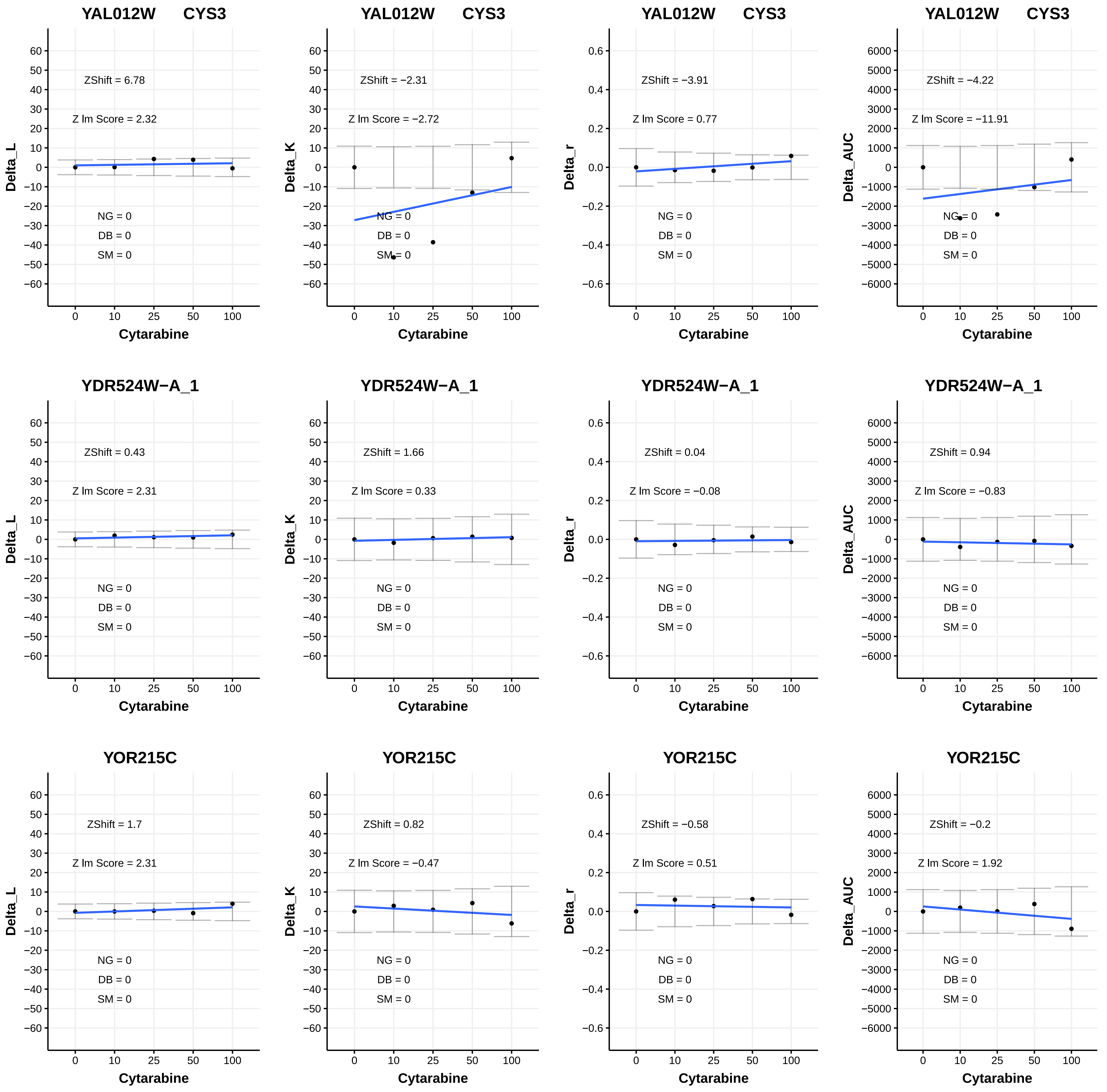

YAL012W CYS3
ZShift = 6.78
Z lm Score = 2.32
NG = 0
DB = 0
SM = 0
Delta_L
Cytarabine
YAL012W CYS3
ZShift = −2.31
Z lm Score = −2.72
NG = 0
DB = 0
SM = 0
Delta_K
Cytarabine
YAL012W CYS3
ZShift = −3.91
Z lm Score = 0.77
NG = 0
DB = 0
SM = 0
Delta_r
Cytarabine
YAL012W CYS3
ZShift = −4.22
Z lm Score = −11.91
NG = 0
DB = 0
SM = 0
Delta_AUC
Cytarabine
YDR524W−A_1
ZShift = 0.43
Z lm Score = 2.31
NG = 0
DB = 0
SM = 0
Delta_L
Cytarabine
YDR524W−A_1
ZShift = 1.66
Z lm Score = 0.33
NG = 0
DB = 0
SM = 0
Delta_K
Cytarabine
YDR524W−A_1
ZShift = 0.04
Z lm Score = −0.08
NG = 0
DB = 0
SM = 0
Delta_r
Cytarabine
YDR524W−A_1
ZShift = 0.94
Z lm Score = −0.83
NG = 0
DB = 0
SM = 0
Delta_AUC
Cytarabine
YOR215C
ZShift = 1.7
Z lm Score = 2.31
NG = 0
DB = 0
SM = 0
Delta_L
Cytarabine
YOR215C
ZShift = 0.82
Z lm Score = −0.47
NG = 0
DB = 0
SM = 0
Delta_K
Cytarabine
YOR215C
ZShift = −0.58
Z lm Score = 0.51
NG = 0
DB = 0
SM = 0
Delta_r
Cytarabine
YOR215C
ZShift = −0.2
Z lm Score = 1.92
NG = 0
DB = 0
SM = 0
Delta_AUC
Cytarabine

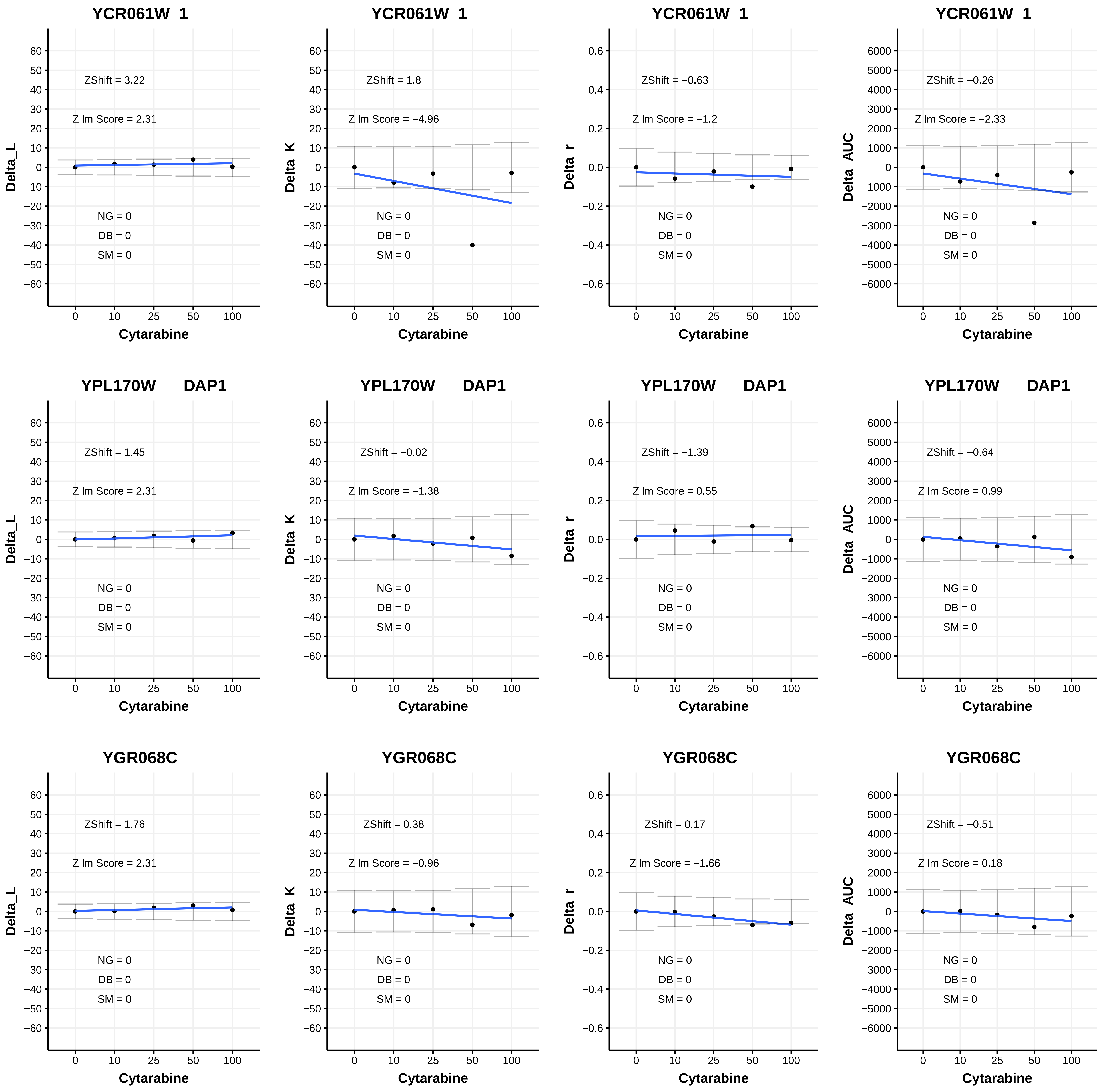

YCR061W_1
ZShift = 3.22
Z lm Score = 2.31
NG = 0
DB = 0
SM = 0
Delta_L
Cytarabine
YCR061W_1
ZShift = 1.8
Z lm Score = −4.96
NG = 0
DB = 0
SM = 0
Delta_K
Cytarabine
YCR061W_1
ZShift = −0.63
Z lm Score = −1.2
NG = 0
DB = 0
SM = 0
Delta_r
Cytarabine
YCR061W_1
ZShift = −0.26
Z lm Score = −2.33
NG = 0
DB = 0
SM = 0
Delta_AUC
Cytarabine
YPL170W DAP1
ZShift = 1.45
Z lm Score = 2.31
NG = 0
DB = 0
SM = 0
Delta_L
Cytarabine
YPL170W DAP1
ZShift = −0.02
Z lm Score = −1.38
NG = 0
DB = 0
SM = 0
Delta_K
Cytarabine
YPL170W DAP1
ZShift = −1.39
Z lm Score = 0.55
NG = 0
DB = 0
SM = 0
Delta_r
Cytarabine
YPL170W DAP1
ZShift = −0.64
Z lm Score = 0.99
NG = 0
DB = 0
SM = 0
Delta_AUC
Cytarabine
YGR068C
ZShift = 1.76
Z lm Score = 2.31
NG = 0
DB = 0
SM = 0
Delta_L
Cytarabine
YGR068C
ZShift = 0.38
Z lm Score = −0.96
NG = 0
DB = 0
SM = 0
Delta_K
Cytarabine
YGR068C
ZShift = 0.17
Z lm Score = −1.66
NG = 0
DB = 0
SM = 0
Delta_r
Cytarabine
YGR068C
ZShift = −0.51
Z lm Score = 0.18
NG = 0
DB = 0
SM = 0
Delta_AUC
Cytarabine

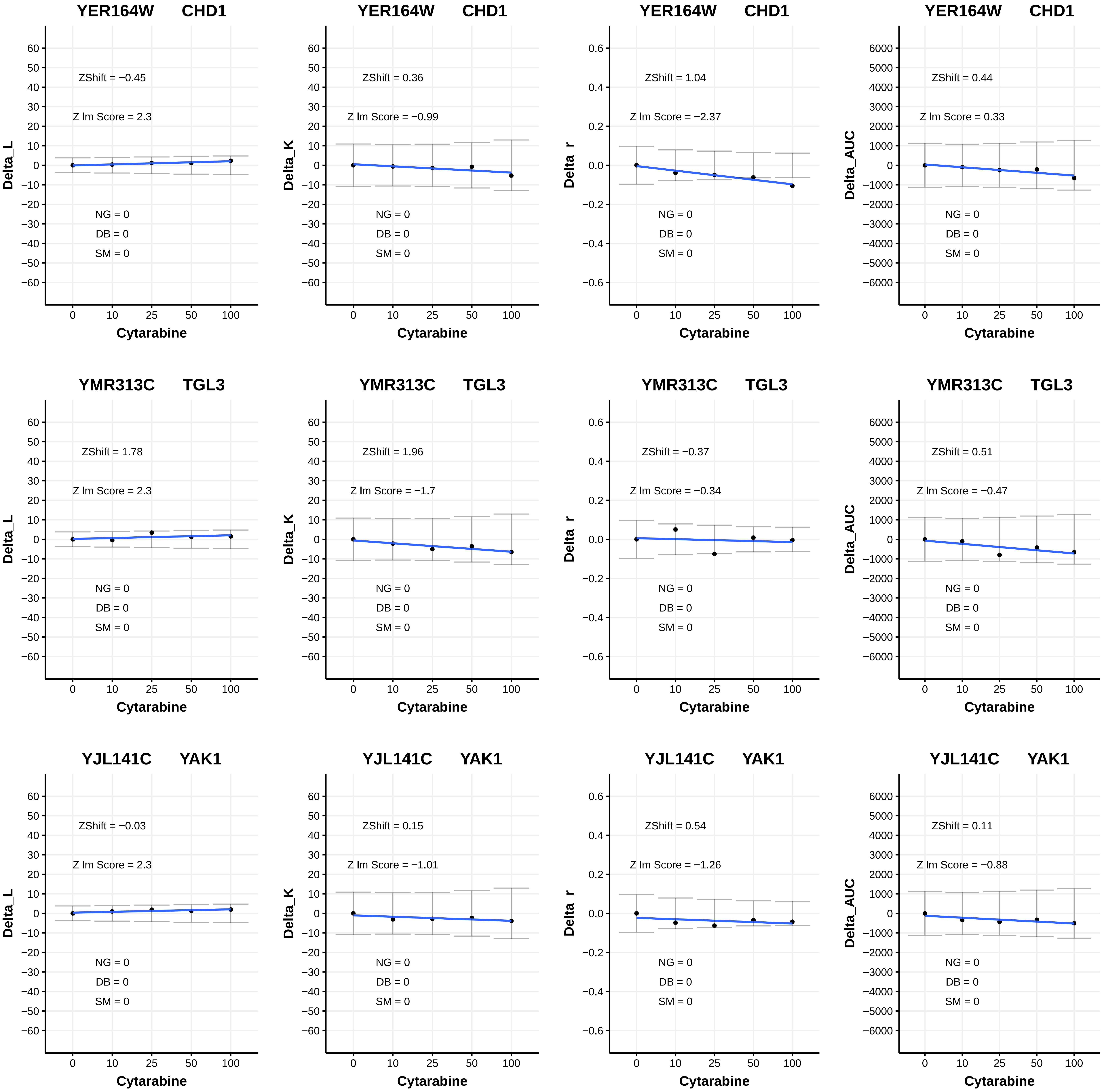

YER164W CHD1
ZShift = −0.45
Z lm Score = 2.3
Delta_L
NG = 0
DB = 0
SM = 0
Cytarabine
YER164W CHD1
ZShift = 0.36
Z lm Score = −0.99
Delta_K
NG = 0
DB = 0
SM = 0
Cytarabine
YER164W CHD1
ZShift = 1.04
Z lm Score = −2.37
Delta_r
NG = 0
DB = 0
SM = 0
Cytarabine
YER164W CHD1
ZShift = 0.44
Z lm Score = 0.33
Delta_AUC
NG = 0
DB = 0
SM = 0
Cytarabine
YMR313C TGL3
ZShift = 1.78
Z lm Score = 2.3
Delta_L
NG = 0
DB = 0
SM = 0
Cytarabine
YMR313C TGL3
ZShift = 1.96
Z lm Score = −1.7
Delta_K
NG = 0
DB = 0
SM = 0
Cytarabine
YMR313C TGL3
ZShift = −0.37
Z lm Score = −0.34
Delta_r
NG = 0
DB = 0
SM = 0
Cytarabine
YMR313C TGL3
ZShift = 0.51
Z lm Score = −0.47
Delta_AUC
NG = 0
DB = 0
SM = 0
Cytarabine
YJL141C YAK1
ZShift = −0.03
Z lm Score = 2.3
Delta_L
NG = 0
DB = 0
SM = 0
Cytarabine
YJL141C YAK1
ZShift = 0.15
Z lm Score = −1.01
Delta_K
NG = 0
DB = 0
SM = 0
Cytarabine
YJL141C YAK1
ZShift = 0.54
Z lm Score = −1.26
Delta_r
NG = 0
DB = 0
SM = 0
Cytarabine
YJL141C YAK1
ZShift = 0.11
Z lm Score = −0.88
Delta_AUC
NG = 0
DB = 0
SM = 0
Cytarabine

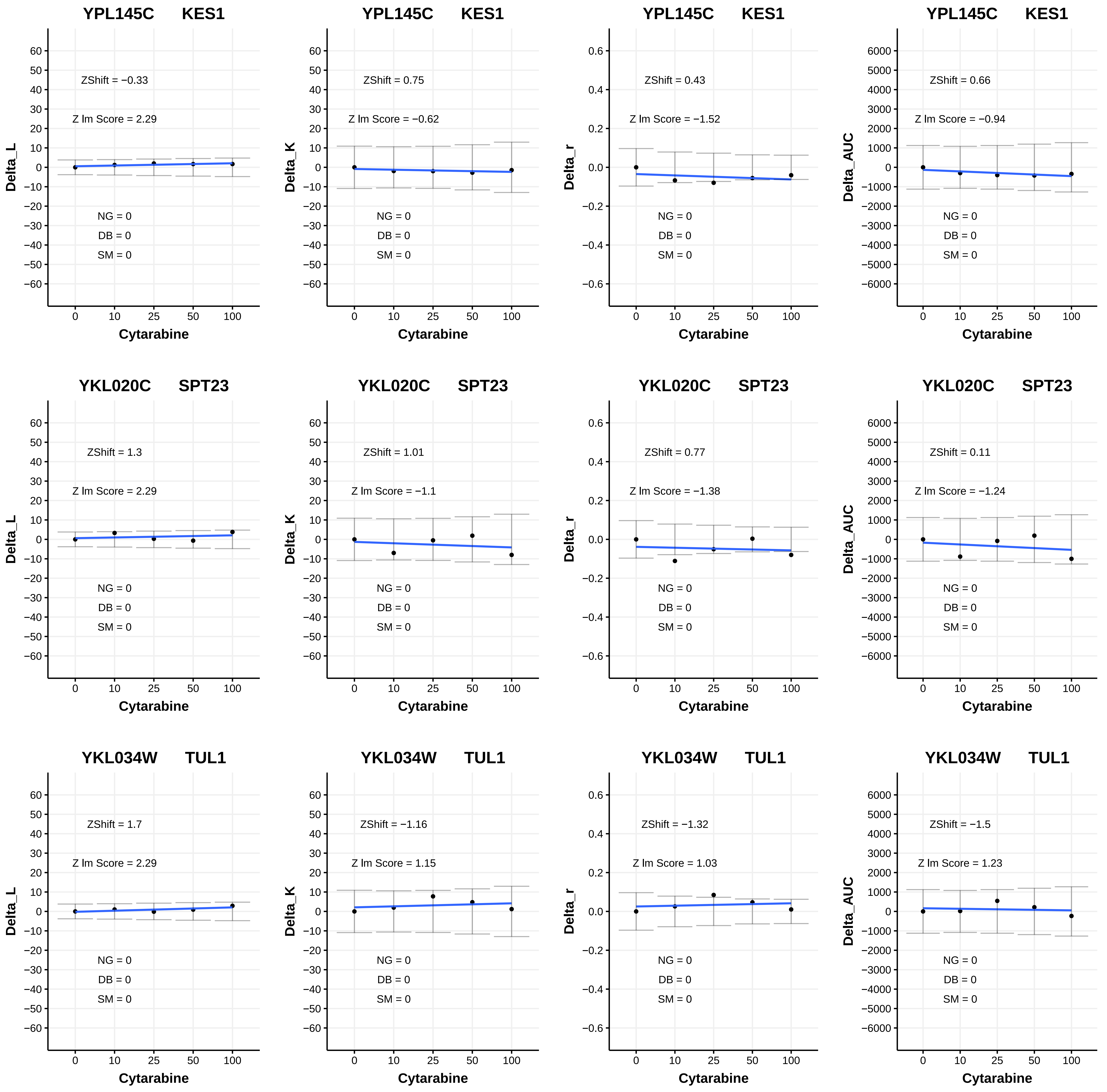

YPL145C KES1
ZShift = −0.33
Z lm Score = 2.29
NG = 0
DB = 0
SM = 0
Delta_L
Cytarabine
YPL145C KES1
ZShift = 0.75
Z lm Score = −0.62
NG = 0
DB = 0
SM = 0
Delta_K
Cytarabine
YPL145C KES1
ZShift = 0.43
Z lm Score = −1.52
NG = 0
DB = 0
SM = 0
Delta_r
Cytarabine
YPL145C KES1
ZShift = 0.66
Z lm Score = −0.94
NG = 0
DB = 0
SM = 0
Delta_AUC
Cytarabine
YKL020C SPT23
ZShift = 1.3
Z lm Score = 2.29
NG = 0
DB = 0
SM = 0
Delta_L
Cytarabine
YKL020C SPT23
ZShift = 1.01
Z lm Score = −1.1
NG = 0
DB = 0
SM = 0
Delta_K
Cytarabine
YKL020C SPT23
ZShift = 0.77
Z lm Score = −1.38
NG = 0
DB = 0
SM = 0
Delta_r
Cytarabine
YKL020C SPT23
ZShift = 0.11
Z lm Score = −1.24
NG = 0
DB = 0
SM = 0
Delta_AUC
Cytarabine
YKL034W TUL1
ZShift = 1.7
Z lm Score = 2.29
NG = 0
DB = 0
SM = 0
Delta_L
Cytarabine
YKL034W TUL1
ZShift = −1.16
Z lm Score = 1.15
NG = 0
DB = 0
SM = 0
Delta_K
Cytarabine
YKL034W TUL1
ZShift = −1.32
Z lm Score = 1.03
NG = 0
DB = 0
SM = 0
Delta_r
Cytarabine
YKL034W TUL1
ZShift = −1.5
Z lm Score = 1.23
NG = 0
DB = 0
SM = 0
Delta_AUC
Cytarabine

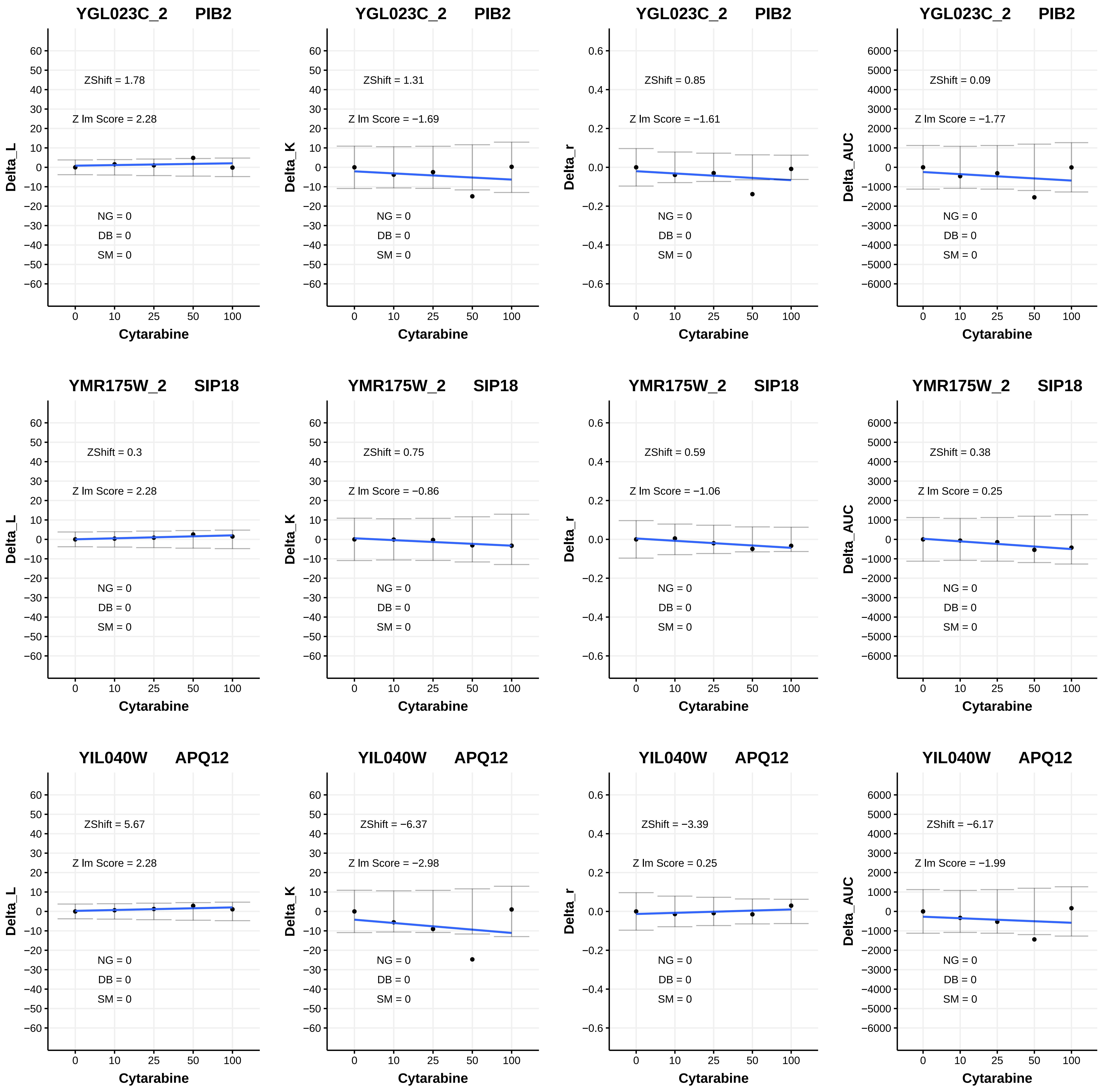
YGL023C_2 PIB2
ZShift = 1.78
Z lm Score = 2.28
NG = 0
DB = 0
SM = 0
Delta_L
Cytarabine
YGL023C_2 PIB2
ZShift = 1.31
Z lm Score = −1.69
NG = 0
DB = 0
SM = 0
Delta_K
Cytarabine
YGL023C_2 PIB2
ZShift = 0.85
Z lm Score = −1.61
NG = 0
DB = 0
SM = 0
Delta_r
Cytarabine
YGL023C_2 PIB2
ZShift = 0.09
Z lm Score = −1.77
NG = 0
DB = 0
SM = 0
Delta_AUC
Cytarabine
YMR175W_2 SIP18
ZShift = 0.3
Z lm Score = 2.28
NG = 0
DB = 0
SM = 0
Delta_L
Cytarabine
YMR175W_2 SIP18
ZShift = 0.75
Z lm Score = −0.86
NG = 0
DB = 0
SM = 0
Delta_K
Cytarabine
YMR175W_2 SIP18
ZShift = 0.59
Z lm Score = −1.06
NG = 0
DB = 0
SM = 0
Delta_r
Cytarabine
YMR175W_2 SIP18
ZShift = 0.38
Z lm Score = 0.25
NG = 0
DB = 0
SM = 0
Delta_AUC
Cytarabine
YIL040W APQ12
ZShift = 5.67
Z lm Score = 2.28
NG = 0
DB = 0
SM = 0
Delta_L
Cytarabine
YIL040W APQ12
ZShift = −6.37
Z lm Score = −2.98
NG = 0
DB = 0
SM = 0
Delta_K
Cytarabine
YIL040W APQ12
ZShift = −3.39
Z lm Score = 0.25
NG = 0
DB = 0
SM = 0
Delta_r
Cytarabine
YIL040W APQ12
ZShift = −6.17
Z lm Score = −1.99
NG = 0
DB = 0
SM = 0
Delta_AUC
Cytarabine

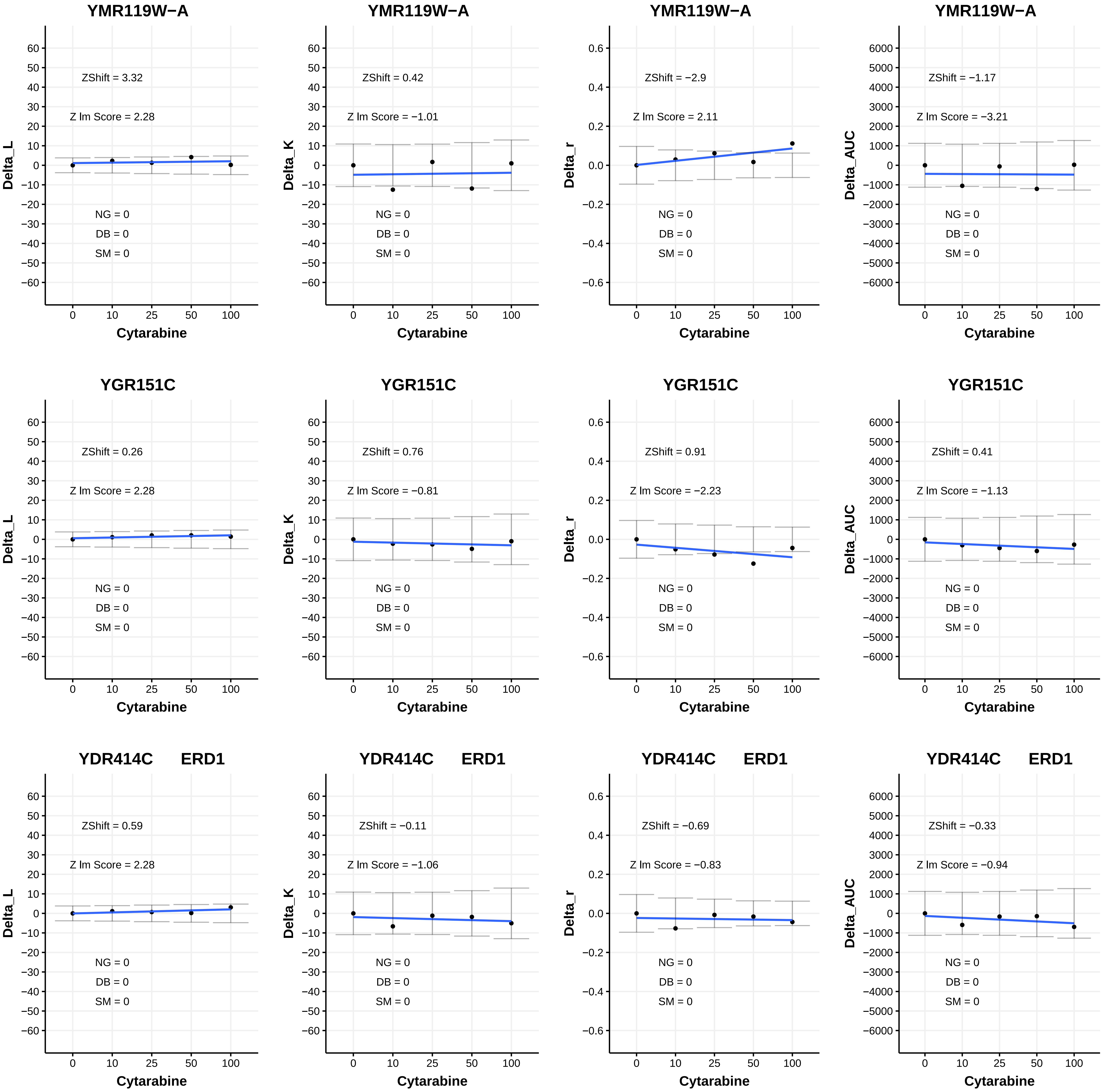
YMR119W−A
ZShift = 3.32
Z lm Score = 2.28
NG = 0
DB = 0
SM = 0
Delta_L
Cytarabine
YMR119W−A
ZShift = 0.42
Z lm Score = −1.01
NG = 0
DB = 0
SM = 0
Delta_K
Cytarabine
YMR119W−A
ZShift = −2.9
Z lm Score = 2.11
NG = 0
DB = 0
SM = 0
Delta_r
Cytarabine
YMR119W−A
ZShift = −1.17
Z lm Score = −3.21
NG = 0
DB = 0
SM = 0
Delta_AUC
Cytarabine
YGR151C
ZShift = 0.26
Z lm Score = 2.28
NG = 0
DB = 0
SM = 0
Delta_L
Cytarabine
YGR151C
ZShift = 0.76
Z lm Score = −0.81
NG = 0
DB = 0
SM = 0
Delta_K
Cytarabine
YGR151C
ZShift = 0.91
Z lm Score = −2.23
NG = 0
DB = 0
SM = 0
Delta_r
Cytarabine
YGR151C
ZShift = 0.41
Z lm Score = −1.13
NG = 0
DB = 0
SM = 0
Delta_AUC
Cytarabine
YDR414C ERD1
ZShift = 0.59
Z lm Score = 2.28
NG = 0
DB = 0
SM = 0
Delta_L
Cytarabine
YDR414C ERD1
ZShift = −0.11
Z lm Score = −1.06
NG = 0
DB = 0
SM = 0
Delta_K
Cytarabine
YDR414C ERD1
ZShift = −0.69
Z lm Score = −0.83
NG = 0
DB = 0
SM = 0
Delta_r
Cytarabine
YDR414C ERD1
ZShift = −0.33
Z lm Score = −0.94
NG = 0
DB = 0
SM = 0
Delta_AUC
Cytarabine

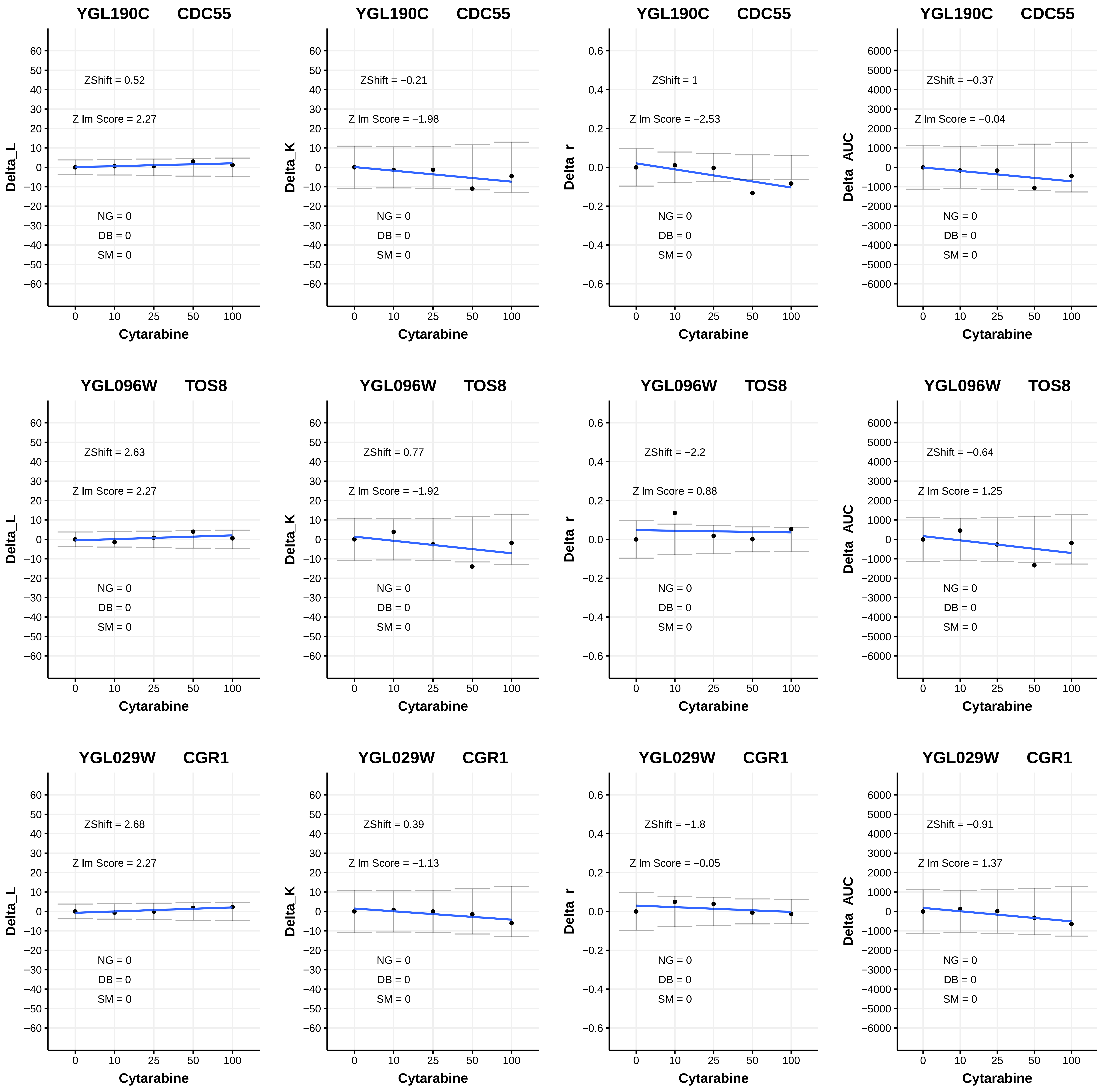

YGL190C CDC55
ZShift = 0.52
Z lm Score = 2.27
NG = 0
DB = 0
SM = 0
Delta_L
Cytarabine
YGL190C CDC55
ZShift = −0.21
Z lm Score = −1.98
NG = 0
DB = 0
SM = 0
Delta_K
Cytarabine
YGL190C CDC55
ZShift = 1
Z lm Score = −2.53
NG = 0
DB = 0
SM = 0
Delta_r
Cytarabine
YGL190C CDC55
ZShift = −0.37
Z lm Score = −0.04
NG = 0
DB = 0
SM = 0
Delta_AUC
Cytarabine
YGL096W TOS8
ZShift = 2.63
Z lm Score = 2.27
NG = 0
DB = 0
SM = 0
Delta_L
Cytarabine
YGL096W TOS8
ZShift = 0.77
Z lm Score = −1.92
NG = 0
DB = 0
SM = 0
Delta_K
Cytarabine
YGL096W TOS8
ZShift = −2.2
Z lm Score = 0.88
NG = 0
DB = 0
SM = 0
Delta_r
Cytarabine
YGL096W TOS8
ZShift = −0.64
Z lm Score = 1.25
NG = 0
DB = 0
SM = 0
Delta_AUC
Cytarabine
YGL029W CGR1
ZShift = 2.68
Z lm Score = 2.27
NG = 0
DB = 0
SM = 0
Delta_L
Cytarabine
YGL029W CGR1
ZShift = 0.39
Z lm Score = −1.13
NG = 0
DB = 0
SM = 0
Delta_K
Cytarabine
YGL029W CGR1
ZShift = −1.8
Z lm Score = −0.05
NG = 0
DB = 0
SM = 0
Delta_r
Cytarabine
YGL029W CGR1
ZShift = −0.91
Z lm Score = 1.37
NG = 0
DB = 0
SM = 0
Delta_AUC
Cytarabine

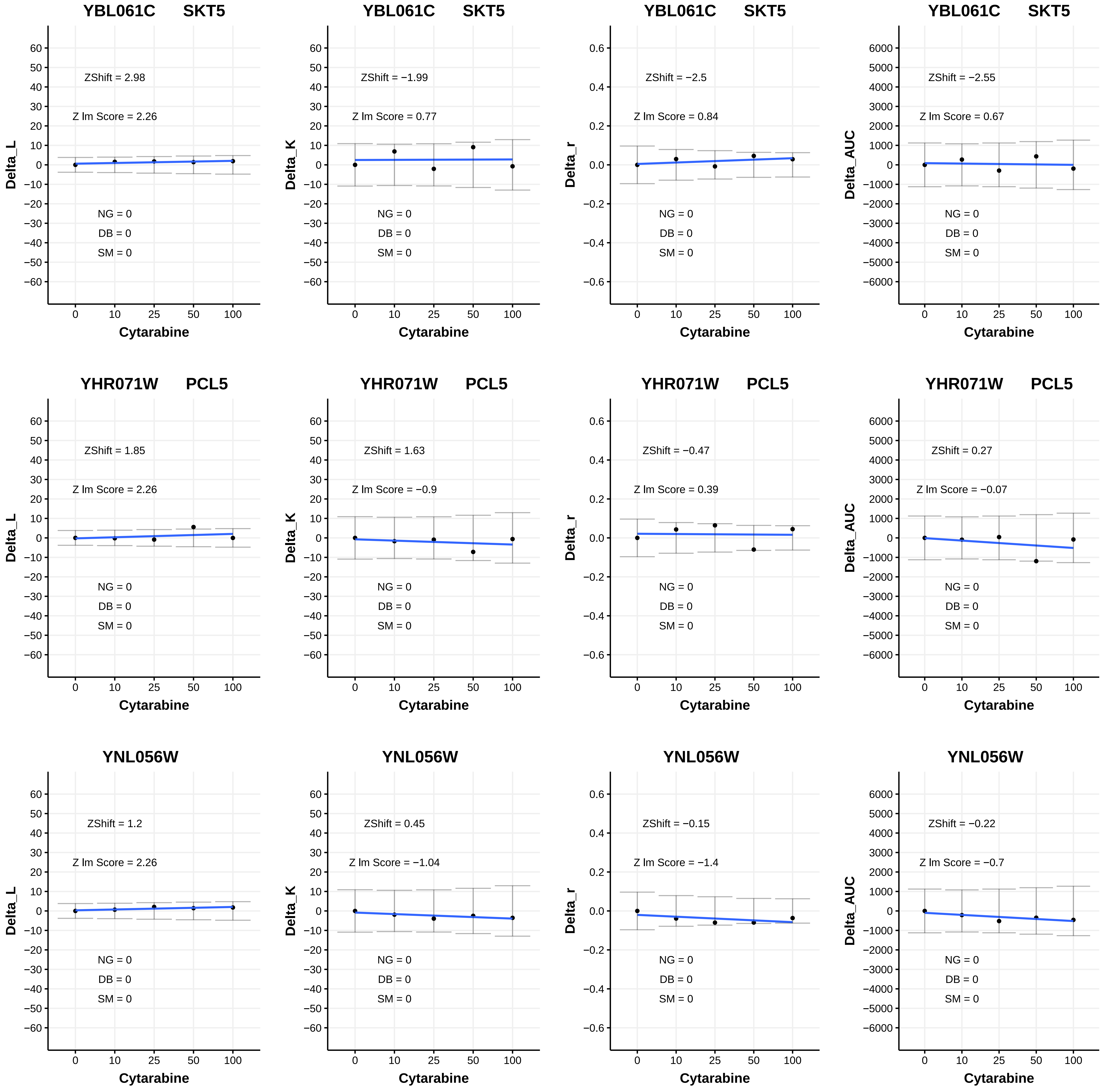
YBL061C SKT5
ZShift = 2.98
Z lm Score = 2.26
NG = 0
DB = 0
SM = 0
Delta_L
Cytarabine
YBL061C SKT5
ZShift = −1.99
Z lm Score = 0.77
NG = 0
DB = 0
SM = 0
Delta_K
Cytarabine
YBL061C SKT5
ZShift = −2.5
Z lm Score = 0.84
NG = 0
DB = 0
SM = 0
Delta_r
Cytarabine
YBL061C SKT5
ZShift = −2.55
Z lm Score = 0.67
NG = 0
DB = 0
SM = 0
Delta_AUC
Cytarabine
YHR071W PCL5
ZShift = 1.85
Z lm Score = 2.26
NG = 0
DB = 0
SM = 0
Delta_L
Cytarabine
YHR071W PCL5
ZShift = 1.63
Z lm Score = −0.9
NG = 0
DB = 0
SM = 0
Delta_K
Cytarabine
YHR071W PCL5
ZShift = −0.47
Z lm Score = 0.39
NG = 0
DB = 0
SM = 0
Delta_r
Cytarabine
YHR071W PCL5
ZShift = 0.27
Z lm Score = −0.07
NG = 0
DB = 0
SM = 0
Delta_AUC
Cytarabine
YNL056W
ZShift = 1.2
Z lm Score = 2.26
NG = 0
DB = 0
SM = 0
Delta_L
Cytarabine
YNL056W
ZShift = 0.45
Z lm Score = −1.04
NG = 0
DB = 0
SM = 0
Delta_K
Cytarabine
YNL056W
ZShift = −0.15
Z lm Score = −1.4
NG = 0
DB = 0
SM = 0
Delta_r
Cytarabine
YNL056W
ZShift = −0.22
Z lm Score = −0.7
NG = 0
DB = 0
SM = 0
Delta_AUC
Cytarabine

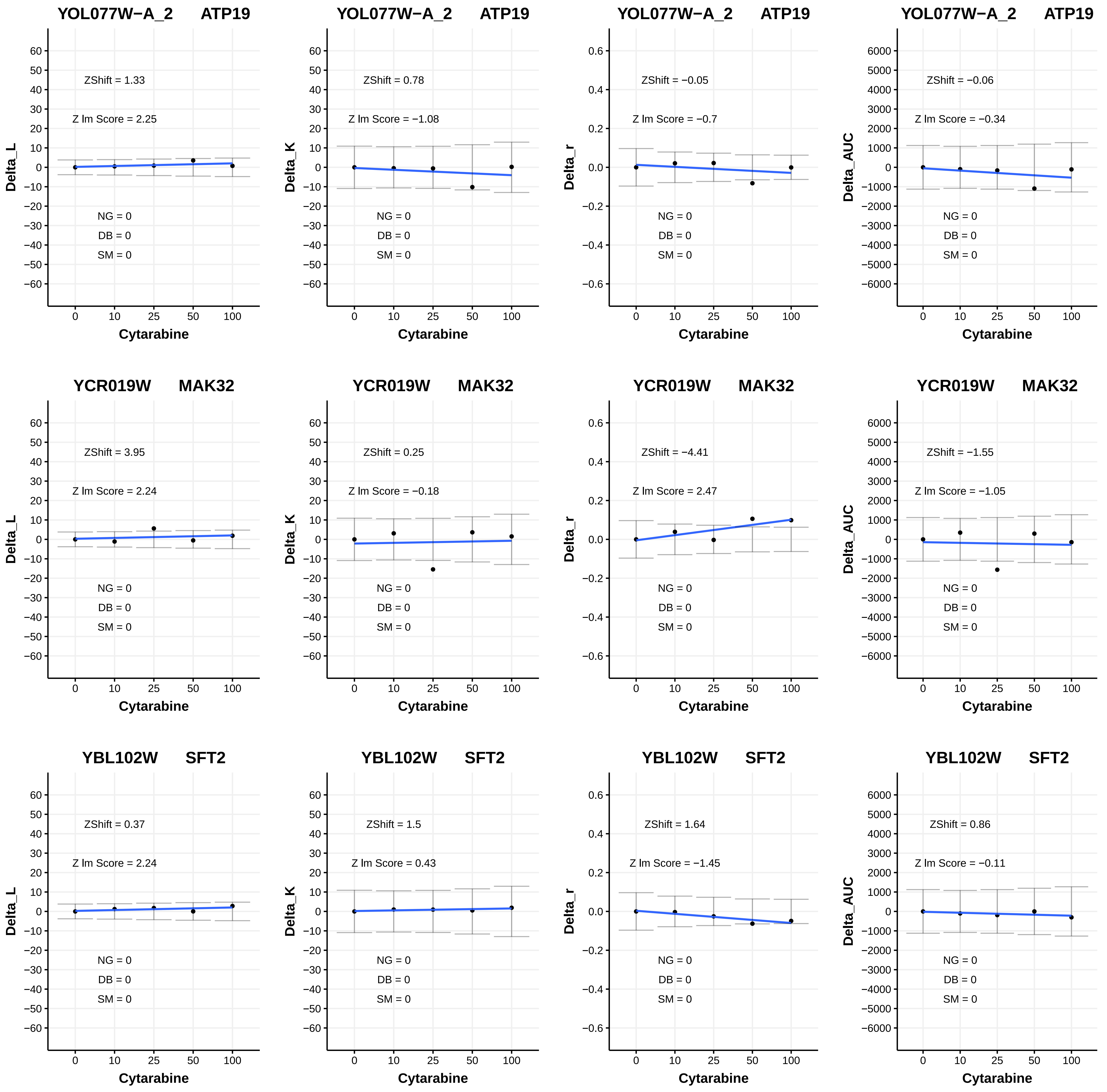

YOL077W−A_2 ATP19
ZShift = 1.33
Z lm Score = 2.25
NG = 0
DB = 0
SM = 0
Delta_L
Cytarabine
YOL077W−A_2 ATP19
ZShift = 0.78
Z lm Score = −1.08
NG = 0
DB = 0
SM = 0
Delta_K
Cytarabine
YOL077W−A_2 ATP19
ZShift = −0.05
Z lm Score = −0.7
NG = 0
DB = 0
SM = 0
Delta_r
Cytarabine
YOL077W−A_2 ATP19
ZShift = −0.06
Z lm Score = −0.34
NG = 0
DB = 0
SM = 0
Delta_AUC
Cytarabine
YCR019W MAK32
ZShift = 3.95
Z lm Score = 2.24
NG = 0
DB = 0
SM = 0
Delta_L
Cytarabine
YCR019W MAK32
ZShift = 0.25
Z lm Score = −0.18
NG = 0
DB = 0
SM = 0
Delta_K
Cytarabine
YCR019W MAK32
ZShift = −4.41
Z lm Score = 2.47
NG = 0
DB = 0
SM = 0
Delta_r
Cytarabine
YCR019W MAK32
ZShift = −1.55
Z lm Score = −1.05
NG = 0
DB = 0
SM = 0
Delta_AUC
Cytarabine
YBL102W SFT2
ZShift = 0.37
Z lm Score = 2.24
NG = 0
DB = 0
SM = 0
Delta_L
Cytarabine
YBL102W SFT2
ZShift = 1.5
Z lm Score = 0.43
NG = 0
DB = 0
SM = 0
Delta_K
Cytarabine
YBL102W SFT2
ZShift = 1.64
Z lm Score = −1.45
NG = 0
DB = 0
SM = 0
Delta_r
Cytarabine
YBL102W SFT2
ZShift = 0.86
Z lm Score = −0.11
NG = 0
DB = 0
SM = 0
Delta_AUC
Cytarabine

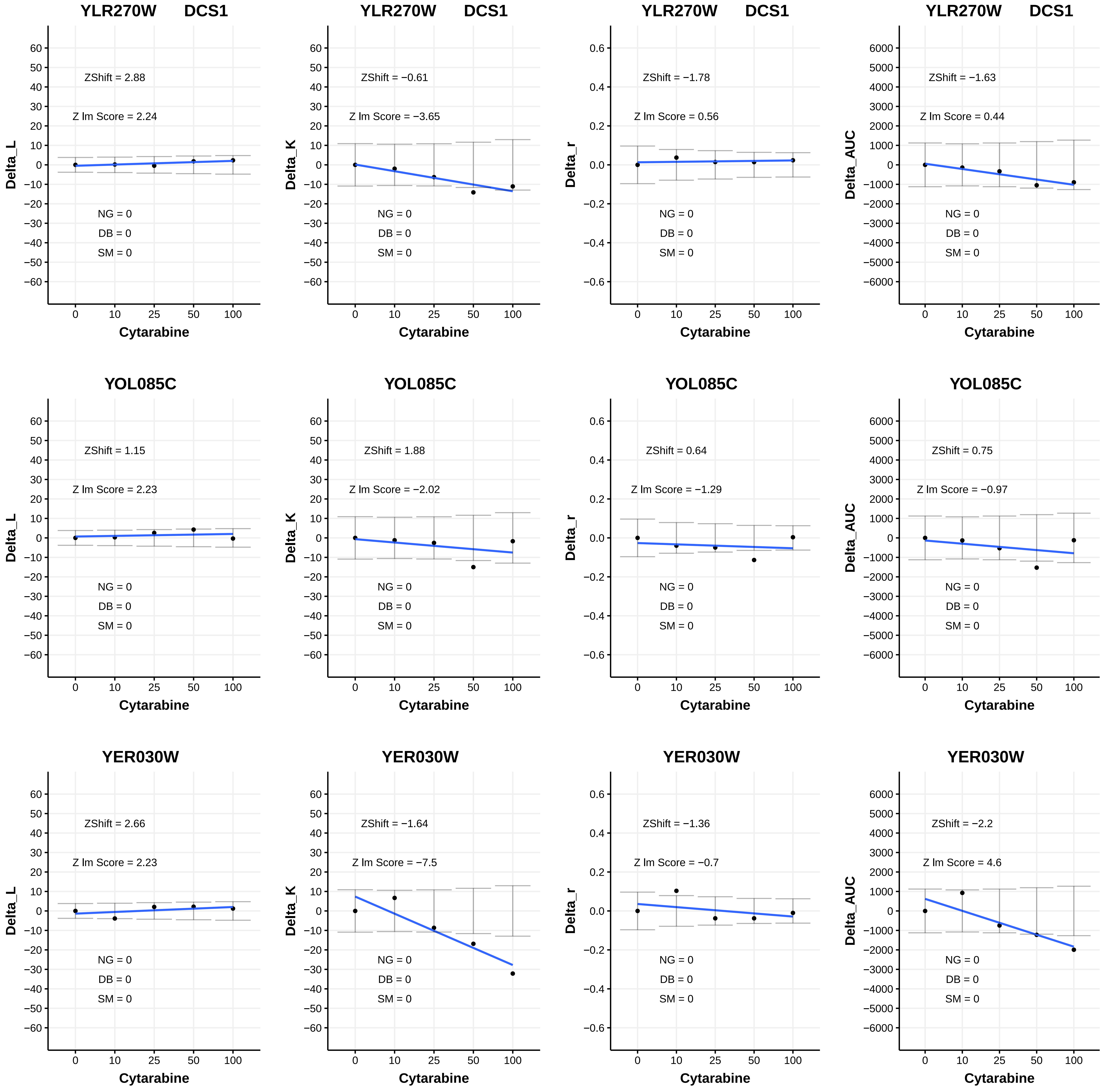
YLR270W DCS1
ZShift = 2.88
Z lm Score = 2.24
NG = 0
DB = 0
SM = 0
Delta_L
Cytarabine
YLR270W DCS1
ZShift = −0.61
Z lm Score = −3.65
NG = 0
DB = 0
SM = 0
Delta_K
Cytarabine
YLR270W DCS1
ZShift = −1.78
Z lm Score = 0.56
NG = 0
DB = 0
SM = 0
Delta_r
Cytarabine
YLR270W DCS1
ZShift = −1.63
Z lm Score = 0.44
NG = 0
DB = 0
SM = 0
Delta_AUC
Cytarabine
YOL085C
ZShift = 1.15
Z lm Score = 2.23
NG = 0
DB = 0
SM = 0
Delta_L
Cytarabine
YOL085C
ZShift = 1.88
Z lm Score = −2.02
NG = 0
DB = 0
SM = 0
Delta_K
Cytarabine
YOL085C
ZShift = 0.64
Z lm Score = −1.29
NG = 0
DB = 0
SM = 0
Delta_r
Cytarabine
YOL085C
ZShift = 0.75
Z lm Score = −0.97
NG = 0
DB = 0
SM = 0
Delta_AUC
Cytarabine
YER030W
ZShift = 2.66
Z lm Score = 2.23
NG = 0
DB = 0
SM = 0
Delta_L
Cytarabine
YER030W
ZShift = −1.64
Z lm Score = −7.5
NG = 0
DB = 0
SM = 0
Delta_K
Cytarabine
YER030W
ZShift = −1.36
Z lm Score = −0.7
NG = 0
DB = 0
SM = 0
Delta_r
Cytarabine
YER030W
ZShift = −2.2
Z lm Score = 4.6
NG = 0
DB = 0
SM = 0
Delta_AUC
Cytarabine

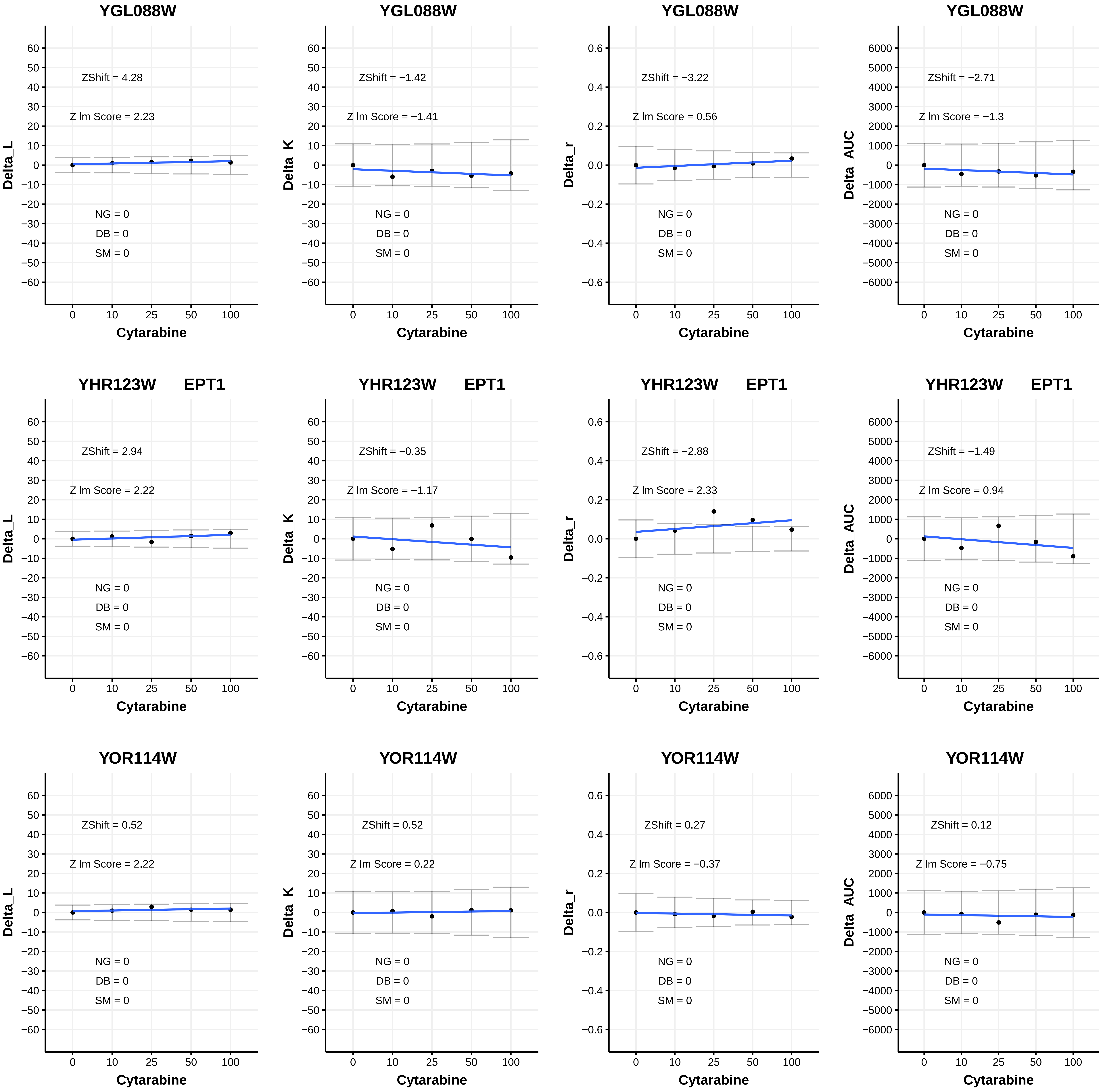

YGL088W
ZShift = 4.28
Z lm Score = 2.23
NG = 0
DB = 0
SM = 0
Delta_L
Cytarabine
YGL088W
ZShift = −1.42
Z lm Score = −1.41
NG = 0
DB = 0
SM = 0
Delta_K
Cytarabine
YGL088W
ZShift = −3.22
Z lm Score = 0.56
NG = 0
DB = 0
SM = 0
Delta_r
Cytarabine
YGL088W
ZShift = −2.71
Z lm Score = −1.3
NG = 0
DB = 0
SM = 0
Delta_AUC
Cytarabine
YHR123W EPT1
ZShift = 2.94
Z lm Score = 2.22
NG = 0
DB = 0
SM = 0
Delta_L
Cytarabine
YHR123W EPT1
ZShift = −0.35
Z lm Score = −1.17
NG = 0
DB = 0
SM = 0
Delta_K
Cytarabine
YHR123W EPT1
ZShift = −2.88
Z lm Score = 2.33
NG = 0
DB = 0
SM = 0
Delta_r
Cytarabine
YHR123W EPT1
ZShift = −1.49
Z lm Score = 0.94
NG = 0
DB = 0
SM = 0
Delta_AUC
Cytarabine
YOR114W
ZShift = 0.52
Z lm Score = 2.22
NG = 0
DB = 0
SM = 0
Delta_L
Cytarabine
YOR114W
ZShift = 0.52
Z lm Score = 0.22
NG = 0
DB = 0
SM = 0
Delta_K
Cytarabine
YOR114W
ZShift = 0.27
Z lm Score = −0.37
NG = 0
DB = 0
SM = 0
Delta_r
Cytarabine
YOR114W
ZShift = 0.12
Z lm Score = −0.75
NG = 0
DB = 0
SM = 0
Delta_AUC
Cytarabine

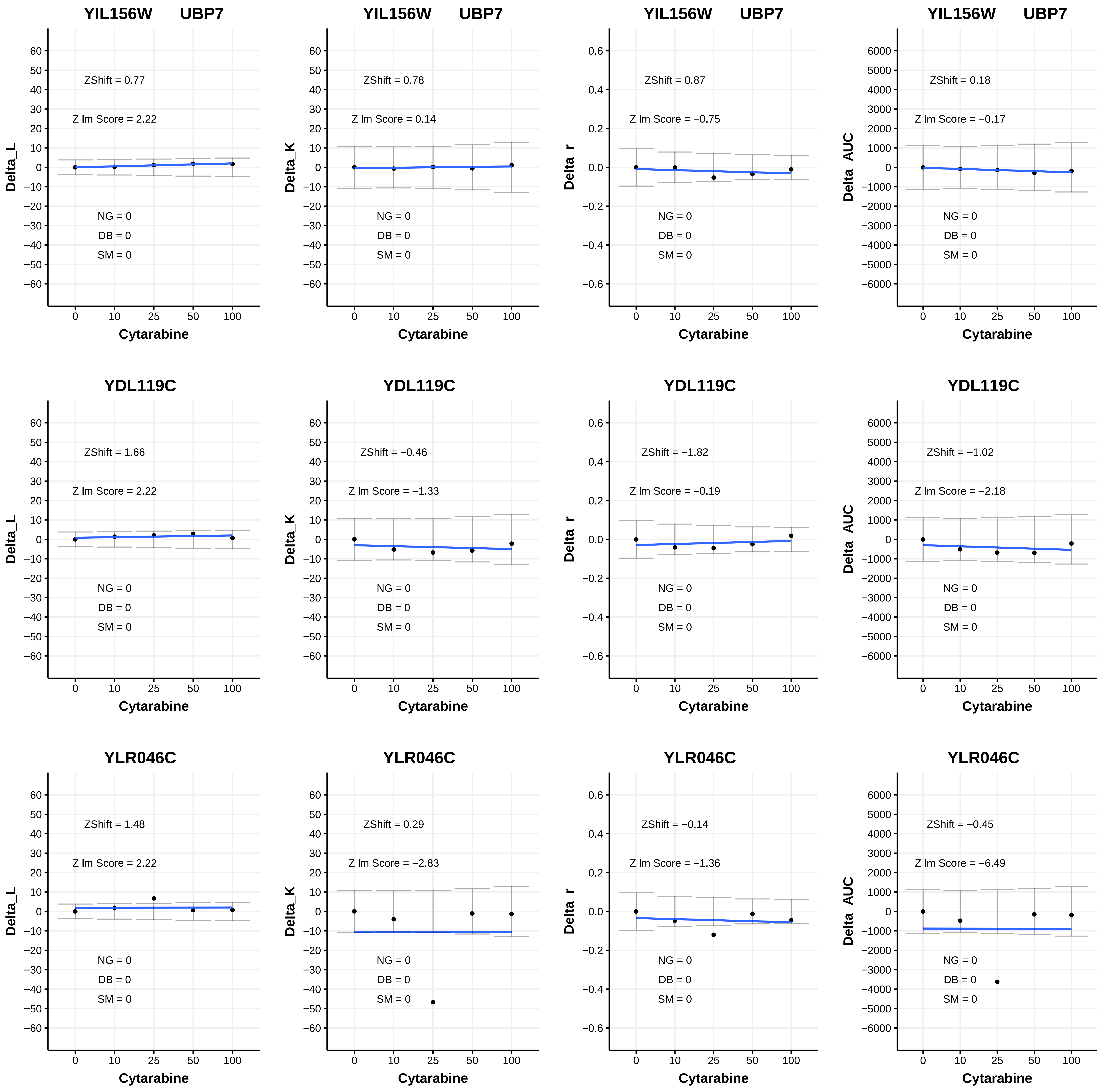

YIL156W UBP7
ZShift = 0.77
Z lm Score = 2.22
NG = 0
DB = 0
SM = 0
Delta_L
Cytarabine
YIL156W UBP7
ZShift = 0.78
Z lm Score = 0.14
NG = 0
DB = 0
SM = 0
Delta_K
Cytarabine
YIL156W UBP7
ZShift = 0.87
Z lm Score = −0.75
NG = 0
DB = 0
SM = 0
Delta_r
Cytarabine
YIL156W UBP7
ZShift = 0.18
Z lm Score = −0.17
NG = 0
DB = 0
SM = 0
Delta_AUC
Cytarabine
YDL119C
ZShift = 1.66
Z lm Score = 2.22
NG = 0
DB = 0
SM = 0
Delta_L
Cytarabine
YDL119C
ZShift = −0.46
Z lm Score = −1.33
NG = 0
DB = 0
SM = 0
Delta_K
Cytarabine
YDL119C
ZShift = −1.82
Z lm Score = −0.19
NG = 0
DB = 0
SM = 0
Delta_r
Cytarabine
YDL119C
ZShift = −1.02
Z lm Score = −2.18
NG = 0
DB = 0
SM = 0
Delta_AUC
Cytarabine
YLR046C
ZShift = 1.48
Z lm Score = 2.22
NG = 0
DB = 0
SM = 0
Delta_L
Cytarabine
YLR046C
ZShift = 0.29
Z lm Score = −2.83
NG = 0
DB = 0
SM = 0
Delta_K
Cytarabine
YLR046C
ZShift = −0.14
Z lm Score = −1.36
NG = 0
DB = 0
SM = 0
Delta_r
Cytarabine
YLR046C
ZShift = −0.45
Z lm Score = −6.49
NG = 0
DB = 0
SM = 0
Delta_AUC
Cytarabine

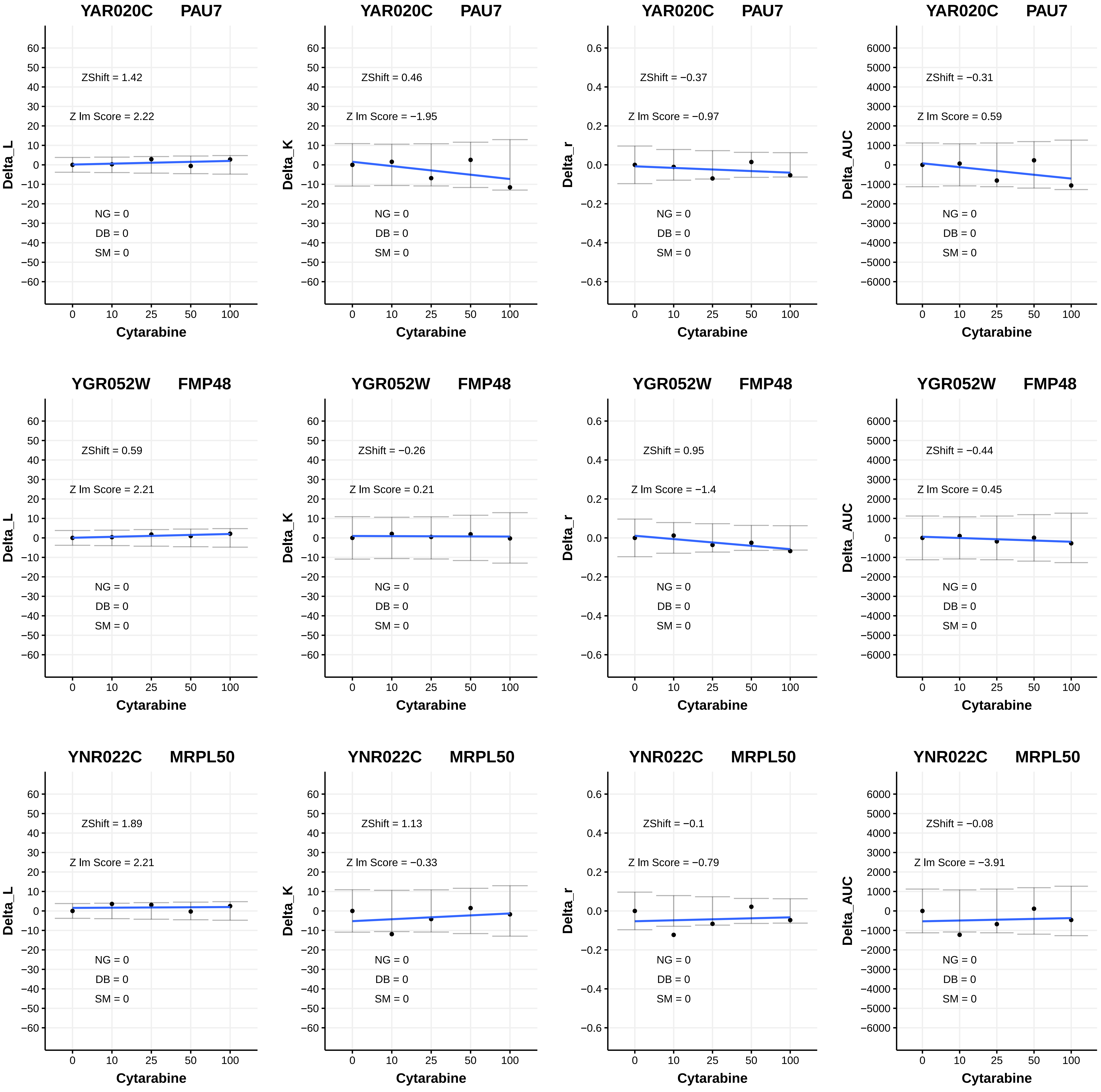

YAR020C PAU7
ZShift = 1.42
Z lm Score = 2.22
NG = 0
DB = 0
SM = 0
Delta_L
Cytarabine
YAR020C PAU7
ZShift = 0.46
Z lm Score = −1.95
NG = 0
DB = 0
SM = 0
Delta_K
Cytarabine
YAR020C PAU7
ZShift = −0.37
Z lm Score = −0.97
NG = 0
DB = 0
SM = 0
Delta_r
Cytarabine
YAR020C PAU7
ZShift = −0.31
Z lm Score = 0.59
NG = 0
DB = 0
SM = 0
Delta_AUC
Cytarabine
YGR052W FMP48
ZShift = 0.59
Z lm Score = 2.21
NG = 0
DB = 0
SM = 0
Delta_L
Cytarabine
YGR052W FMP48
ZShift = −0.26
Z lm Score = 0.21
NG = 0
DB = 0
SM = 0
Delta_K
Cytarabine
YGR052W FMP48
ZShift = 0.95
Z lm Score = −1.4
NG = 0
DB = 0
SM = 0
Delta_r
Cytarabine
YGR052W FMP48
ZShift = −0.44
Z lm Score = 0.45
NG = 0
DB = 0
SM = 0
Delta_AUC
Cytarabine
YNR022C MRPL50
ZShift = 1.89
Z lm Score = 2.21
NG = 0
DB = 0
SM = 0
Delta_L
Cytarabine
YNR022C MRPL50
ZShift = 1.13
Z lm Score = −0.33
NG = 0
DB = 0
SM = 0
Delta_K
Cytarabine
YNR022C MRPL50
ZShift = −0.1
Z lm Score = −0.79
NG = 0
DB = 0
SM = 0
Delta_r
Cytarabine
YNR022C MRPL50
ZShift = −0.08
Z lm Score = −3.91
NG = 0
DB = 0
SM = 0
Delta_AUC
Cytarabine

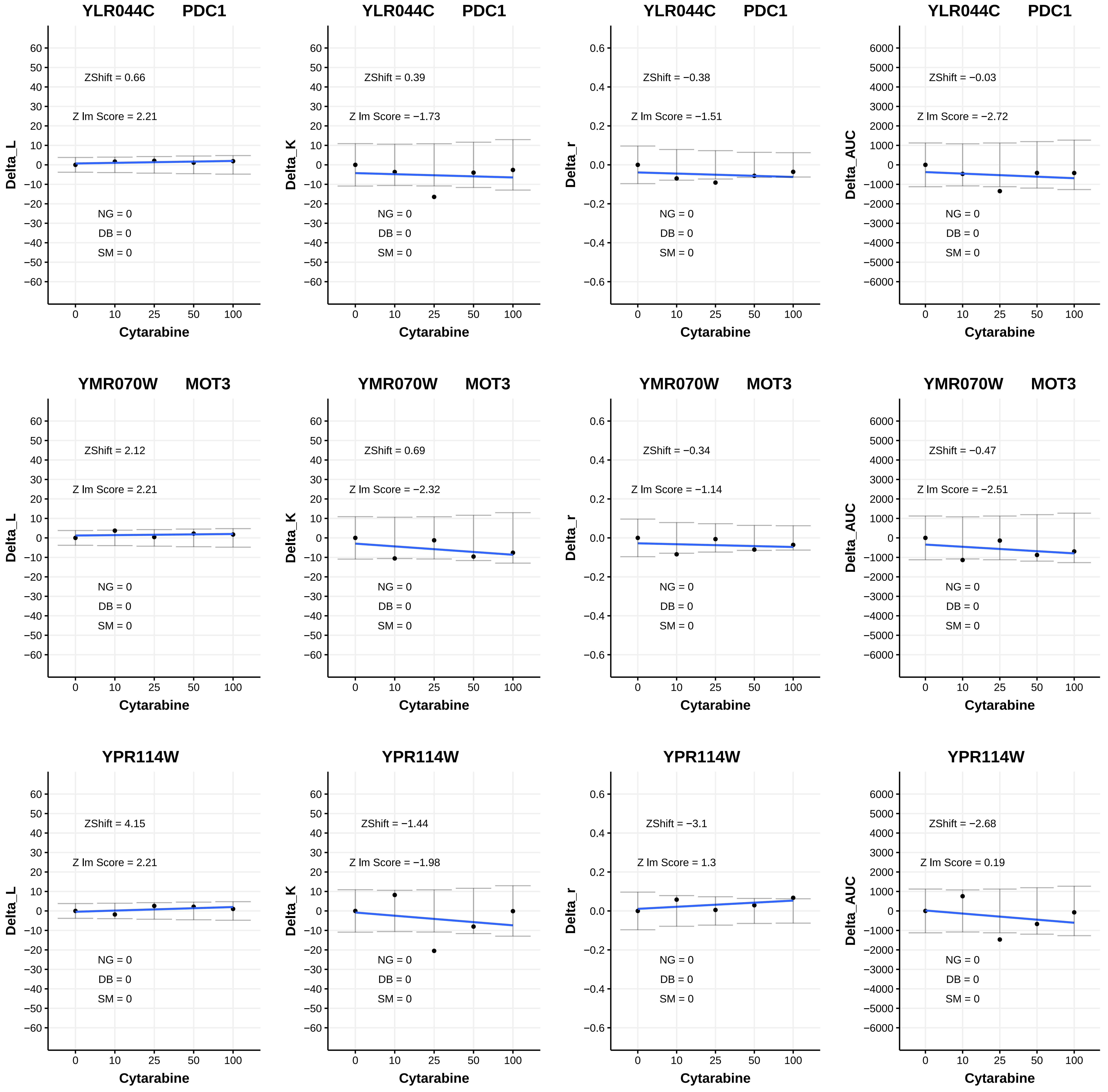

YLR044C PDC1
ZShift = 0.66
Z lm Score = 2.21
NG = 0
DB = 0
SM = 0
Delta_L
Cytarabine
YLR044C PDC1
ZShift = 0.39
Z lm Score = −1.73
NG = 0
DB = 0
SM = 0
Delta_K
Cytarabine
YLR044C PDC1
ZShift = −0.38
Z lm Score = −1.51
NG = 0
DB = 0
SM = 0
Delta_r
Cytarabine
YLR044C PDC1
ZShift = −0.03
Z lm Score = −2.72
NG = 0
DB = 0
SM = 0
Delta_AUC
Cytarabine
YMR070W MOT3
ZShift = 2.12
Z lm Score = 2.21
NG = 0
DB = 0
SM = 0
Delta_L
Cytarabine
YMR070W MOT3
ZShift = 0.69
Z lm Score = −2.32
NG = 0
DB = 0
SM = 0
Delta_K
Cytarabine
YMR070W MOT3
ZShift = −0.34
Z lm Score = −1.14
NG = 0
DB = 0
SM = 0
Delta_r
Cytarabine
YMR070W MOT3
ZShift = −0.47
Z lm Score = −2.51
NG = 0
DB = 0
SM = 0
Delta_AUC
Cytarabine
YPR114W
ZShift = 4.15
Z lm Score = 2.21
NG = 0
DB = 0
SM = 0
Delta_L
Cytarabine
YPR114W
ZShift = −1.44
Z lm Score = −1.98
NG = 0
DB = 0
SM = 0
Delta_K
Cytarabine
YPR114W
ZShift = −3.1
Z lm Score = 1.3
NG = 0
DB = 0
SM = 0
Delta_r
Cytarabine
YPR114W
ZShift = −2.68
Z lm Score = 0.19
NG = 0
DB = 0
SM = 0
Delta_AUC
Cytarabine

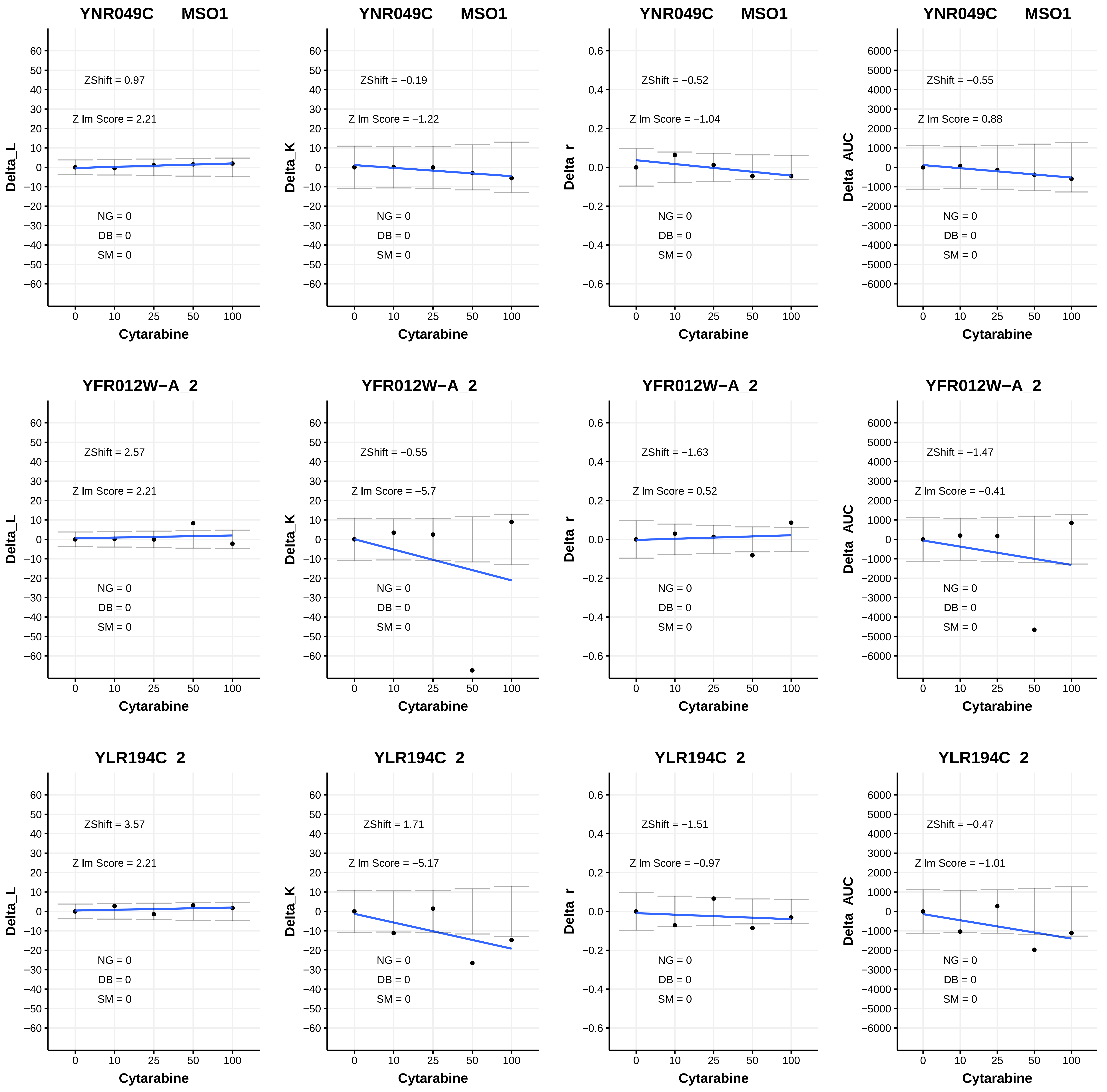
YNR049C MSO1
ZShift = 0.97
Z lm Score = 2.21
NG = 0
DB = 0
SM = 0
Delta_L
Cytarabine
YNR049C MSO1
ZShift = −0.19
Z lm Score = −1.22
NG = 0
DB = 0
SM = 0
Delta_K
Cytarabine
YNR049C MSO1
ZShift = −0.52
Z lm Score = −1.04
NG = 0
DB = 0
SM = 0
Delta_r
Cytarabine
YNR049C MSO1
ZShift = −0.55
Z lm Score = 0.88
NG = 0
DB = 0
SM = 0
Delta_AUC
Cytarabine
YFR012W−A_2
ZShift = 2.57
Z lm Score = 2.21
NG = 0
DB = 0
SM = 0
Delta_L
Cytarabine
YFR012W−A_2
ZShift = −0.55
Z lm Score = −5.7
NG = 0
DB = 0
SM = 0
Delta_K
Cytarabine
YFR012W−A_2
ZShift = −1.63
Z lm Score = 0.52
NG = 0
DB = 0
SM = 0
Delta_r
Cytarabine
YFR012W−A_2
ZShift = −1.47
Z lm Score = −0.41
NG = 0
DB = 0
SM = 0
Delta_AUC
Cytarabine
YLR194C_2
ZShift = 3.57
Z lm Score = 2.21
NG = 0
DB = 0
SM = 0
Delta_L
Cytarabine
YLR194C_2
ZShift = 1.71
Z lm Score = −5.17
NG = 0
DB = 0
SM = 0
Delta_K
Cytarabine
YLR194C_2
ZShift = −1.51
Z lm Score = −0.97
NG = 0
DB = 0
SM = 0
Delta_r
Cytarabine
YLR194C_2
ZShift = −0.47
Z lm Score = −1.01
NG = 0
DB = 0
SM = 0
Delta_AUC
Cytarabine

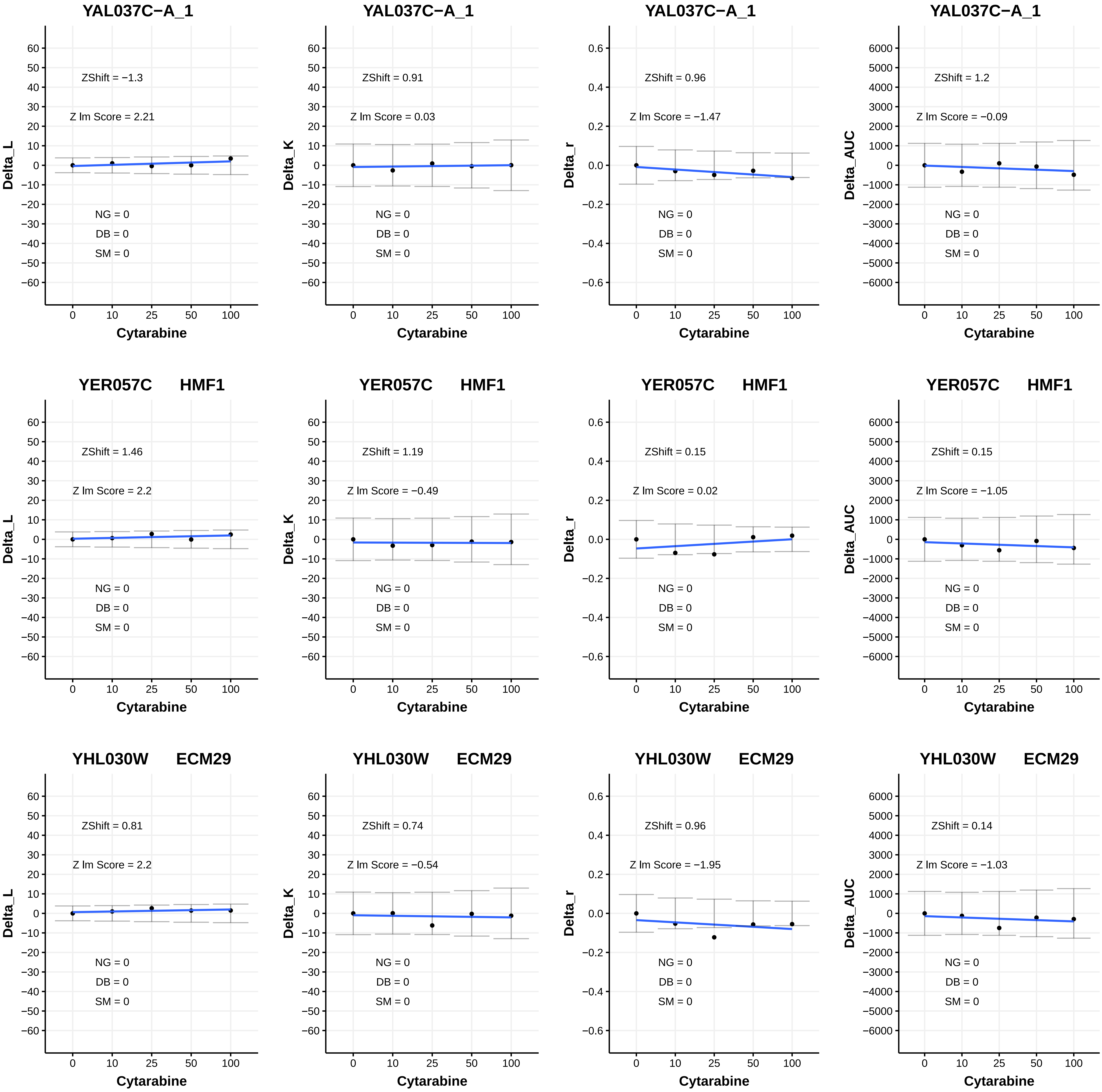
YAL037C−A_1
Delta_L
ZShift = −1.3
Z lm Score = 2.21
NG = 0
DB = 0
SM = 0
Cytarabine
YAL037C−A_1
Delta_K
ZShift = 0.91
Z lm Score = 0.03
NG = 0
DB = 0
SM = 0
Cytarabine
YAL037C−A_1
Delta_r
ZShift = 0.96
Z lm Score = −1.47
NG = 0
DB = 0
SM = 0
Cytarabine
YAL037C−A_1
Delta_AUC
ZShift = 1.2
Z lm Score = −0.09
NG = 0
DB = 0
SM = 0
Cytarabine
YER057C HMF1
Delta_L
ZShift = 1.46
Z lm Score = 2.2
NG = 0
DB = 0
SM = 0
Cytarabine
YER057C HMF1
Delta_K
ZShift = 1.19
Z lm Score = −0.49
NG = 0
DB = 0
SM = 0
Cytarabine
YER057C HMF1
Delta_r
ZShift = 0.15
Z lm Score = 0.02
NG = 0
DB = 0
SM = 0
Cytarabine
YER057C HMF1
Delta_AUC
ZShift = 0.15
Z lm Score = −1.05
NG = 0
DB = 0
SM = 0
Cytarabine
YHL030W ECM29
Delta_L
ZShift = 0.81
Z lm Score = 2.2
NG = 0
DB = 0
SM = 0
Cytarabine
YHL030W ECM29
Delta_K
ZShift = 0.74
Z lm Score = −0.54
NG = 0
DB = 0
SM = 0
Cytarabine
YHL030W ECM29
Delta_r
ZShift = 0.96
Z lm Score = −1.95
NG = 0
DB = 0
SM = 0
Cytarabine
YHL030W ECM29
Delta_AUC
ZShift = 0.14
Z lm Score = −1.03
NG = 0
DB = 0
SM = 0
Cytarabine

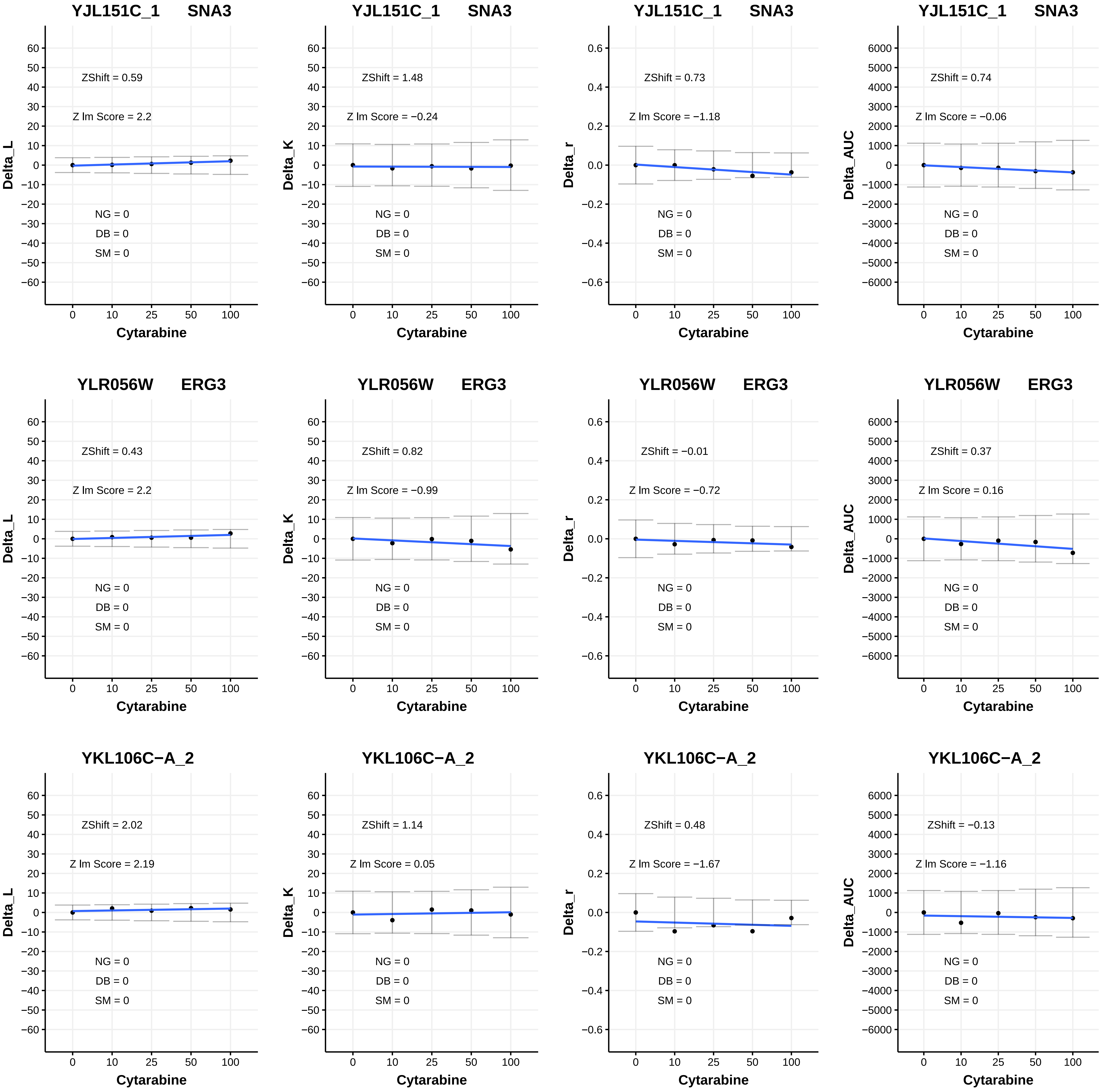

YJL151C_1 SNA3
ZShift = 0.59
Z lm Score = 2.2
NG = 0
DB = 0
SM = 0
Delta_L
Cytarabine
YJL151C_1 SNA3
ZShift = 1.48
Z lm Score = −0.24
NG = 0
DB = 0
SM = 0
Delta_K
Cytarabine
YJL151C_1 SNA3
ZShift = 0.73
Z lm Score = −1.18
NG = 0
DB = 0
SM = 0
Delta_r
Cytarabine
YJL151C_1 SNA3
ZShift = 0.74
Z lm Score = −0.06
NG = 0
DB = 0
SM = 0
Delta_AUC
Cytarabine
YLR056W ERG3
ZShift = 0.43
Z lm Score = 2.2
NG = 0
DB = 0
SM = 0
Delta_L
Cytarabine
YLR056W ERG3
ZShift = 0.82
Z lm Score = −0.99
NG = 0
DB = 0
SM = 0
Delta_K
Cytarabine
YLR056W ERG3
ZShift = −0.01
Z lm Score = −0.72
NG = 0
DB = 0
SM = 0
Delta_r
Cytarabine
YLR056W ERG3
ZShift = 0.37
Z lm Score = 0.16
NG = 0
DB = 0
SM = 0
Delta_AUC
Cytarabine
YKL106C−A_2
ZShift = 2.02
Z lm Score = 2.19
NG = 0
DB = 0
SM = 0
Delta_L
Cytarabine
YKL106C−A_2
ZShift = 1.14
Z lm Score = 0.05
NG = 0
DB = 0
SM = 0
Delta_K
Cytarabine
YKL106C−A_2
ZShift = 0.48
Z lm Score = −1.67
NG = 0
DB = 0
SM = 0
Delta_r
Cytarabine
YKL106C−A_2
ZShift = −0.13
Z lm Score = −1.16
NG = 0
DB = 0
SM = 0
Delta_AUC
Cytarabine

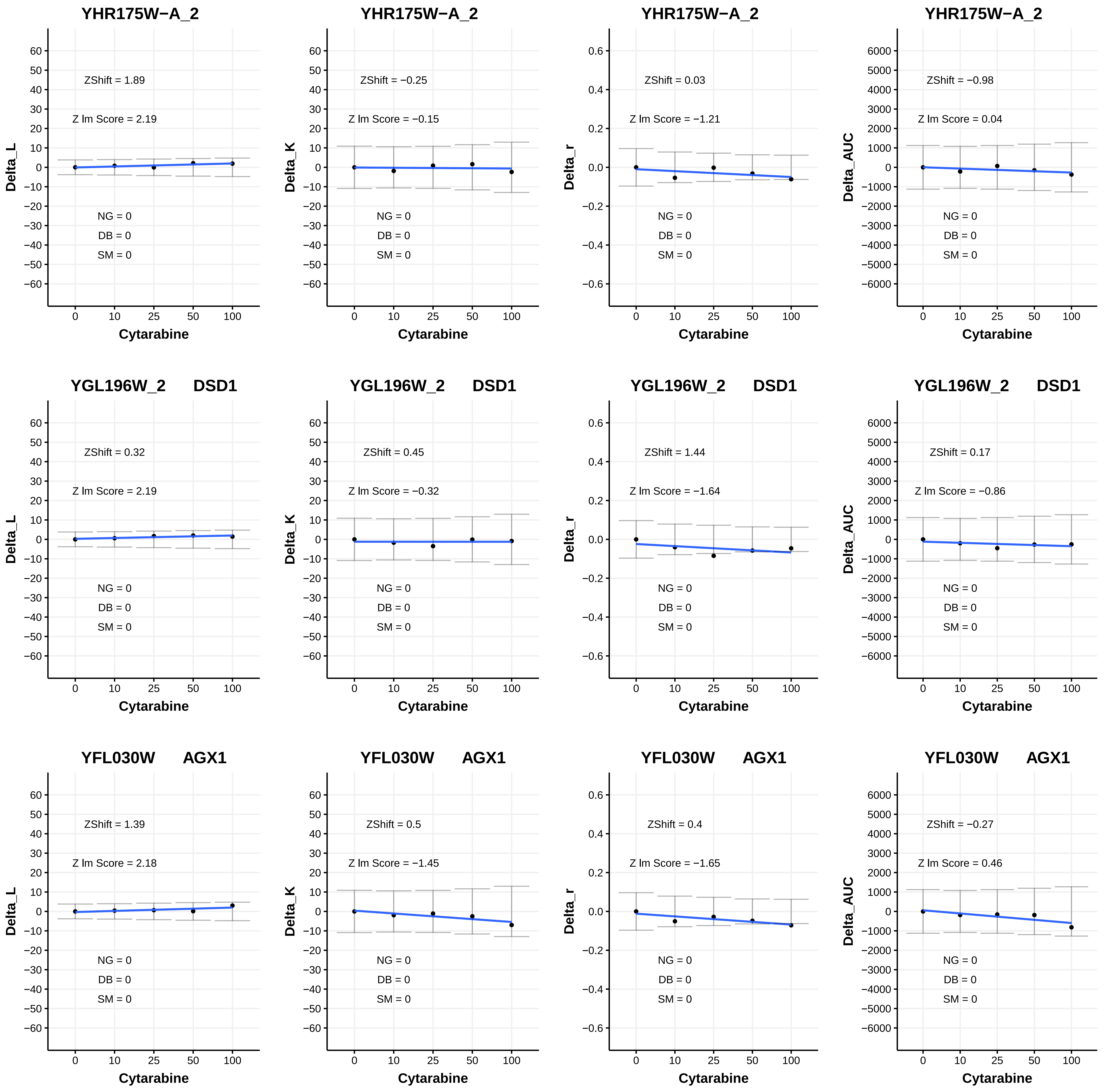

YHR175W−A_2
ZShift = 1.89
Z lm Score = 2.19
NG = 0
DB = 0
SM = 0
Delta_L
Cytarabine
YHR175W−A_2
ZShift = −0.25
Z lm Score = −0.15
NG = 0
DB = 0
SM = 0
Delta_K
Cytarabine
YHR175W−A_2
ZShift = 0.03
Z lm Score = −1.21
NG = 0
DB = 0
SM = 0
Delta_r
Cytarabine
YHR175W−A_2
ZShift = −0.98
Z lm Score = 0.04
NG = 0
DB = 0
SM = 0
Delta_AUC
Cytarabine
YGL196W_2 DSD1
ZShift = 0.32
Z lm Score = 2.19
NG = 0
DB = 0
SM = 0
Delta_L
Cytarabine
YGL196W_2 DSD1
ZShift = 0.45
Z lm Score = −0.32
NG = 0
DB = 0
SM = 0
Delta_K
Cytarabine
YGL196W_2 DSD1
ZShift = 1.44
Z lm Score = −1.64
NG = 0
DB = 0
SM = 0
Delta_r
Cytarabine
YGL196W_2 DSD1
ZShift = 0.17
Z lm Score = −0.86
NG = 0
DB = 0
SM = 0
Delta_AUC
Cytarabine
YFL030W AGX1
ZShift = 1.39
Z lm Score = 2.18
NG = 0
DB = 0
SM = 0
Delta_L
Cytarabine
YFL030W AGX1
ZShift = 0.5
Z lm Score = −1.45
NG = 0
DB = 0
SM = 0
Delta_K
Cytarabine
YFL030W AGX1
ZShift = 0.4
Z lm Score = −1.65
NG = 0
DB = 0
SM = 0
Delta_r
Cytarabine
YFL030W AGX1
ZShift = −0.27
Z lm Score = 0.46
NG = 0
DB = 0
SM = 0
Delta_AUC
Cytarabine

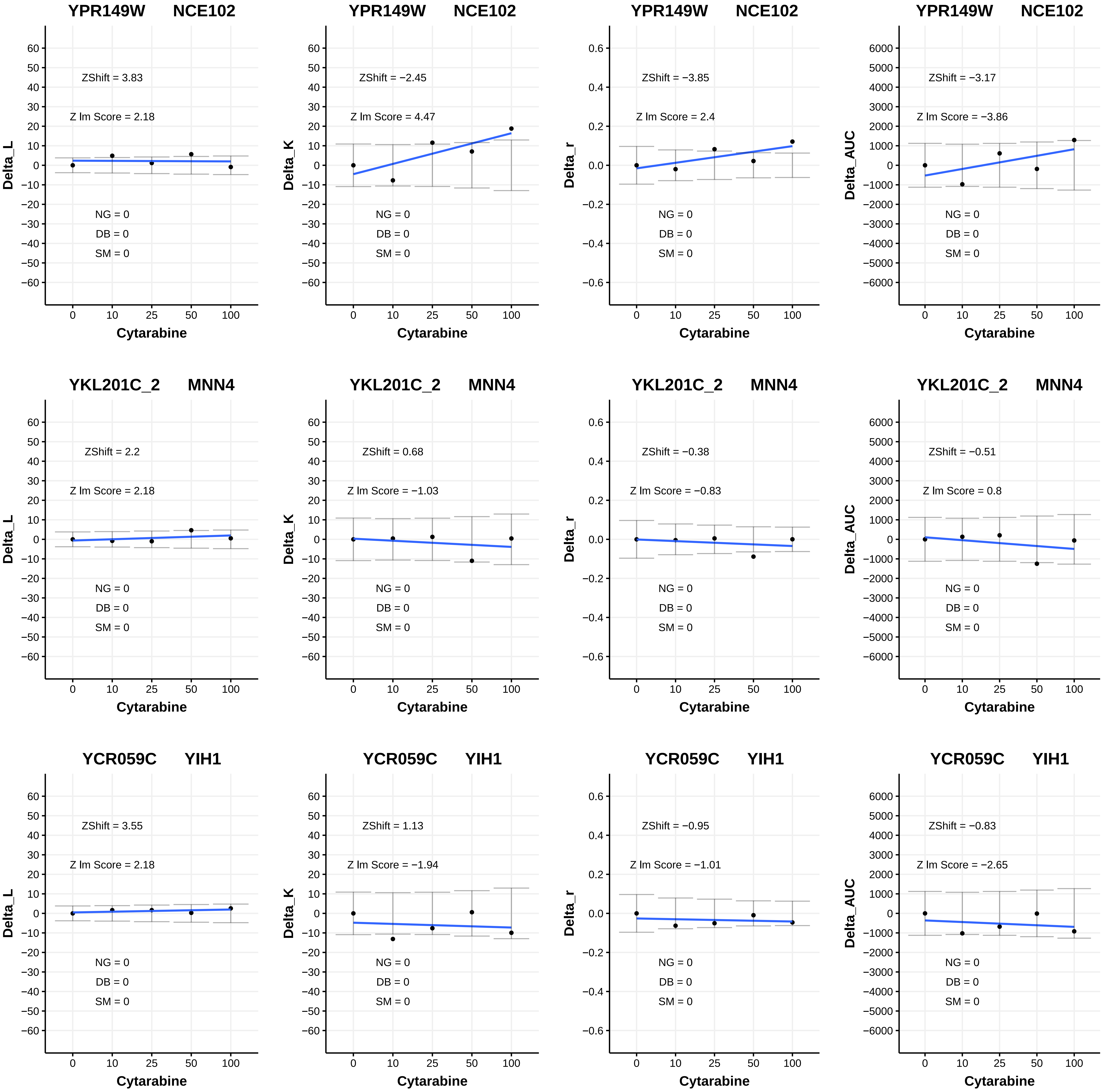

YPR149W NCE102
ZShift = 3.83
Z lm Score = 2.18
NG = 0
DB = 0
SM = 0
Delta_L
Cytarabine
YPR149W NCE102
ZShift = −2.45
Z lm Score = 4.47
NG = 0
DB = 0
SM = 0
Delta_K
Cytarabine
YPR149W NCE102
ZShift = −3.85
Z lm Score = 2.4
NG = 0
DB = 0
SM = 0
Delta_r
Cytarabine
YPR149W NCE102
ZShift = −3.17
Z lm Score = −3.86
NG = 0
DB = 0
SM = 0
Delta_AUC
Cytarabine
YKL201C_2 MNN4
ZShift = 2.2
Z lm Score = 2.18
NG = 0
DB = 0
SM = 0
Delta_L
Cytarabine
YKL201C_2 MNN4
ZShift = 0.68
Z lm Score = −1.03
NG = 0
DB = 0
SM = 0
Delta_K
Cytarabine
YKL201C_2 MNN4
ZShift = −0.38
Z lm Score = −0.83
NG = 0
DB = 0
SM = 0
Delta_r
Cytarabine
YKL201C_2 MNN4
ZShift = −0.51
Z lm Score = 0.8
NG = 0
DB = 0
SM = 0
Delta_AUC
Cytarabine
YCR059C YIH1
ZShift = 3.55
Z lm Score = 2.18
NG = 0
DB = 0
SM = 0
Delta_L
Cytarabine
YCR059C YIH1
ZShift = 1.13
Z lm Score = −1.94
NG = 0
DB = 0
SM = 0
Delta_K
Cytarabine
YCR059C YIH1
ZShift = −0.95
Z lm Score = −1.01
NG = 0
DB = 0
SM = 0
Delta_r
Cytarabine
YCR059C YIH1
ZShift = −0.83
Z lm Score = −2.65
NG = 0
DB = 0
SM = 0
Delta_AUC
Cytarabine

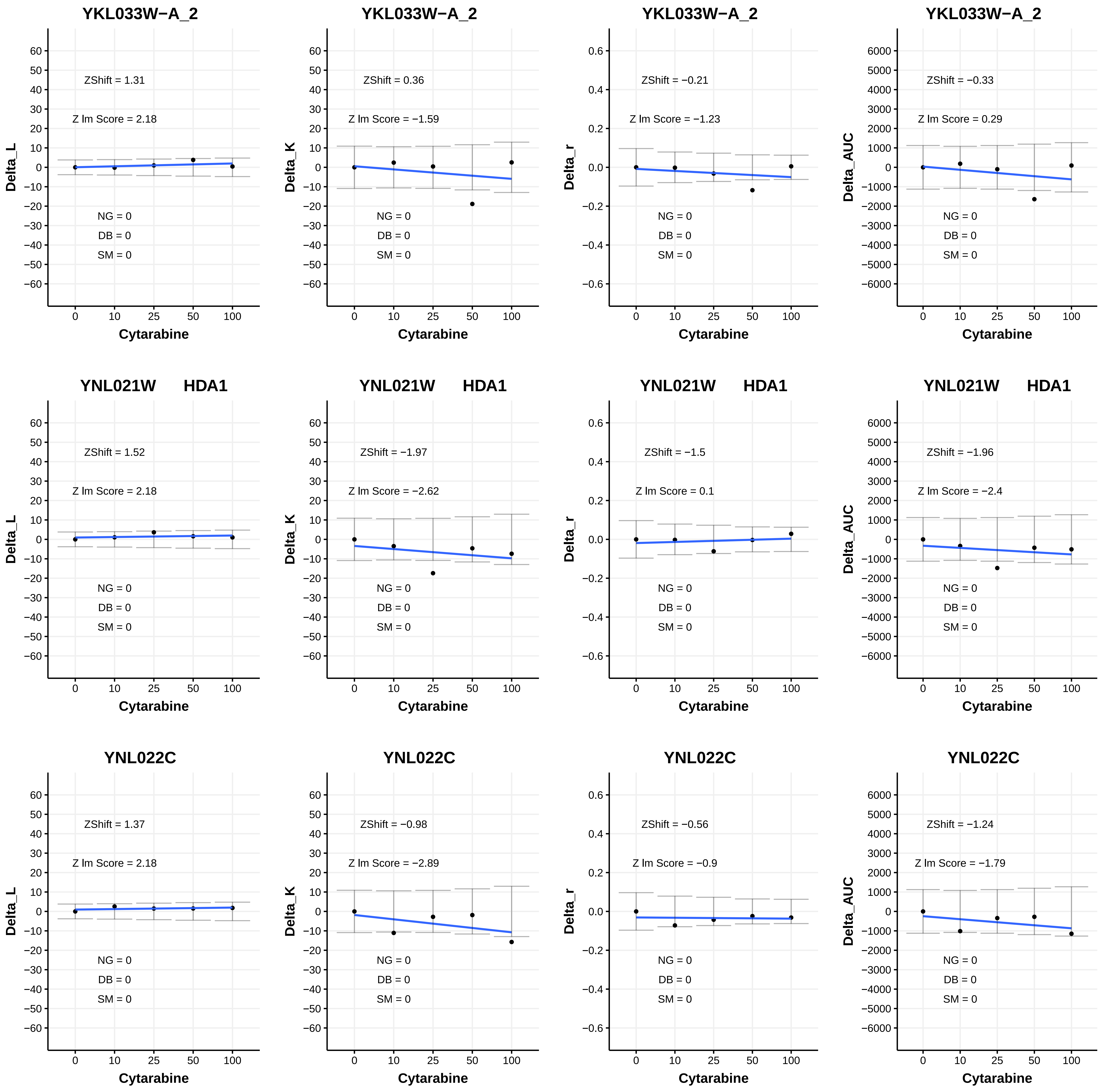
YKL033W−A_2
ZShift = 1.31
Z lm Score = 2.18
NG = 0
DB = 0
SM = 0
Delta_L
Cytarabine
YKL033W−A_2
ZShift = 0.36
Z lm Score = −1.59
NG = 0
DB = 0
SM = 0
Delta_K
Cytarabine
YKL033W−A_2
ZShift = −0.21
Z lm Score = −1.23
NG = 0
DB = 0
SM = 0
Delta_r
Cytarabine
YKL033W−A_2
ZShift = −0.33
Z lm Score = 0.29
NG = 0
DB = 0
SM = 0
Delta_AUC
Cytarabine
YNL021W HDA1
ZShift = 1.52
Z lm Score = 2.18
NG = 0
DB = 0
SM = 0
Delta_L
Cytarabine
YNL021W HDA1
ZShift = −1.97
Z lm Score = −2.62
NG = 0
DB = 0
SM = 0
Delta_K
Cytarabine
YNL021W HDA1
ZShift = −1.5
Z lm Score = 0.1
NG = 0
DB = 0
SM = 0
Delta_r
Cytarabine
YNL021W HDA1
ZShift = −1.96
Z lm Score = −2.4
NG = 0
DB = 0
SM = 0
Delta_AUC
Cytarabine
YNL022C
ZShift = 1.37
Z lm Score = 2.18
NG = 0
DB = 0
SM = 0
Delta_L
Cytarabine
YNL022C
ZShift = −0.98
Z lm Score = −2.89
NG = 0
DB = 0
SM = 0
Delta_K
Cytarabine
YNL022C
ZShift = −0.56
Z lm Score = −0.9
NG = 0
DB = 0
SM = 0
Delta_r
Cytarabine
YNL022C
ZShift = −1.24
Z lm Score = −1.79
NG = 0
DB = 0
SM = 0
Delta_AUC
Cytarabine

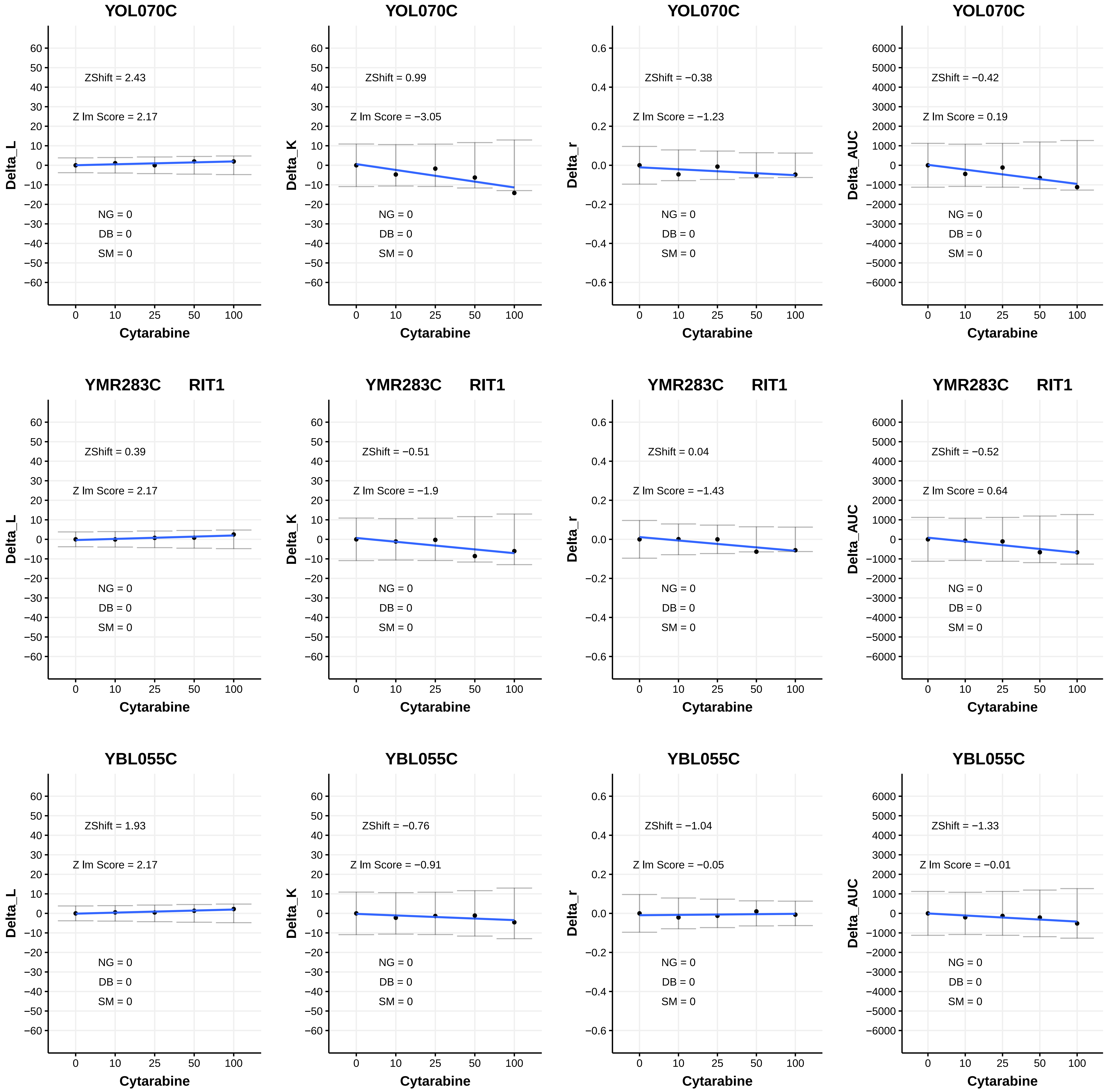
YOL070C
Delta_L
ZShift = 2.43
Z lm Score = 2.17
NG = 0
DB = 0
SM = 0
Cytarabine
YOL070C
Delta_K
ZShift = 0.99
Z lm Score = −3.05
NG = 0
DB = 0
SM = 0
Cytarabine
YOL070C
Delta_r
ZShift = −0.38
Z lm Score = −1.23
NG = 0
DB = 0
SM = 0
Cytarabine
YOL070C
Delta_AUC
ZShift = −0.42
Z lm Score = 0.19
NG = 0
DB = 0
SM = 0
Cytarabine
YMR283C RIT1
Delta_L
ZShift = 0.39
Z lm Score = 2.17
NG = 0
DB = 0
SM = 0
Cytarabine
YMR283C RIT1
Delta_K
ZShift = −0.51
Z lm Score = −1.9
NG = 0
DB = 0
SM = 0
Cytarabine
YMR283C RIT1
Delta_r
ZShift = 0.04
Z lm Score = −1.43
NG = 0
DB = 0
SM = 0
Cytarabine
YMR283C RIT1
Delta_AUC
ZShift = −0.52
Z lm Score = 0.64
NG = 0
DB = 0
SM = 0
Cytarabine
YBL055C
Delta_L
ZShift = 1.93
Z lm Score = 2.17
NG = 0
DB = 0
SM = 0
Cytarabine
YBL055C
Delta_K
ZShift = −0.76
Z lm Score = −0.91
NG = 0
DB = 0
SM = 0
Cytarabine
YBL055C
Delta_r
ZShift = −1.04
Z lm Score = −0.05
NG = 0
DB = 0
SM = 0
Cytarabine
YBL055C
Delta_AUC
ZShift = −1.33
Z lm Score = −0.01
NG = 0
DB = 0
SM = 0
Cytarabine

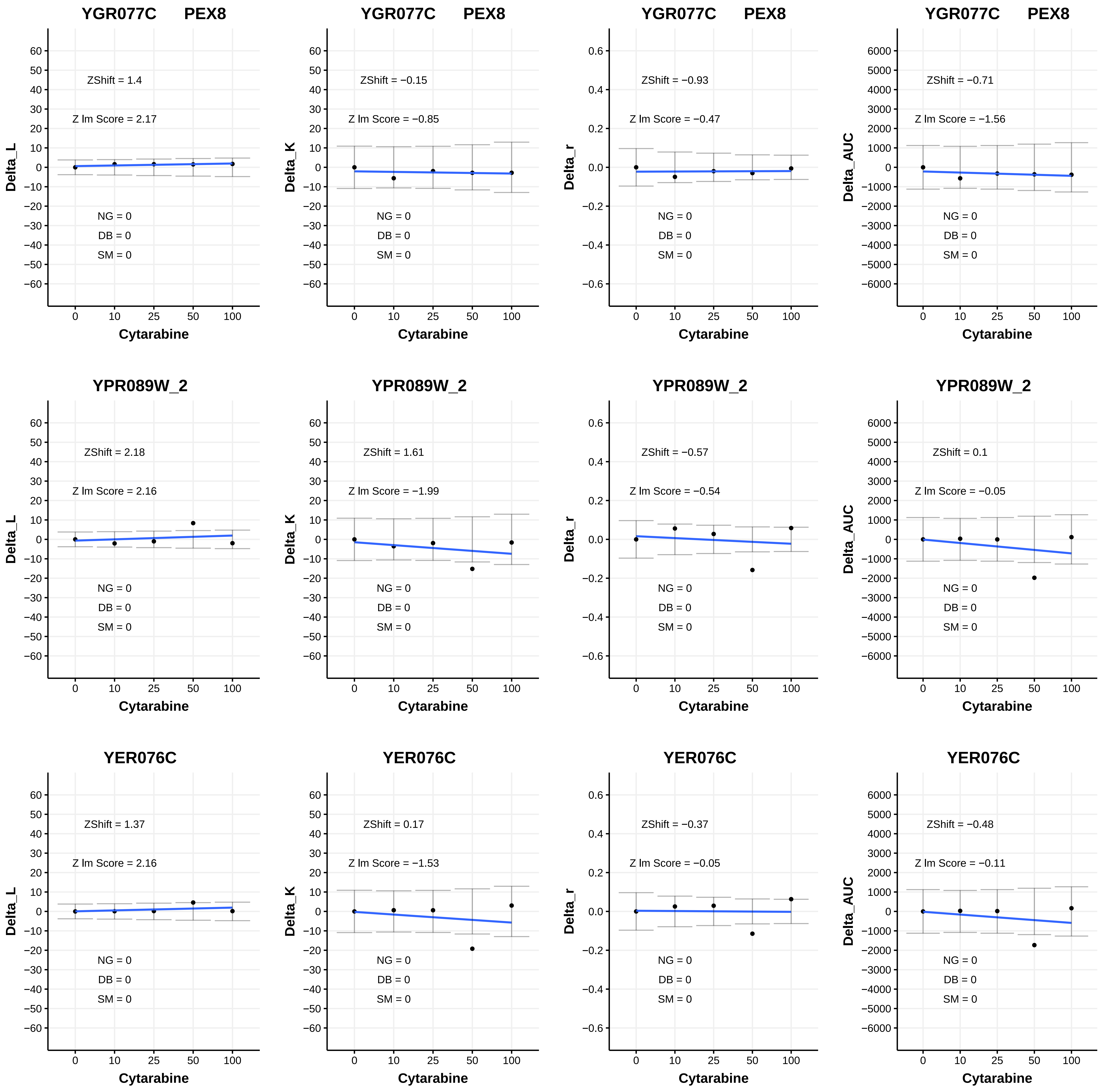

YGR077C PEX8
ZShift = 1.4
Z lm Score = 2.17
NG = 0
DB = 0
SM = 0
Delta_L
Cytarabine
YGR077C PEX8
ZShift = −0.15
Z lm Score = −0.85
NG = 0
DB = 0
SM = 0
Delta_K
Cytarabine
YGR077C PEX8
ZShift = −0.93
Z lm Score = −0.47
NG = 0
DB = 0
SM = 0
Delta_r
Cytarabine
YGR077C PEX8
ZShift = −0.71
Z lm Score = −1.56
NG = 0
DB = 0
SM = 0
Delta_AUC
Cytarabine
YPR089W_2
ZShift = 2.18
Z lm Score = 2.16
NG = 0
DB = 0
SM = 0
Delta_L
Cytarabine
YPR089W_2
ZShift = 1.61
Z lm Score = −1.99
NG = 0
DB = 0
SM = 0
Delta_K
Cytarabine
YPR089W_2
ZShift = −0.57
Z lm Score = −0.54
NG = 0
DB = 0
SM = 0
Delta_r
Cytarabine
YPR089W_2
ZShift = 0.1
Z lm Score = −0.05
NG = 0
DB = 0
SM = 0
Delta_AUC
Cytarabine
YER076C
ZShift = 1.37
Z lm Score = 2.16
NG = 0
DB = 0
SM = 0
Delta_L
Cytarabine
YER076C
ZShift = 0.17
Z lm Score = −1.53
NG = 0
DB = 0
SM = 0
Delta_K
Cytarabine
YER076C
ZShift = −0.37
Z lm Score = −0.05
NG = 0
DB = 0
SM = 0
Delta_r
Cytarabine
YER076C
ZShift = −0.48
Z lm Score = −0.11
NG = 0
DB = 0
SM = 0
Delta_AUC
Cytarabine

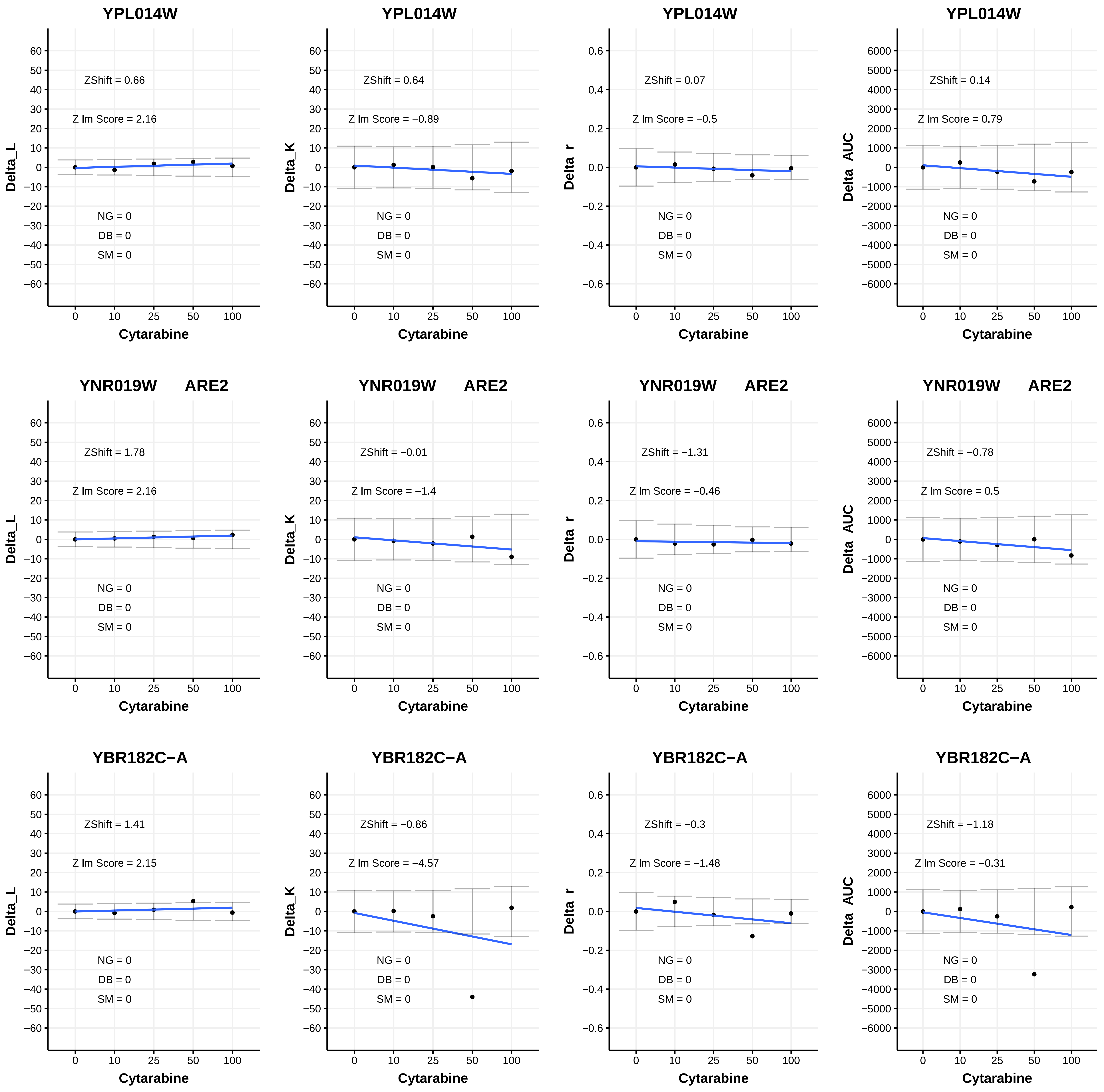

YPL014W
ZShift = 0.66
Z lm Score = 2.16
NG = 0
DB = 0
SM = 0
Delta_L
Cytarabine
YPL014W
ZShift = 0.64
Z lm Score = −0.89
NG = 0
DB = 0
SM = 0
Delta_K
Cytarabine
YPL014W
ZShift = 0.07
Z lm Score = −0.5
NG = 0
DB = 0
SM = 0
Delta_r
Cytarabine
YPL014W
ZShift = 0.14
Z lm Score = 0.79
NG = 0
DB = 0
SM = 0
Delta_AUC
Cytarabine
YNR019W ARE2
ZShift = 1.78
Z lm Score = 2.16
NG = 0
DB = 0
SM = 0
Delta_L
Cytarabine
YNR019W ARE2
ZShift = −0.01
Z lm Score = −1.4
NG = 0
DB = 0
SM = 0
Delta_K
Cytarabine
YNR019W ARE2
ZShift = −1.31
Z lm Score = −0.46
NG = 0
DB = 0
SM = 0
Delta_r
Cytarabine
YNR019W ARE2
ZShift = −0.78
Z lm Score = 0.5
NG = 0
DB = 0
SM = 0
Delta_AUC
Cytarabine
YBR182C−A
ZShift = 1.41
Z lm Score = 2.15
NG = 0
DB = 0
SM = 0
Delta_L
Cytarabine
YBR182C−A
ZShift = −0.86
Z lm Score = −4.57
NG = 0
DB = 0
SM = 0
Delta_K
Cytarabine
YBR182C−A
ZShift = −0.3
Z lm Score = −1.48
NG = 0
DB = 0
SM = 0
Delta_r
Cytarabine
YBR182C−A
ZShift = −1.18
Z lm Score = −0.31
NG = 0
DB = 0
SM = 0
Delta_AUC
Cytarabine

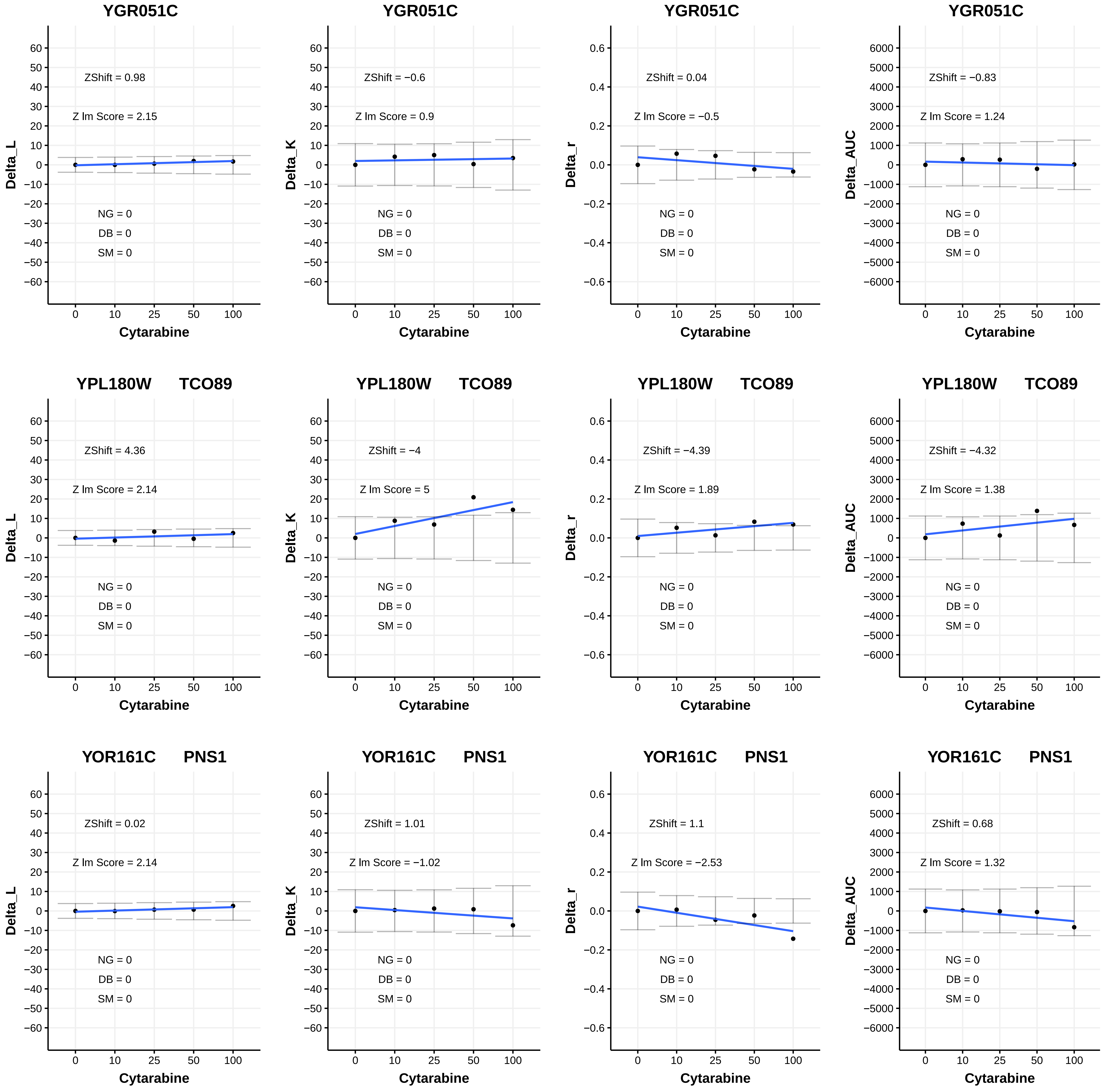

YGR051C
Delta_L
ZShift = 0.98
Z lm Score = 2.15
NG = 0
DB = 0
SM = 0
Cytarabine
YGR051C
Delta_K
ZShift = −0.6
Z lm Score = 0.9
NG = 0
DB = 0
SM = 0
Cytarabine
YGR051C
Delta_r
ZShift = 0.04
Z lm Score = −0.5
NG = 0
DB = 0
SM = 0
Cytarabine
YGR051C
Delta_AUC
ZShift = −0.83
Z lm Score = 1.24
NG = 0
DB = 0
SM = 0
Cytarabine
YPL180W TCO89
Delta_L
ZShift = 4.36
Z lm Score = 2.14
NG = 0
DB = 0
SM = 0
Cytarabine
YPL180W TCO89
Delta_K
ZShift = −4
Z lm Score = 5
NG = 0
DB = 0
SM = 0
Cytarabine
YPL180W TCO89
Delta_r
ZShift = −4.39
Z lm Score = 1.89
NG = 0
DB = 0
SM = 0
Cytarabine
YPL180W TCO89
Delta_AUC
ZShift = −4.32
Z lm Score = 1.38
NG = 0
DB = 0
SM = 0
Cytarabine
YOR161C PNS1
Delta_L
ZShift = 0.02
Z lm Score = 2.14
NG = 0
DB = 0
SM = 0
Cytarabine
YOR161C PNS1
Delta_K
ZShift = 1.01
Z lm Score = −1.02
NG = 0
DB = 0
SM = 0
Cytarabine
YOR161C PNS1
Delta_r
ZShift = 1.1
Z lm Score = −2.53
NG = 0
DB = 0
SM = 0
Cytarabine
YOR161C PNS1
Delta_AUC
ZShift = 0.68
Z lm Score = 1.32
NG = 0
DB = 0
SM = 0
Cytarabine

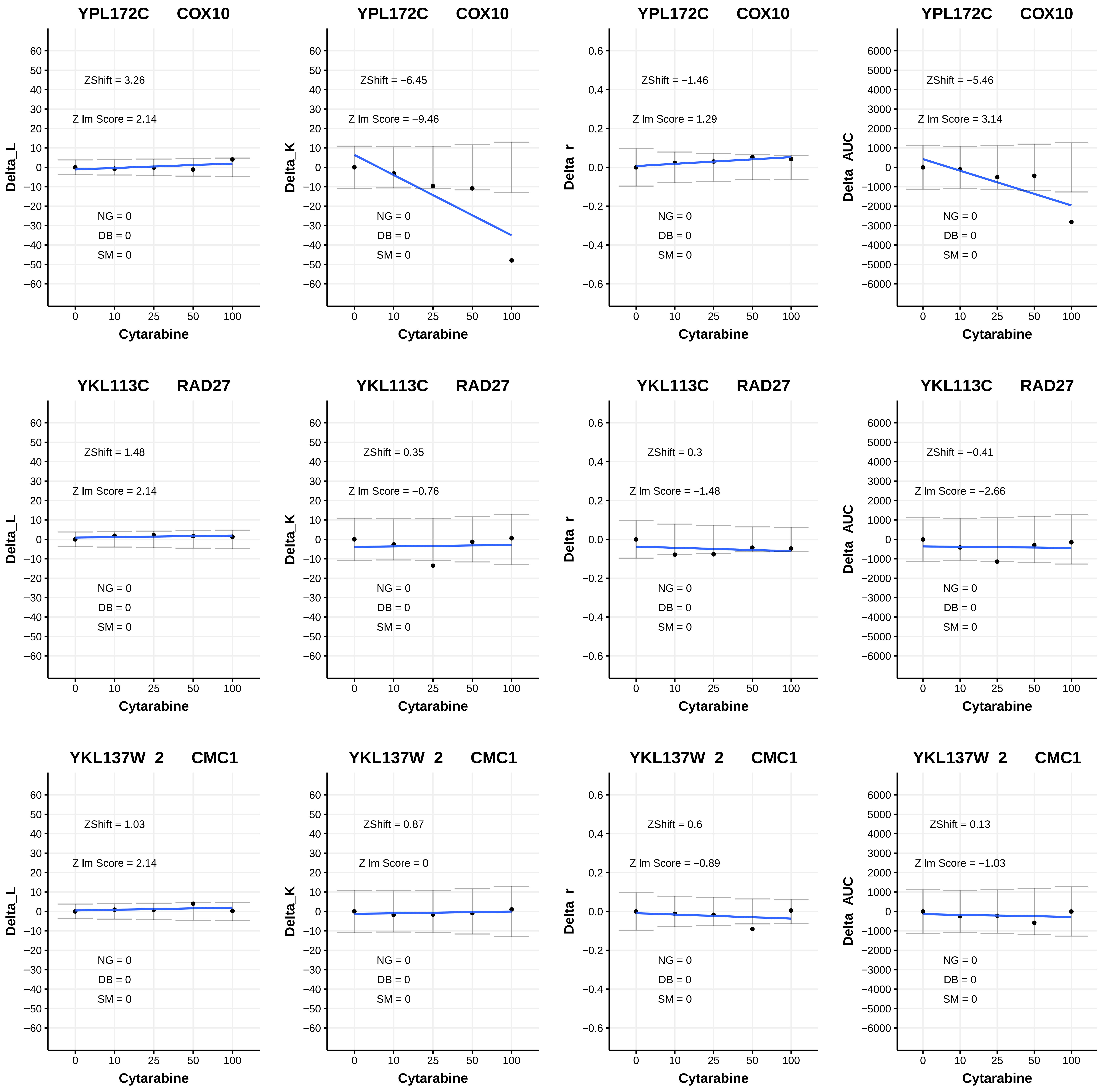
YPL172C COX10
ZShift = 3.26
Z lm Score = 2.14
NG = 0
DB = 0
SM = 0
Delta_L
Cytarabine
YPL172C COX10
ZShift = −6.45
Z lm Score = −9.46
NG = 0
DB = 0
SM = 0
Delta_K
Cytarabine
YPL172C COX10
ZShift = −1.46
Z lm Score = 1.29
NG = 0
DB = 0
SM = 0
Delta_r
Cytarabine
YPL172C COX10
ZShift = −5.46
Z lm Score = 3.14
NG = 0
DB = 0
SM = 0
Delta_AUC
Cytarabine
YKL113C RAD27
ZShift = 1.48
Z lm Score = 2.14
NG = 0
DB = 0
SM = 0
Delta_L
Cytarabine
YKL113C RAD27
ZShift = 0.35
Z lm Score = −0.76
NG = 0
DB = 0
SM = 0
Delta_K
Cytarabine
YKL113C RAD27
ZShift = 0.3
Z lm Score = −1.48
NG = 0
DB = 0
SM = 0
Delta_r
Cytarabine
YKL113C RAD27
ZShift = −0.41
Z lm Score = −2.66
NG = 0
DB = 0
SM = 0
Delta_AUC
Cytarabine
YKL137W_2 CMC1
ZShift = 1.03
Z lm Score = 2.14
NG = 0
DB = 0
SM = 0
Delta_L
Cytarabine
YKL137W_2 CMC1
ZShift = 0.87
Z lm Score = 0
NG = 0
DB = 0
SM = 0
Delta_K
Cytarabine
YKL137W_2 CMC1
ZShift = 0.6
Z lm Score = −0.89
NG = 0
DB = 0
SM = 0
Delta_r
Cytarabine
YKL137W_2 CMC1
ZShift = 0.13
Z lm Score = −1.03
NG = 0
DB = 0
SM = 0
Delta_AUC
Cytarabine

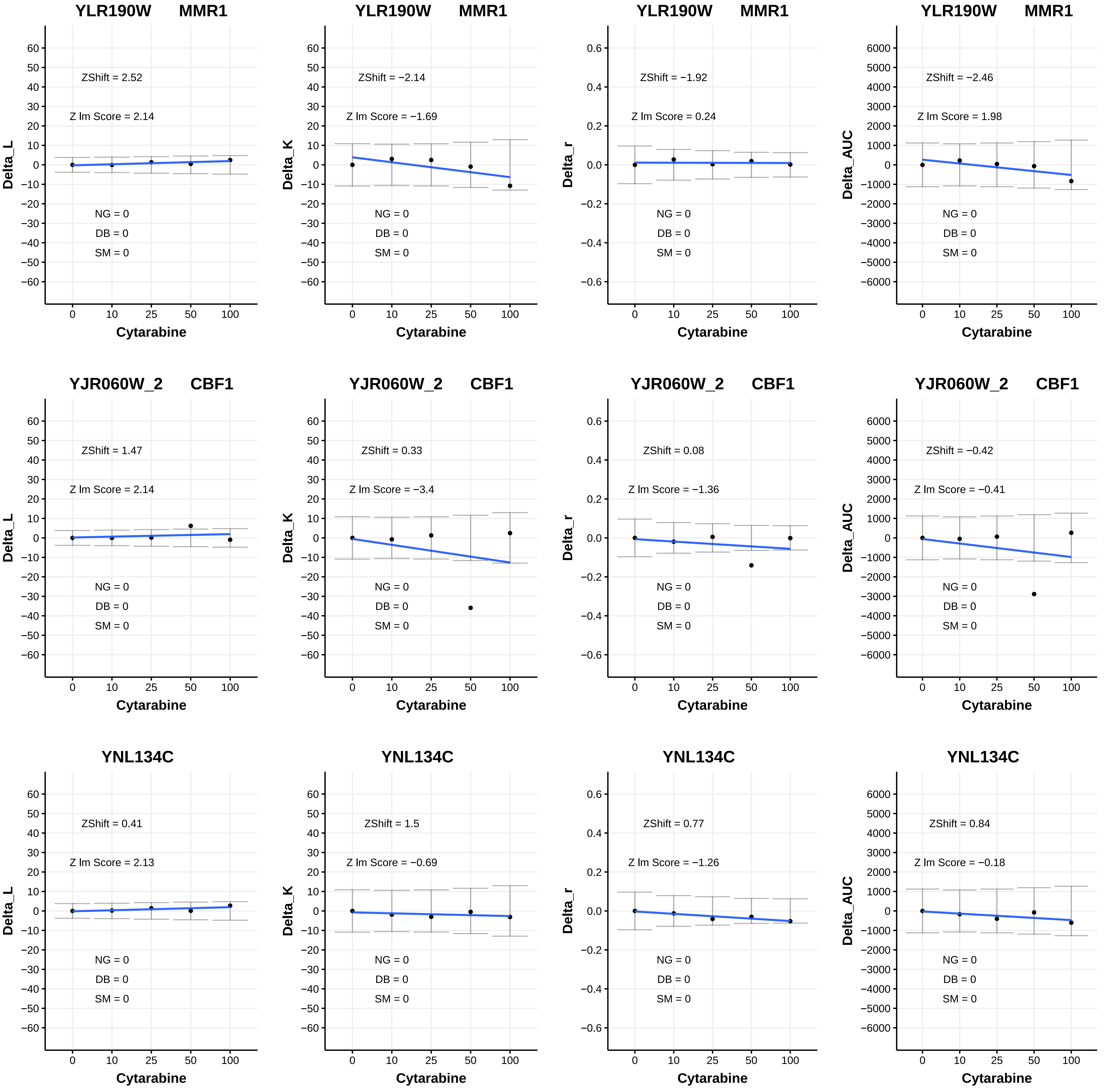
YLR190W MMR1
ZShift = 2.52
Z lm Score = 2.14
NG = 0
DB = 0
SM = 0
Delta_L
Cytarabine
YLR190W MMR1
ZShift = −2.14
Z lm Score = −1.69
NG = 0
DB = 0
SM = 0
Delta_K
Cytarabine
YLR190W MMR1
ZShift = −1.92
Z lm Score = 0.24
NG = 0
DB = 0
SM = 0
Delta_r
Cytarabine
YLR190W MMR1
ZShift = −2.46
Z lm Score = 1.98
NG = 0
DB = 0
SM = 0
Delta_AUC
Cytarabine
YJR060W_2 CBF1
ZShift = 1.47
Z lm Score = 2.14
NG = 0
DB = 0
SM = 0
Delta_L
Cytarabine
YJR060W_2 CBF1
ZShift = 0.33
Z lm Score = −3.4
NG = 0
DB = 0
SM = 0
Delta_K
Cytarabine
YJR060W_2 CBF1
ZShift = 0.08
Z lm Score = −1.36
NG = 0
DB = 0
SM = 0
Delta_r
Cytarabine
YJR060W_2 CBF1
ZShift = −0.42
Z lm Score = −0.41
NG = 0
DB = 0
SM = 0
Delta_AUC
Cytarabine
YNL134C
ZShift = 0.41
Z lm Score = 2.13
NG = 0
DB = 0
SM = 0
Delta_L
Cytarabine
YNL134C
ZShift = 1.5
Z lm Score = −0.69
NG = 0
DB = 0
SM = 0
Delta_K
Cytarabine
YNL134C
ZShift = 0.77
Z lm Score = −1.26
NG = 0
DB = 0
SM = 0
Delta_r
Cytarabine
YNL134C
ZShift = 0.84
Z lm Score = −0.18
NG = 0
DB = 0
SM = 0
Delta_AUC
Cytarabine

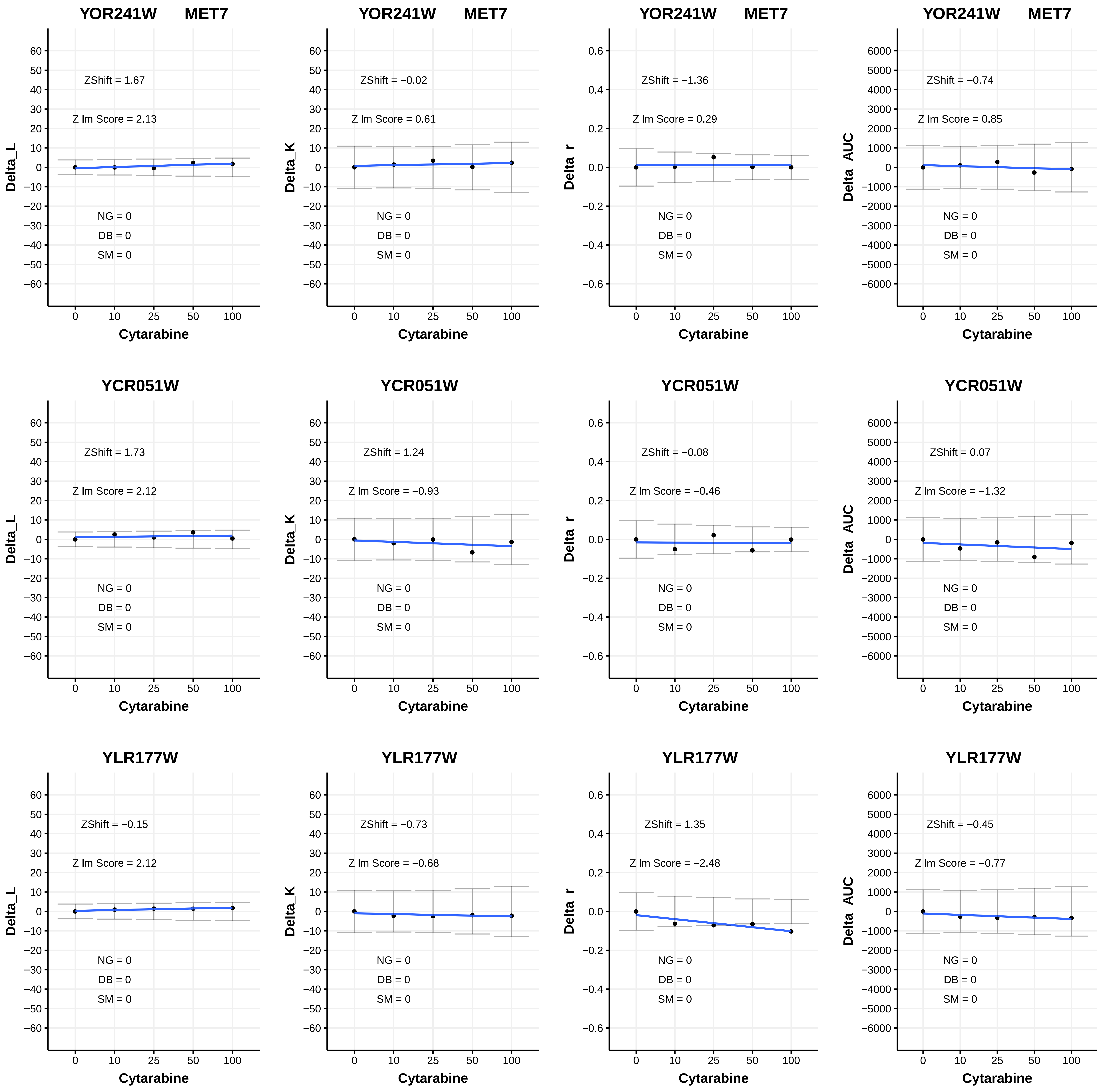
YOR241W MET7
ZShift = 1.67
Z lm Score = 2.13
NG = 0
DB = 0
SM = 0
Delta_L
Cytarabine
YOR241W MET7
ZShift = −0.02
Z lm Score = 0.61
NG = 0
DB = 0
SM = 0
Delta_K
Cytarabine
YOR241W MET7
ZShift = −1.36
Z lm Score = 0.29
NG = 0
DB = 0
SM = 0
Delta_r
Cytarabine
YOR241W MET7
ZShift = −0.74
Z lm Score = 0.85
NG = 0
DB = 0
SM = 0
Delta_AUC
Cytarabine
YCR051W
ZShift = 1.73
Z lm Score = 2.12
NG = 0
DB = 0
SM = 0
Delta_L
Cytarabine
YCR051W
ZShift = 1.24
Z lm Score = −0.93
NG = 0
DB = 0
SM = 0
Delta_K
Cytarabine
YCR051W
ZShift = −0.08
Z lm Score = −0.46
NG = 0
DB = 0
SM = 0
Delta_r
Cytarabine
YCR051W
ZShift = 0.07
Z lm Score = −1.32
NG = 0
DB = 0
SM = 0
Delta_AUC
Cytarabine
YLR177W
ZShift = −0.15
Z lm Score = 2.12
NG = 0
DB = 0
SM = 0
Delta_L
Cytarabine
YLR177W
ZShift = −0.73
Z lm Score = −0.68
NG = 0
DB = 0
SM = 0
Delta_K
Cytarabine
YLR177W
ZShift = 1.35
Z lm Score = −2.48
NG = 0
DB = 0
SM = 0
Delta_r
Cytarabine
YLR177W
ZShift = −0.45
Z lm Score = −0.77
NG = 0
DB = 0
SM = 0
Delta_AUC
Cytarabine

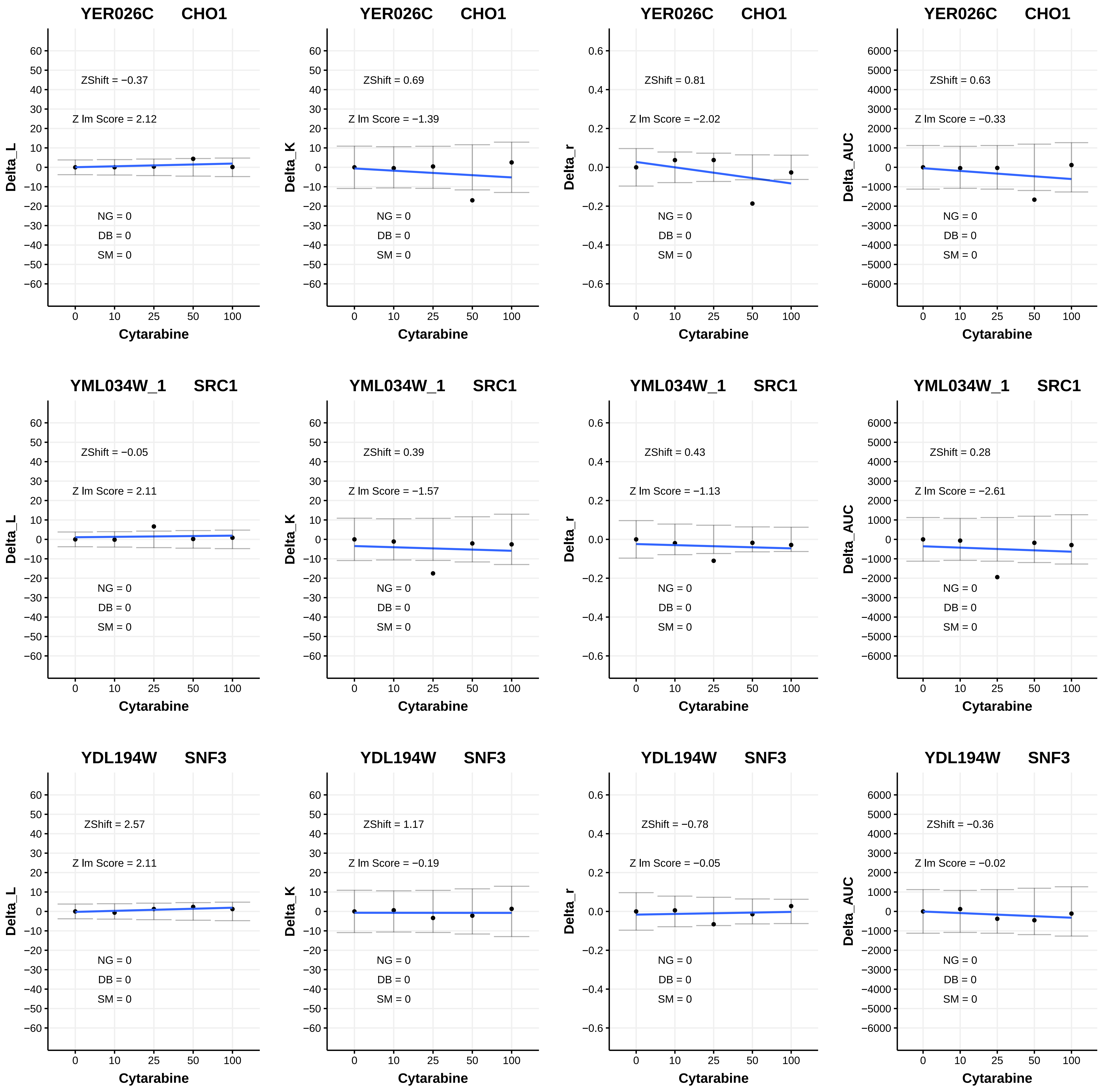
YER026C CHO1
ZShift = −0.37
Z lm Score = 2.12
NG = 0
DB = 0
SM = 0
Delta_L
Cytarabine
YER026C CHO1
ZShift = 0.69
Z lm Score = −1.39
NG = 0
DB = 0
SM = 0
Delta_K
Cytarabine
YER026C CHO1
ZShift = 0.81
Z lm Score = −2.02
NG = 0
DB = 0
SM = 0
Delta_r
Cytarabine
YER026C CHO1
ZShift = 0.63
Z lm Score = −0.33
NG = 0
DB = 0
SM = 0
Delta_AUC
Cytarabine
YML034W_1 SRC1
ZShift = −0.05
Z lm Score = 2.11
NG = 0
DB = 0
SM = 0
Delta_L
Cytarabine
YML034W_1 SRC1
ZShift = 0.39
Z lm Score = −1.57
NG = 0
DB = 0
SM = 0
Delta_K
Cytarabine
YML034W_1 SRC1
ZShift = 0.43
Z lm Score = −1.13
NG = 0
DB = 0
SM = 0
Delta_r
Cytarabine
YML034W_1 SRC1
ZShift = 0.28
Z lm Score = −2.61
NG = 0
DB = 0
SM = 0
Delta_AUC
Cytarabine
YDL194W SNF3
ZShift = 2.57
Z lm Score = 2.11
NG = 0
DB = 0
SM = 0
Delta_L
Cytarabine
YDL194W SNF3
ZShift = 1.17
Z lm Score = −0.19
NG = 0
DB = 0
SM = 0
Delta_K
Cytarabine
YDL194W SNF3
ZShift = −0.78
Z lm Score = −0.05
NG = 0
DB = 0
SM = 0
Delta_r
Cytarabine
YDL194W SNF3
ZShift = −0.36
Z lm Score = −0.02
NG = 0
DB = 0
SM = 0
Delta_AUC
Cytarabine

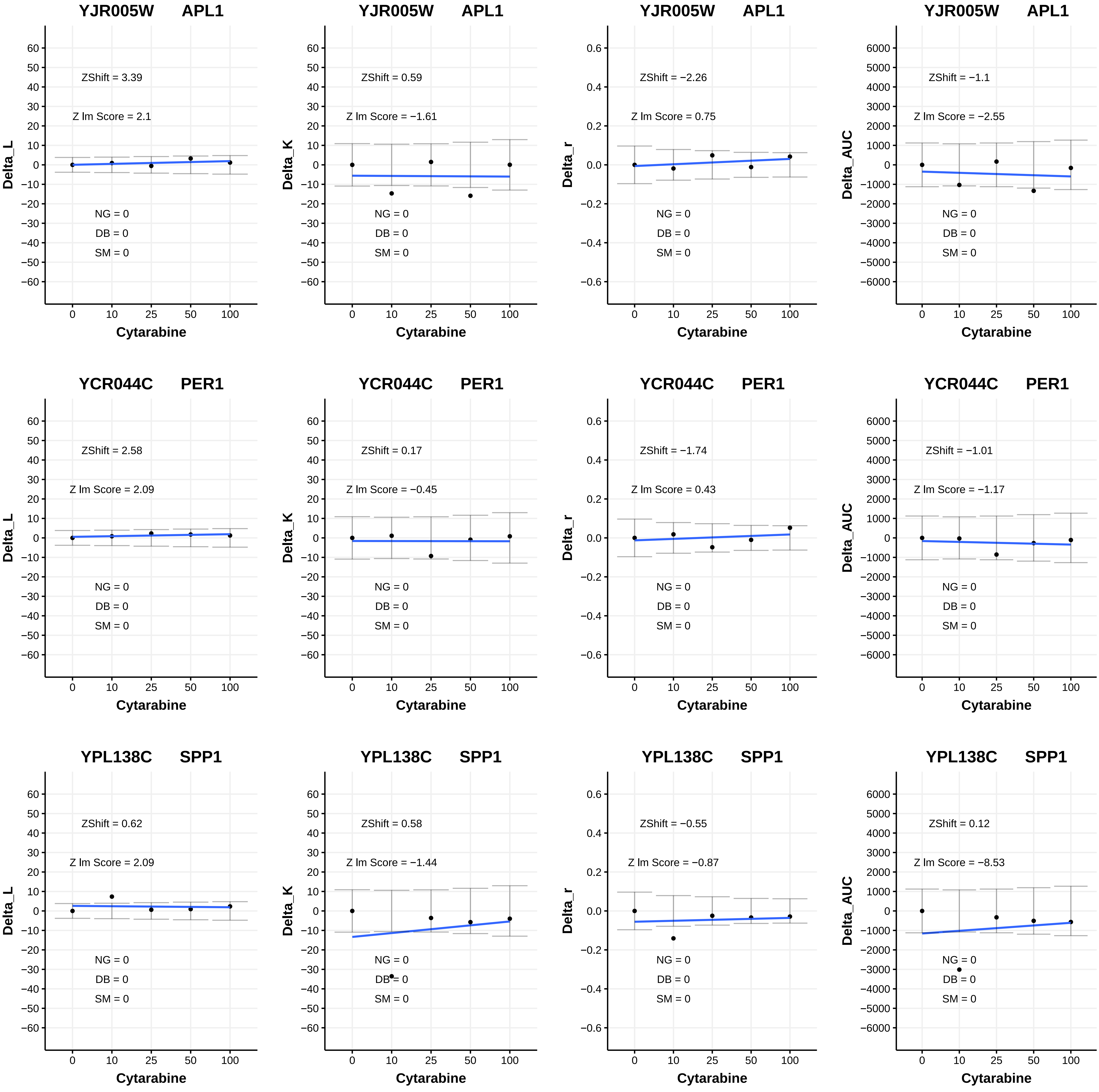

YJR005W APL1
ZShift = 3.39
Z lm Score = 2.1
NG = 0
DB = 0
SM = 0
Delta_L
Cytarabine
YJR005W APL1
ZShift = 0.59
Z lm Score = −1.61
NG = 0
DB = 0
SM = 0
Delta_K
Cytarabine
YJR005W APL1
ZShift = −2.26
Z lm Score = 0.75
NG = 0
DB = 0
SM = 0
Delta_r
Cytarabine
YJR005W APL1
ZShift = −1.1
Z lm Score = −2.55
NG = 0
DB = 0
SM = 0
Delta_AUC
Cytarabine
YCR044C PER1
ZShift = 2.58
Z lm Score = 2.09
NG = 0
DB = 0
SM = 0
Delta_L
Cytarabine
YCR044C PER1
ZShift = 0.17
Z lm Score = −0.45
NG = 0
DB = 0
SM = 0
Delta_K
Cytarabine
YCR044C PER1
ZShift = −1.74
Z lm Score = 0.43
NG = 0
DB = 0
SM = 0
Delta_r
Cytarabine
YCR044C PER1
ZShift = −1.01
Z lm Score = −1.17
NG = 0
DB = 0
SM = 0
Delta_AUC
Cytarabine
YPL138C SPP1
ZShift = 0.62
Z lm Score = 2.09
NG = 0
DB = 0
SM = 0
Delta_L
Cytarabine
YPL138C SPP1
ZShift = 0.58
Z lm Score = −1.44
NG = 0
DB = 0
SM = 0
Delta_K
Cytarabine
YPL138C SPP1
ZShift = −0.55
Z lm Score = −0.87
NG = 0
DB = 0
SM = 0
Delta_r
Cytarabine
YPL138C SPP1
ZShift = 0.12
Z lm Score = −8.53
NG = 0
DB = 0
SM = 0
Delta_AUC
Cytarabine

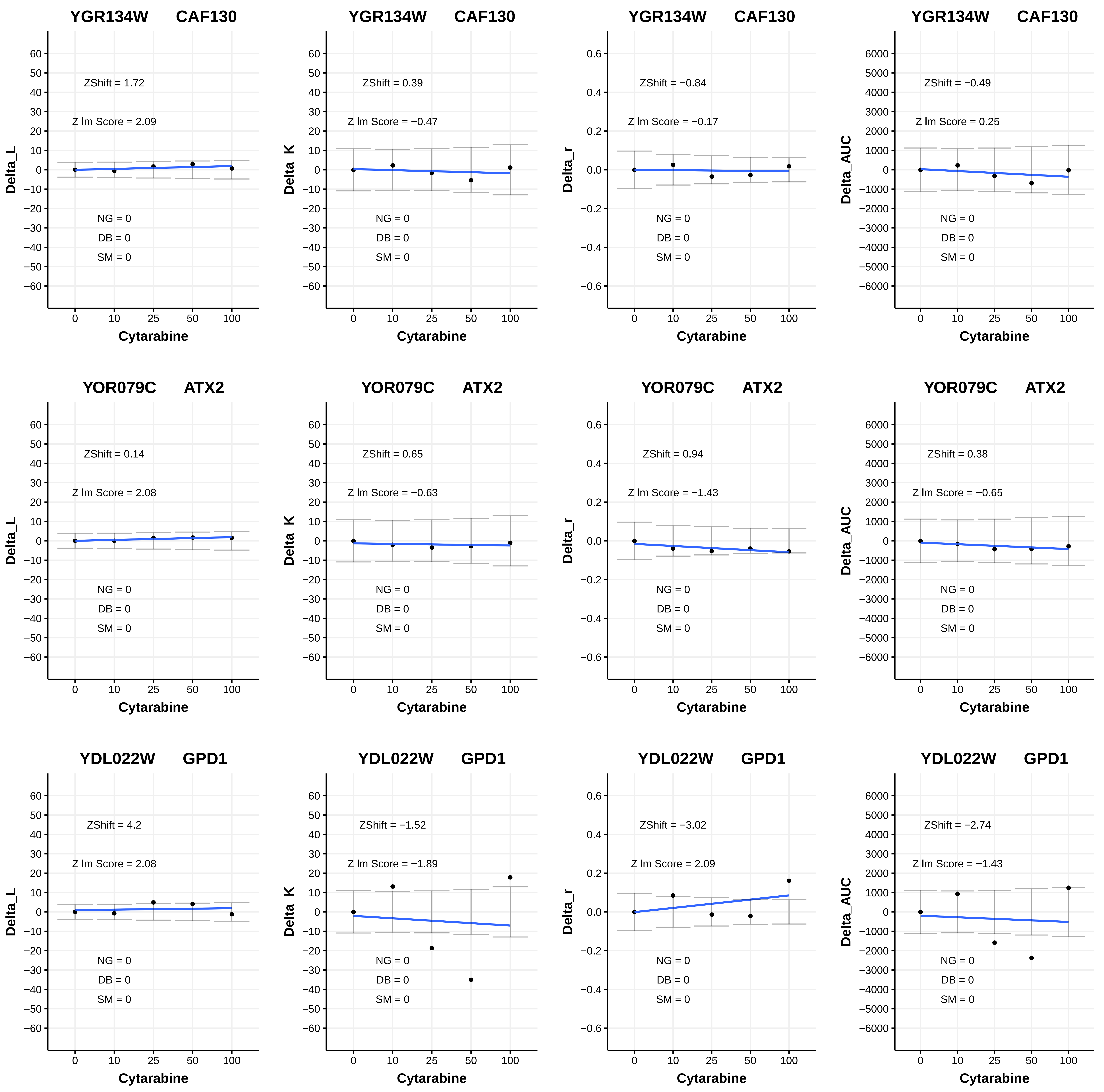
YGR134W CAF130
ZShift = 1.72
Z lm Score = 2.09
NG = 0
DB = 0
SM = 0
Delta_L
Cytarabine
YGR134W CAF130
ZShift = 0.39
Z lm Score = −0.47
NG = 0
DB = 0
SM = 0
Delta_K
Cytarabine
YGR134W CAF130
ZShift = −0.84
Z lm Score = −0.17
NG = 0
DB = 0
SM = 0
Delta_r
Cytarabine
YGR134W CAF130
ZShift = −0.49
Z lm Score = 0.25
NG = 0
DB = 0
SM = 0
Delta_AUC
Cytarabine
YOR079C ATX2
ZShift = 0.14
Z lm Score = 2.08
NG = 0
DB = 0
SM = 0
Delta_L
Cytarabine
YOR079C ATX2
ZShift = 0.65
Z lm Score = −0.63
NG = 0
DB = 0
SM = 0
Delta_K
Cytarabine
YOR079C ATX2
ZShift = 0.94
Z lm Score = −1.43
NG = 0
DB = 0
SM = 0
Delta_r
Cytarabine
YOR079C ATX2
ZShift = 0.38
Z lm Score = −0.65
NG = 0
DB = 0
SM = 0
Delta_AUC
Cytarabine
YDL022W GPD1
ZShift = 4.2
Z lm Score = 2.08
NG = 0
DB = 0
SM = 0
Delta_L
Cytarabine
YDL022W GPD1
ZShift = −1.52
Z lm Score = −1.89
NG = 0
DB = 0
SM = 0
Delta_K
Cytarabine
YDL022W GPD1
ZShift = −3.02
Z lm Score = 2.09
NG = 0
DB = 0
SM = 0
Delta_r
Cytarabine
YDL022W GPD1
ZShift = −2.74
Z lm Score = −1.43
NG = 0
DB = 0
SM = 0
Delta_AUC
Cytarabine

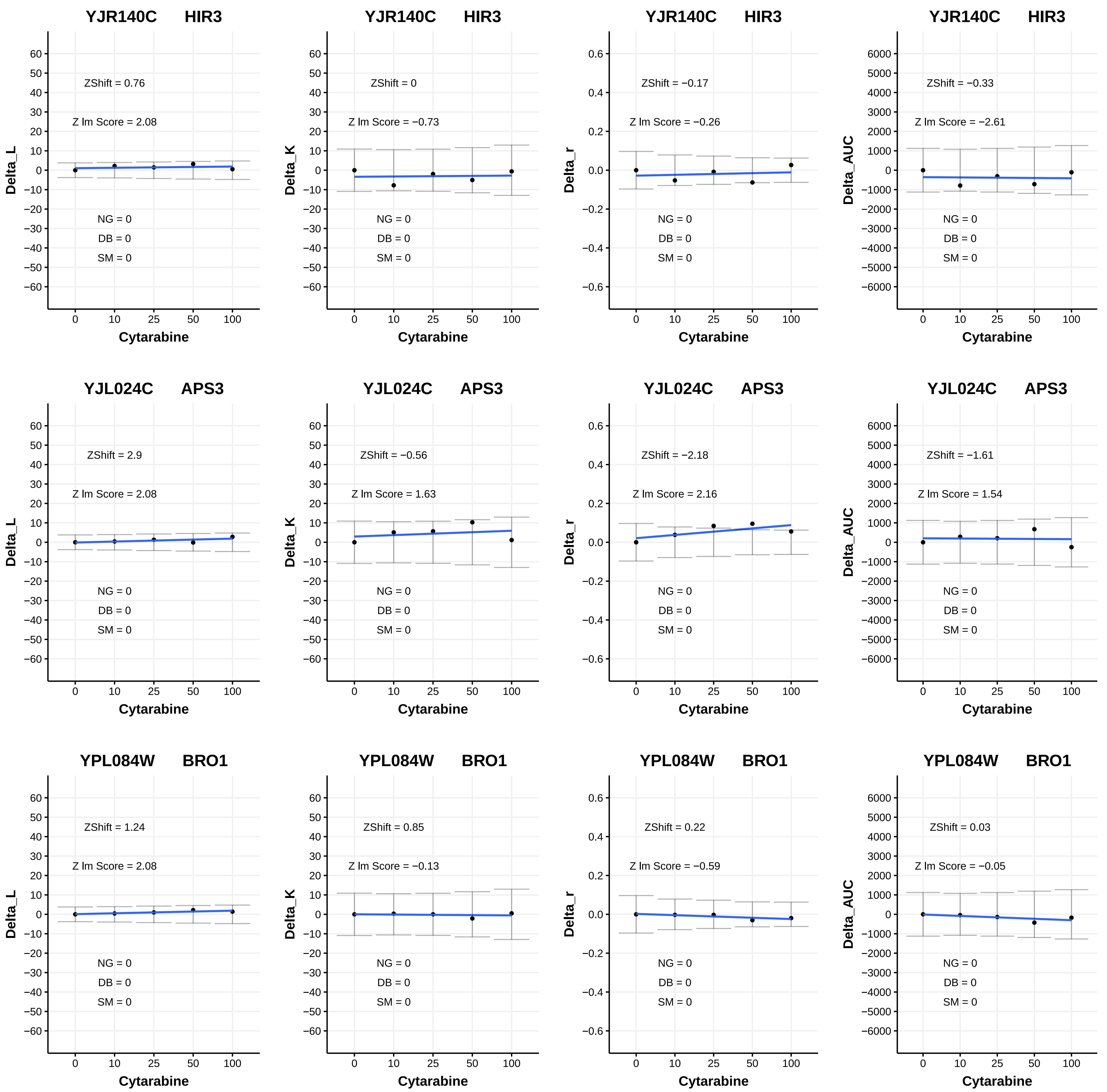

YJR140C HIR3
ZShift = 0.76
Z lm Score = 2.08
NG = 0
DB = 0
SM = 0
Delta_L
Cytarabine
YJR140C HIR3
ZShift = 0
Z lm Score = −0.73
NG = 0
DB = 0
SM = 0
Delta_K
Cytarabine
YJR140C HIR3
ZShift = −0.17
Z lm Score = −0.26
NG = 0
DB = 0
SM = 0
Delta_r
Cytarabine
YJR140C HIR3
ZShift = −0.33
Z lm Score = −2.61
NG = 0
DB = 0
SM = 0
Delta_AUC
Cytarabine
YJL024C APS3
ZShift = 2.9
Z lm Score = 2.08
NG = 0
DB = 0
SM = 0
Delta_L
Cytarabine
YJL024C APS3
ZShift = −0.56
Z lm Score = 1.63
NG = 0
DB = 0
SM = 0
Delta_K
Cytarabine
YJL024C APS3
ZShift = −2.18
Z lm Score = 2.16
NG = 0
DB = 0
SM = 0
Delta_r
Cytarabine
YJL024C APS3
ZShift = −1.61
Z lm Score = 1.54
NG = 0
DB = 0
SM = 0
Delta_AUC
Cytarabine
YPL084W BRO1
ZShift = 1.24
Z lm Score = 2.08
NG = 0
DB = 0
SM = 0
Delta_L
Cytarabine
YPL084W BRO1
ZShift = 0.85
Z lm Score = −0.13
NG = 0
DB = 0
SM = 0
Delta_K
Cytarabine
YPL084W BRO1
ZShift = 0.22
Z lm Score = −0.59
NG = 0
DB = 0
SM = 0
Delta_r
Cytarabine
YPL084W BRO1
ZShift = 0.03
Z lm Score = −0.05
NG = 0
DB = 0
SM = 0
Delta_AUC
Cytarabine

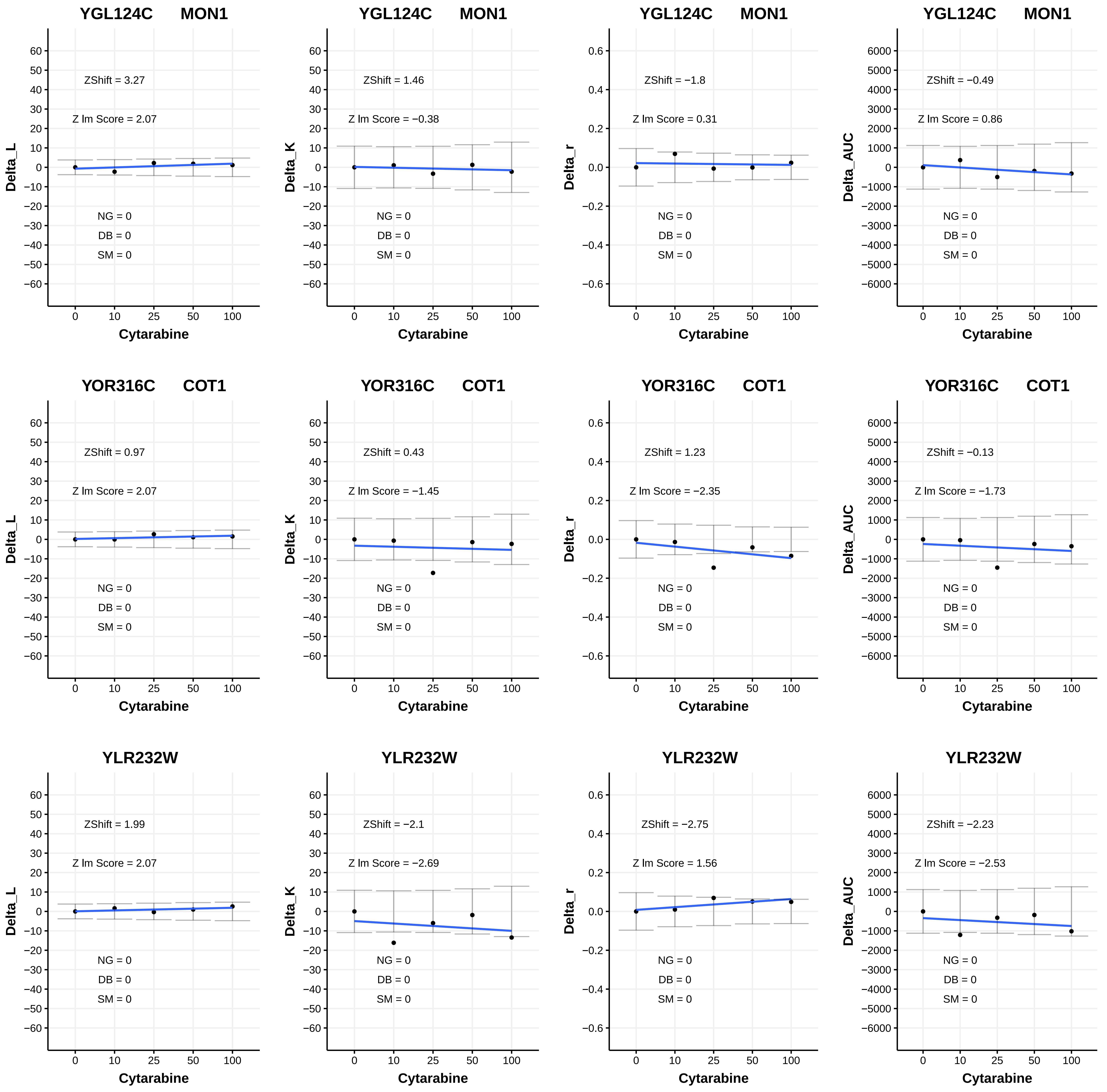
YGL124C MON1
ZShift = 3.27
Z lm Score = 2.07
NG = 0
DB = 0
SM = 0
Delta_L
Cytarabine
YGL124C MON1
ZShift = 1.46
Z lm Score = −0.38
NG = 0
DB = 0
SM = 0
Delta_K
Cytarabine
YGL124C MON1
ZShift = −1.8
Z lm Score = 0.31
NG = 0
DB = 0
SM = 0
Delta_r
Cytarabine
YGL124C MON1
ZShift = −0.49
Z lm Score = 0.86
NG = 0
DB = 0
SM = 0
Delta_AUC
Cytarabine
YOR316C COT1
ZShift = 0.97
Z lm Score = 2.07
NG = 0
DB = 0
SM = 0
Delta_L
Cytarabine
YOR316C COT1
ZShift = 0.43
Z lm Score = −1.45
NG = 0
DB = 0
SM = 0
Delta_K
Cytarabine
YOR316C COT1
ZShift = 1.23
Z lm Score = −2.35
NG = 0
DB = 0
SM = 0
Delta_r
Cytarabine
YOR316C COT1
ZShift = −0.13
Z lm Score = −1.73
NG = 0
DB = 0
SM = 0
Delta_AUC
Cytarabine
YLR232W
ZShift = 1.99
Z lm Score = 2.07
NG = 0
DB = 0
SM = 0
Delta_L
Cytarabine
YLR232W
ZShift = −2.1
Z lm Score = −2.69
NG = 0
DB = 0
SM = 0
Delta_K
Cytarabine
YLR232W
ZShift = −2.75
Z lm Score = 1.56
NG = 0
DB = 0
SM = 0
Delta_r
Cytarabine
YLR232W
ZShift = −2.23
Z lm Score = −2.53
NG = 0
DB = 0
SM = 0
Delta_AUC
Cytarabine

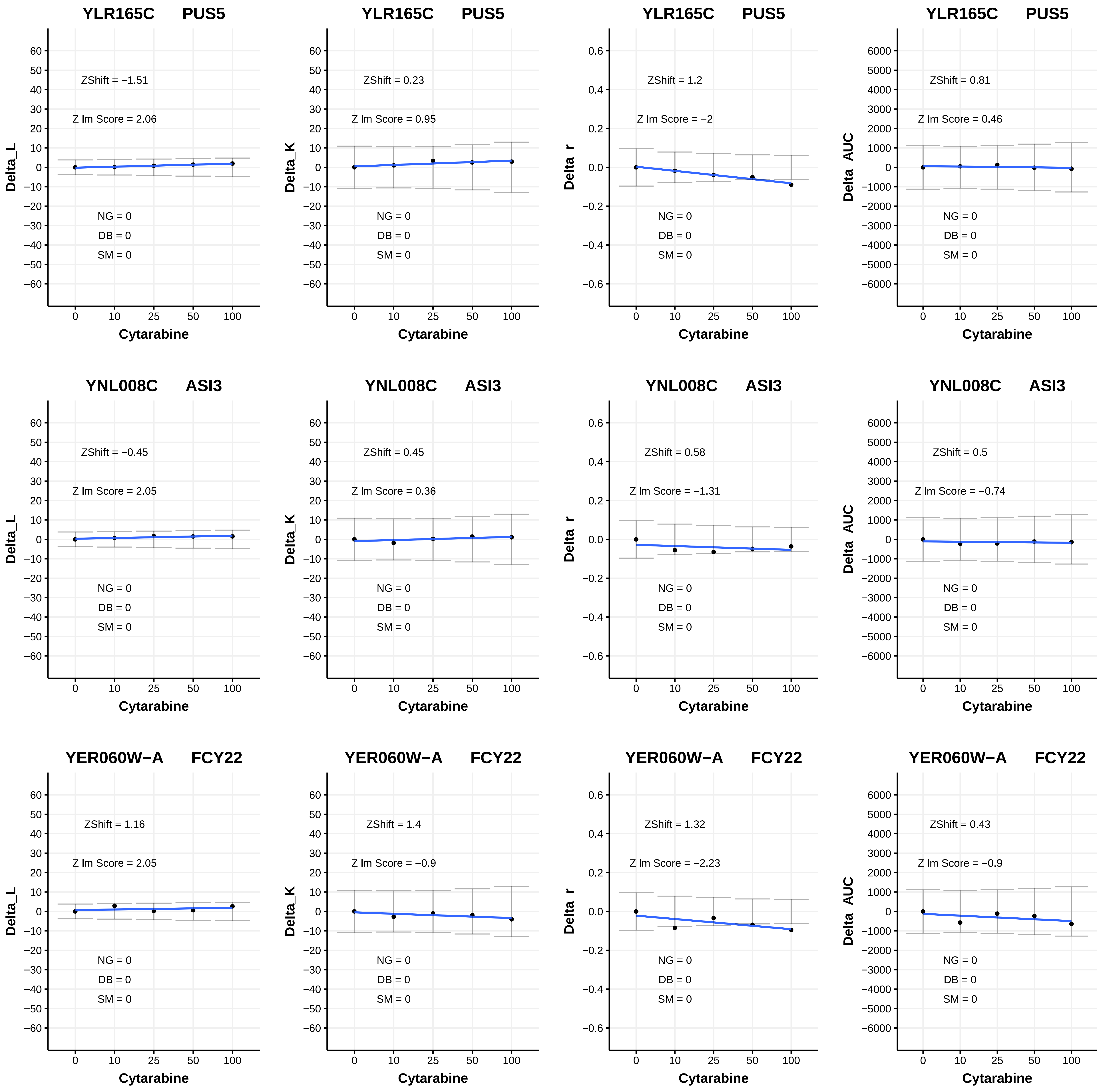
YLR165C PUS5
ZShift = −1.51
Z lm Score = 2.06
NG = 0
DB = 0
SM = 0
Delta_L
Cytarabine
YLR165C PUS5
ZShift = 0.23
Z lm Score = 0.95
NG = 0
DB = 0
SM = 0
Delta_K
Cytarabine
YLR165C PUS5
ZShift = 1.2
Z lm Score = −2
NG = 0
DB = 0
SM = 0
Delta_r
Cytarabine
YLR165C PUS5
ZShift = 0.81
Z lm Score = 0.46
NG = 0
DB = 0
SM = 0
Delta_AUC
Cytarabine
YNL008C ASI3
ZShift = −0.45
Z lm Score = 2.05
NG = 0
DB = 0
SM = 0
Delta_L
Cytarabine
YNL008C ASI3
ZShift = 0.45
Z lm Score = 0.36
NG = 0
DB = 0
SM = 0
Delta_K
Cytarabine
YNL008C ASI3
ZShift = 0.58
Z lm Score = −1.31
NG = 0
DB = 0
SM = 0
Delta_r
Cytarabine
YNL008C ASI3
ZShift = 0.5
Z lm Score = −0.74
NG = 0
DB = 0
SM = 0
Delta_AUC
Cytarabine
YER060W−A FCY22
ZShift = 1.16
Z lm Score = 2.05
NG = 0
DB = 0
SM = 0
Delta_L
Cytarabine
YER060W−A FCY22
ZShift = 1.4
Z lm Score = −0.9
NG = 0
DB = 0
SM = 0
Delta_K
Cytarabine
YER060W−A FCY22
ZShift = 1.32
Z lm Score = −2.23
NG = 0
DB = 0
SM = 0
Delta_r
Cytarabine
YER060W−A FCY22
ZShift = 0.43
Z lm Score = −0.9
NG = 0
DB = 0
SM = 0
Delta_AUC
Cytarabine

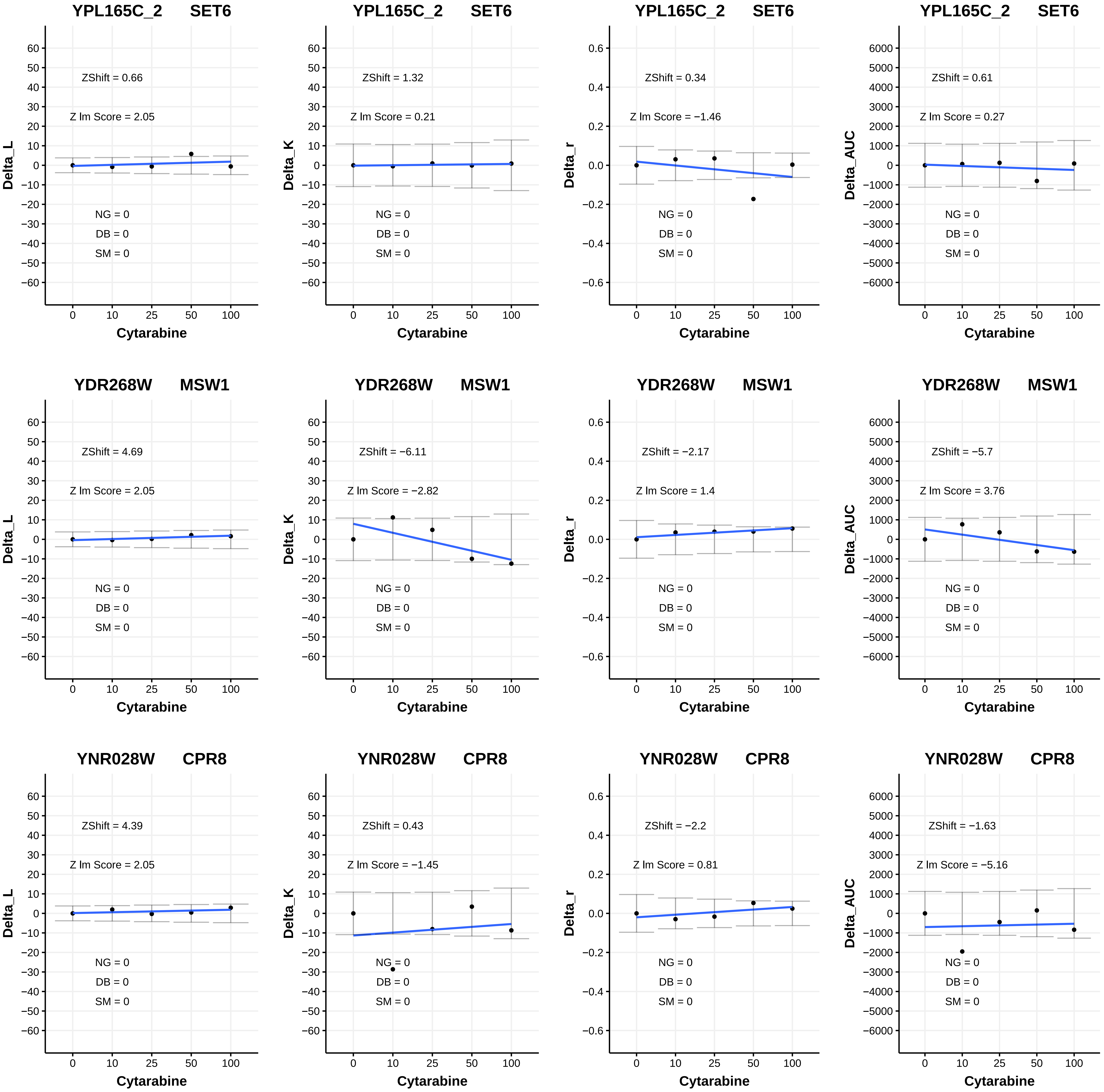

YPL165C_2 SET6
ZShift = 0.66
Z lm Score = 2.05
NG = 0
DB = 0
SM = 0
Delta_L
Cytarabine
YPL165C_2 SET6
ZShift = 1.32
Z lm Score = 0.21
NG = 0
DB = 0
SM = 0
Delta_K
Cytarabine
YPL165C_2 SET6
ZShift = 0.34
Z lm Score = −1.46
NG = 0
DB = 0
SM = 0
Delta_r
Cytarabine
YPL165C_2 SET6
ZShift = 0.61
Z lm Score = 0.27
NG = 0
DB = 0
SM = 0
Delta_AUC
Cytarabine
YDR268W MSW1
ZShift = 4.69
Z lm Score = 2.05
NG = 0
DB = 0
SM = 0
Delta_L
Cytarabine
YDR268W MSW1
ZShift = −6.11
Z lm Score = −2.82
NG = 0
DB = 0
SM = 0
Delta_K
Cytarabine
YDR268W MSW1
ZShift = −2.17
Z lm Score = 1.4
NG = 0
DB = 0
SM = 0
Delta_r
Cytarabine
YDR268W MSW1
ZShift = −5.7
Z lm Score = 3.76
NG = 0
DB = 0
SM = 0
Delta_AUC
Cytarabine
YNR028W CPR8
ZShift = 4.39
Z lm Score = 2.05
NG = 0
DB = 0
SM = 0
Delta_L
Cytarabine
YNR028W CPR8
ZShift = 0.43
Z lm Score = −1.45
NG = 0
DB = 0
SM = 0
Delta_K
Cytarabine
YNR028W CPR8
ZShift = −2.2
Z lm Score = 0.81
NG = 0
DB = 0
SM = 0
Delta_r
Cytarabine
YNR028W CPR8
ZShift = −1.63
Z lm Score = −5.16
NG = 0
DB = 0
SM = 0
Delta_AUC
Cytarabine

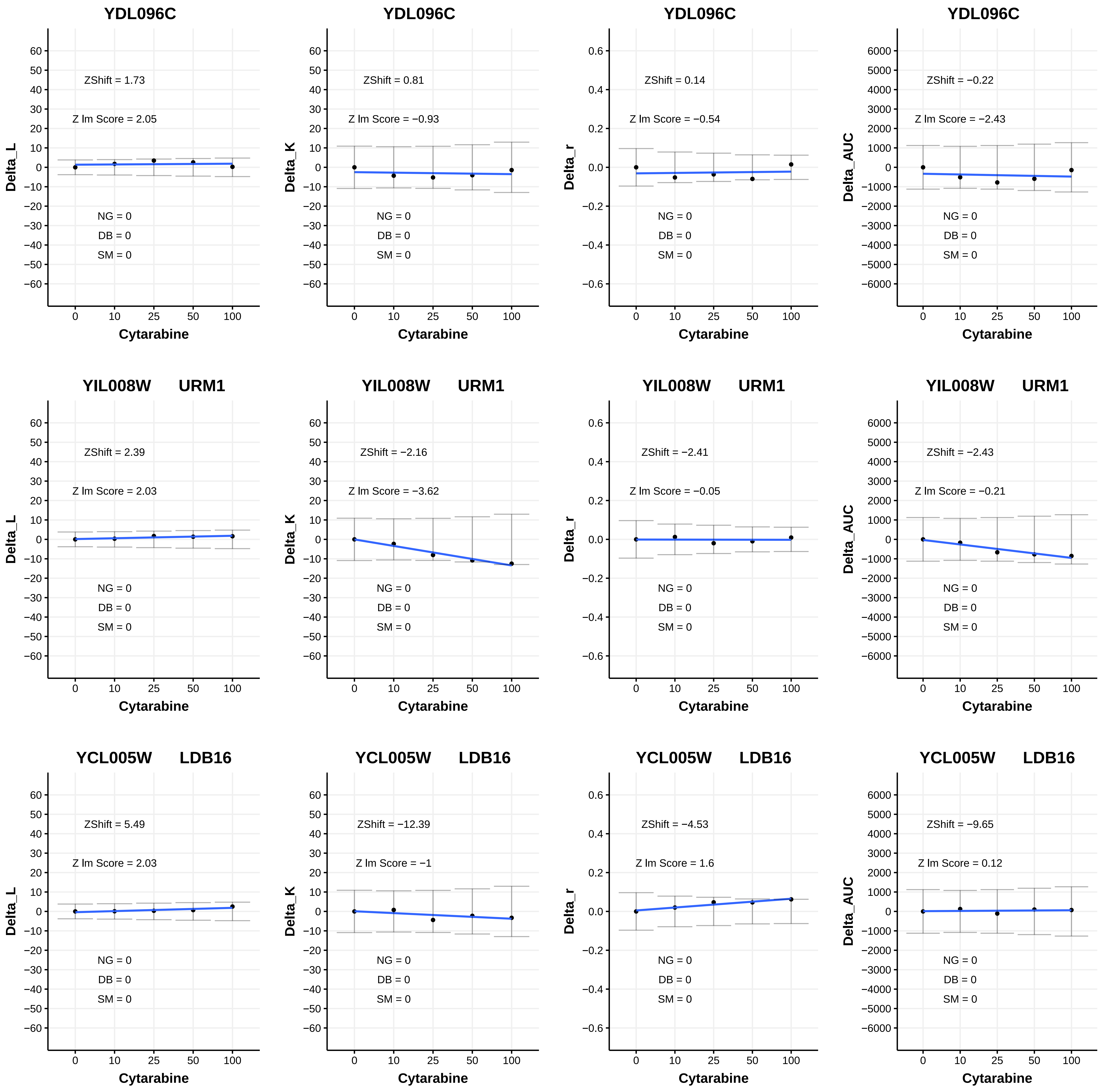
YDL096C
ZShift = 1.73
Z lm Score = 2.05
NG = 0
DB = 0
SM = 0
Delta_L
Cytarabine
YDL096C
ZShift = 0.81
Z lm Score = −0.93
NG = 0
DB = 0
SM = 0
Delta_K
Cytarabine
YDL096C
ZShift = 0.14
Z lm Score = −0.54
NG = 0
DB = 0
SM = 0
Delta_r
Cytarabine
YDL096C
ZShift = −0.22
Z lm Score = −2.43
NG = 0
DB = 0
SM = 0
Delta_AUC
Cytarabine
YIL008W URM1
ZShift = 2.39
Z lm Score = 2.03
NG = 0
DB = 0
SM = 0
Delta_L
Cytarabine
YIL008W URM1
ZShift = −2.16
Z lm Score = −3.62
NG = 0
DB = 0
SM = 0
Delta_K
Cytarabine
YIL008W URM1
ZShift = −2.41
Z lm Score = −0.05
NG = 0
DB = 0
SM = 0
Delta_r
Cytarabine
YIL008W URM1
ZShift = −2.43
Z lm Score = −0.21
NG = 0
DB = 0
SM = 0
Delta_AUC
Cytarabine
YCL005W LDB16
ZShift = 5.49
Z lm Score = 2.03
NG = 0
DB = 0
SM = 0
Delta_L
Cytarabine
YCL005W LDB16
ZShift = −12.39
Z lm Score = −1
NG = 0
DB = 0
SM = 0
Delta_K
Cytarabine
YCL005W LDB16
ZShift = −4.53
Z lm Score = 1.6
NG = 0
DB = 0
SM = 0
Delta_r
Cytarabine
YCL005W LDB16
ZShift = −9.65
Z lm Score = 0.12
NG = 0
DB = 0
SM = 0
Delta_AUC
Cytarabine

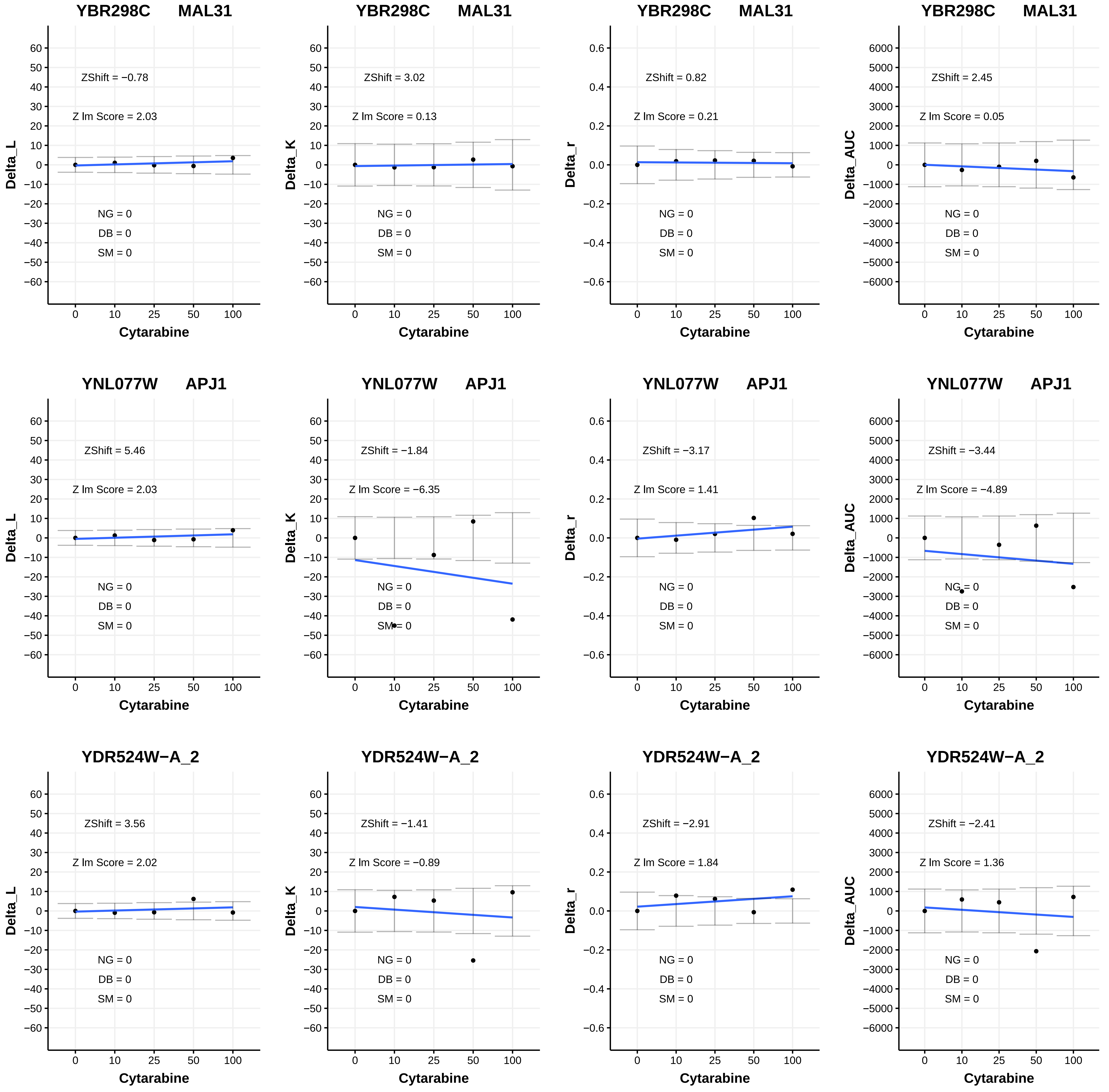

YBR298C MAL31
ZShift = −0.78
Z lm Score = 2.03
ZShift = 3.02
Z lm Score = 0.13
ZShift = 0.82
Z lm Score = 0.21
ZShift = 2.45
Z lm Score = 0.05
YNL077W APJ1
ZShift = 5.46
Z lm Score = 2.03
ZShift = −1.84
Z lm Score = −6.35
ZShift = −3.17
Z lm Score = 1.41
ZShift = −3.44
Z lm Score = −4.89
YDR524W−A_2
ZShift = 3.56
Z lm Score = 2.02
ZShift = −1.41
Z lm Score = −0.89
ZShift = −2.91
Z lm Score = 1.84
ZShift = −2.41
Z lm Score = 1.36
NG = 0
DB = 0
SM = 0
Delta_L
Delta_K
Delta_r
Delta_AUC
Cytarabine
0
10
25
50
100

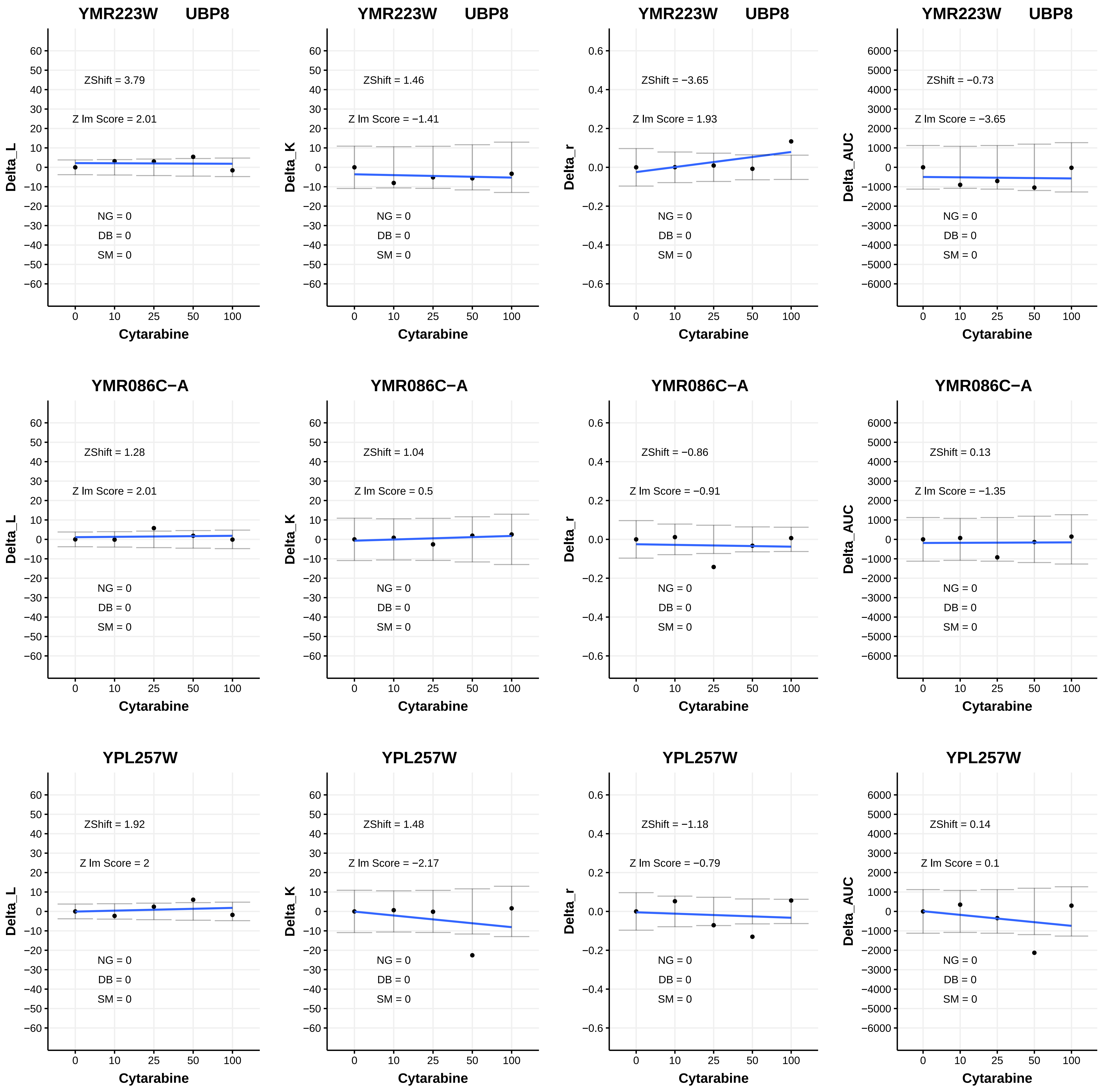

YMR223W UBP8
ZShift = 3.79
Z lm Score = 2.01
NG = 0
DB = 0
SM = 0
Delta_L
Cytarabine
YMR223W UBP8
ZShift = 1.46
Z lm Score = −1.41
NG = 0
DB = 0
SM = 0
Delta_K
Cytarabine
YMR223W UBP8
ZShift = −3.65
Z lm Score = 1.93
NG = 0
DB = 0
SM = 0
Delta_r
Cytarabine
YMR223W UBP8
ZShift = −0.73
Z lm Score = −3.65
NG = 0
DB = 0
SM = 0
Delta_AUC
Cytarabine
YMR086C−A
ZShift = 1.28
Z lm Score = 2.01
NG = 0
DB = 0
SM = 0
Delta_L
Cytarabine
YMR086C−A
ZShift = 1.04
Z lm Score = 0.5
NG = 0
DB = 0
SM = 0
Delta_K
Cytarabine
YMR086C−A
ZShift = −0.86
Z lm Score = −0.91
NG = 0
DB = 0
SM = 0
Delta_r
Cytarabine
YMR086C−A
ZShift = 0.13
Z lm Score = −1.35
NG = 0
DB = 0
SM = 0
Delta_AUC
Cytarabine
YPL257W
ZShift = 1.92
Z lm Score = 2
NG = 0
DB = 0
SM = 0
Delta_L
Cytarabine
YPL257W
ZShift = 1.48
Z lm Score = −2.17
NG = 0
DB = 0
SM = 0
Delta_K
Cytarabine
YPL257W
ZShift = −1.18
Z lm Score = −0.79
NG = 0
DB = 0
SM = 0
Delta_r
Cytarabine
YPL257W
ZShift = 0.14
Z lm Score = 0.1
NG = 0
DB = 0
SM = 0
Delta_AUC
Cytarabine

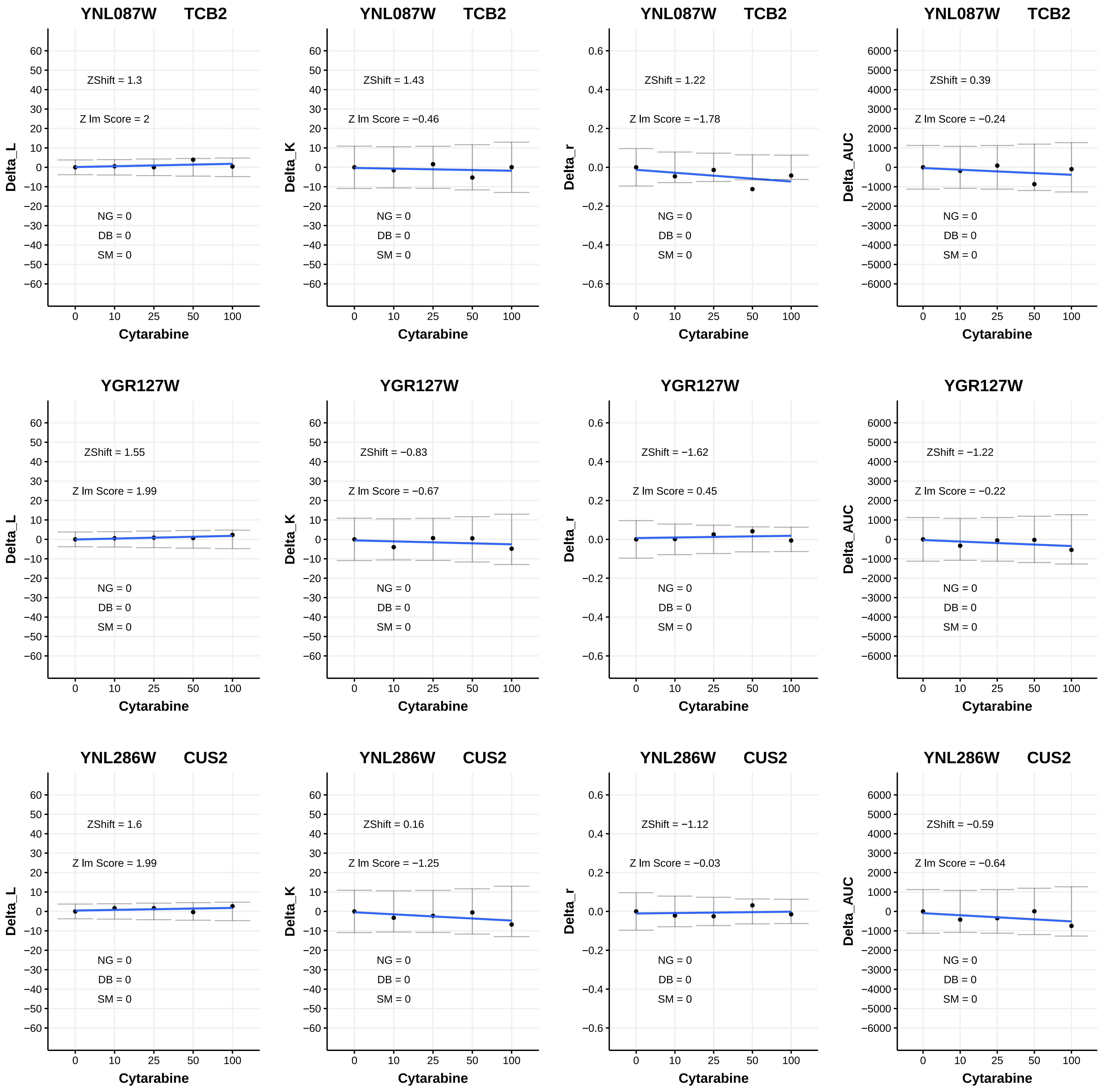
YNL087W TCB2
Delta_L
ZShift = 1.3
Z lm Score = 2
NG = 0
DB = 0
SM = 0
Cytarabine
YNL087W TCB2
Delta_K
ZShift = 1.43
Z lm Score = −0.46
NG = 0
DB = 0
SM = 0
Cytarabine
YNL087W TCB2
Delta_r
ZShift = 1.22
Z lm Score = −1.78
NG = 0
DB = 0
SM = 0
Cytarabine
YNL087W TCB2
Delta_AUC
ZShift = 0.39
Z lm Score = −0.24
NG = 0
DB = 0
SM = 0
Cytarabine
YGR127W
Delta_L
ZShift = 1.55
Z lm Score = 1.99
NG = 0
DB = 0
SM = 0
Cytarabine
YGR127W
Delta_K
ZShift = −0.83
Z lm Score = −0.67
NG = 0
DB = 0
SM = 0
Cytarabine
YGR127W
Delta_r
ZShift = −1.62
Z lm Score = 0.45
NG = 0
DB = 0
SM = 0
Cytarabine
YGR127W
Delta_AUC
ZShift = −1.22
Z lm Score = −0.22
NG = 0
DB = 0
SM = 0
Cytarabine
YNL286W CUS2
Delta_L
ZShift = 1.6
Z lm Score = 1.99
NG = 0
DB = 0
SM = 0
Cytarabine
YNL286W CUS2
Delta_K
ZShift = 0.16
Z lm Score = −1.25
NG = 0
DB = 0
SM = 0
Cytarabine
YNL286W CUS2
Delta_r
ZShift = −1.12
Z lm Score = −0.03
NG = 0
DB = 0
SM = 0
Cytarabine
YNL286W CUS2
Delta_AUC
ZShift = −0.59
Z lm Score = −0.64
NG = 0
DB = 0
SM = 0
Cytarabine

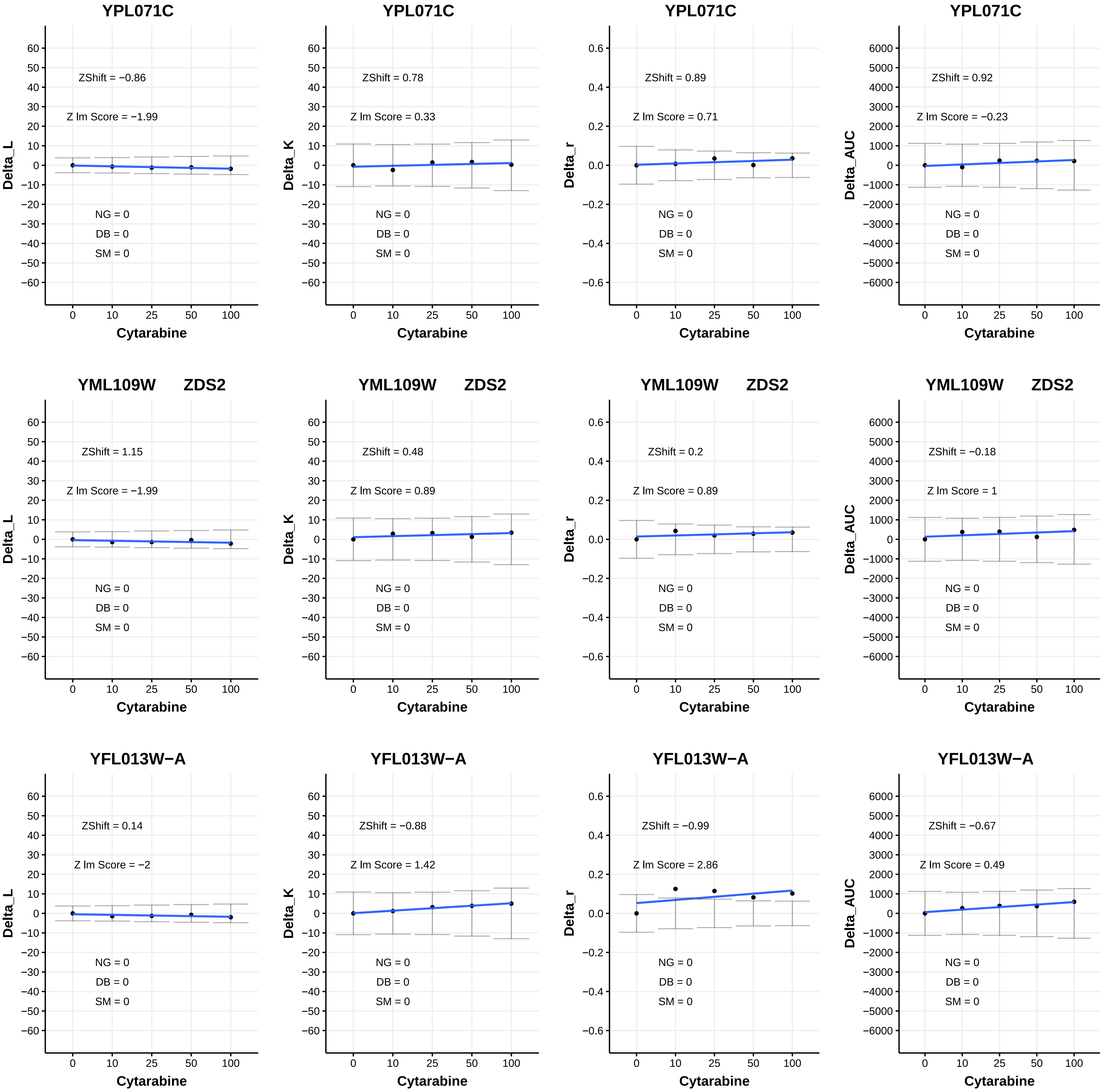

YPL071C
Delta_L
ZShift = −0.86
Z lm Score = −1.99
NG = 0
DB = 0
SM = 0
Cytarabine
YPL071C
Delta_K
ZShift = 0.78
Z lm Score = 0.33
NG = 0
DB = 0
SM = 0
Cytarabine
YPL071C
Delta_r
ZShift = 0.89
Z lm Score = 0.71
NG = 0
DB = 0
SM = 0
Cytarabine
YPL071C
Delta_AUC
ZShift = 0.92
Z lm Score = −0.23
NG = 0
DB = 0
SM = 0
Cytarabine
YML109W ZDS2
Delta_L
ZShift = 1.15
Z lm Score = −1.99
NG = 0
DB = 0
SM = 0
Cytarabine
YML109W ZDS2
Delta_K
ZShift = 0.48
Z lm Score = 0.89
NG = 0
DB = 0
SM = 0
Cytarabine
YML109W ZDS2
Delta_r
ZShift = 0.2
Z lm Score = 0.89
NG = 0
DB = 0
SM = 0
Cytarabine
YML109W ZDS2
Delta_AUC
ZShift = −0.18
Z lm Score = 1
NG = 0
DB = 0
SM = 0
Cytarabine
YFL013W−A
Delta_L
ZShift = 0.14
Z lm Score = −2
NG = 0
DB = 0
SM = 0
Cytarabine
YFL013W−A
Delta_K
ZShift = −0.88
Z lm Score = 1.42
NG = 0
DB = 0
SM = 0
Cytarabine
YFL013W−A
Delta_r
ZShift = −0.99
Z lm Score = 2.86
NG = 0
DB = 0
SM = 0
Cytarabine
YFL013W−A
Delta_AUC
ZShift = −0.67
Z lm Score = 0.49
NG = 0
DB = 0
SM = 0
Cytarabine

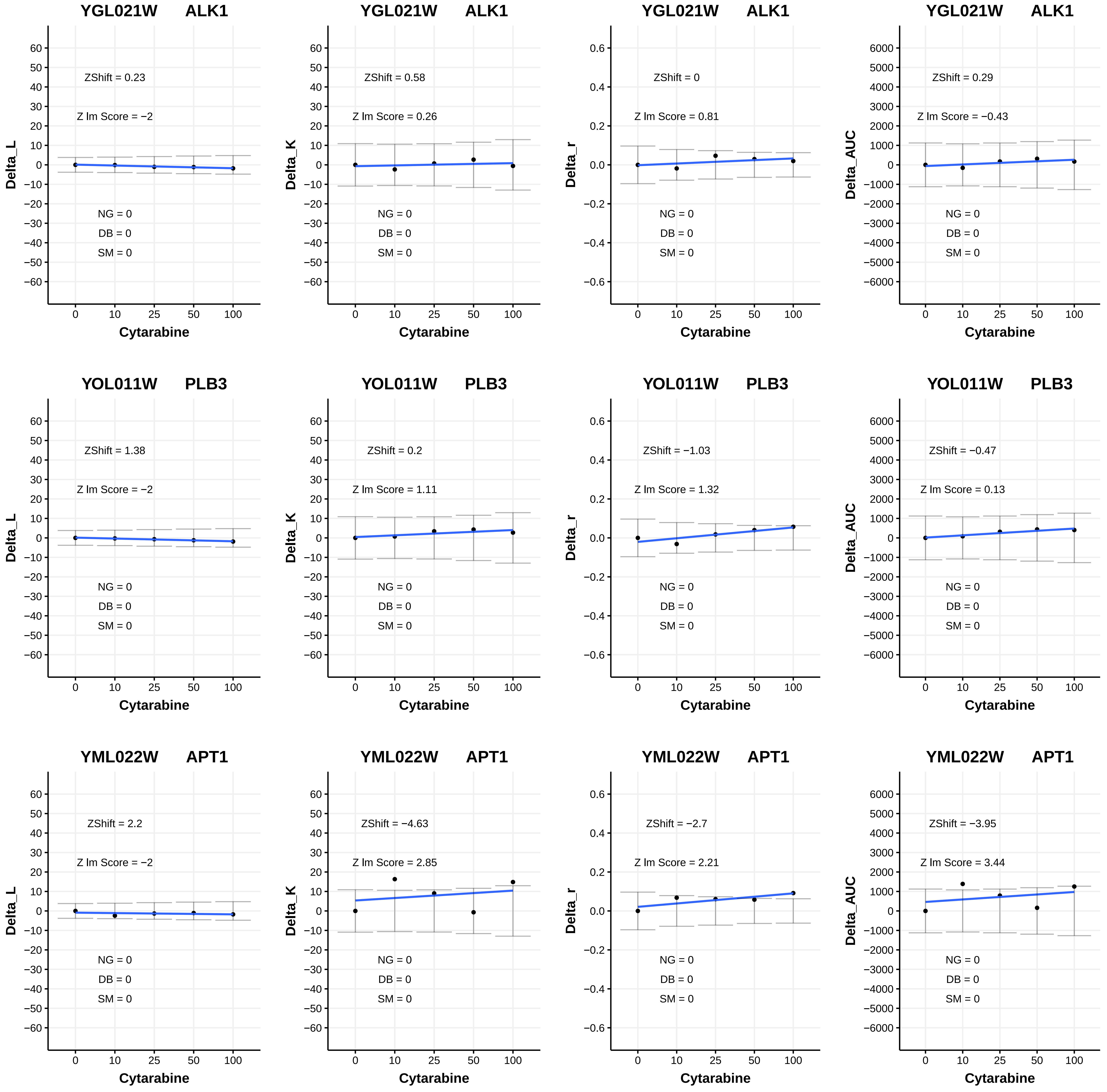
YGL021W ALK1
ZShift = 0.23
Z lm Score = −2
NG = 0
DB = 0
SM = 0
Delta_L
Cytarabine
YGL021W ALK1
ZShift = 0.58
Z lm Score = 0.26
NG = 0
DB = 0
SM = 0
Delta_K
Cytarabine
YGL021W ALK1
ZShift = 0
Z lm Score = 0.81
NG = 0
DB = 0
SM = 0
Delta_r
Cytarabine
YGL021W ALK1
ZShift = 0.29
Z lm Score = −0.43
NG = 0
DB = 0
SM = 0
Delta_AUC
Cytarabine
YOL011W PLB3
ZShift = 1.38
Z lm Score = −2
NG = 0
DB = 0
SM = 0
Delta_L
Cytarabine
YOL011W PLB3
ZShift = 0.2
Z lm Score = 1.11
NG = 0
DB = 0
SM = 0
Delta_K
Cytarabine
YOL011W PLB3
ZShift = −1.03
Z lm Score = 1.32
NG = 0
DB = 0
SM = 0
Delta_r
Cytarabine
YOL011W PLB3
ZShift = −0.47
Z lm Score = 0.13
NG = 0
DB = 0
SM = 0
Delta_AUC
Cytarabine
YML022W APT1
ZShift = 2.2
Z lm Score = −2
NG = 0
DB = 0
SM = 0
Delta_L
Cytarabine
YML022W APT1
ZShift = −4.63
Z lm Score = 2.85
NG = 0
DB = 0
SM = 0
Delta_K
Cytarabine
YML022W APT1
ZShift = −2.7
Z lm Score = 2.21
NG = 0
DB = 0
SM = 0
Delta_r
Cytarabine
YML022W APT1
ZShift = −3.95
Z lm Score = 3.44
NG = 0
DB = 0
SM = 0
Delta_AUC
Cytarabine

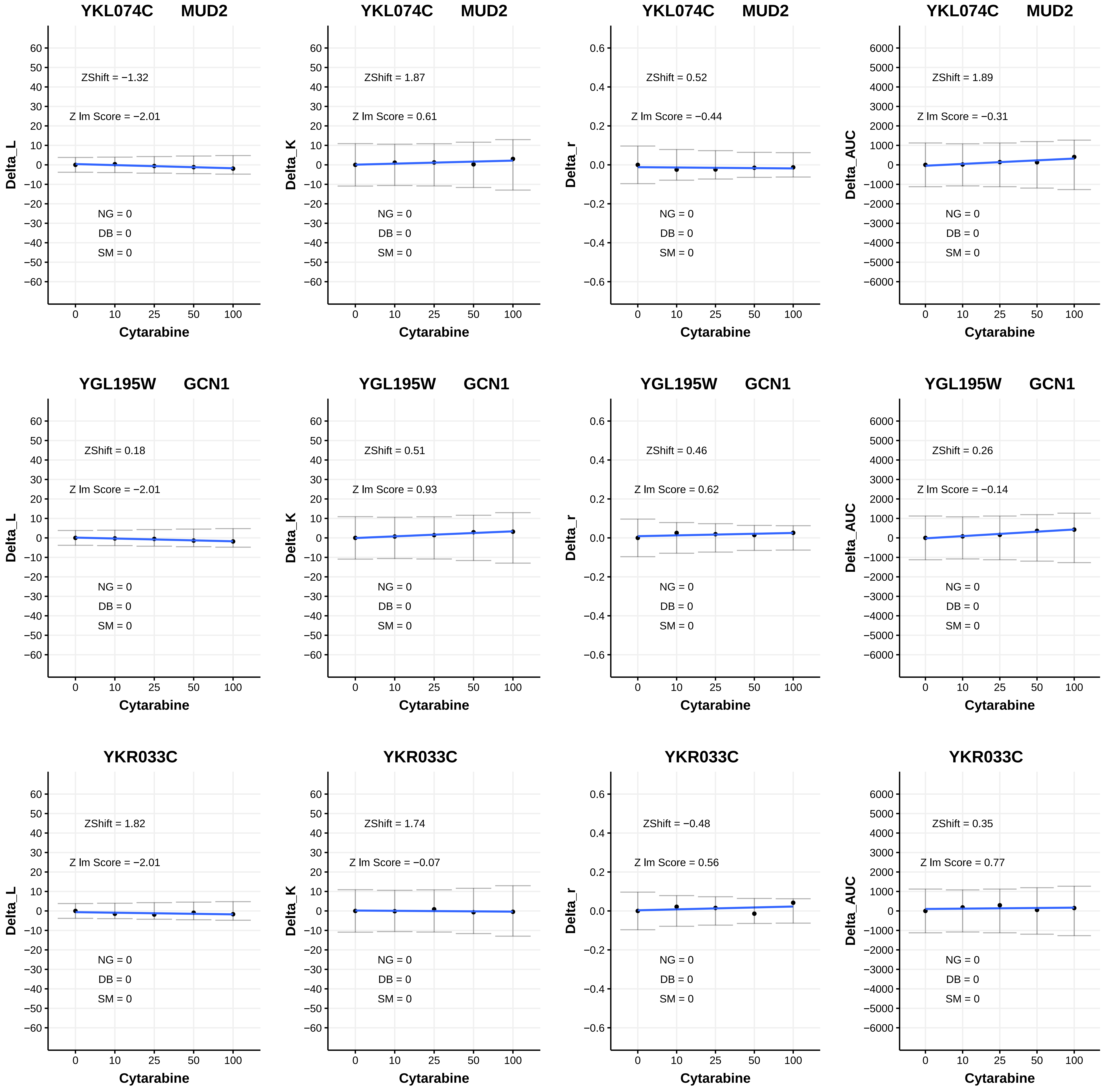

YKL074C MUD2
ZShift = −1.32
Z lm Score = −2.01
NG = 0
DB = 0
SM = 0
Delta_L
Cytarabine
YKL074C MUD2
ZShift = 1.87
Z lm Score = 0.61
NG = 0
DB = 0
SM = 0
Delta_K
Cytarabine
YKL074C MUD2
ZShift = 0.52
Z lm Score = −0.44
NG = 0
DB = 0
SM = 0
Delta_r
Cytarabine
YKL074C MUD2
ZShift = 1.89
Z lm Score = −0.31
NG = 0
DB = 0
SM = 0
Delta_AUC
Cytarabine
YGL195W GCN1
ZShift = 0.18
Z lm Score = −2.01
NG = 0
DB = 0
SM = 0
Delta_L
Cytarabine
YGL195W GCN1
ZShift = 0.51
Z lm Score = 0.93
NG = 0
DB = 0
SM = 0
Delta_K
Cytarabine
YGL195W GCN1
ZShift = 0.46
Z lm Score = 0.62
NG = 0
DB = 0
SM = 0
Delta_r
Cytarabine
YGL195W GCN1
ZShift = 0.26
Z lm Score = −0.14
NG = 0
DB = 0
SM = 0
Delta_AUC
Cytarabine
YKR033C
ZShift = 1.82
Z lm Score = −2.01
NG = 0
DB = 0
SM = 0
Delta_L
Cytarabine
YKR033C
ZShift = 1.74
Z lm Score = −0.07
NG = 0
DB = 0
SM = 0
Delta_K
Cytarabine
YKR033C
ZShift = −0.48
Z lm Score = 0.56
NG = 0
DB = 0
SM = 0
Delta_r
Cytarabine
YKR033C
ZShift = 0.35
Z lm Score = 0.77
NG = 0
DB = 0
SM = 0
Delta_AUC
Cytarabine

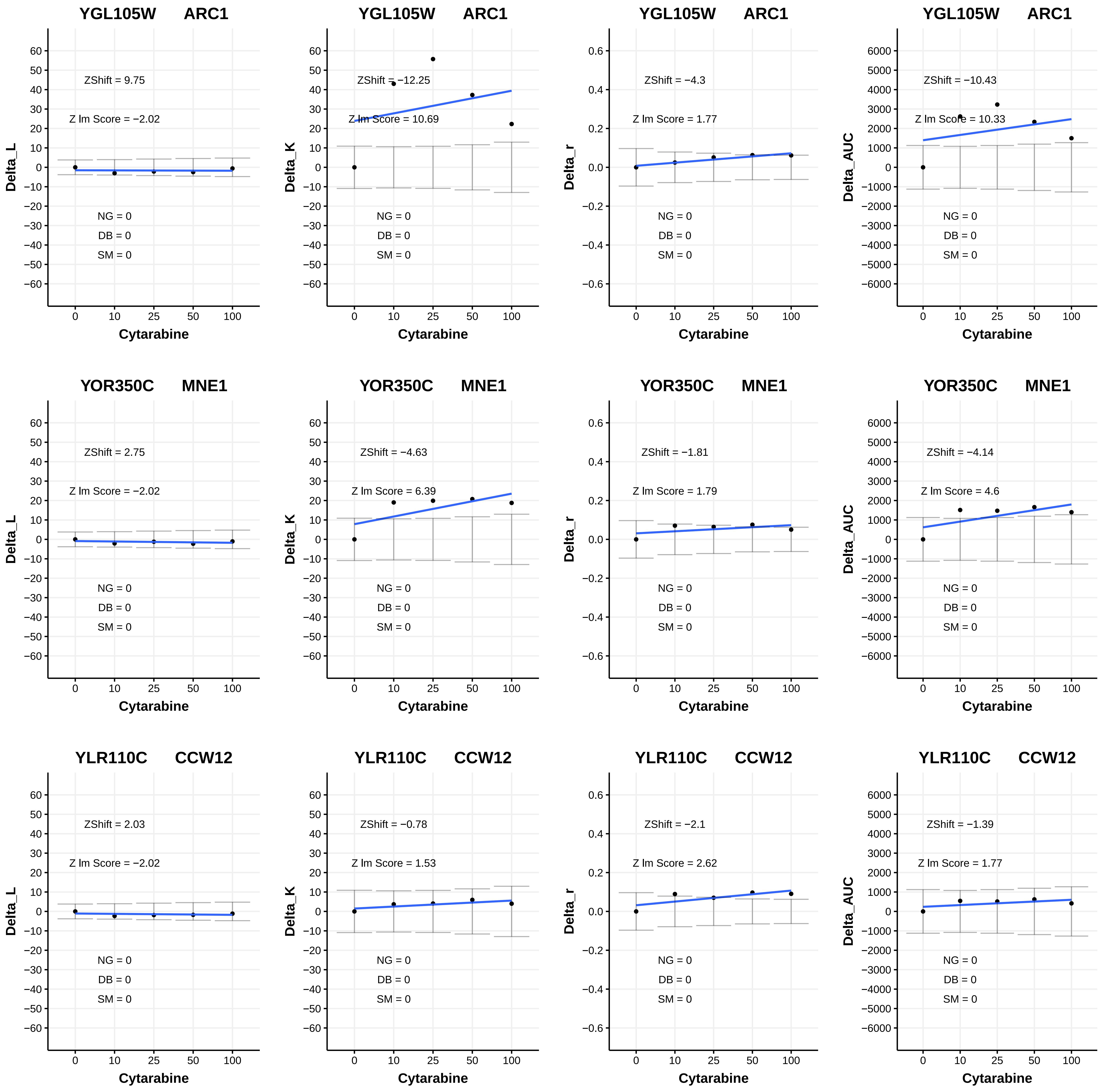

YGL105W ARC1
ZShift = 9.75
Z lm Score = −2.02
NG = 0
DB = 0
SM = 0
Delta_L
Cytarabine
YGL105W ARC1
ZShift = −12.25
Z lm Score = 10.69
NG = 0
DB = 0
SM = 0
Delta_K
Cytarabine
YGL105W ARC1
ZShift = −4.3
Z lm Score = 1.77
NG = 0
DB = 0
SM = 0
Delta_r
Cytarabine
YGL105W ARC1
ZShift = −10.43
Z lm Score = 10.33
NG = 0
DB = 0
SM = 0
Delta_AUC
Cytarabine
YOR350C MNE1
ZShift = 2.75
Z lm Score = −2.02
NG = 0
DB = 0
SM = 0
Delta_L
Cytarabine
YOR350C MNE1
ZShift = −4.63
Z lm Score = 6.39
NG = 0
DB = 0
SM = 0
Delta_K
Cytarabine
YOR350C MNE1
ZShift = −1.81
Z lm Score = 1.79
NG = 0
DB = 0
SM = 0
Delta_r
Cytarabine
YOR350C MNE1
ZShift = −4.14
Z lm Score = 4.6
NG = 0
DB = 0
SM = 0
Delta_AUC
Cytarabine
YLR110C CCW12
ZShift = 2.03
Z lm Score = −2.02
NG = 0
DB = 0
SM = 0
Delta_L
Cytarabine
YLR110C CCW12
ZShift = −0.78
Z lm Score = 1.53
NG = 0
DB = 0
SM = 0
Delta_K
Cytarabine
YLR110C CCW12
ZShift = −2.1
Z lm Score = 2.62
NG = 0
DB = 0
SM = 0
Delta_r
Cytarabine
YLR110C CCW12
ZShift = −1.39
Z lm Score = 1.77
NG = 0
DB = 0
SM = 0
Delta_AUC
Cytarabine

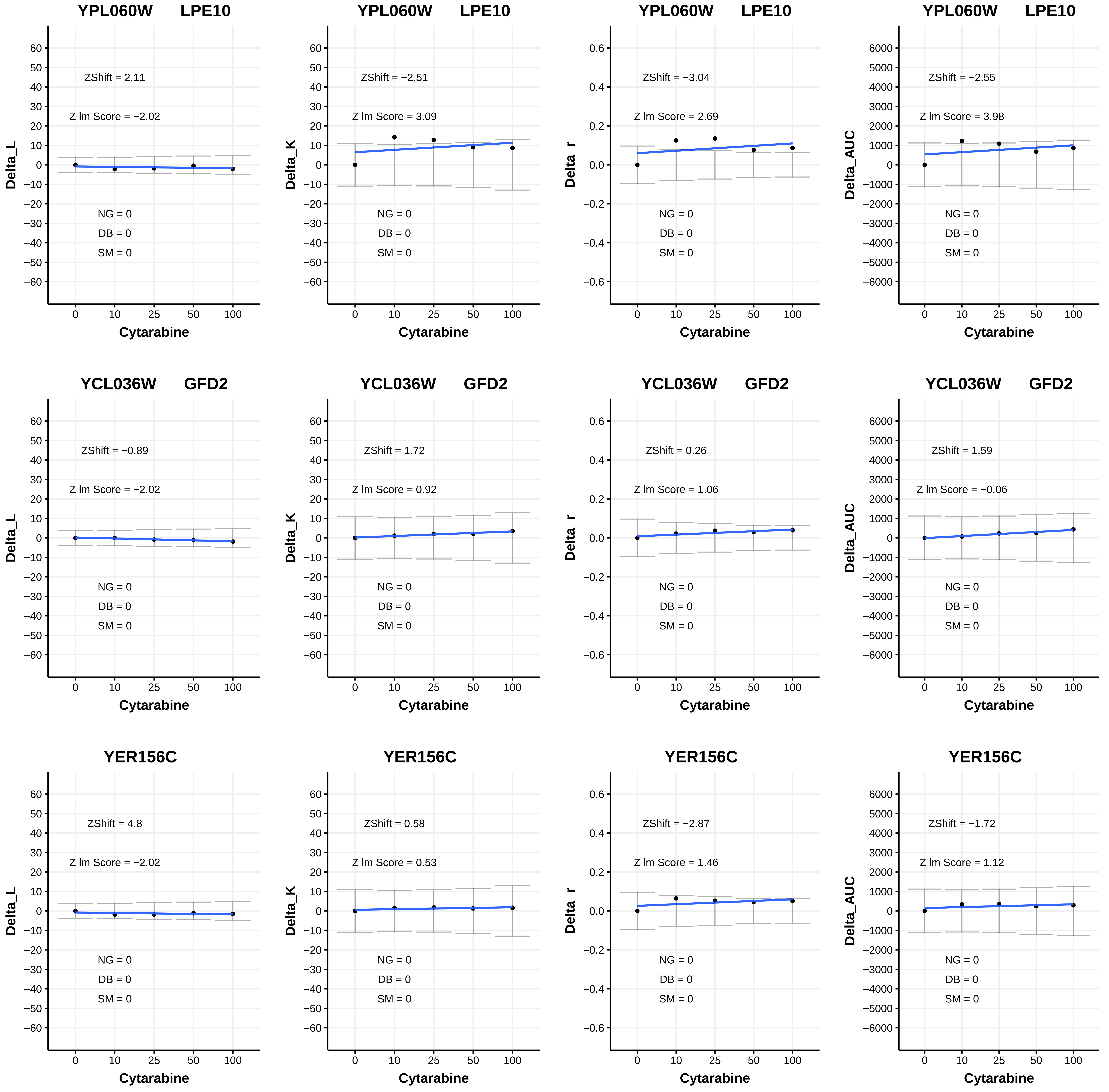

YPL060W LPE10
ZShift = 2.11
Z lm Score = −2.02
NG = 0
DB = 0
SM = 0
Delta_L
Cytarabine
YPL060W LPE10
ZShift = −2.51
Z lm Score = 3.09
NG = 0
DB = 0
SM = 0
Delta_K
Cytarabine
YPL060W LPE10
ZShift = −3.04
Z lm Score = 2.69
NG = 0
DB = 0
SM = 0
Delta_r
Cytarabine
YPL060W LPE10
ZShift = −2.55
Z lm Score = 3.98
NG = 0
DB = 0
SM = 0
Delta_AUC
Cytarabine
YCL036W GFD2
ZShift = −0.89
Z lm Score = −2.02
NG = 0
DB = 0
SM = 0
Delta_L
Cytarabine
YCL036W GFD2
ZShift = 1.72
Z lm Score = 0.92
NG = 0
DB = 0
SM = 0
Delta_K
Cytarabine
YCL036W GFD2
ZShift = 0.26
Z lm Score = 1.06
NG = 0
DB = 0
SM = 0
Delta_r
Cytarabine
YCL036W GFD2
ZShift = 1.59
Z lm Score = −0.06
NG = 0
DB = 0
SM = 0
Delta_AUC
Cytarabine
YER156C
ZShift = 4.8
Z lm Score = −2.02
NG = 0
DB = 0
SM = 0
Delta_L
Cytarabine
YER156C
ZShift = 0.58
Z lm Score = 0.53
NG = 0
DB = 0
SM = 0
Delta_K
Cytarabine
YER156C
ZShift = −2.87
Z lm Score = 1.46
NG = 0
DB = 0
SM = 0
Delta_r
Cytarabine
YER156C
ZShift = −1.72
Z lm Score = 1.12
NG = 0
DB = 0
SM = 0
Delta_AUC
Cytarabine

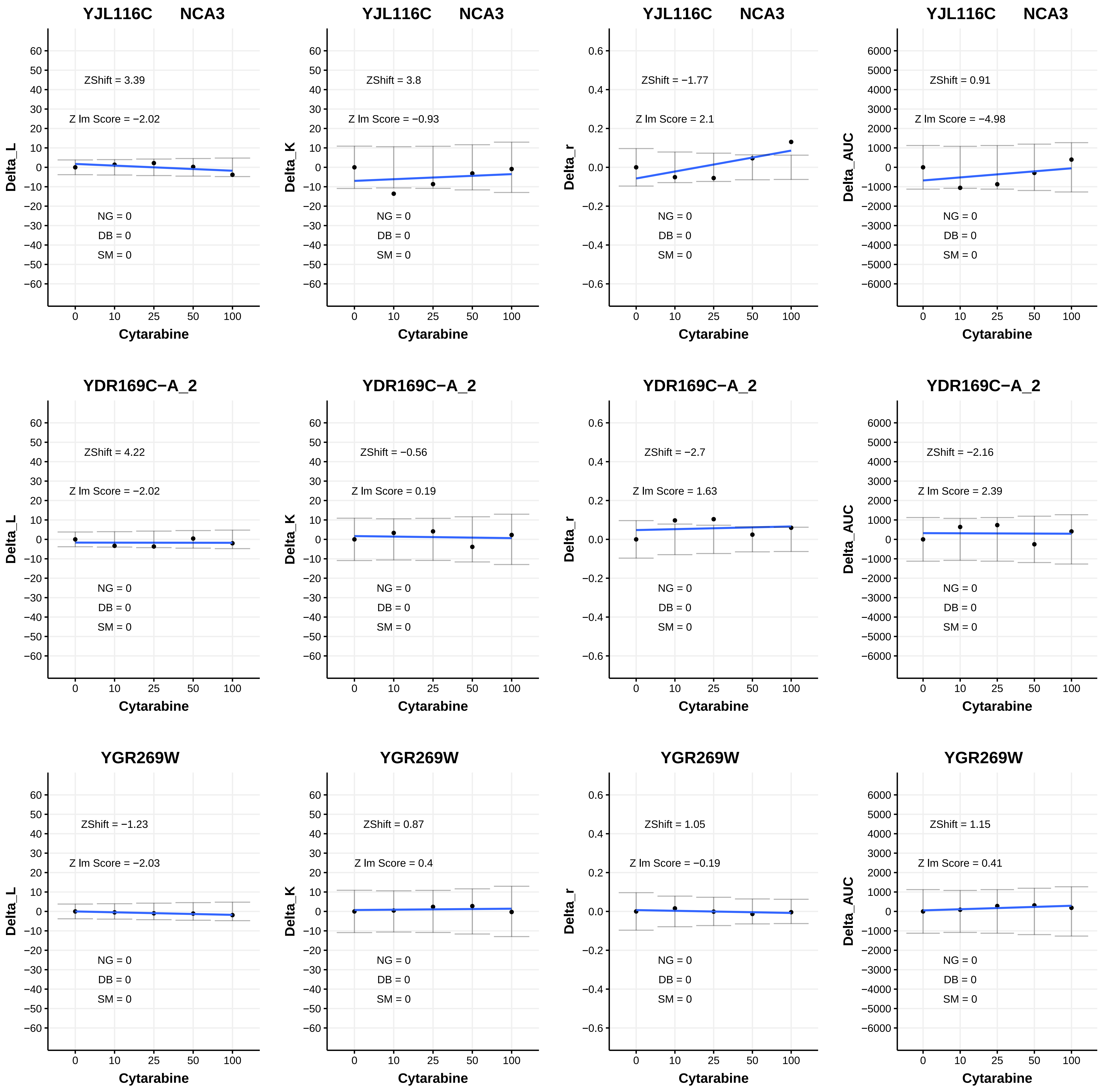

YJL116C NCA3
ZShift = 3.39
Z lm Score = −2.02
NG = 0
DB = 0
SM = 0
Delta_L
Cytarabine
YJL116C NCA3
ZShift = 3.8
Z lm Score = −0.93
NG = 0
DB = 0
SM = 0
Delta_K
Cytarabine
YJL116C NCA3
ZShift = −1.77
Z lm Score = 2.1
NG = 0
DB = 0
SM = 0
Delta_r
Cytarabine
YJL116C NCA3
ZShift = 0.91
Z lm Score = −4.98
NG = 0
DB = 0
SM = 0
Delta_AUC
Cytarabine
YDR169C−A_2
ZShift = 4.22
Z lm Score = −2.02
NG = 0
DB = 0
SM = 0
Delta_L
Cytarabine
YDR169C−A_2
ZShift = −0.56
Z lm Score = 0.19
NG = 0
DB = 0
SM = 0
Delta_K
Cytarabine
YDR169C−A_2
ZShift = −2.7
Z lm Score = 1.63
NG = 0
DB = 0
SM = 0
Delta_r
Cytarabine
YDR169C−A_2
ZShift = −2.16
Z lm Score = 2.39
NG = 0
DB = 0
SM = 0
Delta_AUC
Cytarabine
YGR269W
ZShift = −1.23
Z lm Score = −2.03
NG = 0
DB = 0
SM = 0
Delta_L
Cytarabine
YGR269W
ZShift = 0.87
Z lm Score = 0.4
NG = 0
DB = 0
SM = 0
Delta_K
Cytarabine
YGR269W
ZShift = 1.05
Z lm Score = −0.19
NG = 0
DB = 0
SM = 0
Delta_r
Cytarabine
YGR269W
ZShift = 1.15
Z lm Score = 0.41
NG = 0
DB = 0
SM = 0
Delta_AUC
Cytarabine

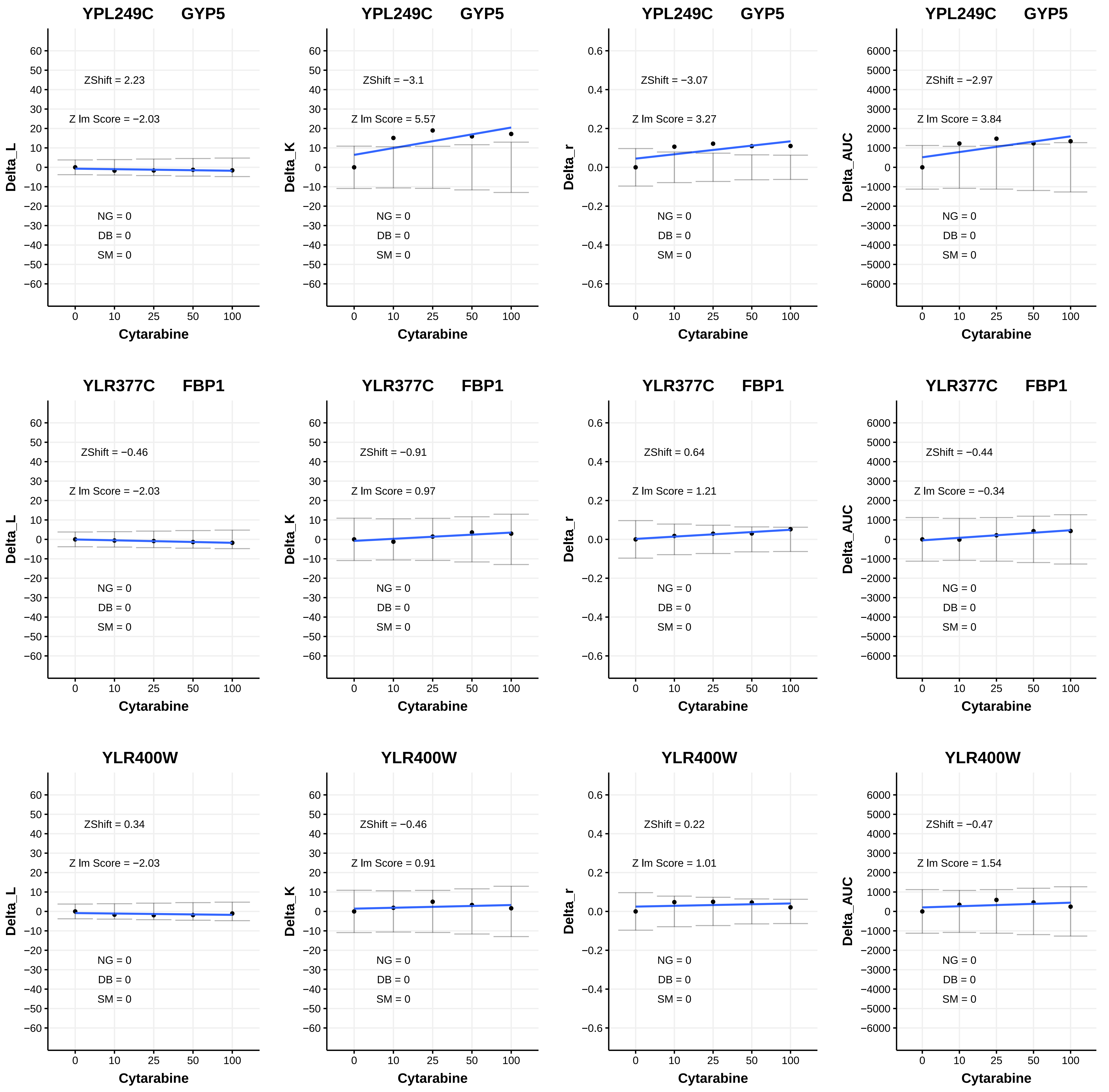

YPL249C GYP5
ZShift = 2.23
Z lm Score = −2.03
NG = 0
DB = 0
SM = 0
Delta_L
Cytarabine
YPL249C GYP5
ZShift = −3.1
Z lm Score = 5.57
NG = 0
DB = 0
SM = 0
Delta_K
Cytarabine
YPL249C GYP5
ZShift = −3.07
Z lm Score = 3.27
NG = 0
DB = 0
SM = 0
Delta_r
Cytarabine
YPL249C GYP5
ZShift = −2.97
Z lm Score = 3.84
NG = 0
DB = 0
SM = 0
Delta_AUC
Cytarabine
YLR377C FBP1
ZShift = −0.46
Z lm Score = −2.03
NG = 0
DB = 0
SM = 0
Delta_L
Cytarabine
YLR377C FBP1
ZShift = −0.91
Z lm Score = 0.97
NG = 0
DB = 0
SM = 0
Delta_K
Cytarabine
YLR377C FBP1
ZShift = 0.64
Z lm Score = 1.21
NG = 0
DB = 0
SM = 0
Delta_r
Cytarabine
YLR377C FBP1
ZShift = −0.44
Z lm Score = −0.34
NG = 0
DB = 0
SM = 0
Delta_AUC
Cytarabine
YLR400W
ZShift = 0.34
Z lm Score = −2.03
NG = 0
DB = 0
SM = 0
Delta_L
Cytarabine
YLR400W
ZShift = −0.46
Z lm Score = 0.91
NG = 0
DB = 0
SM = 0
Delta_K
Cytarabine
YLR400W
ZShift = 0.22
Z lm Score = 1.01
NG = 0
DB = 0
SM = 0
Delta_r
Cytarabine
YLR400W
ZShift = −0.47
Z lm Score = 1.54
NG = 0
DB = 0
SM = 0
Delta_AUC
Cytarabine

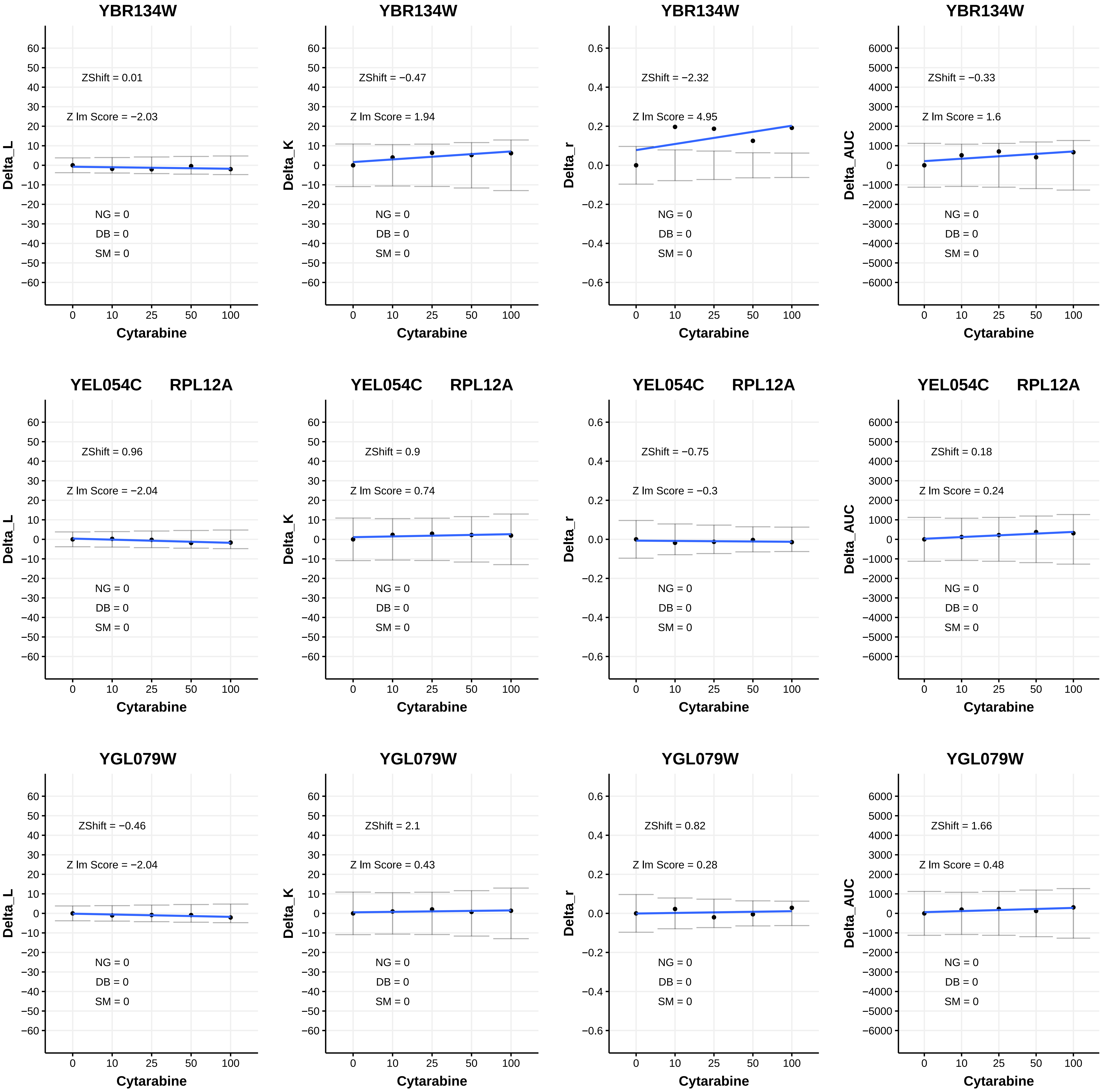

YBR134W
ZShift = 0.01
Z lm Score = −2.03
NG = 0
DB = 0
SM = 0
Delta_L
Cytarabine
YBR134W
ZShift = −0.47
Z lm Score = 1.94
NG = 0
DB = 0
SM = 0
Delta_K
Cytarabine
YBR134W
ZShift = −2.32
Z lm Score = 4.95
NG = 0
DB = 0
SM = 0
Delta_r
Cytarabine
YBR134W
ZShift = −0.33
Z lm Score = 1.6
NG = 0
DB = 0
SM = 0
Delta_AUC
Cytarabine
YEL054C RPL12A
ZShift = 0.96
Z lm Score = −2.04
NG = 0
DB = 0
SM = 0
Delta_L
Cytarabine
YEL054C RPL12A
ZShift = 0.9
Z lm Score = 0.74
NG = 0
DB = 0
SM = 0
Delta_K
Cytarabine
YEL054C RPL12A
ZShift = −0.75
Z lm Score = −0.3
NG = 0
DB = 0
SM = 0
Delta_r
Cytarabine
YEL054C RPL12A
ZShift = 0.18
Z lm Score = 0.24
NG = 0
DB = 0
SM = 0
Delta_AUC
Cytarabine
YGL079W
ZShift = −0.46
Z lm Score = −2.04
NG = 0
DB = 0
SM = 0
Delta_L
Cytarabine
YGL079W
ZShift = 2.1
Z lm Score = 0.43
NG = 0
DB = 0
SM = 0
Delta_K
Cytarabine
YGL079W
ZShift = 0.82
Z lm Score = 0.28
NG = 0
DB = 0
SM = 0
Delta_r
Cytarabine
YGL079W
ZShift = 1.66
Z lm Score = 0.48
NG = 0
DB = 0
SM = 0
Delta_AUC
Cytarabine

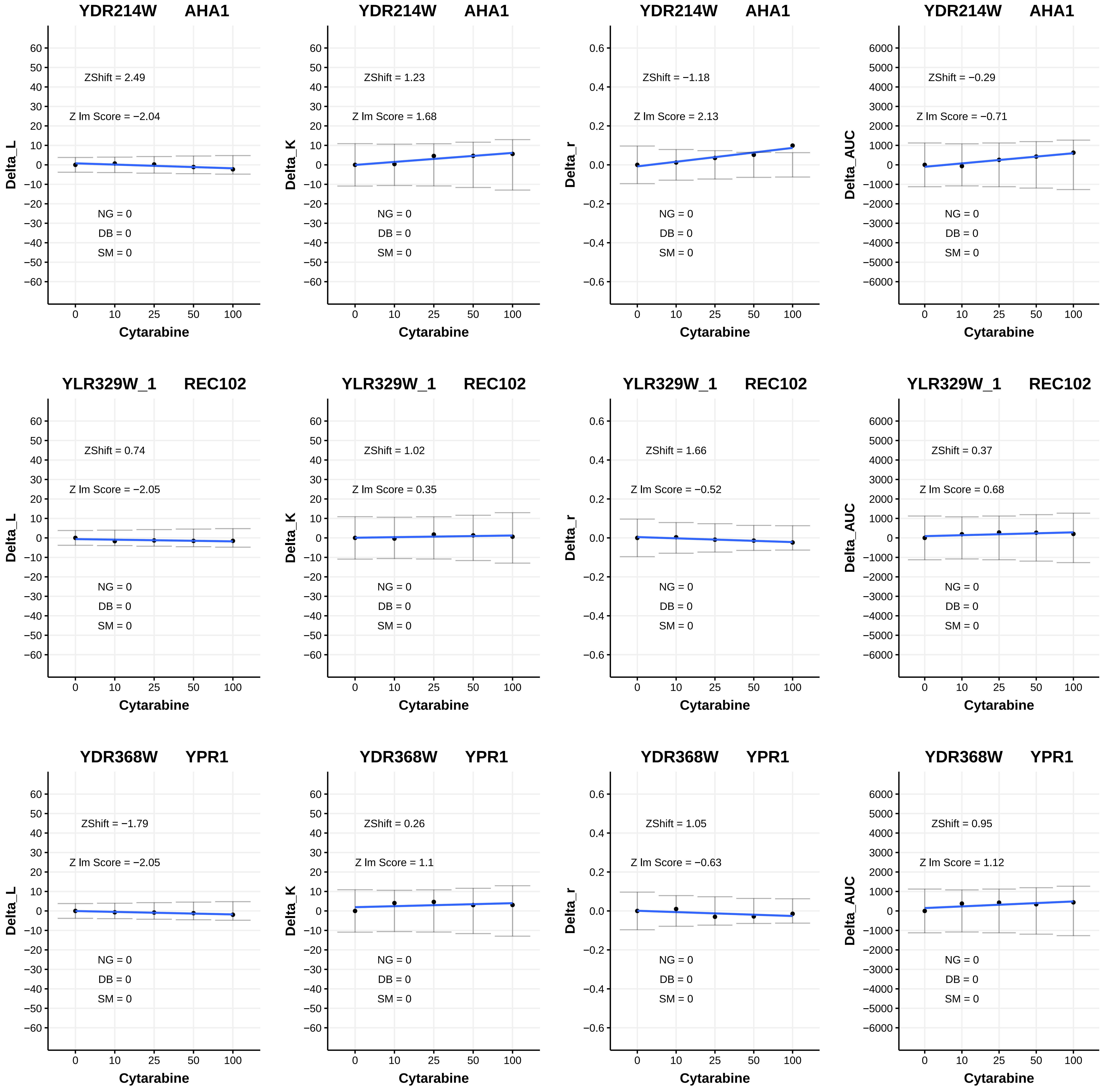

YDR214W AHA1
Delta_L
ZShift = 2.49
Z lm Score = −2.04
NG = 0
DB = 0
SM = 0
Cytarabine
YDR214W AHA1
Delta_K
ZShift = 1.23
Z lm Score = 1.68
NG = 0
DB = 0
SM = 0
Cytarabine
YDR214W AHA1
Delta_r
ZShift = −1.18
Z lm Score = 2.13
NG = 0
DB = 0
SM = 0
Cytarabine
YDR214W AHA1
Delta_AUC
ZShift = −0.29
Z lm Score = −0.71
NG = 0
DB = 0
SM = 0
Cytarabine
YLR329W_1 REC102
Delta_L
ZShift = 0.74
Z lm Score = −2.05
NG = 0
DB = 0
SM = 0
Cytarabine
YLR329W_1 REC102
Delta_K
ZShift = 1.02
Z lm Score = 0.35
NG = 0
DB = 0
SM = 0
Cytarabine
YLR329W_1 REC102
Delta_r
ZShift = 1.66
Z lm Score = −0.52
NG = 0
DB = 0
SM = 0
Cytarabine
YLR329W_1 REC102
Delta_AUC
ZShift = 0.37
Z lm Score = 0.68
NG = 0
DB = 0
SM = 0
Cytarabine
YDR368W YPR1
Delta_L
ZShift = −1.79
Z lm Score = −2.05
NG = 0
DB = 0
SM = 0
Cytarabine
YDR368W YPR1
Delta_K
ZShift = 0.26
Z lm Score = 1.1
NG = 0
DB = 0
SM = 0
Cytarabine
YDR368W YPR1
Delta_r
ZShift = 1.05
Z lm Score = −0.63
NG = 0
DB = 0
SM = 0
Cytarabine
YDR368W YPR1
Delta_AUC
ZShift = 0.95
Z lm Score = 1.12
NG = 0
DB = 0
SM = 0
Cytarabine

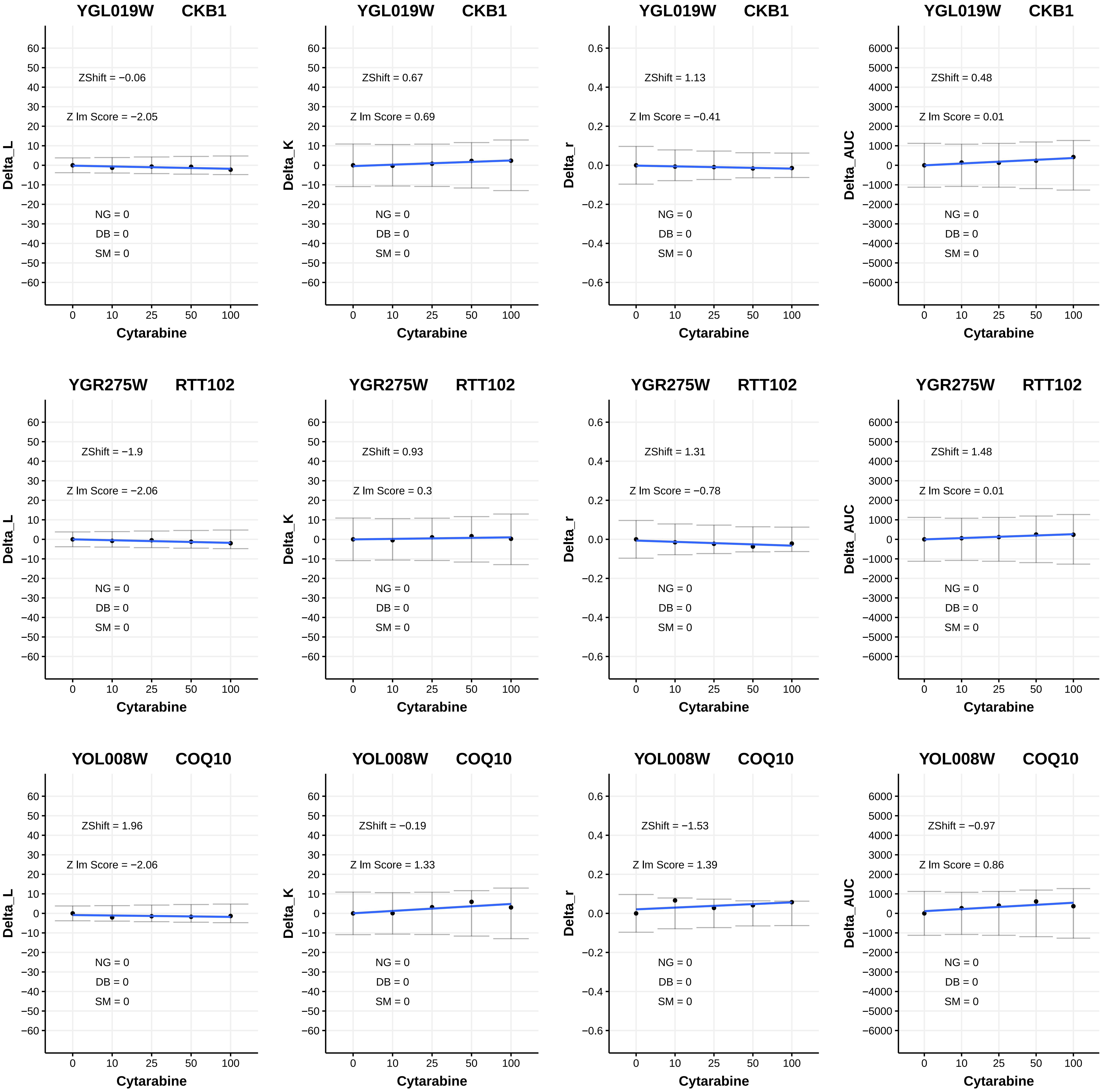

YGL019W CKB1
ZShift = −0.06
Z lm Score = −2.05
NG = 0
DB = 0
SM = 0
Delta_L
Cytarabine
YGL019W CKB1
ZShift = 0.67
Z lm Score = 0.69
NG = 0
DB = 0
SM = 0
Delta_K
Cytarabine
YGL019W CKB1
ZShift = 1.13
Z lm Score = −0.41
NG = 0
DB = 0
SM = 0
Delta_r
Cytarabine
YGL019W CKB1
ZShift = 0.48
Z lm Score = 0.01
NG = 0
DB = 0
SM = 0
Delta_AUC
Cytarabine
YGR275W RTT102
ZShift = −1.9
Z lm Score = −2.06
NG = 0
DB = 0
SM = 0
Delta_L
Cytarabine
YGR275W RTT102
ZShift = 0.93
Z lm Score = 0.3
NG = 0
DB = 0
SM = 0
Delta_K
Cytarabine
YGR275W RTT102
ZShift = 1.31
Z lm Score = −0.78
NG = 0
DB = 0
SM = 0
Delta_r
Cytarabine
YGR275W RTT102
ZShift = 1.48
Z lm Score = 0.01
NG = 0
DB = 0
SM = 0
Delta_AUC
Cytarabine
YOL008W COQ10
ZShift = 1.96
Z lm Score = −2.06
NG = 0
DB = 0
SM = 0
Delta_L
Cytarabine
YOL008W COQ10
ZShift = −0.19
Z lm Score = 1.33
NG = 0
DB = 0
SM = 0
Delta_K
Cytarabine
YOL008W COQ10
ZShift = −1.53
Z lm Score = 1.39
NG = 0
DB = 0
SM = 0
Delta_r
Cytarabine
YOL008W COQ10
ZShift = −0.97
Z lm Score = 0.86
NG = 0
DB = 0
SM = 0
Delta_AUC
Cytarabine

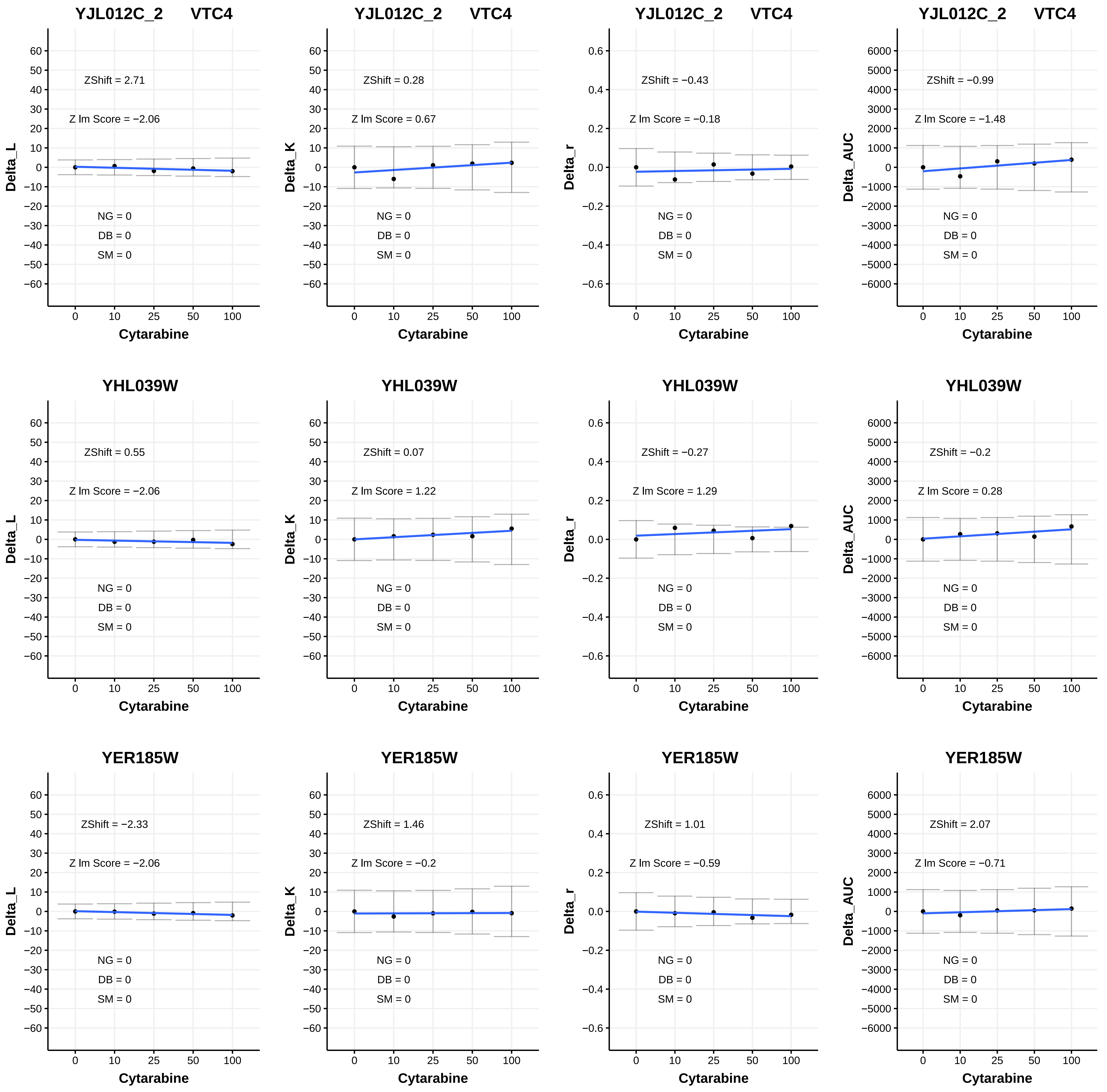
YJL012C_2 VTC4
ZShift = 2.71
Z lm Score = −2.06
NG = 0
DB = 0
SM = 0
Delta_L
Cytarabine
YJL012C_2 VTC4
ZShift = 0.28
Z lm Score = 0.67
NG = 0
DB = 0
SM = 0
Delta_K
Cytarabine
YJL012C_2 VTC4
ZShift = −0.43
Z lm Score = −0.18
NG = 0
DB = 0
SM = 0
Delta_r
Cytarabine
YJL012C_2 VTC4
ZShift = −0.99
Z lm Score = −1.48
NG = 0
DB = 0
SM = 0
Delta_AUC
Cytarabine
YHL039W
ZShift = 0.55
Z lm Score = −2.06
NG = 0
DB = 0
SM = 0
Delta_L
Cytarabine
YHL039W
ZShift = 0.07
Z lm Score = 1.22
NG = 0
DB = 0
SM = 0
Delta_K
Cytarabine
YHL039W
ZShift = −0.27
Z lm Score = 1.29
NG = 0
DB = 0
SM = 0
Delta_r
Cytarabine
YHL039W
ZShift = −0.2
Z lm Score = 0.28
NG = 0
DB = 0
SM = 0
Delta_AUC
Cytarabine
YER185W
ZShift = −2.33
Z lm Score = −2.06
NG = 0
DB = 0
SM = 0
Delta_L
Cytarabine
YER185W
ZShift = 1.46
Z lm Score = −0.2
NG = 0
DB = 0
SM = 0
Delta_K
Cytarabine
YER185W
ZShift = 1.01
Z lm Score = −0.59
NG = 0
DB = 0
SM = 0
Delta_r
Cytarabine
YER185W
ZShift = 2.07
Z lm Score = −0.71
NG = 0
DB = 0
SM = 0
Delta_AUC
Cytarabine

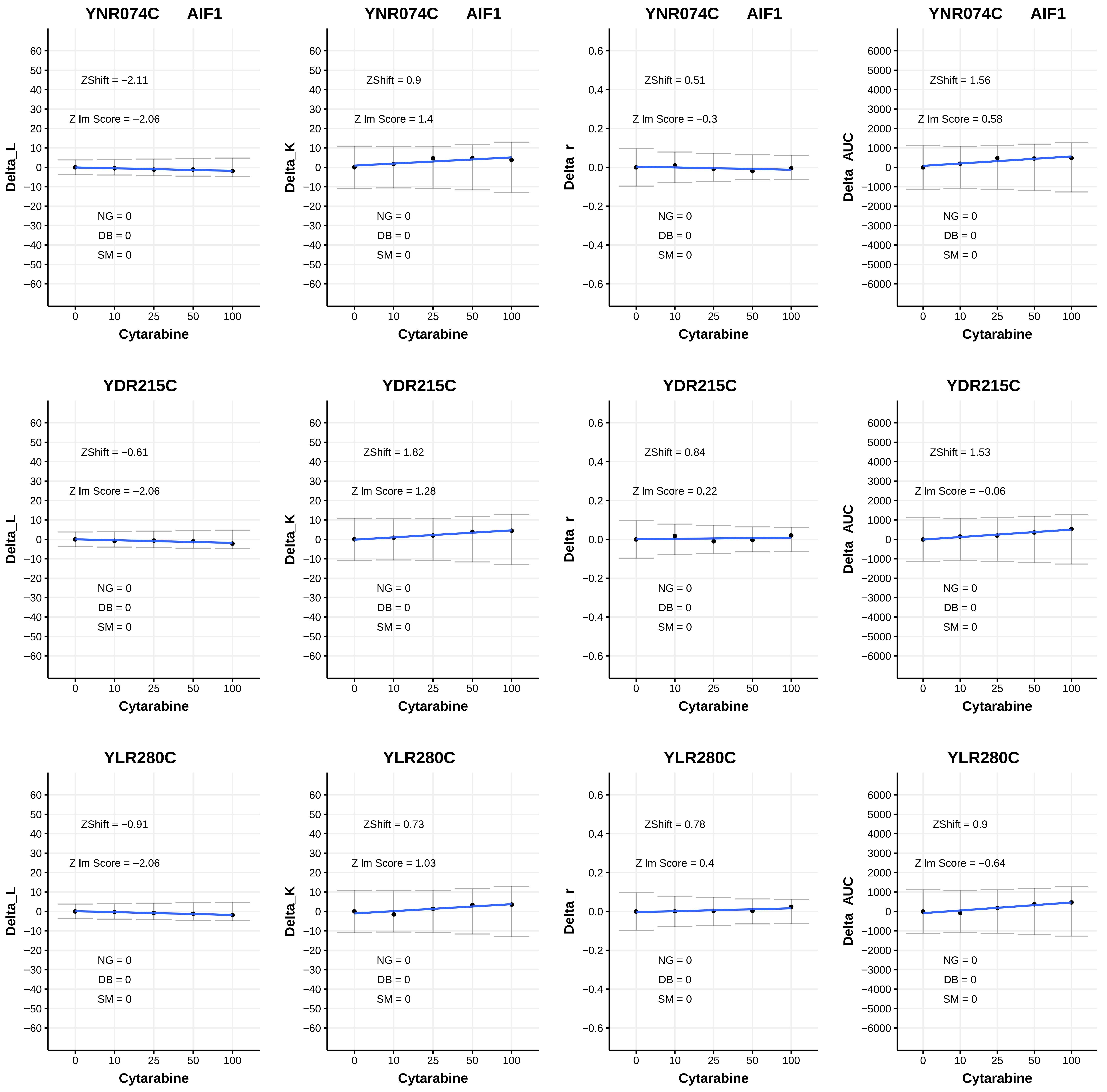

YNR074C AIF1
Delta_L
ZShift = −2.11
Z lm Score = −2.06
NG = 0
DB = 0
SM = 0
Cytarabine
YNR074C AIF1
Delta_K
ZShift = 0.9
Z lm Score = 1.4
NG = 0
DB = 0
SM = 0
Cytarabine
YNR074C AIF1
Delta_r
ZShift = 0.51
Z lm Score = −0.3
NG = 0
DB = 0
SM = 0
Cytarabine
YNR074C AIF1
Delta_AUC
ZShift = 1.56
Z lm Score = 0.58
NG = 0
DB = 0
SM = 0
Cytarabine
YDR215C
Delta_L
ZShift = −0.61
Z lm Score = −2.06
NG = 0
DB = 0
SM = 0
Cytarabine
YDR215C
Delta_K
ZShift = 1.82
Z lm Score = 1.28
NG = 0
DB = 0
SM = 0
Cytarabine
YDR215C
Delta_r
ZShift = 0.84
Z lm Score = 0.22
NG = 0
DB = 0
SM = 0
Cytarabine
YDR215C
Delta_AUC
ZShift = 1.53
Z lm Score = −0.06
NG = 0
DB = 0
SM = 0
Cytarabine
YLR280C
Delta_L
ZShift = −0.91
Z lm Score = −2.06
NG = 0
DB = 0
SM = 0
Cytarabine
YLR280C
Delta_K
ZShift = 0.73
Z lm Score = 1.03
NG = 0
DB = 0
SM = 0
Cytarabine
YLR280C
Delta_r
ZShift = 0.78
Z lm Score = 0.4
NG = 0
DB = 0
SM = 0
Cytarabine
YLR280C
Delta_AUC
ZShift = 0.9
Z lm Score = −0.64
NG = 0
DB = 0
SM = 0
Cytarabine

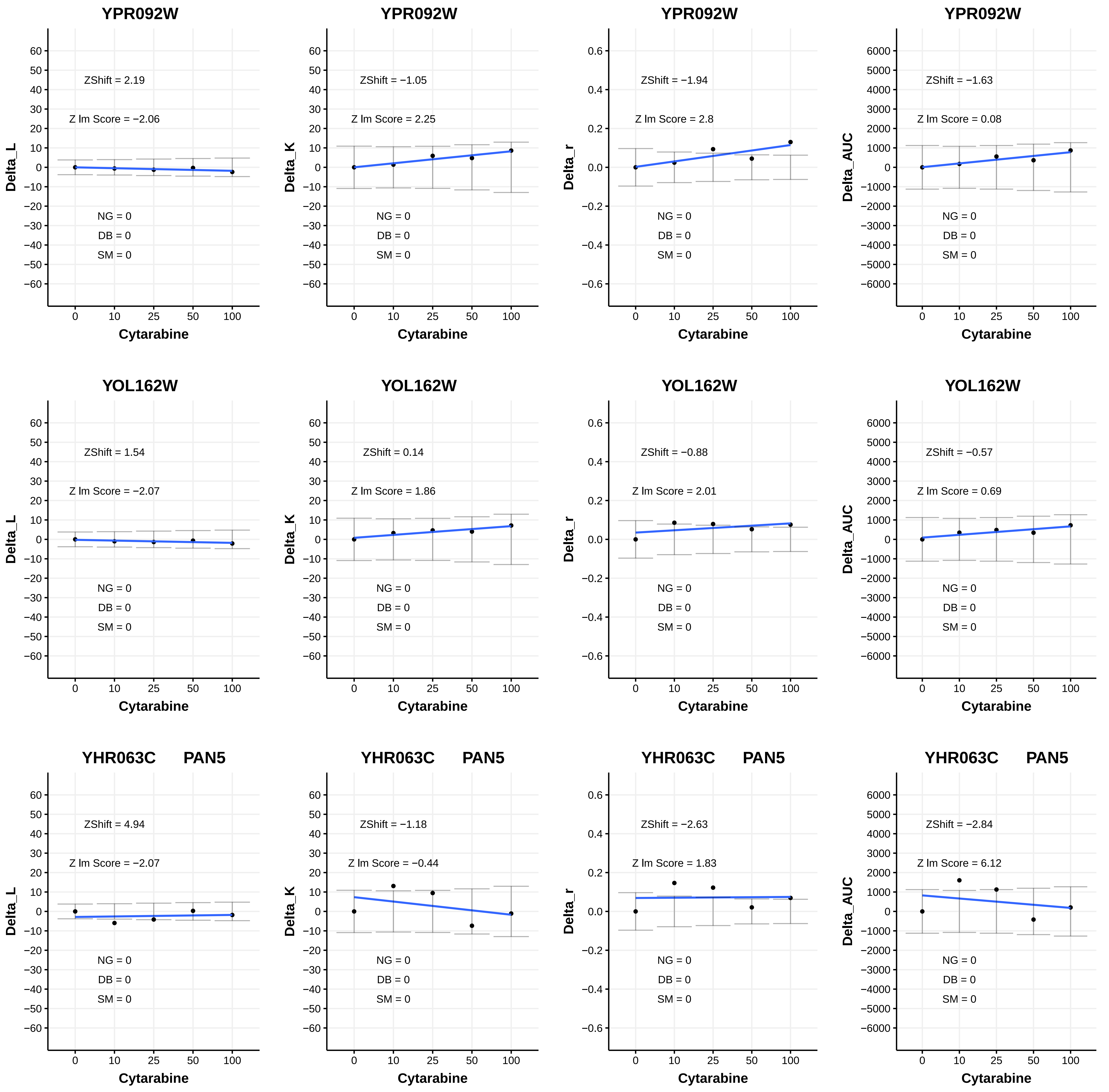
YPR092W
ZShift = 2.19
Z lm Score = −2.06
NG = 0
DB = 0
SM = 0
Delta_L
Cytarabine
YPR092W
ZShift = −1.05
Z lm Score = 2.25
NG = 0
DB = 0
SM = 0
Delta_K
Cytarabine
YPR092W
ZShift = −1.94
Z lm Score = 2.8
NG = 0
DB = 0
SM = 0
Delta_r
Cytarabine
YPR092W
ZShift = −1.63
Z lm Score = 0.08
NG = 0
DB = 0
SM = 0
Delta_AUC
Cytarabine
YOL162W
ZShift = 1.54
Z lm Score = −2.07
NG = 0
DB = 0
SM = 0
Delta_L
Cytarabine
YOL162W
ZShift = 0.14
Z lm Score = 1.86
NG = 0
DB = 0
SM = 0
Delta_K
Cytarabine
YOL162W
ZShift = −0.88
Z lm Score = 2.01
NG = 0
DB = 0
SM = 0
Delta_r
Cytarabine
YOL162W
ZShift = −0.57
Z lm Score = 0.69
NG = 0
DB = 0
SM = 0
Delta_AUC
Cytarabine
YHR063C PAN5
ZShift = 4.94
Z lm Score = −2.07
NG = 0
DB = 0
SM = 0
Delta_L
Cytarabine
YHR063C PAN5
ZShift = −1.18
Z lm Score = −0.44
NG = 0
DB = 0
SM = 0
Delta_K
Cytarabine
YHR063C PAN5
ZShift = −2.63
Z lm Score = 1.83
NG = 0
DB = 0
SM = 0
Delta_r
Cytarabine
YHR063C PAN5
ZShift = −2.84
Z lm Score = 6.12
NG = 0
DB = 0
SM = 0
Delta_AUC
Cytarabine

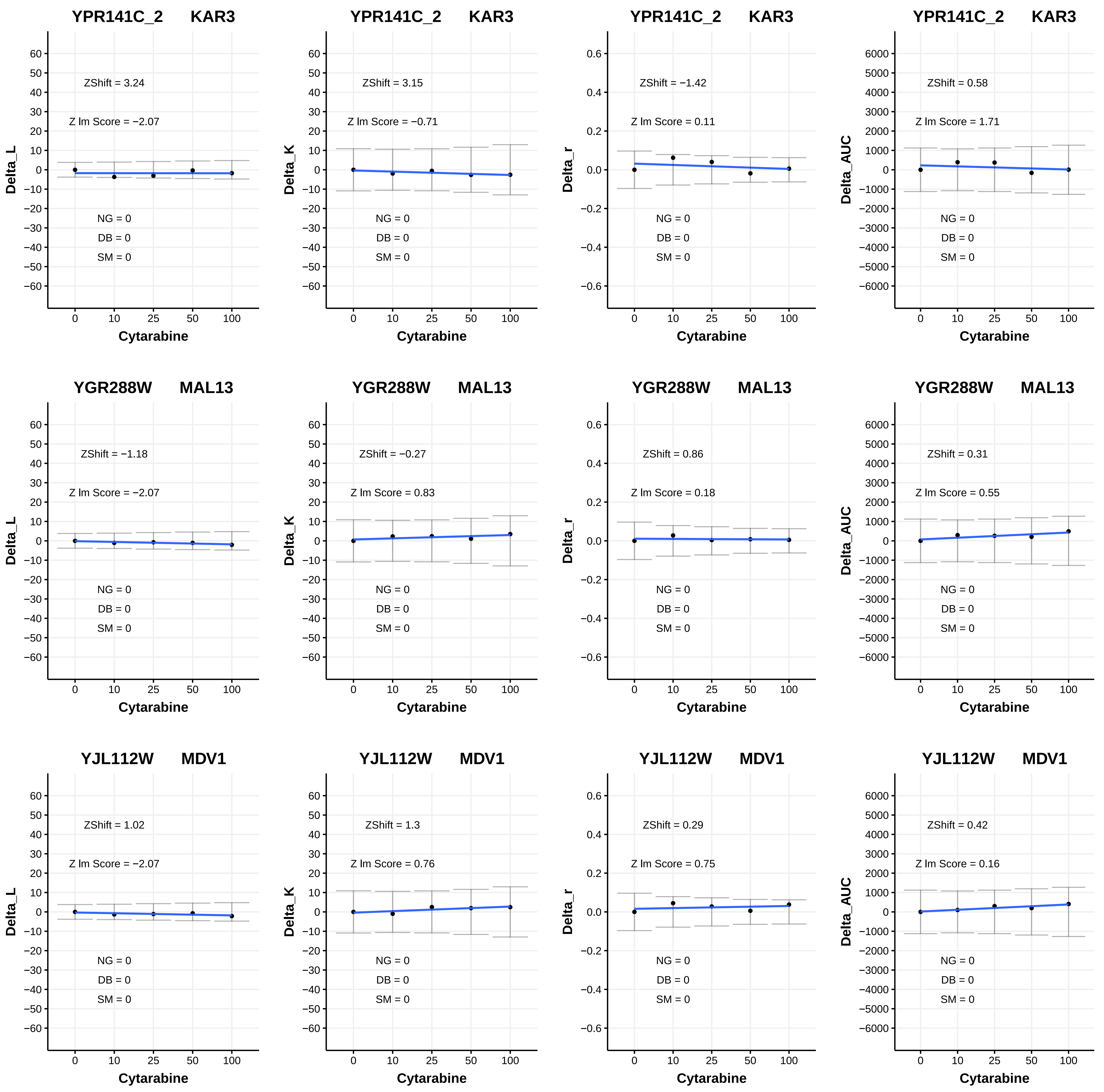
YPR141C_2 KAR3
ZShift = 3.24
Z lm Score = −2.07
NG = 0
DB = 0
SM = 0
Delta_L
Cytarabine
YPR141C_2 KAR3
ZShift = 3.15
Z lm Score = −0.71
NG = 0
DB = 0
SM = 0
Delta_K
Cytarabine
YPR141C_2 KAR3
ZShift = −1.42
Z lm Score = 0.11
NG = 0
DB = 0
SM = 0
Delta_r
Cytarabine
YPR141C_2 KAR3
ZShift = 0.58
Z lm Score = 1.71
NG = 0
DB = 0
SM = 0
Delta_AUC
Cytarabine
YGR288W MAL13
ZShift = −1.18
Z lm Score = −2.07
NG = 0
DB = 0
SM = 0
Delta_L
Cytarabine
YGR288W MAL13
ZShift = −0.27
Z lm Score = 0.83
NG = 0
DB = 0
SM = 0
Delta_K
Cytarabine
YGR288W MAL13
ZShift = 0.86
Z lm Score = 0.18
NG = 0
DB = 0
SM = 0
Delta_r
Cytarabine
YGR288W MAL13
ZShift = 0.31
Z lm Score = 0.55
NG = 0
DB = 0
SM = 0
Delta_AUC
Cytarabine
YJL112W MDV1
ZShift = 1.02
Z lm Score = −2.07
NG = 0
DB = 0
SM = 0
Delta_L
Cytarabine
YJL112W MDV1
ZShift = 1.3
Z lm Score = 0.76
NG = 0
DB = 0
SM = 0
Delta_K
Cytarabine
YJL112W MDV1
ZShift = 0.29
Z lm Score = 0.75
NG = 0
DB = 0
SM = 0
Delta_r
Cytarabine
YJL112W MDV1
ZShift = 0.42
Z lm Score = 0.16
NG = 0
DB = 0
SM = 0
Delta_AUC
Cytarabine

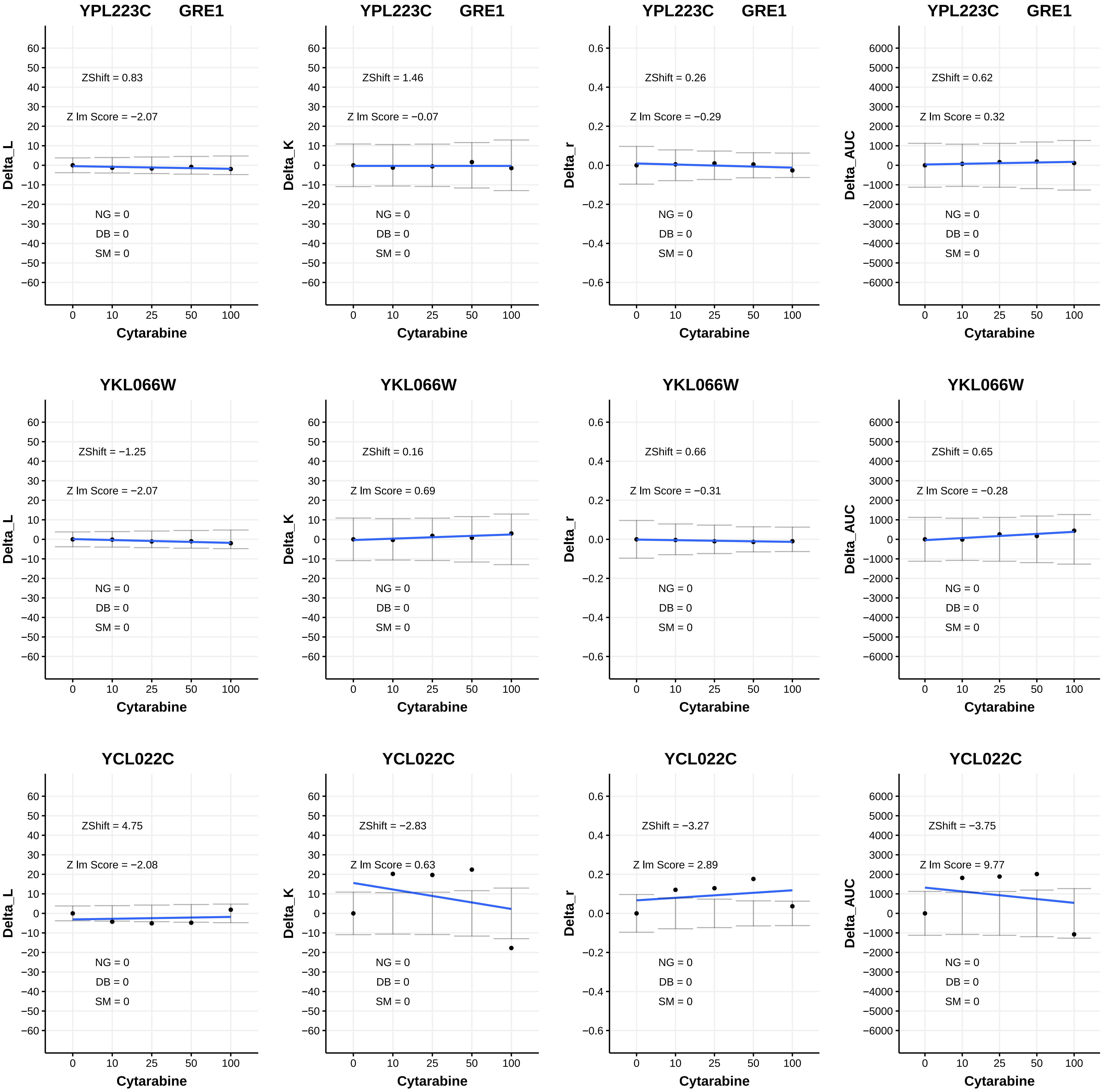

YPL223C GRE1
ZShift = 0.83
Z lm Score = −2.07
NG = 0
DB = 0
SM = 0
Delta_L
Cytarabine
YPL223C GRE1
ZShift = 1.46
Z lm Score = −0.07
NG = 0
DB = 0
SM = 0
Delta_K
Cytarabine
YPL223C GRE1
ZShift = 0.26
Z lm Score = −0.29
NG = 0
DB = 0
SM = 0
Delta_r
Cytarabine
YPL223C GRE1
ZShift = 0.62
Z lm Score = 0.32
NG = 0
DB = 0
SM = 0
Delta_AUC
Cytarabine
YKL066W
ZShift = −1.25
Z lm Score = −2.07
NG = 0
DB = 0
SM = 0
Delta_L
Cytarabine
YKL066W
ZShift = 0.16
Z lm Score = 0.69
NG = 0
DB = 0
SM = 0
Delta_K
Cytarabine
YKL066W
ZShift = 0.66
Z lm Score = −0.31
NG = 0
DB = 0
SM = 0
Delta_r
Cytarabine
YKL066W
ZShift = 0.65
Z lm Score = −0.28
NG = 0
DB = 0
SM = 0
Delta_AUC
Cytarabine
YCL022C
ZShift = 4.75
Z lm Score = −2.08
NG = 0
DB = 0
SM = 0
Delta_L
Cytarabine
YCL022C
ZShift = −2.83
Z lm Score = 0.63
NG = 0
DB = 0
SM = 0
Delta_K
Cytarabine
YCL022C
ZShift = −3.27
Z lm Score = 2.89
NG = 0
DB = 0
SM = 0
Delta_r
Cytarabine
YCL022C
ZShift = −3.75
Z lm Score = 9.77
NG = 0
DB = 0
SM = 0
Delta_AUC
Cytarabine

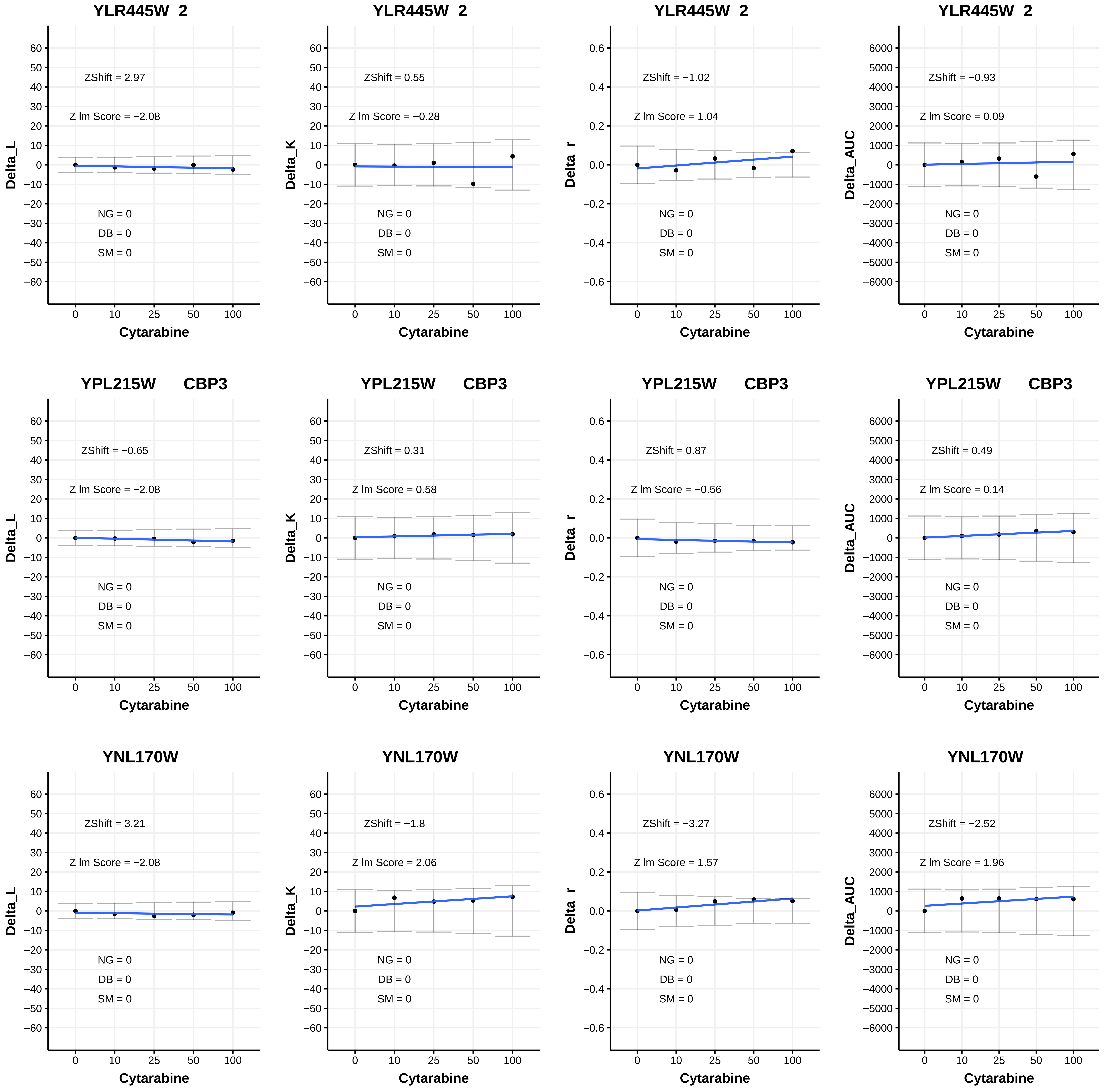

YLR445W_2
ZShift = 2.97
Z lm Score = −2.08
NG = 0
DB = 0
SM = 0
Delta_L
Cytarabine
YLR445W_2
ZShift = 0.55
Z lm Score = −0.28
NG = 0
DB = 0
SM = 0
Delta_K
Cytarabine
YLR445W_2
ZShift = −1.02
Z lm Score = 1.04
NG = 0
DB = 0
SM = 0
Delta_r
Cytarabine
YLR445W_2
ZShift = −0.93
Z lm Score = 0.09
NG = 0
DB = 0
SM = 0
Delta_AUC
Cytarabine
YPL215W CBP3
ZShift = −0.65
Z lm Score = −2.08
NG = 0
DB = 0
SM = 0
Delta_L
Cytarabine
YPL215W CBP3
ZShift = 0.31
Z lm Score = 0.58
NG = 0
DB = 0
SM = 0
Delta_K
Cytarabine
YPL215W CBP3
ZShift = 0.87
Z lm Score = −0.56
NG = 0
DB = 0
SM = 0
Delta_r
Cytarabine
YPL215W CBP3
ZShift = 0.49
Z lm Score = 0.14
NG = 0
DB = 0
SM = 0
Delta_AUC
Cytarabine
YNL170W
ZShift = 3.21
Z lm Score = −2.08
NG = 0
DB = 0
SM = 0
Delta_L
Cytarabine
YNL170W
ZShift = −1.8
Z lm Score = 2.06
NG = 0
DB = 0
SM = 0
Delta_K
Cytarabine
YNL170W
ZShift = −3.27
Z lm Score = 1.57
NG = 0
DB = 0
SM = 0
Delta_r
Cytarabine
YNL170W
ZShift = −2.52
Z lm Score = 1.96
NG = 0
DB = 0
SM = 0
Delta_AUC
Cytarabine

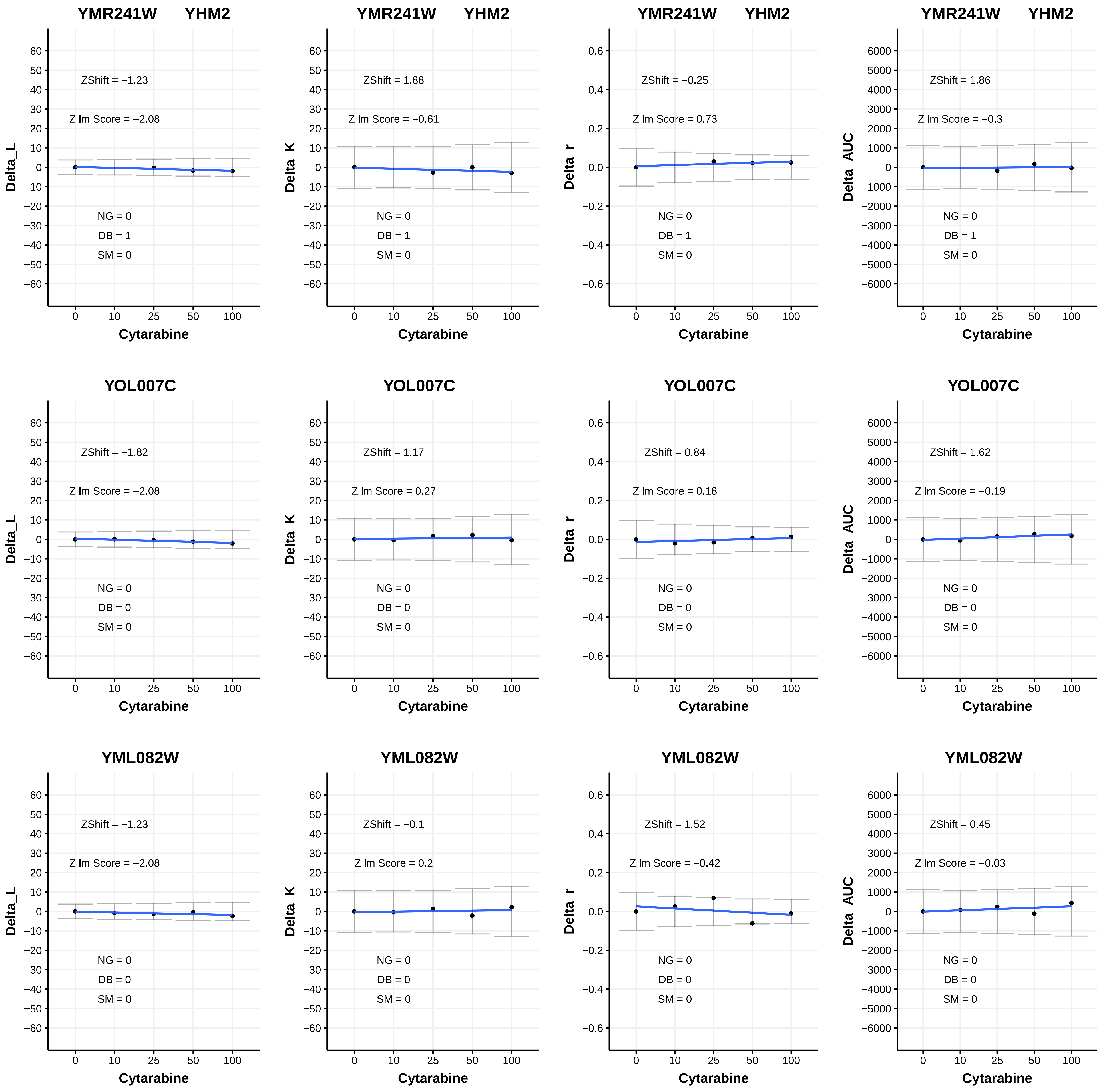

YMR241W YHM2
ZShift = −1.23
Z lm Score = −2.08
NG = 0
DB = 1
SM = 0
Delta_L
Cytarabine
YMR241W YHM2
ZShift = 1.88
Z lm Score = −0.61
NG = 0
DB = 1
SM = 0
Delta_K
Cytarabine
YMR241W YHM2
ZShift = −0.25
Z lm Score = 0.73
NG = 0
DB = 1
SM = 0
Delta_r
Cytarabine
YMR241W YHM2
ZShift = 1.86
Z lm Score = −0.3
NG = 0
DB = 1
SM = 0
Delta_AUC
Cytarabine
YOL007C
ZShift = −1.82
Z lm Score = −2.08
NG = 0
DB = 0
SM = 0
Delta_L
Cytarabine
YOL007C
ZShift = 1.17
Z lm Score = 0.27
NG = 0
DB = 0
SM = 0
Delta_K
Cytarabine
YOL007C
ZShift = 0.84
Z lm Score = 0.18
NG = 0
DB = 0
SM = 0
Delta_r
Cytarabine
YOL007C
ZShift = 1.62
Z lm Score = −0.19
NG = 0
DB = 0
SM = 0
Delta_AUC
Cytarabine
YML082W
ZShift = −1.23
Z lm Score = −2.08
NG = 0
DB = 0
SM = 0
Delta_L
Cytarabine
YML082W
ZShift = −0.1
Z lm Score = 0.2
NG = 0
DB = 0
SM = 0
Delta_K
Cytarabine
YML082W
ZShift = 1.52
Z lm Score = −0.42
NG = 0
DB = 0
SM = 0
Delta_r
Cytarabine
YML082W
ZShift = 0.45
Z lm Score = −0.03
NG = 0
DB = 0
SM = 0
Delta_AUC
Cytarabine

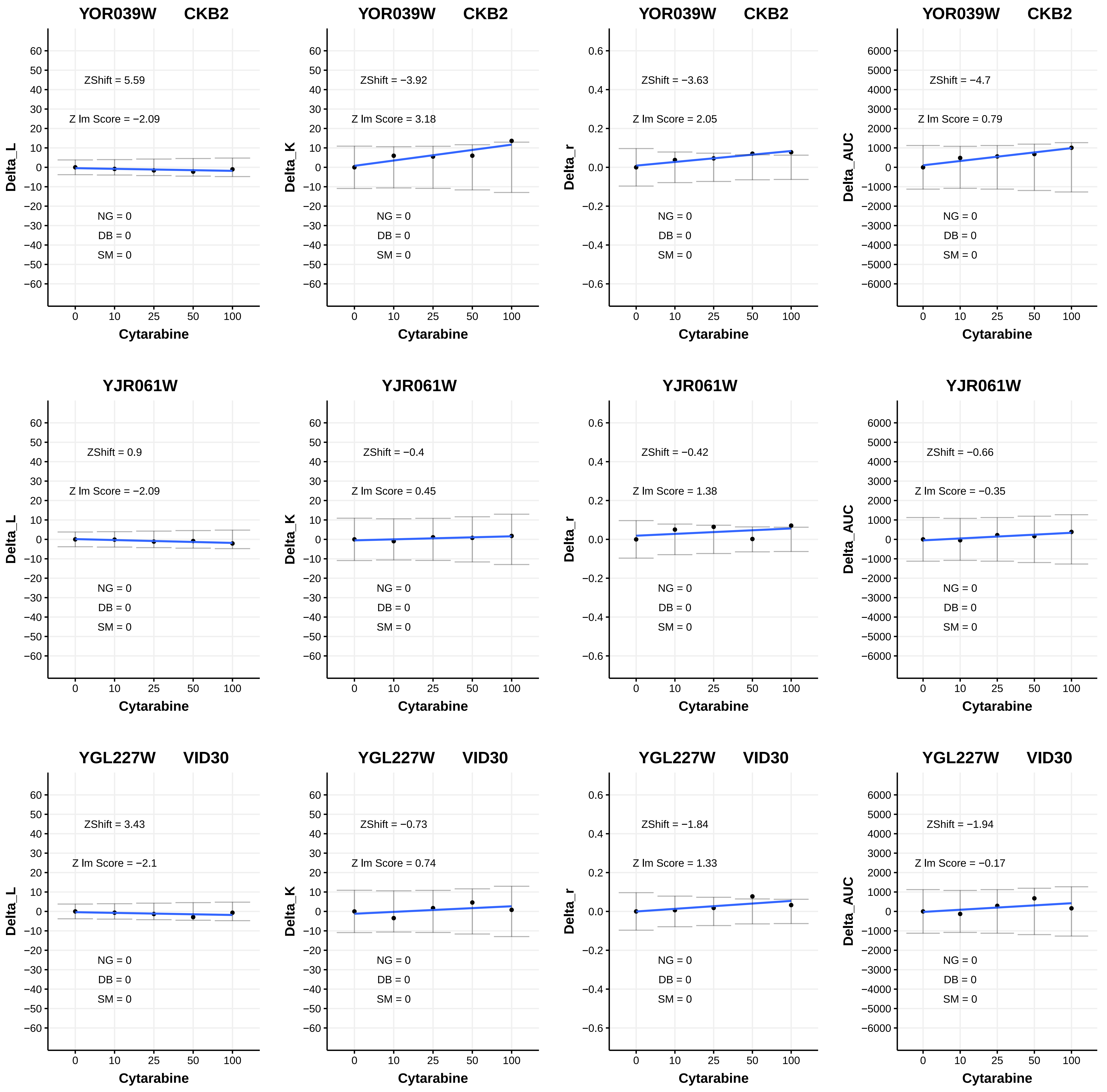
YOR039W CKB2
ZShift = 5.59
Z lm Score = −2.09
NG = 0
DB = 0
SM = 0
Delta_L
Cytarabine
YOR039W CKB2
ZShift = −3.92
Z lm Score = 3.18
NG = 0
DB = 0
SM = 0
Delta_K
Cytarabine
YOR039W CKB2
ZShift = −3.63
Z lm Score = 2.05
NG = 0
DB = 0
SM = 0
Delta_r
Cytarabine
YOR039W CKB2
ZShift = −4.7
Z lm Score = 0.79
NG = 0
DB = 0
SM = 0
Delta_AUC
Cytarabine
YJR061W
ZShift = 0.9
Z lm Score = −2.09
NG = 0
DB = 0
SM = 0
Delta_L
Cytarabine
YJR061W
ZShift = −0.4
Z lm Score = 0.45
NG = 0
DB = 0
SM = 0
Delta_K
Cytarabine
YJR061W
ZShift = −0.42
Z lm Score = 1.38
NG = 0
DB = 0
SM = 0
Delta_r
Cytarabine
YJR061W
ZShift = −0.66
Z lm Score = −0.35
NG = 0
DB = 0
SM = 0
Delta_AUC
Cytarabine
YGL227W VID30
ZShift = 3.43
Z lm Score = −2.1
NG = 0
DB = 0
SM = 0
Delta_L
Cytarabine
YGL227W VID30
ZShift = −0.73
Z lm Score = 0.74
NG = 0
DB = 0
SM = 0
Delta_K
Cytarabine
YGL227W VID30
ZShift = −1.84
Z lm Score = 1.33
NG = 0
DB = 0
SM = 0
Delta_r
Cytarabine
YGL227W VID30
ZShift = −1.94
Z lm Score = −0.17
NG = 0
DB = 0
SM = 0
Delta_AUC
Cytarabine

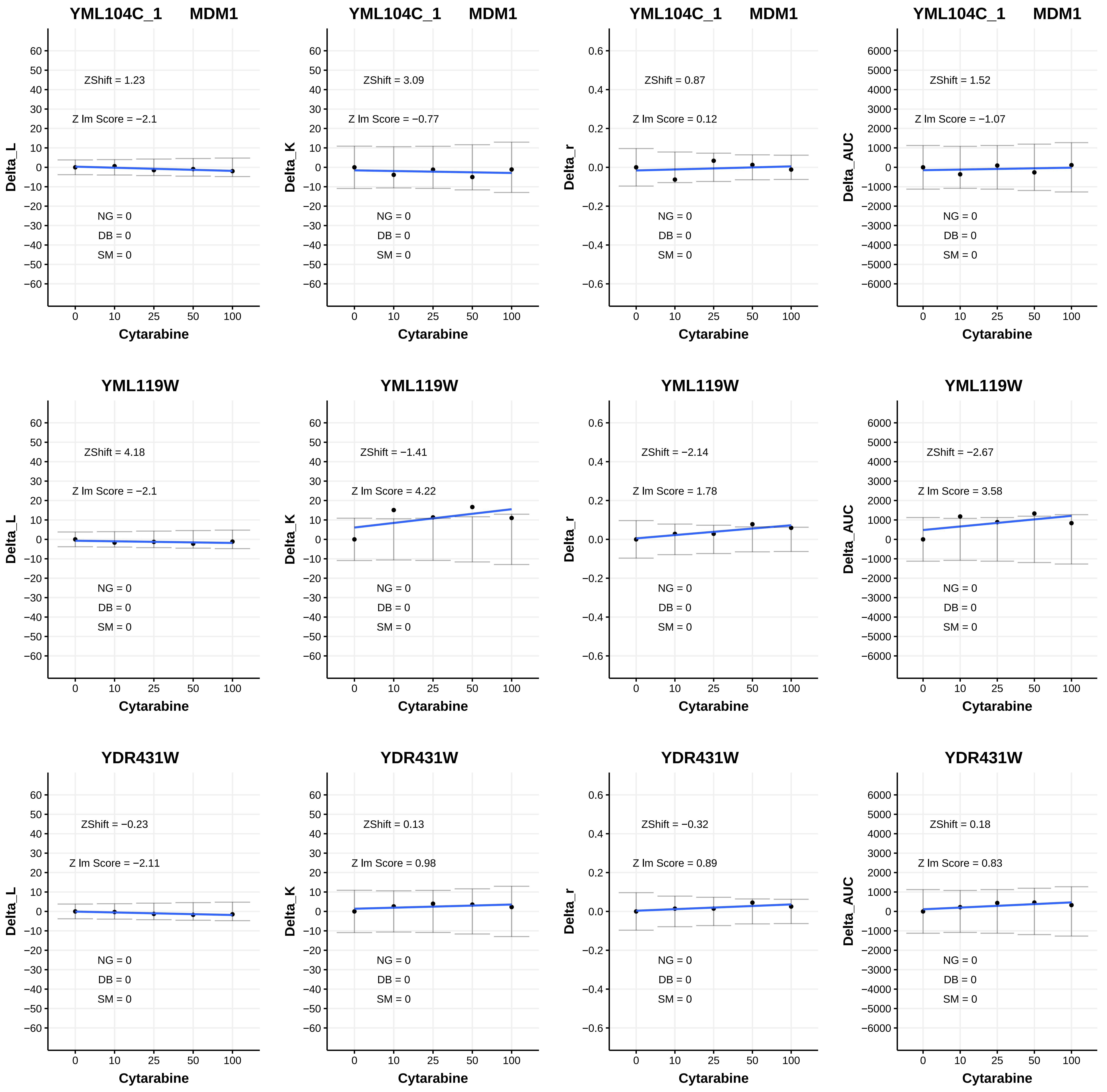
YML104C_1 MDM1
ZShift = 1.23
Z lm Score = −2.1
NG = 0
DB = 0
SM = 0
Delta_L
Cytarabine
YML104C_1 MDM1
ZShift = 3.09
Z lm Score = −0.77
NG = 0
DB = 0
SM = 0
Delta_K
Cytarabine
YML104C_1 MDM1
ZShift = 0.87
Z lm Score = 0.12
NG = 0
DB = 0
SM = 0
Delta_r
Cytarabine
YML104C_1 MDM1
ZShift = 1.52
Z lm Score = −1.07
NG = 0
DB = 0
SM = 0
Delta_AUC
Cytarabine
YML119W
ZShift = 4.18
Z lm Score = −2.1
NG = 0
DB = 0
SM = 0
Delta_L
Cytarabine
YML119W
ZShift = −1.41
Z lm Score = 4.22
NG = 0
DB = 0
SM = 0
Delta_K
Cytarabine
YML119W
ZShift = −2.14
Z lm Score = 1.78
NG = 0
DB = 0
SM = 0
Delta_r
Cytarabine
YML119W
ZShift = −2.67
Z lm Score = 3.58
NG = 0
DB = 0
SM = 0
Delta_AUC
Cytarabine
YDR431W
ZShift = −0.23
Z lm Score = −2.11
NG = 0
DB = 0
SM = 0
Delta_L
Cytarabine
YDR431W
ZShift = 0.13
Z lm Score = 0.98
NG = 0
DB = 0
SM = 0
Delta_K
Cytarabine
YDR431W
ZShift = −0.32
Z lm Score = 0.89
NG = 0
DB = 0
SM = 0
Delta_r
Cytarabine
YDR431W
ZShift = 0.18
Z lm Score = 0.83
NG = 0
DB = 0
SM = 0
Delta_AUC
Cytarabine

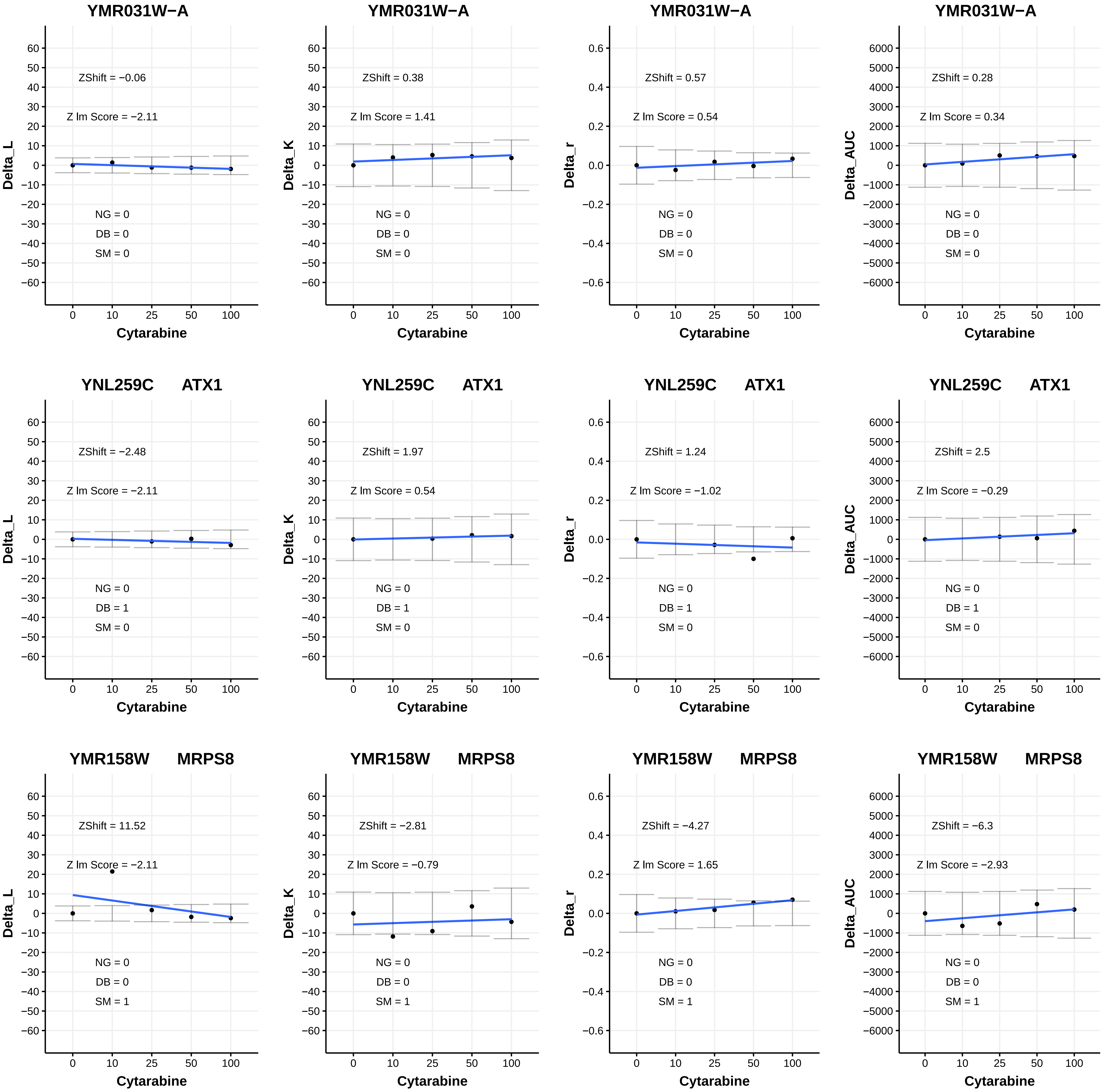

YMR031W−A
ZShift = −0.06
Z lm Score = −2.11
NG = 0
DB = 0
SM = 0
Delta_L
Cytarabine
YMR031W−A
ZShift = 0.38
Z lm Score = 1.41
NG = 0
DB = 0
SM = 0
Delta_K
Cytarabine
YMR031W−A
ZShift = 0.57
Z lm Score = 0.54
NG = 0
DB = 0
SM = 0
Delta_r
Cytarabine
YMR031W−A
ZShift = 0.28
Z lm Score = 0.34
NG = 0
DB = 0
SM = 0
Delta_AUC
Cytarabine
YNL259C ATX1
ZShift = −2.48
Z lm Score = −2.11
NG = 0
DB = 1
SM = 0
Delta_L
Cytarabine
YNL259C ATX1
ZShift = 1.97
Z lm Score = 0.54
NG = 0
DB = 1
SM = 0
Delta_K
Cytarabine
YNL259C ATX1
ZShift = 1.24
Z lm Score = −1.02
NG = 0
DB = 1
SM = 0
Delta_r
Cytarabine
YNL259C ATX1
ZShift = 2.5
Z lm Score = −0.29
NG = 0
DB = 1
SM = 0
Delta_AUC
Cytarabine
YMR158W MRPS8
ZShift = 11.52
Z lm Score = −2.11
NG = 0
DB = 0
SM = 1
Delta_L
Cytarabine
YMR158W MRPS8
ZShift = −2.81
Z lm Score = −0.79
NG = 0
DB = 0
SM = 1
Delta_K
Cytarabine
YMR158W MRPS8
ZShift = −4.27
Z lm Score = 1.65
NG = 0
DB = 0
SM = 1
Delta_r
Cytarabine
YMR158W MRPS8
ZShift = −6.3
Z lm Score = −2.93
NG = 0
DB = 0
SM = 1
Delta_AUC
Cytarabine

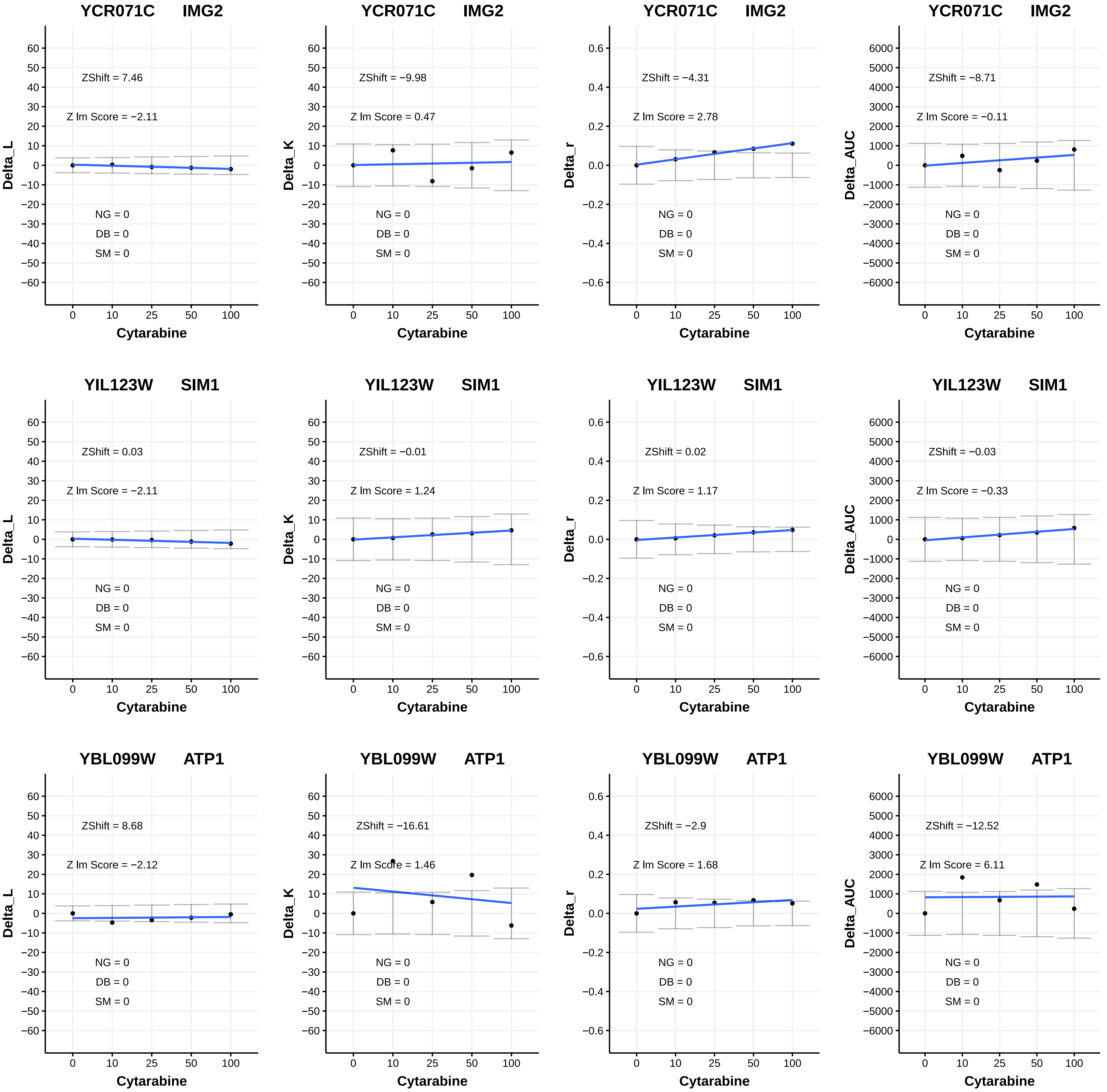

YCR071C IMG2
ZShift = 7.46
Z lm Score = −2.11
NG = 0
DB = 0
SM = 0
Delta_L
Cytarabine
YCR071C IMG2
ZShift = −9.98
Z lm Score = 0.47
NG = 0
DB = 0
SM = 0
Delta_K
Cytarabine
YCR071C IMG2
ZShift = −4.31
Z lm Score = 2.78
NG = 0
DB = 0
SM = 0
Delta_r
Cytarabine
YCR071C IMG2
ZShift = −8.71
Z lm Score = −0.11
NG = 0
DB = 0
SM = 0
Delta_AUC
Cytarabine
YIL123W SIM1
ZShift = 0.03
Z lm Score = −2.11
NG = 0
DB = 0
SM = 0
Delta_L
Cytarabine
YIL123W SIM1
ZShift = −0.01
Z lm Score = 1.24
NG = 0
DB = 0
SM = 0
Delta_K
Cytarabine
YIL123W SIM1
ZShift = 0.02
Z lm Score = 1.17
NG = 0
DB = 0
SM = 0
Delta_r
Cytarabine
YIL123W SIM1
ZShift = −0.03
Z lm Score = −0.33
NG = 0
DB = 0
SM = 0
Delta_AUC
Cytarabine
YBL099W ATP1
ZShift = 8.68
Z lm Score = −2.12
NG = 0
DB = 0
SM = 0
Delta_L
Cytarabine
YBL099W ATP1
ZShift = −16.61
Z lm Score = 1.46
NG = 0
DB = 0
SM = 0
Delta_K
Cytarabine
YBL099W ATP1
ZShift = −2.9
Z lm Score = 1.68
NG = 0
DB = 0
SM = 0
Delta_r
Cytarabine
YBL099W ATP1
ZShift = −12.52
Z lm Score = 6.11
NG = 0
DB = 0
SM = 0
Delta_AUC
Cytarabine

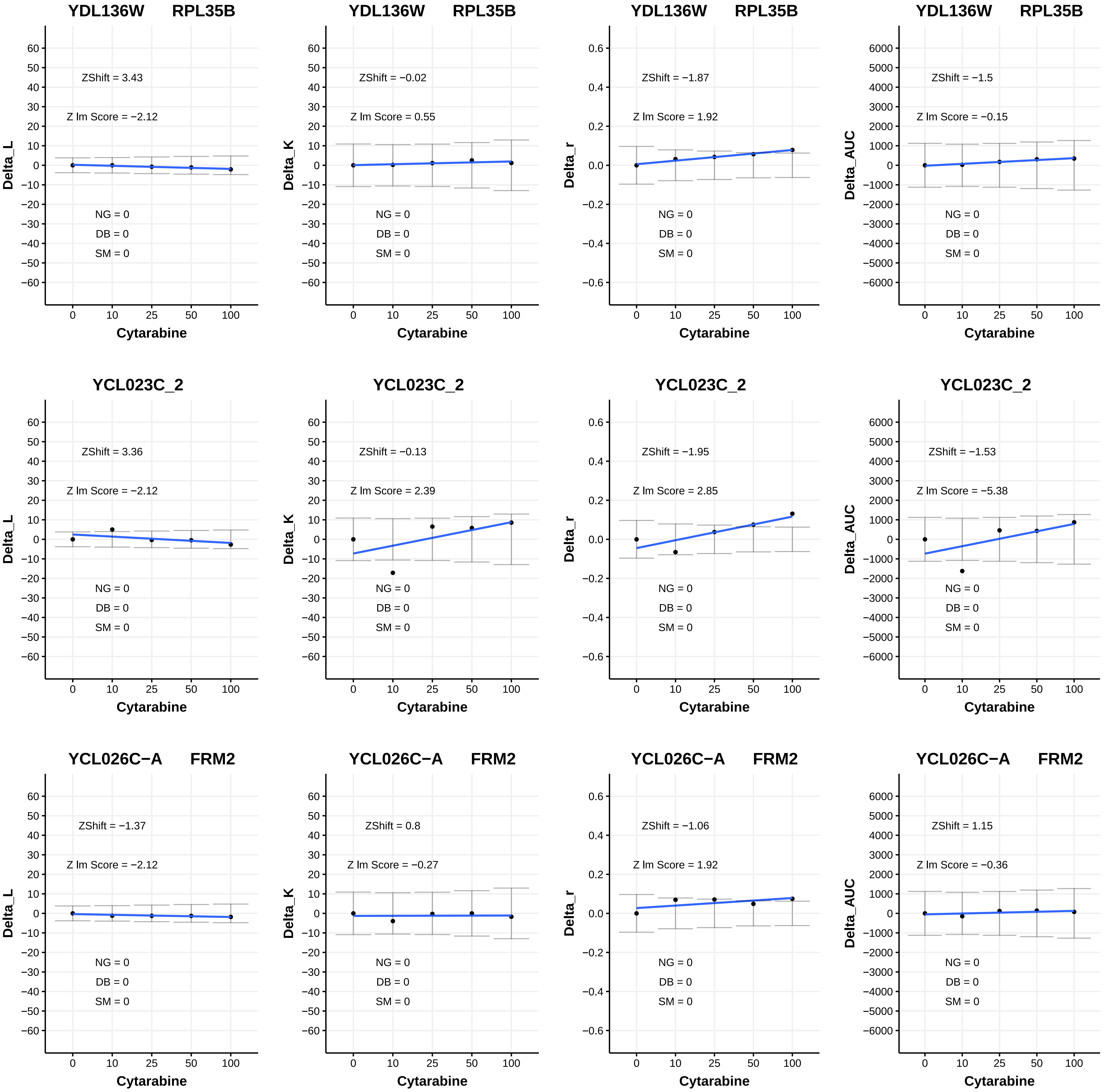

YDL136W RPL35B
ZShift = 3.43
Z lm Score = −2.12
NG = 0
DB = 0
SM = 0
Delta_L
Cytarabine
YDL136W RPL35B
ZShift = −0.02
Z lm Score = 0.55
NG = 0
DB = 0
SM = 0
Delta_K
Cytarabine
YDL136W RPL35B
ZShift = −1.87
Z lm Score = 1.92
NG = 0
DB = 0
SM = 0
Delta_r
Cytarabine
YDL136W RPL35B
ZShift = −1.5
Z lm Score = −0.15
NG = 0
DB = 0
SM = 0
Delta_AUC
Cytarabine
YCL023C_2
ZShift = 3.36
Z lm Score = −2.12
NG = 0
DB = 0
SM = 0
Delta_L
Cytarabine
YCL023C_2
ZShift = −0.13
Z lm Score = 2.39
NG = 0
DB = 0
SM = 0
Delta_K
Cytarabine
YCL023C_2
ZShift = −1.95
Z lm Score = 2.85
NG = 0
DB = 0
SM = 0
Delta_r
Cytarabine
YCL023C_2
ZShift = −1.53
Z lm Score = −5.38
NG = 0
DB = 0
SM = 0
Delta_AUC
Cytarabine
YCL026C−A FRM2
ZShift = −1.37
Z lm Score = −2.12
NG = 0
DB = 0
SM = 0
Delta_L
Cytarabine
YCL026C−A FRM2
ZShift = 0.8
Z lm Score = −0.27
NG = 0
DB = 0
SM = 0
Delta_K
Cytarabine
YCL026C−A FRM2
ZShift = −1.06
Z lm Score = 1.92
NG = 0
DB = 0
SM = 0
Delta_r
Cytarabine
YCL026C−A FRM2
ZShift = 1.15
Z lm Score = −0.36
NG = 0
DB = 0
SM = 0
Delta_AUC
Cytarabine

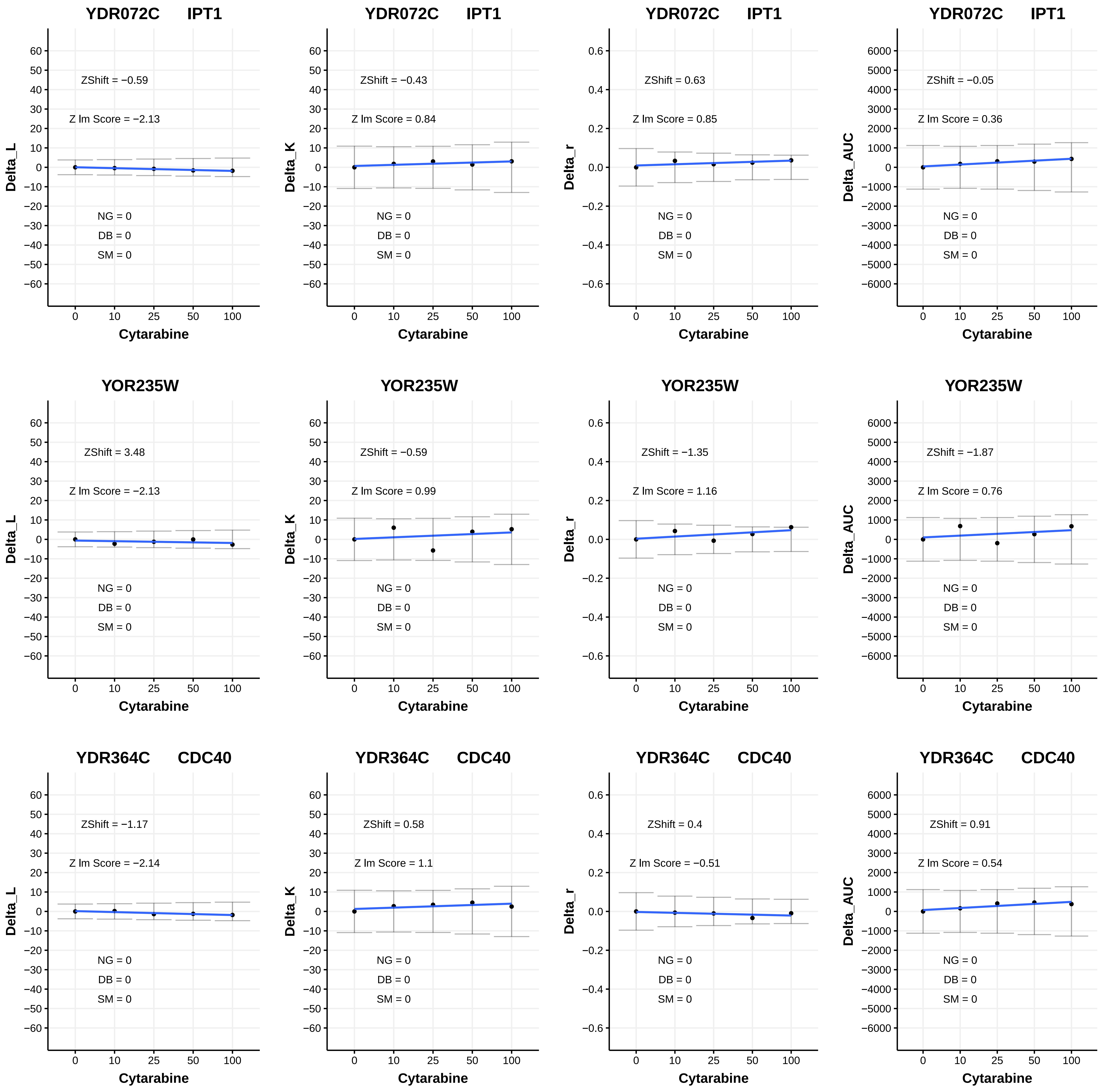

YDR072C IPT1
ZShift = −0.59
Z lm Score = −2.13
NG = 0
DB = 0
SM = 0
Delta_L
Cytarabine
YDR072C IPT1
ZShift = −0.43
Z lm Score = 0.84
NG = 0
DB = 0
SM = 0
Delta_K
Cytarabine
YDR072C IPT1
ZShift = 0.63
Z lm Score = 0.85
NG = 0
DB = 0
SM = 0
Delta_r
Cytarabine
YDR072C IPT1
ZShift = −0.05
Z lm Score = 0.36
NG = 0
DB = 0
SM = 0
Delta_AUC
Cytarabine
YOR235W
ZShift = 3.48
Z lm Score = −2.13
NG = 0
DB = 0
SM = 0
Delta_L
Cytarabine
YOR235W
ZShift = −0.59
Z lm Score = 0.99
NG = 0
DB = 0
SM = 0
Delta_K
Cytarabine
YOR235W
ZShift = −1.35
Z lm Score = 1.16
NG = 0
DB = 0
SM = 0
Delta_r
Cytarabine
YOR235W
ZShift = −1.87
Z lm Score = 0.76
NG = 0
DB = 0
SM = 0
Delta_AUC
Cytarabine
YDR364C CDC40
ZShift = −1.17
Z lm Score = −2.14
NG = 0
DB = 0
SM = 0
Delta_L
Cytarabine
YDR364C CDC40
ZShift = 0.58
Z lm Score = 1.1
NG = 0
DB = 0
SM = 0
Delta_K
Cytarabine
YDR364C CDC40
ZShift = 0.4
Z lm Score = −0.51
NG = 0
DB = 0
SM = 0
Delta_r
Cytarabine
YDR364C CDC40
ZShift = 0.91
Z lm Score = 0.54
NG = 0
DB = 0
SM = 0
Delta_AUC
Cytarabine

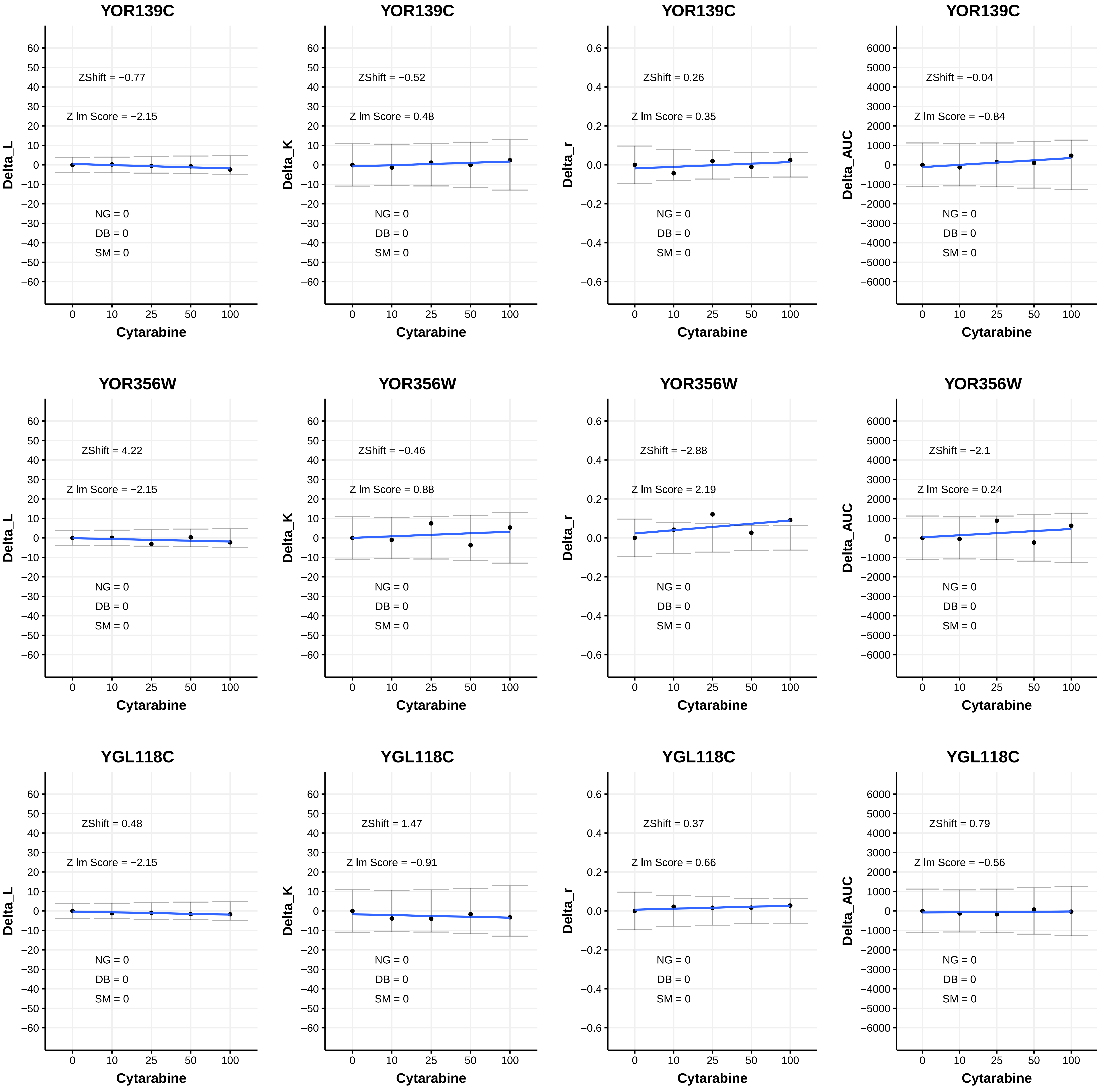

YOR139C
ZShift = −0.77
Z lm Score = −2.15
NG = 0
DB = 0
SM = 0
Delta_L
Cytarabine
YOR139C
ZShift = −0.52
Z lm Score = 0.48
NG = 0
DB = 0
SM = 0
Delta_K
Cytarabine
YOR139C
ZShift = 0.26
Z lm Score = 0.35
NG = 0
DB = 0
SM = 0
Delta_r
Cytarabine
YOR139C
ZShift = −0.04
Z lm Score = −0.84
NG = 0
DB = 0
SM = 0
Delta_AUC
Cytarabine
YOR356W
ZShift = 4.22
Z lm Score = −2.15
NG = 0
DB = 0
SM = 0
Delta_L
Cytarabine
YOR356W
ZShift = −0.46
Z lm Score = 0.88
NG = 0
DB = 0
SM = 0
Delta_K
Cytarabine
YOR356W
ZShift = −2.88
Z lm Score = 2.19
NG = 0
DB = 0
SM = 0
Delta_r
Cytarabine
YOR356W
ZShift = −2.1
Z lm Score = 0.24
NG = 0
DB = 0
SM = 0
Delta_AUC
Cytarabine
YGL118C
ZShift = 0.48
Z lm Score = −2.15
NG = 0
DB = 0
SM = 0
Delta_L
Cytarabine
YGL118C
ZShift = 1.47
Z lm Score = −0.91
NG = 0
DB = 0
SM = 0
Delta_K
Cytarabine
YGL118C
ZShift = 0.37
Z lm Score = 0.66
NG = 0
DB = 0
SM = 0
Delta_r
Cytarabine
YGL118C
ZShift = 0.79
Z lm Score = −0.56
NG = 0
DB = 0
SM = 0
Delta_AUC
Cytarabine

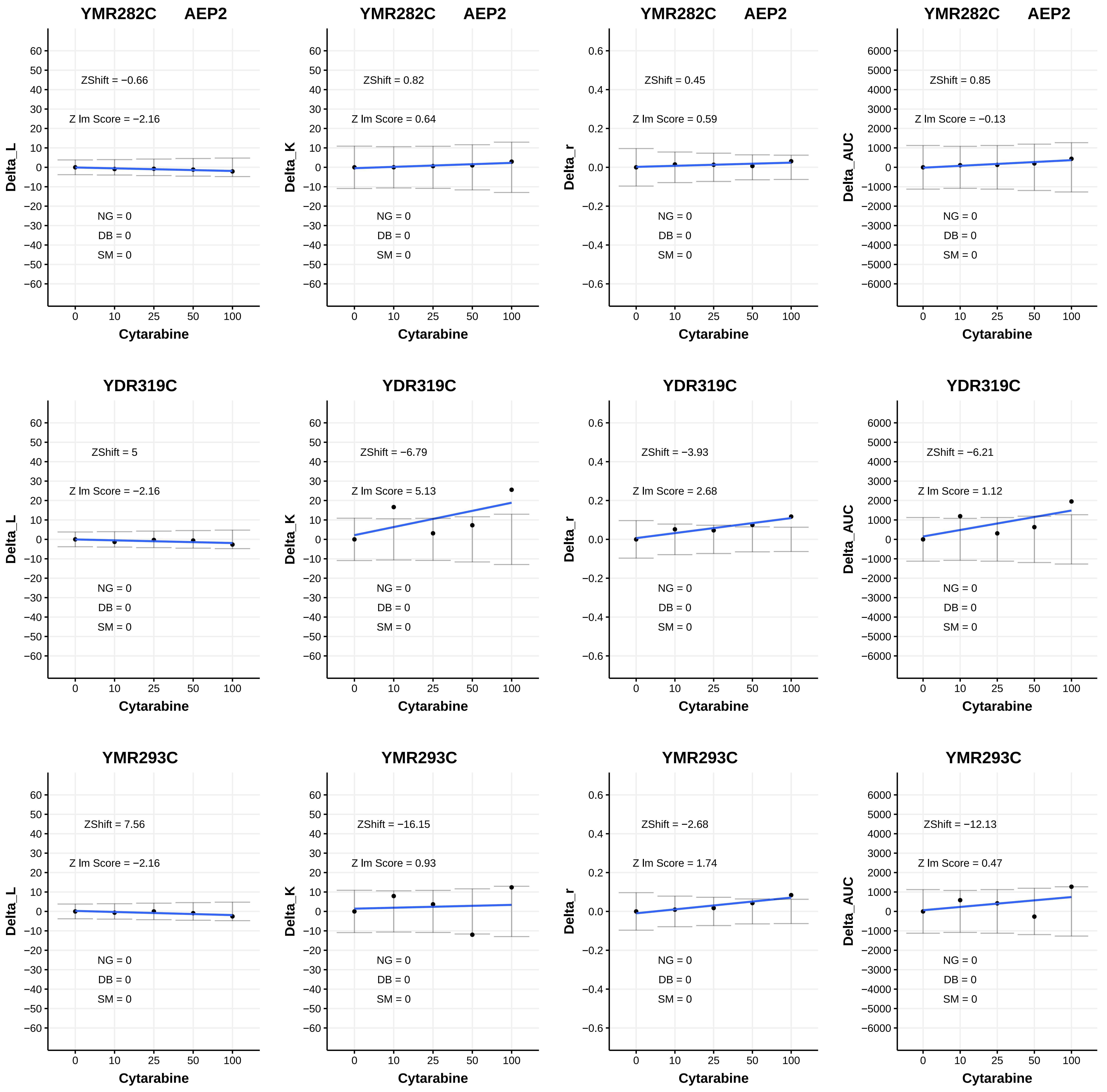

YMR282C AEP2
ZShift = −0.66
Z lm Score = −2.16
NG = 0
DB = 0
SM = 0
Delta_L
Cytarabine
YMR282C AEP2
ZShift = 0.82
Z lm Score = 0.64
NG = 0
DB = 0
SM = 0
Delta_K
Cytarabine
YMR282C AEP2
ZShift = 0.45
Z lm Score = 0.59
NG = 0
DB = 0
SM = 0
Delta_r
Cytarabine
YMR282C AEP2
ZShift = 0.85
Z lm Score = −0.13
NG = 0
DB = 0
SM = 0
Delta_AUC
Cytarabine
YDR319C
ZShift = 5
Z lm Score = −2.16
NG = 0
DB = 0
SM = 0
Delta_L
Cytarabine
YDR319C
ZShift = −6.79
Z lm Score = 5.13
NG = 0
DB = 0
SM = 0
Delta_K
Cytarabine
YDR319C
ZShift = −3.93
Z lm Score = 2.68
NG = 0
DB = 0
SM = 0
Delta_r
Cytarabine
YDR319C
ZShift = −6.21
Z lm Score = 1.12
NG = 0
DB = 0
SM = 0
Delta_AUC
Cytarabine
YMR293C
ZShift = 7.56
Z lm Score = −2.16
NG = 0
DB = 0
SM = 0
Delta_L
Cytarabine
YMR293C
ZShift = −16.15
Z lm Score = 0.93
NG = 0
DB = 0
SM = 0
Delta_K
Cytarabine
YMR293C
ZShift = −2.68
Z lm Score = 1.74
NG = 0
DB = 0
SM = 0
Delta_r
Cytarabine
YMR293C
ZShift = −12.13
Z lm Score = 0.47
NG = 0
DB = 0
SM = 0
Delta_AUC
Cytarabine

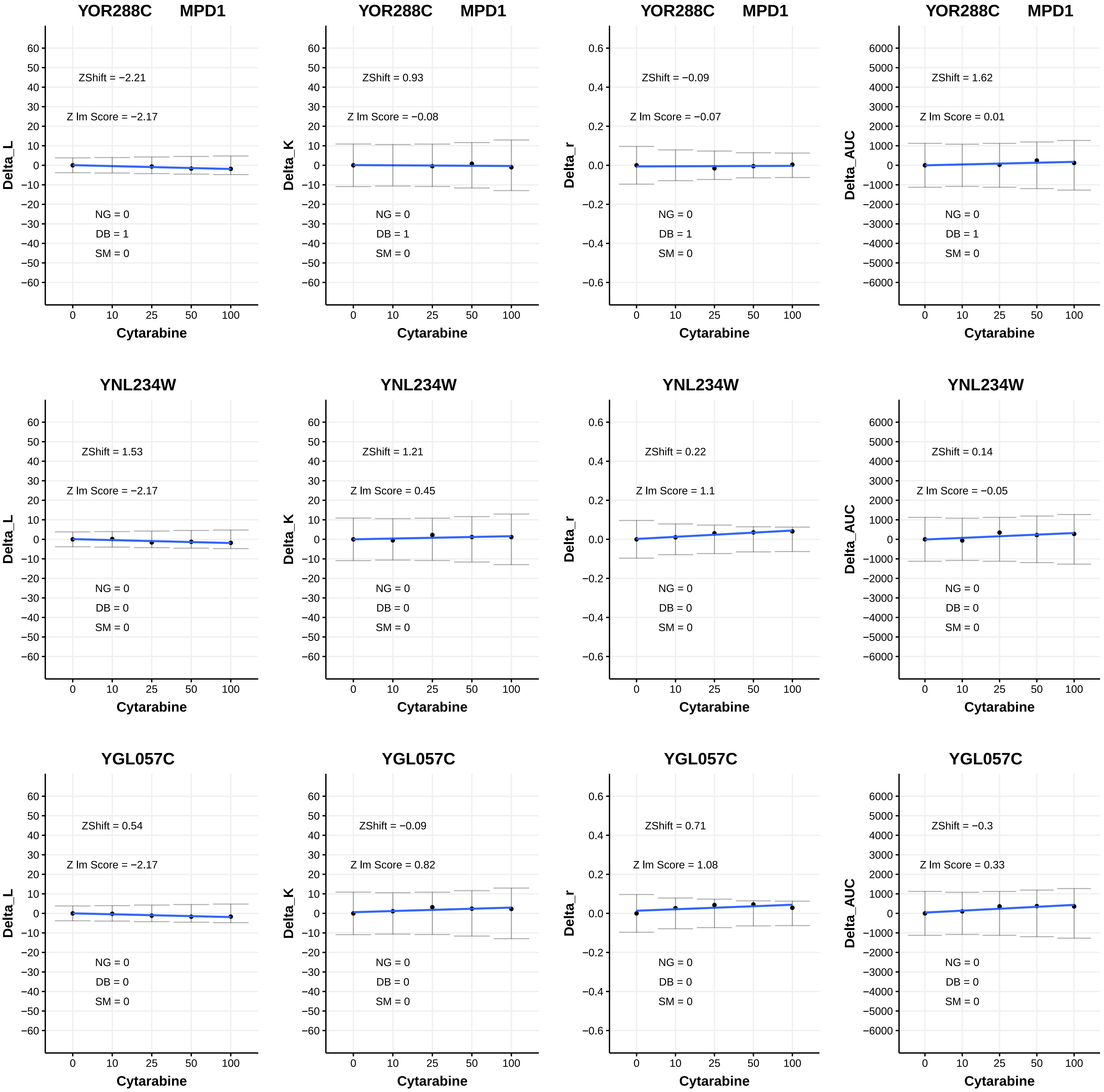
YOR288C MPD1
ZShift = −2.21
Z lm Score = −2.17
NG = 0
DB = 1
SM = 0
Delta_L
Cytarabine
YOR288C MPD1
ZShift = 0.93
Z lm Score = −0.08
NG = 0
DB = 1
SM = 0
Delta_K
Cytarabine
YOR288C MPD1
ZShift = −0.09
Z lm Score = −0.07
NG = 0
DB = 1
SM = 0
Delta_r
Cytarabine
YOR288C MPD1
ZShift = 1.62
Z lm Score = 0.01
NG = 0
DB = 1
SM = 0
Delta_AUC
Cytarabine
YNL234W
ZShift = 1.53
Z lm Score = −2.17
NG = 0
DB = 0
SM = 0
Delta_L
Cytarabine
YNL234W
ZShift = 1.21
Z lm Score = 0.45
NG = 0
DB = 0
SM = 0
Delta_K
Cytarabine
YNL234W
ZShift = 0.22
Z lm Score = 1.1
NG = 0
DB = 0
SM = 0
Delta_r
Cytarabine
YNL234W
ZShift = 0.14
Z lm Score = −0.05
NG = 0
DB = 0
SM = 0
Delta_AUC
Cytarabine
YGL057C
ZShift = 0.54
Z lm Score = −2.17
NG = 0
DB = 0
SM = 0
Delta_L
Cytarabine
YGL057C
ZShift = −0.09
Z lm Score = 0.82
NG = 0
DB = 0
SM = 0
Delta_K
Cytarabine
YGL057C
ZShift = 0.71
Z lm Score = 1.08
NG = 0
DB = 0
SM = 0
Delta_r
Cytarabine
YGL057C
ZShift = −0.3
Z lm Score = 0.33
NG = 0
DB = 0
SM = 0
Delta_AUC
Cytarabine

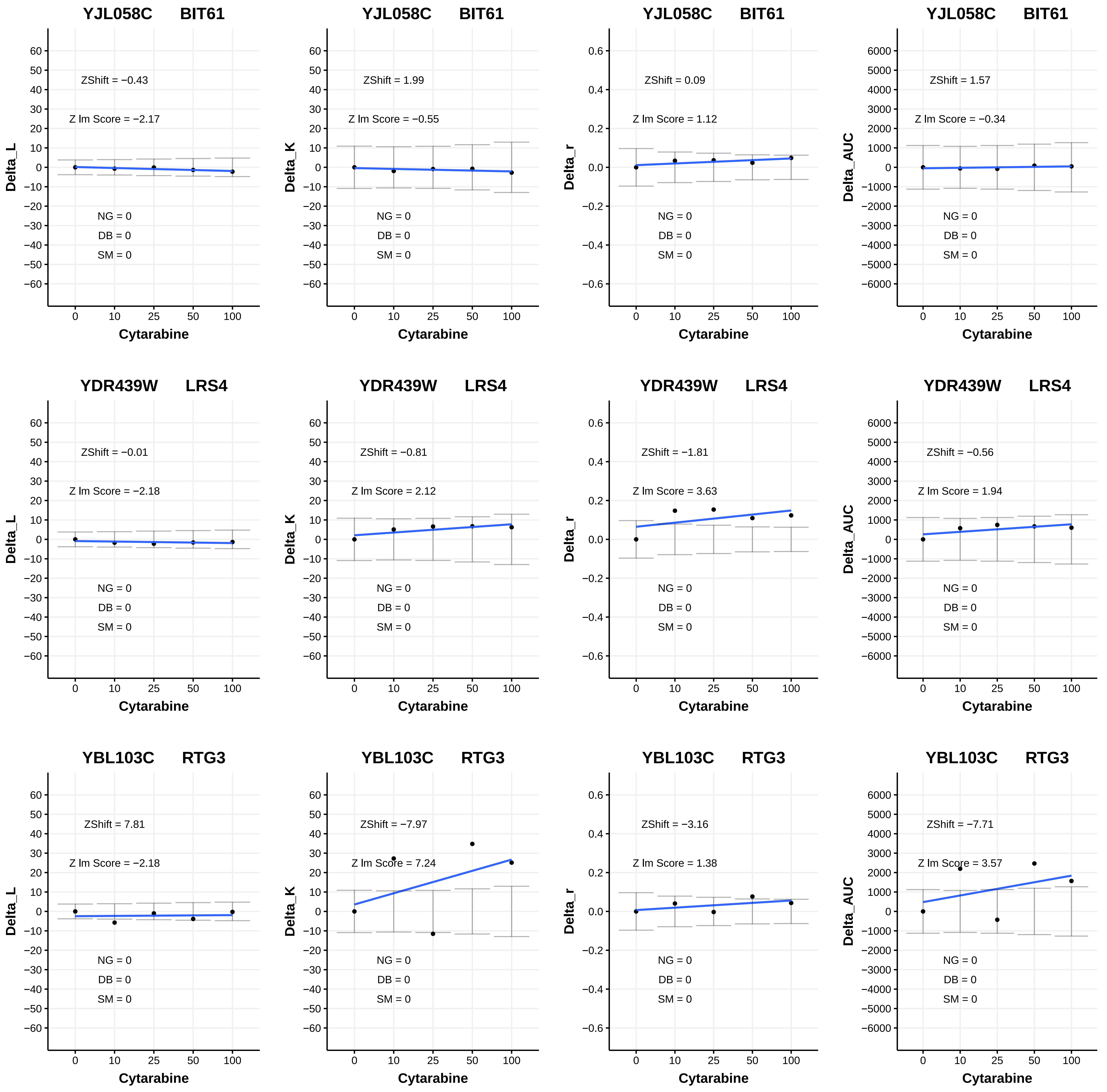

YJL058C BIT61
ZShift = −0.43
Z lm Score = −2.17
NG = 0
DB = 0
SM = 0
Delta_L
Cytarabine
YJL058C BIT61
ZShift = 1.99
Z lm Score = −0.55
NG = 0
DB = 0
SM = 0
Delta_K
Cytarabine
YJL058C BIT61
ZShift = 0.09
Z lm Score = 1.12
NG = 0
DB = 0
SM = 0
Delta_r
Cytarabine
YJL058C BIT61
ZShift = 1.57
Z lm Score = −0.34
NG = 0
DB = 0
SM = 0
Delta_AUC
Cytarabine
YDR439W LRS4
ZShift = −0.01
Z lm Score = −2.18
NG = 0
DB = 0
SM = 0
Delta_L
Cytarabine
YDR439W LRS4
ZShift = −0.81
Z lm Score = 2.12
NG = 0
DB = 0
SM = 0
Delta_K
Cytarabine
YDR439W LRS4
ZShift = −1.81
Z lm Score = 3.63
NG = 0
DB = 0
SM = 0
Delta_r
Cytarabine
YDR439W LRS4
ZShift = −0.56
Z lm Score = 1.94
NG = 0
DB = 0
SM = 0
Delta_AUC
Cytarabine
YBL103C RTG3
ZShift = 7.81
Z lm Score = −2.18
NG = 0
DB = 0
SM = 0
Delta_L
Cytarabine
YBL103C RTG3
ZShift = −7.97
Z lm Score = 7.24
NG = 0
DB = 0
SM = 0
Delta_K
Cytarabine
YBL103C RTG3
ZShift = −3.16
Z lm Score = 1.38
NG = 0
DB = 0
SM = 0
Delta_r
Cytarabine
YBL103C RTG3
ZShift = −7.71
Z lm Score = 3.57
NG = 0
DB = 0
SM = 0
Delta_AUC
Cytarabine

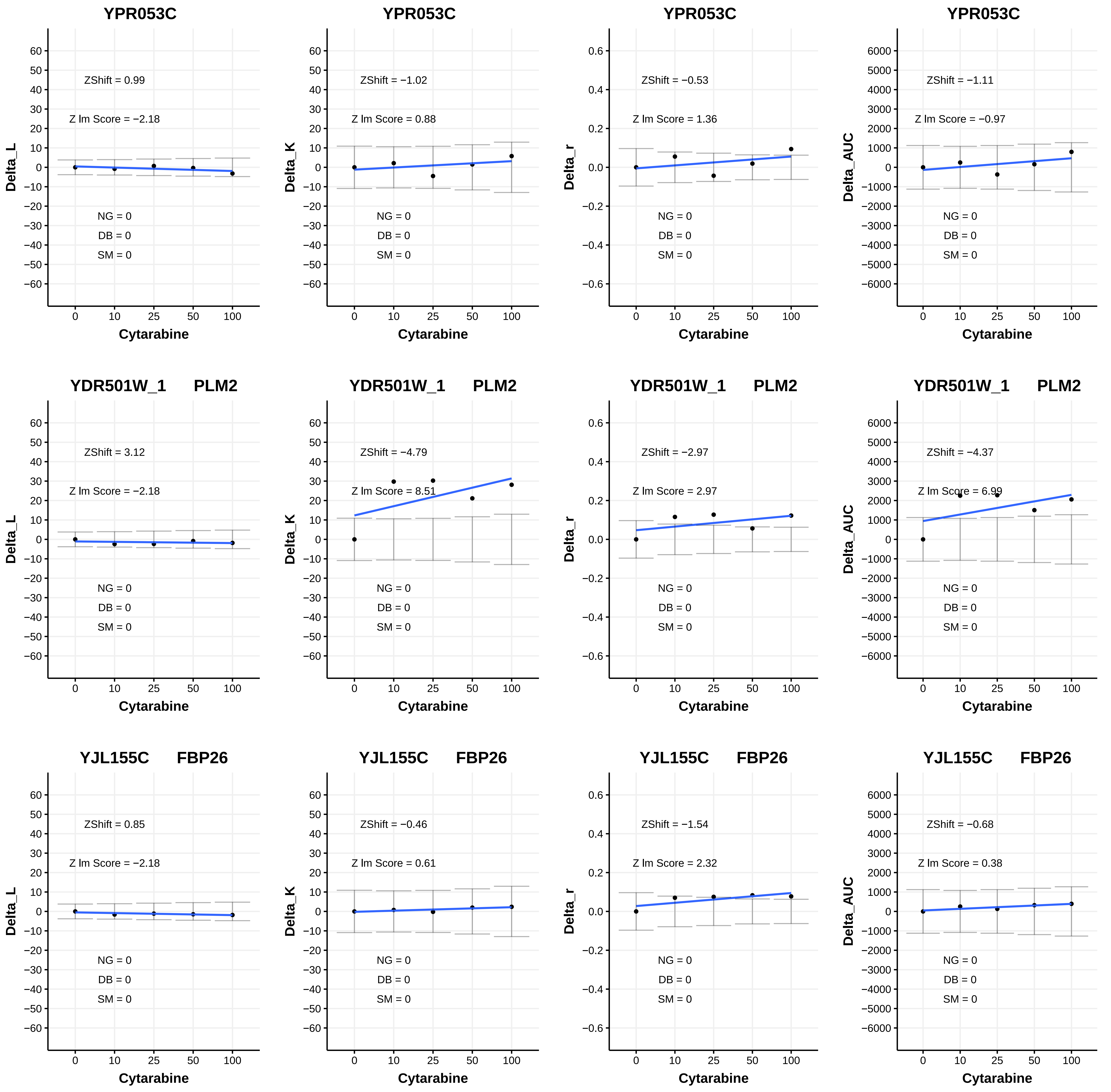
YPR053C
Delta_L
ZShift = 0.99
Z lm Score = −2.18
NG = 0
DB = 0
SM = 0
Cytarabine
YPR053C
Delta_K
ZShift = −1.02
Z lm Score = 0.88
NG = 0
DB = 0
SM = 0
Cytarabine
YPR053C
Delta_r
ZShift = −0.53
Z lm Score = 1.36
NG = 0
DB = 0
SM = 0
Cytarabine
YPR053C
Delta_AUC
ZShift = −1.11
Z lm Score = −0.97
NG = 0
DB = 0
SM = 0
Cytarabine
YDR501W_1 PLM2
Delta_L
ZShift = 3.12
Z lm Score = −2.18
NG = 0
DB = 0
SM = 0
Cytarabine
YDR501W_1 PLM2
Delta_K
ZShift = −4.79
Z lm Score = 8.51
NG = 0
DB = 0
SM = 0
Cytarabine
YDR501W_1 PLM2
Delta_r
ZShift = −2.97
Z lm Score = 2.97
NG = 0
DB = 0
SM = 0
Cytarabine
YDR501W_1 PLM2
Delta_AUC
ZShift = −4.37
Z lm Score = 6.99
NG = 0
DB = 0
SM = 0
Cytarabine
YJL155C FBP26
Delta_L
ZShift = 0.85
Z lm Score = −2.18
NG = 0
DB = 0
SM = 0
Cytarabine
YJL155C FBP26
Delta_K
ZShift = −0.46
Z lm Score = 0.61
NG = 0
DB = 0
SM = 0
Cytarabine
YJL155C FBP26
Delta_r
ZShift = −1.54
Z lm Score = 2.32
NG = 0
DB = 0
SM = 0
Cytarabine
YJL155C FBP26
Delta_AUC
ZShift = −0.68
Z lm Score = 0.38
NG = 0
DB = 0
SM = 0
Cytarabine

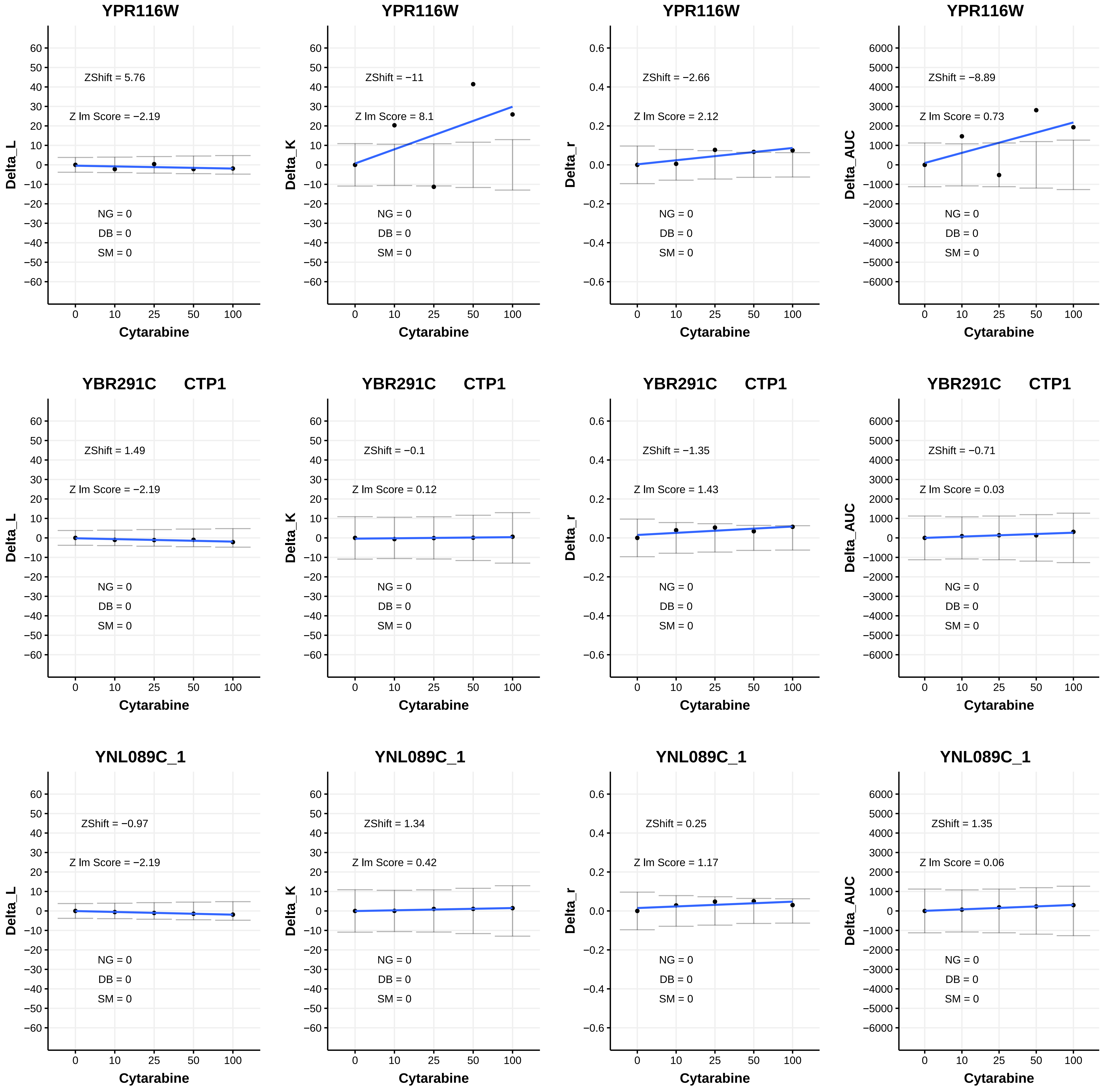
YPR116W
ZShift = 5.76
Z lm Score = −2.19
NG = 0
DB = 0
SM = 0
Delta_L
Cytarabine
YPR116W
ZShift = −11
Z lm Score = 8.1
NG = 0
DB = 0
SM = 0
Delta_K
Cytarabine
YPR116W
ZShift = −2.66
Z lm Score = 2.12
NG = 0
DB = 0
SM = 0
Delta_r
Cytarabine
YPR116W
ZShift = −8.89
Z lm Score = 0.73
NG = 0
DB = 0
SM = 0
Delta_AUC
Cytarabine
YBR291C CTP1
ZShift = 1.49
Z lm Score = −2.19
NG = 0
DB = 0
SM = 0
Delta_L
Cytarabine
YBR291C CTP1
ZShift = −0.1
Z lm Score = 0.12
NG = 0
DB = 0
SM = 0
Delta_K
Cytarabine
YBR291C CTP1
ZShift = −1.35
Z lm Score = 1.43
NG = 0
DB = 0
SM = 0
Delta_r
Cytarabine
YBR291C CTP1
ZShift = −0.71
Z lm Score = 0.03
NG = 0
DB = 0
SM = 0
Delta_AUC
Cytarabine
YNL089C_1
ZShift = −0.97
Z lm Score = −2.19
NG = 0
DB = 0
SM = 0
Delta_L
Cytarabine
YNL089C_1
ZShift = 1.34
Z lm Score = 0.42
NG = 0
DB = 0
SM = 0
Delta_K
Cytarabine
YNL089C_1
ZShift = 0.25
Z lm Score = 1.17
NG = 0
DB = 0
SM = 0
Delta_r
Cytarabine
YNL089C_1
ZShift = 1.35
Z lm Score = 0.06
NG = 0
DB = 0
SM = 0
Delta_AUC
Cytarabine

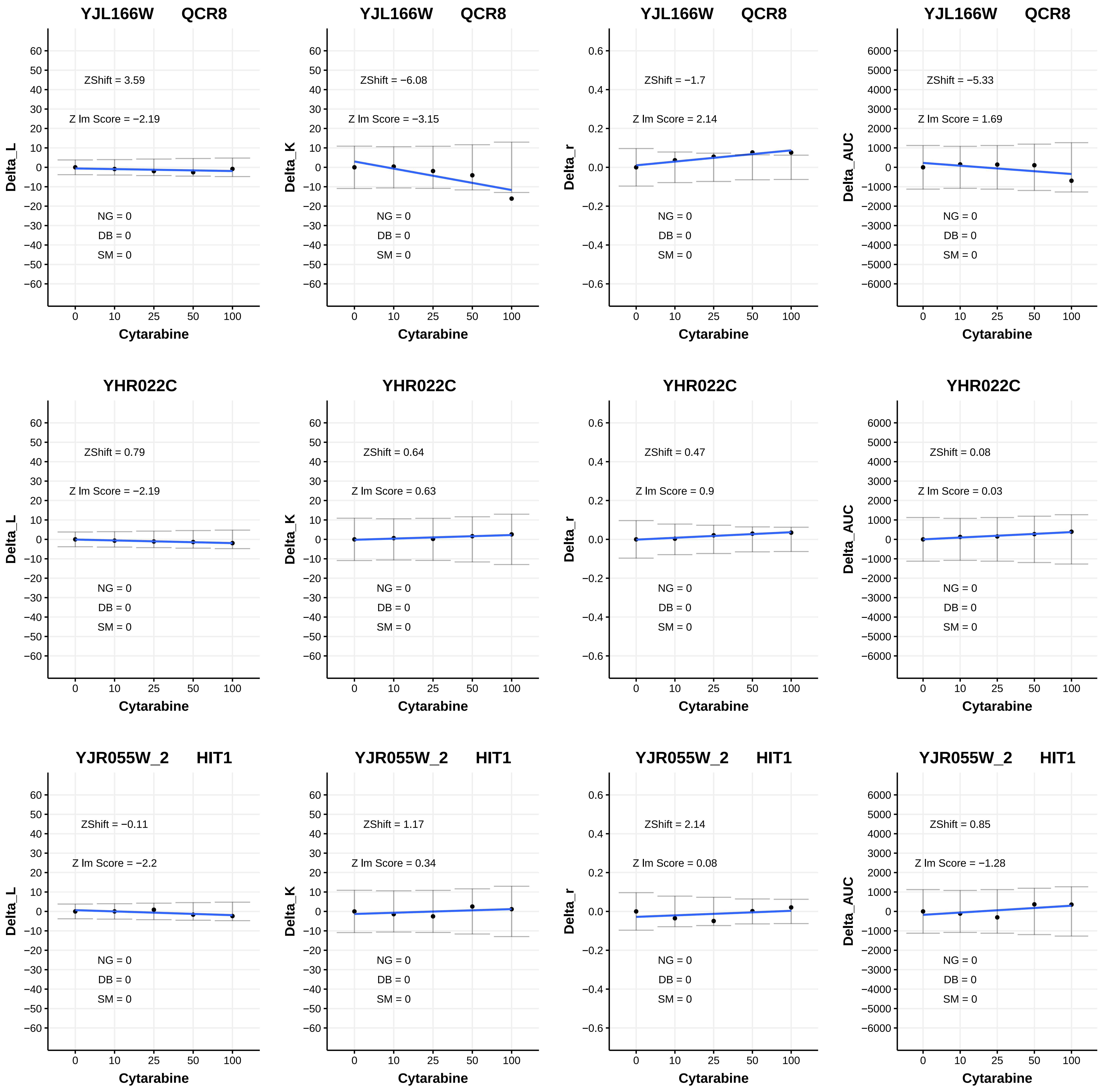

YJL166W QCR8
ZShift = 3.59
Z lm Score = −2.19
NG = 0
DB = 0
SM = 0
Delta_L
Cytarabine
YJL166W QCR8
ZShift = −6.08
Z lm Score = −3.15
NG = 0
DB = 0
SM = 0
Delta_K
Cytarabine
YJL166W QCR8
ZShift = −1.7
Z lm Score = 2.14
NG = 0
DB = 0
SM = 0
Delta_r
Cytarabine
YJL166W QCR8
ZShift = −5.33
Z lm Score = 1.69
NG = 0
DB = 0
SM = 0
Delta_AUC
Cytarabine
YHR022C
ZShift = 0.79
Z lm Score = −2.19
NG = 0
DB = 0
SM = 0
Delta_L
Cytarabine
YHR022C
ZShift = 0.64
Z lm Score = 0.63
NG = 0
DB = 0
SM = 0
Delta_K
Cytarabine
YHR022C
ZShift = 0.47
Z lm Score = 0.9
NG = 0
DB = 0
SM = 0
Delta_r
Cytarabine
YHR022C
ZShift = 0.08
Z lm Score = 0.03
NG = 0
DB = 0
SM = 0
Delta_AUC
Cytarabine
YJR055W_2 HIT1
ZShift = −0.11
Z lm Score = −2.2
NG = 0
DB = 0
SM = 0
Delta_L
Cytarabine
YJR055W_2 HIT1
ZShift = 1.17
Z lm Score = 0.34
NG = 0
DB = 0
SM = 0
Delta_K
Cytarabine
YJR055W_2 HIT1
ZShift = 2.14
Z lm Score = 0.08
NG = 0
DB = 0
SM = 0
Delta_r
Cytarabine
YJR055W_2 HIT1
ZShift = 0.85
Z lm Score = −1.28
NG = 0
DB = 0
SM = 0
Delta_AUC
Cytarabine

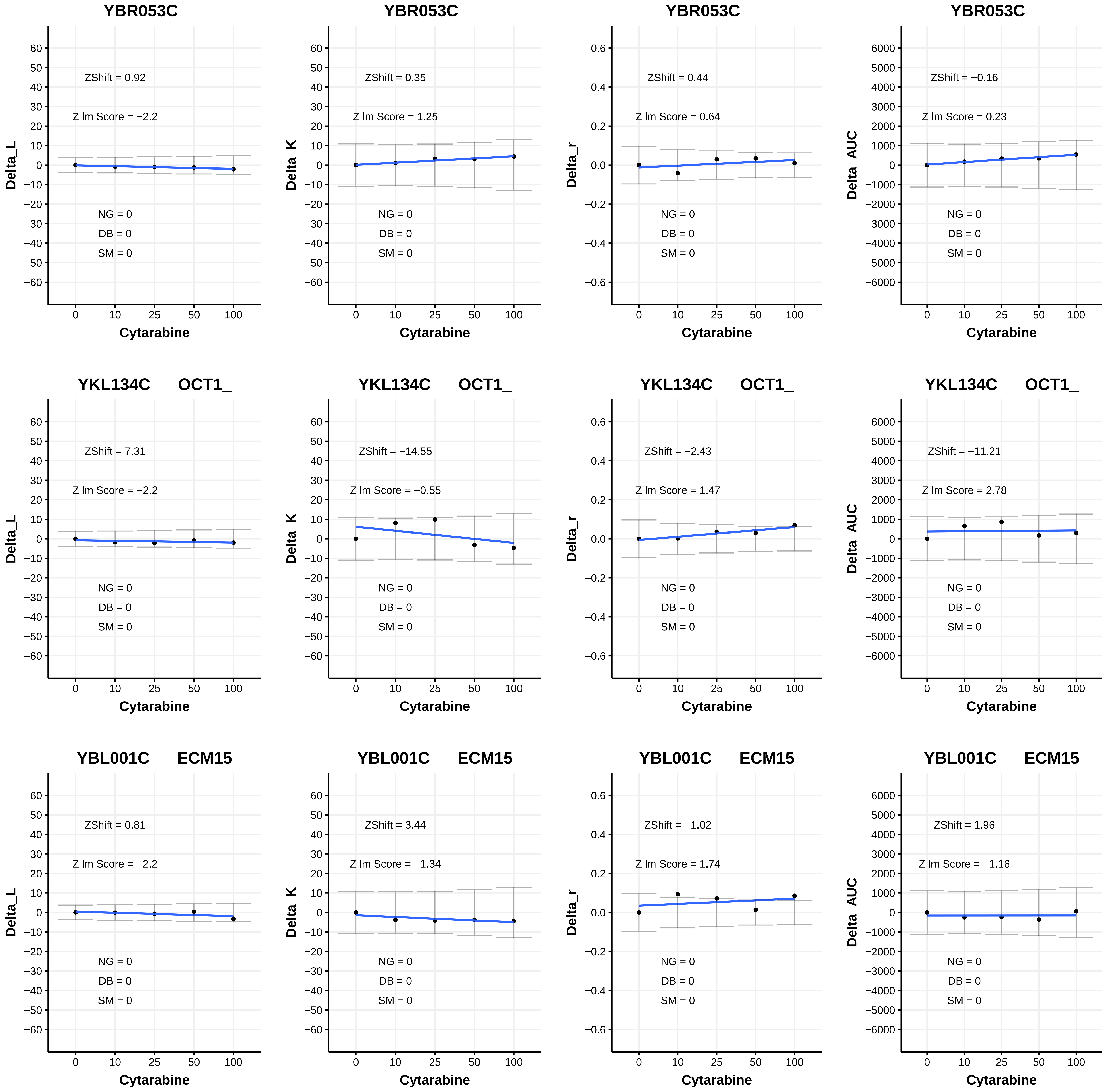
YBR053C
ZShift = 0.92
Z lm Score = −2.2
NG = 0
DB = 0
SM = 0
Delta_L
Cytarabine
YBR053C
ZShift = 0.35
Z lm Score = 1.25
NG = 0
DB = 0
SM = 0
Delta_K
Cytarabine
YBR053C
ZShift = 0.44
Z lm Score = 0.64
NG = 0
DB = 0
SM = 0
Delta_r
Cytarabine
YBR053C
ZShift = −0.16
Z lm Score = 0.23
NG = 0
DB = 0
SM = 0
Delta_AUC
Cytarabine
YKL134C OCT1_
ZShift = 7.31
Z lm Score = −2.2
NG = 0
DB = 0
SM = 0
Delta_L
Cytarabine
YKL134C OCT1_
ZShift = −14.55
Z lm Score = −0.55
NG = 0
DB = 0
SM = 0
Delta_K
Cytarabine
YKL134C OCT1_
ZShift = −2.43
Z lm Score = 1.47
NG = 0
DB = 0
SM = 0
Delta_r
Cytarabine
YKL134C OCT1_
ZShift = −11.21
Z lm Score = 2.78
NG = 0
DB = 0
SM = 0
Delta_AUC
Cytarabine
YBL001C ECM15
ZShift = 0.81
Z lm Score = −2.2
NG = 0
DB = 0
SM = 0
Delta_L
Cytarabine
YBL001C ECM15
ZShift = 3.44
Z lm Score = −1.34
NG = 0
DB = 0
SM = 0
Delta_K
Cytarabine
YBL001C ECM15
ZShift = −1.02
Z lm Score = 1.74
NG = 0
DB = 0
SM = 0
Delta_r
Cytarabine
YBL001C ECM15
ZShift = 1.96
Z lm Score = −1.16
NG = 0
DB = 0
SM = 0
Delta_AUC
Cytarabine

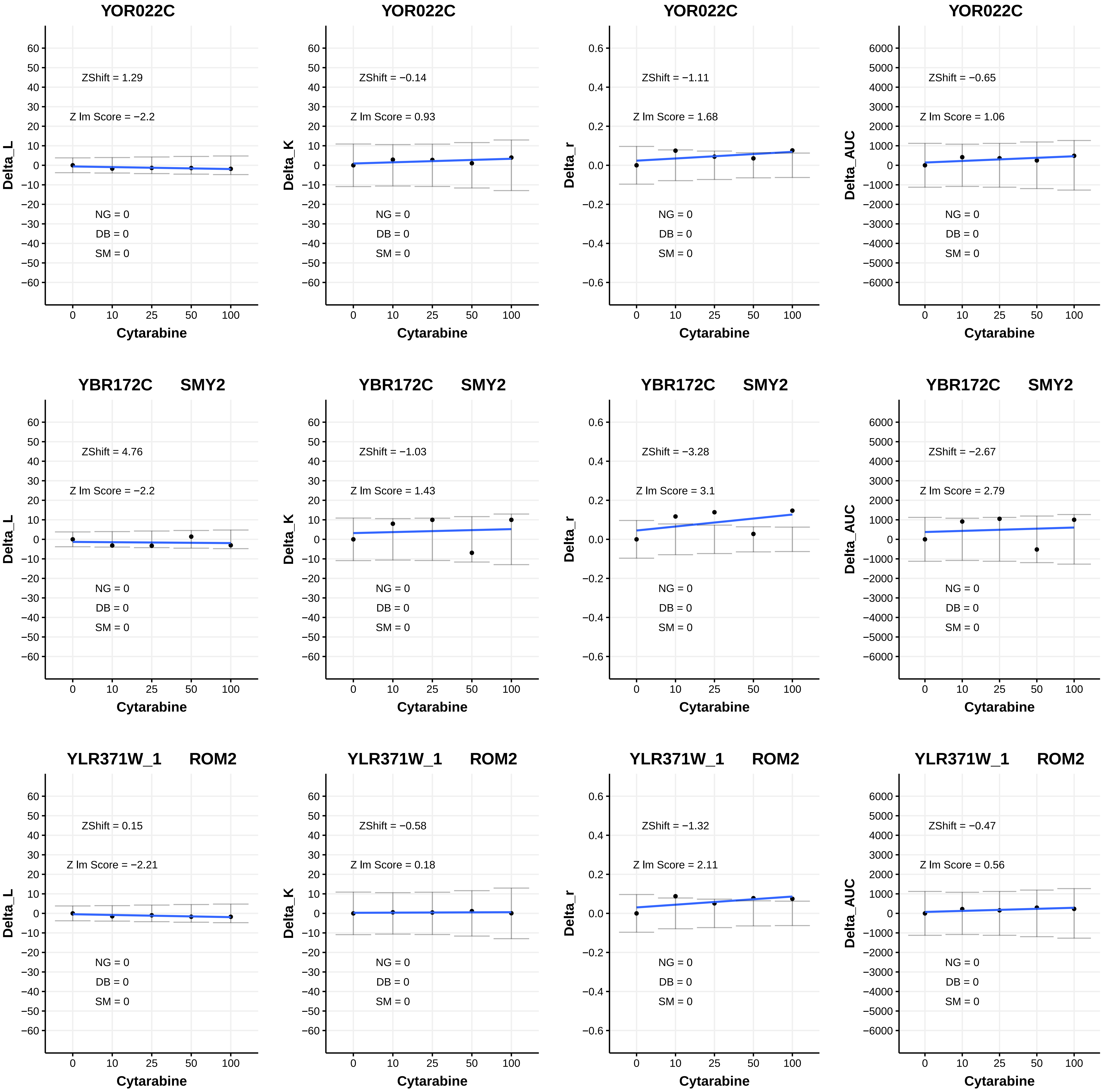
YOR022C
Delta_L
ZShift = 1.29
Z lm Score = −2.2
NG = 0
DB = 0
SM = 0
Cytarabine
YOR022C
Delta_K
ZShift = −0.14
Z lm Score = 0.93
NG = 0
DB = 0
SM = 0
Cytarabine
YOR022C
Delta_r
ZShift = −1.11
Z lm Score = 1.68
NG = 0
DB = 0
SM = 0
Cytarabine
YOR022C
Delta_AUC
ZShift = −0.65
Z lm Score = 1.06
NG = 0
DB = 0
SM = 0
Cytarabine
YBR172C SMY2
Delta_L
ZShift = 4.76
Z lm Score = −2.2
NG = 0
DB = 0
SM = 0
Cytarabine
YBR172C SMY2
Delta_K
ZShift = −1.03
Z lm Score = 1.43
NG = 0
DB = 0
SM = 0
Cytarabine
YBR172C SMY2
Delta_r
ZShift = −3.28
Z lm Score = 3.1
NG = 0
DB = 0
SM = 0
Cytarabine
YBR172C SMY2
Delta_AUC
ZShift = −2.67
Z lm Score = 2.79
NG = 0
DB = 0
SM = 0
Cytarabine
YLR371W_1 ROM2
Delta_L
ZShift = 0.15
Z lm Score = −2.21
NG = 0
DB = 0
SM = 0
Cytarabine
YLR371W_1 ROM2
Delta_K
ZShift = −0.58
Z lm Score = 0.18
NG = 0
DB = 0
SM = 0
Cytarabine
YLR371W_1 ROM2
Delta_r
ZShift = −1.32
Z lm Score = 2.11
NG = 0
DB = 0
SM = 0
Cytarabine
YLR371W_1 ROM2
Delta_AUC
ZShift = −0.47
Z lm Score = 0.56
NG = 0
DB = 0
SM = 0
Cytarabine

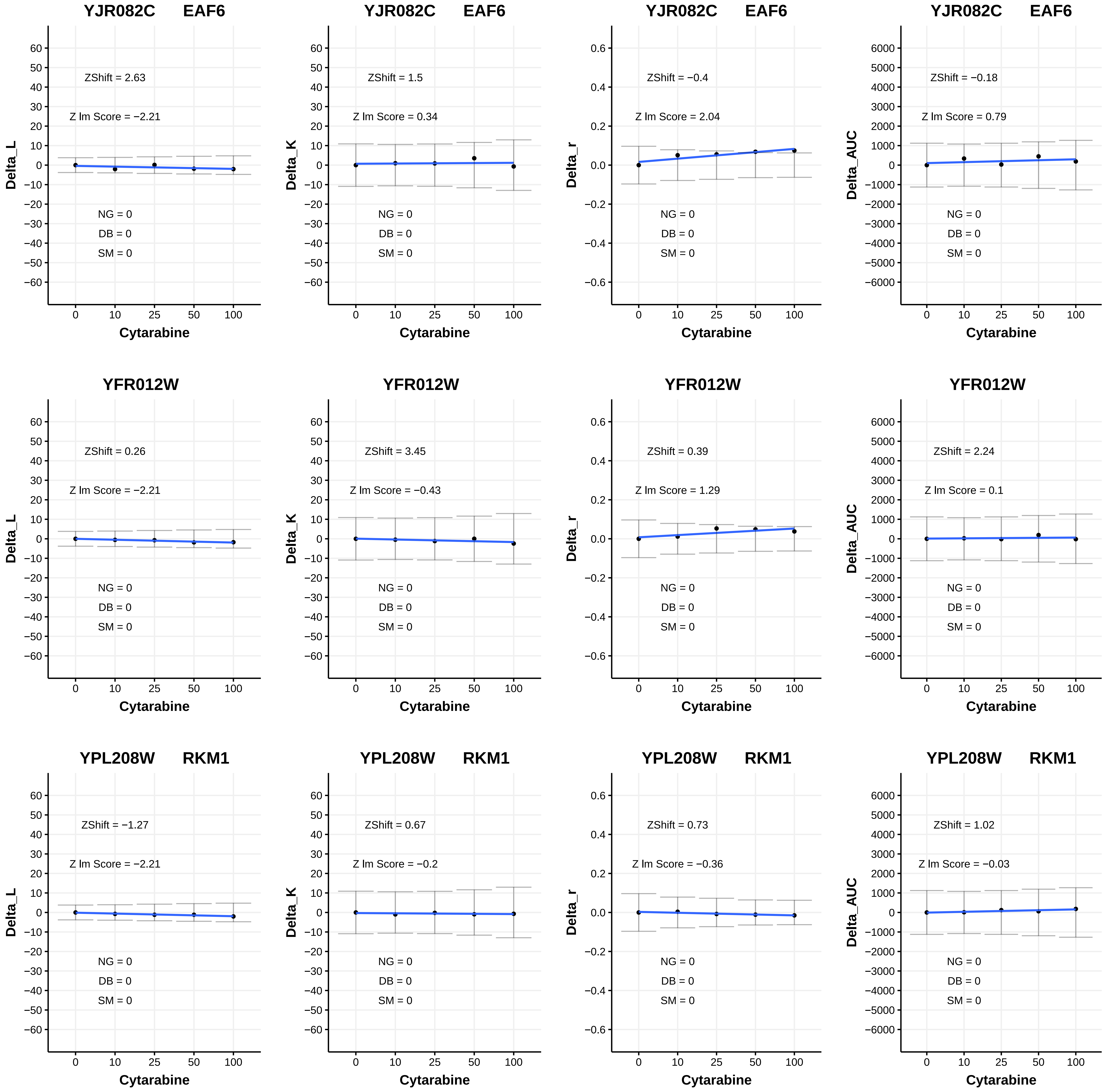

YJR082C EAF6
ZShift = 2.63
Z lm Score = −2.21
NG = 0
DB = 0
SM = 0
Delta_L
Cytarabine
YJR082C EAF6
ZShift = 1.5
Z lm Score = 0.34
NG = 0
DB = 0
SM = 0
Delta_K
Cytarabine
YJR082C EAF6
ZShift = −0.4
Z lm Score = 2.04
NG = 0
DB = 0
SM = 0
Delta_r
Cytarabine
YJR082C EAF6
ZShift = −0.18
Z lm Score = 0.79
NG = 0
DB = 0
SM = 0
Delta_AUC
Cytarabine
YFR012W
ZShift = 0.26
Z lm Score = −2.21
NG = 0
DB = 0
SM = 0
Delta_L
Cytarabine
YFR012W
ZShift = 3.45
Z lm Score = −0.43
NG = 0
DB = 0
SM = 0
Delta_K
Cytarabine
YFR012W
ZShift = 0.39
Z lm Score = 1.29
NG = 0
DB = 0
SM = 0
Delta_r
Cytarabine
YFR012W
ZShift = 2.24
Z lm Score = 0.1
NG = 0
DB = 0
SM = 0
Delta_AUC
Cytarabine
YPL208W RKM1
ZShift = −1.27
Z lm Score = −2.21
NG = 0
DB = 0
SM = 0
Delta_L
Cytarabine
YPL208W RKM1
ZShift = 0.67
Z lm Score = −0.2
NG = 0
DB = 0
SM = 0
Delta_K
Cytarabine
YPL208W RKM1
ZShift = 0.73
Z lm Score = −0.36
NG = 0
DB = 0
SM = 0
Delta_r
Cytarabine
YPL208W RKM1
ZShift = 1.02
Z lm Score = −0.03
NG = 0
DB = 0
SM = 0
Delta_AUC
Cytarabine

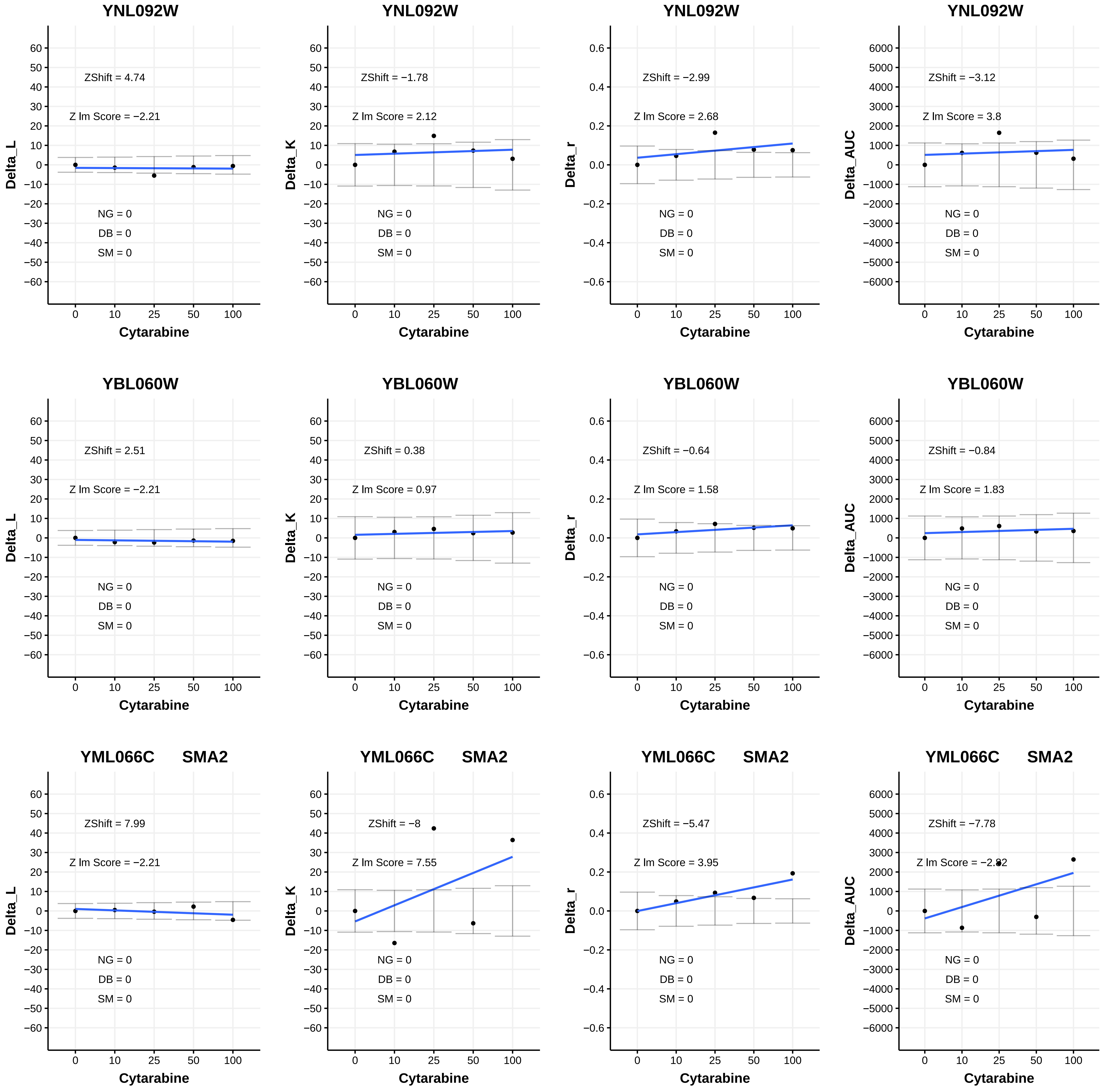
YNL092W
ZShift = 4.74
Z lm Score = −2.21
NG = 0
DB = 0
SM = 0
Delta_L
Cytarabine
YNL092W
ZShift = −1.78
Z lm Score = 2.12
NG = 0
DB = 0
SM = 0
Delta_K
Cytarabine
YNL092W
ZShift = −2.99
Z lm Score = 2.68
NG = 0
DB = 0
SM = 0
Delta_r
Cytarabine
YNL092W
ZShift = −3.12
Z lm Score = 3.8
NG = 0
DB = 0
SM = 0
Delta_AUC
Cytarabine
YBL060W
ZShift = 2.51
Z lm Score = −2.21
NG = 0
DB = 0
SM = 0
Delta_L
Cytarabine
YBL060W
ZShift = 0.38
Z lm Score = 0.97
NG = 0
DB = 0
SM = 0
Delta_K
Cytarabine
YBL060W
ZShift = −0.64
Z lm Score = 1.58
NG = 0
DB = 0
SM = 0
Delta_r
Cytarabine
YBL060W
ZShift = −0.84
Z lm Score = 1.83
NG = 0
DB = 0
SM = 0
Delta_AUC
Cytarabine
YML066C SMA2
ZShift = 7.99
Z lm Score = −2.21
NG = 0
DB = 0
SM = 0
Delta_L
Cytarabine
YML066C SMA2
ZShift = −8
Z lm Score = 7.55
NG = 0
DB = 0
SM = 0
Delta_K
Cytarabine
YML066C SMA2
ZShift = −5.47
Z lm Score = 3.95
NG = 0
DB = 0
SM = 0
Delta_r
Cytarabine
YML066C SMA2
ZShift = −7.78
Z lm Score = −2.82
NG = 0
DB = 0
SM = 0
Delta_AUC
Cytarabine

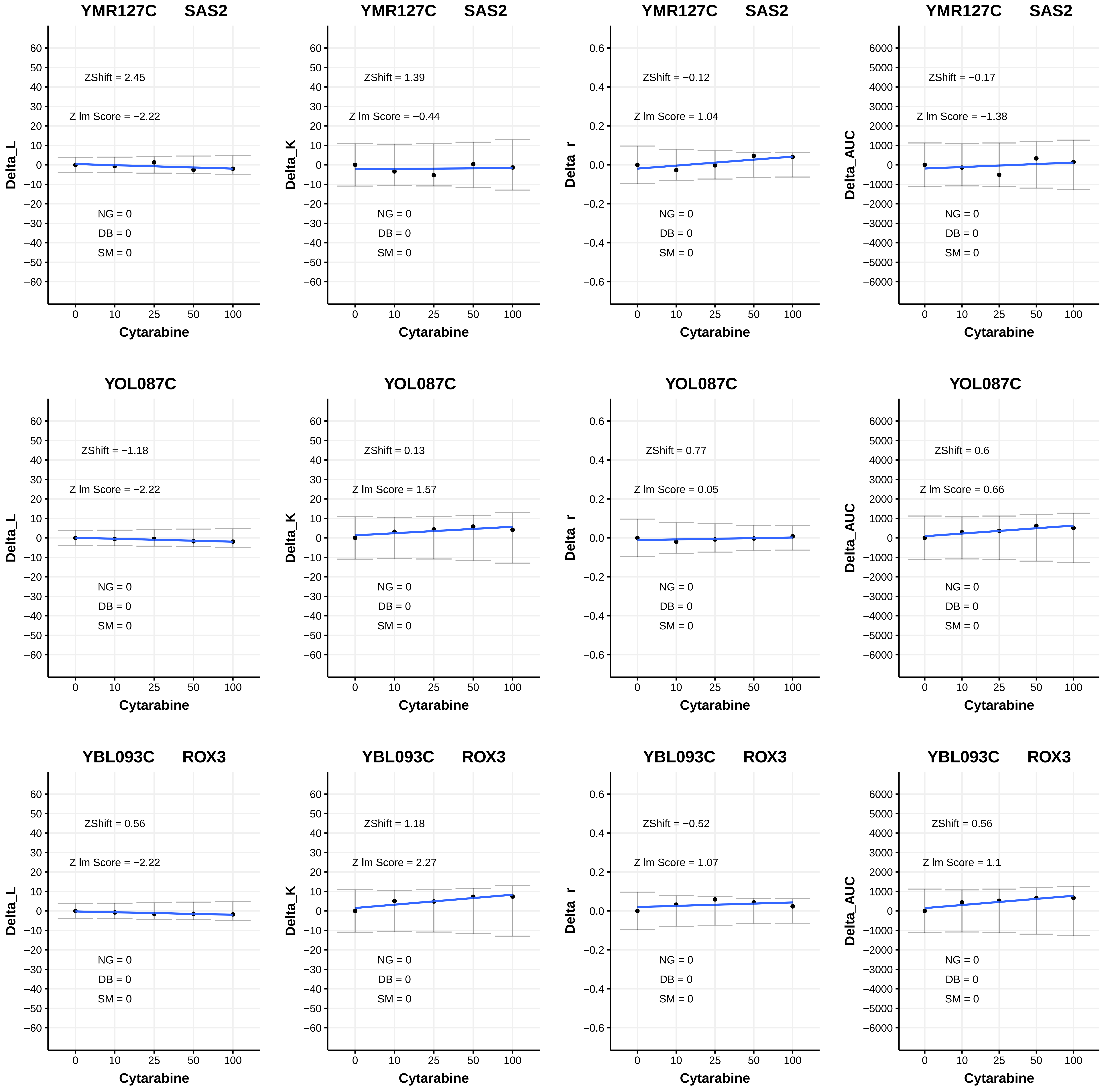
YMR127C SAS2
ZShift = 2.45
Z lm Score = −2.22
NG = 0
DB = 0
SM = 0
Delta_L
Cytarabine
YMR127C SAS2
ZShift = 1.39
Z lm Score = −0.44
NG = 0
DB = 0
SM = 0
Delta_K
Cytarabine
YMR127C SAS2
ZShift = −0.12
Z lm Score = 1.04
NG = 0
DB = 0
SM = 0
Delta_r
Cytarabine
YMR127C SAS2
ZShift = −0.17
Z lm Score = −1.38
NG = 0
DB = 0
SM = 0
Delta_AUC
Cytarabine
YOL087C
ZShift = −1.18
Z lm Score = −2.22
NG = 0
DB = 0
SM = 0
Delta_L
Cytarabine
YOL087C
ZShift = 0.13
Z lm Score = 1.57
NG = 0
DB = 0
SM = 0
Delta_K
Cytarabine
YOL087C
ZShift = 0.77
Z lm Score = 0.05
NG = 0
DB = 0
SM = 0
Delta_r
Cytarabine
YOL087C
ZShift = 0.6
Z lm Score = 0.66
NG = 0
DB = 0
SM = 0
Delta_AUC
Cytarabine
YBL093C ROX3
ZShift = 0.56
Z lm Score = −2.22
NG = 0
DB = 0
SM = 0
Delta_L
Cytarabine
YBL093C ROX3
ZShift = 1.18
Z lm Score = 2.27
NG = 0
DB = 0
SM = 0
Delta_K
Cytarabine
YBL093C ROX3
ZShift = −0.52
Z lm Score = 1.07
NG = 0
DB = 0
SM = 0
Delta_r
Cytarabine
YBL093C ROX3
ZShift = 0.56
Z lm Score = 1.1
NG = 0
DB = 0
SM = 0
Delta_AUC
Cytarabine

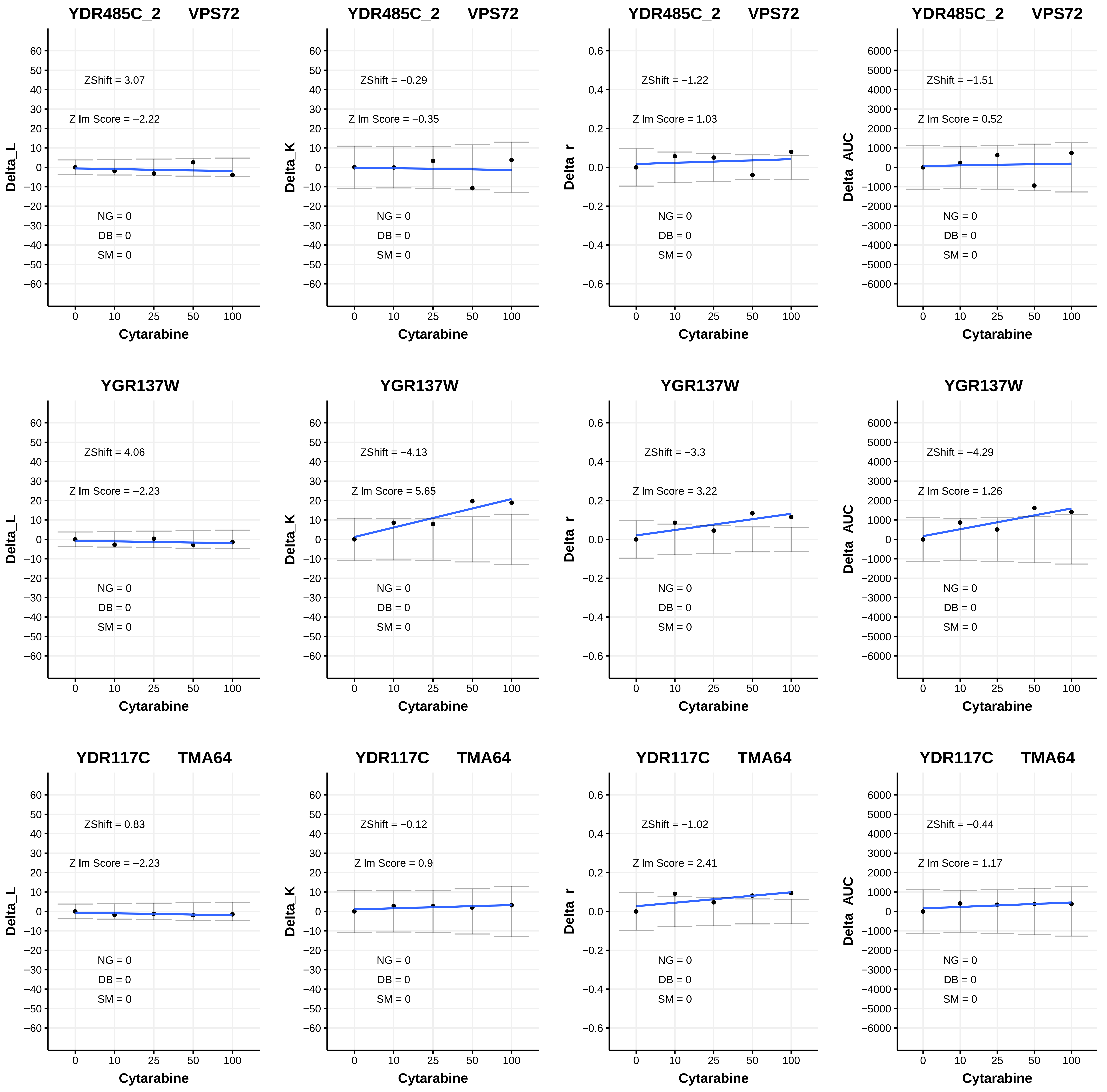

YDR485C_2 VPS72
Delta_L
ZShift = 3.07
Z lm Score = −2.22
NG = 0
DB = 0
SM = 0
Cytarabine
YDR485C_2 VPS72
Delta_K
ZShift = −0.29
Z lm Score = −0.35
NG = 0
DB = 0
SM = 0
Cytarabine
YDR485C_2 VPS72
Delta_r
ZShift = −1.22
Z lm Score = 1.03
NG = 0
DB = 0
SM = 0
Cytarabine
YDR485C_2 VPS72
Delta_AUC
ZShift = −1.51
Z lm Score = 0.52
NG = 0
DB = 0
SM = 0
Cytarabine
YGR137W
Delta_L
ZShift = 4.06
Z lm Score = −2.23
NG = 0
DB = 0
SM = 0
Cytarabine
YGR137W
Delta_K
ZShift = −4.13
Z lm Score = 5.65
NG = 0
DB = 0
SM = 0
Cytarabine
YGR137W
Delta_r
ZShift = −3.3
Z lm Score = 3.22
NG = 0
DB = 0
SM = 0
Cytarabine
YGR137W
Delta_AUC
ZShift = −4.29
Z lm Score = 1.26
NG = 0
DB = 0
SM = 0
Cytarabine
YDR117C TMA64
Delta_L
ZShift = 0.83
Z lm Score = −2.23
NG = 0
DB = 0
SM = 0
Cytarabine
YDR117C TMA64
Delta_K
ZShift = −0.12
Z lm Score = 0.9
NG = 0
DB = 0
SM = 0
Cytarabine
YDR117C TMA64
Delta_r
ZShift = −1.02
Z lm Score = 2.41
NG = 0
DB = 0
SM = 0
Cytarabine
YDR117C TMA64
Delta_AUC
ZShift = −0.44
Z lm Score = 1.17
NG = 0
DB = 0
SM = 0
Cytarabine

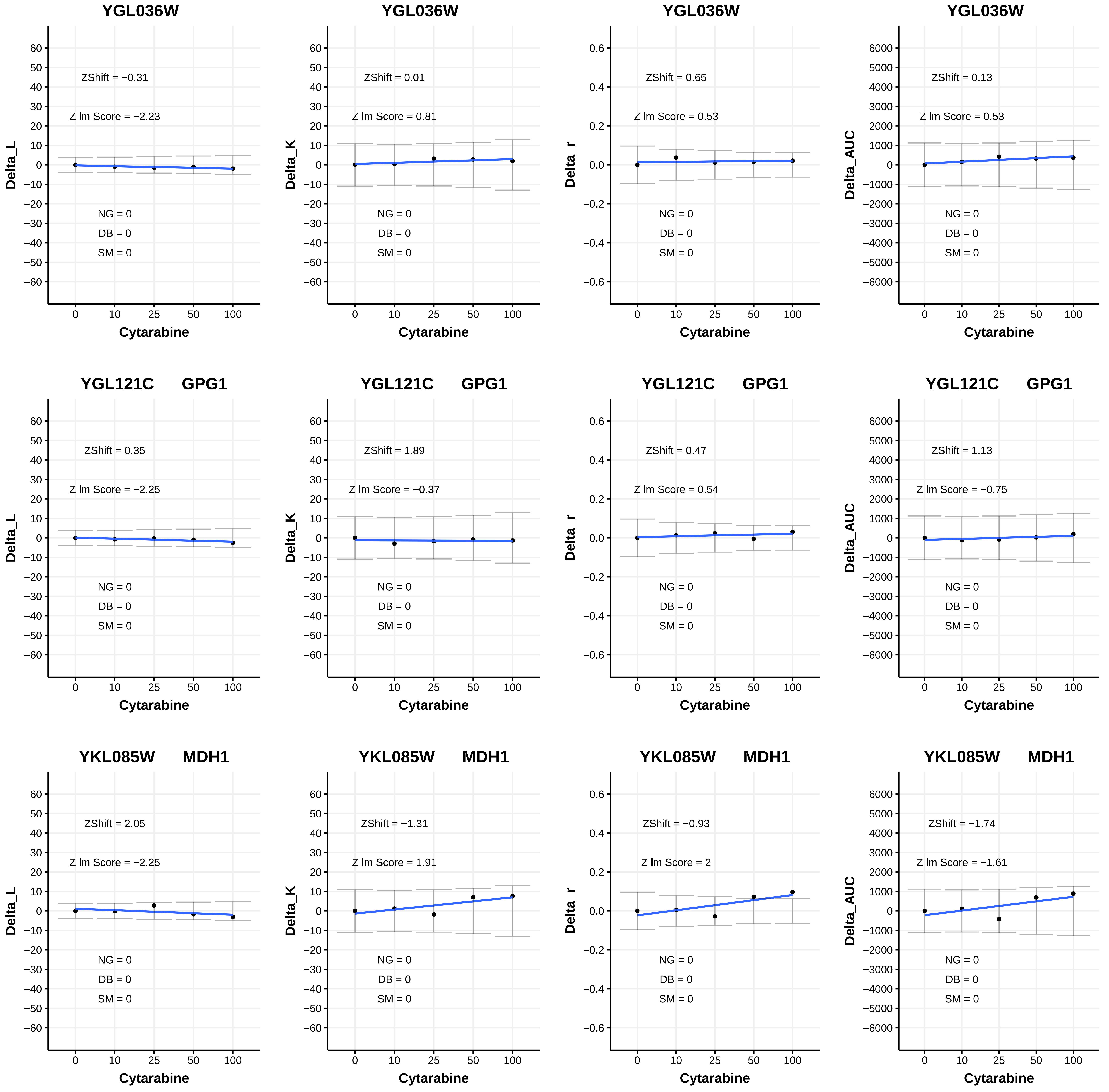
YGL036W
ZShift = −0.31
Z lm Score = −2.23
NG = 0
DB = 0
SM = 0
Delta_L
Cytarabine
YGL036W
ZShift = 0.01
Z lm Score = 0.81
NG = 0
DB = 0
SM = 0
Delta_K
Cytarabine
YGL036W
ZShift = 0.65
Z lm Score = 0.53
NG = 0
DB = 0
SM = 0
Delta_r
Cytarabine
YGL036W
ZShift = 0.13
Z lm Score = 0.53
NG = 0
DB = 0
SM = 0
Delta_AUC
Cytarabine
YGL121C GPG1
ZShift = 0.35
Z lm Score = −2.25
NG = 0
DB = 0
SM = 0
Delta_L
Cytarabine
YGL121C GPG1
ZShift = 1.89
Z lm Score = −0.37
NG = 0
DB = 0
SM = 0
Delta_K
Cytarabine
YGL121C GPG1
ZShift = 0.47
Z lm Score = 0.54
NG = 0
DB = 0
SM = 0
Delta_r
Cytarabine
YGL121C GPG1
ZShift = 1.13
Z lm Score = −0.75
NG = 0
DB = 0
SM = 0
Delta_AUC
Cytarabine
YKL085W MDH1
ZShift = 2.05
Z lm Score = −2.25
NG = 0
DB = 0
SM = 0
Delta_L
Cytarabine
YKL085W MDH1
ZShift = −1.31
Z lm Score = 1.91
NG = 0
DB = 0
SM = 0
Delta_K
Cytarabine
YKL085W MDH1
ZShift = −0.93
Z lm Score = 2
NG = 0
DB = 0
SM = 0
Delta_r
Cytarabine
YKL085W MDH1
ZShift = −1.74
Z lm Score = −1.61
NG = 0
DB = 0
SM = 0
Delta_AUC
Cytarabine

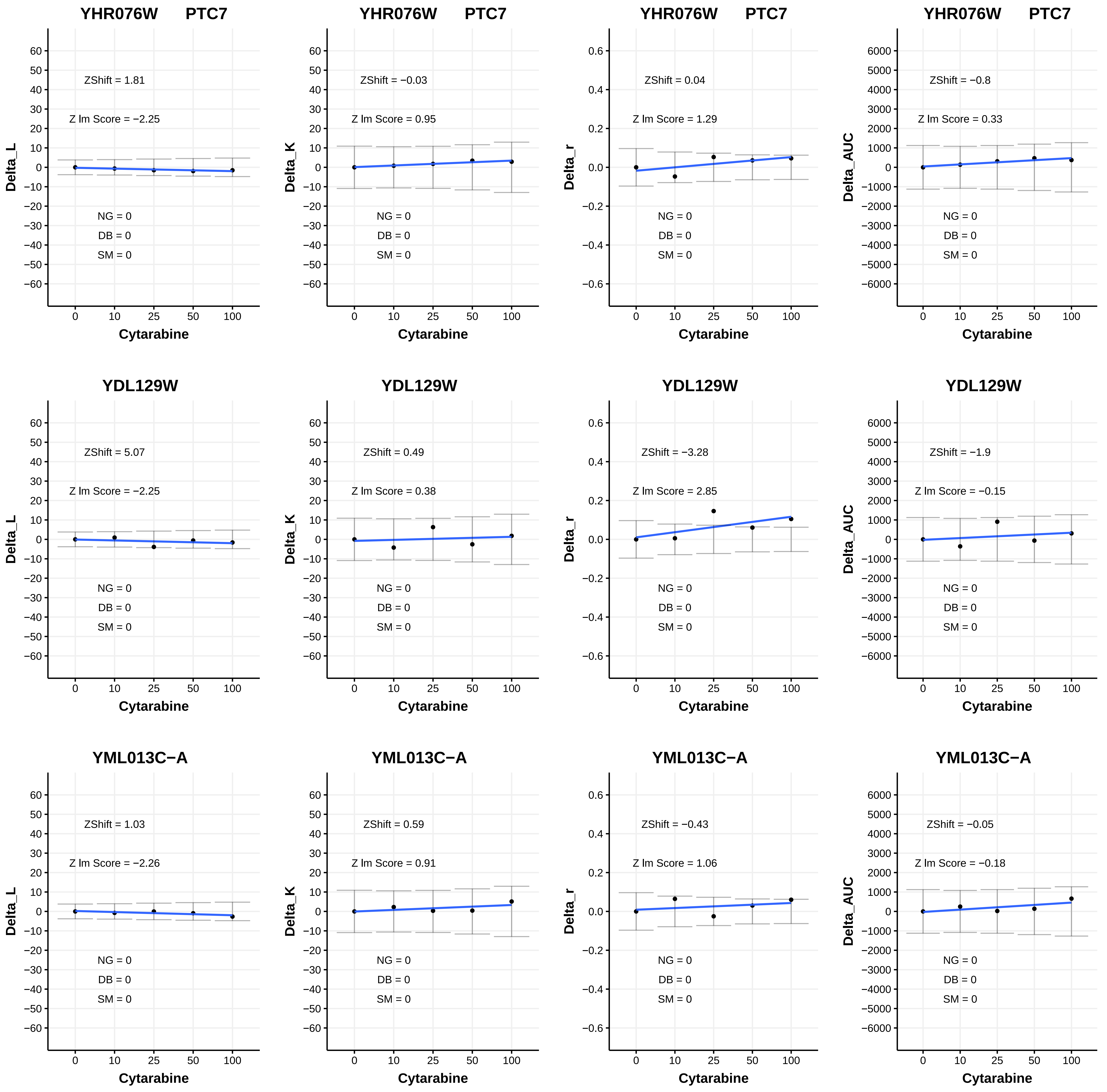

YHR076W PTC7
ZShift = 1.81
Z lm Score = −2.25
NG = 0
DB = 0
SM = 0
Delta_L
Cytarabine
YHR076W PTC7
ZShift = −0.03
Z lm Score = 0.95
NG = 0
DB = 0
SM = 0
Delta_K
Cytarabine
YHR076W PTC7
ZShift = 0.04
Z lm Score = 1.29
NG = 0
DB = 0
SM = 0
Delta_r
Cytarabine
YHR076W PTC7
ZShift = −0.8
Z lm Score = 0.33
NG = 0
DB = 0
SM = 0
Delta_AUC
Cytarabine
YDL129W
ZShift = 5.07
Z lm Score = −2.25
NG = 0
DB = 0
SM = 0
Delta_L
Cytarabine
YDL129W
ZShift = 0.49
Z lm Score = 0.38
NG = 0
DB = 0
SM = 0
Delta_K
Cytarabine
YDL129W
ZShift = −3.28
Z lm Score = 2.85
NG = 0
DB = 0
SM = 0
Delta_r
Cytarabine
YDL129W
ZShift = −1.9
Z lm Score = −0.15
NG = 0
DB = 0
SM = 0
Delta_AUC
Cytarabine
YML013C−A
ZShift = 1.03
Z lm Score = −2.26
NG = 0
DB = 0
SM = 0
Delta_L
Cytarabine
YML013C−A
ZShift = 0.59
Z lm Score = 0.91
NG = 0
DB = 0
SM = 0
Delta_K
Cytarabine
YML013C−A
ZShift = −0.43
Z lm Score = 1.06
NG = 0
DB = 0
SM = 0
Delta_r
Cytarabine
YML013C−A
ZShift = −0.05
Z lm Score = −0.18
NG = 0
DB = 0
SM = 0
Delta_AUC
Cytarabine

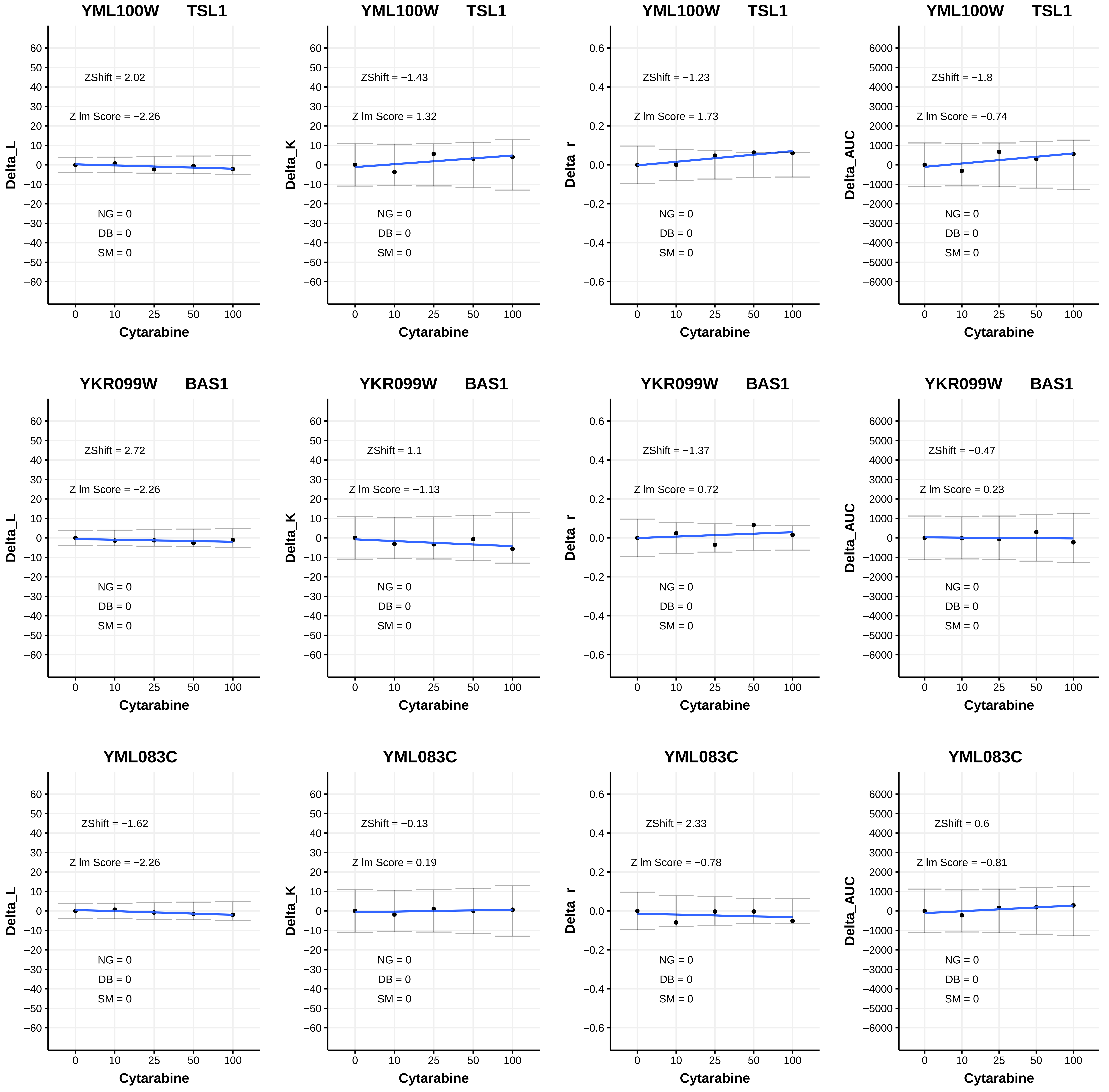
YML100W TSL1
ZShift = 2.02
Z lm Score = −2.26
NG = 0
DB = 0
SM = 0
Delta_L
Cytarabine
YML100W TSL1
ZShift = −1.43
Z lm Score = 1.32
NG = 0
DB = 0
SM = 0
Delta_K
Cytarabine
YML100W TSL1
ZShift = −1.23
Z lm Score = 1.73
NG = 0
DB = 0
SM = 0
Delta_r
Cytarabine
YML100W TSL1
ZShift = −1.8
Z lm Score = −0.74
NG = 0
DB = 0
SM = 0
Delta_AUC
Cytarabine
YKR099W BAS1
ZShift = 2.72
Z lm Score = −2.26
NG = 0
DB = 0
SM = 0
Delta_L
Cytarabine
YKR099W BAS1
ZShift = 1.1
Z lm Score = −1.13
NG = 0
DB = 0
SM = 0
Delta_K
Cytarabine
YKR099W BAS1
ZShift = −1.37
Z lm Score = 0.72
NG = 0
DB = 0
SM = 0
Delta_r
Cytarabine
YKR099W BAS1
ZShift = −0.47
Z lm Score = 0.23
NG = 0
DB = 0
SM = 0
Delta_AUC
Cytarabine
YML083C
ZShift = −1.62
Z lm Score = −2.26
NG = 0
DB = 0
SM = 0
Delta_L
Cytarabine
YML083C
ZShift = −0.13
Z lm Score = 0.19
NG = 0
DB = 0
SM = 0
Delta_K
Cytarabine
YML083C
ZShift = 2.33
Z lm Score = −0.78
NG = 0
DB = 0
SM = 0
Delta_r
Cytarabine
YML083C
ZShift = 0.6
Z lm Score = −0.81
NG = 0
DB = 0
SM = 0
Delta_AUC
Cytarabine

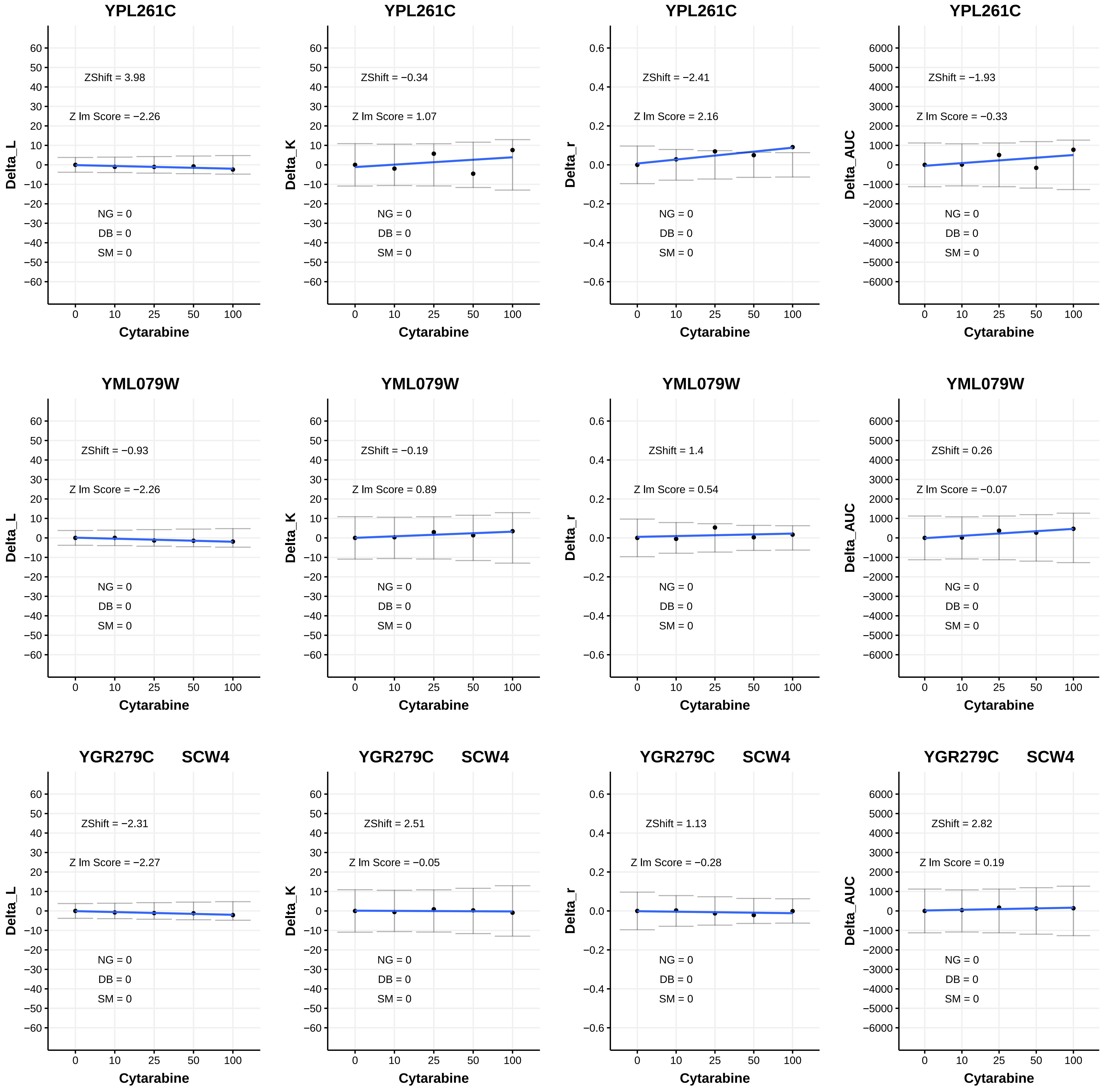
YPL261C
ZShift = 3.98
Z lm Score = −2.26
NG = 0
DB = 0
SM = 0
Delta_L
Cytarabine
YPL261C
ZShift = −0.34
Z lm Score = 1.07
NG = 0
DB = 0
SM = 0
Delta_K
Cytarabine
YPL261C
ZShift = −2.41
Z lm Score = 2.16
NG = 0
DB = 0
SM = 0
Delta_r
Cytarabine
YPL261C
ZShift = −1.93
Z lm Score = −0.33
NG = 0
DB = 0
SM = 0
Delta_AUC
Cytarabine
YML079W
ZShift = −0.93
Z lm Score = −2.26
NG = 0
DB = 0
SM = 0
Delta_L
Cytarabine
YML079W
ZShift = −0.19
Z lm Score = 0.89
NG = 0
DB = 0
SM = 0
Delta_K
Cytarabine
YML079W
ZShift = 1.4
Z lm Score = 0.54
NG = 0
DB = 0
SM = 0
Delta_r
Cytarabine
YML079W
ZShift = 0.26
Z lm Score = −0.07
NG = 0
DB = 0
SM = 0
Delta_AUC
Cytarabine
YGR279C SCW4
ZShift = −2.31
Z lm Score = −2.27
NG = 0
DB = 0
SM = 0
Delta_L
Cytarabine
YGR279C SCW4
ZShift = 2.51
Z lm Score = −0.05
NG = 0
DB = 0
SM = 0
Delta_K
Cytarabine
YGR279C SCW4
ZShift = 1.13
Z lm Score = −0.28
NG = 0
DB = 0
SM = 0
Delta_r
Cytarabine
YGR279C SCW4
ZShift = 2.82
Z lm Score = 0.19
NG = 0
DB = 0
SM = 0
Delta_AUC
Cytarabine

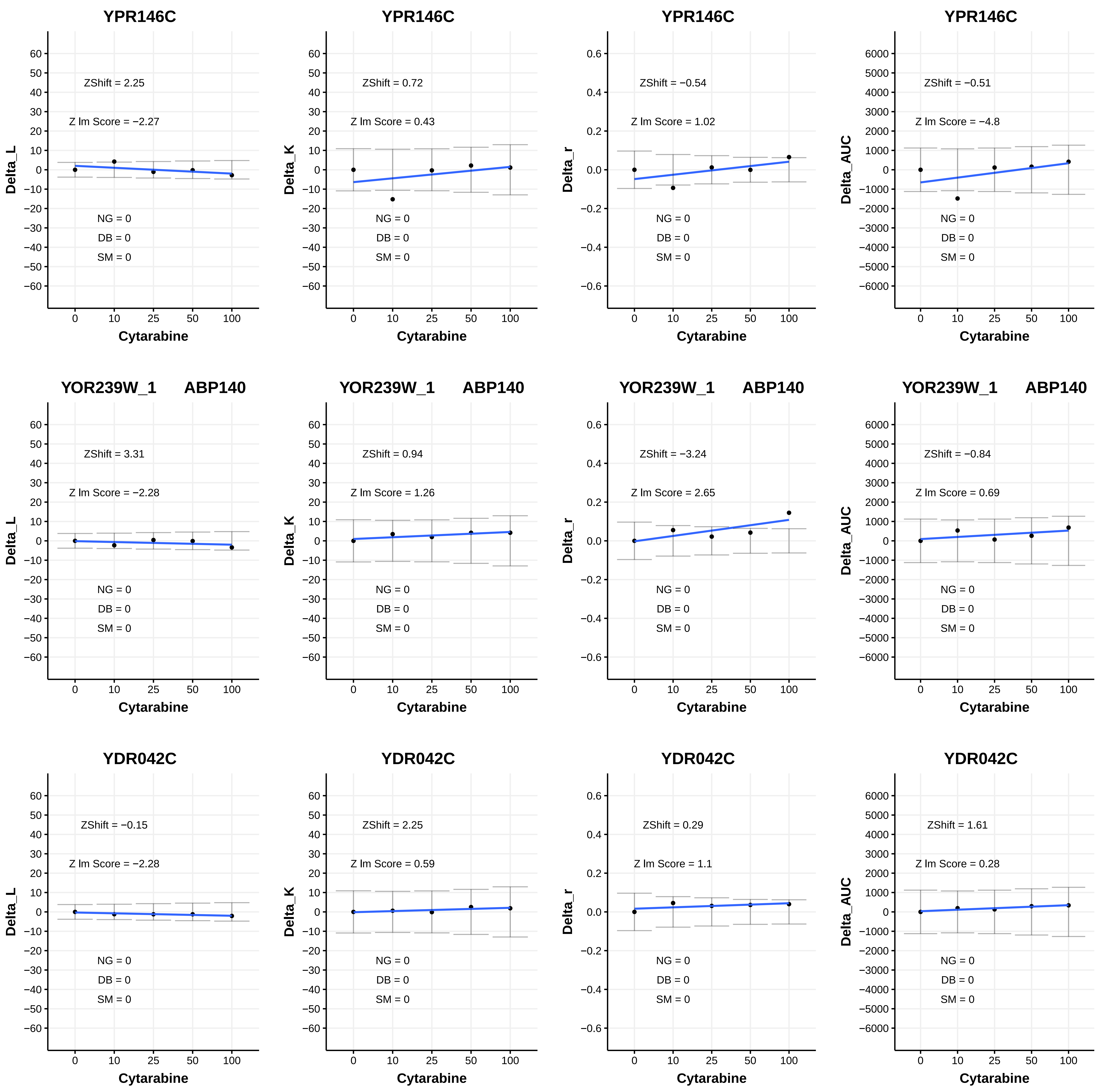

YPR146C
Delta_L
ZShift = 2.25
Z lm Score = −2.27
NG = 0
DB = 0
SM = 0
Cytarabine
YPR146C
Delta_K
ZShift = 0.72
Z lm Score = 0.43
NG = 0
DB = 0
SM = 0
Cytarabine
YPR146C
Delta_r
ZShift = −0.54
Z lm Score = 1.02
NG = 0
DB = 0
SM = 0
Cytarabine
YPR146C
Delta_AUC
ZShift = −0.51
Z lm Score = −4.8
NG = 0
DB = 0
SM = 0
Cytarabine
YOR239W_1 ABP140
Delta_L
ZShift = 3.31
Z lm Score = −2.28
NG = 0
DB = 0
SM = 0
Cytarabine
YOR239W_1 ABP140
Delta_K
ZShift = 0.94
Z lm Score = 1.26
NG = 0
DB = 0
SM = 0
Cytarabine
YOR239W_1 ABP140
Delta_r
ZShift = −3.24
Z lm Score = 2.65
NG = 0
DB = 0
SM = 0
Cytarabine
YOR239W_1 ABP140
Delta_AUC
ZShift = −0.84
Z lm Score = 0.69
NG = 0
DB = 0
SM = 0
Cytarabine
YDR042C
Delta_L
ZShift = −0.15
Z lm Score = −2.28
NG = 0
DB = 0
SM = 0
Cytarabine
YDR042C
Delta_K
ZShift = 2.25
Z lm Score = 0.59
NG = 0
DB = 0
SM = 0
Cytarabine
YDR042C
Delta_r
ZShift = 0.29
Z lm Score = 1.1
NG = 0
DB = 0
SM = 0
Cytarabine
YDR042C
Delta_AUC
ZShift = 1.61
Z lm Score = 0.28
NG = 0
DB = 0
SM = 0
Cytarabine

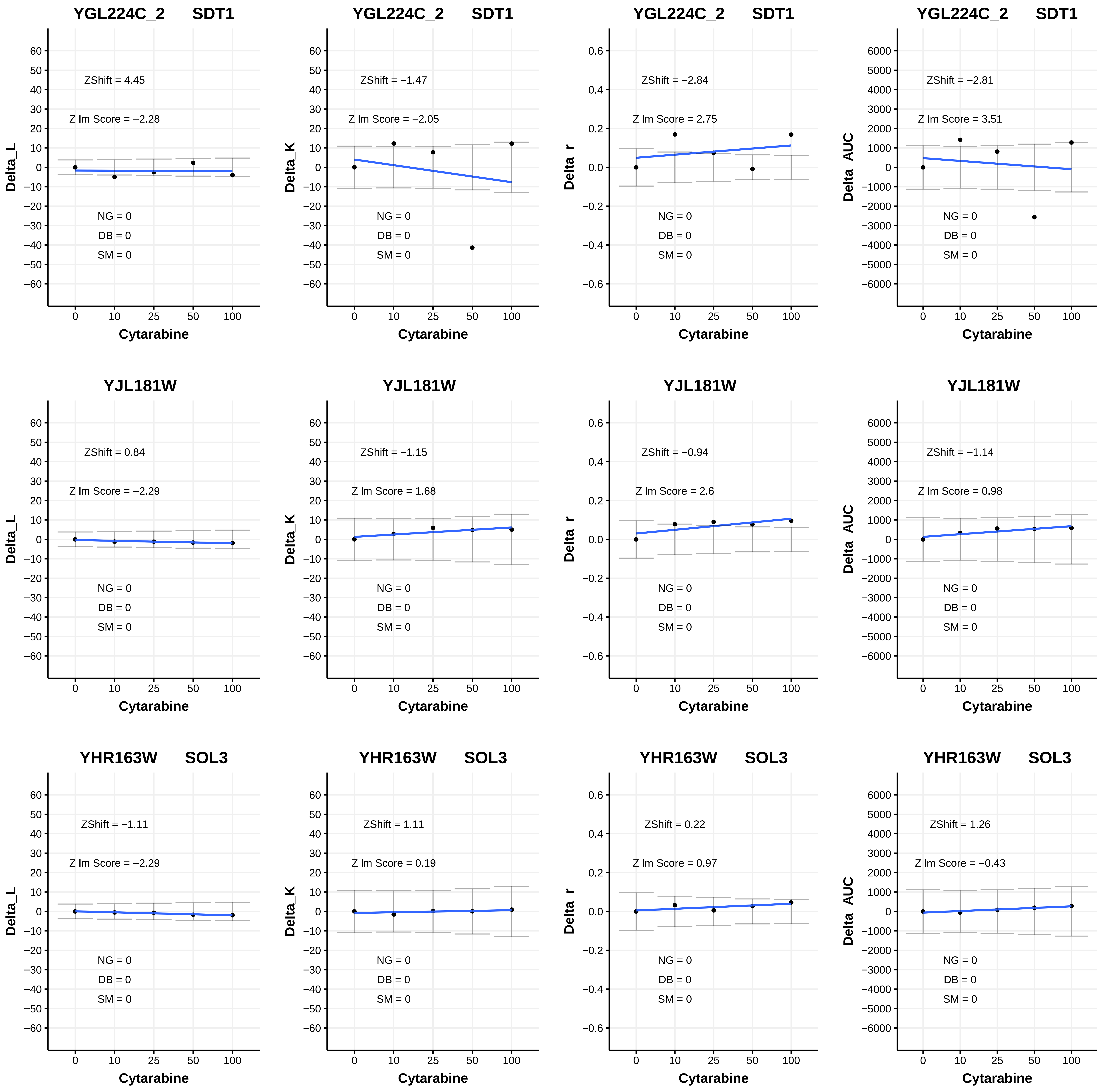
YGL224C_2 SDT1
ZShift = 4.45
Z lm Score = −2.28
NG = 0
DB = 0
SM = 0
Delta_L
Cytarabine
YGL224C_2 SDT1
ZShift = −1.47
Z lm Score = −2.05
NG = 0
DB = 0
SM = 0
Delta_K
Cytarabine
YGL224C_2 SDT1
ZShift = −2.84
Z lm Score = 2.75
NG = 0
DB = 0
SM = 0
Delta_r
Cytarabine
YGL224C_2 SDT1
ZShift = −2.81
Z lm Score = 3.51
NG = 0
DB = 0
SM = 0
Delta_AUC
Cytarabine
YJL181W
ZShift = 0.84
Z lm Score = −2.29
NG = 0
DB = 0
SM = 0
Delta_L
Cytarabine
YJL181W
ZShift = −1.15
Z lm Score = 1.68
NG = 0
DB = 0
SM = 0
Delta_K
Cytarabine
YJL181W
ZShift = −0.94
Z lm Score = 2.6
NG = 0
DB = 0
SM = 0
Delta_r
Cytarabine
YJL181W
ZShift = −1.14
Z lm Score = 0.98
NG = 0
DB = 0
SM = 0
Delta_AUC
Cytarabine
YHR163W SOL3
ZShift = −1.11
Z lm Score = −2.29
NG = 0
DB = 0
SM = 0
Delta_L
Cytarabine
YHR163W SOL3
ZShift = 1.11
Z lm Score = 0.19
NG = 0
DB = 0
SM = 0
Delta_K
Cytarabine
YHR163W SOL3
ZShift = 0.22
Z lm Score = 0.97
NG = 0
DB = 0
SM = 0
Delta_r
Cytarabine
YHR163W SOL3
ZShift = 1.26
Z lm Score = −0.43
NG = 0
DB = 0
SM = 0
Delta_AUC
Cytarabine

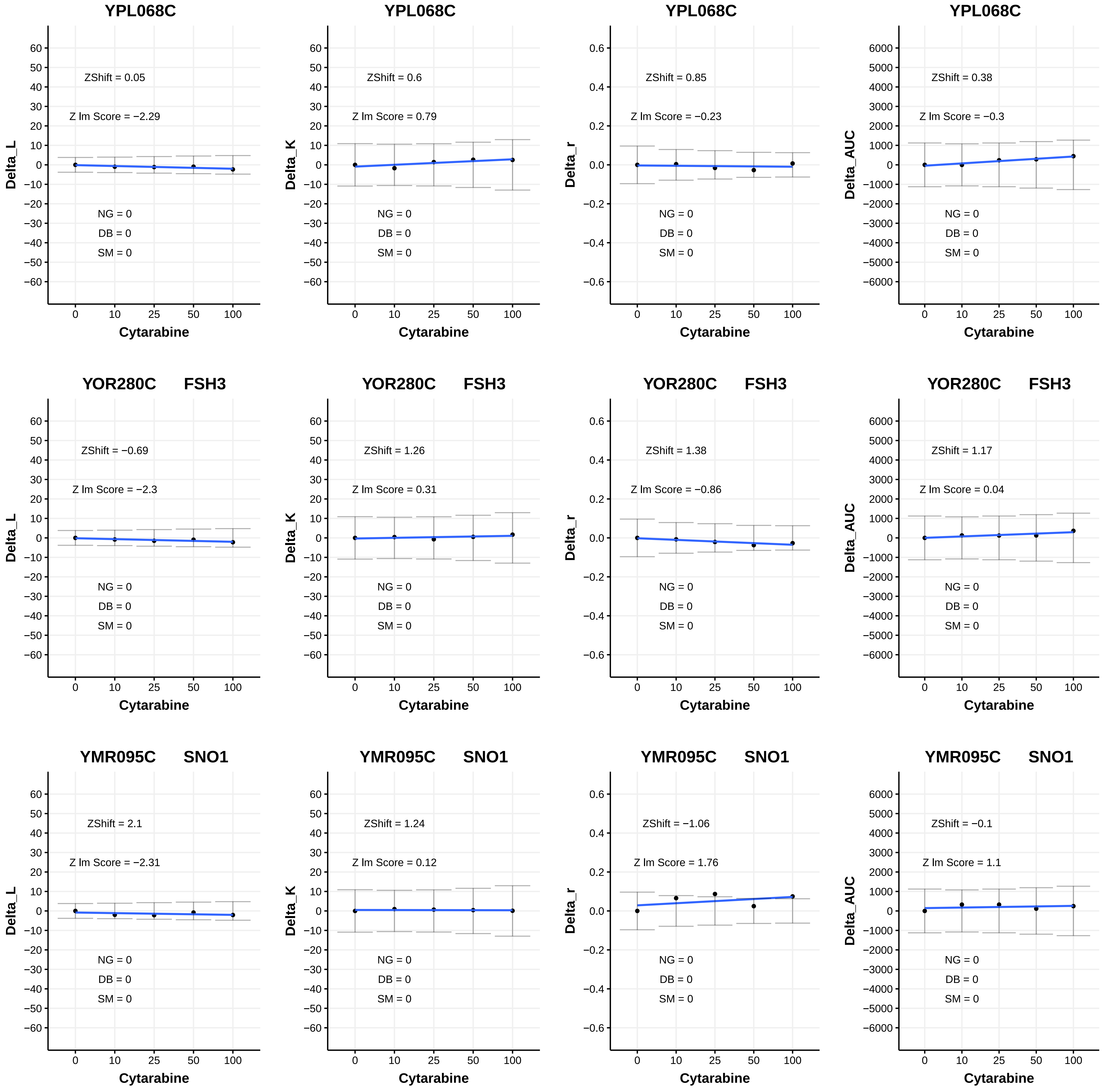
YPL068C
ZShift = 0.05
Z lm Score = −2.29
NG = 0
DB = 0
SM = 0
Delta_L
Cytarabine
YPL068C
ZShift = 0.6
Z lm Score = 0.79
NG = 0
DB = 0
SM = 0
Delta_K
Cytarabine
YPL068C
ZShift = 0.85
Z lm Score = −0.23
NG = 0
DB = 0
SM = 0
Delta_r
Cytarabine
YPL068C
ZShift = 0.38
Z lm Score = −0.3
NG = 0
DB = 0
SM = 0
Delta_AUC
Cytarabine
YOR280C FSH3
ZShift = −0.69
Z lm Score = −2.3
NG = 0
DB = 0
SM = 0
Delta_L
Cytarabine
YOR280C FSH3
ZShift = 1.26
Z lm Score = 0.31
NG = 0
DB = 0
SM = 0
Delta_K
Cytarabine
YOR280C FSH3
ZShift = 1.38
Z lm Score = −0.86
NG = 0
DB = 0
SM = 0
Delta_r
Cytarabine
YOR280C FSH3
ZShift = 1.17
Z lm Score = 0.04
NG = 0
DB = 0
SM = 0
Delta_AUC
Cytarabine
YMR095C SNO1
ZShift = 2.1
Z lm Score = −2.31
NG = 0
DB = 0
SM = 0
Delta_L
Cytarabine
YMR095C SNO1
ZShift = 1.24
Z lm Score = 0.12
NG = 0
DB = 0
SM = 0
Delta_K
Cytarabine
YMR095C SNO1
ZShift = −1.06
Z lm Score = 1.76
NG = 0
DB = 0
SM = 0
Delta_r
Cytarabine
YMR095C SNO1
ZShift = −0.1
Z lm Score = 1.1
NG = 0
DB = 0
SM = 0
Delta_AUC
Cytarabine

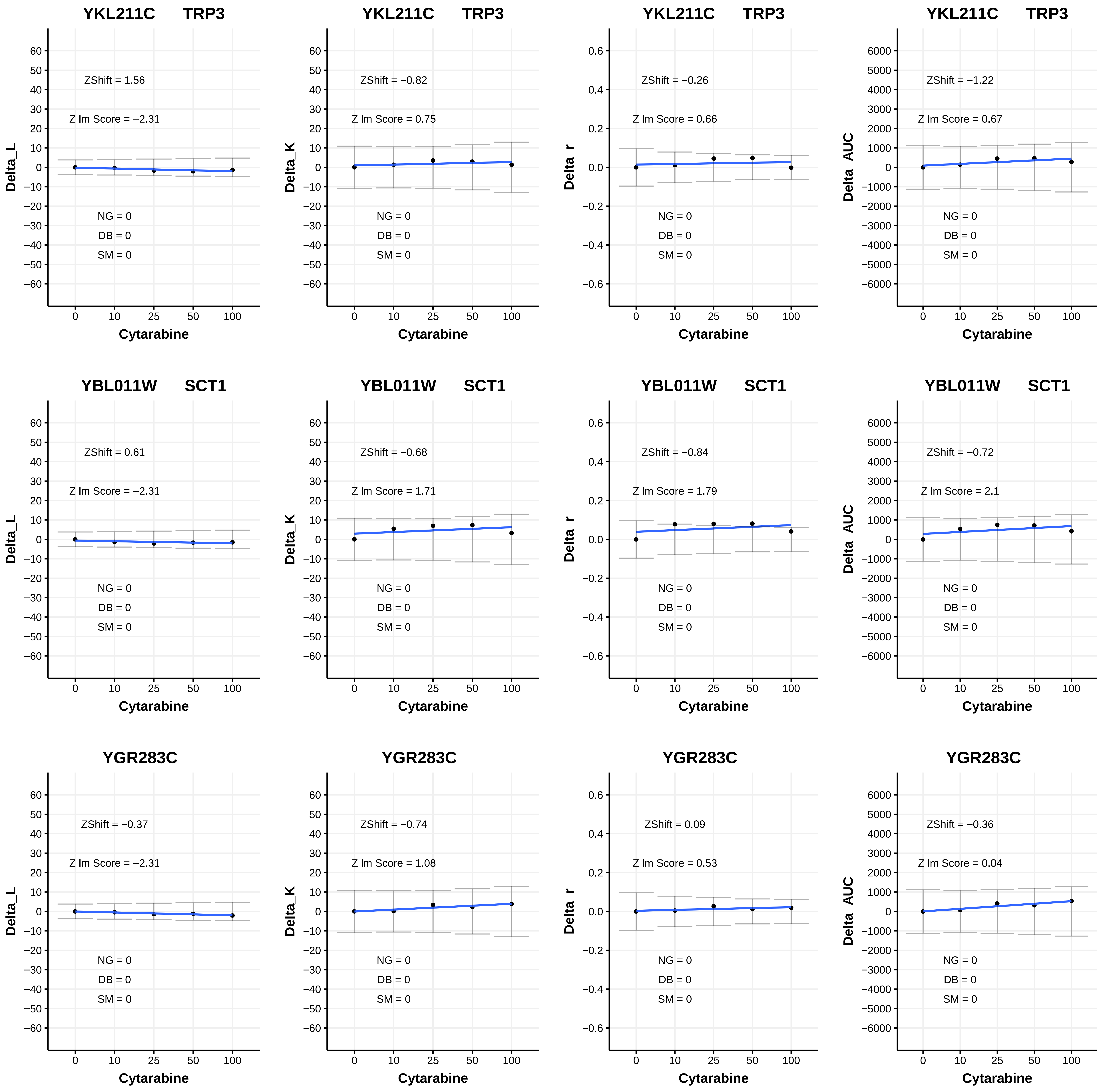

YKL211C TRP3
ZShift = 1.56
Z lm Score = −2.31
NG = 0
DB = 0
SM = 0
Delta_L
Cytarabine
YKL211C TRP3
ZShift = −0.82
Z lm Score = 0.75
NG = 0
DB = 0
SM = 0
Delta_K
Cytarabine
YKL211C TRP3
ZShift = −0.26
Z lm Score = 0.66
NG = 0
DB = 0
SM = 0
Delta_r
Cytarabine
YKL211C TRP3
ZShift = −1.22
Z lm Score = 0.67
NG = 0
DB = 0
SM = 0
Delta_AUC
Cytarabine
YBL011W SCT1
ZShift = 0.61
Z lm Score = −2.31
NG = 0
DB = 0
SM = 0
Delta_L
Cytarabine
YBL011W SCT1
ZShift = −0.68
Z lm Score = 1.71
NG = 0
DB = 0
SM = 0
Delta_K
Cytarabine
YBL011W SCT1
ZShift = −0.84
Z lm Score = 1.79
NG = 0
DB = 0
SM = 0
Delta_r
Cytarabine
YBL011W SCT1
ZShift = −0.72
Z lm Score = 2.1
NG = 0
DB = 0
SM = 0
Delta_AUC
Cytarabine
YGR283C
ZShift = −0.37
Z lm Score = −2.31
NG = 0
DB = 0
SM = 0
Delta_L
Cytarabine
YGR283C
ZShift = −0.74
Z lm Score = 1.08
NG = 0
DB = 0
SM = 0
Delta_K
Cytarabine
YGR283C
ZShift = 0.09
Z lm Score = 0.53
NG = 0
DB = 0
SM = 0
Delta_r
Cytarabine
YGR283C
ZShift = −0.36
Z lm Score = 0.04
NG = 0
DB = 0
SM = 0
Delta_AUC
Cytarabine

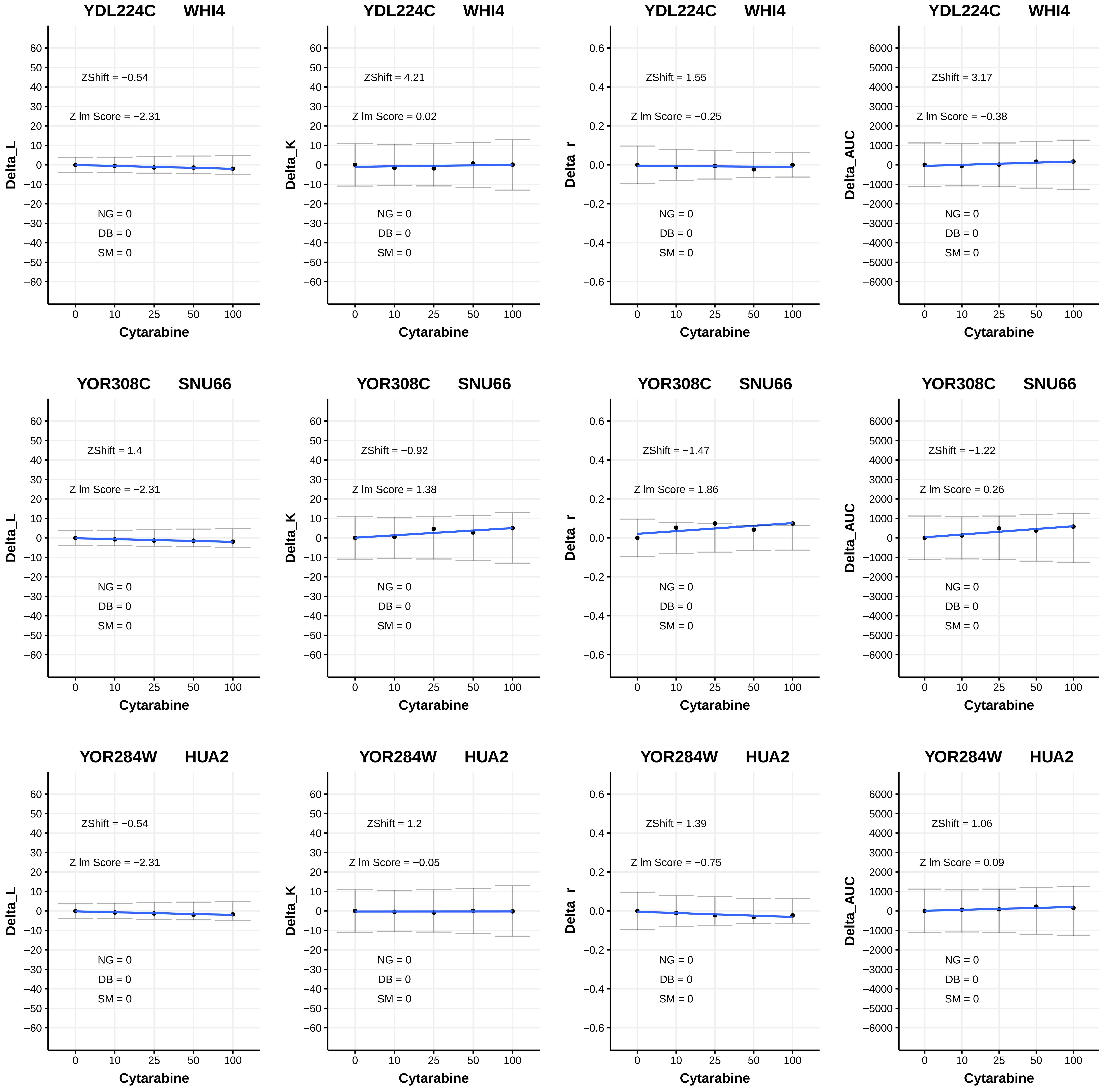
YDL224C WHI4
ZShift = −0.54
Z lm Score = −2.31
NG = 0
DB = 0
SM = 0
Delta_L
Cytarabine
YDL224C WHI4
ZShift = 4.21
Z lm Score = 0.02
NG = 0
DB = 0
SM = 0
Delta_K
Cytarabine
YDL224C WHI4
ZShift = 1.55
Z lm Score = −0.25
NG = 0
DB = 0
SM = 0
Delta_r
Cytarabine
YDL224C WHI4
ZShift = 3.17
Z lm Score = −0.38
NG = 0
DB = 0
SM = 0
Delta_AUC
Cytarabine
YOR308C SNU66
ZShift = 1.4
Z lm Score = −2.31
NG = 0
DB = 0
SM = 0
Delta_L
Cytarabine
YOR308C SNU66
ZShift = −0.92
Z lm Score = 1.38
NG = 0
DB = 0
SM = 0
Delta_K
Cytarabine
YOR308C SNU66
ZShift = −1.47
Z lm Score = 1.86
NG = 0
DB = 0
SM = 0
Delta_r
Cytarabine
YOR308C SNU66
ZShift = −1.22
Z lm Score = 0.26
NG = 0
DB = 0
SM = 0
Delta_AUC
Cytarabine
YOR284W HUA2
ZShift = −0.54
Z lm Score = −2.31
NG = 0
DB = 0
SM = 0
Delta_L
Cytarabine
YOR284W HUA2
ZShift = 1.2
Z lm Score = −0.05
NG = 0
DB = 0
SM = 0
Delta_K
Cytarabine
YOR284W HUA2
ZShift = 1.39
Z lm Score = −0.75
NG = 0
DB = 0
SM = 0
Delta_r
Cytarabine
YOR284W HUA2
ZShift = 1.06
Z lm Score = 0.09
NG = 0
DB = 0
SM = 0
Delta_AUC
Cytarabine

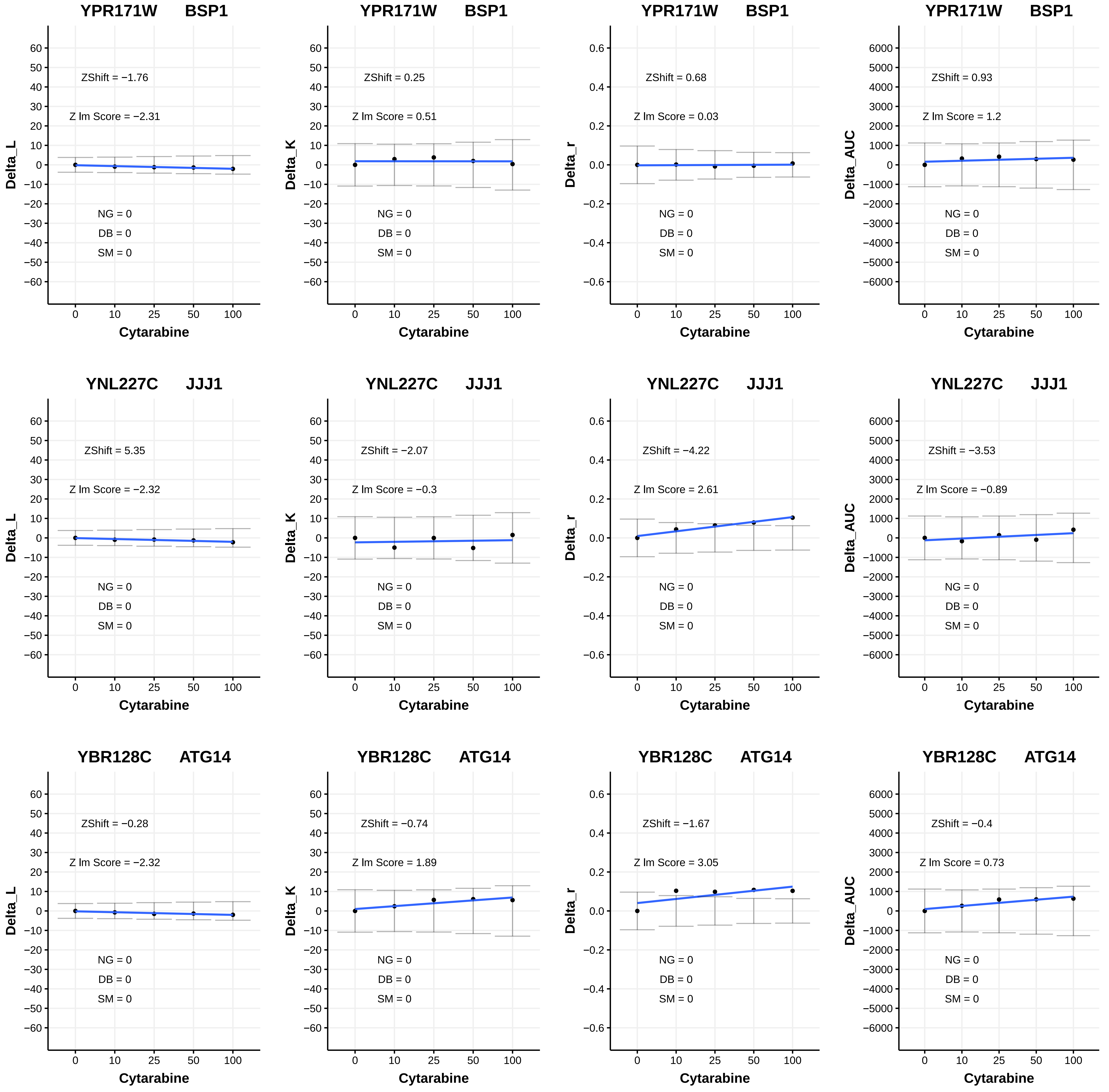
YPR171W BSP1
ZShift = −1.76
Z lm Score = −2.31
NG = 0
DB = 0
SM = 0
Delta_L
Cytarabine
YPR171W BSP1
ZShift = 0.25
Z lm Score = 0.51
NG = 0
DB = 0
SM = 0
Delta_K
Cytarabine
YPR171W BSP1
ZShift = 0.68
Z lm Score = 0.03
NG = 0
DB = 0
SM = 0
Delta_r
Cytarabine
YPR171W BSP1
ZShift = 0.93
Z lm Score = 1.2
NG = 0
DB = 0
SM = 0
Delta_AUC
Cytarabine
YNL227C JJJ1
ZShift = 5.35
Z lm Score = −2.32
NG = 0
DB = 0
SM = 0
Delta_L
Cytarabine
YNL227C JJJ1
ZShift = −2.07
Z lm Score = −0.3
NG = 0
DB = 0
SM = 0
Delta_K
Cytarabine
YNL227C JJJ1
ZShift = −4.22
Z lm Score = 2.61
NG = 0
DB = 0
SM = 0
Delta_r
Cytarabine
YNL227C JJJ1
ZShift = −3.53
Z lm Score = −0.89
NG = 0
DB = 0
SM = 0
Delta_AUC
Cytarabine
YBR128C ATG14
ZShift = −0.28
Z lm Score = −2.32
NG = 0
DB = 0
SM = 0
Delta_L
Cytarabine
YBR128C ATG14
ZShift = −0.74
Z lm Score = 1.89
NG = 0
DB = 0
SM = 0
Delta_K
Cytarabine
YBR128C ATG14
ZShift = −1.67
Z lm Score = 3.05
NG = 0
DB = 0
SM = 0
Delta_r
Cytarabine
YBR128C ATG14
ZShift = −0.4
Z lm Score = 0.73
NG = 0
DB = 0
SM = 0
Delta_AUC
Cytarabine

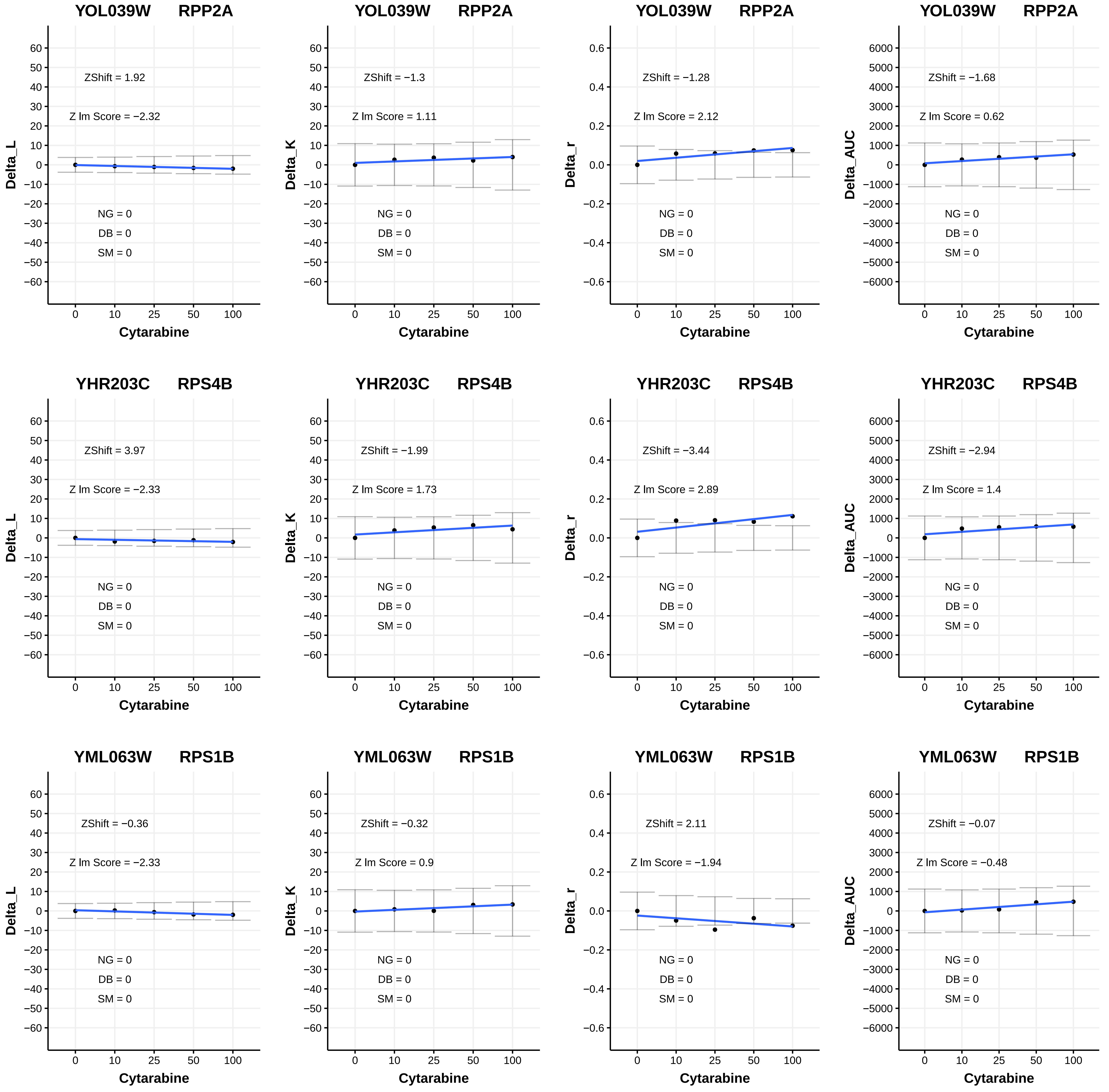
YOL039W RPP2A
ZShift = 1.92
Z lm Score = −2.32
NG = 0
DB = 0
SM = 0
Delta_L
Cytarabine
YOL039W RPP2A
ZShift = −1.3
Z lm Score = 1.11
NG = 0
DB = 0
SM = 0
Delta_K
Cytarabine
YOL039W RPP2A
ZShift = −1.28
Z lm Score = 2.12
NG = 0
DB = 0
SM = 0
Delta_r
Cytarabine
YOL039W RPP2A
ZShift = −1.68
Z lm Score = 0.62
NG = 0
DB = 0
SM = 0
Delta_AUC
Cytarabine
YHR203C RPS4B
ZShift = 3.97
Z lm Score = −2.33
NG = 0
DB = 0
SM = 0
Delta_L
Cytarabine
YHR203C RPS4B
ZShift = −1.99
Z lm Score = 1.73
NG = 0
DB = 0
SM = 0
Delta_K
Cytarabine
YHR203C RPS4B
ZShift = −3.44
Z lm Score = 2.89
NG = 0
DB = 0
SM = 0
Delta_r
Cytarabine
YHR203C RPS4B
ZShift = −2.94
Z lm Score = 1.4
NG = 0
DB = 0
SM = 0
Delta_AUC
Cytarabine
YML063W RPS1B
ZShift = −0.36
Z lm Score = −2.33
NG = 0
DB = 0
SM = 0
Delta_L
Cytarabine
YML063W RPS1B
ZShift = −0.32
Z lm Score = 0.9
NG = 0
DB = 0
SM = 0
Delta_K
Cytarabine
YML063W RPS1B
ZShift = 2.11
Z lm Score = −1.94
NG = 0
DB = 0
SM = 0
Delta_r
Cytarabine
YML063W RPS1B
ZShift = −0.07
Z lm Score = −0.48
NG = 0
DB = 0
SM = 0
Delta_AUC
Cytarabine

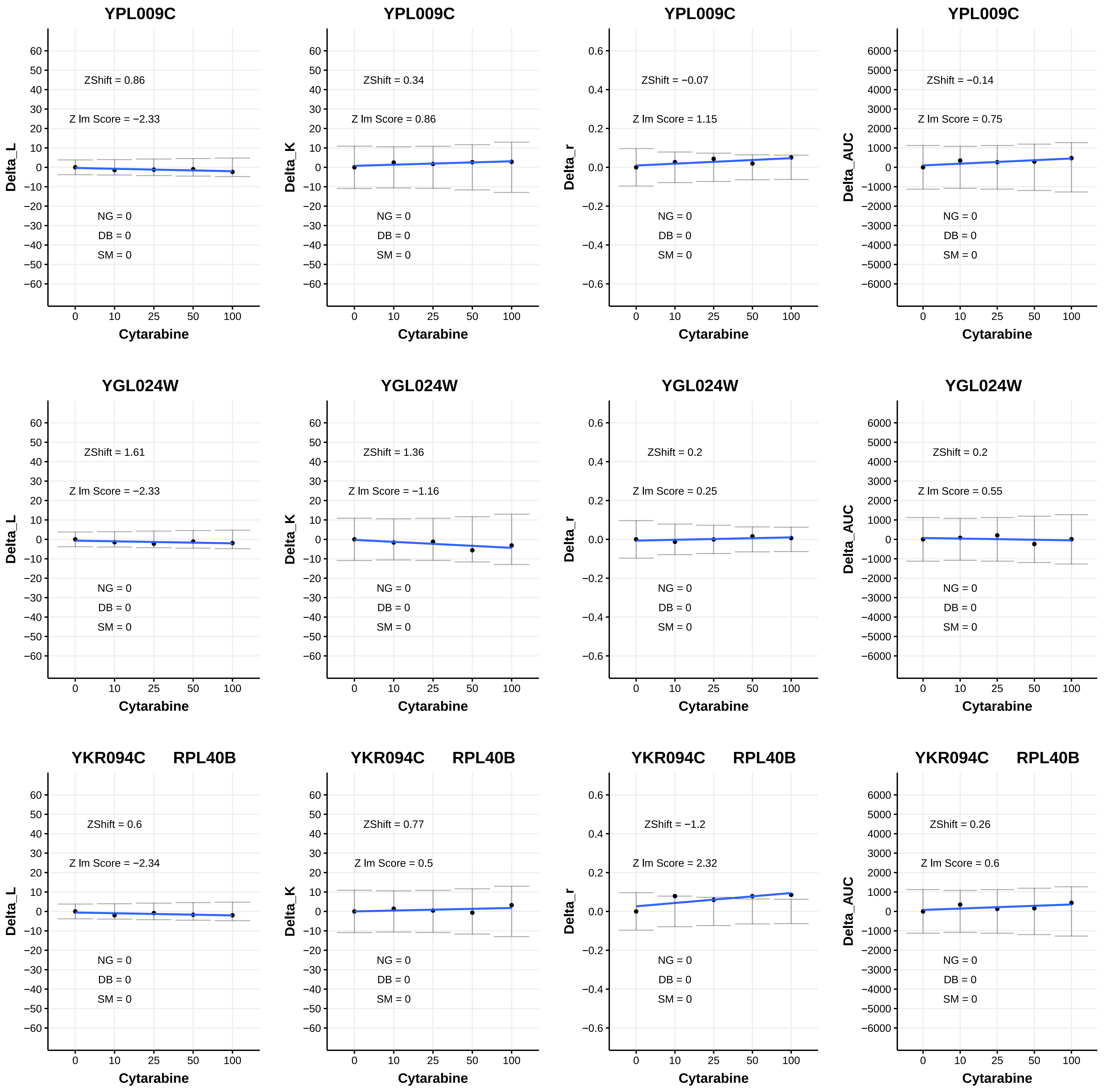

YPL009C
ZShift = 0.86
Z lm Score = −2.33
NG = 0
DB = 0
SM = 0
Delta_L
Cytarabine
YPL009C
ZShift = 0.34
Z lm Score = 0.86
NG = 0
DB = 0
SM = 0
Delta_K
Cytarabine
YPL009C
ZShift = −0.07
Z lm Score = 1.15
NG = 0
DB = 0
SM = 0
Delta_r
Cytarabine
YPL009C
ZShift = −0.14
Z lm Score = 0.75
NG = 0
DB = 0
SM = 0
Delta_AUC
Cytarabine
YGL024W
ZShift = 1.61
Z lm Score = −2.33
NG = 0
DB = 0
SM = 0
Delta_L
Cytarabine
YGL024W
ZShift = 1.36
Z lm Score = −1.16
NG = 0
DB = 0
SM = 0
Delta_K
Cytarabine
YGL024W
ZShift = 0.2
Z lm Score = 0.25
NG = 0
DB = 0
SM = 0
Delta_r
Cytarabine
YGL024W
ZShift = 0.2
Z lm Score = 0.55
NG = 0
DB = 0
SM = 0
Delta_AUC
Cytarabine
YKR094C RPL40B
ZShift = 0.6
Z lm Score = −2.34
NG = 0
DB = 0
SM = 0
Delta_L
Cytarabine
YKR094C RPL40B
ZShift = 0.77
Z lm Score = 0.5
NG = 0
DB = 0
SM = 0
Delta_K
Cytarabine
YKR094C RPL40B
ZShift = −1.2
Z lm Score = 2.32
NG = 0
DB = 0
SM = 0
Delta_r
Cytarabine
YKR094C RPL40B
ZShift = 0.26
Z lm Score = 0.6
NG = 0
DB = 0
SM = 0
Delta_AUC
Cytarabine

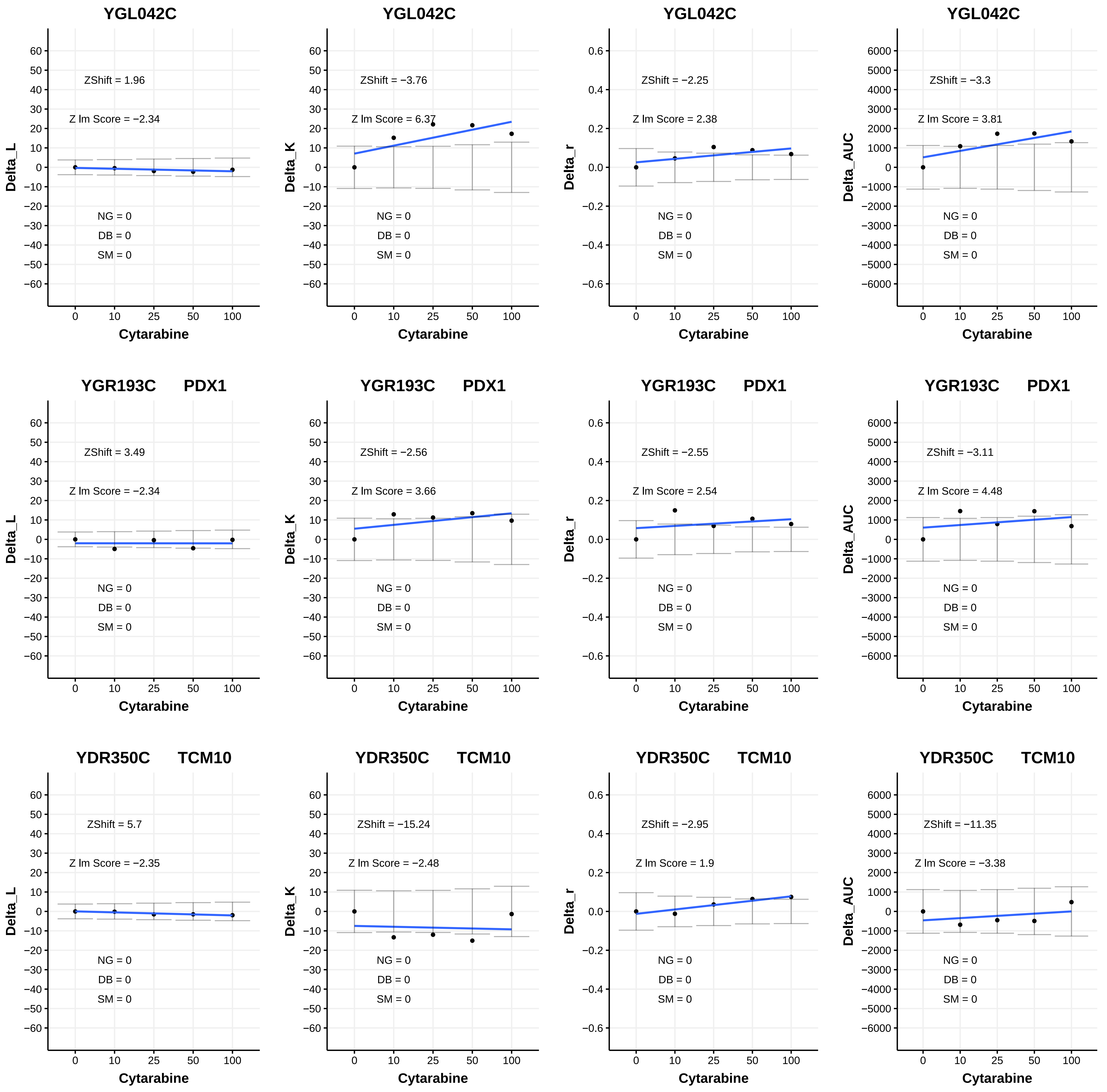
YGL042C
ZShift = 1.96
Z lm Score = −2.34
NG = 0
DB = 0
SM = 0
Delta_L
Cytarabine
YGL042C
ZShift = −3.76
Z lm Score = 6.37
NG = 0
DB = 0
SM = 0
Delta_K
Cytarabine
YGL042C
ZShift = −2.25
Z lm Score = 2.38
NG = 0
DB = 0
SM = 0
Delta_r
Cytarabine
YGL042C
ZShift = −3.3
Z lm Score = 3.81
NG = 0
DB = 0
SM = 0
Delta_AUC
Cytarabine
YGR193C PDX1
ZShift = 3.49
Z lm Score = −2.34
NG = 0
DB = 0
SM = 0
Delta_L
Cytarabine
YGR193C PDX1
ZShift = −2.56
Z lm Score = 3.66
NG = 0
DB = 0
SM = 0
Delta_K
Cytarabine
YGR193C PDX1
ZShift = −2.55
Z lm Score = 2.54
NG = 0
DB = 0
SM = 0
Delta_r
Cytarabine
YGR193C PDX1
ZShift = −3.11
Z lm Score = 4.48
NG = 0
DB = 0
SM = 0
Delta_AUC
Cytarabine
YDR350C TCM10
ZShift = 5.7
Z lm Score = −2.35
NG = 0
DB = 0
SM = 0
Delta_L
Cytarabine
YDR350C TCM10
ZShift = −15.24
Z lm Score = −2.48
NG = 0
DB = 0
SM = 0
Delta_K
Cytarabine
YDR350C TCM10
ZShift = −2.95
Z lm Score = 1.9
NG = 0
DB = 0
SM = 0
Delta_r
Cytarabine
YDR350C TCM10
ZShift = −11.35
Z lm Score = −3.38
NG = 0
DB = 0
SM = 0
Delta_AUC
Cytarabine

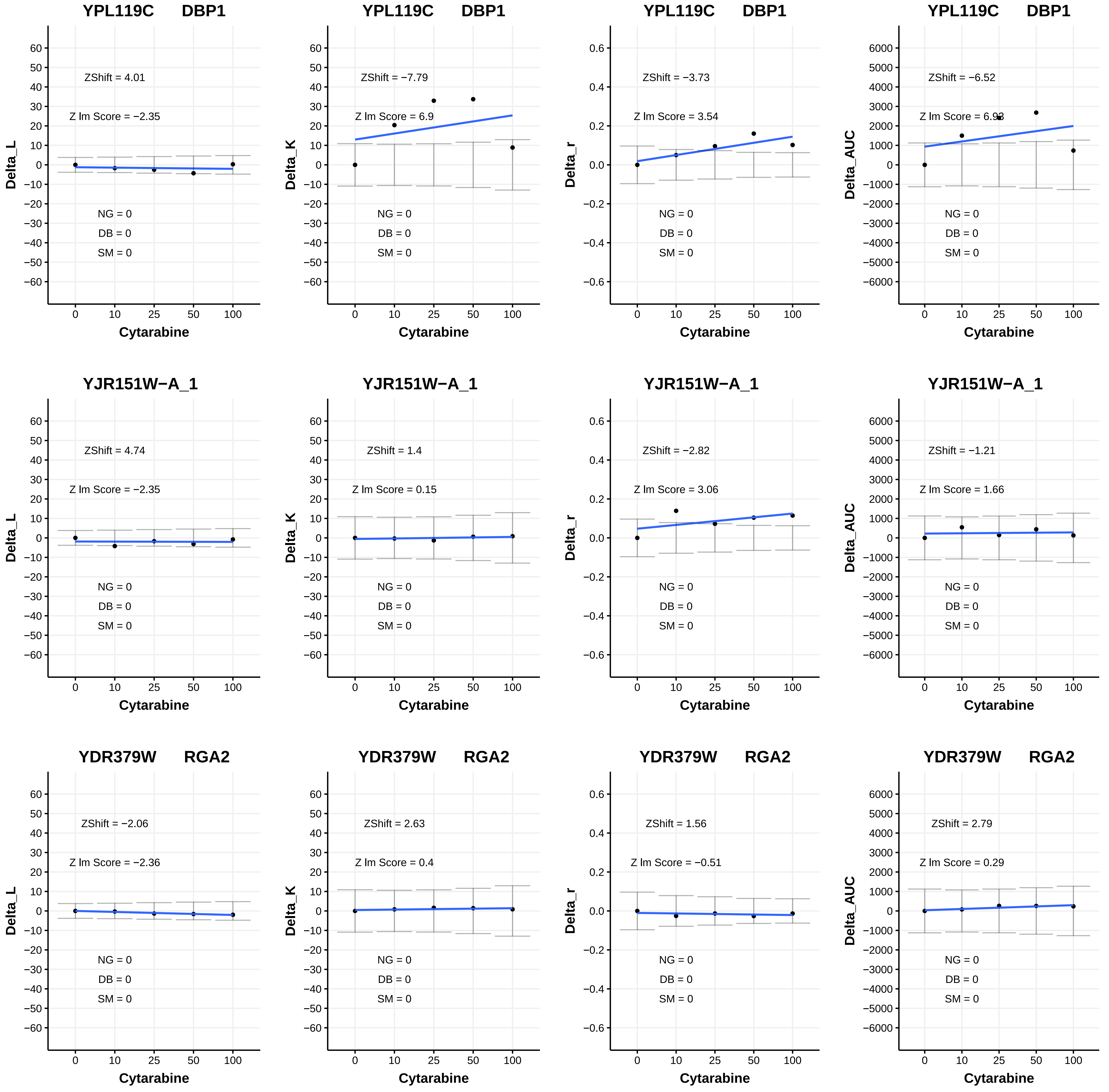

YPL119C DBP1
Delta_L
ZShift = 4.01
Z lm Score = −2.35
NG = 0
DB = 0
SM = 0
Cytarabine
YPL119C DBP1
Delta_K
ZShift = −7.79
Z lm Score = 6.9
NG = 0
DB = 0
SM = 0
Cytarabine
YPL119C DBP1
Delta_r
ZShift = −3.73
Z lm Score = 3.54
NG = 0
DB = 0
SM = 0
Cytarabine
YPL119C DBP1
Delta_AUC
ZShift = −6.52
Z lm Score = 6.93
NG = 0
DB = 0
SM = 0
Cytarabine
YJR151W−A_1
Delta_L
ZShift = 4.74
Z lm Score = −2.35
NG = 0
DB = 0
SM = 0
Cytarabine
YJR151W−A_1
Delta_K
ZShift = 1.4
Z lm Score = 0.15
NG = 0
DB = 0
SM = 0
Cytarabine
YJR151W−A_1
Delta_r
ZShift = −2.82
Z lm Score = 3.06
NG = 0
DB = 0
SM = 0
Cytarabine
YJR151W−A_1
Delta_AUC
ZShift = −1.21
Z lm Score = 1.66
NG = 0
DB = 0
SM = 0
Cytarabine
YDR379W RGA2
Delta_L
ZShift = −2.06
Z lm Score = −2.36
NG = 0
DB = 0
SM = 0
Cytarabine
YDR379W RGA2
Delta_K
ZShift = 2.63
Z lm Score = 0.4
NG = 0
DB = 0
SM = 0
Cytarabine
YDR379W RGA2
Delta_r
ZShift = 1.56
Z lm Score = −0.51
NG = 0
DB = 0
SM = 0
Cytarabine
YDR379W RGA2
Delta_AUC
ZShift = 2.79
Z lm Score = 0.29
NG = 0
DB = 0
SM = 0
Cytarabine

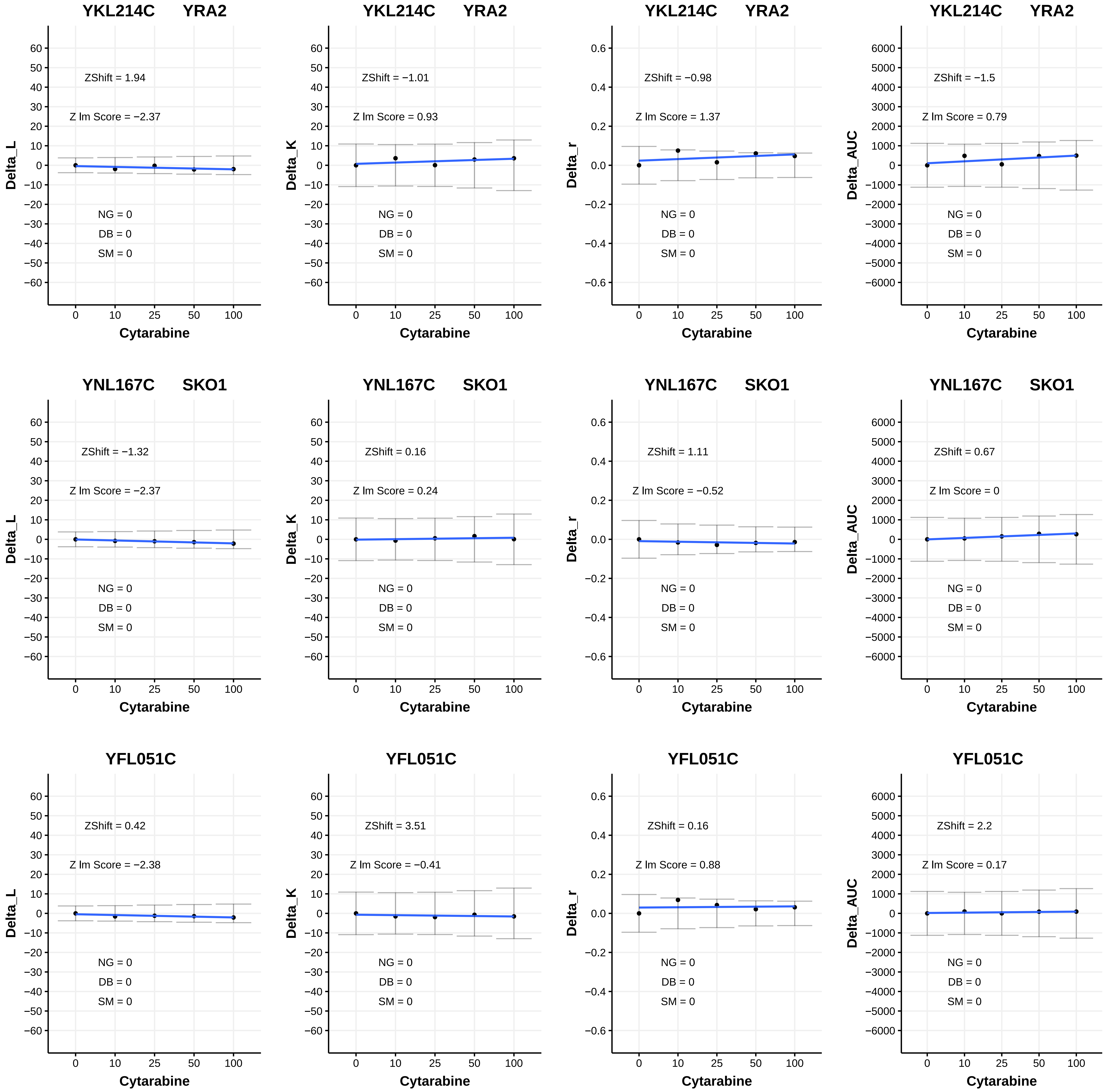

YKL214C YRA2
ZShift = 1.94
Z lm Score = −2.37
NG = 0
DB = 0
SM = 0
Delta_L
Cytarabine
ZShift = −1.01
Z lm Score = 0.93
Delta_K
ZShift = −0.98
Z lm Score = 1.37
Delta_r
ZShift = −1.5
Z lm Score = 0.79
Delta_AUC
YNL167C SKO1
ZShift = −1.32
Z lm Score = −2.37
ZShift = 0.16
Z lm Score = 0.24
ZShift = 1.11
Z lm Score = −0.52
ZShift = 0.67
Z lm Score = 0
YFL051C
ZShift = 0.42
Z lm Score = −2.38
ZShift = 3.51
Z lm Score = −0.41
ZShift = 0.16
Z lm Score = 0.88
ZShift = 2.2
Z lm Score = 0.17
0
10
25
50
100

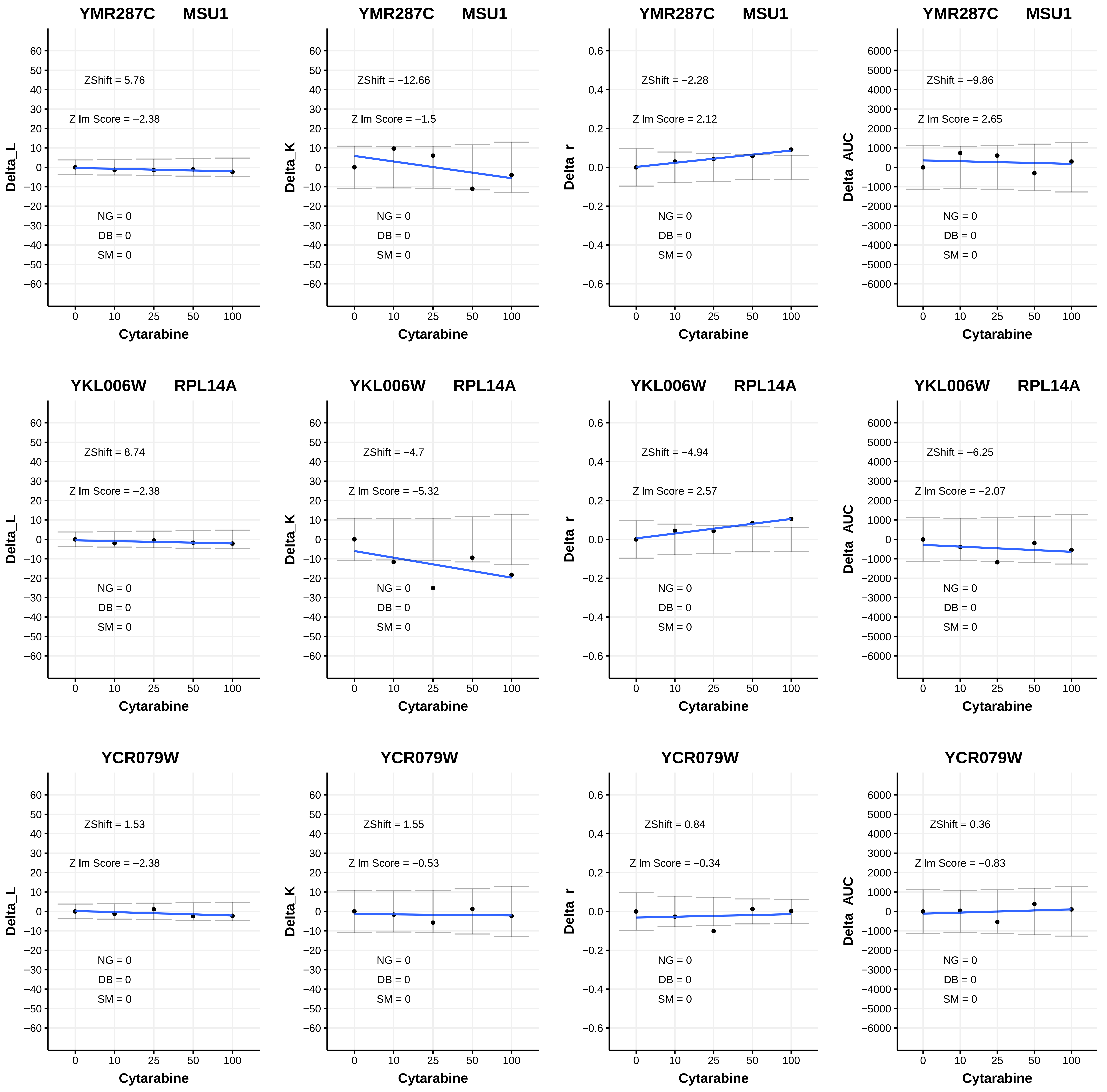
YMR287C MSU1
ZShift = 5.76
Z lm Score = −2.38
NG = 0
DB = 0
SM = 0
Delta_L
Cytarabine
YMR287C MSU1
ZShift = −12.66
Z lm Score = −1.5
NG = 0
DB = 0
SM = 0
Delta_K
Cytarabine
YMR287C MSU1
ZShift = −2.28
Z lm Score = 2.12
NG = 0
DB = 0
SM = 0
Delta_r
Cytarabine
YMR287C MSU1
ZShift = −9.86
Z lm Score = 2.65
NG = 0
DB = 0
SM = 0
Delta_AUC
Cytarabine
YKL006W RPL14A
ZShift = 8.74
Z lm Score = −2.38
NG = 0
DB = 0
SM = 0
Delta_L
Cytarabine
YKL006W RPL14A
ZShift = −4.7
Z lm Score = −5.32
NG = 0
DB = 0
SM = 0
Delta_K
Cytarabine
YKL006W RPL14A
ZShift = −4.94
Z lm Score = 2.57
NG = 0
DB = 0
SM = 0
Delta_r
Cytarabine
YKL006W RPL14A
ZShift = −6.25
Z lm Score = −2.07
NG = 0
DB = 0
SM = 0
Delta_AUC
Cytarabine
YCR079W
ZShift = 1.53
Z lm Score = −2.38
NG = 0
DB = 0
SM = 0
Delta_L
Cytarabine
YCR079W
ZShift = 1.55
Z lm Score = −0.53
NG = 0
DB = 0
SM = 0
Delta_K
Cytarabine
YCR079W
ZShift = 0.84
Z lm Score = −0.34
NG = 0
DB = 0
SM = 0
Delta_r
Cytarabine
YCR079W
ZShift = 0.36
Z lm Score = −0.83
NG = 0
DB = 0
SM = 0
Delta_AUC
Cytarabine

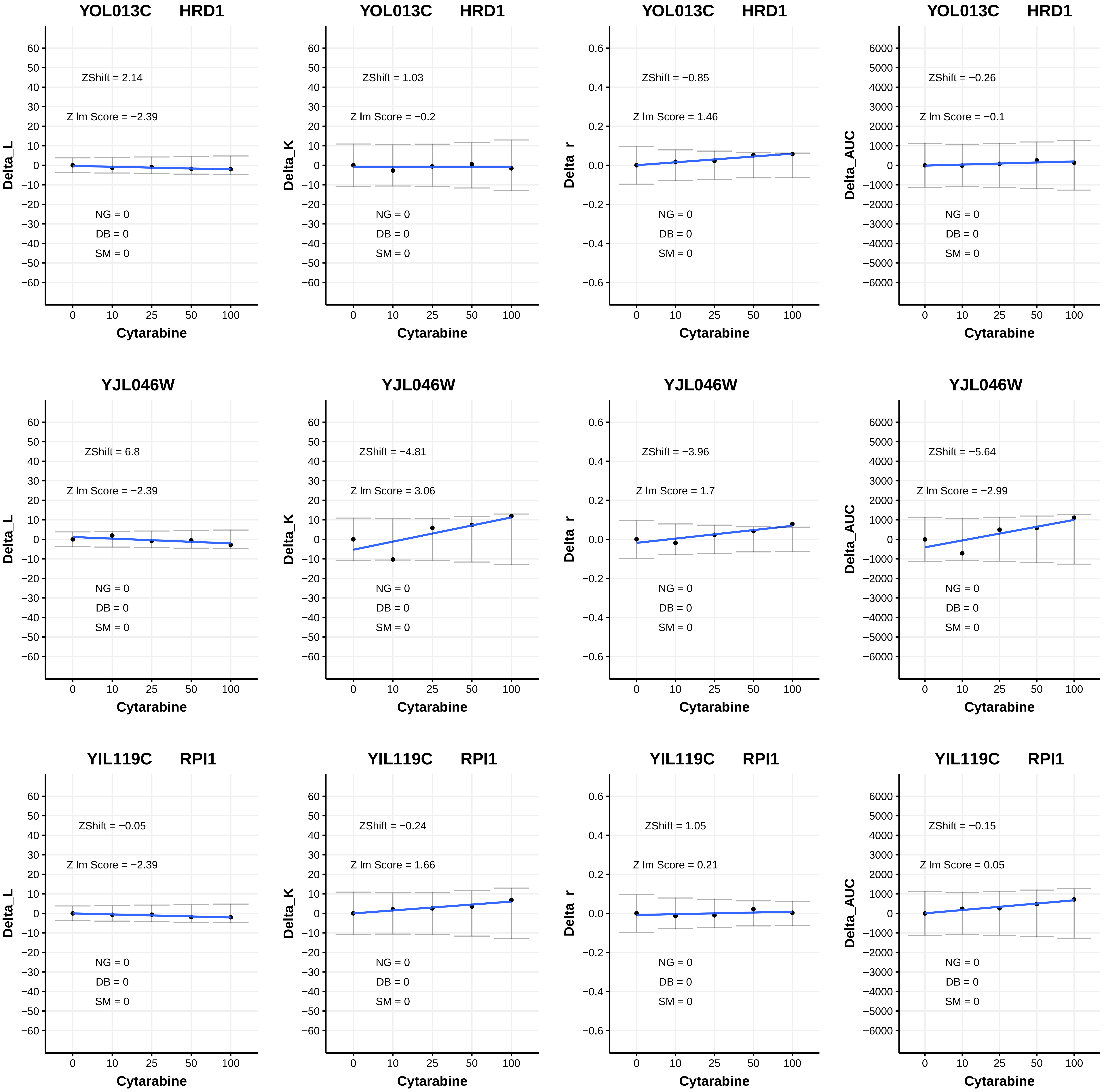

YOL013C HRD1
ZShift = 2.14
Z lm Score = −2.39
NG = 0
DB = 0
SM = 0
Delta_L
Cytarabine
YOL013C HRD1
ZShift = 1.03
Z lm Score = −0.2
NG = 0
DB = 0
SM = 0
Delta_K
Cytarabine
YOL013C HRD1
ZShift = −0.85
Z lm Score = 1.46
NG = 0
DB = 0
SM = 0
Delta_r
Cytarabine
YOL013C HRD1
ZShift = −0.26
Z lm Score = −0.1
NG = 0
DB = 0
SM = 0
Delta_AUC
Cytarabine
YJL046W
ZShift = 6.8
Z lm Score = −2.39
NG = 0
DB = 0
SM = 0
Delta_L
Cytarabine
YJL046W
ZShift = −4.81
Z lm Score = 3.06
NG = 0
DB = 0
SM = 0
Delta_K
Cytarabine
YJL046W
ZShift = −3.96
Z lm Score = 1.7
NG = 0
DB = 0
SM = 0
Delta_r
Cytarabine
YJL046W
ZShift = −5.64
Z lm Score = −2.99
NG = 0
DB = 0
SM = 0
Delta_AUC
Cytarabine
YIL119C RPI1
ZShift = −0.05
Z lm Score = −2.39
NG = 0
DB = 0
SM = 0
Delta_L
Cytarabine
YIL119C RPI1
ZShift = −0.24
Z lm Score = 1.66
NG = 0
DB = 0
SM = 0
Delta_K
Cytarabine
YIL119C RPI1
ZShift = 1.05
Z lm Score = 0.21
NG = 0
DB = 0
SM = 0
Delta_r
Cytarabine
YIL119C RPI1
ZShift = −0.15
Z lm Score = 0.05
NG = 0
DB = 0
SM = 0
Delta_AUC
Cytarabine

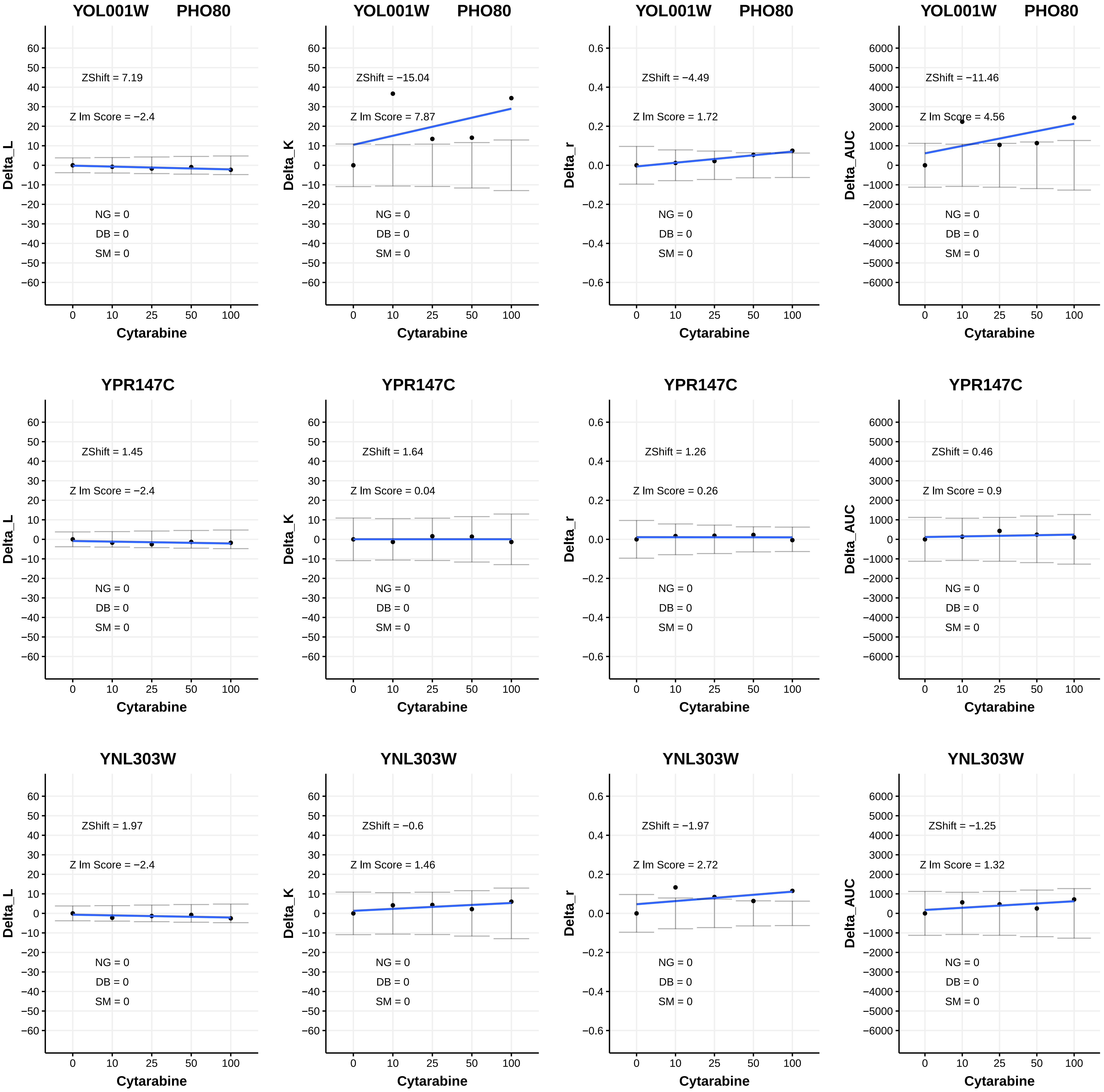
YOL001W PHO80
ZShift = 7.19
Z lm Score = −2.4
NG = 0
DB = 0
SM = 0
Delta_L
Cytarabine
YOL001W PHO80
ZShift = −15.04
Z lm Score = 7.87
NG = 0
DB = 0
SM = 0
Delta_K
Cytarabine
YOL001W PHO80
ZShift = −4.49
Z lm Score = 1.72
NG = 0
DB = 0
SM = 0
Delta_r
Cytarabine
YOL001W PHO80
ZShift = −11.46
Z lm Score = 4.56
NG = 0
DB = 0
SM = 0
Delta_AUC
Cytarabine
YPR147C
ZShift = 1.45
Z lm Score = −2.4
NG = 0
DB = 0
SM = 0
Delta_L
Cytarabine
YPR147C
ZShift = 1.64
Z lm Score = 0.04
NG = 0
DB = 0
SM = 0
Delta_K
Cytarabine
YPR147C
ZShift = 1.26
Z lm Score = 0.26
NG = 0
DB = 0
SM = 0
Delta_r
Cytarabine
YPR147C
ZShift = 0.46
Z lm Score = 0.9
NG = 0
DB = 0
SM = 0
Delta_AUC
Cytarabine
YNL303W
ZShift = 1.97
Z lm Score = −2.4
NG = 0
DB = 0
SM = 0
Delta_L
Cytarabine
YNL303W
ZShift = −0.6
Z lm Score = 1.46
NG = 0
DB = 0
SM = 0
Delta_K
Cytarabine
YNL303W
ZShift = −1.97
Z lm Score = 2.72
NG = 0
DB = 0
SM = 0
Delta_r
Cytarabine
YNL303W
ZShift = −1.25
Z lm Score = 1.32
NG = 0
DB = 0
SM = 0
Delta_AUC
Cytarabine

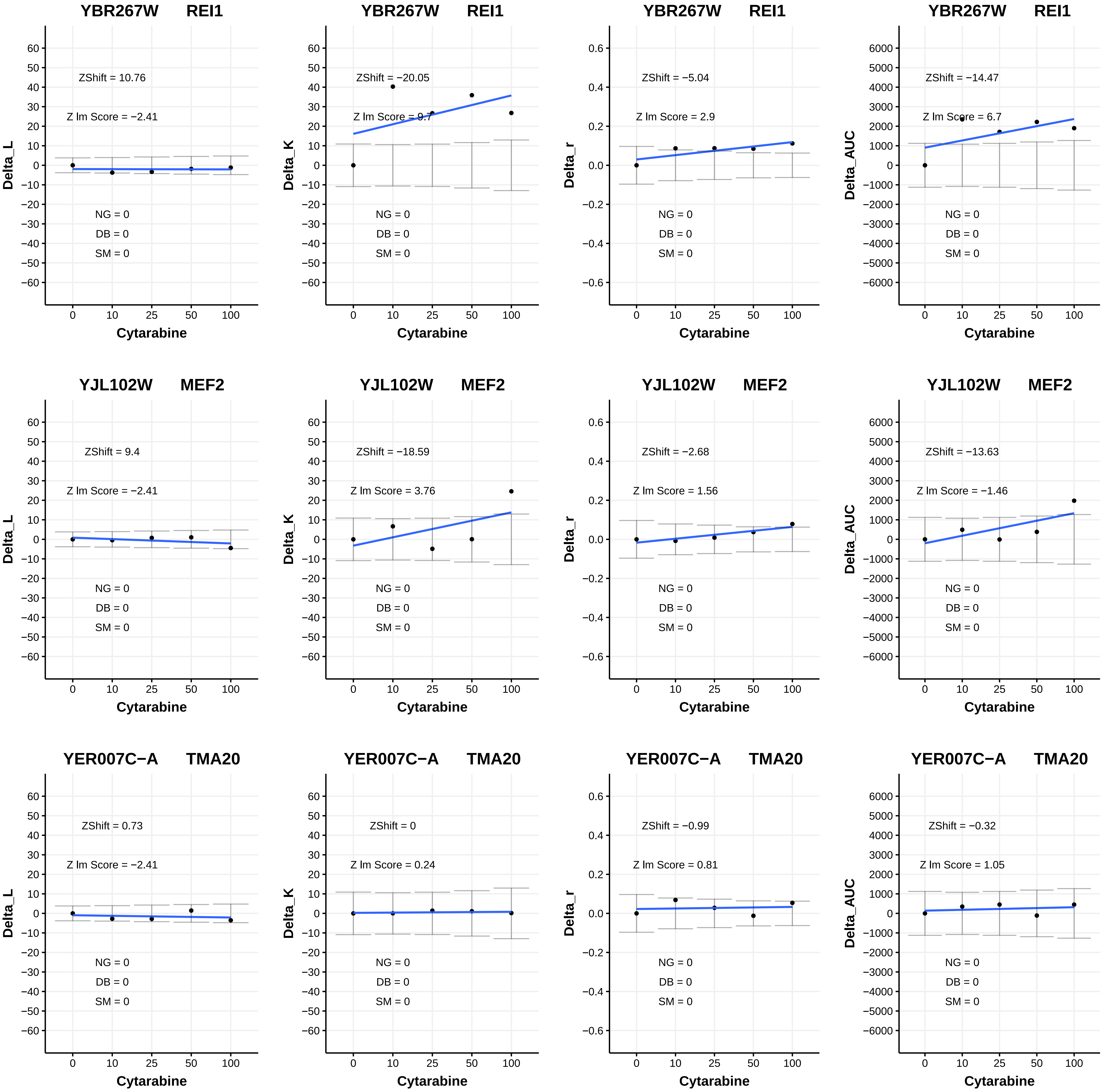

YBR267W REI1
ZShift = 10.76
Z lm Score = −2.41
NG = 0
DB = 0
SM = 0
Delta_L
Cytarabine
YBR267W REI1
ZShift = −20.05
Z lm Score = 9.7
NG = 0
DB = 0
SM = 0
Delta_K
Cytarabine
YBR267W REI1
ZShift = −5.04
Z lm Score = 2.9
NG = 0
DB = 0
SM = 0
Delta_r
Cytarabine
YBR267W REI1
ZShift = −14.47
Z lm Score = 6.7
NG = 0
DB = 0
SM = 0
Delta_AUC
Cytarabine
YJL102W MEF2
ZShift = 9.4
Z lm Score = −2.41
NG = 0
DB = 0
SM = 0
Delta_L
Cytarabine
YJL102W MEF2
ZShift = −18.59
Z lm Score = 3.76
NG = 0
DB = 0
SM = 0
Delta_K
Cytarabine
YJL102W MEF2
ZShift = −2.68
Z lm Score = 1.56
NG = 0
DB = 0
SM = 0
Delta_r
Cytarabine
YJL102W MEF2
ZShift = −13.63
Z lm Score = −1.46
NG = 0
DB = 0
SM = 0
Delta_AUC
Cytarabine
YER007C−A TMA20
ZShift = 0.73
Z lm Score = −2.41
NG = 0
DB = 0
SM = 0
Delta_L
Cytarabine
YER007C−A TMA20
ZShift = 0
Z lm Score = 0.24
NG = 0
DB = 0
SM = 0
Delta_K
Cytarabine
YER007C−A TMA20
ZShift = −0.99
Z lm Score = 0.81
NG = 0
DB = 0
SM = 0
Delta_r
Cytarabine
YER007C−A TMA20
ZShift = −0.32
Z lm Score = 1.05
NG = 0
DB = 0
SM = 0
Delta_AUC
Cytarabine

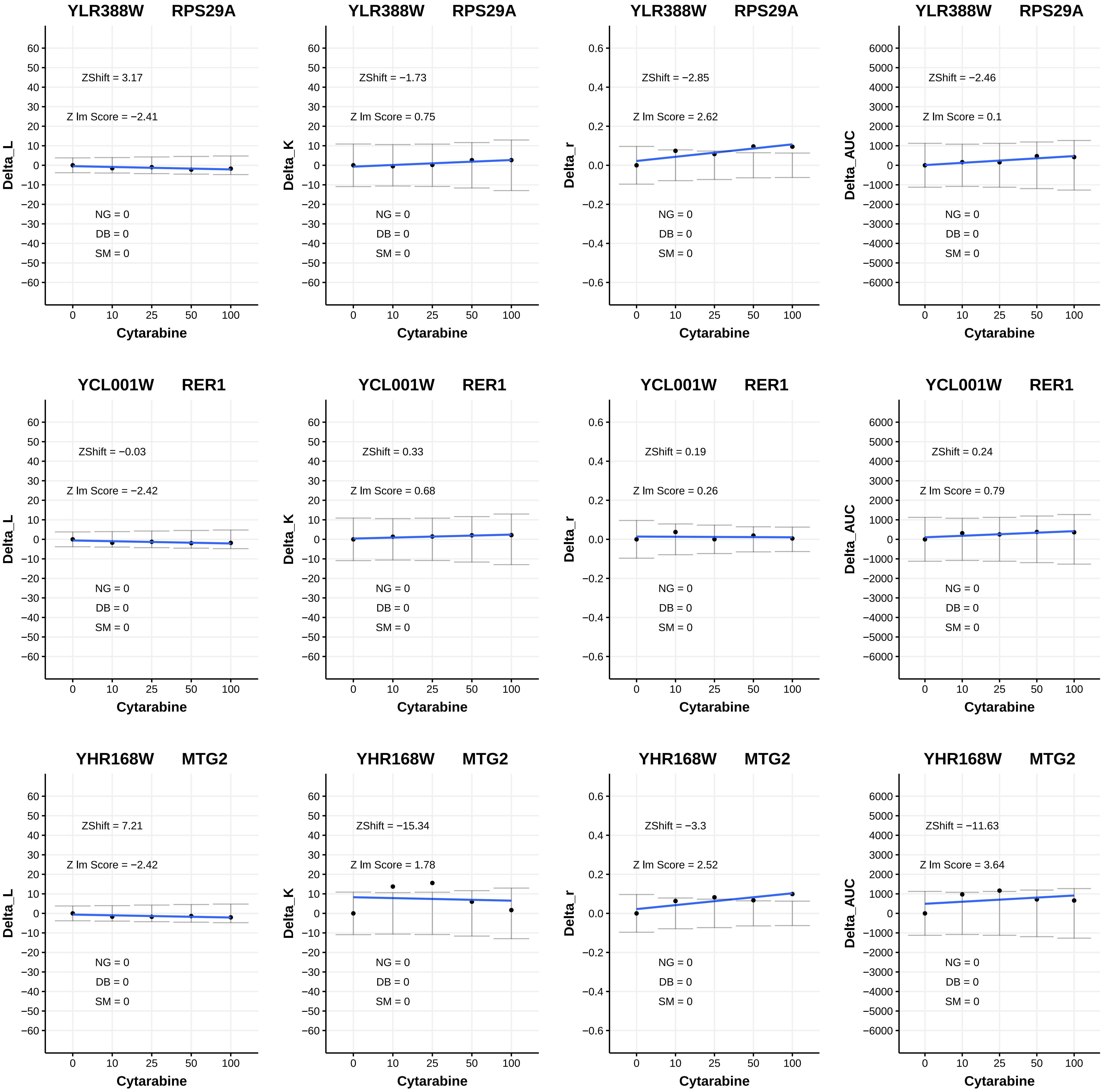
YLR388W RPS29A
ZShift = 3.17
Z lm Score = −2.41
NG = 0
DB = 0
SM = 0
Delta_L
Cytarabine
YLR388W RPS29A
ZShift = −1.73
Z lm Score = 0.75
NG = 0
DB = 0
SM = 0
Delta_K
Cytarabine
YLR388W RPS29A
ZShift = −2.85
Z lm Score = 2.62
NG = 0
DB = 0
SM = 0
Delta_r
Cytarabine
YLR388W RPS29A
ZShift = −2.46
Z lm Score = 0.1
NG = 0
DB = 0
SM = 0
Delta_AUC
Cytarabine
YCL001W RER1
ZShift = −0.03
Z lm Score = −2.42
NG = 0
DB = 0
SM = 0
Delta_L
Cytarabine
YCL001W RER1
ZShift = 0.33
Z lm Score = 0.68
NG = 0
DB = 0
SM = 0
Delta_K
Cytarabine
YCL001W RER1
ZShift = 0.19
Z lm Score = 0.26
NG = 0
DB = 0
SM = 0
Delta_r
Cytarabine
YCL001W RER1
ZShift = 0.24
Z lm Score = 0.79
NG = 0
DB = 0
SM = 0
Delta_AUC
Cytarabine
YHR168W MTG2
ZShift = 7.21
Z lm Score = −2.42
NG = 0
DB = 0
SM = 0
Delta_L
Cytarabine
YHR168W MTG2
ZShift = −15.34
Z lm Score = 1.78
NG = 0
DB = 0
SM = 0
Delta_K
Cytarabine
YHR168W MTG2
ZShift = −3.3
Z lm Score = 2.52
NG = 0
DB = 0
SM = 0
Delta_r
Cytarabine
YHR168W MTG2
ZShift = −11.63
Z lm Score = 3.64
NG = 0
DB = 0
SM = 0
Delta_AUC
Cytarabine

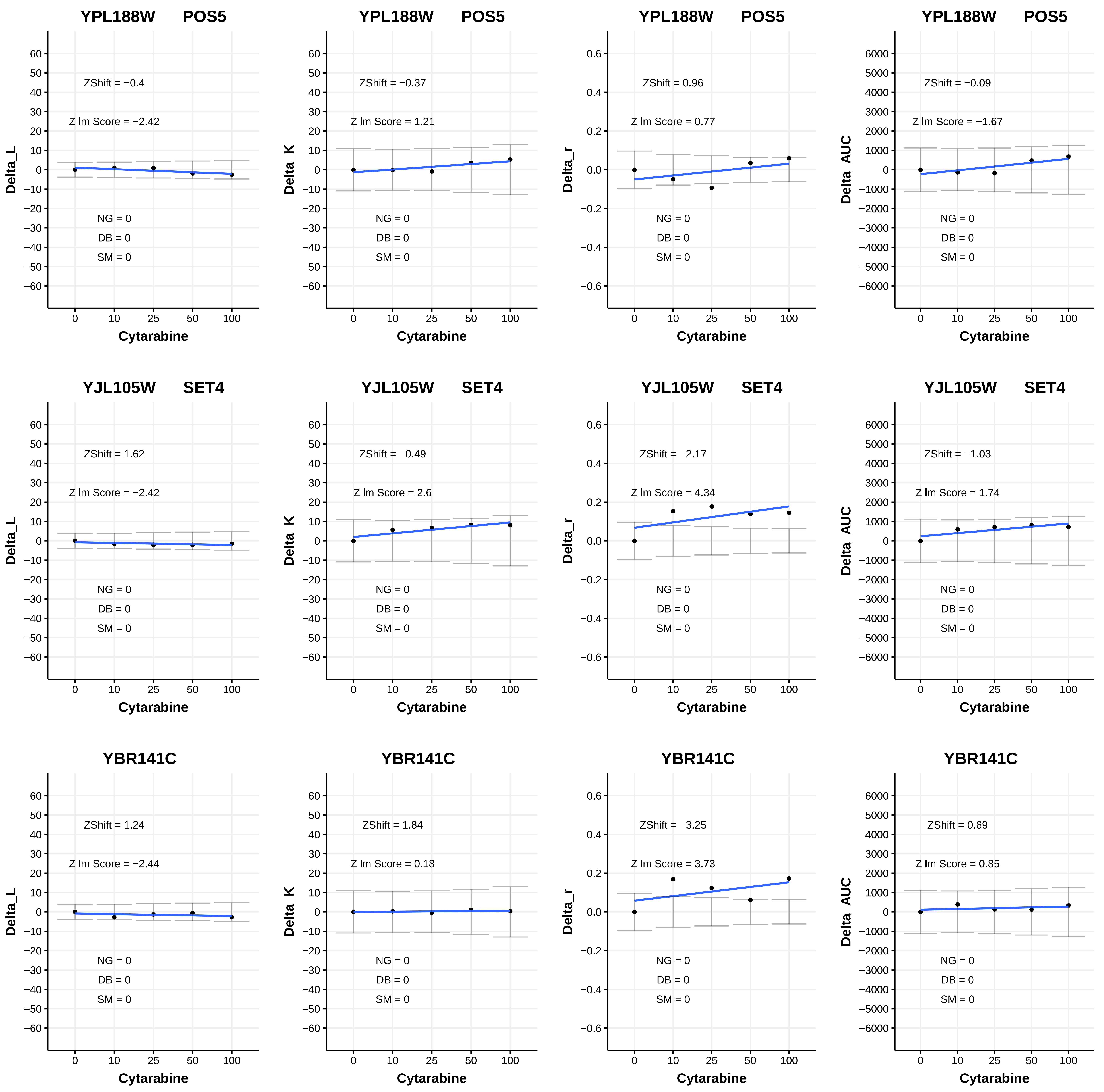
YPL188W POS5
ZShift = −0.4
Z lm Score = −2.42
NG = 0
DB = 0
SM = 0
Delta_L
Cytarabine
YPL188W POS5
ZShift = −0.37
Z lm Score = 1.21
NG = 0
DB = 0
SM = 0
Delta_K
Cytarabine
YPL188W POS5
ZShift = 0.96
Z lm Score = 0.77
NG = 0
DB = 0
SM = 0
Delta_r
Cytarabine
YPL188W POS5
ZShift = −0.09
Z lm Score = −1.67
NG = 0
DB = 0
SM = 0
Delta_AUC
Cytarabine
YJL105W SET4
ZShift = 1.62
Z lm Score = −2.42
NG = 0
DB = 0
SM = 0
Delta_L
Cytarabine
YJL105W SET4
ZShift = −0.49
Z lm Score = 2.6
NG = 0
DB = 0
SM = 0
Delta_K
Cytarabine
YJL105W SET4
ZShift = −2.17
Z lm Score = 4.34
NG = 0
DB = 0
SM = 0
Delta_r
Cytarabine
YJL105W SET4
ZShift = −1.03
Z lm Score = 1.74
NG = 0
DB = 0
SM = 0
Delta_AUC
Cytarabine
YBR141C
ZShift = 1.24
Z lm Score = −2.44
NG = 0
DB = 0
SM = 0
Delta_L
Cytarabine
YBR141C
ZShift = 1.84
Z lm Score = 0.18
NG = 0
DB = 0
SM = 0
Delta_K
Cytarabine
YBR141C
ZShift = −3.25
Z lm Score = 3.73
NG = 0
DB = 0
SM = 0
Delta_r
Cytarabine
YBR141C
ZShift = 0.69
Z lm Score = 0.85
NG = 0
DB = 0
SM = 0
Delta_AUC
Cytarabine

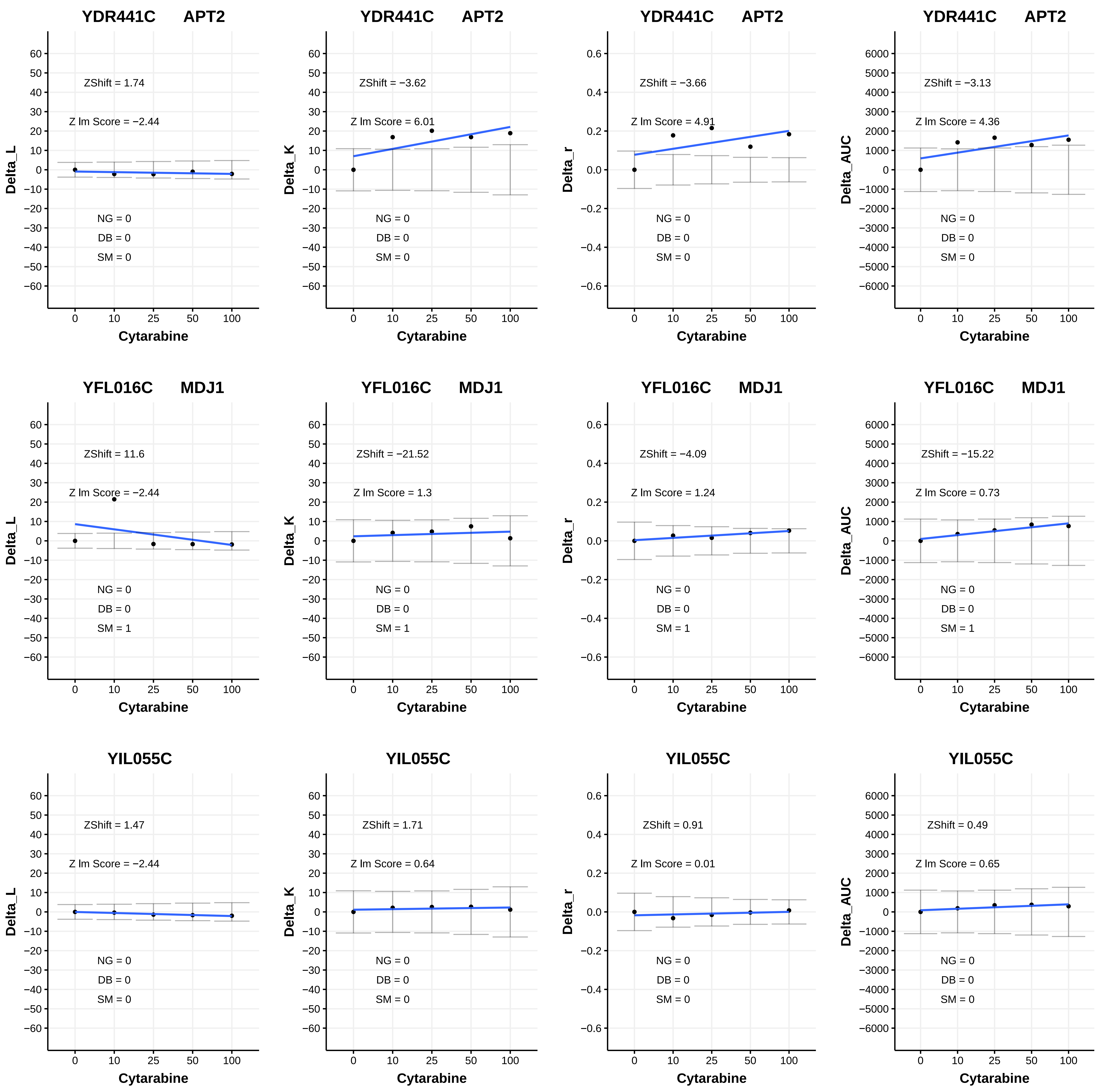
YDR441C APT2
ZShift = 1.74
Z lm Score = −2.44
NG = 0
DB = 0
SM = 0
Delta_L
Cytarabine
YDR441C APT2
ZShift = −3.62
Z lm Score = 6.01
NG = 0
DB = 0
SM = 0
Delta_K
Cytarabine
YDR441C APT2
ZShift = −3.66
Z lm Score = 4.91
NG = 0
DB = 0
SM = 0
Delta_r
Cytarabine
YDR441C APT2
ZShift = −3.13
Z lm Score = 4.36
NG = 0
DB = 0
SM = 0
Delta_AUC
Cytarabine
YFL016C MDJ1
ZShift = 11.6
Z lm Score = −2.44
NG = 0
DB = 0
SM = 1
Delta_L
Cytarabine
YFL016C MDJ1
ZShift = −21.52
Z lm Score = 1.3
NG = 0
DB = 0
SM = 1
Delta_K
Cytarabine
YFL016C MDJ1
ZShift = −4.09
Z lm Score = 1.24
NG = 0
DB = 0
SM = 1
Delta_r
Cytarabine
YFL016C MDJ1
ZShift = −15.22
Z lm Score = 0.73
NG = 0
DB = 0
SM = 1
Delta_AUC
Cytarabine
YIL055C
ZShift = 1.47
Z lm Score = −2.44
NG = 0
DB = 0
SM = 0
Delta_L
Cytarabine
YIL055C
ZShift = 1.71
Z lm Score = 0.64
NG = 0
DB = 0
SM = 0
Delta_K
Cytarabine
YIL055C
ZShift = 0.91
Z lm Score = 0.01
NG = 0
DB = 0
SM = 0
Delta_r
Cytarabine
YIL055C
ZShift = 0.49
Z lm Score = 0.65
NG = 0
DB = 0
SM = 0
Delta_AUC
Cytarabine

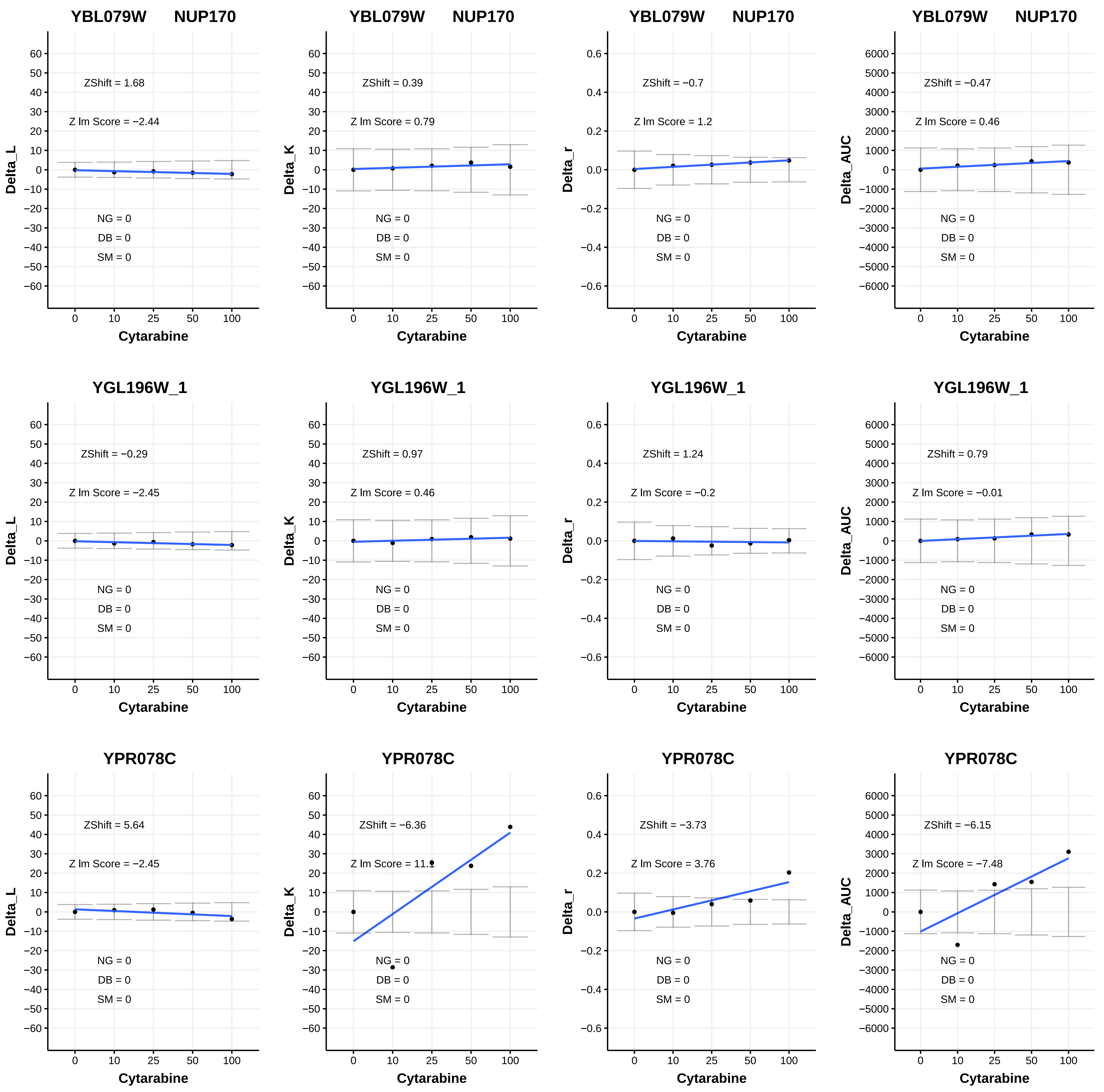
YBL079W NUP170
ZShift = 1.68
Z lm Score = −2.44
NG = 0
DB = 0
SM = 0
Delta_L
Cytarabine
ZShift = 0.39
Z lm Score = 0.79
Delta_K
ZShift = −0.7
Z lm Score = 1.2
Delta_r
ZShift = −0.47
Z lm Score = 0.46
Delta_AUC
YGL196W_1
ZShift = −0.29
Z lm Score = −2.45
ZShift = 0.97
Z lm Score = 0.46
ZShift = 1.24
Z lm Score = −0.2
ZShift = 0.79
Z lm Score = −0.01
YPR078C
ZShift = 5.64
Z lm Score = −2.45
ZShift = −6.36
Z lm Score = 11.1
ZShift = −3.73
Z lm Score = 3.76
ZShift = −6.15
Z lm Score = −7.48

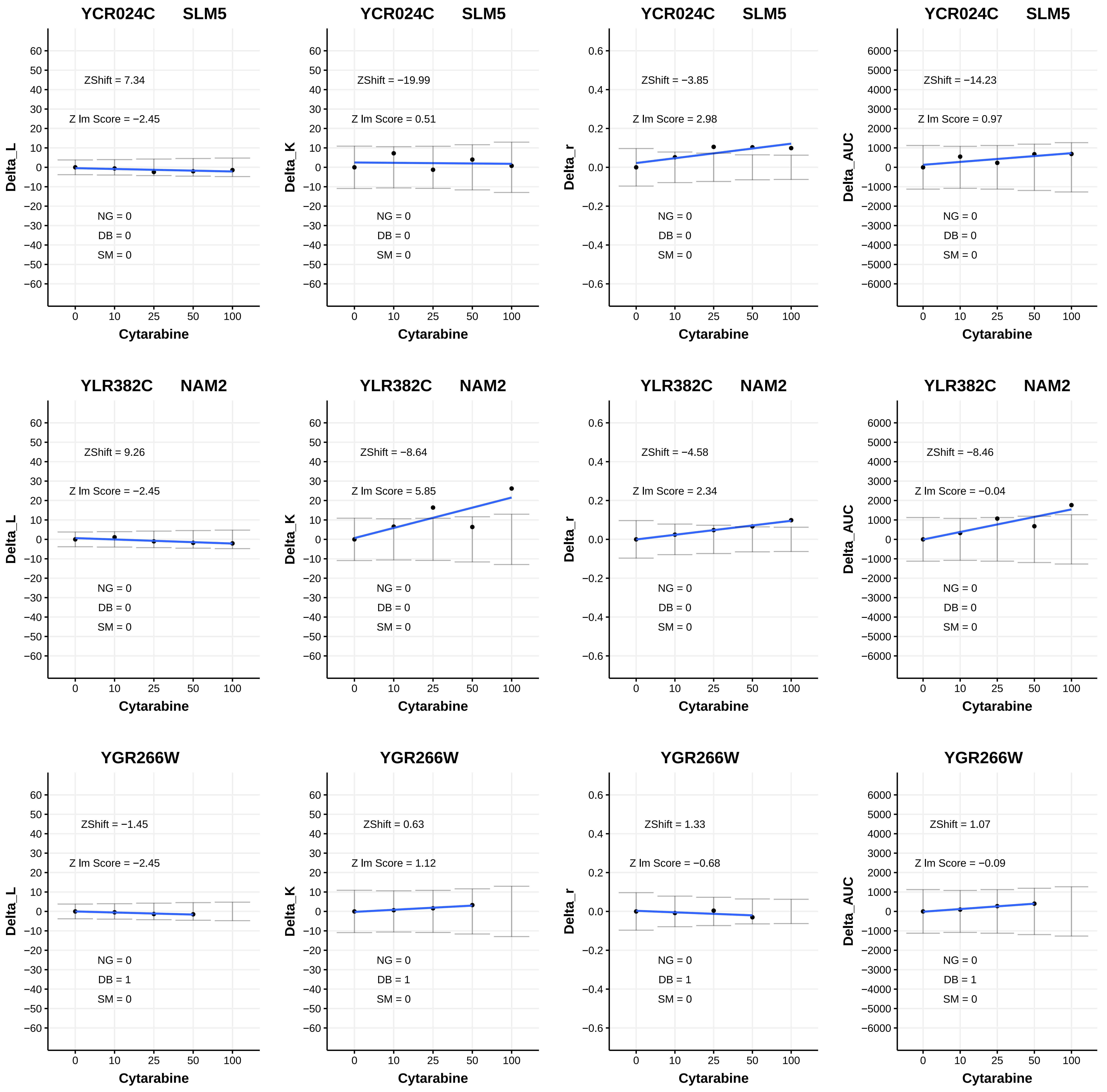
YCR024C SLM5
ZShift = 7.34
Z lm Score = −2.45
NG = 0
DB = 0
SM = 0
Delta_L
Cytarabine
YCR024C SLM5
ZShift = −19.99
Z lm Score = 0.51
NG = 0
DB = 0
SM = 0
Delta_K
Cytarabine
YCR024C SLM5
ZShift = −3.85
Z lm Score = 2.98
NG = 0
DB = 0
SM = 0
Delta_r
Cytarabine
YCR024C SLM5
ZShift = −14.23
Z lm Score = 0.97
NG = 0
DB = 0
SM = 0
Delta_AUC
Cytarabine
YLR382C NAM2
ZShift = 9.26
Z lm Score = −2.45
NG = 0
DB = 0
SM = 0
Delta_L
Cytarabine
YLR382C NAM2
ZShift = −8.64
Z lm Score = 5.85
NG = 0
DB = 0
SM = 0
Delta_K
Cytarabine
YLR382C NAM2
ZShift = −4.58
Z lm Score = 2.34
NG = 0
DB = 0
SM = 0
Delta_r
Cytarabine
YLR382C NAM2
ZShift = −8.46
Z lm Score = −0.04
NG = 0
DB = 0
SM = 0
Delta_AUC
Cytarabine
YGR266W
ZShift = −1.45
Z lm Score = −2.45
NG = 0
DB = 1
SM = 0
Delta_L
Cytarabine
YGR266W
ZShift = 0.63
Z lm Score = 1.12
NG = 0
DB = 1
SM = 0
Delta_K
Cytarabine
YGR266W
ZShift = 1.33
Z lm Score = −0.68
NG = 0
DB = 1
SM = 0
Delta_r
Cytarabine
YGR266W
ZShift = 1.07
Z lm Score = −0.09
NG = 0
DB = 1
SM = 0
Delta_AUC
Cytarabine

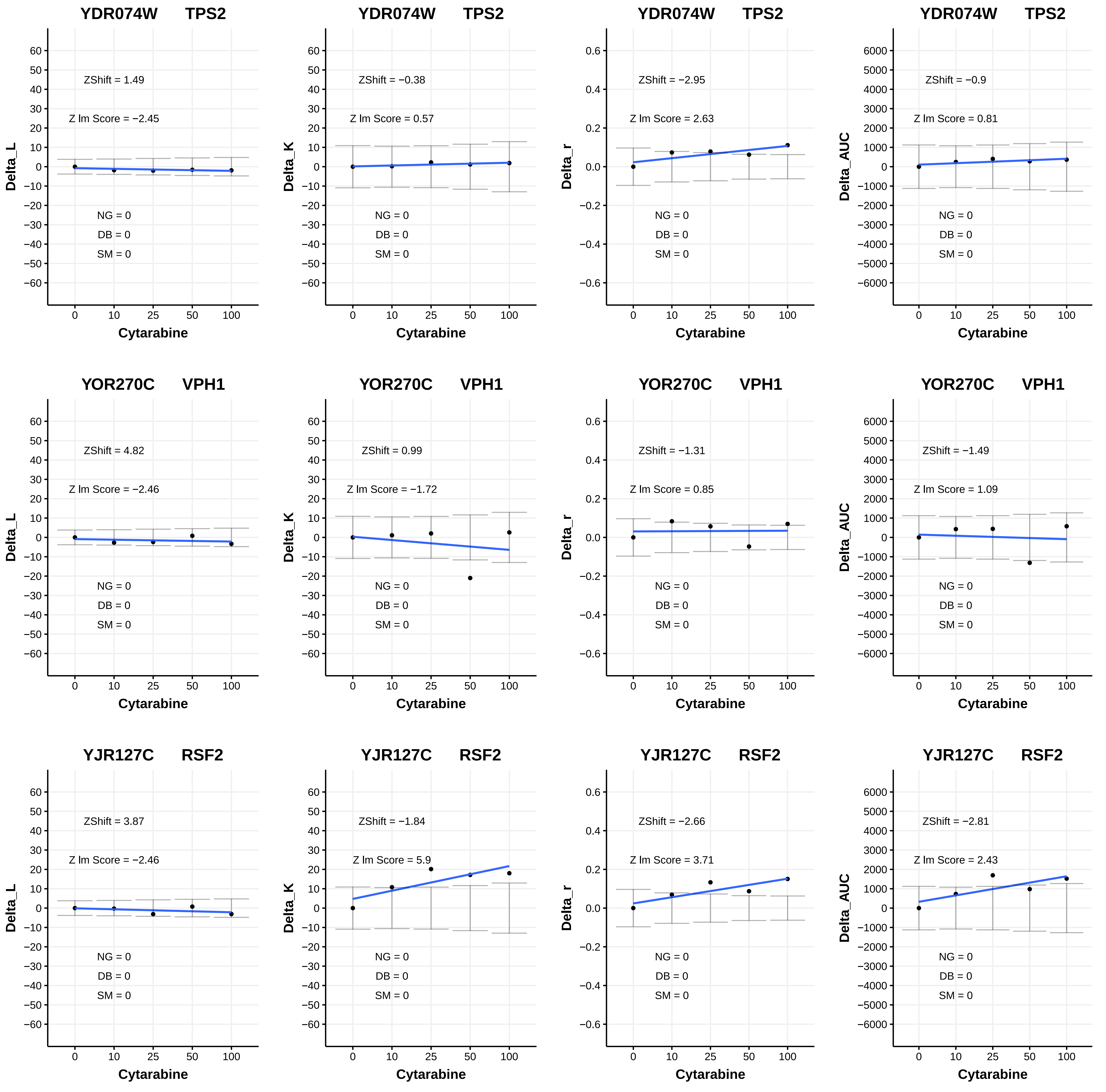
YDR074W TPS2
ZShift = 1.49
Z lm Score = −2.45
NG = 0
DB = 0
SM = 0
Delta_L
Cytarabine
YDR074W TPS2
ZShift = −0.38
Z lm Score = 0.57
NG = 0
DB = 0
SM = 0
Delta_K
Cytarabine
YDR074W TPS2
ZShift = −2.95
Z lm Score = 2.63
NG = 0
DB = 0
SM = 0
Delta_r
Cytarabine
YDR074W TPS2
ZShift = −0.9
Z lm Score = 0.81
NG = 0
DB = 0
SM = 0
Delta_AUC
Cytarabine
YOR270C VPH1
ZShift = 4.82
Z lm Score = −2.46
NG = 0
DB = 0
SM = 0
Delta_L
Cytarabine
YOR270C VPH1
ZShift = 0.99
Z lm Score = −1.72
NG = 0
DB = 0
SM = 0
Delta_K
Cytarabine
YOR270C VPH1
ZShift = −1.31
Z lm Score = 0.85
NG = 0
DB = 0
SM = 0
Delta_r
Cytarabine
YOR270C VPH1
ZShift = −1.49
Z lm Score = 1.09
NG = 0
DB = 0
SM = 0
Delta_AUC
Cytarabine
YJR127C RSF2
ZShift = 3.87
Z lm Score = −2.46
NG = 0
DB = 0
SM = 0
Delta_L
Cytarabine
YJR127C RSF2
ZShift = −1.84
Z lm Score = 5.9
NG = 0
DB = 0
SM = 0
Delta_K
Cytarabine
YJR127C RSF2
ZShift = −2.66
Z lm Score = 3.71
NG = 0
DB = 0
SM = 0
Delta_r
Cytarabine
YJR127C RSF2
ZShift = −2.81
Z lm Score = 2.43
NG = 0
DB = 0
SM = 0
Delta_AUC
Cytarabine

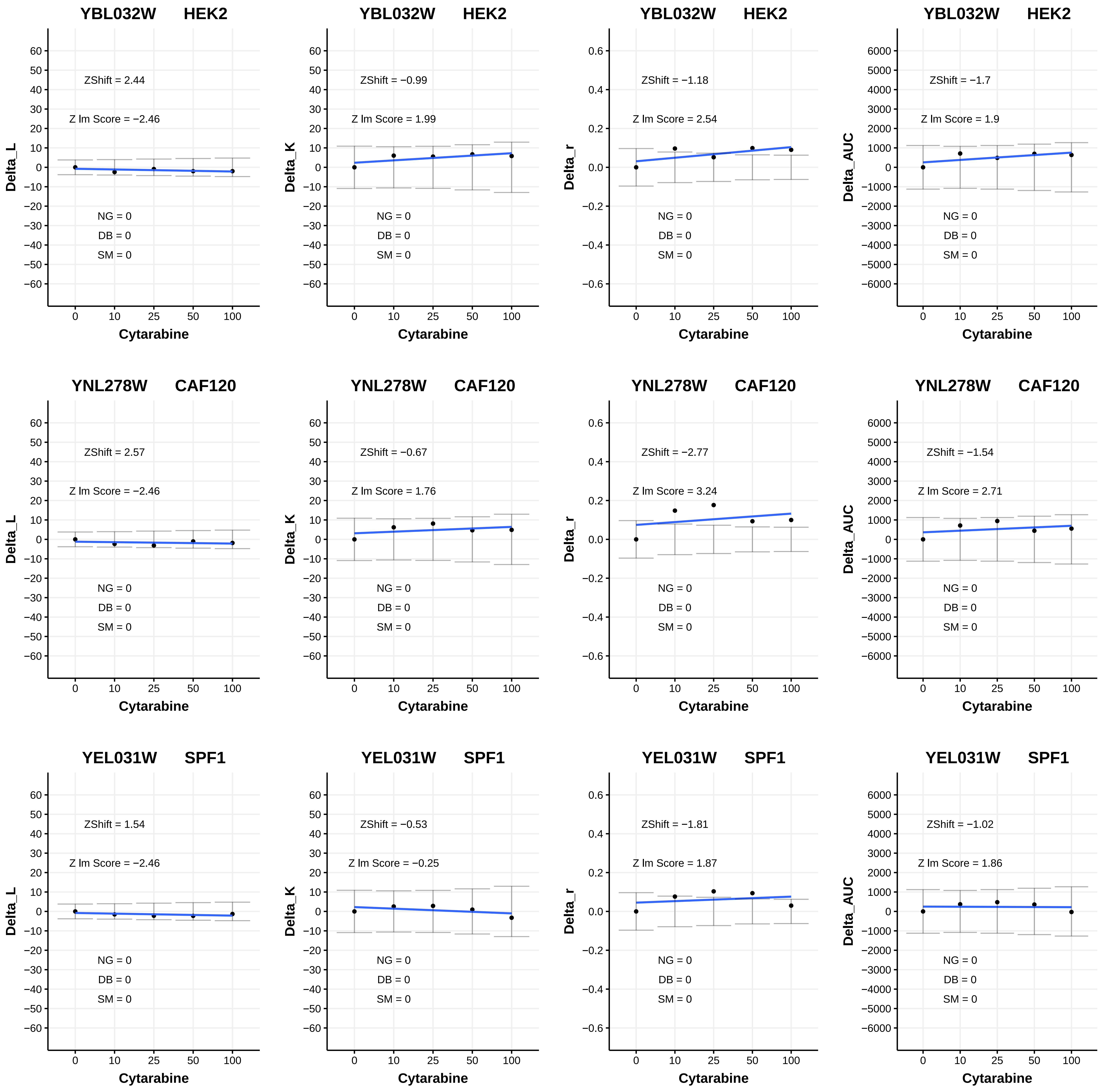

YBL032W HEK2
ZShift = 2.44
Z lm Score = −2.46
NG = 0
DB = 0
SM = 0
Delta_L
Cytarabine
YBL032W HEK2
ZShift = −0.99
Z lm Score = 1.99
NG = 0
DB = 0
SM = 0
Delta_K
Cytarabine
YBL032W HEK2
ZShift = −1.18
Z lm Score = 2.54
NG = 0
DB = 0
SM = 0
Delta_r
Cytarabine
YBL032W HEK2
ZShift = −1.7
Z lm Score = 1.9
NG = 0
DB = 0
SM = 0
Delta_AUC
Cytarabine
YNL278W CAF120
ZShift = 2.57
Z lm Score = −2.46
NG = 0
DB = 0
SM = 0
Delta_L
Cytarabine
YNL278W CAF120
ZShift = −0.67
Z lm Score = 1.76
NG = 0
DB = 0
SM = 0
Delta_K
Cytarabine
YNL278W CAF120
ZShift = −2.77
Z lm Score = 3.24
NG = 0
DB = 0
SM = 0
Delta_r
Cytarabine
YNL278W CAF120
ZShift = −1.54
Z lm Score = 2.71
NG = 0
DB = 0
SM = 0
Delta_AUC
Cytarabine
YEL031W SPF1
ZShift = 1.54
Z lm Score = −2.46
NG = 0
DB = 0
SM = 0
Delta_L
Cytarabine
YEL031W SPF1
ZShift = −0.53
Z lm Score = −0.25
NG = 0
DB = 0
SM = 0
Delta_K
Cytarabine
YEL031W SPF1
ZShift = −1.81
Z lm Score = 1.87
NG = 0
DB = 0
SM = 0
Delta_r
Cytarabine
YEL031W SPF1
ZShift = −1.02
Z lm Score = 1.86
NG = 0
DB = 0
SM = 0
Delta_AUC
Cytarabine

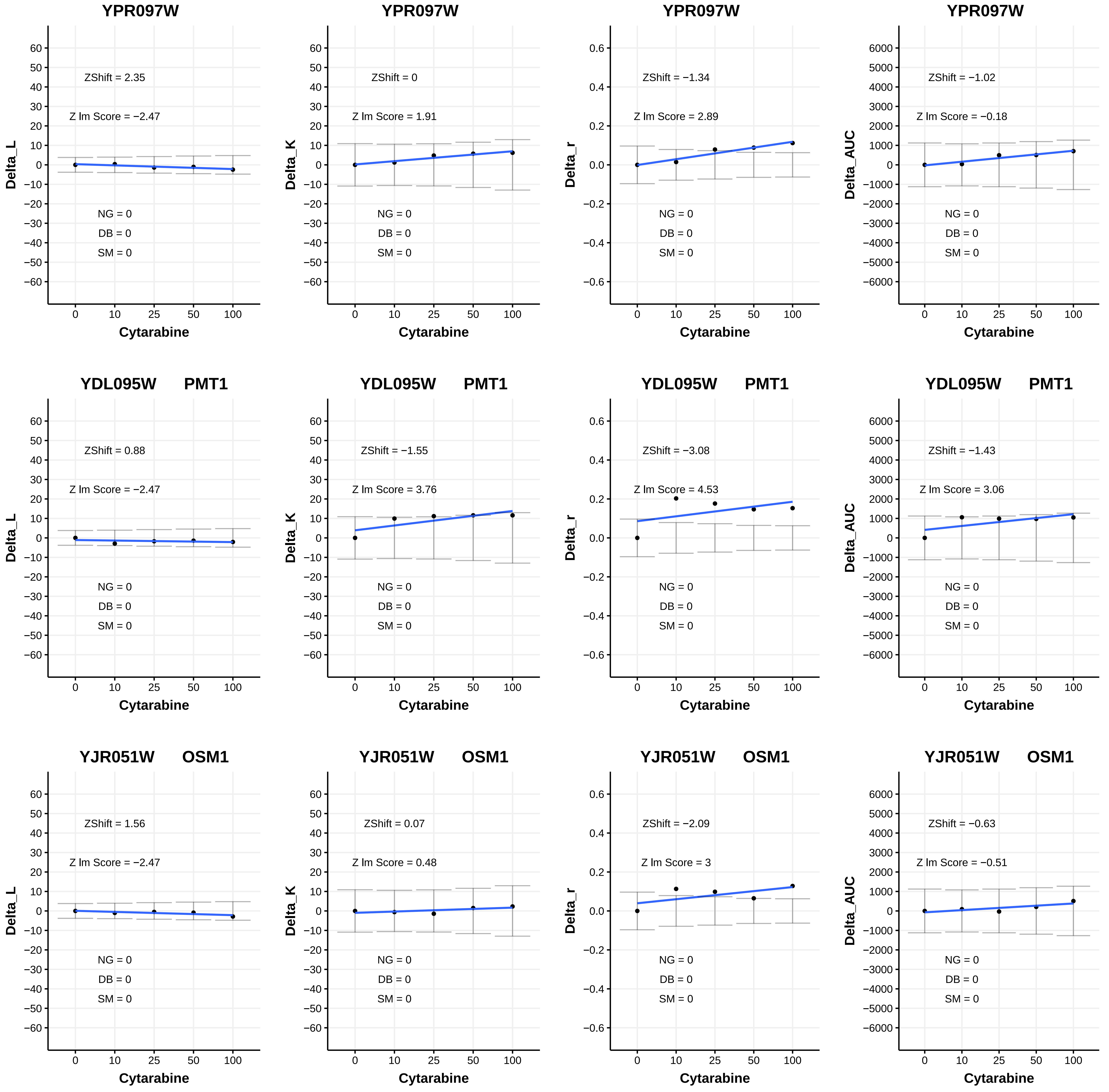
YPR097W
ZShift = 2.35
Z lm Score = −2.47
NG = 0
DB = 0
SM = 0
Delta_L
Cytarabine
YPR097W
ZShift = 0
Z lm Score = 1.91
NG = 0
DB = 0
SM = 0
Delta_K
Cytarabine
YPR097W
ZShift = −1.34
Z lm Score = 2.89
NG = 0
DB = 0
SM = 0
Delta_r
Cytarabine
YPR097W
ZShift = −1.02
Z lm Score = −0.18
NG = 0
DB = 0
SM = 0
Delta_AUC
Cytarabine
YDL095W PMT1
ZShift = 0.88
Z lm Score = −2.47
NG = 0
DB = 0
SM = 0
Delta_L
Cytarabine
YDL095W PMT1
ZShift = −1.55
Z lm Score = 3.76
NG = 0
DB = 0
SM = 0
Delta_K
Cytarabine
YDL095W PMT1
ZShift = −3.08
Z lm Score = 4.53
NG = 0
DB = 0
SM = 0
Delta_r
Cytarabine
YDL095W PMT1
ZShift = −1.43
Z lm Score = 3.06
NG = 0
DB = 0
SM = 0
Delta_AUC
Cytarabine
YJR051W OSM1
ZShift = 1.56
Z lm Score = −2.47
NG = 0
DB = 0
SM = 0
Delta_L
Cytarabine
YJR051W OSM1
ZShift = 0.07
Z lm Score = 0.48
NG = 0
DB = 0
SM = 0
Delta_K
Cytarabine
YJR051W OSM1
ZShift = −2.09
Z lm Score = 3
NG = 0
DB = 0
SM = 0
Delta_r
Cytarabine
YJR051W OSM1
ZShift = −0.63
Z lm Score = −0.51
NG = 0
DB = 0
SM = 0
Delta_AUC
Cytarabine

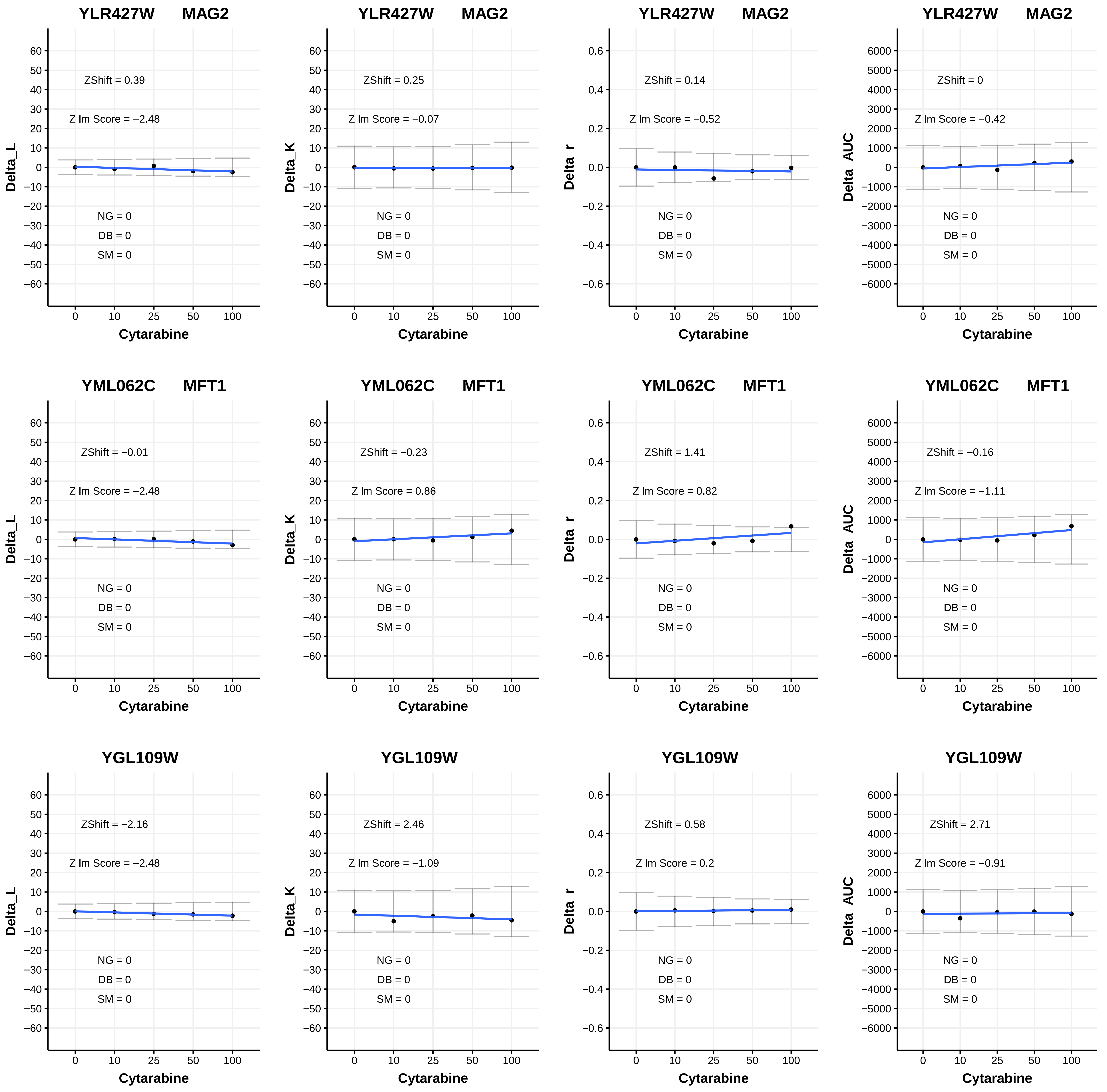
YLR427W MAG2
ZShift = 0.39
Z lm Score = −2.48
NG = 0
DB = 0
SM = 0
Delta_L
Cytarabine
YLR427W MAG2
ZShift = 0.25
Z lm Score = −0.07
NG = 0
DB = 0
SM = 0
Delta_K
Cytarabine
YLR427W MAG2
ZShift = 0.14
Z lm Score = −0.52
NG = 0
DB = 0
SM = 0
Delta_r
Cytarabine
YLR427W MAG2
ZShift = 0
Z lm Score = −0.42
NG = 0
DB = 0
SM = 0
Delta_AUC
Cytarabine
YML062C MFT1
ZShift = −0.01
Z lm Score = −2.48
NG = 0
DB = 0
SM = 0
Delta_L
Cytarabine
YML062C MFT1
ZShift = −0.23
Z lm Score = 0.86
NG = 0
DB = 0
SM = 0
Delta_K
Cytarabine
YML062C MFT1
ZShift = 1.41
Z lm Score = 0.82
NG = 0
DB = 0
SM = 0
Delta_r
Cytarabine
YML062C MFT1
ZShift = −0.16
Z lm Score = −1.11
NG = 0
DB = 0
SM = 0
Delta_AUC
Cytarabine
YGL109W
ZShift = −2.16
Z lm Score = −2.48
NG = 0
DB = 0
SM = 0
Delta_L
Cytarabine
YGL109W
ZShift = 2.46
Z lm Score = −1.09
NG = 0
DB = 0
SM = 0
Delta_K
Cytarabine
YGL109W
ZShift = 0.58
Z lm Score = 0.2
NG = 0
DB = 0
SM = 0
Delta_r
Cytarabine
YGL109W
ZShift = 2.71
Z lm Score = −0.91
NG = 0
DB = 0
SM = 0
Delta_AUC
Cytarabine

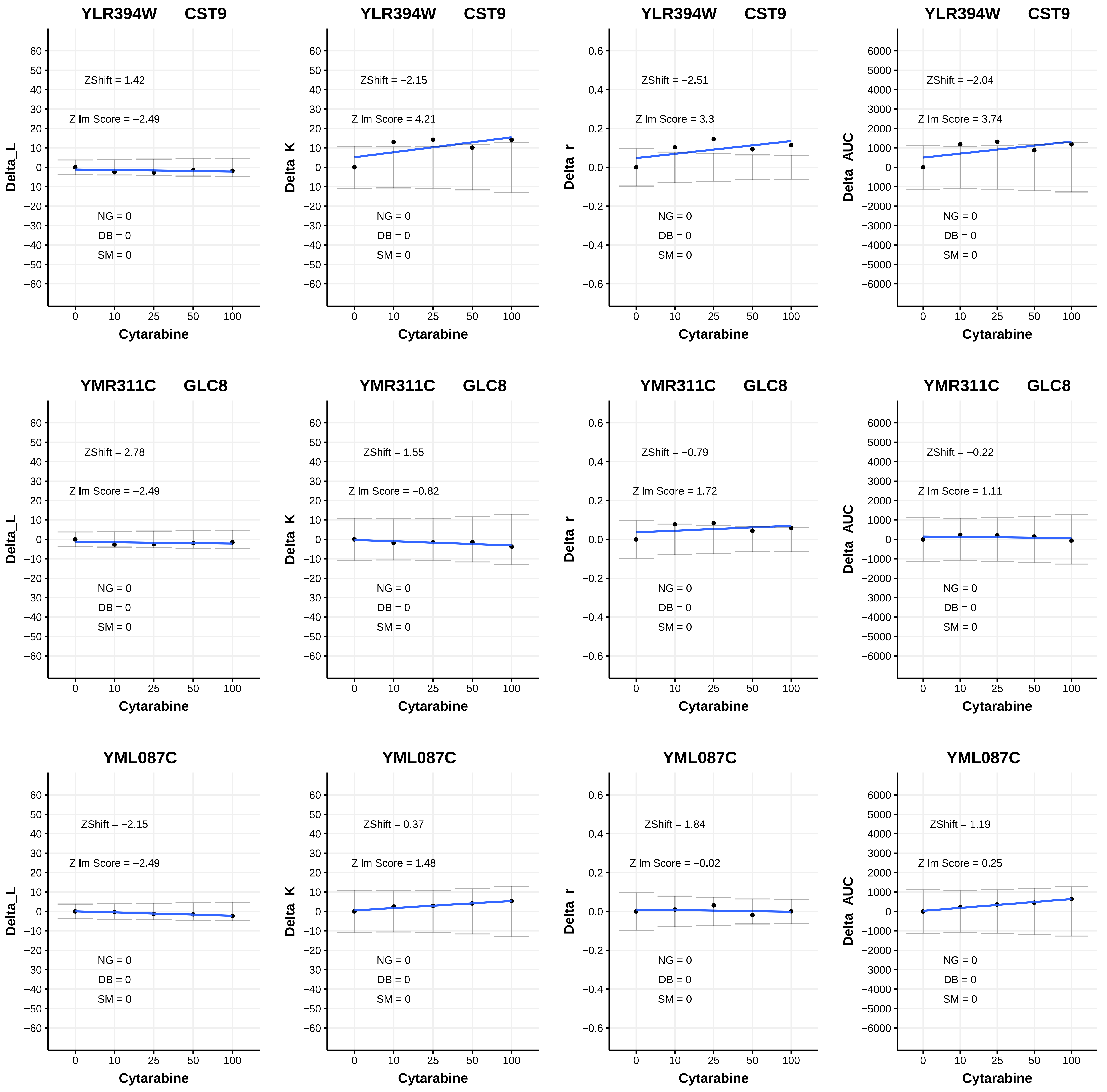

YLR394W CST9
ZShift = 1.42
Z lm Score = −2.49
NG = 0
DB = 0
SM = 0
Delta_L
Cytarabine
YLR394W CST9
ZShift = −2.15
Z lm Score = 4.21
NG = 0
DB = 0
SM = 0
Delta_K
Cytarabine
YLR394W CST9
ZShift = −2.51
Z lm Score = 3.3
NG = 0
DB = 0
SM = 0
Delta_r
Cytarabine
YLR394W CST9
ZShift = −2.04
Z lm Score = 3.74
NG = 0
DB = 0
SM = 0
Delta_AUC
Cytarabine
YMR311C GLC8
ZShift = 2.78
Z lm Score = −2.49
NG = 0
DB = 0
SM = 0
Delta_L
Cytarabine
YMR311C GLC8
ZShift = 1.55
Z lm Score = −0.82
NG = 0
DB = 0
SM = 0
Delta_K
Cytarabine
YMR311C GLC8
ZShift = −0.79
Z lm Score = 1.72
NG = 0
DB = 0
SM = 0
Delta_r
Cytarabine
YMR311C GLC8
ZShift = −0.22
Z lm Score = 1.11
NG = 0
DB = 0
SM = 0
Delta_AUC
Cytarabine
YML087C
ZShift = −2.15
Z lm Score = −2.49
NG = 0
DB = 0
SM = 0
Delta_L
Cytarabine
YML087C
ZShift = 0.37
Z lm Score = 1.48
NG = 0
DB = 0
SM = 0
Delta_K
Cytarabine
YML087C
ZShift = 1.84
Z lm Score = −0.02
NG = 0
DB = 0
SM = 0
Delta_r
Cytarabine
YML087C
ZShift = 1.19
Z lm Score = 0.25
NG = 0
DB = 0
SM = 0
Delta_AUC
Cytarabine

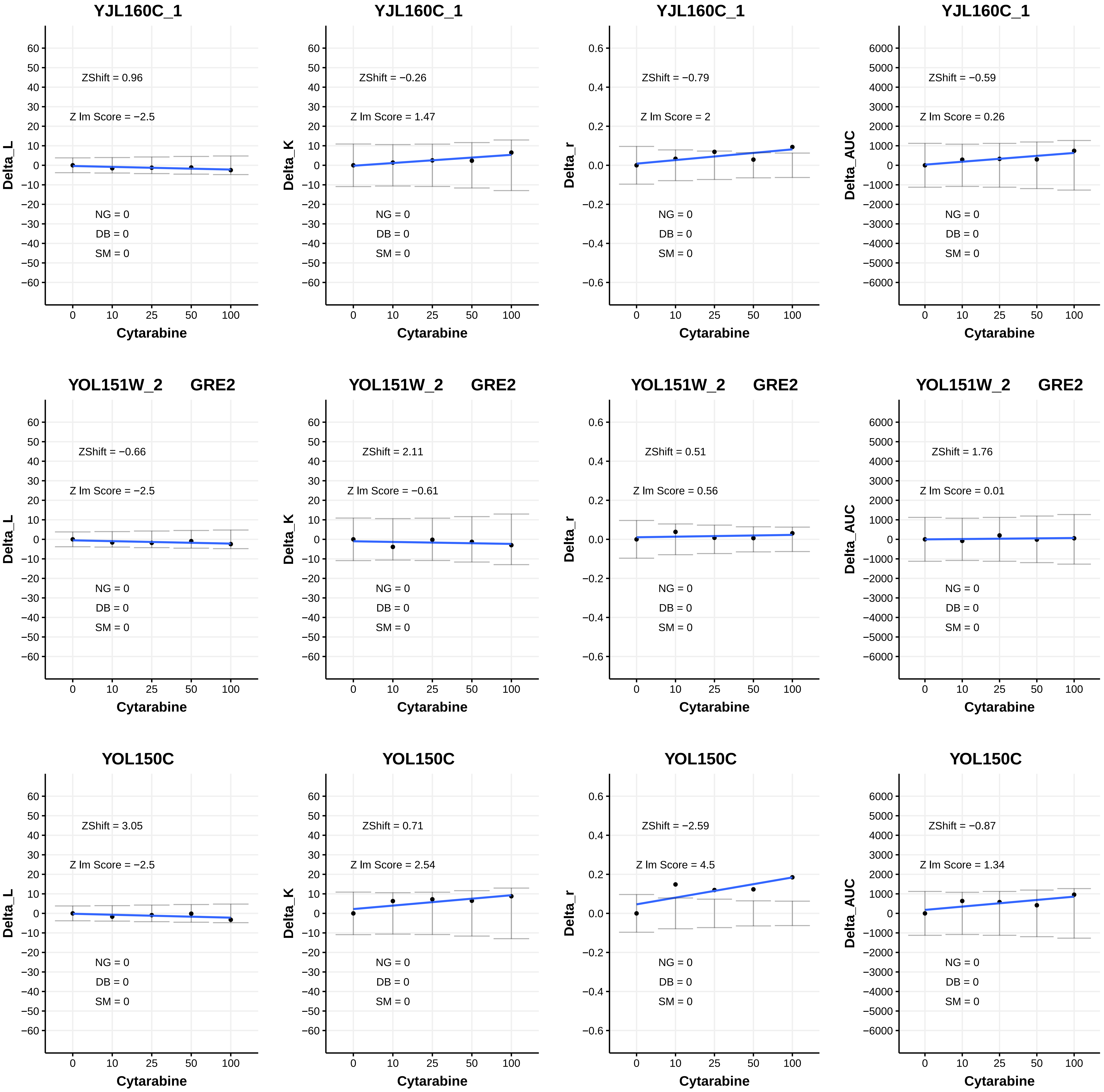

YJL160C_1
ZShift = 0.96
Z lm Score = −2.5
NG = 0
DB = 0
SM = 0
Delta_L
Cytarabine
YJL160C_1
ZShift = −0.26
Z lm Score = 1.47
NG = 0
DB = 0
SM = 0
Delta_K
Cytarabine
YJL160C_1
ZShift = −0.79
Z lm Score = 2
NG = 0
DB = 0
SM = 0
Delta_r
Cytarabine
YJL160C_1
ZShift = −0.59
Z lm Score = 0.26
NG = 0
DB = 0
SM = 0
Delta_AUC
Cytarabine
YOL151W_2 GRE2
ZShift = −0.66
Z lm Score = −2.5
NG = 0
DB = 0
SM = 0
Delta_L
Cytarabine
YOL151W_2 GRE2
ZShift = 2.11
Z lm Score = −0.61
NG = 0
DB = 0
SM = 0
Delta_K
Cytarabine
YOL151W_2 GRE2
ZShift = 0.51
Z lm Score = 0.56
NG = 0
DB = 0
SM = 0
Delta_r
Cytarabine
YOL151W_2 GRE2
ZShift = 1.76
Z lm Score = 0.01
NG = 0
DB = 0
SM = 0
Delta_AUC
Cytarabine
YOL150C
ZShift = 3.05
Z lm Score = −2.5
NG = 0
DB = 0
SM = 0
Delta_L
Cytarabine
YOL150C
ZShift = 0.71
Z lm Score = 2.54
NG = 0
DB = 0
SM = 0
Delta_K
Cytarabine
YOL150C
ZShift = −2.59
Z lm Score = 4.5
NG = 0
DB = 0
SM = 0
Delta_r
Cytarabine
YOL150C
ZShift = −0.87
Z lm Score = 1.34
NG = 0
DB = 0
SM = 0
Delta_AUC
Cytarabine

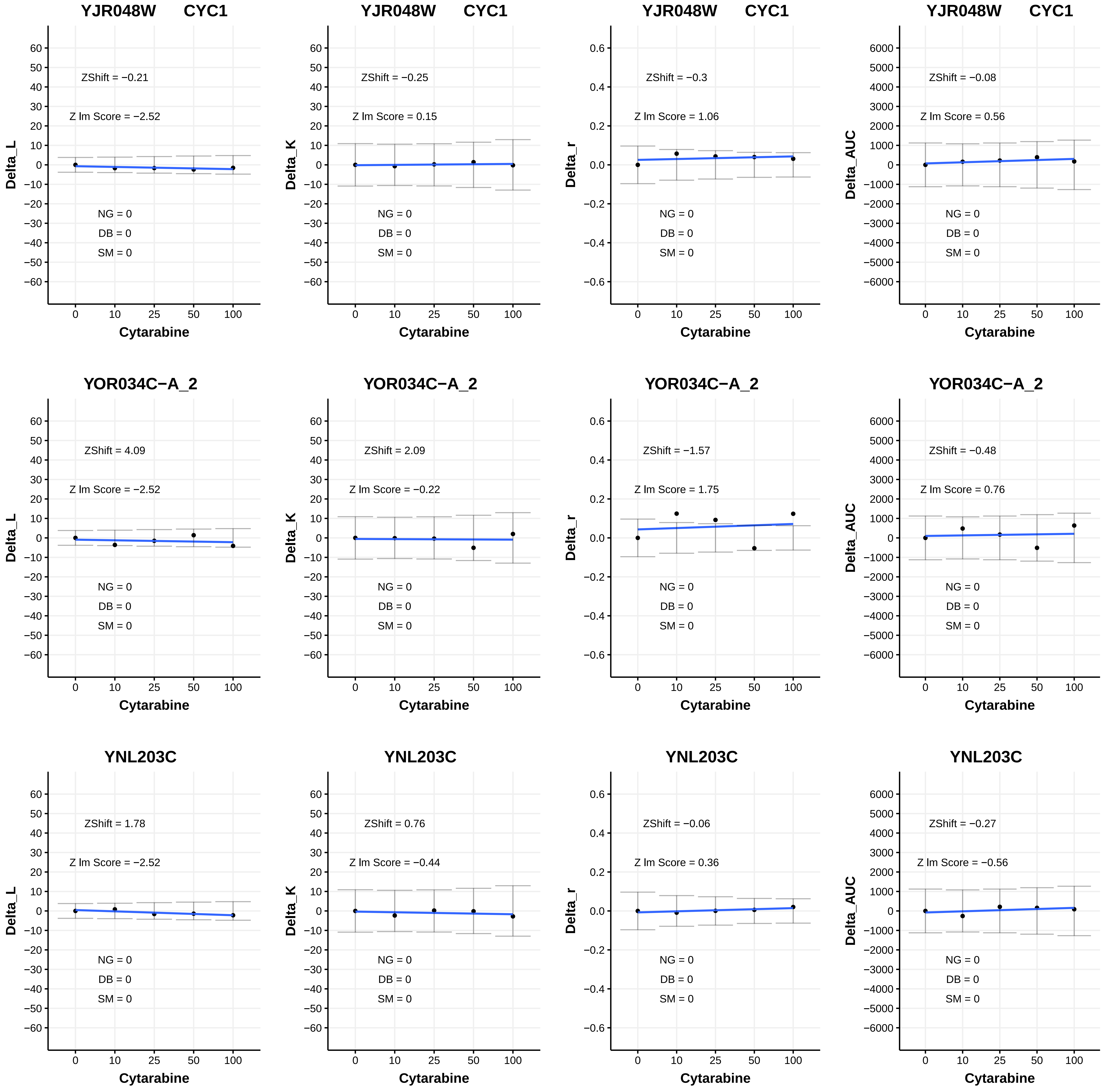

YJR048W CYC1
ZShift = −0.21
Z lm Score = −2.52
NG = 0
DB = 0
SM = 0
Delta_L
Cytarabine
YJR048W CYC1
ZShift = −0.25
Z lm Score = 0.15
NG = 0
DB = 0
SM = 0
Delta_K
Cytarabine
YJR048W CYC1
ZShift = −0.3
Z lm Score = 1.06
NG = 0
DB = 0
SM = 0
Delta_r
Cytarabine
YJR048W CYC1
ZShift = −0.08
Z lm Score = 0.56
NG = 0
DB = 0
SM = 0
Delta_AUC
Cytarabine
YOR034C−A_2
ZShift = 4.09
Z lm Score = −2.52
NG = 0
DB = 0
SM = 0
Delta_L
Cytarabine
YOR034C−A_2
ZShift = 2.09
Z lm Score = −0.22
NG = 0
DB = 0
SM = 0
Delta_K
Cytarabine
YOR034C−A_2
ZShift = −1.57
Z lm Score = 1.75
NG = 0
DB = 0
SM = 0
Delta_r
Cytarabine
YOR034C−A_2
ZShift = −0.48
Z lm Score = 0.76
NG = 0
DB = 0
SM = 0
Delta_AUC
Cytarabine
YNL203C
ZShift = 1.78
Z lm Score = −2.52
NG = 0
DB = 0
SM = 0
Delta_L
Cytarabine
YNL203C
ZShift = 0.76
Z lm Score = −0.44
NG = 0
DB = 0
SM = 0
Delta_K
Cytarabine
YNL203C
ZShift = −0.06
Z lm Score = 0.36
NG = 0
DB = 0
SM = 0
Delta_r
Cytarabine
YNL203C
ZShift = −0.27
Z lm Score = −0.56
NG = 0
DB = 0
SM = 0
Delta_AUC
Cytarabine
0 10 25 50 100

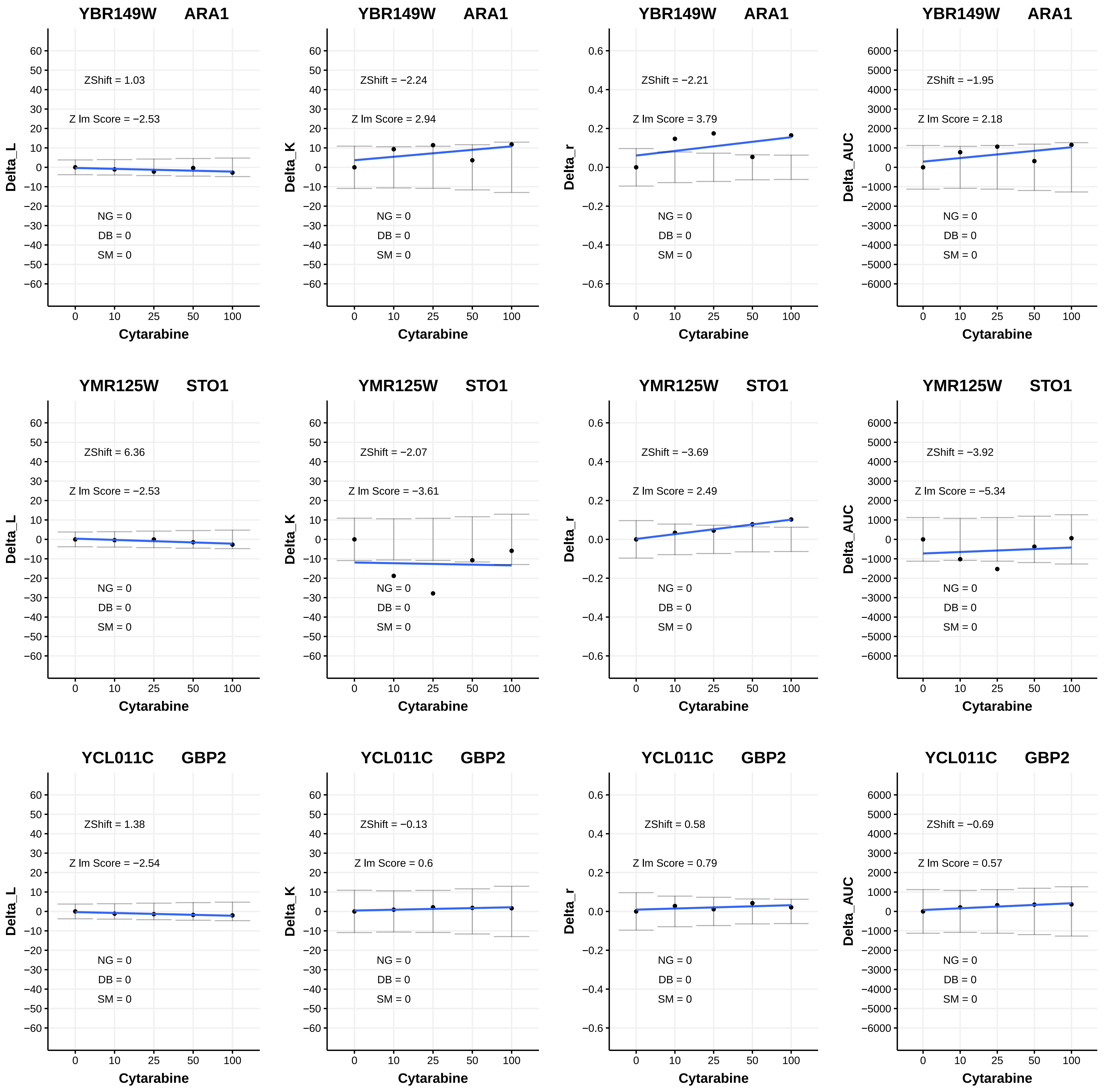
YBR149W ARA1
ZShift = 1.03
Z lm Score = −2.53
NG = 0
DB = 0
SM = 0
Delta_L
Cytarabine
YBR149W ARA1
ZShift = −2.24
Z lm Score = 2.94
NG = 0
DB = 0
SM = 0
Delta_K
Cytarabine
YBR149W ARA1
ZShift = −2.21
Z lm Score = 3.79
NG = 0
DB = 0
SM = 0
Delta_r
Cytarabine
YBR149W ARA1
ZShift = −1.95
Z lm Score = 2.18
NG = 0
DB = 0
SM = 0
Delta_AUC
Cytarabine
YMR125W STO1
ZShift = 6.36
Z lm Score = −2.53
NG = 0
DB = 0
SM = 0
Delta_L
Cytarabine
YMR125W STO1
ZShift = −2.07
Z lm Score = −3.61
NG = 0
DB = 0
SM = 0
Delta_K
Cytarabine
YMR125W STO1
ZShift = −3.69
Z lm Score = 2.49
NG = 0
DB = 0
SM = 0
Delta_r
Cytarabine
YMR125W STO1
ZShift = −3.92
Z lm Score = −5.34
NG = 0
DB = 0
SM = 0
Delta_AUC
Cytarabine
YCL011C GBP2
ZShift = 1.38
Z lm Score = −2.54
NG = 0
DB = 0
SM = 0
Delta_L
Cytarabine
YCL011C GBP2
ZShift = −0.13
Z lm Score = 0.6
NG = 0
DB = 0
SM = 0
Delta_K
Cytarabine
YCL011C GBP2
ZShift = 0.58
Z lm Score = 0.79
NG = 0
DB = 0
SM = 0
Delta_r
Cytarabine
YCL011C GBP2
ZShift = −0.69
Z lm Score = 0.57
NG = 0
DB = 0
SM = 0
Delta_AUC
Cytarabine

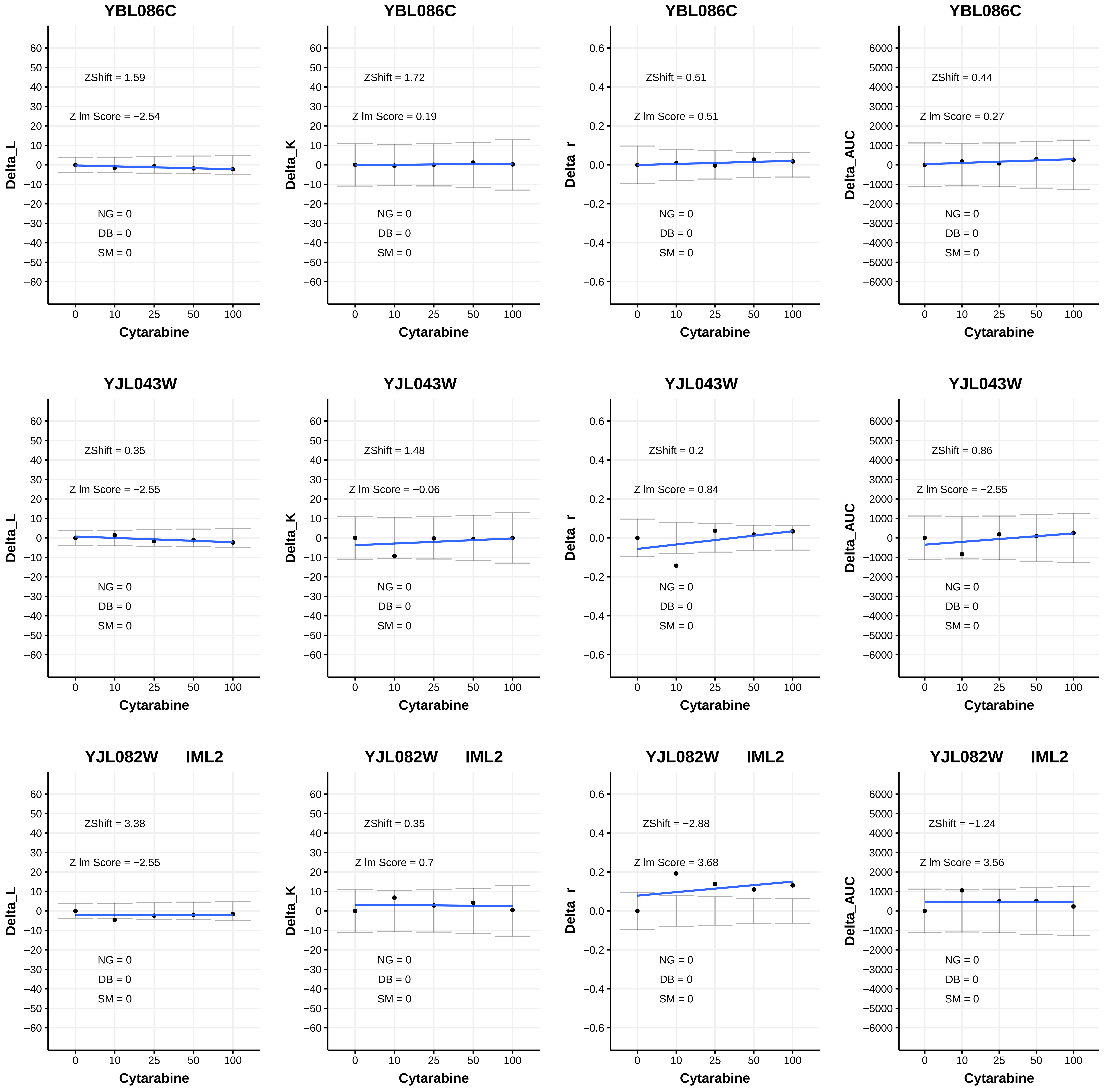
YBL086C
ZShift = 1.59
Z lm Score = −2.54
NG = 0
DB = 0
SM = 0
Delta_L
Cytarabine
YBL086C
ZShift = 1.72
Z lm Score = 0.19
NG = 0
DB = 0
SM = 0
Delta_K
Cytarabine
YBL086C
ZShift = 0.51
Z lm Score = 0.51
NG = 0
DB = 0
SM = 0
Delta_r
Cytarabine
YBL086C
ZShift = 0.44
Z lm Score = 0.27
NG = 0
DB = 0
SM = 0
Delta_AUC
Cytarabine
YJL043W
ZShift = 0.35
Z lm Score = −2.55
NG = 0
DB = 0
SM = 0
Delta_L
Cytarabine
YJL043W
ZShift = 1.48
Z lm Score = −0.06
NG = 0
DB = 0
SM = 0
Delta_K
Cytarabine
YJL043W
ZShift = 0.2
Z lm Score = 0.84
NG = 0
DB = 0
SM = 0
Delta_r
Cytarabine
YJL043W
ZShift = 0.86
Z lm Score = −2.55
NG = 0
DB = 0
SM = 0
Delta_AUC
Cytarabine
YJL082W IML2
ZShift = 3.38
Z lm Score = −2.55
NG = 0
DB = 0
SM = 0
Delta_L
Cytarabine
YJL082W IML2
ZShift = 0.35
Z lm Score = 0.7
NG = 0
DB = 0
SM = 0
Delta_K
Cytarabine
YJL082W IML2
ZShift = −2.88
Z lm Score = 3.68
NG = 0
DB = 0
SM = 0
Delta_r
Cytarabine
YJL082W IML2
ZShift = −1.24
Z lm Score = 3.56
NG = 0
DB = 0
SM = 0
Delta_AUC
Cytarabine

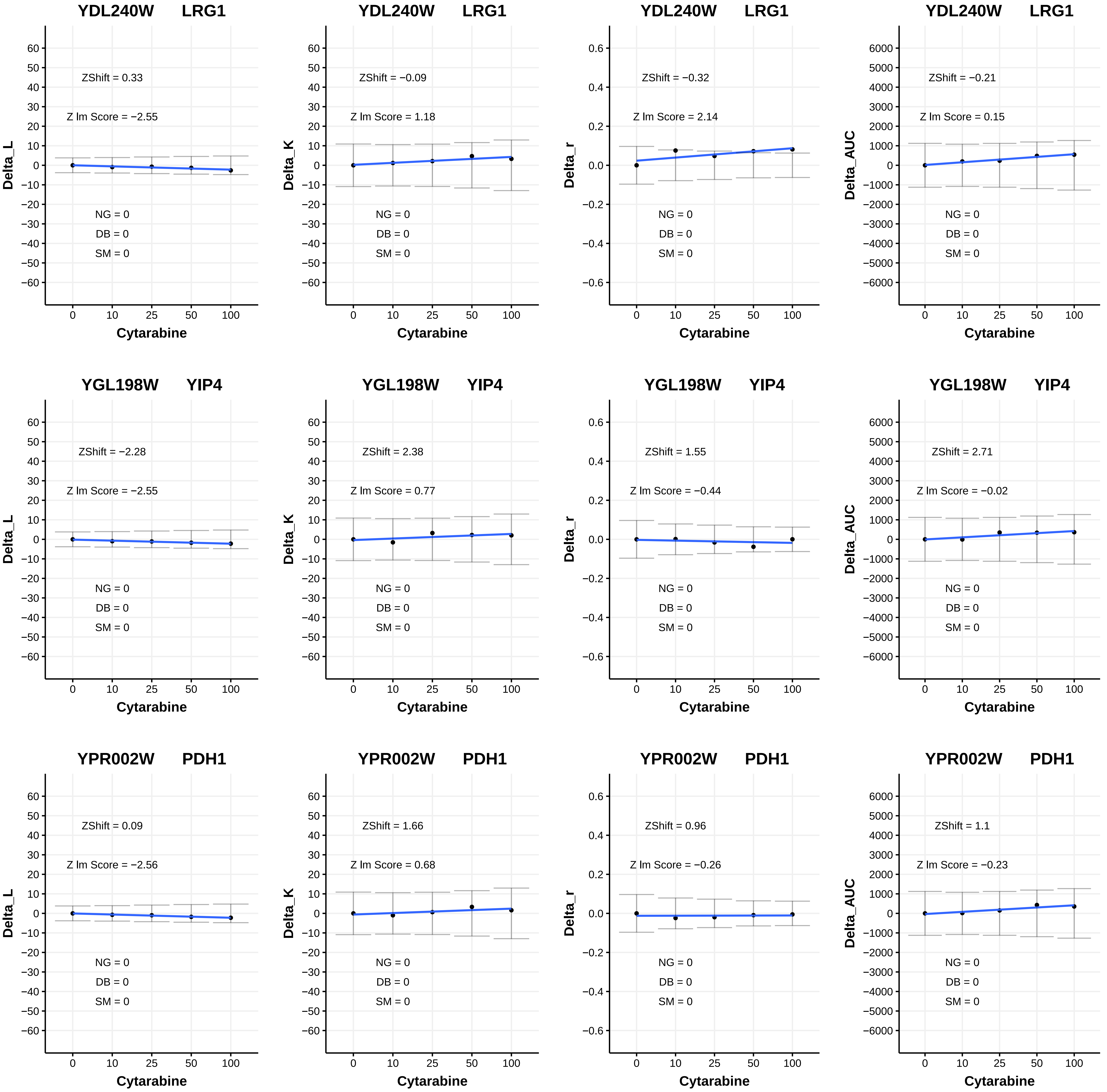

YDL240W LRG1
ZShift = 0.33
Z lm Score = −2.55
NG = 0
DB = 0
SM = 0
Delta_L
Cytarabine
YDL240W LRG1
ZShift = −0.09
Z lm Score = 1.18
NG = 0
DB = 0
SM = 0
Delta_K
Cytarabine
YDL240W LRG1
ZShift = −0.32
Z lm Score = 2.14
NG = 0
DB = 0
SM = 0
Delta_r
Cytarabine
YDL240W LRG1
ZShift = −0.21
Z lm Score = 0.15
NG = 0
DB = 0
SM = 0
Delta_AUC
Cytarabine
YGL198W YIP4
ZShift = −2.28
Z lm Score = −2.55
NG = 0
DB = 0
SM = 0
Delta_L
Cytarabine
YGL198W YIP4
ZShift = 2.38
Z lm Score = 0.77
NG = 0
DB = 0
SM = 0
Delta_K
Cytarabine
YGL198W YIP4
ZShift = 1.55
Z lm Score = −0.44
NG = 0
DB = 0
SM = 0
Delta_r
Cytarabine
YGL198W YIP4
ZShift = 2.71
Z lm Score = −0.02
NG = 0
DB = 0
SM = 0
Delta_AUC
Cytarabine
YPR002W PDH1
ZShift = 0.09
Z lm Score = −2.56
NG = 0
DB = 0
SM = 0
Delta_L
Cytarabine
YPR002W PDH1
ZShift = 1.66
Z lm Score = 0.68
NG = 0
DB = 0
SM = 0
Delta_K
Cytarabine
YPR002W PDH1
ZShift = 0.96
Z lm Score = −0.26
NG = 0
DB = 0
SM = 0
Delta_r
Cytarabine
YPR002W PDH1
ZShift = 1.1
Z lm Score = −0.23
NG = 0
DB = 0
SM = 0
Delta_AUC
Cytarabine

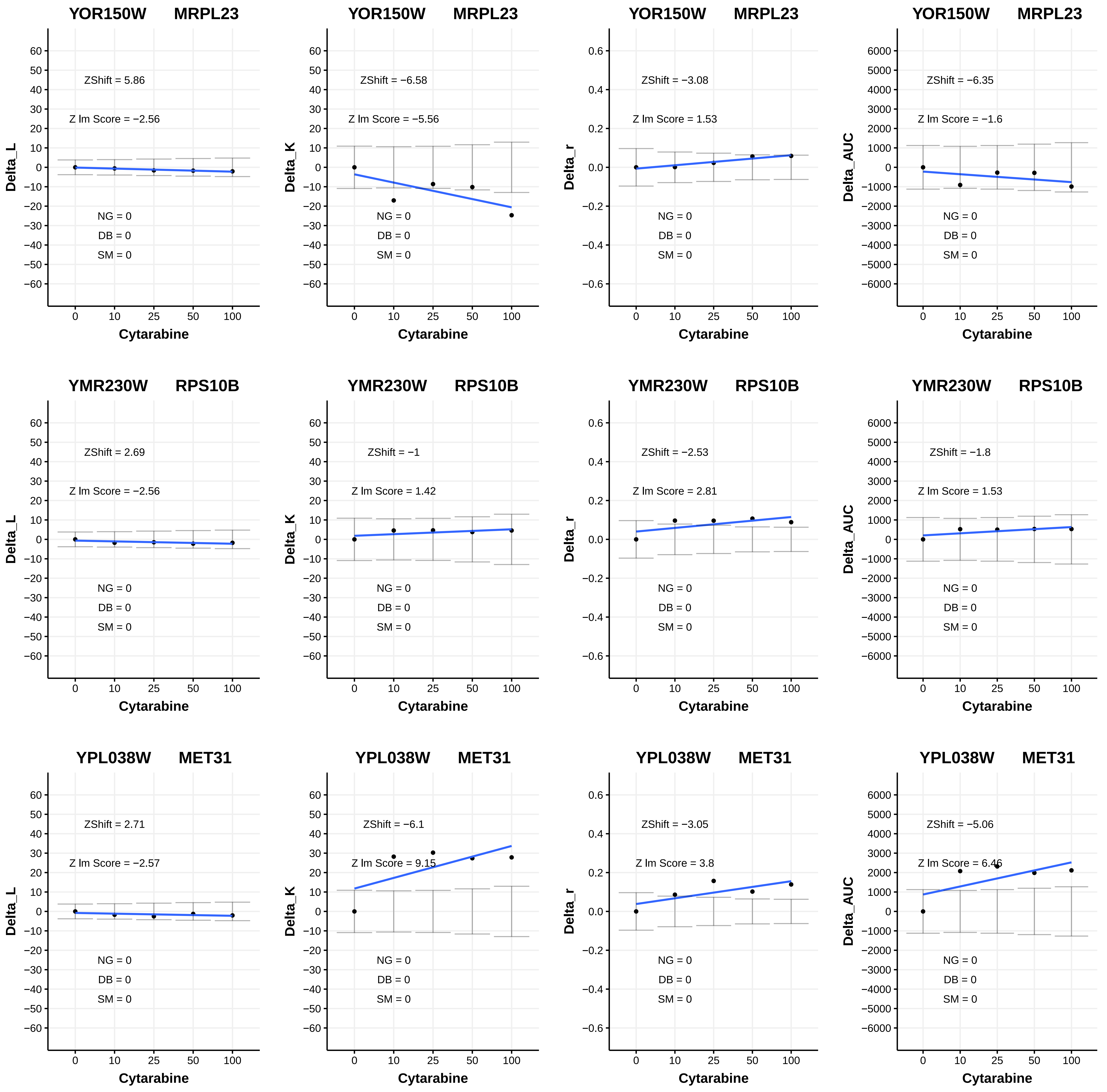

YOR150W MRPL23
ZShift = 5.86
Z lm Score = −2.56
NG = 0
DB = 0
SM = 0
Delta_L
Cytarabine
YOR150W MRPL23
ZShift = −6.58
Z lm Score = −5.56
NG = 0
DB = 0
SM = 0
Delta_K
Cytarabine
YOR150W MRPL23
ZShift = −3.08
Z lm Score = 1.53
NG = 0
DB = 0
SM = 0
Delta_r
Cytarabine
YOR150W MRPL23
ZShift = −6.35
Z lm Score = −1.6
NG = 0
DB = 0
SM = 0
Delta_AUC
Cytarabine
YMR230W RPS10B
ZShift = 2.69
Z lm Score = −2.56
NG = 0
DB = 0
SM = 0
Delta_L
Cytarabine
YMR230W RPS10B
ZShift = −1
Z lm Score = 1.42
NG = 0
DB = 0
SM = 0
Delta_K
Cytarabine
YMR230W RPS10B
ZShift = −2.53
Z lm Score = 2.81
NG = 0
DB = 0
SM = 0
Delta_r
Cytarabine
YMR230W RPS10B
ZShift = −1.8
Z lm Score = 1.53
NG = 0
DB = 0
SM = 0
Delta_AUC
Cytarabine
YPL038W MET31
ZShift = 2.71
Z lm Score = −2.57
NG = 0
DB = 0
SM = 0
Delta_L
Cytarabine
YPL038W MET31
ZShift = −6.1
Z lm Score = 9.15
NG = 0
DB = 0
SM = 0
Delta_K
Cytarabine
YPL038W MET31
ZShift = −3.05
Z lm Score = 3.8
NG = 0
DB = 0
SM = 0
Delta_r
Cytarabine
YPL038W MET31
ZShift = −5.06
Z lm Score = 6.46
NG = 0
DB = 0
SM = 0
Delta_AUC
Cytarabine

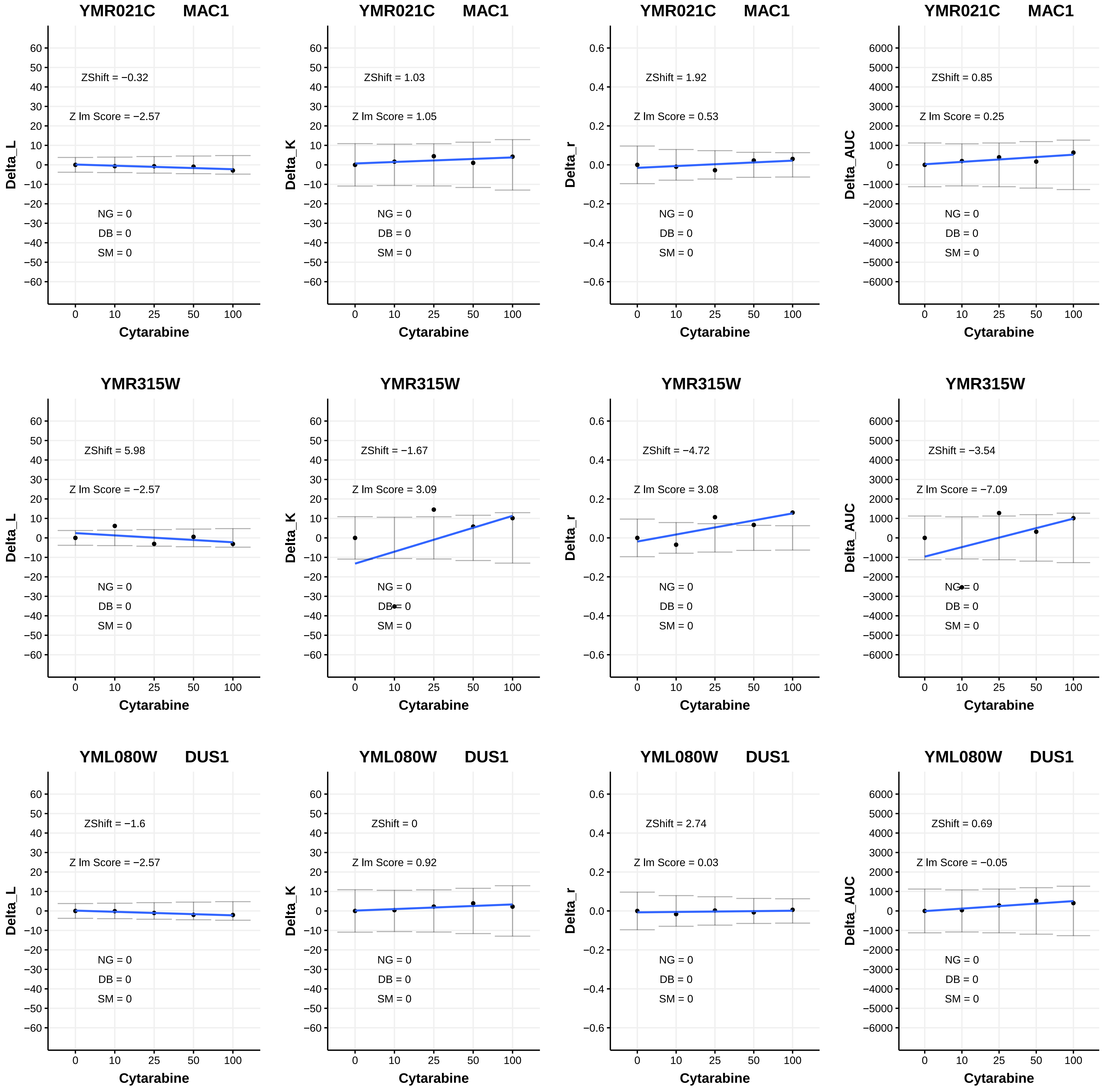

YMR021C MAC1
ZShift = −0.32
Z lm Score = −2.57
NG = 0
DB = 0
SM = 0
Delta_L
Cytarabine
YMR021C MAC1
ZShift = 1.03
Z lm Score = 1.05
NG = 0
DB = 0
SM = 0
Delta_K
Cytarabine
YMR021C MAC1
ZShift = 1.92
Z lm Score = 0.53
NG = 0
DB = 0
SM = 0
Delta_r
Cytarabine
YMR021C MAC1
ZShift = 0.85
Z lm Score = 0.25
NG = 0
DB = 0
SM = 0
Delta_AUC
Cytarabine
YMR315W
ZShift = 5.98
Z lm Score = −2.57
NG = 0
DB = 0
SM = 0
Delta_L
Cytarabine
YMR315W
ZShift = −1.67
Z lm Score = 3.09
NG = 0
DB = 0
SM = 0
Delta_K
Cytarabine
YMR315W
ZShift = −4.72
Z lm Score = 3.08
NG = 0
DB = 0
SM = 0
Delta_r
Cytarabine
YMR315W
ZShift = −3.54
Z lm Score = −7.09
NG = 0
DB = 0
SM = 0
Delta_AUC
Cytarabine
YML080W DUS1
ZShift = −1.6
Z lm Score = −2.57
NG = 0
DB = 0
SM = 0
Delta_L
Cytarabine
YML080W DUS1
ZShift = 0
Z lm Score = 0.92
NG = 0
DB = 0
SM = 0
Delta_K
Cytarabine
YML080W DUS1
ZShift = 2.74
Z lm Score = 0.03
NG = 0
DB = 0
SM = 0
Delta_r
Cytarabine
YML080W DUS1
ZShift = 0.69
Z lm Score = −0.05
NG = 0
DB = 0
SM = 0
Delta_AUC
Cytarabine

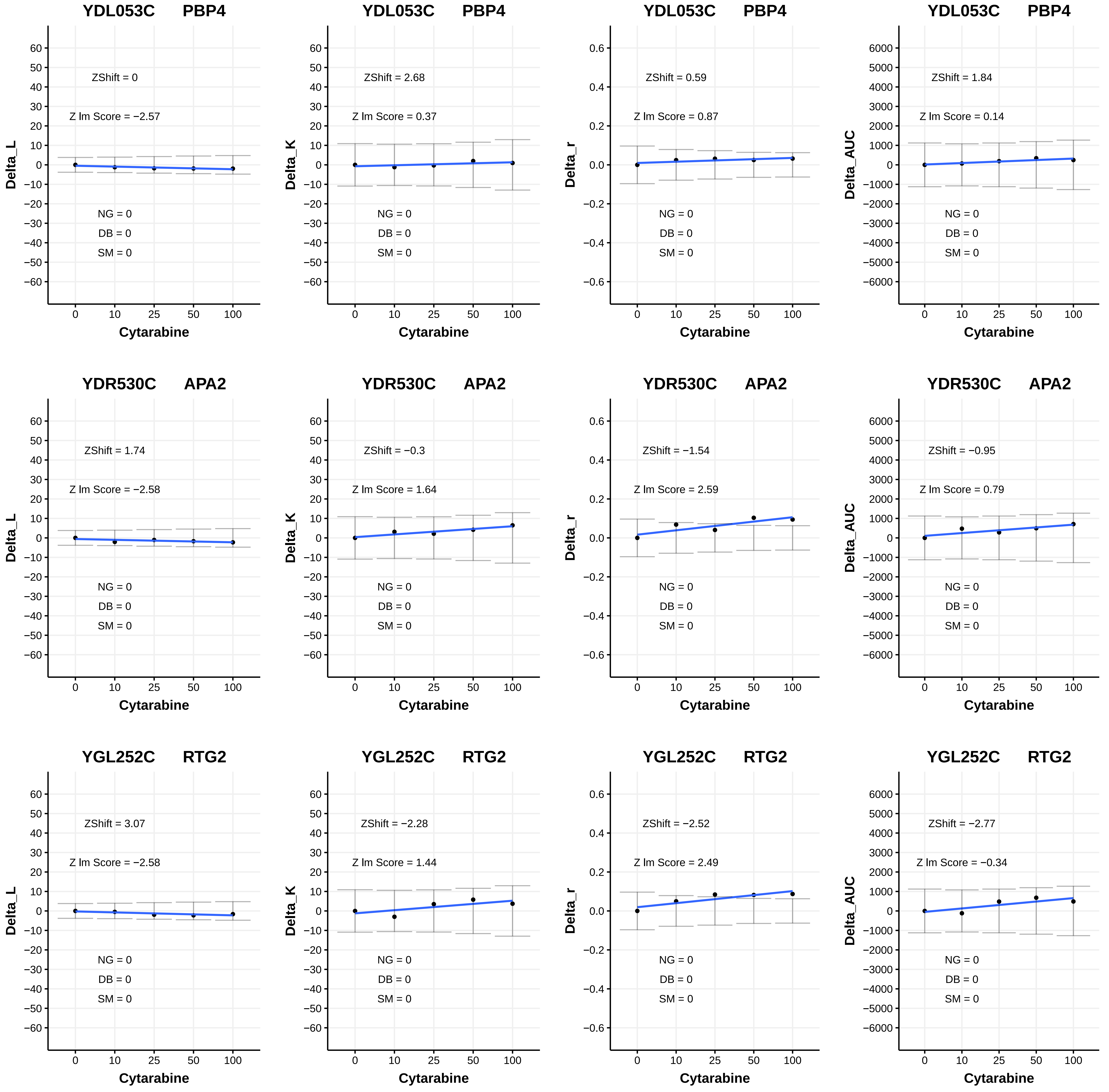

YDL053C PBP4
ZShift = 0
Z lm Score = −2.57
NG = 0
DB = 0
SM = 0
Delta_L
Cytarabine
YDL053C PBP4
ZShift = 2.68
Z lm Score = 0.37
NG = 0
DB = 0
SM = 0
Delta_K
Cytarabine
YDL053C PBP4
ZShift = 0.59
Z lm Score = 0.87
NG = 0
DB = 0
SM = 0
Delta_r
Cytarabine
YDL053C PBP4
ZShift = 1.84
Z lm Score = 0.14
NG = 0
DB = 0
SM = 0
Delta_AUC
Cytarabine
YDR530C APA2
ZShift = 1.74
Z lm Score = −2.58
NG = 0
DB = 0
SM = 0
Delta_L
Cytarabine
YDR530C APA2
ZShift = −0.3
Z lm Score = 1.64
NG = 0
DB = 0
SM = 0
Delta_K
Cytarabine
YDR530C APA2
ZShift = −1.54
Z lm Score = 2.59
NG = 0
DB = 0
SM = 0
Delta_r
Cytarabine
YDR530C APA2
ZShift = −0.95
Z lm Score = 0.79
NG = 0
DB = 0
SM = 0
Delta_AUC
Cytarabine
YGL252C RTG2
ZShift = 3.07
Z lm Score = −2.58
NG = 0
DB = 0
SM = 0
Delta_L
Cytarabine
YGL252C RTG2
ZShift = −2.28
Z lm Score = 1.44
NG = 0
DB = 0
SM = 0
Delta_K
Cytarabine
YGL252C RTG2
ZShift = −2.52
Z lm Score = 2.49
NG = 0
DB = 0
SM = 0
Delta_r
Cytarabine
YGL252C RTG2
ZShift = −2.77
Z lm Score = −0.34
NG = 0
DB = 0
SM = 0
Delta_AUC
Cytarabine

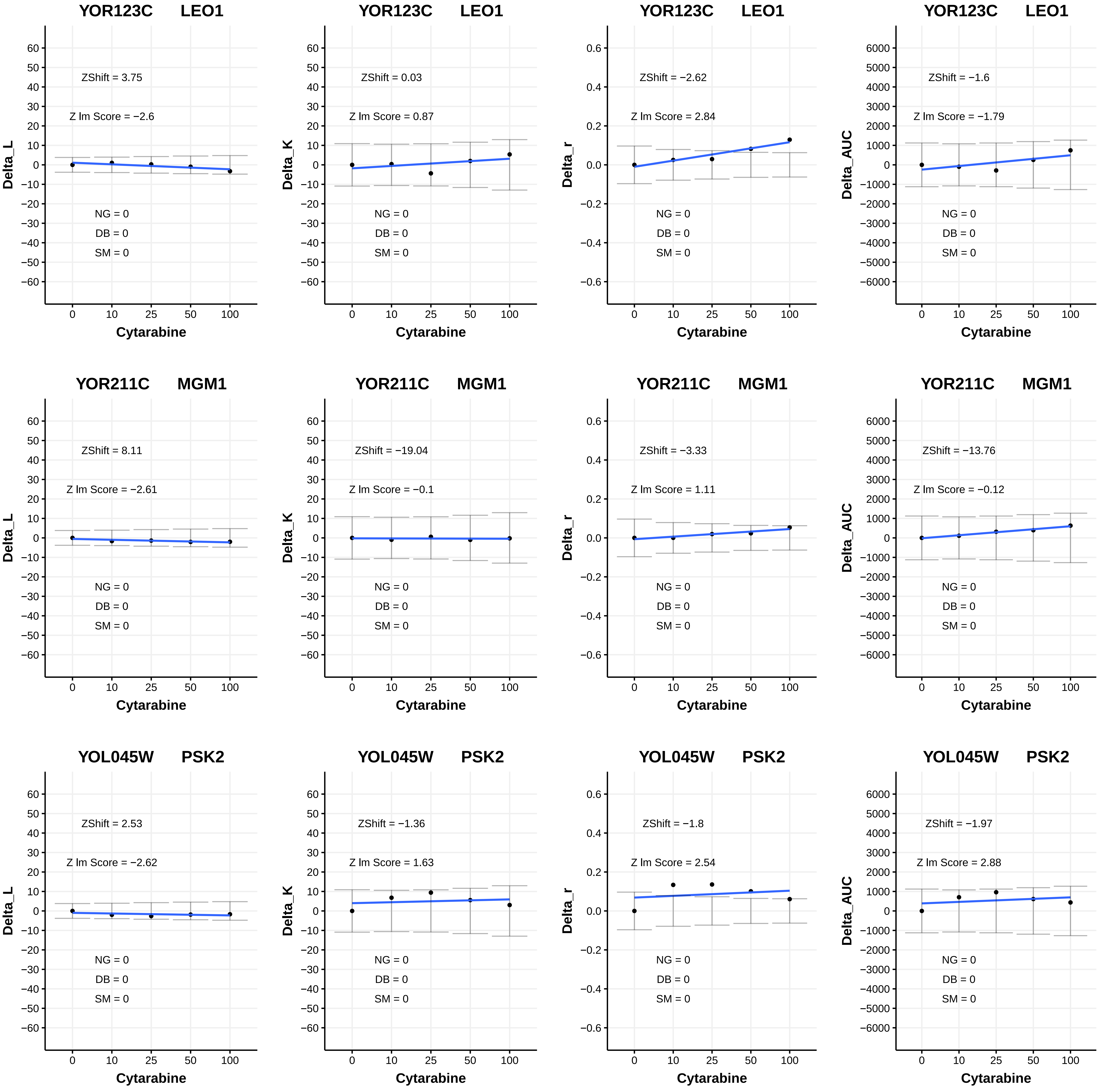

YOR123C LEO1
Delta_L
ZShift = 3.75
Z lm Score = −2.6
NG = 0
DB = 0
SM = 0
Cytarabine
YOR123C LEO1
Delta_K
ZShift = 0.03
Z lm Score = 0.87
NG = 0
DB = 0
SM = 0
Cytarabine
YOR123C LEO1
Delta_r
ZShift = −2.62
Z lm Score = 2.84
NG = 0
DB = 0
SM = 0
Cytarabine
YOR123C LEO1
Delta_AUC
ZShift = −1.6
Z lm Score = −1.79
NG = 0
DB = 0
SM = 0
Cytarabine
YOR211C MGM1
Delta_L
ZShift = 8.11
Z lm Score = −2.61
NG = 0
DB = 0
SM = 0
Cytarabine
YOR211C MGM1
Delta_K
ZShift = −19.04
Z lm Score = −0.1
NG = 0
DB = 0
SM = 0
Cytarabine
YOR211C MGM1
Delta_r
ZShift = −3.33
Z lm Score = 1.11
NG = 0
DB = 0
SM = 0
Cytarabine
YOR211C MGM1
Delta_AUC
ZShift = −13.76
Z lm Score = −0.12
NG = 0
DB = 0
SM = 0
Cytarabine
YOL045W PSK2
Delta_L
ZShift = 2.53
Z lm Score = −2.62
NG = 0
DB = 0
SM = 0
Cytarabine
YOL045W PSK2
Delta_K
ZShift = −1.36
Z lm Score = 1.63
NG = 0
DB = 0
SM = 0
Cytarabine
YOL045W PSK2
Delta_r
ZShift = −1.8
Z lm Score = 2.54
NG = 0
DB = 0
SM = 0
Cytarabine
YOL045W PSK2
Delta_AUC
ZShift = −1.97
Z lm Score = 2.88
NG = 0
DB = 0
SM = 0
Cytarabine

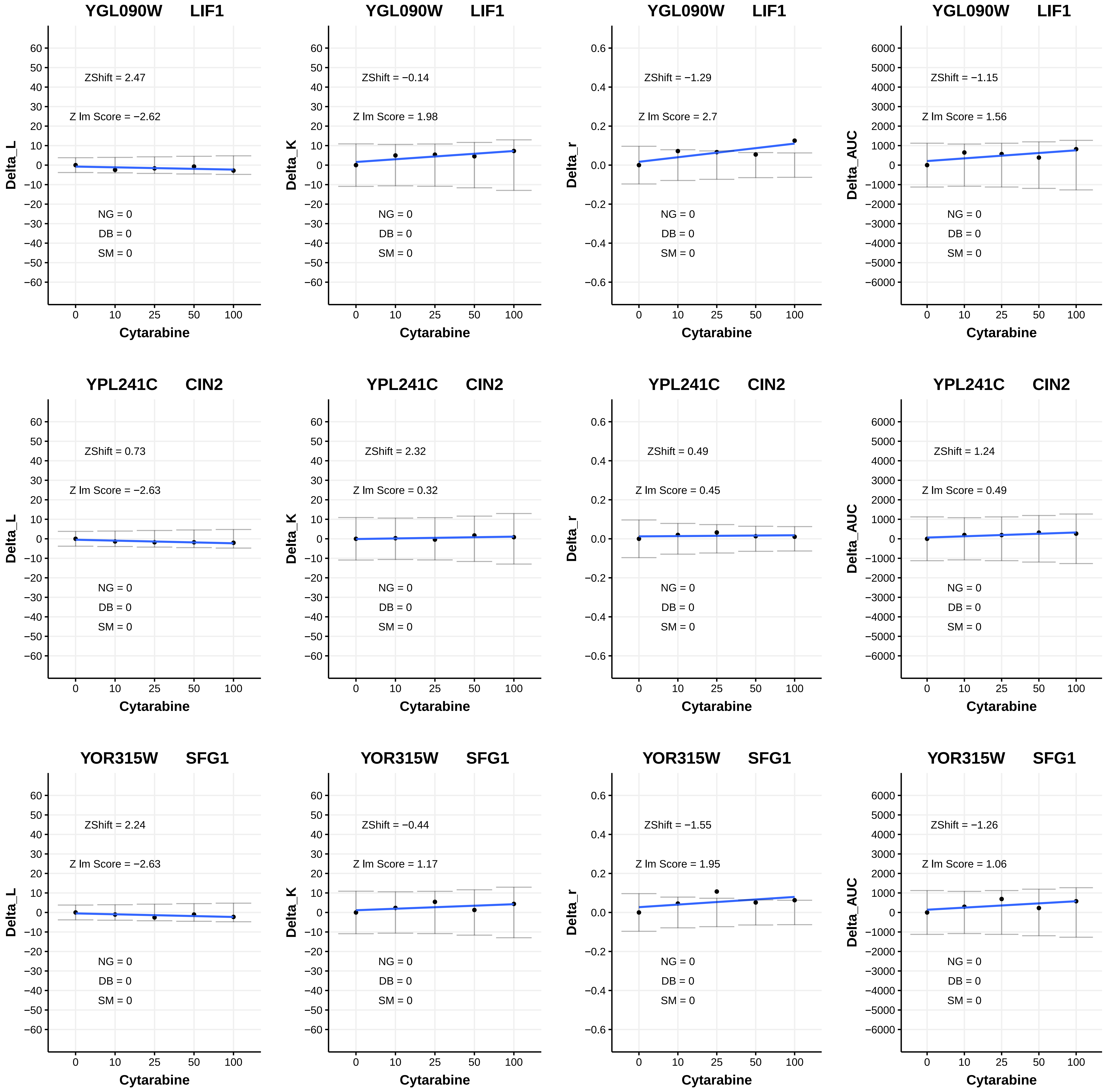

YGL090W LIF1
ZShift = 2.47
Z lm Score = −2.62
NG = 0
DB = 0
SM = 0
Delta_L
Cytarabine
YGL090W LIF1
ZShift = −0.14
Z lm Score = 1.98
NG = 0
DB = 0
SM = 0
Delta_K
Cytarabine
YGL090W LIF1
ZShift = −1.29
Z lm Score = 2.7
NG = 0
DB = 0
SM = 0
Delta_r
Cytarabine
YGL090W LIF1
ZShift = −1.15
Z lm Score = 1.56
NG = 0
DB = 0
SM = 0
Delta_AUC
Cytarabine
YPL241C CIN2
ZShift = 0.73
Z lm Score = −2.63
NG = 0
DB = 0
SM = 0
Delta_L
Cytarabine
YPL241C CIN2
ZShift = 2.32
Z lm Score = 0.32
NG = 0
DB = 0
SM = 0
Delta_K
Cytarabine
YPL241C CIN2
ZShift = 0.49
Z lm Score = 0.45
NG = 0
DB = 0
SM = 0
Delta_r
Cytarabine
YPL241C CIN2
ZShift = 1.24
Z lm Score = 0.49
NG = 0
DB = 0
SM = 0
Delta_AUC
Cytarabine
YOR315W SFG1
ZShift = 2.24
Z lm Score = −2.63
NG = 0
DB = 0
SM = 0
Delta_L
Cytarabine
YOR315W SFG1
ZShift = −0.44
Z lm Score = 1.17
NG = 0
DB = 0
SM = 0
Delta_K
Cytarabine
YOR315W SFG1
ZShift = −1.55
Z lm Score = 1.95
NG = 0
DB = 0
SM = 0
Delta_r
Cytarabine
YOR315W SFG1
ZShift = −1.26
Z lm Score = 1.06
NG = 0
DB = 0
SM = 0
Delta_AUC
Cytarabine

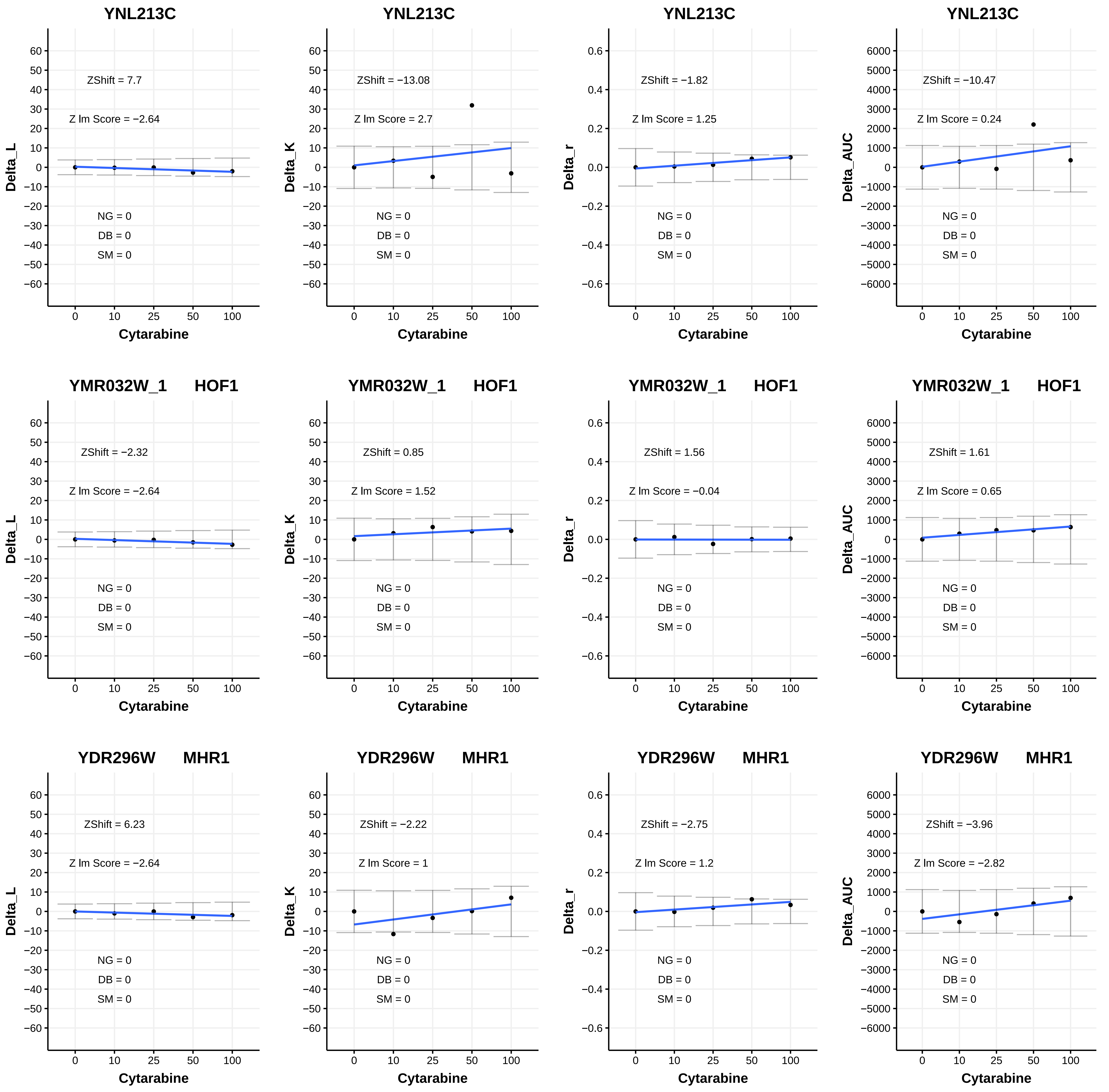
YNL213C
ZShift = 7.7
Z lm Score = −2.64
NG = 0
DB = 0
SM = 0
Delta_L
Cytarabine
YNL213C
ZShift = −13.08
Z lm Score = 2.7
NG = 0
DB = 0
SM = 0
Delta_K
Cytarabine
YNL213C
ZShift = −1.82
Z lm Score = 1.25
NG = 0
DB = 0
SM = 0
Delta_r
Cytarabine
YNL213C
ZShift = −10.47
Z lm Score = 0.24
NG = 0
DB = 0
SM = 0
Delta_AUC
Cytarabine
YMR032W_1 HOF1
ZShift = −2.32
Z lm Score = −2.64
NG = 0
DB = 0
SM = 0
Delta_L
Cytarabine
YMR032W_1 HOF1
ZShift = 0.85
Z lm Score = 1.52
NG = 0
DB = 0
SM = 0
Delta_K
Cytarabine
YMR032W_1 HOF1
ZShift = 1.56
Z lm Score = −0.04
NG = 0
DB = 0
SM = 0
Delta_r
Cytarabine
YMR032W_1 HOF1
ZShift = 1.61
Z lm Score = 0.65
NG = 0
DB = 0
SM = 0
Delta_AUC
Cytarabine
YDR296W MHR1
ZShift = 6.23
Z lm Score = −2.64
NG = 0
DB = 0
SM = 0
Delta_L
Cytarabine
YDR296W MHR1
ZShift = −2.22
Z lm Score = 1
NG = 0
DB = 0
SM = 0
Delta_K
Cytarabine
YDR296W MHR1
ZShift = −2.75
Z lm Score = 1.2
NG = 0
DB = 0
SM = 0
Delta_r
Cytarabine
YDR296W MHR1
ZShift = −3.96
Z lm Score = −2.82
NG = 0
DB = 0
SM = 0
Delta_AUC
Cytarabine

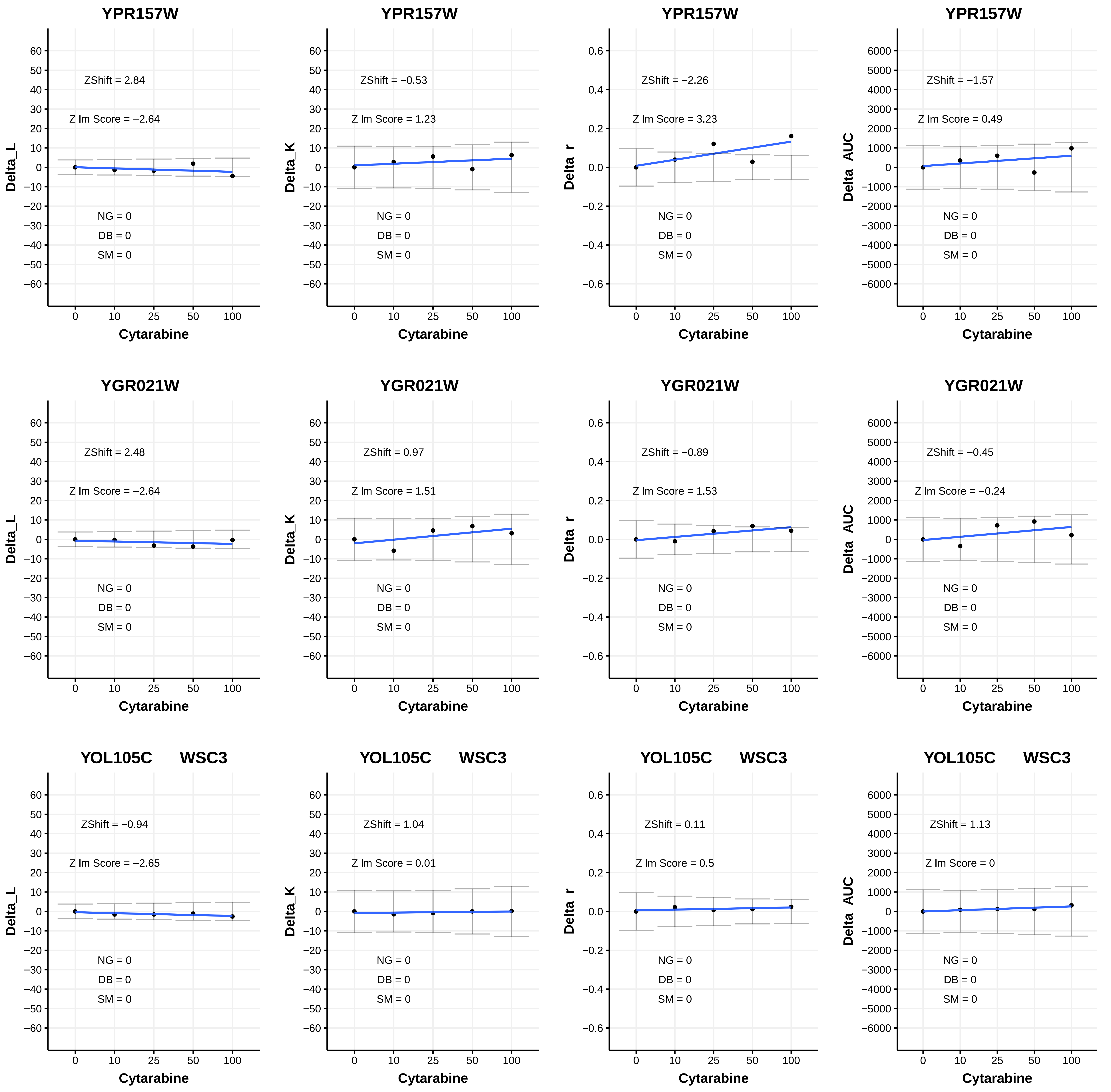

YPR157W
ZShift = 2.84
Z lm Score = −2.64
NG = 0
DB = 0
SM = 0
Delta_L
Cytarabine
YPR157W
ZShift = −0.53
Z lm Score = 1.23
NG = 0
DB = 0
SM = 0
Delta_K
Cytarabine
YPR157W
ZShift = −2.26
Z lm Score = 3.23
NG = 0
DB = 0
SM = 0
Delta_r
Cytarabine
YPR157W
ZShift = −1.57
Z lm Score = 0.49
NG = 0
DB = 0
SM = 0
Delta_AUC
Cytarabine
YGR021W
ZShift = 2.48
Z lm Score = −2.64
NG = 0
DB = 0
SM = 0
Delta_L
Cytarabine
YGR021W
ZShift = 0.97
Z lm Score = 1.51
NG = 0
DB = 0
SM = 0
Delta_K
Cytarabine
YGR021W
ZShift = −0.89
Z lm Score = 1.53
NG = 0
DB = 0
SM = 0
Delta_r
Cytarabine
YGR021W
ZShift = −0.45
Z lm Score = −0.24
NG = 0
DB = 0
SM = 0
Delta_AUC
Cytarabine
YOL105C WSC3
ZShift = −0.94
Z lm Score = −2.65
NG = 0
DB = 0
SM = 0
Delta_L
Cytarabine
YOL105C WSC3
ZShift = 1.04
Z lm Score = 0.01
NG = 0
DB = 0
SM = 0
Delta_K
Cytarabine
YOL105C WSC3
ZShift = 0.11
Z lm Score = 0.5
NG = 0
DB = 0
SM = 0
Delta_r
Cytarabine
YOL105C WSC3
ZShift = 1.13
Z lm Score = 0
NG = 0
DB = 0
SM = 0
Delta_AUC
Cytarabine

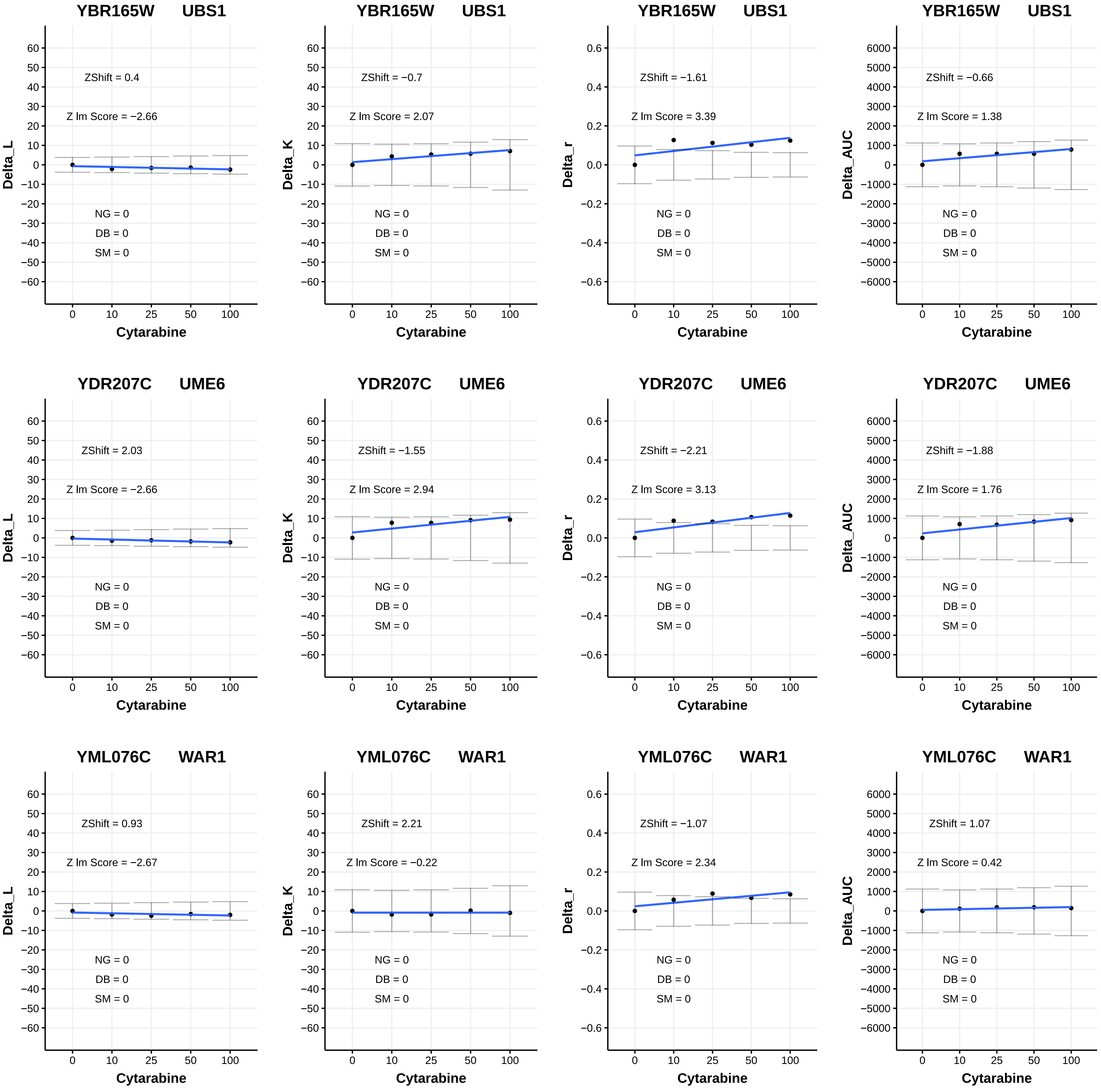

YBR165W UBS1
Delta_L
ZShift = 0.4
Z lm Score = −2.66
NG = 0
DB = 0
SM = 0
Cytarabine
YBR165W UBS1
Delta_K
ZShift = −0.7
Z lm Score = 2.07
NG = 0
DB = 0
SM = 0
Cytarabine
YBR165W UBS1
Delta_r
ZShift = −1.61
Z lm Score = 3.39
NG = 0
DB = 0
SM = 0
Cytarabine
YBR165W UBS1
Delta_AUC
ZShift = −0.66
Z lm Score = 1.38
NG = 0
DB = 0
SM = 0
Cytarabine
YDR207C UME6
Delta_L
ZShift = 2.03
Z lm Score = −2.66
NG = 0
DB = 0
SM = 0
Cytarabine
YDR207C UME6
Delta_K
ZShift = −1.55
Z lm Score = 2.94
NG = 0
DB = 0
SM = 0
Cytarabine
YDR207C UME6
Delta_r
ZShift = −2.21
Z lm Score = 3.13
NG = 0
DB = 0
SM = 0
Cytarabine
YDR207C UME6
Delta_AUC
ZShift = −1.88
Z lm Score = 1.76
NG = 0
DB = 0
SM = 0
Cytarabine
YML076C WAR1
Delta_L
ZShift = 0.93
Z lm Score = −2.67
NG = 0
DB = 0
SM = 0
Cytarabine
YML076C WAR1
Delta_K
ZShift = 2.21
Z lm Score = −0.22
NG = 0
DB = 0
SM = 0
Cytarabine
YML076C WAR1
Delta_r
ZShift = −1.07
Z lm Score = 2.34
NG = 0
DB = 0
SM = 0
Cytarabine
YML076C WAR1
Delta_AUC
ZShift = 1.07
Z lm Score = 0.42
NG = 0
DB = 0
SM = 0
Cytarabine

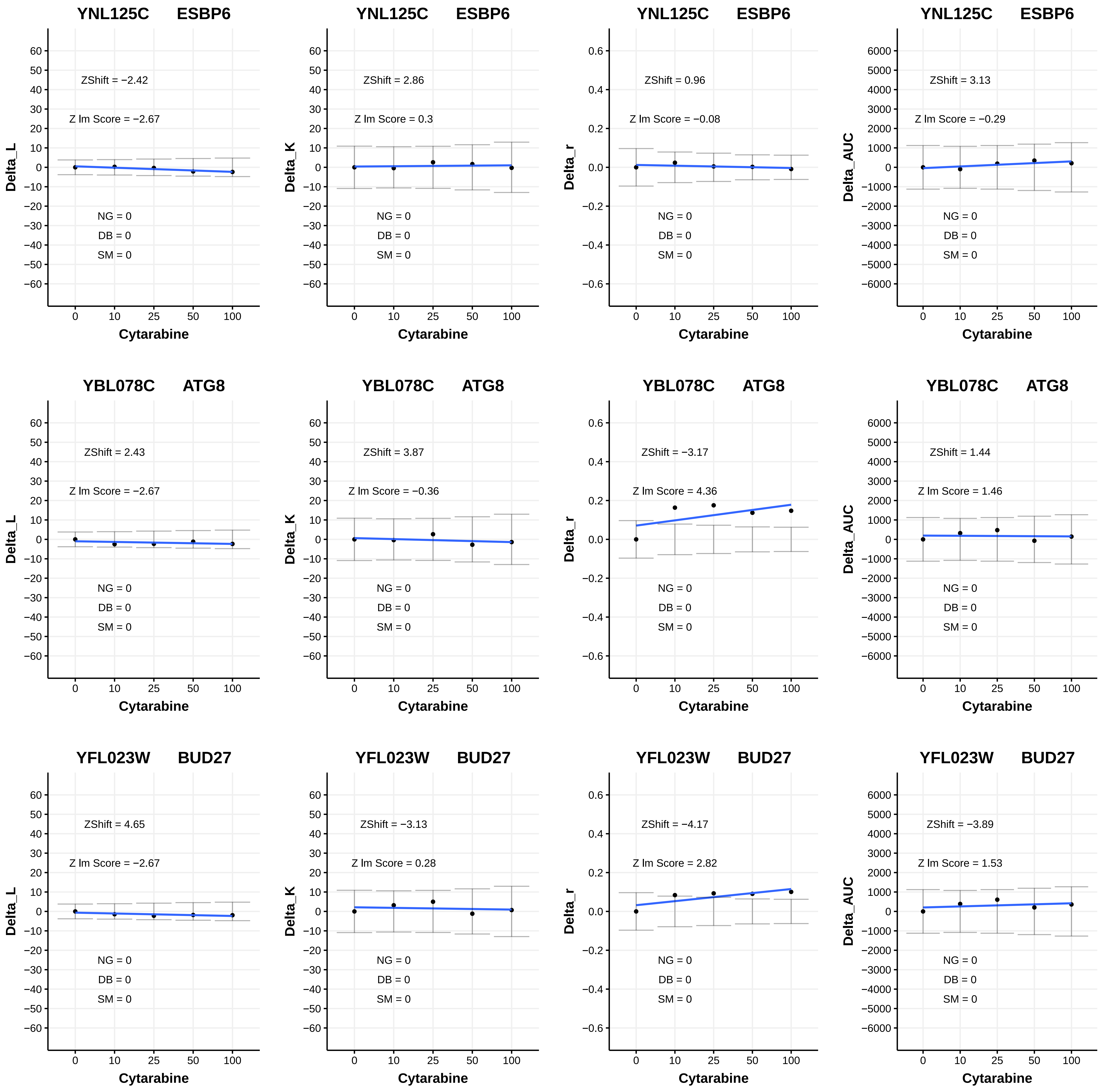
YNL125C ESBP6
ZShift = −2.42
Z lm Score = −2.67
NG = 0
DB = 0
SM = 0
Delta_L
Cytarabine
YNL125C ESBP6
ZShift = 2.86
Z lm Score = 0.3
NG = 0
DB = 0
SM = 0
Delta_K
Cytarabine
YNL125C ESBP6
ZShift = 0.96
Z lm Score = −0.08
NG = 0
DB = 0
SM = 0
Delta_r
Cytarabine
YNL125C ESBP6
ZShift = 3.13
Z lm Score = −0.29
NG = 0
DB = 0
SM = 0
Delta_AUC
Cytarabine
YBL078C ATG8
ZShift = 2.43
Z lm Score = −2.67
NG = 0
DB = 0
SM = 0
Delta_L
Cytarabine
YBL078C ATG8
ZShift = 3.87
Z lm Score = −0.36
NG = 0
DB = 0
SM = 0
Delta_K
Cytarabine
YBL078C ATG8
ZShift = −3.17
Z lm Score = 4.36
NG = 0
DB = 0
SM = 0
Delta_r
Cytarabine
YBL078C ATG8
ZShift = 1.44
Z lm Score = 1.46
NG = 0
DB = 0
SM = 0
Delta_AUC
Cytarabine
YFL023W BUD27
ZShift = 4.65
Z lm Score = −2.67
NG = 0
DB = 0
SM = 0
Delta_L
Cytarabine
YFL023W BUD27
ZShift = −3.13
Z lm Score = 0.28
NG = 0
DB = 0
SM = 0
Delta_K
Cytarabine
YFL023W BUD27
ZShift = −4.17
Z lm Score = 2.82
NG = 0
DB = 0
SM = 0
Delta_r
Cytarabine
YFL023W BUD27
ZShift = −3.89
Z lm Score = 1.53
NG = 0
DB = 0
SM = 0
Delta_AUC
Cytarabine

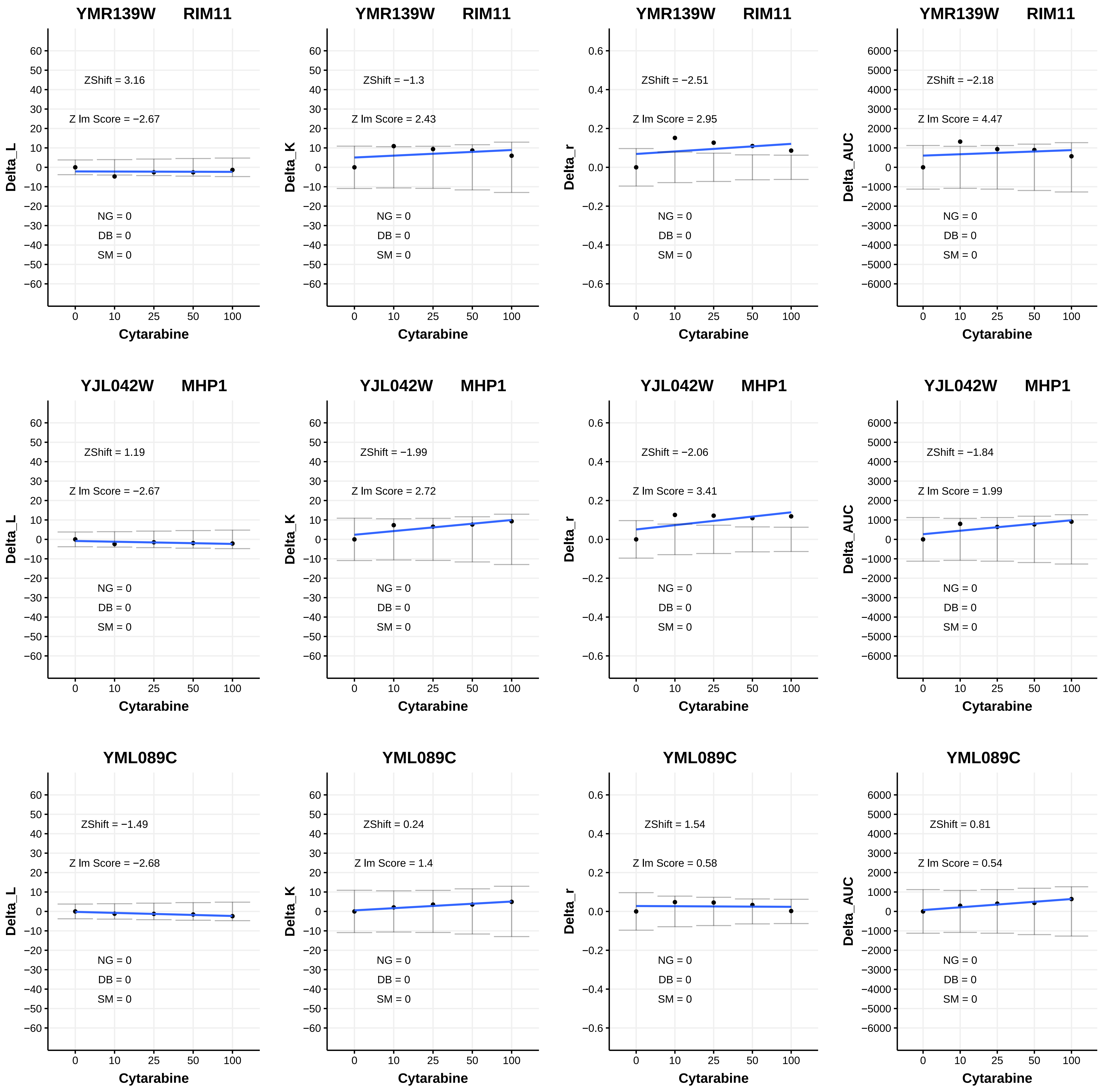

YMR139W RIM11
ZShift = 3.16
Z lm Score = −2.67
NG = 0
DB = 0
SM = 0
Delta_L
Cytarabine
YMR139W RIM11
ZShift = −1.3
Z lm Score = 2.43
NG = 0
DB = 0
SM = 0
Delta_K
Cytarabine
YMR139W RIM11
ZShift = −2.51
Z lm Score = 2.95
NG = 0
DB = 0
SM = 0
Delta_r
Cytarabine
YMR139W RIM11
ZShift = −2.18
Z lm Score = 4.47
NG = 0
DB = 0
SM = 0
Delta_AUC
Cytarabine
YJL042W MHP1
ZShift = 1.19
Z lm Score = −2.67
NG = 0
DB = 0
SM = 0
Delta_L
Cytarabine
YJL042W MHP1
ZShift = −1.99
Z lm Score = 2.72
NG = 0
DB = 0
SM = 0
Delta_K
Cytarabine
YJL042W MHP1
ZShift = −2.06
Z lm Score = 3.41
NG = 0
DB = 0
SM = 0
Delta_r
Cytarabine
YJL042W MHP1
ZShift = −1.84
Z lm Score = 1.99
NG = 0
DB = 0
SM = 0
Delta_AUC
Cytarabine
YML089C
ZShift = −1.49
Z lm Score = −2.68
NG = 0
DB = 0
SM = 0
Delta_L
Cytarabine
YML089C
ZShift = 0.24
Z lm Score = 1.4
NG = 0
DB = 0
SM = 0
Delta_K
Cytarabine
YML089C
ZShift = 1.54
Z lm Score = 0.58
NG = 0
DB = 0
SM = 0
Delta_r
Cytarabine
YML089C
ZShift = 0.81
Z lm Score = 0.54
NG = 0
DB = 0
SM = 0
Delta_AUC
Cytarabine

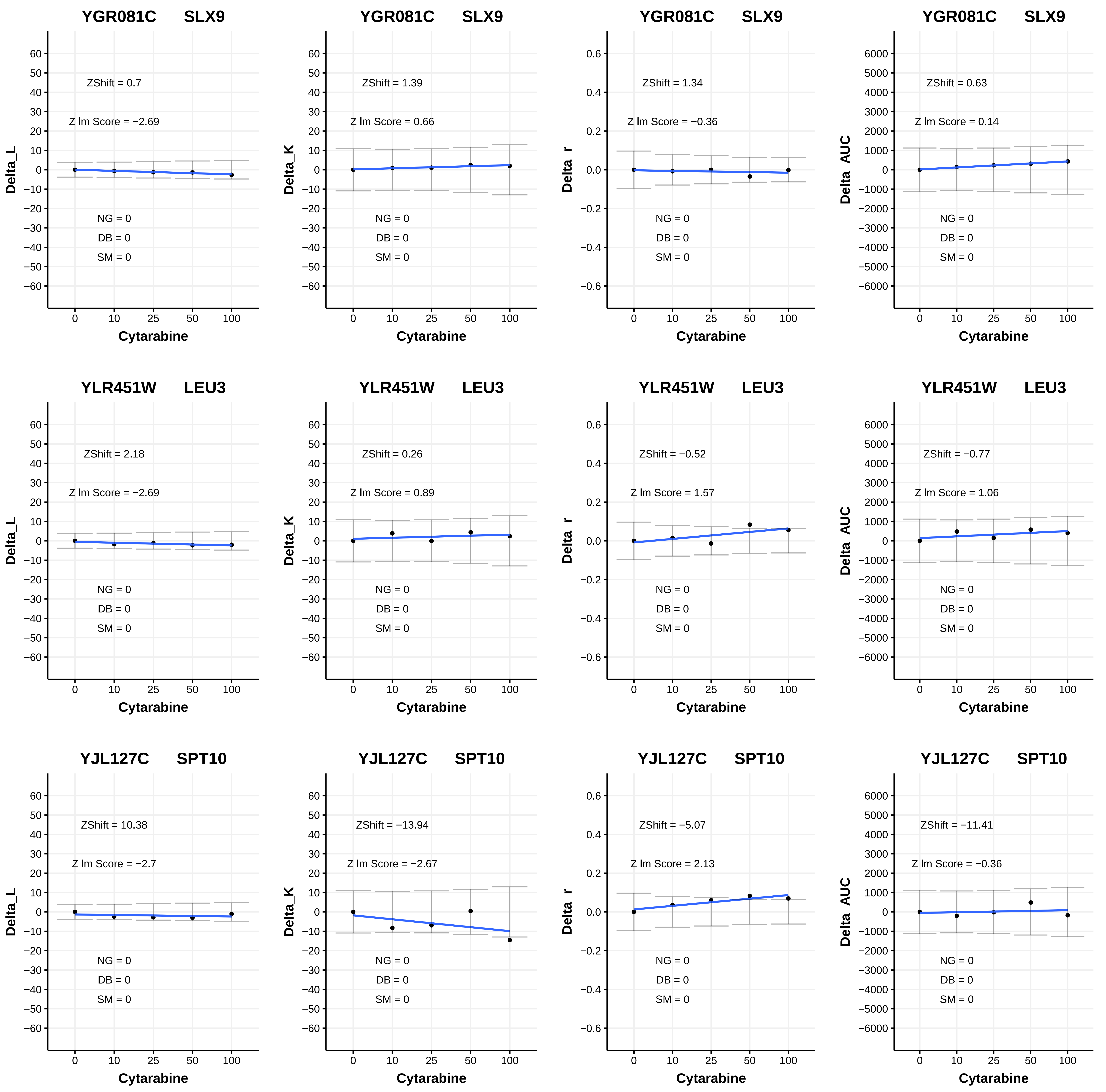
YGR081C SLX9
ZShift = 0.7
Z lm Score = −2.69
NG = 0
DB = 0
SM = 0
Delta_L
Cytarabine
YGR081C SLX9
ZShift = 1.39
Z lm Score = 0.66
NG = 0
DB = 0
SM = 0
Delta_K
Cytarabine
YGR081C SLX9
ZShift = 1.34
Z lm Score = −0.36
NG = 0
DB = 0
SM = 0
Delta_r
Cytarabine
YGR081C SLX9
ZShift = 0.63
Z lm Score = 0.14
NG = 0
DB = 0
SM = 0
Delta_AUC
Cytarabine
YLR451W LEU3
ZShift = 2.18
Z lm Score = −2.69
NG = 0
DB = 0
SM = 0
Delta_L
Cytarabine
YLR451W LEU3
ZShift = 0.26
Z lm Score = 0.89
NG = 0
DB = 0
SM = 0
Delta_K
Cytarabine
YLR451W LEU3
ZShift = −0.52
Z lm Score = 1.57
NG = 0
DB = 0
SM = 0
Delta_r
Cytarabine
YLR451W LEU3
ZShift = −0.77
Z lm Score = 1.06
NG = 0
DB = 0
SM = 0
Delta_AUC
Cytarabine
YJL127C SPT10
ZShift = 10.38
Z lm Score = −2.7
NG = 0
DB = 0
SM = 0
Delta_L
Cytarabine
YJL127C SPT10
ZShift = −13.94
Z lm Score = −2.67
NG = 0
DB = 0
SM = 0
Delta_K
Cytarabine
YJL127C SPT10
ZShift = −5.07
Z lm Score = 2.13
NG = 0
DB = 0
SM = 0
Delta_r
Cytarabine
YJL127C SPT10
ZShift = −11.41
Z lm Score = −0.36
NG = 0
DB = 0
SM = 0
Delta_AUC
Cytarabine

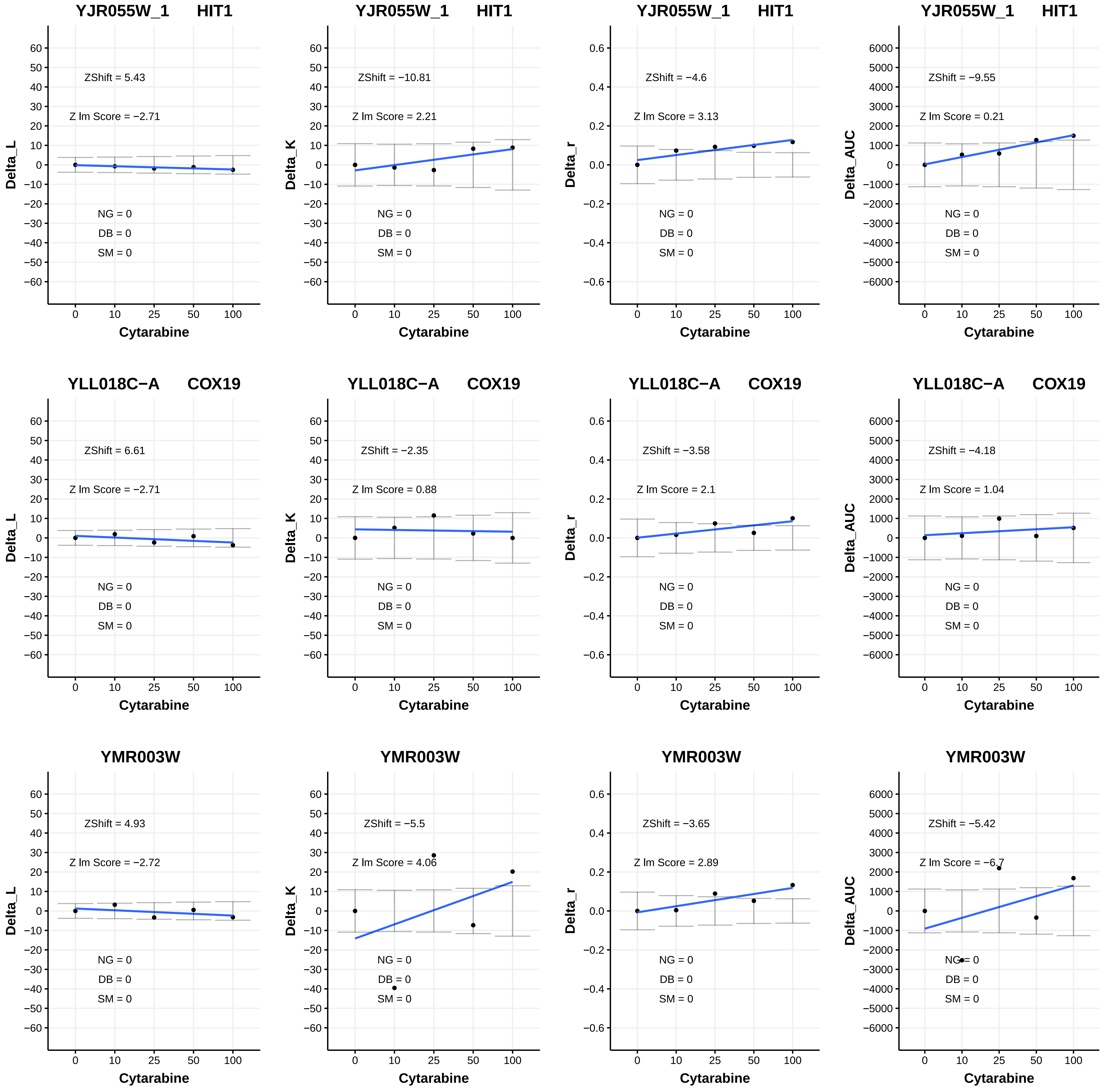
YJR055W_1 HIT1
ZShift = 5.43
Z lm Score = −2.71
NG = 0
DB = 0
SM = 0
Delta_L
Cytarabine
YJR055W_1 HIT1
ZShift = −10.81
Z lm Score = 2.21
NG = 0
DB = 0
SM = 0
Delta_K
Cytarabine
YJR055W_1 HIT1
ZShift = −4.6
Z lm Score = 3.13
NG = 0
DB = 0
SM = 0
Delta_r
Cytarabine
YJR055W_1 HIT1
ZShift = −9.55
Z lm Score = 0.21
NG = 0
DB = 0
SM = 0
Delta_AUC
Cytarabine
YLL018C−A COX19
ZShift = 6.61
Z lm Score = −2.71
NG = 0
DB = 0
SM = 0
Delta_L
Cytarabine
YLL018C−A COX19
ZShift = −2.35
Z lm Score = 0.88
NG = 0
DB = 0
SM = 0
Delta_K
Cytarabine
YLL018C−A COX19
ZShift = −3.58
Z lm Score = 2.1
NG = 0
DB = 0
SM = 0
Delta_r
Cytarabine
YLL018C−A COX19
ZShift = −4.18
Z lm Score = 1.04
NG = 0
DB = 0
SM = 0
Delta_AUC
Cytarabine
YMR003W
ZShift = 4.93
Z lm Score = −2.72
NG = 0
DB = 0
SM = 0
Delta_L
Cytarabine
YMR003W
ZShift = −5.5
Z lm Score = 4.06
NG = 0
DB = 0
SM = 0
Delta_K
Cytarabine
YMR003W
ZShift = −3.65
Z lm Score = 2.89
NG = 0
DB = 0
SM = 0
Delta_r
Cytarabine
YMR003W
ZShift = −5.42
Z lm Score = −6.7
NG = 0
DB = 0
SM = 0
Delta_AUC
Cytarabine

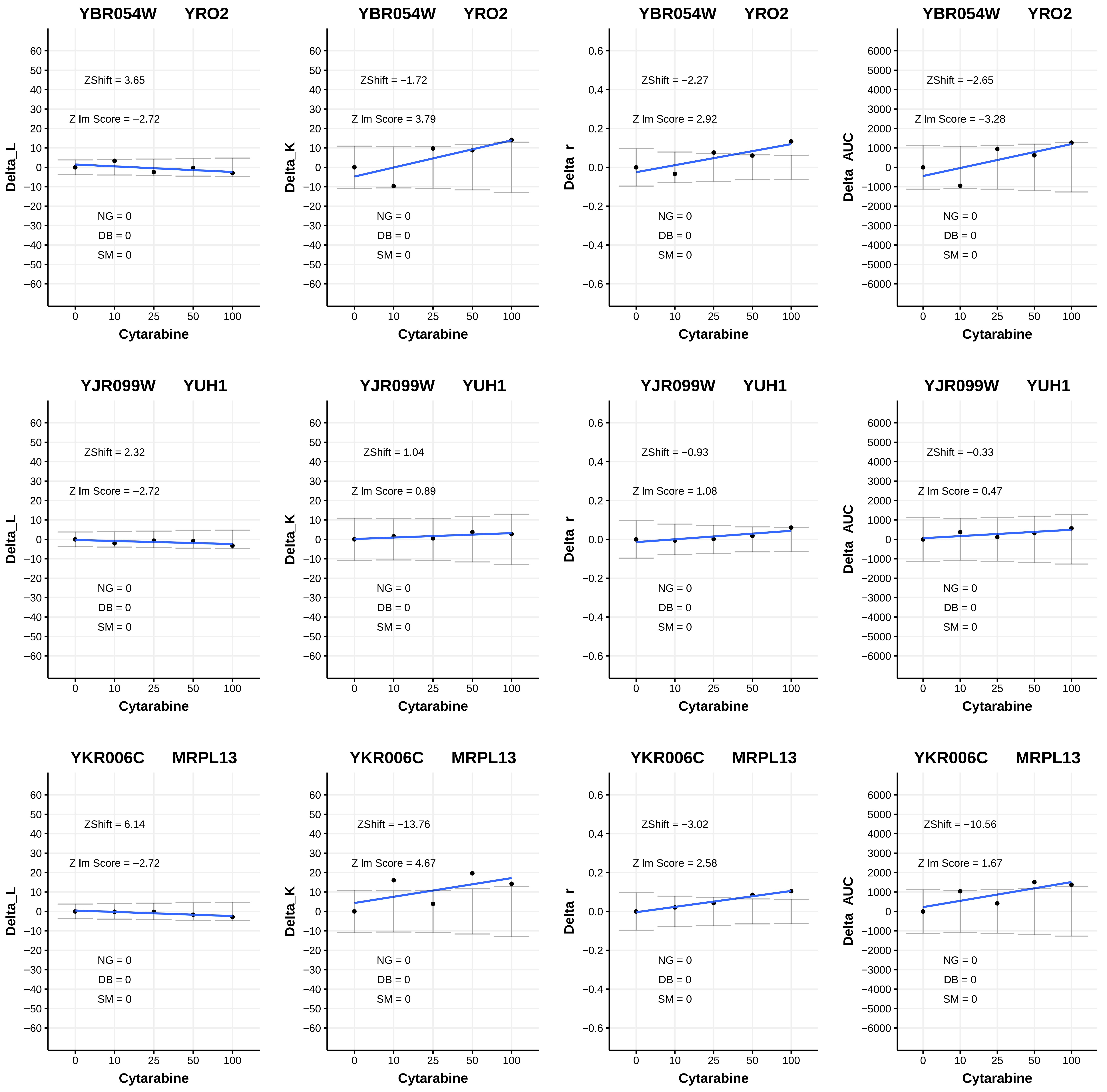
YBR054W YRO2
ZShift = 3.65
Z lm Score = −2.72
NG = 0
DB = 0
SM = 0
Delta_L
Cytarabine
YBR054W YRO2
ZShift = −1.72
Z lm Score = 3.79
NG = 0
DB = 0
SM = 0
Delta_K
Cytarabine
YBR054W YRO2
ZShift = −2.27
Z lm Score = 2.92
NG = 0
DB = 0
SM = 0
Delta_r
Cytarabine
YBR054W YRO2
ZShift = −2.65
Z lm Score = −3.28
NG = 0
DB = 0
SM = 0
Delta_AUC
Cytarabine
YJR099W YUH1
ZShift = 2.32
Z lm Score = −2.72
NG = 0
DB = 0
SM = 0
Delta_L
Cytarabine
YJR099W YUH1
ZShift = 1.04
Z lm Score = 0.89
NG = 0
DB = 0
SM = 0
Delta_K
Cytarabine
YJR099W YUH1
ZShift = −0.93
Z lm Score = 1.08
NG = 0
DB = 0
SM = 0
Delta_r
Cytarabine
YJR099W YUH1
ZShift = −0.33
Z lm Score = 0.47
NG = 0
DB = 0
SM = 0
Delta_AUC
Cytarabine
YKR006C MRPL13
ZShift = 6.14
Z lm Score = −2.72
NG = 0
DB = 0
SM = 0
Delta_L
Cytarabine
YKR006C MRPL13
ZShift = −13.76
Z lm Score = 4.67
NG = 0
DB = 0
SM = 0
Delta_K
Cytarabine
YKR006C MRPL13
ZShift = −3.02
Z lm Score = 2.58
NG = 0
DB = 0
SM = 0
Delta_r
Cytarabine
YKR006C MRPL13
ZShift = −10.56
Z lm Score = 1.67
NG = 0
DB = 0
SM = 0
Delta_AUC
Cytarabine

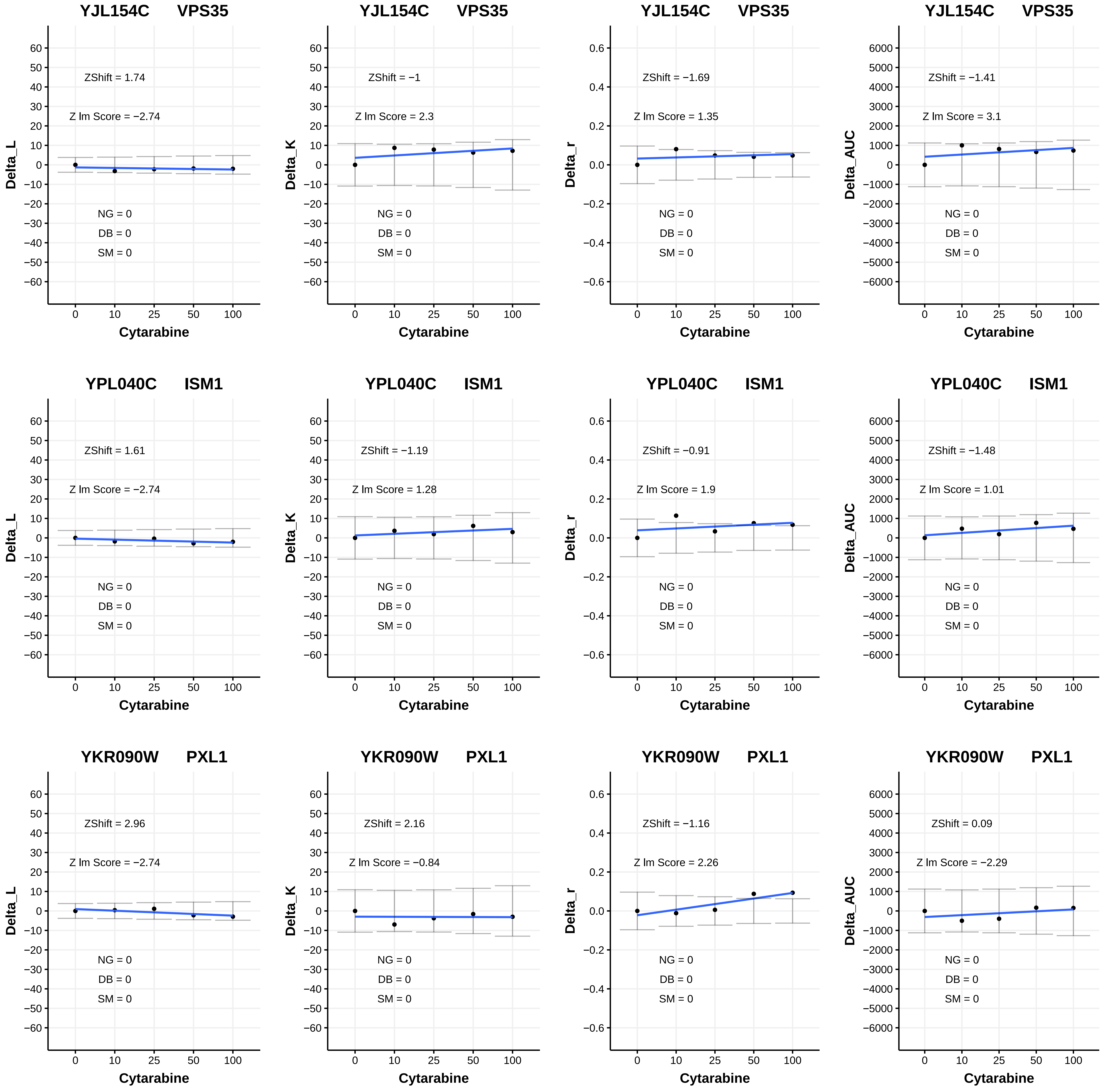
YJL154C VPS35
ZShift = 1.74
Z lm Score = −2.74
NG = 0
DB = 0
SM = 0
Delta_L
Cytarabine
YJL154C VPS35
ZShift = −1
Z lm Score = 2.3
NG = 0
DB = 0
SM = 0
Delta_K
Cytarabine
YJL154C VPS35
ZShift = −1.69
Z lm Score = 1.35
NG = 0
DB = 0
SM = 0
Delta_r
Cytarabine
YJL154C VPS35
ZShift = −1.41
Z lm Score = 3.1
NG = 0
DB = 0
SM = 0
Delta_AUC
Cytarabine
YPL040C ISM1
ZShift = 1.61
Z lm Score = −2.74
NG = 0
DB = 0
SM = 0
Delta_L
Cytarabine
YPL040C ISM1
ZShift = −1.19
Z lm Score = 1.28
NG = 0
DB = 0
SM = 0
Delta_K
Cytarabine
YPL040C ISM1
ZShift = −0.91
Z lm Score = 1.9
NG = 0
DB = 0
SM = 0
Delta_r
Cytarabine
YPL040C ISM1
ZShift = −1.48
Z lm Score = 1.01
NG = 0
DB = 0
SM = 0
Delta_AUC
Cytarabine
YKR090W PXL1
ZShift = 2.96
Z lm Score = −2.74
NG = 0
DB = 0
SM = 0
Delta_L
Cytarabine
YKR090W PXL1
ZShift = 2.16
Z lm Score = −0.84
NG = 0
DB = 0
SM = 0
Delta_K
Cytarabine
YKR090W PXL1
ZShift = −1.16
Z lm Score = 2.26
NG = 0
DB = 0
SM = 0
Delta_r
Cytarabine
YKR090W PXL1
ZShift = 0.09
Z lm Score = −2.29
NG = 0
DB = 0
SM = 0
Delta_AUC
Cytarabine

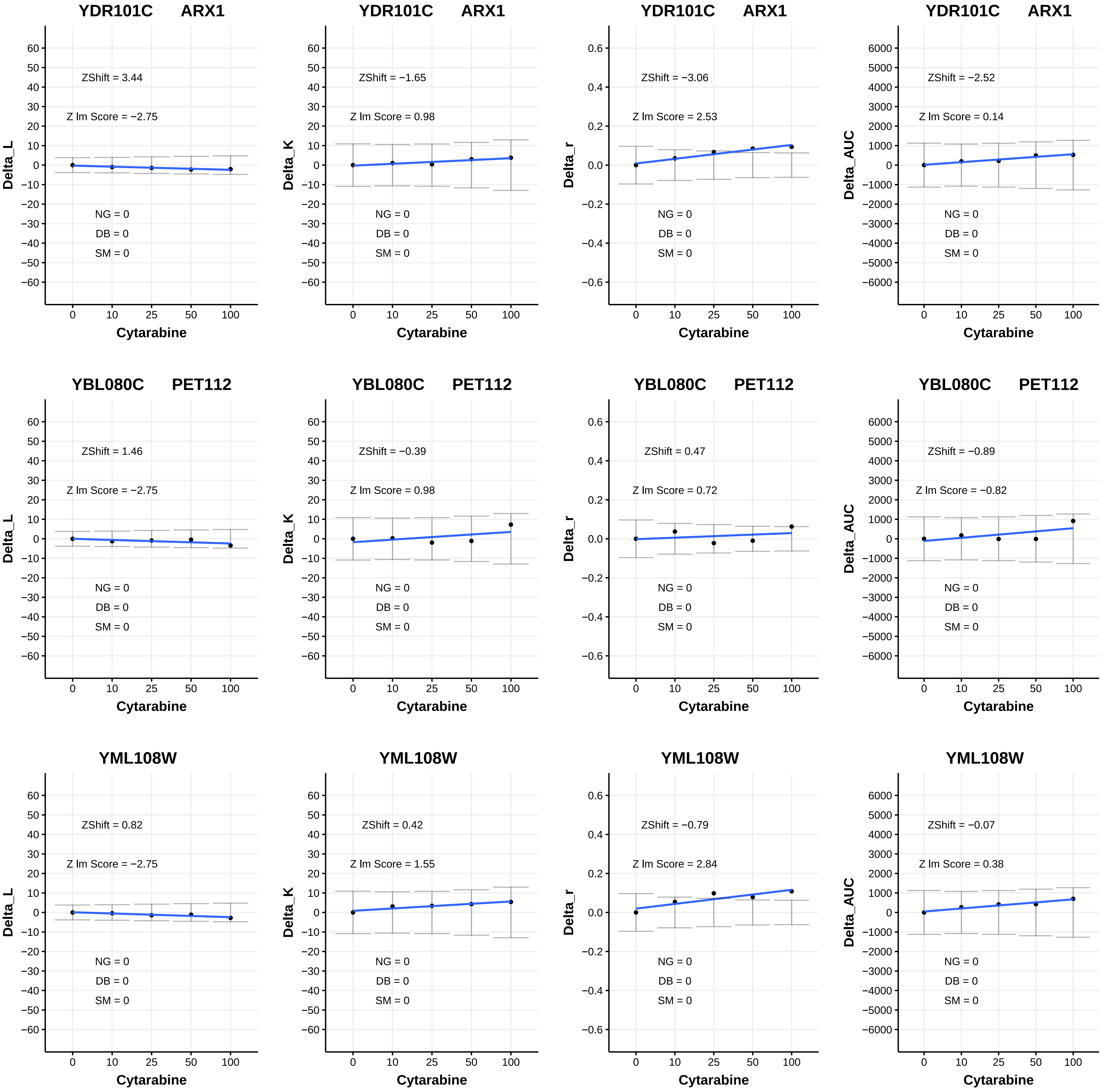
YDR101C ARX1
Delta_L
ZShift = 3.44
Z lm Score = −2.75
NG = 0
DB = 0
SM = 0
Cytarabine
YDR101C ARX1
Delta_K
ZShift = −1.65
Z lm Score = 0.98
NG = 0
DB = 0
SM = 0
Cytarabine
YDR101C ARX1
Delta_r
ZShift = −3.06
Z lm Score = 2.53
NG = 0
DB = 0
SM = 0
Cytarabine
YDR101C ARX1
Delta_AUC
ZShift = −2.52
Z lm Score = 0.14
NG = 0
DB = 0
SM = 0
Cytarabine
YBL080C PET112
Delta_L
ZShift = 1.46
Z lm Score = −2.75
NG = 0
DB = 0
SM = 0
Cytarabine
YBL080C PET112
Delta_K
ZShift = −0.39
Z lm Score = 0.98
NG = 0
DB = 0
SM = 0
Cytarabine
YBL080C PET112
Delta_r
ZShift = 0.47
Z lm Score = 0.72
NG = 0
DB = 0
SM = 0
Cytarabine
YBL080C PET112
Delta_AUC
ZShift = −0.89
Z lm Score = −0.82
NG = 0
DB = 0
SM = 0
Cytarabine
YML108W
Delta_L
ZShift = 0.82
Z lm Score = −2.75
NG = 0
DB = 0
SM = 0
Cytarabine
YML108W
Delta_K
ZShift = 0.42
Z lm Score = 1.55
NG = 0
DB = 0
SM = 0
Cytarabine
YML108W
Delta_r
ZShift = −0.79
Z lm Score = 2.84
NG = 0
DB = 0
SM = 0
Cytarabine
YML108W
Delta_AUC
ZShift = −0.07
Z lm Score = 0.38
NG = 0
DB = 0
SM = 0
Cytarabine

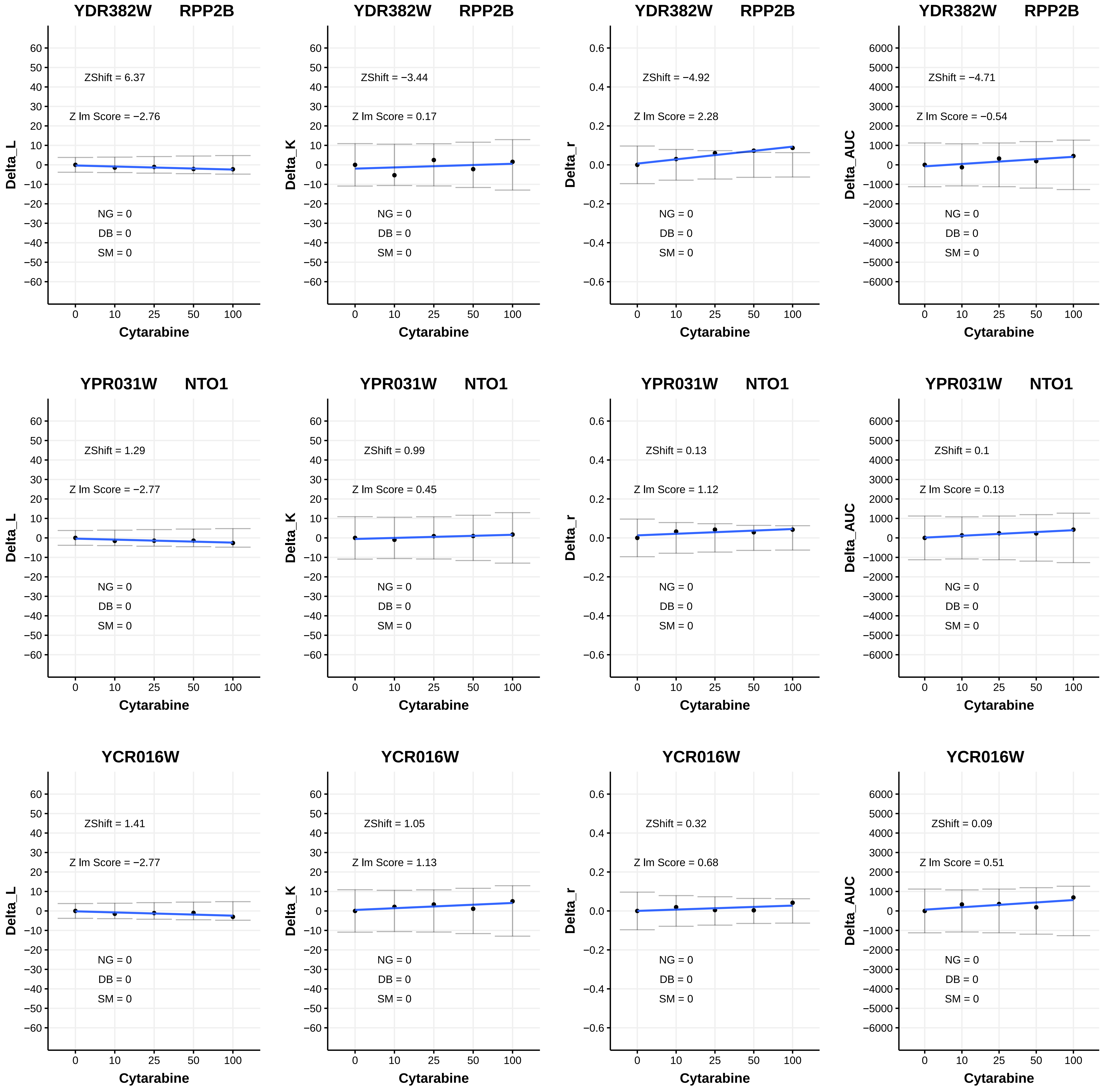
YDR382W RPP2B
ZShift = 6.37
Z lm Score = −2.76
NG = 0
DB = 0
SM = 0
Delta_L
Cytarabine
YDR382W RPP2B
ZShift = −3.44
Z lm Score = 0.17
NG = 0
DB = 0
SM = 0
Delta_K
Cytarabine
YDR382W RPP2B
ZShift = −4.92
Z lm Score = 2.28
NG = 0
DB = 0
SM = 0
Delta_r
Cytarabine
YDR382W RPP2B
ZShift = −4.71
Z lm Score = −0.54
NG = 0
DB = 0
SM = 0
Delta_AUC
Cytarabine
YPR031W NTO1
ZShift = 1.29
Z lm Score = −2.77
NG = 0
DB = 0
SM = 0
Delta_L
Cytarabine
YPR031W NTO1
ZShift = 0.99
Z lm Score = 0.45
NG = 0
DB = 0
SM = 0
Delta_K
Cytarabine
YPR031W NTO1
ZShift = 0.13
Z lm Score = 1.12
NG = 0
DB = 0
SM = 0
Delta_r
Cytarabine
YPR031W NTO1
ZShift = 0.1
Z lm Score = 0.13
NG = 0
DB = 0
SM = 0
Delta_AUC
Cytarabine
YCR016W
ZShift = 1.41
Z lm Score = −2.77
NG = 0
DB = 0
SM = 0
Delta_L
Cytarabine
YCR016W
ZShift = 1.05
Z lm Score = 1.13
NG = 0
DB = 0
SM = 0
Delta_K
Cytarabine
YCR016W
ZShift = 0.32
Z lm Score = 0.68
NG = 0
DB = 0
SM = 0
Delta_r
Cytarabine
YCR016W
ZShift = 0.09
Z lm Score = 0.51
NG = 0
DB = 0
SM = 0
Delta_AUC
Cytarabine

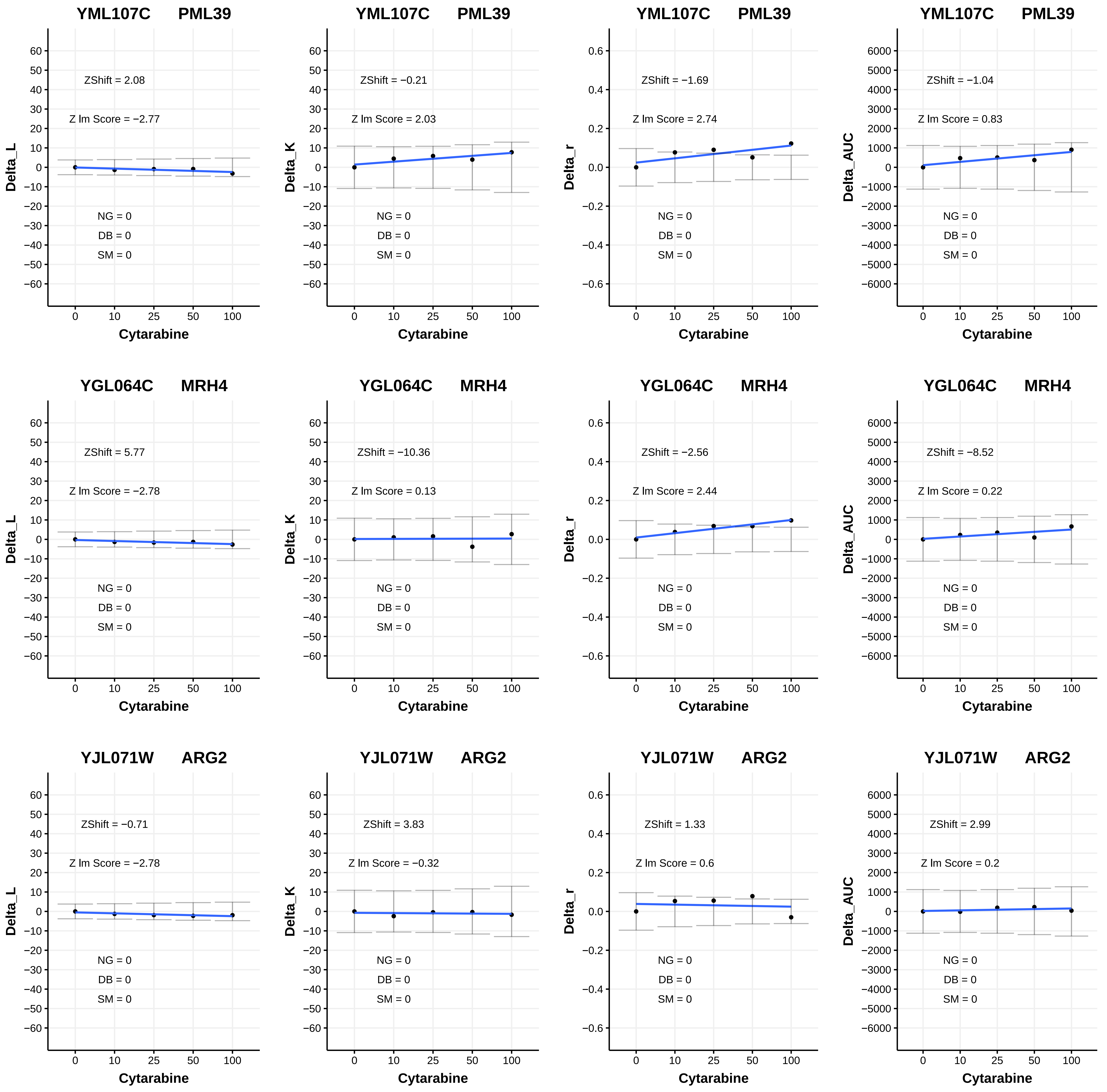
YML107C PML39
ZShift = 2.08
Z lm Score = −2.77
NG = 0
DB = 0
SM = 0
Delta_L
Cytarabine
YML107C PML39
ZShift = −0.21
Z lm Score = 2.03
NG = 0
DB = 0
SM = 0
Delta_K
Cytarabine
YML107C PML39
ZShift = −1.69
Z lm Score = 2.74
NG = 0
DB = 0
SM = 0
Delta_r
Cytarabine
YML107C PML39
ZShift = −1.04
Z lm Score = 0.83
NG = 0
DB = 0
SM = 0
Delta_AUC
Cytarabine
YGL064C MRH4
ZShift = 5.77
Z lm Score = −2.78
NG = 0
DB = 0
SM = 0
Delta_L
Cytarabine
YGL064C MRH4
ZShift = −10.36
Z lm Score = 0.13
NG = 0
DB = 0
SM = 0
Delta_K
Cytarabine
YGL064C MRH4
ZShift = −2.56
Z lm Score = 2.44
NG = 0
DB = 0
SM = 0
Delta_r
Cytarabine
YGL064C MRH4
ZShift = −8.52
Z lm Score = 0.22
NG = 0
DB = 0
SM = 0
Delta_AUC
Cytarabine
YJL071W ARG2
ZShift = −0.71
Z lm Score = −2.78
NG = 0
DB = 0
SM = 0
Delta_L
Cytarabine
YJL071W ARG2
ZShift = 3.83
Z lm Score = −0.32
NG = 0
DB = 0
SM = 0
Delta_K
Cytarabine
YJL071W ARG2
ZShift = 1.33
Z lm Score = 0.6
NG = 0
DB = 0
SM = 0
Delta_r
Cytarabine
YJL071W ARG2
ZShift = 2.99
Z lm Score = 0.2
NG = 0
DB = 0
SM = 0
Delta_AUC
Cytarabine

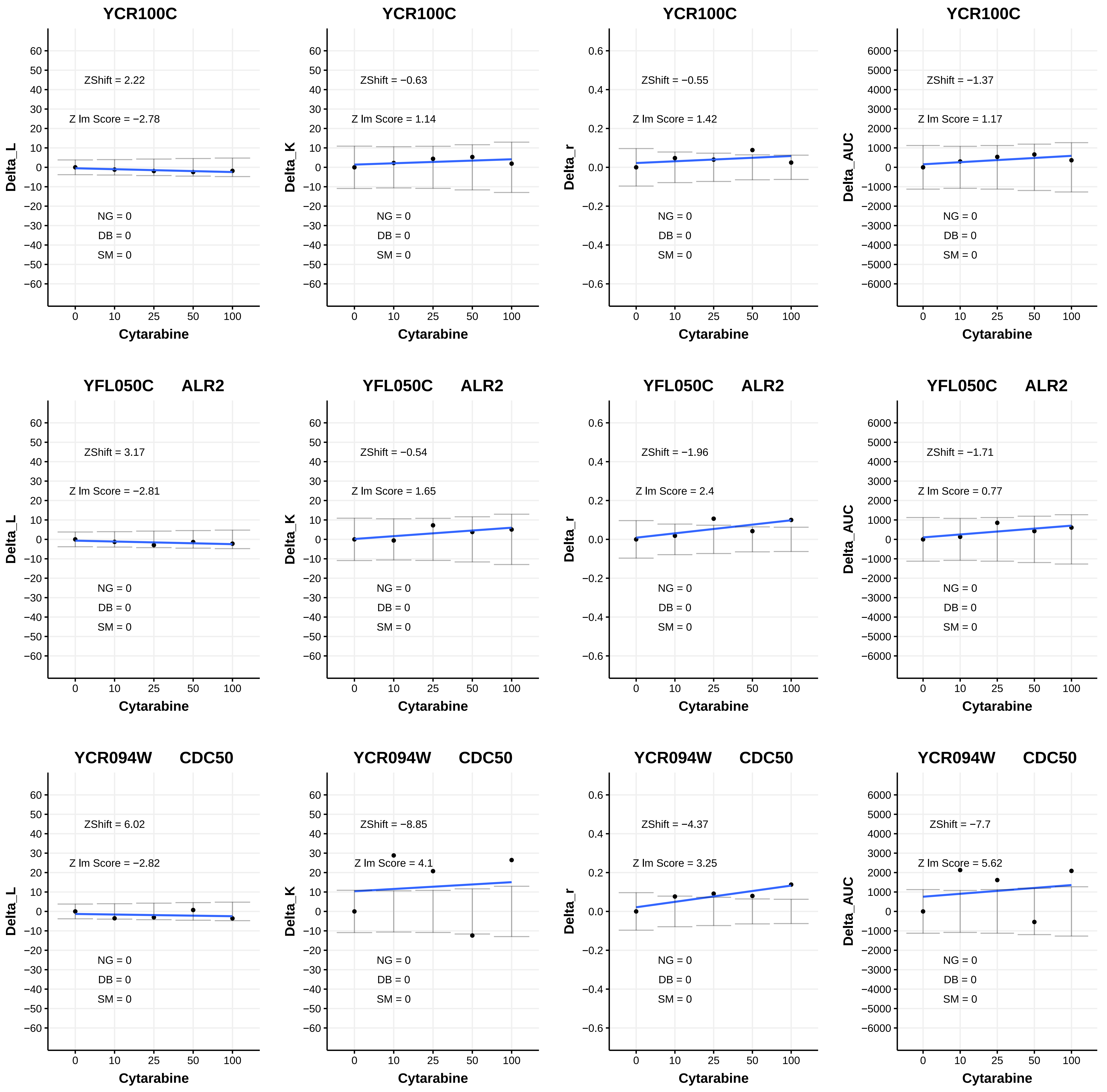
YCR100C
ZShift = 2.22
Z lm Score = −2.78
NG = 0
DB = 0
SM = 0
Delta_L
Cytarabine
YCR100C
ZShift = −0.63
Z lm Score = 1.14
NG = 0
DB = 0
SM = 0
Delta_K
Cytarabine
YCR100C
ZShift = −0.55
Z lm Score = 1.42
NG = 0
DB = 0
SM = 0
Delta_r
Cytarabine
YCR100C
ZShift = −1.37
Z lm Score = 1.17
NG = 0
DB = 0
SM = 0
Delta_AUC
Cytarabine
YFL050C ALR2
ZShift = 3.17
Z lm Score = −2.81
NG = 0
DB = 0
SM = 0
Delta_L
Cytarabine
YFL050C ALR2
ZShift = −0.54
Z lm Score = 1.65
NG = 0
DB = 0
SM = 0
Delta_K
Cytarabine
YFL050C ALR2
ZShift = −1.96
Z lm Score = 2.4
NG = 0
DB = 0
SM = 0
Delta_r
Cytarabine
YFL050C ALR2
ZShift = −1.71
Z lm Score = 0.77
NG = 0
DB = 0
SM = 0
Delta_AUC
Cytarabine
YCR094W CDC50
ZShift = 6.02
Z lm Score = −2.82
NG = 0
DB = 0
SM = 0
Delta_L
Cytarabine
YCR094W CDC50
ZShift = −8.85
Z lm Score = 4.1
NG = 0
DB = 0
SM = 0
Delta_K
Cytarabine
YCR094W CDC50
ZShift = −4.37
Z lm Score = 3.25
NG = 0
DB = 0
SM = 0
Delta_r
Cytarabine
YCR094W CDC50
ZShift = −7.7
Z lm Score = 5.62
NG = 0
DB = 0
SM = 0
Delta_AUC
Cytarabine

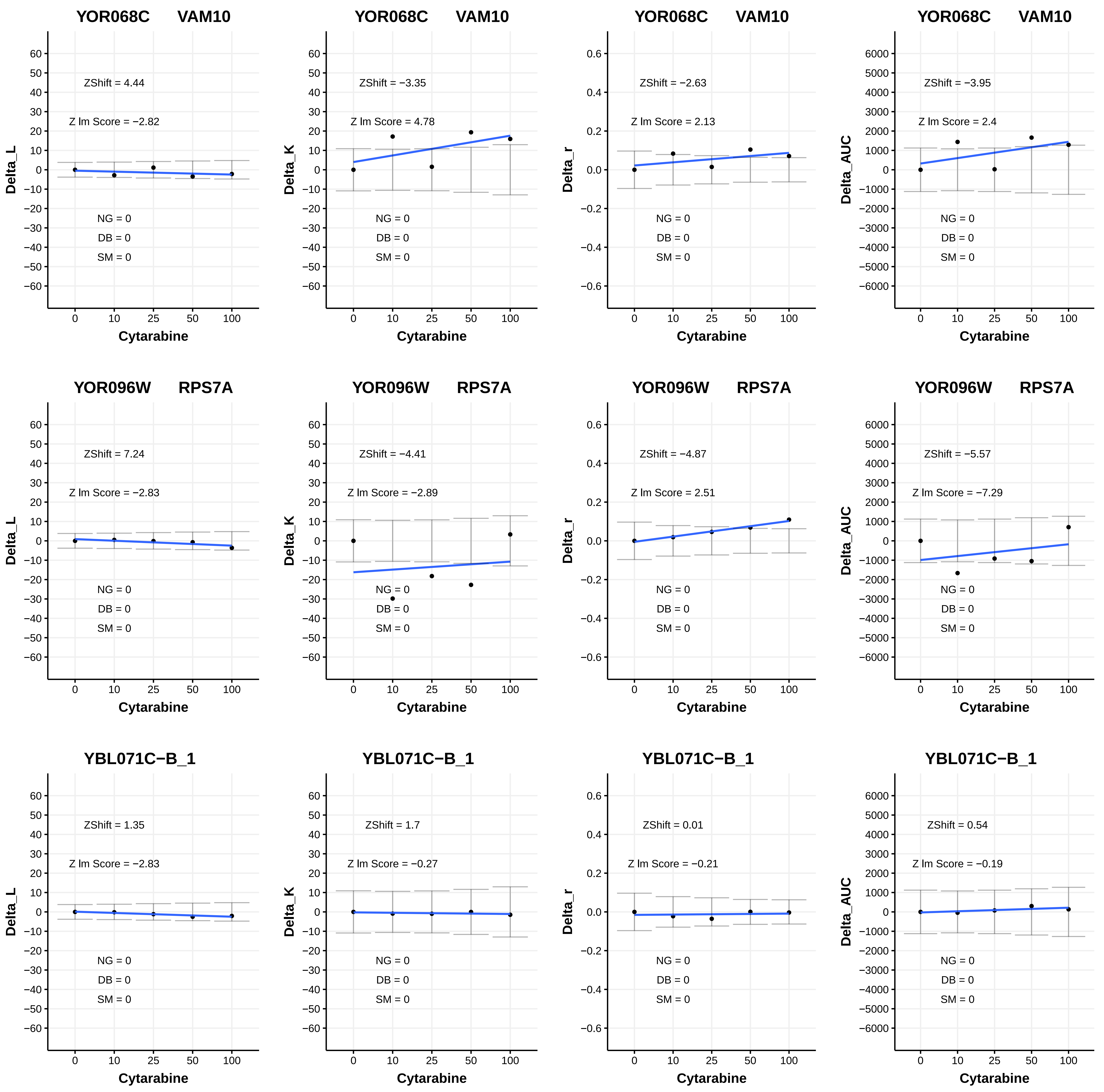
YOR068C VAM10
ZShift = 4.44
Z lm Score = −2.82
NG = 0
DB = 0
SM = 0
Delta_L
Cytarabine
YOR068C VAM10
ZShift = −3.35
Z lm Score = 4.78
NG = 0
DB = 0
SM = 0
Delta_K
Cytarabine
YOR068C VAM10
ZShift = −2.63
Z lm Score = 2.13
NG = 0
DB = 0
SM = 0
Delta_r
Cytarabine
YOR068C VAM10
ZShift = −3.95
Z lm Score = 2.4
NG = 0
DB = 0
SM = 0
Delta_AUC
Cytarabine
YOR096W RPS7A
ZShift = 7.24
Z lm Score = −2.83
NG = 0
DB = 0
SM = 0
Delta_L
Cytarabine
YOR096W RPS7A
ZShift = −4.41
Z lm Score = −2.89
NG = 0
DB = 0
SM = 0
Delta_K
Cytarabine
YOR096W RPS7A
ZShift = −4.87
Z lm Score = 2.51
NG = 0
DB = 0
SM = 0
Delta_r
Cytarabine
YOR096W RPS7A
ZShift = −5.57
Z lm Score = −7.29
NG = 0
DB = 0
SM = 0
Delta_AUC
Cytarabine
YBL071C−B_1
ZShift = 1.35
Z lm Score = −2.83
NG = 0
DB = 0
SM = 0
Delta_L
Cytarabine
YBL071C−B_1
ZShift = 1.7
Z lm Score = −0.27
NG = 0
DB = 0
SM = 0
Delta_K
Cytarabine
YBL071C−B_1
ZShift = 0.01
Z lm Score = −0.21
NG = 0
DB = 0
SM = 0
Delta_r
Cytarabine
YBL071C−B_1
ZShift = 0.54
Z lm Score = −0.19
NG = 0
DB = 0
SM = 0
Delta_AUC
Cytarabine

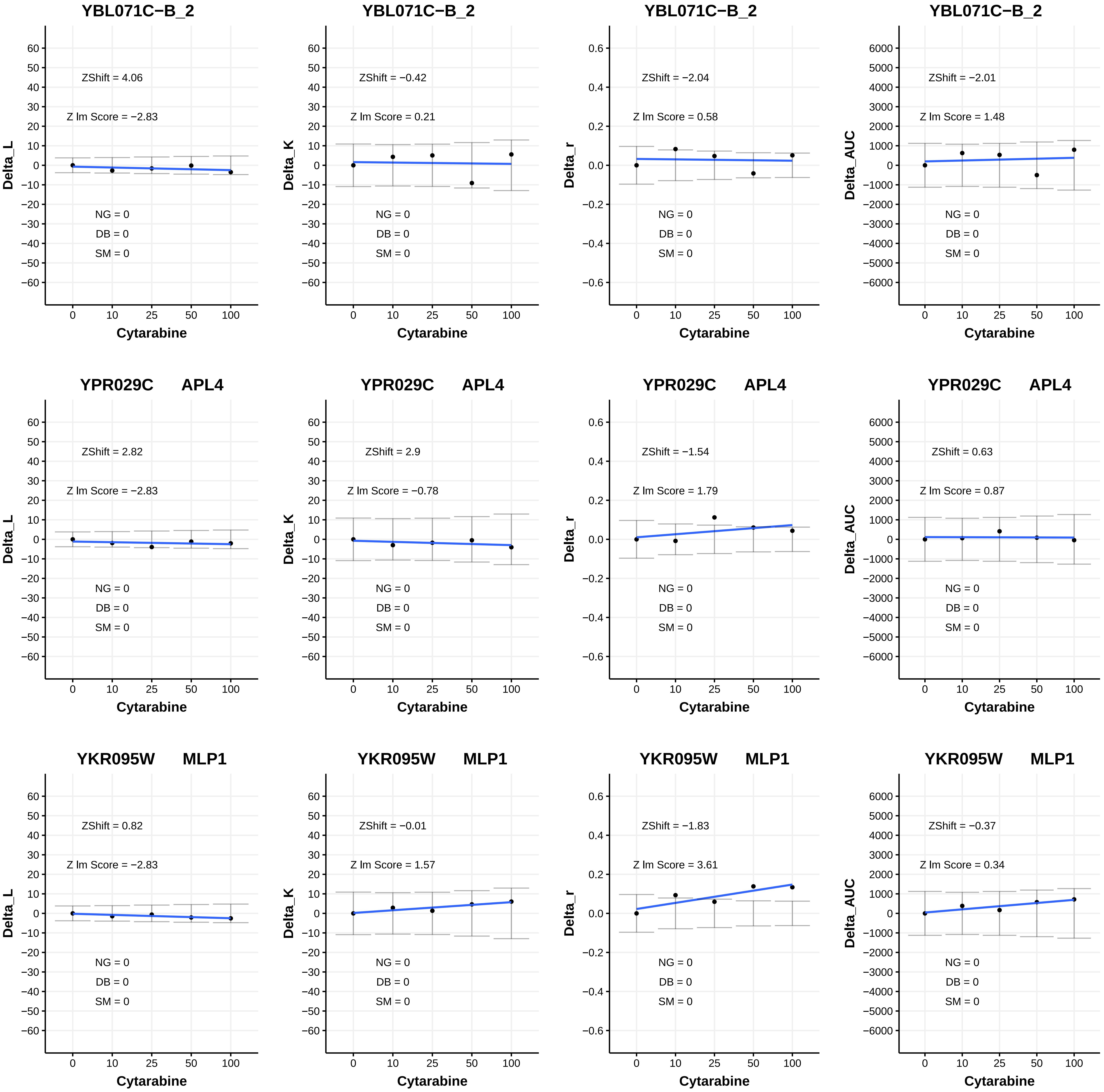

YBL071C−B_2
ZShift = 4.06
Z lm Score = −2.83
NG = 0
DB = 0
SM = 0
Delta_L
Cytarabine
YBL071C−B_2
ZShift = −0.42
Z lm Score = 0.21
NG = 0
DB = 0
SM = 0
Delta_K
Cytarabine
YBL071C−B_2
ZShift = −2.04
Z lm Score = 0.58
NG = 0
DB = 0
SM = 0
Delta_r
Cytarabine
YBL071C−B_2
ZShift = −2.01
Z lm Score = 1.48
NG = 0
DB = 0
SM = 0
Delta_AUC
Cytarabine
YPR029C APL4
ZShift = 2.82
Z lm Score = −2.83
NG = 0
DB = 0
SM = 0
Delta_L
Cytarabine
YPR029C APL4
ZShift = 2.9
Z lm Score = −0.78
NG = 0
DB = 0
SM = 0
Delta_K
Cytarabine
YPR029C APL4
ZShift = −1.54
Z lm Score = 1.79
NG = 0
DB = 0
SM = 0
Delta_r
Cytarabine
YPR029C APL4
ZShift = 0.63
Z lm Score = 0.87
NG = 0
DB = 0
SM = 0
Delta_AUC
Cytarabine
YKR095W MLP1
ZShift = 0.82
Z lm Score = −2.83
NG = 0
DB = 0
SM = 0
Delta_L
Cytarabine
YKR095W MLP1
ZShift = −0.01
Z lm Score = 1.57
NG = 0
DB = 0
SM = 0
Delta_K
Cytarabine
YKR095W MLP1
ZShift = −1.83
Z lm Score = 3.61
NG = 0
DB = 0
SM = 0
Delta_r
Cytarabine
YKR095W MLP1
ZShift = −0.37
Z lm Score = 0.34
NG = 0
DB = 0
SM = 0
Delta_AUC
Cytarabine

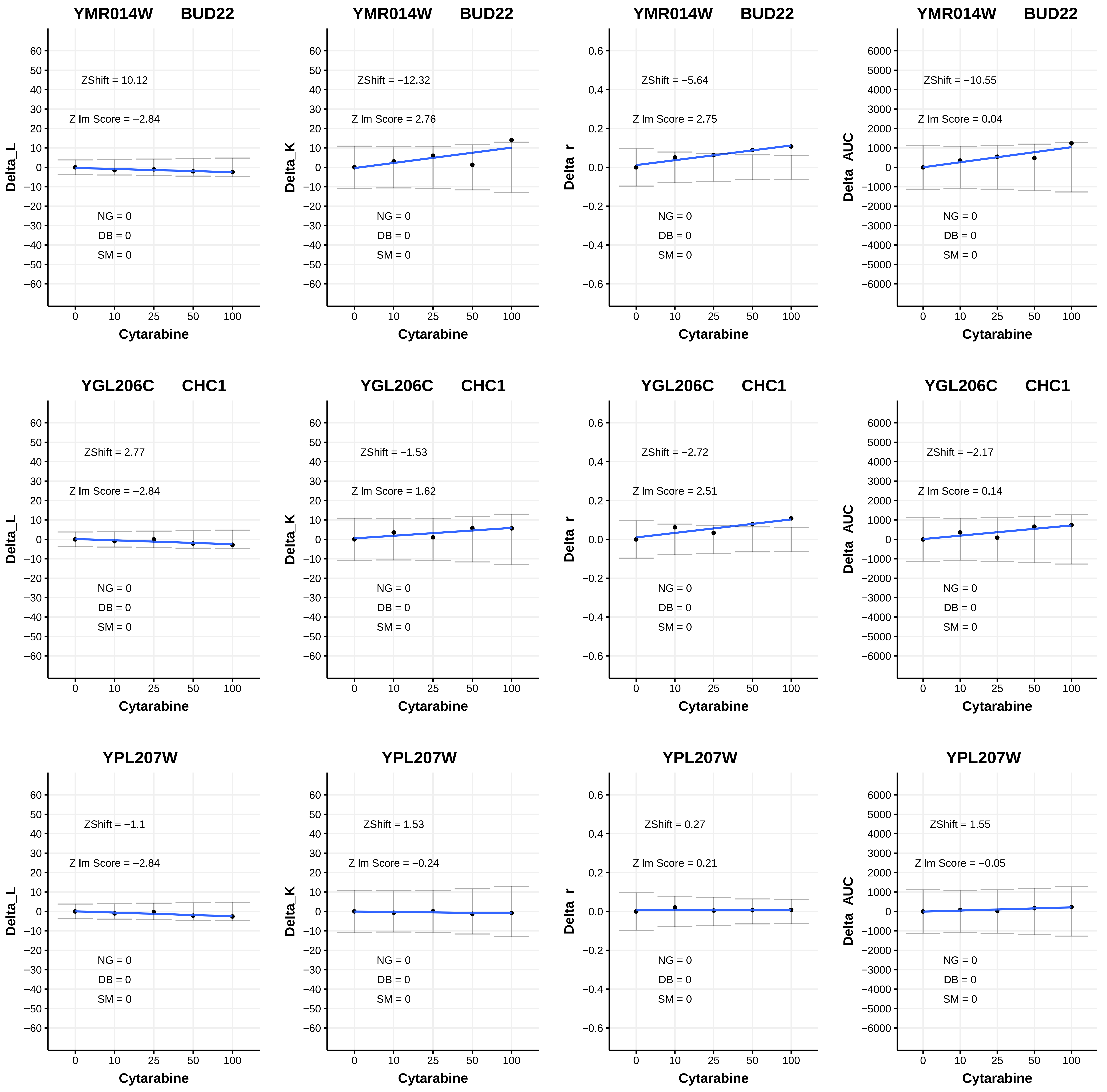

YMR014W BUD22
ZShift = 10.12
Z lm Score = −2.84
NG = 0
DB = 0
SM = 0
Delta_L
Cytarabine
YMR014W BUD22
ZShift = −12.32
Z lm Score = 2.76
NG = 0
DB = 0
SM = 0
Delta_K
Cytarabine
YMR014W BUD22
ZShift = −5.64
Z lm Score = 2.75
NG = 0
DB = 0
SM = 0
Delta_r
Cytarabine
YMR014W BUD22
ZShift = −10.55
Z lm Score = 0.04
NG = 0
DB = 0
SM = 0
Delta_AUC
Cytarabine
YGL206C CHC1
ZShift = 2.77
Z lm Score = −2.84
NG = 0
DB = 0
SM = 0
Delta_L
Cytarabine
YGL206C CHC1
ZShift = −1.53
Z lm Score = 1.62
NG = 0
DB = 0
SM = 0
Delta_K
Cytarabine
YGL206C CHC1
ZShift = −2.72
Z lm Score = 2.51
NG = 0
DB = 0
SM = 0
Delta_r
Cytarabine
YGL206C CHC1
ZShift = −2.17
Z lm Score = 0.14
NG = 0
DB = 0
SM = 0
Delta_AUC
Cytarabine
YPL207W
ZShift = −1.1
Z lm Score = −2.84
NG = 0
DB = 0
SM = 0
Delta_L
Cytarabine
YPL207W
ZShift = 1.53
Z lm Score = −0.24
NG = 0
DB = 0
SM = 0
Delta_K
Cytarabine
YPL207W
ZShift = 0.27
Z lm Score = 0.21
NG = 0
DB = 0
SM = 0
Delta_r
Cytarabine
YPL207W
ZShift = 1.55
Z lm Score = −0.05
NG = 0
DB = 0
SM = 0
Delta_AUC
Cytarabine

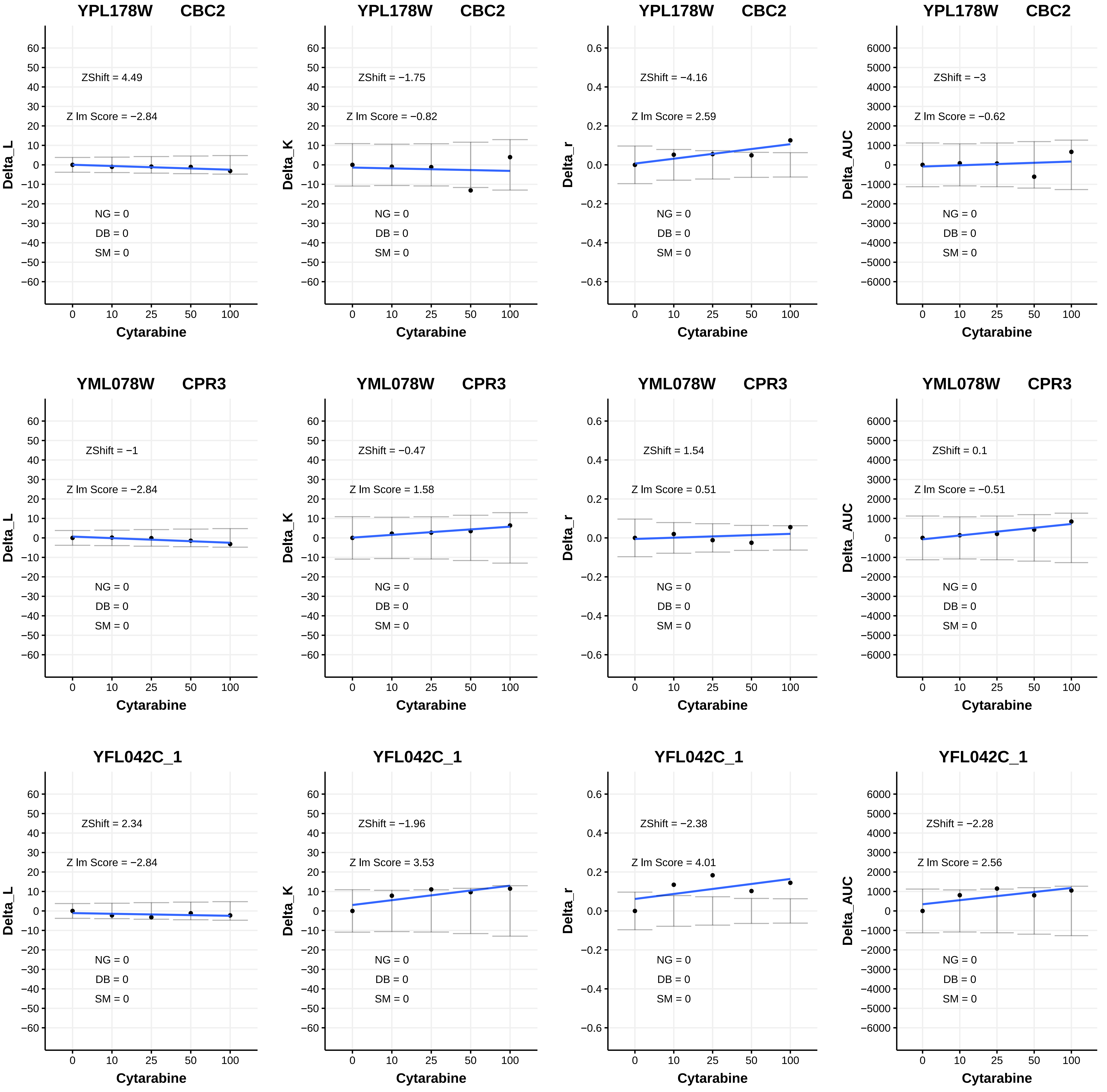
YPL178W CBC2
ZShift = 4.49
Z lm Score = −2.84
NG = 0
DB = 0
SM = 0
Delta_L
Cytarabine
YPL178W CBC2
ZShift = −1.75
Z lm Score = −0.82
NG = 0
DB = 0
SM = 0
Delta_K
Cytarabine
YPL178W CBC2
ZShift = −4.16
Z lm Score = 2.59
NG = 0
DB = 0
SM = 0
Delta_r
Cytarabine
YPL178W CBC2
ZShift = −3
Z lm Score = −0.62
NG = 0
DB = 0
SM = 0
Delta_AUC
Cytarabine
YML078W CPR3
ZShift = −1
Z lm Score = −2.84
NG = 0
DB = 0
SM = 0
Delta_L
Cytarabine
YML078W CPR3
ZShift = −0.47
Z lm Score = 1.58
NG = 0
DB = 0
SM = 0
Delta_K
Cytarabine
YML078W CPR3
ZShift = 1.54
Z lm Score = 0.51
NG = 0
DB = 0
SM = 0
Delta_r
Cytarabine
YML078W CPR3
ZShift = 0.1
Z lm Score = −0.51
NG = 0
DB = 0
SM = 0
Delta_AUC
Cytarabine
YFL042C_1
ZShift = 2.34
Z lm Score = −2.84
NG = 0
DB = 0
SM = 0
Delta_L
Cytarabine
YFL042C_1
ZShift = −1.96
Z lm Score = 3.53
NG = 0
DB = 0
SM = 0
Delta_K
Cytarabine
YFL042C_1
ZShift = −2.38
Z lm Score = 4.01
NG = 0
DB = 0
SM = 0
Delta_r
Cytarabine
YFL042C_1
ZShift = −2.28
Z lm Score = 2.56
NG = 0
DB = 0
SM = 0
Delta_AUC
Cytarabine

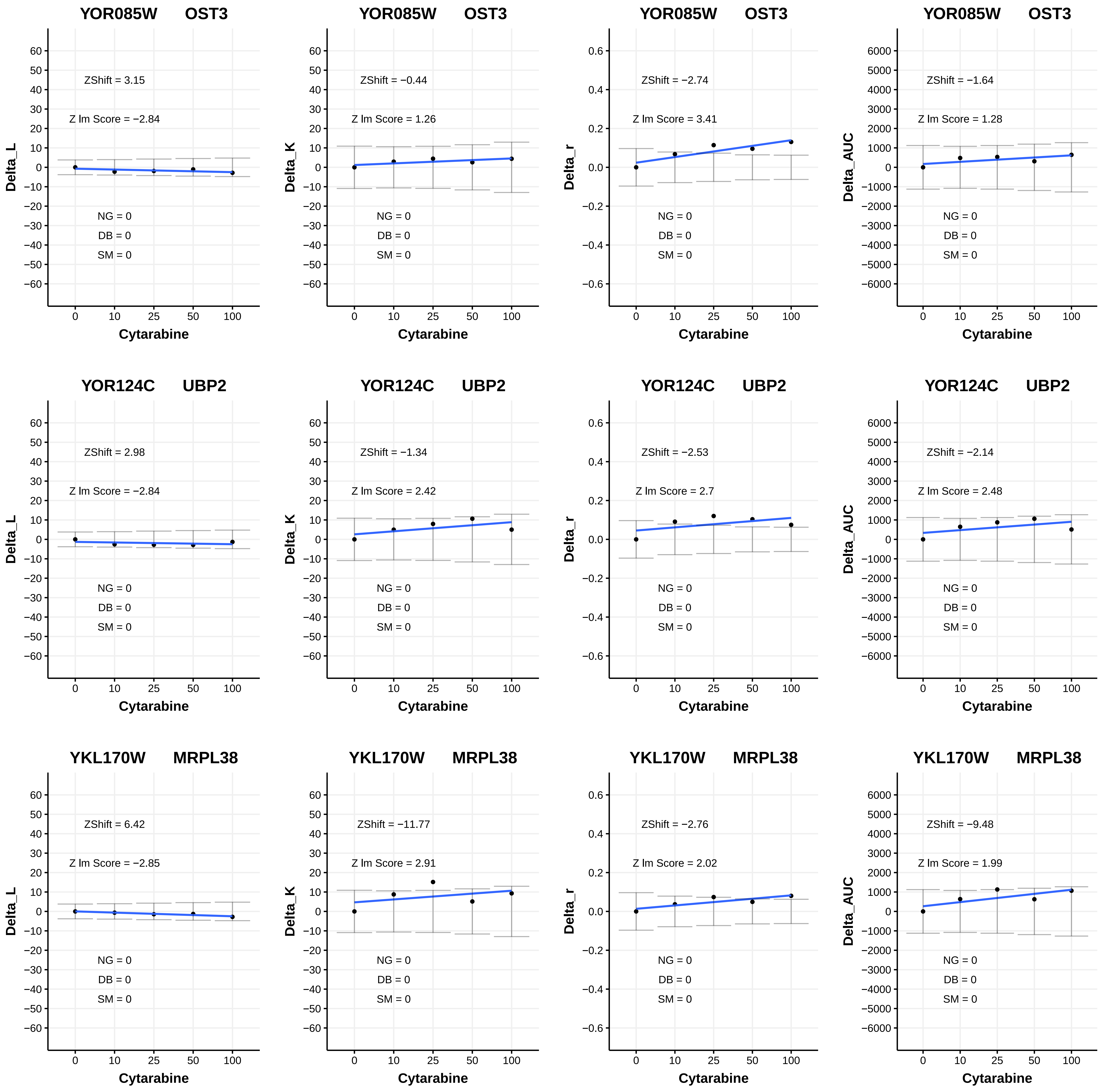

YOR085W OST3
ZShift = 3.15
Z lm Score = −2.84
NG = 0
DB = 0
SM = 0
Delta_L
YOR085W OST3
ZShift = −0.44
Z lm Score = 1.26
NG = 0
DB = 0
SM = 0
Delta_K
YOR085W OST3
ZShift = −2.74
Z lm Score = 3.41
NG = 0
DB = 0
SM = 0
Delta_r
YOR085W OST3
ZShift = −1.64
Z lm Score = 1.28
NG = 0
DB = 0
SM = 0
Delta_AUC
YOR124C UBP2
ZShift = 2.98
Z lm Score = −2.84
NG = 0
DB = 0
SM = 0
Delta_L
YOR124C UBP2
ZShift = −1.34
Z lm Score = 2.42
NG = 0
DB = 0
SM = 0
Delta_K
YOR124C UBP2
ZShift = −2.53
Z lm Score = 2.7
NG = 0
DB = 0
SM = 0
Delta_r
YOR124C UBP2
ZShift = −2.14
Z lm Score = 2.48
NG = 0
DB = 0
SM = 0
Delta_AUC
YKL170W MRPL38
ZShift = 6.42
Z lm Score = −2.85
NG = 0
DB = 0
SM = 0
Delta_L
YKL170W MRPL38
ZShift = −11.77
Z lm Score = 2.91
NG = 0
DB = 0
SM = 0
Delta_K
YKL170W MRPL38
ZShift = −2.76
Z lm Score = 2.02
NG = 0
DB = 0
SM = 0
Delta_r
YKL170W MRPL38
ZShift = −9.48
Z lm Score = 1.99
NG = 0
DB = 0
SM = 0
Delta_AUC
0 10 25 50 100
Cytarabine

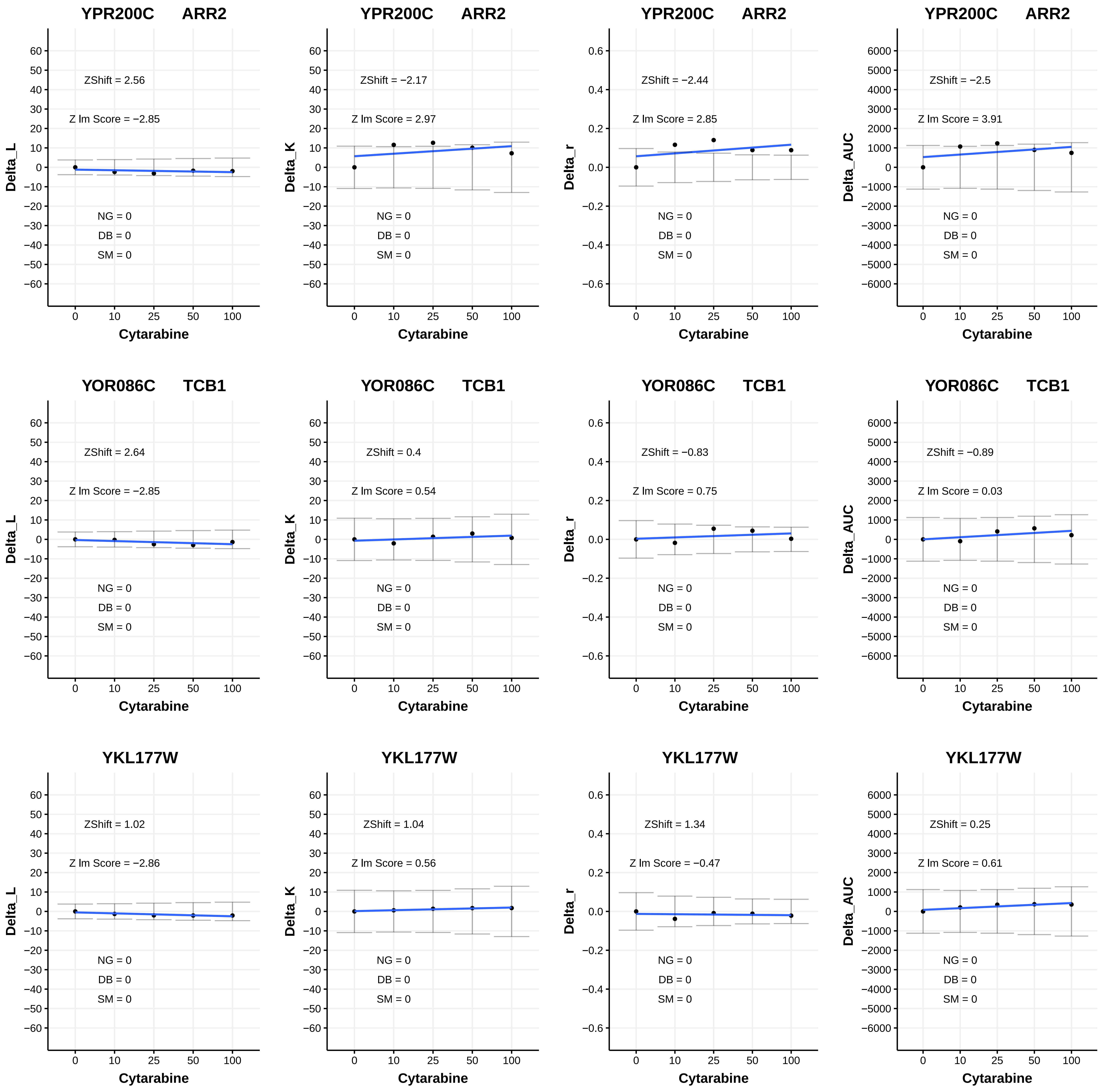
YPR200C ARR2
ZShift = 2.56
Z lm Score = −2.85
NG = 0
DB = 0
SM = 0
Delta_L
Cytarabine
YPR200C ARR2
ZShift = −2.17
Z lm Score = 2.97
NG = 0
DB = 0
SM = 0
Delta_K
Cytarabine
YPR200C ARR2
ZShift = −2.44
Z lm Score = 2.85
NG = 0
DB = 0
SM = 0
Delta_r
Cytarabine
YPR200C ARR2
ZShift = −2.5
Z lm Score = 3.91
NG = 0
DB = 0
SM = 0
Delta_AUC
Cytarabine
YOR086C TCB1
ZShift = 2.64
Z lm Score = −2.85
NG = 0
DB = 0
SM = 0
Delta_L
Cytarabine
YOR086C TCB1
ZShift = 0.4
Z lm Score = 0.54
NG = 0
DB = 0
SM = 0
Delta_K
Cytarabine
YOR086C TCB1
ZShift = −0.83
Z lm Score = 0.75
NG = 0
DB = 0
SM = 0
Delta_r
Cytarabine
YOR086C TCB1
ZShift = −0.89
Z lm Score = 0.03
NG = 0
DB = 0
SM = 0
Delta_AUC
Cytarabine
YKL177W
ZShift = 1.02
Z lm Score = −2.86
NG = 0
DB = 0
SM = 0
Delta_L
Cytarabine
YKL177W
ZShift = 1.04
Z lm Score = 0.56
NG = 0
DB = 0
SM = 0
Delta_K
Cytarabine
YKL177W
ZShift = 1.34
Z lm Score = −0.47
NG = 0
DB = 0
SM = 0
Delta_r
Cytarabine
YKL177W
ZShift = 0.25
Z lm Score = 0.61
NG = 0
DB = 0
SM = 0
Delta_AUC
Cytarabine

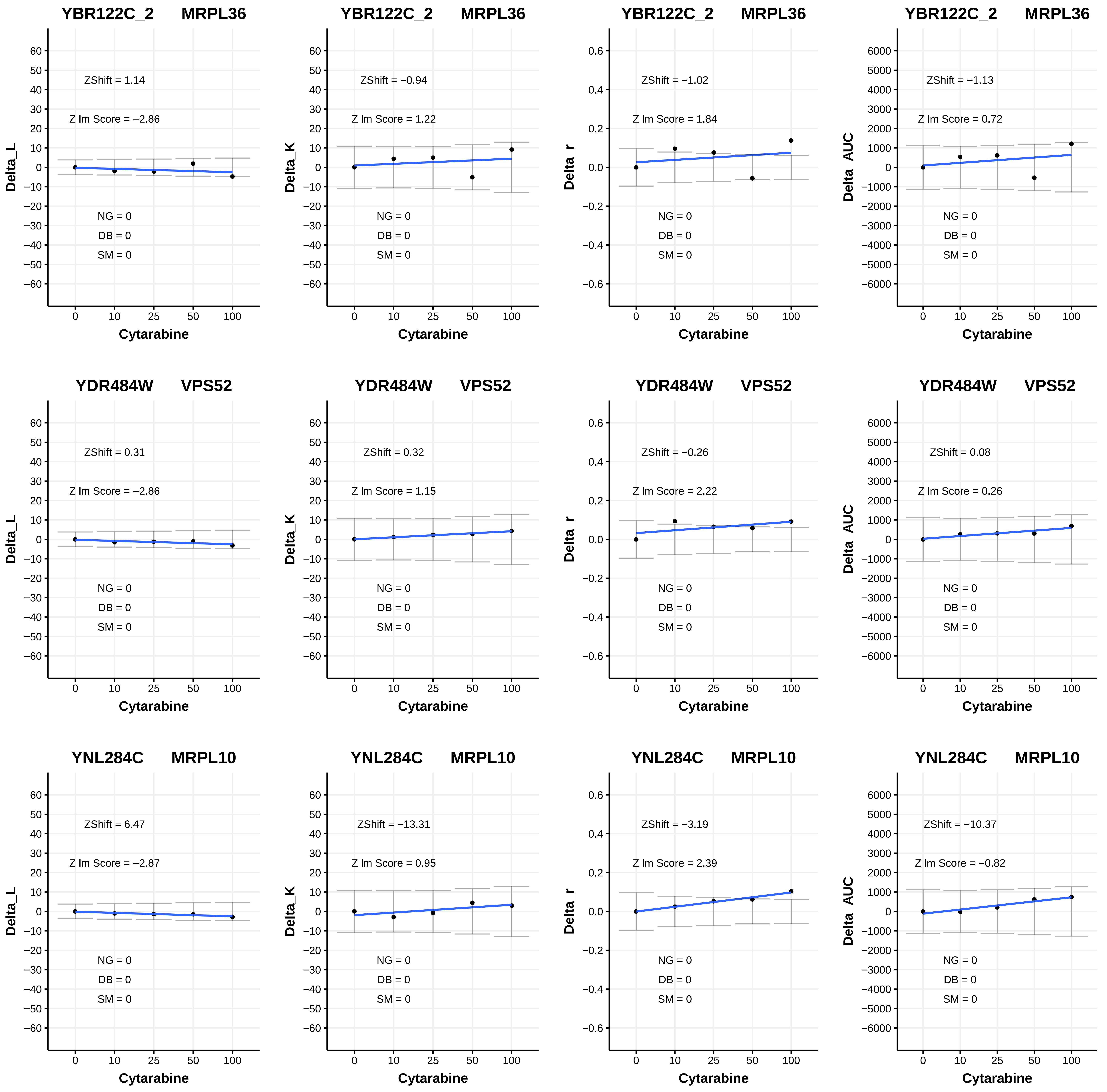

YBR122C_2 MRPL36
ZShift = 1.14
Z lm Score = −2.86
NG = 0
DB = 0
SM = 0
Delta_L
Cytarabine
YBR122C_2 MRPL36
ZShift = −0.94
Z lm Score = 1.22
NG = 0
DB = 0
SM = 0
Delta_K
Cytarabine
YBR122C_2 MRPL36
ZShift = −1.02
Z lm Score = 1.84
NG = 0
DB = 0
SM = 0
Delta_r
Cytarabine
YBR122C_2 MRPL36
ZShift = −1.13
Z lm Score = 0.72
NG = 0
DB = 0
SM = 0
Delta_AUC
Cytarabine
YDR484W VPS52
ZShift = 0.31
Z lm Score = −2.86
NG = 0
DB = 0
SM = 0
Delta_L
Cytarabine
YDR484W VPS52
ZShift = 0.32
Z lm Score = 1.15
NG = 0
DB = 0
SM = 0
Delta_K
Cytarabine
YDR484W VPS52
ZShift = −0.26
Z lm Score = 2.22
NG = 0
DB = 0
SM = 0
Delta_r
Cytarabine
YDR484W VPS52
ZShift = 0.08
Z lm Score = 0.26
NG = 0
DB = 0
SM = 0
Delta_AUC
Cytarabine
YNL284C MRPL10
ZShift = 6.47
Z lm Score = −2.87
NG = 0
DB = 0
SM = 0
Delta_L
Cytarabine
YNL284C MRPL10
ZShift = −13.31
Z lm Score = 0.95
NG = 0
DB = 0
SM = 0
Delta_K
Cytarabine
YNL284C MRPL10
ZShift = −3.19
Z lm Score = 2.39
NG = 0
DB = 0
SM = 0
Delta_r
Cytarabine
YNL284C MRPL10
ZShift = −10.37
Z lm Score = −0.82
NG = 0
DB = 0
SM = 0
Delta_AUC
Cytarabine

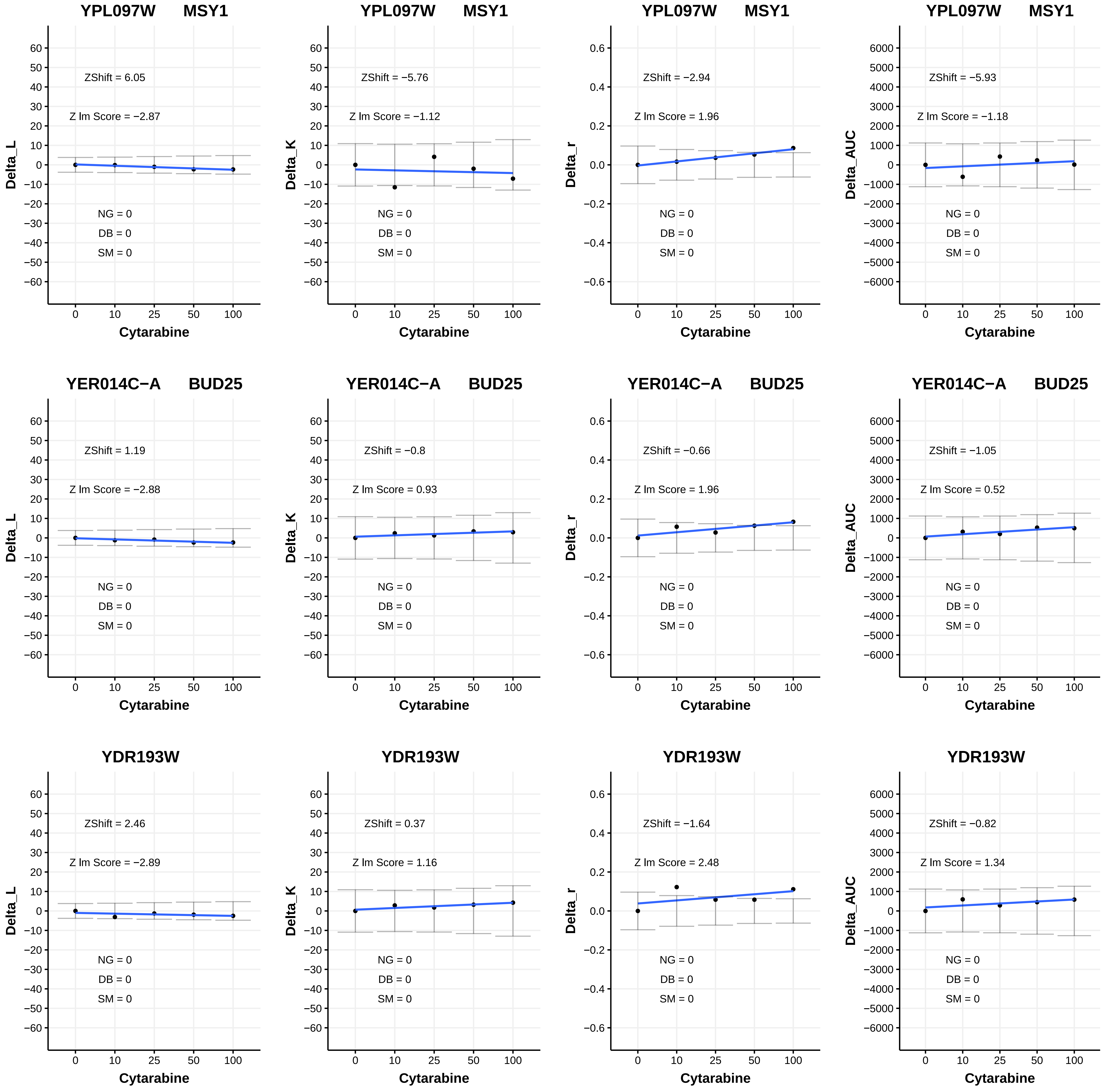

YPL097W MSY1
ZShift = 6.05
Z lm Score = −2.87
NG = 0
DB = 0
SM = 0
Delta_L
Cytarabine
YPL097W MSY1
ZShift = −5.76
Z lm Score = −1.12
NG = 0
DB = 0
SM = 0
Delta_K
Cytarabine
YPL097W MSY1
ZShift = −2.94
Z lm Score = 1.96
NG = 0
DB = 0
SM = 0
Delta_r
Cytarabine
YPL097W MSY1
ZShift = −5.93
Z lm Score = −1.18
NG = 0
DB = 0
SM = 0
Delta_AUC
Cytarabine
YER014C−A BUD25
ZShift = 1.19
Z lm Score = −2.88
NG = 0
DB = 0
SM = 0
Delta_L
Cytarabine
YER014C−A BUD25
ZShift = −0.8
Z lm Score = 0.93
NG = 0
DB = 0
SM = 0
Delta_K
Cytarabine
YER014C−A BUD25
ZShift = −0.66
Z lm Score = 1.96
NG = 0
DB = 0
SM = 0
Delta_r
Cytarabine
YER014C−A BUD25
ZShift = −1.05
Z lm Score = 0.52
NG = 0
DB = 0
SM = 0
Delta_AUC
Cytarabine
YDR193W
ZShift = 2.46
Z lm Score = −2.89
NG = 0
DB = 0
SM = 0
Delta_L
Cytarabine
YDR193W
ZShift = 0.37
Z lm Score = 1.16
NG = 0
DB = 0
SM = 0
Delta_K
Cytarabine
YDR193W
ZShift = −1.64
Z lm Score = 2.48
NG = 0
DB = 0
SM = 0
Delta_r
Cytarabine
YDR193W
ZShift = −0.82
Z lm Score = 1.34
NG = 0
DB = 0
SM = 0
Delta_AUC
Cytarabine

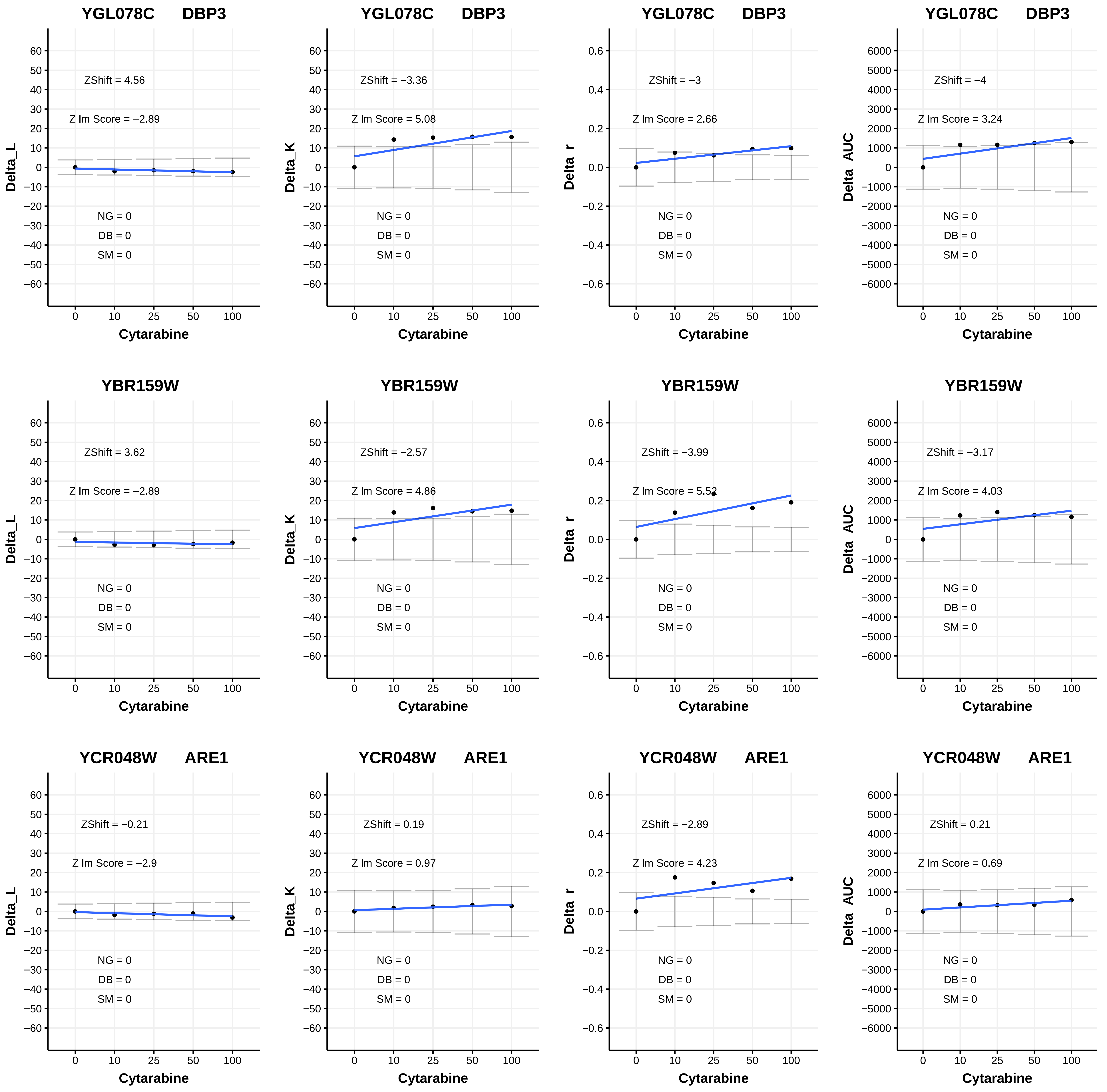
YGL078C DBP3
Delta_L
ZShift = 4.56
Z lm Score = −2.89
NG = 0
DB = 0
SM = 0
Cytarabine
YGL078C DBP3
Delta_K
ZShift = −3.36
Z lm Score = 5.08
NG = 0
DB = 0
SM = 0
Cytarabine
YGL078C DBP3
Delta_r
ZShift = −3
Z lm Score = 2.66
NG = 0
DB = 0
SM = 0
Cytarabine
YGL078C DBP3
Delta_AUC
ZShift = −4
Z lm Score = 3.24
NG = 0
DB = 0
SM = 0
Cytarabine
YBR159W
Delta_L
ZShift = 3.62
Z lm Score = −2.89
NG = 0
DB = 0
SM = 0
Cytarabine
YBR159W
Delta_K
ZShift = −2.57
Z lm Score = 4.86
NG = 0
DB = 0
SM = 0
Cytarabine
YBR159W
Delta_r
ZShift = −3.99
Z lm Score = 5.52
NG = 0
DB = 0
SM = 0
Cytarabine
YBR159W
Delta_AUC
ZShift = −3.17
Z lm Score = 4.03
NG = 0
DB = 0
SM = 0
Cytarabine
YCR048W ARE1
Delta_L
ZShift = −0.21
Z lm Score = −2.9
NG = 0
DB = 0
SM = 0
Cytarabine
YCR048W ARE1
Delta_K
ZShift = 0.19
Z lm Score = 0.97
NG = 0
DB = 0
SM = 0
Cytarabine
YCR048W ARE1
Delta_r
ZShift = −2.89
Z lm Score = 4.23
NG = 0
DB = 0
SM = 0
Cytarabine
YCR048W ARE1
Delta_AUC
ZShift = 0.21
Z lm Score = 0.69
NG = 0
DB = 0
SM = 0
Cytarabine

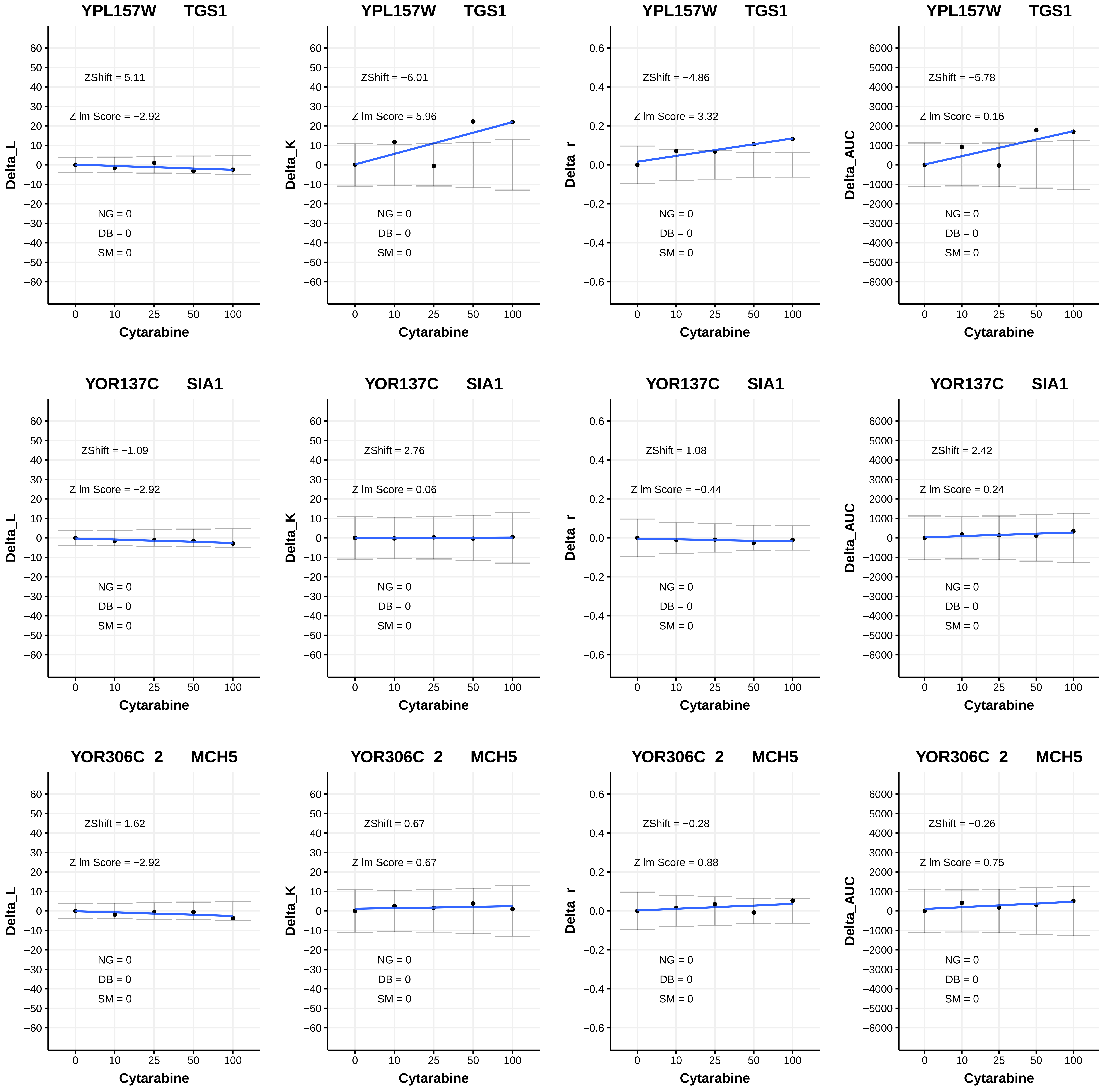

YPL157W TGS1
ZShift = 5.11
Z lm Score = −2.92
NG = 0
DB = 0
SM = 0
Delta_L
Cytarabine
YPL157W TGS1
ZShift = −6.01
Z lm Score = 5.96
NG = 0
DB = 0
SM = 0
Delta_K
Cytarabine
YPL157W TGS1
ZShift = −4.86
Z lm Score = 3.32
NG = 0
DB = 0
SM = 0
Delta_r
Cytarabine
YPL157W TGS1
ZShift = −5.78
Z lm Score = 0.16
NG = 0
DB = 0
SM = 0
Delta_AUC
Cytarabine
YOR137C SIA1
ZShift = −1.09
Z lm Score = −2.92
NG = 0
DB = 0
SM = 0
Delta_L
Cytarabine
YOR137C SIA1
ZShift = 2.76
Z lm Score = 0.06
NG = 0
DB = 0
SM = 0
Delta_K
Cytarabine
YOR137C SIA1
ZShift = 1.08
Z lm Score = −0.44
NG = 0
DB = 0
SM = 0
Delta_r
Cytarabine
YOR137C SIA1
ZShift = 2.42
Z lm Score = 0.24
NG = 0
DB = 0
SM = 0
Delta_AUC
Cytarabine
YOR306C_2 MCH5
ZShift = 1.62
Z lm Score = −2.92
NG = 0
DB = 0
SM = 0
Delta_L
Cytarabine
YOR306C_2 MCH5
ZShift = 0.67
Z lm Score = 0.67
NG = 0
DB = 0
SM = 0
Delta_K
Cytarabine
YOR306C_2 MCH5
ZShift = −0.28
Z lm Score = 0.88
NG = 0
DB = 0
SM = 0
Delta_r
Cytarabine
YOR306C_2 MCH5
ZShift = −0.26
Z lm Score = 0.75
NG = 0
DB = 0
SM = 0
Delta_AUC
Cytarabine

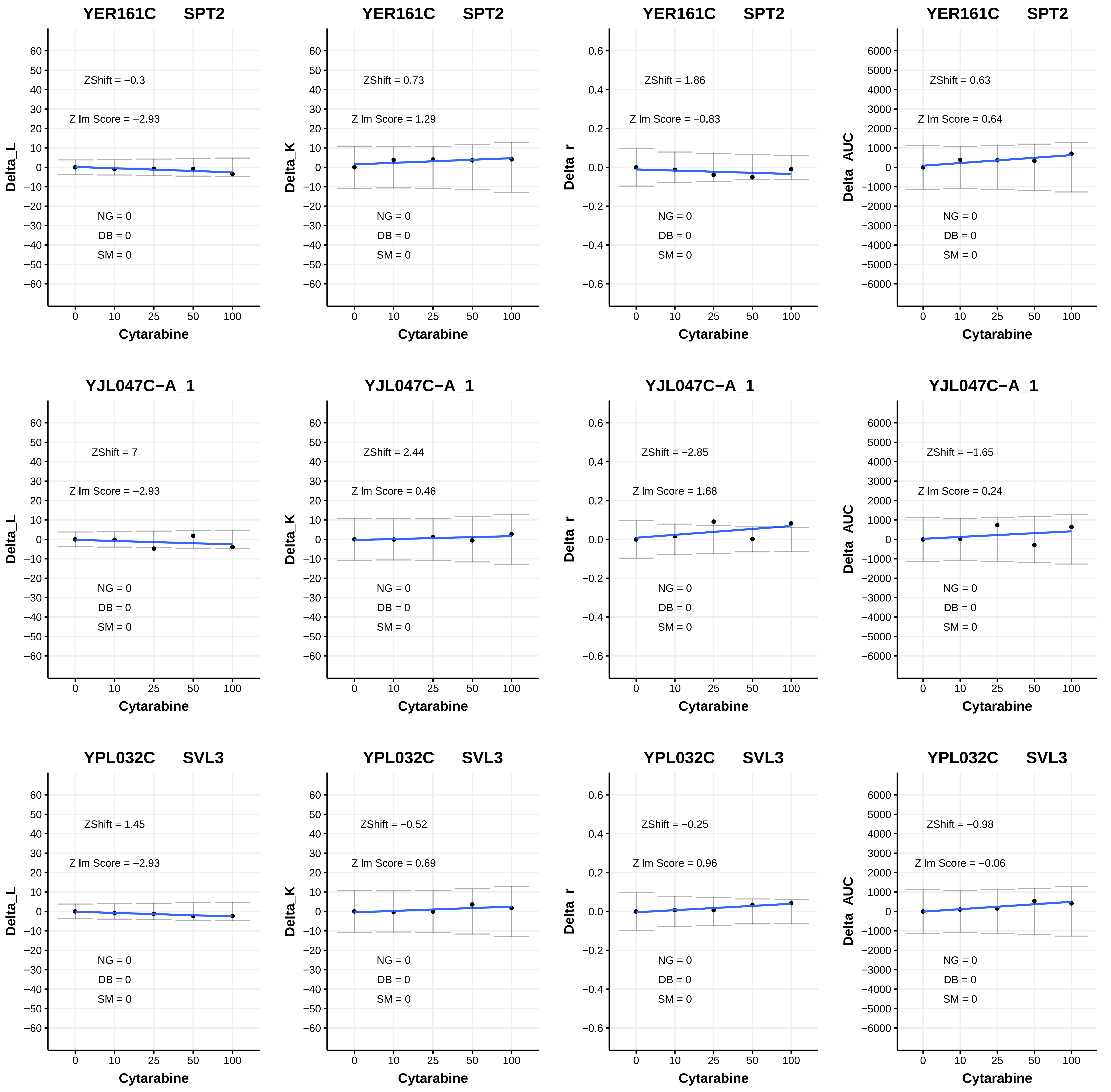
YER161C SPT2
ZShift = −0.3
Z lm Score = −2.93
NG = 0
DB = 0
SM = 0
Delta_L
Cytarabine
YER161C SPT2
ZShift = 0.73
Z lm Score = 1.29
NG = 0
DB = 0
SM = 0
Delta_K
Cytarabine
YER161C SPT2
ZShift = 1.86
Z lm Score = −0.83
NG = 0
DB = 0
SM = 0
Delta_r
Cytarabine
YER161C SPT2
ZShift = 0.63
Z lm Score = 0.64
NG = 0
DB = 0
SM = 0
Delta_AUC
Cytarabine
YJL047C−A_1
ZShift = 7
Z lm Score = −2.93
NG = 0
DB = 0
SM = 0
Delta_L
Cytarabine
YJL047C−A_1
ZShift = 2.44
Z lm Score = 0.46
NG = 0
DB = 0
SM = 0
Delta_K
Cytarabine
YJL047C−A_1
ZShift = −2.85
Z lm Score = 1.68
NG = 0
DB = 0
SM = 0
Delta_r
Cytarabine
YJL047C−A_1
ZShift = −1.65
Z lm Score = 0.24
NG = 0
DB = 0
SM = 0
Delta_AUC
Cytarabine
YPL032C SVL3
ZShift = 1.45
Z lm Score = −2.93
NG = 0
DB = 0
SM = 0
Delta_L
Cytarabine
YPL032C SVL3
ZShift = −0.52
Z lm Score = 0.69
NG = 0
DB = 0
SM = 0
Delta_K
Cytarabine
YPL032C SVL3
ZShift = −0.25
Z lm Score = 0.96
NG = 0
DB = 0
SM = 0
Delta_r
Cytarabine
YPL032C SVL3
ZShift = −0.98
Z lm Score = −0.06
NG = 0
DB = 0
SM = 0
Delta_AUC
Cytarabine

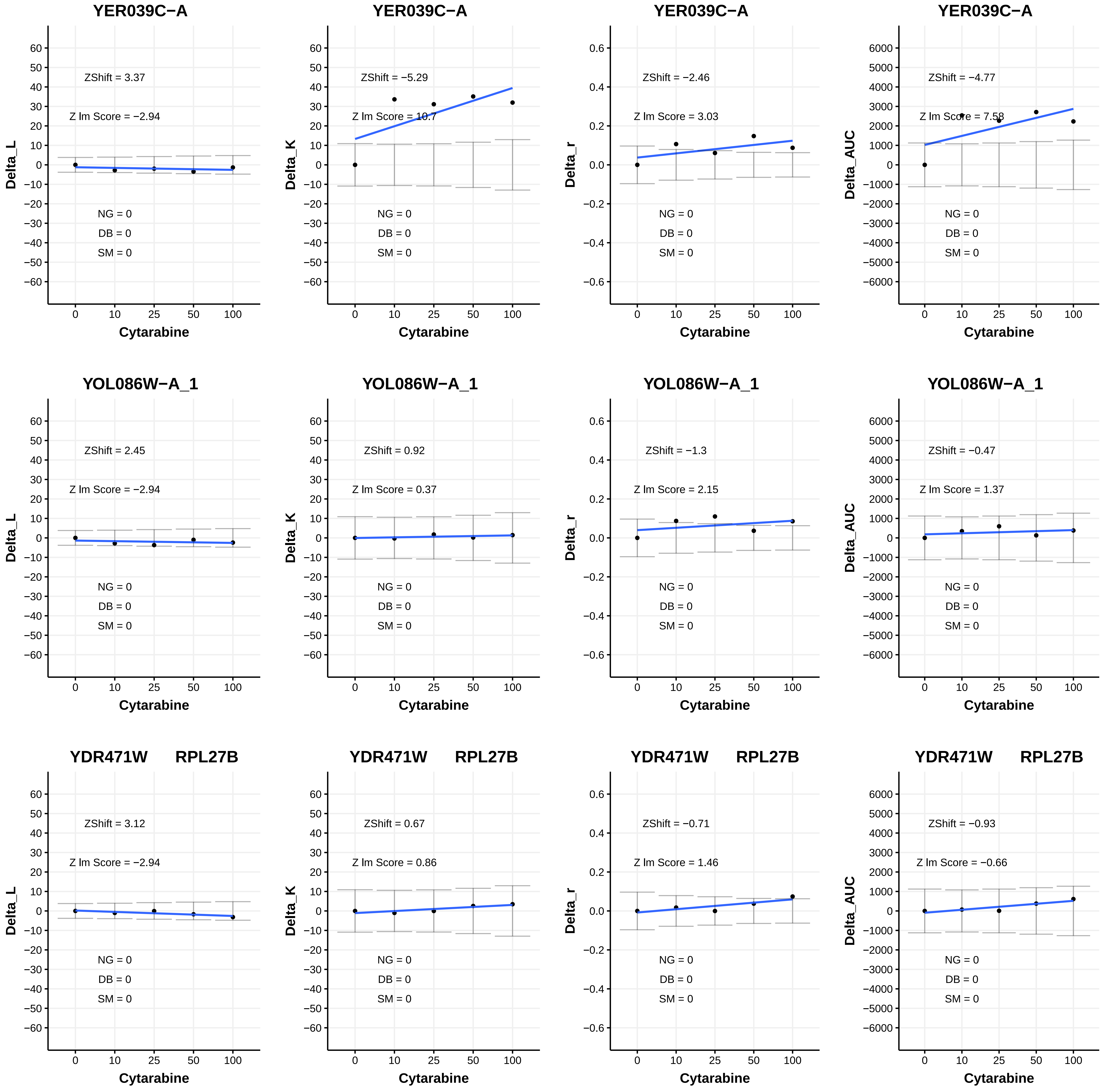

YER039C−A
ZShift = 3.37
Z lm Score = −2.94
NG = 0
DB = 0
SM = 0
Delta_L
Cytarabine
YER039C−A
ZShift = −5.29
Z lm Score = 10.7
NG = 0
DB = 0
SM = 0
Delta_K
Cytarabine
YER039C−A
ZShift = −2.46
Z lm Score = 3.03
NG = 0
DB = 0
SM = 0
Delta_r
Cytarabine
YER039C−A
ZShift = −4.77
Z lm Score = 7.58
NG = 0
DB = 0
SM = 0
Delta_AUC
Cytarabine
YOL086W−A_1
ZShift = 2.45
Z lm Score = −2.94
NG = 0
DB = 0
SM = 0
Delta_L
Cytarabine
YOL086W−A_1
ZShift = 0.92
Z lm Score = 0.37
NG = 0
DB = 0
SM = 0
Delta_K
Cytarabine
YOL086W−A_1
ZShift = −1.3
Z lm Score = 2.15
NG = 0
DB = 0
SM = 0
Delta_r
Cytarabine
YOL086W−A_1
ZShift = −0.47
Z lm Score = 1.37
NG = 0
DB = 0
SM = 0
Delta_AUC
Cytarabine
YDR471W RPL27B
ZShift = 3.12
Z lm Score = −2.94
NG = 0
DB = 0
SM = 0
Delta_L
Cytarabine
YDR471W RPL27B
ZShift = 0.67
Z lm Score = 0.86
NG = 0
DB = 0
SM = 0
Delta_K
Cytarabine
YDR471W RPL27B
ZShift = −0.71
Z lm Score = 1.46
NG = 0
DB = 0
SM = 0
Delta_r
Cytarabine
YDR471W RPL27B
ZShift = −0.93
Z lm Score = −0.66
NG = 0
DB = 0
SM = 0
Delta_AUC
Cytarabine

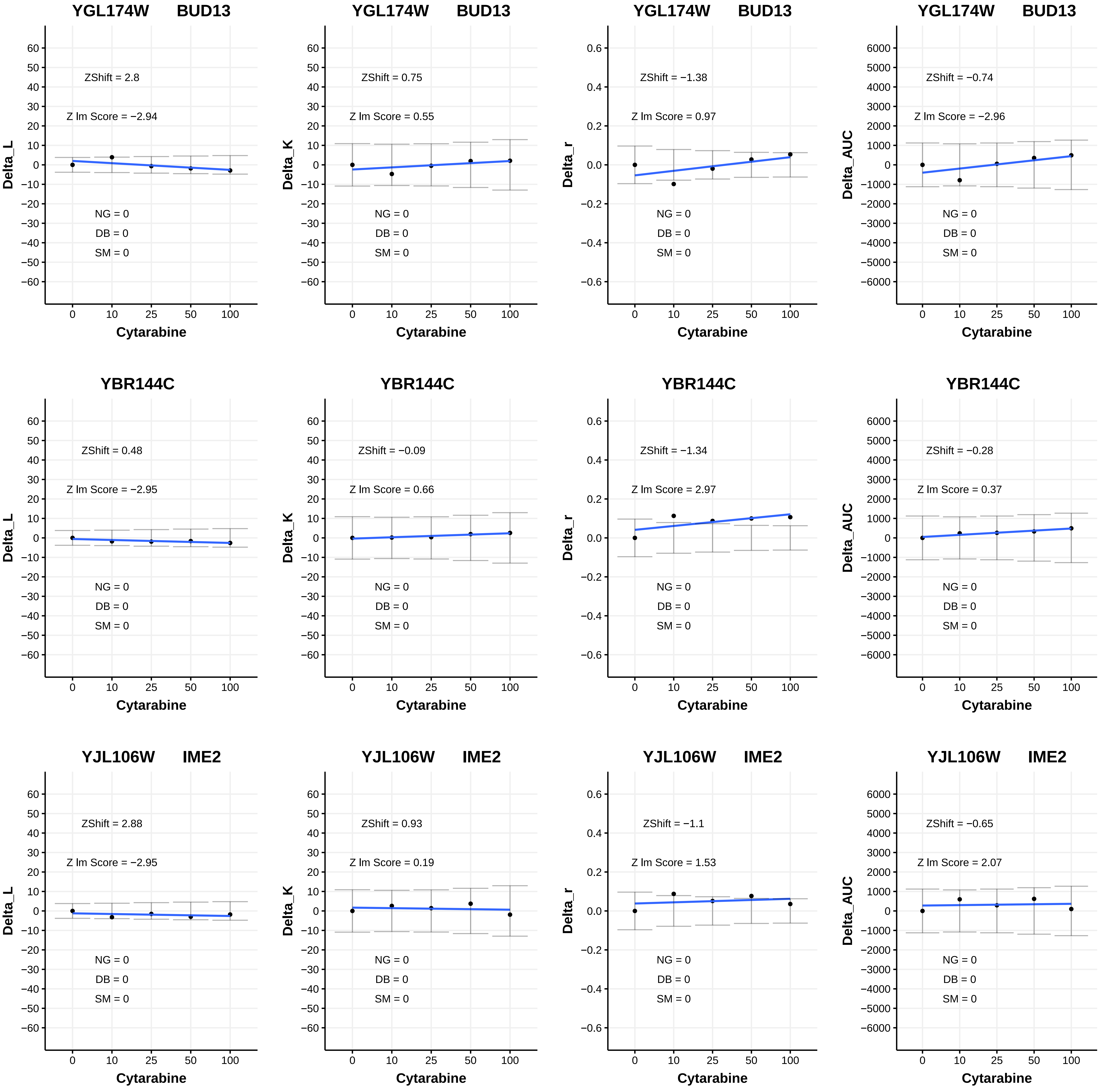
YGL174W BUD13
ZShift = 2.8
Z lm Score = −2.94
NG = 0
DB = 0
SM = 0
Delta_L
0 10 25 50 100
Cytarabine
YGL174W BUD13
ZShift = 0.75
Z lm Score = 0.55
NG = 0
DB = 0
SM = 0
Delta_K
0 10 25 50 100
Cytarabine
YGL174W BUD13
ZShift = −1.38
Z lm Score = 0.97
NG = 0
DB = 0
SM = 0
Delta_r
0 10 25 50 100
Cytarabine
YGL174W BUD13
ZShift = −0.74
Z lm Score = −2.96
NG = 0
DB = 0
SM = 0
Delta_AUC
0 10 25 50 100
Cytarabine
YBR144C
ZShift = 0.48
Z lm Score = −2.95
NG = 0
DB = 0
SM = 0
Delta_L
0 10 25 50 100
Cytarabine
YBR144C
ZShift = −0.09
Z lm Score = 0.66
NG = 0
DB = 0
SM = 0
Delta_K
0 10 25 50 100
Cytarabine
YBR144C
ZShift = −1.34
Z lm Score = 2.97
NG = 0
DB = 0
SM = 0
Delta_r
0 10 25 50 100
Cytarabine
YBR144C
ZShift = −0.28
Z lm Score = 0.37
NG = 0
DB = 0
SM = 0
Delta_AUC
0 10 25 50 100
Cytarabine
YJL106W IME2
ZShift = 2.88
Z lm Score = −2.95
NG = 0
DB = 0
SM = 0
Delta_L
0 10 25 50 100
Cytarabine
YJL106W IME2
ZShift = 0.93
Z lm Score = 0.19
NG = 0
DB = 0
SM = 0
Delta_K
0 10 25 50 100
Cytarabine
YJL106W IME2
ZShift = −1.1
Z lm Score = 1.53
NG = 0
DB = 0
SM = 0
Delta_r
0 10 25 50 100
Cytarabine
YJL106W IME2
ZShift = −0.65
Z lm Score = 2.07
NG = 0
DB = 0
SM = 0
Delta_AUC
0 10 25 50 100
Cytarabine

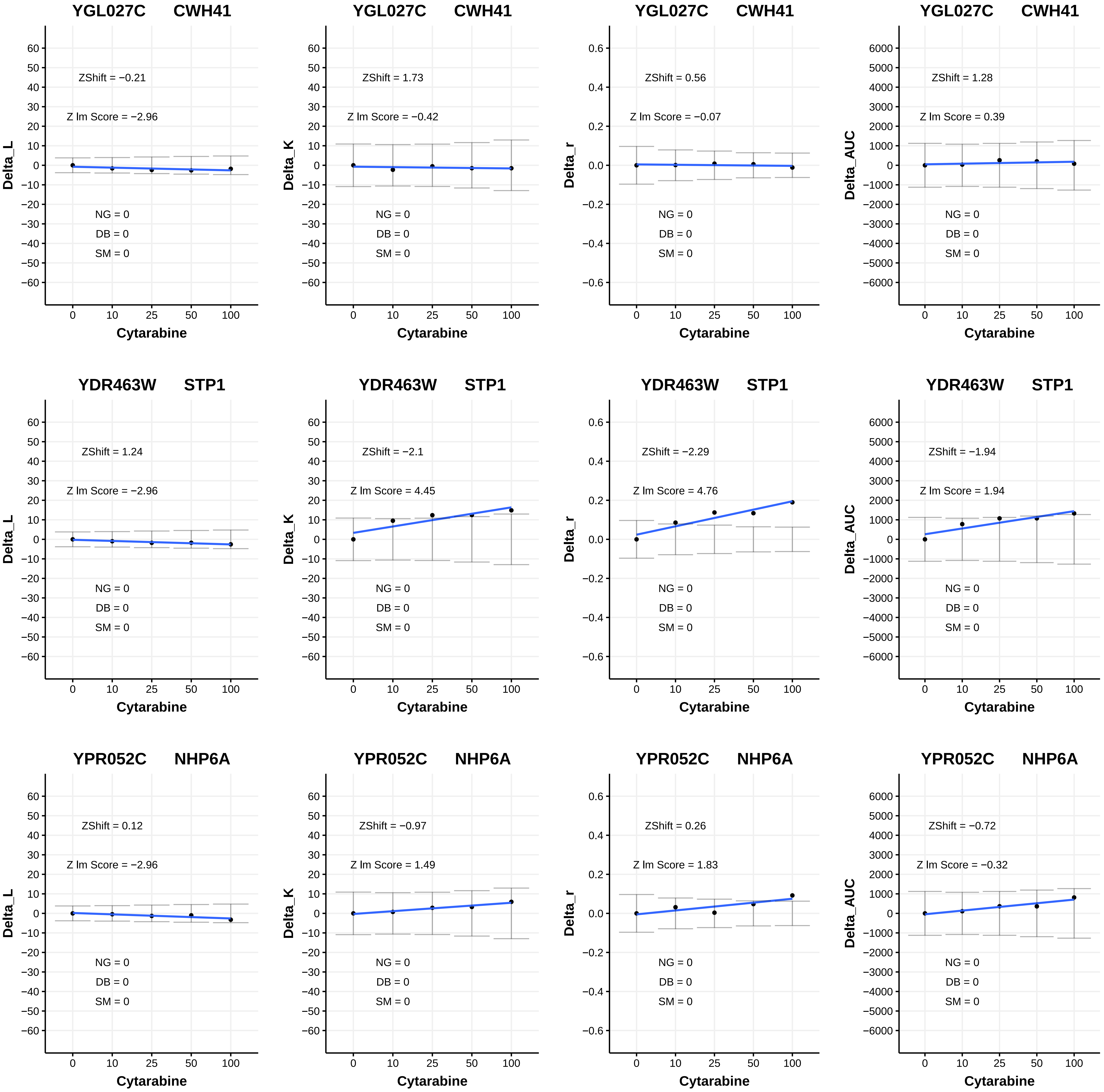

YGL027C CWH41
ZShift = −0.21
Z lm Score = −2.96
NG = 0
DB = 0
SM = 0
Delta_L
Cytarabine
YGL027C CWH41
ZShift = 1.73
Z lm Score = −0.42
NG = 0
DB = 0
SM = 0
Delta_K
Cytarabine
YGL027C CWH41
ZShift = 0.56
Z lm Score = −0.07
NG = 0
DB = 0
SM = 0
Delta_r
Cytarabine
YGL027C CWH41
ZShift = 1.28
Z lm Score = 0.39
NG = 0
DB = 0
SM = 0
Delta_AUC
Cytarabine
YDR463W STP1
ZShift = 1.24
Z lm Score = −2.96
NG = 0
DB = 0
SM = 0
Delta_L
Cytarabine
YDR463W STP1
ZShift = −2.1
Z lm Score = 4.45
NG = 0
DB = 0
SM = 0
Delta_K
Cytarabine
YDR463W STP1
ZShift = −2.29
Z lm Score = 4.76
NG = 0
DB = 0
SM = 0
Delta_r
Cytarabine
YDR463W STP1
ZShift = −1.94
Z lm Score = 1.94
NG = 0
DB = 0
SM = 0
Delta_AUC
Cytarabine
YPR052C NHP6A
ZShift = 0.12
Z lm Score = −2.96
NG = 0
DB = 0
SM = 0
Delta_L
Cytarabine
YPR052C NHP6A
ZShift = −0.97
Z lm Score = 1.49
NG = 0
DB = 0
SM = 0
Delta_K
Cytarabine
YPR052C NHP6A
ZShift = 0.26
Z lm Score = 1.83
NG = 0
DB = 0
SM = 0
Delta_r
Cytarabine
YPR052C NHP6A
ZShift = −0.72
Z lm Score = −0.32
NG = 0
DB = 0
SM = 0
Delta_AUC
Cytarabine

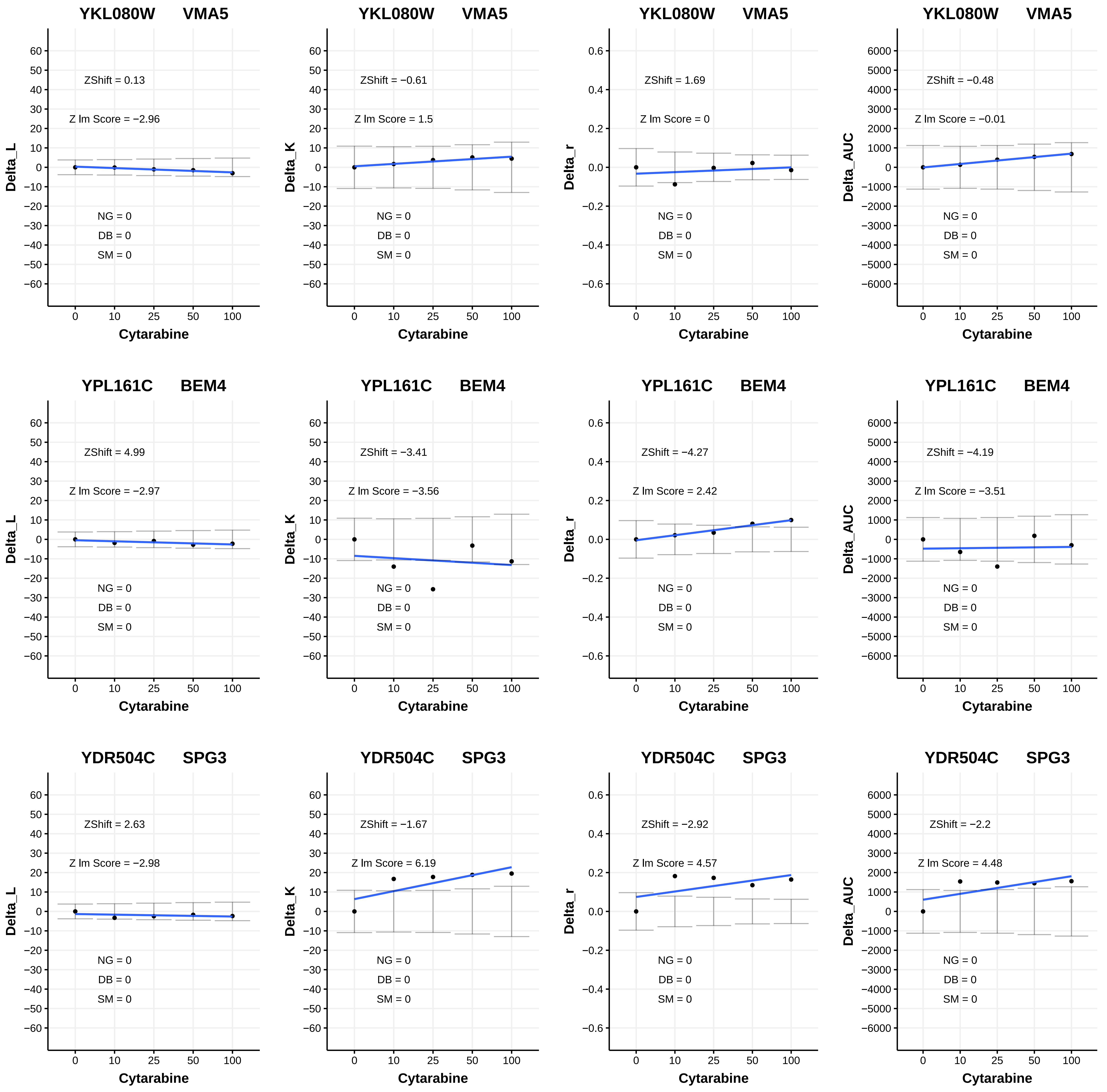
YKL080W VMA5
ZShift = 0.13
Z lm Score = −2.96
NG = 0
DB = 0
SM = 0
Delta_L
Cytarabine
YKL080W VMA5
ZShift = −0.61
Z lm Score = 1.5
NG = 0
DB = 0
SM = 0
Delta_K
Cytarabine
YKL080W VMA5
ZShift = 1.69
Z lm Score = 0
NG = 0
DB = 0
SM = 0
Delta_r
Cytarabine
YKL080W VMA5
ZShift = −0.48
Z lm Score = −0.01
NG = 0
DB = 0
SM = 0
Delta_AUC
Cytarabine
YPL161C BEM4
ZShift = 4.99
Z lm Score = −2.97
NG = 0
DB = 0
SM = 0
Delta_L
Cytarabine
YPL161C BEM4
ZShift = −3.41
Z lm Score = −3.56
NG = 0
DB = 0
SM = 0
Delta_K
Cytarabine
YPL161C BEM4
ZShift = −4.27
Z lm Score = 2.42
NG = 0
DB = 0
SM = 0
Delta_r
Cytarabine
YPL161C BEM4
ZShift = −4.19
Z lm Score = −3.51
NG = 0
DB = 0
SM = 0
Delta_AUC
Cytarabine
YDR504C SPG3
ZShift = 2.63
Z lm Score = −2.98
NG = 0
DB = 0
SM = 0
Delta_L
Cytarabine
YDR504C SPG3
ZShift = −1.67
Z lm Score = 6.19
NG = 0
DB = 0
SM = 0
Delta_K
Cytarabine
YDR504C SPG3
ZShift = −2.92
Z lm Score = 4.57
NG = 0
DB = 0
SM = 0
Delta_r
Cytarabine
YDR504C SPG3
ZShift = −2.2
Z lm Score = 4.48
NG = 0
DB = 0
SM = 0
Delta_AUC
Cytarabine

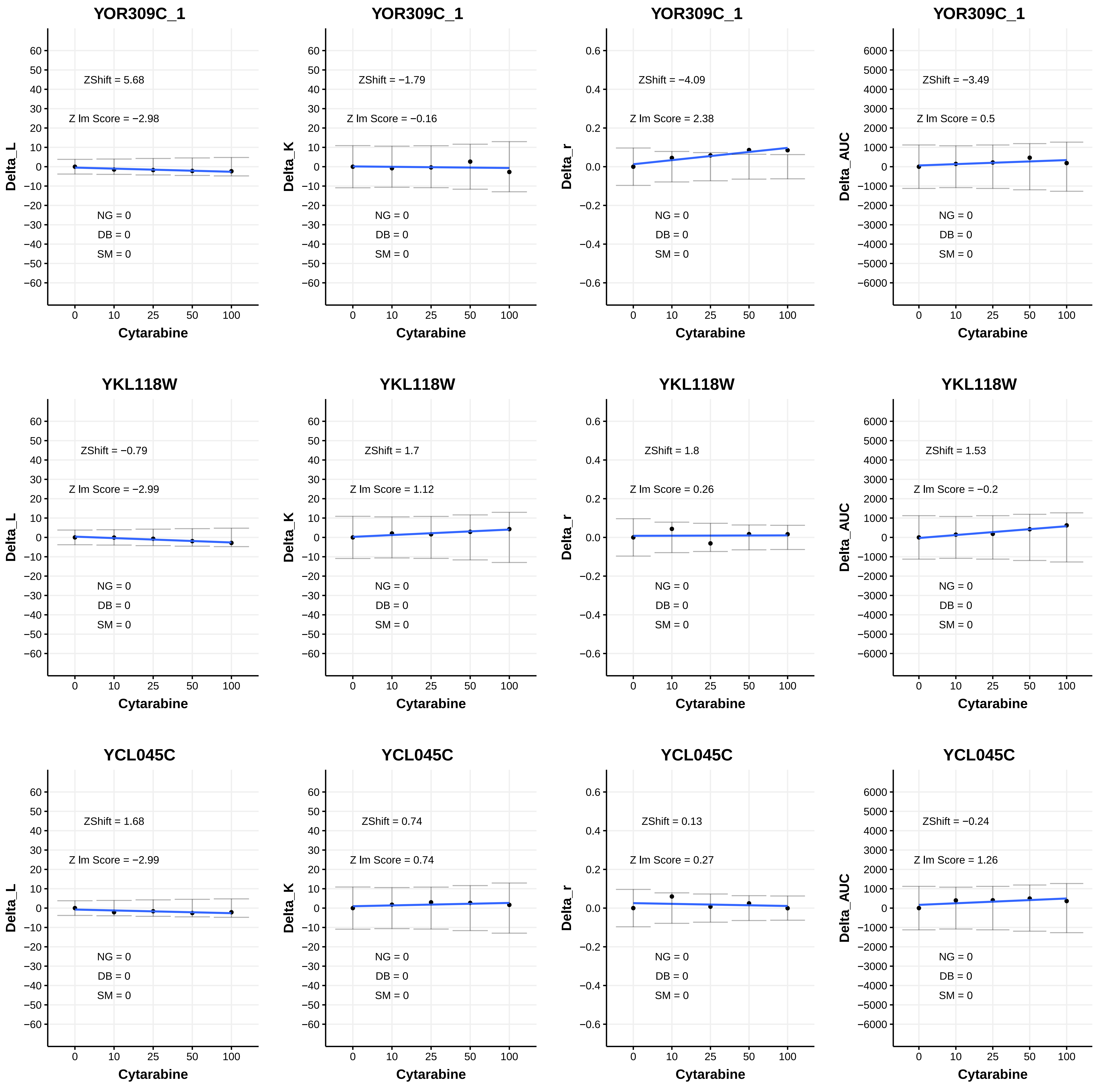
YOR309C_1
Delta_L
ZShift = 5.68
Z lm Score = −2.98
NG = 0
DB = 0
SM = 0
Cytarabine
YOR309C_1
Delta_K
ZShift = −1.79
Z lm Score = −0.16
NG = 0
DB = 0
SM = 0
Cytarabine
YOR309C_1
Delta_r
ZShift = −4.09
Z lm Score = 2.38
NG = 0
DB = 0
SM = 0
Cytarabine
YOR309C_1
Delta_AUC
ZShift = −3.49
Z lm Score = 0.5
NG = 0
DB = 0
SM = 0
Cytarabine
YKL118W
Delta_L
ZShift = −0.79
Z lm Score = −2.99
NG = 0
DB = 0
SM = 0
Cytarabine
YKL118W
Delta_K
ZShift = 1.7
Z lm Score = 1.12
NG = 0
DB = 0
SM = 0
Cytarabine
YKL118W
Delta_r
ZShift = 1.8
Z lm Score = 0.26
NG = 0
DB = 0
SM = 0
Cytarabine
YKL118W
Delta_AUC
ZShift = 1.53
Z lm Score = −0.2
NG = 0
DB = 0
SM = 0
Cytarabine
YCL045C
Delta_L
ZShift = 1.68
Z lm Score = −2.99
NG = 0
DB = 0
SM = 0
Cytarabine
YCL045C
Delta_K
ZShift = 0.74
Z lm Score = 0.74
NG = 0
DB = 0
SM = 0
Cytarabine
YCL045C
Delta_r
ZShift = 0.13
Z lm Score = 0.27
NG = 0
DB = 0
SM = 0
Cytarabine
YCL045C
Delta_AUC
ZShift = −0.24
Z lm Score = 1.26
NG = 0
DB = 0
SM = 0
Cytarabine

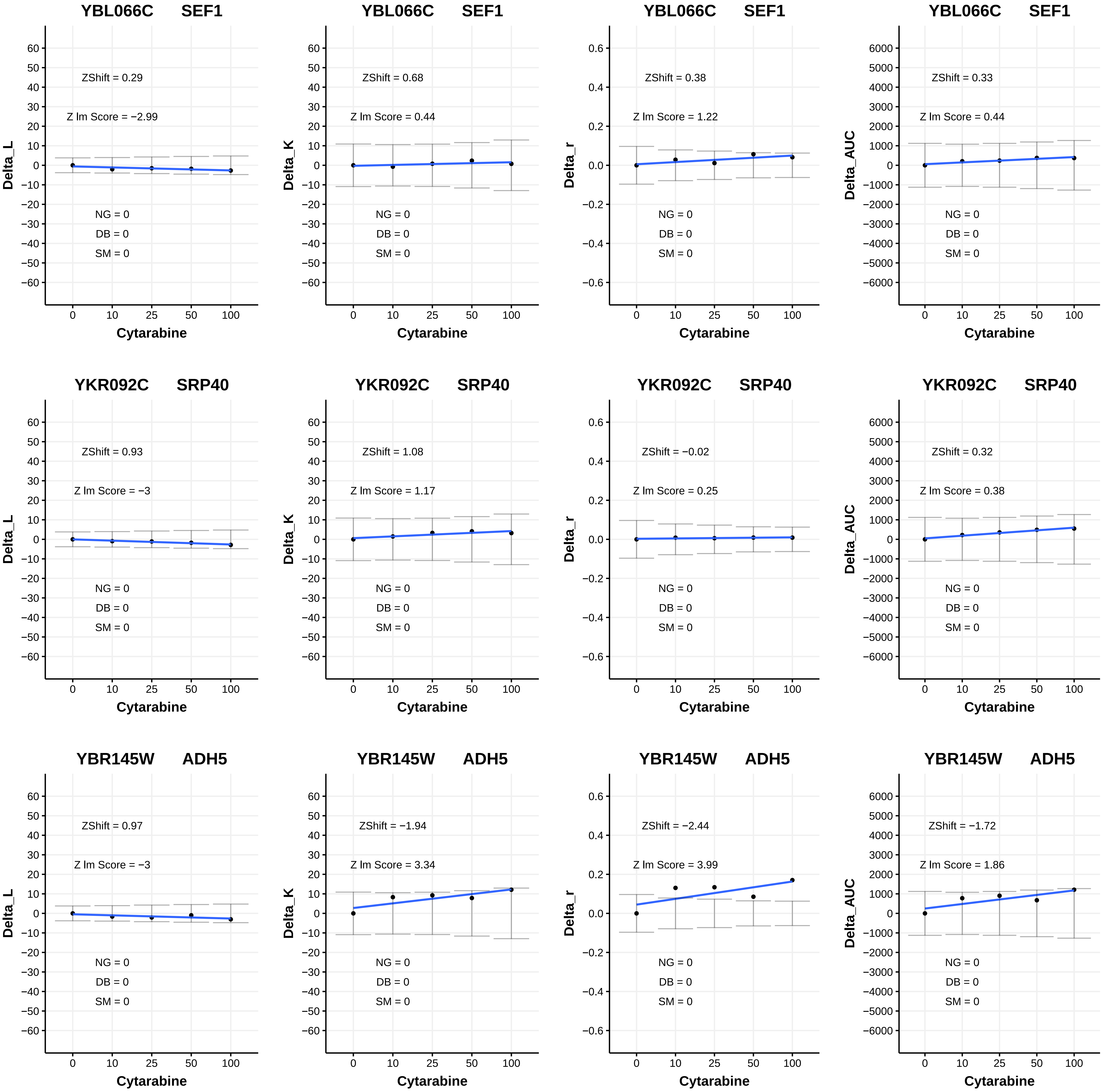
YBL066C SEF1
ZShift = 0.29
Z lm Score = −2.99
NG = 0
DB = 0
SM = 0
Delta_L
Cytarabine
YBL066C SEF1
ZShift = 0.68
Z lm Score = 0.44
NG = 0
DB = 0
SM = 0
Delta_K
Cytarabine
YBL066C SEF1
ZShift = 0.38
Z lm Score = 1.22
NG = 0
DB = 0
SM = 0
Delta_r
Cytarabine
YBL066C SEF1
ZShift = 0.33
Z lm Score = 0.44
NG = 0
DB = 0
SM = 0
Delta_AUC
Cytarabine
YKR092C SRP40
ZShift = 0.93
Z lm Score = −3
NG = 0
DB = 0
SM = 0
Delta_L
Cytarabine
YKR092C SRP40
ZShift = 1.08
Z lm Score = 1.17
NG = 0
DB = 0
SM = 0
Delta_K
Cytarabine
YKR092C SRP40
ZShift = −0.02
Z lm Score = 0.25
NG = 0
DB = 0
SM = 0
Delta_r
Cytarabine
YKR092C SRP40
ZShift = 0.32
Z lm Score = 0.38
NG = 0
DB = 0
SM = 0
Delta_AUC
Cytarabine
YBR145W ADH5
ZShift = 0.97
Z lm Score = −3
NG = 0
DB = 0
SM = 0
Delta_L
Cytarabine
YBR145W ADH5
ZShift = −1.94
Z lm Score = 3.34
NG = 0
DB = 0
SM = 0
Delta_K
Cytarabine
YBR145W ADH5
ZShift = −2.44
Z lm Score = 3.99
NG = 0
DB = 0
SM = 0
Delta_r
Cytarabine
YBR145W ADH5
ZShift = −1.72
Z lm Score = 1.86
NG = 0
DB = 0
SM = 0
Delta_AUC
Cytarabine

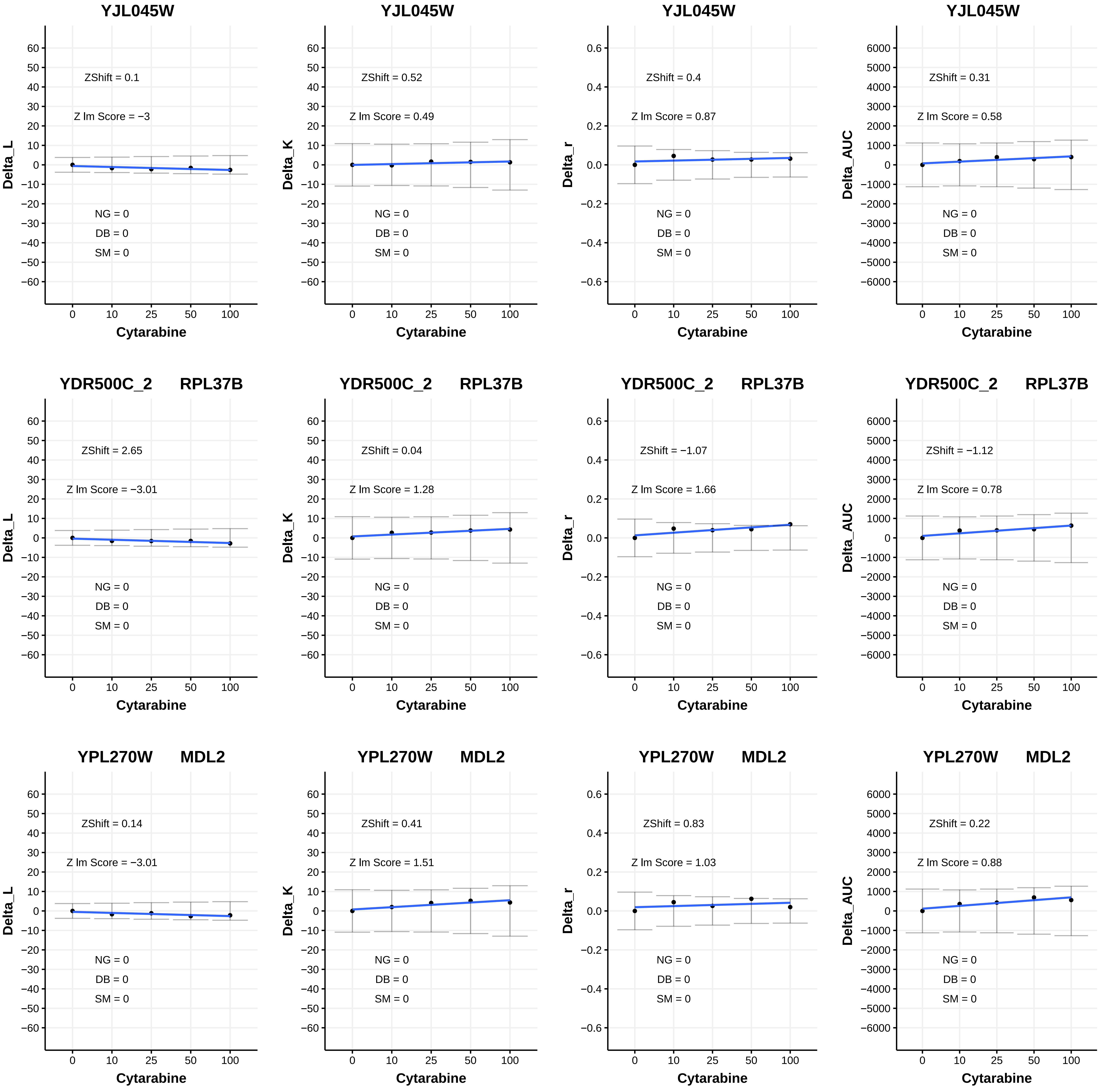

YJL045W
ZShift = 0.1
Z lm Score = −3
NG = 0
DB = 0
SM = 0
Delta_L
Cytarabine
YJL045W
ZShift = 0.52
Z lm Score = 0.49
NG = 0
DB = 0
SM = 0
Delta_K
Cytarabine
YJL045W
ZShift = 0.4
Z lm Score = 0.87
NG = 0
DB = 0
SM = 0
Delta_r
Cytarabine
YJL045W
ZShift = 0.31
Z lm Score = 0.58
NG = 0
DB = 0
SM = 0
Delta_AUC
Cytarabine
YDR500C_2 RPL37B
ZShift = 2.65
Z lm Score = −3.01
NG = 0
DB = 0
SM = 0
Delta_L
Cytarabine
YDR500C_2 RPL37B
ZShift = 0.04
Z lm Score = 1.28
NG = 0
DB = 0
SM = 0
Delta_K
Cytarabine
YDR500C_2 RPL37B
ZShift = −1.07
Z lm Score = 1.66
NG = 0
DB = 0
SM = 0
Delta_r
Cytarabine
YDR500C_2 RPL37B
ZShift = −1.12
Z lm Score = 0.78
NG = 0
DB = 0
SM = 0
Delta_AUC
Cytarabine
YPL270W MDL2
ZShift = 0.14
Z lm Score = −3.01
NG = 0
DB = 0
SM = 0
Delta_L
Cytarabine
YPL270W MDL2
ZShift = 0.41
Z lm Score = 1.51
NG = 0
DB = 0
SM = 0
Delta_K
Cytarabine
YPL270W MDL2
ZShift = 0.83
Z lm Score = 1.03
NG = 0
DB = 0
SM = 0
Delta_r
Cytarabine
YPL270W MDL2
ZShift = 0.22
Z lm Score = 0.88
NG = 0
DB = 0
SM = 0
Delta_AUC
Cytarabine

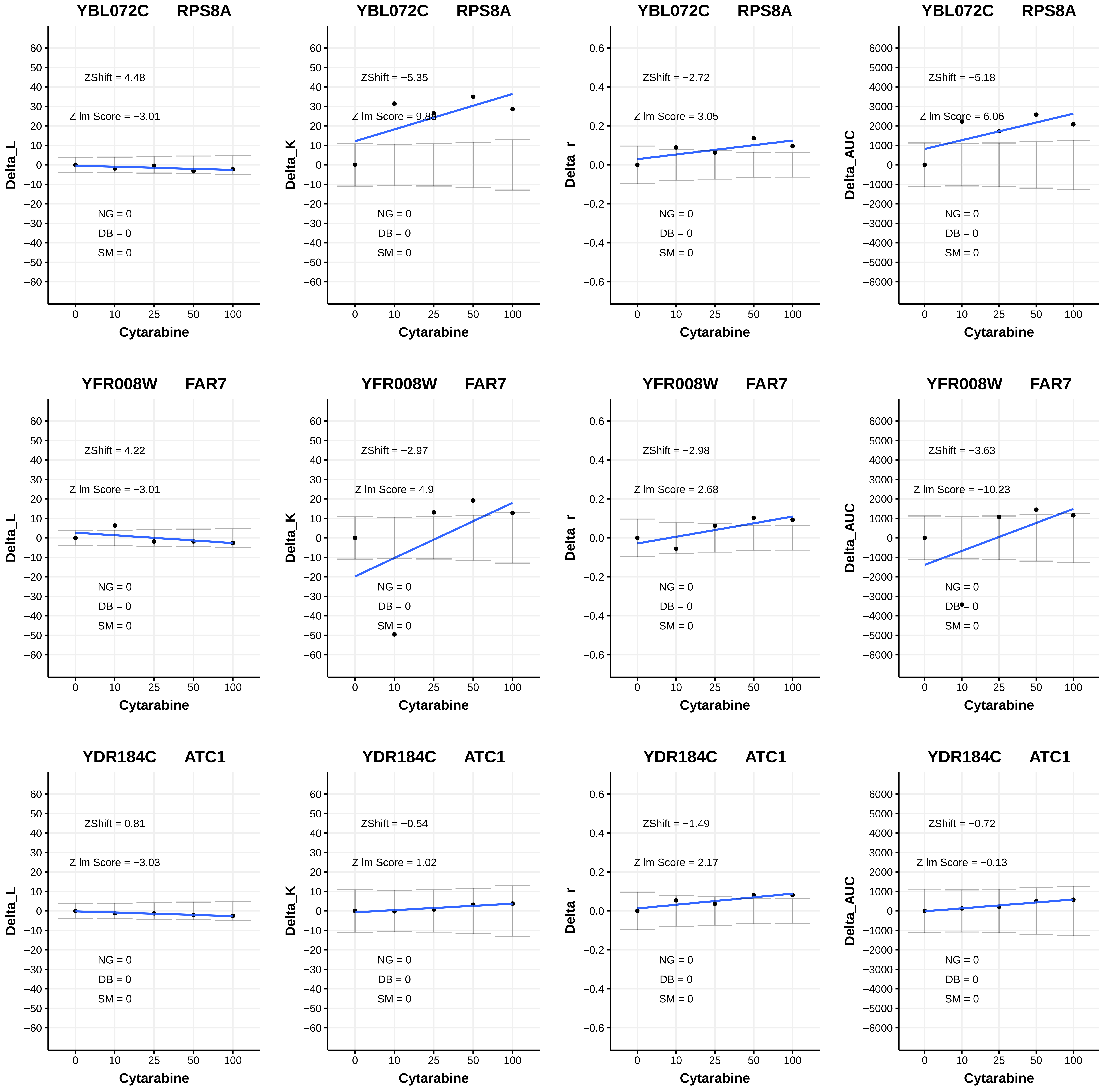

YBL072C RPS8A
ZShift = 4.48
Z lm Score = −3.01
NG = 0
DB = 0
SM = 0
Delta_L
Cytarabine
YBL072C RPS8A
ZShift = −5.35
Z lm Score = 9.88
NG = 0
DB = 0
SM = 0
Delta_K
Cytarabine
YBL072C RPS8A
ZShift = −2.72
Z lm Score = 3.05
NG = 0
DB = 0
SM = 0
Delta_r
Cytarabine
YBL072C RPS8A
ZShift = −5.18
Z lm Score = 6.06
NG = 0
DB = 0
SM = 0
Delta_AUC
Cytarabine
YFR008W FAR7
ZShift = 4.22
Z lm Score = −3.01
NG = 0
DB = 0
SM = 0
Delta_L
Cytarabine
YFR008W FAR7
ZShift = −2.97
Z lm Score = 4.9
NG = 0
DB = 0
SM = 0
Delta_K
Cytarabine
YFR008W FAR7
ZShift = −2.98
Z lm Score = 2.68
NG = 0
DB = 0
SM = 0
Delta_r
Cytarabine
YFR008W FAR7
ZShift = −3.63
Z lm Score = −10.23
NG = 0
DB = 0
SM = 0
Delta_AUC
Cytarabine
YDR184C ATC1
ZShift = 0.81
Z lm Score = −3.03
NG = 0
DB = 0
SM = 0
Delta_L
Cytarabine
YDR184C ATC1
ZShift = −0.54
Z lm Score = 1.02
NG = 0
DB = 0
SM = 0
Delta_K
Cytarabine
YDR184C ATC1
ZShift = −1.49
Z lm Score = 2.17
NG = 0
DB = 0
SM = 0
Delta_r
Cytarabine
YDR184C ATC1
ZShift = −0.72
Z lm Score = −0.13
NG = 0
DB = 0
SM = 0
Delta_AUC
Cytarabine

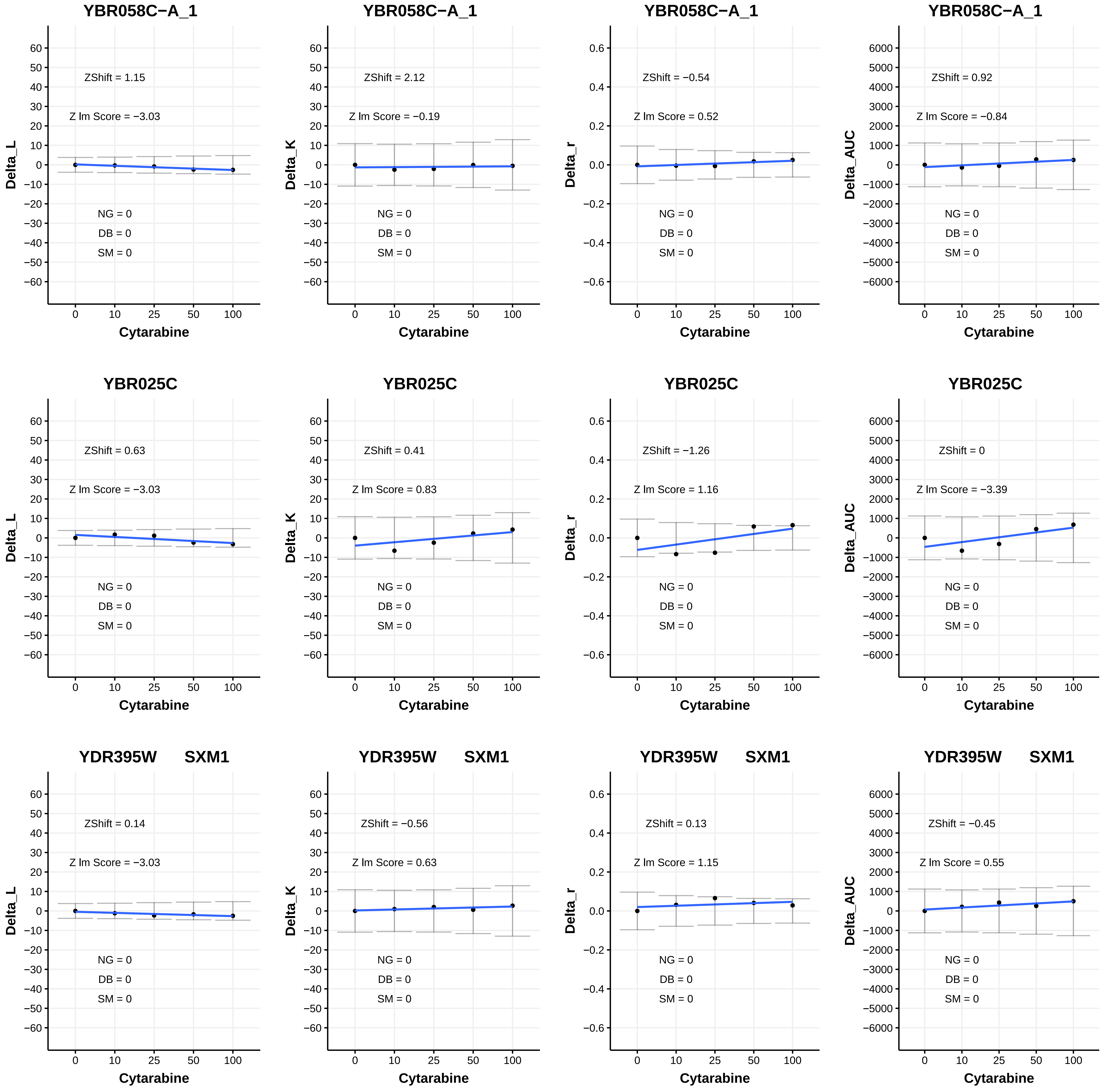
YBR058C−A_1
ZShift = 1.15
Z lm Score = −3.03
NG = 0
DB = 0
SM = 0
Delta_L
Cytarabine
YBR058C−A_1
ZShift = 2.12
Z lm Score = −0.19
NG = 0
DB = 0
SM = 0
Delta_K
Cytarabine
YBR058C−A_1
ZShift = −0.54
Z lm Score = 0.52
NG = 0
DB = 0
SM = 0
Delta_r
Cytarabine
YBR058C−A_1
ZShift = 0.92
Z lm Score = −0.84
NG = 0
DB = 0
SM = 0
Delta_AUC
Cytarabine
YBR025C
ZShift = 0.63
Z lm Score = −3.03
NG = 0
DB = 0
SM = 0
Delta_L
Cytarabine
YBR025C
ZShift = 0.41
Z lm Score = 0.83
NG = 0
DB = 0
SM = 0
Delta_K
Cytarabine
YBR025C
ZShift = −1.26
Z lm Score = 1.16
NG = 0
DB = 0
SM = 0
Delta_r
Cytarabine
YBR025C
ZShift = 0
Z lm Score = −3.39
NG = 0
DB = 0
SM = 0
Delta_AUC
Cytarabine
YDR395W SXM1
ZShift = 0.14
Z lm Score = −3.03
NG = 0
DB = 0
SM = 0
Delta_L
Cytarabine
YDR395W SXM1
ZShift = −0.56
Z lm Score = 0.63
NG = 0
DB = 0
SM = 0
Delta_K
Cytarabine
YDR395W SXM1
ZShift = 0.13
Z lm Score = 1.15
NG = 0
DB = 0
SM = 0
Delta_r
Cytarabine
YDR395W SXM1
ZShift = −0.45
Z lm Score = 0.55
NG = 0
DB = 0
SM = 0
Delta_AUC
Cytarabine

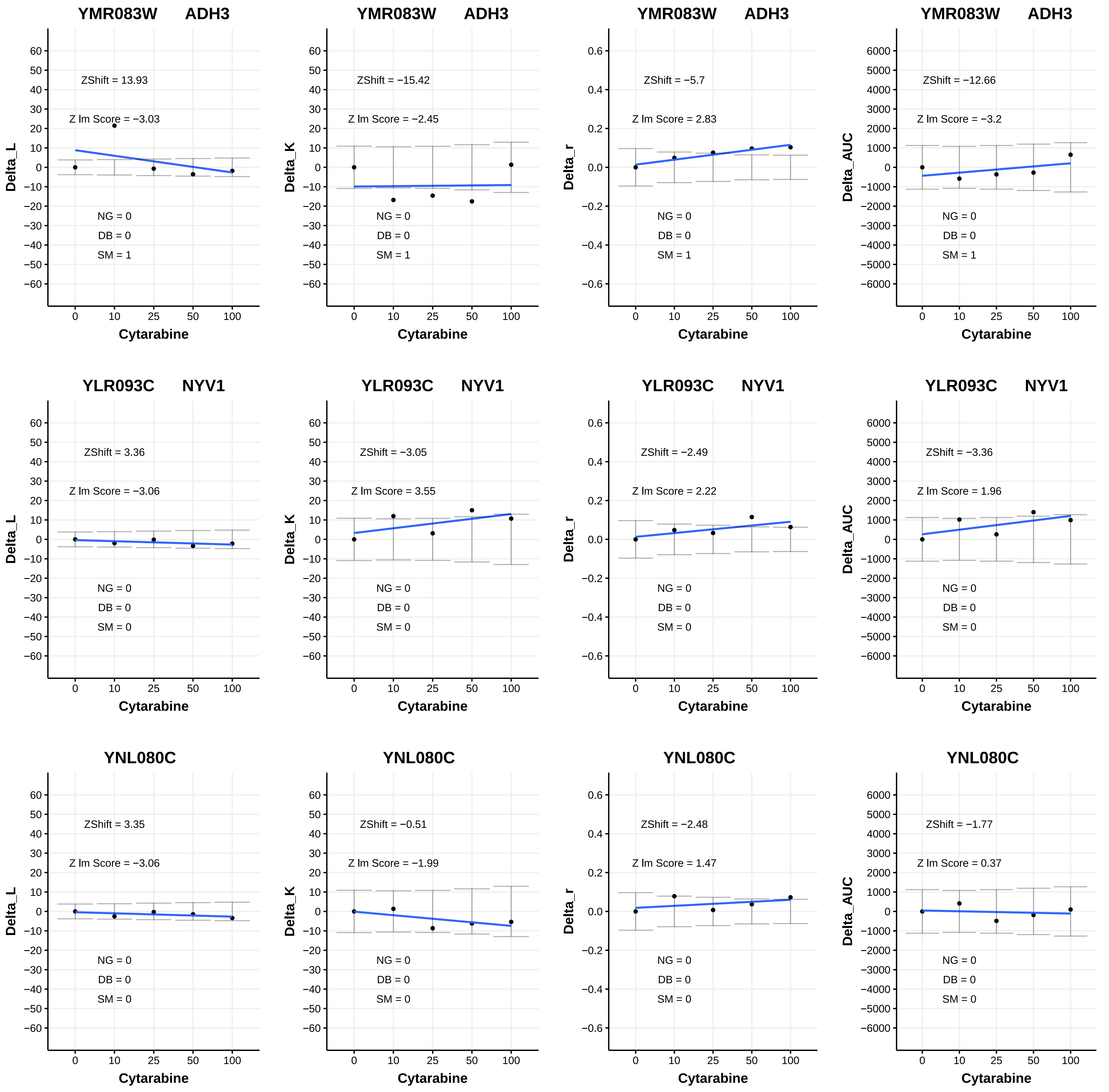

YMR083W ADH3
ZShift = 13.93
Z lm Score = −3.03
NG = 0
DB = 0
SM = 1
Delta_L
Cytarabine
YMR083W ADH3
ZShift = −15.42
Z lm Score = −2.45
NG = 0
DB = 0
SM = 1
Delta_K
Cytarabine
YMR083W ADH3
ZShift = −5.7
Z lm Score = 2.83
NG = 0
DB = 0
SM = 1
Delta_r
Cytarabine
YMR083W ADH3
ZShift = −12.66
Z lm Score = −3.2
NG = 0
DB = 0
SM = 1
Delta_AUC
Cytarabine
YLR093C NYV1
ZShift = 3.36
Z lm Score = −3.06
NG = 0
DB = 0
SM = 0
Delta_L
Cytarabine
YLR093C NYV1
ZShift = −3.05
Z lm Score = 3.55
NG = 0
DB = 0
SM = 0
Delta_K
Cytarabine
YLR093C NYV1
ZShift = −2.49
Z lm Score = 2.22
NG = 0
DB = 0
SM = 0
Delta_r
Cytarabine
YLR093C NYV1
ZShift = −3.36
Z lm Score = 1.96
NG = 0
DB = 0
SM = 0
Delta_AUC
Cytarabine
YNL080C
ZShift = 3.35
Z lm Score = −3.06
NG = 0
DB = 0
SM = 0
Delta_L
Cytarabine
YNL080C
ZShift = −0.51
Z lm Score = −1.99
NG = 0
DB = 0
SM = 0
Delta_K
Cytarabine
YNL080C
ZShift = −2.48
Z lm Score = 1.47
NG = 0
DB = 0
SM = 0
Delta_r
Cytarabine
YNL080C
ZShift = −1.77
Z lm Score = 0.37
NG = 0
DB = 0
SM = 0
Delta_AUC
Cytarabine

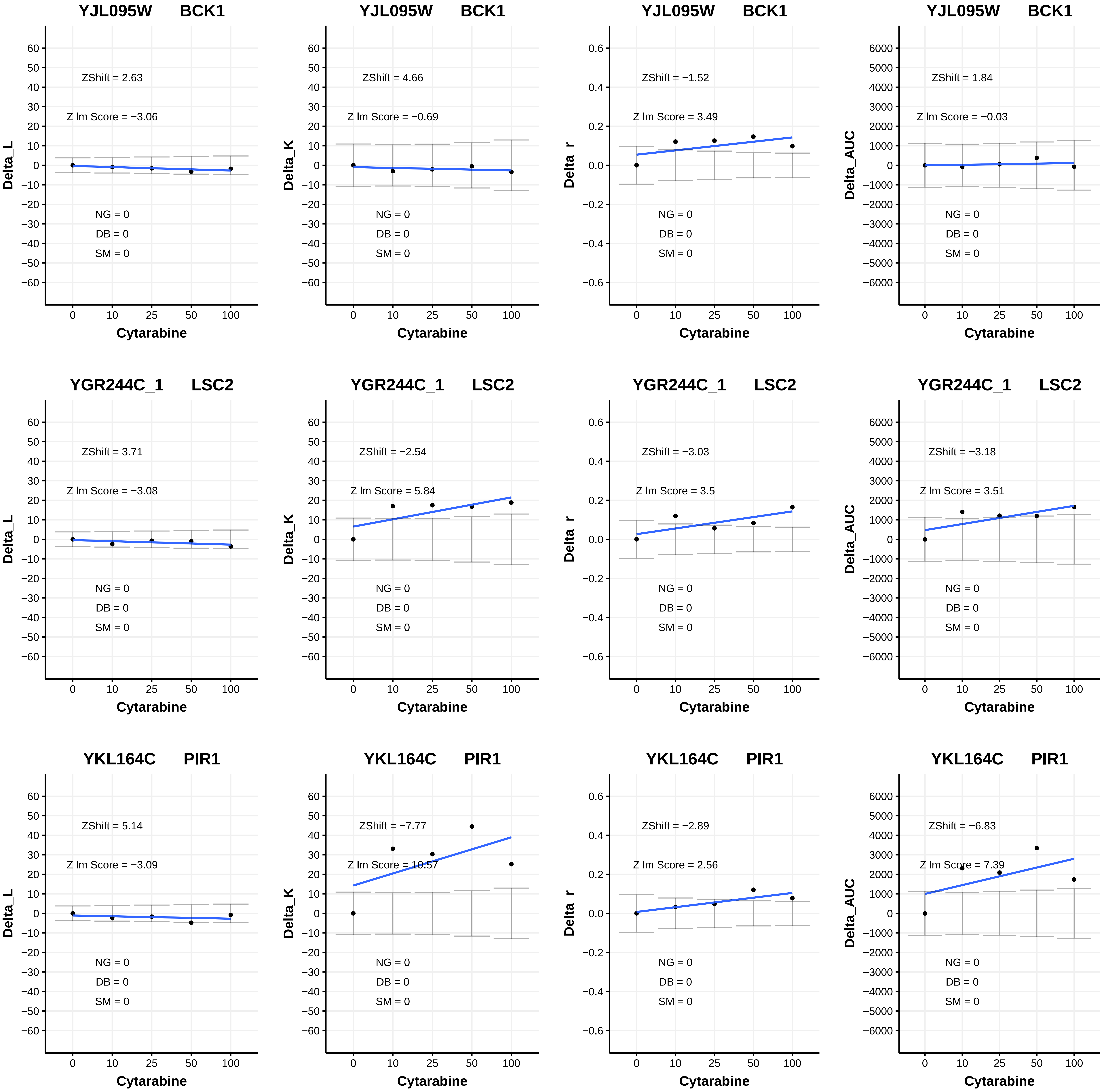
YJL095W BCK1
ZShift = 2.63
Z lm Score = −3.06
NG = 0
DB = 0
SM = 0
Delta_L
Cytarabine
YJL095W BCK1
ZShift = 4.66
Z lm Score = −0.69
NG = 0
DB = 0
SM = 0
Delta_K
Cytarabine
YJL095W BCK1
ZShift = −1.52
Z lm Score = 3.49
NG = 0
DB = 0
SM = 0
Delta_r
Cytarabine
YJL095W BCK1
ZShift = 1.84
Z lm Score = −0.03
NG = 0
DB = 0
SM = 0
Delta_AUC
Cytarabine
YGR244C_1 LSC2
ZShift = 3.71
Z lm Score = −3.08
NG = 0
DB = 0
SM = 0
Delta_L
Cytarabine
YGR244C_1 LSC2
ZShift = −2.54
Z lm Score = 5.84
NG = 0
DB = 0
SM = 0
Delta_K
Cytarabine
YGR244C_1 LSC2
ZShift = −3.03
Z lm Score = 3.5
NG = 0
DB = 0
SM = 0
Delta_r
Cytarabine
YGR244C_1 LSC2
ZShift = −3.18
Z lm Score = 3.51
NG = 0
DB = 0
SM = 0
Delta_AUC
Cytarabine
YKL164C PIR1
ZShift = 5.14
Z lm Score = −3.09
NG = 0
DB = 0
SM = 0
Delta_L
Cytarabine
YKL164C PIR1
ZShift = −7.77
Z lm Score = 10.57
NG = 0
DB = 0
SM = 0
Delta_K
Cytarabine
YKL164C PIR1
ZShift = −2.89
Z lm Score = 2.56
NG = 0
DB = 0
SM = 0
Delta_r
Cytarabine
YKL164C PIR1
ZShift = −6.83
Z lm Score = 7.39
NG = 0
DB = 0
SM = 0
Delta_AUC
Cytarabine

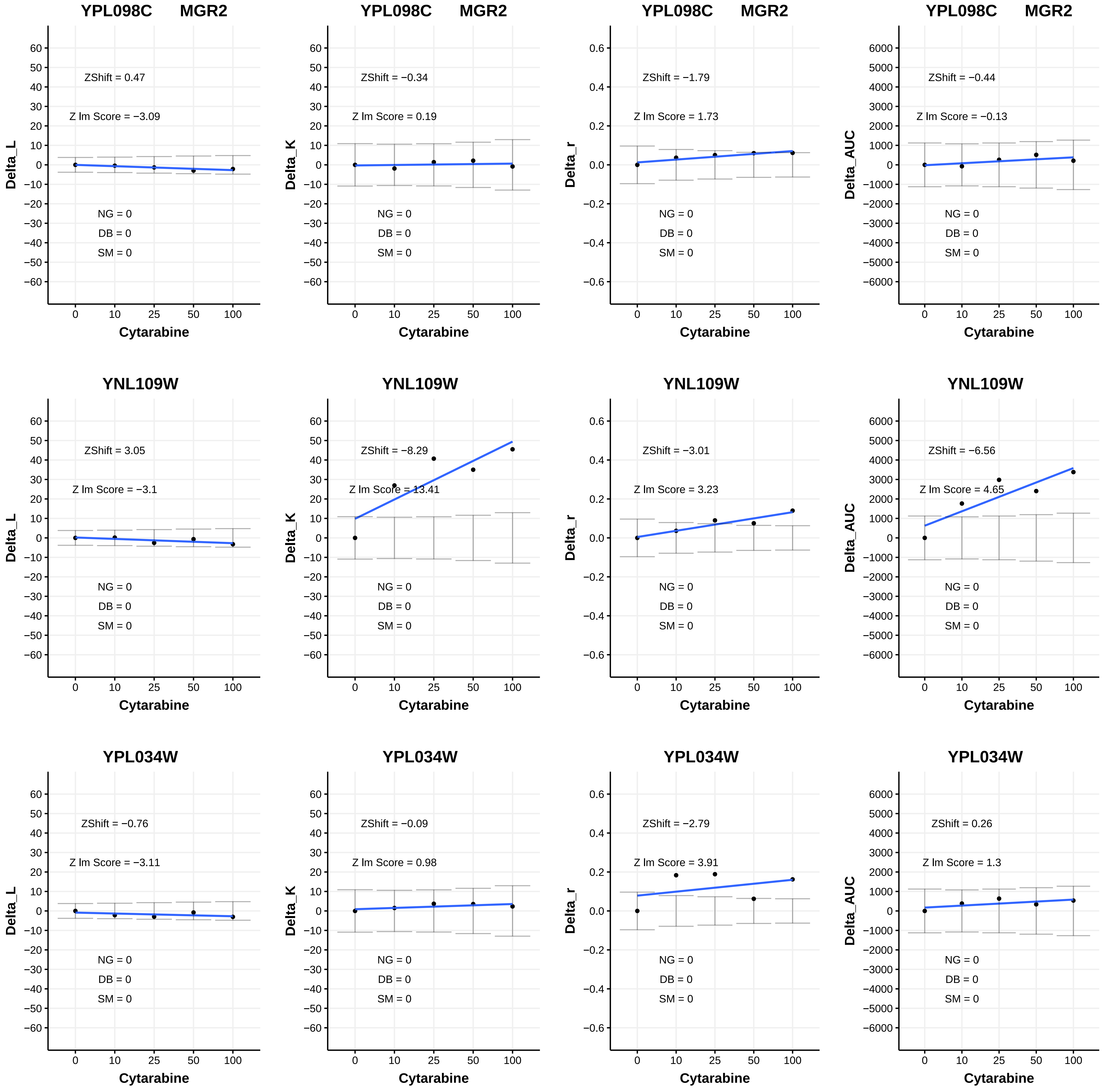
YPL098C MGR2
ZShift = 0.47
Z lm Score = −3.09
NG = 0
DB = 0
SM = 0
Delta_L
Cytarabine
YPL098C MGR2
ZShift = −0.34
Z lm Score = 0.19
NG = 0
DB = 0
SM = 0
Delta_K
Cytarabine
YPL098C MGR2
ZShift = −1.79
Z lm Score = 1.73
NG = 0
DB = 0
SM = 0
Delta_r
Cytarabine
YPL098C MGR2
ZShift = −0.44
Z lm Score = −0.13
NG = 0
DB = 0
SM = 0
Delta_AUC
Cytarabine
YNL109W
ZShift = 3.05
Z lm Score = −3.1
NG = 0
DB = 0
SM = 0
Delta_L
Cytarabine
YNL109W
ZShift = −8.29
Z lm Score = 13.41
NG = 0
DB = 0
SM = 0
Delta_K
Cytarabine
YNL109W
ZShift = −3.01
Z lm Score = 3.23
NG = 0
DB = 0
SM = 0
Delta_r
Cytarabine
YNL109W
ZShift = −6.56
Z lm Score = 4.65
NG = 0
DB = 0
SM = 0
Delta_AUC
Cytarabine
YPL034W
ZShift = −0.76
Z lm Score = −3.11
NG = 0
DB = 0
SM = 0
Delta_L
Cytarabine
YPL034W
ZShift = −0.09
Z lm Score = 0.98
NG = 0
DB = 0
SM = 0
Delta_K
Cytarabine
YPL034W
ZShift = −2.79
Z lm Score = 3.91
NG = 0
DB = 0
SM = 0
Delta_r
Cytarabine
YPL034W
ZShift = 0.26
Z lm Score = 1.3
NG = 0
DB = 0
SM = 0
Delta_AUC
Cytarabine

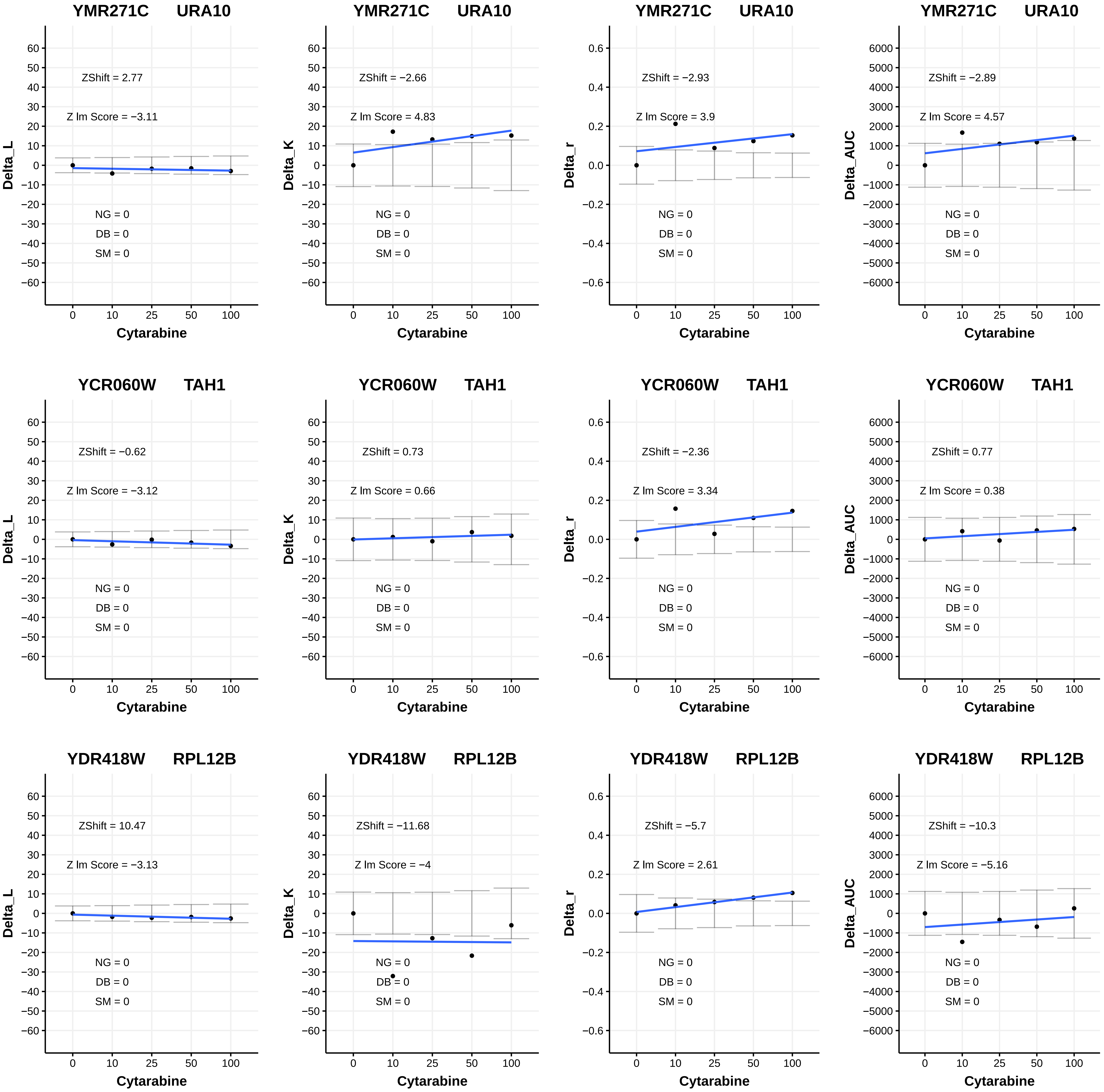
YMR271C URA10
ZShift = 2.77
Z lm Score = −3.11
NG = 0
DB = 0
SM = 0
Delta_L
Cytarabine
YMR271C URA10
ZShift = −2.66
Z lm Score = 4.83
NG = 0
DB = 0
SM = 0
Delta_K
Cytarabine
YMR271C URA10
ZShift = −2.93
Z lm Score = 3.9
NG = 0
DB = 0
SM = 0
Delta_r
Cytarabine
YMR271C URA10
ZShift = −2.89
Z lm Score = 4.57
NG = 0
DB = 0
SM = 0
Delta_AUC
Cytarabine
YCR060W TAH1
ZShift = −0.62
Z lm Score = −3.12
NG = 0
DB = 0
SM = 0
Delta_L
Cytarabine
YCR060W TAH1
ZShift = 0.73
Z lm Score = 0.66
NG = 0
DB = 0
SM = 0
Delta_K
Cytarabine
YCR060W TAH1
ZShift = −2.36
Z lm Score = 3.34
NG = 0
DB = 0
SM = 0
Delta_r
Cytarabine
YCR060W TAH1
ZShift = 0.77
Z lm Score = 0.38
NG = 0
DB = 0
SM = 0
Delta_AUC
Cytarabine
YDR418W RPL12B
ZShift = 10.47
Z lm Score = −3.13
NG = 0
DB = 0
SM = 0
Delta_L
Cytarabine
YDR418W RPL12B
ZShift = −11.68
Z lm Score = −4
NG = 0
DB = 0
SM = 0
Delta_K
Cytarabine
YDR418W RPL12B
ZShift = −5.7
Z lm Score = 2.61
NG = 0
DB = 0
SM = 0
Delta_r
Cytarabine
YDR418W RPL12B
ZShift = −10.3
Z lm Score = −5.16
NG = 0
DB = 0
SM = 0
Delta_AUC
Cytarabine

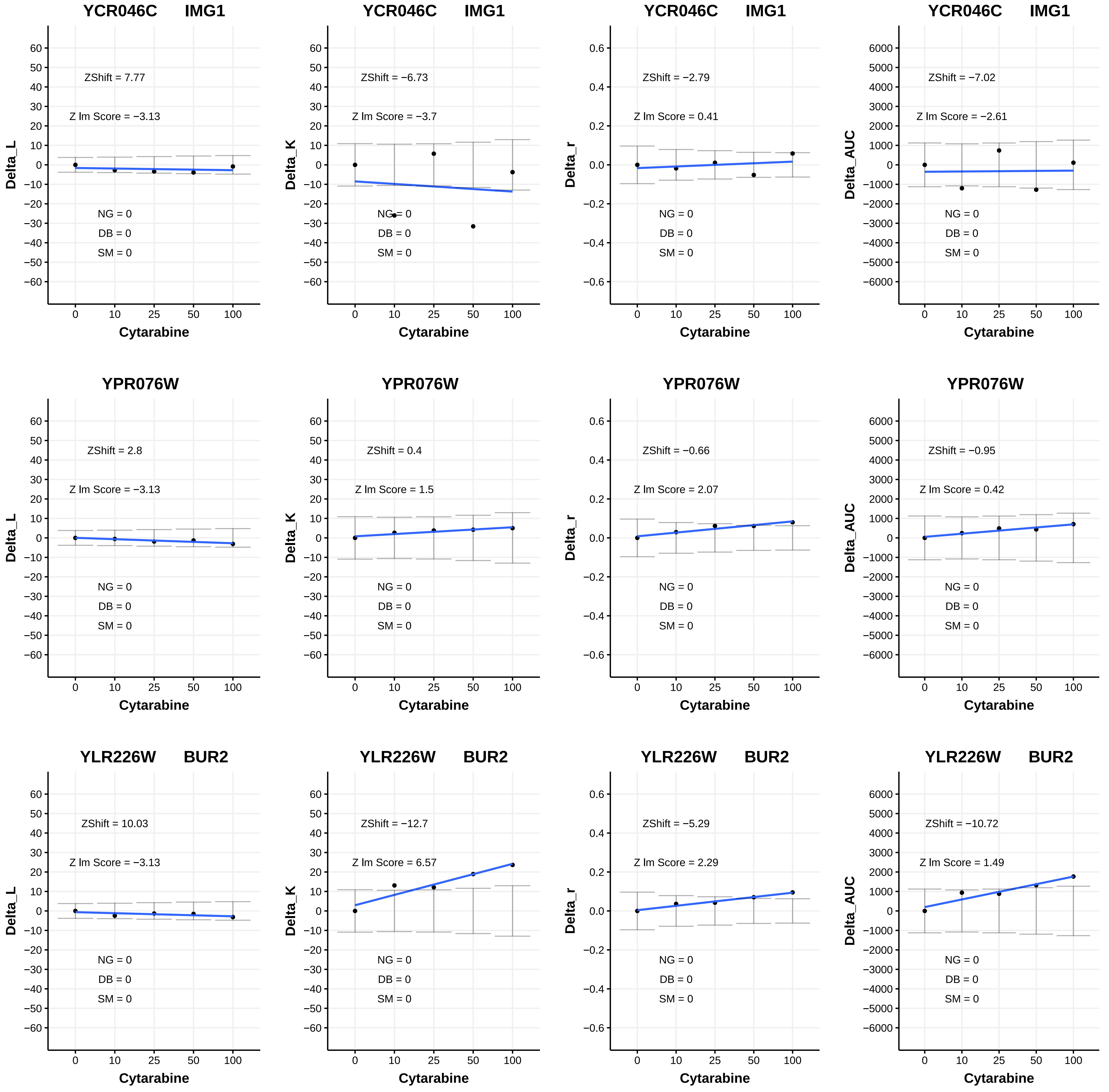
YCR046C IMG1
ZShift = 7.77
Z lm Score = −3.13
NG = 0
DB = 0
SM = 0
Delta_L
Cytarabine
YCR046C IMG1
ZShift = −6.73
Z lm Score = −3.7
NG = 0
DB = 0
SM = 0
Delta_K
Cytarabine
YCR046C IMG1
ZShift = −2.79
Z lm Score = 0.41
NG = 0
DB = 0
SM = 0
Delta_r
Cytarabine
YCR046C IMG1
ZShift = −7.02
Z lm Score = −2.61
NG = 0
DB = 0
SM = 0
Delta_AUC
Cytarabine
YPR076W
ZShift = 2.8
Z lm Score = −3.13
NG = 0
DB = 0
SM = 0
Delta_L
Cytarabine
YPR076W
ZShift = 0.4
Z lm Score = 1.5
NG = 0
DB = 0
SM = 0
Delta_K
Cytarabine
YPR076W
ZShift = −0.66
Z lm Score = 2.07
NG = 0
DB = 0
SM = 0
Delta_r
Cytarabine
YPR076W
ZShift = −0.95
Z lm Score = 0.42
NG = 0
DB = 0
SM = 0
Delta_AUC
Cytarabine
YLR226W BUR2
ZShift = 10.03
Z lm Score = −3.13
NG = 0
DB = 0
SM = 0
Delta_L
Cytarabine
YLR226W BUR2
ZShift = −12.7
Z lm Score = 6.57
NG = 0
DB = 0
SM = 0
Delta_K
Cytarabine
YLR226W BUR2
ZShift = −5.29
Z lm Score = 2.29
NG = 0
DB = 0
SM = 0
Delta_r
Cytarabine
YLR226W BUR2
ZShift = −10.72
Z lm Score = 1.49
NG = 0
DB = 0
SM = 0
Delta_AUC
Cytarabine

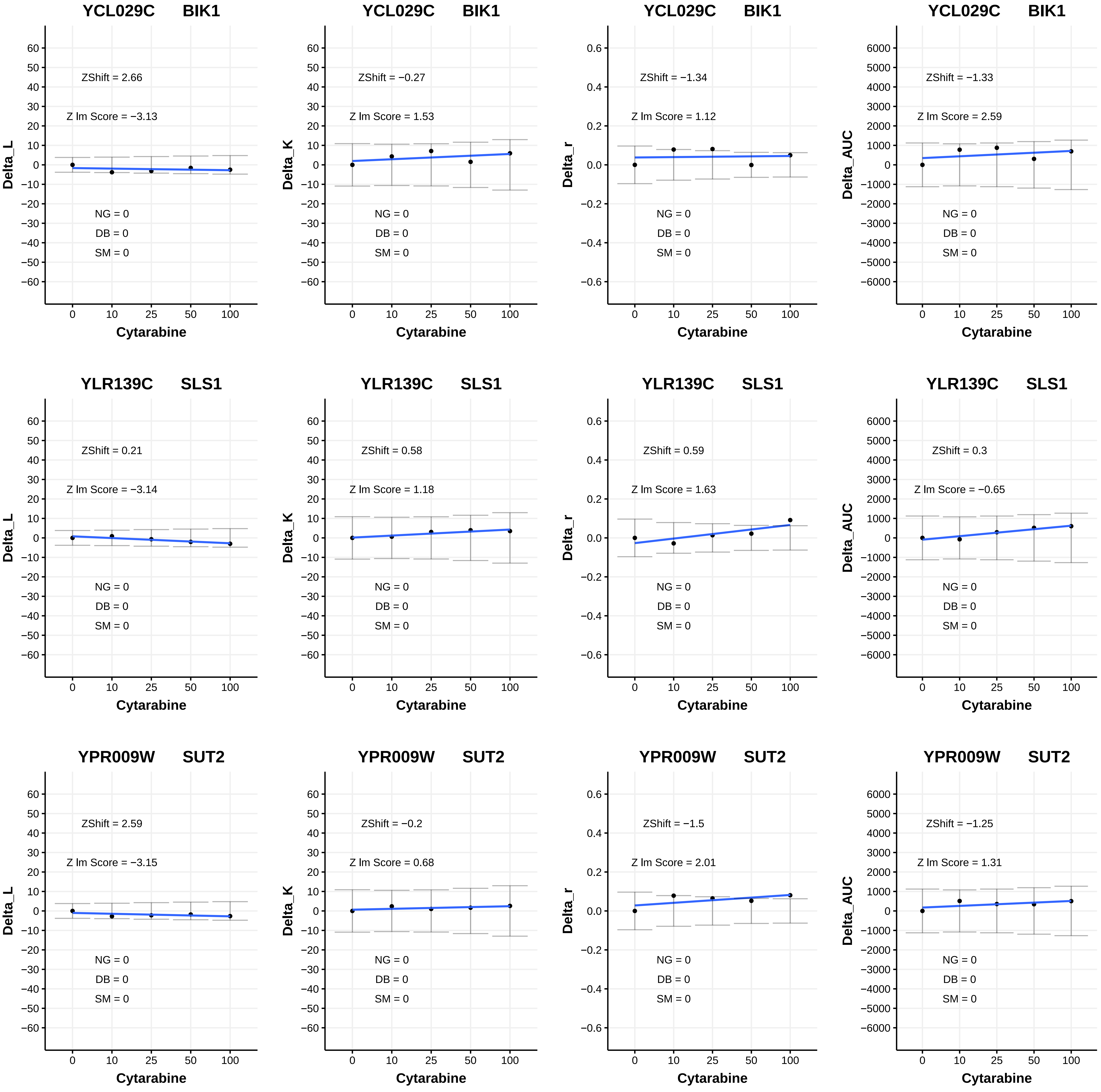

YCL029C BIK1
ZShift = 2.66
Z lm Score = −3.13
NG = 0
DB = 0
SM = 0
Delta_L
Cytarabine
YCL029C BIK1
ZShift = −0.27
Z lm Score = 1.53
NG = 0
DB = 0
SM = 0
Delta_K
Cytarabine
YCL029C BIK1
ZShift = −1.34
Z lm Score = 1.12
NG = 0
DB = 0
SM = 0
Delta_r
Cytarabine
YCL029C BIK1
ZShift = −1.33
Z lm Score = 2.59
NG = 0
DB = 0
SM = 0
Delta_AUC
Cytarabine
YLR139C SLS1
ZShift = 0.21
Z lm Score = −3.14
NG = 0
DB = 0
SM = 0
Delta_L
Cytarabine
YLR139C SLS1
ZShift = 0.58
Z lm Score = 1.18
NG = 0
DB = 0
SM = 0
Delta_K
Cytarabine
YLR139C SLS1
ZShift = 0.59
Z lm Score = 1.63
NG = 0
DB = 0
SM = 0
Delta_r
Cytarabine
YLR139C SLS1
ZShift = 0.3
Z lm Score = −0.65
NG = 0
DB = 0
SM = 0
Delta_AUC
Cytarabine
YPR009W SUT2
ZShift = 2.59
Z lm Score = −3.15
NG = 0
DB = 0
SM = 0
Delta_L
Cytarabine
YPR009W SUT2
ZShift = −0.2
Z lm Score = 0.68
NG = 0
DB = 0
SM = 0
Delta_K
Cytarabine
YPR009W SUT2
ZShift = −1.5
Z lm Score = 2.01
NG = 0
DB = 0
SM = 0
Delta_r
Cytarabine
YPR009W SUT2
ZShift = −1.25
Z lm Score = 1.31
NG = 0
DB = 0
SM = 0
Delta_AUC
Cytarabine

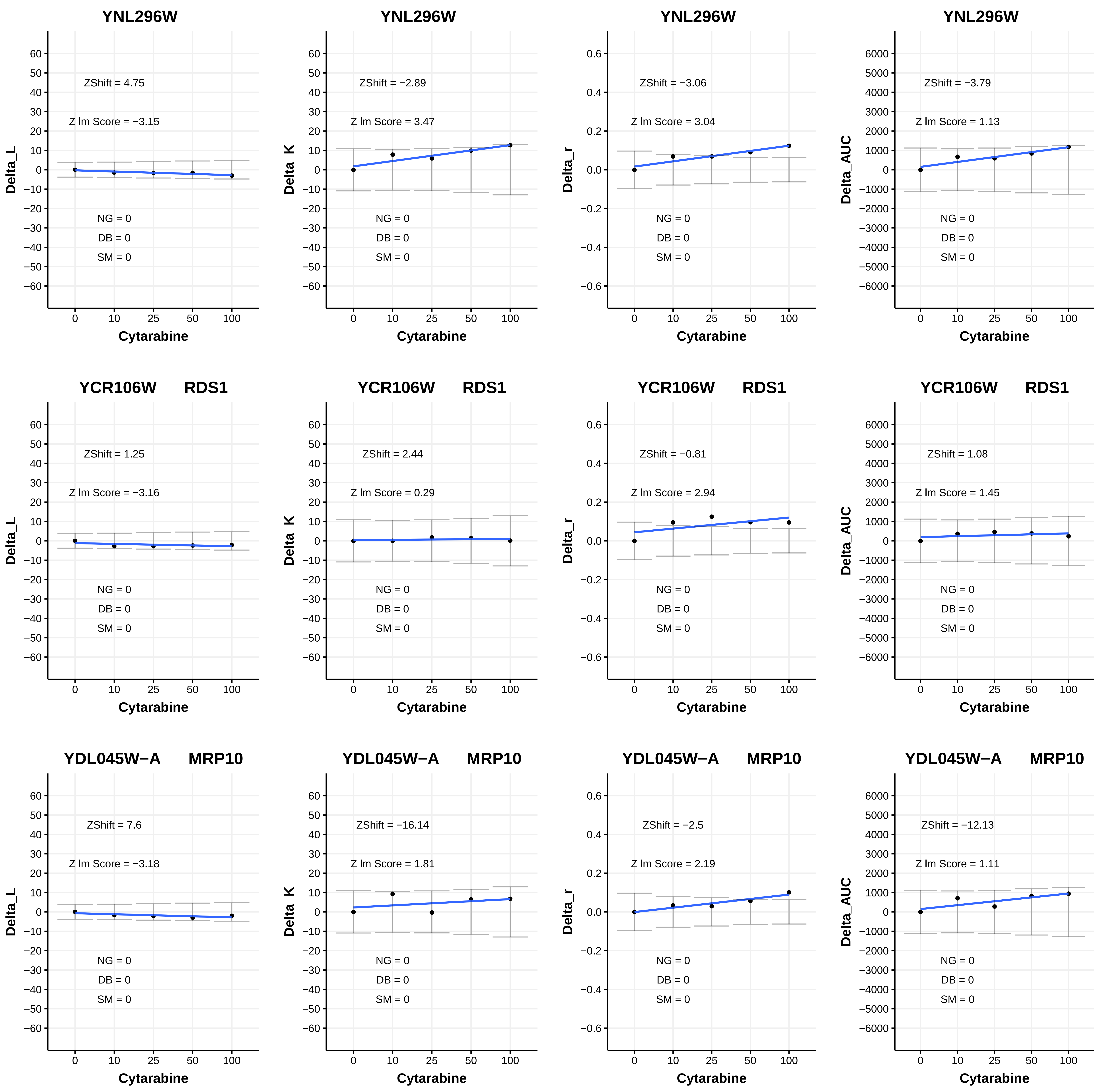

YNL296W
ZShift = 4.75
Z lm Score = −3.15
NG = 0
DB = 0
SM = 0
Delta_L
Cytarabine
YNL296W
ZShift = −2.89
Z lm Score = 3.47
NG = 0
DB = 0
SM = 0
Delta_K
Cytarabine
YNL296W
ZShift = −3.06
Z lm Score = 3.04
NG = 0
DB = 0
SM = 0
Delta_r
Cytarabine
YNL296W
ZShift = −3.79
Z lm Score = 1.13
NG = 0
DB = 0
SM = 0
Delta_AUC
Cytarabine
YCR106W RDS1
ZShift = 1.25
Z lm Score = −3.16
NG = 0
DB = 0
SM = 0
Delta_L
Cytarabine
YCR106W RDS1
ZShift = 2.44
Z lm Score = 0.29
NG = 0
DB = 0
SM = 0
Delta_K
Cytarabine
YCR106W RDS1
ZShift = −0.81
Z lm Score = 2.94
NG = 0
DB = 0
SM = 0
Delta_r
Cytarabine
YCR106W RDS1
ZShift = 1.08
Z lm Score = 1.45
NG = 0
DB = 0
SM = 0
Delta_AUC
Cytarabine
YDL045W−A MRP10
ZShift = 7.6
Z lm Score = −3.18
NG = 0
DB = 0
SM = 0
Delta_L
Cytarabine
YDL045W−A MRP10
ZShift = −16.14
Z lm Score = 1.81
NG = 0
DB = 0
SM = 0
Delta_K
Cytarabine
YDL045W−A MRP10
ZShift = −2.5
Z lm Score = 2.19
NG = 0
DB = 0
SM = 0
Delta_r
Cytarabine
YDL045W−A MRP10
ZShift = −12.13
Z lm Score = 1.11
NG = 0
DB = 0
SM = 0
Delta_AUC
Cytarabine

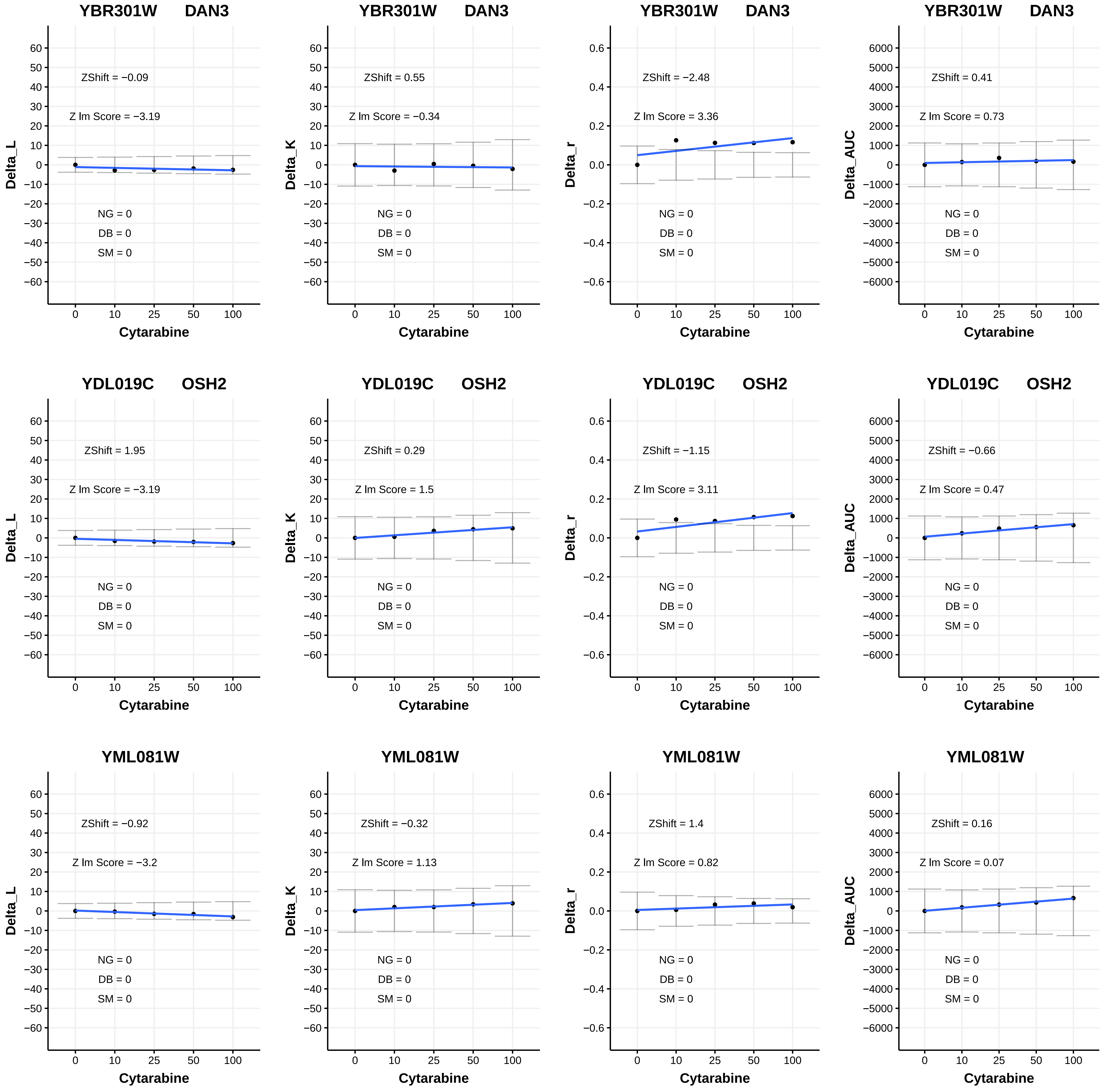
YBR301W DAN3
ZShift = −0.09
Z lm Score = −3.19
NG = 0
DB = 0
SM = 0
Delta_L
Cytarabine
YBR301W DAN3
ZShift = 0.55
Z lm Score = −0.34
NG = 0
DB = 0
SM = 0
Delta_K
Cytarabine
YBR301W DAN3
ZShift = −2.48
Z lm Score = 3.36
NG = 0
DB = 0
SM = 0
Delta_r
Cytarabine
YBR301W DAN3
ZShift = 0.41
Z lm Score = 0.73
NG = 0
DB = 0
SM = 0
Delta_AUC
Cytarabine
YDL019C OSH2
ZShift = 1.95
Z lm Score = −3.19
NG = 0
DB = 0
SM = 0
Delta_L
Cytarabine
YDL019C OSH2
ZShift = 0.29
Z lm Score = 1.5
NG = 0
DB = 0
SM = 0
Delta_K
Cytarabine
YDL019C OSH2
ZShift = −1.15
Z lm Score = 3.11
NG = 0
DB = 0
SM = 0
Delta_r
Cytarabine
YDL019C OSH2
ZShift = −0.66
Z lm Score = 0.47
NG = 0
DB = 0
SM = 0
Delta_AUC
Cytarabine
YML081W
ZShift = −0.92
Z lm Score = −3.2
NG = 0
DB = 0
SM = 0
Delta_L
Cytarabine
YML081W
ZShift = −0.32
Z lm Score = 1.13
NG = 0
DB = 0
SM = 0
Delta_K
Cytarabine
YML081W
ZShift = 1.4
Z lm Score = 0.82
NG = 0
DB = 0
SM = 0
Delta_r
Cytarabine
YML081W
ZShift = 0.16
Z lm Score = 0.07
NG = 0
DB = 0
SM = 0
Delta_AUC
Cytarabine

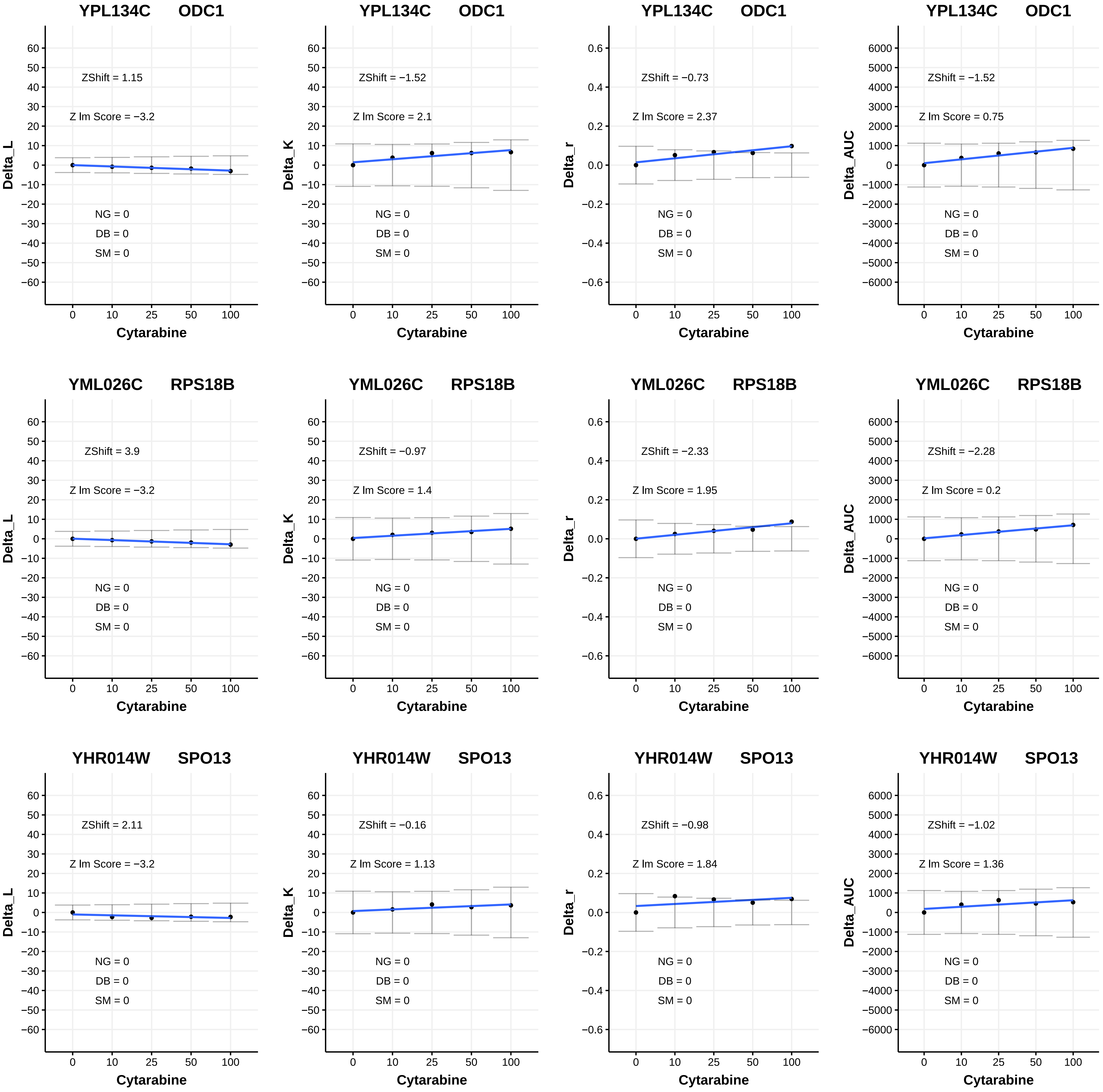
YPL134C ODC1
ZShift = 1.15
Z lm Score = −3.2
NG = 0
DB = 0
SM = 0
Delta_L
Cytarabine
YPL134C ODC1
ZShift = −1.52
Z lm Score = 2.1
NG = 0
DB = 0
SM = 0
Delta_K
Cytarabine
YPL134C ODC1
ZShift = −0.73
Z lm Score = 2.37
NG = 0
DB = 0
SM = 0
Delta_r
Cytarabine
YPL134C ODC1
ZShift = −1.52
Z lm Score = 0.75
NG = 0
DB = 0
SM = 0
Delta_AUC
Cytarabine
YML026C RPS18B
ZShift = 3.9
Z lm Score = −3.2
NG = 0
DB = 0
SM = 0
Delta_L
Cytarabine
YML026C RPS18B
ZShift = −0.97
Z lm Score = 1.4
NG = 0
DB = 0
SM = 0
Delta_K
Cytarabine
YML026C RPS18B
ZShift = −2.33
Z lm Score = 1.95
NG = 0
DB = 0
SM = 0
Delta_r
Cytarabine
YML026C RPS18B
ZShift = −2.28
Z lm Score = 0.2
NG = 0
DB = 0
SM = 0
Delta_AUC
Cytarabine
YHR014W SPO13
ZShift = 2.11
Z lm Score = −3.2
NG = 0
DB = 0
SM = 0
Delta_L
Cytarabine
YHR014W SPO13
ZShift = −0.16
Z lm Score = 1.13
NG = 0
DB = 0
SM = 0
Delta_K
Cytarabine
YHR014W SPO13
ZShift = −0.98
Z lm Score = 1.84
NG = 0
DB = 0
SM = 0
Delta_r
Cytarabine
YHR014W SPO13
ZShift = −1.02
Z lm Score = 1.36
NG = 0
DB = 0
SM = 0
Delta_AUC
Cytarabine

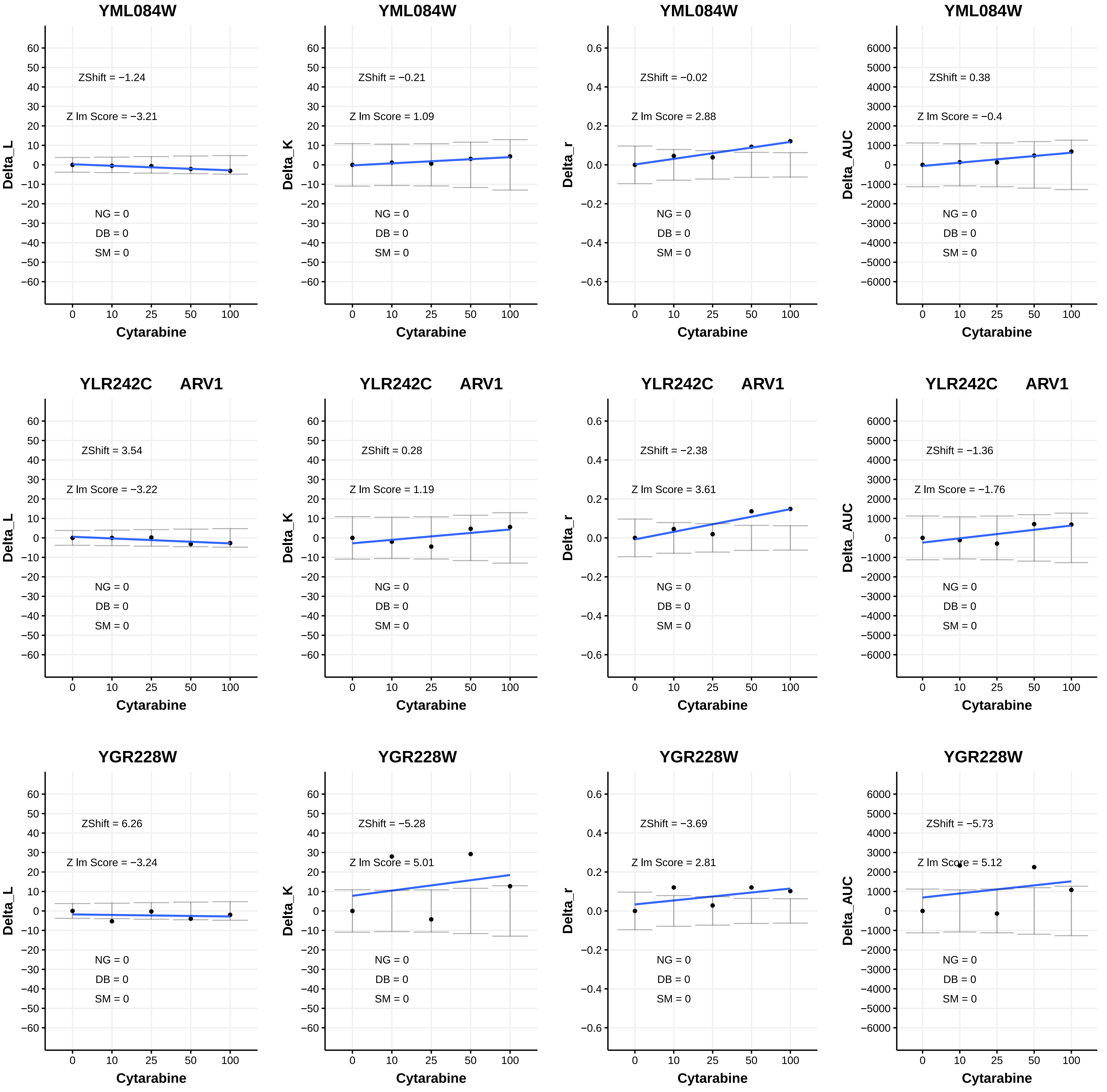

YML084W
ZShift = −1.24
Z lm Score = −3.21
NG = 0
DB = 0
SM = 0
Delta_L
Cytarabine
YML084W
ZShift = −0.21
Z lm Score = 1.09
NG = 0
DB = 0
SM = 0
Delta_K
Cytarabine
YML084W
ZShift = −0.02
Z lm Score = 2.88
NG = 0
DB = 0
SM = 0
Delta_r
Cytarabine
YML084W
ZShift = 0.38
Z lm Score = −0.4
NG = 0
DB = 0
SM = 0
Delta_AUC
Cytarabine
YLR242C ARV1
ZShift = 3.54
Z lm Score = −3.22
NG = 0
DB = 0
SM = 0
Delta_L
Cytarabine
YLR242C ARV1
ZShift = 0.28
Z lm Score = 1.19
NG = 0
DB = 0
SM = 0
Delta_K
Cytarabine
YLR242C ARV1
ZShift = −2.38
Z lm Score = 3.61
NG = 0
DB = 0
SM = 0
Delta_r
Cytarabine
YLR242C ARV1
ZShift = −1.36
Z lm Score = −1.76
NG = 0
DB = 0
SM = 0
Delta_AUC
Cytarabine
YGR228W
ZShift = 6.26
Z lm Score = −3.24
NG = 0
DB = 0
SM = 0
Delta_L
Cytarabine
YGR228W
ZShift = −5.28
Z lm Score = 5.01
NG = 0
DB = 0
SM = 0
Delta_K
Cytarabine
YGR228W
ZShift = −3.69
Z lm Score = 2.81
NG = 0
DB = 0
SM = 0
Delta_r
Cytarabine
YGR228W
ZShift = −5.73
Z lm Score = 5.12
NG = 0
DB = 0
SM = 0
Delta_AUC
Cytarabine

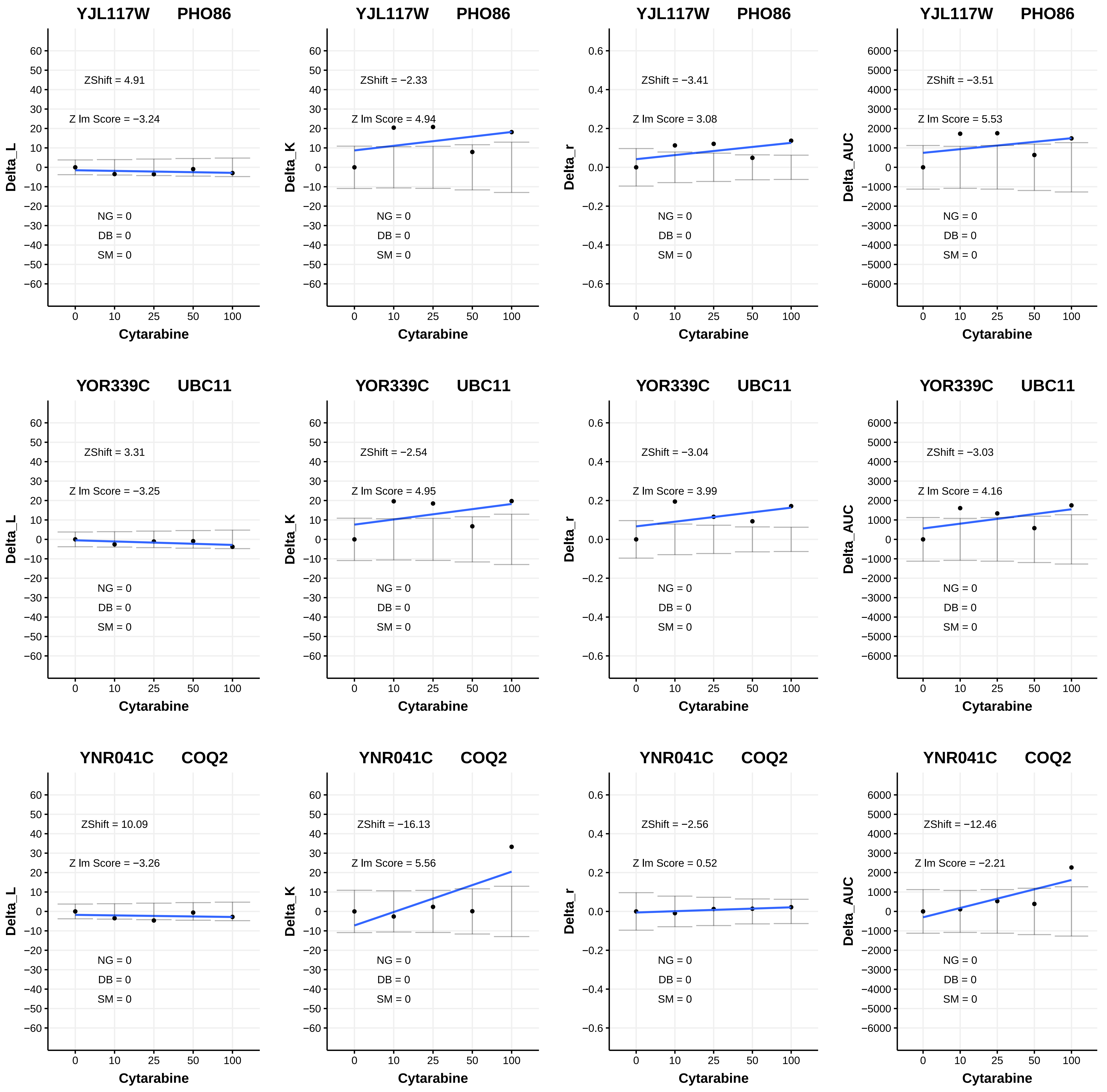

YJL117W PHO86
Delta_L
ZShift = 4.91
Z lm Score = −3.24
NG = 0
DB = 0
SM = 0
Cytarabine
YJL117W PHO86
Delta_K
ZShift = −2.33
Z lm Score = 4.94
NG = 0
DB = 0
SM = 0
Cytarabine
YJL117W PHO86
Delta_r
ZShift = −3.41
Z lm Score = 3.08
NG = 0
DB = 0
SM = 0
Cytarabine
YJL117W PHO86
Delta_AUC
ZShift = −3.51
Z lm Score = 5.53
NG = 0
DB = 0
SM = 0
Cytarabine
YOR339C UBC11
Delta_L
ZShift = 3.31
Z lm Score = −3.25
NG = 0
DB = 0
SM = 0
Cytarabine
YOR339C UBC11
Delta_K
ZShift = −2.54
Z lm Score = 4.95
NG = 0
DB = 0
SM = 0
Cytarabine
YOR339C UBC11
Delta_r
ZShift = −3.04
Z lm Score = 3.99
NG = 0
DB = 0
SM = 0
Cytarabine
YOR339C UBC11
Delta_AUC
ZShift = −3.03
Z lm Score = 4.16
NG = 0
DB = 0
SM = 0
Cytarabine
YNR041C COQ2
Delta_L
ZShift = 10.09
Z lm Score = −3.26
NG = 0
DB = 0
SM = 0
Cytarabine
YNR041C COQ2
Delta_K
ZShift = −16.13
Z lm Score = 5.56
NG = 0
DB = 0
SM = 0
Cytarabine
YNR041C COQ2
Delta_r
ZShift = −2.56
Z lm Score = 0.52
NG = 0
DB = 0
SM = 0
Cytarabine
YNR041C COQ2
Delta_AUC
ZShift = −12.46
Z lm Score = −2.21
NG = 0
DB = 0
SM = 0
Cytarabine

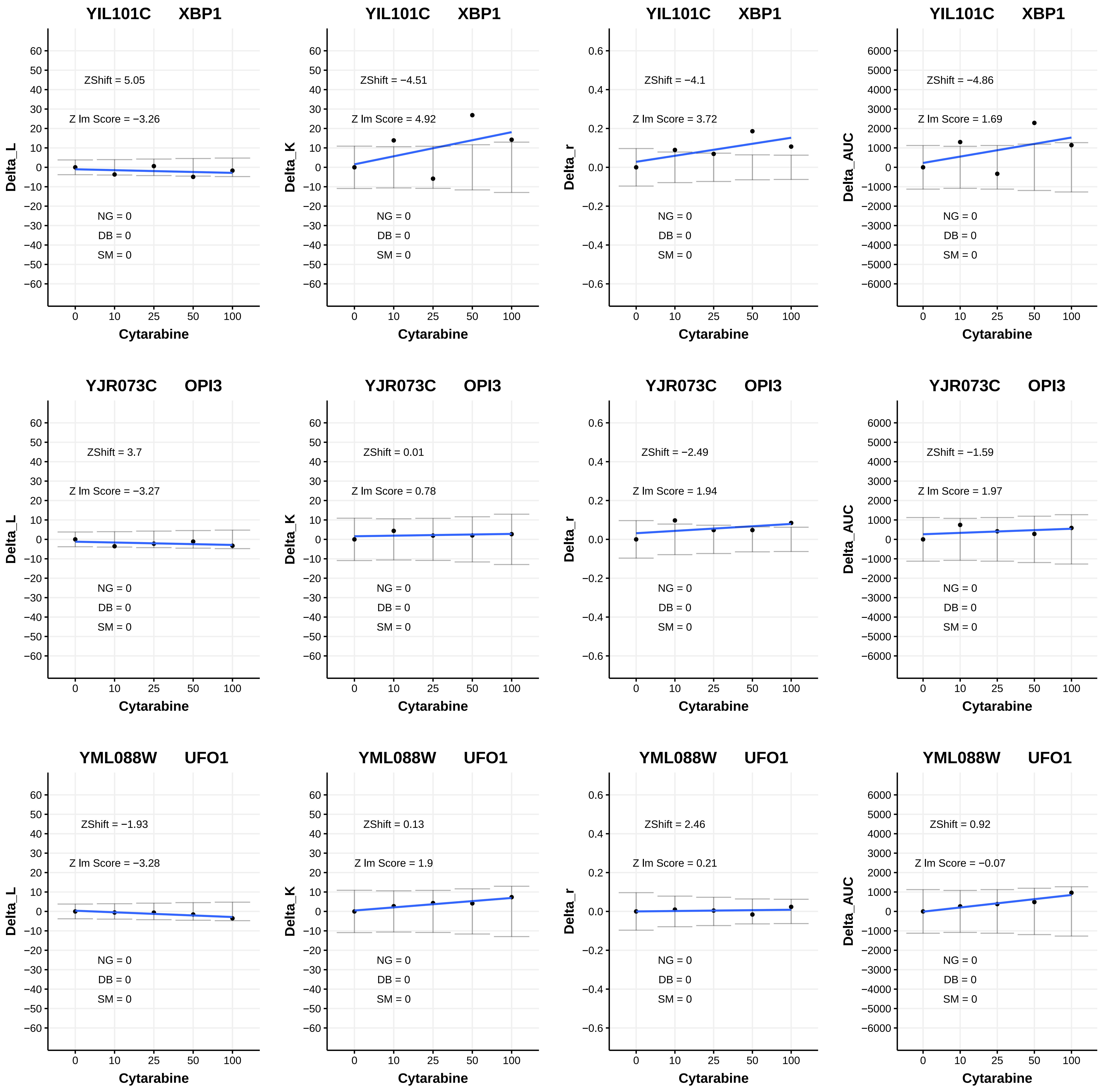

YIL101C XBP1
ZShift = 5.05
Z lm Score = −3.26
NG = 0
DB = 0
SM = 0
Delta_L
Cytarabine
YIL101C XBP1
ZShift = −4.51
Z lm Score = 4.92
NG = 0
DB = 0
SM = 0
Delta_K
Cytarabine
YIL101C XBP1
ZShift = −4.1
Z lm Score = 3.72
NG = 0
DB = 0
SM = 0
Delta_r
Cytarabine
YIL101C XBP1
ZShift = −4.86
Z lm Score = 1.69
NG = 0
DB = 0
SM = 0
Delta_AUC
Cytarabine
YJR073C OPI3
ZShift = 3.7
Z lm Score = −3.27
NG = 0
DB = 0
SM = 0
Delta_L
Cytarabine
YJR073C OPI3
ZShift = 0.01
Z lm Score = 0.78
NG = 0
DB = 0
SM = 0
Delta_K
Cytarabine
YJR073C OPI3
ZShift = −2.49
Z lm Score = 1.94
NG = 0
DB = 0
SM = 0
Delta_r
Cytarabine
YJR073C OPI3
ZShift = −1.59
Z lm Score = 1.97
NG = 0
DB = 0
SM = 0
Delta_AUC
Cytarabine
YML088W UFO1
ZShift = −1.93
Z lm Score = −3.28
NG = 0
DB = 0
SM = 0
Delta_L
Cytarabine
YML088W UFO1
ZShift = 0.13
Z lm Score = 1.9
NG = 0
DB = 0
SM = 0
Delta_K
Cytarabine
YML088W UFO1
ZShift = 2.46
Z lm Score = 0.21
NG = 0
DB = 0
SM = 0
Delta_r
Cytarabine
YML088W UFO1
ZShift = 0.92
Z lm Score = −0.07
NG = 0
DB = 0
SM = 0
Delta_AUC
Cytarabine

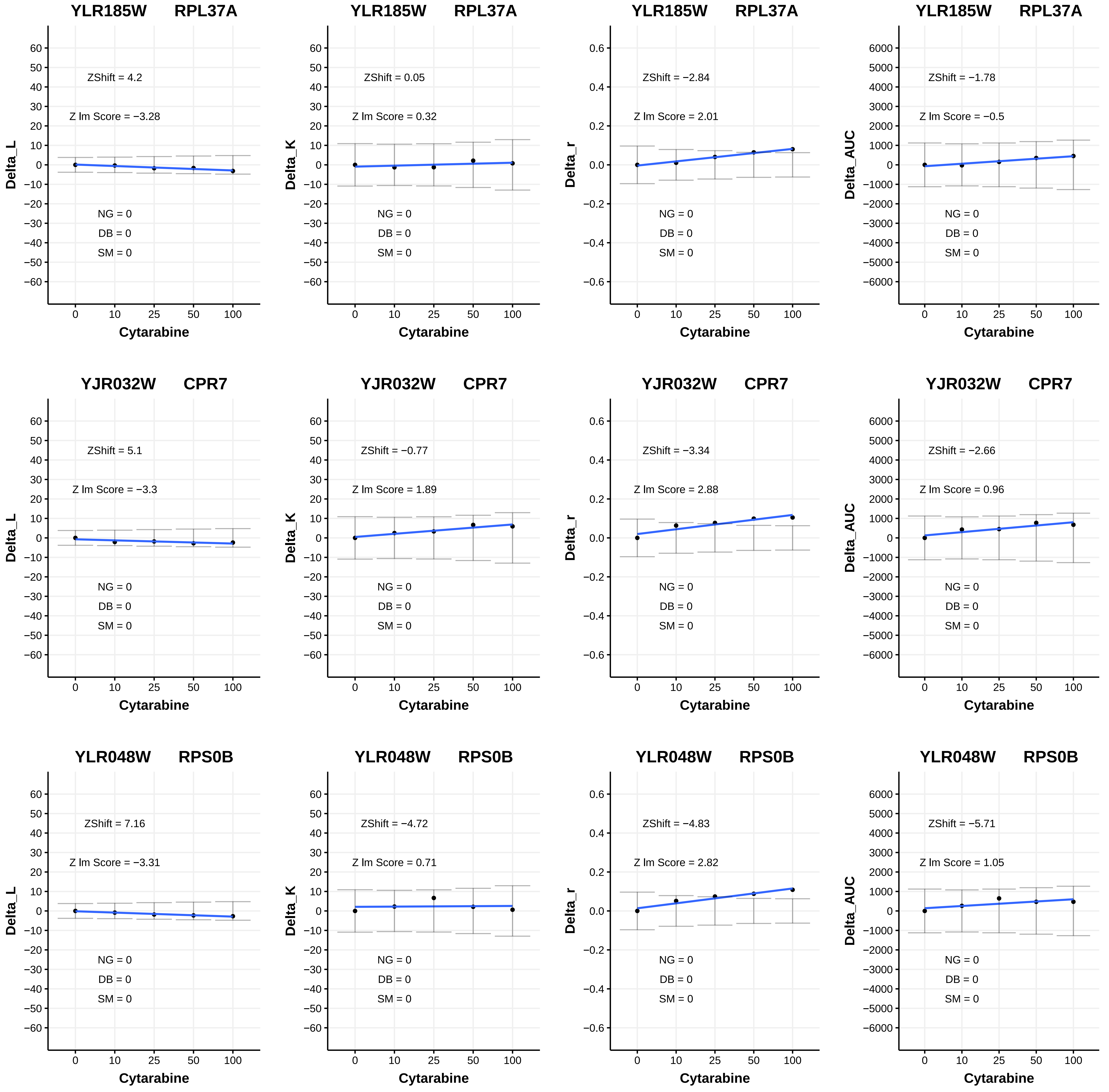
YLR185W RPL37A
ZShift = 4.2
Z lm Score = −3.28
NG = 0
DB = 0
SM = 0
Delta_L
Cytarabine
YLR185W RPL37A
ZShift = 0.05
Z lm Score = 0.32
NG = 0
DB = 0
SM = 0
Delta_K
Cytarabine
YLR185W RPL37A
ZShift = −2.84
Z lm Score = 2.01
NG = 0
DB = 0
SM = 0
Delta_r
Cytarabine
YLR185W RPL37A
ZShift = −1.78
Z lm Score = −0.5
NG = 0
DB = 0
SM = 0
Delta_AUC
Cytarabine
YJR032W CPR7
ZShift = 5.1
Z lm Score = −3.3
NG = 0
DB = 0
SM = 0
Delta_L
Cytarabine
YJR032W CPR7
ZShift = −0.77
Z lm Score = 1.89
NG = 0
DB = 0
SM = 0
Delta_K
Cytarabine
YJR032W CPR7
ZShift = −3.34
Z lm Score = 2.88
NG = 0
DB = 0
SM = 0
Delta_r
Cytarabine
YJR032W CPR7
ZShift = −2.66
Z lm Score = 0.96
NG = 0
DB = 0
SM = 0
Delta_AUC
Cytarabine
YLR048W RPS0B
ZShift = 7.16
Z lm Score = −3.31
NG = 0
DB = 0
SM = 0
Delta_L
Cytarabine
YLR048W RPS0B
ZShift = −4.72
Z lm Score = 0.71
NG = 0
DB = 0
SM = 0
Delta_K
Cytarabine
YLR048W RPS0B
ZShift = −4.83
Z lm Score = 2.82
NG = 0
DB = 0
SM = 0
Delta_r
Cytarabine
YLR048W RPS0B
ZShift = −5.71
Z lm Score = 1.05
NG = 0
DB = 0
SM = 0
Delta_AUC
Cytarabine

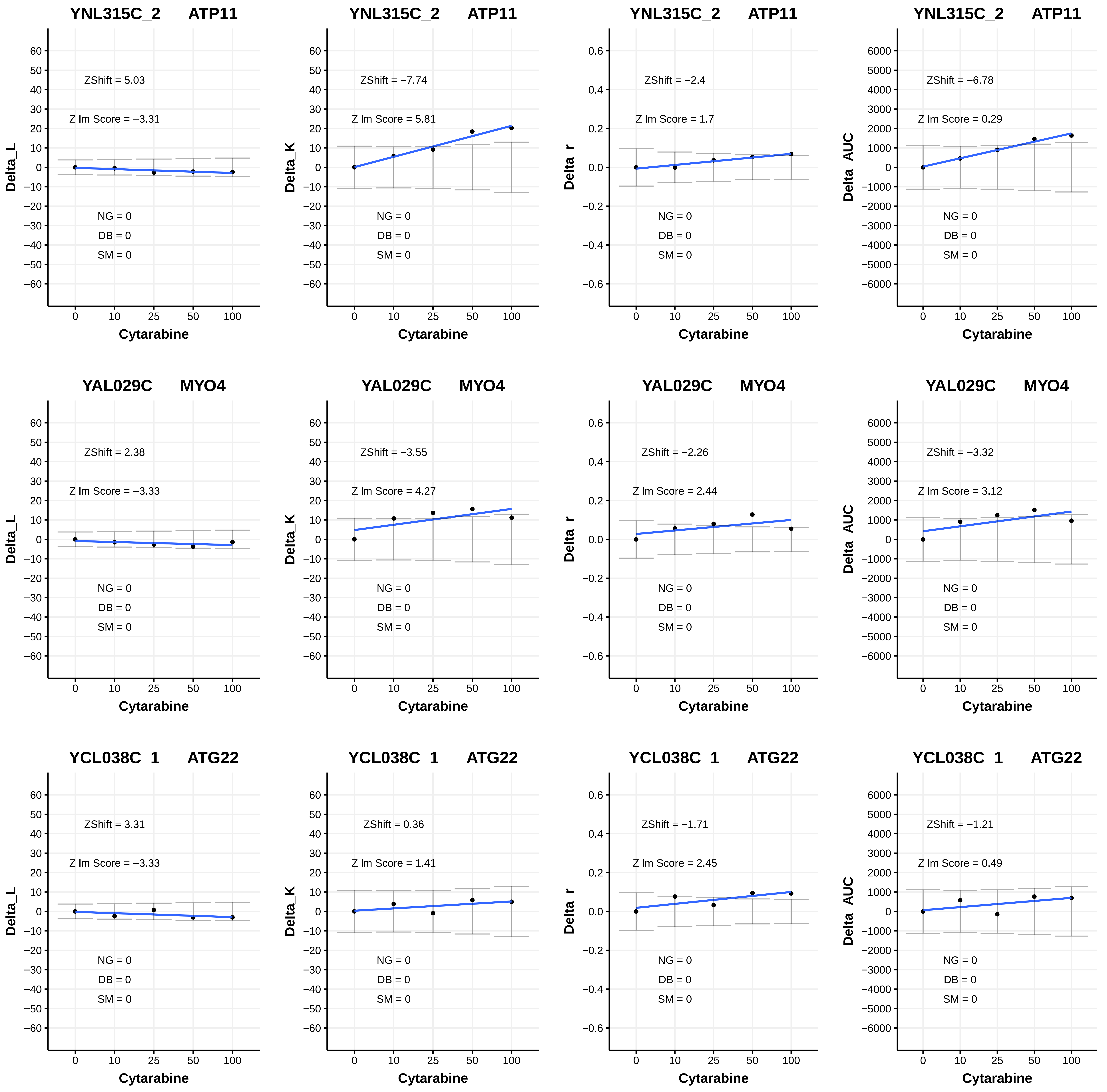
YNL315C_2 ATP11
ZShift = 5.03
Z lm Score = −3.31
Delta_L
NG = 0
DB = 0
SM = 0
Cytarabine
YNL315C_2 ATP11
ZShift = −7.74
Z lm Score = 5.81
Delta_K
NG = 0
DB = 0
SM = 0
Cytarabine
YNL315C_2 ATP11
ZShift = −2.4
Z lm Score = 1.7
Delta_r
NG = 0
DB = 0
SM = 0
Cytarabine
YNL315C_2 ATP11
ZShift = −6.78
Z lm Score = 0.29
Delta_AUC
NG = 0
DB = 0
SM = 0
Cytarabine
YAL029C MYO4
ZShift = 2.38
Z lm Score = −3.33
Delta_L
NG = 0
DB = 0
SM = 0
Cytarabine
YAL029C MYO4
ZShift = −3.55
Z lm Score = 4.27
Delta_K
NG = 0
DB = 0
SM = 0
Cytarabine
YAL029C MYO4
ZShift = −2.26
Z lm Score = 2.44
Delta_r
NG = 0
DB = 0
SM = 0
Cytarabine
YAL029C MYO4
ZShift = −3.32
Z lm Score = 3.12
Delta_AUC
NG = 0
DB = 0
SM = 0
Cytarabine
YCL038C_1 ATG22
ZShift = 3.31
Z lm Score = −3.33
Delta_L
NG = 0
DB = 0
SM = 0
Cytarabine
YCL038C_1 ATG22
ZShift = 0.36
Z lm Score = 1.41
Delta_K
NG = 0
DB = 0
SM = 0
Cytarabine
YCL038C_1 ATG22
ZShift = −1.71
Z lm Score = 2.45
Delta_r
NG = 0
DB = 0
SM = 0
Cytarabine
YCL038C_1 ATG22
ZShift = −1.21
Z lm Score = 0.49
Delta_AUC
NG = 0
DB = 0
SM = 0
Cytarabine

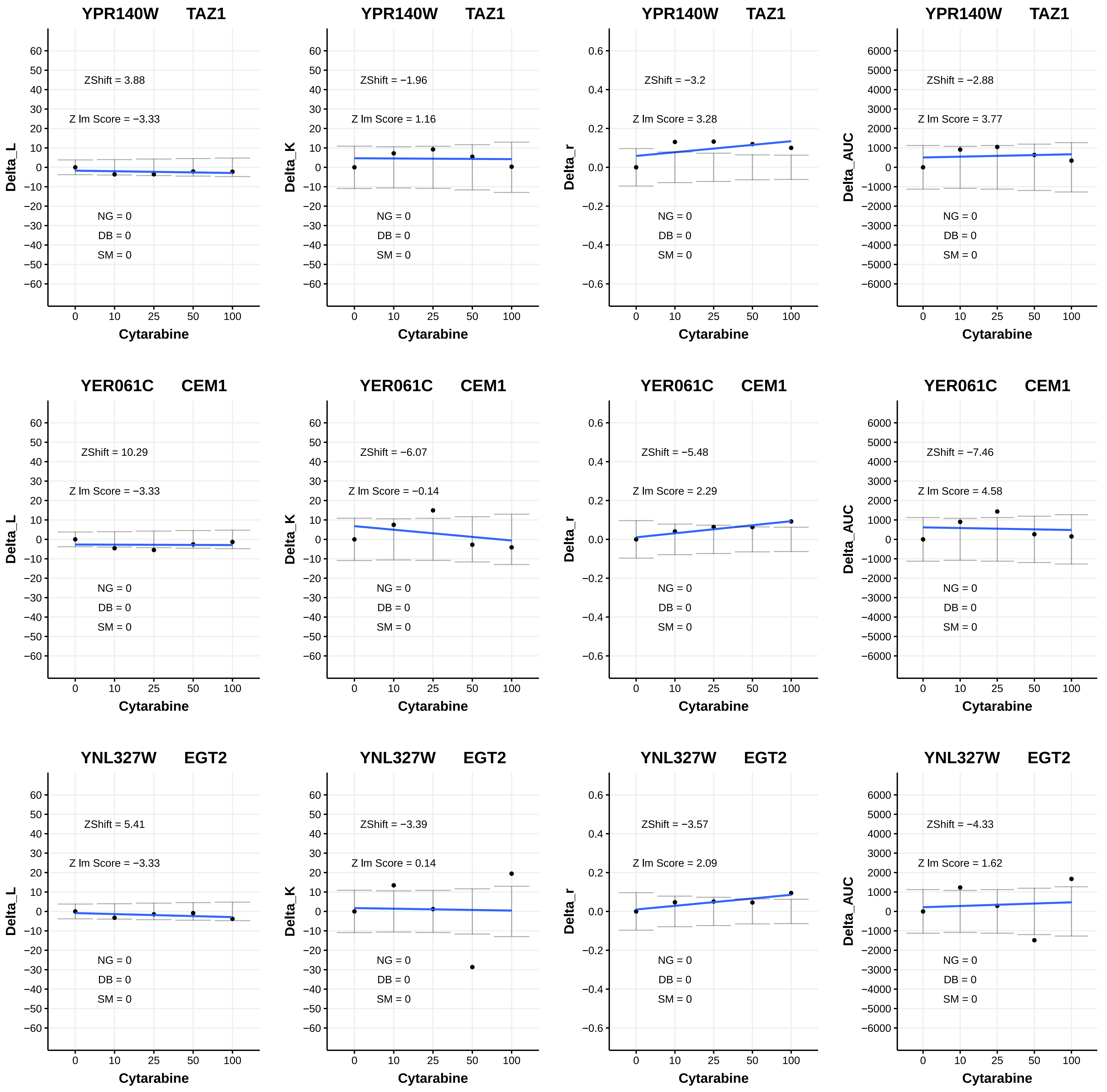

YPR140W TAZ1
ZShift = 3.88
Z lm Score = −3.33
NG = 0
DB = 0
SM = 0
Delta_L
Cytarabine
YPR140W TAZ1
ZShift = −1.96
Z lm Score = 1.16
NG = 0
DB = 0
SM = 0
Delta_K
Cytarabine
YPR140W TAZ1
ZShift = −3.2
Z lm Score = 3.28
NG = 0
DB = 0
SM = 0
Delta_r
Cytarabine
YPR140W TAZ1
ZShift = −2.88
Z lm Score = 3.77
NG = 0
DB = 0
SM = 0
Delta_AUC
Cytarabine
YER061C CEM1
ZShift = 10.29
Z lm Score = −3.33
NG = 0
DB = 0
SM = 0
Delta_L
Cytarabine
YER061C CEM1
ZShift = −6.07
Z lm Score = −0.14
NG = 0
DB = 0
SM = 0
Delta_K
Cytarabine
YER061C CEM1
ZShift = −5.48
Z lm Score = 2.29
NG = 0
DB = 0
SM = 0
Delta_r
Cytarabine
YER061C CEM1
ZShift = −7.46
Z lm Score = 4.58
NG = 0
DB = 0
SM = 0
Delta_AUC
Cytarabine
YNL327W EGT2
ZShift = 5.41
Z lm Score = −3.33
NG = 0
DB = 0
SM = 0
Delta_L
Cytarabine
YNL327W EGT2
ZShift = −3.39
Z lm Score = 0.14
NG = 0
DB = 0
SM = 0
Delta_K
Cytarabine
YNL327W EGT2
ZShift = −3.57
Z lm Score = 2.09
NG = 0
DB = 0
SM = 0
Delta_r
Cytarabine
YNL327W EGT2
ZShift = −4.33
Z lm Score = 1.62
NG = 0
DB = 0
SM = 0
Delta_AUC
Cytarabine

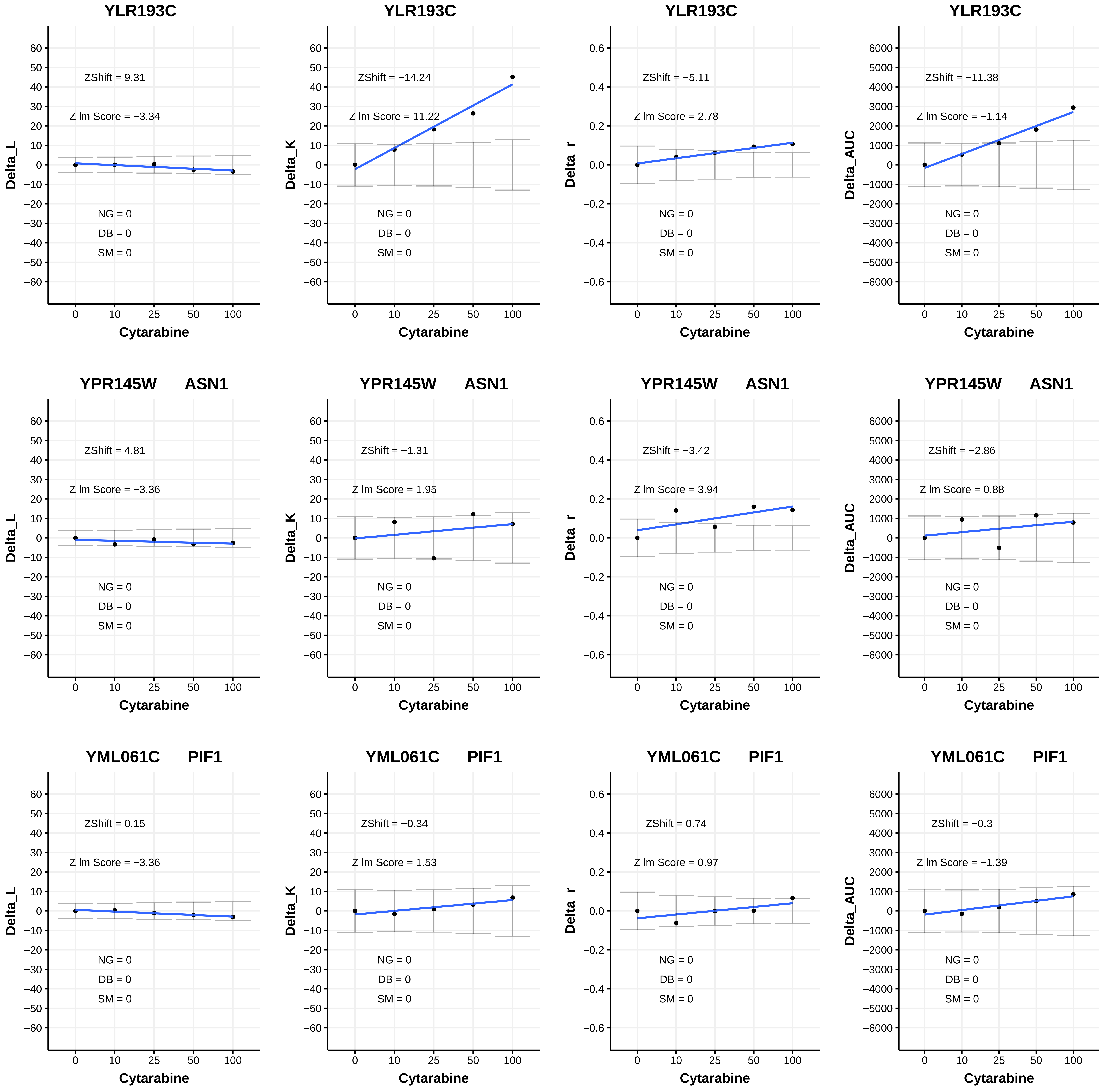

YLR193C
ZShift = 9.31
Z lm Score = −3.34
NG = 0
DB = 0
SM = 0
Delta_L
Cytarabine
YLR193C
ZShift = −14.24
Z lm Score = 11.22
NG = 0
DB = 0
SM = 0
Delta_K
Cytarabine
YLR193C
ZShift = −5.11
Z lm Score = 2.78
NG = 0
DB = 0
SM = 0
Delta_r
Cytarabine
YLR193C
ZShift = −11.38
Z lm Score = −1.14
NG = 0
DB = 0
SM = 0
Delta_AUC
Cytarabine
YPR145W ASN1
ZShift = 4.81
Z lm Score = −3.36
NG = 0
DB = 0
SM = 0
Delta_L
Cytarabine
YPR145W ASN1
ZShift = −1.31
Z lm Score = 1.95
NG = 0
DB = 0
SM = 0
Delta_K
Cytarabine
YPR145W ASN1
ZShift = −3.42
Z lm Score = 3.94
NG = 0
DB = 0
SM = 0
Delta_r
Cytarabine
YPR145W ASN1
ZShift = −2.86
Z lm Score = 0.88
NG = 0
DB = 0
SM = 0
Delta_AUC
Cytarabine
YML061C PIF1
ZShift = 0.15
Z lm Score = −3.36
NG = 0
DB = 0
SM = 0
Delta_L
Cytarabine
YML061C PIF1
ZShift = −0.34
Z lm Score = 1.53
NG = 0
DB = 0
SM = 0
Delta_K
Cytarabine
YML061C PIF1
ZShift = 0.74
Z lm Score = 0.97
NG = 0
DB = 0
SM = 0
Delta_r
Cytarabine
YML061C PIF1
ZShift = −0.3
Z lm Score = −1.39
NG = 0
DB = 0
SM = 0
Delta_AUC
Cytarabine

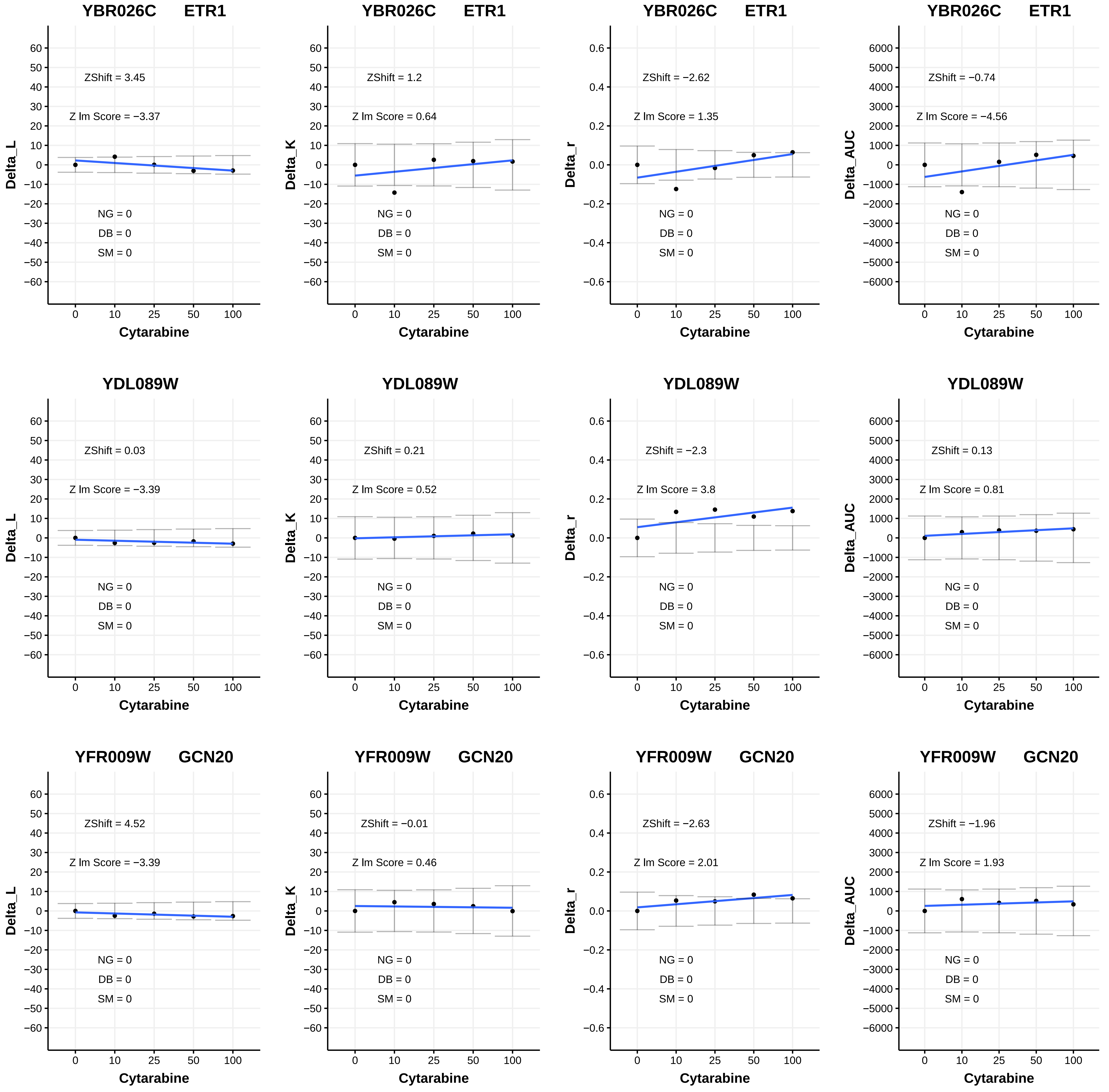

YBR026C ETR1
ZShift = 3.45
Z lm Score = −3.37
Delta_L
NG = 0
DB = 0
SM = 0
Cytarabine
YBR026C ETR1
ZShift = 1.2
Z lm Score = 0.64
Delta_K
NG = 0
DB = 0
SM = 0
Cytarabine
YBR026C ETR1
ZShift = −2.62
Z lm Score = 1.35
Delta_r
NG = 0
DB = 0
SM = 0
Cytarabine
YBR026C ETR1
ZShift = −0.74
Z lm Score = −4.56
Delta_AUC
NG = 0
DB = 0
SM = 0
Cytarabine
YDL089W
ZShift = 0.03
Z lm Score = −3.39
Delta_L
NG = 0
DB = 0
SM = 0
Cytarabine
YDL089W
ZShift = 0.21
Z lm Score = 0.52
Delta_K
NG = 0
DB = 0
SM = 0
Cytarabine
YDL089W
ZShift = −2.3
Z lm Score = 3.8
Delta_r
NG = 0
DB = 0
SM = 0
Cytarabine
YDL089W
ZShift = 0.13
Z lm Score = 0.81
Delta_AUC
NG = 0
DB = 0
SM = 0
Cytarabine
YFR009W GCN20
ZShift = 4.52
Z lm Score = −3.39
Delta_L
NG = 0
DB = 0
SM = 0
Cytarabine
YFR009W GCN20
ZShift = −0.01
Z lm Score = 0.46
Delta_K
NG = 0
DB = 0
SM = 0
Cytarabine
YFR009W GCN20
ZShift = −2.63
Z lm Score = 2.01
Delta_r
NG = 0
DB = 0
SM = 0
Cytarabine
YFR009W GCN20
ZShift = −1.96
Z lm Score = 1.93
Delta_AUC
NG = 0
DB = 0
SM = 0
Cytarabine

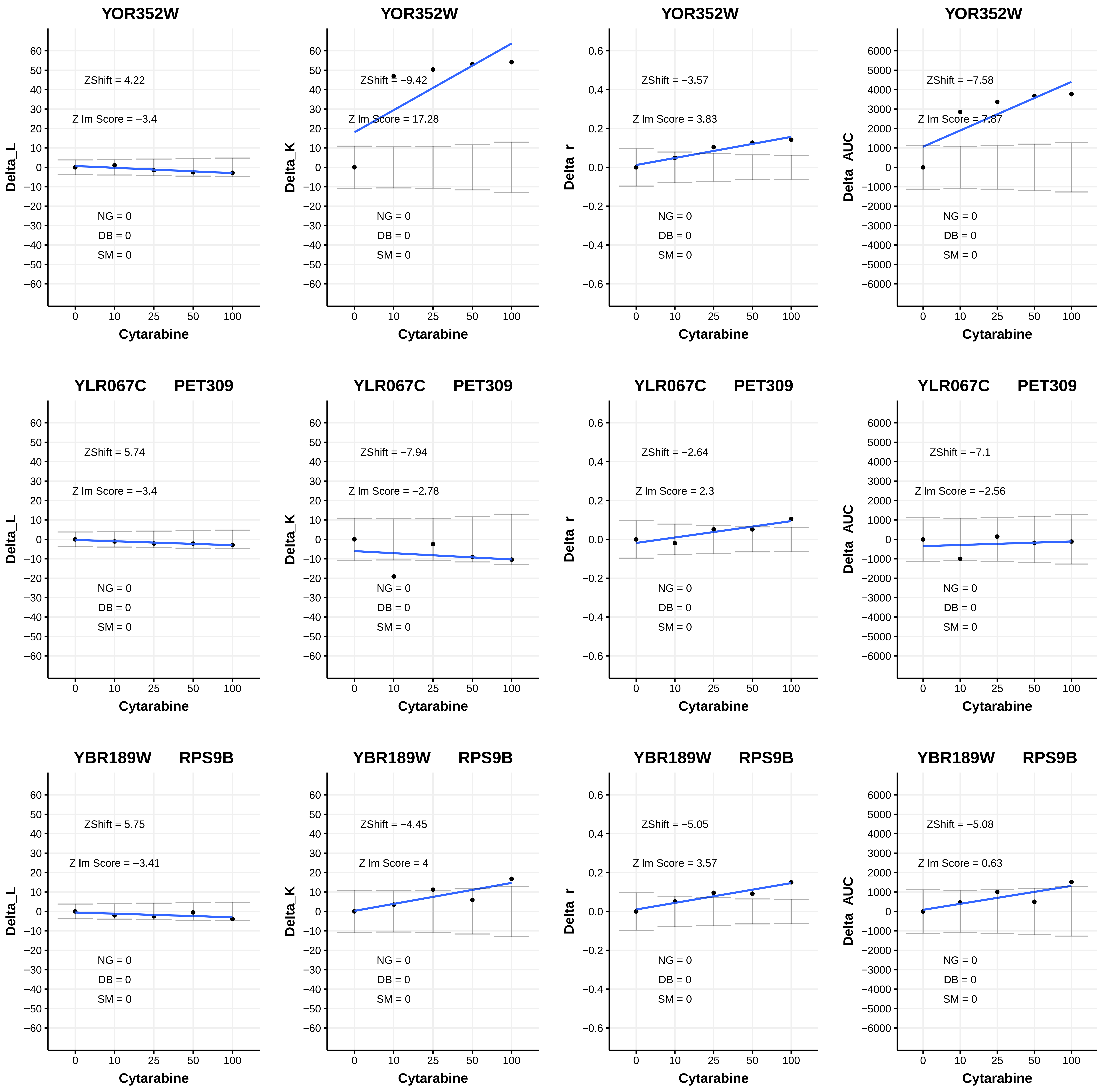
YOR352W
ZShift = 4.22
Z lm Score = −3.4
NG = 0
DB = 0
SM = 0
Delta_L
Cytarabine
YOR352W
ZShift = −9.42
Z lm Score = 17.28
NG = 0
DB = 0
SM = 0
Delta_K
Cytarabine
YOR352W
ZShift = −3.57
Z lm Score = 3.83
NG = 0
DB = 0
SM = 0
Delta_r
Cytarabine
YOR352W
ZShift = −7.58
Z lm Score = 7.87
NG = 0
DB = 0
SM = 0
Delta_AUC
Cytarabine
YLR067C PET309
ZShift = 5.74
Z lm Score = −3.4
NG = 0
DB = 0
SM = 0
Delta_L
Cytarabine
YLR067C PET309
ZShift = −7.94
Z lm Score = −2.78
NG = 0
DB = 0
SM = 0
Delta_K
Cytarabine
YLR067C PET309
ZShift = −2.64
Z lm Score = 2.3
NG = 0
DB = 0
SM = 0
Delta_r
Cytarabine
YLR067C PET309
ZShift = −7.1
Z lm Score = −2.56
NG = 0
DB = 0
SM = 0
Delta_AUC
Cytarabine
YBR189W RPS9B
ZShift = 5.75
Z lm Score = −3.41
NG = 0
DB = 0
SM = 0
Delta_L
Cytarabine
YBR189W RPS9B
ZShift = −4.45
Z lm Score = 4
NG = 0
DB = 0
SM = 0
Delta_K
Cytarabine
YBR189W RPS9B
ZShift = −5.05
Z lm Score = 3.57
NG = 0
DB = 0
SM = 0
Delta_r
Cytarabine
YBR189W RPS9B
ZShift = −5.08
Z lm Score = 0.63
NG = 0
DB = 0
SM = 0
Delta_AUC
Cytarabine
0 10 25 50 100

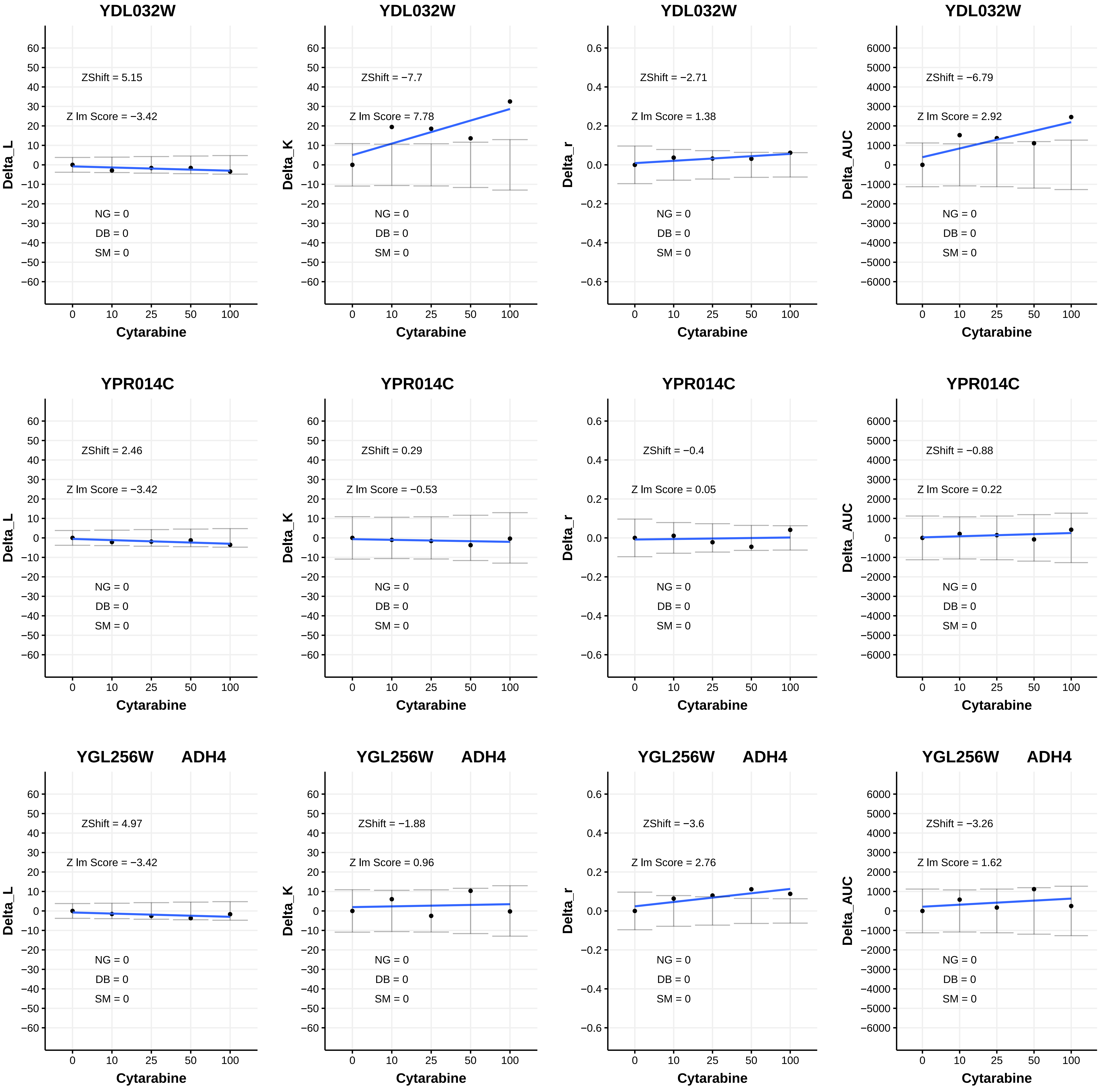

YDL032W
ZShift = 5.15
Z lm Score = −3.42
NG = 0
DB = 0
SM = 0
Delta_L
Cytarabine
YDL032W
ZShift = −7.7
Z lm Score = 7.78
NG = 0
DB = 0
SM = 0
Delta_K
Cytarabine
YDL032W
ZShift = −2.71
Z lm Score = 1.38
NG = 0
DB = 0
SM = 0
Delta_r
Cytarabine
YDL032W
ZShift = −6.79
Z lm Score = 2.92
NG = 0
DB = 0
SM = 0
Delta_AUC
Cytarabine
YPR014C
ZShift = 2.46
Z lm Score = −3.42
NG = 0
DB = 0
SM = 0
Delta_L
Cytarabine
YPR014C
ZShift = 0.29
Z lm Score = −0.53
NG = 0
DB = 0
SM = 0
Delta_K
Cytarabine
YPR014C
ZShift = −0.4
Z lm Score = 0.05
NG = 0
DB = 0
SM = 0
Delta_r
Cytarabine
YPR014C
ZShift = −0.88
Z lm Score = 0.22
NG = 0
DB = 0
SM = 0
Delta_AUC
Cytarabine
YGL256W ADH4
ZShift = 4.97
Z lm Score = −3.42
NG = 0
DB = 0
SM = 0
Delta_L
Cytarabine
YGL256W ADH4
ZShift = −1.88
Z lm Score = 0.96
NG = 0
DB = 0
SM = 0
Delta_K
Cytarabine
YGL256W ADH4
ZShift = −3.6
Z lm Score = 2.76
NG = 0
DB = 0
SM = 0
Delta_r
Cytarabine
YGL256W ADH4
ZShift = −3.26
Z lm Score = 1.62
NG = 0
DB = 0
SM = 0
Delta_AUC
Cytarabine

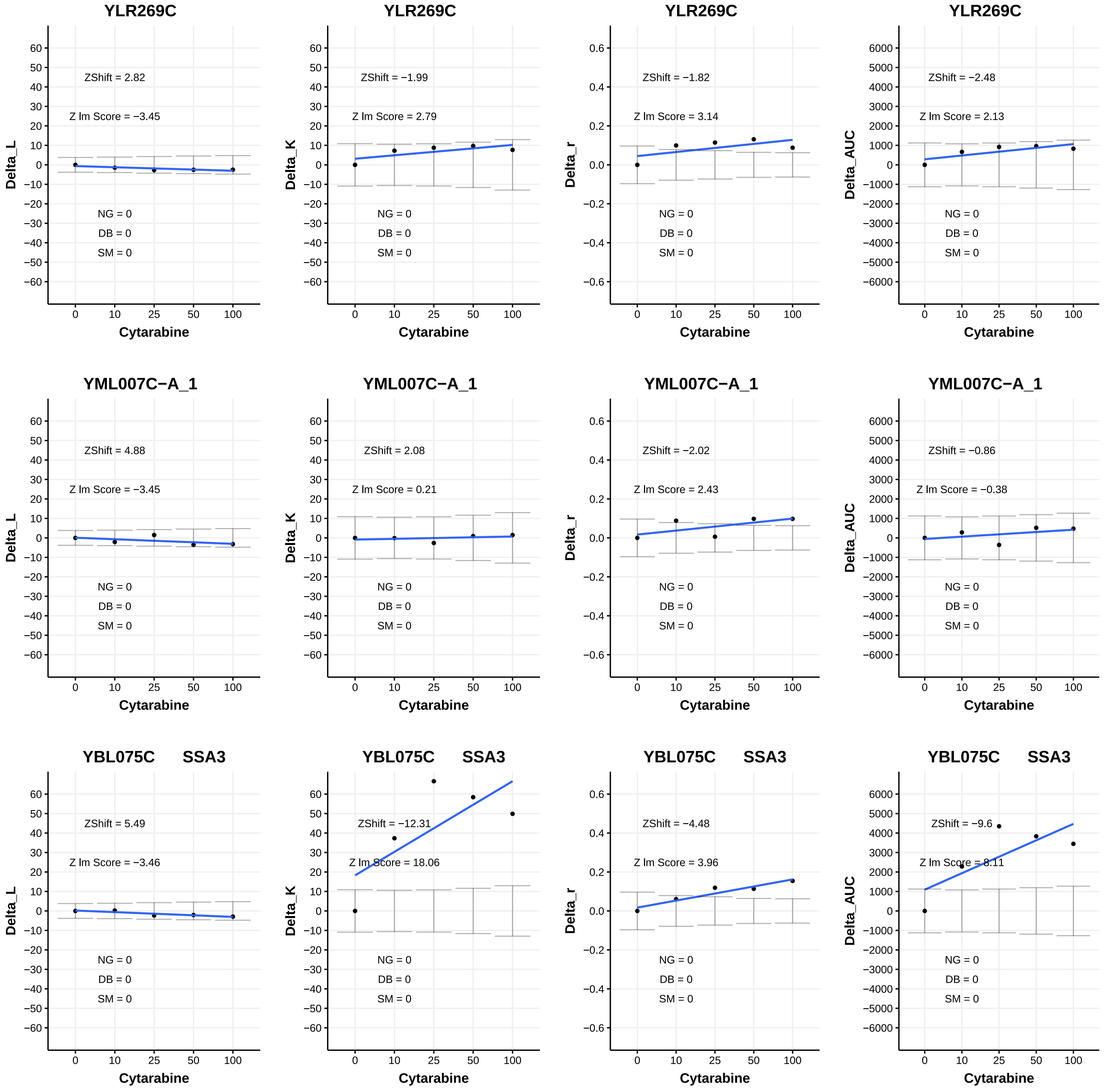
YLR269C
ZShift = 2.82
Z lm Score = −3.45
NG = 0
DB = 0
SM = 0
Delta_L
Cytarabine
YLR269C
ZShift = −1.99
Z lm Score = 2.79
NG = 0
DB = 0
SM = 0
Delta_K
Cytarabine
YLR269C
ZShift = −1.82
Z lm Score = 3.14
NG = 0
DB = 0
SM = 0
Delta_r
Cytarabine
YLR269C
ZShift = −2.48
Z lm Score = 2.13
NG = 0
DB = 0
SM = 0
Delta_AUC
Cytarabine
YML007C−A_1
ZShift = 4.88
Z lm Score = −3.45
NG = 0
DB = 0
SM = 0
Delta_L
Cytarabine
YML007C−A_1
ZShift = 2.08
Z lm Score = 0.21
NG = 0
DB = 0
SM = 0
Delta_K
Cytarabine
YML007C−A_1
ZShift = −2.02
Z lm Score = 2.43
NG = 0
DB = 0
SM = 0
Delta_r
Cytarabine
YML007C−A_1
ZShift = −0.86
Z lm Score = −0.38
NG = 0
DB = 0
SM = 0
Delta_AUC
Cytarabine
YBL075C SSA3
ZShift = 5.49
Z lm Score = −3.46
NG = 0
DB = 0
SM = 0
Delta_L
Cytarabine
YBL075C SSA3
ZShift = −12.31
Z lm Score = 18.06
NG = 0
DB = 0
SM = 0
Delta_K
Cytarabine
YBL075C SSA3
ZShift = −4.48
Z lm Score = 3.96
NG = 0
DB = 0
SM = 0
Delta_r
Cytarabine
YBL075C SSA3
ZShift = −9.6
Z lm Score = 8.11
NG = 0
DB = 0
SM = 0
Delta_AUC
Cytarabine

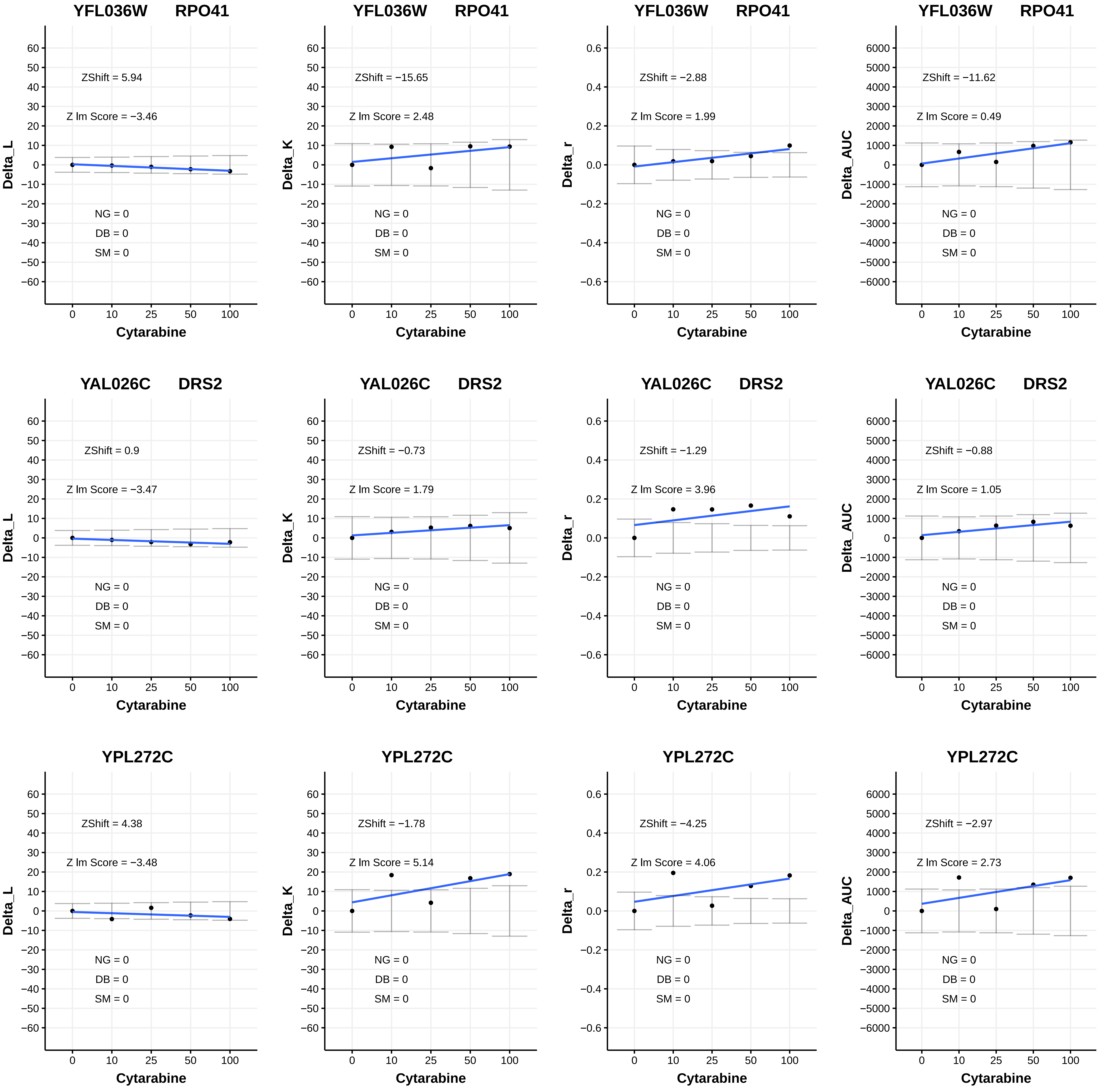

YFL036W RPO41
ZShift = 5.94
Z lm Score = −3.46
NG = 0
DB = 0
SM = 0
Delta_L
Cytarabine
YFL036W RPO41
ZShift = −15.65
Z lm Score = 2.48
NG = 0
DB = 0
SM = 0
Delta_K
Cytarabine
YFL036W RPO41
ZShift = −2.88
Z lm Score = 1.99
NG = 0
DB = 0
SM = 0
Delta_r
Cytarabine
YFL036W RPO41
ZShift = −11.62
Z lm Score = 0.49
NG = 0
DB = 0
SM = 0
Delta_AUC
Cytarabine
YAL026C DRS2
ZShift = 0.9
Z lm Score = −3.47
NG = 0
DB = 0
SM = 0
Delta_L
Cytarabine
YAL026C DRS2
ZShift = −0.73
Z lm Score = 1.79
NG = 0
DB = 0
SM = 0
Delta_K
Cytarabine
YAL026C DRS2
ZShift = −1.29
Z lm Score = 3.96
NG = 0
DB = 0
SM = 0
Delta_r
Cytarabine
YAL026C DRS2
ZShift = −0.88
Z lm Score = 1.05
NG = 0
DB = 0
SM = 0
Delta_AUC
Cytarabine
YPL272C
ZShift = 4.38
Z lm Score = −3.48
NG = 0
DB = 0
SM = 0
Delta_L
Cytarabine
YPL272C
ZShift = −1.78
Z lm Score = 5.14
NG = 0
DB = 0
SM = 0
Delta_K
Cytarabine
YPL272C
ZShift = −4.25
Z lm Score = 4.06
NG = 0
DB = 0
SM = 0
Delta_r
Cytarabine
YPL272C
ZShift = −2.97
Z lm Score = 2.73
NG = 0
DB = 0
SM = 0
Delta_AUC
Cytarabine
0 10 25 50 100

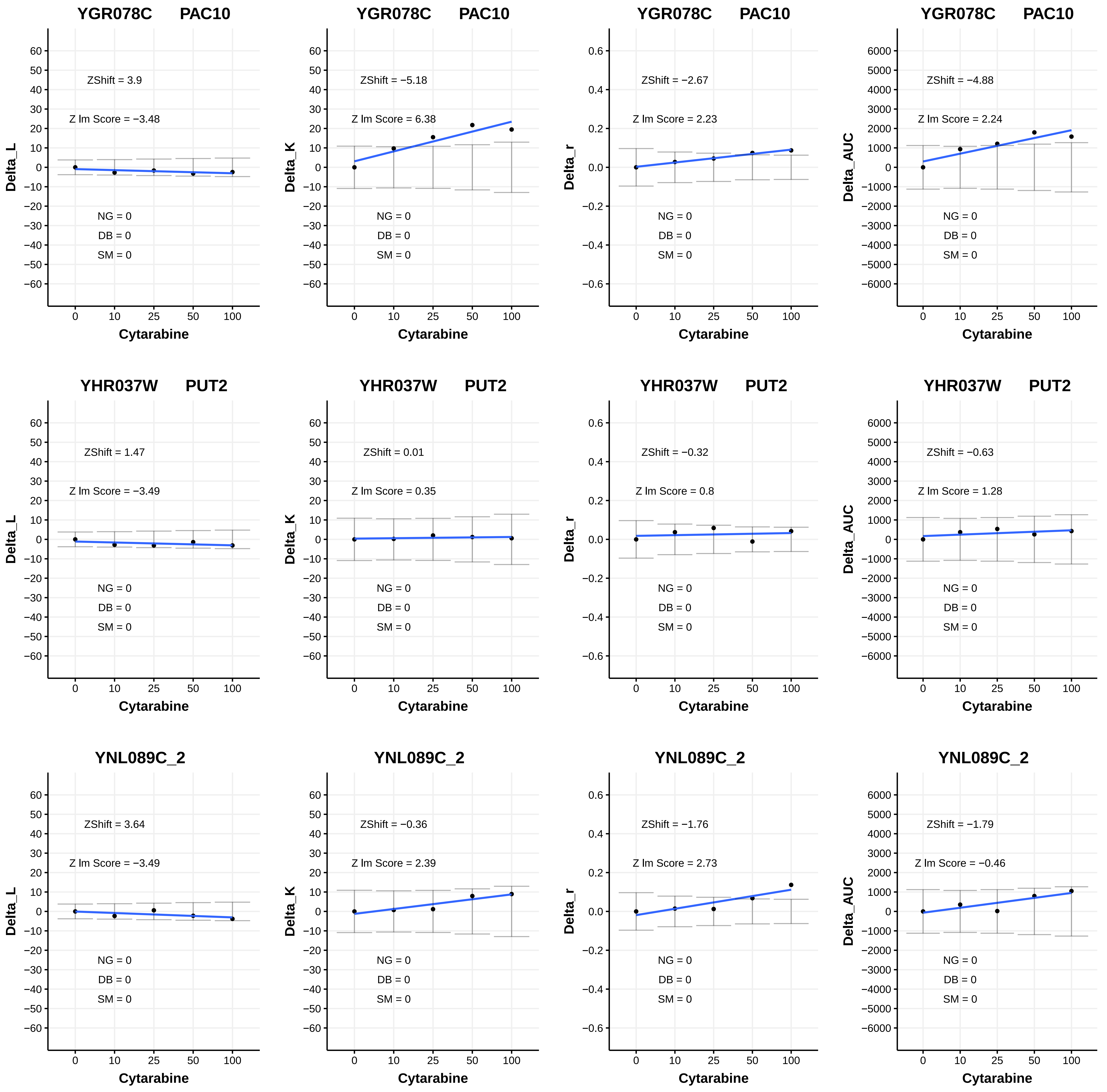
YGR078C PAC10
ZShift = 3.9
Z lm Score = −3.48
NG = 0
DB = 0
SM = 0
Delta_L
Cytarabine
YGR078C PAC10
ZShift = −5.18
Z lm Score = 6.38
NG = 0
DB = 0
SM = 0
Delta_K
Cytarabine
YGR078C PAC10
ZShift = −2.67
Z lm Score = 2.23
NG = 0
DB = 0
SM = 0
Delta_r
Cytarabine
YGR078C PAC10
ZShift = −4.88
Z lm Score = 2.24
NG = 0
DB = 0
SM = 0
Delta_AUC
Cytarabine
YHR037W PUT2
ZShift = 1.47
Z lm Score = −3.49
NG = 0
DB = 0
SM = 0
Delta_L
Cytarabine
YHR037W PUT2
ZShift = 0.01
Z lm Score = 0.35
NG = 0
DB = 0
SM = 0
Delta_K
Cytarabine
YHR037W PUT2
ZShift = −0.32
Z lm Score = 0.8
NG = 0
DB = 0
SM = 0
Delta_r
Cytarabine
YHR037W PUT2
ZShift = −0.63
Z lm Score = 1.28
NG = 0
DB = 0
SM = 0
Delta_AUC
Cytarabine
YNL089C_2
ZShift = 3.64
Z lm Score = −3.49
NG = 0
DB = 0
SM = 0
Delta_L
Cytarabine
YNL089C_2
ZShift = −0.36
Z lm Score = 2.39
NG = 0
DB = 0
SM = 0
Delta_K
Cytarabine
YNL089C_2
ZShift = −1.76
Z lm Score = 2.73
NG = 0
DB = 0
SM = 0
Delta_r
Cytarabine
YNL089C_2
ZShift = −1.79
Z lm Score = −0.46
NG = 0
DB = 0
SM = 0
Delta_AUC
Cytarabine

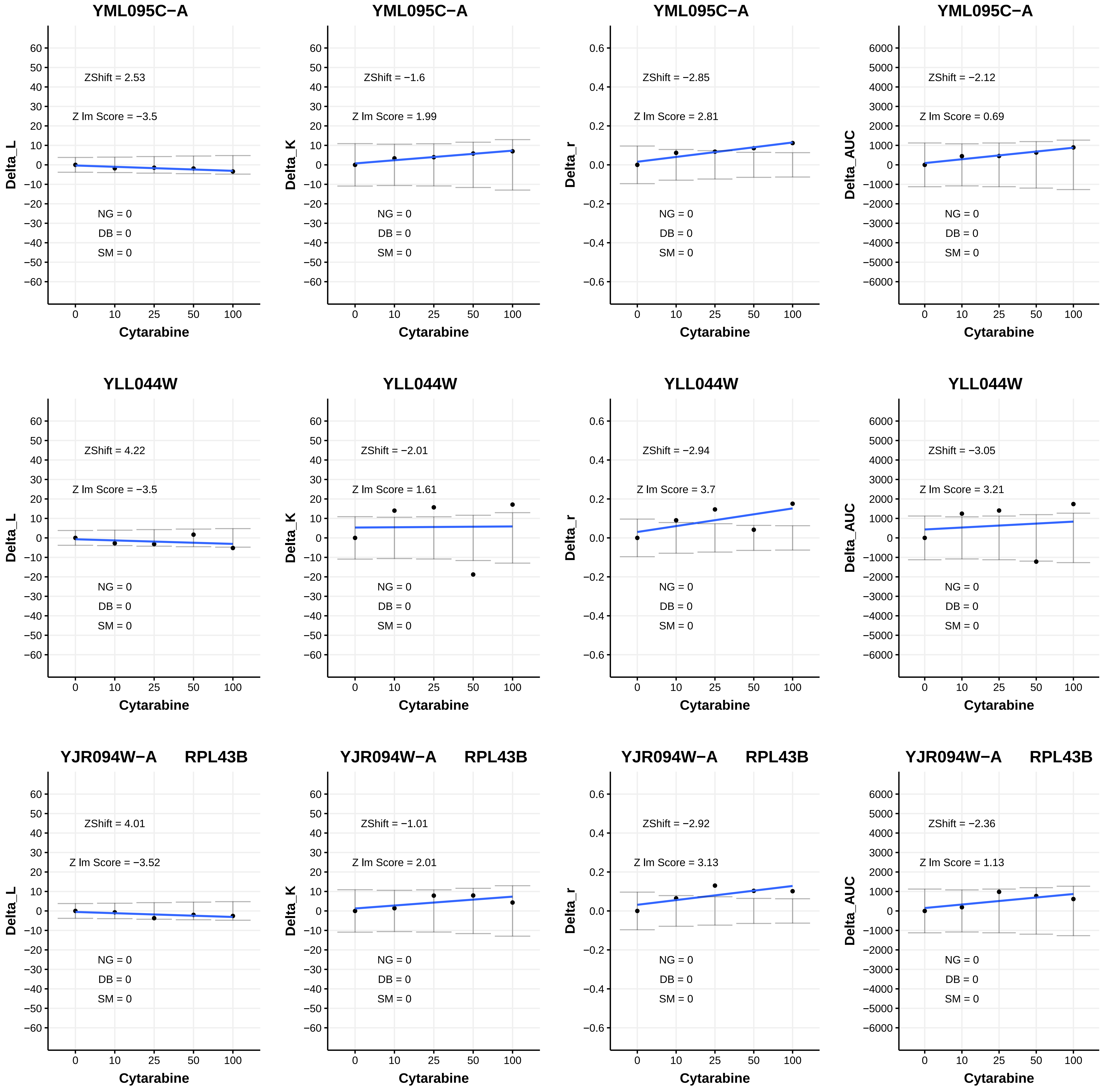

YML095C−A
ZShift = 2.53
Z lm Score = −3.5
NG = 0
DB = 0
SM = 0
Delta_L
Cytarabine
YML095C−A
ZShift = −1.6
Z lm Score = 1.99
NG = 0
DB = 0
SM = 0
Delta_K
Cytarabine
YML095C−A
ZShift = −2.85
Z lm Score = 2.81
NG = 0
DB = 0
SM = 0
Delta_r
Cytarabine
YML095C−A
ZShift = −2.12
Z lm Score = 0.69
NG = 0
DB = 0
SM = 0
Delta_AUC
Cytarabine
YLL044W
ZShift = 4.22
Z lm Score = −3.5
NG = 0
DB = 0
SM = 0
Delta_L
Cytarabine
YLL044W
ZShift = −2.01
Z lm Score = 1.61
NG = 0
DB = 0
SM = 0
Delta_K
Cytarabine
YLL044W
ZShift = −2.94
Z lm Score = 3.7
NG = 0
DB = 0
SM = 0
Delta_r
Cytarabine
YLL044W
ZShift = −3.05
Z lm Score = 3.21
NG = 0
DB = 0
SM = 0
Delta_AUC
Cytarabine
YJR094W−A RPL43B
ZShift = 4.01
Z lm Score = −3.52
NG = 0
DB = 0
SM = 0
Delta_L
Cytarabine
YJR094W−A RPL43B
ZShift = −1.01
Z lm Score = 2.01
NG = 0
DB = 0
SM = 0
Delta_K
Cytarabine
YJR094W−A RPL43B
ZShift = −2.92
Z lm Score = 3.13
NG = 0
DB = 0
SM = 0
Delta_r
Cytarabine
YJR094W−A RPL43B
ZShift = −2.36
Z lm Score = 1.13
NG = 0
DB = 0
SM = 0
Delta_AUC
Cytarabine

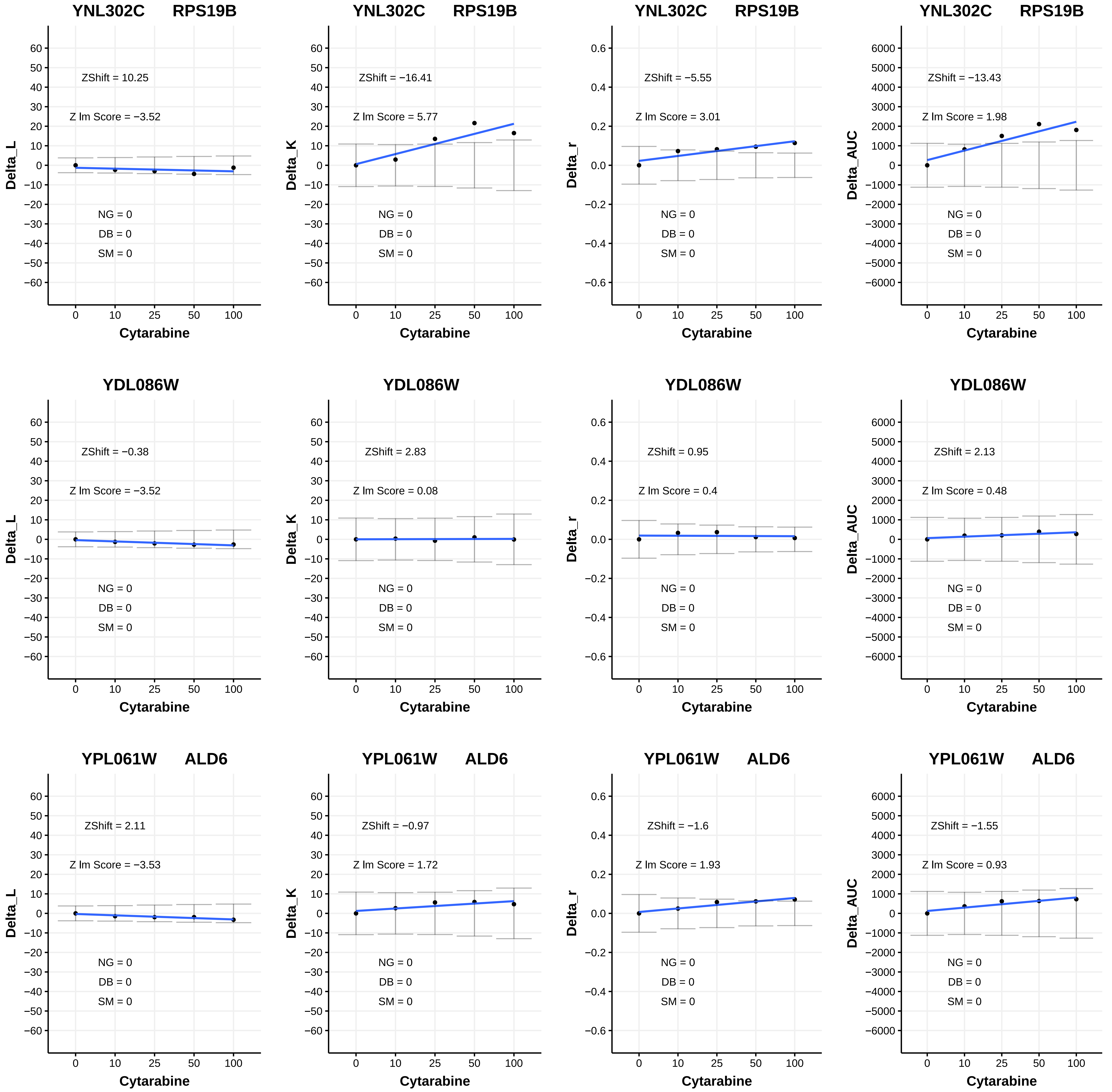
YNL302C RPS19B
ZShift = 10.25
Z lm Score = −3.52
NG = 0
DB = 0
SM = 0
Delta_L
Cytarabine
YNL302C RPS19B
ZShift = −16.41
Z lm Score = 5.77
NG = 0
DB = 0
SM = 0
Delta_K
Cytarabine
YNL302C RPS19B
ZShift = −5.55
Z lm Score = 3.01
NG = 0
DB = 0
SM = 0
Delta_r
Cytarabine
YNL302C RPS19B
ZShift = −13.43
Z lm Score = 1.98
NG = 0
DB = 0
SM = 0
Delta_AUC
Cytarabine
YDL086W
ZShift = −0.38
Z lm Score = −3.52
NG = 0
DB = 0
SM = 0
Delta_L
Cytarabine
YDL086W
ZShift = 2.83
Z lm Score = 0.08
NG = 0
DB = 0
SM = 0
Delta_K
Cytarabine
YDL086W
ZShift = 0.95
Z lm Score = 0.4
NG = 0
DB = 0
SM = 0
Delta_r
Cytarabine
YDL086W
ZShift = 2.13
Z lm Score = 0.48
NG = 0
DB = 0
SM = 0
Delta_AUC
Cytarabine
YPL061W ALD6
ZShift = 2.11
Z lm Score = −3.53
NG = 0
DB = 0
SM = 0
Delta_L
Cytarabine
YPL061W ALD6
ZShift = −0.97
Z lm Score = 1.72
NG = 0
DB = 0
SM = 0
Delta_K
Cytarabine
YPL061W ALD6
ZShift = −1.6
Z lm Score = 1.93
NG = 0
DB = 0
SM = 0
Delta_r
Cytarabine
YPL061W ALD6
ZShift = −1.55
Z lm Score = 0.93
NG = 0
DB = 0
SM = 0
Delta_AUC
Cytarabine

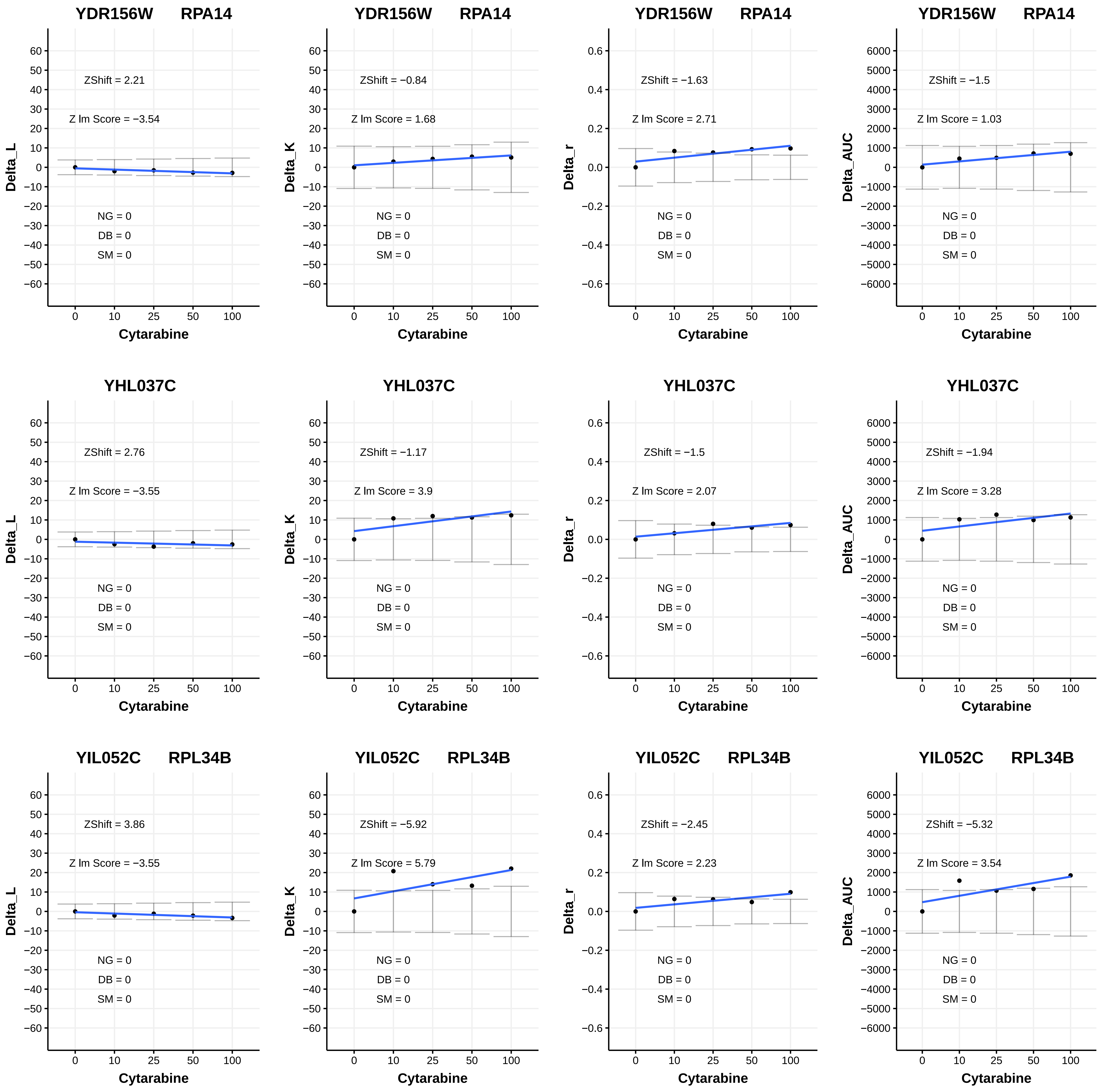

YDR156W RPA14
ZShift = 2.21
Z lm Score = −3.54
NG = 0
DB = 0
SM = 0
Delta_L
Cytarabine
YDR156W RPA14
ZShift = −0.84
Z lm Score = 1.68
NG = 0
DB = 0
SM = 0
Delta_K
Cytarabine
YDR156W RPA14
ZShift = −1.63
Z lm Score = 2.71
NG = 0
DB = 0
SM = 0
Delta_r
Cytarabine
YDR156W RPA14
ZShift = −1.5
Z lm Score = 1.03
NG = 0
DB = 0
SM = 0
Delta_AUC
Cytarabine
YHL037C
ZShift = 2.76
Z lm Score = −3.55
NG = 0
DB = 0
SM = 0
Delta_L
Cytarabine
YHL037C
ZShift = −1.17
Z lm Score = 3.9
NG = 0
DB = 0
SM = 0
Delta_K
Cytarabine
YHL037C
ZShift = −1.5
Z lm Score = 2.07
NG = 0
DB = 0
SM = 0
Delta_r
Cytarabine
YHL037C
ZShift = −1.94
Z lm Score = 3.28
NG = 0
DB = 0
SM = 0
Delta_AUC
Cytarabine
YIL052C RPL34B
ZShift = 3.86
Z lm Score = −3.55
NG = 0
DB = 0
SM = 0
Delta_L
Cytarabine
YIL052C RPL34B
ZShift = −5.92
Z lm Score = 5.79
NG = 0
DB = 0
SM = 0
Delta_K
Cytarabine
YIL052C RPL34B
ZShift = −2.45
Z lm Score = 2.23
NG = 0
DB = 0
SM = 0
Delta_r
Cytarabine
YIL052C RPL34B
ZShift = −5.32
Z lm Score = 3.54
NG = 0
DB = 0
SM = 0
Delta_AUC
Cytarabine

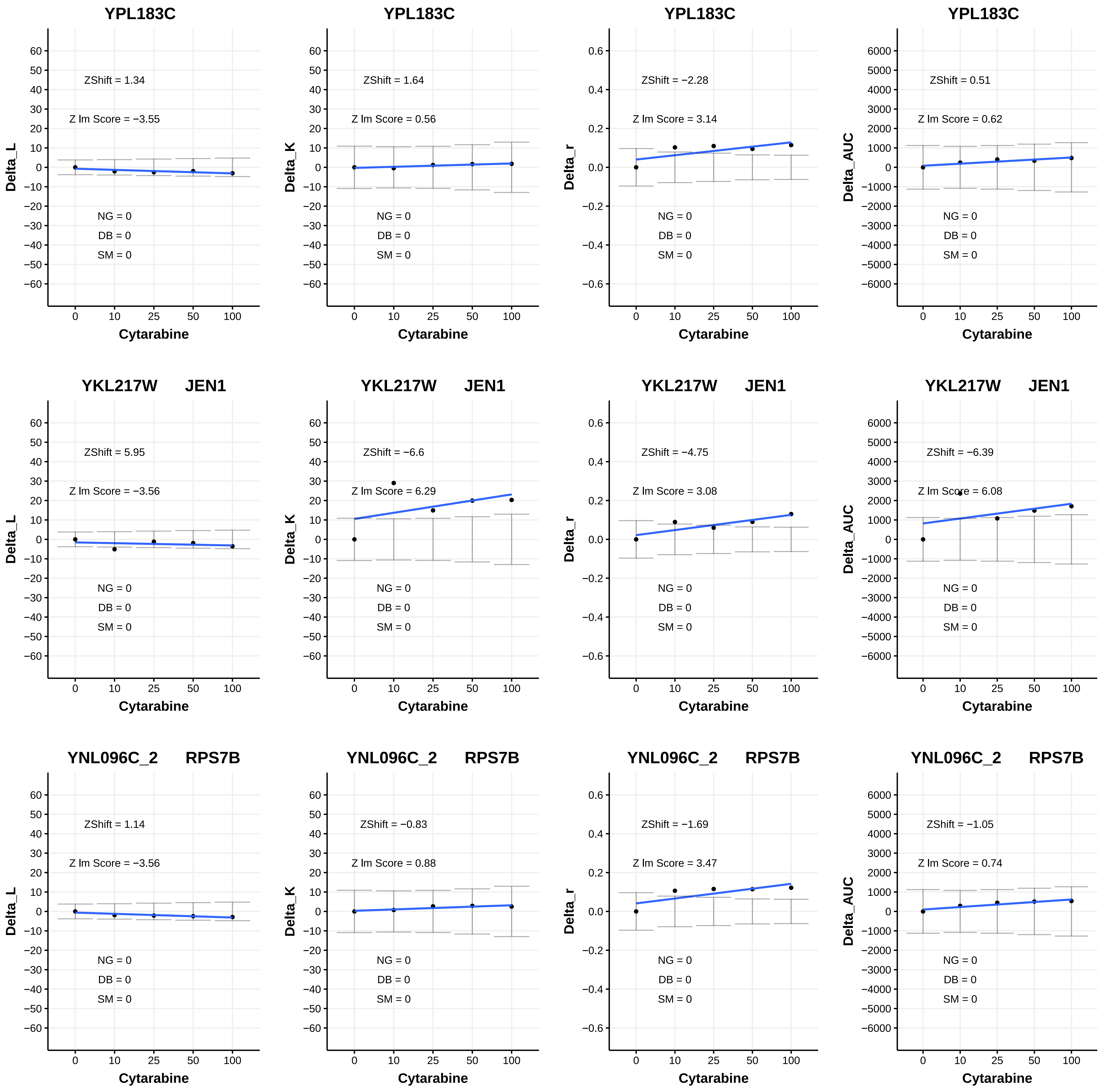

YPL183C
ZShift = 1.34
Z lm Score = −3.55
NG = 0
DB = 0
SM = 0
Delta_L
Cytarabine
YPL183C
ZShift = 1.64
Z lm Score = 0.56
NG = 0
DB = 0
SM = 0
Delta_K
Cytarabine
YPL183C
ZShift = −2.28
Z lm Score = 3.14
NG = 0
DB = 0
SM = 0
Delta_r
Cytarabine
YPL183C
ZShift = 0.51
Z lm Score = 0.62
NG = 0
DB = 0
SM = 0
Delta_AUC
Cytarabine
YKL217W JEN1
ZShift = 5.95
Z lm Score = −3.56
NG = 0
DB = 0
SM = 0
Delta_L
Cytarabine
YKL217W JEN1
ZShift = −6.6
Z lm Score = 6.29
NG = 0
DB = 0
SM = 0
Delta_K
Cytarabine
YKL217W JEN1
ZShift = −4.75
Z lm Score = 3.08
NG = 0
DB = 0
SM = 0
Delta_r
Cytarabine
YKL217W JEN1
ZShift = −6.39
Z lm Score = 6.08
NG = 0
DB = 0
SM = 0
Delta_AUC
Cytarabine
YNL096C_2 RPS7B
ZShift = 1.14
Z lm Score = −3.56
NG = 0
DB = 0
SM = 0
Delta_L
Cytarabine
YNL096C_2 RPS7B
ZShift = −0.83
Z lm Score = 0.88
NG = 0
DB = 0
SM = 0
Delta_K
Cytarabine
YNL096C_2 RPS7B
ZShift = −1.69
Z lm Score = 3.47
NG = 0
DB = 0
SM = 0
Delta_r
Cytarabine
YNL096C_2 RPS7B
ZShift = −1.05
Z lm Score = 0.74
NG = 0
DB = 0
SM = 0
Delta_AUC
Cytarabine

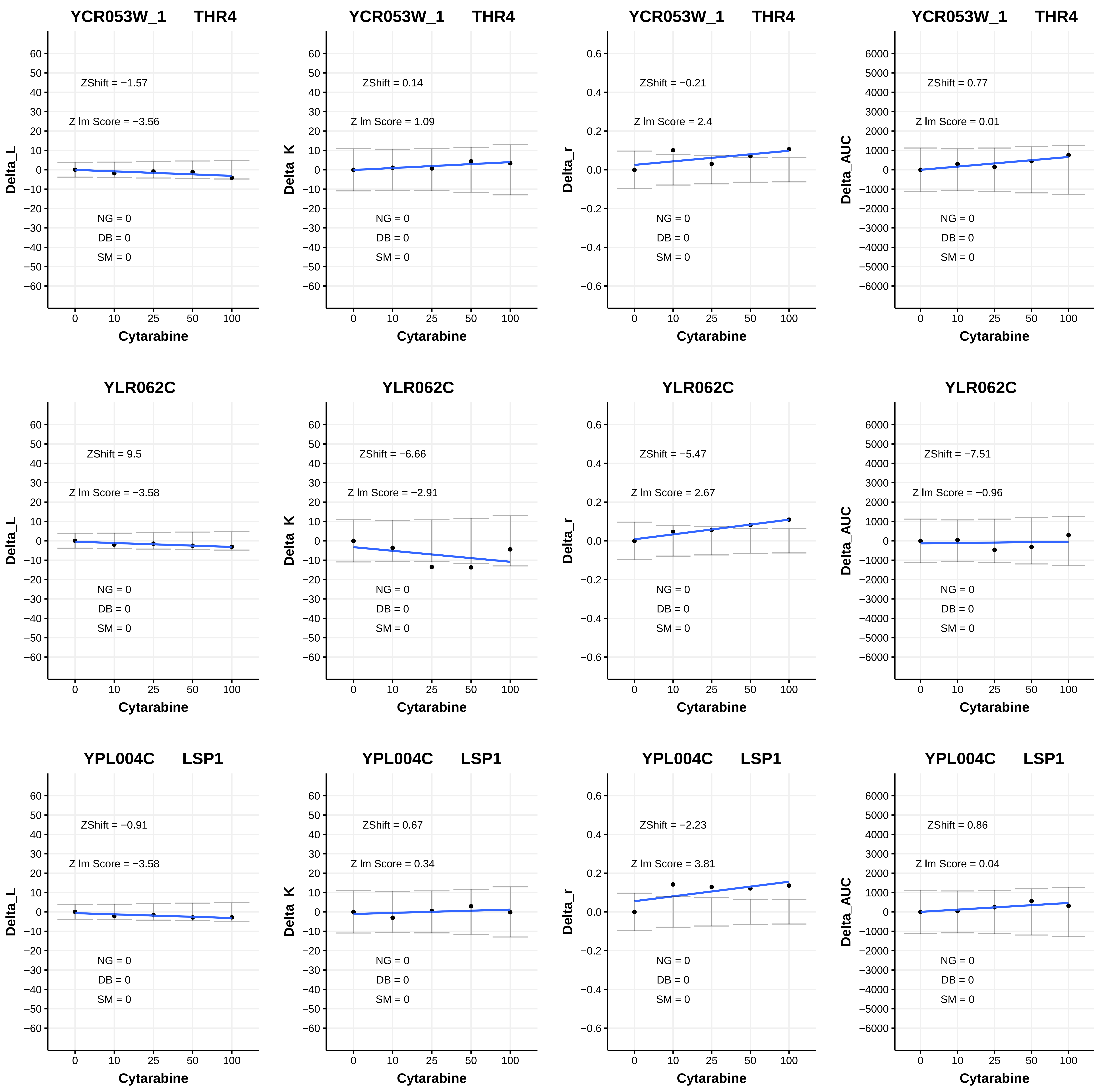

YCR053W_1 THR4
ZShift = −1.57
Z lm Score = −3.56
NG = 0
DB = 0
SM = 0
Delta_L
Cytarabine
YCR053W_1 THR4
ZShift = 0.14
Z lm Score = 1.09
NG = 0
DB = 0
SM = 0
Delta_K
Cytarabine
YCR053W_1 THR4
ZShift = −0.21
Z lm Score = 2.4
NG = 0
DB = 0
SM = 0
Delta_r
Cytarabine
YCR053W_1 THR4
ZShift = 0.77
Z lm Score = 0.01
NG = 0
DB = 0
SM = 0
Delta_AUC
Cytarabine
YLR062C
ZShift = 9.5
Z lm Score = −3.58
NG = 0
DB = 0
SM = 0
Delta_L
Cytarabine
YLR062C
ZShift = −6.66
Z lm Score = −2.91
NG = 0
DB = 0
SM = 0
Delta_K
Cytarabine
YLR062C
ZShift = −5.47
Z lm Score = 2.67
NG = 0
DB = 0
SM = 0
Delta_r
Cytarabine
YLR062C
ZShift = −7.51
Z lm Score = −0.96
NG = 0
DB = 0
SM = 0
Delta_AUC
Cytarabine
YPL004C LSP1
ZShift = −0.91
Z lm Score = −3.58
NG = 0
DB = 0
SM = 0
Delta_L
Cytarabine
YPL004C LSP1
ZShift = 0.67
Z lm Score = 0.34
NG = 0
DB = 0
SM = 0
Delta_K
Cytarabine
YPL004C LSP1
ZShift = −2.23
Z lm Score = 3.81
NG = 0
DB = 0
SM = 0
Delta_r
Cytarabine
YPL004C LSP1
ZShift = 0.86
Z lm Score = 0.04
NG = 0
DB = 0
SM = 0
Delta_AUC
Cytarabine

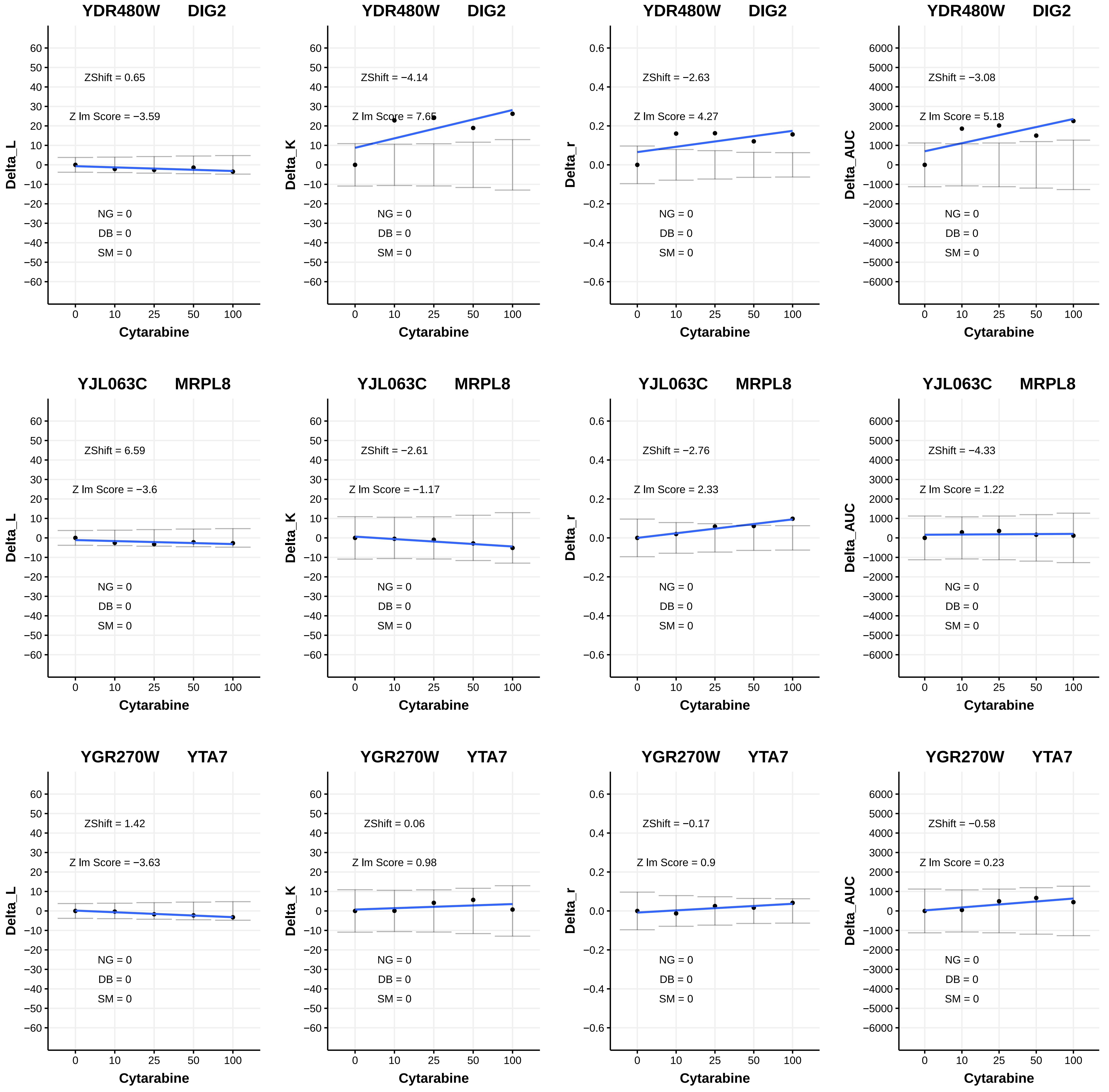
YDR480W DIG2
ZShift = 0.65
Z lm Score = −3.59
NG = 0
DB = 0
SM = 0
Delta_L
Cytarabine
YDR480W DIG2
ZShift = −4.14
Z lm Score = 7.65
NG = 0
DB = 0
SM = 0
Delta_K
Cytarabine
YDR480W DIG2
ZShift = −2.63
Z lm Score = 4.27
NG = 0
DB = 0
SM = 0
Delta_r
Cytarabine
YDR480W DIG2
ZShift = −3.08
Z lm Score = 5.18
NG = 0
DB = 0
SM = 0
Delta_AUC
Cytarabine
YJL063C MRPL8
ZShift = 6.59
Z lm Score = −3.6
NG = 0
DB = 0
SM = 0
Delta_L
Cytarabine
YJL063C MRPL8
ZShift = −2.61
Z lm Score = −1.17
NG = 0
DB = 0
SM = 0
Delta_K
Cytarabine
YJL063C MRPL8
ZShift = −2.76
Z lm Score = 2.33
NG = 0
DB = 0
SM = 0
Delta_r
Cytarabine
YJL063C MRPL8
ZShift = −4.33
Z lm Score = 1.22
NG = 0
DB = 0
SM = 0
Delta_AUC
Cytarabine
YGR270W YTA7
ZShift = 1.42
Z lm Score = −3.63
NG = 0
DB = 0
SM = 0
Delta_L
Cytarabine
YGR270W YTA7
ZShift = 0.06
Z lm Score = 0.98
NG = 0
DB = 0
SM = 0
Delta_K
Cytarabine
YGR270W YTA7
ZShift = −0.17
Z lm Score = 0.9
NG = 0
DB = 0
SM = 0
Delta_r
Cytarabine
YGR270W YTA7
ZShift = −0.58
Z lm Score = 0.23
NG = 0
DB = 0
SM = 0
Delta_AUC
Cytarabine

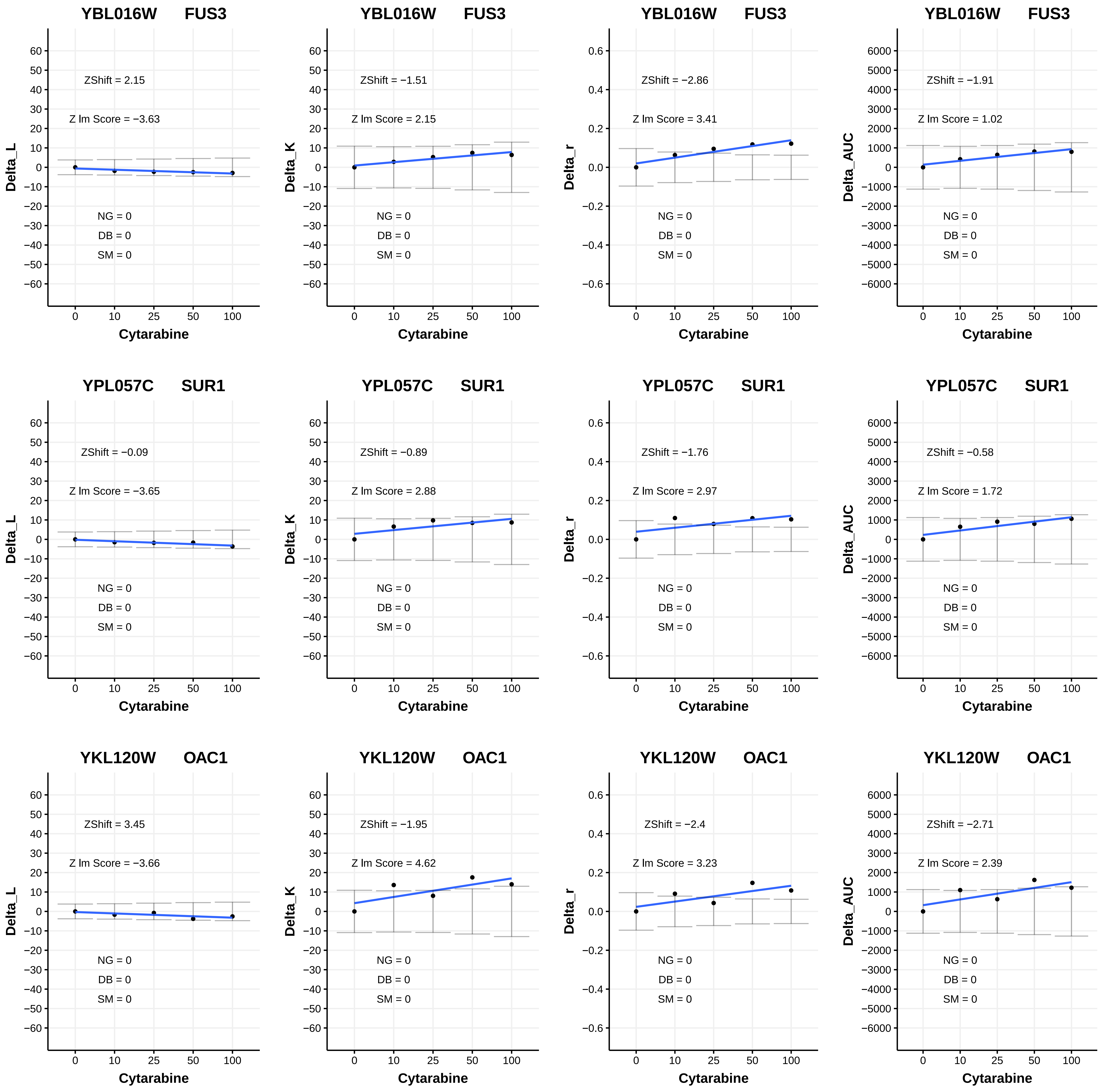
YBL016W FUS3
Delta_L
ZShift = 2.15
Z lm Score = −3.63
NG = 0
DB = 0
SM = 0
Cytarabine
YBL016W FUS3
Delta_K
ZShift = −1.51
Z lm Score = 2.15
NG = 0
DB = 0
SM = 0
Cytarabine
YBL016W FUS3
Delta_r
ZShift = −2.86
Z lm Score = 3.41
NG = 0
DB = 0
SM = 0
Cytarabine
YBL016W FUS3
Delta_AUC
ZShift = −1.91
Z lm Score = 1.02
NG = 0
DB = 0
SM = 0
Cytarabine
YPL057C SUR1
Delta_L
ZShift = −0.09
Z lm Score = −3.65
NG = 0
DB = 0
SM = 0
Cytarabine
YPL057C SUR1
Delta_K
ZShift = −0.89
Z lm Score = 2.88
NG = 0
DB = 0
SM = 0
Cytarabine
YPL057C SUR1
Delta_r
ZShift = −1.76
Z lm Score = 2.97
NG = 0
DB = 0
SM = 0
Cytarabine
YPL057C SUR1
Delta_AUC
ZShift = −0.58
Z lm Score = 1.72
NG = 0
DB = 0
SM = 0
Cytarabine
YKL120W OAC1
Delta_L
ZShift = 3.45
Z lm Score = −3.66
NG = 0
DB = 0
SM = 0
Cytarabine
YKL120W OAC1
Delta_K
ZShift = −1.95
Z lm Score = 4.62
NG = 0
DB = 0
SM = 0
Cytarabine
YKL120W OAC1
Delta_r
ZShift = −2.4
Z lm Score = 3.23
NG = 0
DB = 0
SM = 0
Cytarabine
YKL120W OAC1
Delta_AUC
ZShift = −2.71
Z lm Score = 2.39
NG = 0
DB = 0
SM = 0
Cytarabine

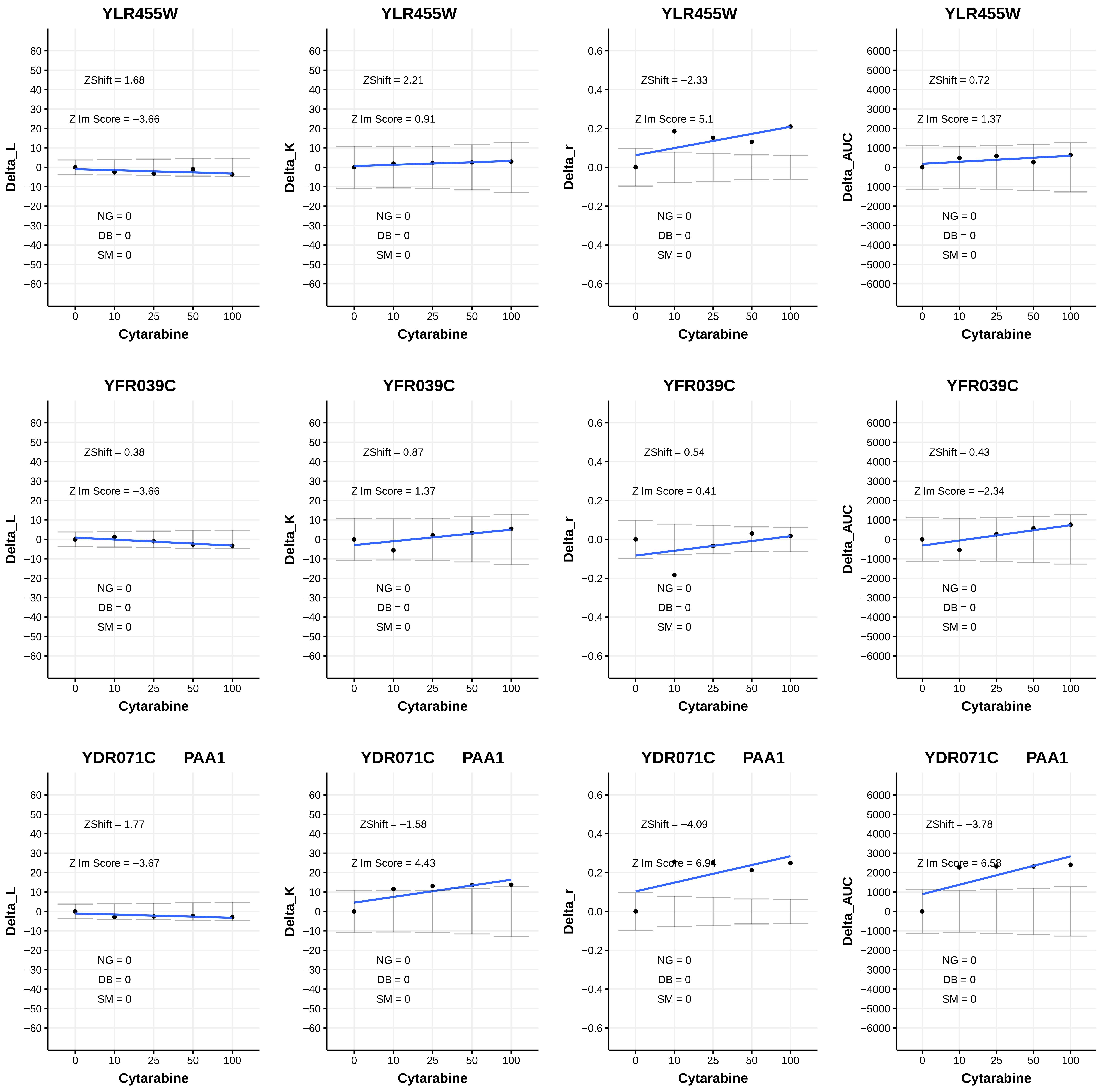

YLR455W
ZShift = 1.68
Z lm Score = −3.66
NG = 0
DB = 0
SM = 0
Delta_L
Cytarabine
YLR455W
ZShift = 2.21
Z lm Score = 0.91
NG = 0
DB = 0
SM = 0
Delta_K
Cytarabine
YLR455W
ZShift = −2.33
Z lm Score = 5.1
NG = 0
DB = 0
SM = 0
Delta_r
Cytarabine
YLR455W
ZShift = 0.72
Z lm Score = 1.37
NG = 0
DB = 0
SM = 0
Delta_AUC
Cytarabine
YFR039C
ZShift = 0.38
Z lm Score = −3.66
NG = 0
DB = 0
SM = 0
Delta_L
Cytarabine
YFR039C
ZShift = 0.87
Z lm Score = 1.37
NG = 0
DB = 0
SM = 0
Delta_K
Cytarabine
YFR039C
ZShift = 0.54
Z lm Score = 0.41
NG = 0
DB = 0
SM = 0
Delta_r
Cytarabine
YFR039C
ZShift = 0.43
Z lm Score = −2.34
NG = 0
DB = 0
SM = 0
Delta_AUC
Cytarabine
YDR071C PAA1
ZShift = 1.77
Z lm Score = −3.67
NG = 0
DB = 0
SM = 0
Delta_L
Cytarabine
YDR071C PAA1
ZShift = −1.58
Z lm Score = 4.43
NG = 0
DB = 0
SM = 0
Delta_K
Cytarabine
YDR071C PAA1
ZShift = −4.09
Z lm Score = 6.94
NG = 0
DB = 0
SM = 0
Delta_r
Cytarabine
YDR071C PAA1
ZShift = −3.78
Z lm Score = 6.58
NG = 0
DB = 0
SM = 0
Delta_AUC
Cytarabine

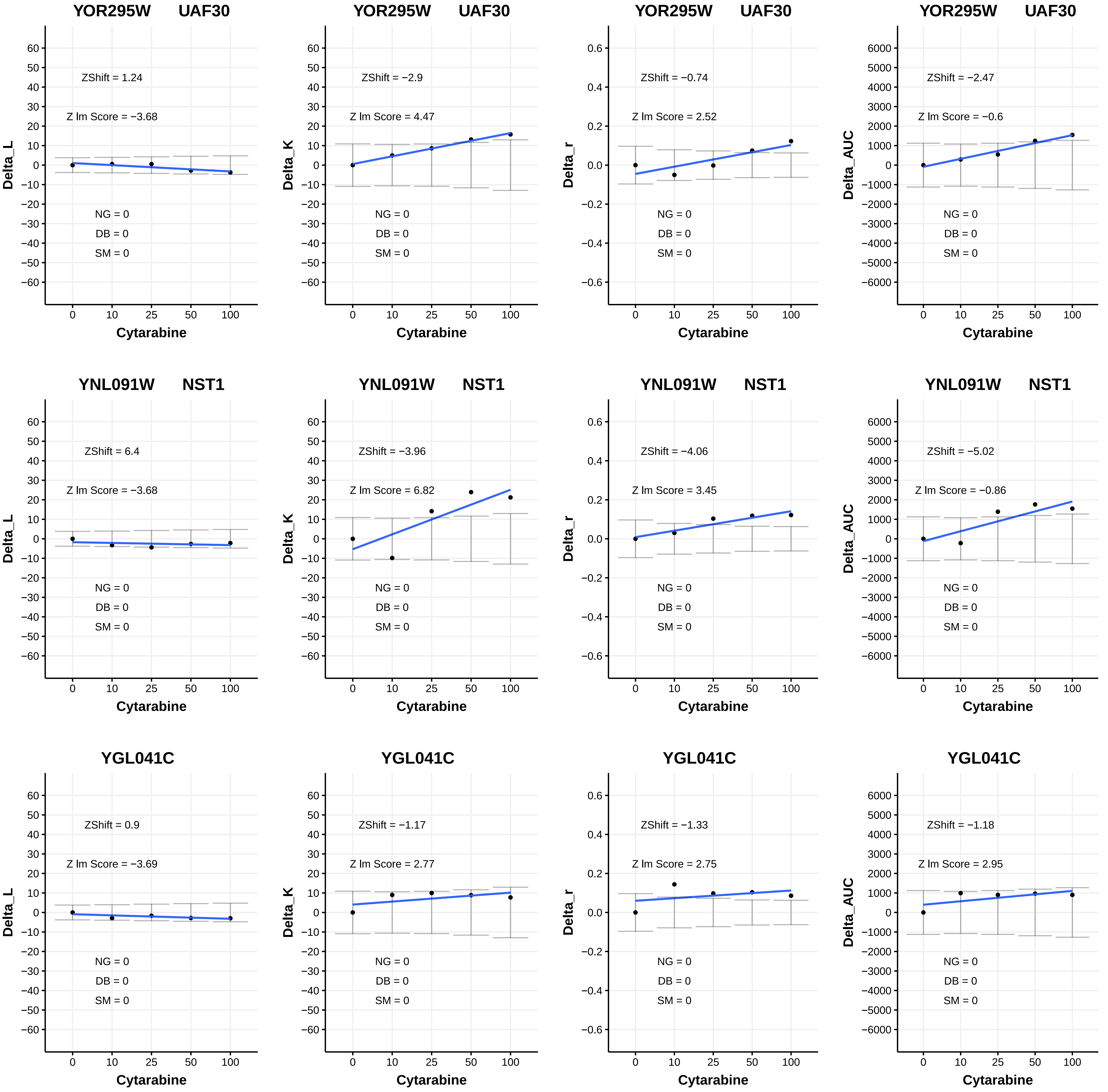

YOR295W UAF30
ZShift = 1.24
Z lm Score = −3.68
NG = 0
DB = 0
SM = 0
Delta_L
Cytarabine
YOR295W UAF30
ZShift = −2.9
Z lm Score = 4.47
NG = 0
DB = 0
SM = 0
Delta_K
Cytarabine
YOR295W UAF30
ZShift = −0.74
Z lm Score = 2.52
NG = 0
DB = 0
SM = 0
Delta_r
Cytarabine
YOR295W UAF30
ZShift = −2.47
Z lm Score = −0.6
NG = 0
DB = 0
SM = 0
Delta_AUC
Cytarabine
YNL091W NST1
ZShift = 6.4
Z lm Score = −3.68
NG = 0
DB = 0
SM = 0
Delta_L
Cytarabine
YNL091W NST1
ZShift = −3.96
Z lm Score = 6.82
NG = 0
DB = 0
SM = 0
Delta_K
Cytarabine
YNL091W NST1
ZShift = −4.06
Z lm Score = 3.45
NG = 0
DB = 0
SM = 0
Delta_r
Cytarabine
YNL091W NST1
ZShift = −5.02
Z lm Score = −0.86
NG = 0
DB = 0
SM = 0
Delta_AUC
Cytarabine
YGL041C
ZShift = 0.9
Z lm Score = −3.69
NG = 0
DB = 0
SM = 0
Delta_L
Cytarabine
YGL041C
ZShift = −1.17
Z lm Score = 2.77
NG = 0
DB = 0
SM = 0
Delta_K
Cytarabine
YGL041C
ZShift = −1.33
Z lm Score = 2.75
NG = 0
DB = 0
SM = 0
Delta_r
Cytarabine
YGL041C
ZShift = −1.18
Z lm Score = 2.95
NG = 0
DB = 0
SM = 0
Delta_AUC
Cytarabine

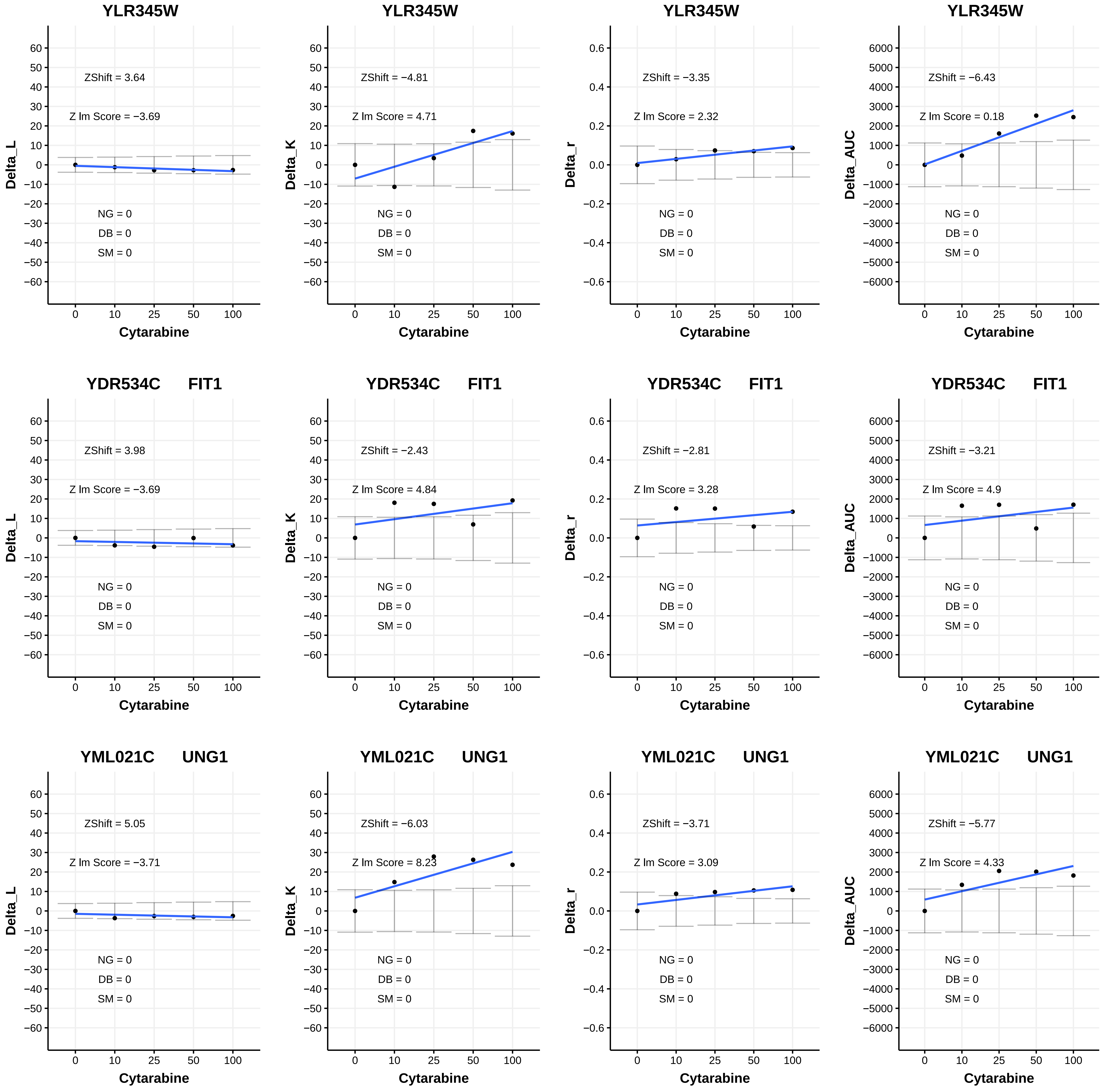
YLR345W
ZShift = 3.64
Z lm Score = −3.69
NG = 0
DB = 0
SM = 0
Delta_L
Cytarabine
YLR345W
ZShift = −4.81
Z lm Score = 4.71
NG = 0
DB = 0
SM = 0
Delta_K
Cytarabine
YLR345W
ZShift = −3.35
Z lm Score = 2.32
NG = 0
DB = 0
SM = 0
Delta_r
Cytarabine
YLR345W
ZShift = −6.43
Z lm Score = 0.18
NG = 0
DB = 0
SM = 0
Delta_AUC
Cytarabine
YDR534C FIT1
ZShift = 3.98
Z lm Score = −3.69
NG = 0
DB = 0
SM = 0
Delta_L
Cytarabine
YDR534C FIT1
ZShift = −2.43
Z lm Score = 4.84
NG = 0
DB = 0
SM = 0
Delta_K
Cytarabine
YDR534C FIT1
ZShift = −2.81
Z lm Score = 3.28
NG = 0
DB = 0
SM = 0
Delta_r
Cytarabine
YDR534C FIT1
ZShift = −3.21
Z lm Score = 4.9
NG = 0
DB = 0
SM = 0
Delta_AUC
Cytarabine
YML021C UNG1
ZShift = 5.05
Z lm Score = −3.71
NG = 0
DB = 0
SM = 0
Delta_L
Cytarabine
YML021C UNG1
ZShift = −6.03
Z lm Score = 8.23
NG = 0
DB = 0
SM = 0
Delta_K
Cytarabine
YML021C UNG1
ZShift = −3.71
Z lm Score = 3.09
NG = 0
DB = 0
SM = 0
Delta_r
Cytarabine
YML021C UNG1
ZShift = −5.77
Z lm Score = 4.33
NG = 0
DB = 0
SM = 0
Delta_AUC
Cytarabine

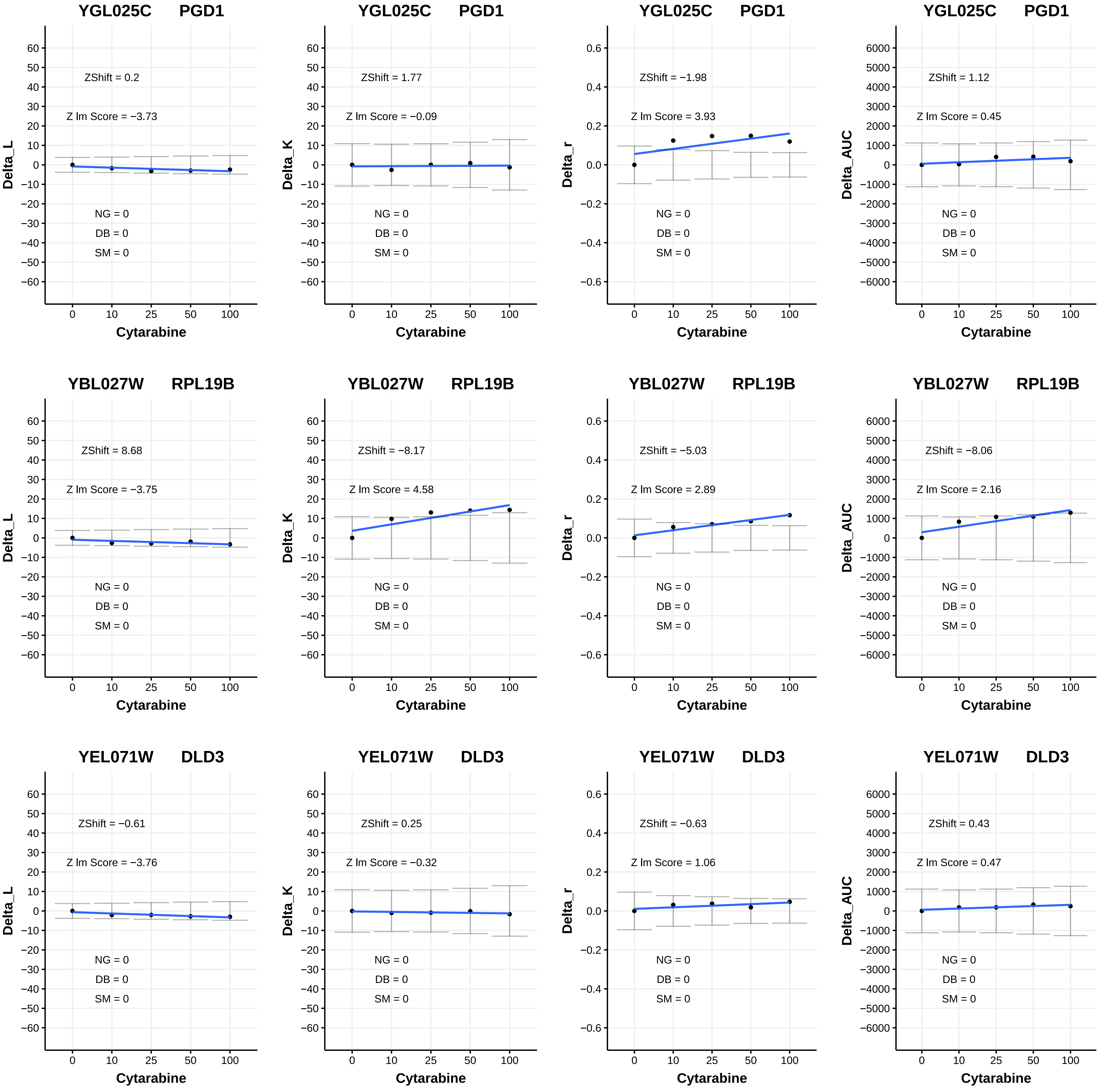

YGL025C PGD1
ZShift = 0.2
Z lm Score = −3.73
NG = 0
DB = 0
SM = 0
Delta_L
Cytarabine
YGL025C PGD1
ZShift = 1.77
Z lm Score = −0.09
NG = 0
DB = 0
SM = 0
Delta_K
Cytarabine
YGL025C PGD1
ZShift = −1.98
Z lm Score = 3.93
NG = 0
DB = 0
SM = 0
Delta_r
Cytarabine
YGL025C PGD1
ZShift = 1.12
Z lm Score = 0.45
NG = 0
DB = 0
SM = 0
Delta_AUC
Cytarabine
YBL027W RPL19B
ZShift = 8.68
Z lm Score = −3.75
NG = 0
DB = 0
SM = 0
Delta_L
Cytarabine
YBL027W RPL19B
ZShift = −8.17
Z lm Score = 4.58
NG = 0
DB = 0
SM = 0
Delta_K
Cytarabine
YBL027W RPL19B
ZShift = −5.03
Z lm Score = 2.89
NG = 0
DB = 0
SM = 0
Delta_r
Cytarabine
YBL027W RPL19B
ZShift = −8.06
Z lm Score = 2.16
NG = 0
DB = 0
SM = 0
Delta_AUC
Cytarabine
YEL071W DLD3
ZShift = −0.61
Z lm Score = −3.76
NG = 0
DB = 0
SM = 0
Delta_L
Cytarabine
YEL071W DLD3
ZShift = 0.25
Z lm Score = −0.32
NG = 0
DB = 0
SM = 0
Delta_K
Cytarabine
YEL071W DLD3
ZShift = −0.63
Z lm Score = 1.06
NG = 0
DB = 0
SM = 0
Delta_r
Cytarabine
YEL071W DLD3
ZShift = 0.43
Z lm Score = 0.47
NG = 0
DB = 0
SM = 0
Delta_AUC
Cytarabine

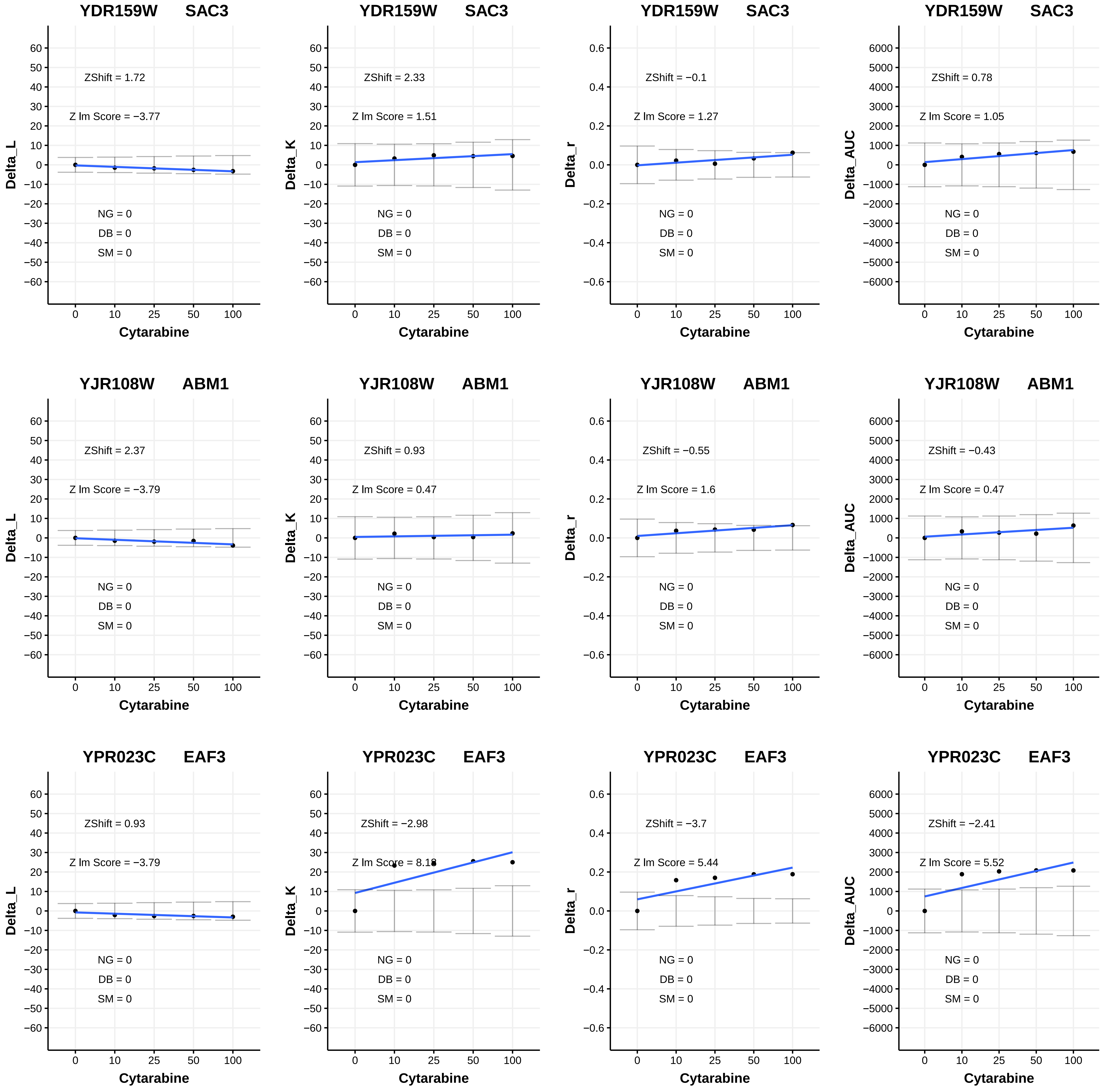

YDR159W SAC3
ZShift = 1.72
Z lm Score = −3.77
NG = 0
DB = 0
SM = 0
Delta_L
Cytarabine
YDR159W SAC3
ZShift = 2.33
Z lm Score = 1.51
NG = 0
DB = 0
SM = 0
Delta_K
Cytarabine
YDR159W SAC3
ZShift = −0.1
Z lm Score = 1.27
NG = 0
DB = 0
SM = 0
Delta_r
Cytarabine
YDR159W SAC3
ZShift = 0.78
Z lm Score = 1.05
NG = 0
DB = 0
SM = 0
Delta_AUC
Cytarabine
YJR108W ABM1
ZShift = 2.37
Z lm Score = −3.79
NG = 0
DB = 0
SM = 0
Delta_L
Cytarabine
YJR108W ABM1
ZShift = 0.93
Z lm Score = 0.47
NG = 0
DB = 0
SM = 0
Delta_K
Cytarabine
YJR108W ABM1
ZShift = −0.55
Z lm Score = 1.6
NG = 0
DB = 0
SM = 0
Delta_r
Cytarabine
YJR108W ABM1
ZShift = −0.43
Z lm Score = 0.47
NG = 0
DB = 0
SM = 0
Delta_AUC
Cytarabine
YPR023C EAF3
ZShift = 0.93
Z lm Score = −3.79
NG = 0
DB = 0
SM = 0
Delta_L
Cytarabine
YPR023C EAF3
ZShift = −2.98
Z lm Score = 8.18
NG = 0
DB = 0
SM = 0
Delta_K
Cytarabine
YPR023C EAF3
ZShift = −3.7
Z lm Score = 5.44
NG = 0
DB = 0
SM = 0
Delta_r
Cytarabine
YPR023C EAF3
ZShift = −2.41
Z lm Score = 5.52
NG = 0
DB = 0
SM = 0
Delta_AUC
Cytarabine

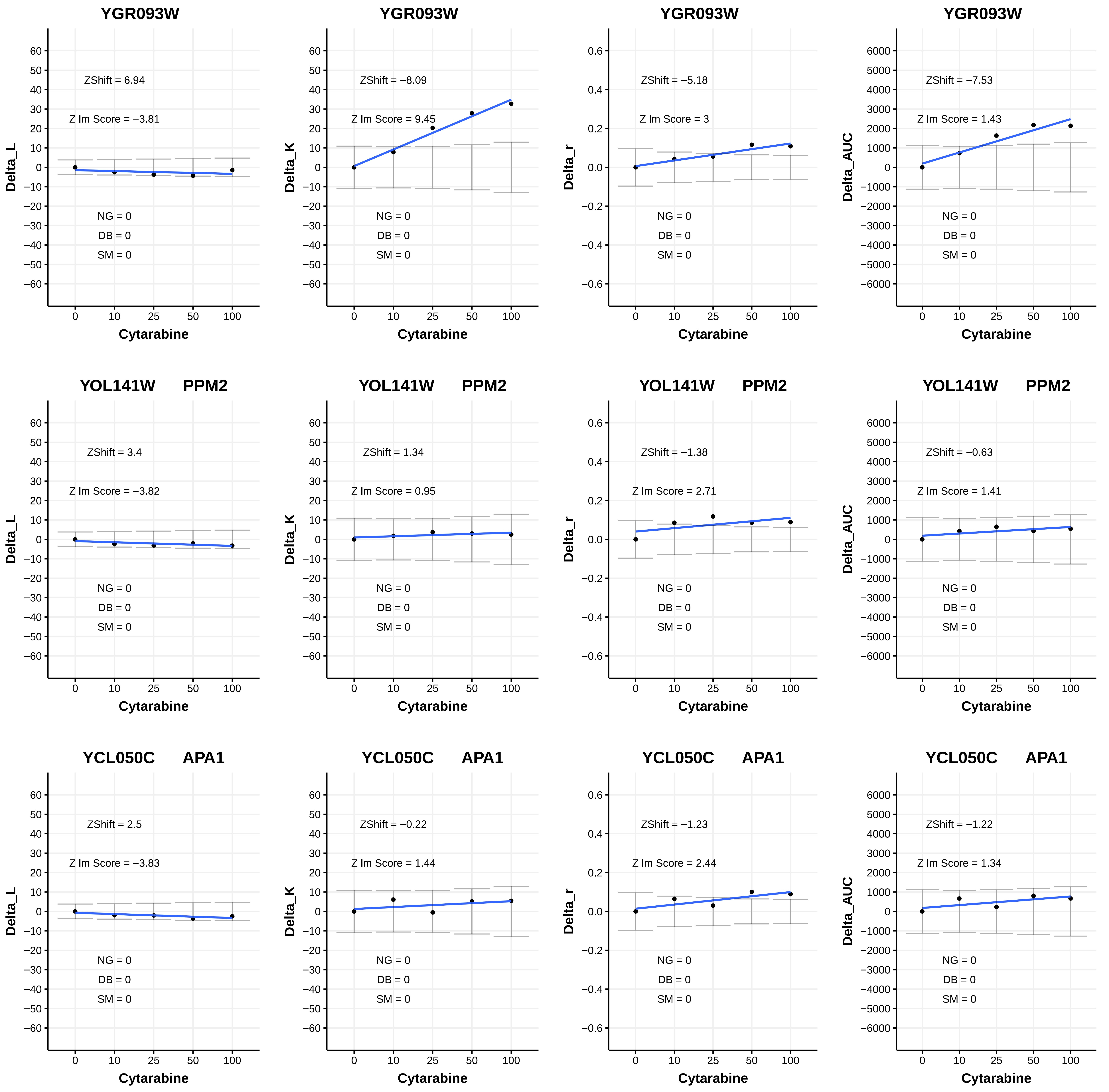

YGR093W
ZShift = 6.94
Z lm Score = −3.81
NG = 0
DB = 0
SM = 0
Delta_L
Cytarabine
YGR093W
ZShift = −8.09
Z lm Score = 9.45
NG = 0
DB = 0
SM = 0
Delta_K
Cytarabine
YGR093W
ZShift = −5.18
Z lm Score = 3
NG = 0
DB = 0
SM = 0
Delta_r
Cytarabine
YGR093W
ZShift = −7.53
Z lm Score = 1.43
NG = 0
DB = 0
SM = 0
Delta_AUC
Cytarabine
YOL141W PPM2
ZShift = 3.4
Z lm Score = −3.82
NG = 0
DB = 0
SM = 0
Delta_L
Cytarabine
YOL141W PPM2
ZShift = 1.34
Z lm Score = 0.95
NG = 0
DB = 0
SM = 0
Delta_K
Cytarabine
YOL141W PPM2
ZShift = −1.38
Z lm Score = 2.71
NG = 0
DB = 0
SM = 0
Delta_r
Cytarabine
YOL141W PPM2
ZShift = −0.63
Z lm Score = 1.41
NG = 0
DB = 0
SM = 0
Delta_AUC
Cytarabine
YCL050C APA1
ZShift = 2.5
Z lm Score = −3.83
NG = 0
DB = 0
SM = 0
Delta_L
Cytarabine
YCL050C APA1
ZShift = −0.22
Z lm Score = 1.44
NG = 0
DB = 0
SM = 0
Delta_K
Cytarabine
YCL050C APA1
ZShift = −1.23
Z lm Score = 2.44
NG = 0
DB = 0
SM = 0
Delta_r
Cytarabine
YCL050C APA1
ZShift = −1.22
Z lm Score = 1.34
NG = 0
DB = 0
SM = 0
Delta_AUC
Cytarabine

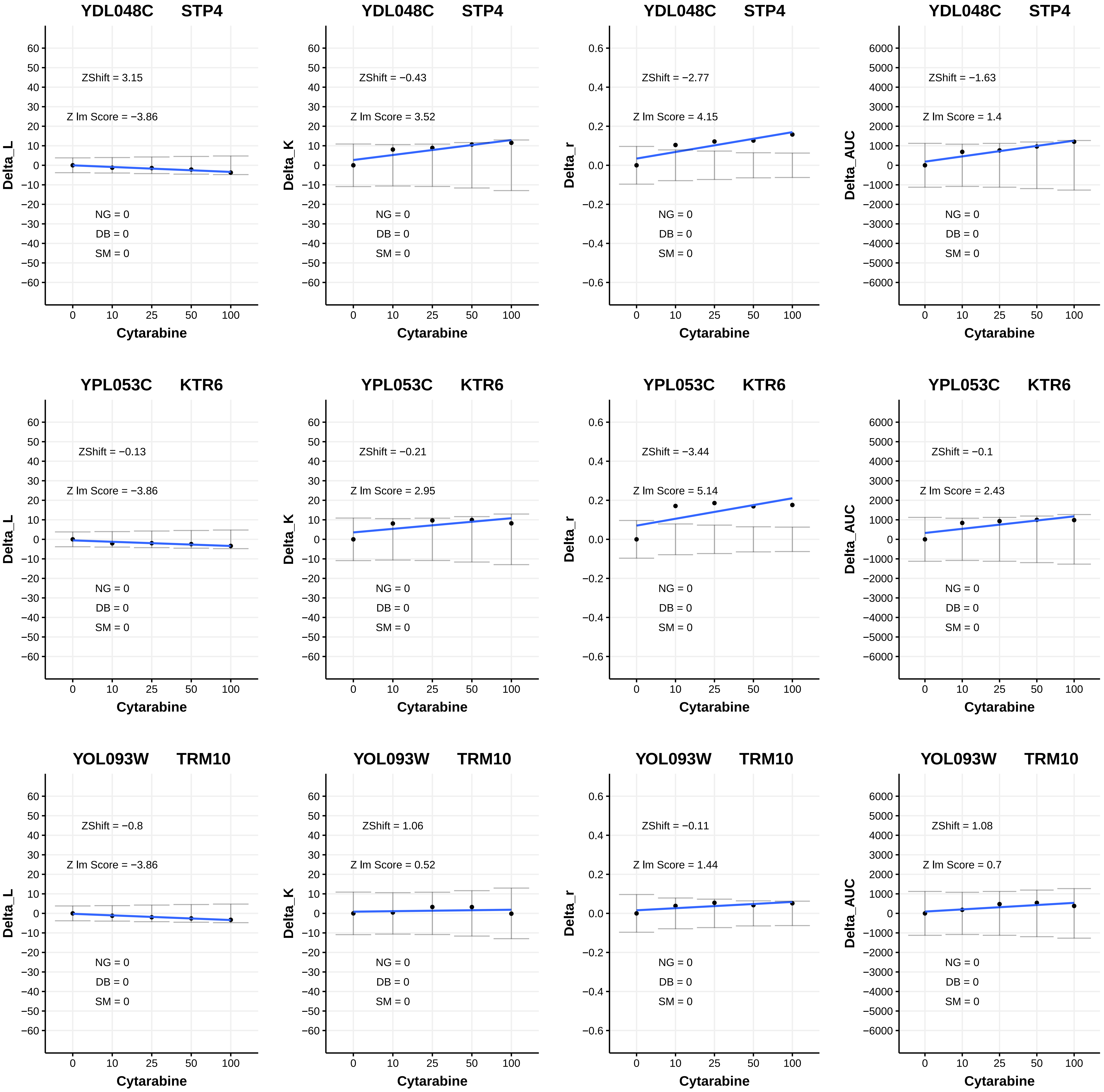

YDL048C STP4
ZShift = −3.15
Z lm Score = −3.86
NG = 0
DB = 0
SM = 0
Delta_L
Cytarabine
YDL048C STP4
ZShift = −0.43
Z lm Score = 3.52
NG = 0
DB = 0
SM = 0
Delta_K
Cytarabine
YDL048C STP4
ZShift = −2.77
Z lm Score = 4.15
NG = 0
DB = 0
SM = 0
Delta_r
Cytarabine
YDL048C STP4
ZShift = −1.63
Z lm Score = 1.4
NG = 0
DB = 0
SM = 0
Delta_AUC
Cytarabine
YPL053C KTR6
ZShift = −0.13
Z lm Score = −3.86
NG = 0
DB = 0
SM = 0
Delta_L
Cytarabine
YPL053C KTR6
ZShift = −0.21
Z lm Score = 2.95
NG = 0
DB = 0
SM = 0
Delta_K
Cytarabine
YPL053C KTR6
ZShift = −3.44
Z lm Score = 5.14
NG = 0
DB = 0
SM = 0
Delta_r
Cytarabine
YPL053C KTR6
ZShift = −0.1
Z lm Score = 2.43
NG = 0
DB = 0
SM = 0
Delta_AUC
Cytarabine
YOL093W TRM10
ZShift = −0.8
Z lm Score = −3.86
NG = 0
DB = 0
SM = 0
Delta_L
Cytarabine
YOL093W TRM10
ZShift = 1.06
Z lm Score = 0.52
NG = 0
DB = 0
SM = 0
Delta_K
Cytarabine
YOL093W TRM10
ZShift = −0.11
Z lm Score = 1.44
NG = 0
DB = 0
SM = 0
Delta_r
Cytarabine
YOL093W TRM10
ZShift = 1.08
Z lm Score = 0.7
NG = 0
DB = 0
SM = 0
Delta_AUC
Cytarabine

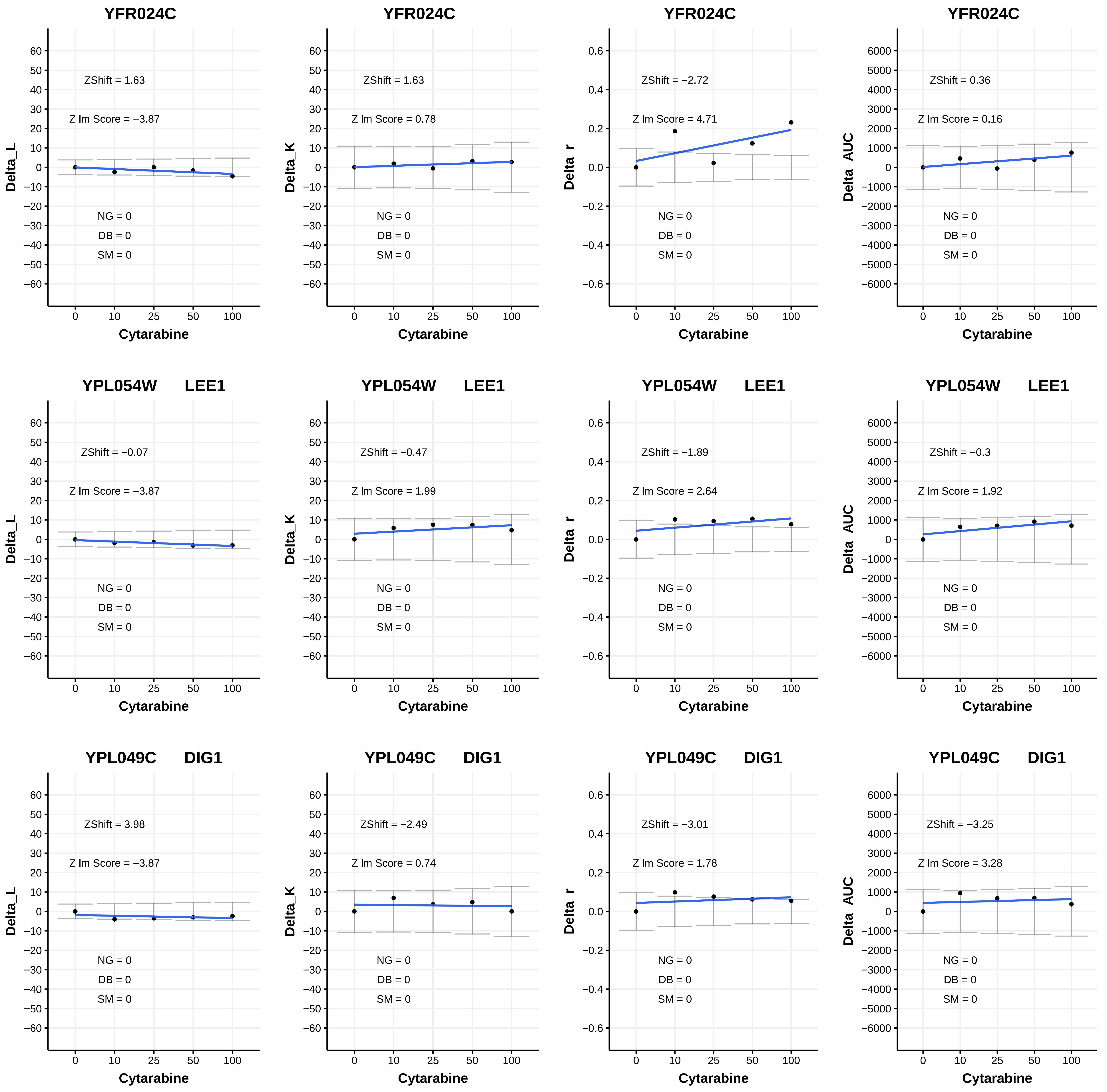
YFR024C
ZShift = 1.63
Z lm Score = −3.87
NG = 0
DB = 0
SM = 0
Delta_L
Cytarabine
YFR024C
ZShift = 1.63
Z lm Score = 0.78
NG = 0
DB = 0
SM = 0
Delta_K
Cytarabine
YFR024C
ZShift = −2.72
Z lm Score = 4.71
NG = 0
DB = 0
SM = 0
Delta_r
Cytarabine
YFR024C
ZShift = 0.36
Z lm Score = 0.16
NG = 0
DB = 0
SM = 0
Delta_AUC
Cytarabine
YPL054W LEE1
ZShift = −0.07
Z lm Score = −3.87
NG = 0
DB = 0
SM = 0
Delta_L
Cytarabine
YPL054W LEE1
ZShift = −0.47
Z lm Score = 1.99
NG = 0
DB = 0
SM = 0
Delta_K
Cytarabine
YPL054W LEE1
ZShift = −1.89
Z lm Score = 2.64
NG = 0
DB = 0
SM = 0
Delta_r
Cytarabine
YPL054W LEE1
ZShift = −0.3
Z lm Score = 1.92
NG = 0
DB = 0
SM = 0
Delta_AUC
Cytarabine
YPL049C DIG1
ZShift = 3.98
Z lm Score = −3.87
NG = 0
DB = 0
SM = 0
Delta_L
Cytarabine
YPL049C DIG1
ZShift = −2.49
Z lm Score = 0.74
NG = 0
DB = 0
SM = 0
Delta_K
Cytarabine
YPL049C DIG1
ZShift = −3.01
Z lm Score = 1.78
NG = 0
DB = 0
SM = 0
Delta_r
Cytarabine
YPL049C DIG1
ZShift = −3.25
Z lm Score = 3.28
NG = 0
DB = 0
SM = 0
Delta_AUC
Cytarabine

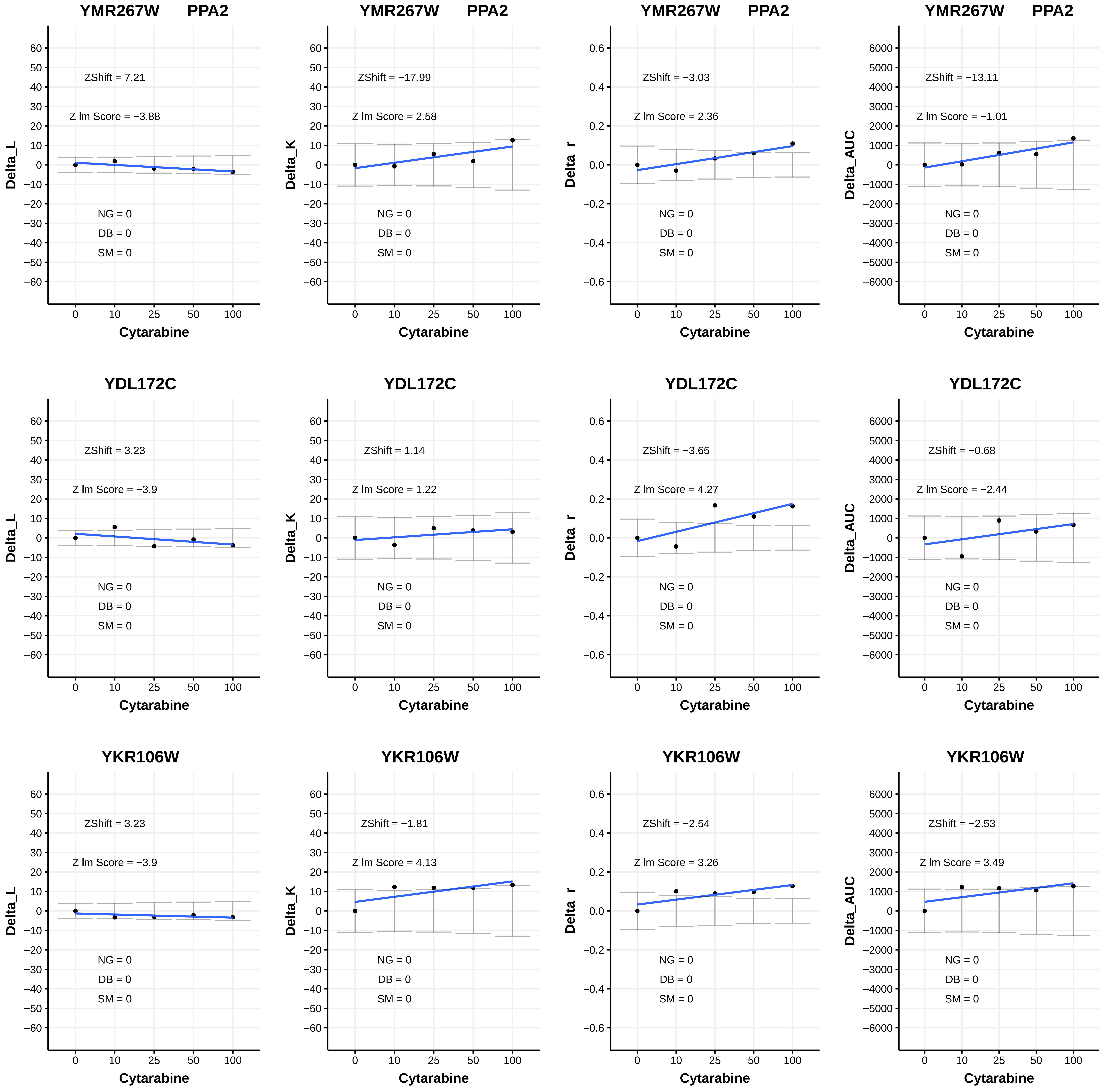

YMR267W PPA2
ZShift = 7.21
Z lm Score = −3.88
NG = 0
DB = 0
SM = 0
Delta_L
Cytarabine
YMR267W PPA2
ZShift = −17.99
Z lm Score = 2.58
NG = 0
DB = 0
SM = 0
Delta_K
Cytarabine
YMR267W PPA2
ZShift = −3.03
Z lm Score = 2.36
NG = 0
DB = 0
SM = 0
Delta_r
Cytarabine
YMR267W PPA2
ZShift = −13.11
Z lm Score = −1.01
NG = 0
DB = 0
SM = 0
Delta_AUC
Cytarabine
YDL172C
ZShift = 3.23
Z lm Score = −3.9
NG = 0
DB = 0
SM = 0
Delta_L
Cytarabine
YDL172C
ZShift = 1.14
Z lm Score = 1.22
NG = 0
DB = 0
SM = 0
Delta_K
Cytarabine
YDL172C
ZShift = −3.65
Z lm Score = 4.27
NG = 0
DB = 0
SM = 0
Delta_r
Cytarabine
YDL172C
ZShift = −0.68
Z lm Score = −2.44
NG = 0
DB = 0
SM = 0
Delta_AUC
Cytarabine
YKR106W
ZShift = 3.23
Z lm Score = −3.9
NG = 0
DB = 0
SM = 0
Delta_L
Cytarabine
YKR106W
ZShift = −1.81
Z lm Score = 4.13
NG = 0
DB = 0
SM = 0
Delta_K
Cytarabine
YKR106W
ZShift = −2.54
Z lm Score = 3.26
NG = 0
DB = 0
SM = 0
Delta_r
Cytarabine
YKR106W
ZShift = −2.53
Z lm Score = 3.49
NG = 0
DB = 0
SM = 0
Delta_AUC
Cytarabine

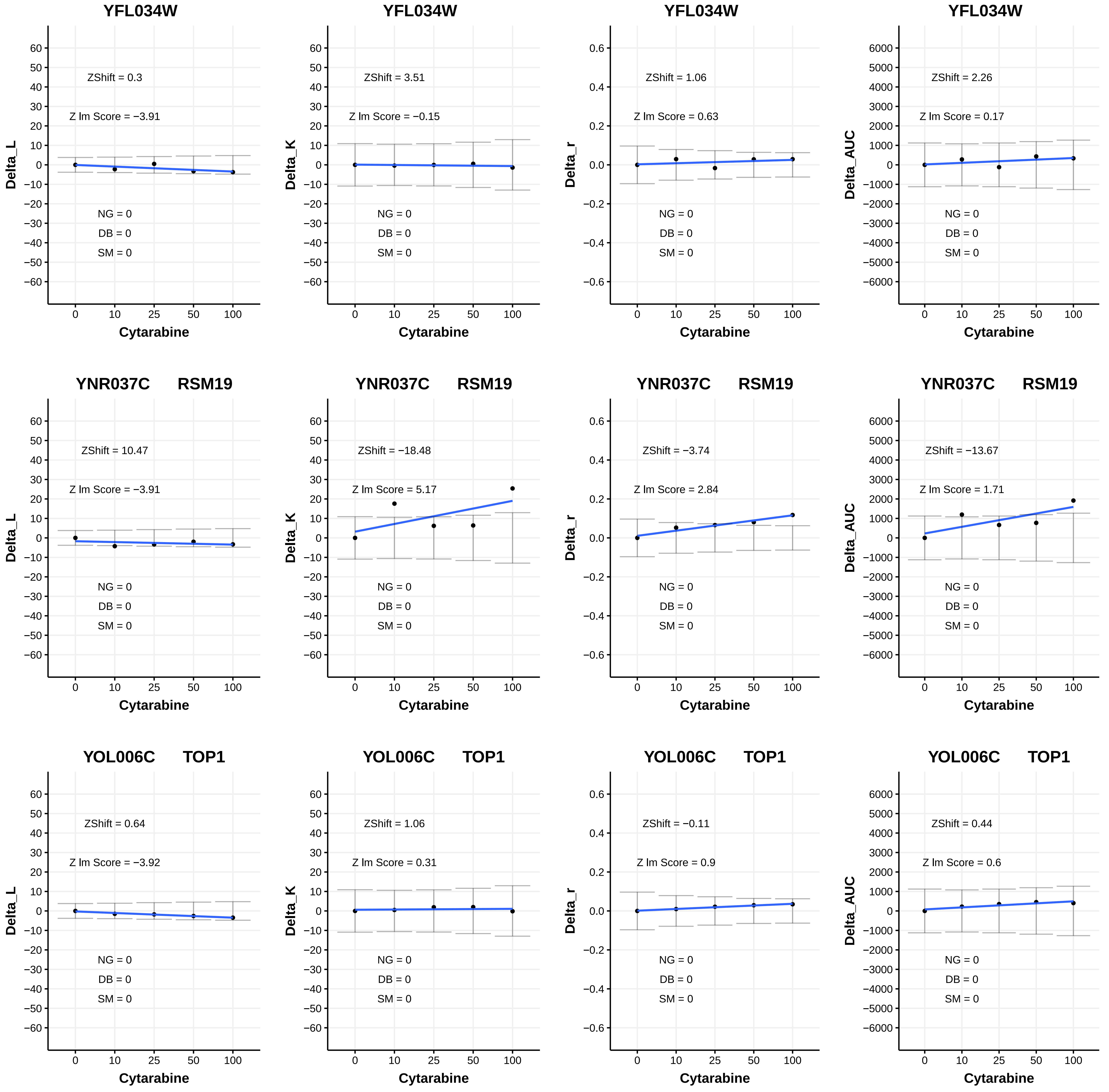

YFL034W
ZShift = 0.3
Z lm Score = −3.91
NG = 0
DB = 0
SM = 0
Delta_L
Cytarabine
YFL034W
ZShift = 3.51
Z lm Score = −0.15
NG = 0
DB = 0
SM = 0
Delta_K
Cytarabine
YFL034W
ZShift = 1.06
Z lm Score = 0.63
NG = 0
DB = 0
SM = 0
Delta_r
Cytarabine
YFL034W
ZShift = 2.26
Z lm Score = 0.17
NG = 0
DB = 0
SM = 0
Delta_AUC
Cytarabine
YNR037C RSM19
ZShift = 10.47
Z lm Score = −3.91
NG = 0
DB = 0
SM = 0
Delta_L
Cytarabine
YNR037C RSM19
ZShift = −18.48
Z lm Score = 5.17
NG = 0
DB = 0
SM = 0
Delta_K
Cytarabine
YNR037C RSM19
ZShift = −3.74
Z lm Score = 2.84
NG = 0
DB = 0
SM = 0
Delta_r
Cytarabine
YNR037C RSM19
ZShift = −13.67
Z lm Score = 1.71
NG = 0
DB = 0
SM = 0
Delta_AUC
Cytarabine
YOL006C TOP1
ZShift = 0.64
Z lm Score = −3.92
NG = 0
DB = 0
SM = 0
Delta_L
Cytarabine
YOL006C TOP1
ZShift = 1.06
Z lm Score = 0.31
NG = 0
DB = 0
SM = 0
Delta_K
Cytarabine
YOL006C TOP1
ZShift = −0.11
Z lm Score = 0.9
NG = 0
DB = 0
SM = 0
Delta_r
Cytarabine
YOL006C TOP1
ZShift = 0.44
Z lm Score = 0.6
NG = 0
DB = 0
SM = 0
Delta_AUC
Cytarabine

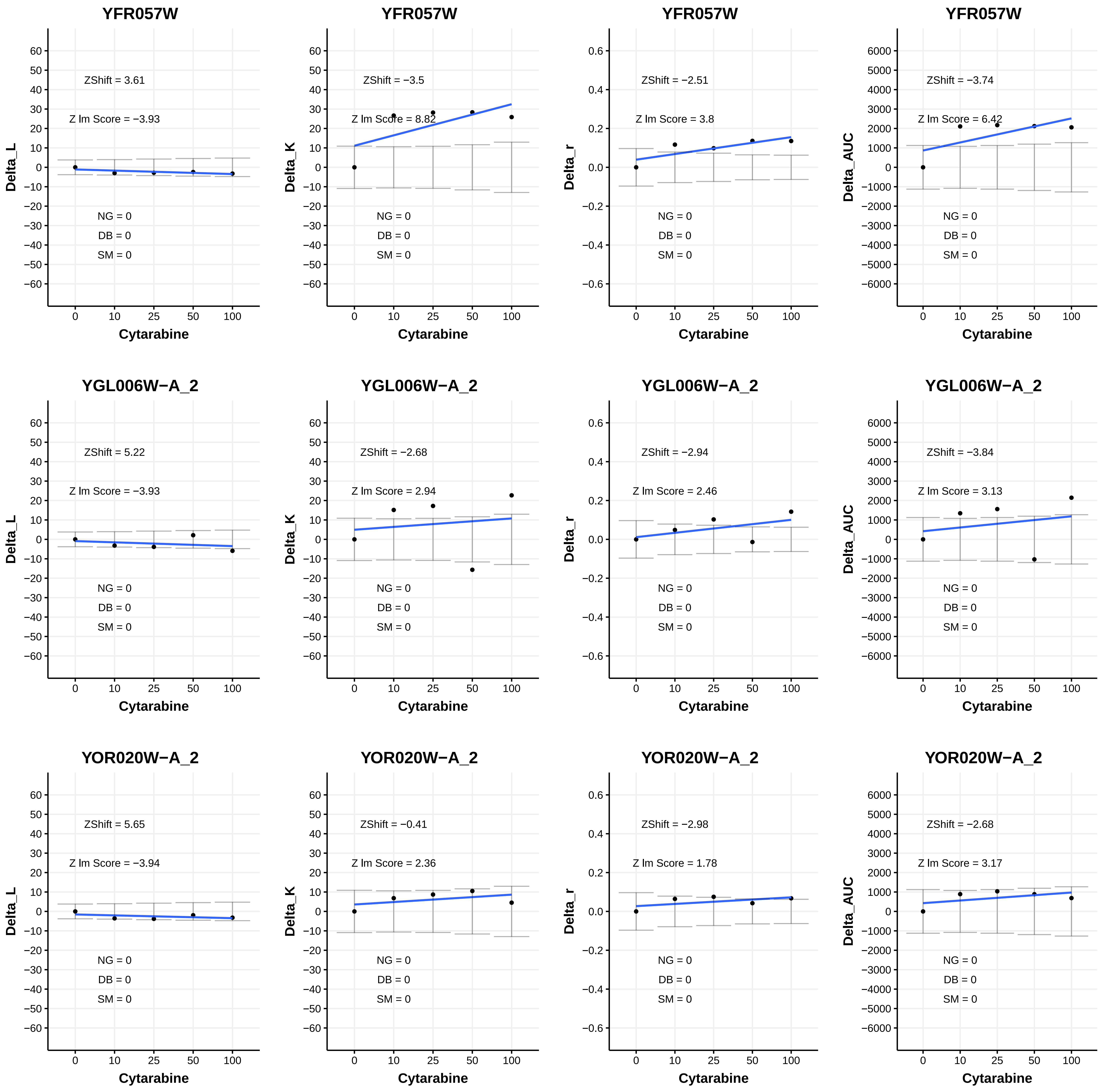
YFR057W
ZShift = 3.61
Z lm Score = −3.93
NG = 0
DB = 0
SM = 0
Delta_L
Cytarabine
YFR057W
ZShift = −3.5
Z lm Score = 8.82
NG = 0
DB = 0
SM = 0
Delta_K
Cytarabine
YFR057W
ZShift = −2.51
Z lm Score = 3.8
NG = 0
DB = 0
SM = 0
Delta_r
Cytarabine
YFR057W
ZShift = −3.74
Z lm Score = 6.42
NG = 0
DB = 0
SM = 0
Delta_AUC
Cytarabine
YGL006W−A_2
ZShift = 5.22
Z lm Score = −3.93
NG = 0
DB = 0
SM = 0
Delta_L
Cytarabine
YGL006W−A_2
ZShift = −2.68
Z lm Score = 2.94
NG = 0
DB = 0
SM = 0
Delta_K
Cytarabine
YGL006W−A_2
ZShift = −2.94
Z lm Score = 2.46
NG = 0
DB = 0
SM = 0
Delta_r
Cytarabine
YGL006W−A_2
ZShift = −3.84
Z lm Score = 3.13
NG = 0
DB = 0
SM = 0
Delta_AUC
Cytarabine
YOR020W−A_2
ZShift = 5.65
Z lm Score = −3.94
NG = 0
DB = 0
SM = 0
Delta_L
Cytarabine
YOR020W−A_2
ZShift = −0.41
Z lm Score = 2.36
NG = 0
DB = 0
SM = 0
Delta_K
Cytarabine
YOR020W−A_2
ZShift = −2.98
Z lm Score = 1.78
NG = 0
DB = 0
SM = 0
Delta_r
Cytarabine
YOR020W−A_2
ZShift = −2.68
Z lm Score = 3.17
NG = 0
DB = 0
SM = 0
Delta_AUC
Cytarabine

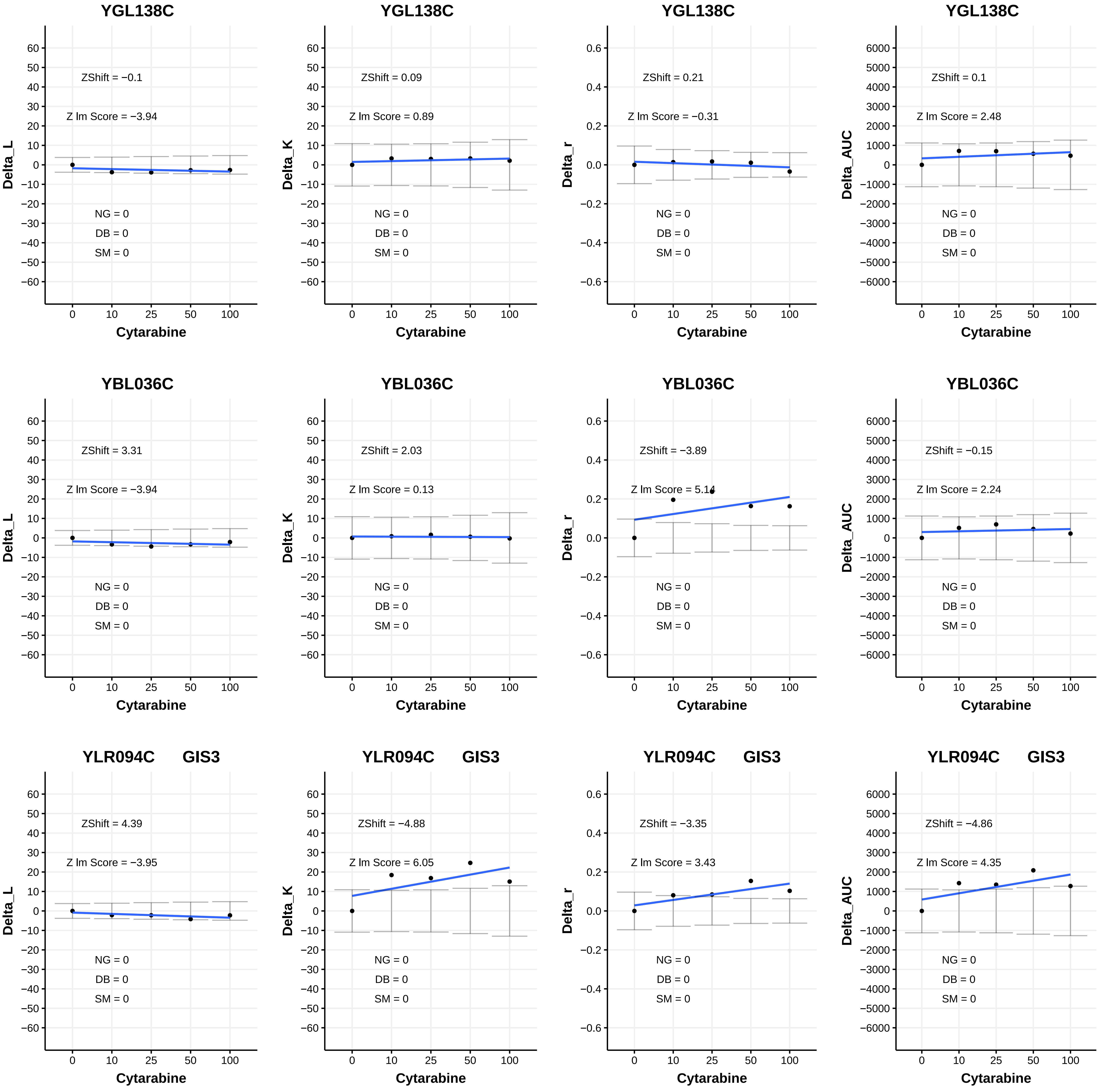
YGL138C
ZShift = −0.1
Z lm Score = −3.94
NG = 0
DB = 0
SM = 0
Delta_L
Cytarabine
YGL138C
ZShift = 0.09
Z lm Score = 0.89
NG = 0
DB = 0
SM = 0
Delta_K
Cytarabine
YGL138C
ZShift = 0.21
Z lm Score = −0.31
NG = 0
DB = 0
SM = 0
Delta_r
Cytarabine
YGL138C
ZShift = 0.1
Z lm Score = 2.48
NG = 0
DB = 0
SM = 0
Delta_AUC
Cytarabine
YBL036C
ZShift = 3.31
Z lm Score = −3.94
NG = 0
DB = 0
SM = 0
Delta_L
Cytarabine
YBL036C
ZShift = 2.03
Z lm Score = 0.13
NG = 0
DB = 0
SM = 0
Delta_K
Cytarabine
YBL036C
ZShift = −3.89
Z lm Score = 5.14
NG = 0
DB = 0
SM = 0
Delta_r
Cytarabine
YBL036C
ZShift = −0.15
Z lm Score = 2.24
NG = 0
DB = 0
SM = 0
Delta_AUC
Cytarabine
YLR094C GIS3
ZShift = 4.39
Z lm Score = −3.95
NG = 0
DB = 0
SM = 0
Delta_L
Cytarabine
YLR094C GIS3
ZShift = −4.88
Z lm Score = 6.05
NG = 0
DB = 0
SM = 0
Delta_K
Cytarabine
YLR094C GIS3
ZShift = −3.35
Z lm Score = 3.43
NG = 0
DB = 0
SM = 0
Delta_r
Cytarabine
YLR094C GIS3
ZShift = −4.86
Z lm Score = 4.35
NG = 0
DB = 0
SM = 0
Delta_AUC
Cytarabine

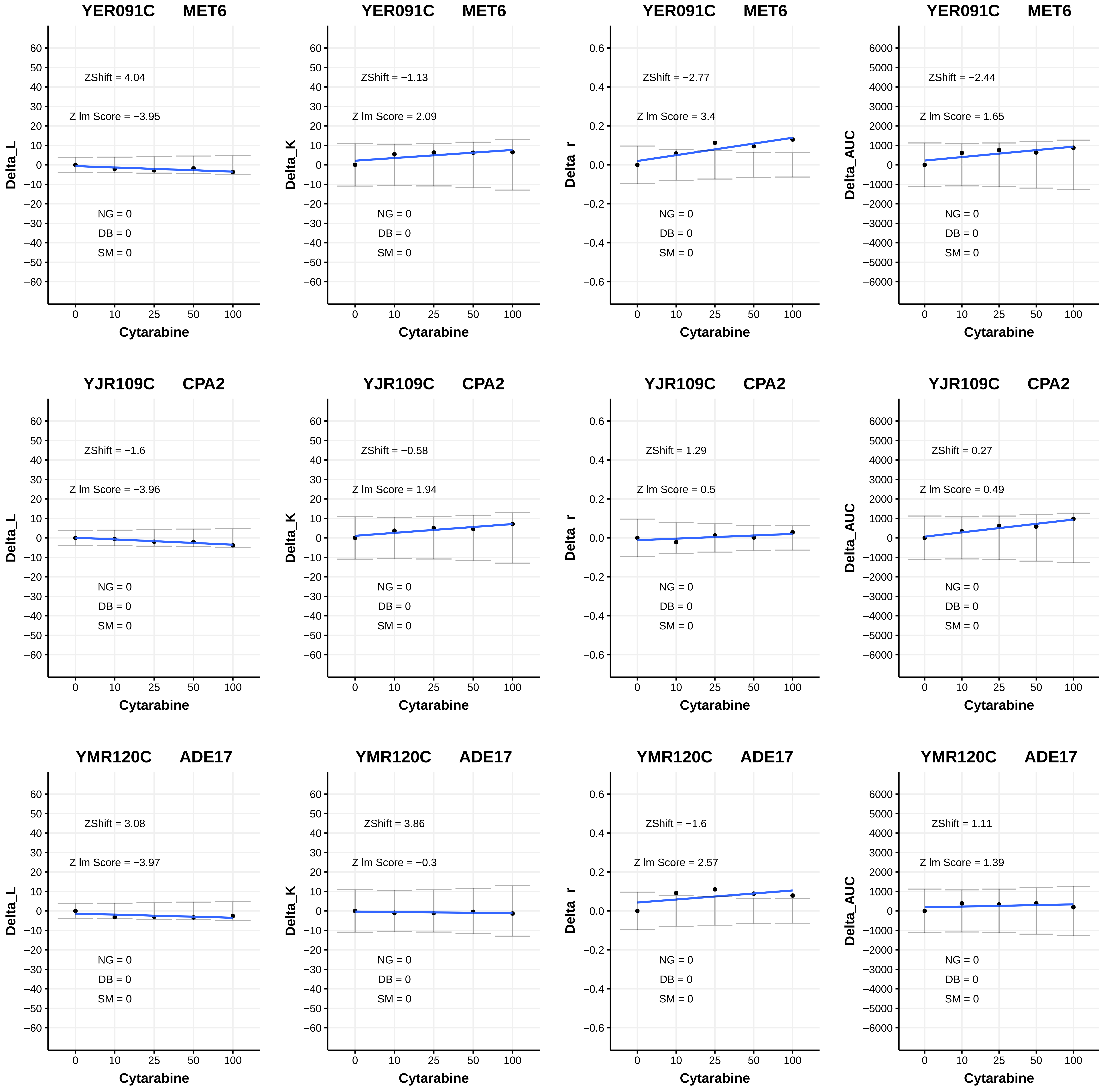

YER091C MET6
ZShift = 4.04
Z lm Score = −3.95
NG = 0
DB = 0
SM = 0
Delta_L
Cytarabine
YER091C MET6
ZShift = −1.13
Z lm Score = 2.09
NG = 0
DB = 0
SM = 0
Delta_K
Cytarabine
YER091C MET6
ZShift = −2.77
Z lm Score = 3.4
NG = 0
DB = 0
SM = 0
Delta_r
Cytarabine
YER091C MET6
ZShift = −2.44
Z lm Score = 1.65
NG = 0
DB = 0
SM = 0
Delta_AUC
Cytarabine
YJR109C CPA2
ZShift = −1.6
Z lm Score = −3.96
NG = 0
DB = 0
SM = 0
Delta_L
Cytarabine
YJR109C CPA2
ZShift = −0.58
Z lm Score = 1.94
NG = 0
DB = 0
SM = 0
Delta_K
Cytarabine
YJR109C CPA2
ZShift = 1.29
Z lm Score = 0.5
NG = 0
DB = 0
SM = 0
Delta_r
Cytarabine
YJR109C CPA2
ZShift = 0.27
Z lm Score = 0.49
NG = 0
DB = 0
SM = 0
Delta_AUC
Cytarabine
YMR120C ADE17
ZShift = 3.08
Z lm Score = −3.97
NG = 0
DB = 0
SM = 0
Delta_L
Cytarabine
YMR120C ADE17
ZShift = 3.86
Z lm Score = −0.3
NG = 0
DB = 0
SM = 0
Delta_K
Cytarabine
YMR120C ADE17
ZShift = −1.6
Z lm Score = 2.57
NG = 0
DB = 0
SM = 0
Delta_r
Cytarabine
YMR120C ADE17
ZShift = 1.11
Z lm Score = 1.39
NG = 0
DB = 0
SM = 0
Delta_AUC
Cytarabine

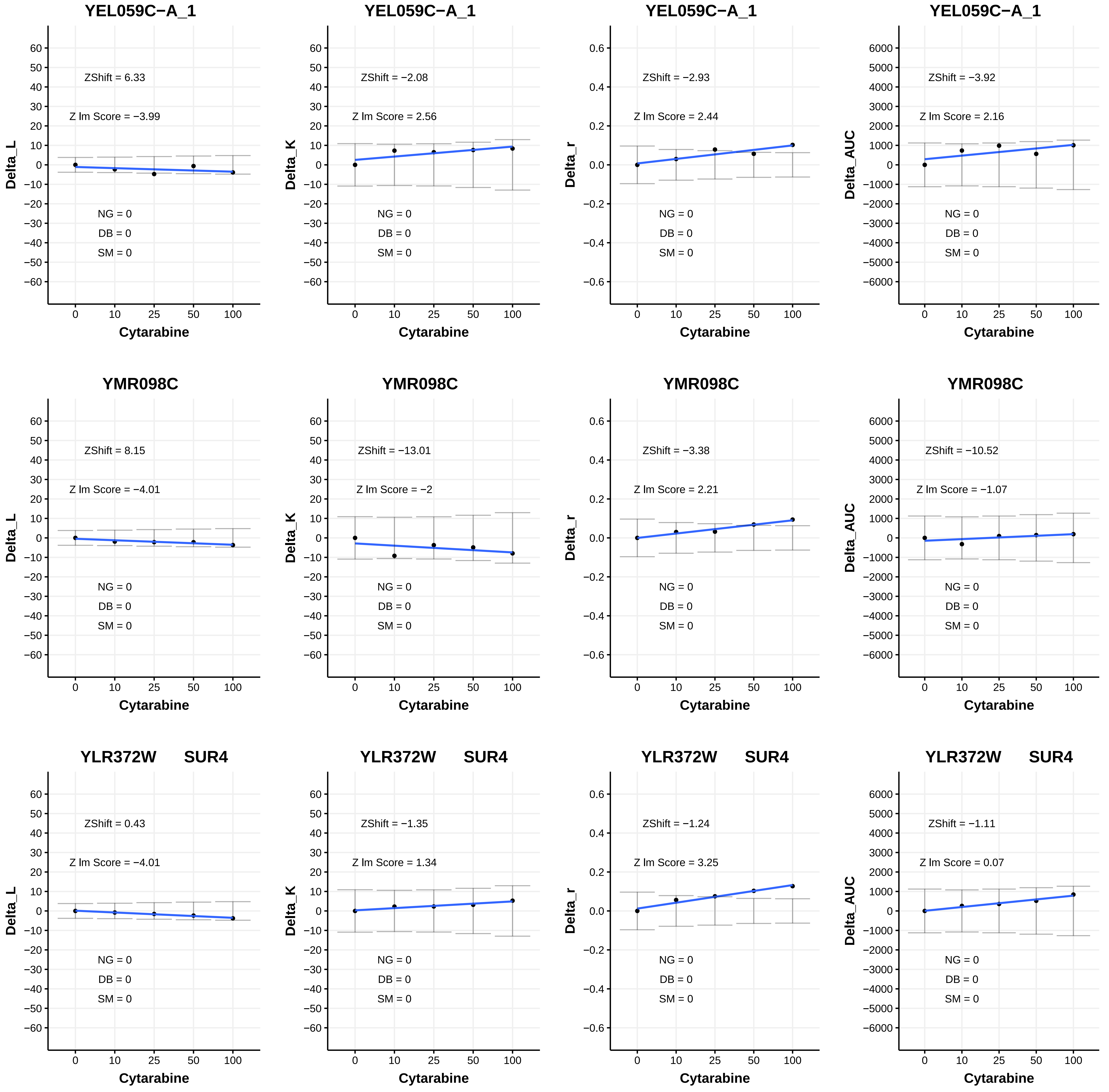
YEL059C−A_1
ZShift = 6.33
Z lm Score = −3.99
NG = 0
DB = 0
SM = 0
Delta_L
Cytarabine
YEL059C−A_1
ZShift = −2.08
Z lm Score = 2.56
NG = 0
DB = 0
SM = 0
Delta_K
Cytarabine
YEL059C−A_1
ZShift = −2.93
Z lm Score = 2.44
NG = 0
DB = 0
SM = 0
Delta_r
Cytarabine
YEL059C−A_1
ZShift = −3.92
Z lm Score = 2.16
NG = 0
DB = 0
SM = 0
Delta_AUC
Cytarabine
YMR098C
ZShift = 8.15
Z lm Score = −4.01
NG = 0
DB = 0
SM = 0
Delta_L
Cytarabine
YMR098C
ZShift = −13.01
Z lm Score = −2
NG = 0
DB = 0
SM = 0
Delta_K
Cytarabine
YMR098C
ZShift = −3.38
Z lm Score = 2.21
NG = 0
DB = 0
SM = 0
Delta_r
Cytarabine
YMR098C
ZShift = −10.52
Z lm Score = −1.07
NG = 0
DB = 0
SM = 0
Delta_AUC
Cytarabine
YLR372W SUR4
ZShift = 0.43
Z lm Score = −4.01
NG = 0
DB = 0
SM = 0
Delta_L
Cytarabine
YLR372W SUR4
ZShift = −1.35
Z lm Score = 1.34
NG = 0
DB = 0
SM = 0
Delta_K
Cytarabine
YLR372W SUR4
ZShift = −1.24
Z lm Score = 3.25
NG = 0
DB = 0
SM = 0
Delta_r
Cytarabine
YLR372W SUR4
ZShift = −1.11
Z lm Score = 0.07
NG = 0
DB = 0
SM = 0
Delta_AUC
Cytarabine

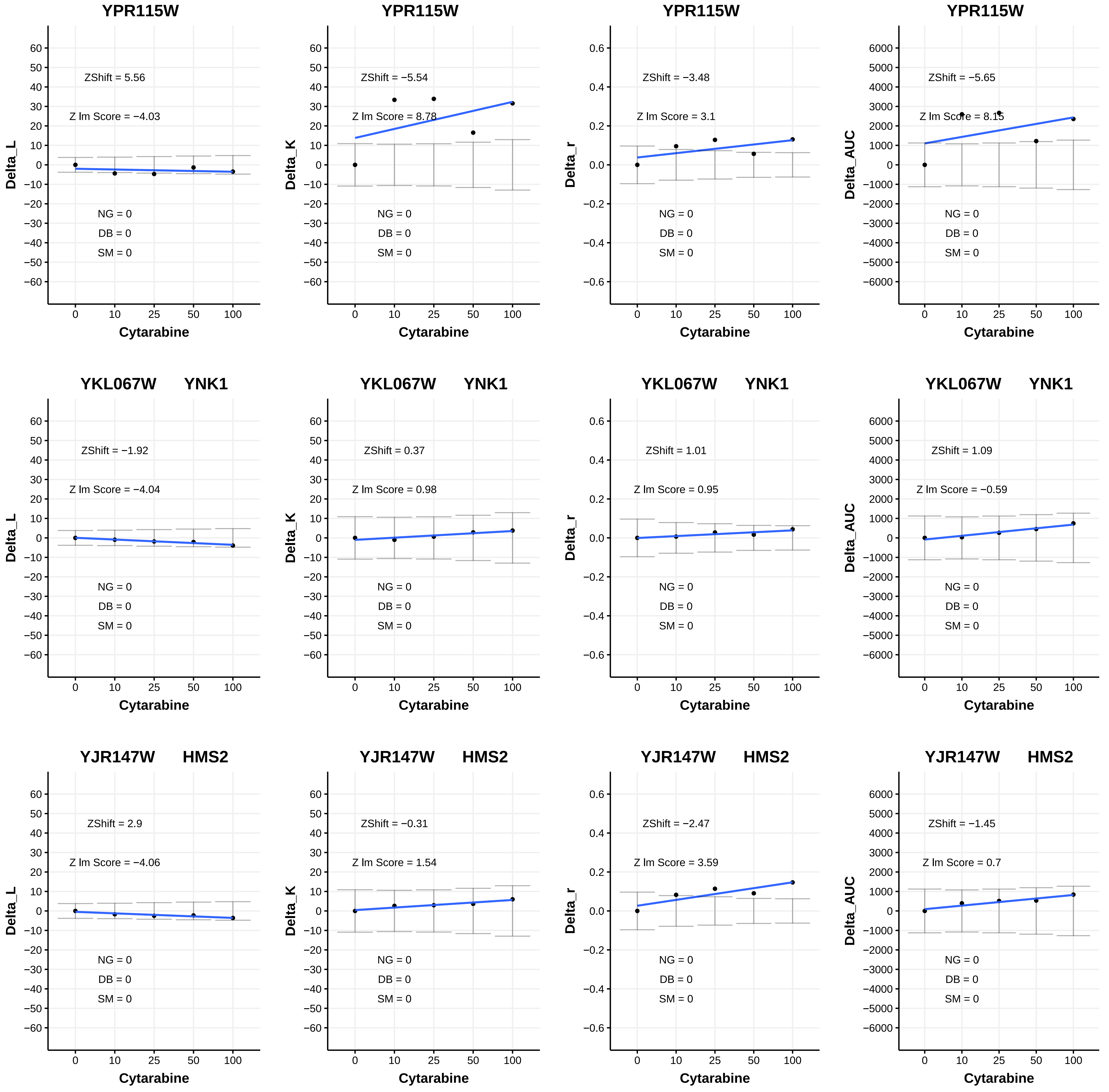
YPR115W
ZShift = 5.56
Z lm Score = −4.03
NG = 0
DB = 0
SM = 0
Delta_L
Cytarabine
YPR115W
ZShift = −5.54
Z lm Score = 8.78
NG = 0
DB = 0
SM = 0
Delta_K
Cytarabine
YPR115W
ZShift = −3.48
Z lm Score = 3.1
NG = 0
DB = 0
SM = 0
Delta_r
Cytarabine
YPR115W
ZShift = −5.65
Z lm Score = 8.15
NG = 0
DB = 0
SM = 0
Delta_AUC
Cytarabine
YKL067W YNK1
ZShift = −1.92
Z lm Score = −4.04
NG = 0
DB = 0
SM = 0
Delta_L
Cytarabine
YKL067W YNK1
ZShift = 0.37
Z lm Score = 0.98
NG = 0
DB = 0
SM = 0
Delta_K
Cytarabine
YKL067W YNK1
ZShift = 1.01
Z lm Score = 0.95
NG = 0
DB = 0
SM = 0
Delta_r
Cytarabine
YKL067W YNK1
ZShift = 1.09
Z lm Score = −0.59
NG = 0
DB = 0
SM = 0
Delta_AUC
Cytarabine
YJR147W HMS2
ZShift = 2.9
Z lm Score = −4.06
NG = 0
DB = 0
SM = 0
Delta_L
Cytarabine
YJR147W HMS2
ZShift = −0.31
Z lm Score = 1.54
NG = 0
DB = 0
SM = 0
Delta_K
Cytarabine
YJR147W HMS2
ZShift = −2.47
Z lm Score = 3.59
NG = 0
DB = 0
SM = 0
Delta_r
Cytarabine
YJR147W HMS2
ZShift = −1.45
Z lm Score = 0.7
NG = 0
DB = 0
SM = 0
Delta_AUC
Cytarabine

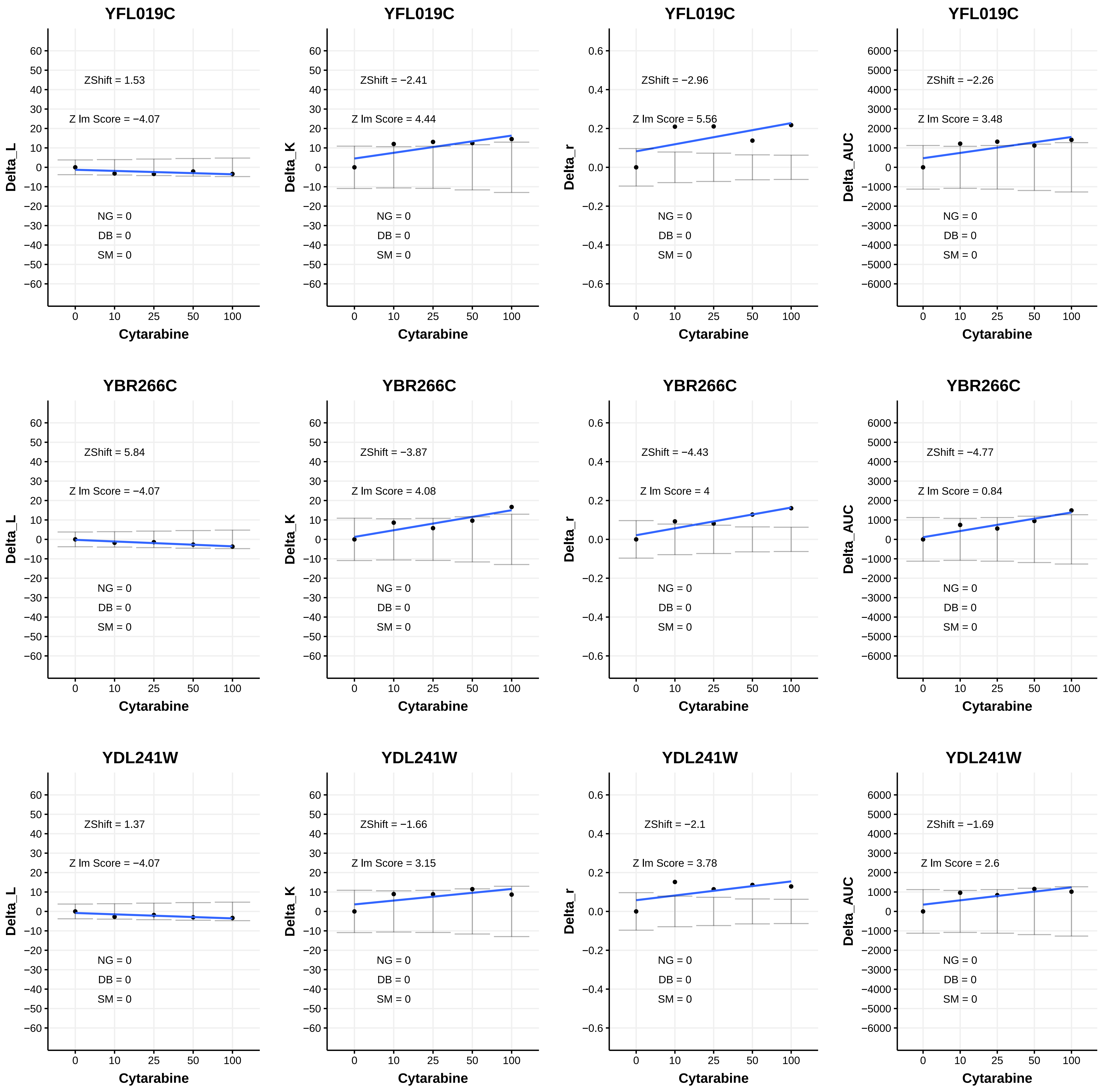

YFL019C
ZShift = 1.53
Z lm Score = −4.07
NG = 0
DB = 0
SM = 0
Delta_L
Cytarabine
YFL019C
ZShift = −2.41
Z lm Score = 4.44
NG = 0
DB = 0
SM = 0
Delta_K
Cytarabine
YFL019C
ZShift = −2.96
Z lm Score = 5.56
NG = 0
DB = 0
SM = 0
Delta_r
Cytarabine
YFL019C
ZShift = −2.26
Z lm Score = 3.48
NG = 0
DB = 0
SM = 0
Delta_AUC
Cytarabine
YBR266C
ZShift = 5.84
Z lm Score = −4.07
NG = 0
DB = 0
SM = 0
Delta_L
Cytarabine
YBR266C
ZShift = −3.87
Z lm Score = 4.08
NG = 0
DB = 0
SM = 0
Delta_K
Cytarabine
YBR266C
ZShift = −4.43
Z lm Score = 4
NG = 0
DB = 0
SM = 0
Delta_r
Cytarabine
YBR266C
ZShift = −4.77
Z lm Score = 0.84
NG = 0
DB = 0
SM = 0
Delta_AUC
Cytarabine
YDL241W
ZShift = 1.37
Z lm Score = −4.07
NG = 0
DB = 0
SM = 0
Delta_L
Cytarabine
YDL241W
ZShift = −1.66
Z lm Score = 3.15
NG = 0
DB = 0
SM = 0
Delta_K
Cytarabine
YDL241W
ZShift = −2.1
Z lm Score = 3.78
NG = 0
DB = 0
SM = 0
Delta_r
Cytarabine
YDL241W
ZShift = −1.69
Z lm Score = 2.6
NG = 0
DB = 0
SM = 0
Delta_AUC
Cytarabine

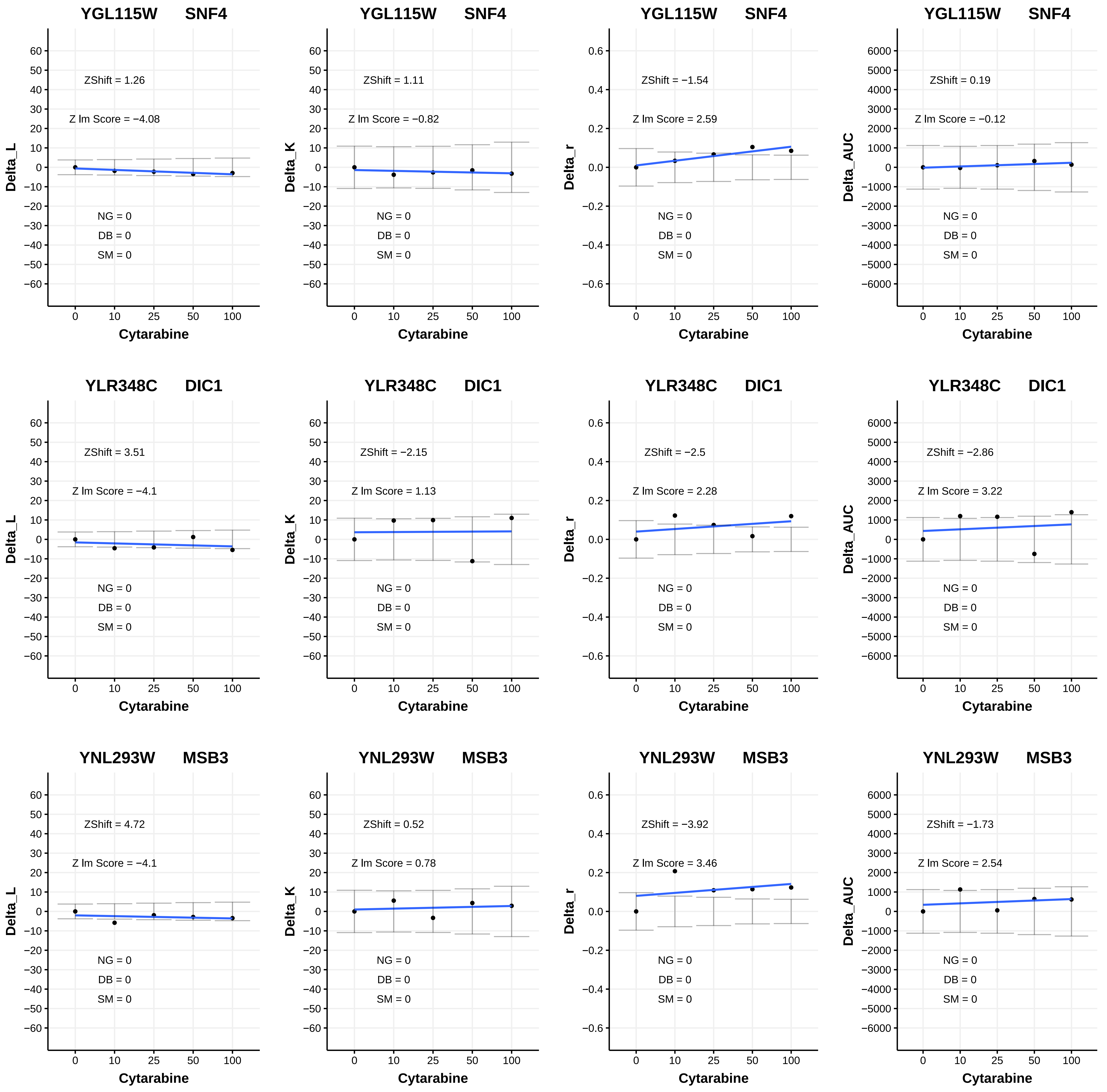
YGL115W SNF4
ZShift = 1.26
Z lm Score = −4.08
NG = 0
DB = 0
SM = 0
Delta_L
Cytarabine
YGL115W SNF4
ZShift = 1.11
Z lm Score = −0.82
NG = 0
DB = 0
SM = 0
Delta_K
Cytarabine
YGL115W SNF4
ZShift = −1.54
Z lm Score = 2.59
NG = 0
DB = 0
SM = 0
Delta_r
Cytarabine
YGL115W SNF4
ZShift = 0.19
Z lm Score = −0.12
NG = 0
DB = 0
SM = 0
Delta_AUC
Cytarabine
YLR348C DIC1
ZShift = 3.51
Z lm Score = −4.1
NG = 0
DB = 0
SM = 0
Delta_L
Cytarabine
YLR348C DIC1
ZShift = −2.15
Z lm Score = 1.13
NG = 0
DB = 0
SM = 0
Delta_K
Cytarabine
YLR348C DIC1
ZShift = −2.5
Z lm Score = 2.28
NG = 0
DB = 0
SM = 0
Delta_r
Cytarabine
YLR348C DIC1
ZShift = −2.86
Z lm Score = 3.22
NG = 0
DB = 0
SM = 0
Delta_AUC
Cytarabine
YNL293W MSB3
ZShift = 4.72
Z lm Score = −4.1
NG = 0
DB = 0
SM = 0
Delta_L
Cytarabine
YNL293W MSB3
ZShift = 0.52
Z lm Score = 0.78
NG = 0
DB = 0
SM = 0
Delta_K
Cytarabine
YNL293W MSB3
ZShift = −3.92
Z lm Score = 3.46
NG = 0
DB = 0
SM = 0
Delta_r
Cytarabine
YNL293W MSB3
ZShift = −1.73
Z lm Score = 2.54
NG = 0
DB = 0
SM = 0
Delta_AUC
Cytarabine

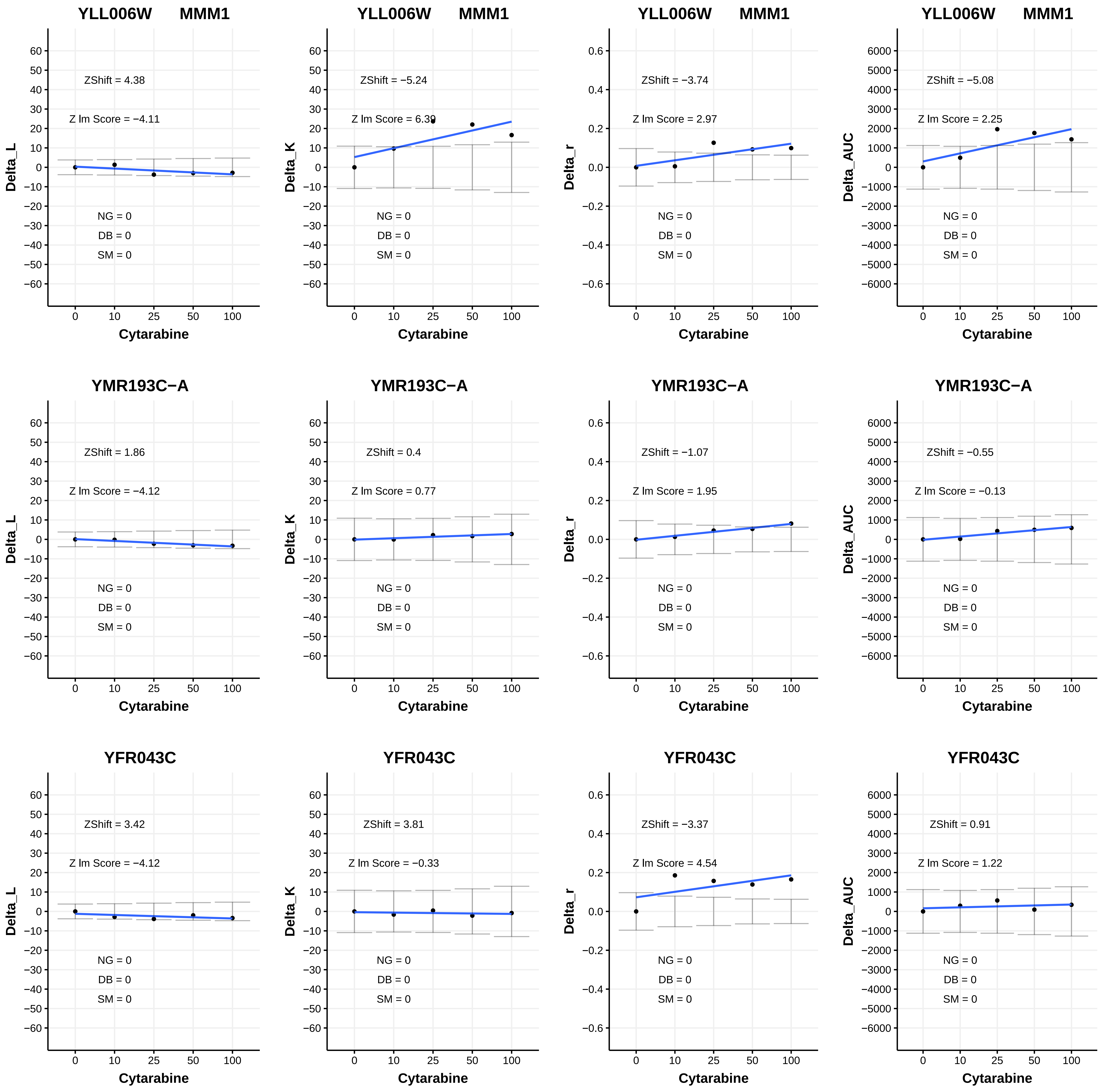

YLL006W MMM1
ZShift = 4.38
Z lm Score = −4.11
NG = 0
DB = 0
SM = 0
Delta_L
Cytarabine
YLL006W MMM1
ZShift = −5.24
Z lm Score = 6.39
NG = 0
DB = 0
SM = 0
Delta_K
Cytarabine
YLL006W MMM1
ZShift = −3.74
Z lm Score = 2.97
NG = 0
DB = 0
SM = 0
Delta_r
Cytarabine
YLL006W MMM1
ZShift = −5.08
Z lm Score = 2.25
NG = 0
DB = 0
SM = 0
Delta_AUC
Cytarabine
YMR193C−A
ZShift = 1.86
Z lm Score = −4.12
NG = 0
DB = 0
SM = 0
Delta_L
Cytarabine
YMR193C−A
ZShift = 0.4
Z lm Score = 0.77
NG = 0
DB = 0
SM = 0
Delta_K
Cytarabine
YMR193C−A
ZShift = −1.07
Z lm Score = 1.95
NG = 0
DB = 0
SM = 0
Delta_r
Cytarabine
YMR193C−A
ZShift = −0.55
Z lm Score = −0.13
NG = 0
DB = 0
SM = 0
Delta_AUC
Cytarabine
YFR043C
ZShift = 3.42
Z lm Score = −4.12
NG = 0
DB = 0
SM = 0
Delta_L
Cytarabine
YFR043C
ZShift = 3.81
Z lm Score = −0.33
NG = 0
DB = 0
SM = 0
Delta_K
Cytarabine
YFR043C
ZShift = −3.37
Z lm Score = 4.54
NG = 0
DB = 0
SM = 0
Delta_r
Cytarabine
YFR043C
ZShift = 0.91
Z lm Score = 1.22
NG = 0
DB = 0
SM = 0
Delta_AUC
Cytarabine

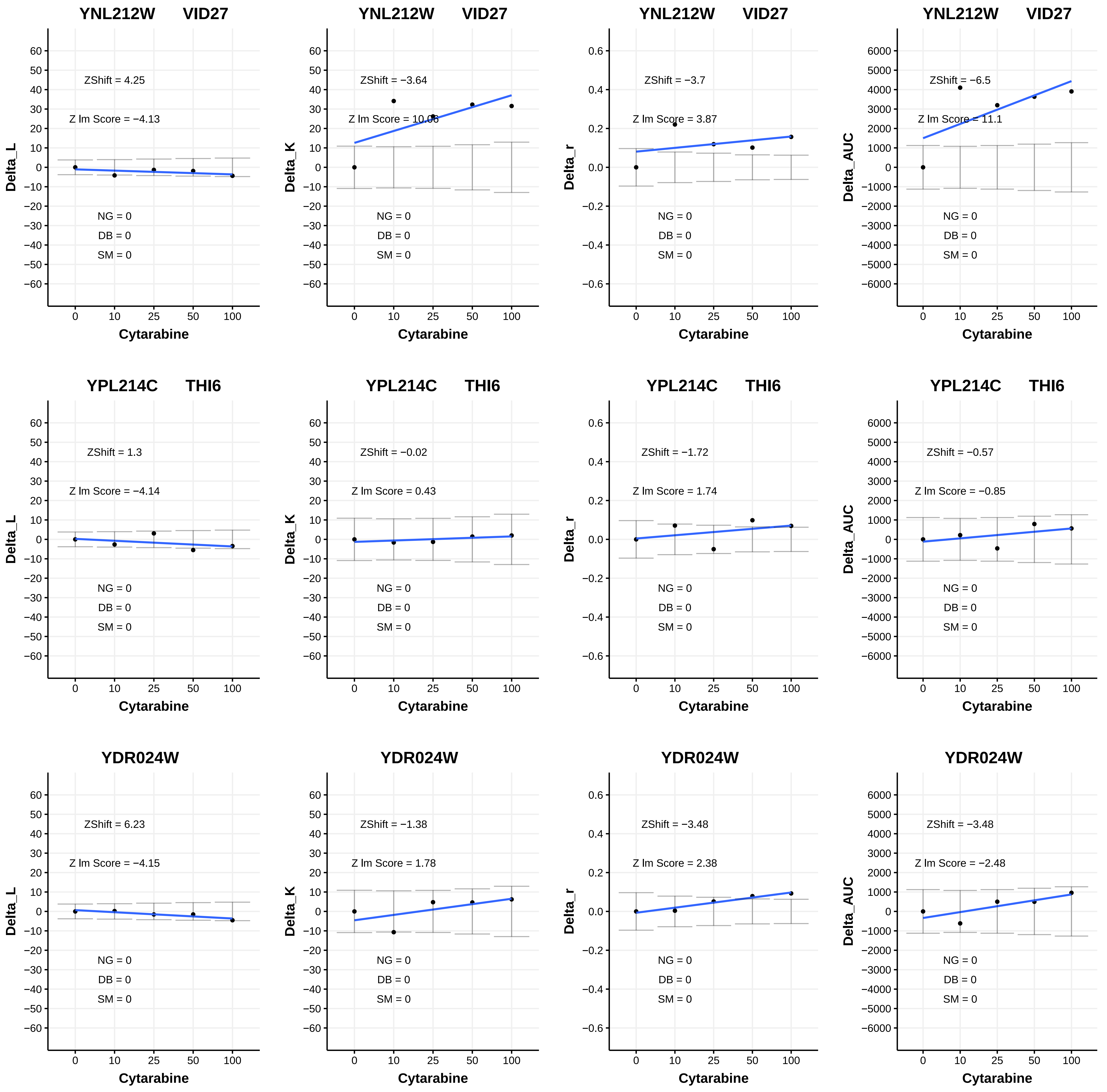
YNL212W VID27
ZShift = 4.25
Z lm Score = −4.13
NG = 0
DB = 0
SM = 0
Delta_L
Cytarabine
YNL212W VID27
ZShift = −3.64
Z lm Score = 10.06
NG = 0
DB = 0
SM = 0
Delta_K
Cytarabine
YNL212W VID27
ZShift = −3.7
Z lm Score = 3.87
NG = 0
DB = 0
SM = 0
Delta_r
Cytarabine
YNL212W VID27
ZShift = −6.5
Z lm Score = 11.1
NG = 0
DB = 0
SM = 0
Delta_AUC
Cytarabine
YPL214C THI6
ZShift = 1.3
Z lm Score = −4.14
NG = 0
DB = 0
SM = 0
Delta_L
Cytarabine
YPL214C THI6
ZShift = −0.02
Z lm Score = 0.43
NG = 0
DB = 0
SM = 0
Delta_K
Cytarabine
YPL214C THI6
ZShift = −1.72
Z lm Score = 1.74
NG = 0
DB = 0
SM = 0
Delta_r
Cytarabine
YPL214C THI6
ZShift = −0.57
Z lm Score = −0.85
NG = 0
DB = 0
SM = 0
Delta_AUC
Cytarabine
YDR024W
ZShift = 6.23
Z lm Score = −4.15
NG = 0
DB = 0
SM = 0
Delta_L
Cytarabine
YDR024W
ZShift = −1.38
Z lm Score = 1.78
NG = 0
DB = 0
SM = 0
Delta_K
Cytarabine
YDR024W
ZShift = −3.48
Z lm Score = 2.38
NG = 0
DB = 0
SM = 0
Delta_r
Cytarabine
YDR024W
ZShift = −3.48
Z lm Score = −2.48
NG = 0
DB = 0
SM = 0
Delta_AUC
Cytarabine

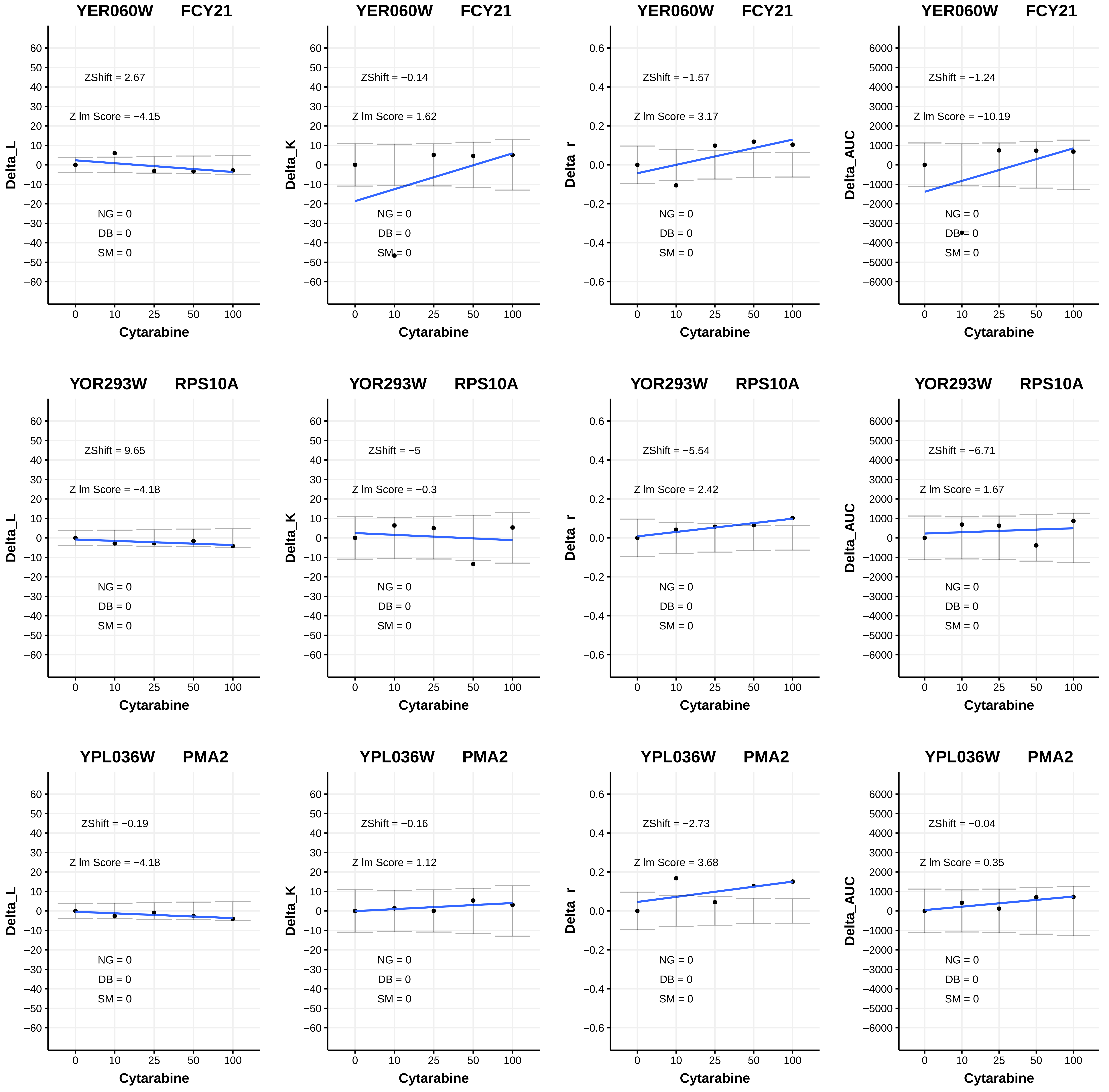

YER060W FCY21
ZShift = 2.67
Z lm Score = −4.15
NG = 0
DB = 0
SM = 0
Delta_L
Cytarabine
YER060W FCY21
ZShift = −0.14
Z lm Score = 1.62
NG = 0
DB = 0
SM = 0
Delta_K
Cytarabine
YER060W FCY21
ZShift = −1.57
Z lm Score = 3.17
NG = 0
DB = 0
SM = 0
Delta_r
Cytarabine
YER060W FCY21
ZShift = −1.24
Z lm Score = −10.19
NG = 0
DB = 0
SM = 0
Delta_AUC
Cytarabine
YOR293W RPS10A
ZShift = 9.65
Z lm Score = −4.18
NG = 0
DB = 0
SM = 0
Delta_L
Cytarabine
YOR293W RPS10A
ZShift = −5
Z lm Score = −0.3
NG = 0
DB = 0
SM = 0
Delta_K
Cytarabine
YOR293W RPS10A
ZShift = −5.54
Z lm Score = 2.42
NG = 0
DB = 0
SM = 0
Delta_r
Cytarabine
YOR293W RPS10A
ZShift = −6.71
Z lm Score = 1.67
NG = 0
DB = 0
SM = 0
Delta_AUC
Cytarabine
YPL036W PMA2
ZShift = −0.19
Z lm Score = −4.18
NG = 0
DB = 0
SM = 0
Delta_L
Cytarabine
YPL036W PMA2
ZShift = −0.16
Z lm Score = 1.12
NG = 0
DB = 0
SM = 0
Delta_K
Cytarabine
YPL036W PMA2
ZShift = −2.73
Z lm Score = 3.68
NG = 0
DB = 0
SM = 0
Delta_r
Cytarabine
YPL036W PMA2
ZShift = −0.04
Z lm Score = 0.35
NG = 0
DB = 0
SM = 0
Delta_AUC
Cytarabine

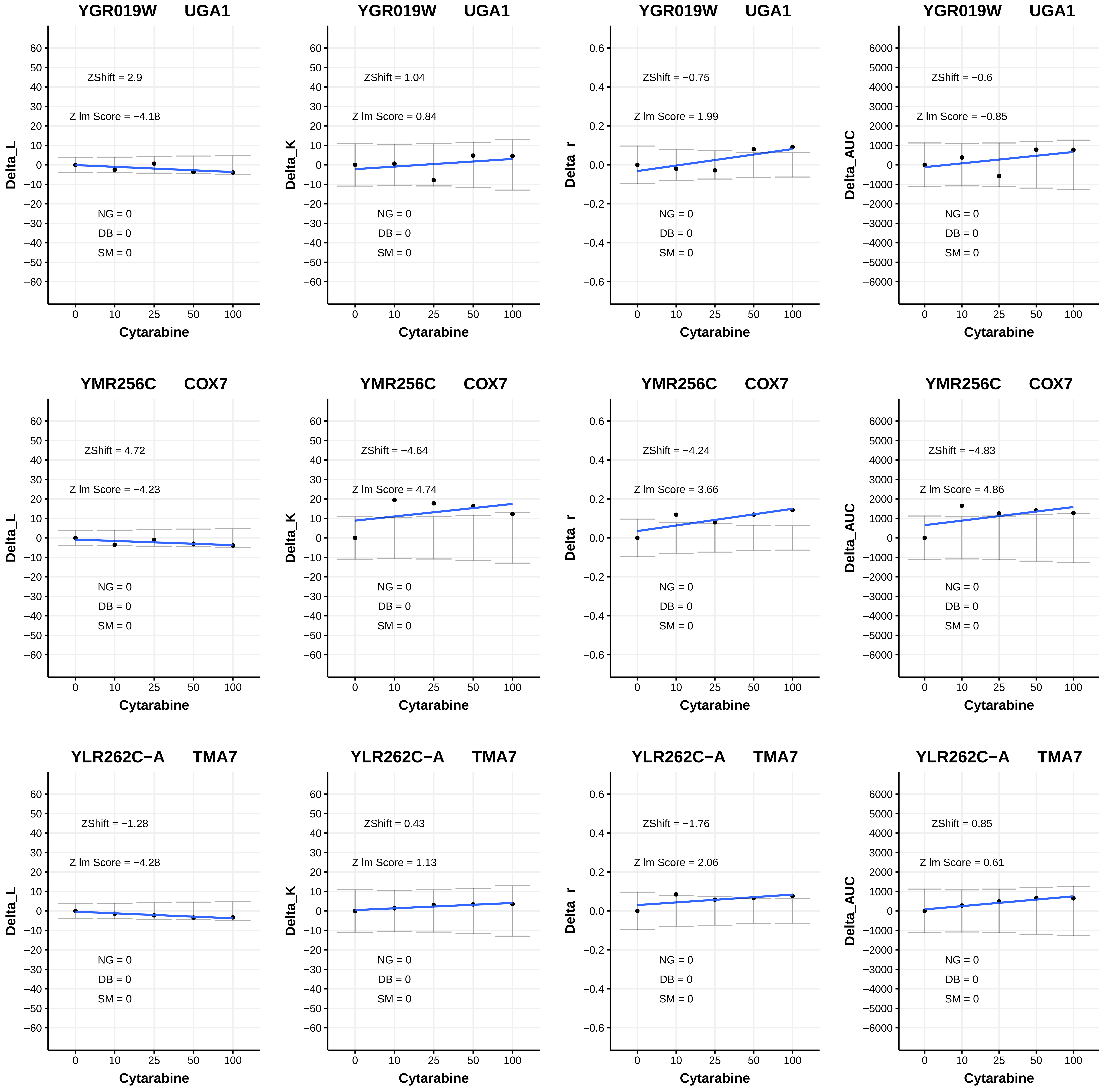
YGR019W UGA1
ZShift = 2.9
Z lm Score = −4.18
NG = 0
DB = 0
SM = 0
Delta_L
Cytarabine
YGR019W UGA1
ZShift = 1.04
Z lm Score = 0.84
NG = 0
DB = 0
SM = 0
Delta_K
Cytarabine
YGR019W UGA1
ZShift = −0.75
Z lm Score = 1.99
NG = 0
DB = 0
SM = 0
Delta_r
Cytarabine
YGR019W UGA1
ZShift = −0.6
Z lm Score = −0.85
NG = 0
DB = 0
SM = 0
Delta_AUC
Cytarabine
YMR256C COX7
ZShift = 4.72
Z lm Score = −4.23
NG = 0
DB = 0
SM = 0
Delta_L
Cytarabine
YMR256C COX7
ZShift = −4.64
Z lm Score = 4.74
NG = 0
DB = 0
SM = 0
Delta_K
Cytarabine
YMR256C COX7
ZShift = −4.24
Z lm Score = 3.66
NG = 0
DB = 0
SM = 0
Delta_r
Cytarabine
YMR256C COX7
ZShift = −4.83
Z lm Score = 4.86
NG = 0
DB = 0
SM = 0
Delta_AUC
Cytarabine
YLR262C−A TMA7
ZShift = −1.28
Z lm Score = −4.28
NG = 0
DB = 0
SM = 0
Delta_L
Cytarabine
YLR262C−A TMA7
ZShift = 0.43
Z lm Score = 1.13
NG = 0
DB = 0
SM = 0
Delta_K
Cytarabine
YLR262C−A TMA7
ZShift = −1.76
Z lm Score = 2.06
NG = 0
DB = 0
SM = 0
Delta_r
Cytarabine
YLR262C−A TMA7
ZShift = 0.85
Z lm Score = 0.61
NG = 0
DB = 0
SM = 0
Delta_AUC
Cytarabine

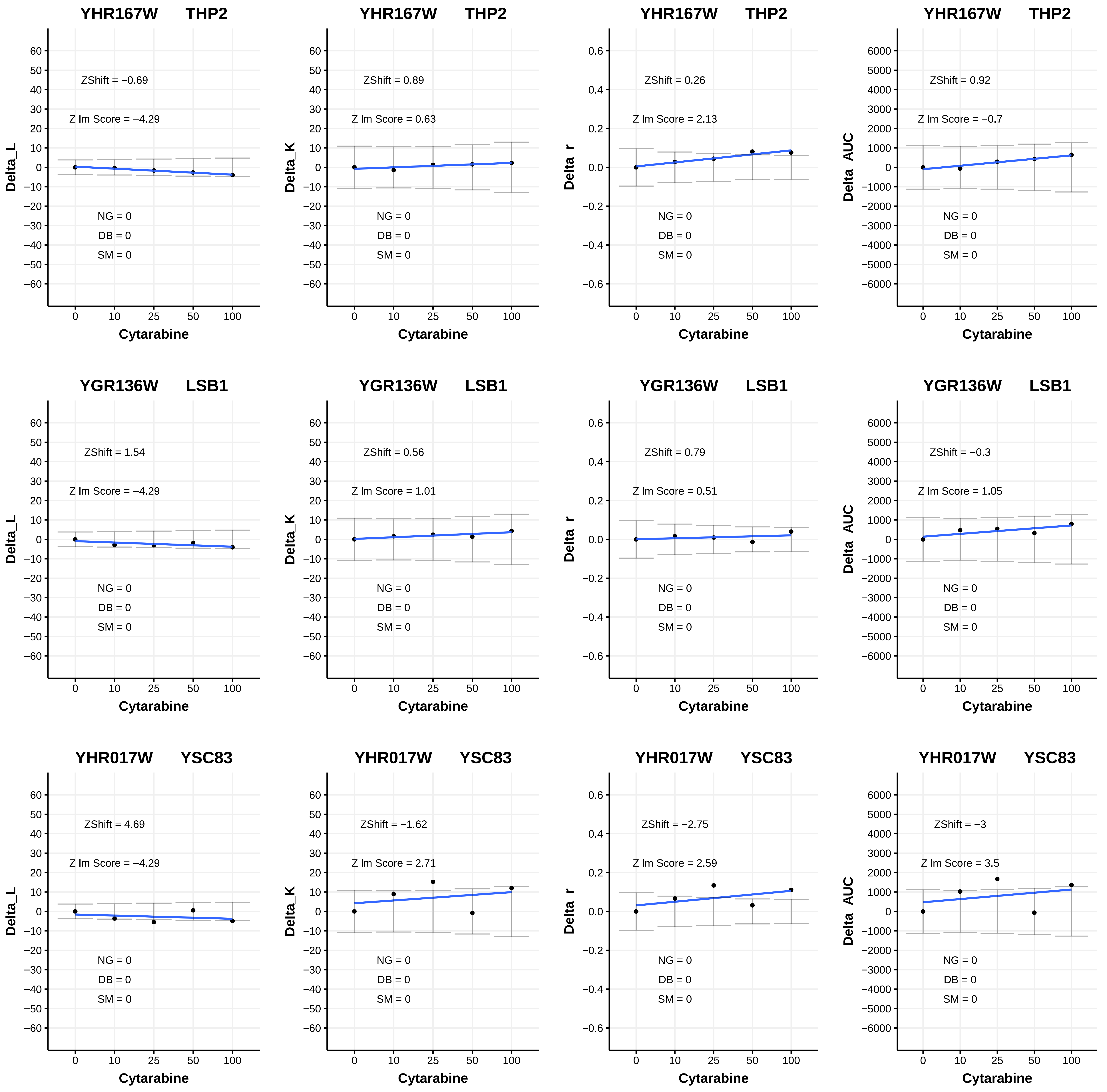

YHR167W THP2
ZShift = −0.69
Z lm Score = −4.29
NG = 0
DB = 0
SM = 0
Delta_L
Cytarabine
YHR167W THP2
ZShift = 0.89
Z lm Score = 0.63
NG = 0
DB = 0
SM = 0
Delta_K
Cytarabine
YHR167W THP2
ZShift = 0.26
Z lm Score = 2.13
NG = 0
DB = 0
SM = 0
Delta_r
Cytarabine
YHR167W THP2
ZShift = 0.92
Z lm Score = −0.7
NG = 0
DB = 0
SM = 0
Delta_AUC
Cytarabine
YGR136W LSB1
ZShift = 1.54
Z lm Score = −4.29
NG = 0
DB = 0
SM = 0
Delta_L
Cytarabine
YGR136W LSB1
ZShift = 0.56
Z lm Score = 1.01
NG = 0
DB = 0
SM = 0
Delta_K
Cytarabine
YGR136W LSB1
ZShift = 0.79
Z lm Score = 0.51
NG = 0
DB = 0
SM = 0
Delta_r
Cytarabine
YGR136W LSB1
ZShift = −0.3
Z lm Score = 1.05
NG = 0
DB = 0
SM = 0
Delta_AUC
Cytarabine
YHR017W YSC83
ZShift = 4.69
Z lm Score = −4.29
NG = 0
DB = 0
SM = 0
Delta_L
Cytarabine
YHR017W YSC83
ZShift = −1.62
Z lm Score = 2.71
NG = 0
DB = 0
SM = 0
Delta_K
Cytarabine
YHR017W YSC83
ZShift = −2.75
Z lm Score = 2.59
NG = 0
DB = 0
SM = 0
Delta_r
Cytarabine
YHR017W YSC83
ZShift = −3
Z lm Score = 3.5
NG = 0
DB = 0
SM = 0
Delta_AUC
Cytarabine

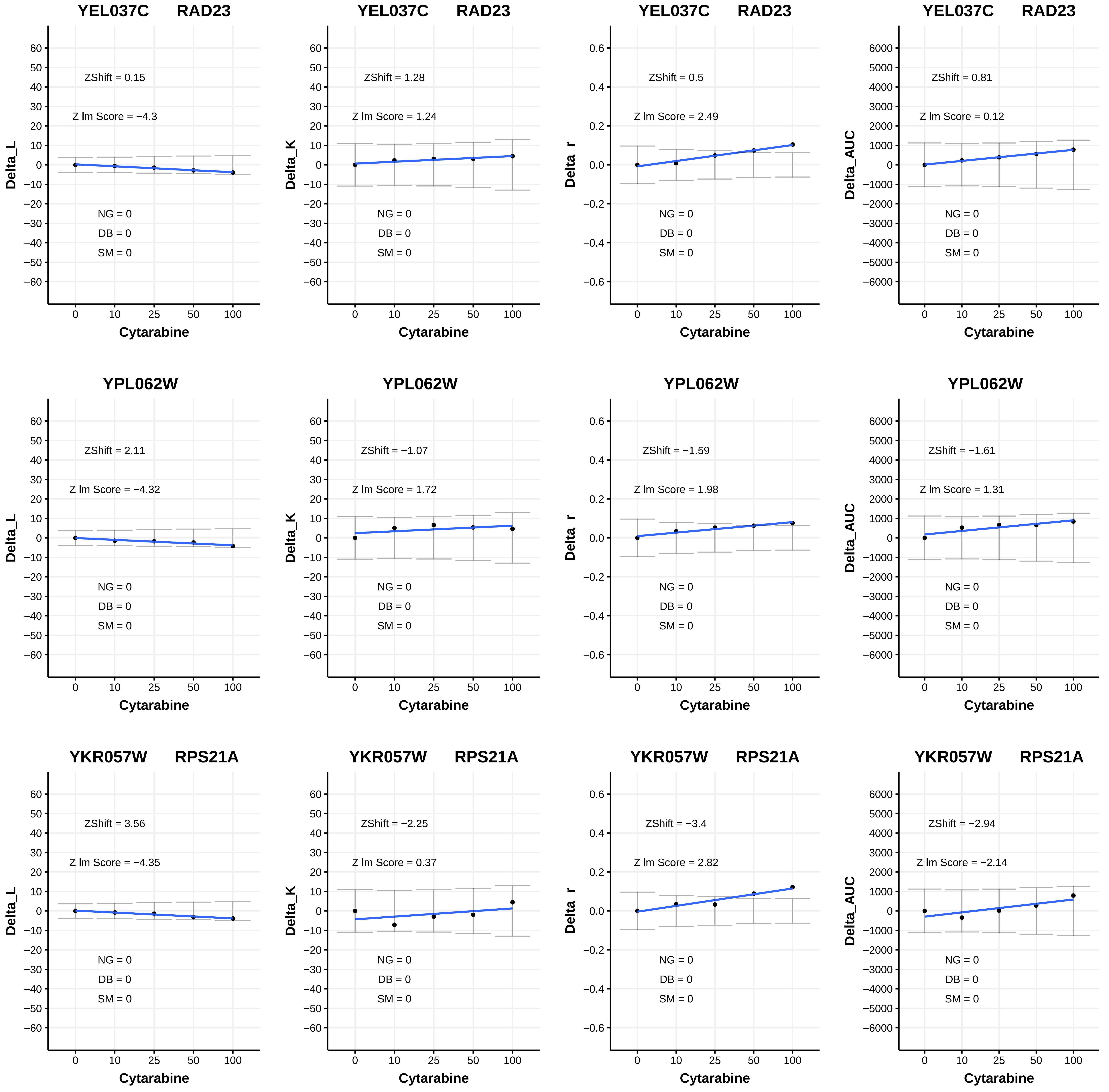

YEL037C RAD23
ZShift = 0.15
Z lm Score = −4.3
NG = 0
DB = 0
SM = 0
Delta_L
Cytarabine
YEL037C RAD23
ZShift = 1.28
Z lm Score = 1.24
NG = 0
DB = 0
SM = 0
Delta_K
Cytarabine
YEL037C RAD23
ZShift = 0.5
Z lm Score = 2.49
NG = 0
DB = 0
SM = 0
Delta_r
Cytarabine
YEL037C RAD23
ZShift = 0.81
Z lm Score = 0.12
NG = 0
DB = 0
SM = 0
Delta_AUC
Cytarabine
YPL062W
ZShift = 2.11
Z lm Score = −4.32
NG = 0
DB = 0
SM = 0
Delta_L
Cytarabine
YPL062W
ZShift = −1.07
Z lm Score = 1.72
NG = 0
DB = 0
SM = 0
Delta_K
Cytarabine
YPL062W
ZShift = −1.59
Z lm Score = 1.98
NG = 0
DB = 0
SM = 0
Delta_r
Cytarabine
YPL062W
ZShift = −1.61
Z lm Score = 1.31
NG = 0
DB = 0
SM = 0
Delta_AUC
Cytarabine
YKR057W RPS21A
ZShift = 3.56
Z lm Score = −4.35
NG = 0
DB = 0
SM = 0
Delta_L
Cytarabine
YKR057W RPS21A
ZShift = −2.25
Z lm Score = 0.37
NG = 0
DB = 0
SM = 0
Delta_K
Cytarabine
YKR057W RPS21A
ZShift = −3.4
Z lm Score = 2.82
NG = 0
DB = 0
SM = 0
Delta_r
Cytarabine
YKR057W RPS21A
ZShift = −2.94
Z lm Score = −2.14
NG = 0
DB = 0
SM = 0
Delta_AUC
Cytarabine

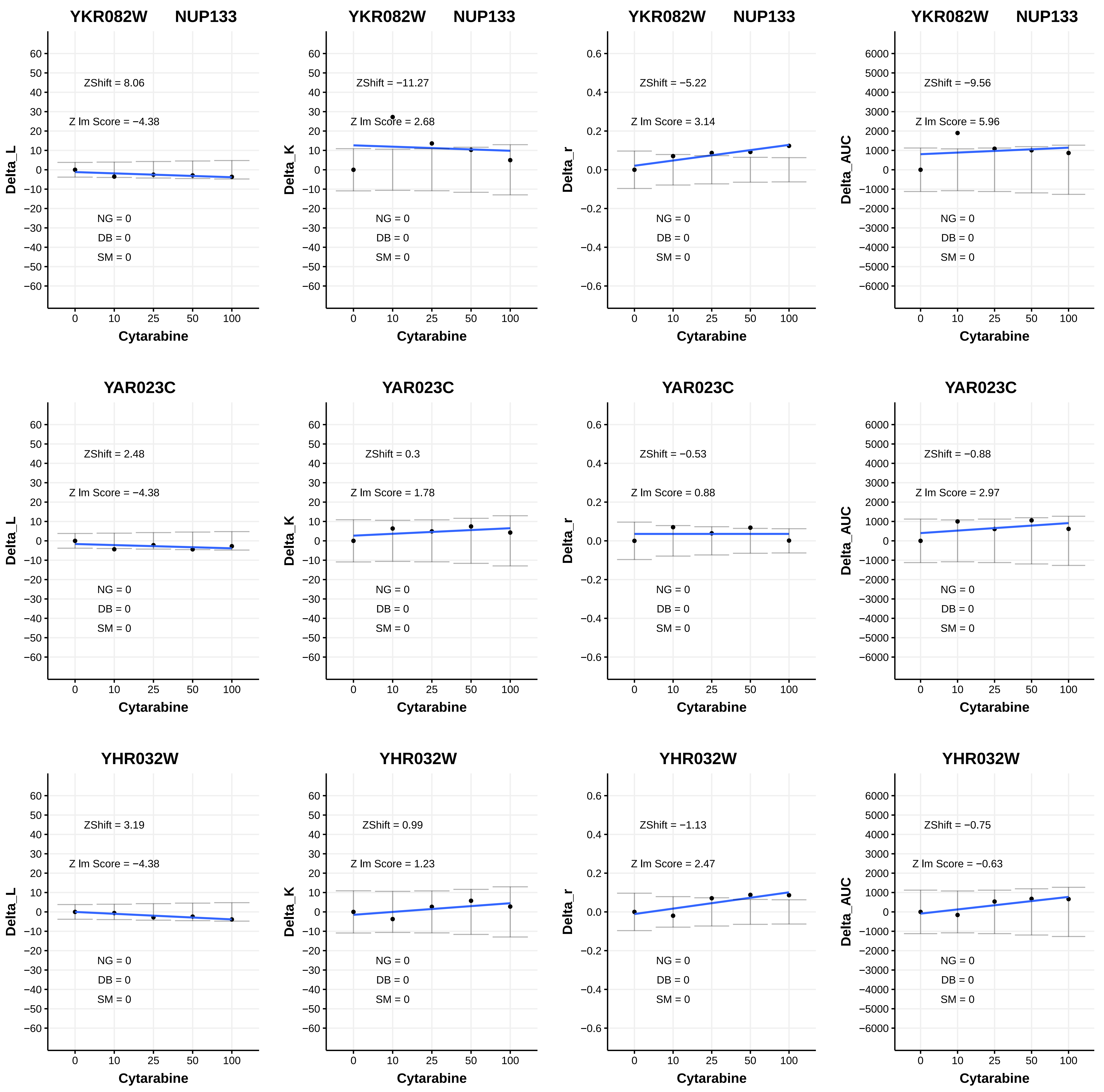

YKR082W NUP133
ZShift = 8.06
Z lm Score = −4.38
NG = 0
DB = 0
SM = 0
Delta_L
Cytarabine
YKR082W NUP133
ZShift = −11.27
Z lm Score = 2.68
NG = 0
DB = 0
SM = 0
Delta_K
Cytarabine
YKR082W NUP133
ZShift = −5.22
Z lm Score = 3.14
NG = 0
DB = 0
SM = 0
Delta_r
Cytarabine
YKR082W NUP133
ZShift = −9.56
Z lm Score = 5.96
NG = 0
DB = 0
SM = 0
Delta_AUC
Cytarabine
YAR023C
ZShift = 2.48
Z lm Score = −4.38
NG = 0
DB = 0
SM = 0
Delta_L
Cytarabine
YAR023C
ZShift = 0.3
Z lm Score = 1.78
NG = 0
DB = 0
SM = 0
Delta_K
Cytarabine
YAR023C
ZShift = −0.53
Z lm Score = 0.88
NG = 0
DB = 0
SM = 0
Delta_r
Cytarabine
YAR023C
ZShift = −0.88
Z lm Score = 2.97
NG = 0
DB = 0
SM = 0
Delta_AUC
Cytarabine
YHR032W
ZShift = 3.19
Z lm Score = −4.38
NG = 0
DB = 0
SM = 0
Delta_L
Cytarabine
YHR032W
ZShift = 0.99
Z lm Score = 1.23
NG = 0
DB = 0
SM = 0
Delta_K
Cytarabine
YHR032W
ZShift = −1.13
Z lm Score = 2.47
NG = 0
DB = 0
SM = 0
Delta_r
Cytarabine
YHR032W
ZShift = −0.75
Z lm Score = −0.63
NG = 0
DB = 0
SM = 0
Delta_AUC
Cytarabine

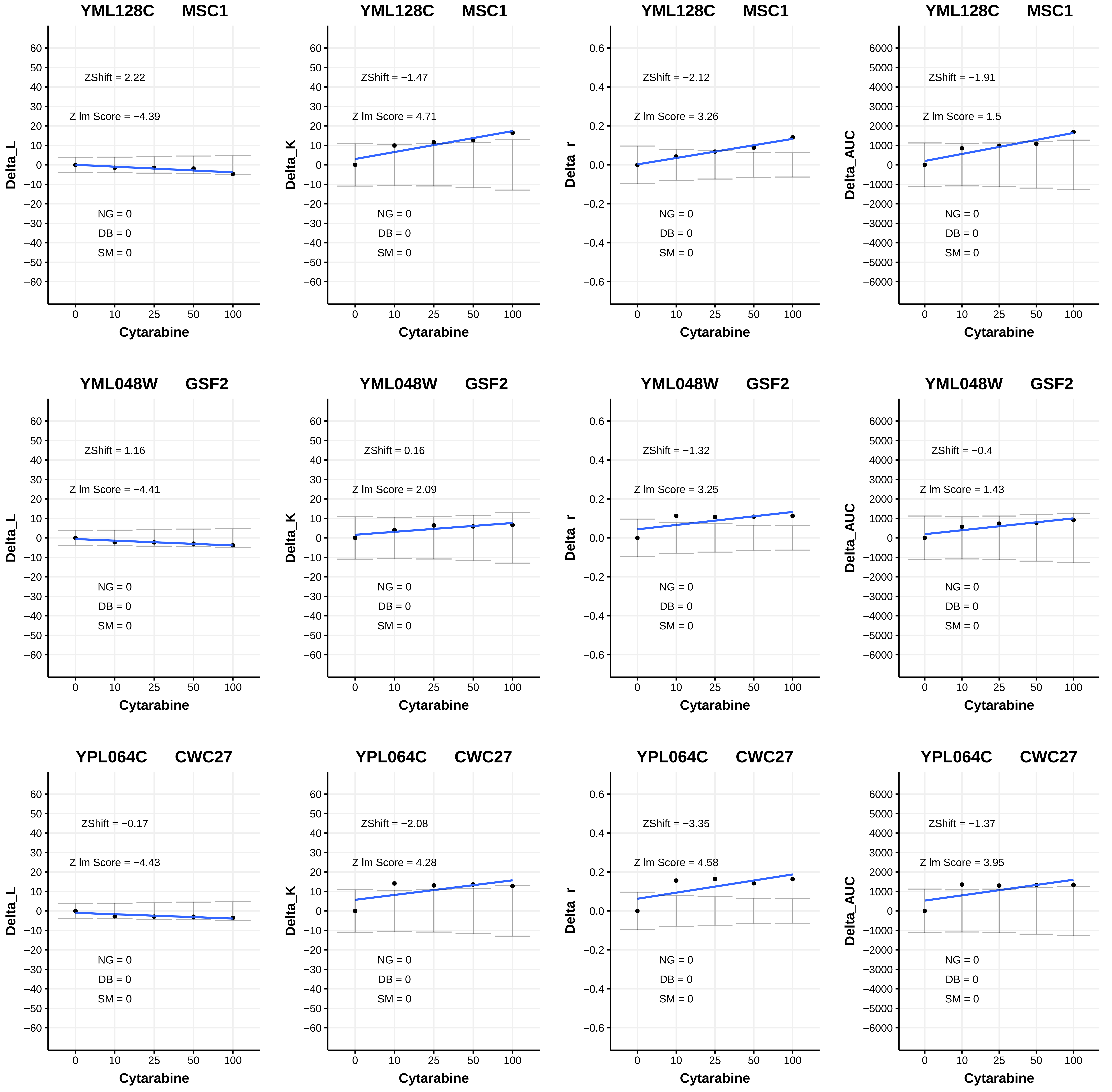
YML128C MSC1
ZShift = 2.22
Z lm Score = −4.39
NG = 0
DB = 0
SM = 0
Delta_L
Cytarabine
YML128C MSC1
ZShift = −1.47
Z lm Score = 4.71
NG = 0
DB = 0
SM = 0
Delta_K
Cytarabine
YML128C MSC1
ZShift = −2.12
Z lm Score = 3.26
NG = 0
DB = 0
SM = 0
Delta_r
Cytarabine
YML128C MSC1
ZShift = −1.91
Z lm Score = 1.5
NG = 0
DB = 0
SM = 0
Delta_AUC
Cytarabine
YML048W GSF2
ZShift = 1.16
Z lm Score = −4.41
NG = 0
DB = 0
SM = 0
Delta_L
Cytarabine
YML048W GSF2
ZShift = 0.16
Z lm Score = 2.09
NG = 0
DB = 0
SM = 0
Delta_K
Cytarabine
YML048W GSF2
ZShift = −1.32
Z lm Score = 3.25
NG = 0
DB = 0
SM = 0
Delta_r
Cytarabine
YML048W GSF2
ZShift = −0.4
Z lm Score = 1.43
NG = 0
DB = 0
SM = 0
Delta_AUC
Cytarabine
YPL064C CWC27
ZShift = −0.17
Z lm Score = −4.43
NG = 0
DB = 0
SM = 0
Delta_L
Cytarabine
YPL064C CWC27
ZShift = −2.08
Z lm Score = 4.28
NG = 0
DB = 0
SM = 0
Delta_K
Cytarabine
YPL064C CWC27
ZShift = −3.35
Z lm Score = 4.58
NG = 0
DB = 0
SM = 0
Delta_r
Cytarabine
YPL064C CWC27
ZShift = −1.37
Z lm Score = 3.95
NG = 0
DB = 0
SM = 0
Delta_AUC
Cytarabine

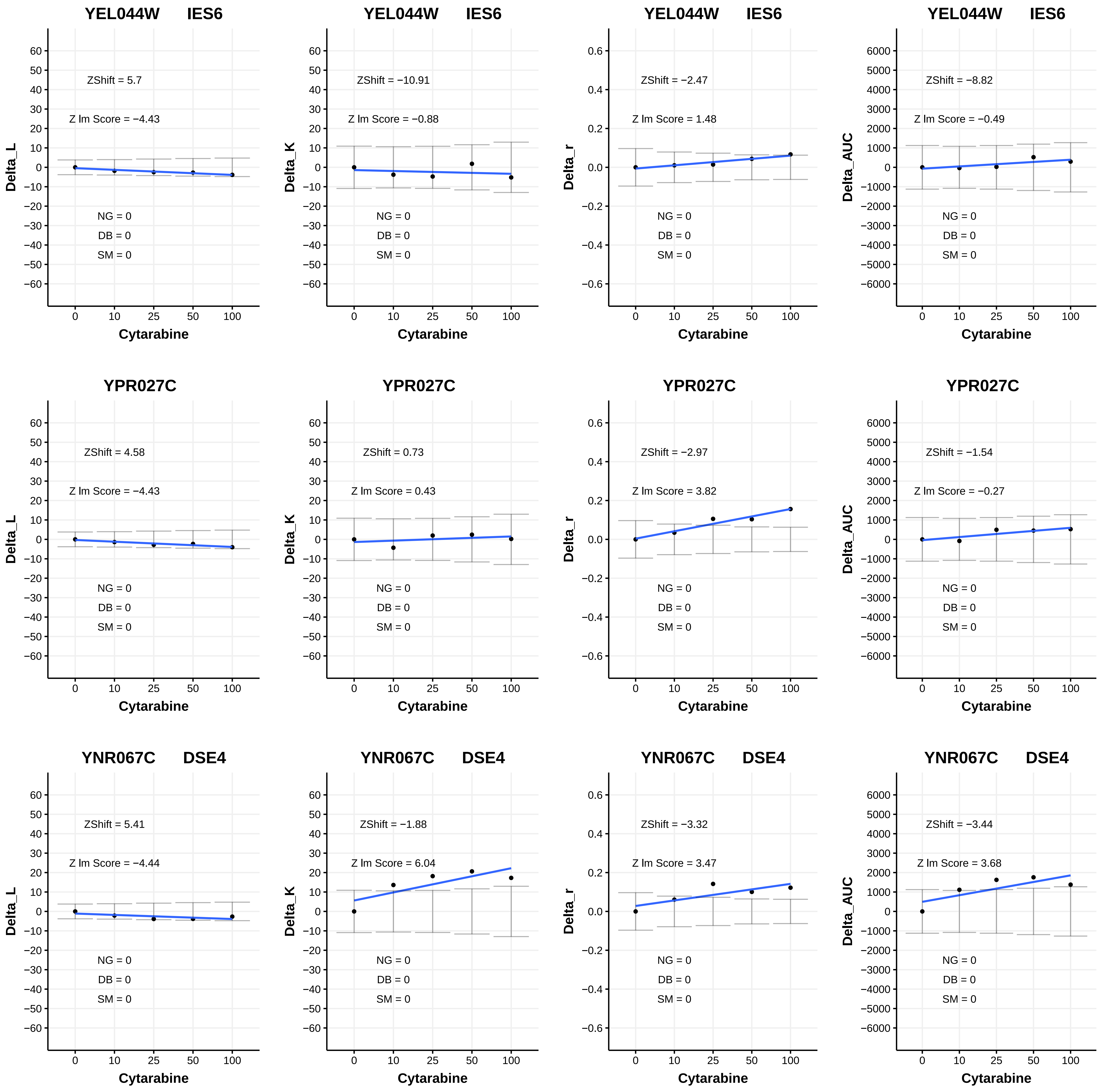

YEL044W IES6
ZShift = 5.7
Z lm Score = −4.43
NG = 0
DB = 0
SM = 0
Delta_L
Cytarabine
YEL044W IES6
ZShift = −10.91
Z lm Score = −0.88
NG = 0
DB = 0
SM = 0
Delta_K
Cytarabine
YEL044W IES6
ZShift = −2.47
Z lm Score = 1.48
NG = 0
DB = 0
SM = 0
Delta_r
Cytarabine
YEL044W IES6
ZShift = −8.82
Z lm Score = −0.49
NG = 0
DB = 0
SM = 0
Delta_AUC
Cytarabine
YPR027C
ZShift = 4.58
Z lm Score = −4.43
NG = 0
DB = 0
SM = 0
Delta_L
Cytarabine
YPR027C
ZShift = 0.73
Z lm Score = 0.43
NG = 0
DB = 0
SM = 0
Delta_K
Cytarabine
YPR027C
ZShift = −2.97
Z lm Score = 3.82
NG = 0
DB = 0
SM = 0
Delta_r
Cytarabine
YPR027C
ZShift = −1.54
Z lm Score = −0.27
NG = 0
DB = 0
SM = 0
Delta_AUC
Cytarabine
YNR067C DSE4
ZShift = 5.41
Z lm Score = −4.44
NG = 0
DB = 0
SM = 0
Delta_L
Cytarabine
YNR067C DSE4
ZShift = −1.88
Z lm Score = 6.04
NG = 0
DB = 0
SM = 0
Delta_K
Cytarabine
YNR067C DSE4
ZShift = −3.32
Z lm Score = 3.47
NG = 0
DB = 0
SM = 0
Delta_r
Cytarabine
YNR067C DSE4
ZShift = −3.44
Z lm Score = 3.68
NG = 0
DB = 0
SM = 0
Delta_AUC
Cytarabine

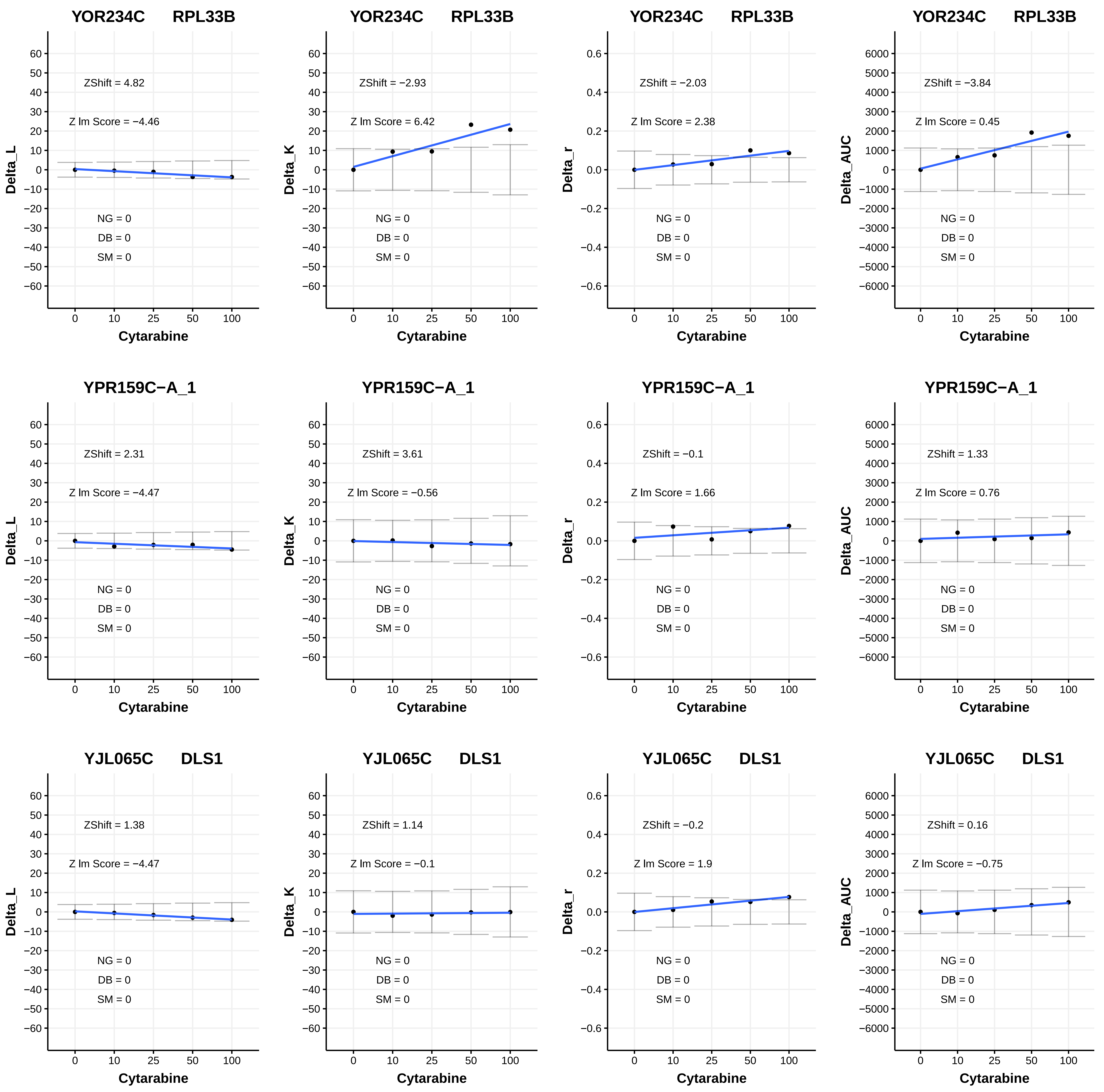

YOR234C RPL33B
ZShift = 4.82
Z lm Score = −4.46
NG = 0
DB = 0
SM = 0
Delta_L
Cytarabine
YOR234C RPL33B
ZShift = −2.93
Z lm Score = 6.42
NG = 0
DB = 0
SM = 0
Delta_K
Cytarabine
YOR234C RPL33B
ZShift = −2.03
Z lm Score = 2.38
NG = 0
DB = 0
SM = 0
Delta_r
Cytarabine
YOR234C RPL33B
ZShift = −3.84
Z lm Score = 0.45
NG = 0
DB = 0
SM = 0
Delta_AUC
Cytarabine
YPR159C−A_1
ZShift = 2.31
Z lm Score = −4.47
NG = 0
DB = 0
SM = 0
Delta_L
Cytarabine
YPR159C−A_1
ZShift = 3.61
Z lm Score = −0.56
NG = 0
DB = 0
SM = 0
Delta_K
Cytarabine
YPR159C−A_1
ZShift = −0.1
Z lm Score = 1.66
NG = 0
DB = 0
SM = 0
Delta_r
Cytarabine
YPR159C−A_1
ZShift = 1.33
Z lm Score = 0.76
NG = 0
DB = 0
SM = 0
Delta_AUC
Cytarabine
YJL065C DLS1
ZShift = 1.38
Z lm Score = −4.47
NG = 0
DB = 0
SM = 0
Delta_L
Cytarabine
YJL065C DLS1
ZShift = 1.14
Z lm Score = −0.1
NG = 0
DB = 0
SM = 0
Delta_K
Cytarabine
YJL065C DLS1
ZShift = −0.2
Z lm Score = 1.9
NG = 0
DB = 0
SM = 0
Delta_r
Cytarabine
YJL065C DLS1
ZShift = 0.16
Z lm Score = −0.75
NG = 0
DB = 0
SM = 0
Delta_AUC
Cytarabine

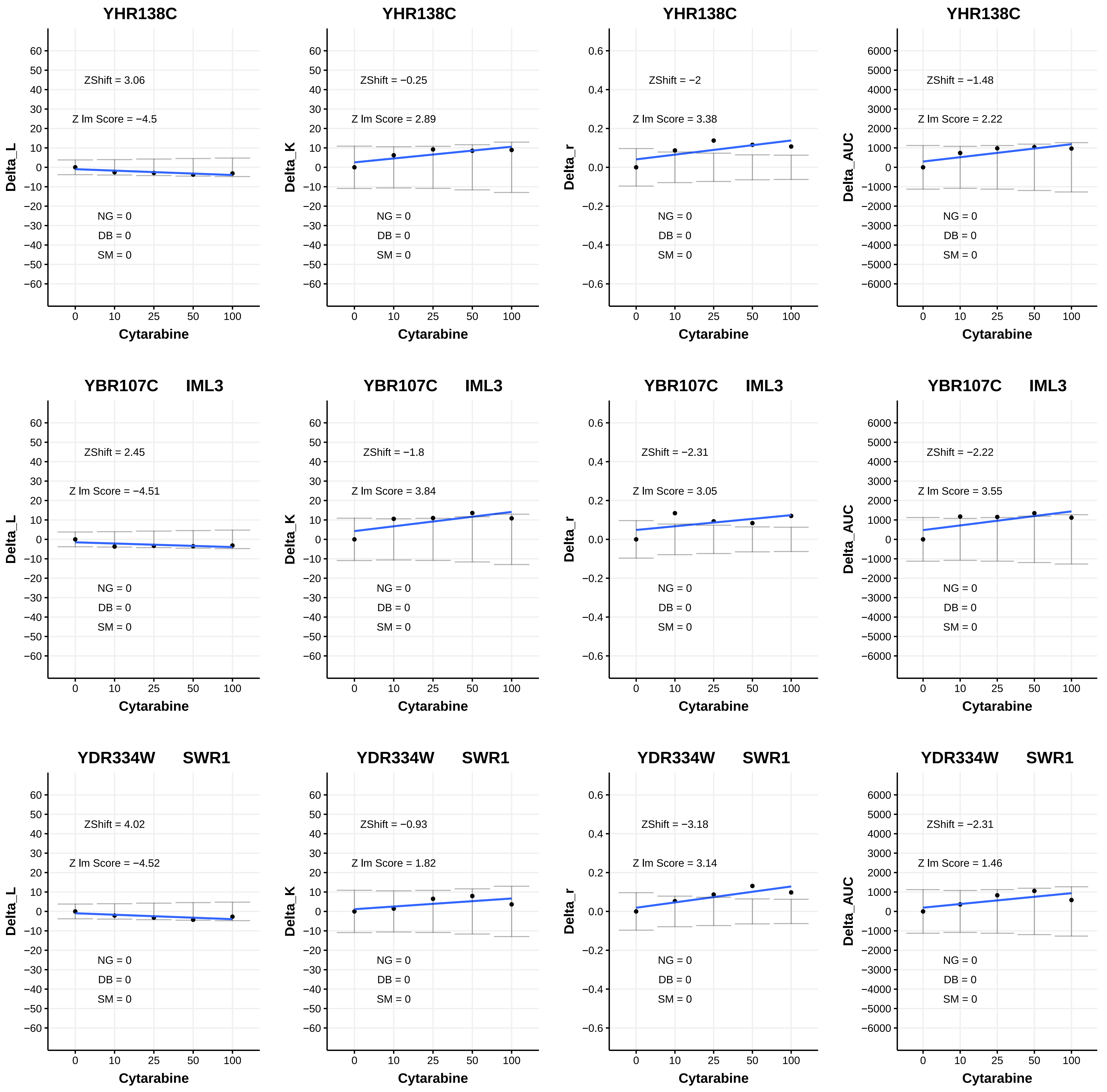

YHR138C
ZShift = 3.06
Z lm Score = −4.5
NG = 0
DB = 0
SM = 0
Delta_L
Cytarabine
YHR138C
ZShift = −0.25
Z lm Score = 2.89
NG = 0
DB = 0
SM = 0
Delta_K
Cytarabine
YHR138C
ZShift = −2
Z lm Score = 3.38
NG = 0
DB = 0
SM = 0
Delta_r
Cytarabine
YHR138C
ZShift = −1.48
Z lm Score = 2.22
NG = 0
DB = 0
SM = 0
Delta_AUC
Cytarabine
YBR107C IML3
ZShift = 2.45
Z lm Score = −4.51
NG = 0
DB = 0
SM = 0
Delta_L
Cytarabine
YBR107C IML3
ZShift = −1.8
Z lm Score = 3.84
NG = 0
DB = 0
SM = 0
Delta_K
Cytarabine
YBR107C IML3
ZShift = −2.31
Z lm Score = 3.05
NG = 0
DB = 0
SM = 0
Delta_r
Cytarabine
YBR107C IML3
ZShift = −2.22
Z lm Score = 3.55
NG = 0
DB = 0
SM = 0
Delta_AUC
Cytarabine
YDR334W SWR1
ZShift = 4.02
Z lm Score = −4.52
NG = 0
DB = 0
SM = 0
Delta_L
Cytarabine
YDR334W SWR1
ZShift = −0.93
Z lm Score = 1.82
NG = 0
DB = 0
SM = 0
Delta_K
Cytarabine
YDR334W SWR1
ZShift = −3.18
Z lm Score = 3.14
NG = 0
DB = 0
SM = 0
Delta_r
Cytarabine
YDR334W SWR1
ZShift = −2.31
Z lm Score = 1.46
NG = 0
DB = 0
SM = 0
Delta_AUC
Cytarabine

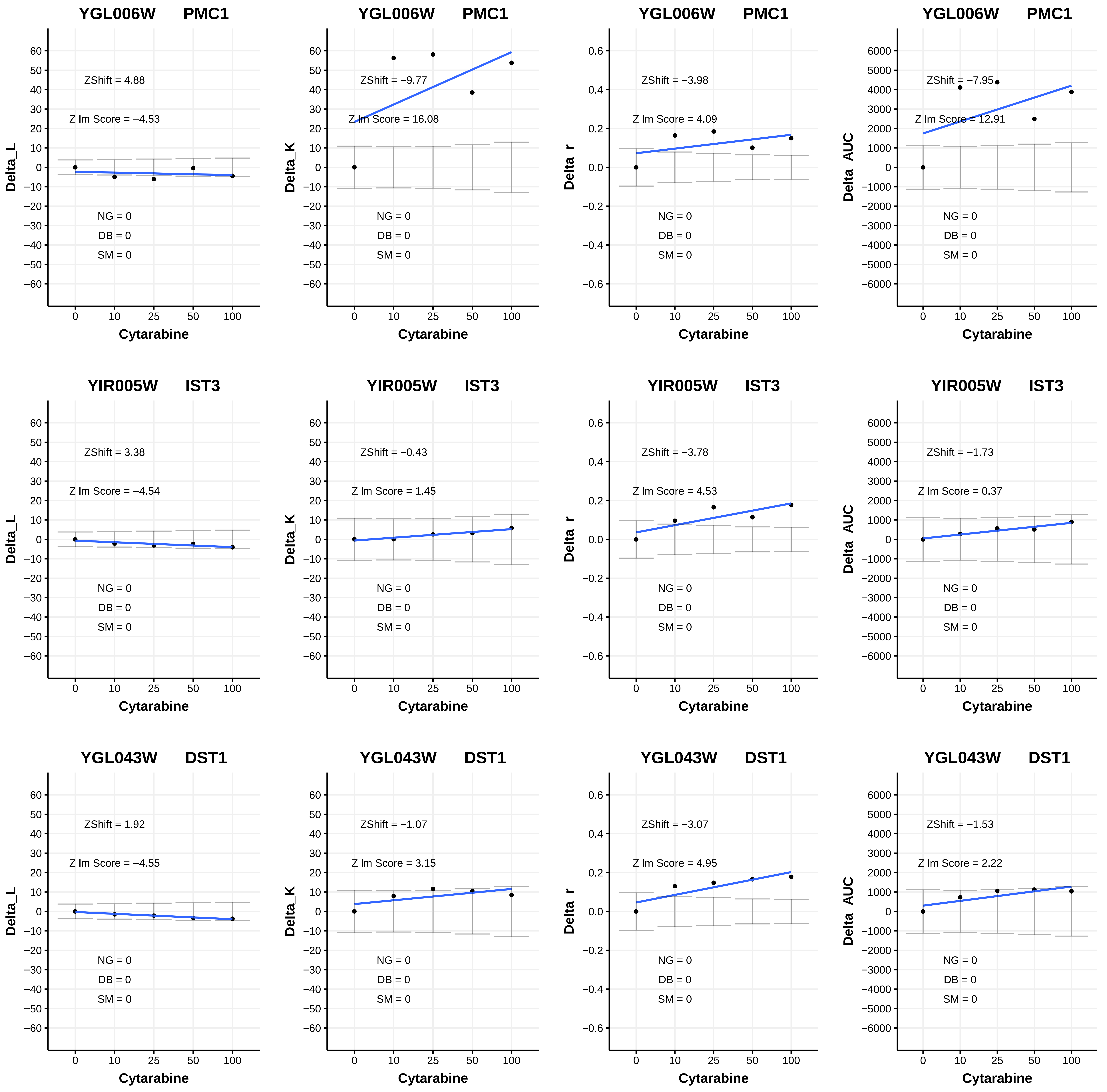
YGL006W PMC1
ZShift = 4.88
Z lm Score = −4.53
NG = 0
DB = 0
SM = 0
Delta_L
Cytarabine
YGL006W PMC1
ZShift = −9.77
Z lm Score = 16.08
NG = 0
DB = 0
SM = 0
Delta_K
Cytarabine
YGL006W PMC1
ZShift = −3.98
Z lm Score = 4.09
NG = 0
DB = 0
SM = 0
Delta_r
Cytarabine
YGL006W PMC1
ZShift = −7.95
Z lm Score = 12.91
NG = 0
DB = 0
SM = 0
Delta_AUC
Cytarabine
YIR005W IST3
ZShift = 3.38
Z lm Score = −4.54
NG = 0
DB = 0
SM = 0
Delta_L
Cytarabine
YIR005W IST3
ZShift = −0.43
Z lm Score = 1.45
NG = 0
DB = 0
SM = 0
Delta_K
Cytarabine
YIR005W IST3
ZShift = −3.78
Z lm Score = 4.53
NG = 0
DB = 0
SM = 0
Delta_r
Cytarabine
YIR005W IST3
ZShift = −1.73
Z lm Score = 0.37
NG = 0
DB = 0
SM = 0
Delta_AUC
Cytarabine
YGL043W DST1
ZShift = 1.92
Z lm Score = −4.55
NG = 0
DB = 0
SM = 0
Delta_L
Cytarabine
YGL043W DST1
ZShift = −1.07
Z lm Score = 3.15
NG = 0
DB = 0
SM = 0
Delta_K
Cytarabine
YGL043W DST1
ZShift = −3.07
Z lm Score = 4.95
NG = 0
DB = 0
SM = 0
Delta_r
Cytarabine
YGL043W DST1
ZShift = −1.53
Z lm Score = 2.22
NG = 0
DB = 0
SM = 0
Delta_AUC
Cytarabine

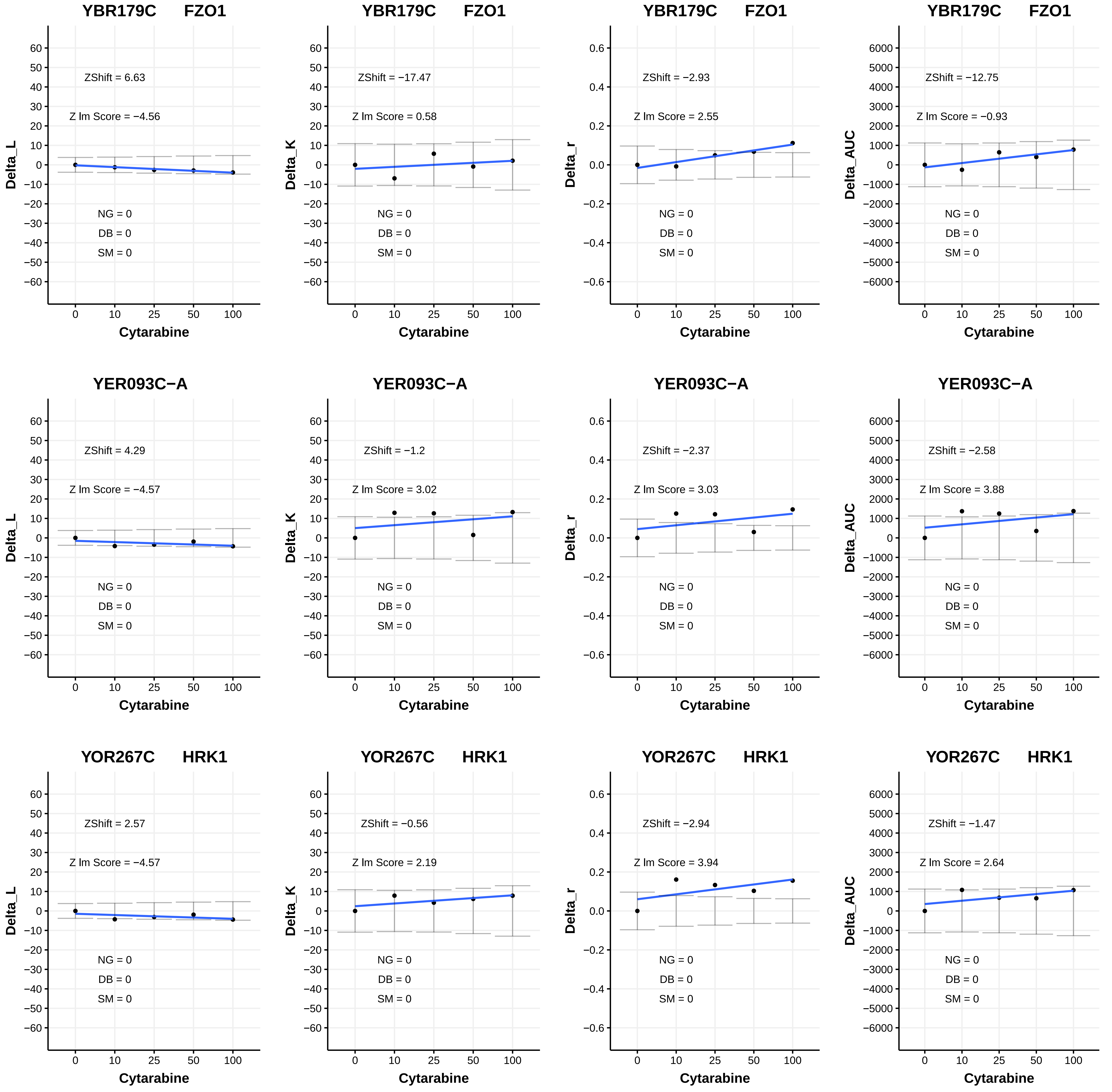
YBR179C FZO1
ZShift = 6.63
Z lm Score = −4.56
NG = 0
DB = 0
SM = 0
Delta_L
Cytarabine
YBR179C FZO1
ZShift = −17.47
Z lm Score = 0.58
NG = 0
DB = 0
SM = 0
Delta_K
Cytarabine
YBR179C FZO1
ZShift = −2.93
Z lm Score = 2.55
NG = 0
DB = 0
SM = 0
Delta_r
Cytarabine
YBR179C FZO1
ZShift = −12.75
Z lm Score = −0.93
NG = 0
DB = 0
SM = 0
Delta_AUC
Cytarabine
YER093C−A
ZShift = 4.29
Z lm Score = −4.57
NG = 0
DB = 0
SM = 0
Delta_L
Cytarabine
YER093C−A
ZShift = −1.2
Z lm Score = 3.02
NG = 0
DB = 0
SM = 0
Delta_K
Cytarabine
YER093C−A
ZShift = −2.37
Z lm Score = 3.03
NG = 0
DB = 0
SM = 0
Delta_r
Cytarabine
YER093C−A
ZShift = −2.58
Z lm Score = 3.88
NG = 0
DB = 0
SM = 0
Delta_AUC
Cytarabine
YOR267C HRK1
ZShift = 2.57
Z lm Score = −4.57
NG = 0
DB = 0
SM = 0
Delta_L
Cytarabine
YOR267C HRK1
ZShift = −0.56
Z lm Score = 2.19
NG = 0
DB = 0
SM = 0
Delta_K
Cytarabine
YOR267C HRK1
ZShift = −2.94
Z lm Score = 3.94
NG = 0
DB = 0
SM = 0
Delta_r
Cytarabine
YOR267C HRK1
ZShift = −1.47
Z lm Score = 2.64
NG = 0
DB = 0
SM = 0
Delta_AUC
Cytarabine

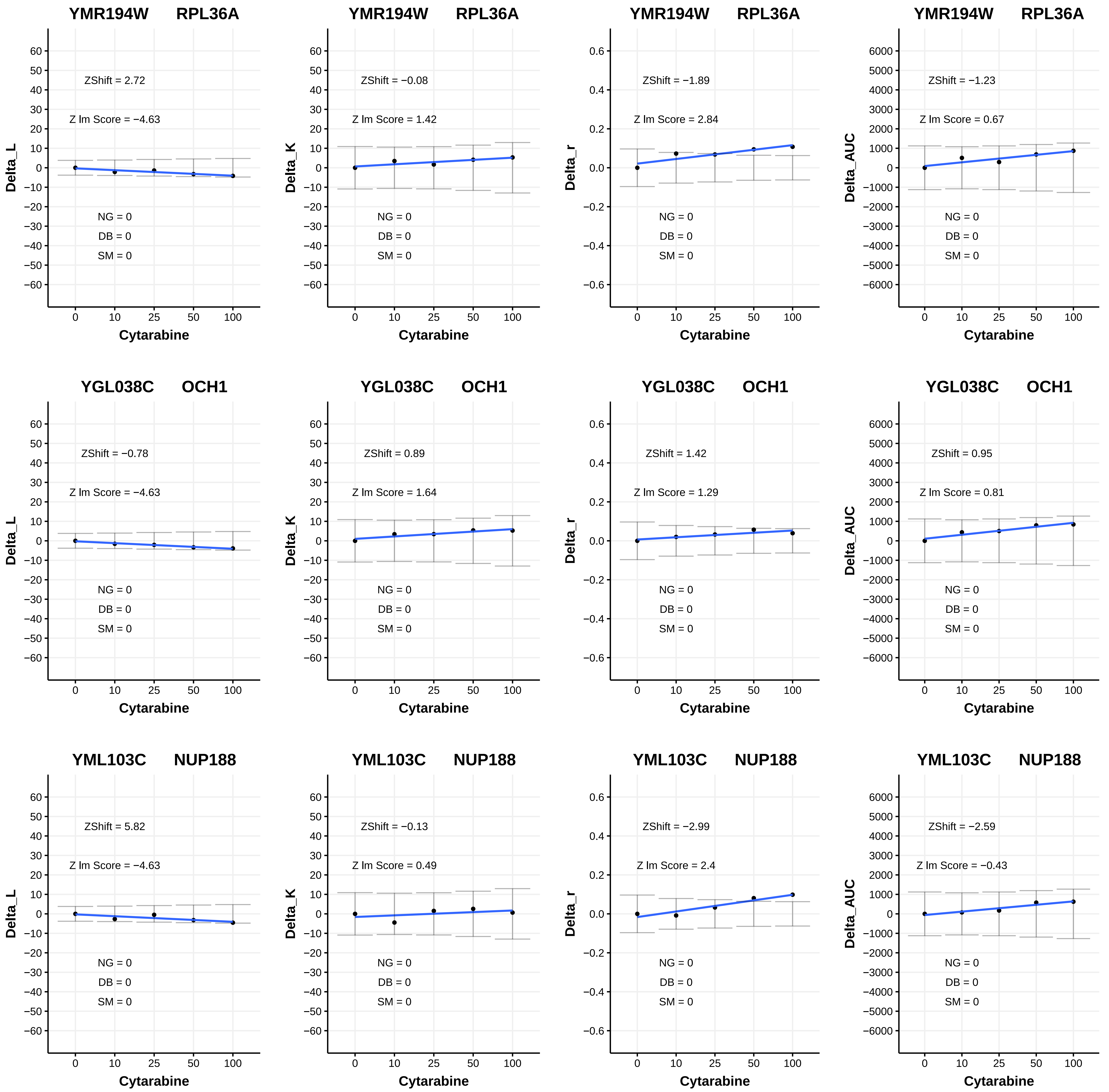

YMR194W RPL36A
Delta_L
ZShift = 2.72
Z lm Score = −4.63
NG = 0
DB = 0
SM = 0
Cytarabine
YMR194W RPL36A
Delta_K
ZShift = −0.08
Z lm Score = 1.42
NG = 0
DB = 0
SM = 0
Cytarabine
YMR194W RPL36A
Delta_r
ZShift = −1.89
Z lm Score = 2.84
NG = 0
DB = 0
SM = 0
Cytarabine
YMR194W RPL36A
Delta_AUC
ZShift = −1.23
Z lm Score = 0.67
NG = 0
DB = 0
SM = 0
Cytarabine
YGL038C OCH1
Delta_L
ZShift = −0.78
Z lm Score = −4.63
NG = 0
DB = 0
SM = 0
Cytarabine
YGL038C OCH1
Delta_K
ZShift = 0.89
Z lm Score = 1.64
NG = 0
DB = 0
SM = 0
Cytarabine
YGL038C OCH1
Delta_r
ZShift = 1.42
Z lm Score = 1.29
NG = 0
DB = 0
SM = 0
Cytarabine
YGL038C OCH1
Delta_AUC
ZShift = 0.95
Z lm Score = 0.81
NG = 0
DB = 0
SM = 0
Cytarabine
YML103C NUP188
Delta_L
ZShift = 5.82
Z lm Score = −4.63
NG = 0
DB = 0
SM = 0
Cytarabine
YML103C NUP188
Delta_K
ZShift = −0.13
Z lm Score = 0.49
NG = 0
DB = 0
SM = 0
Cytarabine
YML103C NUP188
Delta_r
ZShift = −2.99
Z lm Score = 2.4
NG = 0
DB = 0
SM = 0
Cytarabine
YML103C NUP188
Delta_AUC
ZShift = −2.59
Z lm Score = −0.43
NG = 0
DB = 0
SM = 0
Cytarabine

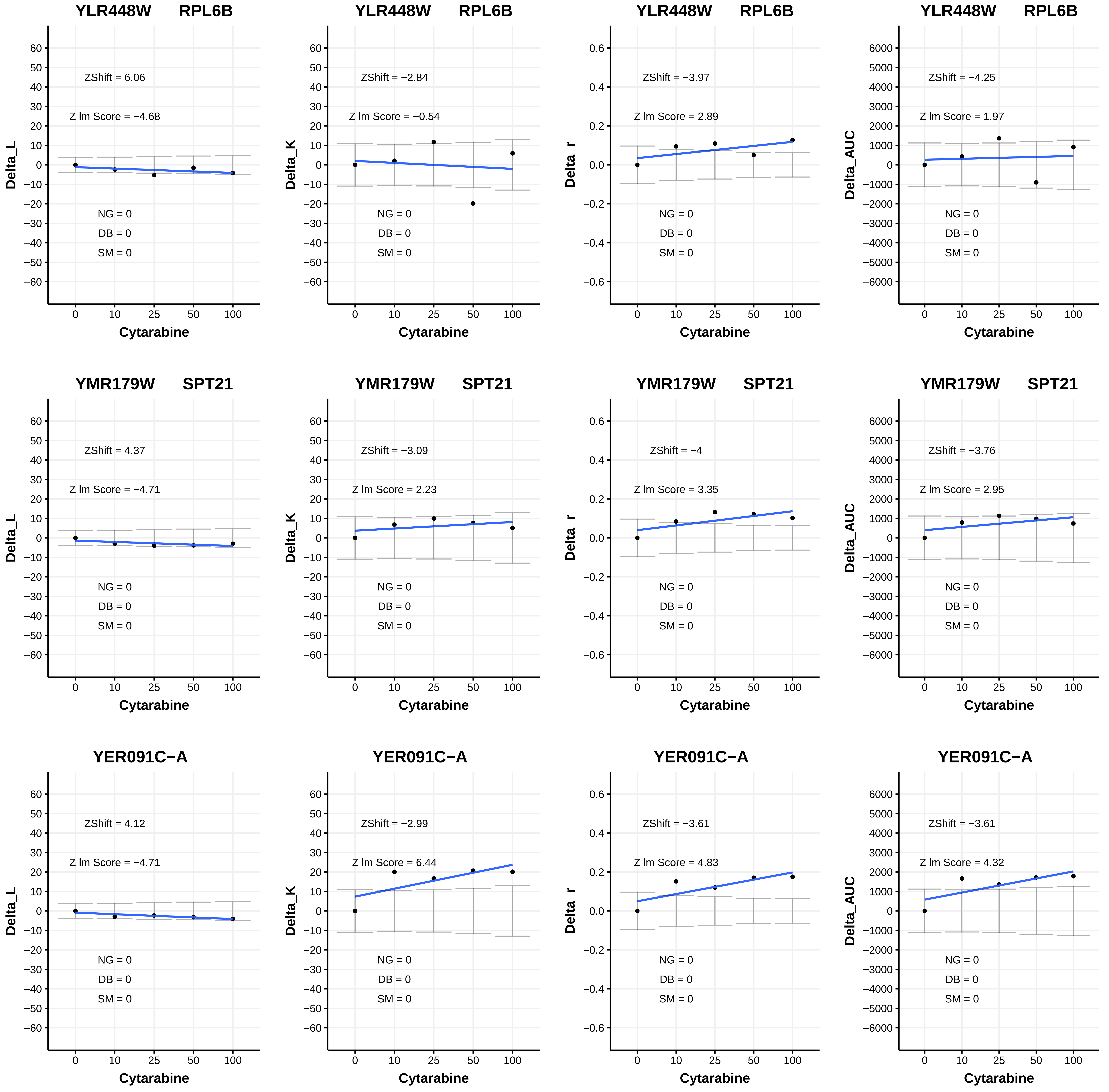
YLR448W RPL6B
ZShift = 6.06
Z lm Score = −4.68
NG = 0
DB = 0
SM = 0
Delta_L
Cytarabine
YLR448W RPL6B
ZShift = −2.84
Z lm Score = −0.54
NG = 0
DB = 0
SM = 0
Delta_K
Cytarabine
YLR448W RPL6B
ZShift = −3.97
Z lm Score = 2.89
NG = 0
DB = 0
SM = 0
Delta_r
Cytarabine
YLR448W RPL6B
ZShift = −4.25
Z lm Score = 1.97
NG = 0
DB = 0
SM = 0
Delta_AUC
Cytarabine
YMR179W SPT21
ZShift = 4.37
Z lm Score = −4.71
NG = 0
DB = 0
SM = 0
Delta_L
Cytarabine
YMR179W SPT21
ZShift = −3.09
Z lm Score = 2.23
NG = 0
DB = 0
SM = 0
Delta_K
Cytarabine
YMR179W SPT21
ZShift = −4
Z lm Score = 3.35
NG = 0
DB = 0
SM = 0
Delta_r
Cytarabine
YMR179W SPT21
ZShift = −3.76
Z lm Score = 2.95
NG = 0
DB = 0
SM = 0
Delta_AUC
Cytarabine
YER091C−A
ZShift = 4.12
Z lm Score = −4.71
NG = 0
DB = 0
SM = 0
Delta_L
Cytarabine
YER091C−A
ZShift = −2.99
Z lm Score = 6.44
NG = 0
DB = 0
SM = 0
Delta_K
Cytarabine
YER091C−A
ZShift = −3.61
Z lm Score = 4.83
NG = 0
DB = 0
SM = 0
Delta_r
Cytarabine
YER091C−A
ZShift = −3.61
Z lm Score = 4.32
NG = 0
DB = 0
SM = 0
Delta_AUC
Cytarabine

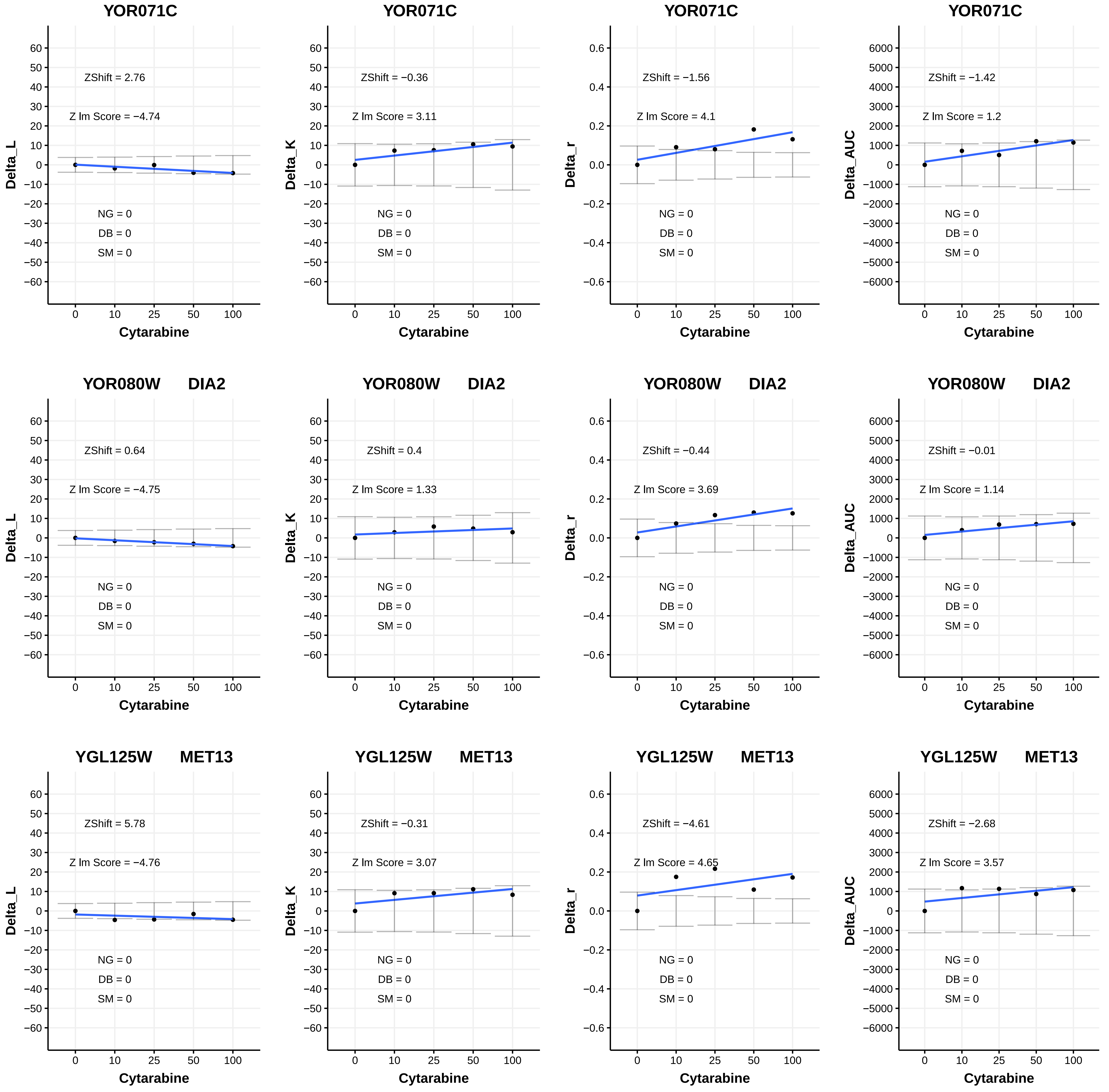

YOR071C
ZShift = 2.76
Z lm Score = −4.74
NG = 0
DB = 0
SM = 0
Delta_L
Cytarabine
YOR071C
ZShift = −0.36
Z lm Score = 3.11
NG = 0
DB = 0
SM = 0
Delta_K
Cytarabine
YOR071C
ZShift = −1.56
Z lm Score = 4.1
NG = 0
DB = 0
SM = 0
Delta_r
Cytarabine
YOR071C
ZShift = −1.42
Z lm Score = 1.2
NG = 0
DB = 0
SM = 0
Delta_AUC
Cytarabine
YOR080W DIA2
ZShift = 0.64
Z lm Score = −4.75
NG = 0
DB = 0
SM = 0
Delta_L
Cytarabine
YOR080W DIA2
ZShift = 0.4
Z lm Score = 1.33
NG = 0
DB = 0
SM = 0
Delta_K
Cytarabine
YOR080W DIA2
ZShift = −0.44
Z lm Score = 3.69
NG = 0
DB = 0
SM = 0
Delta_r
Cytarabine
YOR080W DIA2
ZShift = −0.01
Z lm Score = 1.14
NG = 0
DB = 0
SM = 0
Delta_AUC
Cytarabine
YGL125W MET13
ZShift = 5.78
Z lm Score = −4.76
NG = 0
DB = 0
SM = 0
Delta_L
Cytarabine
YGL125W MET13
ZShift = −0.31
Z lm Score = 3.07
NG = 0
DB = 0
SM = 0
Delta_K
Cytarabine
YGL125W MET13
ZShift = −4.61
Z lm Score = 4.65
NG = 0
DB = 0
SM = 0
Delta_r
Cytarabine
YGL125W MET13
ZShift = −2.68
Z lm Score = 3.57
NG = 0
DB = 0
SM = 0
Delta_AUC
Cytarabine

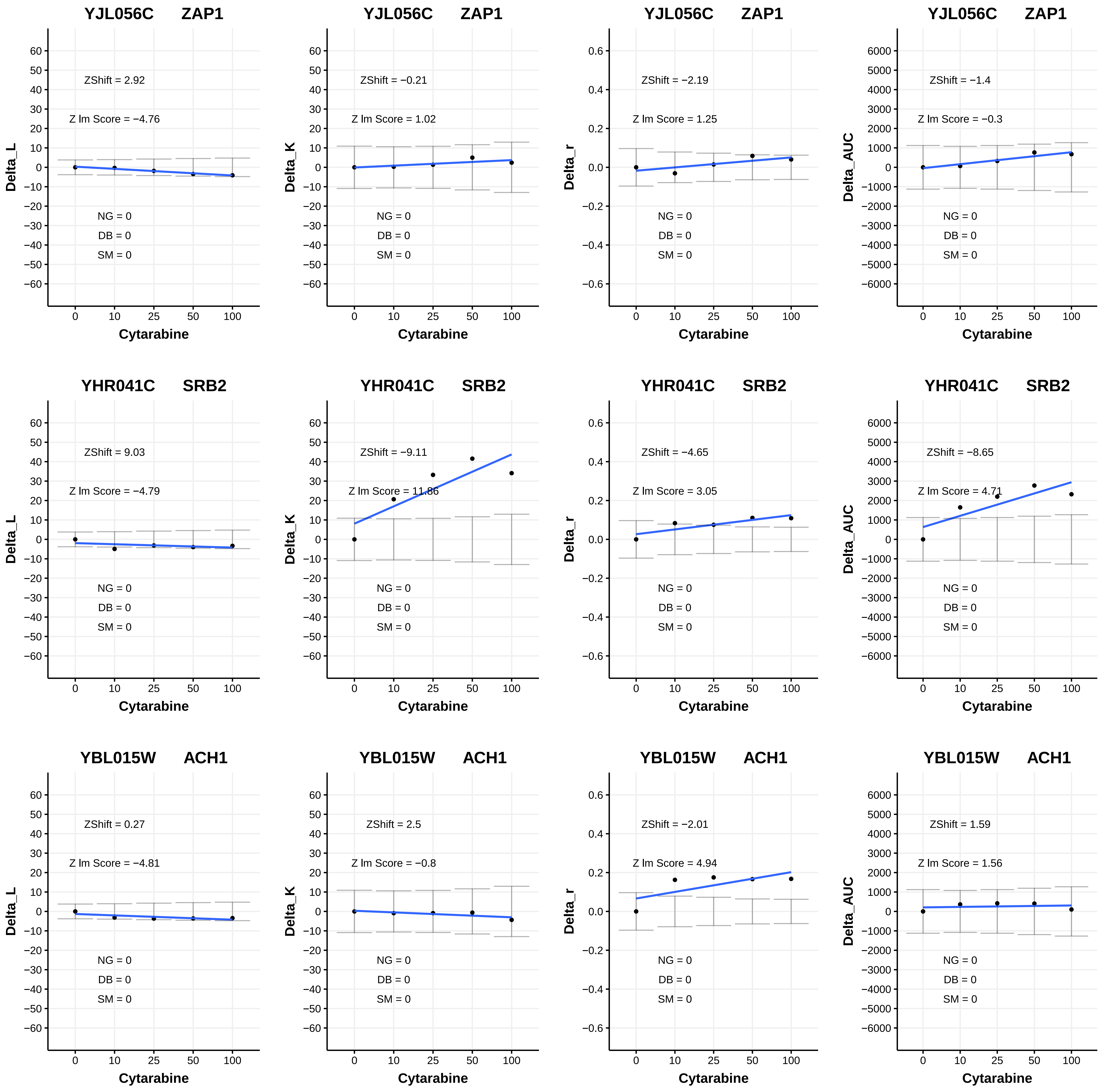

YJL056C ZAP1
Delta_L
ZShift = 2.92
Z lm Score = −4.76
NG = 0
DB = 0
SM = 0
Cytarabine
YJL056C ZAP1
Delta_K
ZShift = −0.21
Z lm Score = 1.02
NG = 0
DB = 0
SM = 0
Cytarabine
YJL056C ZAP1
Delta_r
ZShift = −2.19
Z lm Score = 1.25
NG = 0
DB = 0
SM = 0
Cytarabine
YJL056C ZAP1
Delta_AUC
ZShift = −1.4
Z lm Score = −0.3
NG = 0
DB = 0
SM = 0
Cytarabine
YHR041C SRB2
Delta_L
ZShift = 9.03
Z lm Score = −4.79
NG = 0
DB = 0
SM = 0
Cytarabine
YHR041C SRB2
Delta_K
ZShift = −9.11
Z lm Score = 11.86
NG = 0
DB = 0
SM = 0
Cytarabine
YHR041C SRB2
Delta_r
ZShift = −4.65
Z lm Score = 3.05
NG = 0
DB = 0
SM = 0
Cytarabine
YHR041C SRB2
Delta_AUC
ZShift = −8.65
Z lm Score = 4.71
NG = 0
DB = 0
SM = 0
Cytarabine
YBL015W ACH1
Delta_L
ZShift = 0.27
Z lm Score = −4.81
NG = 0
DB = 0
SM = 0
Cytarabine
YBL015W ACH1
Delta_K
ZShift = 2.5
Z lm Score = −0.8
NG = 0
DB = 0
SM = 0
Cytarabine
YBL015W ACH1
Delta_r
ZShift = −2.01
Z lm Score = 4.94
NG = 0
DB = 0
SM = 0
Cytarabine
YBL015W ACH1
Delta_AUC
ZShift = 1.59
Z lm Score = 1.56
NG = 0
DB = 0
SM = 0
Cytarabine

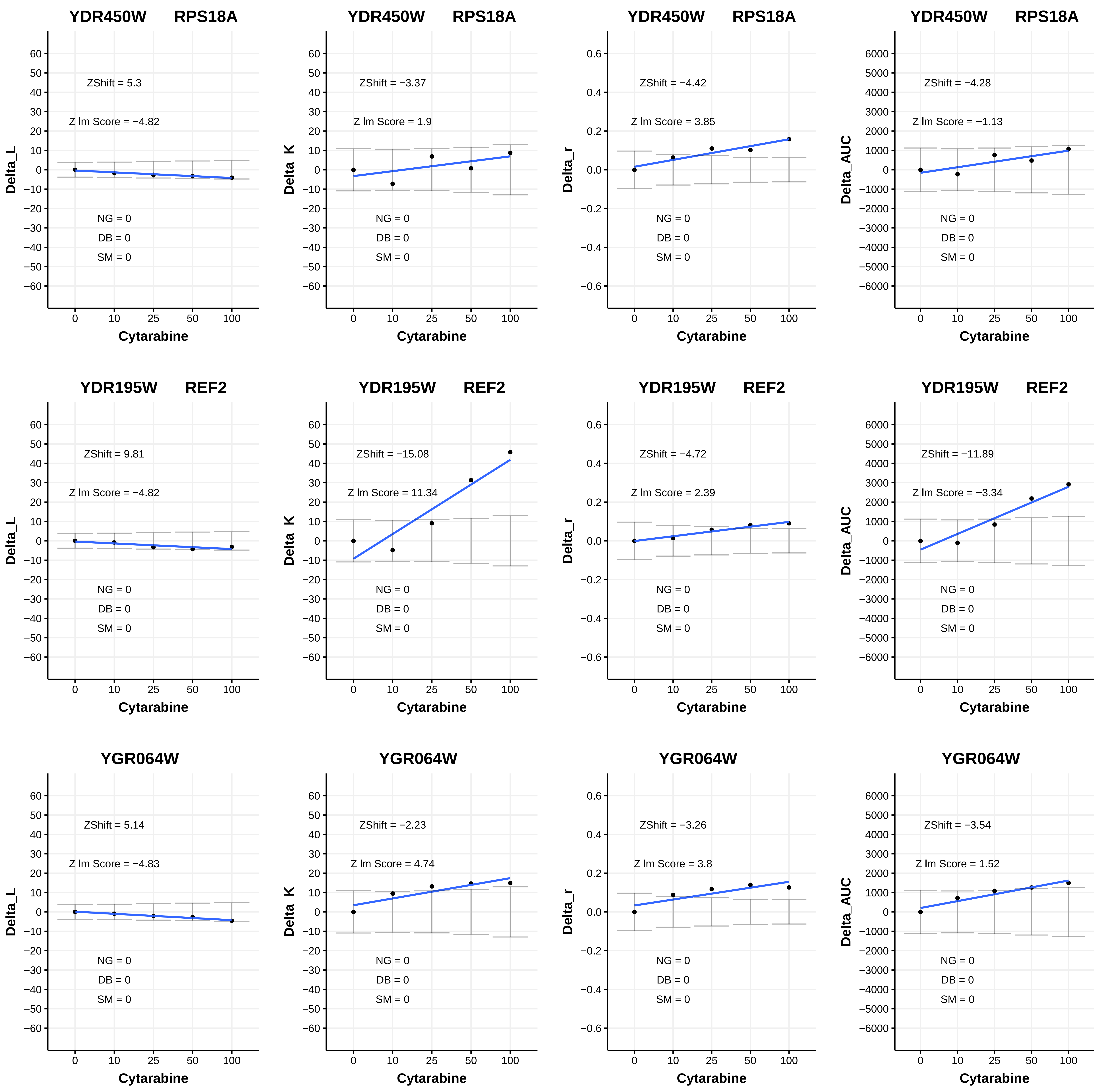

YDR450W RPS18A
ZShift = 5.3
Z lm Score = −4.82
NG = 0
DB = 0
SM = 0
Delta_L
Cytarabine
YDR450W RPS18A
ZShift = −3.37
Z lm Score = 1.9
NG = 0
DB = 0
SM = 0
Delta_K
Cytarabine
YDR450W RPS18A
ZShift = −4.42
Z lm Score = 3.85
NG = 0
DB = 0
SM = 0
Delta_r
Cytarabine
YDR450W RPS18A
ZShift = −4.28
Z lm Score = −1.13
NG = 0
DB = 0
SM = 0
Delta_AUC
Cytarabine
YDR195W REF2
ZShift = 9.81
Z lm Score = −4.82
NG = 0
DB = 0
SM = 0
Delta_L
Cytarabine
YDR195W REF2
ZShift = −15.08
Z lm Score = 11.34
NG = 0
DB = 0
SM = 0
Delta_K
Cytarabine
YDR195W REF2
ZShift = −4.72
Z lm Score = 2.39
NG = 0
DB = 0
SM = 0
Delta_r
Cytarabine
YDR195W REF2
ZShift = −11.89
Z lm Score = −3.34
NG = 0
DB = 0
SM = 0
Delta_AUC
Cytarabine
YGR064W
ZShift = 5.14
Z lm Score = −4.83
NG = 0
DB = 0
SM = 0
Delta_L
Cytarabine
YGR064W
ZShift = −2.23
Z lm Score = 4.74
NG = 0
DB = 0
SM = 0
Delta_K
Cytarabine
YGR064W
ZShift = −3.26
Z lm Score = 3.8
NG = 0
DB = 0
SM = 0
Delta_r
Cytarabine
YGR064W
ZShift = −3.54
Z lm Score = 1.52
NG = 0
DB = 0
SM = 0
Delta_AUC
Cytarabine

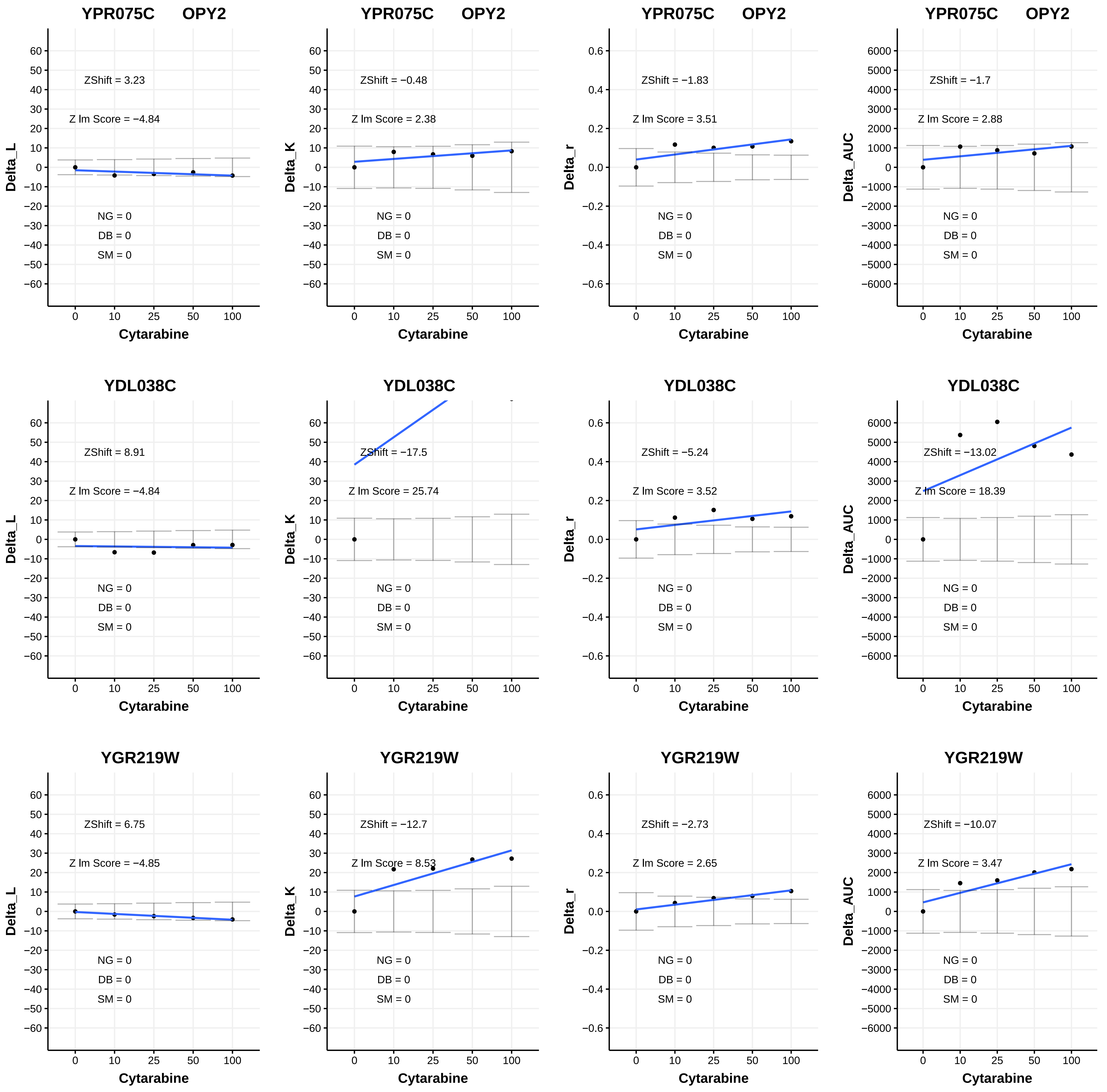
YPR075C OPY2
Delta_L
ZShift = 3.23
Z lm Score = −4.84
NG = 0
DB = 0
SM = 0
Cytarabine
YPR075C OPY2
Delta_K
ZShift = −0.48
Z lm Score = 2.38
NG = 0
DB = 0
SM = 0
Cytarabine
YPR075C OPY2
Delta_r
ZShift = −1.83
Z lm Score = 3.51
NG = 0
DB = 0
SM = 0
Cytarabine
YPR075C OPY2
Delta_AUC
ZShift = −1.7
Z lm Score = 2.88
NG = 0
DB = 0
SM = 0
Cytarabine
YDL038C
Delta_L
ZShift = 8.91
Z lm Score = −4.84
NG = 0
DB = 0
SM = 0
Cytarabine
YDL038C
Delta_K
ZShift = −17.5
Z lm Score = 25.74
NG = 0
DB = 0
SM = 0
Cytarabine
YDL038C
Delta_r
ZShift = −5.24
Z lm Score = 3.52
NG = 0
DB = 0
SM = 0
Cytarabine
YDL038C
Delta_AUC
ZShift = −13.02
Z lm Score = 18.39
NG = 0
DB = 0
SM = 0
Cytarabine
YGR219W
Delta_L
ZShift = 6.75
Z lm Score = −4.85
NG = 0
DB = 0
SM = 0
Cytarabine
YGR219W
Delta_K
ZShift = −12.7
Z lm Score = 8.53
NG = 0
DB = 0
SM = 0
Cytarabine
YGR219W
Delta_r
ZShift = −2.73
Z lm Score = 2.65
NG = 0
DB = 0
SM = 0
Cytarabine
YGR219W
Delta_AUC
ZShift = −10.07
Z lm Score = 3.47
NG = 0
DB = 0
SM = 0
Cytarabine

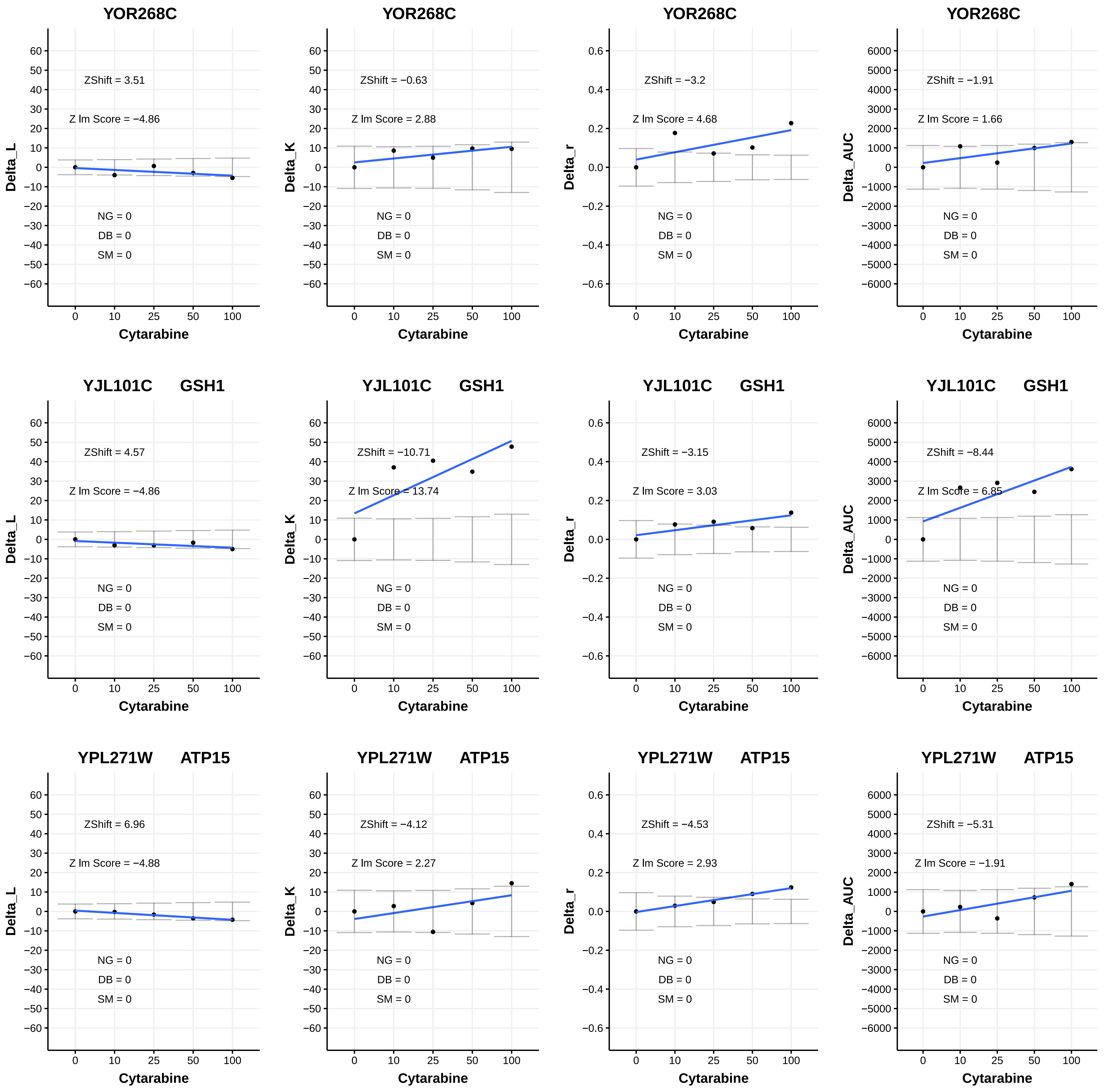
YOR268C
Delta_L
ZShift = 3.51
Z lm Score = −4.86
NG = 0
DB = 0
SM = 0
Cytarabine
YOR268C
Delta_K
ZShift = −0.63
Z lm Score = 2.88
NG = 0
DB = 0
SM = 0
Cytarabine
YOR268C
Delta_r
ZShift = −3.2
Z lm Score = 4.68
NG = 0
DB = 0
SM = 0
Cytarabine
YOR268C
Delta_AUC
ZShift = −1.91
Z lm Score = 1.66
NG = 0
DB = 0
SM = 0
Cytarabine
YJL101C GSH1
Delta_L
ZShift = 4.57
Z lm Score = −4.86
NG = 0
DB = 0
SM = 0
Cytarabine
YJL101C GSH1
Delta_K
ZShift = −10.71
Z lm Score = 13.74
NG = 0
DB = 0
SM = 0
Cytarabine
YJL101C GSH1
Delta_r
ZShift = −3.15
Z lm Score = 3.03
NG = 0
DB = 0
SM = 0
Cytarabine
YJL101C GSH1
Delta_AUC
ZShift = −8.44
Z lm Score = 6.85
NG = 0
DB = 0
SM = 0
Cytarabine
YPL271W ATP15
Delta_L
ZShift = 6.96
Z lm Score = −4.88
NG = 0
DB = 0
SM = 0
Cytarabine
YPL271W ATP15
Delta_K
ZShift = −4.12
Z lm Score = 2.27
NG = 0
DB = 0
SM = 0
Cytarabine
YPL271W ATP15
Delta_r
ZShift = −4.53
Z lm Score = 2.93
NG = 0
DB = 0
SM = 0
Cytarabine
YPL271W ATP15
Delta_AUC
ZShift = −5.31
Z lm Score = −1.91
NG = 0
DB = 0
SM = 0
Cytarabine

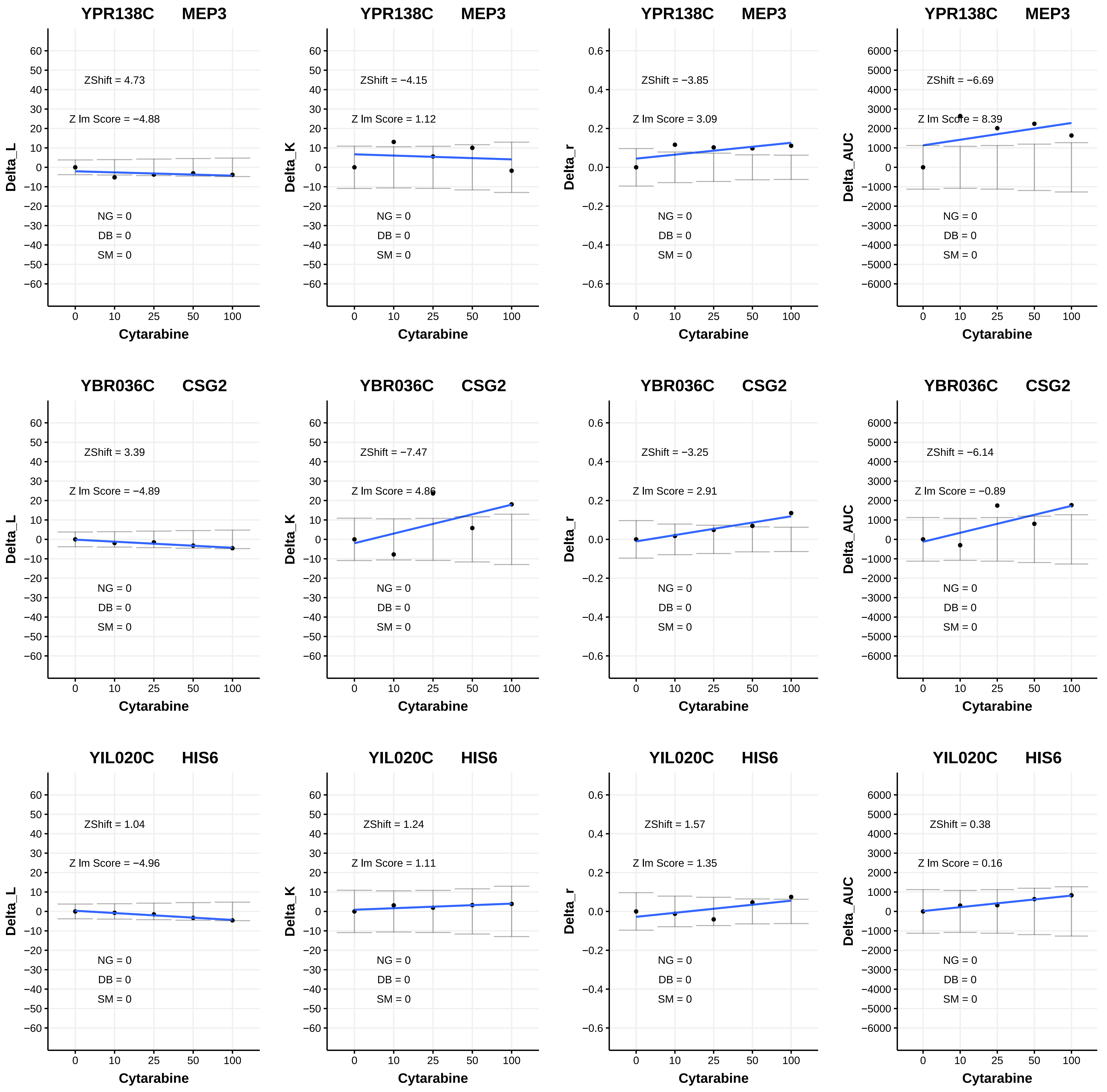

YPR138C MEP3
ZShift = 4.73
Z lm Score = −4.88
NG = 0
DB = 0
SM = 0
Delta_L
Cytarabine
YPR138C MEP3
ZShift = −4.15
Z lm Score = 1.12
NG = 0
DB = 0
SM = 0
Delta_K
Cytarabine
YPR138C MEP3
ZShift = −3.85
Z lm Score = 3.09
NG = 0
DB = 0
SM = 0
Delta_r
Cytarabine
YPR138C MEP3
ZShift = −6.69
Z lm Score = 8.39
NG = 0
DB = 0
SM = 0
Delta_AUC
Cytarabine
YBR036C CSG2
ZShift = 3.39
Z lm Score = −4.89
NG = 0
DB = 0
SM = 0
Delta_L
Cytarabine
YBR036C CSG2
ZShift = −7.47
Z lm Score = 4.86
NG = 0
DB = 0
SM = 0
Delta_K
Cytarabine
YBR036C CSG2
ZShift = −3.25
Z lm Score = 2.91
NG = 0
DB = 0
SM = 0
Delta_r
Cytarabine
YBR036C CSG2
ZShift = −6.14
Z lm Score = −0.89
NG = 0
DB = 0
SM = 0
Delta_AUC
Cytarabine
YIL020C HIS6
ZShift = 1.04
Z lm Score = −4.96
NG = 0
DB = 0
SM = 0
Delta_L
Cytarabine
YIL020C HIS6
ZShift = 1.24
Z lm Score = 1.11
NG = 0
DB = 0
SM = 0
Delta_K
Cytarabine
YIL020C HIS6
ZShift = 1.57
Z lm Score = 1.35
NG = 0
DB = 0
SM = 0
Delta_r
Cytarabine
YIL020C HIS6
ZShift = 0.38
Z lm Score = 0.16
NG = 0
DB = 0
SM = 0
Delta_AUC
Cytarabine

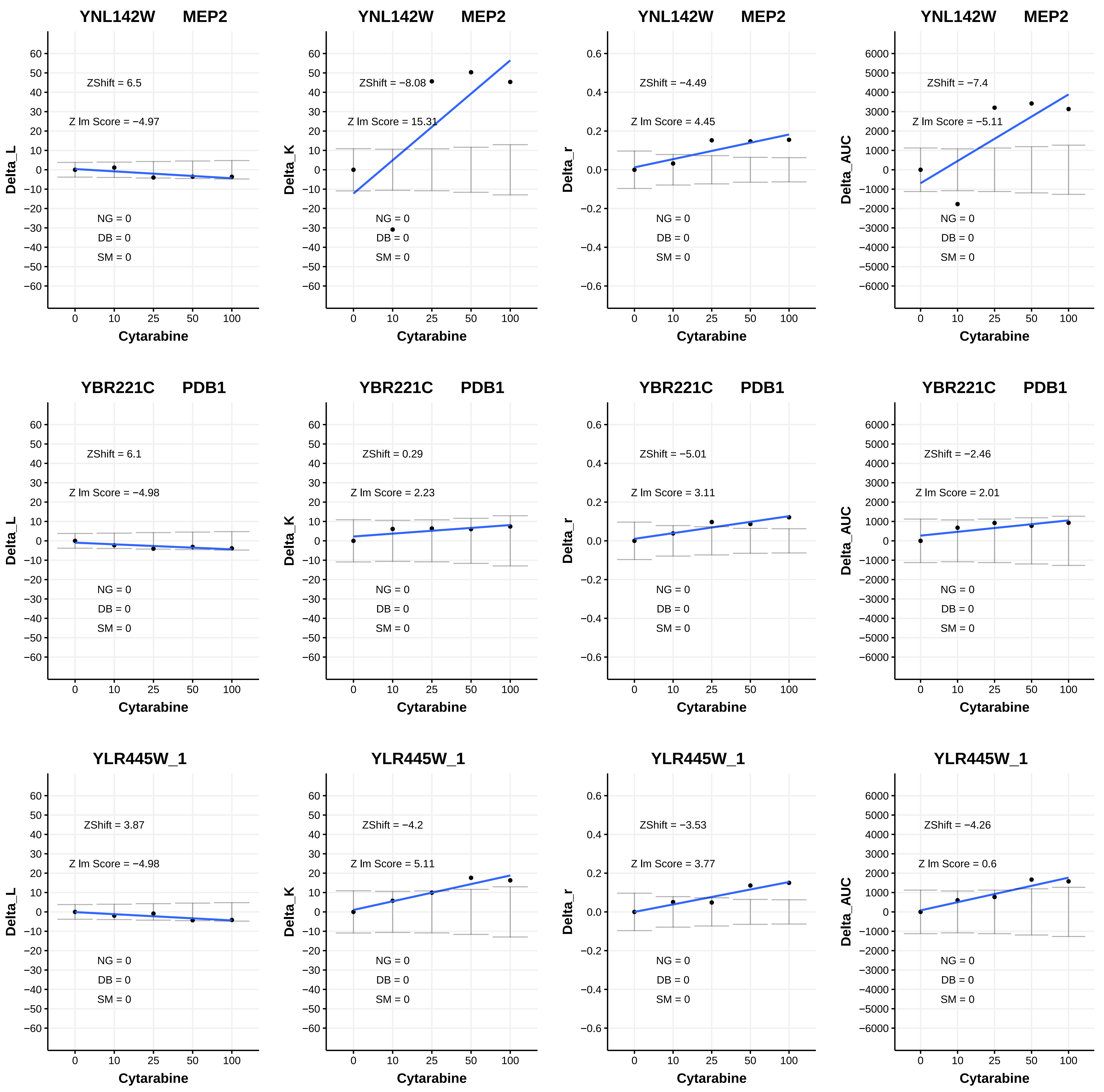

YNL142W MEP2
Delta_L
ZShift = 6.5
Z lm Score = −4.97
NG = 0
DB = 0
SM = 0
Cytarabine
YNL142W MEP2
Delta_K
ZShift = −8.08
Z lm Score = 15.31
NG = 0
DB = 0
SM = 0
Cytarabine
YNL142W MEP2
Delta_r
ZShift = −4.49
Z lm Score = 4.45
NG = 0
DB = 0
SM = 0
Cytarabine
YNL142W MEP2
Delta_AUC
ZShift = −7.4
Z lm Score = −5.11
NG = 0
DB = 0
SM = 0
Cytarabine
YBR221C PDB1
Delta_L
ZShift = 6.1
Z lm Score = −4.98
NG = 0
DB = 0
SM = 0
Cytarabine
YBR221C PDB1
Delta_K
ZShift = 0.29
Z lm Score = 2.23
NG = 0
DB = 0
SM = 0
Cytarabine
YBR221C PDB1
Delta_r
ZShift = −5.01
Z lm Score = 3.11
NG = 0
DB = 0
SM = 0
Cytarabine
YBR221C PDB1
Delta_AUC
ZShift = −2.46
Z lm Score = 2.01
NG = 0
DB = 0
SM = 0
Cytarabine
YLR445W_1
Delta_L
ZShift = 3.87
Z lm Score = −4.98
NG = 0
DB = 0
SM = 0
Cytarabine
YLR445W_1
Delta_K
ZShift = −4.2
Z lm Score = 5.11
NG = 0
DB = 0
SM = 0
Cytarabine
YLR445W_1
Delta_r
ZShift = −3.53
Z lm Score = 3.77
NG = 0
DB = 0
SM = 0
Cytarabine
YLR445W_1
Delta_AUC
ZShift = −4.26
Z lm Score = 0.6
NG = 0
DB = 0
SM = 0
Cytarabine

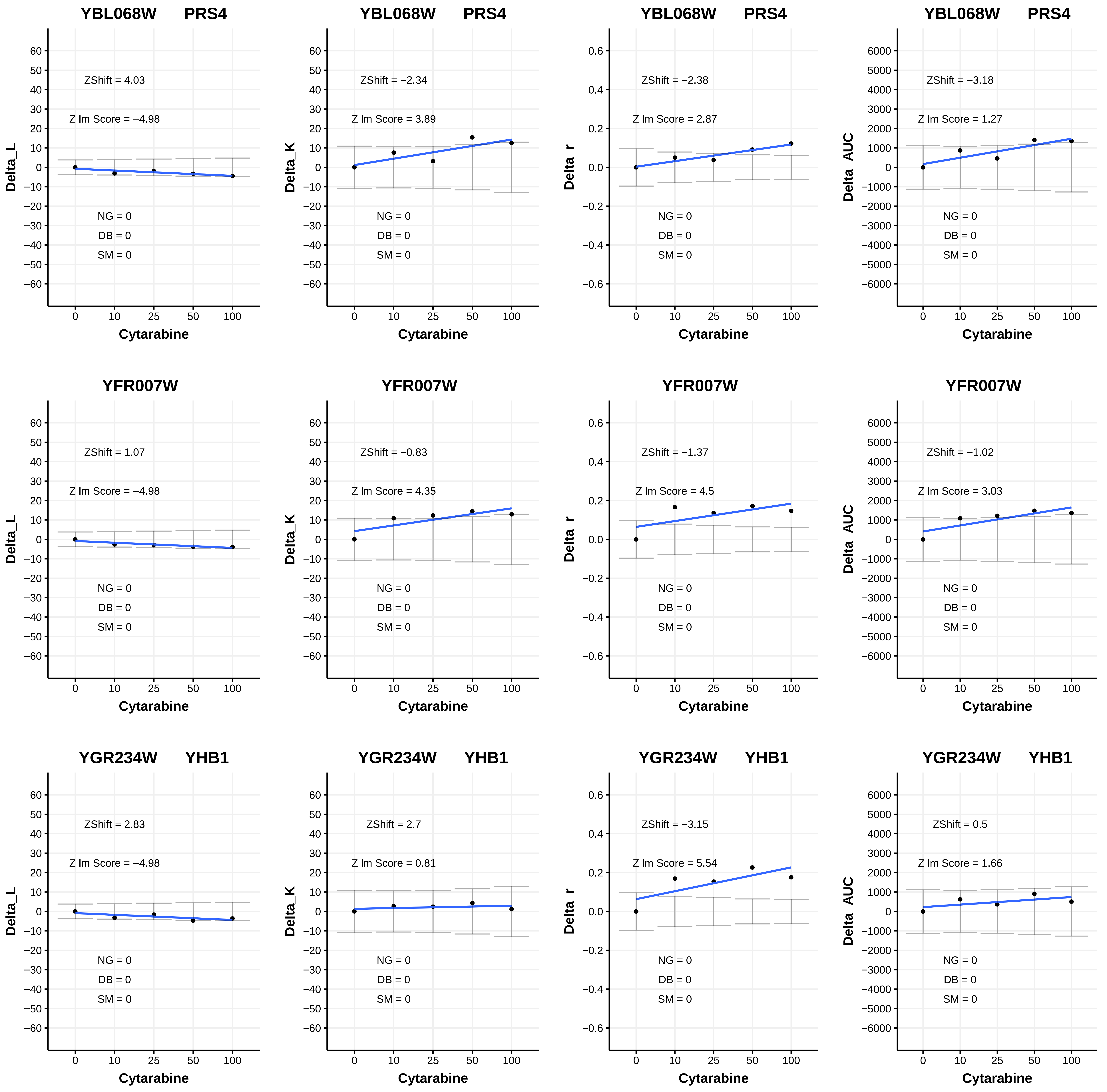

YBL068W PRS4
ZShift = 4.03
Z lm Score = −4.98
NG = 0
DB = 0
SM = 0
Delta_L
Cytarabine
YBL068W PRS4
ZShift = −2.34
Z lm Score = 3.89
NG = 0
DB = 0
SM = 0
Delta_K
Cytarabine
YBL068W PRS4
ZShift = −2.38
Z lm Score = 2.87
NG = 0
DB = 0
SM = 0
Delta_r
Cytarabine
YBL068W PRS4
ZShift = −3.18
Z lm Score = 1.27
NG = 0
DB = 0
SM = 0
Delta_AUC
Cytarabine
YFR007W
ZShift = 1.07
Z lm Score = −4.98
NG = 0
DB = 0
SM = 0
Delta_L
Cytarabine
YFR007W
ZShift = −0.83
Z lm Score = 4.35
NG = 0
DB = 0
SM = 0
Delta_K
Cytarabine
YFR007W
ZShift = −1.37
Z lm Score = 4.5
NG = 0
DB = 0
SM = 0
Delta_r
Cytarabine
YFR007W
ZShift = −1.02
Z lm Score = 3.03
NG = 0
DB = 0
SM = 0
Delta_AUC
Cytarabine
YGR234W YHB1
ZShift = 2.83
Z lm Score = −4.98
NG = 0
DB = 0
SM = 0
Delta_L
Cytarabine
YGR234W YHB1
ZShift = 2.7
Z lm Score = 0.81
NG = 0
DB = 0
SM = 0
Delta_K
Cytarabine
YGR234W YHB1
ZShift = −3.15
Z lm Score = 5.54
NG = 0
DB = 0
SM = 0
Delta_r
Cytarabine
YGR234W YHB1
ZShift = 0.5
Z lm Score = 1.66
NG = 0
DB = 0
SM = 0
Delta_AUC
Cytarabine

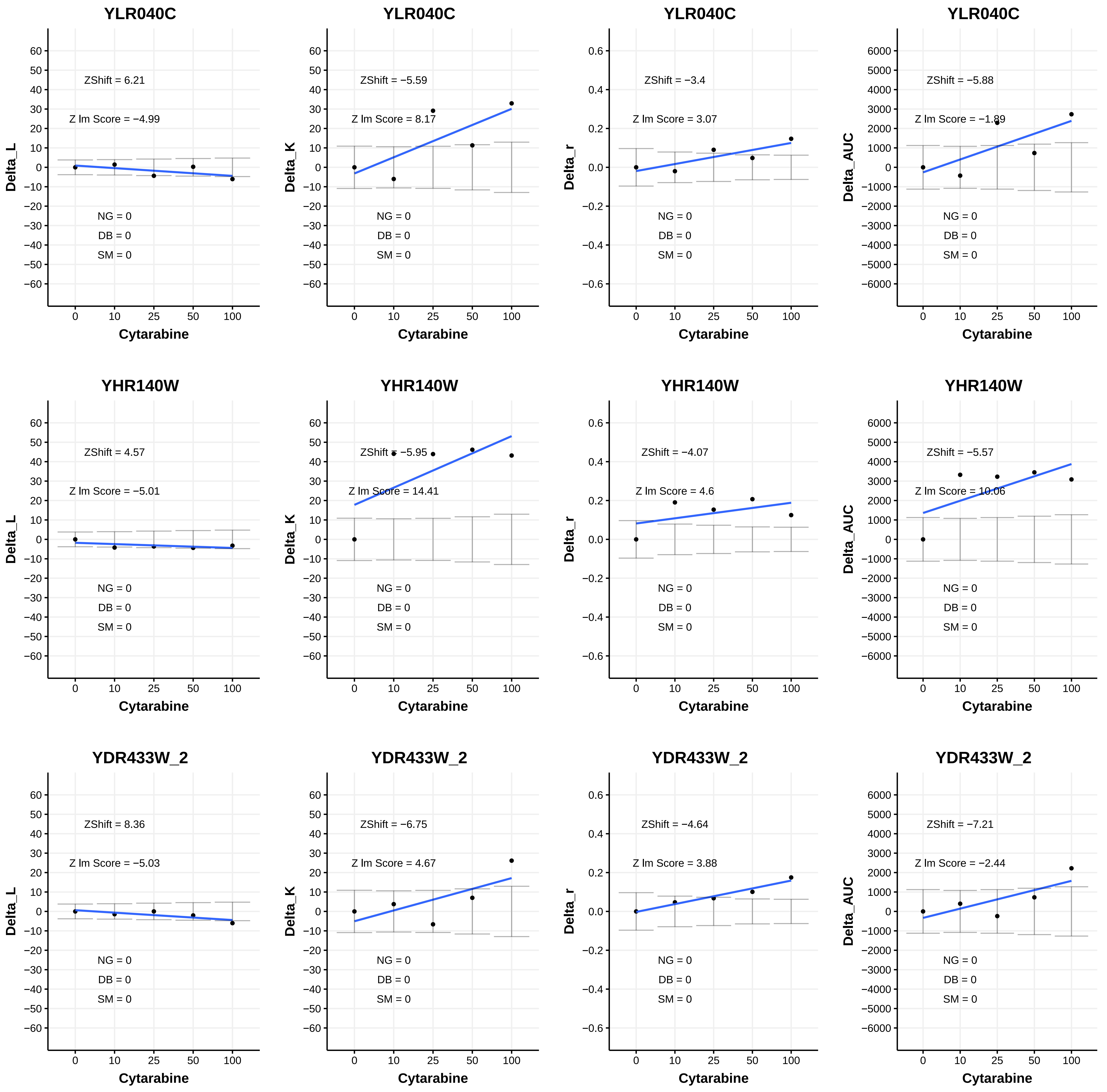
YLR040C
ZShift = 6.21
Z lm Score = −4.99
NG = 0
DB = 0
SM = 0
Delta_L
Cytarabine
YLR040C
ZShift = −5.59
Z lm Score = 8.17
NG = 0
DB = 0
SM = 0
Delta_K
Cytarabine
YLR040C
ZShift = −3.4
Z lm Score = 3.07
NG = 0
DB = 0
SM = 0
Delta_r
Cytarabine
YLR040C
ZShift = −5.88
Z lm Score = −1.89
NG = 0
DB = 0
SM = 0
Delta_AUC
Cytarabine
YHR140W
ZShift = 4.57
Z lm Score = −5.01
NG = 0
DB = 0
SM = 0
Delta_L
Cytarabine
YHR140W
ZShift = −5.95
Z lm Score = 14.41
NG = 0
DB = 0
SM = 0
Delta_K
Cytarabine
YHR140W
ZShift = −4.07
Z lm Score = 4.6
NG = 0
DB = 0
SM = 0
Delta_r
Cytarabine
YHR140W
ZShift = −5.57
Z lm Score = 10.06
NG = 0
DB = 0
SM = 0
Delta_AUC
Cytarabine
YDR433W_2
ZShift = 8.36
Z lm Score = −5.03
NG = 0
DB = 0
SM = 0
Delta_L
Cytarabine
YDR433W_2
ZShift = −6.75
Z lm Score = 4.67
NG = 0
DB = 0
SM = 0
Delta_K
Cytarabine
YDR433W_2
ZShift = −4.64
Z lm Score = 3.88
NG = 0
DB = 0
SM = 0
Delta_r
Cytarabine
YDR433W_2
ZShift = −7.21
Z lm Score = −2.44
NG = 0
DB = 0
SM = 0
Delta_AUC
Cytarabine

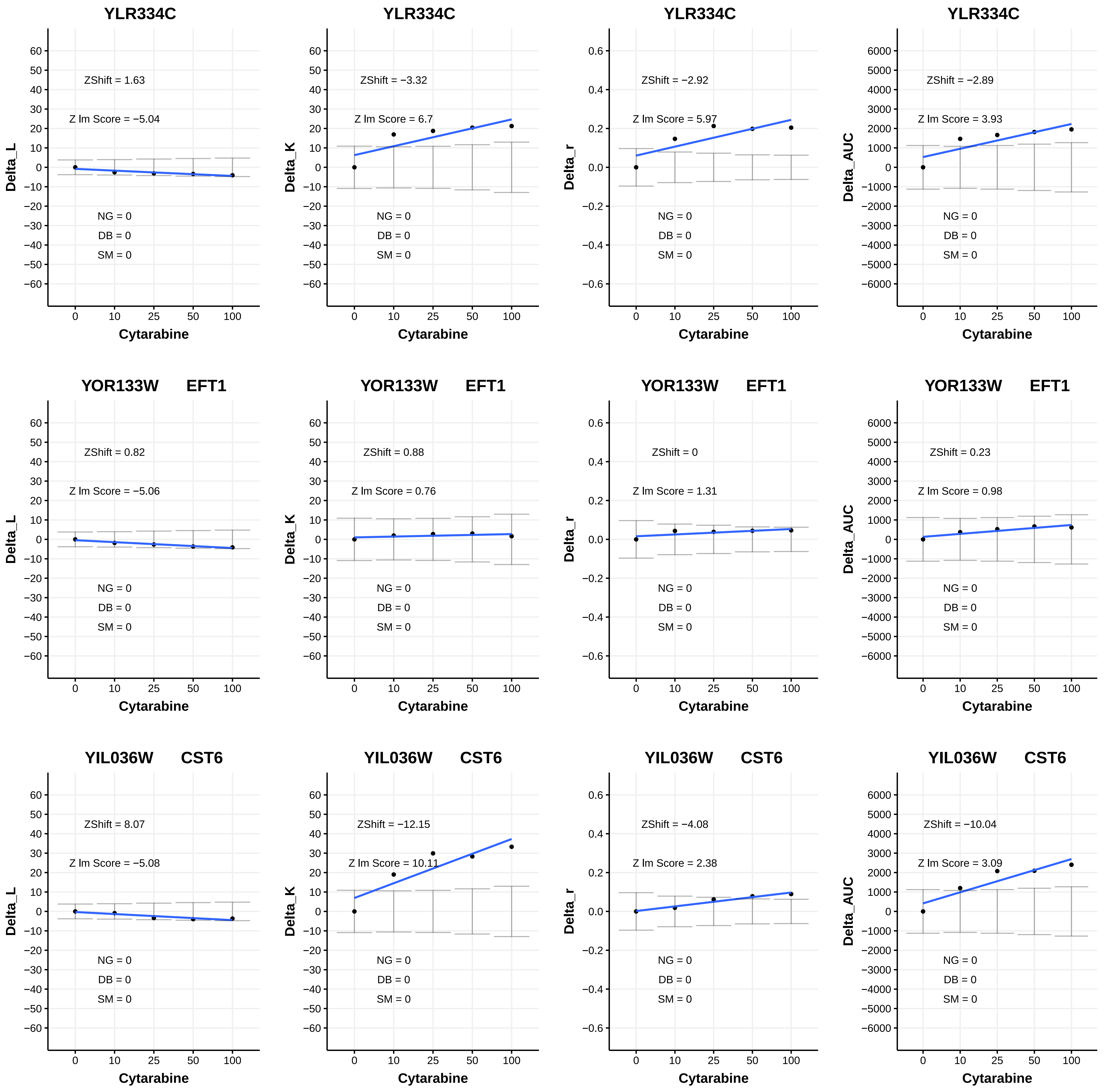
YLR334C
ZShift = 1.63
Z lm Score = −5.04
NG = 0
DB = 0
SM = 0
Delta_L
Cytarabine
YLR334C
ZShift = −3.32
Z lm Score = 6.7
NG = 0
DB = 0
SM = 0
Delta_K
Cytarabine
YLR334C
ZShift = −2.92
Z lm Score = 5.97
NG = 0
DB = 0
SM = 0
Delta_r
Cytarabine
YLR334C
ZShift = −2.89
Z lm Score = 3.93
NG = 0
DB = 0
SM = 0
Delta_AUC
Cytarabine
YOR133W EFT1
ZShift = 0.82
Z lm Score = −5.06
NG = 0
DB = 0
SM = 0
Delta_L
Cytarabine
YOR133W EFT1
ZShift = 0.88
Z lm Score = 0.76
NG = 0
DB = 0
SM = 0
Delta_K
Cytarabine
YOR133W EFT1
ZShift = 0
Z lm Score = 1.31
NG = 0
DB = 0
SM = 0
Delta_r
Cytarabine
YOR133W EFT1
ZShift = 0.23
Z lm Score = 0.98
NG = 0
DB = 0
SM = 0
Delta_AUC
Cytarabine
YIL036W CST6
ZShift = 8.07
Z lm Score = −5.08
NG = 0
DB = 0
SM = 0
Delta_L
Cytarabine
YIL036W CST6
ZShift = −12.15
Z lm Score = 10.11
NG = 0
DB = 0
SM = 0
Delta_K
Cytarabine
YIL036W CST6
ZShift = −4.08
Z lm Score = 2.38
NG = 0
DB = 0
SM = 0
Delta_r
Cytarabine
YIL036W CST6
ZShift = −10.04
Z lm Score = 3.09
NG = 0
DB = 0
SM = 0
Delta_AUC
Cytarabine
0
10
25
50
100

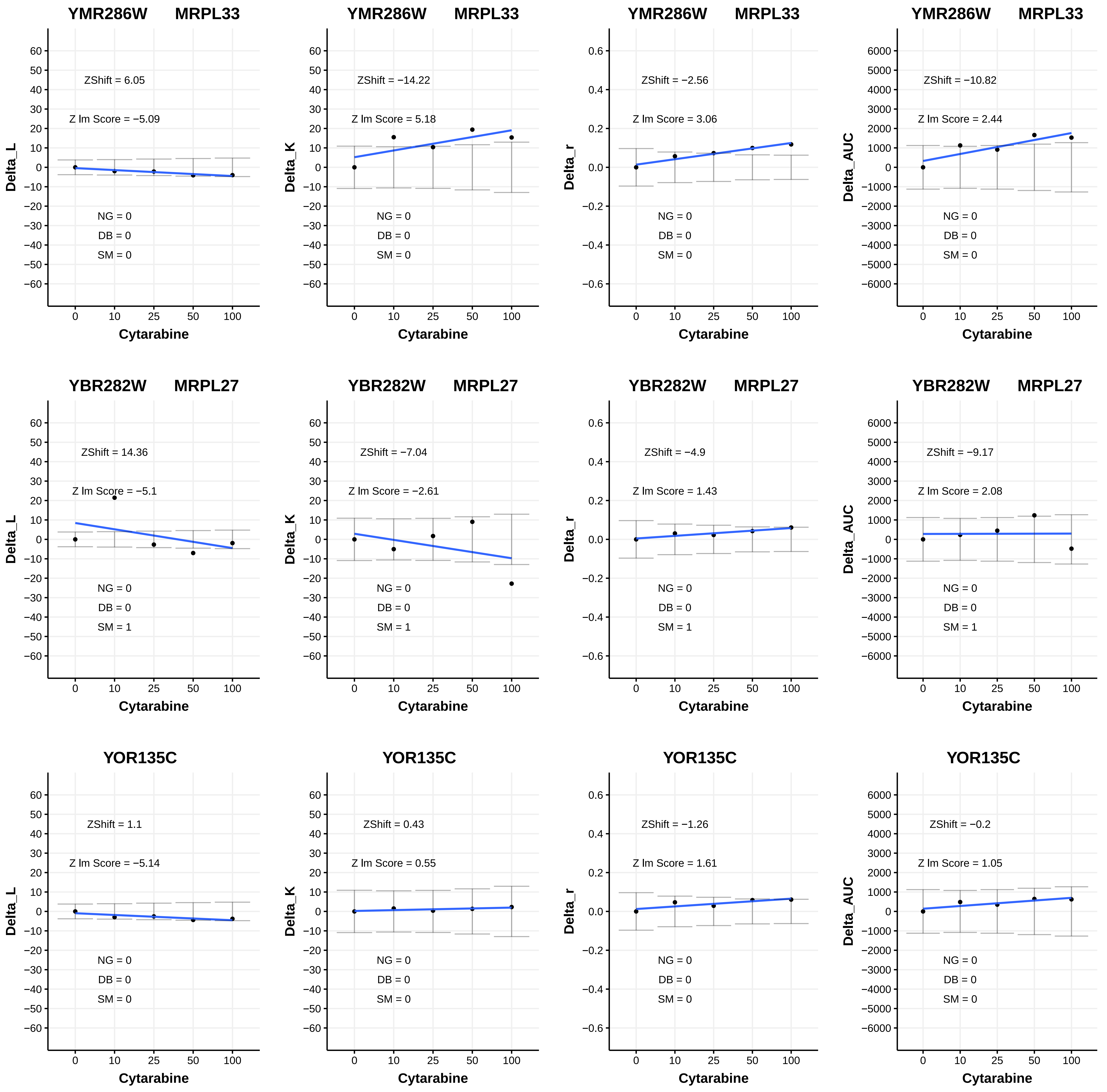

YMR286W MRPL33
ZShift = 6.05
Z lm Score = −5.09
NG = 0
DB = 0
SM = 0
Delta_L
Cytarabine
YMR286W MRPL33
ZShift = −14.22
Z lm Score = 5.18
NG = 0
DB = 0
SM = 0
Delta_K
Cytarabine
YMR286W MRPL33
ZShift = −2.56
Z lm Score = 3.06
NG = 0
DB = 0
SM = 0
Delta_r
Cytarabine
YMR286W MRPL33
ZShift = −10.82
Z lm Score = 2.44
NG = 0
DB = 0
SM = 0
Delta_AUC
Cytarabine
YBR282W MRPL27
ZShift = 14.36
Z lm Score = −5.1
NG = 0
DB = 0
SM = 1
Delta_L
Cytarabine
YBR282W MRPL27
ZShift = −7.04
Z lm Score = −2.61
NG = 0
DB = 0
SM = 1
Delta_K
Cytarabine
YBR282W MRPL27
ZShift = −4.9
Z lm Score = 1.43
NG = 0
DB = 0
SM = 1
Delta_r
Cytarabine
YBR282W MRPL27
ZShift = −9.17
Z lm Score = 2.08
NG = 0
DB = 0
SM = 1
Delta_AUC
Cytarabine
YOR135C
ZShift = 1.1
Z lm Score = −5.14
NG = 0
DB = 0
SM = 0
Delta_L
Cytarabine
YOR135C
ZShift = 0.43
Z lm Score = 0.55
NG = 0
DB = 0
SM = 0
Delta_K
Cytarabine
YOR135C
ZShift = −1.26
Z lm Score = 1.61
NG = 0
DB = 0
SM = 0
Delta_r
Cytarabine
YOR135C
ZShift = −0.2
Z lm Score = 1.05
NG = 0
DB = 0
SM = 0
Delta_AUC
Cytarabine

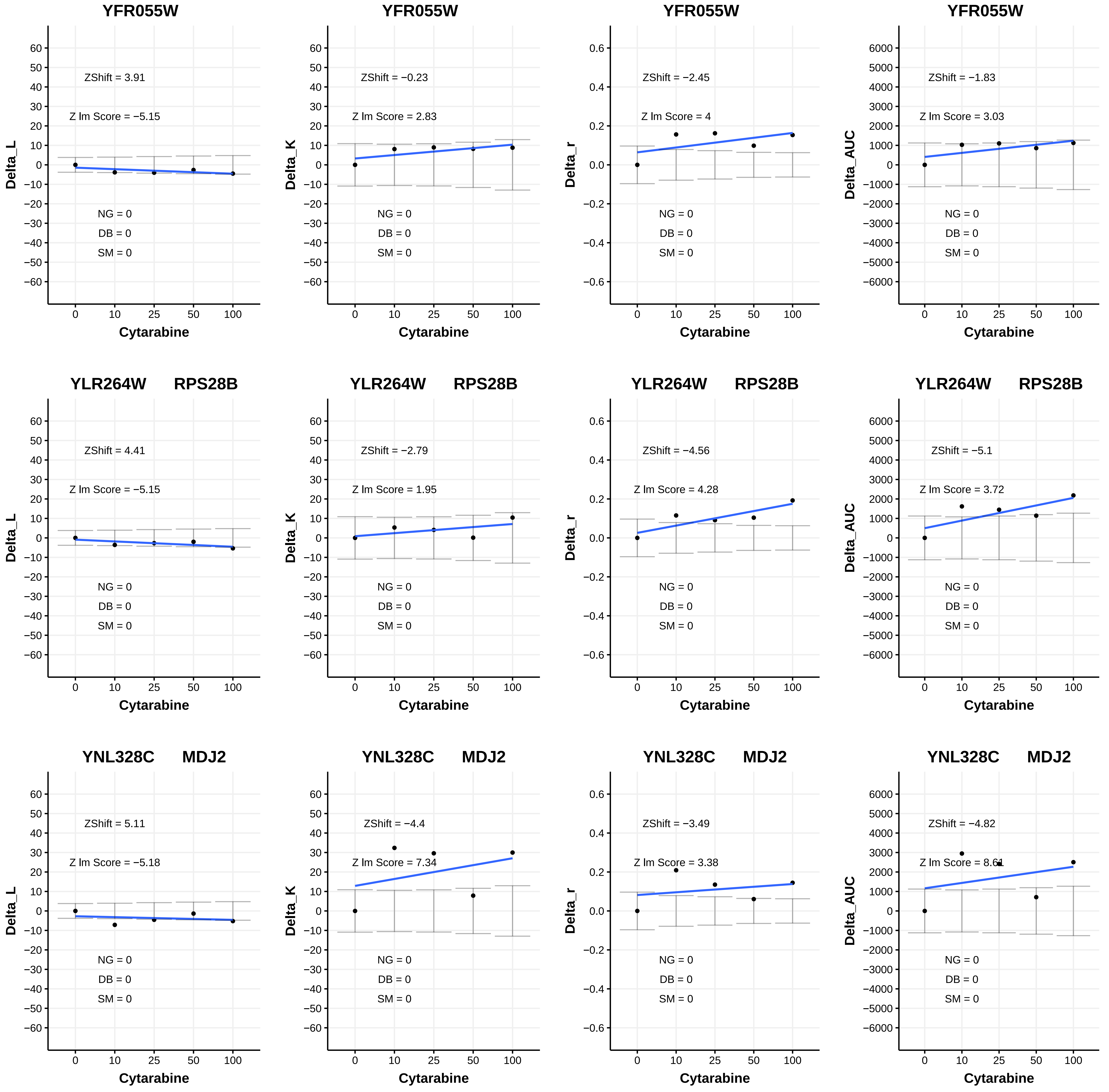

YFR055W
ZShift = 3.91
Z lm Score = −5.15
NG = 0
DB = 0
SM = 0
Delta_L
Cytarabine
YFR055W
ZShift = −0.23
Z lm Score = 2.83
NG = 0
DB = 0
SM = 0
Delta_K
Cytarabine
YFR055W
ZShift = −2.45
Z lm Score = 4
NG = 0
DB = 0
SM = 0
Delta_r
Cytarabine
YFR055W
ZShift = −1.83
Z lm Score = 3.03
NG = 0
DB = 0
SM = 0
Delta_AUC
Cytarabine
YLR264W RPS28B
ZShift = 4.41
Z lm Score = −5.15
NG = 0
DB = 0
SM = 0
Delta_L
Cytarabine
YLR264W RPS28B
ZShift = −2.79
Z lm Score = 1.95
NG = 0
DB = 0
SM = 0
Delta_K
Cytarabine
YLR264W RPS28B
ZShift = −4.56
Z lm Score = 4.28
NG = 0
DB = 0
SM = 0
Delta_r
Cytarabine
YLR264W RPS28B
ZShift = −5.1
Z lm Score = 3.72
NG = 0
DB = 0
SM = 0
Delta_AUC
Cytarabine
YNL328C MDJ2
ZShift = 5.11
Z lm Score = −5.18
NG = 0
DB = 0
SM = 0
Delta_L
Cytarabine
YNL328C MDJ2
ZShift = −4.4
Z lm Score = 7.34
NG = 0
DB = 0
SM = 0
Delta_K
Cytarabine
YNL328C MDJ2
ZShift = −3.49
Z lm Score = 3.38
NG = 0
DB = 0
SM = 0
Delta_r
Cytarabine
YNL328C MDJ2
ZShift = −4.82
Z lm Score = 8.61
NG = 0
DB = 0
SM = 0
Delta_AUC
Cytarabine

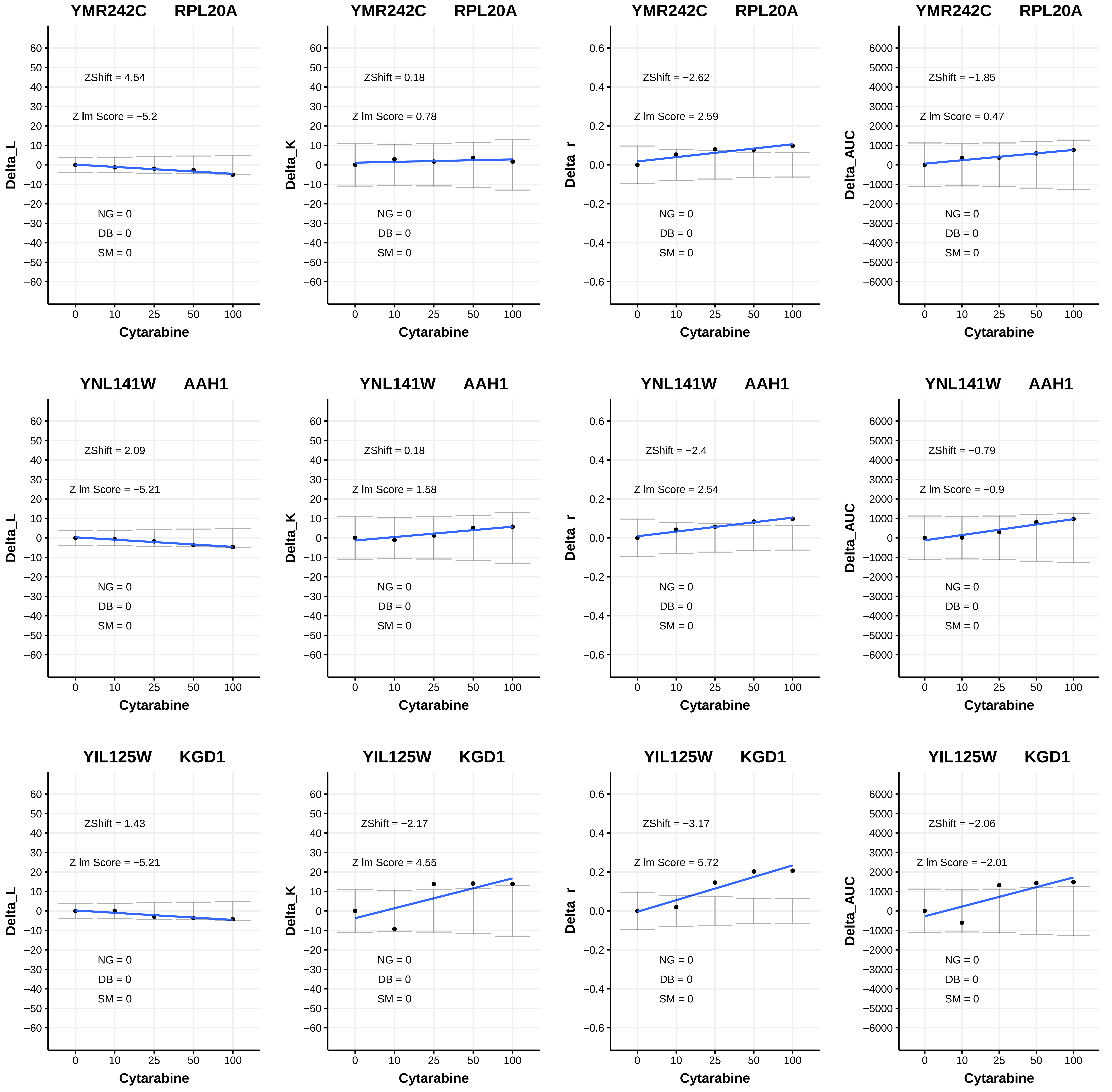
YMR242C RPL20A
ZShift = 4.54
Z lm Score = −5.2
NG = 0
DB = 0
SM = 0
Delta_L
Cytarabine
YMR242C RPL20A
ZShift = 0.18
Z lm Score = 0.78
NG = 0
DB = 0
SM = 0
Delta_K
Cytarabine
YMR242C RPL20A
ZShift = −2.62
Z lm Score = 2.59
NG = 0
DB = 0
SM = 0
Delta_r
Cytarabine
YMR242C RPL20A
ZShift = −1.85
Z lm Score = 0.47
NG = 0
DB = 0
SM = 0
Delta_AUC
Cytarabine
YNL141W AAH1
ZShift = 2.09
Z lm Score = −5.21
NG = 0
DB = 0
SM = 0
Delta_L
Cytarabine
YNL141W AAH1
ZShift = 0.18
Z lm Score = 1.58
NG = 0
DB = 0
SM = 0
Delta_K
Cytarabine
YNL141W AAH1
ZShift = −2.4
Z lm Score = 2.54
NG = 0
DB = 0
SM = 0
Delta_r
Cytarabine
YNL141W AAH1
ZShift = −0.79
Z lm Score = −0.9
NG = 0
DB = 0
SM = 0
Delta_AUC
Cytarabine
YIL125W KGD1
ZShift = 1.43
Z lm Score = −5.21
NG = 0
DB = 0
SM = 0
Delta_L
Cytarabine
YIL125W KGD1
ZShift = −2.17
Z lm Score = 4.55
NG = 0
DB = 0
SM = 0
Delta_K
Cytarabine
YIL125W KGD1
ZShift = −3.17
Z lm Score = 5.72
NG = 0
DB = 0
SM = 0
Delta_r
Cytarabine
YIL125W KGD1
ZShift = −2.06
Z lm Score = −2.01
NG = 0
DB = 0
SM = 0
Delta_AUC
Cytarabine

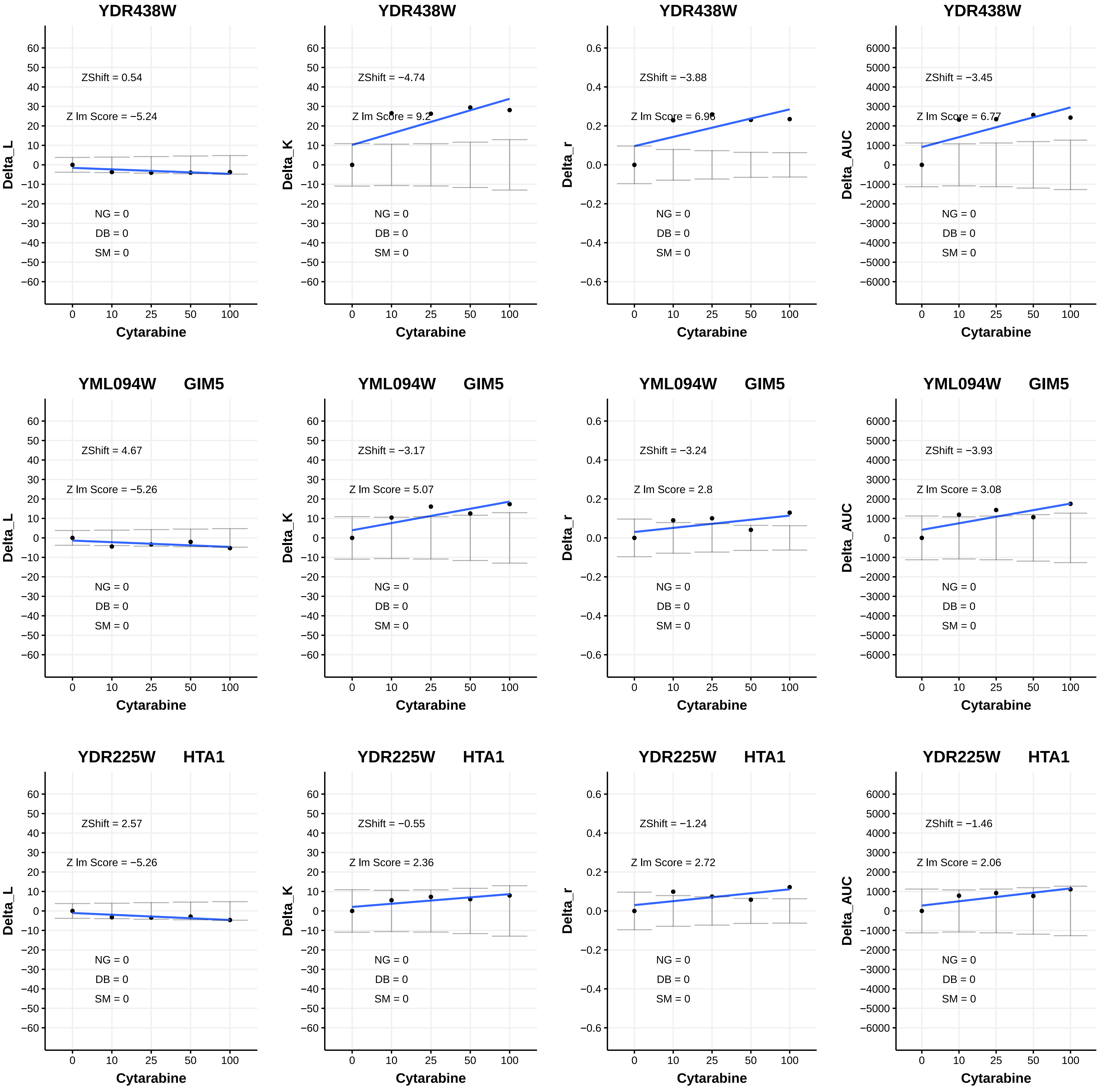
YDR438W
ZShift = 0.54
Z lm Score = −5.24
NG = 0
DB = 0
SM = 0
Delta_L
Cytarabine
YDR438W
ZShift = −4.74
Z lm Score = 9.2
NG = 0
DB = 0
SM = 0
Delta_K
Cytarabine
YDR438W
ZShift = −3.88
Z lm Score = 6.96
NG = 0
DB = 0
SM = 0
Delta_r
Cytarabine
YDR438W
ZShift = −3.45
Z lm Score = 6.77
NG = 0
DB = 0
SM = 0
Delta_AUC
Cytarabine
YML094W GIM5
ZShift = 4.67
Z lm Score = −5.26
NG = 0
DB = 0
SM = 0
Delta_L
Cytarabine
YML094W GIM5
ZShift = −3.17
Z lm Score = 5.07
NG = 0
DB = 0
SM = 0
Delta_K
Cytarabine
YML094W GIM5
ZShift = −3.24
Z lm Score = 2.8
NG = 0
DB = 0
SM = 0
Delta_r
Cytarabine
YML094W GIM5
ZShift = −3.93
Z lm Score = 3.08
NG = 0
DB = 0
SM = 0
Delta_AUC
Cytarabine
YDR225W HTA1
ZShift = 2.57
Z lm Score = −5.26
NG = 0
DB = 0
SM = 0
Delta_L
Cytarabine
YDR225W HTA1
ZShift = −0.55
Z lm Score = 2.36
NG = 0
DB = 0
SM = 0
Delta_K
Cytarabine
YDR225W HTA1
ZShift = −1.24
Z lm Score = 2.72
NG = 0
DB = 0
SM = 0
Delta_r
Cytarabine
YDR225W HTA1
ZShift = −1.46
Z lm Score = 2.06
NG = 0
DB = 0
SM = 0
Delta_AUC
Cytarabine

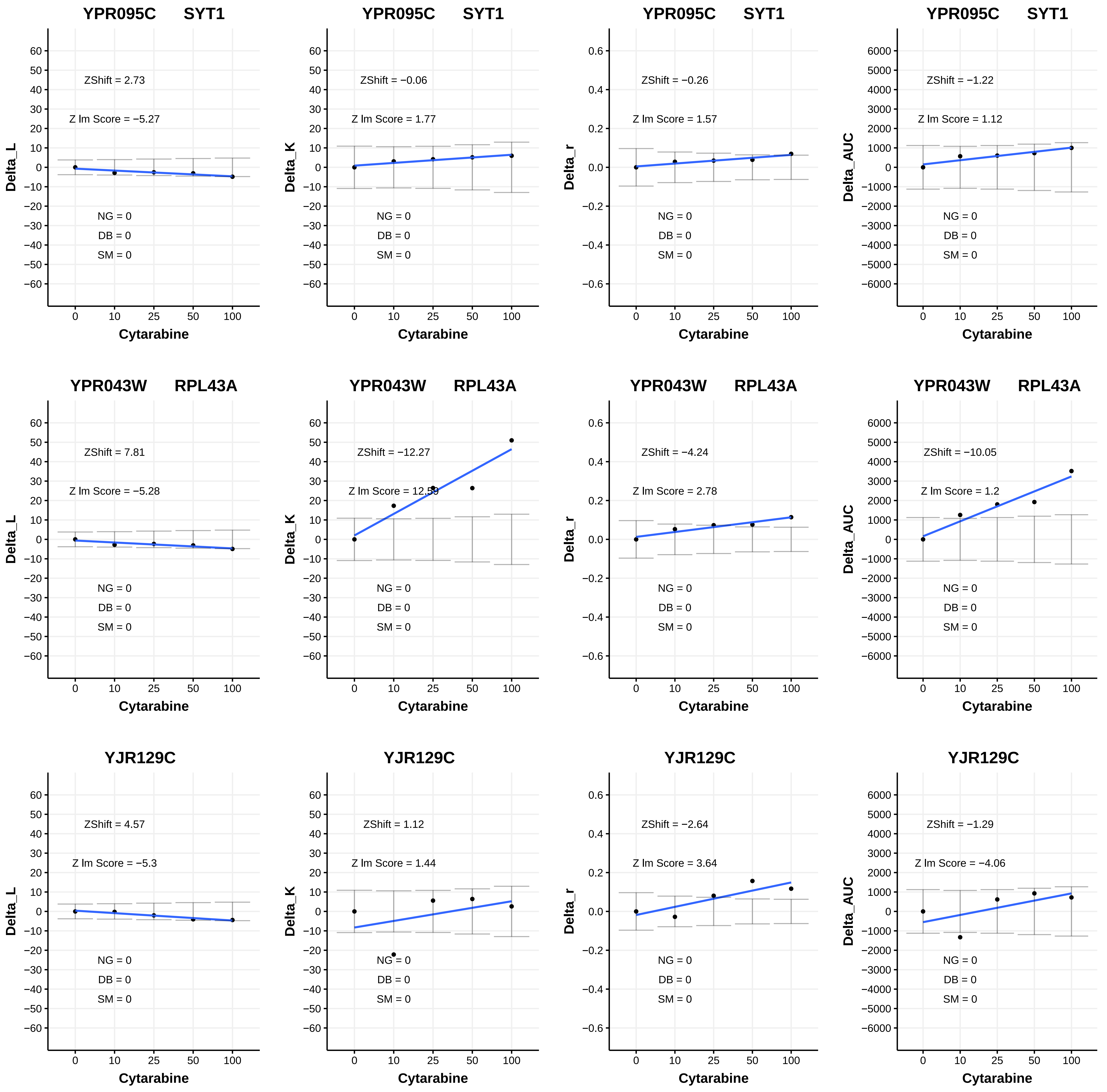

YPR095C SYT1
ZShift = 2.73
Z lm Score = −5.27
NG = 0
DB = 0
SM = 0
Delta_L
Cytarabine
YPR095C SYT1
ZShift = −0.06
Z lm Score = 1.77
NG = 0
DB = 0
SM = 0
Delta_K
Cytarabine
YPR095C SYT1
ZShift = −0.26
Z lm Score = 1.57
NG = 0
DB = 0
SM = 0
Delta_r
Cytarabine
YPR095C SYT1
ZShift = −1.22
Z lm Score = 1.12
NG = 0
DB = 0
SM = 0
Delta_AUC
Cytarabine
YPR043W RPL43A
ZShift = 7.81
Z lm Score = −5.28
NG = 0
DB = 0
SM = 0
Delta_L
Cytarabine
YPR043W RPL43A
ZShift = −12.27
Z lm Score = 12.59
NG = 0
DB = 0
SM = 0
Delta_K
Cytarabine
YPR043W RPL43A
ZShift = −4.24
Z lm Score = 2.78
NG = 0
DB = 0
SM = 0
Delta_r
Cytarabine
YPR043W RPL43A
ZShift = −10.05
Z lm Score = 1.2
NG = 0
DB = 0
SM = 0
Delta_AUC
Cytarabine
YJR129C
ZShift = 4.57
Z lm Score = −5.3
NG = 0
DB = 0
SM = 0
Delta_L
Cytarabine
YJR129C
ZShift = 1.12
Z lm Score = 1.44
NG = 0
DB = 0
SM = 0
Delta_K
Cytarabine
YJR129C
ZShift = −2.64
Z lm Score = 3.64
NG = 0
DB = 0
SM = 0
Delta_r
Cytarabine
YJR129C
ZShift = −1.29
Z lm Score = −4.06
NG = 0
DB = 0
SM = 0
Delta_AUC
Cytarabine

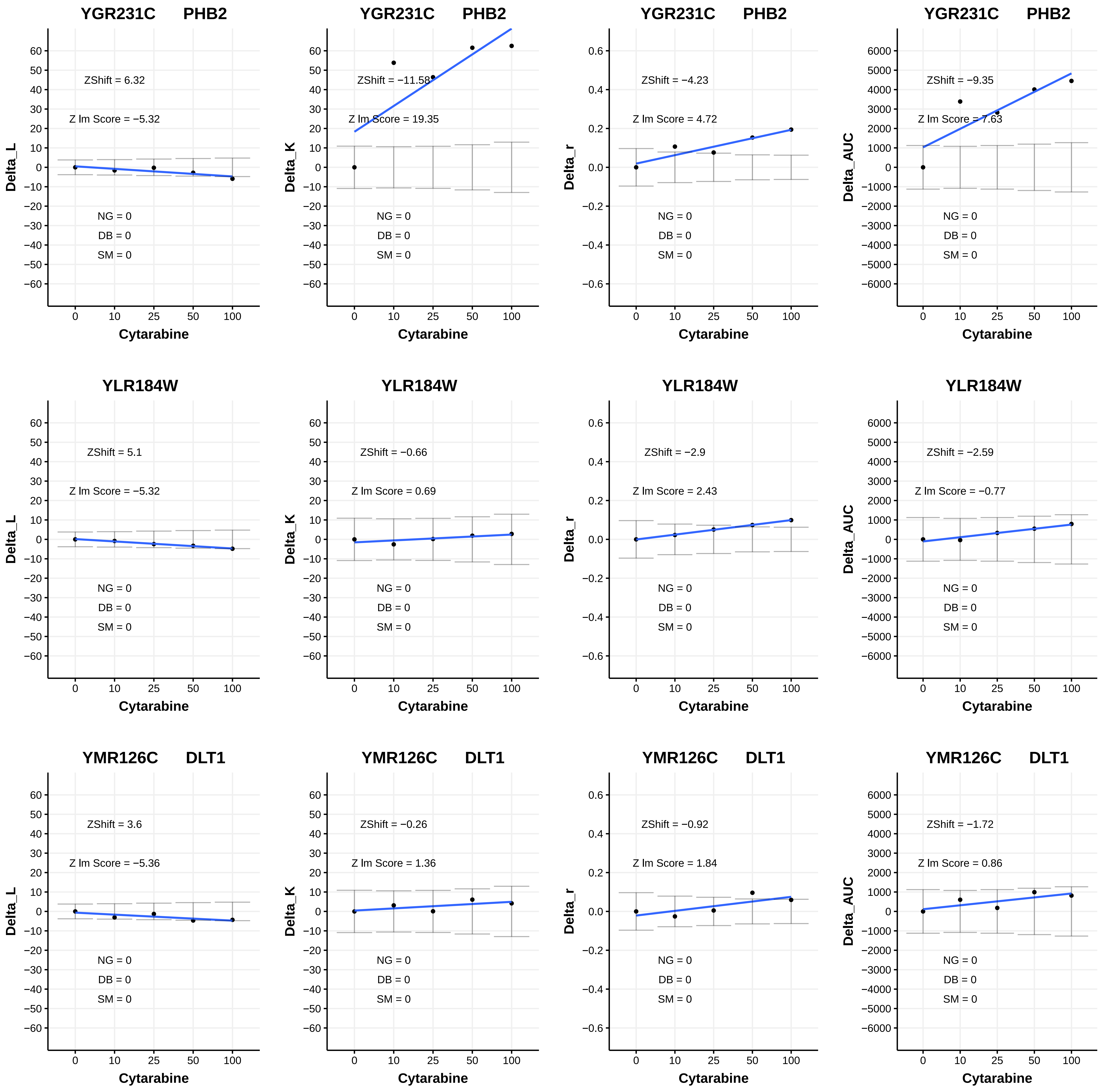

YGR231C PHB2
ZShift = 6.32
Z lm Score = −5.32
NG = 0
DB = 0
SM = 0
Delta_L
Cytarabine
YGR231C PHB2
ZShift = −11.58
Z lm Score = 19.35
NG = 0
DB = 0
SM = 0
Delta_K
Cytarabine
YGR231C PHB2
ZShift = −4.23
Z lm Score = 4.72
NG = 0
DB = 0
SM = 0
Delta_r
Cytarabine
YGR231C PHB2
ZShift = −9.35
Z lm Score = 7.63
NG = 0
DB = 0
SM = 0
Delta_AUC
Cytarabine
YLR184W
ZShift = 5.1
Z lm Score = −5.32
NG = 0
DB = 0
SM = 0
Delta_L
Cytarabine
YLR184W
ZShift = −0.66
Z lm Score = 0.69
NG = 0
DB = 0
SM = 0
Delta_K
Cytarabine
YLR184W
ZShift = −2.9
Z lm Score = 2.43
NG = 0
DB = 0
SM = 0
Delta_r
Cytarabine
YLR184W
ZShift = −2.59
Z lm Score = −0.77
NG = 0
DB = 0
SM = 0
Delta_AUC
Cytarabine
YMR126C DLT1
ZShift = 3.6
Z lm Score = −5.36
NG = 0
DB = 0
SM = 0
Delta_L
Cytarabine
YMR126C DLT1
ZShift = −0.26
Z lm Score = 1.36
NG = 0
DB = 0
SM = 0
Delta_K
Cytarabine
YMR126C DLT1
ZShift = −0.92
Z lm Score = 1.84
NG = 0
DB = 0
SM = 0
Delta_r
Cytarabine
YMR126C DLT1
ZShift = −1.72
Z lm Score = 0.86
NG = 0
DB = 0
SM = 0
Delta_AUC
Cytarabine

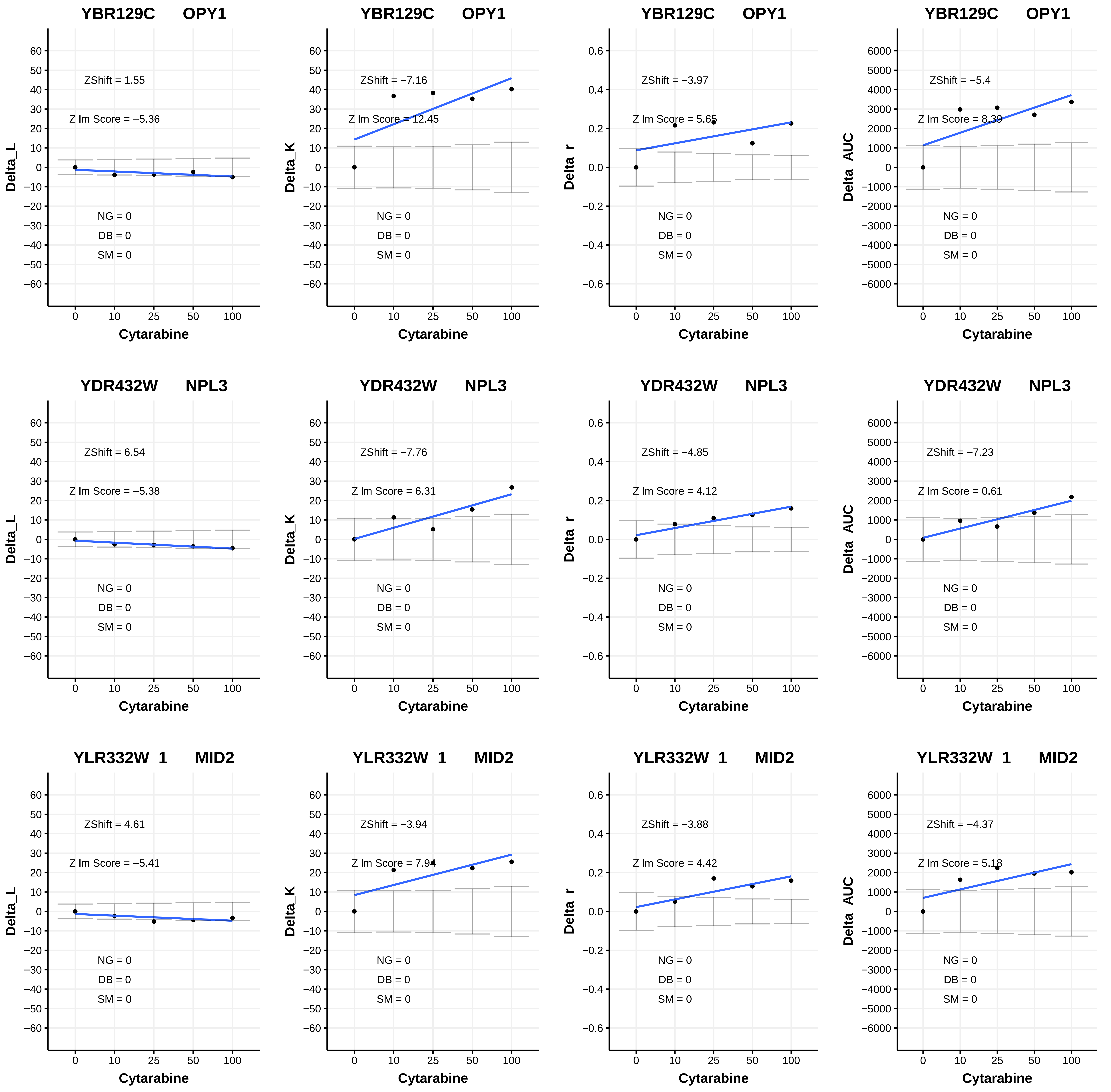
YBR129C OPY1
ZShift = 1.55
Z lm Score = −5.36
NG = 0
DB = 0
SM = 0
Delta_L
Cytarabine
YBR129C OPY1
ZShift = −7.16
Z lm Score = 12.45
NG = 0
DB = 0
SM = 0
Delta_K
Cytarabine
YBR129C OPY1
ZShift = −3.97
Z lm Score = 5.65
NG = 0
DB = 0
SM = 0
Delta_r
Cytarabine
YBR129C OPY1
ZShift = −5.4
Z lm Score = 8.39
NG = 0
DB = 0
SM = 0
Delta_AUC
Cytarabine
YDR432W NPL3
ZShift = 6.54
Z lm Score = −5.38
NG = 0
DB = 0
SM = 0
Delta_L
Cytarabine
YDR432W NPL3
ZShift = −7.76
Z lm Score = 6.31
NG = 0
DB = 0
SM = 0
Delta_K
Cytarabine
YDR432W NPL3
ZShift = −4.85
Z lm Score = 4.12
NG = 0
DB = 0
SM = 0
Delta_r
Cytarabine
YDR432W NPL3
ZShift = −7.23
Z lm Score = 0.61
NG = 0
DB = 0
SM = 0
Delta_AUC
Cytarabine
YLR332W_1 MID2
ZShift = 4.61
Z lm Score = −5.41
NG = 0
DB = 0
SM = 0
Delta_L
Cytarabine
YLR332W_1 MID2
ZShift = −3.94
Z lm Score = 7.94
NG = 0
DB = 0
SM = 0
Delta_K
Cytarabine
YLR332W_1 MID2
ZShift = −3.88
Z lm Score = 4.42
NG = 0
DB = 0
SM = 0
Delta_r
Cytarabine
YLR332W_1 MID2
ZShift = −4.37
Z lm Score = 5.18
NG = 0
DB = 0
SM = 0
Delta_AUC
Cytarabine

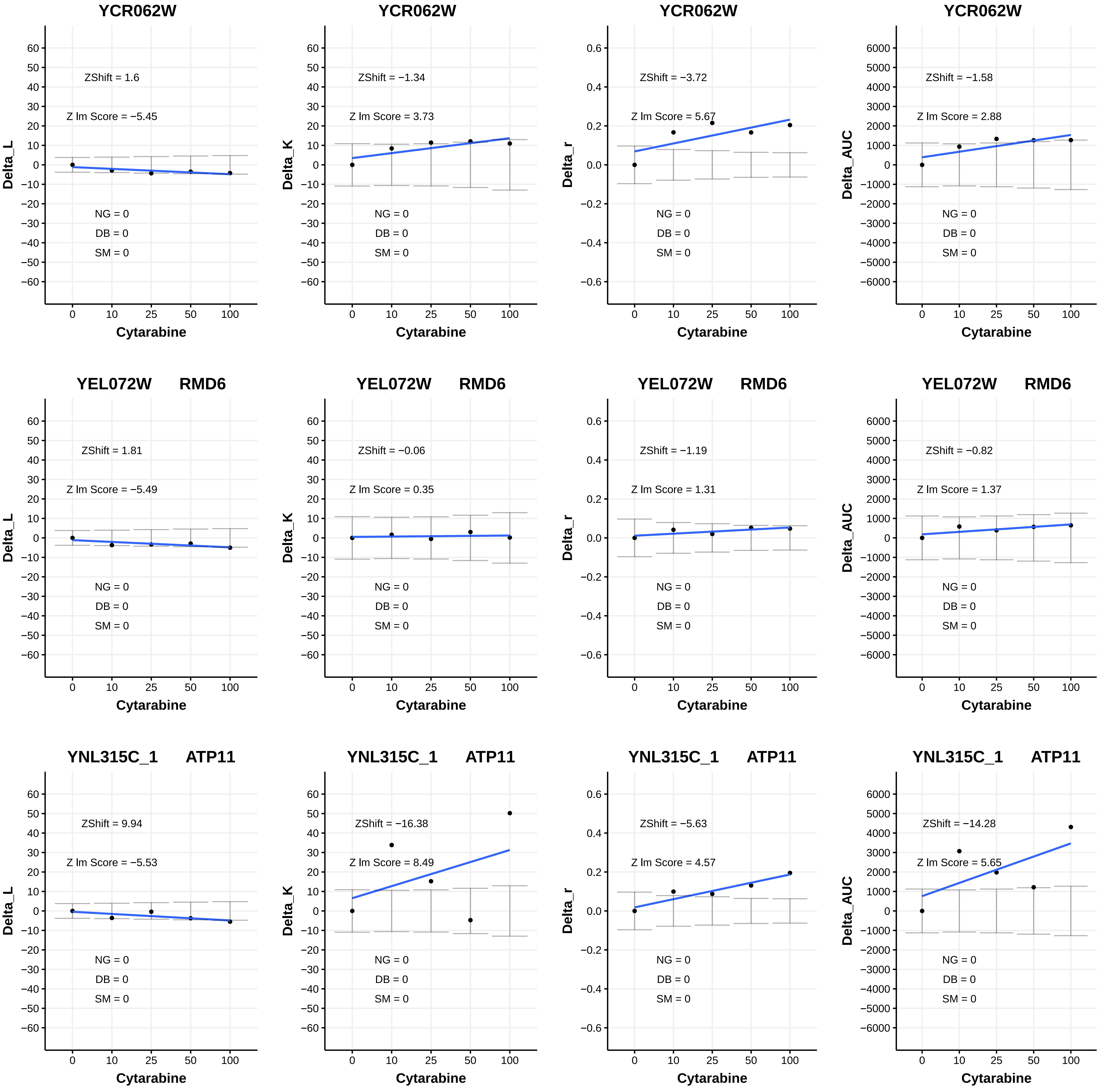
YCR062W
ZShift = 1.6
Z lm Score = −5.45
NG = 0
DB = 0
SM = 0
Delta_L
Cytarabine
YCR062W
ZShift = −1.34
Z lm Score = 3.73
NG = 0
DB = 0
SM = 0
Delta_K
Cytarabine
YCR062W
ZShift = −3.72
Z lm Score = 5.67
NG = 0
DB = 0
SM = 0
Delta_r
Cytarabine
YCR062W
ZShift = −1.58
Z lm Score = 2.88
NG = 0
DB = 0
SM = 0
Delta_AUC
Cytarabine
YEL072W RMD6
ZShift = 1.81
Z lm Score = −5.49
NG = 0
DB = 0
SM = 0
Delta_L
Cytarabine
YEL072W RMD6
ZShift = −0.06
Z lm Score = 0.35
NG = 0
DB = 0
SM = 0
Delta_K
Cytarabine
YEL072W RMD6
ZShift = −1.19
Z lm Score = 1.31
NG = 0
DB = 0
SM = 0
Delta_r
Cytarabine
YEL072W RMD6
ZShift = −0.82
Z lm Score = 1.37
NG = 0
DB = 0
SM = 0
Delta_AUC
Cytarabine
YNL315C_1 ATP11
ZShift = 9.94
Z lm Score = −5.53
NG = 0
DB = 0
SM = 0
Delta_L
Cytarabine
YNL315C_1 ATP11
ZShift = −16.38
Z lm Score = 8.49
NG = 0
DB = 0
SM = 0
Delta_K
Cytarabine
YNL315C_1 ATP11
ZShift = −5.63
Z lm Score = 4.57
NG = 0
DB = 0
SM = 0
Delta_r
Cytarabine
YNL315C_1 ATP11
ZShift = −14.28
Z lm Score = 5.65
NG = 0
DB = 0
SM = 0
Delta_AUC
Cytarabine

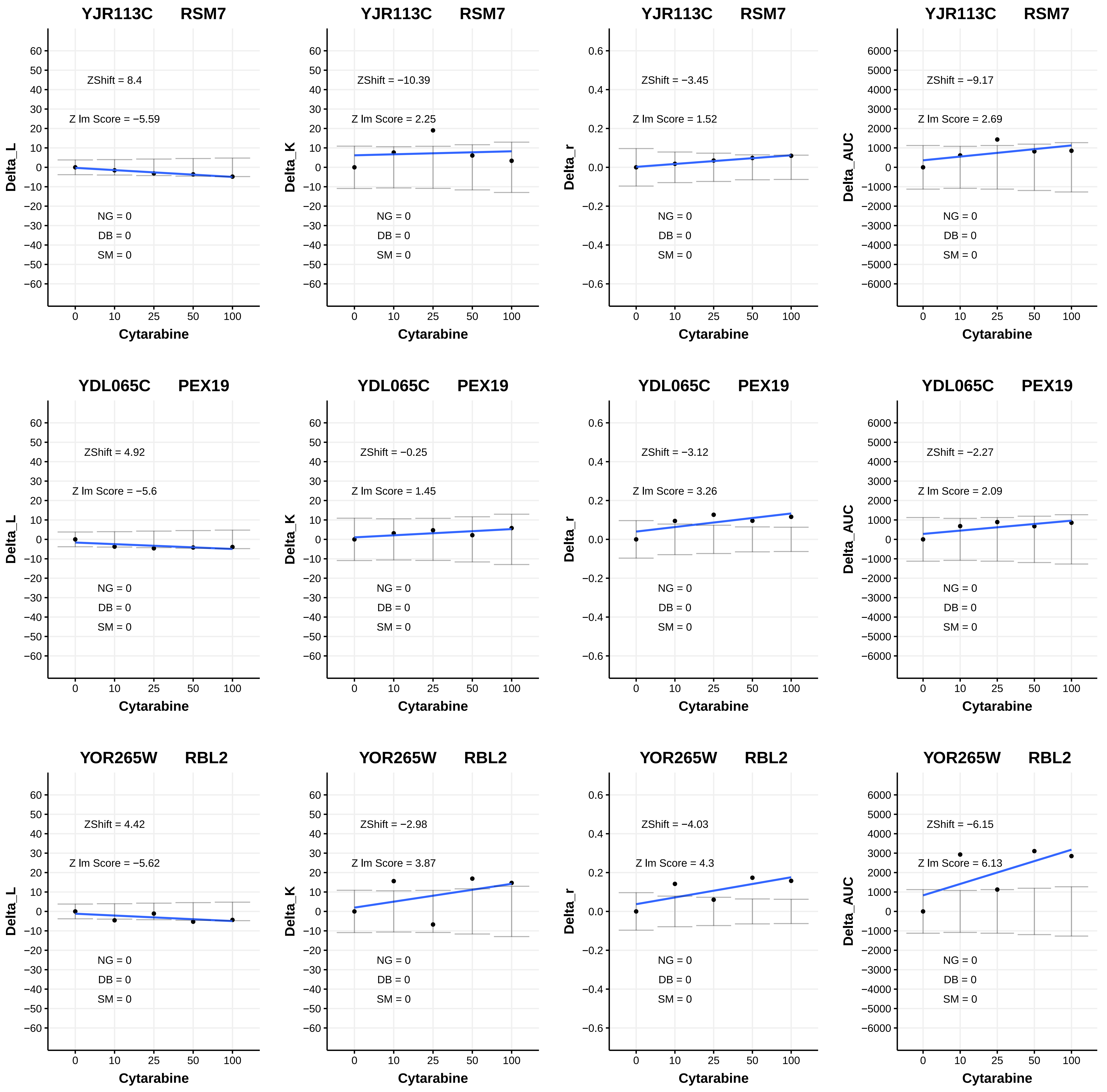

YJR113C RSM7
ZShift = −3.45
Z lm Score = 1.52
ZShift = 8.4
Z lm Score = −5.59
ZShift = −10.39
Z lm Score = 2.25
ZShift = −9.17
Z lm Score = 2.69
YDL065C PEX19
ZShift = 4.92
Z lm Score = −5.6
ZShift = −0.25
Z lm Score = 1.45
ZShift = −3.12
Z lm Score = 3.26
ZShift = −2.27
Z lm Score = 2.09
YOR265W RBL2
ZShift = 4.42
Z lm Score = −5.62
ZShift = −2.98
Z lm Score = 3.87
ZShift = −4.03
Z lm Score = 4.3
ZShift = −6.15
Z lm Score = 6.13
NG = 0
DB = 0
SM = 0
Delta_L
Delta_K
Delta_r
Delta_AUC
Cytarabine
0
10
25
50
100

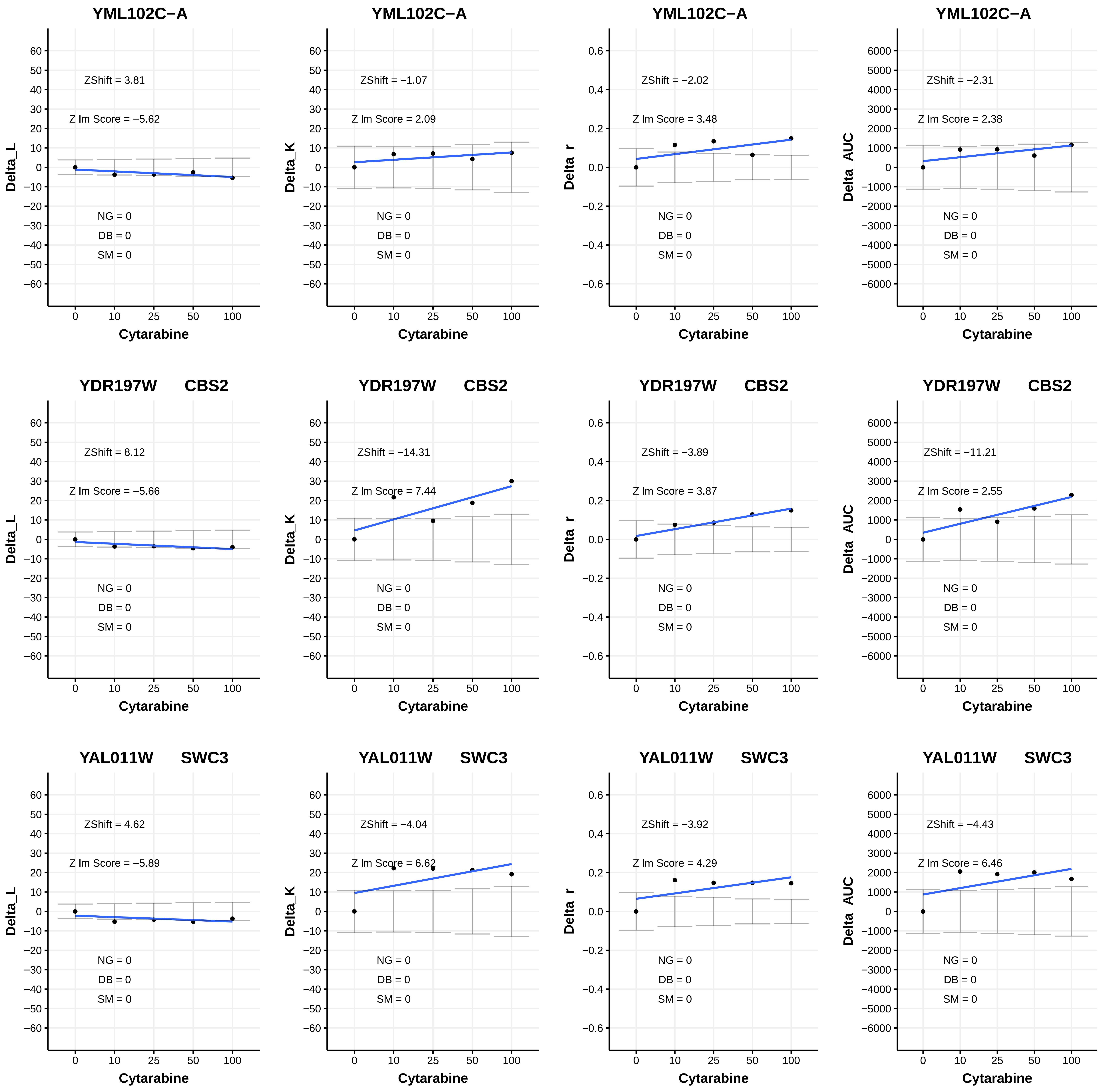
YML102C−A
ZShift = 3.81
Z lm Score = −5.62
NG = 0
DB = 0
SM = 0
Delta_L
Cytarabine
YML102C−A
ZShift = −1.07
Z lm Score = 2.09
NG = 0
DB = 0
SM = 0
Delta_K
Cytarabine
YML102C−A
ZShift = −2.02
Z lm Score = 3.48
NG = 0
DB = 0
SM = 0
Delta_r
Cytarabine
YML102C−A
ZShift = −2.31
Z lm Score = 2.38
NG = 0
DB = 0
SM = 0
Delta_AUC
Cytarabine
YDR197W CBS2
ZShift = 8.12
Z lm Score = −5.66
NG = 0
DB = 0
SM = 0
Delta_L
Cytarabine
YDR197W CBS2
ZShift = −14.31
Z lm Score = 7.44
NG = 0
DB = 0
SM = 0
Delta_K
Cytarabine
YDR197W CBS2
ZShift = −3.89
Z lm Score = 3.87
NG = 0
DB = 0
SM = 0
Delta_r
Cytarabine
YDR197W CBS2
ZShift = −11.21
Z lm Score = 2.55
NG = 0
DB = 0
SM = 0
Delta_AUC
Cytarabine
YAL011W SWC3
ZShift = 4.62
Z lm Score = −5.89
NG = 0
DB = 0
SM = 0
Delta_L
Cytarabine
YAL011W SWC3
ZShift = −4.04
Z lm Score = 6.62
NG = 0
DB = 0
SM = 0
Delta_K
Cytarabine
YAL011W SWC3
ZShift = −3.92
Z lm Score = 4.29
NG = 0
DB = 0
SM = 0
Delta_r
Cytarabine
YAL011W SWC3
ZShift = −4.43
Z lm Score = 6.46
NG = 0
DB = 0
SM = 0
Delta_AUC
Cytarabine

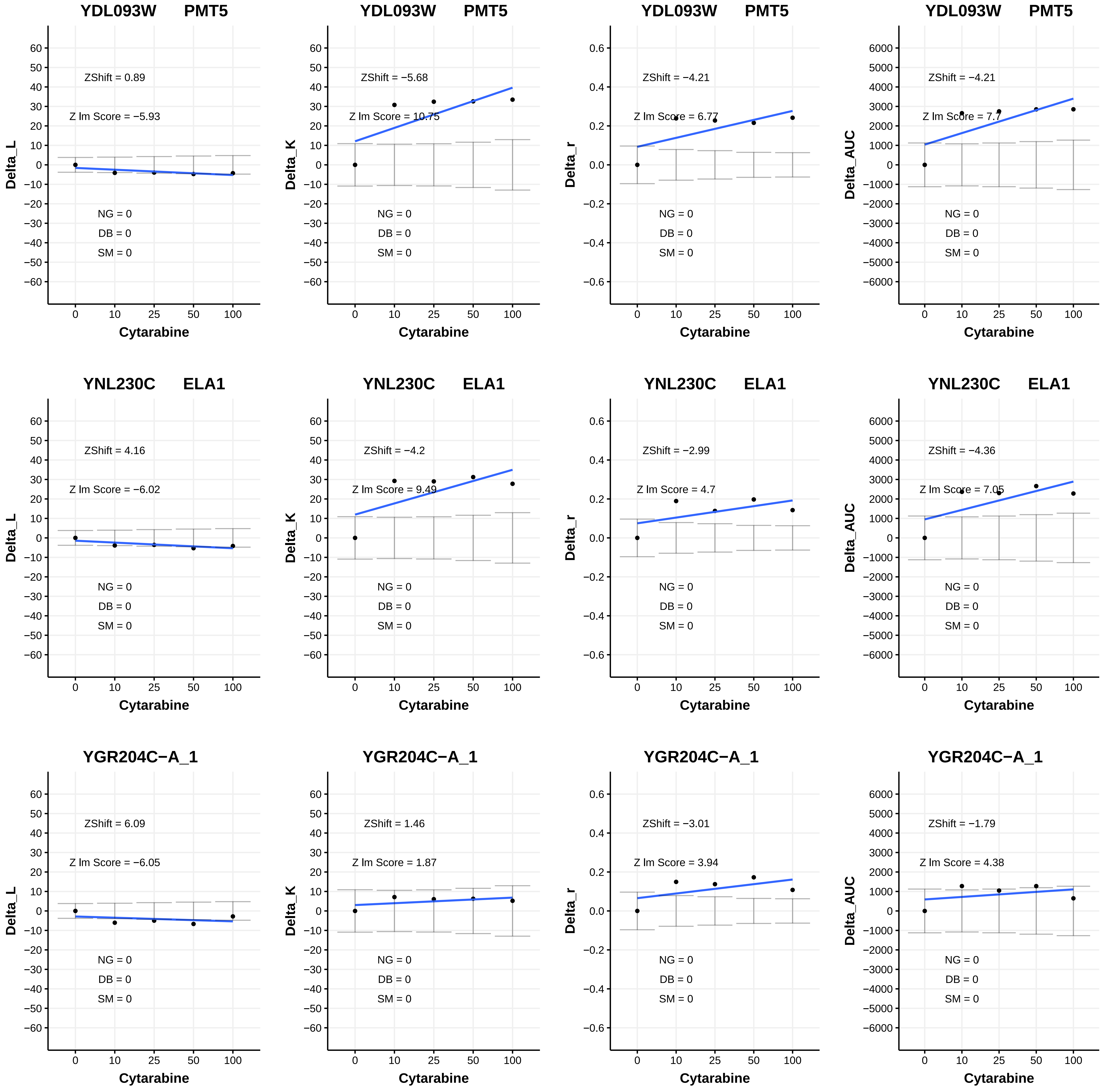

YDL093W PMT5
ZShift = 0.89
Z lm Score = −5.93
NG = 0
DB = 0
SM = 0
Delta_L
Cytarabine
YDL093W PMT5
ZShift = −5.68
Z lm Score = 10.75
NG = 0
DB = 0
SM = 0
Delta_K
Cytarabine
YDL093W PMT5
ZShift = −4.21
Z lm Score = 6.77
NG = 0
DB = 0
SM = 0
Delta_r
Cytarabine
YDL093W PMT5
ZShift = −4.21
Z lm Score = 7.7
NG = 0
DB = 0
SM = 0
Delta_AUC
Cytarabine
YNL230C ELA1
ZShift = 4.16
Z lm Score = −6.02
NG = 0
DB = 0
SM = 0
Delta_L
Cytarabine
YNL230C ELA1
ZShift = −4.2
Z lm Score = 9.49
NG = 0
DB = 0
SM = 0
Delta_K
Cytarabine
YNL230C ELA1
ZShift = −2.99
Z lm Score = 4.7
NG = 0
DB = 0
SM = 0
Delta_r
Cytarabine
YNL230C ELA1
ZShift = −4.36
Z lm Score = 7.05
NG = 0
DB = 0
SM = 0
Delta_AUC
Cytarabine
YGR204C−A_1
ZShift = 6.09
Z lm Score = −6.05
NG = 0
DB = 0
SM = 0
Delta_L
Cytarabine
YGR204C−A_1
ZShift = 1.46
Z lm Score = 1.87
NG = 0
DB = 0
SM = 0
Delta_K
Cytarabine
YGR204C−A_1
ZShift = −3.01
Z lm Score = 3.94
NG = 0
DB = 0
SM = 0
Delta_r
Cytarabine
YGR204C−A_1
ZShift = −1.79
Z lm Score = 4.38
NG = 0
DB = 0
SM = 0
Delta_AUC
Cytarabine

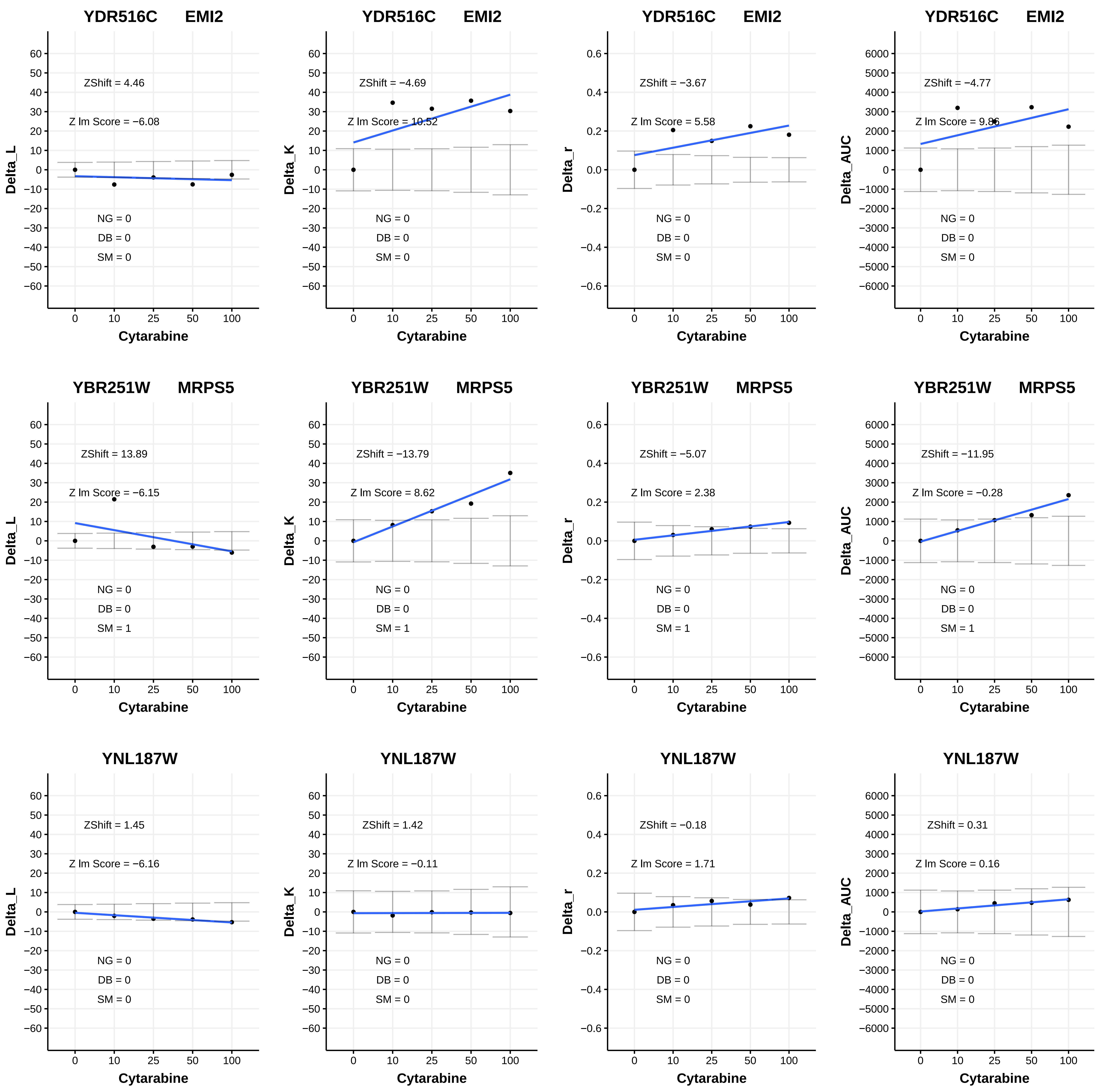

YDR516C EMI2
ZShift = 4.46
Z lm Score = −6.08
NG = 0
DB = 0
SM = 0
Delta_L
Cytarabine
YDR516C EMI2
ZShift = −4.69
Z lm Score = 10.52
NG = 0
DB = 0
SM = 0
Delta_K
Cytarabine
YDR516C EMI2
ZShift = −3.67
Z lm Score = 5.58
NG = 0
DB = 0
SM = 0
Delta_r
Cytarabine
YDR516C EMI2
ZShift = −4.77
Z lm Score = 9.86
NG = 0
DB = 0
SM = 0
Delta_AUC
Cytarabine
YBR251W MRPS5
ZShift = 13.89
Z lm Score = −6.15
NG = 0
DB = 0
SM = 1
Delta_L
Cytarabine
YBR251W MRPS5
ZShift = −13.79
Z lm Score = 8.62
NG = 0
DB = 0
SM = 1
Delta_K
Cytarabine
YBR251W MRPS5
ZShift = −5.07
Z lm Score = 2.38
NG = 0
DB = 0
SM = 1
Delta_r
Cytarabine
YBR251W MRPS5
ZShift = −11.95
Z lm Score = −0.28
NG = 0
DB = 0
SM = 1
Delta_AUC
Cytarabine
YNL187W
ZShift = 1.45
Z lm Score = −6.16
NG = 0
DB = 0
SM = 0
Delta_L
Cytarabine
YNL187W
ZShift = 1.42
Z lm Score = −0.11
NG = 0
DB = 0
SM = 0
Delta_K
Cytarabine
YNL187W
ZShift = −0.18
Z lm Score = 1.71
NG = 0
DB = 0
SM = 0
Delta_r
Cytarabine
YNL187W
ZShift = 0.31
Z lm Score = 0.16
NG = 0
DB = 0
SM = 0
Delta_AUC
Cytarabine

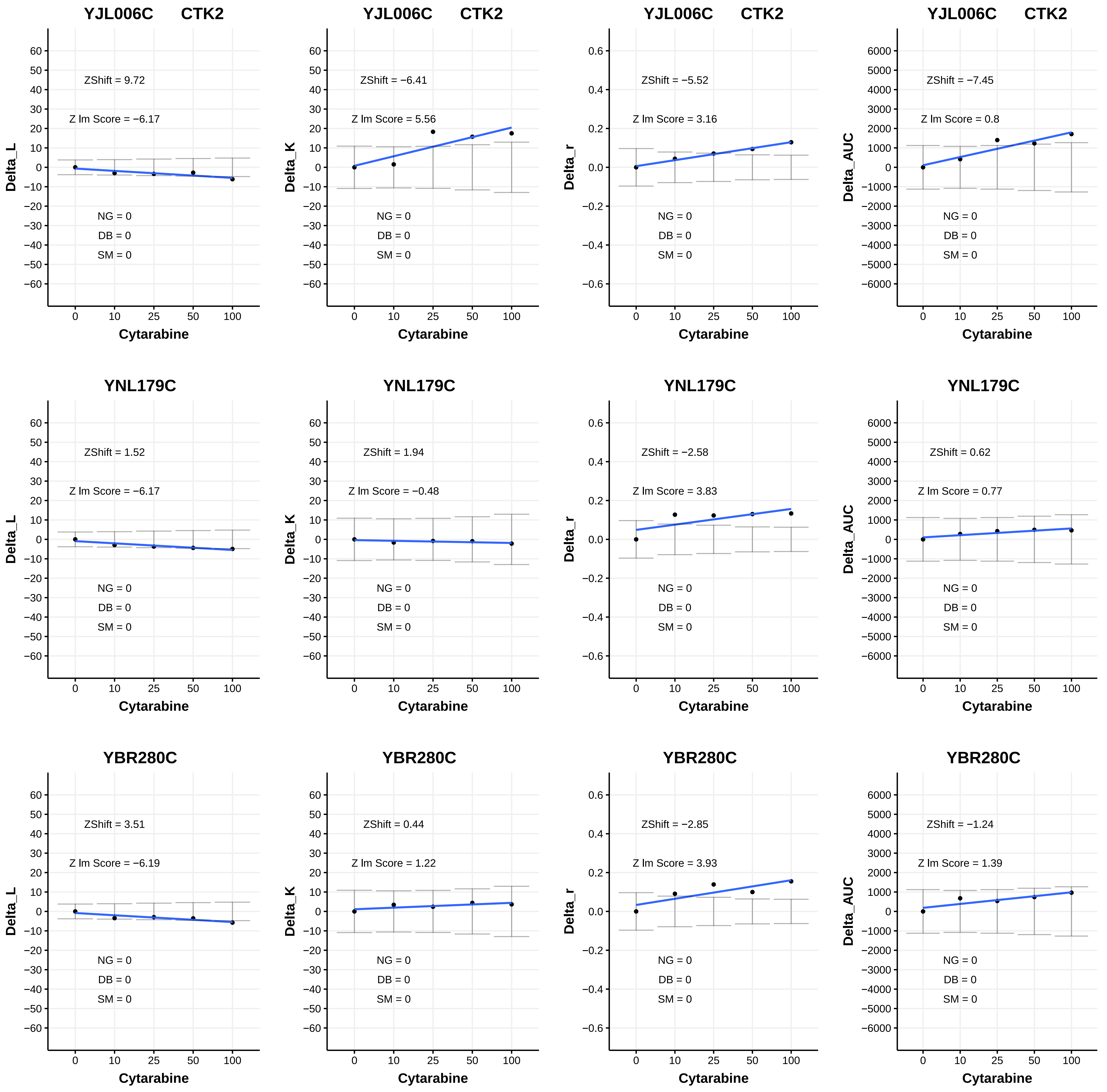

YJL006C CTK2
ZShift = 9.72
Z lm Score = −6.17
NG = 0
DB = 0
SM = 0
Delta_L
Cytarabine
YJL006C CTK2
ZShift = −6.41
Z lm Score = 5.56
NG = 0
DB = 0
SM = 0
Delta_K
Cytarabine
YJL006C CTK2
ZShift = −5.52
Z lm Score = 3.16
NG = 0
DB = 0
SM = 0
Delta_r
Cytarabine
YJL006C CTK2
ZShift = −7.45
Z lm Score = 0.8
NG = 0
DB = 0
SM = 0
Delta_AUC
Cytarabine
YNL179C
ZShift = 1.52
Z lm Score = −6.17
NG = 0
DB = 0
SM = 0
Delta_L
Cytarabine
YNL179C
ZShift = 1.94
Z lm Score = −0.48
NG = 0
DB = 0
SM = 0
Delta_K
Cytarabine
YNL179C
ZShift = −2.58
Z lm Score = 3.83
NG = 0
DB = 0
SM = 0
Delta_r
Cytarabine
YNL179C
ZShift = 0.62
Z lm Score = 0.77
NG = 0
DB = 0
SM = 0
Delta_AUC
Cytarabine
YBR280C
ZShift = 3.51
Z lm Score = −6.19
NG = 0
DB = 0
SM = 0
Delta_L
Cytarabine
YBR280C
ZShift = 0.44
Z lm Score = 1.22
NG = 0
DB = 0
SM = 0
Delta_K
Cytarabine
YBR280C
ZShift = −2.85
Z lm Score = 3.93
NG = 0
DB = 0
SM = 0
Delta_r
Cytarabine
YBR280C
ZShift = −1.24
Z lm Score = 1.39
NG = 0
DB = 0
SM = 0
Delta_AUC
Cytarabine

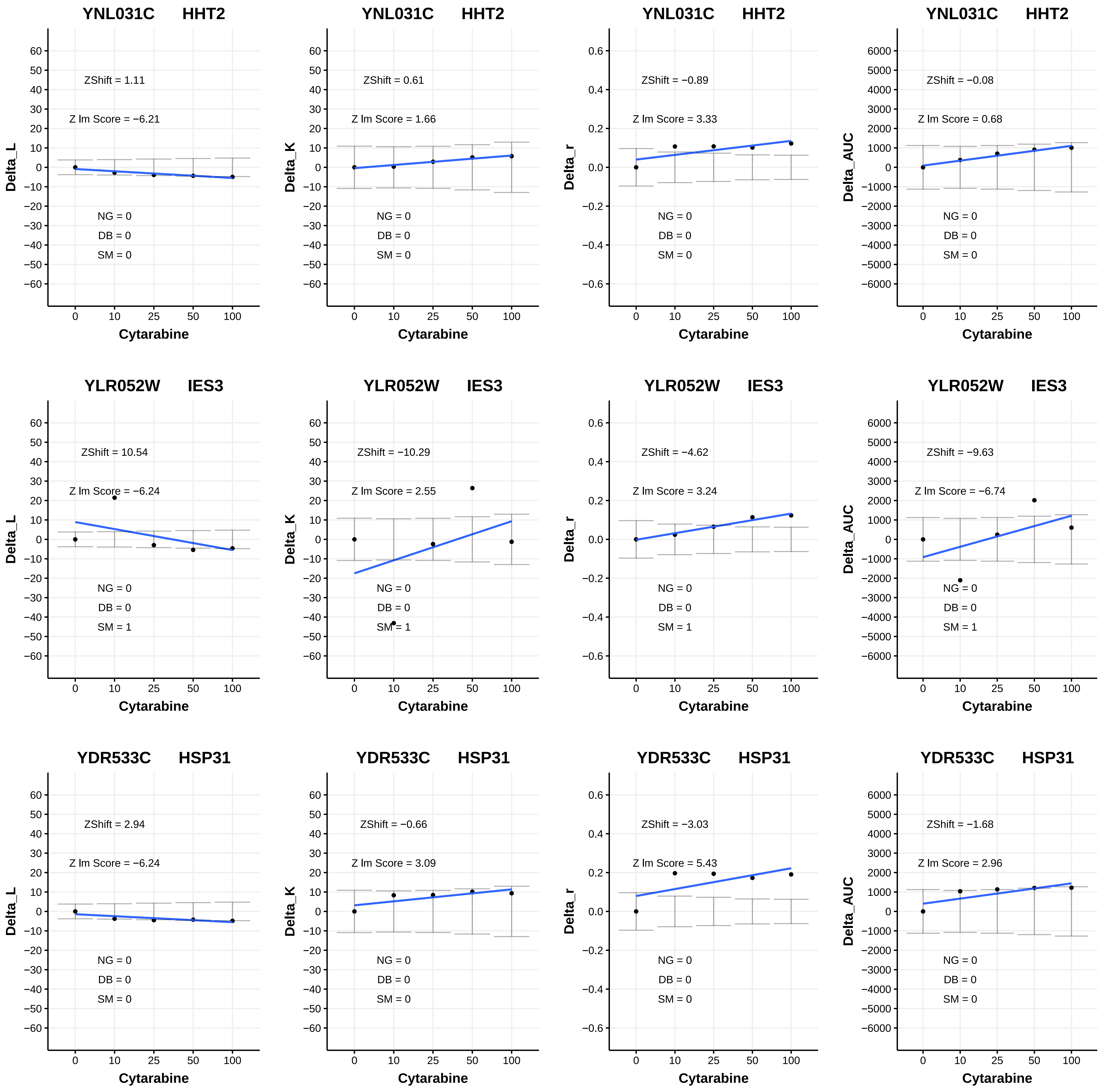

YNL031C HHT2
ZShift = 1.11
Z lm Score = −6.21
NG = 0
DB = 0
SM = 0
Delta_L
Cytarabine
YNL031C HHT2
ZShift = 0.61
Z lm Score = 1.66
NG = 0
DB = 0
SM = 0
Delta_K
Cytarabine
YNL031C HHT2
ZShift = −0.89
Z lm Score = 3.33
NG = 0
DB = 0
SM = 0
Delta_r
Cytarabine
YNL031C HHT2
ZShift = −0.08
Z lm Score = 0.68
NG = 0
DB = 0
SM = 0
Delta_AUC
Cytarabine
YLR052W IES3
ZShift = 10.54
Z lm Score = −6.24
NG = 0
DB = 0
SM = 1
Delta_L
Cytarabine
YLR052W IES3
ZShift = −10.29
Z lm Score = 2.55
NG = 0
DB = 0
SM = 1
Delta_K
Cytarabine
YLR052W IES3
ZShift = −4.62
Z lm Score = 3.24
NG = 0
DB = 0
SM = 1
Delta_r
Cytarabine
YLR052W IES3
ZShift = −9.63
Z lm Score = −6.74
NG = 0
DB = 0
SM = 1
Delta_AUC
Cytarabine
YDR533C HSP31
ZShift = 2.94
Z lm Score = −6.24
NG = 0
DB = 0
SM = 0
Delta_L
Cytarabine
YDR533C HSP31
ZShift = −0.66
Z lm Score = 3.09
NG = 0
DB = 0
SM = 0
Delta_K
Cytarabine
YDR533C HSP31
ZShift = −3.03
Z lm Score = 5.43
NG = 0
DB = 0
SM = 0
Delta_r
Cytarabine
YDR533C HSP31
ZShift = −1.68
Z lm Score = 2.96
NG = 0
DB = 0
SM = 0
Delta_AUC
Cytarabine

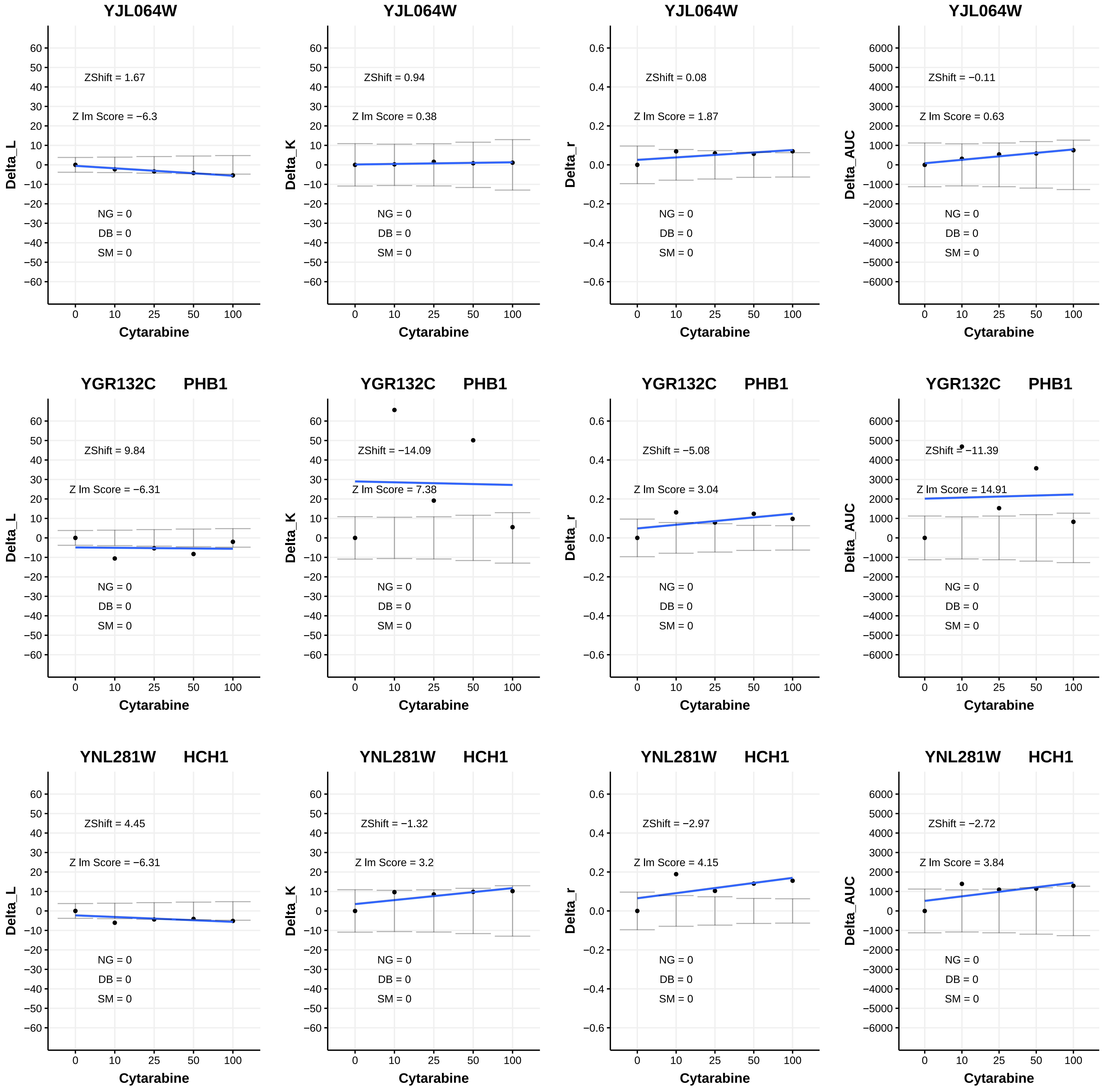

YJL064W
ZShift = 1.67
Z lm Score = −6.3
NG = 0
DB = 0
SM = 0
Delta_L
Cytarabine
YJL064W
ZShift = 0.94
Z lm Score = 0.38
NG = 0
DB = 0
SM = 0
Delta_K
Cytarabine
YJL064W
ZShift = 0.08
Z lm Score = 1.87
NG = 0
DB = 0
SM = 0
Delta_r
Cytarabine
YJL064W
ZShift = −0.11
Z lm Score = 0.63
NG = 0
DB = 0
SM = 0
Delta_AUC
Cytarabine
YGR132C PHB1
ZShift = 9.84
Z lm Score = −6.31
NG = 0
DB = 0
SM = 0
Delta_L
Cytarabine
YGR132C PHB1
ZShift = −14.09
Z lm Score = 7.38
NG = 0
DB = 0
SM = 0
Delta_K
Cytarabine
YGR132C PHB1
ZShift = −5.08
Z lm Score = 3.04
NG = 0
DB = 0
SM = 0
Delta_r
Cytarabine
YGR132C PHB1
ZShift = −11.39
Z lm Score = 14.91
NG = 0
DB = 0
SM = 0
Delta_AUC
Cytarabine
YNL281W HCH1
ZShift = 4.45
Z lm Score = −6.31
NG = 0
DB = 0
SM = 0
Delta_L
Cytarabine
YNL281W HCH1
ZShift = −1.32
Z lm Score = 3.2
NG = 0
DB = 0
SM = 0
Delta_K
Cytarabine
YNL281W HCH1
ZShift = −2.97
Z lm Score = 4.15
NG = 0
DB = 0
SM = 0
Delta_r
Cytarabine
YNL281W HCH1
ZShift = −2.72
Z lm Score = 3.84
NG = 0
DB = 0
SM = 0
Delta_AUC
Cytarabine

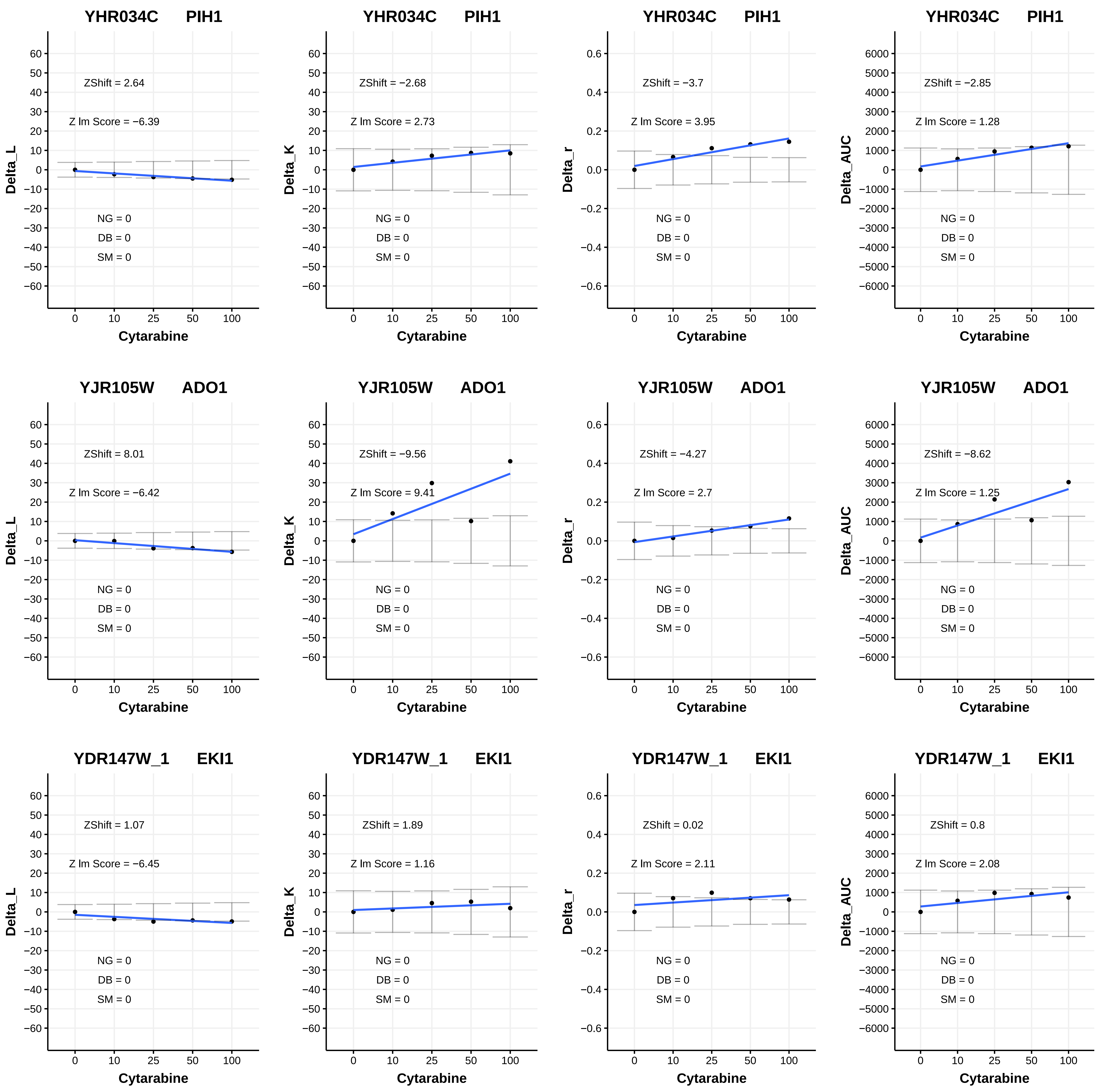
YHR034C PIH1
ZShift = 2.64
Z lm Score = −6.39
NG = 0
DB = 0
SM = 0
Delta_L
Cytarabine
YHR034C PIH1
ZShift = −2.68
Z lm Score = 2.73
NG = 0
DB = 0
SM = 0
Delta_K
Cytarabine
YHR034C PIH1
ZShift = −3.7
Z lm Score = 3.95
NG = 0
DB = 0
SM = 0
Delta_r
Cytarabine
YHR034C PIH1
ZShift = −2.85
Z lm Score = 1.28
NG = 0
DB = 0
SM = 0
Delta_AUC
Cytarabine
YJR105W ADO1
ZShift = 8.01
Z lm Score = −6.42
NG = 0
DB = 0
SM = 0
Delta_L
Cytarabine
YJR105W ADO1
ZShift = −9.56
Z lm Score = 9.41
NG = 0
DB = 0
SM = 0
Delta_K
Cytarabine
YJR105W ADO1
ZShift = −4.27
Z lm Score = 2.7
NG = 0
DB = 0
SM = 0
Delta_r
Cytarabine
YJR105W ADO1
ZShift = −8.62
Z lm Score = 1.25
NG = 0
DB = 0
SM = 0
Delta_AUC
Cytarabine
YDR147W_1 EKI1
ZShift = 1.07
Z lm Score = −6.45
NG = 0
DB = 0
SM = 0
Delta_L
Cytarabine
YDR147W_1 EKI1
ZShift = 1.89
Z lm Score = 1.16
NG = 0
DB = 0
SM = 0
Delta_K
Cytarabine
YDR147W_1 EKI1
ZShift = 0.02
Z lm Score = 2.11
NG = 0
DB = 0
SM = 0
Delta_r
Cytarabine
YDR147W_1 EKI1
ZShift = 0.8
Z lm Score = 2.08
NG = 0
DB = 0
SM = 0
Delta_AUC
Cytarabine

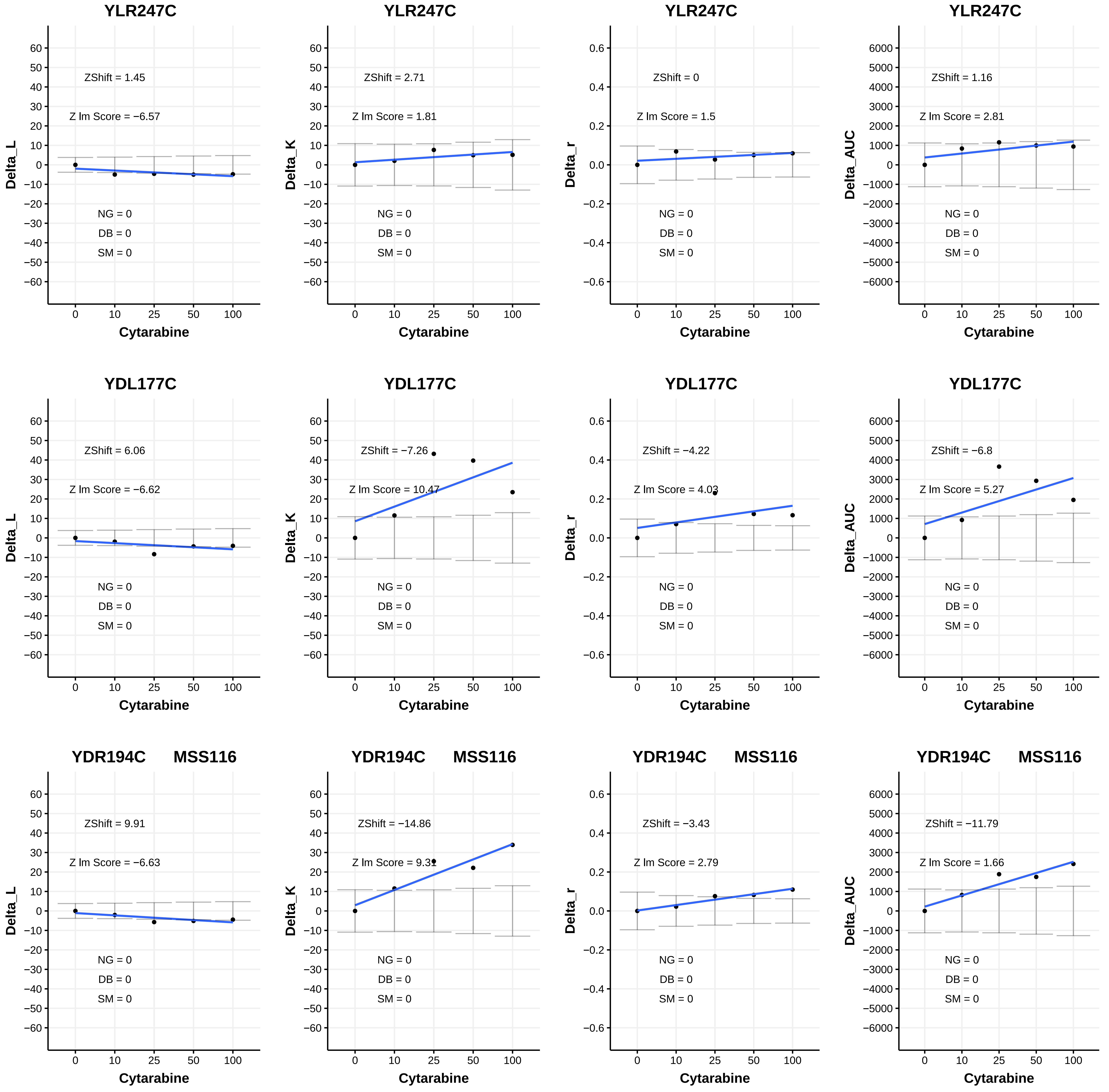
YLR247C
ZShift = 1.45
Z lm Score = −6.57
NG = 0
DB = 0
SM = 0
Delta_L
Cytarabine
YLR247C
ZShift = 2.71
Z lm Score = 1.81
NG = 0
DB = 0
SM = 0
Delta_K
Cytarabine
YLR247C
ZShift = 0
Z lm Score = 1.5
NG = 0
DB = 0
SM = 0
Delta_r
Cytarabine
YLR247C
ZShift = 1.16
Z lm Score = 2.81
NG = 0
DB = 0
SM = 0
Delta_AUC
Cytarabine
YDL177C
ZShift = 6.06
Z lm Score = −6.62
NG = 0
DB = 0
SM = 0
Delta_L
Cytarabine
YDL177C
ZShift = −7.26
Z lm Score = 10.47
NG = 0
DB = 0
SM = 0
Delta_K
Cytarabine
YDL177C
ZShift = −4.22
Z lm Score = 4.03
NG = 0
DB = 0
SM = 0
Delta_r
Cytarabine
YDL177C
ZShift = −6.8
Z lm Score = 5.27
NG = 0
DB = 0
SM = 0
Delta_AUC
Cytarabine
YDR194C MSS116
ZShift = 9.91
Z lm Score = −6.63
NG = 0
DB = 0
SM = 0
Delta_L
Cytarabine
YDR194C MSS116
ZShift = −14.86
Z lm Score = 9.31
NG = 0
DB = 0
SM = 0
Delta_K
Cytarabine
YDR194C MSS116
ZShift = −3.43
Z lm Score = 2.79
NG = 0
DB = 0
SM = 0
Delta_r
Cytarabine
YDR194C MSS116
ZShift = −11.79
Z lm Score = 1.66
NG = 0
DB = 0
SM = 0
Delta_AUC
Cytarabine

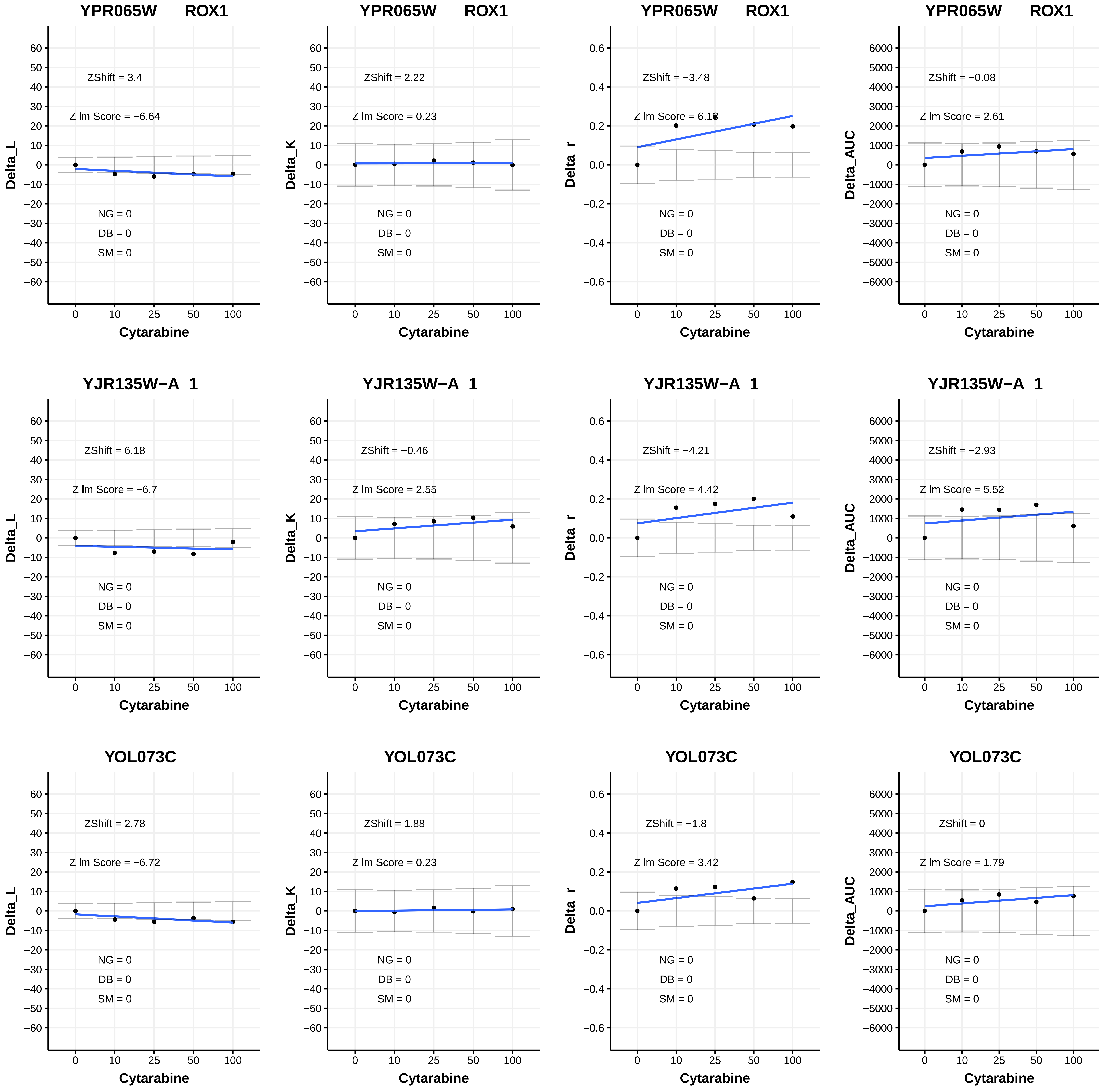

YPR065W ROX1
ZShift = 3.4
Z lm Score = −6.64
NG = 0
DB = 0
SM = 0
Delta_L
Cytarabine
YPR065W ROX1
ZShift = 2.22
Z lm Score = 0.23
NG = 0
DB = 0
SM = 0
Delta_K
Cytarabine
YPR065W ROX1
ZShift = −3.48
Z lm Score = 6.18
NG = 0
DB = 0
SM = 0
Delta_r
Cytarabine
YPR065W ROX1
ZShift = −0.08
Z lm Score = 2.61
NG = 0
DB = 0
SM = 0
Delta_AUC
Cytarabine
YJR135W−A_1
ZShift = 6.18
Z lm Score = −6.7
NG = 0
DB = 0
SM = 0
Delta_L
Cytarabine
YJR135W−A_1
ZShift = −0.46
Z lm Score = 2.55
NG = 0
DB = 0
SM = 0
Delta_K
Cytarabine
YJR135W−A_1
ZShift = −4.21
Z lm Score = 4.42
NG = 0
DB = 0
SM = 0
Delta_r
Cytarabine
YJR135W−A_1
ZShift = −2.93
Z lm Score = 5.52
NG = 0
DB = 0
SM = 0
Delta_AUC
Cytarabine
YOL073C
ZShift = 2.78
Z lm Score = −6.72
NG = 0
DB = 0
SM = 0
Delta_L
Cytarabine
YOL073C
ZShift = 1.88
Z lm Score = 0.23
NG = 0
DB = 0
SM = 0
Delta_K
Cytarabine
YOL073C
ZShift = −1.8
Z lm Score = 3.42
NG = 0
DB = 0
SM = 0
Delta_r
Cytarabine
YOL073C
ZShift = 0
Z lm Score = 1.79
NG = 0
DB = 0
SM = 0
Delta_AUC
Cytarabine

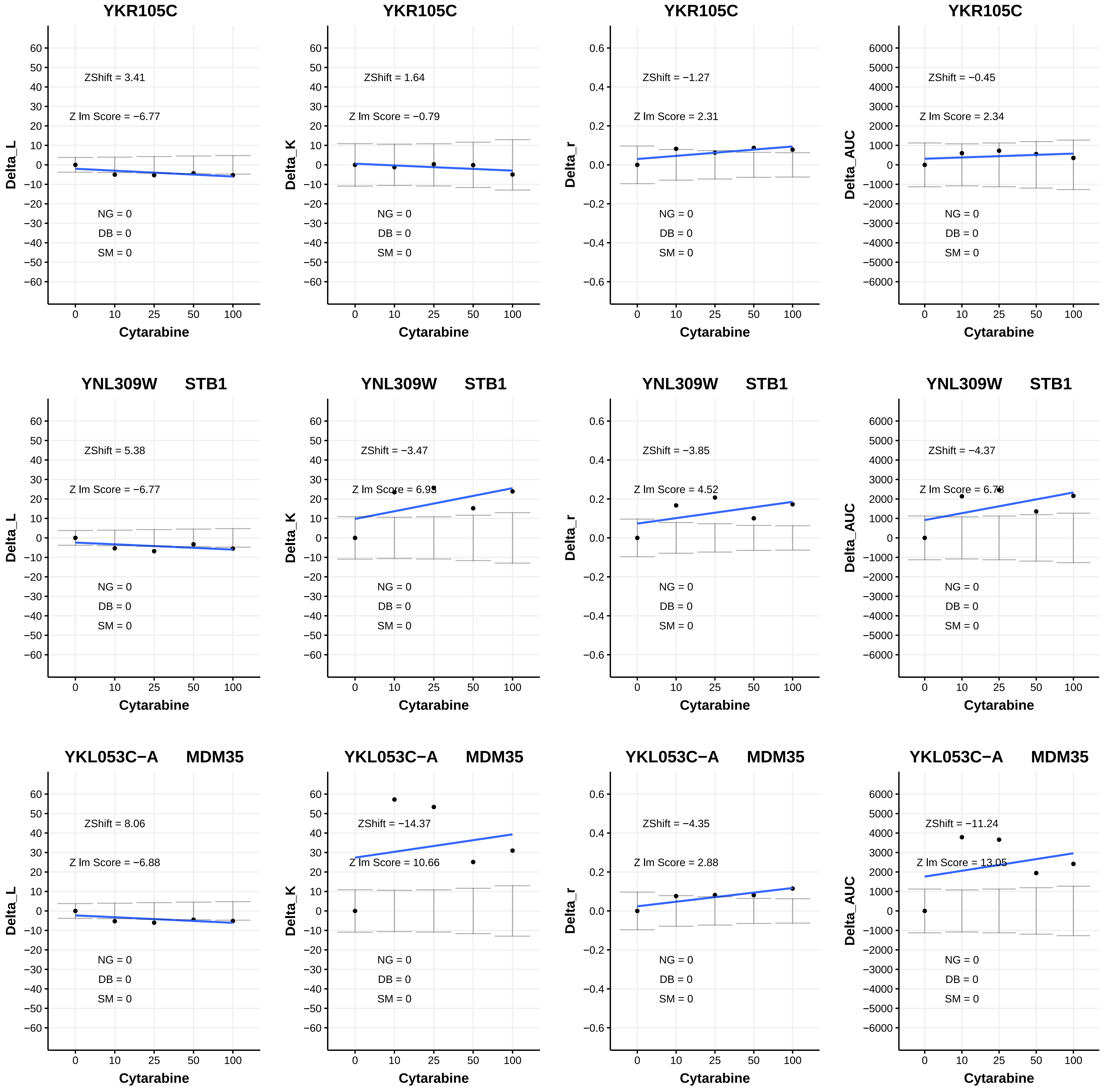

YKR105C
ZShift = 3.41
Z lm Score = −6.77
NG = 0
DB = 0
SM = 0
Delta_L
Cytarabine
YKR105C
ZShift = 1.64
Z lm Score = −0.79
NG = 0
DB = 0
SM = 0
Delta_K
Cytarabine
YKR105C
ZShift = −1.27
Z lm Score = 2.31
NG = 0
DB = 0
SM = 0
Delta_r
Cytarabine
YKR105C
ZShift = −0.45
Z lm Score = 2.34
NG = 0
DB = 0
SM = 0
Delta_AUC
Cytarabine
YNL309W STB1
ZShift = 5.38
Z lm Score = −6.77
NG = 0
DB = 0
SM = 0
Delta_L
Cytarabine
YNL309W STB1
ZShift = −3.47
Z lm Score = 6.93
NG = 0
DB = 0
SM = 0
Delta_K
Cytarabine
YNL309W STB1
ZShift = −3.85
Z lm Score = 4.52
NG = 0
DB = 0
SM = 0
Delta_r
Cytarabine
YNL309W STB1
ZShift = −4.37
Z lm Score = 6.78
NG = 0
DB = 0
SM = 0
Delta_AUC
Cytarabine
YKL053C−A MDM35
ZShift = 8.06
Z lm Score = −6.88
NG = 0
DB = 0
SM = 0
Delta_L
Cytarabine
YKL053C−A MDM35
ZShift = −14.37
Z lm Score = 10.66
NG = 0
DB = 0
SM = 0
Delta_K
Cytarabine
YKL053C−A MDM35
ZShift = −4.35
Z lm Score = 2.88
NG = 0
DB = 0
SM = 0
Delta_r
Cytarabine
YKL053C−A MDM35
ZShift = −11.24
Z lm Score = 13.05
NG = 0
DB = 0
SM = 0
Delta_AUC
Cytarabine

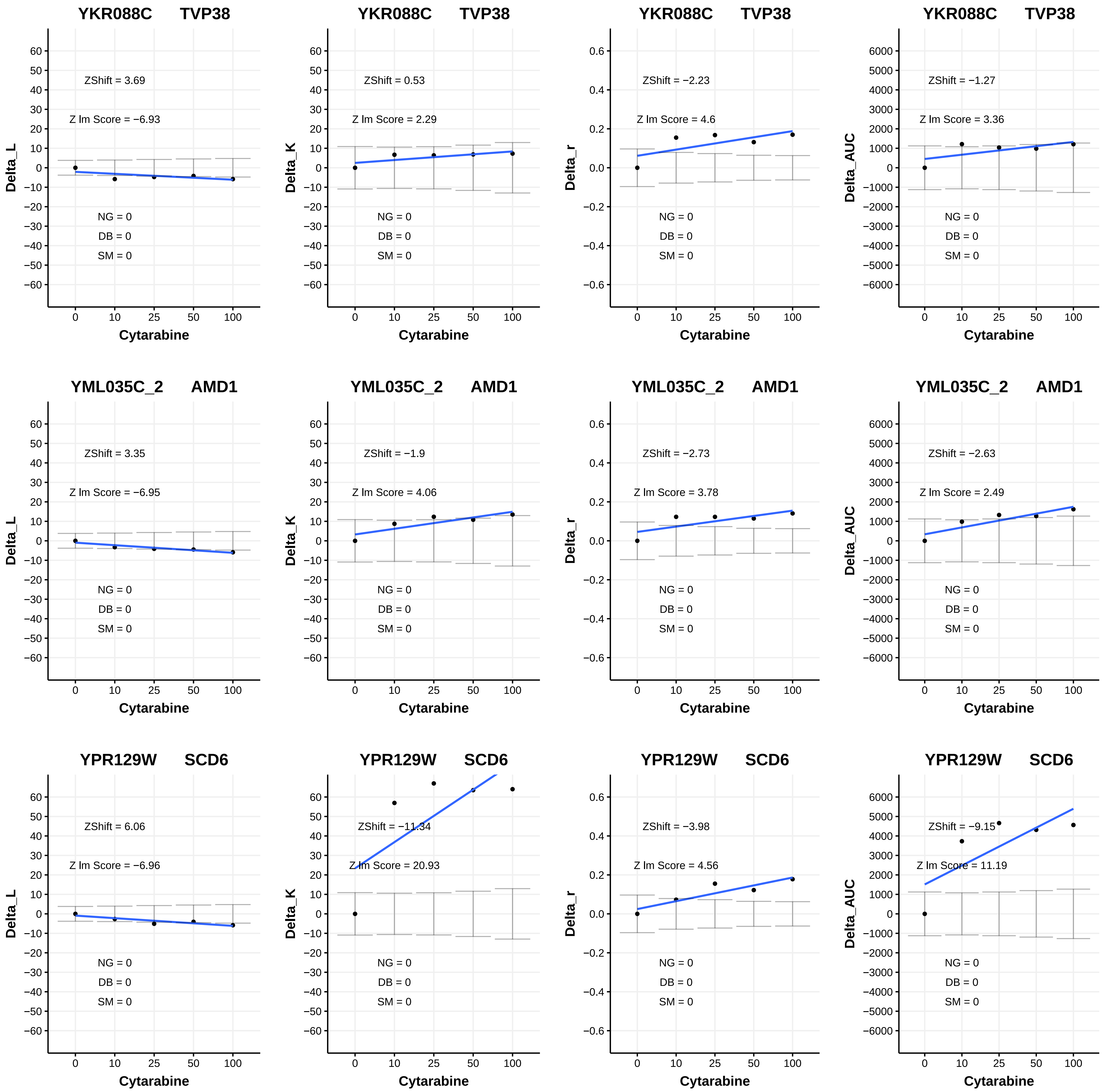
YKR088C TVP38
ZShift = 3.69
Z lm Score = −6.93
NG = 0
DB = 0
SM = 0
Delta_L
Cytarabine
YKR088C TVP38
ZShift = 0.53
Z lm Score = 2.29
NG = 0
DB = 0
SM = 0
Delta_K
Cytarabine
YKR088C TVP38
ZShift = −2.23
Z lm Score = 4.6
NG = 0
DB = 0
SM = 0
Delta_r
Cytarabine
YKR088C TVP38
ZShift = −1.27
Z lm Score = 3.36
NG = 0
DB = 0
SM = 0
Delta_AUC
Cytarabine
YML035C_2 AMD1
ZShift = 3.35
Z lm Score = −6.95
NG = 0
DB = 0
SM = 0
Delta_L
Cytarabine
YML035C_2 AMD1
ZShift = −1.9
Z lm Score = 4.06
NG = 0
DB = 0
SM = 0
Delta_K
Cytarabine
YML035C_2 AMD1
ZShift = −2.73
Z lm Score = 3.78
NG = 0
DB = 0
SM = 0
Delta_r
Cytarabine
YML035C_2 AMD1
ZShift = −2.63
Z lm Score = 2.49
NG = 0
DB = 0
SM = 0
Delta_AUC
Cytarabine
YPR129W SCD6
ZShift = 6.06
Z lm Score = −6.96
NG = 0
DB = 0
SM = 0
Delta_L
Cytarabine
YPR129W SCD6
ZShift = −11.34
Z lm Score = 20.93
NG = 0
DB = 0
SM = 0
Delta_K
Cytarabine
YPR129W SCD6
ZShift = −3.98
Z lm Score = 4.56
NG = 0
DB = 0
SM = 0
Delta_r
Cytarabine
YPR129W SCD6
ZShift = −9.15
Z lm Score = 11.19
NG = 0
DB = 0
SM = 0
Delta_AUC
Cytarabine

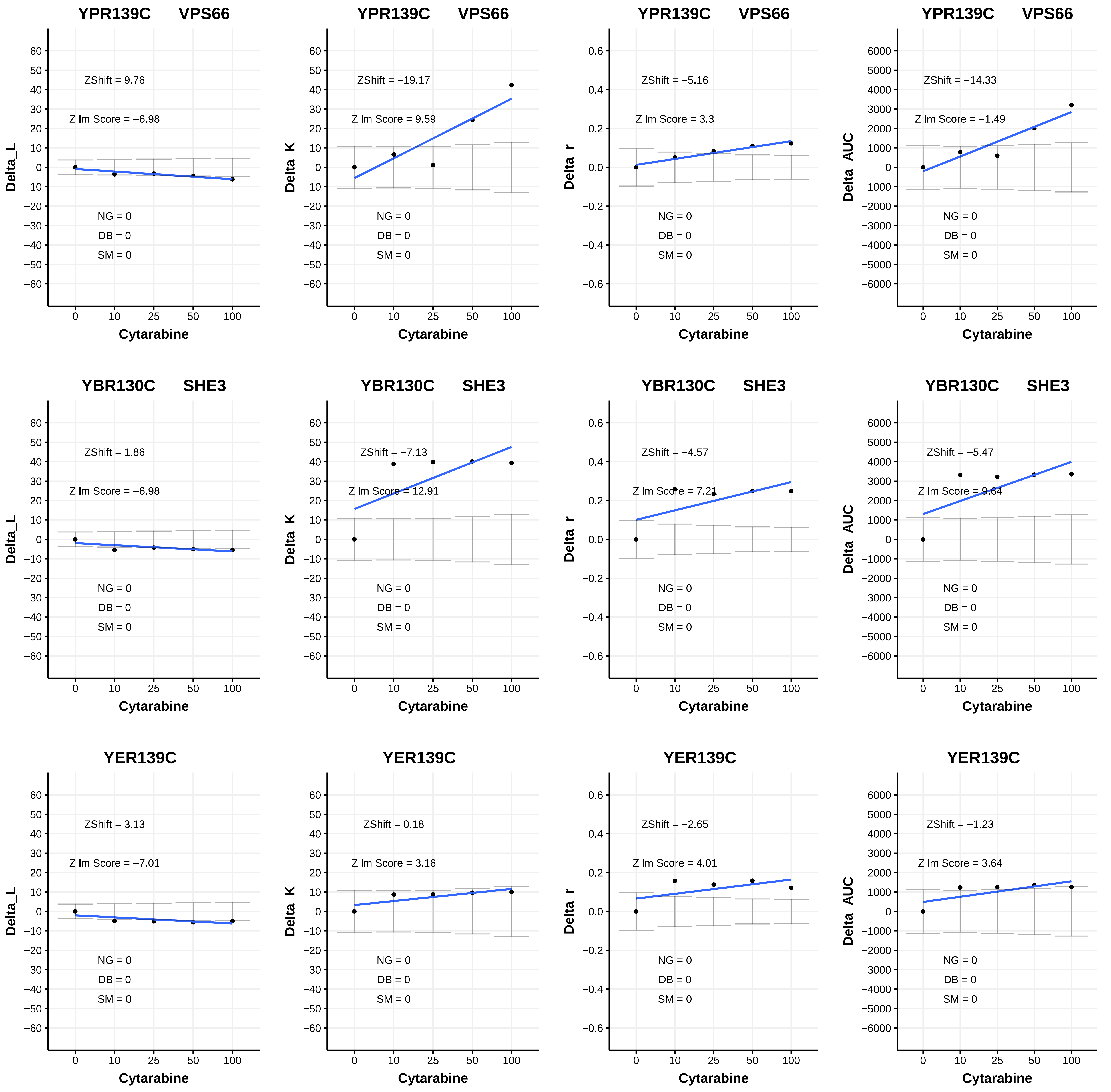
YPR139C VPS66
ZShift = 9.76
Z lm Score = −6.98
NG = 0
DB = 0
SM = 0
Delta_L
Cytarabine
YPR139C VPS66
ZShift = −19.17
Z lm Score = 9.59
NG = 0
DB = 0
SM = 0
Delta_K
Cytarabine
YPR139C VPS66
ZShift = −5.16
Z lm Score = 3.3
NG = 0
DB = 0
SM = 0
Delta_r
Cytarabine
YPR139C VPS66
ZShift = −14.33
Z lm Score = −1.49
NG = 0
DB = 0
SM = 0
Delta_AUC
Cytarabine
YBR130C SHE3
ZShift = 1.86
Z lm Score = −6.98
NG = 0
DB = 0
SM = 0
Delta_L
Cytarabine
YBR130C SHE3
ZShift = −7.13
Z lm Score = 12.91
NG = 0
DB = 0
SM = 0
Delta_K
Cytarabine
YBR130C SHE3
ZShift = −4.57
Z lm Score = 7.21
NG = 0
DB = 0
SM = 0
Delta_r
Cytarabine
YBR130C SHE3
ZShift = −5.47
Z lm Score = 9.64
NG = 0
DB = 0
SM = 0
Delta_AUC
Cytarabine
YER139C
ZShift = 3.13
Z lm Score = −7.01
NG = 0
DB = 0
SM = 0
Delta_L
Cytarabine
YER139C
ZShift = 0.18
Z lm Score = 3.16
NG = 0
DB = 0
SM = 0
Delta_K
Cytarabine
YER139C
ZShift = −2.65
Z lm Score = 4.01
NG = 0
DB = 0
SM = 0
Delta_r
Cytarabine
YER139C
ZShift = −1.23
Z lm Score = 3.64
NG = 0
DB = 0
SM = 0
Delta_AUC
Cytarabine

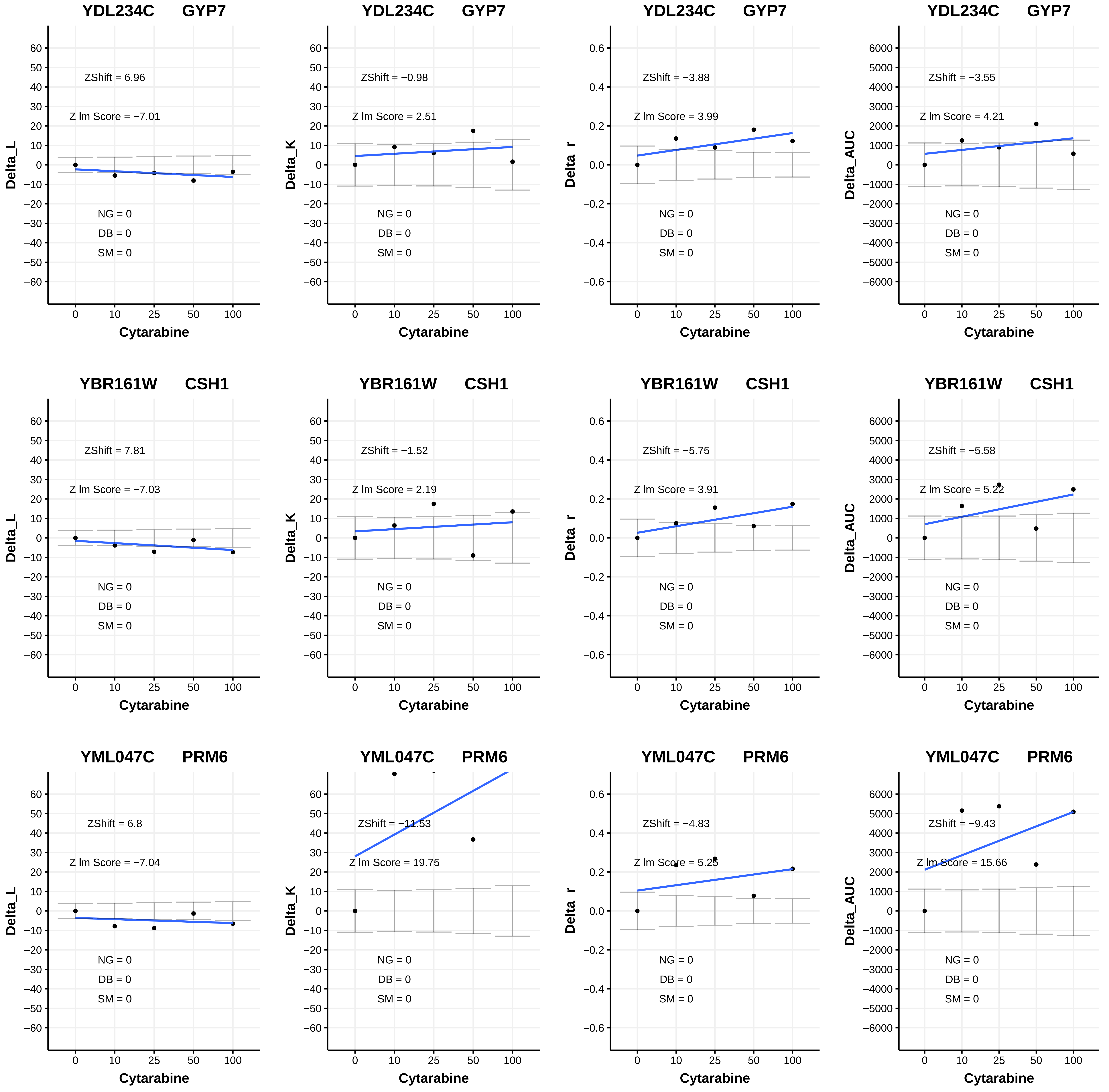
YDL234C GYP7
ZShift = 6.96
Z lm Score = −7.01
Delta_L
NG = 0
DB = 0
SM = 0
Cytarabine
YDL234C GYP7
ZShift = −0.98
Z lm Score = 2.51
Delta_K
NG = 0
DB = 0
SM = 0
Cytarabine
YDL234C GYP7
ZShift = −3.88
Z lm Score = 3.99
Delta_r
NG = 0
DB = 0
SM = 0
Cytarabine
YDL234C GYP7
ZShift = −3.55
Z lm Score = 4.21
Delta_AUC
NG = 0
DB = 0
SM = 0
Cytarabine
YBR161W CSH1
ZShift = 7.81
Z lm Score = −7.03
Delta_L
NG = 0
DB = 0
SM = 0
Cytarabine
YBR161W CSH1
ZShift = −1.52
Z lm Score = 2.19
Delta_K
NG = 0
DB = 0
SM = 0
Cytarabine
YBR161W CSH1
ZShift = −5.75
Z lm Score = 3.91
Delta_r
NG = 0
DB = 0
SM = 0
Cytarabine
YBR161W CSH1
ZShift = −5.58
Z lm Score = 5.22
Delta_AUC
NG = 0
DB = 0
SM = 0
Cytarabine
YML047C PRM6
ZShift = 6.8
Z lm Score = −7.04
Delta_L
NG = 0
DB = 0
SM = 0
Cytarabine
YML047C PRM6
ZShift = −11.53
Z lm Score = 19.75
Delta_K
NG = 0
DB = 0
SM = 0
Cytarabine
YML047C PRM6
ZShift = −4.83
Z lm Score = 5.25
Delta_r
NG = 0
DB = 0
SM = 0
Cytarabine
YML047C PRM6
ZShift = −9.43
Z lm Score = 15.66
Delta_AUC
NG = 0
DB = 0
SM = 0
Cytarabine

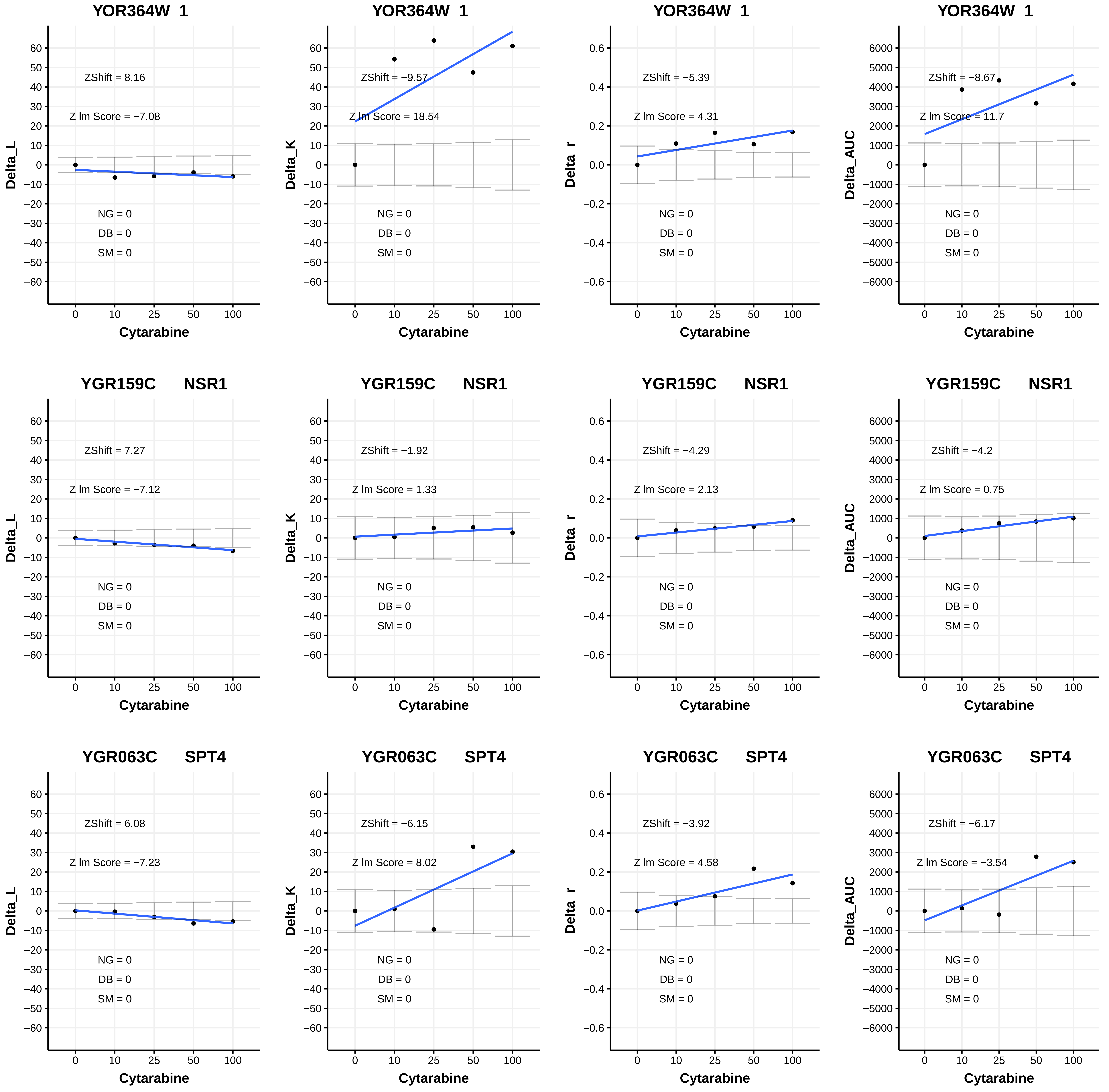
YOR364W_1
ZShift = 8.16
Z lm Score = −7.08
NG = 0
DB = 0
SM = 0
Delta_L
Cytarabine
YOR364W_1
ZShift = −9.57
Z lm Score = 18.54
NG = 0
DB = 0
SM = 0
Delta_K
Cytarabine
YOR364W_1
ZShift = −5.39
Z lm Score = 4.31
NG = 0
DB = 0
SM = 0
Delta_r
Cytarabine
YOR364W_1
ZShift = −8.67
Z lm Score = 11.7
NG = 0
DB = 0
SM = 0
Delta_AUC
Cytarabine
YGR159C NSR1
ZShift = 7.27
Z lm Score = −7.12
NG = 0
DB = 0
SM = 0
Delta_L
Cytarabine
YGR159C NSR1
ZShift = −1.92
Z lm Score = 1.33
NG = 0
DB = 0
SM = 0
Delta_K
Cytarabine
YGR159C NSR1
ZShift = −4.29
Z lm Score = 2.13
NG = 0
DB = 0
SM = 0
Delta_r
Cytarabine
YGR159C NSR1
ZShift = −4.2
Z lm Score = 0.75
NG = 0
DB = 0
SM = 0
Delta_AUC
Cytarabine
YGR063C SPT4
ZShift = 6.08
Z lm Score = −7.23
NG = 0
DB = 0
SM = 0
Delta_L
Cytarabine
YGR063C SPT4
ZShift = −6.15
Z lm Score = 8.02
NG = 0
DB = 0
SM = 0
Delta_K
Cytarabine
YGR063C SPT4
ZShift = −3.92
Z lm Score = 4.58
NG = 0
DB = 0
SM = 0
Delta_r
Cytarabine
YGR063C SPT4
ZShift = −6.17
Z lm Score = −3.54
NG = 0
DB = 0
SM = 0
Delta_AUC
Cytarabine

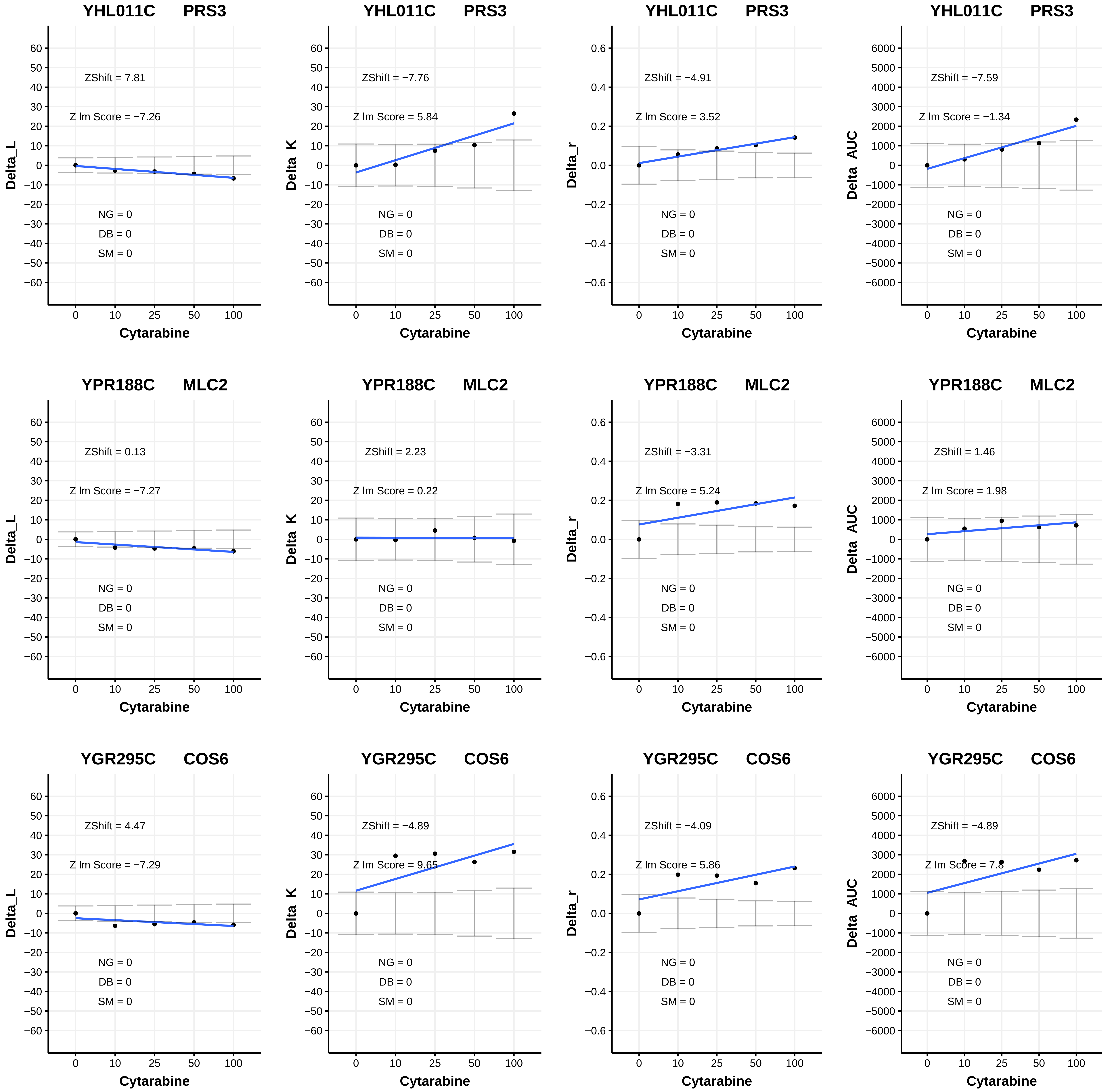
YHL011C PRS3
ZShift = 7.81
Z lm Score = −7.26
NG = 0
DB = 0
SM = 0
Delta_L
Cytarabine
YHL011C PRS3
ZShift = −7.76
Z lm Score = 5.84
NG = 0
DB = 0
SM = 0
Delta_K
Cytarabine
YHL011C PRS3
ZShift = −4.91
Z lm Score = 3.52
NG = 0
DB = 0
SM = 0
Delta_r
Cytarabine
YHL011C PRS3
ZShift = −7.59
Z lm Score = −1.34
NG = 0
DB = 0
SM = 0
Delta_AUC
Cytarabine
YPR188C MLC2
ZShift = 0.13
Z lm Score = −7.27
NG = 0
DB = 0
SM = 0
Delta_L
Cytarabine
YPR188C MLC2
ZShift = 2.23
Z lm Score = 0.22
NG = 0
DB = 0
SM = 0
Delta_K
Cytarabine
YPR188C MLC2
ZShift = −3.31
Z lm Score = 5.24
NG = 0
DB = 0
SM = 0
Delta_r
Cytarabine
YPR188C MLC2
ZShift = 1.46
Z lm Score = 1.98
NG = 0
DB = 0
SM = 0
Delta_AUC
Cytarabine
YGR295C COS6
ZShift = 4.47
Z lm Score = −7.29
NG = 0
DB = 0
SM = 0
Delta_L
Cytarabine
YGR295C COS6
ZShift = −4.89
Z lm Score = 9.65
NG = 0
DB = 0
SM = 0
Delta_K
Cytarabine
YGR295C COS6
ZShift = −4.09
Z lm Score = 5.86
NG = 0
DB = 0
SM = 0
Delta_r
Cytarabine
YGR295C COS6
ZShift = −4.89
Z lm Score = 7.8
NG = 0
DB = 0
SM = 0
Delta_AUC
Cytarabine

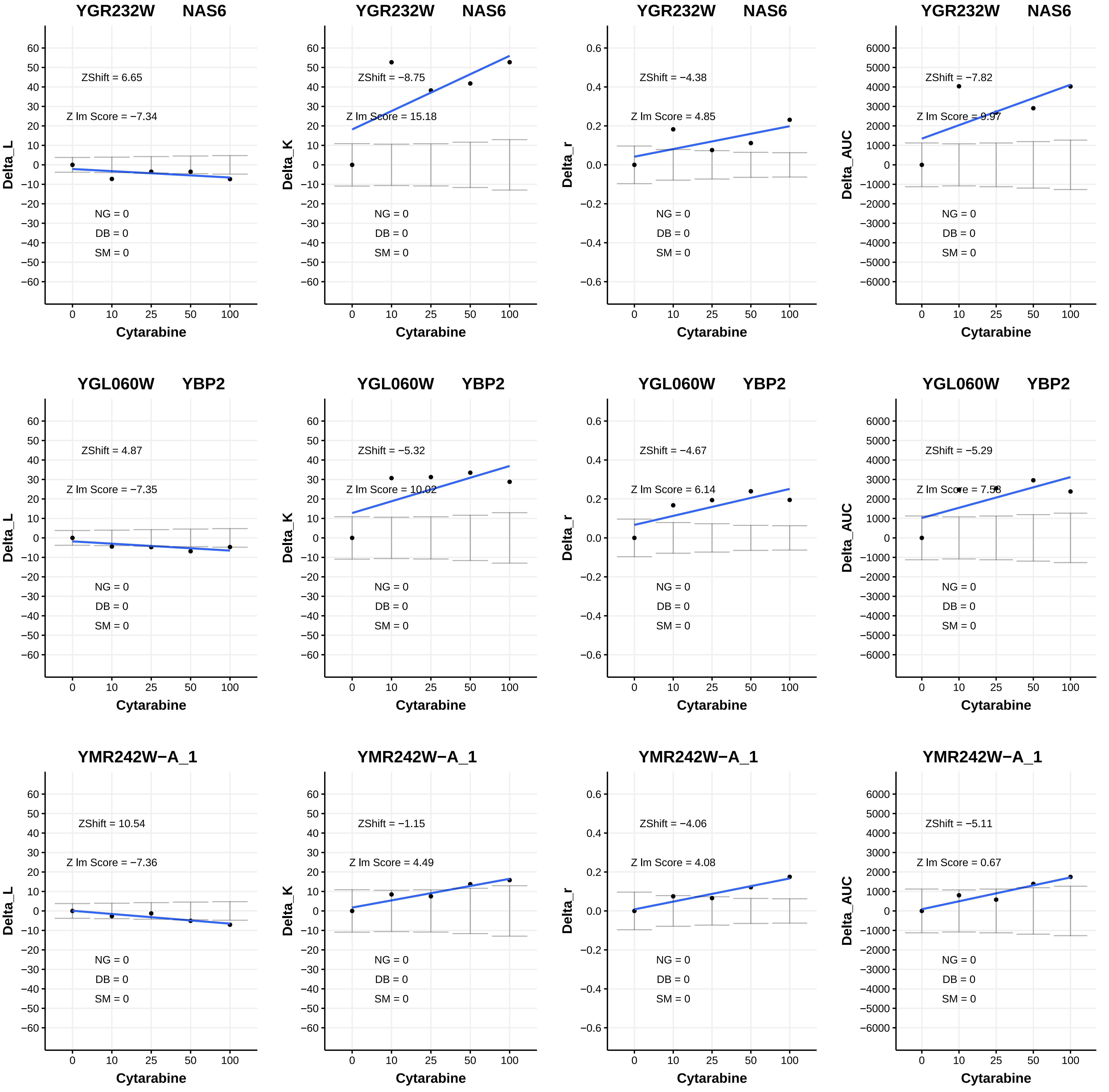
YGR232W NAS6
ZShift = 6.65
Z lm Score = −7.34
NG = 0
DB = 0
SM = 0
Delta_L
Cytarabine
YGR232W NAS6
ZShift = −8.75
Z lm Score = 15.18
NG = 0
DB = 0
SM = 0
Delta_K
Cytarabine
YGR232W NAS6
ZShift = −4.38
Z lm Score = 4.85
NG = 0
DB = 0
SM = 0
Delta_r
Cytarabine
YGR232W NAS6
ZShift = −7.82
Z lm Score = 9.97
NG = 0
DB = 0
SM = 0
Delta_AUC
Cytarabine
YGL060W YBP2
ZShift = 4.87
Z lm Score = −7.35
NG = 0
DB = 0
SM = 0
Delta_L
Cytarabine
YGL060W YBP2
ZShift = −5.32
Z lm Score = 10.02
NG = 0
DB = 0
SM = 0
Delta_K
Cytarabine
YGL060W YBP2
ZShift = −4.67
Z lm Score = 6.14
NG = 0
DB = 0
SM = 0
Delta_r
Cytarabine
YGL060W YBP2
ZShift = −5.29
Z lm Score = 7.58
NG = 0
DB = 0
SM = 0
Delta_AUC
Cytarabine
YMR242W−A_1
ZShift = 10.54
Z lm Score = −7.36
NG = 0
DB = 0
SM = 0
Delta_L
Cytarabine
YMR242W−A_1
ZShift = −1.15
Z lm Score = 4.49
NG = 0
DB = 0
SM = 0
Delta_K
Cytarabine
YMR242W−A_1
ZShift = −4.06
Z lm Score = 4.08
NG = 0
DB = 0
SM = 0
Delta_r
Cytarabine
YMR242W−A_1
ZShift = −5.11
Z lm Score = 0.67
NG = 0
DB = 0
SM = 0
Delta_AUC
Cytarabine

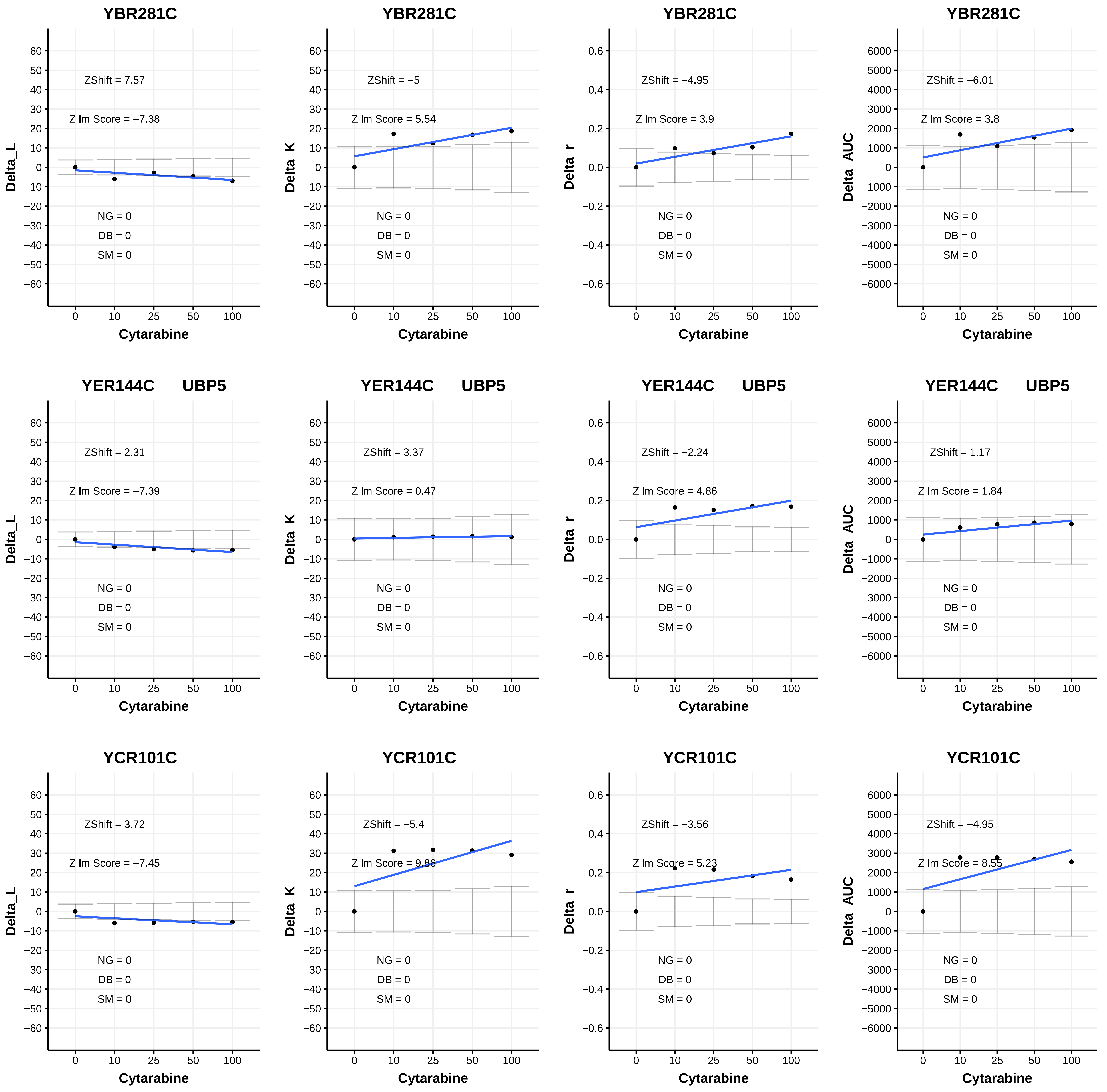

YBR281C
ZShift = 7.57
Z lm Score = −7.38
NG = 0
DB = 0
SM = 0
Delta_L
Cytarabine
YBR281C
ZShift = −5
Z lm Score = 5.54
NG = 0
DB = 0
SM = 0
Delta_K
Cytarabine
YBR281C
ZShift = −4.95
Z lm Score = 3.9
NG = 0
DB = 0
SM = 0
Delta_r
Cytarabine
YBR281C
ZShift = −6.01
Z lm Score = 3.8
NG = 0
DB = 0
SM = 0
Delta_AUC
Cytarabine
YER144C UBP5
ZShift = 2.31
Z lm Score = −7.39
NG = 0
DB = 0
SM = 0
Delta_L
Cytarabine
YER144C UBP5
ZShift = 3.37
Z lm Score = 0.47
NG = 0
DB = 0
SM = 0
Delta_K
Cytarabine
YER144C UBP5
ZShift = −2.24
Z lm Score = 4.86
NG = 0
DB = 0
SM = 0
Delta_r
Cytarabine
YER144C UBP5
ZShift = 1.17
Z lm Score = 1.84
NG = 0
DB = 0
SM = 0
Delta_AUC
Cytarabine
YCR101C
ZShift = 3.72
Z lm Score = −7.45
NG = 0
DB = 0
SM = 0
Delta_L
Cytarabine
YCR101C
ZShift = −5.4
Z lm Score = 9.86
NG = 0
DB = 0
SM = 0
Delta_K
Cytarabine
YCR101C
ZShift = −3.56
Z lm Score = 5.23
NG = 0
DB = 0
SM = 0
Delta_r
Cytarabine
YCR101C
ZShift = −4.95
Z lm Score = 8.55
NG = 0
DB = 0
SM = 0
Delta_AUC
Cytarabine

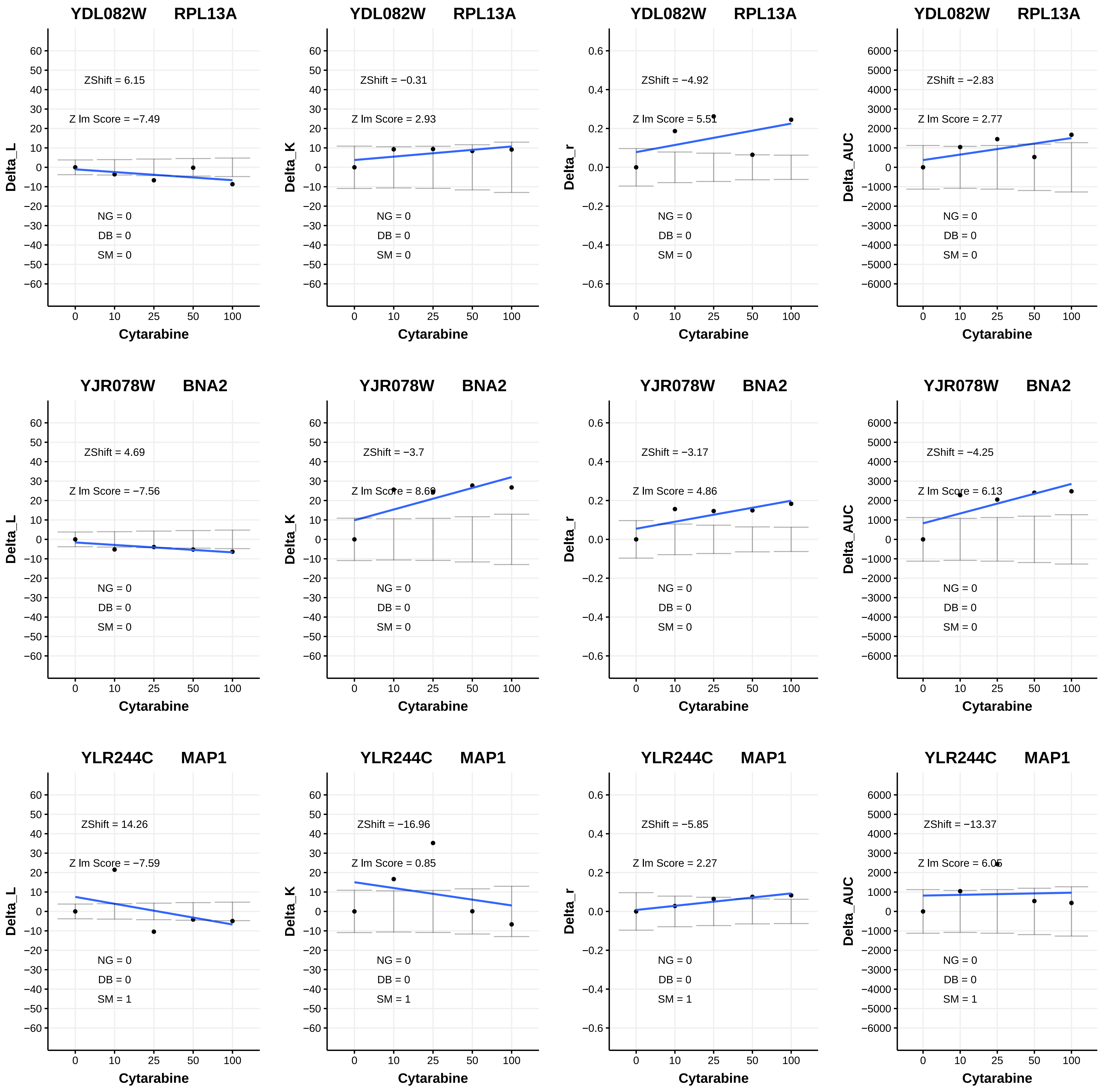

YDL082W RPL13A
ZShift = 6.15
Z lm Score = −7.49
NG = 0
DB = 0
SM = 0
Delta_L
Cytarabine
YDL082W RPL13A
ZShift = −0.31
Z lm Score = 2.93
NG = 0
DB = 0
SM = 0
Delta_K
Cytarabine
YDL082W RPL13A
ZShift = −4.92
Z lm Score = 5.51
NG = 0
DB = 0
SM = 0
Delta_r
Cytarabine
YDL082W RPL13A
ZShift = −2.83
Z lm Score = 2.77
NG = 0
DB = 0
SM = 0
Delta_AUC
Cytarabine
YJR078W BNA2
ZShift = 4.69
Z lm Score = −7.56
NG = 0
DB = 0
SM = 0
Delta_L
Cytarabine
YJR078W BNA2
ZShift = −3.7
Z lm Score = 8.69
NG = 0
DB = 0
SM = 0
Delta_K
Cytarabine
YJR078W BNA2
ZShift = −3.17
Z lm Score = 4.86
NG = 0
DB = 0
SM = 0
Delta_r
Cytarabine
YJR078W BNA2
ZShift = −4.25
Z lm Score = 6.13
NG = 0
DB = 0
SM = 0
Delta_AUC
Cytarabine
YLR244C MAP1
ZShift = 14.26
Z lm Score = −7.59
NG = 0
DB = 0
SM = 1
Delta_L
Cytarabine
YLR244C MAP1
ZShift = −16.96
Z lm Score = 0.85
NG = 0
DB = 0
SM = 1
Delta_K
Cytarabine
YLR244C MAP1
ZShift = −5.85
Z lm Score = 2.27
NG = 0
DB = 0
SM = 1
Delta_r
Cytarabine
YLR244C MAP1
ZShift = −13.37
Z lm Score = 6.05
NG = 0
DB = 0
SM = 1
Delta_AUC
Cytarabine

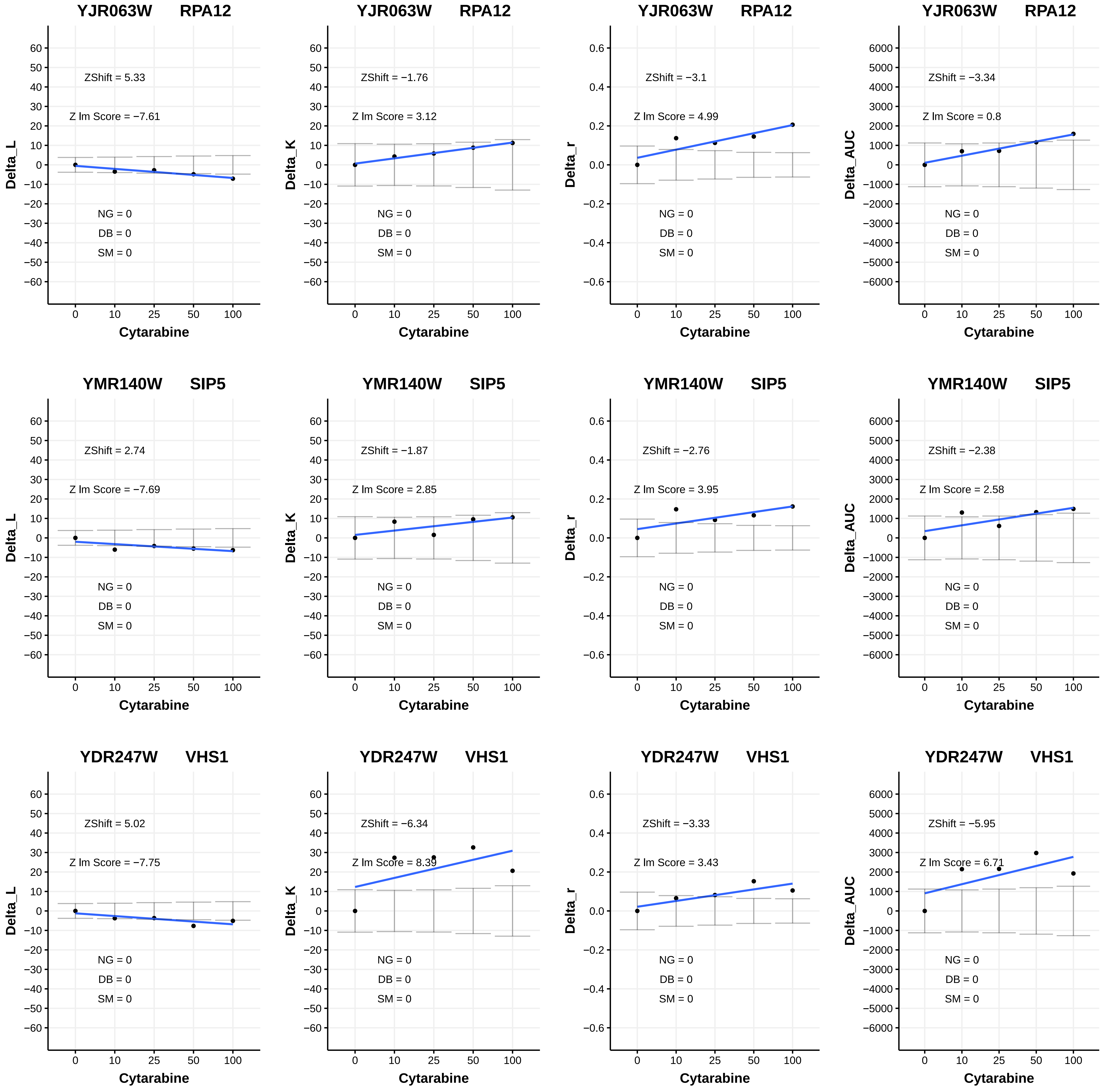
YJR063W RPA12
ZShift = 5.33
Z lm Score = −7.61
NG = 0
DB = 0
SM = 0
Delta_L
Cytarabine
YJR063W RPA12
ZShift = −1.76
Z lm Score = 3.12
NG = 0
DB = 0
SM = 0
Delta_K
Cytarabine
YJR063W RPA12
ZShift = −3.1
Z lm Score = 4.99
NG = 0
DB = 0
SM = 0
Delta_r
Cytarabine
YJR063W RPA12
ZShift = −3.34
Z lm Score = 0.8
NG = 0
DB = 0
SM = 0
Delta_AUC
Cytarabine
YMR140W SIP5
ZShift = 2.74
Z lm Score = −7.69
NG = 0
DB = 0
SM = 0
Delta_L
Cytarabine
YMR140W SIP5
ZShift = −1.87
Z lm Score = 2.85
NG = 0
DB = 0
SM = 0
Delta_K
Cytarabine
YMR140W SIP5
ZShift = −2.76
Z lm Score = 3.95
NG = 0
DB = 0
SM = 0
Delta_r
Cytarabine
YMR140W SIP5
ZShift = −2.38
Z lm Score = 2.58
NG = 0
DB = 0
SM = 0
Delta_AUC
Cytarabine
YDR247W VHS1
ZShift = 5.02
Z lm Score = −7.75
NG = 0
DB = 0
SM = 0
Delta_L
Cytarabine
YDR247W VHS1
ZShift = −6.34
Z lm Score = 8.39
NG = 0
DB = 0
SM = 0
Delta_K
Cytarabine
YDR247W VHS1
ZShift = −3.33
Z lm Score = 3.43
NG = 0
DB = 0
SM = 0
Delta_r
Cytarabine
YDR247W VHS1
ZShift = −5.95
Z lm Score = 6.71
NG = 0
DB = 0
SM = 0
Delta_AUC
Cytarabine

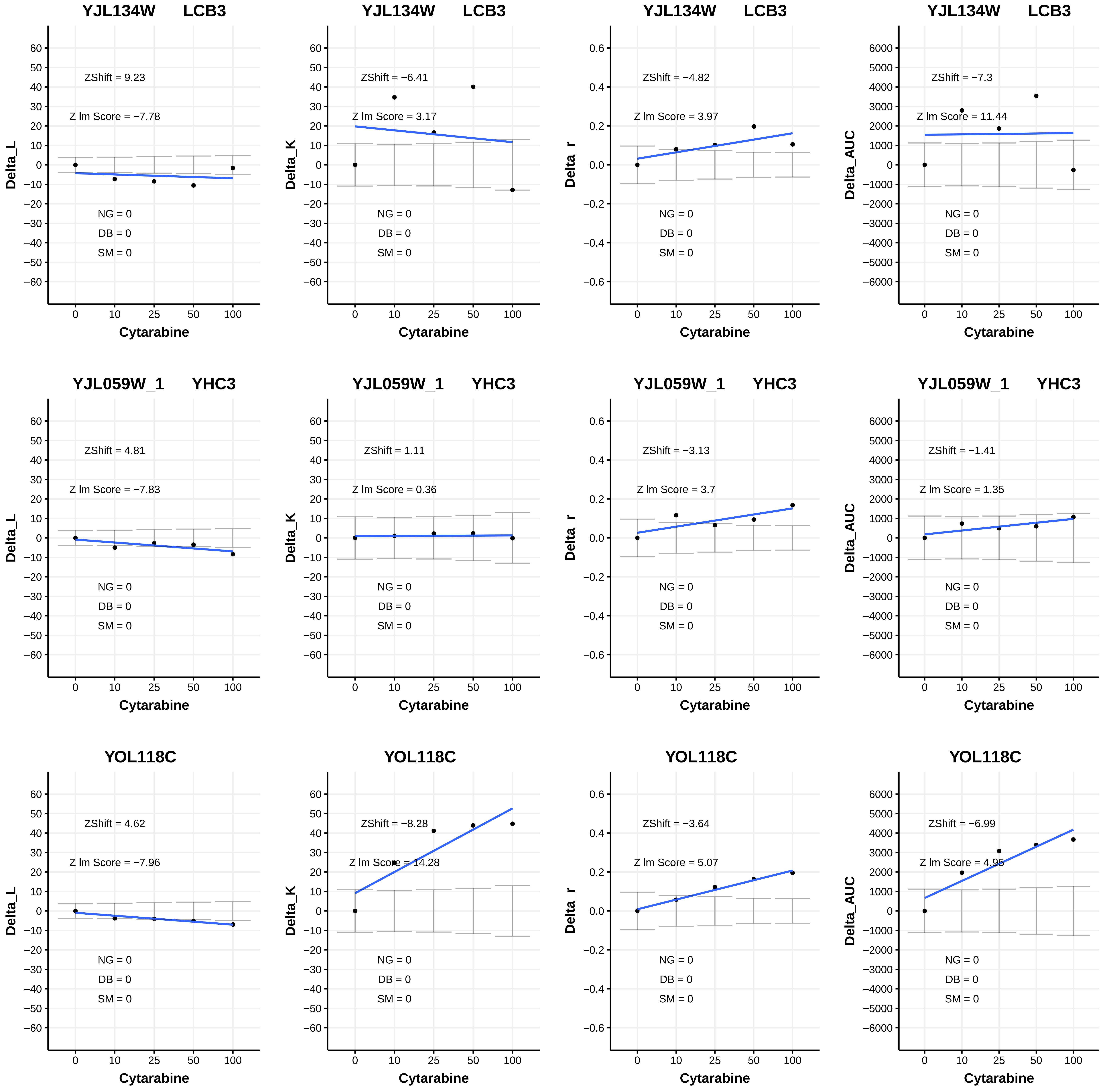
YJL134W LCB3
Delta_L
ZShift = 9.23
Z lm Score = −7.78
NG = 0
DB = 0
SM = 0
Cytarabine
YJL134W LCB3
Delta_K
ZShift = −6.41
Z lm Score = 3.17
NG = 0
DB = 0
SM = 0
Cytarabine
YJL134W LCB3
Delta_r
ZShift = −4.82
Z lm Score = 3.97
NG = 0
DB = 0
SM = 0
Cytarabine
YJL134W LCB3
Delta_AUC
ZShift = −7.3
Z lm Score = 11.44
NG = 0
DB = 0
SM = 0
Cytarabine
YJL059W_1 YHC3
Delta_L
ZShift = 4.81
Z lm Score = −7.83
NG = 0
DB = 0
SM = 0
Cytarabine
YJL059W_1 YHC3
Delta_K
ZShift = 1.11
Z lm Score = 0.36
NG = 0
DB = 0
SM = 0
Cytarabine
YJL059W_1 YHC3
Delta_r
ZShift = −3.13
Z lm Score = 3.7
NG = 0
DB = 0
SM = 0
Cytarabine
YJL059W_1 YHC3
Delta_AUC
ZShift = −1.41
Z lm Score = 1.35
NG = 0
DB = 0
SM = 0
Cytarabine
YOL118C
Delta_L
ZShift = 4.62
Z lm Score = −7.96
NG = 0
DB = 0
SM = 0
Cytarabine
YOL118C
Delta_K
ZShift = −8.28
Z lm Score = 14.28
NG = 0
DB = 0
SM = 0
Cytarabine
YOL118C
Delta_r
ZShift = −3.64
Z lm Score = 5.07
NG = 0
DB = 0
SM = 0
Cytarabine
YOL118C
Delta_AUC
ZShift = −6.99
Z lm Score = 4.95
NG = 0
DB = 0
SM = 0
Cytarabine

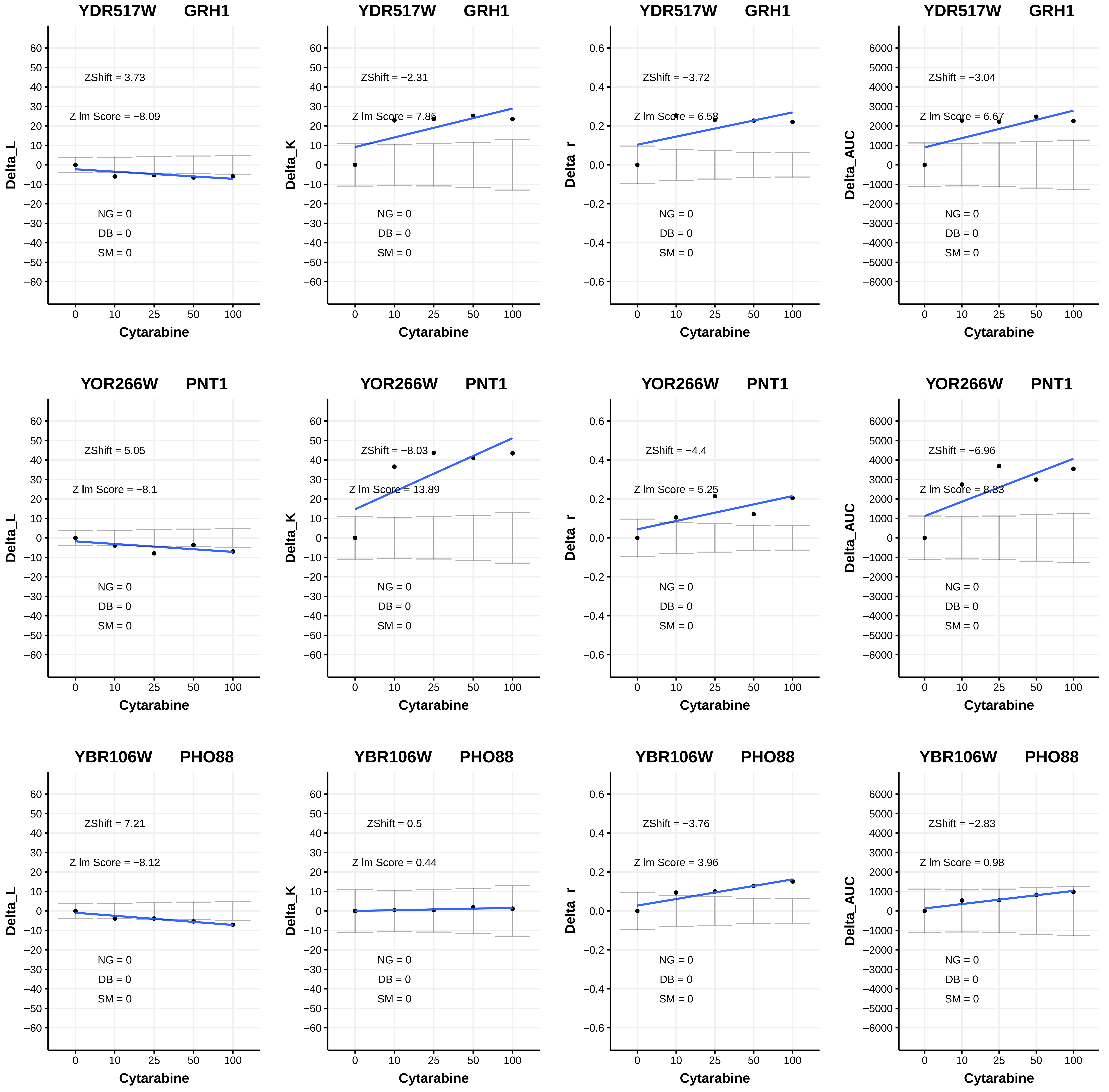
YDR517W GRH1
ZShift = 3.73
Z lm Score = −8.09
NG = 0
DB = 0
SM = 0
Delta_L
Cytarabine
YDR517W GRH1
ZShift = −2.31
Z lm Score = 7.85
NG = 0
DB = 0
SM = 0
Delta_K
Cytarabine
YDR517W GRH1
ZShift = −3.72
Z lm Score = 6.58
NG = 0
DB = 0
SM = 0
Delta_r
Cytarabine
YDR517W GRH1
ZShift = −3.04
Z lm Score = 6.67
NG = 0
DB = 0
SM = 0
Delta_AUC
Cytarabine
YOR266W PNT1
ZShift = 5.05
Z lm Score = −8.1
NG = 0
DB = 0
SM = 0
Delta_L
Cytarabine
YOR266W PNT1
ZShift = −8.03
Z lm Score = 13.89
NG = 0
DB = 0
SM = 0
Delta_K
Cytarabine
YOR266W PNT1
ZShift = −4.4
Z lm Score = 5.25
NG = 0
DB = 0
SM = 0
Delta_r
Cytarabine
YOR266W PNT1
ZShift = −6.96
Z lm Score = 8.33
NG = 0
DB = 0
SM = 0
Delta_AUC
Cytarabine
YBR106W PHO88
ZShift = 7.21
Z lm Score = −8.12
NG = 0
DB = 0
SM = 0
Delta_L
Cytarabine
YBR106W PHO88
ZShift = 0.5
Z lm Score = 0.44
NG = 0
DB = 0
SM = 0
Delta_K
Cytarabine
YBR106W PHO88
ZShift = −3.76
Z lm Score = 3.96
NG = 0
DB = 0
SM = 0
Delta_r
Cytarabine
YBR106W PHO88
ZShift = −2.83
Z lm Score = 0.98
NG = 0
DB = 0
SM = 0
Delta_AUC
Cytarabine

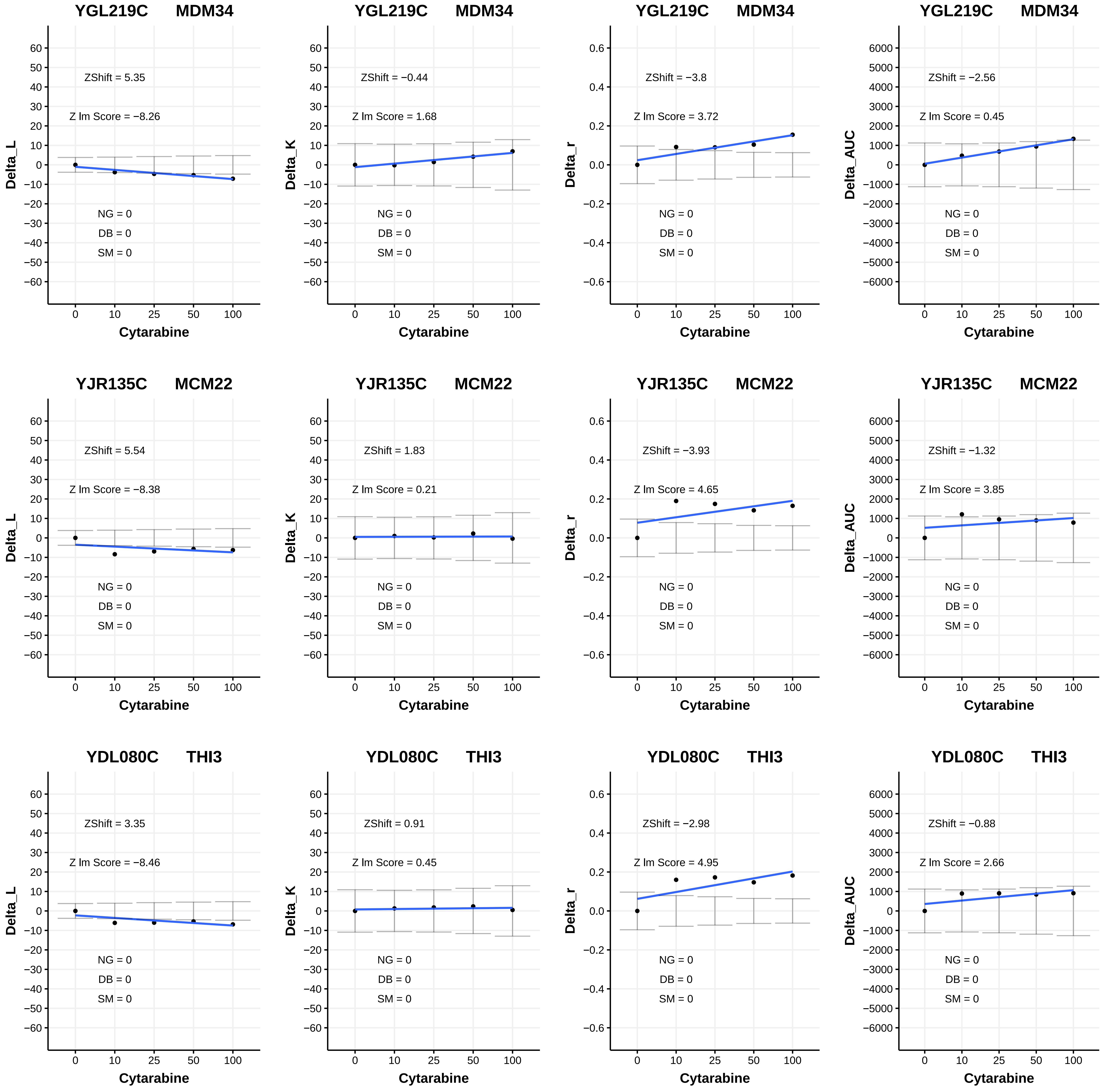

YGL219C MDM34
ZShift = 5.35
Z lm Score = −8.26
NG = 0
DB = 0
SM = 0
Delta_L
Cytarabine
YGL219C MDM34
ZShift = −0.44
Z lm Score = 1.68
NG = 0
DB = 0
SM = 0
Delta_K
Cytarabine
YGL219C MDM34
ZShift = −3.8
Z lm Score = 3.72
NG = 0
DB = 0
SM = 0
Delta_r
Cytarabine
YGL219C MDM34
ZShift = −2.56
Z lm Score = 0.45
NG = 0
DB = 0
SM = 0
Delta_AUC
Cytarabine
YJR135C MCM22
ZShift = 5.54
Z lm Score = −8.38
NG = 0
DB = 0
SM = 0
Delta_L
Cytarabine
YJR135C MCM22
ZShift = 1.83
Z lm Score = 0.21
NG = 0
DB = 0
SM = 0
Delta_K
Cytarabine
YJR135C MCM22
ZShift = −3.93
Z lm Score = 4.65
NG = 0
DB = 0
SM = 0
Delta_r
Cytarabine
YJR135C MCM22
ZShift = −1.32
Z lm Score = 3.85
NG = 0
DB = 0
SM = 0
Delta_AUC
Cytarabine
YDL080C THI3
ZShift = 3.35
Z lm Score = −8.46
NG = 0
DB = 0
SM = 0
Delta_L
Cytarabine
YDL080C THI3
ZShift = 0.91
Z lm Score = 0.45
NG = 0
DB = 0
SM = 0
Delta_K
Cytarabine
YDL080C THI3
ZShift = −2.98
Z lm Score = 4.95
NG = 0
DB = 0
SM = 0
Delta_r
Cytarabine
YDL080C THI3
ZShift = −0.88
Z lm Score = 2.66
NG = 0
DB = 0
SM = 0
Delta_AUC
Cytarabine
0 10 25 50 100

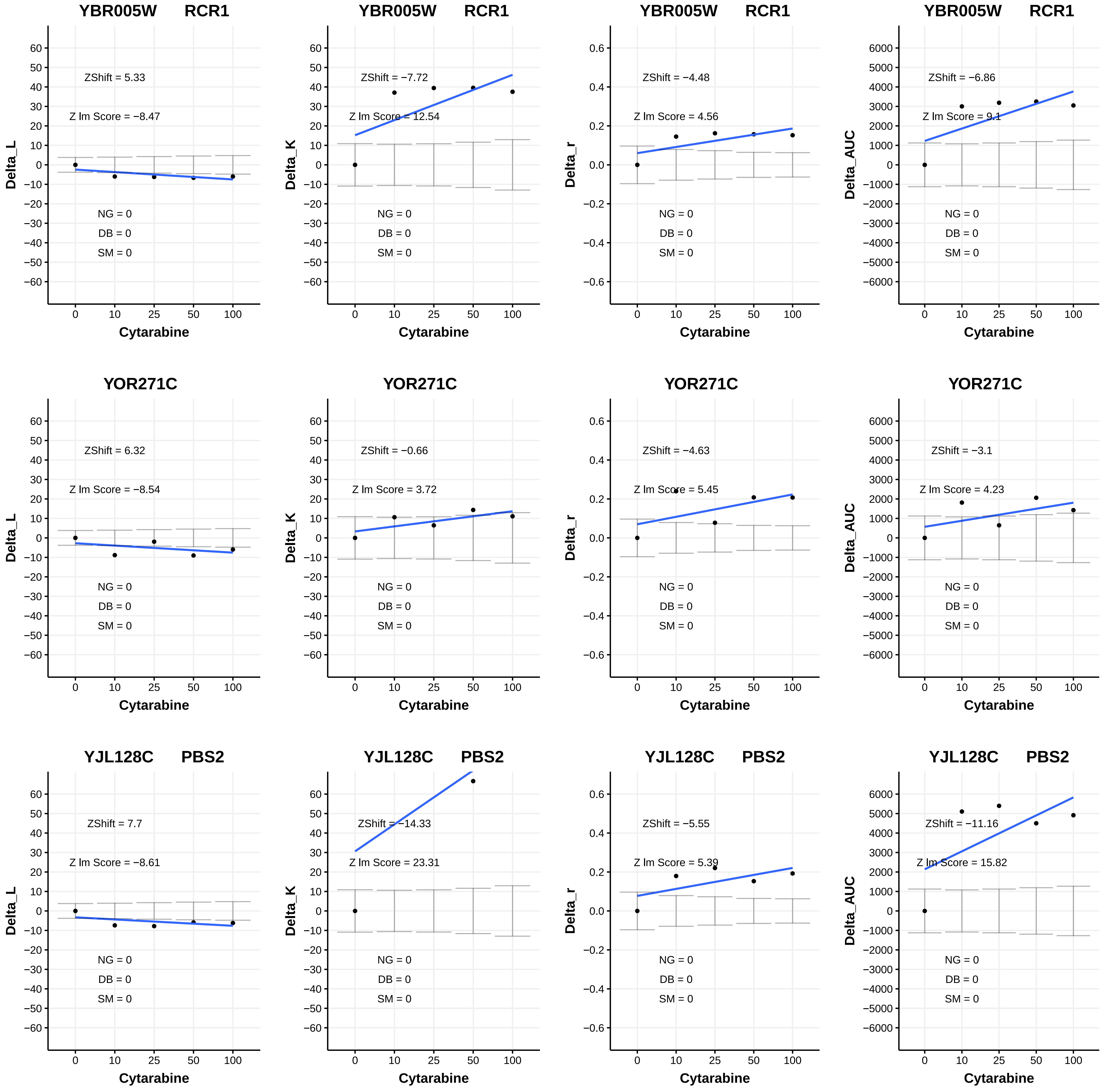

YBR005W RCR1
ZShift = 5.33
Z lm Score = −8.47
NG = 0
DB = 0
SM = 0
Delta_L
Cytarabine
YBR005W RCR1
ZShift = −7.72
Z lm Score = 12.54
NG = 0
DB = 0
SM = 0
Delta_K
Cytarabine
YBR005W RCR1
ZShift = −4.48
Z lm Score = 4.56
NG = 0
DB = 0
SM = 0
Delta_r
Cytarabine
YBR005W RCR1
ZShift = −6.86
Z lm Score = 9.1
NG = 0
DB = 0
SM = 0
Delta_AUC
Cytarabine
YOR271C
ZShift = 6.32
Z lm Score = −8.54
NG = 0
DB = 0
SM = 0
Delta_L
Cytarabine
YOR271C
ZShift = −0.66
Z lm Score = 3.72
NG = 0
DB = 0
SM = 0
Delta_K
Cytarabine
YOR271C
ZShift = −4.63
Z lm Score = 5.45
NG = 0
DB = 0
SM = 0
Delta_r
Cytarabine
YOR271C
ZShift = −3.1
Z lm Score = 4.23
NG = 0
DB = 0
SM = 0
Delta_AUC
Cytarabine
YJL128C PBS2
ZShift = 7.7
Z lm Score = −8.61
NG = 0
DB = 0
SM = 0
Delta_L
Cytarabine
YJL128C PBS2
ZShift = −14.33
Z lm Score = 23.31
NG = 0
DB = 0
SM = 0
Delta_K
Cytarabine
YJL128C PBS2
ZShift = −5.55
Z lm Score = 5.39
NG = 0
DB = 0
SM = 0
Delta_r
Cytarabine
YJL128C PBS2
ZShift = −11.16
Z lm Score = 15.82
NG = 0
DB = 0
SM = 0
Delta_AUC
Cytarabine

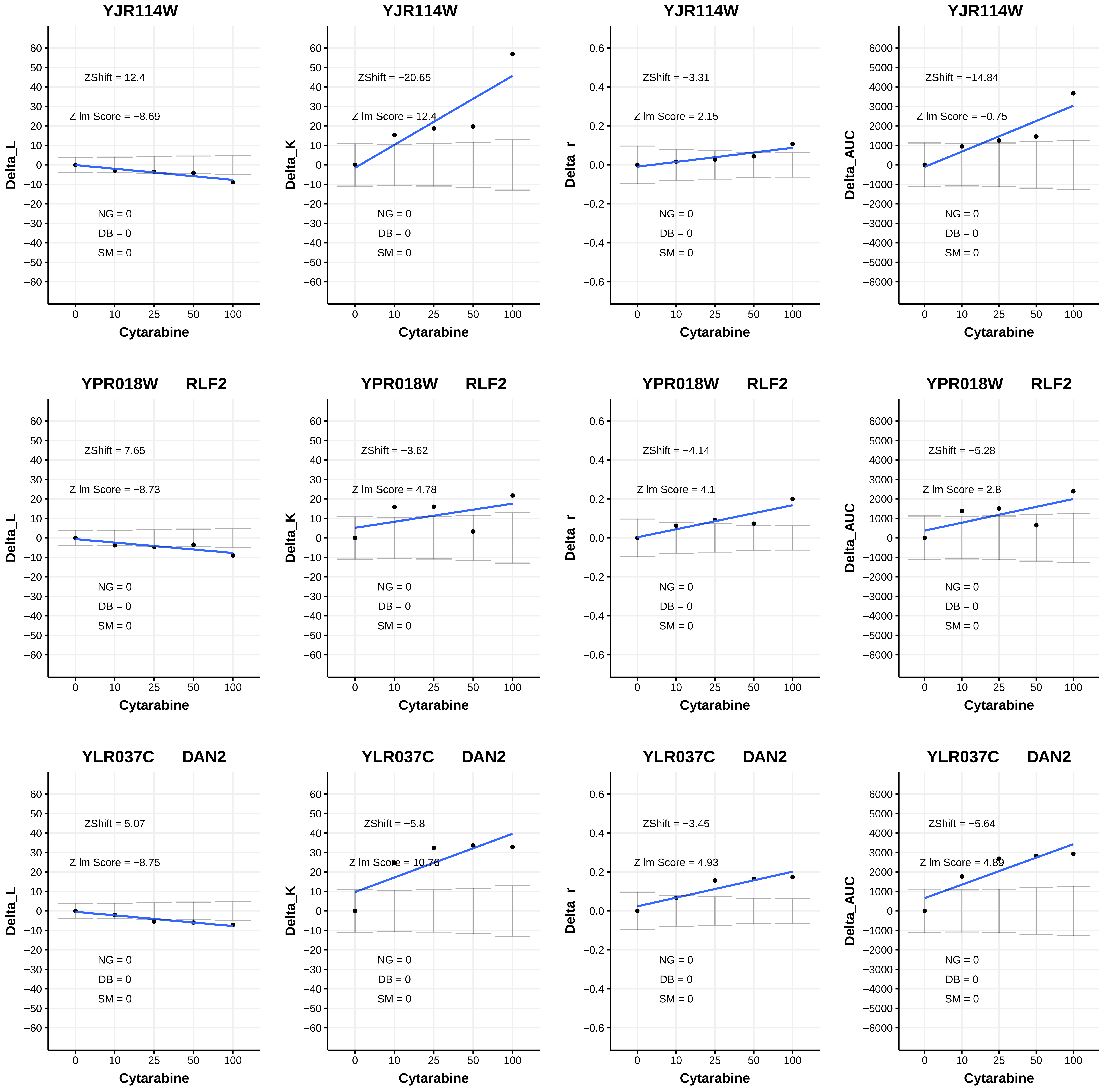

YJR114W
ZShift = 12.4
Z lm Score = −8.69
NG = 0
DB = 0
SM = 0
Delta_L
Cytarabine
YJR114W
ZShift = −20.65
Z lm Score = 12.4
NG = 0
DB = 0
SM = 0
Delta_K
Cytarabine
YJR114W
ZShift = −3.31
Z lm Score = 2.15
NG = 0
DB = 0
SM = 0
Delta_r
Cytarabine
YJR114W
ZShift = −14.84
Z lm Score = −0.75
NG = 0
DB = 0
SM = 0
Delta_AUC
Cytarabine
YPR018W RLF2
ZShift = 7.65
Z lm Score = −8.73
NG = 0
DB = 0
SM = 0
Delta_L
Cytarabine
YPR018W RLF2
ZShift = −3.62
Z lm Score = 4.78
NG = 0
DB = 0
SM = 0
Delta_K
Cytarabine
YPR018W RLF2
ZShift = −4.14
Z lm Score = 4.1
NG = 0
DB = 0
SM = 0
Delta_r
Cytarabine
YPR018W RLF2
ZShift = −5.28
Z lm Score = 2.8
NG = 0
DB = 0
SM = 0
Delta_AUC
Cytarabine
YLR037C DAN2
ZShift = 5.07
Z lm Score = −8.75
NG = 0
DB = 0
SM = 0
Delta_L
Cytarabine
YLR037C DAN2
ZShift = −5.8
Z lm Score = 10.76
NG = 0
DB = 0
SM = 0
Delta_K
Cytarabine
YLR037C DAN2
ZShift = −3.45
Z lm Score = 4.93
NG = 0
DB = 0
SM = 0
Delta_r
Cytarabine
YLR037C DAN2
ZShift = −5.64
Z lm Score = 4.89
NG = 0
DB = 0
SM = 0
Delta_AUC
Cytarabine

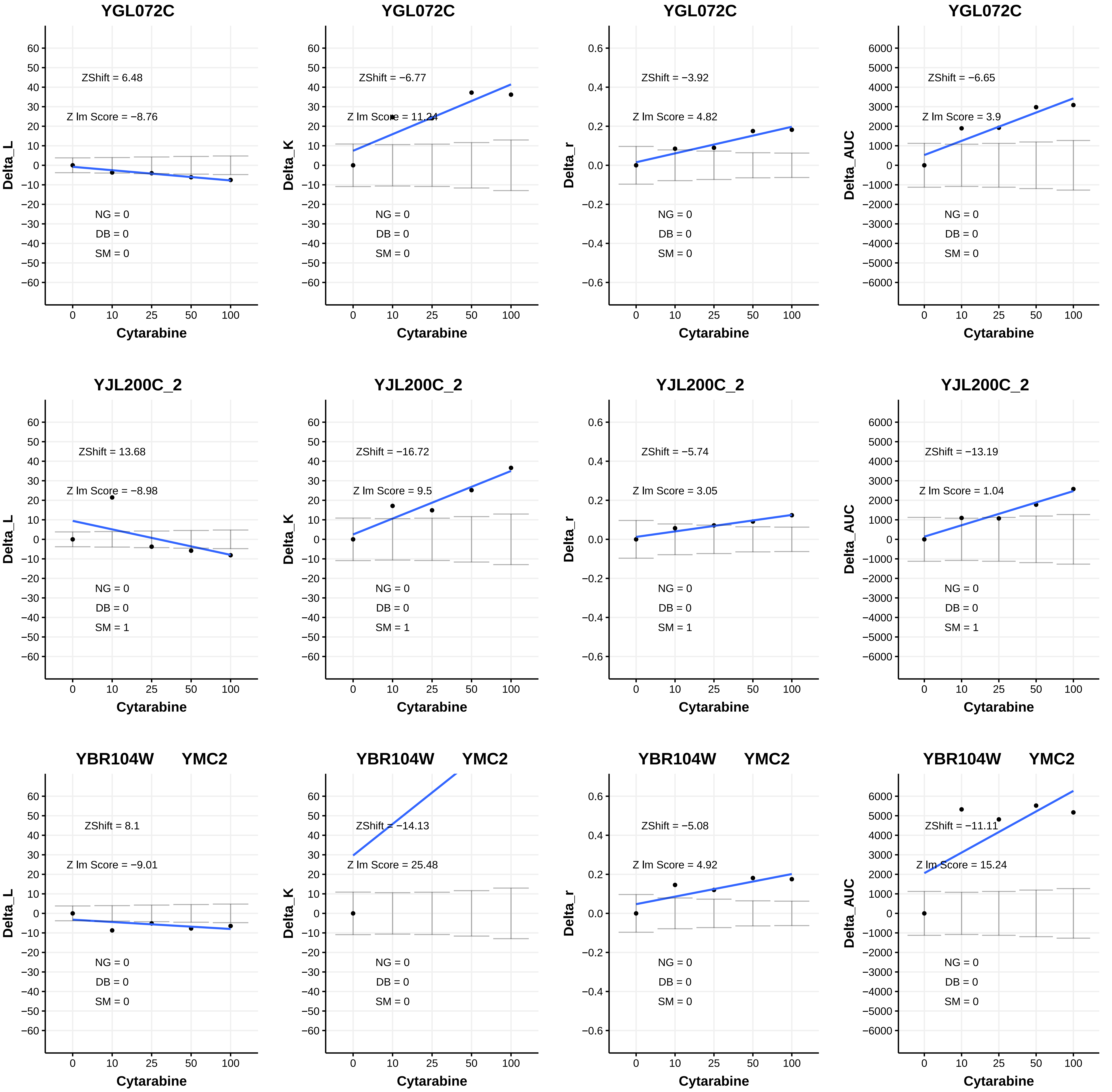
YGL072C
ZShift = 6.48
Z lm Score = −8.76
NG = 0
DB = 0
SM = 0
Delta_L
Cytarabine
YGL072C
ZShift = −6.77
Z lm Score = 11.24
NG = 0
DB = 0
SM = 0
Delta_K
Cytarabine
YGL072C
ZShift = −3.92
Z lm Score = 4.82
NG = 0
DB = 0
SM = 0
Delta_r
Cytarabine
YGL072C
ZShift = −6.65
Z lm Score = 3.9
NG = 0
DB = 0
SM = 0
Delta_AUC
Cytarabine
YJL200C_2
ZShift = 13.68
Z lm Score = −8.98
NG = 0
DB = 0
SM = 1
Delta_L
Cytarabine
YJL200C_2
ZShift = −16.72
Z lm Score = 9.5
NG = 0
DB = 0
SM = 1
Delta_K
Cytarabine
YJL200C_2
ZShift = −5.74
Z lm Score = 3.05
NG = 0
DB = 0
SM = 1
Delta_r
Cytarabine
YJL200C_2
ZShift = −13.19
Z lm Score = 1.04
NG = 0
DB = 0
SM = 1
Delta_AUC
Cytarabine
YBR104W YMC2
ZShift = 8.1
Z lm Score = −9.01
NG = 0
DB = 0
SM = 0
Delta_L
Cytarabine
YBR104W YMC2
ZShift = −14.13
Z lm Score = 25.48
NG = 0
DB = 0
SM = 0
Delta_K
Cytarabine
YBR104W YMC2
ZShift = −5.08
Z lm Score = 4.92
NG = 0
DB = 0
SM = 0
Delta_r
Cytarabine
YBR104W YMC2
ZShift = −11.11
Z lm Score = 15.24
NG = 0
DB = 0
SM = 0
Delta_AUC
Cytarabine

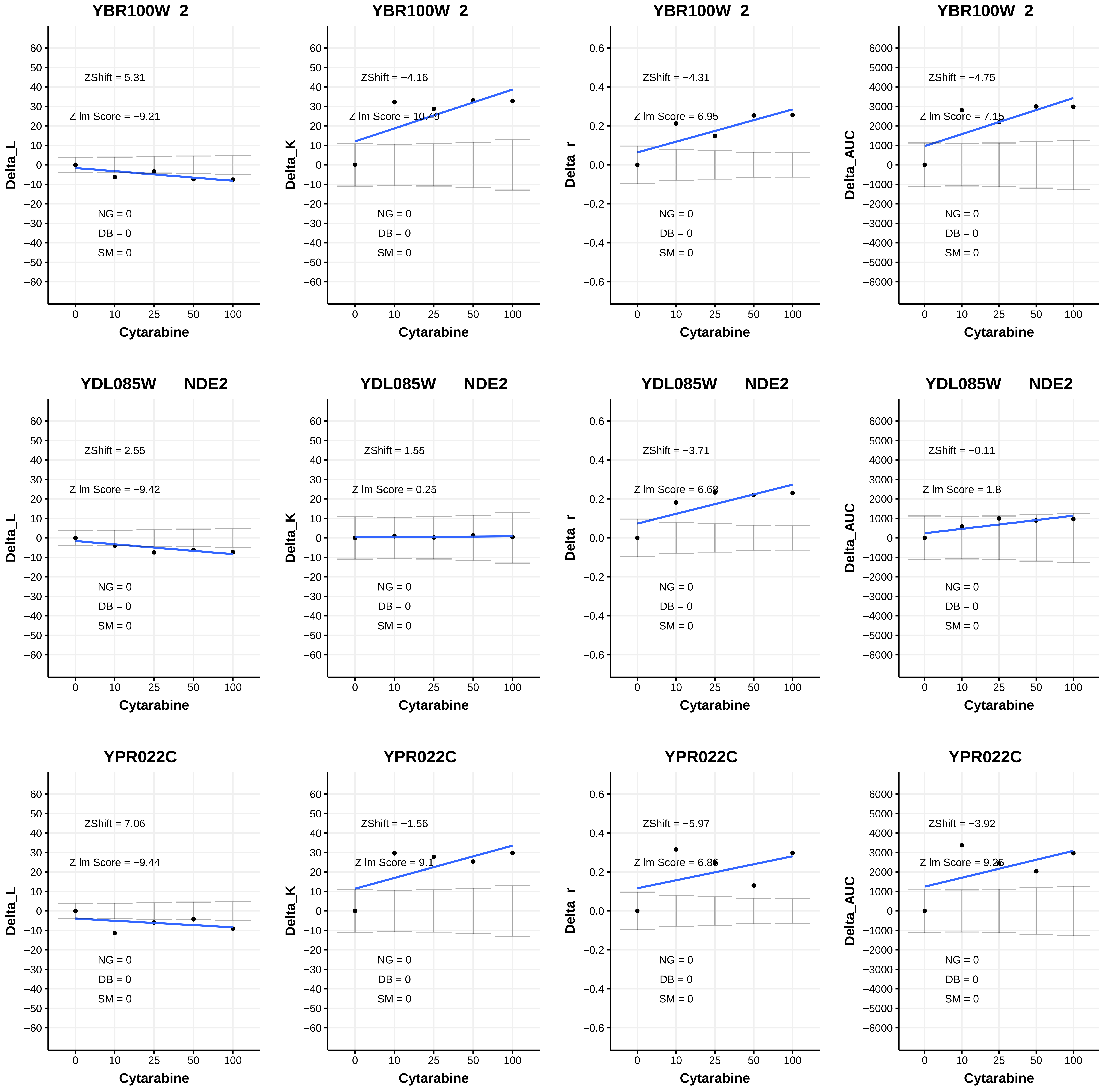

YBR100W_2
ZShift = 5.31
Z lm Score = −9.21
NG = 0
DB = 0
SM = 0
Delta_L
Cytarabine
YBR100W_2
ZShift = −4.16
Z lm Score = 10.49
NG = 0
DB = 0
SM = 0
Delta_K
Cytarabine
YBR100W_2
ZShift = −4.31
Z lm Score = 6.95
NG = 0
DB = 0
SM = 0
Delta_r
Cytarabine
YBR100W_2
ZShift = −4.75
Z lm Score = 7.15
NG = 0
DB = 0
SM = 0
Delta_AUC
Cytarabine
YDL085W NDE2
ZShift = 2.55
Z lm Score = −9.42
NG = 0
DB = 0
SM = 0
Delta_L
Cytarabine
YDL085W NDE2
ZShift = 1.55
Z lm Score = 0.25
NG = 0
DB = 0
SM = 0
Delta_K
Cytarabine
YDL085W NDE2
ZShift = −3.71
Z lm Score = 6.68
NG = 0
DB = 0
SM = 0
Delta_r
Cytarabine
YDL085W NDE2
ZShift = −0.11
Z lm Score = 1.8
NG = 0
DB = 0
SM = 0
Delta_AUC
Cytarabine
YPR022C
ZShift = 7.06
Z lm Score = −9.44
NG = 0
DB = 0
SM = 0
Delta_L
Cytarabine
YPR022C
ZShift = −1.56
Z lm Score = 9.1
NG = 0
DB = 0
SM = 0
Delta_K
Cytarabine
YPR022C
ZShift = −5.97
Z lm Score = 6.86
NG = 0
DB = 0
SM = 0
Delta_r
Cytarabine
YPR022C
ZShift = −3.92
Z lm Score = 9.25
NG = 0
DB = 0
SM = 0
Delta_AUC
Cytarabine

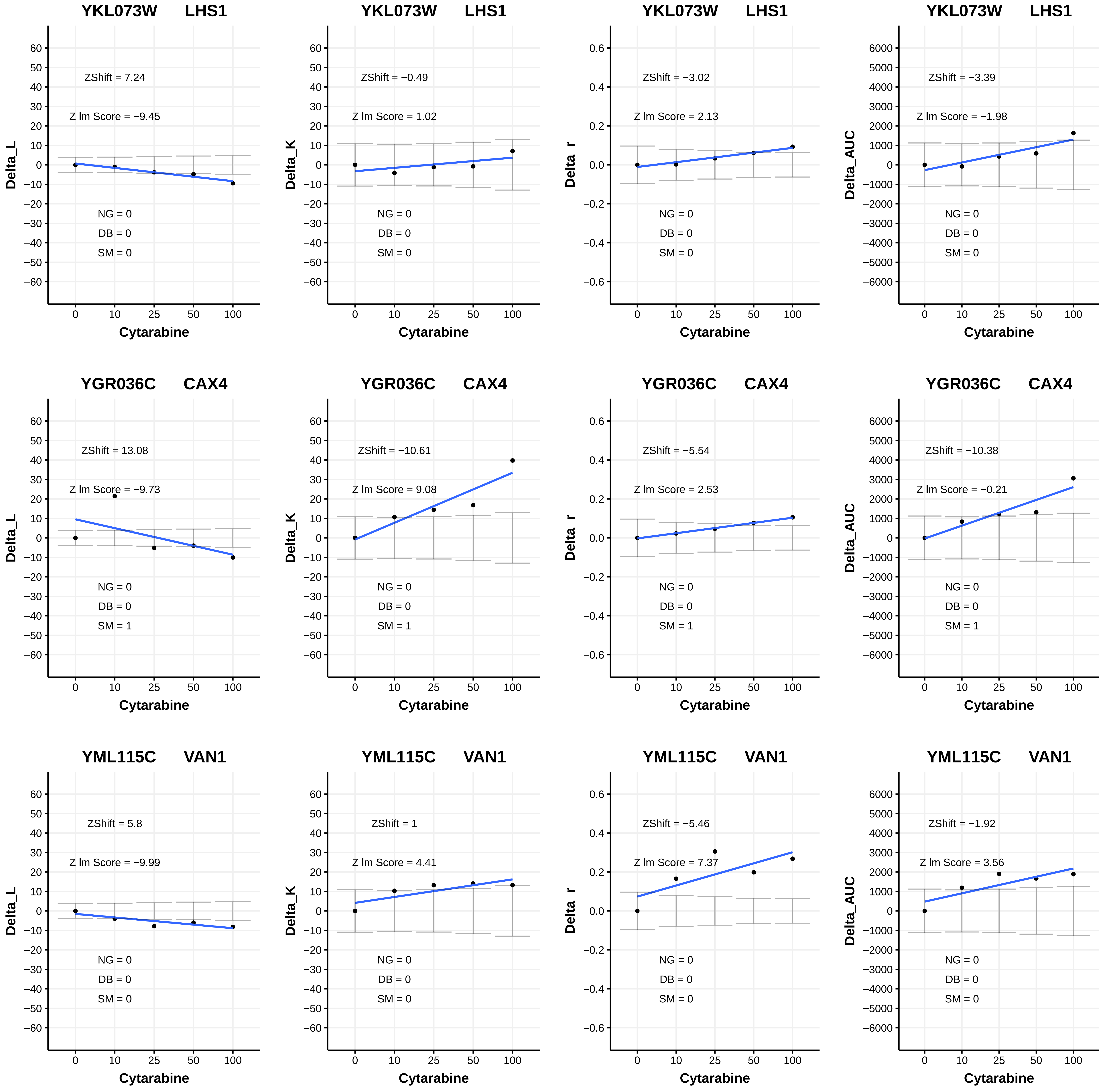

YKL073W LHS1
ZShift = 7.24
Z lm Score = −9.45
NG = 0
DB = 0
SM = 0
Delta_L
Cytarabine
YKL073W LHS1
ZShift = −0.49
Z lm Score = 1.02
NG = 0
DB = 0
SM = 0
Delta_K
Cytarabine
YKL073W LHS1
ZShift = −3.02
Z lm Score = 2.13
NG = 0
DB = 0
SM = 0
Delta_r
Cytarabine
YKL073W LHS1
ZShift = −3.39
Z lm Score = −1.98
NG = 0
DB = 0
SM = 0
Delta_AUC
Cytarabine
YGR036C CAX4
ZShift = 13.08
Z lm Score = −9.73
NG = 0
DB = 0
SM = 1
Delta_L
Cytarabine
YGR036C CAX4
ZShift = −10.61
Z lm Score = 9.08
NG = 0
DB = 0
SM = 1
Delta_K
Cytarabine
YGR036C CAX4
ZShift = −5.54
Z lm Score = 2.53
NG = 0
DB = 0
SM = 1
Delta_r
Cytarabine
YGR036C CAX4
ZShift = −10.38
Z lm Score = −0.21
NG = 0
DB = 0
SM = 1
Delta_AUC
Cytarabine
YML115C VAN1
ZShift = 5.8
Z lm Score = −9.99
NG = 0
DB = 0
SM = 0
Delta_L
Cytarabine
YML115C VAN1
ZShift = 1
Z lm Score = 4.41
NG = 0
DB = 0
SM = 0
Delta_K
Cytarabine
YML115C VAN1
ZShift = −5.46
Z lm Score = 7.37
NG = 0
DB = 0
SM = 0
Delta_r
Cytarabine
YML115C VAN1
ZShift = −1.92
Z lm Score = 3.56
NG = 0
DB = 0
SM = 0
Delta_AUC
Cytarabine

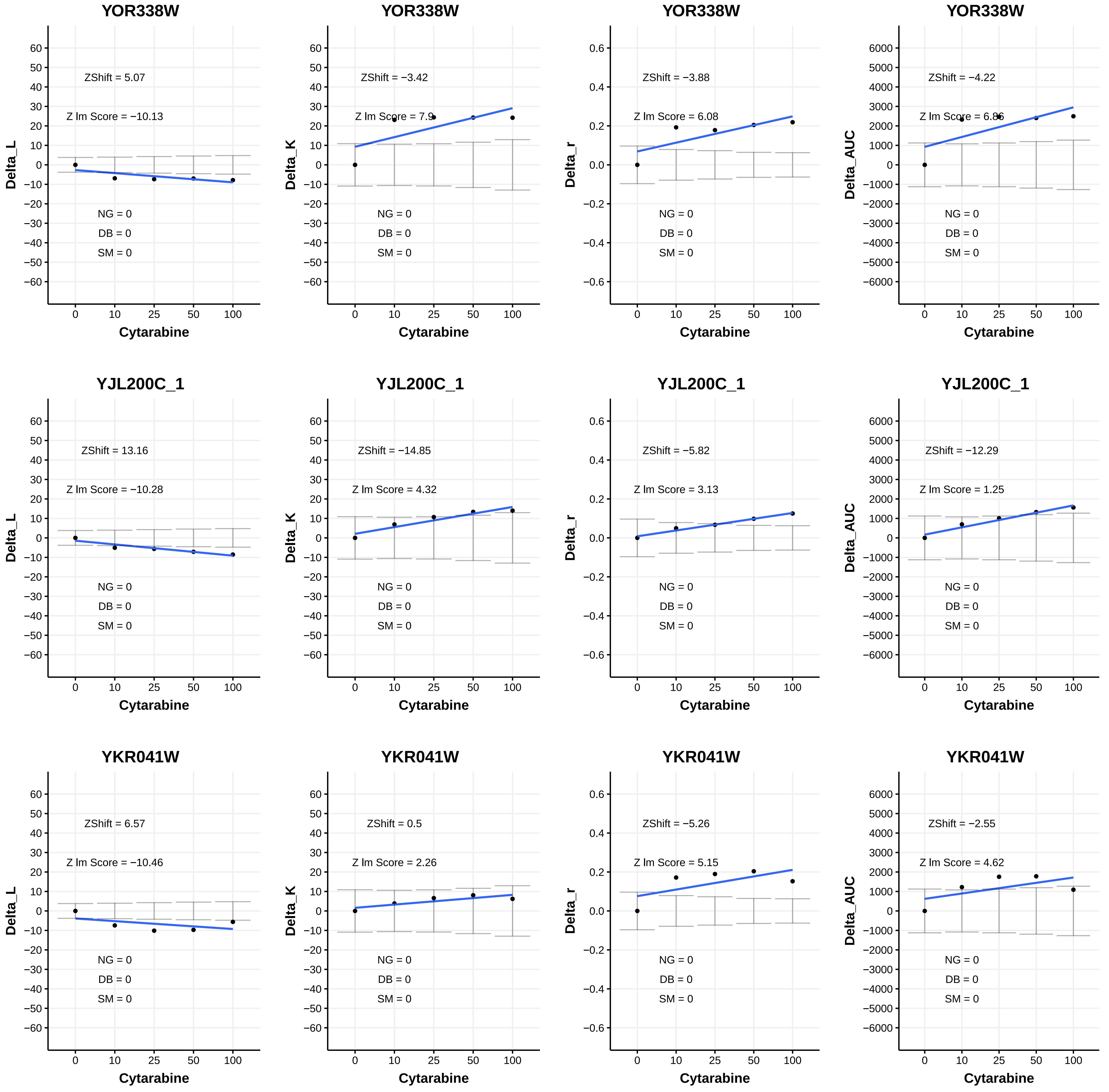
YOR338W
ZShift = 5.07
Z lm Score = −10.13
NG = 0
DB = 0
SM = 0
Delta_L
Cytarabine
YOR338W
ZShift = −3.42
Z lm Score = 7.9
NG = 0
DB = 0
SM = 0
Delta_K
Cytarabine
YOR338W
ZShift = −3.88
Z lm Score = 6.08
NG = 0
DB = 0
SM = 0
Delta_r
Cytarabine
YOR338W
ZShift = −4.22
Z lm Score = 6.86
NG = 0
DB = 0
SM = 0
Delta_AUC
Cytarabine
YJL200C_1
ZShift = 13.16
Z lm Score = −10.28
NG = 0
DB = 0
SM = 0
Delta_L
Cytarabine
YJL200C_1
ZShift = −14.85
Z lm Score = 4.32
NG = 0
DB = 0
SM = 0
Delta_K
Cytarabine
YJL200C_1
ZShift = −5.82
Z lm Score = 3.13
NG = 0
DB = 0
SM = 0
Delta_r
Cytarabine
YJL200C_1
ZShift = −12.29
Z lm Score = 1.25
NG = 0
DB = 0
SM = 0
Delta_AUC
Cytarabine
YKR041W
ZShift = 6.57
Z lm Score = −10.46
NG = 0
DB = 0
SM = 0
Delta_L
Cytarabine
YKR041W
ZShift = 0.5
Z lm Score = 2.26
NG = 0
DB = 0
SM = 0
Delta_K
Cytarabine
YKR041W
ZShift = −5.26
Z lm Score = 5.15
NG = 0
DB = 0
SM = 0
Delta_r
Cytarabine
YKR041W
ZShift = −2.55
Z lm Score = 4.62
NG = 0
DB = 0
SM = 0
Delta_AUC
Cytarabine

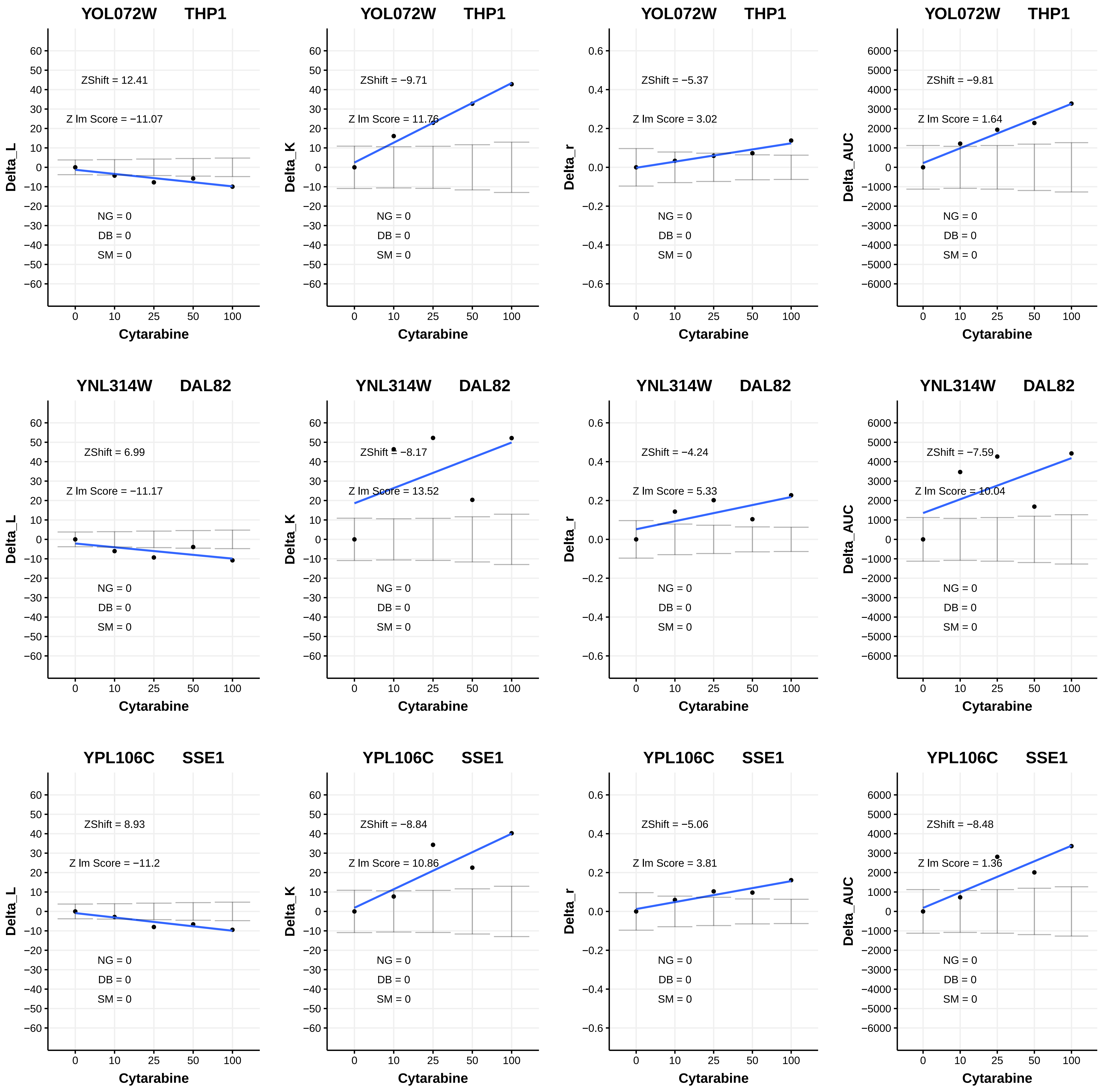

YOL072W THP1
ZShift = 12.41
Z lm Score = −11.07
NG = 0
DB = 0
SM = 0
Delta_L
Cytarabine
YOL072W THP1
ZShift = −9.71
Z lm Score = 11.76
NG = 0
DB = 0
SM = 0
Delta_K
Cytarabine
YOL072W THP1
ZShift = −5.37
Z lm Score = 3.02
NG = 0
DB = 0
SM = 0
Delta_r
Cytarabine
YOL072W THP1
ZShift = −9.81
Z lm Score = 1.64
NG = 0
DB = 0
SM = 0
Delta_AUC
Cytarabine
YNL314W DAL82
ZShift = 6.99
Z lm Score = −11.17
NG = 0
DB = 0
SM = 0
Delta_L
Cytarabine
YNL314W DAL82
ZShift = −8.17
Z lm Score = 13.52
NG = 0
DB = 0
SM = 0
Delta_K
Cytarabine
YNL314W DAL82
ZShift = −4.24
Z lm Score = 5.33
NG = 0
DB = 0
SM = 0
Delta_r
Cytarabine
YNL314W DAL82
ZShift = −7.59
Z lm Score = 10.04
NG = 0
DB = 0
SM = 0
Delta_AUC
Cytarabine
YPL106C SSE1
ZShift = 8.93
Z lm Score = −11.2
NG = 0
DB = 0
SM = 0
Delta_L
Cytarabine
YPL106C SSE1
ZShift = −8.84
Z lm Score = 10.86
NG = 0
DB = 0
SM = 0
Delta_K
Cytarabine
YPL106C SSE1
ZShift = −5.06
Z lm Score = 3.81
NG = 0
DB = 0
SM = 0
Delta_r
Cytarabine
YPL106C SSE1
ZShift = −8.48
Z lm Score = 1.36
NG = 0
DB = 0
SM = 0
Delta_AUC
Cytarabine

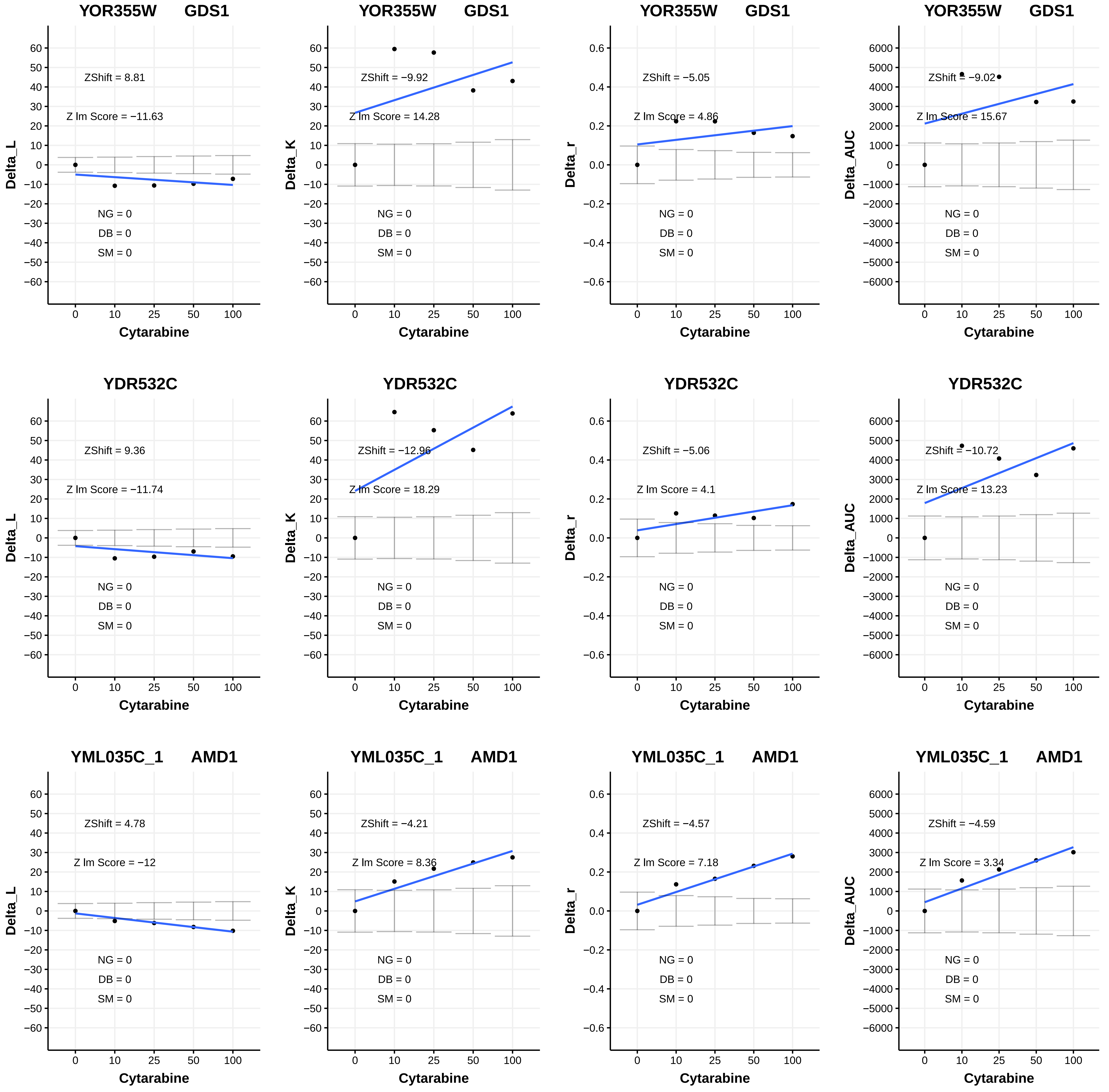

YOR355W GDS1
ZShift = 8.81
Z lm Score = −11.63
NG = 0
DB = 0
SM = 0
Delta_L
Cytarabine
YOR355W GDS1
ZShift = −9.92
Z lm Score = 14.28
NG = 0
DB = 0
SM = 0
Delta_K
Cytarabine
YOR355W GDS1
ZShift = −5.05
Z lm Score = 4.86
NG = 0
DB = 0
SM = 0
Delta_r
Cytarabine
YOR355W GDS1
ZShift = −9.02
Z lm Score = 15.67
NG = 0
DB = 0
SM = 0
Delta_AUC
Cytarabine
YDR532C
ZShift = 9.36
Z lm Score = −11.74
NG = 0
DB = 0
SM = 0
Delta_L
Cytarabine
YDR532C
ZShift = −12.96
Z lm Score = 18.29
NG = 0
DB = 0
SM = 0
Delta_K
Cytarabine
YDR532C
ZShift = −5.06
Z lm Score = 4.1
NG = 0
DB = 0
SM = 0
Delta_r
Cytarabine
YDR532C
ZShift = −10.72
Z lm Score = 13.23
NG = 0
DB = 0
SM = 0
Delta_AUC
Cytarabine
YML035C_1 AMD1
ZShift = 4.78
Z lm Score = −12
NG = 0
DB = 0
SM = 0
Delta_L
Cytarabine
YML035C_1 AMD1
ZShift = −4.21
Z lm Score = 8.36
NG = 0
DB = 0
SM = 0
Delta_K
Cytarabine
YML035C_1 AMD1
ZShift = −4.57
Z lm Score = 7.18
NG = 0
DB = 0
SM = 0
Delta_r
Cytarabine
YML035C_1 AMD1
ZShift = −4.59
Z lm Score = 3.34
NG = 0
DB = 0
SM = 0
Delta_AUC
Cytarabine

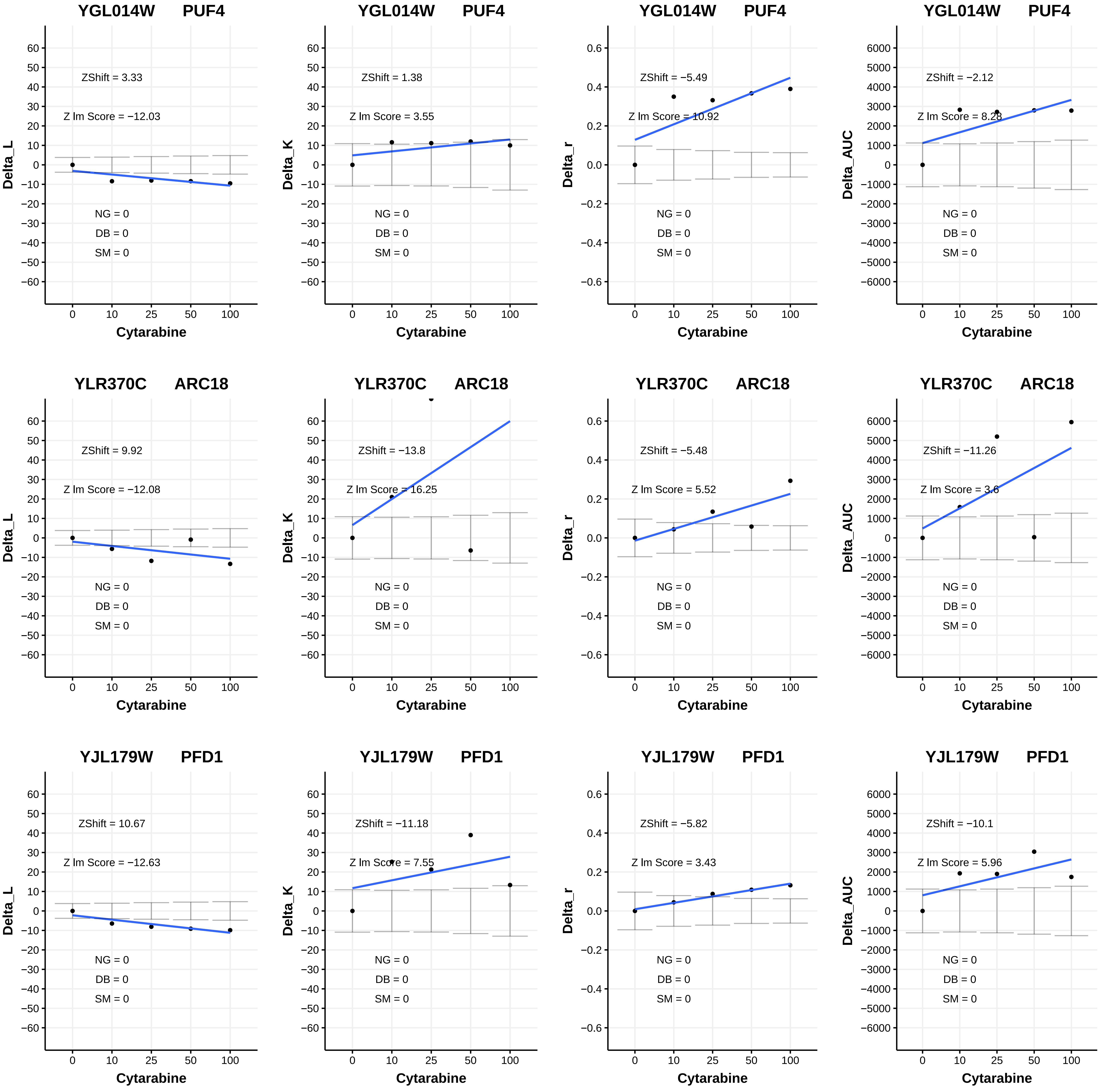

YGL014W PUF4
ZShift = 3.33
Z lm Score = −12.03
NG = 0
DB = 0
SM = 0
Delta_L
Cytarabine
YGL014W PUF4
ZShift = 1.38
Z lm Score = 3.55
NG = 0
DB = 0
SM = 0
Delta_K
Cytarabine
YGL014W PUF4
ZShift = −5.49
Z lm Score = 10.92
NG = 0
DB = 0
SM = 0
Delta_r
Cytarabine
YGL014W PUF4
ZShift = −2.12
Z lm Score = 8.28
NG = 0
DB = 0
SM = 0
Delta_AUC
Cytarabine
YLR370C ARC18
ZShift = 9.92
Z lm Score = −12.08
NG = 0
DB = 0
SM = 0
Delta_L
Cytarabine
YLR370C ARC18
ZShift = −13.8
Z lm Score = 16.25
NG = 0
DB = 0
SM = 0
Delta_K
Cytarabine
YLR370C ARC18
ZShift = −5.48
Z lm Score = 5.52
NG = 0
DB = 0
SM = 0
Delta_r
Cytarabine
YLR370C ARC18
ZShift = −11.26
Z lm Score = 3.6
NG = 0
DB = 0
SM = 0
Delta_AUC
Cytarabine
YJL179W PFD1
ZShift = 10.67
Z lm Score = −12.63
NG = 0
DB = 0
SM = 0
Delta_L
Cytarabine
YJL179W PFD1
ZShift = −11.18
Z lm Score = 7.55
NG = 0
DB = 0
SM = 0
Delta_K
Cytarabine
YJL179W PFD1
ZShift = −5.82
Z lm Score = 3.43
NG = 0
DB = 0
SM = 0
Delta_r
Cytarabine
YJL179W PFD1
ZShift = −10.1
Z lm Score = 5.96
NG = 0
DB = 0
SM = 0
Delta_AUC
Cytarabine

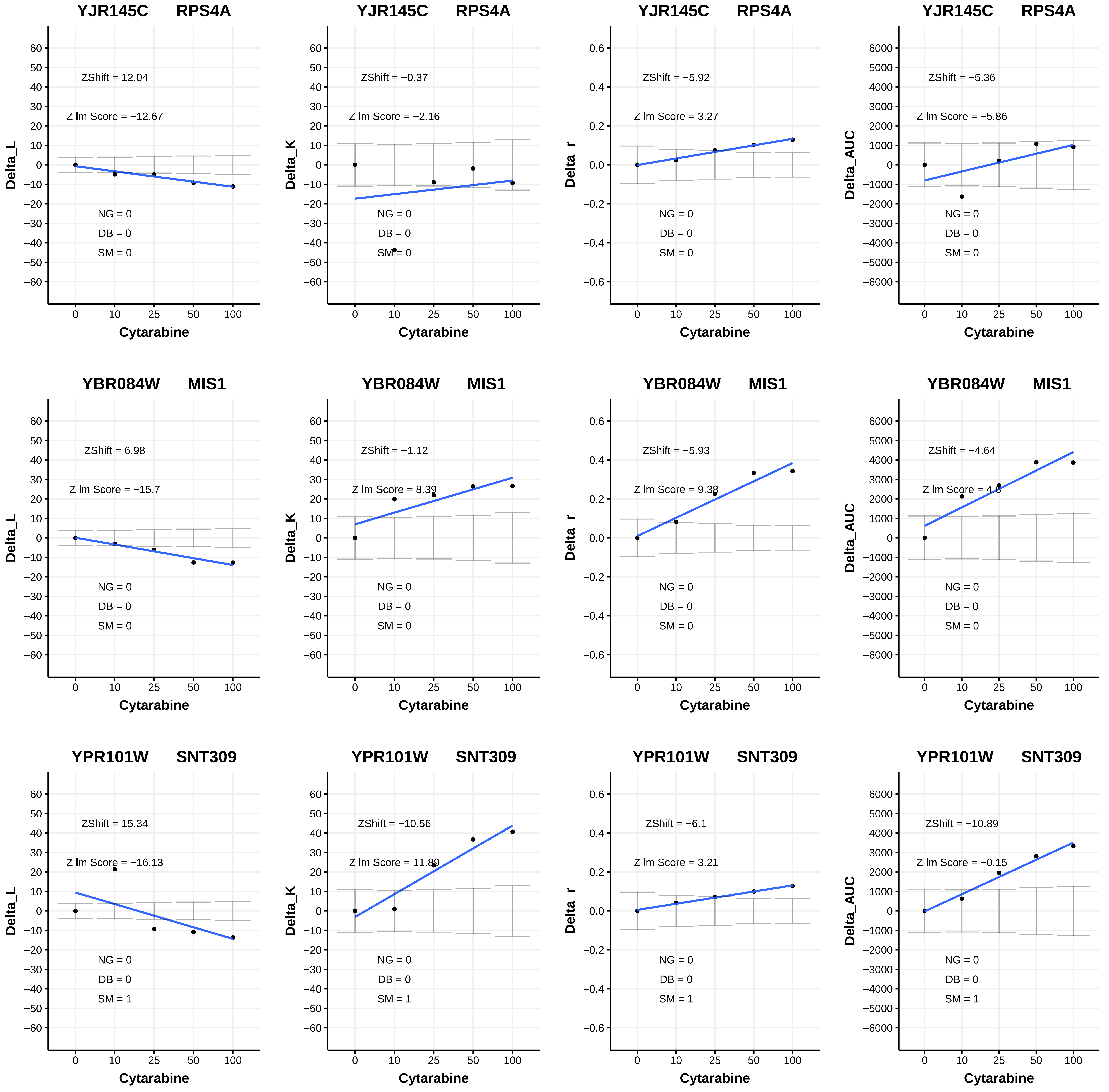

YJR145C RPS4A
ZShift = 12.04
Z lm Score = −12.67
NG = 0
DB = 0
SM = 0
Delta_L
Cytarabine
YJR145C RPS4A
ZShift = −0.37
Z lm Score = −2.16
NG = 0
DB = 0
SM = 0
Delta_K
Cytarabine
YJR145C RPS4A
ZShift = −5.92
Z lm Score = 3.27
NG = 0
DB = 0
SM = 0
Delta_r
Cytarabine
YJR145C RPS4A
ZShift = −5.36
Z lm Score = −5.86
NG = 0
DB = 0
SM = 0
Delta_AUC
Cytarabine
YBR084W MIS1
ZShift = 6.98
Z lm Score = −15.7
NG = 0
DB = 0
SM = 0
Delta_L
Cytarabine
YBR084W MIS1
ZShift = −1.12
Z lm Score = 8.39
NG = 0
DB = 0
SM = 0
Delta_K
Cytarabine
YBR084W MIS1
ZShift = −5.93
Z lm Score = 9.38
NG = 0
DB = 0
SM = 0
Delta_r
Cytarabine
YBR084W MIS1
ZShift = −4.64
Z lm Score = 4.6
NG = 0
DB = 0
SM = 0
Delta_AUC
Cytarabine
YPR101W SNT309
ZShift = 15.34
Z lm Score = −16.13
NG = 0
DB = 0
SM = 1
Delta_L
Cytarabine
YPR101W SNT309
ZShift = −10.56
Z lm Score = 11.89
NG = 0
DB = 0
SM = 1
Delta_K
Cytarabine
YPR101W SNT309
ZShift = −6.1
Z lm Score = 3.21
NG = 0
DB = 0
SM = 1
Delta_r
Cytarabine
YPR101W SNT309
ZShift = −10.89
Z lm Score = −0.15
NG = 0
DB = 0
SM = 1
Delta_AUC
Cytarabine

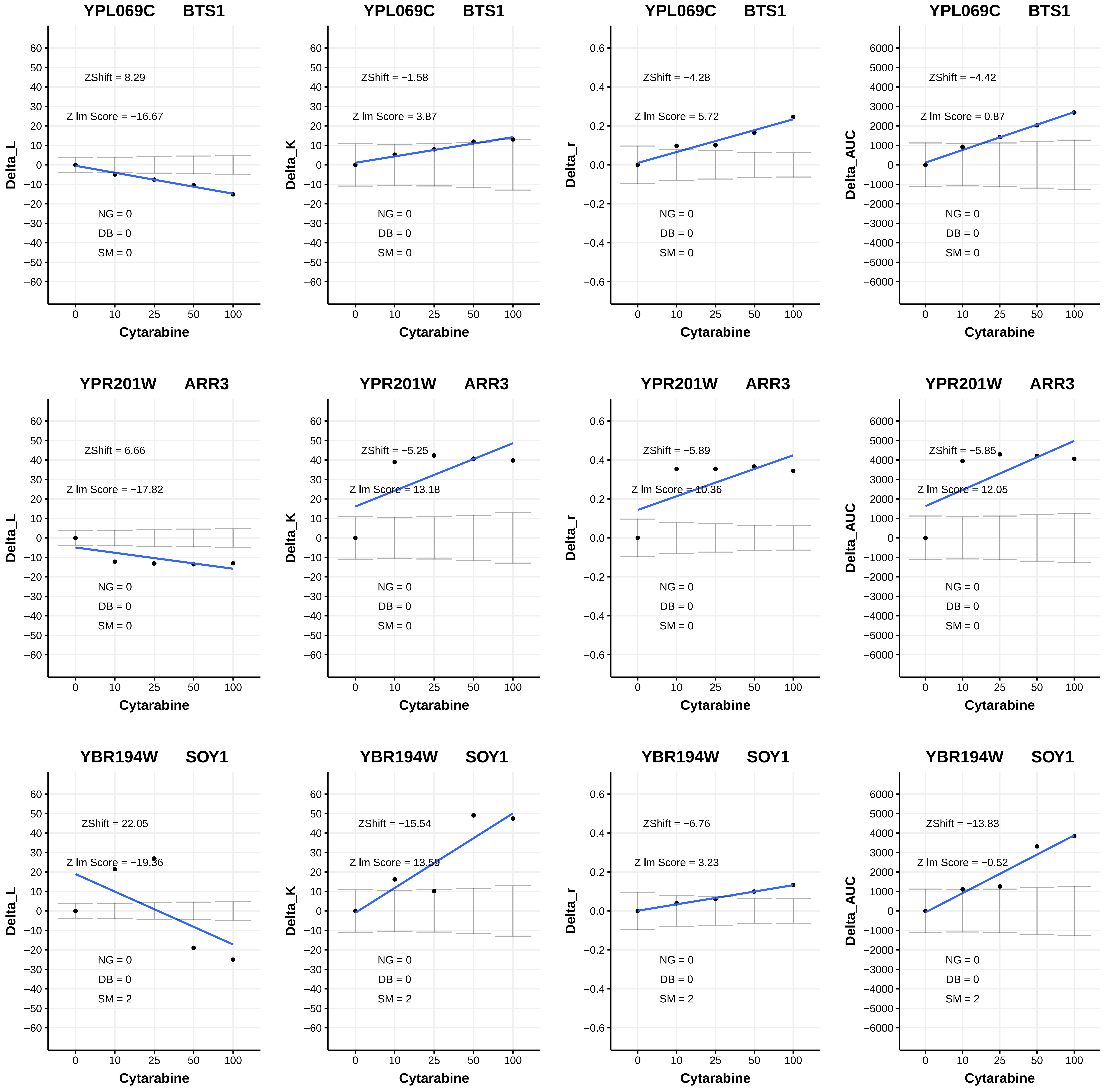
YPL069C BTS1
ZShift = 8.29
Z lm Score = −16.67
NG = 0
DB = 0
SM = 0
Delta_L
Cytarabine
YPL069C BTS1
ZShift = −1.58
Z lm Score = 3.87
NG = 0
DB = 0
SM = 0
Delta_K
Cytarabine
YPL069C BTS1
ZShift = −4.28
Z lm Score = 5.72
NG = 0
DB = 0
SM = 0
Delta_r
Cytarabine
YPL069C BTS1
ZShift = −4.42
Z lm Score = 0.87
NG = 0
DB = 0
SM = 0
Delta_AUC
Cytarabine
YPR201W ARR3
ZShift = 6.66
Z lm Score = −17.82
NG = 0
DB = 0
SM = 0
Delta_L
Cytarabine
YPR201W ARR3
ZShift = −5.25
Z lm Score = 13.18
NG = 0
DB = 0
SM = 0
Delta_K
Cytarabine
YPR201W ARR3
ZShift = −5.89
Z lm Score = 10.36
NG = 0
DB = 0
SM = 0
Delta_r
Cytarabine
YPR201W ARR3
ZShift = −5.85
Z lm Score = 12.05
NG = 0
DB = 0
SM = 0
Delta_AUC
Cytarabine
YBR194W SOY1
ZShift = 22.05
Z lm Score = −19.36
NG = 0
DB = 0
SM = 2
Delta_L
Cytarabine
YBR194W SOY1
ZShift = −15.54
Z lm Score = 13.59
NG = 0
DB = 0
SM = 2
Delta_K
Cytarabine
YBR194W SOY1
ZShift = −6.76
Z lm Score = 3.23
NG = 0
DB = 0
SM = 2
Delta_r
Cytarabine
YBR194W SOY1
ZShift = −13.83
Z lm Score = −0.52
NG = 0
DB = 0
SM = 2
Delta_AUC
Cytarabine

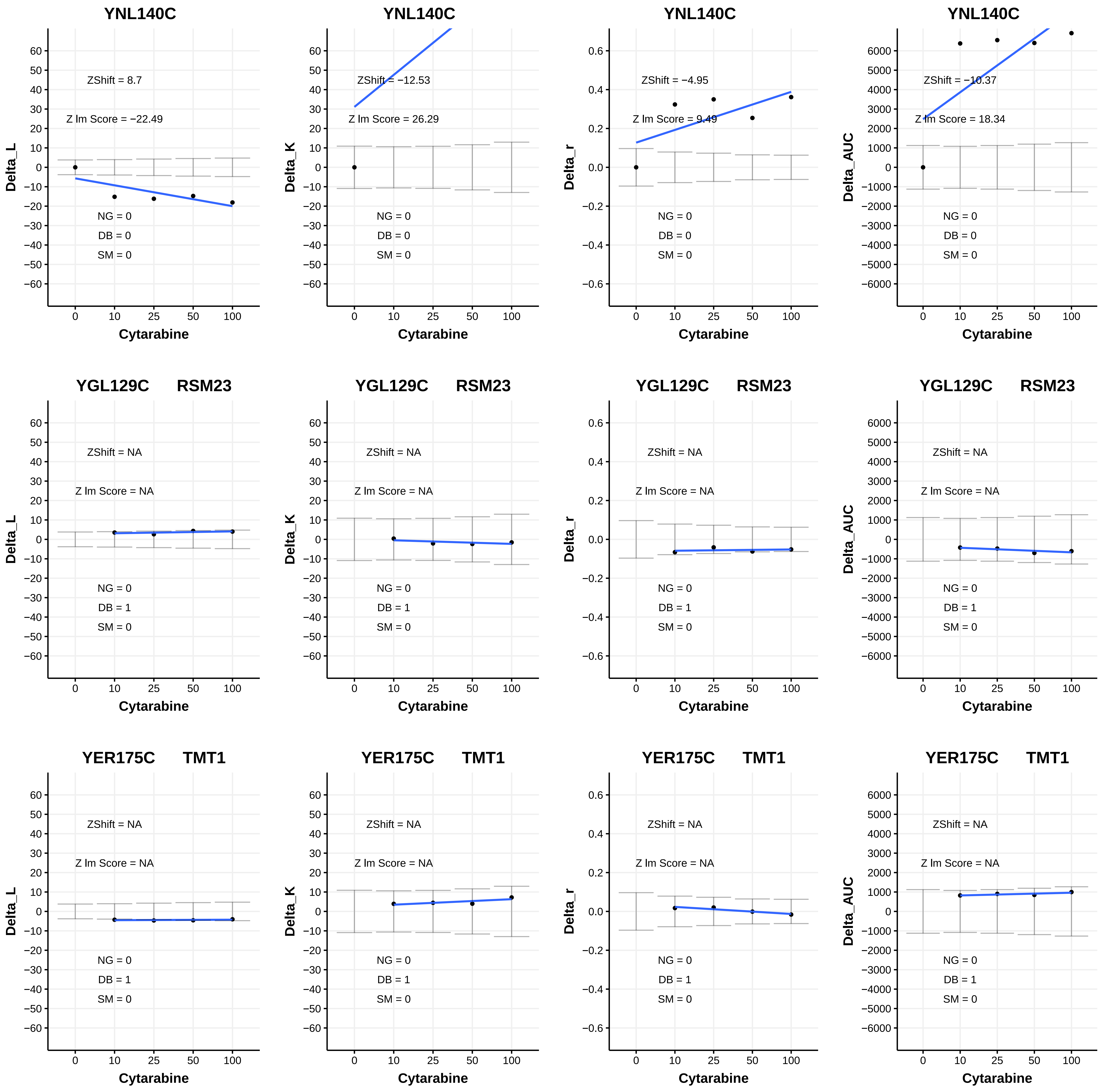

YNL140C
ZShift = 8.7
Z lm Score = −22.49
NG = 0
DB = 0
SM = 0
Delta_L
Cytarabine
YNL140C
ZShift = −12.53
Z lm Score = 26.29
NG = 0
DB = 0
SM = 0
Delta_K
Cytarabine
YNL140C
ZShift = −4.95
Z lm Score = 9.49
NG = 0
DB = 0
SM = 0
Delta_r
Cytarabine
YNL140C
ZShift = −10.37
Z lm Score = 18.34
NG = 0
DB = 0
SM = 0
Delta_AUC
Cytarabine
YGL129C RSM23
ZShift = NA
Z lm Score = NA
NG = 0
DB = 1
SM = 0
Delta_L
Cytarabine
YGL129C RSM23
ZShift = NA
Z lm Score = NA
NG = 0
DB = 1
SM = 0
Delta_K
Cytarabine
YGL129C RSM23
ZShift = NA
Z lm Score = NA
NG = 0
DB = 1
SM = 0
Delta_r
Cytarabine
YGL129C RSM23
ZShift = NA
Z lm Score = NA
NG = 0
DB = 1
SM = 0
Delta_AUC
Cytarabine
YER175C TMT1
ZShift = NA
Z lm Score = NA
NG = 0
DB = 1
SM = 0
Delta_L
Cytarabine
YER175C TMT1
ZShift = NA
Z lm Score = NA
NG = 0
DB = 1
SM = 0
Delta_K
Cytarabine
YER175C TMT1
ZShift = NA
Z lm Score = NA
NG = 0
DB = 1
SM = 0
Delta_r
Cytarabine
YER175C TMT1
ZShift = NA
Z lm Score = NA
NG = 0
DB = 1
SM = 0
Delta_AUC
Cytarabine
